# Comparative genomics of the social amoebae *Dictyostelium discoideum *and *Dictyostelium purpureum*

**DOI:** 10.1186/gb-2011-12-2-r20

**Published:** 2011-02-28

**Authors:** Richard Sucgang, Alan Kuo, Xiangjun Tian, William Salerno, Anup Parikh, Christa L Feasley, Eileen Dalin, Hank Tu, Eryong Huang, Kerrie Barry, Erika Lindquist, Harris Shapiro, David Bruce, Jeremy Schmutz, Asaf Salamov, Petra Fey, Pascale Gaudet, Christophe Anjard, M Madan Babu, Siddhartha Basu, Yulia Bushmanova, Hanke van der Wel, Mariko Katoh-Kurasawa, Christopher Dinh, Pedro M Coutinho, Tamao Saito, Marek Elias, Pauline Schaap, Robert R Kay, Bernard Henrissat, Ludwig Eichinger, Francisco Rivero, Nicholas H Putnam, Christopher M West, William F Loomis, Rex L Chisholm, Gad Shaulsky, Joan E Strassmann, David C Queller, Adam Kuspa, Igor V Grigoriev

**Affiliations:** 1 Verna and Marrs McLean Department of Biochemistry and Molecular Biology, Baylor College of Medicine, One Baylor Plaza, Houston, TX 77030, USA; 2 US Department of Energy Joint Genome Institute, 2800 Mitchell Drive, Walnut Creek, CA 9458, USA; 3 Department of Ecology and Evolutionary Biology, Rice University, 6100 Main Street, Houston, TX 77005, USA; 4 Department of Molecular and Human Genetics, Baylor College of Medicine, One Baylor Plaza, Houston, TX 77030, USA; 5 Department of Biochemistry and Molecular Biology, Oklahoma Center for Medical Glycobiology, University of Oklahoma Health Sciences Center, 110 N. Lindsay, Oklahoma City, OK 73104, USA; 6 dictyBase, Center for Genetic Medicine, Northwestern University, 750 N. Lake Shore Drive, Chicago, IL 60611, USA; 7 Section of Cell and Developmental Biology, Division of Biology, University of California, 9500 Gilman Dr, San Diego, La Jolla, CA 92093, USA; 8 Laboratory of Molecular Biology, MRC Centre, Hills Road, Cambridge CB2 2QH, UK; 9 Architecture et Fonction des Macromolécules Biologiques, UMR6098, CNRS, Universities of Aix-Marseille I & II, 13288 Marseille, France; 10 Department of Materials and Life Sciences, Sophia University 7-1 Kioi-Cho, Chiyoda-Ku, Tokyo 102-8554, Japan; 11 Departments of Botany and Parasitology, Faculty of Science, Charles University in Prague, Albertov 6, Prague 128 43, Czech Republic; 12 College of Life Sciences, University of Dundee, Dow Street, Dundee, DD15EH, UK; 13 Center for Molecular Medicine Cologne, University of Cologne, Joseph-Stelzmann-Str. 52, 50931 Cologne, Germany; 14 Centre for Biomedical Research, The Hull York Medical School and Department of Biological Sciences, University of Hull, Hull, HU6 7RX, UK

## Abstract

**Background:**

The social amoebae (Dictyostelia) are a diverse group of Amoebozoa that achieve multicellularity by aggregation and undergo morphogenesis into fruiting bodies with terminally differentiated spores and stalk cells. There are four groups of dictyostelids, with the most derived being a group that contains the model species *Dictyostelium discoideum*.

**Results:**

We have produced a draft genome sequence of another group dictyostelid, *Dictyostelium purpureum*, and compare it to the *D. discoideum *genome. The assembly (8.41 × coverage) comprises 799 scaffolds totaling 33.0 Mb, comparable to the *D. discoideum *genome size. Sequence comparisons suggest that these two dictyostelids shared a common ancestor approximately 400 million years ago. In spite of this divergence, most orthologs reside in small clusters of conserved synteny. Comparative analyses revealed a core set of orthologous genes that illuminate dictyostelid physiology, as well as differences in gene family content. Interesting patterns of gene conservation and divergence are also evident, suggesting function differences; some protein families, such as the histidine kinases, have undergone little functional change, whereas others, such as the polyketide synthases, have undergone extensive diversification. The abundant amino acid homopolymers encoded in both genomes are generally not found in homologous positions within proteins, so they are unlikely to derive from ancestral DNA triplet repeats. Genes involved in the social stage evolved more rapidly than others, consistent with either relaxed selection or accelerated evolution due to social conflict.

**Conclusions:**

The findings from this new genome sequence and comparative analysis shed light on the biology and evolution of the Dictyostelia.

## Background

The social amoebae have been used to study mechanisms of eukaryotic cell chemotaxis and cell differentiation for over 70 years. The completion of the *Dictyostelium discoideum *genome sequence provided a wealth of information about the basic cell and developmental biology of these organisms and highlighted an unexpected similarity between the cell motility and signaling systems of the social amoebae and the metazoa [1]. For example, the *D. discoideum *genome encodes numerous G-protein coupled receptors (GPCRs) of the frizzled/smoothened, metabotropic glutamate, and secretin families that were previously thought to be specific to animals, suggesting that the GPCR gene families branched prior to the animal/fungal split. Numerous other examples, such as SH2 domain based phosphoprotein signaling, the full complement of ATP-binding cassette (ABC) transporter gene families, and the apparently complex actin cytoskeleton, served to strengthen the idea that amoeba and amoeboid animal cells are related in a more fundamental way than one might have guessed based on their gross physiological traits. We compared the *D. discoideum *genome with a second dictyostelid genome, that of *Dictyostelium purpureum*, in order to determine the set of genes they share, as well as their genomic differences that might illuminate variations in physiology within the social amoeba.

The Amoebozoa are closely related to the opisthokonts (animals and fungi) and include unicellular amoebae (for example, *Acanthamoeba castellani*), obligate parasitic amoeba (for example, *Entamoeba histolytica*), the true slime molds (for example, *Physarum polycephalum*) and the social amoebae, or Dictyostelia (often incorrectly referred to as 'slime molds'). In the 10 years since the monophyly of the Amoebozoa was proposed [2], genomic-scale analysis has confirmed the hypothesis [3] and the phylogenetic relationships between the major amoeboid lineages have been clarified [4-6]. A molecular phylogeny of the Dictyostelia has been constructed and suggests four major groups; the basal, group 1 parvisporids that produce small spores; the group 2 heterostelids; the group 3 rhizostelids; and the group 4 dictyostelids, which include *D. purpureum *and the well-studied *D. discoideum *[7]. The dictyostelid group contains the largest number of described species of social amoeba and all of them produce large fruiting bodies with single sori, containing oblong spores, held aloft on a single cellular stalk.

*D. purpureum *differs from *D. discoideum *in a number of developmental and morphological ways [8]. In particular, during the social stage, *D. discoideum *delays irreversible commitment by cells to sterile stalk tissue until slug migration is complete. *D. purpureum*, by contrast, forms a stalk of dead cells as the slug moves towards light, increasing its ability to cross gaps [9]. In addition, *D. purpureum *makes taller fruiting bodies with smaller spores than *D. discoideum *[7]. *D. purpureum *fruiting bodies are purple with a triangular base formed from specialized stalk cells, whereas *D. discoideum *fruiting bodies are yellow and supported by a basal disc. *D. purpureum *also exhibits greater sorting into kin groups in the social stage than does *D. discoideum *[10,11].

The *D. discoideum *genome sequence was the first amoebozoan genome to become available, and the deduced gene list improved our understanding of the facultative multicellular lifestyle of the social amoeba [1,12]. Here we present our initial analysis of the *D. purpureum *genome and compare it to the *D. discoideum *genome. Since these two species represent the two major clades of the group 4 dictyostelids, a comparison of their genomes has revealed much of the genomic diversity and conservation within this group of social amoebae. Overall, the two genomes are similar in size and gene content, sharing at least 7,619 orthologous protein coding genes and many more paralogous genes. A global analysis of sequence divergence suggests that the genetic diversity of the dictyostelids is similar to that of the vertebrates, from the bony fishes to the mammals. Some large gene families are nearly completely conserved between these two dictyostelids, while others have markedly diverged. Our analyses highlight general characteristics that are conserved among the dictyostelids, as well as potential differences, linking the genomic potential with the physiology of these soil microbes.

## Results and Discussion

### Structure and comparative genomics of the *D. purpureum *genome

#### Genome assembly

The genome of *D. purpureum *strain DpAX1, an axenic derivative of QSDP1, was sequenced using a whole genome shotgun sequencing approach (see Materials and methods) and assembled into 1,213 contigs arranged into 799 scaffolds with 240 larger than 50 kb (Additional file 1). There were 12,410 genes predicted and annotated using the JGI annotation pipeline (see Materials and methods); these are available from the JGI Genome Portal [13] and from dictyBase [14]. Thirty-three percent of the genes were supported by at least one EST clone and 89% of genes displayed some similarity to a gene in the NCBI non-redundant gene databases (Additional file 1). The genome size, gene count and average gene structure are very similar to those of *D. discoideum *(Table 1). Moreover, a recent comparative transcriptome analysis of *D. purpureum *and *D. discoideum*, using 'RNA-sequence' (RNA-seq), provides evidence for the transcription of 7,619 genes encoding protein orthologs within these species, or approximately 61% of the predicted *D. purpureum *genes [15].

**Table 1:** Comparison between the predicted protein coding genes of *D. purpureum *and *D. discoideum*

Feature	*D. purpureum*	***D. discoideum***^**a**^
Genome size (Mb)	33	34
Number of genes	12,410	13,541
Gene density (kb per gene)	2.66	2.5
Mean gene length (nucleotides)	1,760	1,756
Intron per gene (spliced genes)	1.51	1.9
Mean intron length (nucleotides)	177	146
Mean protein length (amino acids)	483	518

^a^From [1].

#### Repetitive elements and simple sequence repeats

The *D. purpureum *genome contains 1.1 Mb of transposons (3.4%), fewer than in D. discoideum. The largest families of transposons are Gypsy (approximately 400 kb, 35.8% of total transposons), Mariner (approximately 186 kb, 16.7%), MSAT1_Dpu (126 kb, 11.4%), and hAT (105 kb, 9.5%).

**Figure 1:**
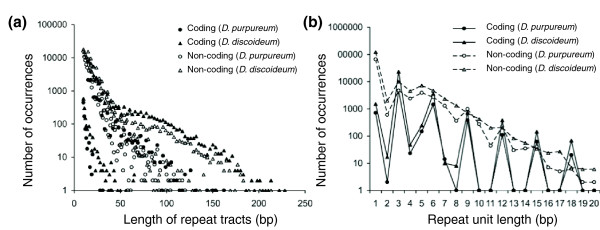
**Number of occurrences of simple sequence repeats in *D. purpureum *and *D. discoideum *genomes**. **(a,b) **The numbers of repeats were classified by the length of repeat tracts (a) and the length of repeat units (b). The *D. purpureum *genome (circles) has fewer and shorter microsatellites than the *D. discoideum *genome (triangles) in both coding regions (solid circles and triangles, and solid lines) and non-coding regions (open circles and triangles, and dashed lines). Not shown are three *D. discoideum *repeats above 250 nucleotides in (a). The minimum number of repeats of the unit motif was 10 repeats for mononucleotides, 7 repeats for dinucleotides, 5 repeats for trinucleotides, 4 repeats for tetranucleotides, 3 repeats for pentanucleotides and longer (6- to 20-nucleotide) motifs.

The previously sequenced *D. discoideum *genome showed an unusually high number, length, and density of simple sequence repeats, including triplet repeats that code for amino acid homopolymers [1]. If unopposed by selection, simple sequence repeats can accumulate in genomes because of their high mutation rates and mutation to different repeat numbers that occur by misalignment and slippage during replication [16]. They are often thought of as non-functional 'junk' DNA, though some are known to be functional [17], and the expansion of some triplet repeats in humans are known to cause disease when the number of repeats exceeds a particular threshold [18]. Despite its considerable evolutionary distance from *D. discoideum *(see below), *D. purpureum *also has a considerable density of simple sequence repeats (Figure 1a). Simple sequence repeats comprise 4.4% of the *D. purpureum *genome, compared to 11% in *D. discoideum *[1]. There are fewer long repeats that exceed 100 bp in length; 54 in *D. purpureum *compared to 1,436 in *D. discoideum*. The lower proportion of simple repeats in the *D. purpureum *genome and their shorter length may be due to current status of the assembly relative to the *D. discoideum *genome, since these repeats are difficult to assemble. Dinucleotide repeats, often the most common repeat in other species, are comparatively rare in both dictyostelid genomes (Figure 1b) [1].

#### Amino acid homopolymers

**Figure 2:**
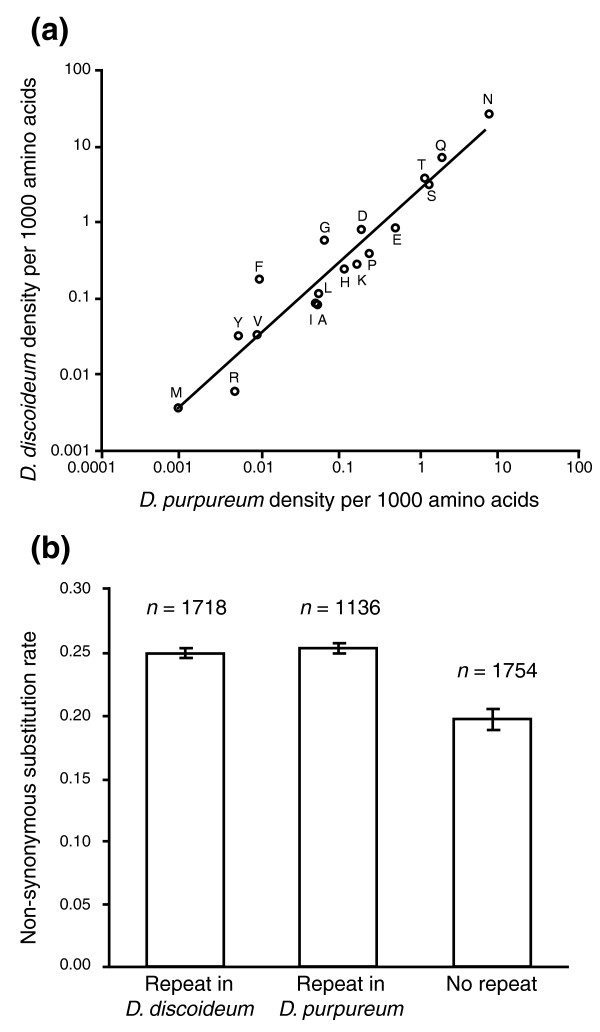
**Densities of different homopolymer amino acid repeats in *D. purpureum *and *D. discoideum***. **(a) **The density of each kind of amino acid repeat was calculated by summing the lengths of non-random repeats of that amino acid (Table S1 in Additional file 1) over protein sequences of all genes from *D. purpureum *and *D. discoideum*, dividing by the total length of coding sequence, and multiplying by 1,000. Letters indicate which amino acid each point represents. The Pearson's correlation coefficient between them is 0.997, *P *< 0.001. **(b) **Mean (± standard error) non-synonymous substitution rates (dNs) of genes with and without amino acid repeats. The non-synonymous substitution rates were calculated between orthologs (excluding repeat sequences) of *D. purpureum *and *D. discoideum*. Orthologs without amino acid repeats have significantly lower dN than orthologs with repeats in either *D. discoideum *and *D. purpureum *(Students *t*-test, both tests *P *< 0.0001). Error bars show standard errors of the means.

One of the most distinctive characteristics of the *D. discoideum *genome is the extreme abundance of amino acid homopolymers within coding sequences [1]. As in *D. discoideum*, simple sequence repeats are common in *D. purpureum *coding sequences (Figure 1a), particularly those with repeat motifs of three nucleotides or multiples of three (Figure 1b). These types of repeats contribute to many amino acid homopolymers (Figure S1 in Additional file 1), including 2,645 that are longer than expected by chance (>5 to >9 residues, depending on the amino acid; Table S1 in Additional file 1). Though the abundance and density is lower than in *D. discoideum*, the relative abundance of different amino acids repeats in *D. purpureum *is very similar, with asparagine and glutamine repeats dominating, followed by serine and threonine (Figure 2a). The correlation between the two species in the densities of different amino acid repeats is 0.997 (Pearson's correlation coefficient, *P *< 0.001), much higher than either species' correlation with *Saccharomyces cerevisiae *(0.516 for *D. discoideum*, and 0.486 for *D. purpureum*), or with *Drosophila melanogaster *(0.241 and 0.238). However, the correlations are also high for the densities of amino acid repeats with the A/T-rich protist *Plasmodium falciparum *(0.917 and 0.923), in agreement with a study showing that A/T content exerts a major influence on which amino acid repeats accumulate and persist within genomes [19].

Codon usage within these amino acid homopolymers is quite similar to codon usage for the same amino acids outside of repeats, with a pattern quite similar to *D. discoideum *(Figure S2 in Additional file 1). Again, as in *D. discoideum*, many amino acid homopolymers contain a single codon, consistent with the relatively recent expansion of those triplet repeats. However, the codon diversity of *D. purpureum *amino acid repeats is significantly higher than it is for *D. discoideum *(Figure S3 in Additional file 1), consistent with the *D. discoideum *repeats being younger, with less time to accumulate changes from the original codon.

The potential function of most amino acid repeats is unknown, but the availability of the *D. purpureum *genome permits some new tests. If amino acid repeats are generally functionally important, they should tend to be conserved in their position within orthologous proteins. Sixty-four percent of the 2,645 *D. purpureum *amino acid repeats and 68% of the 11,243 *D. discoideum *repeats occur in genes that do not have homologs in the other species. Even in those with orthologs, only 19% of *D. purpureum *repeats and 5% of the *D. discoideum *repeats appeared to be homologous within global alignments of their respective proteins. The count of homologous repeats would be higher if we included matches where at least one falls below the threshold expectation for non-random homopolymers (for example, a match between 25 asparagines in *D. discoideum *and 8 in *D. purpureum *would be excluded as a chance event; *P *> 0.01; Table S1 in Additional file 1). On the other hand, some could be fortuitous matches forced by a large number of repeated amino acids that are not truly homologous. Inspection of selected sequences shows at least some that appear to be convincing homologs, with strong identity on both sides of the repeat (Figure S4 in Additional file 1). Still, the apparent small fraction of homologous repeats suggests that the very similar patterns of amino acid homopolymer abundance and distribution do not come primarily from conserved ancestral repeats. Instead they may come from some shared physiological properties - perhaps distinctive DNA polymerases or repair enzymes or high AT-content - that generate similar patterns independently.

In addition to the lack of homology for amino acid homopolymers between *D. discoideum *and *D. purpureum*, several pieces of evidence suggest that these triplet repeats may be 'junk' that accumulates due to weak selection on proteins that are relatively unimportant for fitness. For genes that have homologs in the two species, those with amino acid repeats in either species have higher non-synonymous substitution rates in the non-repeat regions, as expected if genes with repeats are generally less subject to purifying selection (Figure 2b). Another indicator of the degree of selective constraint on a gene is its expression level, particularly in the single-celled, vegetative stage where the selective pressure is likely to be the greatest. If amino acid repeats accumulate in genes where selective constraints are low, we would predict that they will be more common in genes expressed in the social or developmental stages, as opposed to vegetative stages. Using the recent comparison of the transcriptional profiles of *D. discoideum *and *D. purpureum *development by RNA-seq analysis [15], this prediction is confirmed (Figure S5a,c in Additional file 1). Similarly, we would predict, looking only at RNA-seq reads from the vegetative stage, that genes coding for amino acid repeats would be less abundant and this is also confirmed (Figure S5b,d in Additional file 1). In sum, although a small number of repeats appear to be conserved over long periods of time, most appear to have arisen relatively recently in genes where selection against amino acid changes is weak.

#### Phylogeny of *D. purpureum*

**Figure 3:**
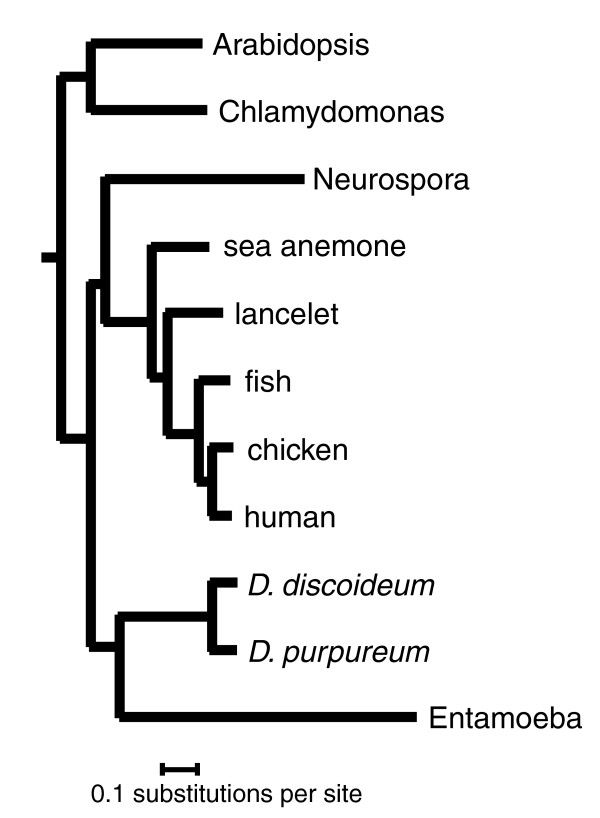
**Phylogeny of the dictyostelids**. Orthologs (389) defined by pairwise genome comparisons for reciprocal best hits using BLASTP from human [100] versus each of *Oryzias latipes *[100], *Gallus gallus *[100], *Branchiostoma floridae *[101], *Nematostella vectensis *[28], *Neurospora crassa *(Broad release 7) [102], *Arabidopsis thaliana *(TAIR8) [103], *Chlamydomonas reinhardtii *[104], *Dictyostelium discoideum *[14], plus *D. discoideum *versus each of *D. purpureum*, and *Entamoeba histolytica *[22]. A concatenated alignment of the orthologs was analyzed with mrBayes 3.1.2 using the WAG model, I + Gamma for 100,000 generations, with the first 50% of sampled trees discarded. The resulting consensus tree was rooted at the midpoint of the branch connecting the green plants to the rest of the tree.

A phylogeny based on small subunit ribosomal RNA gene sequences places *D. purpureum *and *D. discoideum *into distinct clades within the most derived of the four groups of social amoebae, the group 4 dictyostelids [7]. Thus, these two species should represent much of the diversity of the group. We constructed a global phylogeny of representative plant, animal, fungal and amoebal species, based on 389 orthologous gene clusters, in order to estimate the divergence of *D. purpureum *and *D. discoideum *relative to other eukaryotes (Figure 3). This analysis suggests that the group 4 dictyostelids span a comparable degree of protein sequence divergence as occurs among vertebrate species ranging from the bony fishes to the mammals. Recent comprehensive analyses of orthologous protein clusters from complete predicted proteomes suggests that the rates of protein evolution in the Amoebozoa are comparable to those of the plants and animals [20]. If gene sequence evolution occurs at the same rate in the two groups, these two observations suggest that *D. purpureum *and *D. discoideum *shared a common ancestor approximately 400 million years ago.

#### Horizontal gene transfer

The initial description of the *D. discoideum *genome included 18 genes that were proposed to be horizontal gene transfer (HGT) events from bacterial species [1]. After 5 years of refinement of the underlying genome sequence, 16 *D. discoideum *genes remain potential HGT events. They have not been recognized in the characterized plant, animal or fungal genomes, and each of them is phylogenetically embedded within a bacterial clade. In addition, the thymidylate synthase gene, *thyA*, has been confirmed as an HGT; it is present only in a minority of the described bacterial species and is structurally unrelated to the canonical eukaryotic thymidylate synthase [21]. To narrow the time frame wherein the HGT events might have occurred, we searched the *D. purpureum *genome for orthologs to these genes. Each of the proposed *D. discoideum *HGT genes have an ortholog in the *D. purpureum *genome (Table 2). This suggests that all 16 of these potential HGT events occurred after the divergence of the Amoebozoa from the plants and animals, but prior to the radiation of the group 4 dictyostelids.

**Table 2:** Candidate horizontal gene transfers from Bacteria

**Pfam domain**^**a**^	**Function in bacteria**^**b**^	***D. discoideum *dictyBase ID**^**c**^	**Function in *D. discoideum***^**c**^	***D. purpureum *protein ID**^**d**^	*D. purpureum *dictyBase ID
Beta_elim_lyase	Aromatic amino acid lyase	DDB_G0281127	Unknown	154359	DPU_G0057350
BioY	Biotin metabolism	DDB_G0292424	Unknown	79107	DPU_G0053374
Cna_B	Unknown	DDB_G0292696	*colA*, Colossin A slug protein	96318	DPU_G0069302
Peroxidase	Dyp_peroxidase	DDB_G0273083	Unknown	35644	DPU_G0056076
Endotoxin_N	Insecticidal crystal protein	DDB_G0289249	Unknown	96621	DPU_G0058298
IPT	Isopentenyl transferase	DDB_G0277215	Discadenine production	92712	DPU_G0062048
IucA_IucC	Siderophore synthesis	DDB_G0294004	Unknown	No model^e^	No model^e^
OsmC	Osmoregulation	DDB_G0268884	Unknown	93234	DPU_G0070822
Peptidase S13	Dipeptidase/β-lactamase	DDB_G0271902	Penicillin-sensitive carboxypeptidase	6688	DPU_G0063426
PP_kinase	Polyphosphate synthesis	DDB_G0293524	Polyphosphate synthesis	45674	DPU_G0062710
TerD	Tellurium resistance	DDB_G0277501	capA/B	57536	DPU_G0062378
Thy1	Thymidylate synthesis	DDB_G0280045	thyA, thymidylate synthesis	149635	DPU_G0069806
DUF885	Unknown	DDB_G0278355	Unknown	155362	DPU_G0059974
DUF1121	Unknown	DDB_G0277411	Unknown	39626	DPU_G0062812
DUF1289	Unknown	DDB_G0282477	Unknown	27078	DPU_G0056950
DUF1294	Unknown	DDB_G0285825	Unknown	86664	DPU_G0067456

^a^The Pfam domain designation [99]. ^b^Confirmed or proposed function of the prokaryotic ortholog is given. ^c^The *D. discoideum *gene ID number and functional annotation are from dictyBase [14]. ^d^*D. purpureum *ortholog protein ID numbers [13]. All orthologs are 90 to 100% similar in amino acid sequence to the *D. discoideum *protein over >90% of their length. ^e^A related sequence is present, but no protein model could be produced from the current assembly.

Functional information now exists for 6 of the 16 proposed HGT genes and it is interesting to see how the dictyostelids have utilized these contributions from bacteria. ThyA has completely replaced an essential enzyme in central metabolism [21]. Since it is also present in the amoebozoan slime mold *Physarum polycephalum *(GenBank accession number [GenBank:AAY87038] [22]), the change over to the rare bacterial enzyme must have taken place quite early in the radiation of the amoebozoa. The isopentenyl transferase, IptA, produces discadenine, which is a sporulation inducer and spore germination inhibitor [23]. Another gene, *pscA*, encodes an active penicillin-sensitive peptidase but its function is not known [24], and Ppk1 is a bacterial type polyphosphate synthase [25]. Colossin A (ColA) appears to be a structural protein of the slug that was fashioned out of hundreds of repeats of a bacterial Cna_B domain [1]. CapA and CapB are two cAMP-binding proteins whose carboxy-terminal half is derived from a subunit of a bacterial tellurium resistance complex [26]. Recently, CapB was identified in a proteomic screen for centrosomal proteins [27].

#### Conserved gene order between the *D. purpureum *and *D. discoideum *genomes

Genomes evolve through base substitution and insertion/deletion, and also through rearrangements that alter the order and orientation of genes on chromosomes. Synteny, the nature and extent of conserved gene order between species, serves as an important gauge of the dynamics of genome evolution [28]. To characterize the potential synteny between *D. purpureum *and *D. discoideum*, we identified blocks of approximately conserved gene order between their genomes, and compared the number and sizes of these potential conserved syntenic blocks to control genomes in which the gene orders were artificially scrambled. Although the *D. purpureum *genome is not fully assembled, the current level of contiguity allows for an analysis of conserved gene order on a small scale (approximately 50 kb). Blocks of potential synteny were constructed by single-linkage clustering of *D. purpureum *genes, where pairs of genes are considered linked if (i) they fall on the same scaffold of the assembly with at most *w *intervening genes that have *D. discoideum *orthologs, and (ii) their *D. discoideum *orthologs all fall on a single chromosome, with no more than *w *intervening genes that have *D. purpureum *orthologs. For stretches of perfectly conserved gene order (blocks constructed with *w *= 0), 4,734 (63%) of the 1:1 ortholog pairs used in the analysis lie in a genomic block of conserved gene order involving at least two genes in each genome. The mean size of such blocks is 2.8 genes in each genome, with the longest perfectly conserved stretch containing 10 genes.

To determine the maximum length scale over which significant conservation of gene order persists, we compared the increase in potential syntenic clusters as a function of an increasing number of intervening genes (*w*) for *D. purpureum *versus *D. discoideum *to the rate obtained for the permutation controls (Figure S6 in Additional file 1). We found that for up to about 15 intervening genes, potential conserved gene clusters grow significantly faster than what is expected for the same two genomes with randomized gene orders, which provides a conservative threshold for identifying blocks of conserved gene order. With this estimate, 76% of orthologous gene pairs participate in a block of approximately conserved gene order, compared to 5.8 ± 0.4% in controls, with a false positive rate, on a gene-by-gene basis, of approximately 7%. The 5,793 genes contained in these blocks, and their positions in the genome, are listed in Additional file 2. This indicates that the majority of orthologs in *D. purpureum *and *D. discoideum *are found in small neighborhoods of exactly conserved gene order between the two species, and that these neighborhoods are themselves clustered into larger regions of approximately conserved gene order.

### Gene content comparisons of *D. purpureum *and *D. discoideum *genomes

#### Non-coding RNA genes

The described catalog of non-coding RNAs (ncRNAs) in the Dictyostelia was long limited to tRNAs, rRNAs, and a handful of experimentally identified short RNAs, all found in *D. discoideum *(for review, see [29]). Recent work has expanded this repertoire to include a family of spliceosomal ncRNAs and two classes (class I and class II) of novel ncRNAs [30,31]. The spliceosomal RNAs identified in *D. discoideum*, U1, U2, U4, U5, and U6, are each characterized by both specific RNA-binding motifs and the ability to fold into characterized secondary structures [30,31]. Using a modified BLAST search (Additional file 1), we have identified a set of *D. purpureum *spliceosomal homologs that are predicted to fold into the appropriate secondary structures (Table S3a in Additional file 1).

In *D. discoideum *a '*Dictyostelium *upstream sequence element' (DUSE) has been described that sits approximately 63 bp upstream of many ncRNAs, including the class I and II ncRNAs [31]. Identification of the DUSE motif ([AT]CCCA[AT]AA) in *D. purpureum *revealed that a DUSE also sits upstream of all *D. purpureum *spliceosomal RNA genes. The DUSE also enriches for a family of putative *D. purpureum *ncRNAs that are homologous to the two novel classes of *D. discoideum *ncRNAs. This suggests that the DUSE is not specific to *D. discoideum*.

**Figure 4:**
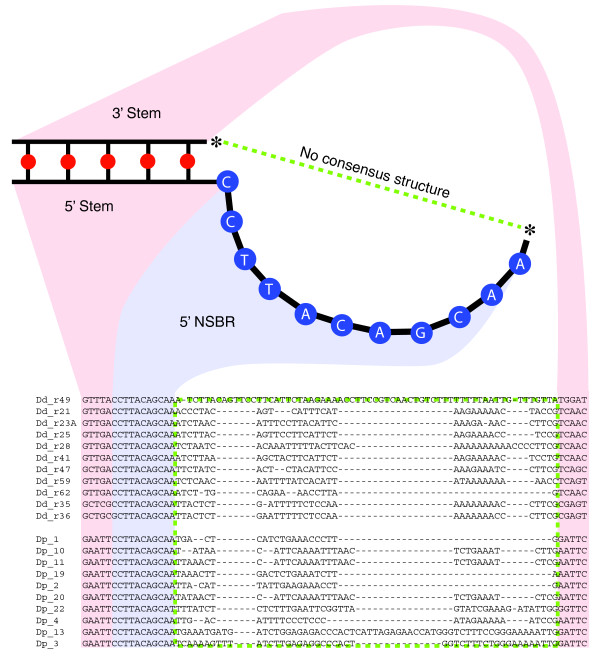
**Putative novel ncRNAs in *D. purpureum***. The sequences and predicted structures of select class I and II ncRNAs in both *D. discoideum *and *D. purpureum*. The red dots indicate base pair positions that possess high mutual information but lack sequence identity. This region contains the 5' and 3' stem sequences, which are conserved among each species but not between both. Blue dots indicate base positions where sequences are perfectly conserved, corresponding to the non-self-binding region (NSBR). The starred positions are connected via a variable sequence (green box in alignment), which lacks primary sequence or secondary structure conservation (see Figure S8 in Additional file 1 for complete alignment).

Operating under the assumption that the DUSE sits upstream of certain ncRNAs in *D. purpureum*, we sought to identify novel ncRNAs by focusing on DUSE-enriched 8-bp sequences (see Additional file 1 for methods). Two of the three 8-mers that were found to be highly enriched, CCTTACAG and CTTACAGC, also occur in the novel classes of *D. discoideum *ncRNAs. These ncRNA gene products are 50 to 60 bp long and have distinct 5' and 3' sequences predicted to form 5-bp stem structures that are conserved within each class (Figure 4). Both classes share a 12-bp 'bulge' sequence, CCTTACAGCCAA, which is immediately 3' to the 5' stem sequence [30]. This 'bulge' sequence is predicted to not bind with any other region of the ncRNA, thus constituting a non-self-binding region (NSBR). The two 8-mers both sit within this NSBR.

To identify putative homologs to the class I and II ncRNAs in *D. purpureum*, we used the structural characteristics of these ncRNAs to filter all sequences containing the DUSE-enriched 8-mers. Forty members of the class I and II ncRNAs were originally identified in *D. discoideum*. Some are described as putative, with nine lacking the canonical bulge sequence, and five others lacking an upstream DUSE, or having a degenerate DUSE. The class I ncRNAs have a 5' stem sequence of GTTGA, while two class II ncRNAs have a 5' stem sequence of GCTCG, and all members have a 3' stem sequence complementary to the 5' stem sitting 40 to 70 bp away from the 5' stem [29].

In our analysis of the masked *D. discoideum *genome, we identified 46 occurrences of the CTTACAGC 8-mer (Additional file 1). Of these, 26 possess both an upstream DUSE and a 5'/3' stem pair sitting 40 to 70 bp apart, and each corresponds to a previously identified class I or II ncRNA. In the masked *D. purpureum *genome there are 61 occurrences of the CCTTACAG 8-mer; 26 of these 8-mers have both an upstream DUSE and a 5'/3' stem pair consisting of an identical 5' sequence (GAATT) (Figure 4). These results suggest a class of ncRNAs in *D. purpureum *similar to the class I and II ncRNAs found in *D. discoideum*.

The comparative genomics approach to identifying these ncRNAs in *D. purpureum *lends deeper insight into their function. The 5' and 3' stem sequences have diverged between species, but have done so in a compensatory manner that maintains the predicted 5'/3' structure. The NSBR sequence, however, has remained perfectly conserved between species, and in neither species is it predicted to self-bind. This suggests a functional role for the NSBR beyond self-interaction, possibly as a binding site for another functional element. Initial genomic analysis of the dictyostelids *Dictyostelium citrinum *and *Polysphondylium violaceum *also revealed putative ncRNAs with an upstream DUSE, the conserved NSBR sequence, a 5'/3' stem structure, but 5'/3' stem sequences different from those of *D. discoideum *and *D. purpureum *(unpublished data).

#### Determination of protein orthologs

Of the 12,410 predicted *D. purpureum *proteins, we identified 7,619 that are likely to be orthologous to *D. discoideum *proteins using the Inparanoid algorithm, best reciprocal blast hits, and manual curation (Additional file 3). An additional 2,759 predicted proteins are similar to genes in *D. discoideum*, while 2,001 appear to be unique to *D. purpureum *(Additional file 4). Thus, at least 84% of the protein-coding genes in *D. purpureum *share orthologs or paralogs in the *D. discoideum *genome. The gene product predictions from the *D. purpureum *genome should be enormously useful for further refinement of the predicted proteome of *D. discoideum*. Some gene families are completely conserved between *D. purpureum *and *D. discoideum*, with clear orthologs for every member of the family, while other families appear to have undergone considerable divergence between the two species (Figure S9 in Additional file 1, and Additional file 4). The differences amongst gene family members should illuminate the physiological differences between these two dictyostelids, whereas the similarities may indicate where the selective pressures, exerted by their common environment, have resulted in stable gene inventories required for survival.

#### Polyketide synthases

Polyketide synthases (PKSs) are enzymatic production lines for making small molecules by the repeated condensation of malonyl-CoA and other thio-esters of coenzyme A (CoA). A large number of polyketides exist and are probably made for ecological purposes, but they also serve as model natural products for the development of drugs, antibiotics and food additives. Soil amoebae are not commonly regarded as polyketide producers, but they too must face complex ecological challenges, which could be met by polyketide production; competition from other amoebae, infection by bacteria and predation by nematodes, amoebae and fungi. A small number of potential eco-chemicals have been identified from social amoebae [32,33], but the completed *D. discoideum *genome sequence revealed a much larger potential [1,34,35]. These PKSs are large, modular proteins of 2,000 to 3,500 amino acids, each having a core of domains for the condensation reaction, together with optional domains for methylation, carbonyl reduction and product release. Two have a unique, 'steely', architecture in which a second PKS - a chalcone synthase - is fused to the carboxyl terminus of a modular PKS [36]. One of these steely proteins makes the precursor of differentiation-inducing factor (DIF)-1, a chlorinated signal molecule for stalk cell differentiation [37], and the other a pyrone or an olivetol derivative [35,36,38].

**Figure 5:**
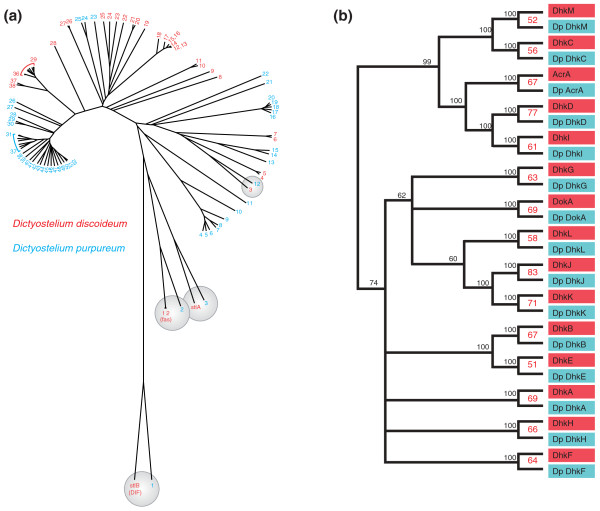
**Polyketide synthases and histidine kinases of *D. purpureum***. **(a) **The phylogram of putative polyketide synthases was constructed from the ketoacyl synthase and acyltransferase domains of each predicted protein. Red numbers indicate *D. discoideum *genes and blue numbers indicate *D. purpureum *genes, with the corresponding genomic loci given in Table S6 in Additional file 1. Orthologous genes are circled in grey; the steely (*stlA*, *stlB*) and the putative fatty acid synthase (*fas*) genes are indicated. **(b) **Unrooted phylogram of the putative histidine kinases and the AcrA protein of *D. discoideum *and *D. purpureum *(denoted with 'Dp' before the gene names). Bootstrap values at each node are given for 1,000 iterations of tree building. The red numbers indicate the percent amino acid sequence identity between each pair of predicted proteins. Note the striking one-to-one correspondence between each gene in the two species.

The *D. purpureum *genome has 50 predicted PKS genes. We constructed phylogenetic trees using the highly conserved ketoacyl synthase and acyl transfer domains of the PKS genes from both species to discern evolutionary relationships (Figure 5a; see Table S6 in Additional file 1 for corresponding genomic loci). The two steely genes within each species are only distantly related to each other but are clearly orthologous between species. This implies that both genes were present in the last common ancestor and that their function has been maintained in both species. There is also a clear ortholog in *D. purpureum *of the methyltransferase catalyzing the last step of DIF-1 biosynthesis [39] and so *D. purpureum *is likely to make DIF-1, like *D. discoideum*, and *Dictyostelium mucoroides *[40], another group 4 dictyostelid [7]. Two other clear orthologous pairs of genes are apparent. *Dp2 *and the very similar *Dd1*/*Dd2 *likely encode fatty acid synthases based on their similarity to other fatty acid synthases and their high expression levels. *Dp12 *and *Dd3 *are of unknown function, though mutation of *Dd3 *causes a 'cheater' phenotype, suggesting that it may produce a developmental signal [41].

In contrast to the four *D. purpureum *genes described above, most *D. purpureum *PKS genes do not have obvious orthologs in *D. discoideum*, indicating species-specific expansions. Given the overall gene conservation between these two species, the divergence of the PKS gene sets is striking. We speculate that this greater evolutionary fluidity reflects different selective pressures placed on the two species, perhaps by different competitor species in their ecological niches, and therefore that most of their polyketides are produced for ecological purposes.

The *D. purpureum *genome confirms the high potential of social amoebae for polyketide production. The relative paucity of orthologs to *D. discoideum *PKSs raises the possibility that polyketide production varies substantially from species to species amongst the dictyostelids. As natural products remain the major source of drugs [42], this diversity suggests that natural products of social amoebae deserve systematic exploration.

#### The ATP-binding cassette transporters

The ABC transporters are one of the largest protein superfamilies that are encoded by any genome. In stark contrast to the lineage-specific radiation of the PKS proteins, the complement of ABC transporters has remained remarkably stable since the divergence of *D. purpureum *and *D. discoideum*. ABC proteins all have a conserved domain of 200 to 250 amino acids, the ATP-binding cassette, and typically have 12 transmembrane domains. Seven different eukaryotic families have been defined on the basis of sequence homology, domain topology and function. The superfamily has been extensively analyzed in *D. discoideum *[43] and this allowed a detailed comparison to the predicted *D. purpureum *ABC superfamily members. Both genomes carry similar numbers of ABC genes overall, but differences in gene number can be observed within groups of closely related genes belonging to the largest families (Tables S7 and S8 in Additional file 1). Only 58 genes can be considered clear orthologs; the remaining genes should be considered paralogs (Figure S10 in Additional file 1). These genes may play partially redundant roles and this might allow their sequences to drift to a point of uncertain orthology.

The Tag subfamily proteins (TagA-D) of the ABC B family have a novel domain structure with a serine protease domain on the amino terminus, a single set of six transmembrane domains, and one ABC domain on the carboxyl terminus. Three of the Tag proteins have defined roles in cell differentiation; TagA is involved in early cell fate determination [44], TagB is required for pre-stalk cell differentiation [45], and TagC is expressed in pre-stalk cells and required to process acyl-CoA binding protein into a spore differentiation peptide signal [46]. Interestingly, TagA, B and C are conserved between *D. purpureum *and *D. discoideum*, but whereas the TagA orthologs are quite similar, the relationship between the TagB and TagC proteins in the two species is not as clear (they were named based on their gene order within a block of synteny between *D. discoideum *and *D. purpureum*).

#### Protein kinases

*D. purpureum *has a similar complement of protein kinases compared to *D. discoideum*. Like *D. discoideum*, *D. purpureum *does not appear to have receptor tyrosine kinases, or other notable protein kinases such as P70, ATM, and PASK. There are 262 eukaryotic protein kinases and 41 atypical protein kinases, including potential pseudogenes (Table S9 in Additional file 1). This compares to 247 identified eukaryotic protein kinases and 39 atypical protein kinases in *D. discoideum *[47].

The 14 *D. purpureum *histidine kinase genes, and the related *acrA *gene, each have an unambiguous ortholog in *D discoideum *(Figure 5b). There is little homology between non-orthologous genes outside of the kinase domain. Thus, the histidine kinases appear to have diverged from a common ancestor before the radiation of the dictyostelids, suggesting that each one of them carries out a distinct and conserved function. The adenylyl cyclase of *D. discodeum*, AcrA, carries a non-functional histidine kinase domain with mutations in key amino acids that preclude kinase activity [48]. This domain and its variations are well conserved in the *D. purpureum *AcrA, suggesting that there is a selective advantage to maintaining this non-catalytic domain, probably as a dimerization domain.

The catalytic subunit of cAMP dependent protein kinase (PKA), PkaC, in *D. purpureum *shows 65% amino acid identity with its *D. discoideum *ortholog. The homology is highest in the catalytic core and lowest in the low complexity amino-terminal domain, with the exception of the region encompassing the αA amphipathic helix [49]. This helix, which is predicted to interact with a hydrophobic pocket on the catalytic core of the enzyme, is 95% identical in these dictyostelids, which is suggestive of a conserved regulatory function. The regulatory subunit of PKA, PkaR, of *D. purpureum *and *D. discoideum *shows 79% amino acid identity and each of them lack the dimerization domain found in metazoa.

#### G-protein coupled receptors

GPCRs are found in all eukaryotes and transduce a variety of extracellular signals via heterotrimeric G-proteins and effector proteins inside the cell to elicit physiological responses. GPCRs are characterized by an extracellular domain, an intracellular domain, and a core domain that contains seven transmembrane regions. The GPCRs are subdivided into six major families that, aside from their conserved secondary domain structure, do not share significant sequence similarity. The *D. purpureum *genome encodes the same families of GPCRs as in *D. discoideum*, but has a reduced total number, which is mainly due to differences in the numbers of cAMP, family 3 and family 5 receptors (Figure S12 and Table S10 in Additional file 1). There are only two cAMP receptors in the *D. purpureum *genome, namely orthologs of *Dictyostelium carA *and *carB*, but there are no orthologs of *carC *and *carD*. In addition, there are 35% fewer family 3 receptors and 40% fewer family 5 receptors. This difference must be due either to an expansion of family 3, 5 and cAR receptors in *D. discoideum *or to a reduction in the *D. purpureum *genome. Either *D. discoideum *has evolved many new functions for GPCRs compared to *D. purpureum *or else there is more functional overlap amongst the *D. discoideum *receptors.

#### Transcription factors

The overall comparison of transcription factors in *D. discoideum *and *D. purpureum *shows gross conservation both in the total number of genes in each family, and at the protein sequence level (Table S11 in Additional file 1). There are only 11 basic leucine zipper (bZIP) domains in *D. purpureum*, versus 19 in *D. discoideum*. Among the 11 bZIPs found in both species are DimA and DimB, which are involved in DIF signaling in *D. discoideum*, as well as bZIP candidates for CREB and GCN4, which are the most conserved bZIPs among eukaryotes (E. Huang, M. Katoh-Kurasawa and G. Shaulsky; unpublished). There are an equal number of STAT transcription factors in *D. purpureum *and *D. discoideum *(four), each with a high degree of protein sequence identity. In the original description of the *D. discoideum *genome, the paucity of transcription factors was noted [1]. One explanation for the small number of recognized transcription factors was the possibility of new classes of transcription factors that evade conventional detection based on sequence searches. One example is the recently defined CudA nuclear protein that binds *in vivo *to the promoter of the *cotC *prespore gene [50]. CudA-related proteins have recently been defined as being specific to the amoebozoa [51], but there are distantly related proteins in plants [50].

#### The actin cytoskeleton and its regulation

The *D. purpureum *repertoire of microfilament system proteins is almost an exact replica of that described in *D. discoideum *(Table S12 in Additional file 1) [52]. In contrast, the actin-depolymerizing factor (ADF) protein family differs between the *Dictyostelium *species. A phylogenetic tree of all ADF domains encoded by the genomes of both species shows three major groups (Figure S13 in Additional file 1). The ADF domains present in cofilin, twinfilin and GMF (glia maturation factor) constitute one group. *D. purpureum *has two genes encoding cofilins, *cofA *and *cofG*. Only *cofA *has a direct ortholog amongst the eight *D. discoideum *genes. An additional group of ADF domains is present in *D. purpureum *that includes three proteins, one of which (DPU_G0064410) has no direct ortholog in *D. discoideum *and another (DPU_G0060306) that is related to two *D. discoideum *genes (DDB_G0270134 and DDB_G0270132).

A family of proteins where there has been some expansion in *D. purpureum *is that of the I/LWEQ domain-containing proteins. Besides two talins and a single Sla2/HIP1, *D. purpureum *harbors three more genes related to *hipA *encoding only a carboxy-terminal fragment that encompasses the I/LWEQ domain. It is not clear whether these are actually pseudogenes. Similarly, we have found a group of at least eight genes that encode short proteins related to the carboxy-terminal part of HIP1 immediately upstream of the I/LWEQ domain. The extensive family of calponin homology (CH) domain proteins in *D. purpureum *has two members absent in *D. discoideum*. One (DPU_G0069574) is related to conventional fimbrins but lacks EF hands and has a weakly conserved fourth CH domain. The other (DPU_G0074288) is a protein with a carboxy-terminal CH domain.

#### Rho signaling

Cytoskeletal remodeling during chemotaxis and phagocytosis is regulated by a considerable number of upstream signaling components. Especially important are those components involved in signaling to and from small GTPases of the Rho family, as recently described in *D. discoideum *[53]. In general terms the repertoire of genes encoding proteins that participate in Rho signaling is very similar in both dictyostelid species, with some exceptions (Tables S13 and S14 in Additional file 1). The Rho GTPase family itself has diversified considerably in *D. purpureum *and *D. discoideum *(Figure S14 in Additional file 1). This family currently comprises 20 *rac *genes and one pseudogene in *D. discoideum *and 18 genes in *D. purpureum*. Most *D. discoideum rac *genes have a direct ortholog in *D. purpureum*, but the degree of conservation is variable. There is a second *rac1a*-related gene, indicating that the ancestral *rac1 *gene duplicated independently in each organism. There is no ortholog for *D. discoideum rac1b*, *rac1c*, *racF1*, *racF2*, *racI *and *racM *to *racO*, and the pseudogene *racK *and, conversely, *D. purpureum *has five more *rac *genes without a *D. discoideum *counterpart (*racR *to *racW*), again indicating that the *rac *family has undergone independent divergence in both species.

Among the Rho regulators *D. purpureum *appears to have one RhoGAP gene less than *D. discoideum*. The missing RhoGAP gene is *gacII*; the corresponding protein consists of a RhoGAP domain followed by a SH3 domain. The protein is very similar to the amino-terminal half of RacGAP1 (*xacA *gene), suggesting that *gacII *resulted from a partial duplication of *xacA *in *D. discoideum*. Among the Rho effectors, the class PI4P 5 kinases have undergone a notable expansion in *D. purpureum *(Table S14 in Additional file 1). Additional descriptions of Ras superfamily members can be found in Additional file 1.

### The *D. purpureum *glycome

Glycosylation is an extensive post-translational modification of proteins, and also occurs on lipids, nucleic acids and, of course, polysaccharides, in all forms of life. Though basic glycosylation pathways tend to be conserved among eukaryotes, glycosylation details can vary between species and cell types, and even between individual proteins as 'microheterogeneities'. In *D. discoideum*, protein glycosylation has been implicated in protein sorting and stability, cell proliferation, adhesion and sorting, spore coat assembly, resistance to cisplatin, and oxygen signaling. The inventory of predicted glycogenes likely to be associated with both anabolic and catabolic aspects of glycan metabolism approaches 2.5% of the genome (Tables S16, S17, S18, and S19 in Additional file 1), typical for metazoans but lower than for higher plants. As discussed below, a comparison of *D. purpureum *with the previously annotated glycogenes of *D. discoideum *[54], in the context of the global CAZy classification [55,56], suggests examples of both considerable conservation and diversification of their glycomes.

#### N-linked glycosylation

Protein N-glycosylation, the most prevalent and highly conserved type of protein glycosylation, is initiated in the rough endoplasmic reticulum of *D. discoideum *by the transfer of a 14-sugar chain from a lipid-linked precursor [57] that is identical to the yeast and human precursor but distinct from that of many protists [58]. Maturation of the sugar chain leads to a preponderance of high-mannose glycans with bisecting and novel intersecting β-linked GlcNAc, and α3-linked core fucose characteristic of plants and invertebrates, followed by increased α-mannosidase processing during development [59,60]. *D. discoideum *N-glycans are often rendered anionic by phosphorylation and sulfation [57,59], in contrast to the typical sialic acid or uronic acid modifications of animal glycans.

A genomic comparison suggests that the N-glycome of *D. purpureum *will be similar to that of *D. discoideum *but with some interesting differences (Table S16 in Additional file 1). For example, putative CAZy GT49 β3-GlcNAc transferases, GT10 α3/4-fucosyltransferases, and glycophosphotransferases, expected to mediate peripheral modifications of N-linked and perhaps other glycans, are represented by much smaller gene families in *D. purpureum*, and low amino acid sequence similarities make ortholog predictions for individual family members less certain. Thus, *D. purpureum *may exhibit reduced prevalence and diversity of its peripheral glycan modifications.

**Figure 6:**
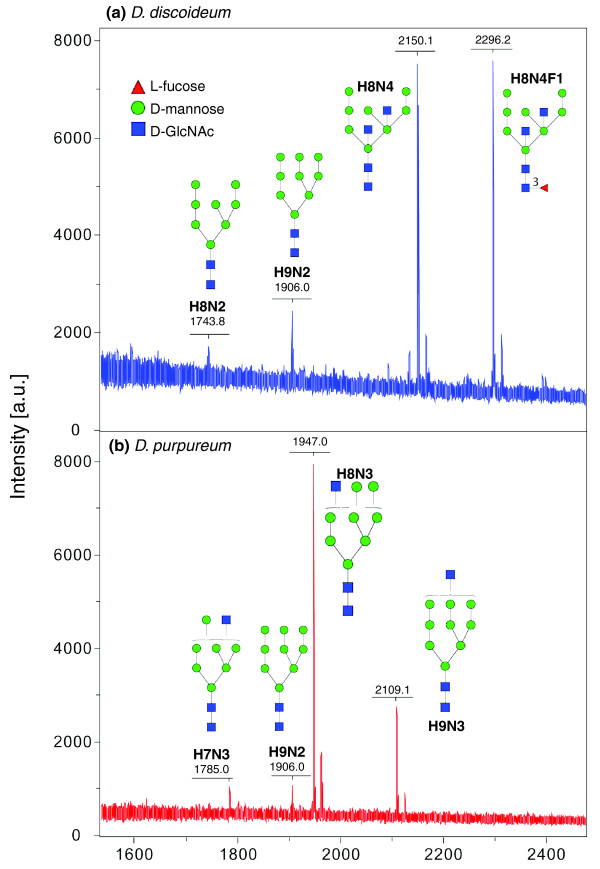
**Comparison of the N-glycomes of *D. purpureum *and *D. discoideum *cells**. Cells were harvested from co-cultures with *Klebsiella aerogenes*, and N-glycans were released from total CHAPS-solubilized, pepsin-digested protein using PNGase A [59,60]. **(a) **Matrix-assisted laser desorption/ionization-time of flight (MALDI-TOF)/TOF mass spectrometry spectrum of underivatized *D. discoideum *N-glycans. **(b) **Corresponding spectrum from *D. purpureum*. Structure assignments are based on glycan compositions derived from m/z values (H = Hex, N = HexNAc, F = Fuc), tandem mass spectrometry analysis, linkage analysis and exoglycosidase digestions. Brackets indicate uncertainties in the positions of peripheral GlcNAc (= N) and mannose (= H) residues. The major ions are [M + Na]^+^; minor [M + K]^+ ^ions are also present. *D. purpureum *N-glycans lack α3-linked core fucose and the fourth peripheral β4-linked GlcNAc consistent with the absence of CAZy GT10 and GT17 genes predicted to encode the glycosyltransferases responsible for these peripheral modifications in *D. discoideum *(Table S16 in Additional file 1). A.u., arbitrary units.

The most dramatic predicted difference between the two dictyostelid glycomes stems from the apparent absence in *D. purpureum *of the four-member CAZy GT17 class of GT-like proteins expected to mediate addition of peripheral bisecting and/or intersecting β4-GlcNAc residues. We tested this by performing a matrix-assisted laser desorption/ionization-time of flight (MALDI-TOF) mass spectrometry glycomic analysis, which confirmed the presence in *D. discoideum *of N-glycans containing two peripheral GlcNAc residues and/or an α3-linked core fucose, and revealed an apparent absence of these species in *D. purpureum *(Figure 6). The results suggest that CAZy family GT17 and GT10 sequences present in *D. discoideum *but absent from *D. purpureum *encode a novel N-glycan β-GlcNAc transferase and a core α3-fucosyltransferase, respectively, emphasizing the value of comparative genomics for predicting gene functions. Other studies have indicated that N-glycans are dominant contributors to the cell surface glycocalyx, and therefore may strongly influence intra- and inter-specific encounters with other amoebae, and interactions with potential predators, pathogens and prey. Thus, the dramatically different N-glycomes of these species might contribute to, for example, their differential sorting in interspecific mixtures [61].

#### Other glycosylation events associated with the secretory pathway

A previous inspection of the predicted *D. discoideum *proteome also indicated the existence of some major classes of biosynthetic enzymes associated with mucin-type O-glycans, O-phosphoglycans, and glycosylphosphatidylinisotol (GPI) anchors [54], in agreement with biochemical studies [57]. For example, mucin-type O-glycosylation is initiated in the Golgi by a CAZy GT60 polypeptide α-GlcNAc transferase, conserved in both dictyostelids and related to the polypeptide α-GalNAc transferases associated with mucin-type O-glycosylation in animals [62]. Glycophosphorylation of the hydroxyamino acids threonine and serine may be less prevalent in *D. purpureum *owing to the much smaller size of its glycophosphotransferase-like gene family (Table S16). Although the glycogene comparison suggests a general conservation of these other aspects of the glycome, differences suggest that there may be equally dramatic variations as observed for N-glycosylation.

#### Cytoplasmic glycome

Whereas glycosylation occurs predominantly in the secretory compartments, formation of the precursors for these pathways generally originates in the cytoplasm, and the cytoplasm is also a site for catabolic deglycosylation. The genome encodes proteins associated with these functions as expected. In addition, like most eukaryotes, *D. discoideum *encodes a potential nucleo-cytoplasmic Spy-like GT41 O-GlcNAc transferase (OGT or Ser/Thr-βGlcNAc transferase) and *D. purpureum *encodes two; the physiological function(s) of O-GlcNAc in protists is currently unknown [63]. *D. discoideum *also possesses a complex cytoplasmic O-glycosylation pathway that modifies hydroxyproline and has an ancient evolutionary relationship with O-glycosylation in the secretory pathway and bacterial glycosylation [64]. The genes of this pathway are highly conserved in *D. purpureum*, and bioinformatics and biochemical data indicate its partial conservation across at least four major protist phyla. This pathway is devoted to the modification of the E3 ubiquitin ligase subunit Skp1, and is involved in oxygen regulation of development in *D. discoideum *[65].

#### Carbohydrate binding proteins

Many glycan functions are mediated *in trans *via carbohydrate binding domains (CBDs) or lectins. During initial remodeling within the rough endoplasmic reticulum, N-glycans are recognized by lectins in a folding/quality control cycle and, unlike many protists, this pathway appears to be highly conserved between the dictyostelids and animals [66]. *D. discoideum *encodes numerous cytoplasmically localized lectins, including multiple discoidin, Cup and comitin proteins [67-69], and glycogen-binding proteins involved in metabolic regulation (Tables S18 and S19 in Additional file 1). Except for the latter, the natural glycan ligands in the cytoplasm are unknown. Interestingly, discoidins, like galectins of animals, exit cells via a non-classical process and potentially bind self, prey or predator glycans containing Gal or GalNAc [70]. Discoidin and Cup CBDs appear to be dictyostelid-specific and evolutionarily dynamic, suggesting they serve species-specific functions as suggested for other lineage-specific expansions [71].

#### Carbohydrate catabolism

Both genomes encode a few more glycohydrolases (Table S16 in Additional file 1) than glycosyltransferases, with suspected substrates ranging from dietary polysaccharides and glycans of bacterial, yeast and perhaps other prey to endogenous glycans for recycling. Potentially 11 of the glycohydrolase domains are fused to carbohydrate binding modules (CBMs), a subset of CBDs associated with enzymes. As described for some cellulases such as CelA, the CBM may localize the enzyme to the target substrate after secretion, and may also directly promote catalysis [72]. Peptidases may be localized at the cell surface by a similar mechanism. Cellulases are likely to be involved in remodeling of slime sheath cellulose during morphogenesis and spore coat breakdown during germination. *D. purpureum *and *D. discoideum *also have a cellulase associated with extracellular digestion in fungi and other cellulose-digesting organisms (CAZy GH7), suggesting a similar role in the social amoebae. Though the number of glycosyltransferases and known glycan binding proteins is 10 to 20% smaller in *D. purpureum *than *D. discoideum*, correlating with fewer peripheral modifications, the number of potential glycohydrolases is approximately 10% greater. The latter differences occur in lysozyme-, chitinase-, and alpha-mannosidase-like enzymes, suggesting variation in the spectrum of bacterial and yeast prey between the species.

### Multicellular development and dictyostelid sociality

The dictyostelid social amoebae undergo multicellular development when nutrients become limiting for vegetative growth. The ensuing events of aggregation of individual cells into an initial mound, slug migration, and ultimately fruiting body morphogenesis, require several cooperative interactions between the cells. These cooperative cellular behaviors include: cellular chemotaxis to self-generated, field-wide spiral waves of extracellular cAMP; the coordinated movements of cells within specialized tissues of the mounds and slugs requiring differential cell adhesion; an innate immune system; and the apparent altruism displayed by the pre-stalk cells that die as they construct the stalk, presumably to aid the dispersal of the spores in the sorus. The initial analyses of the *D. discoideum *genome uncovered a number of protein classes that might mediate this extensive cellular cooperation, and that were previously thought to be unique to metazoa [1]. These proteins included certain subfamilies of ABC transporters, metabotropic GPCRs, and cell surface proteins predicted to contain repeated epidermal growth factor (EGF) or Ig-like domains that had not previously been seen in plants, fungi or amoebae.

One large family of 37 metazoan-like proteins described in *D. discoideum*, the Tiger (transmembrane, IPT, Ig-like, E-SET repeat) proteins, contain family members that mediate cell-cell interactions during development. Mutations in *tgrB1*, *tgrC1 *(formerly *lagC*), *tgrD1 *and *tgrE1 *all result in the arrest of development at the mound stage, and TgrB1 and TgrC1 have been implicated in a self/non-self recognition system that may mediate kin recognition [73]. Twenty-six Tiger-protein encoding genes are present in the *D. purpureum *genome, including orthologs to *D. discoideum*'s *tgrC1*, *tgrD1*, *tgrF1*, *tgrK1*, *tgrM2*, and *tgrN1 *genes. Their presence suggests that the Tiger protein family may be generally involved in allorecognition in the dictyostelids.

An innate immune system has been recently described that functions during slug migration of *D. discoideum *and appears to be present in other group 4 dictyostelids, including *D. purpureum *[74]. It consists of a population of sentinel cells that patrol the body of the slug, engulf any bacteria that are present, bind to the slime sheath, and then exit the slug by being left behind in the slime trail. Sentinel cells are 1% of all slug cells and express particular genes that are related to innate immunity signaling genes in plants and animals, such as *slrA *and *tirA*. In particular, *tirA*, a Toll/interleukin receptor I domain containing protein, is required for some aspects of sentinel cell function [74]. *D. purpureum *has orthologs for both *slrA *and *tirA*, as well as three amoeba-specific lysozymes (Additional file 4).

#### Cyclic nucleotide signalling genes

cAMP controls many aspects of dictyostelid development. As a dynamically secreted chemoattractant it directs the cell movement that causes cells to aggregate, and aggregates to transform into fruiting structures. Secreted cAMP also triggers pre-spore differentiation, up-regulates the expression of aggregation genes and down-regulates stalk gene expression. As an intracellular messenger for other stimuli, cAMP induces spore and stalk encapsulation and maintains spore dormancy [23,46,75,76].

**Figure 7:**
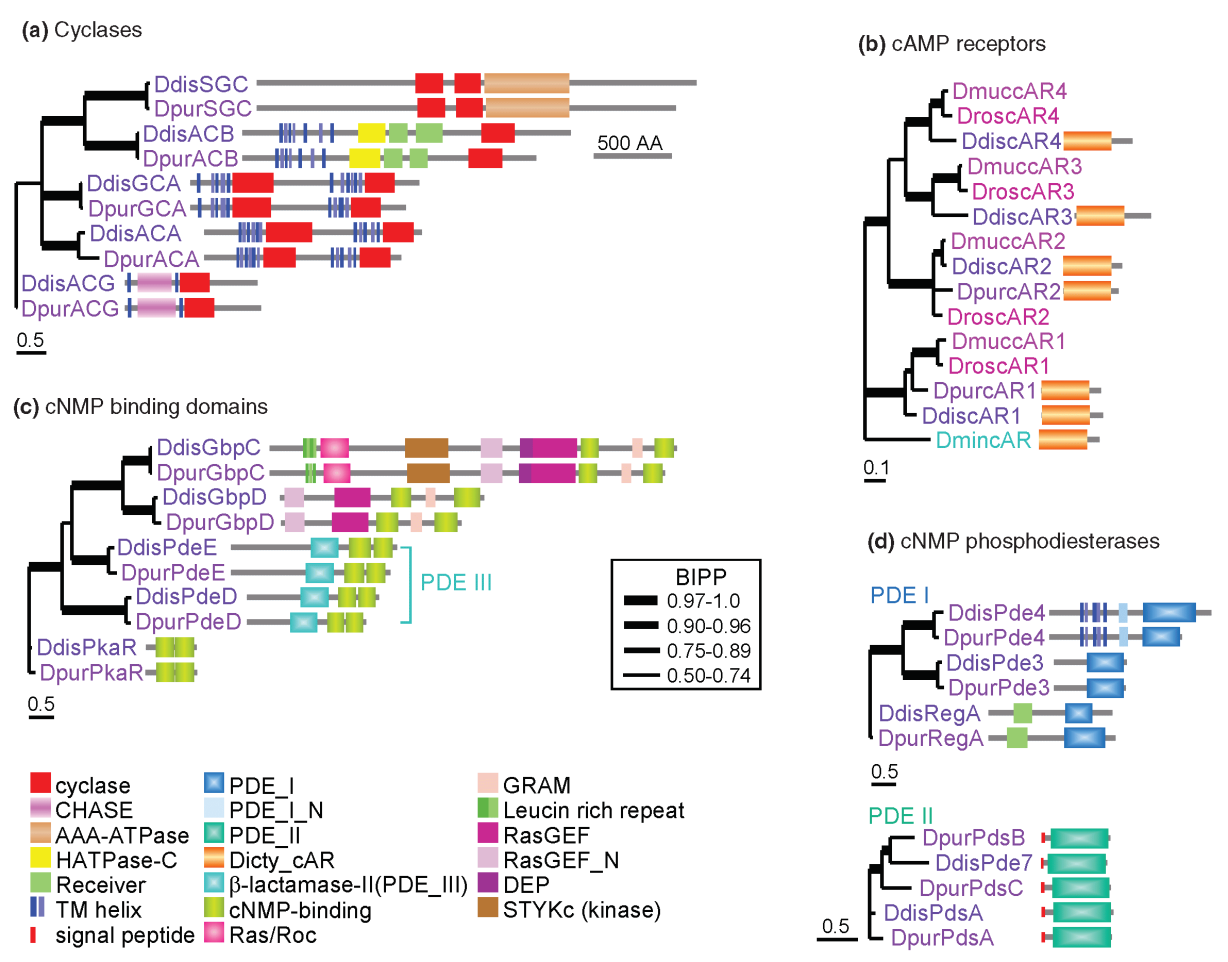
**Architectural conservation of cyclic nucleotide signaling genes**. Deduced sequences of *D. discoideum *(*Ddis*) and *D. purpureum *(*Dpur*) proteins were analyzed by SMART [105] for the presence of functional domains, signal peptides and transmembrane helices. To build protein phylogenies, conserved shared functional domains were aligned using CLUSTAL-W [106] and edited when necessary in BioEdit [107] to juxtapose functionally essential amino acid residues. Regions that did not align unambiguously were deleted. For proteins with two similar domains (cyclases and cyclic nucleotide (cNMP) binding proteins), a tandem alignment of both domains was used, with the single domains of ACB and ACG used twice. Phylogenetic relationships between aligned sequences were determined by Bayesian inference [108] using a mixed amino acid model. Rate variation between sites was estimated by a gamma distribution with a proportion of invariable sites. Analyses were run for 100,000 generations or until the standard deviation of split frequences was <0.01. The phylogenetic trees are decorated with the domain architectures of the proteins, except for the *D. mucoroides *(*Dmuc*) and *D. rosarium *(*Dros*) cAMP receptor (cAR) sequences, which were derived from genes that were only partially amplified by PCR [79]. All trees are unrooted, except for the cAR tree, which is rooted on the single cAR of the group 3 taxon *Dictyostelium minutum *(*Dmin*). The posterior probabilities (BIPP) of nodes are represented by line thickness. **(a) **Cyclases; **(b) **cAMP receptors; **(c) **cNMP binding domains; **(d) **cNMP phosphodiesterases. *Dpur *protein IDs and, if available, dictyBase IDs: SGC, 153022 (DPU_G0054494); ACB, 154751 (DPU_G0058520); GCA, 151484 (DPU_G0075774); ACA, 51614 (DPU_G0071214); ACG, 38950 (DPU_G0061484); GbpC, 168746; GbpD, 88426 (DPU_G0055716); PdeD, 98774 (DPU_G0059600); PdeE, 56777 (DPU_G0059268); PkaR, 157660 (DPU_G0065616); cAR1, 99295 (DPU_G0064058); cAR2, 92050 (DPU_G0053090); Pde3, 34050 (DPU_G0053756); Pde4, 150656 (DPU_G0073898); PdsA, 98685 (DPU_G0058978); PdsB, 168741 (DPU_G0056384); PdsC, 91767 (DPU_G0073930). GenBank accession numbers for *Ddis *sequences: ACB, [GenBank:AAD50121]; GCA, [GenBank:CAB42641]; ACA, [GenBank:AAA33163]; ACG, [GenBank:Q03101]; GbpC, [GenBank:AAM34041]; GbpD, [GenBank:AAM34042]; PdeD, [GenBank:AAL06059]; PdeE, [GenBank:AAL06060]; PkaR, [GenBank:P05987]; cAR1, [GenBank:AAA33177]; cAR2, [GenBank:AAB25436]; cAR3, [GenBank:AAB25437]; cAR4, [GenBank:AAB32419]; Pde3, [GenBank:B0G0Y8]; Pde4, [GenBank:AAO59486]; PdsA, [GenBank:XP_637948]; Pde7, [GenBank:EAL62880]. GenBank accession numbers for *Dmuc *sequences: cAR1, [GenBank:ACF17575]; cAR2, [GenBank:ACF17576]; cAR3, [GenBank:ACF17577]; cAR4, [GenBank:ACF17578]. GenBank accession numbers for *Dros *sequences: cAR1, [GenBank:AAW24476]; cAR2, [GenBank:AAW24477]; cAR3, [GenBank:ACF17573]; cAR4, [GenBank:ACF17574]. GenBank accession number for *Dmin *cAR, [GenBank:AAS59250].

In *D. discoideum*, 19 proteins are directly responsible for synthesis, detection and degradation of cAMP and its sister molecule, cGMP, which acts as a signaling intermediate for chemotaxis [77]. To assess whether cyclic nucleotides play similar roles in *D. purpureum *development, we analyzed conservation and change in all genes that are directly involved in cyclic nucleotide signaling. *D. discoideum *uses the adenylate cyclases ACA, ACB and ACG and the guanylate cyclases sGC and GCA for synthesis of cAMP and cGMP, respectively [76,78]. All five cyclases are present in *D. purpureum *inclusive of their functional domain architecture (Figure 7a). The structurally distinct cell surface cAMP receptors (cARs) and intracellular cyclic nucleotide (cNMP) binding domains are the sole targets for cyclic nucleotides in dictyostelids. *D. discoideum *has four cARs, which are conserved in two other group 4 taxa, *D. mucoroides *and *Dictyostelium rosarium *[79]. Nonetheless, only the *cAR1 *and *cAR2 *genes were detected in the *D. purpureum *genome (Figure 7b). No firm conclusions about the absence of *cAR3 *and *cAR4 *can yet be drawn, since the assembly of this genome is not fully complete.

The cNMP binding domains are found in the regulatory subunit of PKA (PkaR), the cGMP binding proteins GbpC and GbpD and the phosphodiesterases (PDEs) PdeD and PdeE. PdeD is a cGMP phosphodiesterase that is stimulated by cGMP binding to its cNMP binding domains, while PdeE is a cAMP-stimulated cAMP phosphodiesterase [76,80]. GbpC is a complex multidomain protein in which cGMP binding to its cNMP binding domains sequentially activates the intrinsic RasGEF, Ras/Roc and protein kinase domain, which eventually leads to increased cell polarization. GbpD also contains a RasGEF domain, but no output protein kinase domain. Its cNMP binding domains are not functional and it functions as an antagonist of GbpC in the chemotactic response [81,82]. Genes encoding all five cNMP binding proteins with their complete sets of functional domains are present in the *D. purpureum *genome (Figure 7c).

In *D. discoideum *cyclic nucleotides are hydrolyzed by three structurally distinct PDEs [80]. The cAMP PDEs RegA and Pde4 and the cGMP PDE Pde3 harbor a PDE_I type domain with HDc motif that is common to mammalian PDEs. The dual-specificity PDEs PdsA and PDE7 harbor a PDE_II type domain with HSHLDH motif. PdeD and PdeE, which hydrolyze cGMP and cAMP, respectively, carry a related HCHADHDS motif, but are structurally more similar to the lactamase_B protein family. The PDE_I and PDE_III enzymes are fully conserved between *D. discoideum *and *D. purpureum*, but the latter species has three instead of two type II PDEs (Figure 7d).

The high level of conservation between *D. discoideum *and *D. purpureum *of all adenylate and guanylate cyclases, cNMP binding domains and seven out of eight PDEs, combined with the complete conservation of functional domain architecture of these proteins, is indicative of the central roles of cAMP and cGMP in the control of chemotaxis, morphogenesis and gene regulation in the dictyostelids.

#### DIF signaling

DIF is produced predominantly by pre-spore cells during *D. discoideum *development and is part of a signaling mechanism that sets the ratio of stalk and spore cells produced in the fruiting body. It both limits the number of pre-spore cells produced and induces differentiation of a subset of pre-stalk cells. DIF is made by a three step biosynthetic pathway, in which a 12-carbon polyketide is assembled by the StlB polyketide synthase, then successively chlorinated by a chlorinating enzyme, and methylated by the DmtA methyltransferase [36,83,84]. Clear *stlB *and *dmtA *homologs exist in the *D. purpureum *genome, as does a homologue of a recently identified FAD-dependent chlorinating enzyme (C Neumann, C Walsh and RR Kay, unpublished). DIF is inactivated by glutathione-dependent dechlorination [85], and again this enzyme has recently been identified and has a clear homolog in *D. purpureum *(F Velazquez and RR Kay, unpublished). It thus appears certain that *D. purpureum *makes and degrades DIF in a similar way to *D. discoideum*, and presumably utilizes it in a similar way to regulate multicellular development.

#### Social genes

Dictyostelids are interesting social organisms because about 20% of the cells in each fruiting body sacrifice themselves to build the stalk. Groups form through aggregation of formerly separate cells, so different clones can aggregate together, and do so in both the lab and in the field [86]. Clones that successfully compete to get into spores, relegating their partners to the sterile stalk, will be more successful. Some degree of conflict is therefore predicted, and the resulting evolution of strategies and counter strategies may drive rapid adaptive evolution, as appears to be true for genes involved in host-parasite conflicts and male-female conflicts [87]. But there is also a second reason to expect that social genes may evolve more rapidly than genes expressed primarily in the solitary stage. If the social stage occurs relatively infrequently, which seems likely but is unknown, then social genes are less scrutinized by selection and could accumulate more changes through genetic drift.

We tested for more rapid evolution using two ways of defining social genes. The first was to examine the set of genes that emerged from a selection for mutants that cheat (make more than their fair share of spores in mixtures) and compare them with all other genes [41]. The two sets do not differ significantly in the probability of having homologs, suggesting that they neither differentially disappear nor differentially evolve beyond the point of clear homology (Figure S18a in Additional file 1). The two sets also do not differ in probability of having paralogs, suggesting that they do not duplicate at different rates (Figure S18b in Additional file 1). Finally, the two sets do not differ for either dN (the rate of non-synonymous change) or conservation score (a measure that declines with both point differences and with non-aligned portions of the sequences) (Figures S18c and S19d in Additional file 1). However, this set of social genes is relatively small, and some will be false positives (of 198 genes identified, 40 were tested for cheating, of which 31 were cheaters).

A larger set of social genes can be identified using RNA-seq reads from the vegetative stage and six social time points (4, 8, 12, 16, 20, and 24 hours after starving) [15]. Using genes with sufficient reads and high reproducibility (Additional file 1), we defined a gene's index of social expression as the average percentage representation in the social-stage libraries over that average plus the percentage representation in vegetative stage (that is, Social expression/Social expression + Vegetative expression).

Using this classification, social genes showed higher rates of change, and manifested fewer orthologs, higher rates of non-synonymous substitution, and lower conservation scores. Genes with orthologs in *D. discoideum *and *D. purpureum *have a significantly lower social expression index in *D. discoideum *than those without orthologs (Figure S19a in Additional file 1; *n *= 1,739, 1,300, *P *< 2.2e-16, Mann-Whitney U test). This is driven by significant differences in each time point of the developmental stages (data not shown). An analysis using genes with *D. purpureum *RNA-seq data following the above criteria gives a similar overall result (Figure S19b in Additional file 1; *n *= 3,649, 2,102, *P *< 2.2e-16, Mann-Whitney U test). Thus, genes with more social expression are less likely to have orthologs, indicating more rapid evolution in the gain or loss of genes, or in change of genes beyond the point where they are identifiable as homologs. Homologs that had inparalogs show no significant difference of social indices from those that lacked inparalogs when the social expression is measured with RNA-seq reads from *D. discoideum *(Figure S19c in Additional file 1; *n *= 95, 1,644, *P *= 0.93, Mann-Whitney U test) and *D. purpureum *(Figure S19d in Additional file 1; *n *= 137, 3,512, *P *= 0.24, Mann-Whitney U test), suggesting no significant bias of duplication for the social genes of dictyostelids.

**Figure 8:**
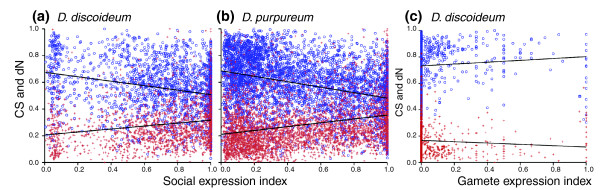
**Conservation score (CS, blue open circles) and non-synonymous substitution rate (dN, red crosses) as a function of the degree of a gene's expression in social versus vegetative stages (a,b) or of sexual versus vegetative stages (c)**. **(a) **For *D. discoideum *RNA-seq reads (1,739 genes) both regressions are significant (CS, y = -0.17x + 0.68, R^2 ^= 0.063, *P *< 0.0001; dN, y = 0.11x + 0.21, R^2 ^= 0.032, *P *< 0.0001). **(b) **For *D. purpureum *RNA-seq reads (3,649 genes), both regressions are also significant (CS, y = -0.20x + 0.69, R^2 ^= 0.11, *P *< 0.0001; dN, y = 0.14x + 0.21, R^2 ^= 0.017, *P *< 0.0001). **(c) **Conservation score and non-synonymous substitution rate as a function of the percentage of *D. discoideum *ESTs expressed in the gamete stage (932 genes, including 835 and 16 genes with a gamete expression index of 0% and 100%, respectively). Both regressions are significant: CS, y = -0.0070x + 0.73, R^2 ^= 0.0072, *P *< 0.01; dN, y = 0.00051x + 0.17, R^2 ^= 0.00708, *P *= 0.01.

Figure 8a plots conservation score and rates of non-synonymous substitution (dN) as a function of the *D. discoideum *social expression index. As the percentage of a gene's RNA-seq reads found in social stages increases, there is a significant drop in conservation score and a significant increase in dN, supporting the hypothesis that social genes change more rapidly than vegetative ones. The same significant results hold if we use the RNA-seq reads of *D. purpureum *genes (Figure 8b). Figure S20 in Additional file 1 shows how the effect is partitioned between different social stages. Previous studies have suggested that individual social genes or small sets of them evolve rapidly because of evolutionary arms races, with conflict driving continuing adaptation and counter-adaptation [88]. This is the first such evidence on a genomic scale. Nonetheless, we cannot rule out the alternative hypotheses that the lower selective scrutiny of social genes might arise if the social stage is not very frequent and not as selectively important as the vegetative stage. Distinguishing these hypotheses further will have to await the more sensitive tests that can be applied to genomes that are more closely related than *D. discoideum *and *D. purpureum*.

*Dictyostelium *has a sexual cycle in which two cells fuse and then engulf many other cells to form a giant macrocyst that undergoes meiosis. However, with the exception of one successful cross [89], the sexual system has not been available in lab studies of *D. discoideum*. Although macrocysts are readily formed, there are problems with germination [90] and, when there is germination, there may be no recombinants [91]. Finding the right conditions for sex would add a valuable genetic dimension to *D. discoideum *studies, but this search would be fruitless if most strains have lost the ability to have sex. If they have lost this ability, we would expect that sex-specific genes would have degraded. We tested this hypothesis using ESTs from gamete-stage libraries made from cells grown in conditions that make them competent for fusion [92]. Figure 8c shows that genes expressed disproportionately in the gamete stage are actually more conserved than other genes, as measured by both dN and conservation score. Provided these truly are sex-specific genes, then it appears that the macrocyst system is functional and not degenerating in *D. discoideum*. This is supported by an analysis showing that 13 meiosis genes [93] have normal dN and conservation score values compared to other genes (Figure S21 in Additional file 1).

## Conclusions

Comparisons of the *D. purpureum *genome, the second group 4 dictyostelid to be sequenced, with the previously sequenced *D. discoideum *have provided insights into the evolution of this clade of social amoebae. Like *D. discoideum*, the genome of *D. purpureum *encodes a high number of triplet nucleotide repeats distributed in both exonic and non-protein-coding regions. However, these tracts are not generally congruent between the two genomes, indicating that their expansion is a consequence of an intrinsic physiology favoring high rates of triplet repeat formation, rather than retention and accumulation of ancient triplet repeats. Although the *D. purpureum *genome was not finished to the same extent as the *D. discoideum *genome, syntenic regions containing orthologous genes are detected. Genes that have been hypothesized as having been acquired through horizontal gene transfer in the *D. discoideum *genome have orthologs in the *D. purpureum *genome; thus, any HGT events involving these genes likely occurred in the common ancestor of the group 4 dictyostelids. Likewise, large gene families of ABC transporters and histidine kinases underwent expansion in the common ancestor before the species line split, while the expansion of polyketide synthase genes occurred in a lineage-specific manner. The repertoire of microfilament system proteins is virtually identical between the two species, but the regulatory proteins differ. Two distinct transferases involved in the N-linked glycosylation were detected in *D. discoideum *but not in *D. purpureum*, and the predicted change in the glycosylation state of the respective species proteins was validated through glycomic analysis. Comparative analyses also enabled the identification of two novel classes of ncRNAs specific to the dictyostelid lineage. High conservation of enzymes involved in cNMP metabolism and DIF production and degradation indicate the central role these signaling systems play in the social behavior of these amoebozoa. A detailed comparison of the variation between cohorts of genes with specific expression patterns between the two genomes demonstrate that genes involved in sociality evolve more rapidly, probably due to continuous adaptation and counter-adaptation.

## Materials and methods

### Sequence and assembly

*D. purpureum *was described in 1902 by Olive [94]. *D. purpureum *isolate QSDP1 from the Queller and Strassmann laboratories at Rice University, and its axenic derivative DpAX1, were used in this study. DpAX1 was selected from QSDP1 for the ability to grow axenically, in defined liquid media, by culturing in plastic Petri dishes containing HL5 medium supplemented with 10% fetal bovine serum [95].

QSDP1 was used for EST production and sequencing. Cells were grown in association with *Klebsiella pneumoniae*, harvested and developed on nitrocellulose filters as described [95]. RNA samples were prepared from developing cells at 0, 6, 12, and 18 hours [45]. Two cDNA libraries were prepared from each of these four RNA samples and a total of 14,949 validated EST clones were sequenced from them. Briefly, polyA-selected RNA was reverse transcribed with superscript reverse transcriptase III (Invitrogen, Carlsbad, CA, USA) using dT primer (5' GACTAGTTCTAGATCGCGAG CGGCCGCCCTTTTTTTTTTTTTTTVN-3'). cDNA was synthesized with *Escherichia coli *DNA polymerase I, *E. coli *DNA ligase, and *E. coli *RNaseH. The DNA ends were repaired with T4 DNA polymerase. SalI adapters (5'-TCGACCCACGCGTCCG-3' and 5'-P0_4_-CGGACGCGTGGG-3') were ligated to cDNA and the product was digested with NotI. The cDNA digestion products were gel purified and directionally ligated into SalI- and NotI-digested pCMVsport6 and transformed into ElectroMAX, T1 DH10B *E. coli *cells (Invitrogen). Plasmid DNA was amplified by a rolling circle method (Templiphi, GE Healthcare, Piscataway, NJ, USA) and purified. The insert of each clone was sequenced from both ends with primers complementary to flanking vector sequences using Big Dye terminator chemistry and resolved by an ABI 3730 sequenator (ABI, Foster City, CA, USA).

To prepare high quality genomic DNA, DpAX1 cells were grown in shaking cultures in HL5 medium, and DNA was prepared from isolated nuclei by cesium chloride equilibrium density gradients. Genomic libraries were constructed by shearing genomic DNA with a Hydroshear (Genomic Solutions Inc., Ann Arbor, MI, USA) to create 6- to 10-kb fragments. The DNA fragments were size selected, purified, blunt-end repaired (End-It Kit, Epicentre Biotechnologies Madison, WI, USA) and ligated into a pMCL200 vector. The ligation product was purified and precipitated, and then transformed into ElectroMax DH10B competent cells (Invitrogen). The percentage of no-insert clones in the library was assessed by colony PCR, using primers flanking the cloning site (Expand long Template PCR system, Roche Applied Science, Indianapolis, IND, USA). Genomic DNA libraries with average insert sizes of 2.3 to 3.0 kb and 3 to 4 kb were produced by similar methods.

Primary sequence data were derived from whole-genome shotgun sequencing of the three plasmid libraries [96]. The reads were screened for vector sequence with cross_match [97] and trimmed for vector and low quality sequences. Reads shorter than 100 bases after trimming were excluded from the assembly. The trimmed read sequence data were assembled with release 1.0.3 of Jazz, a whole genome shotgun assembler [98]. The assembly was next filtered for redundant scaffolds that matched larger scaffolds (<5 kb length where >80% matched a scaffold of >5 kb length). Finally, scaffolds that showed homology to prokaryotic and non-cellular contaminants (viroids and viruses) were identified and removed. The filtered assembly contains 799 scaffolds, comprising 33.0 Mb, with an estimated sequence coverage of 8.41 × (Additional file 1). The data were deposited in GenBank under project ID 30991 [GenBank:ADID00000000].

### The JGI genome annotation pipeline

For genome annotation we use the JGI annotation pipeline, which combines several gene prediction, annotation and analysis tools. First, the genome assembly is masked using RepeatMasker and a custom repeat library. Next, available ESTs and full-length cDNAs are clustered and aligned to the scaffolds with BLAT. Model organism protein sequences from the non-redundant set of proteins from the National Center for Biotechnology Information (GenBank) are aligned to the scaffolds with BLASTX [22]. Gene models and associated transcripts/proteins are predicted or mapped using (i) data from putative full-length cDNAs derived from available mRNA, ESTs and EST clusters, (ii) homology-based methods Genewise and Fgenesh+, and (iii) *ab initio *method Fgenesh trained on putative full-length genes (see above), manually curated genes (if available), and reliable homology-based models. Additional gene models generated externally with other gene predictors trained for a particular genome can be added as well. The clustered ESTs/cDNAs are used to extend and correct predicted gene models where the exons overlap and splice junctions are not consistent in comparing EST sequences to gene models. This often adds 5' and/or 3' UTRs to the models. With gene structure in place, function is assigned to models based on Smith-Waterman homology to annotated genes from nr, KEGG, and KOG databases. InterproScan is used to identify predicted domains and the Gene Ontology is used to identify function and/or subcellular location. SignalP is used to assist with identification of secreted proteins. Since multiple models with overlapping sequences are generated for each locus, a single model is chosen to produce a non-redundant set of genes. Model selection is based on homology to known proteins from other organisms, EST support, as well as protein and transcript completeness (that is, inclusion of 5' methionine, 3' stop codon, and UTRs). This automatically generated set was further refined by manual curation and submitted to GenBank. Whole genome analysis is performed on the non-redundant set of gene models or a snapshot of a manually curated gene catalog assuming the latter includes significant number of changes compared to the automatically generated non-redundant set.

## Abbreviations

ABC: ATP-binding cassette; ADF: actin-depolymerizing factor; bp: base pair; bZIP: basic leucine zipper; cAR: cAMP receptor; CBD: carbohydrate binding domain; CBM: carbohydrate binding module; CH: calponin homology; cNMP: cyclic nucleoside monophosphate; DIF: differentiation-inducing factor; DUSE: *Dictyostelium *upstream sequence element; EST: expressed sequence tag; GPCR: G-protein coupled receptor; HGT: horizontal gene transfer; ncRNA: non-coding RNA; NSBR: non self-binding region; PDE: phosphodiesterase; PKA: cAMP dependent protein kinase; PKS: polyketide synthase; UTR: untranslated region.

## Competing interests

The authors declare that they have no competing interests.

## Authors' contributions

MK-K and GS derived the DpAx1 strain and purified the nucleic acids used in the project; Alan K, ED, HT, KB, EL, HS, DB, JS, and AS produced the primary DNA sequence, genome assembly, and gene annotation within the JGI pipeline; PF, PG, SB, YB and RLC carried out annotation and data accessibility at dictyBase; CA, MMB, XT, WS, AP, CLF, HvdW EH, CMW, WFL, GS, CD, PMC, TS, ME, PS, RRK, BH, LE, FR, and GS were involved in the analysis of the data and drafted sections of the paper; AP carried out the ortholog and paralog predictions; WS annotated the non-coding RNAs; NHP carried out the phylogeny and synteny analyses; CLF, HvdW, PMC, BH, and CMW carried out the glycoprotein analyses; JES, DCQ, RS, Adam K, and IVG provided overall project management; and Adam K assembled and edited the manuscript.

## Supplementary Material

Additional file 1 **Supplementary text, figures and tables**. Supplementary text, figures and tables that include many details of the genome annotation. Click here for file

Additional file 2 **Supplementary Table S2**. A table listing blocks of partially conserved gene order between the *D. discoideum *and *D. purpureum *genomes. Click here for file

Additional file 3 **Supplementary Table S4**. A table listing the predicted orthologs that are shared between *D. discoideum *and *D. purpureum*. Click here for file

Additional file 4 **Supplementary Tables S5**. A table listing the predicted paralogs that are shared between *D. discoideum *and *D. purpureum*. Click here for file
