# Supplementary material for: Comparative genomics of the social amoebae Dictyostelium discoideum and Dictyostelium purpureum
Source: Genome Biol. 2011 Feb 28;12(2):R20. doi: 10.1186/gb-2011-12-2-r20 (PMC3188802; doi:10.1186/gb-2011-12-2-r20)
Supplement: Additional file 1 — Supplementary text, figures and tables. Supplementary text, figures and tables that include many details of the genome annotation. [file gb-2011-12-2-r20-S1.DOC]

Additional data File 1.

Supplemental Material for:

Comparative genomics of the social amoebae *Dictyostelium discoideum* and *Dictyostelium purpureum.*

Richard Sucgang^1*^, Alan Kuo^2*^, Xiangjun Tian^3*^, William Salerno^1*^, Anup Parikh^4^, Christa L. Feasley^5^, Eileen Dalin^2^, Hank Tu^2^, Eryong Huang^4^, Kerrie Barry^2^, Erika Lindquist^2^, Harris Shapiro^2^, David Bruce^2^, Jeremy Schmutz^2^, Asaf Salamov^2^, Petra Fey^6^, Pascale Gaudet^6^, Christophe Anjard^7^, Madan Babu Mohan^8^, Siddhartha Basu^6^, Yulia Bushmanova^6^, Hanke van der Wel^5^, Mariko Katoh-Kurasawa ^4^, Christopher Dinh^1^, Pedro M. Coutinho^9^, Tamao Saito^10^, Marek Elias^11^, Pauline Schaap^12^, Robert R. Kay^8^, Bernard Henrissat^9^, Ludwig Eichinger^13^, Francisco Rivero^14^, Nicholas H. Putnam^3^, Christopher M. West^5^, William F. Loomis^7^, Rex L. Chisholm^6^, Gad Shaulsky^3,4^, Joan E. Strassmann^3^, David C. Queller^3^, Adam Kuspa^1,3,4, §^, and Igor V. Grigoriev^2^

1 – Verna and Marrs McLean Department of Biochemistry and Molecular Biology, Baylor College of Medicine, Houston, TX 77030; 2 – Joint Genome Institute, Department of Energy, Walnut Creek CA; 3 – Department of Ecology and Evolutionary Biology, Rice University, Houston, TX 77005; 4 – Department of Molecular and Human Genetics, Baylor College of Medicine, Houston, TX 77030; 5 - Department of Biochemistry & Molecular Biology, Oklahoma Center for Medical Glycobiology, University of Oklahoma Health Sciences Center, Oklahoma City, OK 73104; 6 – dictyBase, Center for Genetic Medicine, Northwestern University, 750 N Lake Shore Drive, Chicago, Illinois 60611; 7 - Section of Cell and Developmental Biology, Division of Biology, University of California, San Diego, La Jolla, California 92093; 8 - Laboratory of Molecular Biology, MRC Centre, Cambridge CB2 2QH, UK; 9 - Architecture et Fonction des Macromolécules Biologiques, UMR6098, CNRS, Universities of Aix-Marseille I & II, 13288 Marseille, France; 10 – Department of Materials and Life Sciences, Sophia University 7-1 Kioi-Cho, Chiyoda-Ku, Tokyo, Japan 102-8554; 11- Departments of Botany and Parasitology, Faculty of Science, Charles University in Prague, Prague, Czech Republic; 12- College of Life Sciences, University of Dundee, DD15EH Dundee, UK; 13 - Center for Molecular Medicine Cologne, University of Cologne, Joseph-Stelzmann-Str. 52, 50931 Cologne, Germany; 14- Centre for Biomedical Research, The Hull York Medical School and Department of Biological Sciences, University of Hull, Hull HU6 7RX, UK.

* Equal Contributors

^§^ Corresponding author

**Description of the genome**

The initial annotation of the 799 scaffolds and 33 Mbp of JGI's 8.41X assembly of the *Dictyostelium purpureum* DpAX1 genome [13].

**Current statistics at 8.41x coverage:**

Main genome scaffold total: 799

Main genome contig total: 1213

Main genome scaffold sequence total: 33.0 MB

Main genome contig sequence total: 32.9 MB (with 0.4% as gaps)

Main genome scaffold N/L50: 156/66.9 KB

Main genome contig N/L50: 237/45.2 KB

Number of scaffolds > 50 KB: 240

% main genome in scaffolds > 50 KB: 64.9%

# Prediction of protein coding genes

**We predict 12410 genes, with the following average properties:**

Gene length 1760.19 nt

Transcript length 1495.22 nt

Protein length 482.64 aa

Exon frequency 2.51 exons/gene

Exon length 595.25 nt

Intron length 177.26 nt

Gene density 376.4 genes/Mbp scaffold (2.66 kb/gene)

**The genes were found by the following methods:**

Total models: 12410 (100%)

cDNAs and ESTs: 721 (6%)

Similarity to nr (non-redundant): 4050 (33%)

ab initio: 7639 (62%)

The genes were validated by the following evidence:

start+stop codons: 11170 (90%)

EST support: 4039 (33%)

nr (non-redundant) hit: 11086 (89%)

Pfam hit: 6029 (49%)

**Simple sequence repeats and Repetitive elements.**

Many proteins of *D. discoideum*, including members of the ABC and histidine kinase families, have long runs (≥ 20residues) of polyasparagine and polyglutamine. Orthologs in *D. purpureum* have many fewer such runs and the ones they do have are shorter. It appears that these and other simple sequence repeats are not essential for the function of these proteins.

The lower abundance of simple sequence repeats, their shorter lengths, and their greater complexity in amino acid homopolymers – are all consistent in direction with a possible bias. The *D. purpureum* genome is less complete, and some simple sequence repeats could therefore have been missed. Specifically, simple sequence repeats make assembly harder, particularly if they fall in generally low-complexity regions. If such losses do occur, they may be most severe for long repeats and for perfect repeats, which could explain the relative underrepresentation of both of these kinds of repeats relative to *D. discoideum*. Consistent with this kind of bias, the 100-bp regions that terminate contigs in *D. purpureum* are enriched in repeat lengths above 10 repeats compared to sequence inside the contigs, though the difference is not large (density = 0.06 vs 0.09, p < 0.001, Mann-Whitney U test, n = 667). Any bias is unlikely to explain the entire difference between the two species. If the two species actually had the same distributions, it would imply that over 3/4 of the amino acid repeats were missed in *D. purpureum* (2645 found versus 11243 in *D. discoideum*). At the upper end of the range it would imply that nearly all long *D. purpureum* repeats were missed: *D. discoideum* has 252 repeats longer than 45 amino acids, but *D. purpureum* shows only one (Supp. Fig S1b). Both of these possibilities seem very unlikely. It is clear that *D. purpureum* is similar to *D. discoideum* in having a large amount of simple sequence repeats and amino acid homopolymers, but is probably not as extreme.

Homologous amino acid homopolymers were determined by identifying aligned amino acid repeats of the same type from global sequence alignments of pairwise orthologous proteins [109]. But here we restricted homologous repeats (or conserved repeats) to those where both species had repeats longer than expected by chance (Table S1).

**Table S1. Genomic amino acid composition and minimum length of non-random homopolymers.**

| Amino acids | Composition^a^ | p<0.05^b^ | p<0.01 ^b^ | p<0.001 ^b^ |
| --- | --- | --- | --- | --- |
| Alanine (A) | 0.035 | 6 | 7 | 7 |
| Cysteine (C) | 0.015 | 5 | 5 | 6 |
| Aspartic acid (D) | 0.053 | 7 | 7 | 8 |
| Glutamic acid (E) | 0.062 | 7 | 8 | 9 |
| Phenylalanine (F) | 0.049 | 7 | 7 | 8 |
| Glycine (G) | 0.044 | 6 | 7 | 8 |
| Histidine (H) | 0.019 | 5 | 6 | 6 |
| Isoleucine (I) | 0.083 | 8 | 9 | 10 |
| Lysine (K) | 0.079 | 8 | 8 | 9 |
| Leucine (L) | 0.09 | 8 | 9 | 10 |
| Methionine (M) | 0.016 | 5 | 5 | 6 |
| Asparagine (N) | 0.093 | 8 | 9 | 10 |
| Proline (P) | 0.042 | 6 | 7 | 8 |
| Glutamine (Q) | 0.045 | 7 | 7 | 8 |
| Arginine (R) | 0.03 | 6 | 6 | 7 |
| Serine (S) | 0.095 | 8 | 9 | 10 |
| Threonine (T) | 0.057 | 7 | 8 | 8 |
| Valine (V) | 0.047 | 7 | 7 | 8 |
| Tryptophan (W) | 0.008 | 4 | 5 | 5 |
| Tyrosine (Y) | 0.039 | 6 | 7 | 7 |

^a^The fraction of the proteins in the complete predicted proteome consisting of the indicated amino acid.

^b^The minimum length of a non-random amino acid homopolymer, for a given confidence level. Below these length values, a given amino acid homopolymer could occur by chance as predicted by the overall amino acid composition of the predicted proteome.

**
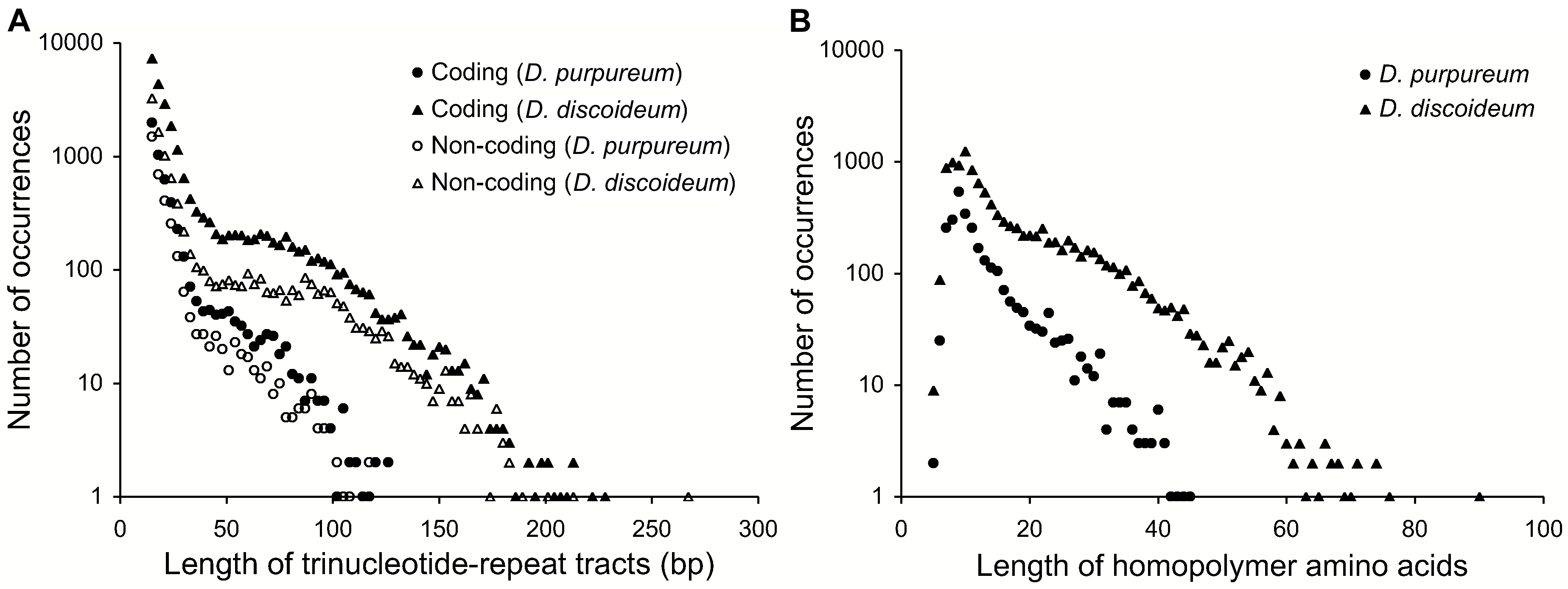
**

**Figure S1. Number of occurrences of trinucleotide repeats (A) and homopolymer amino acids (B) in *Dictyostelium* genomes.** A. *D. purpureum* genome (circles) contains fewer and shorter trinucleotide repeats than *D. discoideum* (triangles). Coding (solid circles and triangles) sequences has more repeats than non-coding (open circles and triangles) sequences in both species. B. *D. purpureum* proteome (circles) contains fewer and shorter homopolymer amino acids than *D. discoideum* (triangles). Not shown are 1 *D. purpureum* and 2 *D. discoideum* repeats above 100 amino acids.

**Figure S2. Codon usage within amino acid repeats and outside the repeats.**


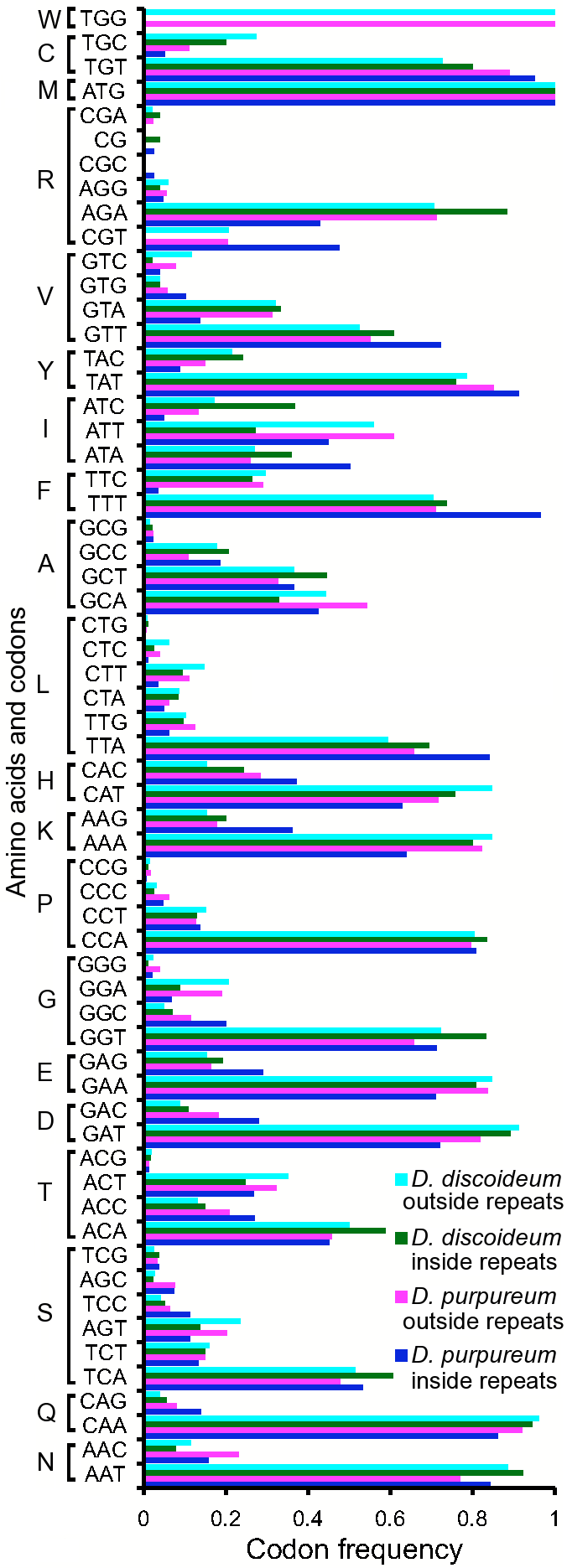


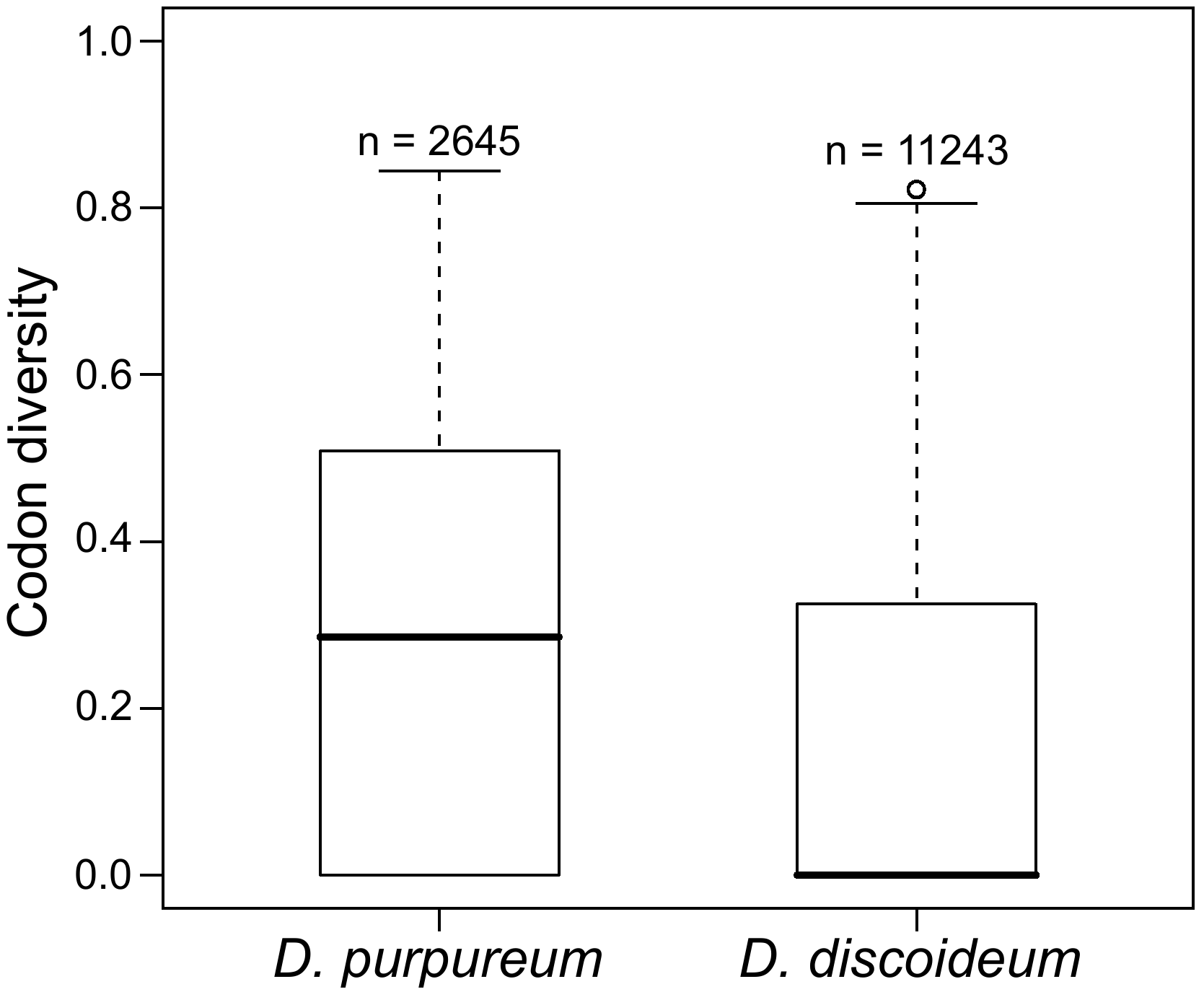


**Figure S3. Codon diversity of homopolymer amino acid repeats.** Codon diversity was calculated as the probability that two random codons in a perfect amino acid repeat match each other: where *N* is the number of codons, *i* indexes the different codon types, and *p_i_*. is the proportion of the amino acids in that sequence that are the *i*^th^ codon. Codons of homopolymer amino acids have higher diversity in *D. purpureum* than in *D. discoideum* (Mann-Whitney U test, *p* < 0.0001). Of the box-and-whisker diagram, boxes show quartiles, whiskers show the most extreme data point which is no more than 1.5 times the interquartile range from the box, and circles show outliers.

**
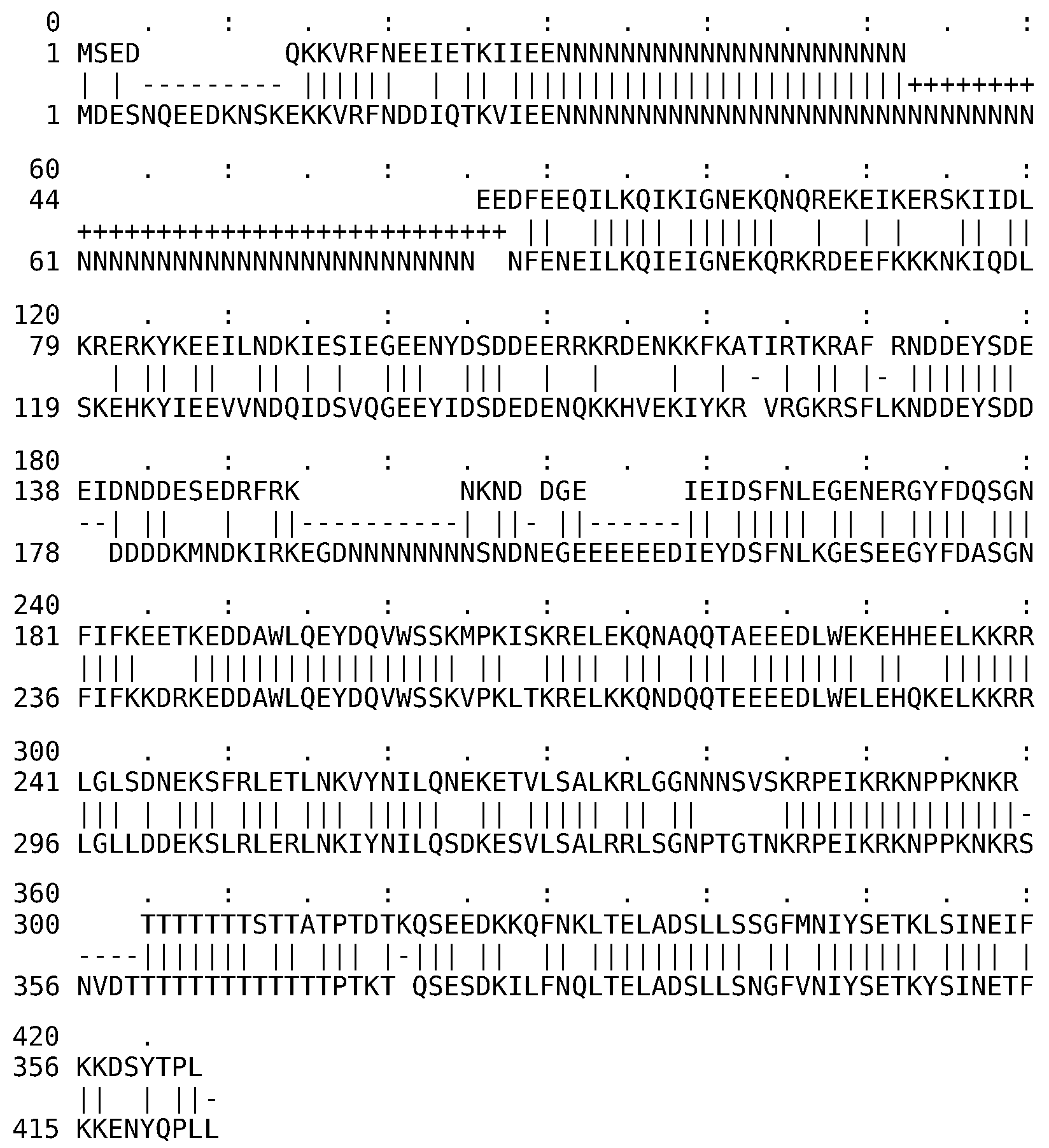
**

**Figure S4. An example of pairwise sequence alignment of proteins with amino acid homopolymers in both species.** Protein sequences of DPU0062537 (dictyBase locus tag, upper sequence, 363 residues) and DDB0233538 (lower sequence, 423 residues) from *D. purpureum* and *D. discoideum*, respectively, were aligned by the program GAP4 and the BLOSUM62 matrix. The ‘.’ and ‘:’ above upper sequences mark the position of the consensus sequence; Between the upper and the lower sequences, the ‘|’ mark matched residues; the ‘-’ and ‘+’ mark InDels; while mismatched residues are showed by spaces.


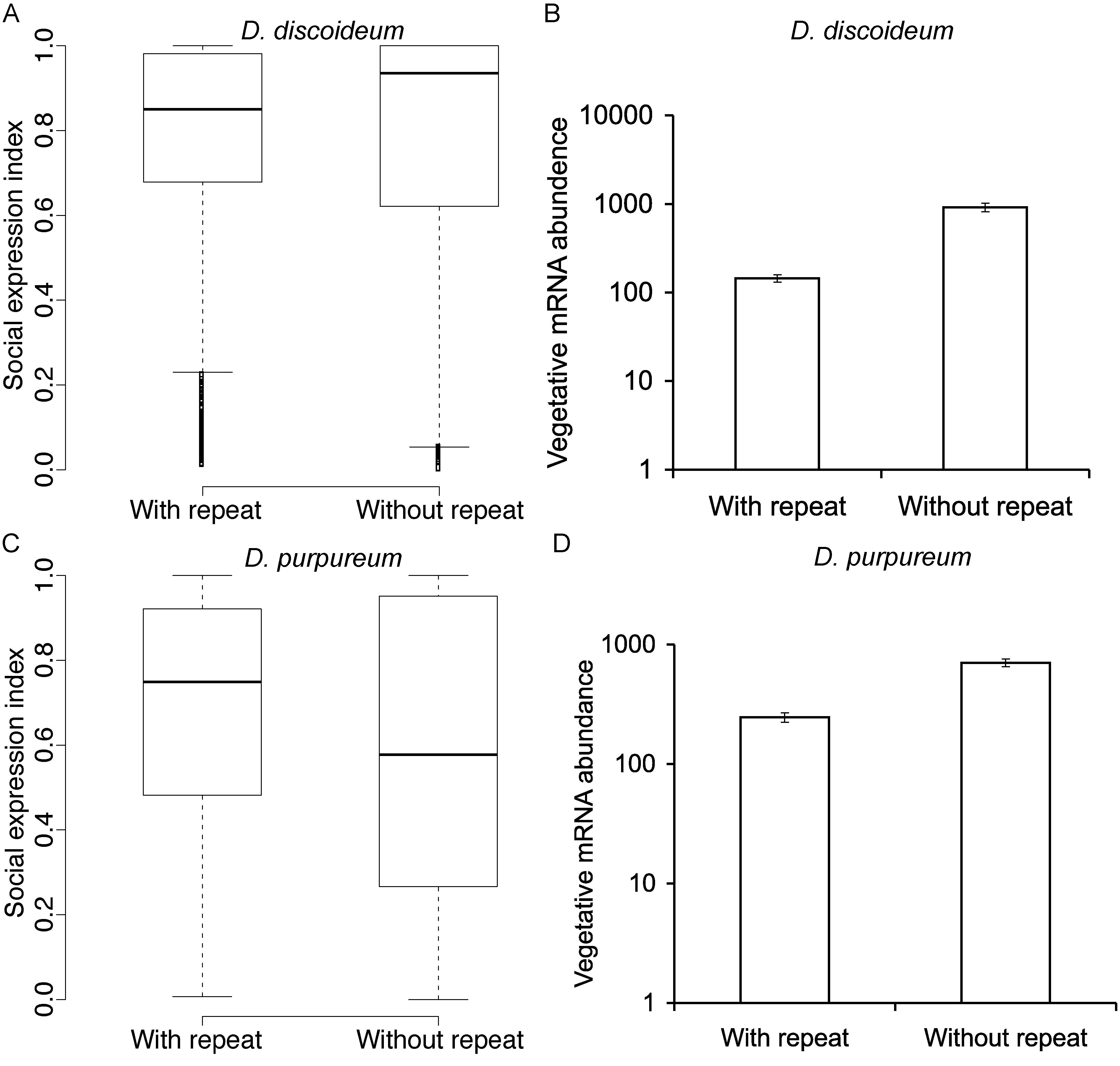


**Figure S5. Expression stages of genes with or without amino acid repeats.** Panels A and C. Genes with amino acid repeats have higher relative expression in the social or developmental stages in *D. purpureum* (n = 1905, 3846, p < 0.0001, both Student’s t-test and Mann-Whitney U test) and *D. discoideum* (n = 1600, 1439, *p* < 0.05, Student’s t-test; p < 0.0001, Mann-Whitney U test). The social expression index measures a gene's relative expression in the social or developmental stage compared to the vegetative stage (see *Analysis of Social Genes* below). Panels B and D. Genes with repeats had lower vegetative-stage mRNA abundance, as measured by RNA-seq reads. Boxes show quartiles, whiskers show the most extreme data point which is no more than 1.5 times the interquartile range from the box, and circles show the outliers. Panels B and D. Genes with amino acid repeats have lower abundance of vegetative ESTs than genes without amino acid repeats both in *D. purpureum* and *D. discoideum* (Students t-test, both test *p* < 0.0001). Error bars show standard errors of the means.

# Phylogeny

Orthologs were grouped by single linkage clustering of protein sequences on mutual-best BLASTP hits from a colletion of pairwise genome comparisions:  human [100] versus each of (Oryzias latipes [100]; Gallus gallus, [100]; Branchiostoma floridae, JGI version 2 [13]; Nematostella vectensis, JGI version 1 [13]; Neurospora crassa, Broad release 7 [102]; Arabidopsis thaliana, TAIR8 [103]; Chlamydomonas reinhardtii, JGI version 4 [13]; Dictyostelium discoideum [14], plus D. discoideum vs each of (D. purpureum, Entamoeba histolytica [22]).  There were 389 clusters that had exactly one gene from each genome.  For each of these clusters, the protein sequences were aligned by ClustalW, and the alignments were trimmed with default settings in Gblocks, leaving a mean of 216 filtered alignment columns per ortholog group, and 83888 aligned columns in total, with no gaps or missing data.  The concatenated alignment was analyzed with mrbayes 3.1.2 using the WAG model, I+Gamma for 100,000 generations, with the first 50% of sampled trees discarded.  The resulting consensus tree was rooted at the midpoint of the branch connecting the green plants to the rest of the tree.


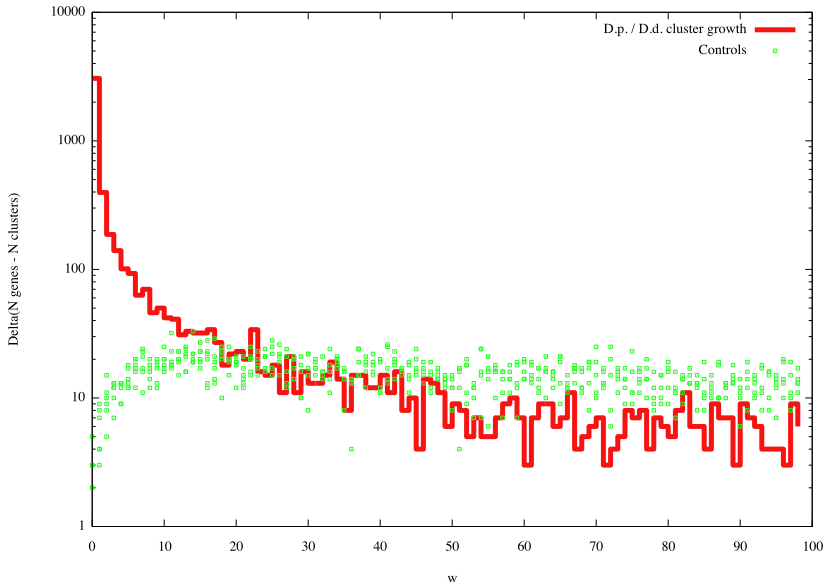


**Figure S6. Synteny conservation between the *D. purpureum* and *D. discoideum* genomes**

We identified blocks of approximately conserved gene order (supplemental Table S2) by clustering together in the same block all pairs of genes that are within a threshold distance (w) of one another, and have their orthologs in the other genome also within a distance (w), where w is the number of intervening genes that have an ortholog in the other genome.  The plot shows the rate of growth of these blocks of conserved gene order as a function of increasing w for for *D. purpureum* versus *D. discoideum* to that for permutation controls in which the gene orders were scrambled in both genomes.  We found that up to w values of approximately 15 intervening genes, clusters grow significantly faster than in the random genome order expectation, which provides a conservative threshold for identifying blocks of approximately conserved gene order.  With this estimate 79% of orthologous gene pairs participate in a block of approximately conserved gene order, compared to 5.8 +- 0.4% in controls, with a false positive rate, on a gene-by-gene basis of ~7%.

**Table S2. Syntenic blocks of genes (Separate Table).**

The genes contained in the blocks of approximately conserved gene order are listed in the order they are found in the *D. discoideum* genome.

**Non-coding RNAs**

*Spliceosomal RNAs*

The spliceosomal RNAs (rnUs) identified in *D. discoideum*, U1, U2, U4, U5, and U6, are each characterized by both specific RNA-binding motifs and the ability to fold into specific secondary structures [30, 31]. An initial BLAST search of these sequences against the *D. purpureum* genome generated a collection of very short (~12 bp), exact matches. Each match was aligned against its *D. discoideum* query sequence, and then extended in both directions to match the length of the query sequence. Extended matches that overlapped in the *D. purpureum* genome were merged. This generated a group of hits for each *D. discoideum* rnU. Each group was aligned with the corresponding *D. discoideum* rnU, and the best-aligned sequences were selected as putative homologs. These best hits all possess high sequence identity in the RNA-binding regions of the rnU. Sequences with low conservation are mostly involved in self-binding, and the differences between species are compensatory, in that similar structures can be formed. When considering the large divergence between the two Dictyostelids, it is not surprising that while secondary structures are conserved, the constituent bases are not.

*tRNAs*

We searched for tRNA genes and found at least one gene representing each amino acid (Table S3b) [110]. Genes corresponding to the rare alanine tRNA with a GCG anti-codon were absent in the intial assembly of the *D. discoideum* and presumed to be the unassembled portion of the genome [1]. The presence of one such tRNA gene in the *D. purpureum* genome would support the notion that these tRNA genes were not lost from the Dictystelids (Table S3b).

Table S3a. Non-coding RNA genes.

| ncRNA | *D. discoideum* | *D. purpureum* |
| --- | --- | --- |
| In other Eukaryotes |  |  |
| Spliceosomal RNAs | U1, U2, U4, U5, U6 | U1, U2, U4, U5, U6 |
| D2/D9 | First dictyostelid ncRNA | - |
| snoRNAs | Box C/D, Box H/ACA | - |
| Antisense RNA | psvA | possible psvA |
| RNase RNA | tRNA, rRNA processing | - |
| tRNA | 418 genes, all anticodons | 353 genes, all anticodons |
| Found only in *D.* *discoideum* |  |  |
| Classes I & II | Class I & II | yes |
| D1/Dd8 | yes | - |
| dutA | yes | - |
| msRNA | yes | - |

***Table S3b. tRNA genes in*** D. purpureum

| Amino acid | Anti-codon | Number of genes | Amino acid | Anti-codon | Number of genes | Amino acid | Anti-codon | Number of genes |
| --- | --- | --- | --- | --- | --- | --- | --- | --- |
| Ala | AGC | 17 | Gly | GCC | 18 | Pro | AGG | 1 |
| Ala | CGC | 1 | Gly | TCC | 4 | Pro | TGG | 15 |
| Ala | TGC | 4 | His | GTG | 7 | Ser | AGA | 5 |
| Arg | ACG | 7 | Ile | AAT | 16 | Ser | CGA | 1 |
| Arg | CCT | 1 | Ile | TAT | 4 | Ser | GCT | 6 |
| Arg | TCG | 1 | Leu | AAG | 11 | Ser | TGA | 13 |
| Arg | TCT | 9 | Leu | CAA | 1 | Thr | AGT | 15 |
| Asn | GTT | 17 | Leu | CAG | 1 | Thr | CGT | 1 |
| Asp | GTC | 21 | Leu | TAA | 16 | Thr | TGT | 5 |
| Cys | GCA | 6 | Leu | TAG | 1 | Trp | CCA | 7 |
| Gln | CTG | 1 | Lys | CTT | 6 | Tyr | GTA | 12 |
| Gln | TTG | 12 | Lys | TTT | 20 | Val | AAC | 17 |
| Glu | CTC | 2 | Met | CAT | 13 | Val | TAC | 4 |
| Glu | TTC | 20 | Phe | GAA | 14 |  |  |  |

***The DUSE.*** A number of ncRNAs have a “*Dictyostelium* upstream sequence element” (DUSE), an eight-nucleotide motif ([AT]CCCA[AT]AA) that sits ~63 base pairs upstream [111]. While the *D. purpureum* spliceosomal RNAs were identified using only the *D. discoideum* spliceosomal RNA sequences, they also had a DUSE sitting ~63bp upstream.

To identify any potential ncRNAs downstream of the DUSE both genomes were first masked for protein coding regions for *D. pururpeum* [112] and for *D. discoideum* [113]. Then each genome was masked for low-complexity sequence via the dustmasker program in the NCBI toolkit [22].

From these masked genomes, every instance of a relaxed DUSE motif (/([ATCG]CCC(?(?<=[AC]CCC)A[^A]AA|A[ATCG]AA))/ as a Perl regular expression) was identified, and each was associated with its Downstream Region (DSR), which consists of the 100 bp that sits 50 bp downstream of the DUSE. In *D. discoideum*, there are 7,409 DUSEs, whose DSRs represent 2.1% of the entire genome and 3.1% of the unmasked bases in the genome. In *D. purpureum*, there are 11,234 DUSEs, whose DSRs represent 3.4% of the entire genome and 3.4% of the unmasked bases in the genome.

Next each 8-mer was counted in both the entire masked genome and the DSR, for each dictyostelid, and combined to give an occurrence ratio (the number of times an 8-mer occurs in the DSRs divided by the number of times an 8-mer occurs in unmasked genome) (Figure S7, Table S4). Three 8-mers are found to have the three highest DSR occurrence ratios in both Dictyostelids (>0.29 in each genome). The significance of this trio was assessed by performing the same analysis for all 8-mers that occur in the genome approximately the same number of times as the relaxed DUSE motif (between 4,500 and 14,500 times in each genome). For each of these control 8-mers, DSRs were extracted from each masked genome, and downstream 8-mer occurrence ratios were calculated as they were for the DUSE. Out of 879 control 8-mers, there were none that had an 8-mer with occurrence ratios > 0.3 in both genomes, and one that had two

# A.

#
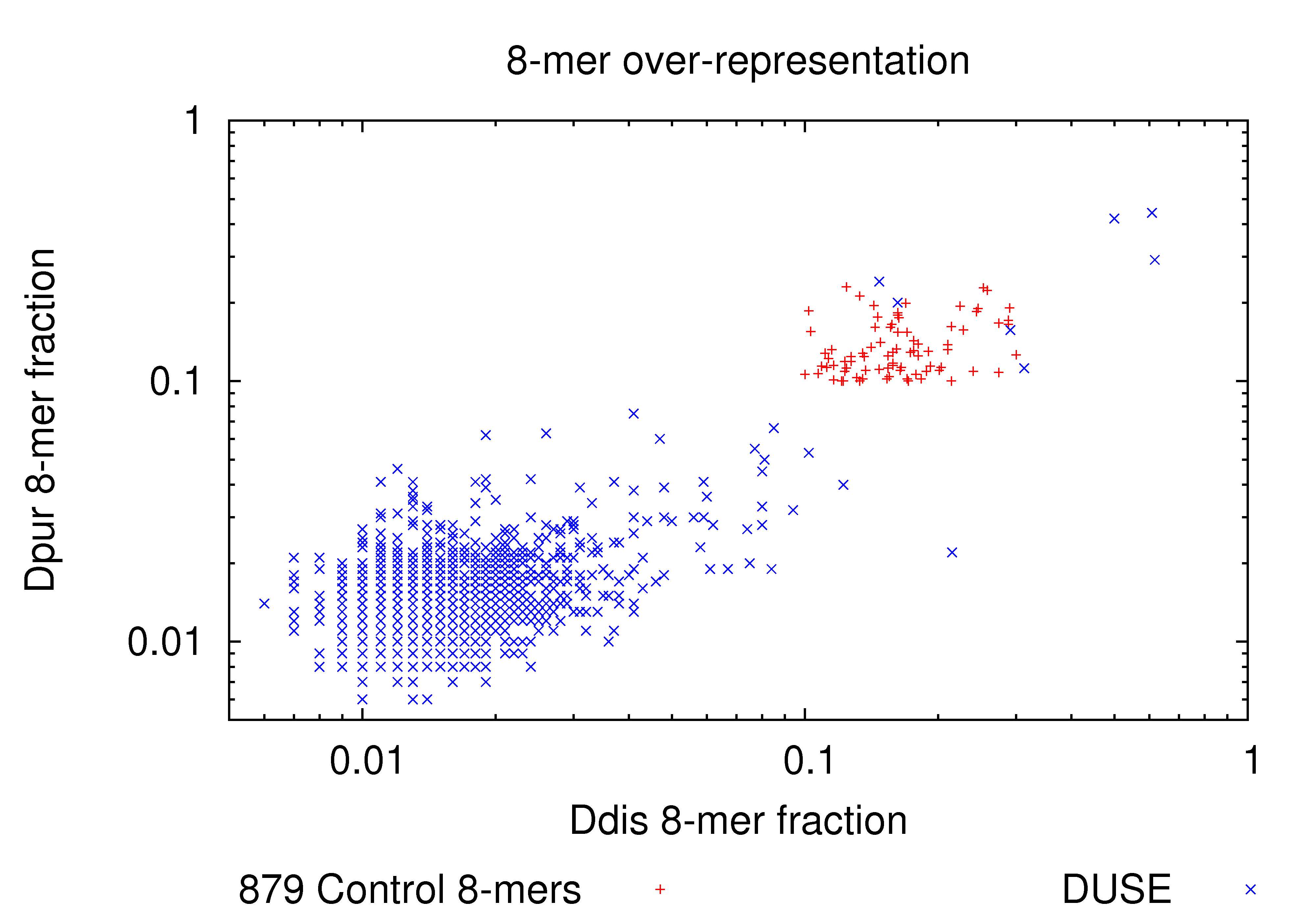


# B.

**Figure S7. Occurrence of 8-mer sequences downstream of DUSE elements.** A. In blue are the all the DUSE-enriched 8-mers with occurrence ratios >0.2 in both genomes. Six of these seven 8-mers are subsequences of the NSBR in the classes I and II ncRNAs. In red are all 8-mers (second column) enriched for by some control DUSE (first column) with occurrence ratios >0.15 in both genomes. B. Each blue point represents an 8-mer that sits in the DSRs of the DUSE. Only those 8-mers that have occurrence ratios >0.1 in both genomes are shown for the 879 Control DUSEs (red). The three 8-mers with the highest ratios, in the upper right-hand corner of the plot, are CCTTACAG, CTTACAGC, and CTAGCAGA.

8-mers with occurrence ratios >0.2. Figure S7 shows that the 8-mers of the DUSE trio each have higher occurrence ratios than any of the sequences enriched for by the control 8-mers.

***Identification of D. purpureum class I and class II homologs.*** The most DUSE-enriched 8-mer, CTTACAGC, starts in the second position of NSBR of the class I and class II ncRNAs (Figure 4). To identify putative homologs in *D. purpureum*, each occurrence of this 8-mer was identified in both *D. purpureum* and *D. discoideum*. Of the 46 occurrences of the 8-mer in *D. discoideum*, 28 possess an upstream DUSE within 100 bp. For each of these 28, a 5'-stem sequence was identified as the 5 bp that sit 6 bp upstream of the 8-mer. The closest sequence that was both complementary to the 5'-stem and sat between 20 bp and 100 bp downstream of the 8-mer was identified as the 3'-stem. 26 of the 28 sequences had 5'/3' complementary sequences. These 26 all corresponded to previously identified classes I and II ncRNAs in *D. discoideum* (Figure S8). The same analysis was performed on *D. purpureum*, identifying 61 instances of the 8-mer, 27 of which had an upstream DUSE. 26 of those also had 5'/3' complementary sequences. These 26 sequences constitute the putative *D. purpureum* ncRNAs. Because they all share the same 5'-stem sequence, they might be considered putative class I ncRNAs.

To assess the secondary structure of these putative ncRNAs, all 52 sequences, from both dictyostelids, were aligned structurally using the locARNA [114] (Figure S8). This alignment and structure was then passed to RNAlogo server [115]. This provided an image that was adapted for Figure 4.

**Figure S8.** **Sequences of putative novel non-coding RNAs in *D. purpureum* and *D. discoideum*.** All 52 identified classes I and II ncRNAs are structurally aligned.

## Protein-coding gene product prediction

**Protein** **Ortholog Discovery**

A list of orthologous genes between *D. purpureum* and *D. discoideum* was created using three sources (in order of confidence): manual curation (as of February 2009), and the Inparanoid algorithm [116], and Best bidirectional blast algorithm. Briefly, both algorithms find reciprocal best match between amino acid sequences using BLASTP. All of the *D. discoideum* gene models from the May 2009 genome build of dictyBase were blasted against all the *D. purpureum* gene models. The Inparanoid algorithm has the advantage of finding one-to-many or many-to-many relationships, by using the reciprocal best matches as seeds to cluster additional orthologs. A minimum cutoff for similarity of 50 bits and 50% overlap was used to avoid inclusion of insignificant hits.

The predictions from the Inparanoid algorithm were validated using the manually curated list. Of the 5,826 one-to-one ortholog pairs identified, 205 had been manually curated. Of those 205 Inparanoid predictions, 202 agreed with the manual curation.

**Table S4.** (**Summary of Additional Data File 2)**

| **Source** | **Number of orthologs** |
| --- | --- |
| **Manual Curation (February 2009)** | 257 |
| **Inparanoid** | 5,826 |
| **Reciprocal Best Match** | 1,536 |
| **Total Orthologs** | 7,619 |

The complete list of orthologs can be found in the Supplemental Table S4 Excel file.

There are 2,759 additional *D. pupureum* genes and 2,990 genes in *D. discoideum* that are paralogous, but where one-to-one orthologous relationships could not be determined (Supplemental Table S5, as separate Excel Table). These families probably represent small gene groups that underwent duplication and divergence soon after the split of the last common ancestor of *D. pupureum* and *D. discoideum*. There are also 2,001 genes in *D. pupureum* genes and 3,531 genes in *D. discoideum* that failed to match any gene in the other genome with a bit score higher than 50.


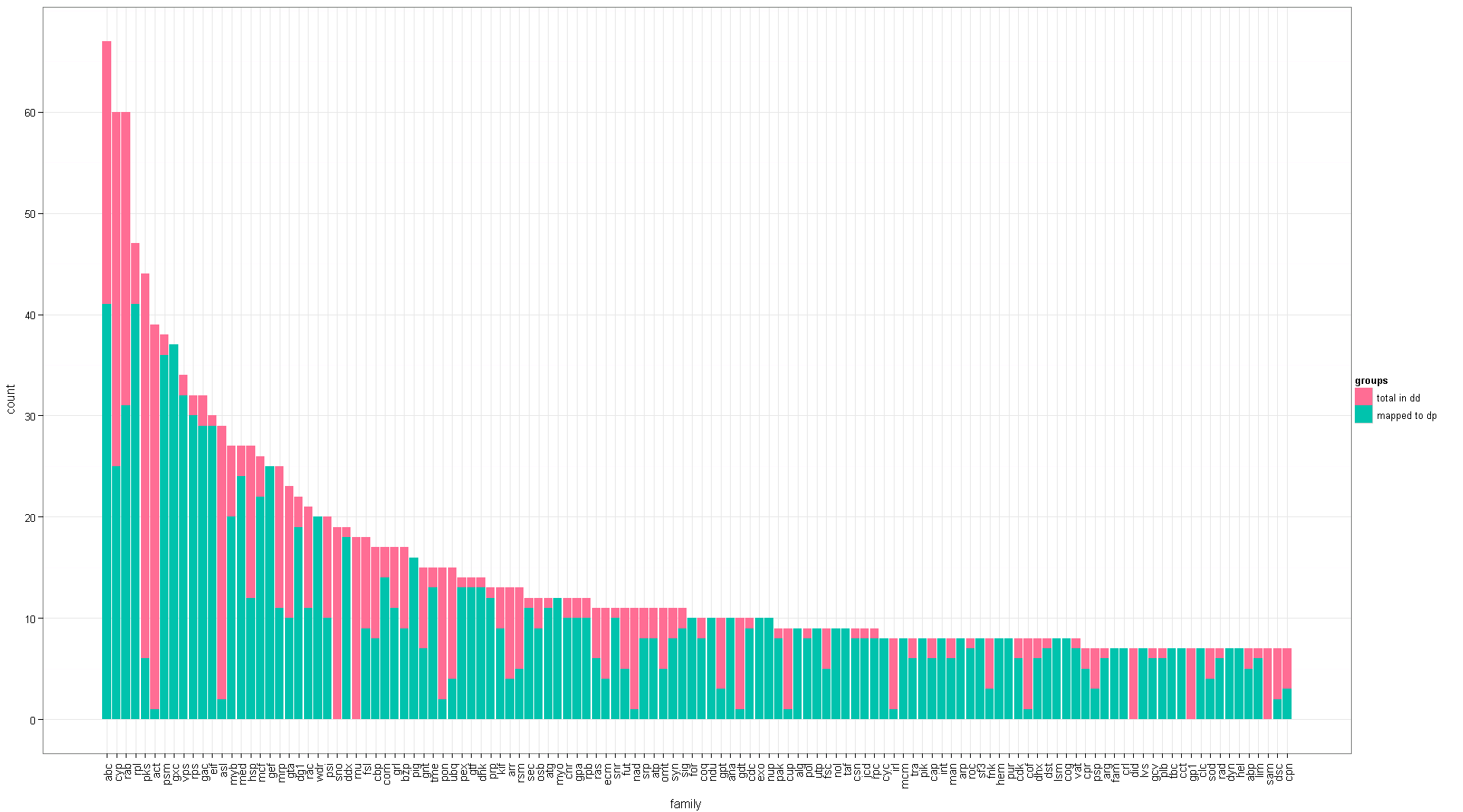


Total genes in *D. discoideum*

Genes mapped to *D. purpureum* orthologs

**Figure S9. Proportion of orthologous genes between *D. purpureum* and *D. discoideum* in major gene families.** Histogram of the number of *D. discoideum* genes that have clear othologs in *D. purpureum* for the 127 gene families listed in Legend below. Gene families (in blue) are listed with their annotations, the number of members in the *D. discoideum* reference genome and the number of those members that have clear orthologs in *D. purpureum*. Only automated gene product annotations were used, so the numbers of genes in each family will change as manual annotation refines the gene product predictions.

| Gene Family | Total in D.d. | Annotation | Ortho-  logs in D.p. | Gene  Family | Total in D.d. | Annotation | Ortho-  logs in D.p. |
| --- | --- | --- | --- | --- | --- | --- | --- |
| abc | 67 | ABC transporter G family protein | 41 | irl | 8 | IRE family protein kinase | 1 |
| abp | 7 | alpha actinin, actin bundling protein | 5 | jcd | 9 | transcription factor jumonji, jmjC domain-containing protein | 8 |
| act | 39 | actin | 1 | kif | 13 | kinesin family member 9 | 9 |
| alg | 9 | glycosyltransferase, beta-1,4-mannosyltransferase | 9 | tgr | 16 | IPT/TIG domain-containing protein, immunoglobulin E-set domain-containing protein (Tiger protein) | 3 |
| ana | 10 | anaphase promoting complex subunit 11 | 10 | lim | 7 | LIM-type zinc finger-containing protein | 6 |
| arg | 7 | acetylornithine deacetylase | 6 | lsm | 8 | LSM (like-Sm) protein, U6 small nuclear RNP-associated protein | 8 |
| arp | 8 | actin related protein 2, polyphosphate kinase component | 8 | lvs | 7 | BEACH domain-containing protein | 7 |
| arr | 13 | ADP-ribosylation factor-related, ARF-related | 4 | man | 8 | alpha-mannosidase | 6 |
| asl | 29 | putative acetyl-CoA synthetase | 2 | mcf | 26 | mitochondrial substrate carrier family protein | 22 |
| atg | 13 | autophagy protein 4 | 13 | mcm | 8 | MCM family protein, putative DNA replication licensing factor | 8 |
| atp | 11 | ATPase subunit 1, ATP synthase F1 alpha | 8 | med | 27 | putative mediator complex subunit 8 | 24 |
| bzp | 17 | putative basic-leucine zipper (bZIP) transcription factor | 9 | mrp | 25 | S60 ribosomal protein L11, ribosomal protein L11, mitochondrial | 11 |
| cap | 8 | cAMP-binding protein | 6 | myb | 27 | myb transcription factor | 20 |
| cbp | 17 | calcium-binding protein | 8 | myo | 12 | class VII unconventional myosin, myosin VII | 12 |
| cct | 7 | chaperonin containing TCP1 eta subunit | 7 | nad | 11 | NADH dehydrogenase subunit 4 | 1 |
| cdc | 10 | cell division cycle protein 48, CDC48 family AAA ATPase | 9 | nag | 7 | beta-N-acetylhexosaminidase, glycoside hydrolase family 20 protein, beta-hexosaminidase | 5 |
| cdk | 8 | p34-cdc2 protein, protein serine/threonine kinase, cyclin-dependent kinase, CMGC group | 6 | ndu | 10 | ubiquinone oxidoreductase, NADH dehydrogenase ubiquinone flavoprotein 1 | 10 |
| clc | 7 | clathrin light chain | 7 | nol | 9 | pseudouridine synthase family protein, DKCLD domain-co protein, PUA domain, H/ACA RNP complex subunit 4 | 9 |
| cnr | 12 | cleft lip and palate transmembrane 1 family protein, putative cell number regulator | 10 | nup | 10 | nucleoporin 107 | 10 |
| cof | 8 | cofilin-2 | 1 | omt | 11 | O-methyltransferase family 2 protein | 5 |
| cog | 8 | oligomeric Golgi complex component | 8 | osb | 12 | oxysterol binding family protein, member 1 | 9 |
| com | 18 | NAD-dependent aldehyde dehydrogenase | 14 | pak | 9 | p21-activated protein kinase, protein serine/threonine kinase, STE20 family protein kinase, PAKA subfamily protein kinase | 8 |
| coq | 10 | ubiE/COQ5 methyltransferase family protein | 8 | pex | 14 | WD40 repeat-containing protein, peroxisomal biogenesis factor 7, peroxin 7, peroxisome targeting signal type 2 receptor | 13 |
| cpn | 7 | phospholipid-binding protein, copine A | 3 | pig | 16 | GlcNAc transferase, phosphatidylinositol glycan, class A, phosphatidylinositol N-acetylglucosaminyltransferase subunit A, glycosyltrasferase | 16 |
| cpr | 9 | cysteine proteinase 5 precursor | 7 | pik | 8 | phosphatidylinositol-4,5-diphosphate 3-kinase, PI3kinase | 8 |
| crl | 7 | cAMP receptor-like protein, G-protein-coupled receptor (GPCR) family protein | 7 | pks | 44 | short-chain dehydrogenase/reductase (SDR) family protein, 3-oxoacyl-[acyl-carrier protein] reductase | 6 |
| csn | 9 | putative casein kinase II beta chain (CK2) | 8 | plb | 7 | phospholipase B | 6 |
| cup | 9 | calcium up-regulated protein, ricin B lectin domain | 1 | pol | 9 | mitochondrial DNA polymerase A, 5'-3' exonuclease domain-containing protein, 3'-5' exonuclease domain | 8 |
| cyc | 8 | cyclin, G2/M-specific cyclinB | 8 | pon | 15 | actin binding protein, ponticulin | 2 |
| cyp | 60 | cyclophilin E, cyclophilin-type peptidylprolyl cis-trans isomerase (PPIase) | 25 | prp | 13 | Propionate catabolic protein PrpD. | 12 |
| ddx | 19 | putative RNA helicase, DEAD/DEAH box helicase | 18 | psi | 20 | PA14 domain-containing protein | 10 |
| dg1 | 22 | RING zinc finger-containing protein, Kelch repeat-containing protein | 19 | psm | 42 | proteasome subunit alpha type 7, 20S proteasome subunit alpha-7 | 39 |
| dhk | 14 | Atypical group, HisK family protein kinase | 13 | psp | 7 | prespore protein | 3 |
| dhx | 8 | DEAD/DEAH box helicase, putative RNA splicing factor | 6 | pur | 8 | adenylosuccinate synthetase | 8 |
| did | 7 |  | NA | rab | 60 | Rab GTPase | 31 |
| dsc | 7 | discoidin II | 2 | rac | 21 | Rho GTPase, RhoBTB family protein | 11 |
| dst | 8 | signal transducer and activator of transcription (STAT) family protein | 7 | rad | 7 | DNA repair protein Rad4 family protein | 6 |
| dyn | 7 | dynactin 150 kDa subunit | 7 | ras | 11 | Ras GTPase | 6 |
| ecm | 11 | extracellular matrix protein ST310 | 4 | rnu | 18 | snRNA, small nuclear RNA | NA |
| eif | 30 | eukaryotic translation initiation factor 5A | 29 | roc | 8 | WD40 repeat-containing protein, protein kinase, tyrosine kinase-like protein, ROCO family protein kinase | 7 |
| exo | 10 | 5'3'-exonuclease N- and I-domain-containing protein, putative exodeoxyribonuclease I | 10 | rpb | 12 | RNA polymerase II largest subunit, RNA polymerase II core subunit | 10 |
| fam | 9 | DUF59 family protein, FAM96 family protein | 9 | rpc | 9 | putative RNA polymerase III subunit, putative RNA polymerase I subunit | 9 |
| fnk | 8 | FNIP repeat-containing protein, protein kinase, STE group | 3 | rpl | 48 | S60 ribosomal protein L36 | 42 |
| for | 10 | actin binding protein, formin homology domain-containing protein | 10 | rps | 32 | 40S ribosomal protein S4 | 30 |
| fsc | 9 | G-protein-coupled receptor (GPCR) family protein, frizzled and smoothened-like sans CRD | 5 | rsm | 13 | small GTPase | 5 |
| fsl | 18 | G-protein-coupled receptor (GPCR) family protein, frizzled and smoothened-like protein | 9 | sam | 7 | SAM domain-containing protein, SAMK family protein kinase | NA |
| fut | 11 | glycosyltransferase, alpha-3/4-fucosyltransferase | 5 | sec | 12 | ankyrin repeat-containing protein, pleckstrin homology (PH) domain-containing protein, Arf guanyl-nucleotide exchange factor | 11 |
| gac | 32 | RhoGAP domain-containing protein | 29 | sf3 | 8 | C2H2-type zinc finger-containing protein, splicing factor 3A subunit 3 | 8 |
| gcv | 7 | glycine dehydrogenase (decarboxylating), glycine cleavage system P-protein | 6 | sig | 11 | unknown | 10 |
| gdt | 10 | GDT family protein kinase | 1 | sno | 19 | C/D-box small nucleolar RNA, C/D box snoRNA | NA |
| gef | 25 | Ras guanine nucleotide exchange factor | 25 | snr | 11 | U1 small nuclear ribonucleoprotein 70 kDa protein | 10 |
| gnt | 15 | GlcNAc transferase, UDP-GlcNAc:hydroxyproline polypeptide alpha-N-acetylglucosaminetransferase | 7 | sod | 7 | superoxide dismutase | 4 |
| gp1 | 7 | cell surface glycoprotein gp138 | NA | srp | 11 | signal recognition particle 54 kDa subunit | 8 |
| gpa | 12 | G-protein subunit alpha 7 | 10 | syn | 11 | putative t-SNARE family protein, putative syntaxin 6 | 8 |
| gpt | 10 | putative glycophosphotransferase | 3 | taf | 9 | putative protein serine/threonine kinase, bromodomain-containing protein, transcription initiation factor TFIID subunit, TFIID subunit, protein kinase, Atypical group, TAF1 family protein kinase, HAF group protein | 9 |
| grl | 16 | G-protein-coupled receptor (GPCR) family 3 protein 14 | 10 | tbc | 7 | RabGAP/TBC domain-containing protein, TBCK family protein kinase | 7 |
| gta | 23 | putative GATA-binding transcription factor | 10 | tme | 16 | transmembrane protein, DUF786 family protein | 14 |
| gtf | 14 | transcription initiation factor IIA gamma chain | 13 | tra | 8 | heat shock protein Hsp90 family protein | 6 |
| gxc | 37 | pleckstrin homology (PH) domain-containing protein, RhoGEF domain-containing protein, armadillo repeat-containing protein | 37 | ubq | 15 | ubiquitin/ribosomal protein S27a fusion protein | 4 |
| hbx | 14 | putative homeobox transcription factor, homeodomain (HOX) | 11 | utp | 9 | U3 snoRNP protein, U3 small nucleolar ribonucleoprotein | 9 |
| hel | 7 | putative RNA helicase, DEAD/DEAH box helicase | 7 | vat | 8 | vacuolar H+-ATPase E subunit | 8 |
| hem | 8 | putative uroporphyrinogen III synthase | 8 | vps | 38 | vacuolar protein sorting-associated protein 26 | 36 |
| hsp | 27 | heat shock protein, heat shock cognate protein Hsc70-2 | 12 | wdr | 21 | WD40 repeat-containing protein | 21 |
| int | 8 | type A von Willebrand factor (VWFA) domain-containing protein, initiation factor eIF-4 gamma middle domain-containing protein, integrator complex subunit 6 | 8 |  |  |  |  |

# Predicted proteins present in *D. purpureum* and absent in *D. discoideum*

There are 2,001 of the 12,410 predicted *D. purpureum* genes that have no obvious counterpart in *D. discoideum* (Table S5). Many of these are likely to be erroneous predictions or gene fragments that will be clarified upon more extensive annotation over time. Even given this uncertainty, there are a number of well annotated proteins in *D. purpureum* that appear to be absent in *D. discoideum*, although some of these may point to gaps in the *D. discoideum* genome. Also, as the manual annotation of both genomes progresses, the presence of any of these predicted gene products may be revealed. Among the annotated genes only 8 characterized *D. discoideum* genes did not have identifiable homologs in the current *D. purpureum* assembly. Two of these 8, *ponA* and *ponB*, are related to each other and reside on different chromosomes in *D. discoideum,* so they are likely to be a true difference in gene content between the two species. If all of the other 6 missing genes represent true gaps in the *D. purpureum* sequence, it would suggest that we are missing, roughly, <0.1% of the *D. purpureum* genes in our current assembly.

* 158581 (*D. purpureum* protein I.D) / DPU_G0068098 (dictyBase Gene I.D): *D. purpureum* has 2 neks, and *D. discoideum* has 3. *D. discoideum* has nek2, nek3,and nek4, and *D. purpureum* has only nek3 and the nek6-like.

* 160185 / DPU_G0073346: TruB homolog; IPR002501 and IPR014780 tRNA pseudouridine synthase B, N-terminal, bacterial-type; while *D. discoideum* seems to have tRNA pseudouridine synthase D, there is no tRNA pseudouridine synthase B; TruB1 homolog animals.

*  75000 / DPU_G0055928: Chitin binding protein.

* 91849 / DPU_G0075114, 96643 / DPU_G0058838, 151171 / DPU_G0075100, 149996 / DPU_G0071494: several copies of chitinase II (the related family PTHR11177 is present, but not IPR011583 (SM00636) in Entamoeba histolytica as well.

* 96521 / DPU_G0054712, 91204 / DPU_G0054716, 147640 / DPU_G0054714: Sulfatase-modifying factor; this domain is absent from *D. discoideum* and *S. cerevisiae*. From InterPro entry (IPR005532): SUMF1 is a paralogue of oxoalanine-generating enzyme, also called C(alpha)-formylglycine generating enzyme (FGE). SUMF1 converts newly synthesized inactive sulfatases to their active form by modifying an active site cysteine residue to oxoalanine. Sulfatases are essential for the degradation of sulphate esters, whose catalytic activity is dependent upon an oxoalanine residue.

* 148893 / DPU_G0065326: CORD and CS domain protein (cysteine and histidine-rich domain (chord)-containing protein), similar to Integrin beta-1-binding protein 2 (Q9UKP3).

* 11178 / DPU_G0066964, 11201 / DPU_G0069136: keratin associated protein; domain PD010562 - neither protein nor domain is in *D. discoideum.*

* 78213 / DPU_G0075718: barnase inhibitor: Extracellular ribonuclease of Bacillus amyloliquefaciens. The gene is not present in eukaryotes, so this gene may be in the *D. purpureum* genome as the result of a horizontal gene transfer.

* 91798 / DPU_G0074254: phosphocarrier protein HPr - bacterial ptsH; however this is a complex, and the other subunits, ptsI or crr, do not seem to be present. The gene is not present in eukaryotes, so this gene may be in the *D. purpureum* genome as the result of a horizontal gene transfer.

* 152726 / DPU_G0053860: cation transport regulator ChaB; however, chaA is absent, chaB function is unknown, and predicted to be a regulator. chaC is present in *D. discoideum* and in most eukaryotes (associated with the putative ChaA Ca2+/H+ cation transport protein in Escherichia coli; function unknown; Dicty has another family of Ca2+/H+ transporters). The chaB gene is not present in eukaryotes, so this gene may be in the *D. purpureum* genome as the result of a horizontal gene transfer.

* 156106 / DPU_G0061782: very conserved to fungal and bacterial proteins; IPR005247 YbhB and YbcL; bacterial PBP family phospholipid-binding protein / PEBP family protein; there are no blast hits to any other amoeba.

* 157748 / DPU_G0065844: conserved in tetrahymena and branchiostoma, a chordate, but no conserved domains.

* 82874 / DPU_G0063878 minD, 84186 / DPU_G0067936 minE: involved in septum formation in bacterial species (possibly involved in mitochondrial replication).

## Polyketide Synthases

Pfam HMMs were used to identify the keto-acyl synthase (ketoacyl-synt and Ketoacyl-synt_C) and the acyl-transferase (Acyl_transf_1) domains from the 40 *D. discoideum* and 52 *D. purpureum* PKS sequences [117]. The sequences of these domains were aligned using the Muscle program [118] and the alignment manually edited. Phylogenetic analysis was carried out by the UPGMA and neighborjoining methods using the MEGA4 package [119]. The UPGMA and neighbour-joining trees were obtained using the MEGA4 package with distances calculated using the JTT matrix. Trees were similar and were independent of the method used. All file manipulations and data processing were carried out using custom written PERL scripts. All the files can be obtained from the MRC website [120].

**Table S6. The Polyketide Synthases of *D. purpureum* and *D. discoideum*.** The identifying numbers of the genes used in Figure 5A and the corresponding gene predictions at dictyBase at [14], as well as the number of amino acids, is given. Only the genes used in the figure are included. Predictions as well as the number of amino acids are given.

| D.d. Gene | AA | *D. discoideum*  dictyBase  Gene I.D. | D.p. Gene | AA | *D.purpureum* Protein  I.D. Number | D.purpureum dictyBase  Gene I.D. |
| --- | --- | --- | --- | --- | --- | --- |
| 1 | 2603 | DDB_G0275069 | 1 | 2736 | 159351 | DPU_G0070338 |
| 2 | 2604 | DDB_G0275077 | 2 | 2516 | 147207 | DPU_G0074440 |
| 3 | 3010 | DDB_G0270572 | 3 | 3003 | 56948 | DPU_G0059924 |
| 4 | 2499 | DDB_G0283931 | 4 | 2500 | 148809 | DPU_G0064650 |
| 5 | 2499 | DDB_G0284001 | 5 | 2557 | 148808 | DPU_G0064648 |
| 6 | 3097 | DDB_G0293912 | 6 | 2497 | 28523 | DPU_G0064654 |
| 7 | 3078 | DDB_G0293902 | 7 | 1925 | 98568 | DPU_G0058036 |
| 8 | 2531 | DDB_G0288457 | 8 | 2482 | 76035 | DPU_G0065412 |
| 9 | 2485 | DDB_G0271662 | 9 | 2504 | 148800 | DPU_G0064628 |
| 10 | 2542 | DDB_G0291684 | 10 | 2373 | 38743 | DPU_G0061162 |
| 11 | 2552 | DDB_G0291614 | 11 | 2481 | 54214 | DPU_G0072748 |
| 12 | 2931 | DDB_G0295659 | 12 | 2973 | 153779 | DPU_G0056128 |
| 13 | 2931 | DDB_G0271530 | 13 | 2917 | 88429 | DPU_G0055732 |
| 14 | 2924 | DDB_G0271524 | 14 | 2971 | 147609 | DPU_G0054664 |
| 15 | 2514 | DDB_G0295667 | 15 | 2966 | 156533 | DPU_G0062936 |
| 16 | 2514 | DDB_G0271618 | 16 | 2756 | 149063 | DPU_G0066546 |
| 17 | 2513 | DDB_G0271614 | 17 | 2336 | 149064 | DPU_G0066548 |
| 18 | 2512 | DDB_G0271520 | 18 | 2499 | 149067 | DPU_G0066554 |
| 19 | 2837 | DDB_G0271000 | 19 | 2737 | 149068 | DPU_G0066556 |
| 20 | 2380 | DDB_G0287095 | 20 | 2735 | 149069 | DPU_G0066558 |
| 21 | 2471 | DDB_G0287119 | 21 | 2882 | 14379 | DPU_G0060068 |
| 22 | 2551 | DDB_G0272981 | 22 | 2722 | 14334 | DPU_G0075164 |
| 23 | 3174 | DDB_G0273007 | 23 | 2646 | 147748 | DPU_G0055892 |
| 24 | 2999 | DDB_G0281475 | 24 | 2919 | 36677 | DPU_G0057886 |
| 25 | 2998 | DDB_G0272859 | 25 | 2560 | 155872 | DPU_G0061120 |
| 26 | 2779 | DDB_G0282029 | 26 | 2361 | 96815 | DPU_G0062778 |
| 27 | 2778 | DDB_G0282027 | 27 | 2525 | 48793 | DPU_G0056916 |
| 28 | 2658 | DDB_G0292544 | 28 | 2936 | 56925 | DPU_G0059880 |
| 29 | 3075 | DDB_G0290701 | 29 | 2939 | 157297 | DPU_G0064756 |
| 30 | 3106 | DDB_G0290699 | 30 | 2930 | 157296 | DPU_G0064754 |
| 31 | 3101 | DDB_G0290709 | 31 | 2953 | 153367 |  |
| 32 | 2623 | DDB_G0290703 | 32 | 2974 | 94806 | DPU_G0055200 |
| 33 | 3078 | DDB_G0290737 | 33 | 2961 | 148728 | DPU_G0063950 |
| 34 | 3127 | DDB_G0290729 | 34 | 2956 | 158551 | DPU_G0068050 |
| 35 | 3108 | DDB_G0290943 | 35 | 2535 | 153368 | DPU_G0055202 |
| 36 | 3133 | DDB_G0290937 | 36 | 2949 | 153366 |  |
| 37 | 2690 | DDB_G0290469 | 37 | 2952 | 158552 | DPU_G0068052 |
| 38 | 2684 | DDB_G0290467 | 38 | 2493 | 89013 | DPU_G0059302 |
| stlA | 3147 | DDB_G0269364 | 39 | 2392 | 86974 | DPU_G0071454 |
| stlB | 2968 | DDB_G0290853 | 40 | 2527 | 148565 | DPU_G0062782 |
| N.D. |  |  | 41 | 2524 | 152464 | DPU_G0053260 |
| N.D. |  |  | 42 | 2361 | 84141 | DPU_G0067840 |
| N.D. |  |  | 43 | 2532 | 55899 | DPU_G0055516 |
| N.D. |  |  | 44 | 2530 | 158676 | DPU_G0068352 |
| N.D. |  |  | 45 | 2523 | 87448 | DPU_G0075108 |
| N.D. |  |  | 46 | 2420 | 88993 | DPU_G0059218 |
| N.D. |  |  | 47 | 2531 | 146538 | DPU_G0051208 |
| N.D. |  |  | 48 | 2523 | 153476 | DPU_G0055518 |
| N.D. |  |  | 49 | 2535 | 35211 | DPU_G0055514 |
| N.D. |  |  | 50 | 2536 | 154925 | DPU_G0058958 |
| N.D. |  |  | 51 | 2535 | 28418 | DPU_G0063914 |
| N.D. |  |  | 52 | 2534 | 75833 | DPU_G0063918 |

# ATP-binding Cassettee (ABC) Transporters

The ABC super-family is one of the largest in both bacteria and eukaryotes. ABC proteins all have a conserved 200-250 amino acid long domain, the ATP-binding cassette, that gives them their family name. In addition many have transmembrane domains that function in transport of a variety of compounds across membranes. Seven different eukaryotic families have been defined on the basis of sequence homology, domain topology and function. The ABC super-family in *D. discoideum* was analyzed early during genome assembly and annotation [43]. Members of the D. *purpureum* ABC super-family were manually curated and compared in detail to the *D. discoideum* members. Both genomes carry similar numbers of ABC genes overall, but gene “gains” and “losses” can be observed within specific families (Table 1). However, only 58 genes can be considered clear orthologs, the others being paralogs. Most of the variation in gene number is observed in groups of closely related genes belonging to the largest families. These genes probably play partially redundant roles and their number can drift randomly. On the other hand, unique genes tends to be conserved and to present only one ortholog. Orthologous ABCs in these Dictyostelids show 66% amino acid identity, on average, similar to other genes.

**Table S7: Genes in the ABC Superfamily.**

| Gene families | *D.* *discoideum* | *D. purpureum* | Differences* |
| --- | --- | --- | --- |
| ABCA family  Full  Half | 7 genes  5 genes | 6 genes  3 genes | +1  +2 |
| ABCB family  Full  Half | 2 genes  9 genes | 3 genes  9 genes | -1  0 |
| ABCC family | 13 genes | 8 genes | +5 |
| ABCD family  Full  Half | None  3 genes | None  3-4 genes | 0  0 |
| ABCE family | 1 gene | 1 gene | 0 |
| ABCF family | 4 genes | 4 genes | 0 |
| ABCG family  Full  half | 14 genes  9 genes | 17 genes  14 genes | -3  -5 |
| Other** | 6 genes | 6 genes | 0 |
| Total | 73 genes | 75 genes | -2 |

* The difference in gene number is *D.* *discoideum, minus D*. *purpureum.*

**** “Other” ABC genes includes the ArsA homologues but not the SMC group.

**Table S8. ABC transporter genes of *D. purpureum* and *D. discoideum.***

| D. purpureum | | | | D. discoideum | |
| --- | --- | --- | --- | --- | --- |
| Gene | dictyBase  Gene I.D. | Gene I.D. | Protein  I.D. | ortholog | dictyBase  Gene I.D. |
| abcA1 | DPU_G0075094 | e_gw1.89.29.1 | 31990 | abcA1 | DDB_G0291994 |
| abcA2 | DPU_G0061498 | GID1.0046674 | 156020 | abcA2 | DDB_G0267438 |
| abcA3 | DPU_G0067284 | estExt_Genewise1.C_4230003 | 50951 | abcA3 | DDB_G0293436 |
| abcA4 | DPU_G0052882 | estExt_fgeneshDP_pm.C_1200013 | 94555 | abcA4 | DDB_G0274121 |
| abcA5 | DPU_G0069080 | e_gw1.49.29.1 | 29588 | abcA5 | DDB_G0274119 |
| abcA6 | DPU_G0063470 | e_gw1.319.2.1 | 40017 | abcA6 | DDB_G0291245 |
| abcA8 | DPU_G0065562 | e_gw1.376.3.1 | 41231 | abcA8 | DDB_G0271138 |
| abcA9 | DPU_G0075092DPU_G0075090 | e_gw1.89.48.1 + e_gw1.89.35.1 | 32029 31972 | abcA9 | DDB_G0291980 |
| abcB1 | DPU_G0062718 | GID1.0039219 | 148530 | abcB1 | DDB_G0293416 |
| abcB2 | DPU_G0067740 | GID1.0039969 | 149280 | abcB2 | DDB_G0293438 |
| abcB3 | DPU_G0057272 | estExt_Genewise1.C_1950002 | 48852 | abcB3 | DDB_G0291714 |
| abcB4 | DPU_G0061998 | e_gw1.286.4.1 | 39254 | abcB4 | DDB_G0279915 |
| abcB5 | DPU_G0054084 | fgeneshDP_pm.C_scaffold_14000002 | 85995 | abcB5 | DDB_G0292554 |
| abcB6 | DPU_G0052826 | GID1.0042921 | 152232 | abcB6 | DDB_G0282931 |
| abcB7 | DPU_G0062650 | fgeneshDP_pg.C_scaffold_3000082 | 74128 | abcB7 | DDB_G0269720 |
| abcB8 | DPU_G0057426 | fgeneshDP_pg.C_scaffold_198000020 | 80697 | none |  |
| tagA | DPU_G0071860 | GID1.0040785 | 150096 | tagA | DDB_G0293002 |
| tagB | DPU_G0059398 | estExt_fgeneshDP_pg.C_230043 | 96674 | tagB | DDB_G0286119 |
| tagC | DPU_G0059394 | fgeneshDP_pm.C_scaffold_23000019 | 86237 | tagC | DDB_G0286121 |
| tagD |  | N.A. |  | tagD | DDB_G0286123 |
| abcC2 | DPU_G0071556 | GID1.0050317 | 159691 | abcC2 | DDB_G0280055 |
| abcC3 | DPU_G0054216 | estExt_Genewise1Plus.C_1400016 | 55519 | abcC3 | DDB_G0287691 |
| abcC5 | DPU_G0065712 | GID1.0039677 | 148988 | abcC5 | DDB_G0286559 |
| abcC8 | DPU_G0075734 | estExt_Genewise1.C_970030 | 47315 | abcC8 | DDB_G0284867 |
| abcC9 | DPU_G0068514 | fgeneshDP_pg.C_scaffold_47000023 | 76449 | abcC9 | DDB_G0285165 |
| abcC10 | DPU_G0053610 | estExt_Genewise1.C_1310018 | 47873 | abcC10 | DDB_G0280977 |
| abcC12 | DPU_G0059808 | GID1.0038880 | 148191 | abcC12 | DDB_G0280973 |
| abcC16 | -  DPU_G0051826 | e_gw1.106.25.1 instead of GID1.0042473 | 32770  151784 |  |  |
| abcD1 | DPU_G0073960 | GID1.0050952 | 160365 | abcD1 | DDB_G0279917 |
| abcD2 | DPU_G0066722 | estExt_Genewise1.C_4040012 | 50838 | abcD2 | DDB_G0293194 |
| abcD3 | DPU_G0058190 | estExt_Genewise1.C_2080019 | 48982 | abcD3 | DDB_G0279919 |
| abcE1 | DPU_G0053338 | estExt_fgeneshDP_pg.C_1280016 | 97974 | abcE1 | DDB_G0290483 |
| abcF1 | DPU_G0064134 | GID1.0047706 | 157052 | abcF1 | DDB_G0285997 |
| abcF2 | DPU_G0070874 | GID1.0050163 | 159537 | abcF2 | DDB_G0284047 |
| abcF3 | DPU_G0059914 | e_gw1.241.22.1 | 37987 | abcF3 | DDB_G0275637 |
| abcF4 | DPU_G0074414 | estExt_Genewise1Plus.C_80085 | 52338 | abcF4 | DDB_G0267436 |
| abcG1 | DPU_G0072486 | estExt_Genewise1Plus.C_660033 | 54176 | abcG1 | DDB_G0269214 |
| abcG2 | DPU_G0060990 | estExt_fgeneshDP_pg.C_2640005 | 98941 | abcG2 | DDB_G0275689 |
| abcG3 | DPU_G0053626 | estExt_Genewise1Plus.C_1310032 | 55375 | abcG3 | DDB_G0287461 |
| abcG4 | DPU_G0055092 | GID1.0043994 | 153305 | abcG4 | DDB_G0289657 |
| abcG5 | DPU_G0067272 | estExt_Genewise1.C_4220006 | 50946 | abcG5 | DDB_G0281391 |
| abcG7 | DPU_G0055090 | GID1.0043993 | 153304 | abcG7 | DDB_G0289655 |
| abcG8 | DPU_G0070800 | estExt_Genewise1Plus.C_570059 | 53928 | abcG8 | DDB_G0274117 |
| abcG11 | DPU_G0056894 | GID1.0044821 | 154132 | abcG11 | DDB_G0269212 |
| abcG12 | DPU_G0059036 | GID1.0045623 | 154969 | abcG12 | DDB_G0274115 |
| abcG14 | DPU_G0066602 | GID1.0039780 | 149091 | abcG14 | DDB_G0269210 |
| abcG15 | DPU_G0058848 | GID1.0038770 | 148081 | abcG15 | DDB_G0267432 |
| abcG16 | DPU_G0058224 | e_gw1.209.12.1 | 36905 | abcG16 | DDB_G0289331 |
| abcG17 | DPU_G0064052 | e_gw1.333.5.1 | 40327 | abcG17 | DDB_G0273073 |
| abcG18 | DPU_G0060986 | GID1.0046454 | 155800 | abcG18 | DDB_G0275687 |
| abcG20 | DPU_G0053174 | GID1.0043107 | 152418 | abcG20 | DDB_G0267430 |
| abcG21 | DPU_G0059566 | GID1.0045860 | 155206 | abcG21 | DDB_G0269206 |
| abcG22 | DPU_G0062512 | GID1.0037392 | 146703 | abcG22 | DDB_G0270826 |
| abcG23 | DPU_G0062252 | estExt_Genewise1Plus.C_2920005 | 57493 | abcG23 | DDB_G0269026 |
| abcG24 | DPU_G0064878 | estExt_Genewise1Plus.C_3560012 | 58057 | abcG24 | DDB_G0282103 |
| abcG25 | DPU_G0074636 | gw1.82.6.1 | 5674 | none |  |
| abcG26 | DPU_G0065042 | e_gw1.36.6.1 | 28616 | none |  |
| abcG27 | DPU_G0057694 | e_gw1.20.4.1 | 27275 | none |  |
| abcG28 | DPU_G0057746 | e_gw1.20.35.1 | 27204 | none |  |
| abcG29 | DPU_G0053204 | e_gw1.126.15.1 | 33697 | none |  |
| abcG30 | DPU_G0059038 | estExt_fgeneshDP_pg.C_2230012 | 98693 | none |  |
| abcG31 | DPU_G0055496 | GID1.0044153 | 153464 |  |  |
| abcG32 | DPU_G0059586 | GID1.0045870 | 155216 |  |  |
| abcG33 | DPU_G0059396 | GID1.0038839 | 148150 |  |  |
| abcG34 | DPU_G0066902 | fgeneshDP_pg.C_scaffold_41000029 | 76201 |  |  |
| arsA | DPU_G0056324 | estExt_Genewise1.C_1780012 | 48651 | arsA | DDB_G0293528 |
| arsB | DPU_G0067778 | fgeneshDP_pg.C_scaffold_44000048 | 76346 | arsB | DDB_G0289879 |
| abcH1 | DPU_G0074228 | e_gw1.79.40.1 | 31462 | abcH1 | DDB_G0292384 |
| abcH2 | DPU_G0074244 | GID1.0041478 | 150789 | abcH2 | DDB_G0267428 |
| abcH3 | DPU_G0054400 | gw1.143.15.1 | 15107 | abcH3 | DDB_G0275697 |
| abcH4 | DPU_G0074876 | fgeneshDP_pg.C_scaffold_85000020 | 77869 | abcH4 | DDB_G0283479 |
| abcH5 | DPU_G0062188 | fgeneshDP_pg.C_scaffold_29000039 | 75661 | none |  |


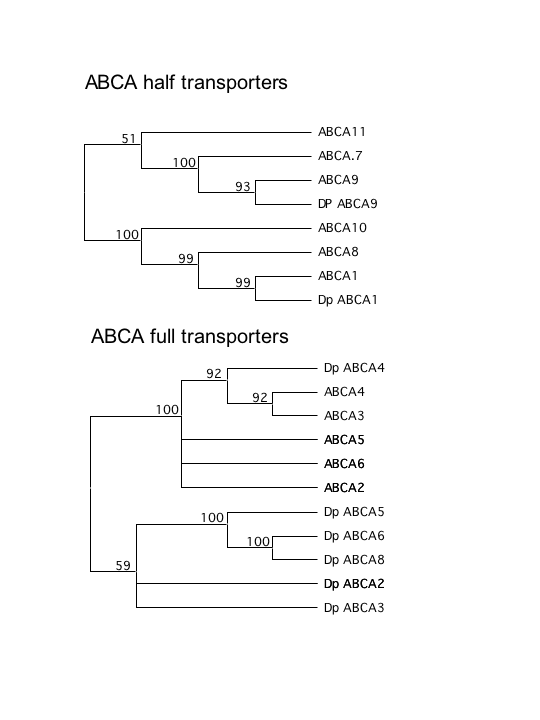


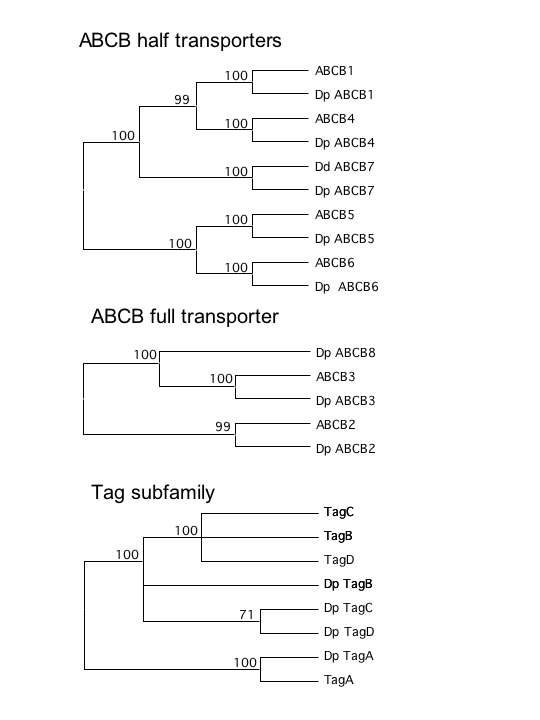


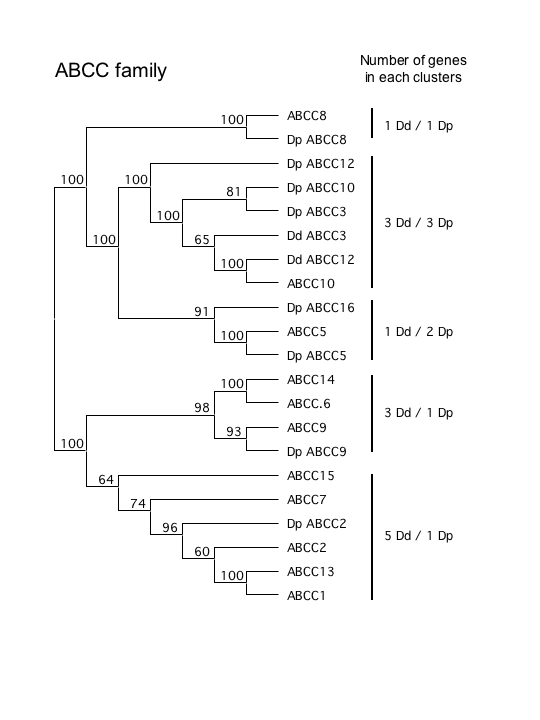


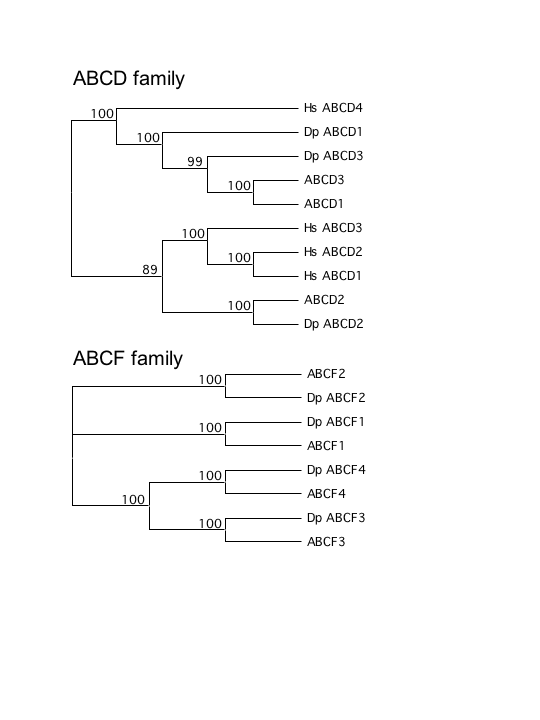


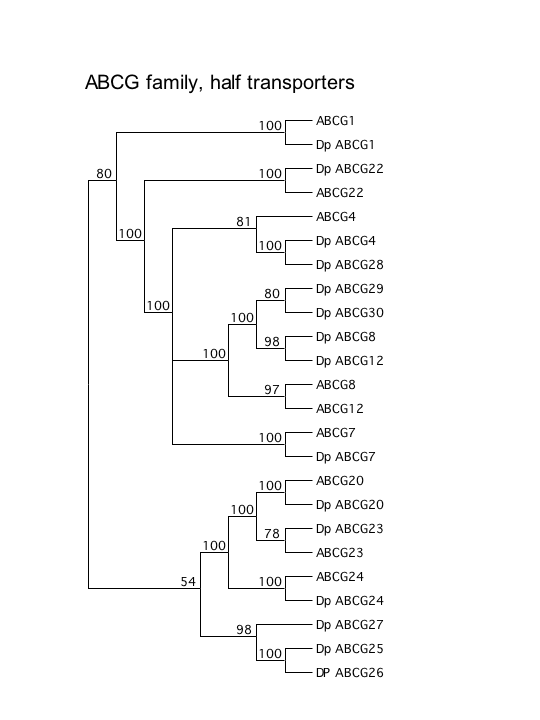


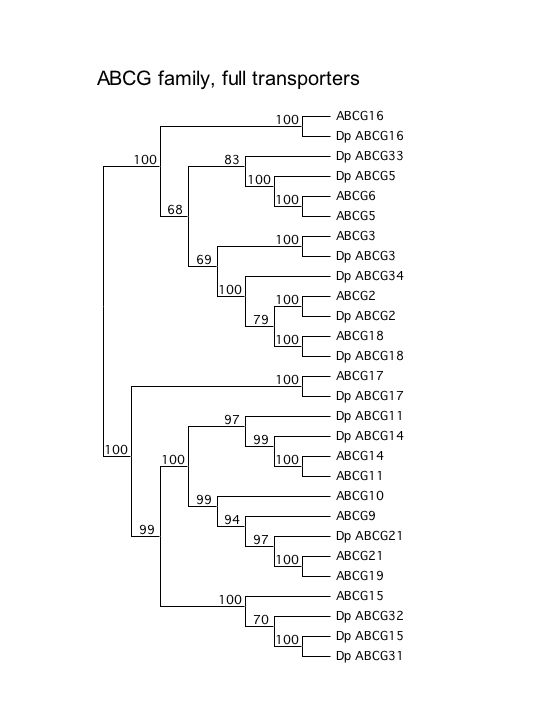


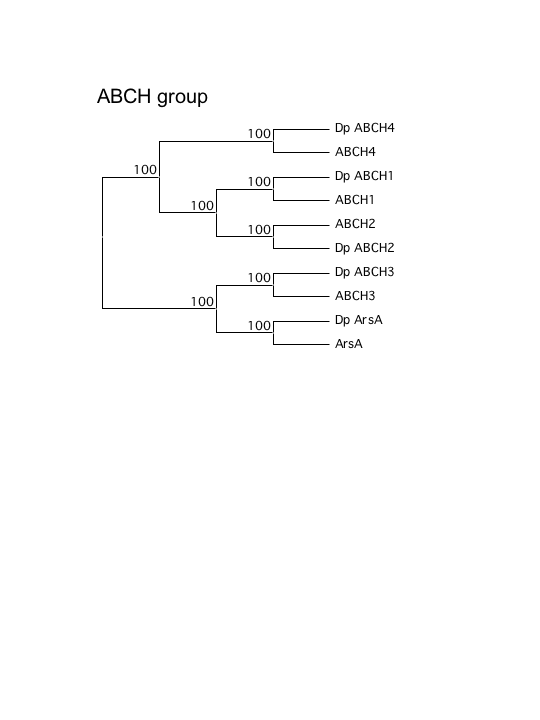
**Figure S10. ABC transporter genes of *D. purpureum* and *D. discoideum*.** Dendrograms of the families of ABC proteins from the *D. discoideum* and *D. purpureum* genomes.

## Protein kinases

*D. purpureum* has a similar complement of protein kinases as compared to *D. discoideum*. There are 262 eukaryotic protein kinases (ePKs; having at least one ePK domain, IPR000719) and 41 atypical protein kinases (aPKs) that are listed in Table S9. The main groups of protein kinases are separated by grey bars, and families within those groups are roughly indicated by various colors. The aPK groups are each colored differently and also separated by grey bars.

**Table S9. The protein kinases of *D. purpureum* and *D discoideum*.** Grey bars indicate major divisions in protein kinase groups (ePK, unclassified, atypical) and colored blocks roughly divide the predicted genes/proteins into protein kinase subfamilies.

| Eukaryotic Protein Kinases (ePK) | | | | | |
| --- | --- | --- | --- | --- | --- |
| D. purpureum | | | D. discoideum | | |
| dictyBase Gene I.D. | Protein I.D. | Proposed Gene Name | Closest paralog dictyBase Gene I.D. | Closest paralog Name | Description from annotation (modified to be consistent with the predicted D. purpureum protein) |
| DPU_G0068090 | 42383 | pkaC | DDB_G0283907 | pkaC | cAMP-dependent protein kinase, AGC group |
| DPU_G0058066 | 8174 | pkaD | DDB_G0277145 | pkaD | cAMP-dependent protein kinase, AGC group |
| DPU_G0072818 | 100116 | pkgD | DDB_G0284029 | pkgD | cAMP-dependent protein kinase, AGC group |
| DPU_G0061370 | 38849 | pkbA1 | DDB_G0268620 | pkbA | AKT/PKB protein kinase, AGC group |
| DPU_G0053200 | 152431 | pkbA2 | DDB_G0268620 | pkbA | AKT/PKB protein kinase, AGC group |
| DPU_G0068224 | 149347 |  | DDB_G0288795 | DDB_G0288795 | AKT family protein kinase, AGC group |
| DPU_G0071804 | 159783 | pkgA | DDB_G0276157 | pkgA | MAST family protein kinase, AGC group |
| DPU_G0064080 | 50356 |  | DDB_G0272282 | DDB_G0272282 | MAST family protein kinase, AGC group |
| DPU_G0054528 | 34584 |  | DDB_G0293276 | DDB_G0293276 | MAST family protein kinase, AGC group |
| DPU_G0064200 | 50393 | drA | DDB_G0278457 | ndrA | NDR family protein kinase, AGC group |
| DPU_G0070842 | 51572 | ndrB | DDB_G0288753 | ndrB | NDR family protein kinase, AGC group |
| DPU_G0074558 | 87338 |  | DDB_G0278845 | DDB_G0278845 | NDR family protein kinase, AGC group |
| DPU_G0053304 | 92065 |  | DDB_G0290859 | DDB_G0290859 | NDR family protein kinase, AGC group |
| DPU_G0058964 | 56680 | pdkA | DDB_G0281471 | pdkA | PDK1 family protein kinase, AGC group |
| DPU_G0056736 | 36122 | pdkB | DDB_G0284489 | pdkB | PDK1 family protein kinase, AGC group |
| DPU_G0060130 | 98847 |  | DDB_G0290157 | pkgB | SGK family protein kinase, AGC group |
| DPU_G0062384 | 39515 |  | DDB_G0277449 | DDB_G0277449 | SGK family protein kinase, AGC group |
| DPU_G0069160 | 58802 | snfA | DDB_G0277905 | snfA | AMP-activated protein kinase alpha subunit, A (AMPK), CAMK group |
| DPU_G0072054 | 90836 | prkag | DDB_G0272542 | prkag | AMP-activated protein kinase gamma subunit (AMPK), CBS (cystathionine-beta-synthase) domain |
| DPU_G0064894 | 157371 | mrkB | DDB_G0285643 | mrkB | similar to SNF1 kinases, MARK kinases, and AMPK kinases |
| DPU_G0058454 | 154713 | mrkC | DDB_G0281895 | mrkC | similar to MARK kinases, SNF1 kinases, and AMPK kinases |
| DPU_G0064378 | 99342 |  | (DDB_G0281895) | (mrkC) | very weak similarity to MARK kinases |
| DPU_G0072942 | 150403 | fhkA | DDB_G0293656 | fhkA | FHA domain-containing protein, RAD53 family protein kinase, CAMK group |
| DPU_G0064488 | 157188 | fhkC | DDB_G0281567 | fhkC | FHA domain-containing protein,similar to mammalian chk2 and yeast RAD53 |
| DPU_G0060718 | 38535 | fhkD | DDB_G0285963 | fhkD | FHA domain-containing protein, RAD53 family protein kinase, CAMK group |
| DPU_G0066926 | 29047 | fhkE | DDB_G0280321 | fhkE | FHA domain-containing protein, RAD53 family protein kinase, CAMK group |
| DPU_G0074230 | 47007 | lkb1 | DDB_G0279629 | lkb1 | CAMKL family, LKB subfamily protein kinase, CAMK group |
| DPU_G0066302 | 157928 | mlkA | DDB_G0279925 | mlkA | CAMK1 family, phosphorylates myosin II regulatory light chain at S13 |
| DPU_G0074364 | 52310 |  | DDB_G0275057 | DDB_G0275057 | CAMK1 family, CAMK group, putative myosin light chain kinase |
| DPU_G0058438 | 49032 |  | DDB_G0282429 | DDB_G0282429 | CAMK1 family, CAMK group, putative myosin light chain kinase |
| DPU_G0064206 | 95599 |  | DDB_G0292624 | DDB_G0292624 | CAMK1 family, CAMK group, putative myosin light chain kinase |
| DPU_G0059860 | 37955 |  | DDB_G0271550 | DDB_G0271550 | CAMK1 family, CAMK group, putative myosin light chain kinase |
| DPU_G0070118 | 51418 | mrkA | DDB_G0292304 | mrkA | CAMKL family, MARK subfamily protein kinase, CAMK group |
| DPU_G0061116 | 57246 | pXi | DDB_G0289119 | pXi | CAMK1 family, CAMK group |
| DPU_G0063552 | 148677 |  | DDB_G0279405 | DDB_G0279405 | CAMKK family, Meta subfamily protein kinase |
| DPU_G0066846 | 76173 |  | DDB_G0279831 | DDB_G0279831 | CAMK1 family, CAMK group |
| DPU_G0071624 | 59197 |  | DDB_G0284661 | DDB_G0284661 | CAMK1 family, CAMK group |
| DPU_G0051654 | 151676 |  | DDB_G0280133 | DDB_G0280133 | CAMKL family, QIK subfamily protein kinase, CAMK group |
| DPU_G0060118 | 49516 |  | DDB_G0277165 | DDB_G0277165 | CAMKL family, BRSK subfamily, CAMK group |
| DPU_G0064538 | 50476 |  | DDB_G0292354 | DDB_G0292354 | TTBK family protein kinase, CK1 group, putative casein kinase I, putative tau-tubulin kinase |
| DPU_G0064746 | 50512 |  | DDB_G0273737 | DDB_G0273737 | Similar to Dictyostelium discoideum casein kinase 1, CK1 group |
| DPU_G0069324 | 96328 | glkA | DDB_G0270218 | glkA | GSK family protein kinase, CMGC group |
| DPU_G0052036 | 26270 | casK | DDB_G0276885 | casK | CK2 family protein kinase, casein kinase II alpha chain, CMGC group |
| DPU_G0072090 | 43974 |  | DDB_G0286481 | DDB_G0286481 | CK2 family protein kinase, CMGC group, putative casein kinase II alpha chain (CK2) |
| DPU_G0064536 | 40649 | clkA | DDB_G0281179 | clkA | CLK family protein kinase, CMGC group |
| DPU_G0067960 | 91498 | sky1 | (DDB_G0275627) | sky1 | SRPK family protein kinase, CMGC group |
| DPU_G0058602 | 98641 |  | DDB_G0274613 | DDB_G0274613 | RING zinc finger-containing protein, CMGC group |
| DPU_G0067110 | 41903 | cdk1 | DDB_G0272813 | cdk1 | CDC2 subfamily protein kinase, CDK family protein kinase, p34-cdc2 protein, CMGC group |
| DPU_G0064348 | 92862 | cdk5 | DDB_G0288677 | cdk5 | CDK family cyclin-dependent kinase, CMGC group |
| DPU_G0067682 | 29225 | cdk7 | DDB_G0285417 | cdk7 | CDK family cyclin-dependent kinase, p34-cdc2 protein, CMGC group |
| DPU_G0068446 | 58676 | cdk8 | DDB_G0267442 | cdk8 | CDK family cyclin-dependent kinase, CMGC group |
| DPU_G0064748 | 157293 | cdk9 | DDB_G0273207 | cdk9-1 | similar cdc2-related protein kinase 7 (CRK7) and other cell division cycle 2-like protein kinases |
| DPU_G0053558 | 18522 | cdk10 | DDB_G0268480 | cdk10 | cell division cycle 2 (CDC2)-like protein kinase; has similarity to PITSLRE proteins and other CDK2s |
| DPU_G0071398 | 74328 | cdk11 | DDB_G0283279 | cdk11 | CDK family cyclin-dependent kinase, CMGC group |
| DPU_G0074212 | 160441 |  | DDB_G0274007 |  | similar to cyclin dependent protein kinases, CMGC group |
| DPU_G0068044 | 42322 |  | DDB_G0278487 | DDB_G0278487 | CDK family protein kinase, CMGC group |
| DPU_G0059916 | 155330 | cdc7 | DDB_G0292152 | cdc7 | similar to S. cerevisiae cell division control protein 7 (CDC7), a S/T kinase required for mitosis and meiosis |
| DPU_G0057164 | 154263 | mps1 | DDB_G0280995 | mps1 | similar to yeast and zebrafish mps1 (monopolar spindle) kinase, a threonine/tyrosine kinase |
| DPU_G0060506 | 38421 | bub1 | DDB_G0292676 | bub1 | BUB family protein kinase, putative mitotic checkpoint protein |
| DPU_G0063456 | 156778 | rio2 | DDB_G0282099 | rio2 | contains RIO1 domain; winged helix DNA-binding domain; RIO1 plays a role in cell cycle progression |
| DPU_G0063564 | 96852 | aurK | DDB_G0279343 | aurK | Aurora family protein kinase |
| DPU_G0071982 | 24715 | nek3 | DDB_G0275241 | nek3 | NEK family protein kinase, putative protein serine/threonine kinase |
| DPU_G0073774 | 44395 |  | DDB_G0283927 | DDB_G0283927 | NEK family protein kinase, putative protein serine/threonine kinase |
| DPU_G0057408 | 154392 |  | DDB_G0270444 | DDB_G0270444 | similar to MAPK, D.d. lacks resdiues required for kinase function; contains WD40 repeats, CMGC group |
| DPU_G0069580 | 97131 | yakA | DDB_G0283605 | yakA | DYRK family protein kinase, CMGC group |
| DPU_G0051834 | 97778 | dyrk1 | DDB_G0277485 | dyrk1 | DYRK family protein kinase, CMGC group |
| DPU_G0051982 | 91968 | dyrk2 | DDB_G0293750 | dyrk2 | DYRK family protein kinase, CMGC group |
| DPU_G0071516 | 54020 | prpf4B | DDB_G0279703 | prpf4B | DYRK family protein kinase, PRP4 - pre-mRNA processing factor 4 homolog B, CMGC group |
| DPU_G0057548 | 146611 | fnkA | DDB_G0275561 | fnkA | FNIP repeat-containing protein kinase, STE group |
| DPU_G0053468 | 74778 |  | (DDB_G0275561) |  | FNIP repeat-containing protein kinase, STE group |
| DPU_G0066830 | 158080 |  | (DDB_G0275561) |  | FNIP repeat-containing protein kinase, STE group |
| DPU_G0066832 | 158081 |  | (DDB_G0275561) |  | FNIP repeat-containing protein,kinase, STE group |
| DPU_G0051700 | 47438 | fnkB | DDB_G0267934 | fnkB | FNIP repeat-containing protein kinase, STE group |
| DPU_G0055908 | 147757 | fnkC | DDB_G0276349 | fnkC | FNIP repeat-containing protein kinase, STE group |
| DPU_G0063898 | 75824 | fnkD | (DDB_G0273129) | fnkD | FNIP containing protein kinase |
| DPU_G0053466 | 147525 | fnkE | DDB_G0275879 | fnkE | FNIP repeat-containing protein kinase, STE group |
| DPU_G0053464 | 74775 |  | (DDB_G0275879) |  | FNIP repeat-containing protein kinase, STE group |
| DPU_G0057098 | 98488 |  | (DDB_G0275879) |  | FNIP repeat-containing protein kinase, STE group |
| DPU_G0054654 | 34641 |  | (DDB_G0275879) |  | FNIP repeat-containing protein kinase, STE group |
| DPU_G0062608 | 146753 |  | (DDB_G0275879) |  | FNIP repeat-containing protein kinase, STE group |
| DPU_G0054652 | 34650 |  | (DDB_G0275879) |  | FNIP repeat-containing protein kinase, STE group |
| DPU_G0053462 | 147523 |  | (DDB_G0275879) |  | FNIP repeat-containing protein kinase, STE group |
| DPU_G0053448 | 147516 |  | (DDB_G0275879) |  | FNIP repeat-containing protein kinase, STE group |
| DPU_G0069014 | 149485 |  | (DDB_G0275879) |  | FNIP repeat-containing protein kinase, STE group |
| DPU_G0061836 | 49896 | mkkA | DDB_G0283265 | mkkA | F-box MEK kinase, STE11 family protein kinase, WD40 repeat, octicosapeptide/Phox/Bem1p domain |
|  | 57414 | mkkB | (DDB_G0283265) | (mkkA) | F-box MEK kinase, STE11 family protein kinase, WD40 repeat, D.p. protein highly similar to mkkA |
| DPU_G0061140 | 155882 |  | (DDB_G0283265) | (mkkA) | weak similarity to DDB_G0283265 (mkkA) |
| DPU_G0072542 | 160010 |  | (DDB_G0283265) | (mkkA) | weak similarity to DDB_G0283265 (mkkA) |
| DPU_G0074488 | 150832 | dst1 | DDB_G0274593 | dst1 | similar to mammalian STK3 and STK4 (MST) kinases |
| DPU_G0055740 | 153614 | dst3 | DDB_G0291267 | dst3 | similar to mammalian STK3 and STK4 (MST) kinases, and other STE20-like kinases |
| DPU_G0066164 | 83651 | dst4 | DDB_G0288071 | dst4 | STE20 family protein kinase, protein kinase, STE group |
| DPU_G0052246 | 33056 |  | DDB_G0278901 | DDB_G0278901 | STE group, putative protein serine/threonine kinase |
| DPU_G0051338 | 91138 | fray1 | DDB_G0278863 | fray1 | FRAY subfamily, STE20 family, STE group |
| DPU_G0060884 | 57173 | fray2 | DDB_G0276577 | fray2 | FRAY subfamily, STE20 family, STE group |
| DPU_G0058244 | 96609 | sepA | DDB_G0276465 | sepA | armadillo repeat-containing protein, STE group, septase |
| DPU_G0061144 | 38722 |  | (DDB_G0276465) | (sepA) | STE group septase (Possible pseudogene) |
| DPU_G0062510 | 96263 | pakA | DDB_G0269166 | pakA | PAKA subfamily, STE20 family protein kinase, p21-activated protein kinase, STE group |
| DPU_G0074072 | 31335 | pakB | DDB_G0276459 | pakB | PAKA subfamily, STE20 family protein kinase, myosin I heavy chain kinase, p21-activated protein kinase |
| DPU_G0074390 | 91113 | pakC | DDB_G0267450 | pakC | PAKA subfamily, STE20 family protein kinase, PH domain, STE group |
| DPU_G0059930 | 155336 | pakD | DDB_G0269696 | pakD | similar to D.d. pakA (Ste20/PAK family); putative p21-activated kinase; calponin-like actin-binding domain |
| DPU_G0072662 | 44150 | pakE | DDB_G0293932 | pakE | PAKL subfamily protein kinase, STE20 family protein kinase, protein kinase, STE group |
| DPU_G0074510 | 150846 | pakF | DDB_G0274409 | pakF | similar to mammalian STK3 and STK4 (MST) kinases |
| DPU_G0056426 | 147802 | pakG | DDB_G0282891 | pakG | PAKL subfamily protein kinase, STE20 family protein kinase, STE group |
| DPU_G0064392 | 157139 |  | (DDB_G0282891) | (pakG) | similar to mammalian STK3 and STK4 (MST) kinases, and other STE20-like kinases |
| DPU_G0065958 | 90051 | pakH | DDB_G0273865 | pakH | PAKL subfamily protein kinase, STE20 family protein kinase, STE group |
| DPU_G0061606 | 92678 | svkA | DDB_G0286359 | svkA | STE20 family protein kinase, YST subfamily, STE group, severin kinase |
| DPU_G0052716 | 93521 | krsA | DDB_G0284181 | krsA | STE group, MST subfamily kinase, STE20 family, krs1-like kinase, protein serine/threonine kinase |
| DPU_G0054724 | 93560 | krsB | DDB_G0267978 | krsB | STE group, MST subfamily kinase, STE20 family, calpain-like cysteine protease, peptidase C2 family protein |
| DPU_G0058304 | 93640 | dst2 | DDB_G0267730 | dst2 | STE20 family protein kinase, STE group |
| DPU_G0059330 | 148117 | mkcA | DDB_G0285427 | mkcA | MKC subfamily, STE20 family protein kinase, STE group |
| DPU_G0068038 | 158544 | mkcB | DDB_G0290723 | mkcB | MKC subfamily, STE20 family protein kinase, STE group |
| DPU_G0052150 | 151903 | mkcC | DDB_G0287853 | mkcC | MKC subfamily, STE20 family protein kinase, STE group |
| DPU_G0059750 | 95210 | mkcD | DDB_G0277413 | mkcD | MKC subfamily, STE20 family protein kinase, STE group |
| DPU_G0070094 | 96000 | mkcE | DDB_G0281649 | mkcE | MKC subfamily, STE20 family protein kinase, STE group |
| DPU_G0057256 | 154313 | mkcF | DDB_G0270102 | mkcF | MKC subfamily, STE20 family protein kinase, STE group |
| DPU_G0068018 | 53558 | erkA | DDB_G0286353 | erkA | ERK subfamily, MAPK family protein kinase |
| DPU_G0063478 | 93783 | erkB | DDB_G0283903 | erkB | ERK subfamily, MAPK family protein kinase |
| DPU_G0060050 | 98833 |  | DDB_G0269152 | mekA | MAP kinase kinase (MAP2K), STE7 family protein kinase, STE group |
| DPU_G0069236 | 52129 | kxcB | DDB_G0293124 | kxcB | DH and PH domain-containing protein, RhoGEF domain-containing protein, STE group |
| DPU_G0062136 | 96792 |  | DDB_G0293184 | DDB_G0293184 | RhoGAP domain-containing protein, STE group |
| DPU_G0070478 | 90653 |  | DDB_G0270146 | DDB_G0270146 | STE group, putative protein serine/threonine kinase |
| DPU_G0063436 | 95528 |  | DDB_G0284251 | DDB_G0284251 | STE group, putative protein serine/threonine kinase |
| DPU_G0072724 | 96158 |  | DDB_G0292350 | DDB_G0292350 | STE group, putative protein serine/threonine kinase |
| DPU_G0054990 | 48249 |  | DDB_G0283821 | DDB_G0283821 | STE group, putative protein serine/threonine kinase |
| DPU_G0051704 | 78437 |  | DDB_G0268550 | DDB_G0268550 | STE group, putative protein serine/threonine kinase |
| DPU_G0051702 | 78436 |  | (DDB_G0268550) |  | STE group STE20 family (Possible fragment of 78437 above) |
| DPU_G0062518 | 146705 |  | (DDB_G0276187) | (pyk4) | N-terminal kinase domain related to STE group |
| DPU_G0074738 | 77798 |  | (DDB_G0276187) | (pyk4) | N-terminal kinase domain related to STE group |
| DPU_G0054656 | 98147 | ifkB | DDB_G0276829 | ifkB | GCN2 subfamily protein kinase, PEK family protein, putative eIF2alpha kinase |
| DPU_G0068358 | 158682 | ifkC | DDB_G0276043 | ifkC | GCN2 subfamily protein kinase, PEK family protein, putative eIF2alpha kinase |
| DPU_G0073386 | 160208 |  | DDB_G0268642 |  | related to GCN2 family; it does not contain a catalytic aspartate |
| DPU_G0065112 | 9913 |  | DDB_G0292734 |  | related to GCN2 family of protein kinases |
| DPU_G0064512 | 83097 |  | DDB_G0283065 | DDB_G0283065 | PEK family protein kinase, putative eIF2alpha kinase |
| DPU_G0054406 | 98108 |  | DDB_G0268642 |  | related to GCN2 family; unlikely to function as a kinase- no catalytic aspartate |
| DPU_G0075598 | 78158 | irlC | DDB_G0270894 | irlC | IRE family the kinase domain is similar to yeast IRE1 kinase required for inositol phototroph |
| DPU_G0057530 | 96238 |  | (DDB_G0270894) |  | IRE family protein kinase |
| DPU_G0061794 | 92689 |  | (DDB_G0270894) |  | IRE family protein kinase similar to yeast IRE1 kinase required for inositol phototrophy; contains SWIM Zn-finger |
| DPU_G0057530 | 96238 |  | (DDB_G0270894) |  | IRE family protein kinase |
| DPU_G0057562 | 96243 | irlD | DDB_G0269632 | irlD | IRE1 kinase required for inositol phototrophy |
| DPU_G0069426 | 58819 | ireA | DDB_G0267650 | ireA | IRE family protein kinase, putative endoribonuclease |
| DPU_G0051864 | 78515 |  | DDB_G0291350 | DDB_G0291350 | NAK (Numb-associated kinase) family protein kinase, putative MPSK |
| DPU_G0055080 | 98203 |  | DDB_G0276461 | DDB_G0276461 | NAK family protein kinase, putative protein serine/threonine kinase |
| DPU_G0065850 | 95721 |  | DDB_G0280111 | DDB_G0280111 | NAK family protein kinase, putative protein serine/threonine kinase |
| DPU_G0070418 | 94041 | tsuA | DDB_G0267962 | tsuA | ULK family protein kinase, fused-like protein kinase, putative protein serine/threonine kinase |
| DPU_G0069554 | 159087 | atg1 | DDB_G0292390 | atg1 | Dictyostelium homolog of yeast atg1, belongs to the ULK (Unc-51-like kinase) family of kinases; required for macroautophagy |
| DPU_G0071576 | 51646 | cct3 | DDB_G0281741 | cct3 | chaperonin containing TCP1 gamma subunit |
| DPU_G0053554 | 55347 | scy1 | DDB_G0267540 | scy1 | SCY1 family encodes highly conserved non-canonical kinases that are believed to function |
| DPU_G0067502 | 158338 | scy2 | DDB_G0270808 | scy2 | SCY1 family encodes highly conserved non-canonical kinases that are believed to function |
| DPU_G0055694 | 153587 | vps15 | DDB_G0282627 | vps15 | similar to human PIK3R4 and yeast VPS15, which regulate VPS34 (hVps34); known to be involved in vacuolar protein sorting |
| DPU_G0056214 | 153832 |  | DDB_G0276527 | DDB_G0276527 | putative protein containing 3 major domains: one RCC1 domain, one protein kinase domain, and one HECT domain |
| DPU_G0054788 | 79620 | slob1 | DDB_G0281863 | slob1 | Slob family protein kinase, FVYE and WH2 domains |
| DPU_G0058362 | 75243 | slob2 | DDB_G0279303 | slob2 | Slob family protein kinase |
| DPU_G0062732 | 75685 |  | (DDB_G0277989) |  | Two ser/thr kinase domains |
| DPU_G0063474 | 82742 | tbck | DDB_G0267760 | tbck | RabGAP/TBC domain-containing protein, TBCK family protein kinase |
| DPU_G0055504 | 98258 |  | DDB_G0274503 | PLK | polo family protein kinase, putative protein serine/threonine kinase |
| DPU_G0057080 | 98486 |  | DDB_G0277071 | DDB_G0277071 | putative protein tyrosine phosphatase, dual specificity |
| DPU_G0073194 | 150424 |  | DDB_G0291436 | DDB_G0291436 | Q86SQ9 Dehydrodolichyl diphosphate synthase (Dedol-PP synthase). Similarity to D.d. not in the kinase domain of the D.p. protein. |
| DPU_G0075140 | 74505 |  | (DDB_G0277989) |  | contains two kinase domains |
| DPU_G0071390 | 147000 | iksA | DDB_G0283109 | iksA | IKS family protein kinase, IQ calmodulin-binding domain, putative transmembrane protein |
| DPU_G0051978 | 97806 |  | DDB_G0293746 | DDB_G0293746 | kinase motif-containing (KMC) protein |
| Tyrosine Kinase like | |  |  |  |  |
| DPU_G0075364 | 91861 | arkA | (DDB_G0289555) | arkA | ARK (ankyrin repeat-containing kinase) family, suppressor of spalten (spnA) mutant; member of the TKL (tyrosine kinase-like) group |
| DPU_G0051824 | 32789 | phg2 | DDB_G0283699 | phg2 | ARK family protein kinase, TKL group |
| DPU_G0064186 | 82991 |  | DDB_G0282963 | DDB_G0282963 | ARK family protein kinase, TKL group |
| DPU_G0065390 | 148930 |  | DDB_G0290471 | DDB_G0290471 | ARK family protein kinase, TKL group, tyrosine kinase-like protein |
| DPU_G0064056 | 95570 | spkA-2 | DDB_G0273531 | spkA-2 | ARK family protein kinase, SAM domain-containing protein, TKL group, stress-activated MEK-like kinase |
| DPU_G0058284 | 75207 |  | DDB_G0267686 | DDB_G0267686 | ARK family protein kinase, RGS domain-containing protein, protein kinase, TKL group |
| DPU_G0070650 | 43457 |  | DDB_G0272092 | DDB_G0272092 | ARK family protein kinase, C2 domain-containing protein, SAM domain-containing protein, TKL group |
| DPU_G0056958 | 16456 |  | DDB_G0278535 |  | ARK family protein kinase; SAM (Sterile alpha motif) domain, TKL group |
| DPU_G0063252 | 99201 |  | DDB_G0278521 | DDB_G0278521 | HH498 subfamily protein kinase, MLK family protein kinase, TKL group |
| DPU_G0058188 | 8978 |  | DDB_G0271682 |  | member of the TKL group; belongs to the ARK (ankyrin repeat-containing kinase) family, although it does not contain ankyrin repeats |
| DPU_G0051656 | 78414 |  | DDB_G0280131 | DDB_G0280131 | ARMK family protein kinase, armadillo repeat-containing protein, TKL group |
| DPU_G0051314 | 147302 |  | DDB_G0278909 | DDB_G0278909 | ARMK family protein kinase, armadillo repeat-containing protein, leucine-rich repeats (LRR), TKL group |
| DPU_G0068520 | 53617 | splA | DDB_G0283385 | splA | CZAK (C-terminal domain of ZakA) family protein kinase, dual-specificity protein kinase, TKL group |
| DPU_G0061764 | 91361 |  | (DDB_G0283385) | (splA) | CZAK (C-terminal domain of ZakA) family protein kinase, dual-specificity protein kinase, TKL group |
| DPU_G0065758 | 92958 |  | (DDB_G0283385) | (splA) | CZAK (C-terminal domain of ZakA) family protein kinase, dual-specificity protein kinase, TKL group |
| DPU_G0074712 | 19597 |  | (DDB_G0283385) | (splA) | weak similarity to splA, CZAK, member of the TKL group |
| DPU_G0058658 | 56632 | splB | DDB_G0285321 | splB | CZAK (C-terminal domain of ZakA) family, TKL group |
| DPU_G0066514 | 25407 |  | (DDB_G0276025) |  | kinase domain similar to TKL group zakA |
| DPU_G0051780 | 78473 |  | (DDB_G0276025) |  | kinase domain similar to TKL group zakA |
| DPU_G0073450 | 46831 |  | (DDB_G0276025) |  | kinase domain similar to TKL group zakA |
| DPU_G0063812 | 40205 |  | DDB_G0285463 | DDB_G0285463 | kinase domain similar to TKL group zakA, putative transmembrane protein, tyrosine kinase-like protein |
| DPU_G0064800 | 4928 |  | DDB_G0283397 | DDB_G0283397 | kinase domain similar to TKL group zakA |
| DPU_G0073902 | 10036 |  | (DDB_G0283397) |  | weak similarity to DDB_G0283397, member of the TKL (tyrosine kinase-like) group and the CZAK (C-terminal domain of ZakA) family |
| DPU_G0071248 | 100013 | pkyA | DDB_G0289001 | pkyA | DPYK family protein kinase, TKL group |
| DPU_G0075472 | 91876 |  | (DDB_G0289001) | (pykA) | DPYK family protein kinase, TKL group |
| DPU_G0069888 | 159186 | drkB | DDB_G0289709 | drkB | DRK (Dictyostelium receptor-like kinase) subfamily, TKL group |
| DPU_G0064592 | 157247 |  | (DDB_G0289709) | (drkB) | DRK (Dictyostelium receptor-like kinase) subfamily, TKL group |
| DPU_G0066438 | 25383 | drkC | DDB_G0281899 | drkC | DRK subfamily protein kinase, E set domain-containing protein, TKL grop |
| DPU_G0052888 | 33484 | drkD | DDB_G0281557 | drkD | DRK subfamily protein kinase, leucine-rich repeats (LRR), TKL group |
| DPU_G0072350 | 97331 |  | DDB_G0270544 | gdt6 | GDT family protein kinase, TKL group, putative transmembrane protein |
| DPU_G0072354 | 77168 |  | DDB_G0278879 | gdt9 | GDT family protein kinase, TKL group, putative transmembrane protein |
| DPU_G0065986 | 157827 | kxcA | DDB_G0289859 | kxcA | LISK (LIM domain and testis-specific kinase) family; contains calmodulin-binding, RhoGEFand PH domains |
| DPU_G0066732 | 41714 | kinX | DDB_G0283391 | kinX | LISK (LIM domain and testis-specific kinase) family protein kinase, TKL group |
| DPU_G0061012 | 155812 |  | DDB_G0289661 | kinY | LISK family; similar to LIM kinases which regulate actin dynamics, TKL group |
| DPU_G0053516 | 152543 |  | DDB_G0271538 | DDB_G0271538 | LISK (LIM domain and testis-specific kinase) family; expressed in pstO cells, TKL group |
| DPU_G0053790 | 152685 | gefX | DDB_G0269298 | gefX | LISK (LIM domain and testis-specific kinase) family; RasGEF nucleotide exchange factor domain, TKL group |
| DPU_G0071580 | 159703 |  | DDB_G0281745 |  | LISK (LIM domain and testis-specific kinase) family, TKL group |
| DPU_G0069360 | 149540 |  | DDB_G0275165 | DDB_G0275165 | LISK family protein kinase, TKL group |
| DPU_G0059584 | 49348 |  | DDB_G0280461 | DDB_G0280461 | LISK family protein kinase, TKL group |
| DPU_G0069936 | 99895 |  | DDB_G0271402 | DDB_G0271402 | LISK family protein kinase, TKL group |
| DPU_G0066116 | 99535 |  | DDB_G0282895 | DDB_G0282895 | MORN repeat-containing protein, TKL group |
| DPU_G0071386 | 96342 | shkA | DDB_G0283267 | shkA | SH2 domain-containing protein, SHK subfamily protein kinase, TKL group |
| DPU_G0067818 | 158466 |  | DDB_G0288617 | shkB | SH2 domain-containing protein, SHK subfamily protein kinase, TKL group |
| DPU_G0054978 | 55744 | shkC | DDB_G0278409 | shkC | SH2 domain-containing protein, SHK subfamily protein kinase, TKL group |
| DPU_G0071724 | 159737 | shkE | DDB_G0290451 | shkE | SH2 domain-containing protein, SHK subfamily protein kinase, TKL group, contains a C-terminal SH2 (Src homology 2) domain |
| DPU_G0061708 | 148427 |  | (DDB_G0290451) | (shkE) | SH2 domain-containing protein, SHK subfamily protein kinase, TKL group |
| DPU_G0052914 | 152279 | rckA | DDB_G0278737 | rckA | RGS (Regulator of G protein Signaling) domain, TKL group |
| DPU_G0070698 | 159478 | pats1 | DDB_G0269250 | pats1 | ROCO family protein kinases; contains LRR, Roc, COR, WD40 domains, TKL group |
| DPU_G0059624 | 95196 | gbpC | DDB_G0291079 | gbpC | RasGEF domain-containing protein, cyclic GMP-binding protein, leucine-rich repeats (LRR), TKL group |
| DPU_G0058498 | 49056 | roco4 | DDB_G0288251 | roco4 | ROCO family protein kinase, leucine-rich repeats(LRR), TKL group |
| DPU_G0063182 | 148631 | roco5 | DDB_G0294533 | roco5 | ROCO family protein kinase, RhoGEF domain, leucine-rich repeats (LRR), PH domain, TKL group |
| DPU_G0065240 | 95686 | roco6 | DDB_G0279417 | roco6 | ROCO family protein kinase, leucine-rich repeats (LRR), PH domain, TKL group |
| DPU_G0059300 | 81310 | roco7 | DDB_G0267472 | roco7 | ROCO family protein kinase, WD40 repeat-containing protein, TKL group |
| DPU_G0058976 | 98684 | roco8 | DDB_G0286127 | roco8 | ROCO family protein kinase, leucine-rich repeats (LRR), DEP domain, TKL group |
| DPU_G0072160 | 150174 |  | DDB_G0288183 | roco9 | ROCO family protein kinase, RhoGAP domain, leucine-rich repeats (LRR), TKL group |
| DPU_G0063892 | 148693 |  | DDB_G0291710 | roco10 | ROCO family protein kinase, RhoGAP, RGS, Kelch domain-containing protein, leucine-rich repeats (LRR), TKL group |
| DPU_G0051594 | 91930 |  | DDB_G0287001 | DDB_G0287001 | LIM-type zinc finger-containing protein, TKL group |
| DPU_G0054076 | 147550 |  | DDB_G0278509 | DDB_G0278509 | protein kinase, TKL group |
| DPU_G0053128 | 152395 |  | DDB_G0288147 | DDB_G0288147 | similar to plant serine/threonine kinases; contains a phorbol ester/diacylglycerol-binding domain, TKL group |
| DPU_G0055048 | 79745 |  | DDB_G0276181 |  | TKL group of protein kinases; contains a PH (pleckstrin homology) domain |
| DPU_G0070696 | 96054 |  | DDB_G0267514 | DDB_G0267514 | TKL group |
| DPU_G0056114 | 94884 |  | DDB_G0272254 | DDB_G0272254 | TKL group, Kelch repeat-containing protein, galactose oxidase domain-containing protein |
| DPU_G0072288 | 87059 |  | DDB_G0278665 | DDB_G0278665 | TKL group |
| DPU_G0060552 | 38450 |  | DDB_G0268876 | DDB_G0268876 | filamin/ABP280 repeat-containing protein, protein kinase,TKL group |
| DPU_G0057092 | 154223 |  | DDB_G0290621 |  | member of the TKL group of protein kinases |
| DPU_G0068556 | 29443 |  | DDB_G0291842 | DDB_G0291842 | WEE1 family protein kinase, putative protein tyrosine kinase |
| DPU_G0058790 | 148051 |  | DDB_G0277539 | DDB_G0277539 | WEE1 family protein kinase, putative protein tyrosine kinase |
| DPU_G0068882 | 158867 |  | DDB_G0291133 | DDB_G0291133 | similar to S. pombe wee1, inhibitor of mitosis through phosphorylation of cdc2 |
| Unclassified kinases and proteins with weak similarity to characterized kinases. | | | | |  |
| DPU_G0072986 | 160136 |  | DDB_G0277989 | DDB_G0277989 | putative kinase |
| DPU_G0067288 | 41971 |  | DDB_G0271366 | DDB_G0271366 | putative kinase |
| DPU_G0060500 | 155584 |  | (DDB_G0277989) |  | putative protein serine/threonine kinase |
| DPU_G0066578 | 53438 |  | DDB_G0269628 | DDB_G0269628 | putative protein serine/threonine kinase |
| DPU_G0053258 | 79056 |  | DDB_G0293958 | DDB_G0293958 | putative protein serine/threonine kinase |
| DPU_G0072554 | 51786 |  | DDB_G0279719 | DDB_G0279719 | putative protein serine/threonine kinase |
| DPU_G0075162 | 147224 |  | DDB_G0293292 | DDB_G0293292 | putative protein serine/threonine kinase |
| DPU_G0063940 | 96861 |  | DDB_G0291918 | DDB_G0291918 | putative protein serine/threonine kinase |
| DPU_G0053292 | 33770 |  | DDB_G0281331 | DDB_G0281331 | putative protein serine/threonine kinase, putative transmembrane protein |
| DPU_G0056750 | 36123 |  | DDB_G0284491 | DDB_G0284491 | Serine/threonine protein kinase |
| DPU_G0051972 | 151859 |  | (DDB_G0279945) |  | DDB_G0279945 Closest paralog in D. discoideum does not encode a kinase domain |
| DPU_G0067366 | 42055 | pan3 | DDB_G0279129 | pan3 | ortholog of PAN3, a member of poly(A)-ribonuclease complex controls poly(A) tail; no catalytic aspartate |
| DPU_G0068318 | 158653 |  | DDB_G0274821 |  |  |

| DPU_G0053256 | 79055 |  | Not Identified |  | very weak similarity to Nek kinases |
| --- | --- | --- | --- | --- | --- |
| DPU_G0058714 | 81086 |  | Not Identified |  | very weak similarity to Nek kinases |
| DPU_G0058712 | 154850 |  | Not Identified |  | very weak similarity to Nek kinases |
| DPU_G0074516 | 160477 |  | Not Identified |  | weak similarity to DDB_G0283391 LISK family protein kinase |
| DPU_G0058100 | 9742 |  | Not Identified |  | weak similarity to DDB_G0270146 |
| DPU_G0072540 | 160009 |  | Not Identified |  | weak similarity to DDB_G0278901. |
| DPU_G0067570 | 13537 |  | Not Identified |  | weak similarity to Oryza sativa PK, XP_482452i |
| DPU_G0073256 | 160171 |  | Not Identified |  | weak similarity to Vibrio S/T kinase |
| DPU_G0061138 | 155881 |  | Not Identified |  | apnB (DDB_G0277247) has similarity to the C-terminus (non-kinase) of this protein |
| DPU_G0058098 | 154589 |  | Not Identified |  | XP_445391 unnamed protein product [Candida glabrata] |
| DPU_G0058102 | 154592 |  | Not Identified |  | CAA42788 ssp31 protein kinase S. cerevisiae |
| DPU_G0066626 | 157967 |  | Not Identified |  | weak hit to E. histolitica P. kinase |
| DPU_G0068098 | 158581 |  | Not Identified |  | weak hit to E. histolitica P. kinase |
| DPU_G0073686 | 150586 |  | Not Identified |  | YP_643722.1| serine/threonine protein kinase [Rubrobacter xylanophilus DSM 9941] |
| DPU_G0073198 | 150426 |  | Not Identified |  | (XP_653371. protein kinase [Entamoeba histolytica HM-1:IMSS].) |
| DPU_G0073674 | 77467 |  | Not Identified |  | (XP_824253.1. Protein kinase Trypanosome brucei.) |
| DPU_G0071050 | 85033 |  | Not Identified |  | (XP_450193. putative serine/threonine-protein kinase ctr1 [Oryza sativa].) |
| DPU_G0069498 | 84604 |  | Not Identified |  | CAJ09507 protein kinase, putative [Leishmania major].) |
| DPU_G0070004 | 76697 |  | Not Identified |  | (XP_725148 hypothetical protein [Plasmodium yoelii yoelii str. 17XNL].) |
| DPU_G0057808 | 27259 |  | Not Identified |  | (BAD28399 putative MAP3K alpha 1 protein kinase [Oryza sativa Japonica Group]. |
| DPU_G0064382 | 83033 |  | Not Identified |  | (XP_727189. calcium-dependent protein kinase [Plasmodium yoelii]) |
|  |  |  |  |  |  |

| Atypical Kinases |  |  |  |  |  |
| --- | --- | --- | --- | --- | --- |
| D. purpureum I.D. | Protein I.D. | D. purpureum name | D. discoideum I.D. | D. discoideum ortholog | Description from D. discoideum |
| DPU_G0055828 | 153663 | dhkA | DDB_G0280961 | dhkA | HisK family protein kinase, histidine kinase |
| DPU_G0071678 | 150045 | dhkB | DDB_G0277845 | dhkB | HisK family protein kinase, histidine kinase |
| DPU_G0066966 | 158106 | dhkC | DDB_G0274191 | dhkC | HisK family protein kinase, histidine kinase |
| DPU_G0053234 | 152450 | dhkD | DDB_G0282289 | dhkD | HisK family protein kinase, histidine kinase |
| DPU_G0060290 | 148245 | dhkE | DDB_G0269204 | dhkE | HisK family protein kinase, histidine kinase |
| DPU_G0069672 | 51346 | dhkF | DDB_G0276143 | dhkF | HisK family protein kinase, histidine kinase |
| DPU_G0059448 | 155144 | dhkG | DDB_G0284045 | dhkG | HisK family protein kinase, histidine kinase |
| DPU_G0051968 | 151857 | dhkH | DDB_G0279913 | dhkH | HisK family protein kinase, histidine kinase |
| DPU_G0064064 | 157014 | dhkI | DDB_G0273475 | dhkI | HisK family protein kinase, histidine kinase |
| DPU_G0055632 | 35308 | dhkJ | DDB_G0277883 | dhkJ | HisK family protein kinase, histidine kinase |
| DPU_G0067300 | 50957 | dhkK | DDB_G0277887 | dhkK | HisK family protein kinase, histidine kinase |
| DPU_G0067586 | 42128 | dhkL | DDB_G0282927 | dhkL | HisK family protein kinase, histidine kinase |
| DPU_G0059468 | 155154 | dhkM | DDB_G0282377 | dhkM | HisK family protein kinase, histidine kinase |
| DPU_G0063058 | 156601 | dokA | DDB_G0274101 | dokA | HisK family protein kinase, histidine kinase |
|  |  |  |  |  |  |
| DPU_G0054288 | 34378 | vwkA | DDB_G0268144 | vwkA | Alpha kinase family, type A von Willebrand factor (VWFA) domain protein |
| DPU_G0054290 | 79436 | vwkB | DDB_G0268144 | (vwkA) | Alpha kinase family, type A von Willebrand factor (VWFA) domain protein |
| DPU_G0059918 | 98812 | ak1 | DDB_G0292150 | ak1 | Alpha kinase family, Arf GTPase activating protein |
| DPU_G0063328 | 95520 | mhkD | DDB_G0282489 | mhkD | Alpha kinase family, MHCK subfamily protein kinase |
| DPU_G0073190 | 94172 | mhkA | DDB_G0291231 | mhkA | Alpha kinase family, MHCK subfamily protein kinase, myosin heavy chain kinase |
| DPU_G0056802 | 48766 | mhkB | DDB_G0289115 | mhkB | Alpha kinase family, MHCK subfamily protein kinase, myosin heavy chain kinase |
| DPU_G0070676 | 51532 | mhkC | DDB_G0290687 | mhkC | Alpha kinase family, MHCK subfamily protein kinase, myosin heavy chain kinase |
| DPU_G0065756 | 83521 |  | Not Identified |  |  |
|  |  |  |  |  |  |
| DPU_G0070838 | 159516 | abkA | DDB_G0288749 | abkA | ABC1 family protein kinase, ABC1-A subfamily protein kinase |
| DPU_G0066890 | 16409 | abkB | DDB_G0281799 | abkB | ABC1 kinase domain; yeast ABC1 essential for electron transfer in the BC(1) complex; E.coli homolog required for ubiquinone biosynthesis |
| DPU_G0065070 | 83271 | abkC | DDB_G0267774 | abkC | ABC1 family protein kinase, ABC1-C subfamily protein kinase |
|  |  |  |  |  |  |
| DPU_G0069658 | 42973 |  | Not Identified | (DDB_G0268748) | putative actin fragmin kinase |
| DPU_G0062264 | 7275 |  | DDB_G0268748 | DDB_G0268748 | putative AFK family protein kinase, putative actin fragmin kinase |
| DPU_G0065292 | 99462 |  | Not Identified | (DDB_G0268748) | putative actin fragmin kinase |
| DPU_G0068144 | 90360 |  | DDB_G0279609 | DDB_G0279609 | AFK family protein kinase, actin fragmin kinase, protein kinase, Atypical group |
|  |  |  |  |  |  |
| DPU_G0060816 | 75494 |  | DDB_G0270170 | DDB_G0270170 | BRD family protein kinase, GTE group protein, bromodomain-containing protein |
| DPU_G0061336 | 98971 |  | DDB_G0293800 | DDB_G0293800 | BRD family protein kinase, BTB/POZ domain-containing protein, GTE group protein, ankyrin repeat-containing protein |
|  |  |  |  |  |  |
| DPU_G0073684 | 150585 |  | DDB_G0279761 | DDB_G0279761 | G11 family protein kinase, protein kinase, Atypical group, putative protein serine/threonine kinase |
|  |  |  |  |  |  |
| DPU_G0052622 | 33395 | rio1 | DDB_G0280431 | rio1 | RIO family protein kinase, RIO1 subfamily protein kinase, protein kinase, Atypical group, putative protein serine/threonine kinase |
| DPU_G0063456 | 156778 | rio2 | DDB_G0282099 | rio2 | RIO family protein kinase, RIO2 subfamily protein kinase, protein kinase, Atypical group, putative protein serine/threonine kinase |
|  |  |  |  |  |  |
| DPU_G0059476 | 49312 | torA | DDB_G0281569 | tor | FRAP subfamily protein kinase, PIKK family protein kinase, phosphatidylinositol 3-kinase-related protein kinase |
| DPU_G0073170 | 150412 | atr1 | DDB_G0291380 | atr1 | ATR subfamily protein kinase, PIKK family protein kinase, phosphatidylinositol 3-kinase-related protein kinase |
| DPU_G0064214 | 157099 | smg1 | DDB_G0275845 | smg1 | PIKK family protein kinase, SMG1 subfamily protein kinase, phosphatidylinositol 3-kinase-related protein kinase |
| DPU_G0073432 | 150499 | dnapkcs | DDB_G0281167 | dnapkcs | DNA-dependent protein kinase (DNAPK) subunit, PIKK family protein kinase, phosphatidylinositol 3-kinase-related protein kinase |
| DPU_G0074948 | 151088 | tra1 | DDB_G0281947 | tra1 | PIKK family protein kinase, TRRAP subfamily protein kinase, phosphatidylinositol 3-kinase-related protein kinase |
|  |  |  |  |  |  |
| DPU_G0059428 | 98741 | taf1 | DDB_G0292242 | taf1 | HAF group protein, TAF1 family protein kinase, TFIID subunit, bromodomain-containing protein |
| DPU_G0074374 | 93469 | tif1 | DDB_G0271372 | DDB_G0271372 | TIF1 family protein kinase, B-box zinc finger-containing protein, Kelch repeat-containing protein, RING zinc finger-containing protein. |

Grey bars indicate major divisions in protein kinase groups (ePK, unclassified, atypical) and colored blocks roughly divide the predicted genes into protein kinase subfamilies.

**G-protein coupled receptors (or seven transmembrane domain receptors)**

G-protein coupled receptors (GPCRs) are subdivided into six major families that, aside from their conserved secondary domain structure, do not share significant sequence similarity across families [121]. Family 1 includes the β-adrenergic, light and odorant receptors, family 2 receptors are activated by large peptides like glucagon or secretin, family 3 comprises the metabotropic glutamate receptors, Ca^2+^-sensing receptors, a group of putative pheromone receptors coupled to G_0_ and the GABA_B_ receptors, family 4 contains pheromone receptors associated with G_i_, family 5 includes the frizzled and smoothened receptors involved in embryonic development and family 6 the *Dictyostelium* cAMP receptors [121]. Table S10 lists all *D. purpureum* and *D. discoideum* members of the six major GPCR families.

Family 1 includes the β-adrenergic, light and odorant receptors; the family 2 receptors are activated by large peptides like glucagon or secretin; the family 3 receptors include the metabotropic glutamate receptors, Ca^2+^-sensing receptors, a group of putative pheromone receptors coupled to G_0_, and the GABA_B_ receptors; and the family 4 receptors include pheromone receptors associated with G_i_; the family 5 receptors include the frizzled and smoothened receptors involved in embryonic development; and the family 6 receptors include the cAMP receptors of the social amoeba [121].

The original analysis of the *D. discoideum* genome revealed 55 genes predicted to encode GPCRs; eight cAMP receptors (cAR) and cAMP receptor-like (crl) proteins, one secretin-like receptor (family 2), 17 GABA_B_-like receptors (family 3), and 25 frizzled-like receptors (family 5)[1, 122]. The analysis of a group 1 and group 2 dictyostelid genome (*Dictyostelium fasciculatum* and *Polysphondylium pallidum*, respectively) resulted in the identification of seven additional putative GPCR genes in *D. discoideum* genome, bringing the number to 62. These belong to two subgroups of orphan receptors. Six putative GPCRs, of which one appears incorrectly predicted because it lacks two transmembrane domains, are related to vertebrate transmembrane protein 145. The seventh putative receptor is highly conserved from amoeba to man and belongs to the GPR89 group of receptors. There are two such receptors in *Arabidopsis thaliana*, GTG1 and GTG2 (GPCR-type G protein 1 and 2) and recently it was reported that they constitute abscisic acid receptors [123].

Most of the receptors in both species were already present in the common ancestor, while some, such as *carB*, appear to have expanded only in *D. discoideum*, or else duplicated versions were lost in *D. purpureum*. Other GPCR’s such as *grlK* and *grlM* appear to have been duplicated from an ancestral gene in both organisms. The data suggest an evolutionary trend in the Dictyostelids towards a higher number and larger diversity of GPCRs. Interestingly, our analysis revealed three exceptions to this observation; there is exactly one ortholog for the GPR89 gene, an ortholog for each *D. discoideum* *crl* gene*,* and the *D. purpureum* genome encodes two latrophilin-like (family 2) receptors, while *D. discoideum* has one full length latrophilin and a second smaller predicted protein that may or may not be functional. Before their description in the Dictyostelids family 2 receptors were thought to be of relatively recent origin, appearing closer to the time of the divergence of animals [124].

**
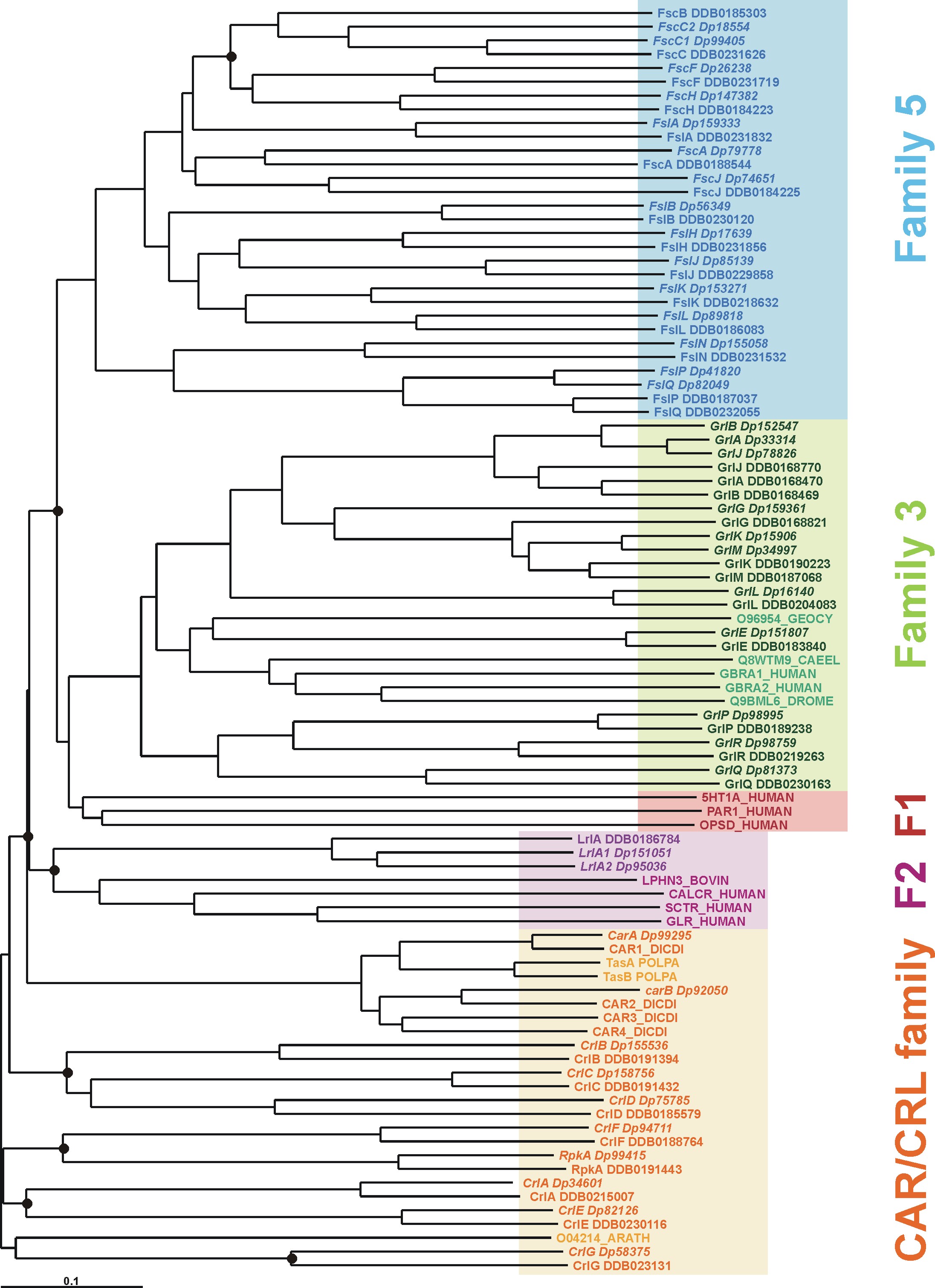
**

**Figure S11. The repertoire of *D. purpureum* and *D. discoideum* G-protein coupled receptors.** A CLUSTALX alignment of the sequences encompassing the seven transmembrane domains of all *D. purpureum* and *D. discoideum* GPCRs, as well as selected GPCRs from other organisms, was used to create a dendrogram with the TreeView program. A black circle marks the innermost node of each branch supported by >50% bootstraps. Protein Ids (Dp) were used for the *D. purpureum,* dictyBase identifiers (DDB) for the *D. discoideum*, gene names for the *Polysphondylium pallidum* and SwissProt entry names for all other receptors. F1: Family 1; F2: Family 2; CAR/CRL: cAMP receptor/cAMP receptor-like. ARATH: *Arabidopsis thaliana*, POLPA: *P. pallidum*, CAEEL: *Caenorhabditis elegans*, DROME: *Drosophila melanogaster*; BOVIN: *Bos taurus*; GEOCY: *Geodia cydonium*; HUMAN: *Homo sapiens*.

**Table S10. The G-protein coupled receptors of *D. purpureum* and *D. discoideum***.

| D. discoideum | | | | D. purpureum | | | | |
| --- | --- | --- | --- | --- | --- | --- | --- | --- |
|  | dictyBase  Gene I.D. | Gene Name(s) | Protein Name | dictyBase  Gene I.D. | Protein ID | Proposed Gene Name | Proposed Protein Name | Evidence* |
| cAMP Receptors: | DDB_G0273397 | *CAR1/carA** | cAR1 | DPU_G0064058 | 99295 | *carA* | cAR1 | KPGIBL |
|  | DDB_G0288179 | *CAR2/carB* | cAR2 | DPU_G0053090 | 92050 | *carB* | cAR2 | BKPGIBL |
|  | DDB_G0277829 | *CAR3/carC* | cAR3 | --- | --- | --- | --- | --- |
|  | DDB_G0277831 | *CAR4/carD* | cAR4 | --- | --- | --- | --- | --- |
|  |  |  |  |  |  |  |  |  |
| cAMP Receptor-like: | DDB_G0280983 | *crlA* | CrlA | DPU_G0054582 | 34601 | *crlA* | CrlA | BKPGIBL |
|  | DDB_G0289395 | *crlB* | CrlB | DPU_G0060408 | 155536 | *crlB* | CrlB | BBL |
|  | DDB_G0283619 | *crlC* | CrlC | DPU_G0068592 | 158756 | *crlC* | CrlC | BGIBL |
|  | DDB_G0283579 | *crlD* | CrlD | DPU_G0063516 | 75785 | *crlD* | CrlD | IBL |
|  | DDB_G0286301 | *crlE* | CrlE | DPU_G0061572 | 82126 | *crlE* | CrlE | BIBL |
|  | DDB_G0290185 | *crlF* | CrlF | DPU_G0054366 | 94711 | *crlF* | CrlF | GIBL |
|  | DDB_G0279599 | *crlG* | CrlG | DPU_G0066748 | 58375 | *crlG* | CrlG | BGIBL |
|  |  |  |  |  |  |  |  |  |
| Serpentine receptor PIP5K family | DDB_G0283615 | *rpkA* | RpkA | DPU_G0064944 | 99415 | *rpkA* | RpkA | BGIBL |
|  |  |  |  |  |  |  |  |  |
| Latrophilin receptor-like (Family 2): | DDB_G0286037 | *lrlA* | LrlA | DPU_G0074886 | 151051 | *lrlA1* | LrlA1 | KPGIBL |
|  |  |  |  | DPU_G0057912 | 95036 | *lrlA2* | LrlA2 | KPGIBL |
|  | DDB_G0286109 | --- | --- | --- | --- | --- | --- | --- |
| GABA(B) or metabotropic glutamate receptor-like (Family 3): | DDB_G0271684 | *grlA* | GrlA | DPU_G0052600 | 33314 | *grlA* | GrlA | BKPGIBL |
|  | DDB_G0271686 | *grlB* | GrlB | DPU_G0053524 | 152547 | *grlB* | GrlB | KPGIBL |
|  | DDB_G0282461 | *grlC* | GrlC | --- | --- | --- | --- | --- |
|  | DDB_G0286895 | *grlD* | GrlD | --- | --- | --- | --- | --- |
|  | DDB_G0291356 | *grlE* | GrlE | DPU_G0051870 | 151807 | *grlE* | GrlE | BKPGIBL |
|  | DDB_G0282175 | *grlF* | GrlF | --- | --- | --- | --- | --- |
|  | DDB_G0272244 | *grlG* | GrlG | DPU_G0070356 | 159361 | *grlG* | GrlG | BKPGIBL |
|  | DDB_G0282459 | *grlH* | GrlH | --- | --- | --- | --- | --- |
|  | DDB_G0272150 | *grlJ* | GrlJ | DPU_G0052598 | 78826 | *grlJ* | GrlJ | KPGIBL |
|  | DDB_G0269386 | *grlK* | GrlK | DPU_G0055180 | 15906 | *grlK* | GrlK | KPGIBL** |
|  | DDB_G0281211 | *grlL* | GrlL | DPU_G0053314 | 16140 | *grlL* | GrlL | BKPGIBL |
|  | DDB_G0286643 | *grlM* | GrlM | DPU_G0055176 | 34997 | *grlM* | GrlM | BKPGIBL** |
|  | DDB_G0283839 | *grlN* | GrlN | --- | --- | --- | --- | --- |
|  | DDB_G0271688 | *grlO* | GrlO | --- | --- | --- | --- | --- |
|  | DDB_G0291095 | *grlP* | GrlP | DPU_G0061448 | 98995 | *grlP* | GrlP | BPGIBL |
|  | DDB_G0287905 | *grlQ* | GrlQ | DPU_G0059522 | 81373 | *grlQ* | GrlQ | BKGIBL |
|  | DDB_G0287681 | *grlR* | GrlR | DPU_G0059524 | 98759 | *grlR* | GrlR | BGIBL |
| Frizzled/Smoothened-like or FSL (Family 5): |  |  |  |  |  |  |  |  |
| *fsl* Group (Frizzled/Smoothened-Like) | DDB_G0284761 | *fslA* | FslA | DPU_G0070308 | 159333 | *fslA* | FslA | BGIBL |
|  | DDB_G0270730 | *fslB* | FslB | DPU_G0057226 | 56349 | *fslB* | FslB | BKGIBL |
|  | DDB_G0274287 | *fslC* | FslC | --- | --- | --- | --- | --- |
|  | DDB_G0269528 | *fslD* | FslD | --- | --- | --- | --- | --- |
|  | DDB_G0288269 | *fslE* | FslE | --- | --- | --- | --- | --- |
|  | DDB_G0288253 | *fslF* | FslF | --- | --- | --- | --- | --- |
|  | DDB_G0288261 | *fslG* | FslG | --- | --- | --- | --- | --- |
|  | DDB_G0274773 | *fslH* | FslH | DPU_G0067680 | 17639 | *fslH* | FslH | BGIBL |
|  | DDB_G0272885 | *fslJ* | FslJ | DPU_G0071614 | 85139 | *fslJ* | FslJ | GIBL |
|  | DDB_G0284729 | *fslK* | FslK | DPU_G0055030 | 153271 | *fslK* | FslK | BGIBL |
|  | DDB_G0284585 | *fslL* | FslL | DPU_G0064402 | 89818 | *fslL* | FslL | BGIBL |
|  | DDB_G0273035 | *fslM* | FslM | --- | --- | --- | --- | --- |
|  | DDB_G0270672 | *fslN* | FslN | DPU_G0059206 | 155058 | *fslN* | FslN | BBL |
|  | DDB_G0278289 | *fslO* | FslO | --- | --- | --- | --- | --- |
|  | DDB_G0286607 | *fslP* | FslP | DPU_G0066984 | 41820 | *fslP* | FslP | BBL |
|  | DDB_G0286609 | *fslQ* | FslQ | DPU_G0061414 | 82049 | *fslQ* | FslQ | BL |
| *fsc* Group (Frizzled/Smoothened-like Sans CRD) | DDB_G0289725 | *fscA* | FscA | DPU_G0055116 | 79778 | *fscA* | FscA | BBL |
|  | DDB_G0282989 | *fscB* | FscB | --- | --- | --- | --- | --- |
|  | DDB_G0292204 | *fscC* | FscC | DPU_G0064888 | 99405 | *fscC1* | FscC1 | BGIBL |
|  |  |  |  | DPU_G0055752 | 18554 | *fscC2* | FscC2 | GIBL |
|  | DDB_G0272240 | *fscD* | FscD | --- | --- | --- | --- | --- |
|  | DDB_G0272306 | *fscE* | FscE | --- | --- | --- | --- | --- |
|  | DDB_G0292064 | *fscF* | FscF | DPU_G0052082 | 26238 | *fscF* | FscF | BKGIBL |
|  | DDB_G0292156 | *fscG* | FscG | --- | --- | --- | --- | --- |
|  | DDB_G0292100 | *fscH* | FscH | DPU_G0052084 | 147382 | *fscH* | FscH | BBL |
|  | DDB_G0292102 | *fscJ* | FscJ | DPU_G0052086 | 74651 | *fscJ* | FscJ | BBL |

*B: Best Bidirectional BLAST Hit; K: KOG; P: Pfam; G: GO; I: InterPro; BL: manual BLAST

** 15906 is likely the orthologue of GrlK (based on BBB 34997 is the orthologue of GrlM; see also the phylogenetic tree)

# Transcription Factors

The overall comparison of the recognizable transcription factors in *D. discoideum* and *D. purpureum* shows gross conservation both in terms of the total number of genes in each of the transcription factor families and also at the protein sequence level for each predicted protein.

# Table S11. The Transcription factors of *D. purpureum* – Summary.

| TF family | Total number in  *D. purpureum* | Total number in  *D. discoideum* |
| --- | --- | --- |
| Zinc finger GATA | 16 | 25 |
| STAT | 4 | 4 |
| Helix-turn-helix | 12 | 15 |
| bZIP | 11 | 19 |
| MADS box | 2 | 4 |
| Myb | 32 | 36 |
| bHLH | 0 | 0 |
| CBF/NF-Y | 3 | 2 |
| SNF5 | 3 | 3 |
| E2F/DP | 1 | 1 |
| WRKY | 2 | 1 |
| Zinc-Cluster TF | 1 | 2 |
| Fork Head activators | 4 | 2 |
| Other unique TFs | 5 | 4 |

Transcription Factor Families (detail).

| *D. purpureum*  Protein I.D. | *D. purpureum*  dictyBase  Gene I.D. | Predicted *D. discoideum*  ortholog | *D. discoideum*  dictyBase  Gene I.D. | Annotation |
| --- | --- | --- | --- | --- |
| BZIP Family |  |  |  |  |
| 91709 | DPU_G0073166 | BzpB | DDB_G0291372 | DIF-signaling |
| 152822 | DPU_G0054020 | BzpA/DimA | DDB_G0278971 | DIF-signaling |
| 96504 | DPU_G0054134 | BzpC | DDB_G0279439 |  |
| 149168 | DPU_G0067176 | BzpD | DDB_G0278379 |  |
| 150254 | DPU_G0072470 | BzpE | DDB_G0269338 |  |
| 92949 | DPU_G0065610 | BzpF | DDB_G0279529 | CREB candidate |
| 41441 | DPU_G0066112 | BzpG | DDB_G0282749 | CREB candidate |
| 156583 | DPU_G0063022 | BzpH | DDB_G0277681 |  |
| N.D. |  | BzpI | DDB_G0290173 |  |
| 93022 | DPU_G0066952 | BzpJ | DDB_G0274993 |  |
| N.D. |  | BzpK | DDB_G0282049 |  |
| 96995 | DPU_G0067218 | BzpL | DDB_G0284023 |  |
| N.D. |  | BzpM | DDB_G0282047 |  |
| N.D. |  | BzpN | DDB_G0286411 | GCN4 homolgue |
| N.D. |  | BzpO | DDB_G0290169 |  |
| N.D. |  | BzpP | DDB_G0290303 |  |
| 98369 | DPU_G0056250 | BzpQ | DDB_G0288705 |  |
| N.D. |  | BzpR | DDB_G0290165 |  |
| N.D. |  | BzpS | DDB_G0290171 |  |
| 150629 | DPU_G0073814 | No bZIP domain |  | mafK |
| 155482 | DPU_G0060208 | No bZIP domain |  |  |
| 42334 | DPU_G0068042 | No bZIP domain |  | mRNA capping enzyme |
| STAT Family |  |  |  |  |
| 57361 | DPU_G0061618 | DstA | DDB_G0281381 |  |
| 157342 | DPU_G0064844 | DstD | DDB_G0275167 |  |
| 147158 | DPU_G0074350 | DstC | DDB_G0268638 |  |
| 51917 | DPU_G0073854 | DstB | DDB_G0293532 |  |
| MADS Box Family |  |  |  |  |
| 92150 | DPU_G0054446 | SrfA | DDB_G0281387 |  |
| N.D. |  | SrfB | DDB_G0282835 |  |
| N.D. |  | SrfC | DDB_G0268920 |  |
| 96067 | DPU_G0070868 | SrfD | DDB_G0283483 |  |
| GATA Family |  |  |  |  |
|  |  | StkA | DDB_G0277147 |  |
| N.D. |  | ComH | DDB_G0280547 |  |
| 51958 | DPU_G0074602 | GtaC | DDB_G0277589 |  |
| N.D. |  | GtaD | DDB_G0286855 |  |
| 96381 | DPU_G0074318 | GtaE | DDB_G0267640 |  |
| 90623 | DPU_G0070286 | GtaF | DDB_G0268792 |  |
| 96721 | DPU_G0060732 | GtaG | DDB_G0270756 |  |
| 55210 | DPU_G0052890 | GtaH | DDB_G0277591 |  |
| 55210 | DPU_G0052890 | GtaI | DDB_G0281661 |  |
| 97535 | DPU_G0074600 | GtaJ | DDB_G0281829 |  |
| 99939 | DPU_G0070362 | GtaK | DDB_G0282811 |  |
| 77444 | DPU_G0073586 | GtaL | DDB_G0285139 |  |
| 82915 | DPU_G0064036 | GtaM | DDB_G0286837 |  |
| 99939 | DPU_G0070362 | GtaN | DDB_G0287057 |  |
| 155826 | DPU_G0061040 | GtaO | DDB_G0289651 |  |
| 159043 | DPU_G0069464 | GtaP | DDB_G0295707 |  |
| 151306 | DPU_G0075458 | GtaQ | DDB_G0286843 |  |
| N.D. |  | GtaR | DDB_G0279331 |  |
| N.D. |  | GtaS | DDB_G0280627 |  |
| N.D. |  | GtaT | DDB_G0280639 |  |
| 83500 | DPU_G0065622 | GtaU | DDB_G0280853 |  |
| N.D. |  | GtaV | DDB_G0281087 |  |
| 83500 | DPU_G0065622 | GtaW | DDB_G0286839 |  |
| 83500 (49.7) | DPU_G0065622 | GtaX | DDB_G0290665 |  |
| N.D. |  | GtaY | DDB_G0279239 |  |
| 91945 | DPU_G0051784 | N.D. |  |  |
| 92966 | DPU_G0065836 | N.D. |  |  |
| 72172 | DPU_G0074742 | N.D. |  |  |
| 81331 | DPU_G0059436 | N.D. |  |  |
| 150926 | DPU_G0074694 | N.D. |  |  |
| 77360 | DPU_G0073296 | N.D. |  |  |
| 82915 | DPU_G0064036 | N.D. |  |  |
| Myb Family | Myb Family |  |  |  |
| 26731 | DPU_G0055346 | MybA | DDB_G0293900 |  |
| 94790 | DPU_G0055088 | MybB | DDB_G0275445 |  |
| 44316 | DPU_G0073496 | MybC | DDB_G0281563 |  |
| (99144) | DPU_G0062828 | MybD | DDB_G0287637 |  |
| 99144 | DPU_G0062828 | MybE | DDB_G0281969 |  |
| (99144) | DPU_G0062828 | MybF | DDB_G0278317 |  |
| 51636 | DPU_G0071282 | MybG | DDB_G0288783 |  |
| 150027 | DPU_G0071646 | MybH | DDB_G0277927 |  |
| 98959 | DPU_G0061114 | MybI | DDB_G0289151 |  |
| 152142 | DPU_G0052522 | MybJ | DDB_G0274463 |  |
| 159839 | DPU_G0071950 | MybK | DDB_G0276877 |  |
| 75617 | DPU_G0061760 | MybL | DDB_G0285373 |  |
| 92812 | DPU_G0063666 | MybM | DDB_G0267636 |  |
| 59013 | DPU_G0070466 | MybN | DDB_G0292782 |  |
| 96894 | DPU_G0064680 | MybO | DDB_G0290787 |  |
| 76145 | DPU_G0066540 | MybP | DDB_G0270560 |  |
| 93079 | DPU_G0067830 | MybQ | DDB_G0289319 |  |
| N.D. |  | MybR | DDB_G0292182 |  |
| 79414 | DPU_G0054254 | MybS | DDB_G0293468 |  |
| N.D. |  | MybT | DDB_G0292180 |  |
| 99744 | DPU_G0068302 | MybU | DDB_G0283953 | only myb domain |
| 99058 | DPU_G0061846 | MybV | DDB_G0279281 |  |
| 156055 | DPU_G0061568 | MybW | DDB_G0270346 |  |
| 152374 | DPU_G0053088 | MybX | DDB_G0288285 |  |
| 97062 | DPU_G0068490 | MybY | DDB_G0284399 |  |
| N.D. |  | MybZ | DDB_G0284103 |  |
| 38843 | DPU_G0061406 | MybAA | DDB_G0268368 |  |
| 76451 | DPU_G0068518 | bdp1 | DDB_G0283405 |  |
| 77715 | DPU_G0074540 | cdc5l | DDB_G0279311 | human CDC5L |
| 29919 | DPU_G0070078 | DDB_G0278179 | DDB_G0278179 |  |
| 159517 | DPU_G0070840 | DDB_G0288747 | DDB_G0288747 |  |
| 26655 | DPU_G0054730 | DDB_G0271272 | DDB_G0271272 |  |
| 156868 | DPU_G0063714 | DDB_G0280079 | DDB_G0280079 |  |
| 148146 | DPU_G0059390 | DDB_G0286135 | DDB_G0286135 |  |
| 149794 | DPU_G0070570 | DDB_G0277033 | DDB_G0277033 |  |
| 85378 | DPU_G0072576 | DDB_G0267638 | DDB_G0267638 |  |
| 150076 | DPU_G0071826 | DDB_G0280705 | DDB_G0280705 |  |
| 4822 | DPU_G0071212 | Isw | DDB_G0292948 |  |
| 26731 | DPU_G0055346 |  |  |  |
| 11392 | DPU_G0073554 |  |  |  |
| 75617 | DPU_G0061760 |  |  |  |
| 155440 | DPU_G0060138 |  |  |  |
| 154597 | DPU_G0058108 |  |  |  |
| 77715 | DPU_G0074540 |  |  |  |
| 55593 | DPU_G0054422 |  |  |  |
| 151821 | DPU_G0051898 |  |  |  |
| Homeobox Family |  |  |  |  |
| 96898 | DPU_G0064692 | WarA | DDB_G0291075 |  |
| 96404 | DPU_G0074442 | Hbx2 | DDB_G0275173 |  |
| 159280 | DPU_G0070146 | DDB_G0284293 | DDB_G0284293 |  |
| 149222 | DPU_G0067434 | DDB_G0286733 | DDB_G0286733 |  |
| N.D. |  | DDB_G0289677 | DDB_G0289677 |  |
| 14027 | DPU_G0061474 | DDB_G0291197 | DDB_G0291197 |  |
| 91524 | DPU_G0068748 | DDB_G0273127 | DDB_G0273127 |  |
| N.D. |  | DDB_G0277505 | DDB_G0277505 |  |
| 92118? | DPU_G0054012 | DDB_G0278225 | DDB_G0278225 |  |
| 24166 | DPU_G0053458 | DDB_G0272967 | DDB_G0272967 |  |
| 39700 | DPU_G0062888 | DDB_G0280473 | DDB_G0280473 |  |
| 160463 | DPU_G0074296 | DDB_G0279257 | DDB_G0279257 |  |
| 91760 | DPU_G0073884 | DDB_G0288975 | DDB_G0288975 |  |
| 154980 | DPU_G0059052 | DDB_G0295787 | DDB_G0295787 |  |
| 91524 | DPU_G0068748 | DDB_G0273645 | DDB_G0273645 |  |
|  |  |  |  |  |

| *D. purpureum*  Protein I.D. | *D. purpureum*  dictyBase Gene I.D. | Predicted *D. discoideum*  ortholog | *D. discoideum*  dictyBase Gene I.D. | Annotation |
| --- | --- | --- | --- | --- |
| Other Families |  |  |  |  |
| 13314 | DPU_G0063568 | DDB_G0279419 | DDB_G0279419 | putative CCAAT-binding transcription factor  putative histone-like transcription factor  N-terminus is similar to CCAAT-box binding transcription factor |
| N.D. |  | DDB_G0282697 | DDB_G0282697 |
| 159469 | DPU_G0070684 | N.D. |  |
| 92261 | DPU_G0055668 | N.D. |  |
| 158603 | DPU_G0068148 | cbfA | DDB_G0279409 | C-module-binding factor  transcription factor jumonji, jmjC domain-containing protein |
| 158872 | DPU_G0068892 | cbfB | DDB_G0293470 |
| 98517 | DPU_G0057322 | E2F | DDB_G0284129 | transcription factor E2F/dimerisation partner (TDP) family protein |
| 86591 | DPU_G0066084 | wrky1 | DDB_G0275267 | putative WRKY transcription factor |
| 13896 | DPU_G0066086 | N.D. |  |
| 157864 | DPU_G0066170 | crtF | DDB_G0278077 | CAR1 Transcription Factor  required for expression of aggregation genes |
| 94676 | DPU_G0053996 |  |  |
| 99541 | DPU_G0066152 | gbfA | DDB_G0288755 |  |
| 151803 | DPU_G0051862 | DDB_G0291348 | DDB_G0291348 | zinc cluster transcription factor |
| N.D. |  | DDB_G0270590 | DDB_G0270590 |
| 55079 | DPU_G0052332 | DDB_G0290591 | DDB_G0290591 | winged helix DNA-binding domain-containing protein  Fork head activators |
| 157374 | DPU_G0064900 | tipA | DDB_G0281561 |
| 49599 | DPU_G0060490 | N.D. |  |
| 97029 | DPU_G0067962 | N.D. |  |
| N.D. |  | DDB_G0277305 | DDB_G0277305 | SNF5/SMARCB1/INI1 family protein, chromatin remodeling complex subunit E (CHE) protein; SNF5 homolog |
| 84008 | DPU_G0067382 | DDB_G0279373 | DDB_G0279373 |
| 100178 | DPU_G0074078 | DDB_G0284591 | DDB_G0284591 |

N.D. No ortholog detected.

**The microfilament system**

In *D. purpureum* 31 actin and actin-related genes and two putative pseudogenes have been identified (Table S12; Figure S12). This contrasts with the 41 genes and 7 pseudogenes of the *D. discoideum* actinome [125]. The *D. purpureum* actinome encodes 10 different proteins (versus 17 in *D. discoideum*). There is a large group of genes (12) encoding an identical protein in turn identical to *D. discoideum* actin 10. In *D. discoideum* a group of 17 genes encodes an identical product, actin 8. A smaller group of 3 genes of *D. purpureum* encodes an identical actin (Actin S) that differs in only one residue from actin 10 (M190L) and in two residues from actin 8 (D2E, M190L). Of the two pseudogenes one appears to be an *act10* gene with a frame shift caused by two missing bases and the other is a truncated gene with additional missing bases in the coding region. One gene (*fia*) encodes a filactin ortholog and 7 genes encode fairly divergent actins without direct orthologs in the *D. discoideum* actinome (Figure S12). Almost all *D. purpureum* actin genes have a single conserved intron position, whereas *D. discoideum* genes are intronless, indicating that all conventional *D. purpureum* actin genes resulted from the duplication of a single ancestral gene. *D. purpureum* has the same set of actin related proteins (Arp’s) as *D. discoideum*, although with variable degree of amino acid sequence similarity (98% for Arp1, versus 58% for Arp8). Among the proteins involved in nucleation of actin filaments, *D. purpureum* has an additional formin gene, *forK*. The C-terminal part of ForK is closer to ForA than to any other formin but lacks the DAD (diaphanous autoinhibitory domain) region. Since the *forK* gene is intronless and the *forA* gene has 4 introns, we speculate that *forK* resulted from the integration and subsequent divergence of a retrotranscribed *forA* gene.

*D. purpureum* has a single gene encoding the G-actin sequestering protein actobindin. In *D. discoideum* the family is represented by a tandem of three genes, two encoding the same protein. *D. purpureum* actobindin is equally similar to both *D. discoideum* actobindins. There are also some differences in the set of profilins. *D. purpureum* has 4 profilin genes, compared to the 3 of *D. discoideum*. Two of them, *proA* and *proC*, have direct orthologs in *D. discoideum*. There is no *proB*, which is probably the result of a duplication of an ancestral *proC* in *D. discoideum* only, but the unique *D. purpureum* genes *proD* and *proE* share two intron positions with *proA*, suggesting that they arose by duplications of this gene in *D. purpureum*.

The ADF/cofilin family also differs between both *Dictyostelium* species. A phylogenetic tree of all ADF domains encoded by the genomes of both species allows distinguishing three major groups (Figure S13).

Hisactophilin is represented by a single protein in *D. purpureum* that is related to the *D. discoideum* hisactophilins A and B. Both annexin genes of *D. discoideum* are represented in *D. purpureum*. Additionally, one more gene in *D. purpureum*, *nxnC*, appears to encode a truncated protein identical to the C-terminus of annexin I. Among the translation elongation factors with actin-binding properties, *D. purpureum* has one single gene encoding an EF1a (two in *D. discoideum*) and two genes encoding EF1b and related proteins (three in *D. discoideum*).

*D. purpureum* contains 10 genes (and two pseudogenes) that are weakly similar to the group of 16 *D.discoideum* ponticulins (ponA, DDB_G0293522; ponB, DDB_G0286247; ponC1, DDB_G0286717; ponC2, DDB_G0286715; ponC3, DDB_G0286721; ponC4, DDB_G0286719; ponC5, DDB_G0286723; ponD, DDB_G0282423; ponE, DDB_G0293634; ponF, DDB_G0286631; ponG/pspA, DDB_G0267412; ponH, DDB_G0286573; ponJ, DDB_G0289919; ponK, DDB_G0268460; ponL, DDB_G0288649; ponM, DDB_G028198). Apart from 4 *D. purpureum* ponticulins and 5 *D. discoideum* *ponC* isoforms that constitute two separate clusters of very similar proteins, in general all ponticulins are equally distant, and direct orthologs between both species cannot be identified. One *D. purpureum* ponticulin (92055) consists of an internal duplication of the central region, and therefore contains two blocks of 6 cysteines.

**Table S12. The microfiliament system of *D. purpureum***.

| D. purpurem Protein ID | D. purpureum  dictyBase Gene I.D. | Proposed  gene name | Proposed D. purpureum  protein name | D. discoideum ortholog | D. discoideum  gene ID | Relevant domains | |
| --- | --- | --- | --- | --- | --- | --- | --- |
| Actins | | | | | | | |
| 73394 | DPU_G0065618 | act10 | Actin 10 | act10 | DDB_G0289811 | Actin | |
| 92832 | DPU_G0063988 | act10 | Actin 10 | act10 | DDB_G0289811 | Actin | |
| 73240 | DPU_G0063614 | act10 | Actin 10 | act10 | DDB_G0289811 | Actin | |
| 92793 | DPU_G0063284 | act10 | Actin 10 | act10 | DDB_G0289811 | Actin | |
| 72679 | DPU_G0056118 | act10 | Actin 10 | act10 | DDB_G0289811 | Actin | |
| 92254 | DPU_G0055648 | act10 | Actin 10 | act10 | DDB_G0289811 | Actin | |
| 54009 | DPU_G0071500 | act10 | Actin 10 | act10 | DDB_G0289811 | Actin | |
| 46519 | DPU_G0071452 | act10 | Actin 10 | act10 | DDB_G0289811 | Actin | |
| 91566 | DPU_G0069822 | act10 | Actin 10 | act10 | DDB_G0289811 | Actin | |
| 91392 | DPU_G0063498 | act10 | Actin 10 | act10 | DDB_G0289811 | Actin | |
| 91170 | DPU_G0053430 | act10 | Actin 10 | act10 | DDB_G0289811 | Actin | |
| 91068 | DPU_G0069316 | act10 | Actin 10 | act10 | DDB_G0289811 | Actin | |
| 90232 | DPU_G0067308 | pseudogene (act10) |  | act10/act8 | DDB_G0289811, DDB_G0269234 | Actin | |
| 92405 | DPU_G0057900 | actS |  | act10/act8 | DDB_G0289811, DDB_G0269234 | Actin | |
| 48914 | DPU_G0057898 | actS |  | act10/act8 | DDB_G0289811, DDB_G0269234 | Actin | |
| 72359 | DPU_G0052194 | actS |  | none |  | Actin | |
| 80660 | DPU_G0057356 | fia | Filactin | fia | DDB_G0284389 | Actin, FLMN | |
| 74418 | DPU_G0073124 | actT |  | none |  | Actin | |
| 74419 | DPU_G0073126 | actU |  | none |  | Actin | |
| 87084 | DPU_G0072460 | actV |  | none |  | Actin | |
| 74427 | DPU_G0073142 | actW |  | none |  | Actin | |
| 78745 | DPU_G0052426 | actX |  | none |  | Actin | |
| 88965 | DPU_G0059126 | actY |  | none |  | Actin | |
| 95143 | DPU_G0059050 | actZ |  | none |  | Actin | |
| 74420 | DPU_G0073128 | pseudogene |  |  |  | Actin | |
| Actin-related proteins | | | | | | | |
| 88548 | DPU_G0056364 | arpA | Arp1 | arpA | DDB_G0288937 | Actin | |
| 46175 | DPU_G0068768 | arpB | Arp2 | arpB | DDB_G0272106 | Actin | |
| 50380 | DPU_G0064164 | arpC | Arp3 | arpC | DDB_G0283755 | Actin | |
| 48789 | DPU_G0056898 | arpD | Arp4 | arpD | DDB_G0280887 | Actin | |
| 154327 | DPU_G0057282 | arpE | Arp5 | arpE | DDB_G0291728 | Actin | |
| 99023 | DPU_G0061582 | arpF | Arp6 | arpF | DDB_G0287007 | Actin | |
| 158218 | DPU_G0067228 | arpG | Arp8 | arpG | DDB_G0287779 | Actin | |
| 155548 | DPU_G0060434 | arpH | Arp11 | arpH | DDB_G0287739 | Actin | |
| Nucleators and WH2 domain-containing proteins | | | | | | | |
| 150581 | DPU_G0073676 | forA | Formin A | forA | DDB_G0279607 | FH2 | |
| 33735 | DPU_G0053232 | forB | Formin B | forB | DDB_G0282297 | FH2 | |
| 50865 | DPU_G0066776 | forC | Formin C | forC | DDB_G0287295 | FH2 | |
| 46912 | DPU_G0073848 | forD | Formin D | forD | DDB_G0282245 | FH2 | |
| 154316 | DPU_G0057262 | forE | Formin E | forE | DDB_G0269626 | FH2 | |
| 148204 | DPU_G0059834 | forF | Formin F | forF | DDB_G0289763 | FH2 | |
| 153797 | DPU_G0056158 | forG | Formin G | forG | DDB_G0277175 | FH2 | |
| 156963 | DPU_G0063964 | forH | Formin H | forH | DDB_G0285589 | FH2 | |
| 36102 | DPU_G0056740 | forI | Formin I | forI | DDB_G0284519 | FH2 | |
| 97202 | DPU_G0070450 | forJ | Formin J | forJ | DDB_G0291378 | FH2 | |
| 99159 | DPU_G0062882 | forK | Formin K | none |  | FH2 | |
| 94694 | DPU_G0054232 | abn | Actobindin | abnA and abnB/abnC | DDB_G0270884, DDB_G0269518, DDB_G0269520 | WH2 | |
| 156917 and 40920 | DPU_G0063804 and DPU_G0065084 | scrA | Scar | scrA | DDB_G0285253 | WH2 | |
| 87126 | DPU_G0072896 | wasA | WASP | wasA | DDB_G0293834 | WH2 | |
| 81492 | DPU_G0059876 |  | WASP-related 1 | DDB_G0272811 | DDB_G0272811 | WH2 | |
| (no model) |  |  | WASP-related 2 | DDB_G0283827 | DDB_G0283827 | WH2 | |
| 74538 | DPU_G0075208 |  | Scar/WASP-related | DDB_G0292878 | DDB_G0292878 | WH2 | |
| 76111 | DPU_G0066048 | wipA | WIPa | wipA | DDB_G0270430 | WH2 | |
| 150808 | DPU_G0074280 |  | WH2 and SH3 containing | DDB_G0269708 | DDB_G0269708 | WH2 | |
| 153613 | DPU_G0055738 |  | WH2 and LIM containing | limD | DDB_G0291251 | WH2, LIM | |
| 79620 | DPU_G0054788 | slob1 | WH2 and FYVE containing prot. kinase | slob1 | DDB_G0281863 | WH2 | |
| (no model) |  |  | WH2 domain-containing | DDB_G0281723 | DDB_G0281723 | WH2 | |
| 91862 | DPU_G0075368 | vasP | VASP | vasP | DDB_G0289541 |  | |
| 98559 | DPU_G0057980 | carmil | CARMIL | carmil | DDB_G0292386 |  | |
| 46589 | DPU_G0071848 | pirA | PIR121 (Scar complex) | pirA | DDB_G0287855 |  | |
| 153775 | DPU_G0056120 | napA | Nap1 (Scar complex) | napA | DDB_G0274519 |  | |
| 87985 | DPU_G0053536 | abiA | Abi2 (Scar complex) | abiA | DDB_G0267956 |  | |
| 88267 | DPU_G0054936 | hspc300 | HSPC300 (Scar complex) | hspc300 | DDB_G0279175 |  | |
| 95056 | DPU_G0058120 | arcA | ARPC1/p41-Arc (Arp2/3 complex) | arcA | DDB_G0277825 |  | |
| 159207 | DPU_G0069920 | arcB | ARPC2/p34-Arc (Arp2/3 complex) | arcB | DDB_G0282813 |  | |
| 58101 | DPU_G0065124 | acrC | ARPC3/p21-Arc (Arp2/3 complex) | acrC | DDB_G0292804 |  | |
| 85721 | DPU_G0062552 | arcD | ARPC4/p20-Arc (Arp2/3 complex) | arcD | DDB_G0269102 |  | |
| 59079 | DPU_G0070712 | arcE | ARPC5/p16-Arc (Arp2/3 complex) | arcE | DDB_G0288319 |  | |
| Profilins | | | | | | | |
| 91884 | DPU_G0075524 | proA | Profilin 1 | proA | DDB_G0287125 | PRO | |
| 90584 | DPU_G0069940 | proC | Profilin 3 | proC | DDB_G0271142 | PRO | |
| 157060 | DPU_G0064146 | proD | Profilin 4 | none |  | PRO | |
| 157061 | DPU_G0064148 | proE | Profilin 5 | none |  | PRO | |
| Cofilin family | | | | | | | |
| 57070 | DPU_G0060220 | cofA | Cofilin 1 | cofA (and cofB) | DDB_G0277833 (DDB_G0291970) | ADF | |
| 155430 | DPU_G0060116 | cofG | Cofilin-related | none |  | ADF | |
| 88870 | DPU_G0058504 | gmfA | Glia maturation factor | gmfA | DDB_G0282595 | ADF | |
| 44803 | DPU_G0073040 | twfA | Twinfilin | twfA | DDB_G0274437 | ADF | |
| 45162 | DPU_G0055352 | coaA | Coactosin | coaA | DDB_G0293898 | ADF | |
| 84230 | DPU_G0068122 | abpE | Abp1 | abpE | DDB_G0273447 | ADF | |
| 84356 | DPU_G0068594 |  | Coactosin-related | DDB_G0283837 | DDB_G0283837 | ADF | |
| 88560 | DPU_G0056402 |  | Coactosin-related | DDB_G0273569_ps, DDBG_0273405_ps | DDB_G0273569, DDBG_0273405 | ADF | |
| 91304 | DPU_G0059796 |  | Coactosin-related | DDB_G0277615 | DDB_G0277615 | ADF | |
| 41497 | DPU_G0066212 |  | Coactosin-related LIM-containing | DDB_G0295683 | DDB_G0295683 | ADF | |
| 48521 | DPU_G0055848 |  | Coactosin-related | DDB_G0290593 | DDB_G0290593 | ADF | |
| 148253 | DPU_G0060306 |  | Coactosin-related | DDB_G0270134, DDB_G0270132 | DDB_G0270134, DDB_G0270132 | ADF | |
| 57957 | DPU_G0064410 |  | Coactosin-related | none |  | ADF | |
| Gelsolin family | | | | | | | |
| 46298 | DPU_G0069786 | vilA | Villidin | vilA | DDB_G0288557 | WD, GEL, VHP | |
| 153835 | DPU_G0056220 | vilB | Protovillin | vilB | DDB_G0276453 | GEL, VHP | |
| (no model) |  | vilC |  | vilC | DDB_G0271058 | GEL, VHP | |
| 51061 | DPU_G0067882 | vilD | Flightless/villin-related | vilD | DDB_G0282725 | GEL, VHP | |
| 99974 | DPU_G0070708 | sevA | Severin | sevA | DDB_G0289327 | GEL | |
| 4927 | DPU_G0067876 | gnrA | GRP125 | gnrA | DDB_G0291125 | GEL | |
| 95982 | DPU_G0069738 | gnrB | Gelsolin-related | gnrB | DDB_G0291536 | GEL | |
| 90830 | DPU_G0071952 | gnrC | Gelsolin-related | gnrC | DDB_G0276571 | GEL | |
| Varia | | | | | | | |
| 82038 |  | corA | Coronin | corA | DDB_G0267382 | WD | |
| 99654 | DPU_G0067358 | corB | Coronin 7 | corB | DDB_G0269388 | WD | |
| 53913 | DPU_G0070758 | cap | CAP | cap | DDB_G0288769 |  | |
| 45371 | DPU_G0058872 | acpA | Cap32 | acpA | DDB_G0267374 |  | |
| 97675 | DPU_G0075686 | acpB | Cap34 | acpB | DDB_G0272104 |  | |
| 51240 | DPU_G0068950 | wdpA | Aip | wdpA | DDB_G0278733 | WD | |
| 94447 | DPU_G0051748 | abpB | ABP34 | abpB | DDB_G0279081 |  | |
| 92199 | DPU_G0054986 | dct | Dynacortin | dct | DDB_G0283767 |  | |
| 38686 | DPU_G0061038 | comA | Comitin | comA | DDB_G0289599 |  | |
| 75958 | DPU_G0064986 | cmr | Comitin-related | cmr | DDB_G0279711 |  | |
| 96363 | DPU_G0073056 | hat | Hisactophilin | hatA and hatB | DDB_G0282141, DDB_G0282143 |  | |
| 97523 | DPU_G0074506 | nxnA | Annexin VII (C1) | nxnA | DDB_G0269160 |  | |
| 12667 | DPU_G0074620 | nxnB | Annexin I | nxnB | DDB_G0284261 |  | |
| 83254 | DPU_G0064926 | nxnC | Annexin I | nxnB | DDB_G0284261 |  | |
| 153006 | DPU_G0054464 |  | Vinculin/alpha-catenin-related | DDB_G0281499 | DDB_G0281499 |  | |
| 49816 | DPU_G0061526 |  | Vinculin/alpha-catenin-related | DDB_G0285939 | DDB_G0285939 |  | |
| 52946 | DPU_G0060246 | efaAI | Elongation factor IA | efaAI | DDB_G0269134 |  | |
| 149388 | DPU_G0068476 | efa1B | Elongation factor IB | efa1B | DDB_G0284035 |  | |
| 93663 | DPU_G0059322 |  | Elongation factor IB-related | DDB_G0286023 | DDB_G0286023 |  | |
| 94172 | DPU_G0073190 | mhkA | MHCKA | mhkA | DDB_G0291231 |  | |
| 87008 | DPU_G0071698 |  |  | DDB_G0278025 | DDB_G0278025 | VHP | |
| 152393 | DPU_G0053124 |  |  | DDB_G0288031 | DDB_G0288031 | VHP | |
| 79698 | DPU_G0054948 |  |  | DDB_G0279449 | DDB_G0279449 | VHP | |
| 54776 and 91894 | 54776 and 91894 |  | Kelch-related | DDB_G0279093 | DDB_G0279093 | KELCH | |
| 91686 | DPU_G0072514 | limC | LimC | limC | DDB_G0277881 | LIM | |
| 32515 | DPU_G0051412 | limD1 | LimD1 | limD1 | DDB_G0287507 | LIM | |
| 94684 | DPU_G0054034 | limE | LimE | limE | DDB_G0279415 | LIM | |
| 99774 | DPU_G0068600 |  | Filamin and HECT containing | DDB_G0283983 | DDB_G0283983 | FLMN | |
| 50592 | DPU_G0065142 |  | LRR and filamin repeat containing | DDB_G0289585 | DDB_G0289585 | FLMN | |
| 91016 | DPU_G0057526 |  | Ponticulin | ponA-M | Ids in text |  | |
| 92054 | DPU_G0053170 |  | Ponticulin | ponA-M | Ids in text |  | |
| 159218 | DPU_G0069944 |  | Ponticulin | ponA-M | Ids in text |  | |
| 97951 | DPU_G0053176 |  | Ponticulin | ponA-M | Ids in text |  | |
| 152487 | DPU_G0053306 |  | Ponticulin | ponA-M | Ids in text |  | |
| 152347 | DPU_G0053044 |  | Ponticulin | ponA-M | Ids in text |  | |
| 153835 | DPU_G0056220 |  | Ponticulin | ponA-M | Ids in text |  | |
| 76224 | DPU_G0067146 |  | Ponticulin | ponA-M | Ids in text |  | |
| 155499 | DPU_G0060344 | pseudogene | Ponticulin | ponA-M | Ids in text |  | |
| 80458 | DPU_G0056822 | pseudogene | Ponticulin | ponA-M | Ids in text |  | |
| 148939 | DPU_G0065406 |  | Ponticulin | ponA-M | Ids in text |  | |
| 92055 | DPU_G0053172 |  | Ponticulin | ponA-M | Ids in text |  | |
| I/LWEQ domain containing proteins | | | | | | | |
| 48677 | DPU_G0056558 | hipA | Sla2/HIP-1 | hipA | DDB_G0285703 | I/LWEQ | |
| 50735 | DPU_G0065934 | talA | Talin A (Filopodin) | talA | DDB_G0290481 | I/LWEQ | |
| 148964 | DPU_G0065666 | talB | Talin B | talB | DDB_G0287505 | I/LWEQ, VHP | |
| 81926 | DPU_G0061044 |  |  | none |  | I/LWEQ | |
| 85023 | DPU_G0071020 |  |  | none |  | I/LWEQ | |
| 158187 | DPU_G0067092 |  |  | none |  | I/LWEQ | |
| Myosins | | | | | | | |
| 48616 | DPU_G0056248 | mhcA | Myosin II | mhcA | DDB_G0286355 | MYO | |
| 157695 | DPU_G0065750 | myoA | Myosin IA | myoA | DDB_G0280039 | MYO | |
| 46917 | DPU_G0073894 | myoB | Myosin IB | myoB | DDB_G0289117 | MYO | |
| 149813 | DPU_G0070600 | myoC | Myosin IC | myoC | DDB_G0276617 | MYO | |
| 34447 | DPU_G0054394 | myoD | Myosin ID | myoD | DDB_G0275447 | MYO | |
| 98367 | DPU_G0056238 | myoE | Myosin IE | myoE | DDB_G0288679 | MYO | |
| 159347 | DPU_G0070330 | myoF | Myosin IF | myoF | DDB_G0289177 | MYO | |
| 149337 | DPU_G0068010 | myoG | Myosin N | myoG | DDB_G0276363 | MYO | |
| 98394 | DPU_G0056360 | myoH | Myosin H | myoH | DDB_G0289447 | MYO | |
| 52275 | DPU_G0073054 | myoI | Myosin VII | myoI | DDB_G0274455 | MYO | |
| 92763 | DPU_G0062932 | myoJ | Myosin J | myoJ | DDB_G0272112 | MYO | |
| 25874 | DPU_G0073006 | myoK | Myosin IK | myoK | DDB_G0274575 | MYO | |
| 159515 | DPU_G0070836 | myoM | Myosin M | myoM | DDB_G0292262 | MYO | |
| Calponin homology domain family | | | | | | |  |
| 92251 | DPU_G0055626 | fimA | Fimbrin | fimA | DDB_G0277855 | 4xCHf | |
| 149579 | DPU_G0069574 |  | Fimbrin-related protein | none |  | 4xCHf | |
| 99698 | DPU_G0067808 | fimB/enlA | Enlazin/Fimbrin-2 | enlA/fimB | DDB_G0276103 | 4xCHf | |
| 14945 | DPU_G0075678 | fimC | Fimbrin-3 | fimC | DDB_G0278545 | 4xCHf | |
| 100074 | DPU_G0072112 | fimD | Fimbrin-4 | fimD | DDB_G0270762 | 4xCHf | |
| 147379 | DPU_G0052076 | frpA | Fimbrin-related protein | frpA | DDB_G0292068 | 2xCHf | |
| 158441 | DPU_G0067794 |  | Fimbrin-related RasGAP | DDB_G0275731 | DDB_G0275731 | 2xCHf | |
| 54845 | DPU_G0051436 | abpD | Interaptin | abpD | DDB_G0287291 | CH1-CH2 | |
| 91322 | DPU_G0060312 | abpC | Filamin | abpC | DDB_G0269100 | CH1-CH2, FLMN | |
| 47922 | DPU_G0053758 | abpA | a-actinin | abpA | DDB_G0268632 | CH1-CH2, SPEC | |
| 80288 | DPU_G0056358 | ctxA | Cortexillin 1 | ctxA | DDB_G0289483 | CH1-CH2 | |
| 52744 | DPU_G0057754 | ctxB | Cortexillin 2 | ctxB | DDB_G0276893 | CH1-CH2 | |
| 73997 | DPU_G0057590 |  |  | DDB_G0294557 | DDB_G0294557 | CH1-CH2 | |
| 32330 | DPU_G0075670 | gxcCC | RacGEF | gxcCC | DDB_G0279123 | CH1-CH2 | |
| 158954 | DPU_G0069126 |  |  | DDB_G0287875 | DDB_G0287875 | CH1-CH2 | |
| 153438 | DPU_G0055442 |  | NAV/Unc-53-related | DDB_G0284113 | DDB_G0284113 | CH1 | |
| 153591 | DPU_G0055702 | gxcAA | RacGEF | gxcAA | DDB_G0282717 | CH1 | |
| 46997 | DPU_G0074182 | gxcDD | RacGEF | gxcDD | DDB_G0279733 | CH1 | |
| 99638 | DPU_G0067266 |  |  | DDB_G0280565 | DDB_G0280565 | CH2 | |
| (no model) |  |  |  | DDB_G0283441 | DDB_G0283441 | CH2 | |
| 13370 | DPU_G0064322 |  | Smoothelin-related | DDB_G0272472 | DDB_G0272472 | CH2 | |
| 100179 | DPU_G0074096 |  |  | DDB_G0277777 | DDB_G0277777 | CH2 | |
| 97668 | DPU_G0075630 |  |  | DDB_G0270220 | DDB_G0270220 | CH3 | |
| 96403 | DPU_G0074430 | ChLim |  | ChLim | DDB_G0267490 | CH3, LIM | |
| 27726 | DPU_G0060288 | gxcB | Trix | gxcB | DDB_G0269424 | CH3 | |
| 96538 | DPU_G0055330 | gxcZ | RacGEF | gxcZ | DDB_G0293928 | CH3, VHP | |
| 4855 | DPU_G0067656 | gxcD | RacGEF | gxcD | DDB_G0267854 | CH3, VHP | |
| 51654 | DPU_G0071592 | gxcBB | RacGEF | gxcBB | DDB_G0277131 | CH3 | |
| 99548 | DPU_G0066182 | gxcA | RacGEF1 | gxcA | DDB_G0277987 | CH3 | |
| 151950 | DPU_G0052204 | gefP | RasGEF P | gefP | DDB_G0281573 | CH3 | |
| 155336 | DPU_G0059930 | pakD | PAK D | pakD | DDB_G0269696 | CH3 | |
| 159654 | DPU_G0071250 |  |  | DDB_G0289033 | DDB_G0289033 | CH3 | |
| 147823 | DPU_G0056468 |  | Transgelin-related | DDB_G0292664 | DDB_G0292664 | CH3 | |
| 156854 | DPU_G0063686 |  |  | DDB_G0269824 | DDB_G0269824 | CH3 | |
| 156272 | DPU_G0062084 |  |  | DDB_G0267974 | DDB_G0267974 | CH3 | |
| 75049 | DPU_G0056458 |  |  | DDB_G0282859 | DDB_G0282859 | CH3 | |
| 14225 | DPU_G0074288 |  |  | none |  | CH3 | |
| 99773 | DPU_G0068590 | eb1 | EB1 | eb1 | DDB_G0283607 | CHe | |
|  |  |  |  |  |  |  | |


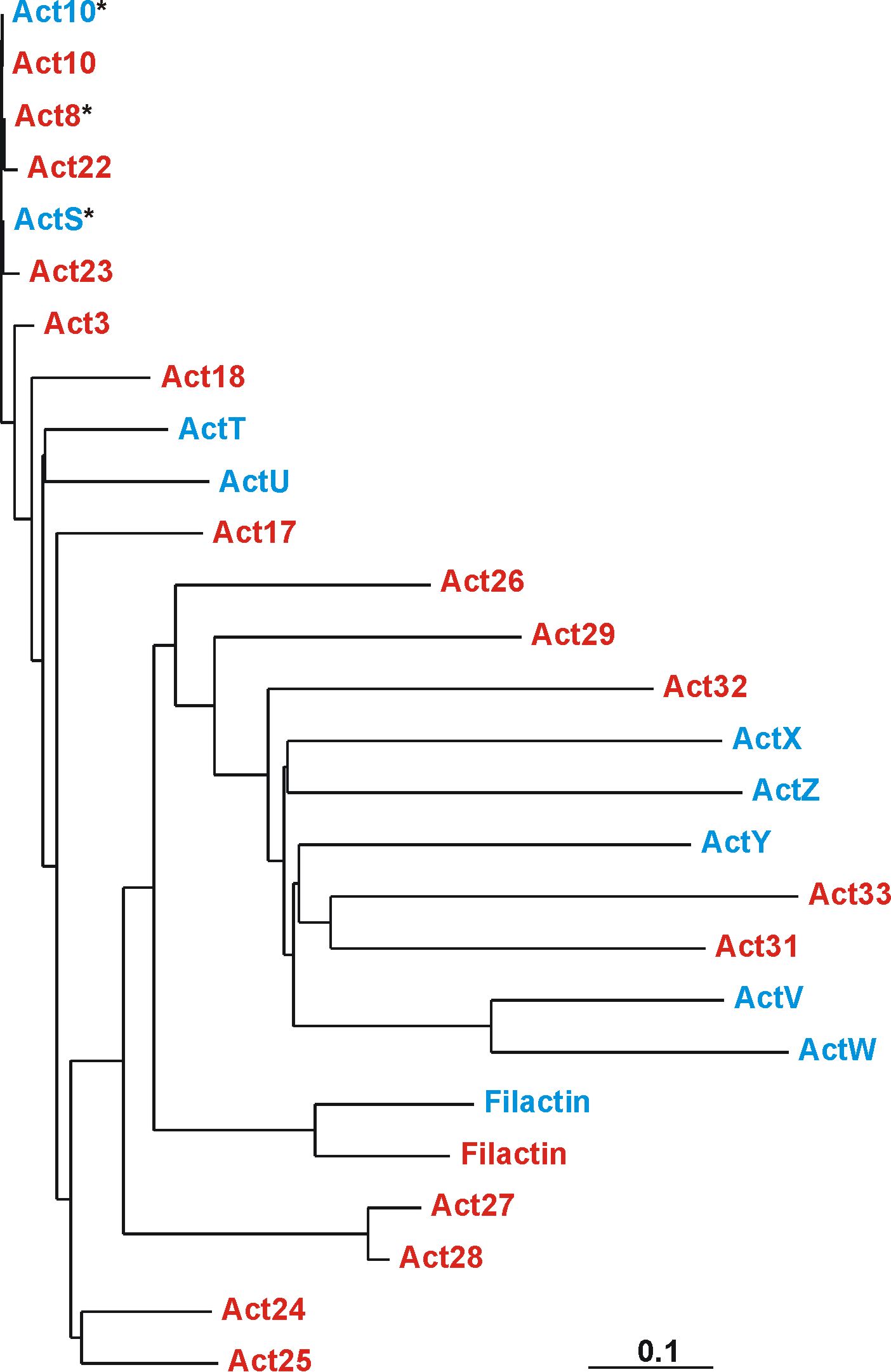


**Figure S12. A phylogenetic tree of the actins of *D. purpureum* and *D. discoideum*.** The phylogenetic tree was constructed using the neighbor joining method. *D. purpureum* and *D. discoideum* actins are in blue and red, respectively. The scale bar represents amino acid substitutions per site. Asterisks indicate groups of identical proteins encoded by different genes.


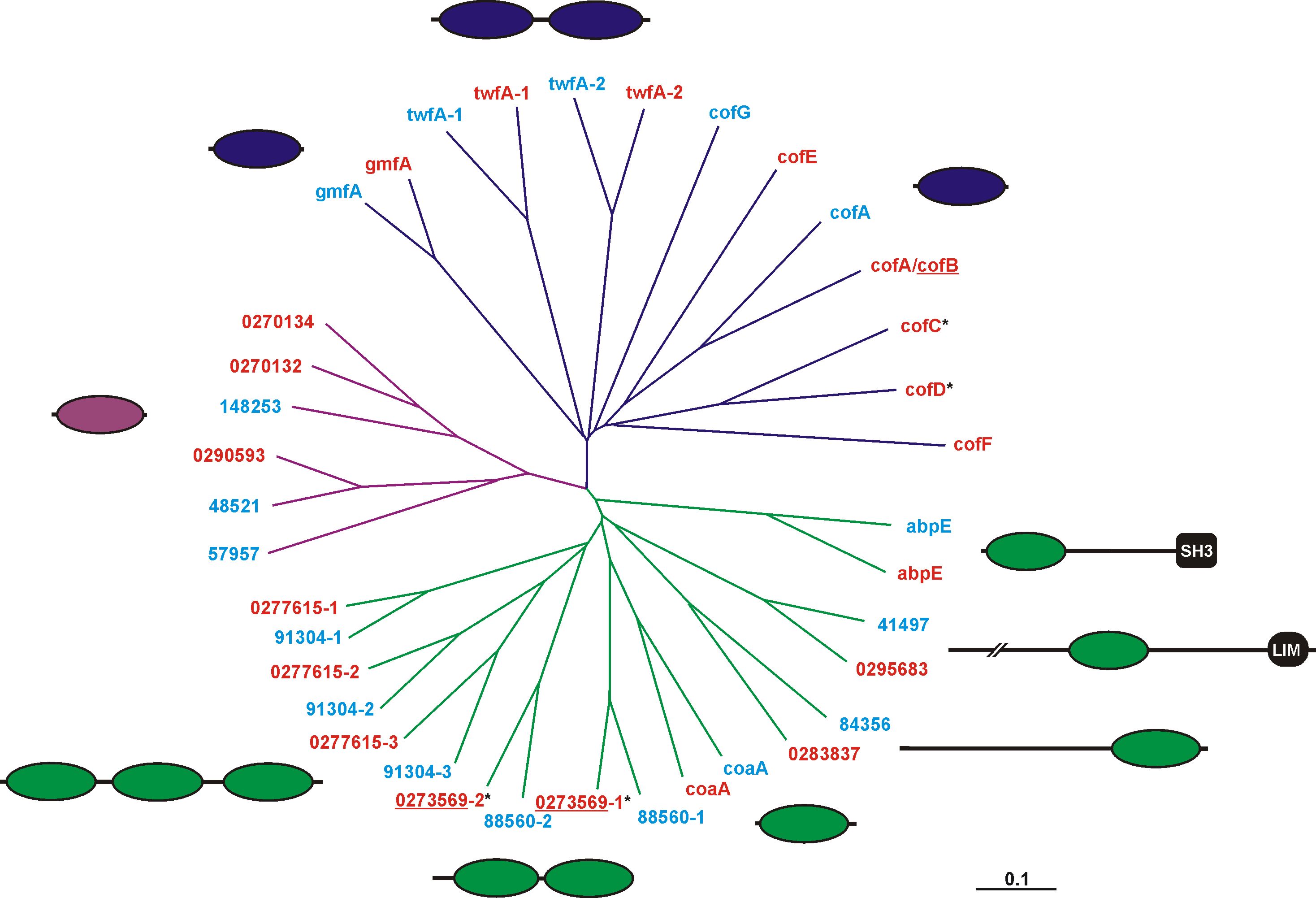


**Figure S13. A phylogenetic tree of the ADF domains in *D. purpureum* and *D. discoideum*.** The phylogenetic tree was constructed using the neighbor joining method. *D. purpureum* and *D. discoideum* proteins are in blue and in red, respectively. Protein identification numbers are given for *D. purpureum*. For *D. discoideum* non-annotated genes are given as gene identification numbers devoid of the DDB_G heading. Only the sequences of the ADF domains were used. Domains were aligned separately in those proteins consisting of duplications or triplications of the domain. The scale bar represents amino acid substitutions per site. Asterisks denote genes present in two copies in the *D. discoideum* genome as the result of the chromosome 2 duplication in the sequenced strain. Underlined are genes annotated as pseudogenes in the *D discoideum* genome. *D. discoideum* *cofA* and *cofB* encode an identical protein but *cofB* is not expressed. The three major groups of ADF domains are indicated by different colors of the tree branches and the domain architecture schemes.

Two proteins containing filamin repeats, filamin and filactin (6 and 2 repeats, respectively) were known previously and are present in both Dictyostelid genomes. We have also identified two proteins with a single filamin repeat each that are present in both Dictyostelid species. One protein, 99774 (DPU_G0068600), contains an N-terminal filamin repeat and a C-terminal HECT domain, which is an E3 ubiquitin ligase domain. Proteins with the same domain architecture can be found in insects and chordates, but there is no information on their functional roles. The other protein, 50592 (DPU_G0065142), consists of leucine-rich repeats and a C-terminal filamin repeat and is apparently unique to *Dictyostelium*.

# Rho signalling

The repertoire of genes encoding proteins that reportedly or presumably participate in Rho signaling is very similar in both *Dictyostelium* species (Tables S13 and S14).

Among the Rho effectors, there are three CIP4-related proteins (PCH family) in both species, after an additional ortholog has been identified in *D. discoideum* in the course of the present study. The class of PI4P 5 kinases has undergone a notable expansion in *D. purpureum*. The *D. discoideum* gene DDB_G0286115 is represented by 9 orthologs in *D. purpureum*, the gene DDB_G0267588 by two orthologs and one more *D. purpureum* gene lacks an ortholog in *D. discoideum*.

**Table S13. Proteins involved in Rho signaling in *D. purpureum*** (summary).

| Protein Class | D. purpureum Genes (No.) | D. discoideum  Genes (No.) | Relevant domain or component^1^ | Closest relative(s) in other organisms |
| --- | --- | --- | --- | --- |
| Rho GTPases | | | | |
| Rac-like | 3 | 6 | GTPase | Rac |
| RhoBTB-like | 1 | 1 | GTPase | RhoBTB |
| Other RhoGTPases^2^ | 14 | 14 | GTPase | Rac (Unique) |
| Dissociation inhibitors | | | | |
| RhoGDI1 | 1 | 1 | RhoGDI | RhoGDI |
| RhoGDI2 | 1 | 1 | RhoGDI | RhoGDI (Unique) |
| Exchange factors |  |  |  |  |
| RhoGEF^3^ | 46 | 47 | RhoGEF (DH) | Mostly unique |
| CZH | 8 | 8 | CZH2 (DHR-2) | DOCK180, MBC, CED-5 |
| Darlin | 1 | 1 |  | SmgGDS, Yeb3p |
| GTPase activating proteins | | | | |
| RhoGAP^3^ | 45 | 46 | RhoGAP | Mostly unique |
| Effectors and other Rho GTPase-binding proteins | | | | |
| Scar complex | 5 | 5 | PIR121 | WAVE complex |
| WASP | 1 | 1 | CRIB | WASP |
| WASP-related | 2 | 2 | CRIB | WASP/WAVE (Unique) |
| PAK^4^ | 8 | 9 | CRIB | PAK kinases |
| Gelsolin-related | 1 | 1 | CRIB |  |
| Formins | 11 | 10 | GBD | Formins |
| IQGAP-related | 4 | 4 | GRD | IQGAP |
| PCH family | 3 | 3 | HR1 | CIP4, Toca-1, Cdc15p |
| Lipid kinases^5^ | 25 | 15 | p85 | Class I PI-3-kinases (p110), PI-4-P5K, DGK |
| Phospholipases^5^ | 5 | 5 |  | Phospholipase C, D1/2 |
| NADPH oxidase | >5 | >5 | p67^phox^ | NADPH oxidase |
| Exocyst complex | 8 | 8 | Sec3, Exo70 | Exocyst complex |
| LIS1 | 1 | 1 |  | LIS1, Pac1 |
| LimE | 1 | 1 |  | LIM proteins |

The table is based on [53]. Note that for many of the protein families included in the table participation in Rho signaling has been documented in some species but not in others, therefore a role in *Dictyostelium* should be taken as tentative.

^1^Relevant domains or components refer, apart from the GTPase, to those involved in interactions with the Rho GTPase if they have been determined. Abbreviations of the relevant domains: CRIB, Cdcd42 and Rac interactive binding (also known as PBD, p21-binding domain); CZH2, CDM-zizimin homology 2 domain (also known as DHR-2, Dock homology region 2); GAP, GTPase activating protein; GBD, Rho GTPase-binding domain of formins; GDI, guanine nucleotide dissociation inhibitor; GEF, guanine nucleotide exchange factor (the RhoGEF domain is also known as DH, Dbl homology domain); GRD, RasGAP-related domain; HR1, protein kinase C-related kinase homology region 1.

^2^ In *D.discoideum* this group includes RacC to E, RacG to Q and the pseudogene RacK. In *D. purpureum* this group includes RacC to E, RacG, RacH, RacJ, RacL, RacP and RacR to RacW.

^3^ Three genes encode proteins with both RhoGAP and RhoGEF domains.

^4^ In *D. discoideum* PakH exists as two copies, one on the chromosome 2 duplication

^5^ Includes 6 PI3 kinases (p110 subunit), one DAG kinase and 9 (in *D. discoideum*) or 18 (in *D purpureum*) PI-4-P5 kinase genes. The p85 regulatory subunit of the PI3 kinase, which in vertebrates interacts with Cdc42 and Rac1, is apparently missing in *D. discoideum* and *D. purpureum*. This does not exclude a potential regulation by Rho GTPases through other mechanisms, therefore PI 3-kinases have not been excluded from the table.

^6^ Includes a single phospholipase C as well four phospholipase D proteins related to mammalian PLD1 and PLD2.

**Table S14. Proteins involved in Rho signaling in *D. purpureum*** (detail).

| D. purpureum  Protein ID | D. purpureum  dictyBase Gene I.D. | Proposed  D. purpureum  gene name | D. discoideum  dictyBase Gene ID | D. discoideum  Ortholog |  |
| --- | --- | --- | --- | --- | --- |
| Rho GTPases | | | | |  |
| 92985 | DPU_G0066198 | *rac1a1* | DDB_G0277869 | *rac1a* |  |
| 168754# | DPU_G0057068 | *rac1a2* | DDB_G0277869 | *rac1a* |  |
| 50894 | DPU_G0066986 | *racA* | DDB_G0286555 | *racA* |  |
| 168757# | DPU_G0061650 | *racB* | DDB_G0279605 | *racB* |  |
| 55703 | DPU_G0054880 | *racC* | DDB_G0293526 | *racC* |  |
| 93635# | DPU_G0058262 | *racD* | DDB_G0291976 | *racD* |  |
| 98135 | DPU_G0054576 | *racE* | DDB_G0280975 | *racE* |  |
| 75463 | DPU_G0060756 | *racG* | DDB_G0269178 | *racG* |  |
| 41217 | DPU_G0065542 | *racH* | DDB_G0269240 | *racH* |  |
| 147341 | DPU_G0051392 | *racJ* | DDB_G0292560 | *racJ* |  |
| 52376 | DPU_G0075220 | *racL* | DDB_G0292816 | *racL* |  |
| 89172 | DPU_G0060224 | *racP* | DDB_G0285453 | *racP* |  |
| 80321# | DPU_G0056546 | *racR* |  |  |  |
| 81024# | DPU_G0058588 | *racS* |  |  |  |
| 149292 | DPU_G0067766 | *racT* |  |  |  |
| 81474 | DPU_G0059740 | *racU* |  |  |  |
| 81475 | DPU_G0059742 | *racV* |  |  |  |
| 158834 | DPU_G0068822 | *racW* |  |  |  |
| RhoGDI | | | | |  |
| 95353 | DPU_G0061434 | *rdiA* | DDB_G0291077 | *rdiA* |  |
| 78427 | DPU_G0051684 | *rdiB* | DDB_G0280049 | *rdiB* |  |
| RhoGEF (DH) | | | | |  |
| 99548 | DPU_G0066182 | *gxcA* | DDB_G0277987 | *gxcA* |  |
| 27726 | DPU_G0060288 | *gxcB* | DDB_G0269424 | *gxcB* |  |
| 156926 | DPU_G0063820 | *gxcC* | DDB_G0284845 | *gxcC* |  |
| 4855 | DPU_G0067656 | *gxcD* | DDB_G0267854 | *gxcD* |  |
| 81422 | DPU_G0059622 | *gxcE* | DDB_G0291085 | *gxcE* |  |
| 85191 | DPU_G0071806 | *gxcF* | DDB_G0282475 | *gxcF* |  |
| 153584 | DPU_G0055688 | *gxcG* | DDB_G0282073 | *gxcG* |  |
| 77025 | DPU_G0071818 | *gxcH* | DDB_G0288377 | *gxcH* |  |
| 77030 | DPU_G0071828 | *gxcI* | DDB_G0288383 | *gxcI* |  |
| 45161 | DPU_G0055350 | *gxcJ* | DDB_G0293978 | *gxcJ* |  |
| 148367 | DPU_G0061232 | *gxcK* | DDB_G0291007 | *gxcK* |  |
| 83267 | DPU_G0064952 | *gxcL* | DDB_G0290023 | *gxcL* |  |
| 148839 | DPU_G0064972 | *gxcM* | DDB_G0272372 | *gxcM* |  |
| 96166 | DPU_G0072848 | *gxcN* | DDB_G0277017 | *gxcN* |  |
| 97823 | DPU_G0052190 | *gxcO* | DDB_G0293396 | *gxcO* |  |
| 41248 | DPU_G0065600 | *gxcP* | DDB_G0285859 | *gxcP* |  |
| 155148 | DPU_G0059456 | *gxcQ* | DDB_G0284501 | *gxcQ* |  |
| 89777 | DPU_G0064124 | *gxcR* | DDB_G0285303 | *gxcR* |  |
| 82447 | DPU_G0062438 | *gxcS* | DDB_G0280087 | *gxcS* |  |
| 84596 | DPU_G0069476 | *gxcT* | DDB_G0269610 | *gxcT* |  |
| 26275 | DPU_G0052094 | *gxcU* | DDB_G0291996 | *gxcU* |  |
| 33732 | DPU_G0053238 | *gxcV* | DDB_G0282271 | *gxcV* |  |
| 153285 | DPU_G0055058 | *gxcW* | DDB_G0278147 | *gxcW* |  |
| 158102 | DPU_G0066958 | *gxcX* | DDB_G0274889 | *gxcX* |  |
| 39928 | DPU_G0063332 | *gxcY* | DDB_G0293266 | *gxcY* |  |
| 96538 | DPU_G0055330 | *gxcZ* | DDB_G0293928 | *gxcZ* |  |
| 153591 | DPU_G0055702 | *gxcAA* | DDB_G0282717 | *gxcAA* |  |
| 51654 | DPU_G0071592 | *gxcBB* | DDB_G0277131 | *gxcBB* |  |
| 32330 | DPU_G0075670 | *gxcCC* | DDB_G0279123 | *gxcCC* |  |
| 46997 | DPU_G0074182 | *gxcDD* | DDB_G0279733 | *gxcDD* |  |
| 82194 | DPU_G0061832 | *gxcEE* | DDB_G0281047 | *gxcEE* |  |
| 152605 | DPU_G0053642 | *gxcFF* | DDB_G0284739 | *gxcFF* |  |
| 92938 | DPU_G0065484 | *gxcGG* | DDB_G0268354 | *gxcGG* |  |
| 148817 | DPU_G0064668 | *gxcHH* | DDB_G0290493 | *gxcHH* |  |
| 99010 | DPU_G0061516 | *gxcII* | DDB_G0278703 | *gxcII* |  |
| 99199 | DPU_G0063228 | *gxcJJ* | DDB_G0275679 | *gxcJJ* |  |
| 26788 | DPU_G0055312 | *gxcKK* | DDB_G0293340 | *gxcKK* |  |
| 157827 | DPU_G0065986 | *kxcA* | DDB_G0289859 | *kxcA* |  |
| 52129 | DPU_G0069236 | *kxcB* | DDB_G0293124 | *kxcB* |  |
| 148631 | DPU_G0063182 | *roco5* | DDB_G0294533 | *roco5* |  |
| 157834 | DPU_G0066114 | *gefC* | DDB_G0282381 | *gefC* |  |
| 159515 | DPU_G0070836 | *myoM* | DDB_G0292262 | *myoM* |  |
| 79261 | DPU_G0053802 |  | DDB_G0269934 | *DDB_G0269934* |  |
| RhoGEF (Dock180 and ELMO) | | | | |  |
| 47503 | DPU_G0051892 | *docA* | DDB_G0291974 | *docA* |  |
| 34798 | DPU_G0054908 | *docB* | DDB_G0270404 | *docB* |  |
| 81789 | DPU_G0060636 | *docC* | DDB_G0292004 | *docC* |  |
| 27977 | DPU_G0061656 | *docD* | DDB_G0284725 | *docD* |  |
| 151994 | DPU_G0052276 | *zizA* | DDB_G0275035 | *zizA* |  |
| 153700 | DPU_G0056004 | *zizB* | DDB_G0293084 | *zizB* |  |
| 152501 | DPU_G0053332 | *zizC* | DDB_G0275239 | *zizC* |  |
| 149722 | DPU_G0070224 | *zizD* | DDB_G0268888 | *zizD* |  |
| 96234 | DPU_G0057496 | *elmoA* | DDB_G0278051 | *elmoA* |  |
| 86597 | DPU_G0066098 | *elmoB* | DDB_G0280179 | *elmoB* |  |
| 50960 | DPU_G0067304 | *elmoC* | DDB_G0282949 | *elmoC* |  |
| 99508 | DPU_G0065782 | *elmoD* | DDB_G0280943 | *elmoD* |  |
| 94211 | DPU_G0073680 | *elmoE* | DDB_G0279657 | *elmoE* |  |
| 76775 | DPU_G0070396 | *elmoF* | DDB_G0267548 | *elmoF* |  |
| Darlin | | | | |  |
| 40381 | DPU_G0064106 | *darA* | DDB_G0288771 | *darA* |  |
| RhoGAP | | | | |  |
| 29142 | DPU_G0067174 | *gacA* | DDB_G0278381 | *gacA* |  |
| 96526 | DPU_G0054746 | *gacB* | DDB_G0278549 | *gacB* |  |
| (no model) |  | *gacC* | DDB_G0284571 | *gacC* |  |
| 82068 | DPU_G0061452 | *gacD* | DDB_G0291021 | *gacD* |  |
| 153891 | DPU_G0056322 | *gacE* | DDB_G0293654 | *gacE* |  |
| 146520 | DPU_G0051180 | *gacF* | DDB_G0274243 | *gacF* |  |
| (no model) |  | *gacG* | DDB_G0283955 | *gacG* |  |
| 147510 | DPU_G0053436 | *gacH* | DDB_G0272694 | *gacH* |  |
| 50285 | DPU_G0063832 | *gacI* | DDB_G0275185 | *gacI* |  |
| 89900 | DPU_G0064892 | *gacJ* | DDB_G0285641 | *gacJ* |  |
| 93648 | DPU_G0058818 | *gacK* | DDB_G0285915 | *gacK* |  |
| 95356 | DPU_G0061454 | *gacL* | DDB_G0290891 | *gacL* |  |
| 88032 | DPU_G0053702 | *gacM* | DDB_G0287895 | *gacM* |  |
| 158397 | DPU_G0067616 | *gacN* | DDB_G0279315 | *gacN* |  |
| 10267 | DPU_G0053570 | *gacO* | DDB_G0267568 | *gacO* |  |
| 155202 | DPU_G0059558 | *gacP* | DDB_G0279009 | *gacP* |  |
| 158606 | DPU_G0068154 | *gacQ* | DDB_G0282395 | *gacQ* |  |
| 85916 | DPU_G0051352 | *gacR* | DDB_G0278755 | *gacR* |  |
| 158993 | DPU_G0069188 | *gacS* | DDB_G0276833 | *gacS* |  |
| 97430 | DPU_G0073602 | *gacT* | DDB_G0285163 | *gacT* |  |
| 98229 | DPU_G0055212 | *gacU* | DDB_G0272925 | *gacU* |  |
| 98974 | DPU_G0061350 | *gacV* | DDB_G0293510 | *gacV* |  |
| 15574 | DPU_G0055276 | *gacW* | DDB_G0290439 | *gacW* |  |
| 150164 | DPU_G0072142 | *gacX* | DDB_G0288205 | *gacX* |  |
| 159700 | DPU_G0071574 | *gacY* | DDB_G0281739 | *gacY* |  |
| 97170 | DPU_G0070034 | *gacZ* | DDB_G0269496 | *gacZ* |  |
| 56354 | DPU_G0057264 | *mgp3/mpgAA* | DDB_G0269624 | *gacAA* |  |
| 155654 | DPU_G0060628 | *mgp1/mpgBB* | DDB_G0290233 | *gacBB* |  |
| 15662 | DPU_G0066588 | *mgp2/gacCC* | DDB_G0270024 | *mgp2/gacCC* |  |
| 42386 | DPU_G0068110 | *mgp4/gacDD* | DDB_G0270024 | *mgp4/gacDD* |  |
| 149424 | DPU_G0068558 | *gacEE* | DDB_G0291840 | *gacEE* |  |
| 149898 | DPU_G0070966 | *gacFF* | DDB_G0275085 | *gacFF* |  |
| 98143 | DPU_G0054624 | *gacGG* | DDB_G0280093 | *gacGG* |  |
| 77981 | DPU_G0075120 | *gacHH* | DDB_G0272080 | *gacHH* |  |
| 153400 | DPU_G0055260 | *gacJJ* | DDB_G0290873 | *gacJJ* |  |
| 98122 | DPU_G0054482 | *Dp5p4* | DDB_G0267462 | *Dd5p4* |  |
| 96792 | DPU_G0062136 |  | DDB_G0293184 | *DDB_G0293184* |  |
| 150174 | DPU_G0072160 | *roco9* | DDB_G0288183 | *roco9* |  |
| 148693 | DPU_G0063892 | *roco10* | DDB_G0291710 | *roco10* |  |
| 40694 | DPU_G0064598 | *gefD* | DDB_G0289667 | *gefD* |  |
| 4417 | DPU_G0061560 | *gflB* | DDB_G0286773 | *gflB* |  |
| 77513 | DPU_G0073816 | *gflD* | DDB_G0279569 | *gflD* |  |
| RhoGEF + RhoGAP | | | | |  |
| 96450 | DPU_G0052100 | *xacA* | DDB_G0291978 | *xacA* |  |
| 154253 | DPU_G0057146 | *xacB* | DDB_G0278417 | *xacB* |  |
| 86690 | DPU_G0067748 | *xacC* | DDB_G0285391 | *xacC* |  |
| *Effectors with CRIB/PBD* | | | | | |
| 87126 | DPU_G0072896 | *wasA* | DDB_G0293834 | *wasA* |  |
| 81492 | DPU_G0059876 |  | DDB_G0272811 | *DDB_G0272811* |  |
| (no model) |  |  | DDB_G0283827 | *DDB_G0283827* |  |
| 90830 | DPU_G0071952 | *gnrC* | DDB_G0276571 | *gnrC* |  |
| 96263 | DPU_G0062510 | *pakA* | DDB_G0269166 | *pakA* |  |
| 31335 | DPU_G0074072 | *pakB* | DDB_G0276459 | *pakB* |  |
| 91113 | DPU_G0074390 | *pakC* | DDB_G0267450 | *pakC* |  |
| 155336 | DPU_G0059930 | *pakD* | DDB_G0269696 | *pakD* |  |
| 44150 | DPU_G0072662 | *pakE* | DDB_G0293932 | *pakE* |  |
| 150846 | DPU_G0074510 | *pakF* | DDB_G0274409 | *pakF* |  |
| 147802 | DPU_G0056426 | *pakG* | DDB_G0282891 | *pakG* |  |
| 90051 | DPU_G0065958 | *pakH* | DDB_G0269166 | *pakH* |  |
| Scar complex | | | | |  |
| 40920 | DPU_G0065084 | *scrA* | DDB_G0285253 | *scrA* |  |
| 87985 | DPU_G0053536 | *abiA* | DDB_G0267956 | *abiA* |  |
| 46589 | DPU_G0071848 | *pirA* | DDB_G0287855 | *pirA* |  |
| 153775 | DPU_G0056120 | *napA* | DDB_G0274519 | *napA* |  |
| 88267 | DPU_G0054936 | *hspc300* | DDB_G0279175 | *hspc300* |  |
| Formins | | | | |  |
| 150581 | DPU_G0073676 | *forA* | DDB_G0279607 | *forA* |  |
| 33735 | DPU_G0053232 | *forB* | DDB_G0282297 | *forB* |  |
| 50865 | DPU_G0066776 | *forC* | DDB_G0287295 | *forC* |  |
| 46912 | DPU_G0073848 | *forD* | DDB_G0282245 | *forD* |  |
| 154316 | DPU_G0057262 | *forE* | DDB_G0269626 | *forE* |  |
| 148204 | DPU_G0059834 | *forF* | DDB_G0289763 | *forF* |  |
| 153797 | DPU_G0056158 | *forG* | DDB_G0277175 | *forG* |  |
| 156963 | DPU_G0063964 | *forH* | DDB_G0285589 | *forH* |  |
| 36102 | DPU_G0056740 | *forI* | DDB_G0284519 | *forI* |  |
| 97202 | DPU_G0070450 | *forJ* | DDB_G0291378 | *forJ* |  |
| 99159 | DPU_G0062882 | *forK* |  | *none* |  |
| IQGAP | | | | |  |
| 42926 | DPU_G0069466 | *gapA* | DDB_G0269140 | *gapA* |  |
| 49582 | DPU_G0060430 | *rgaA* | DDB_G0287585 | *rgaA* |  |
| 158441 | DPU_G0067794 |  | DDB_G0275731 | *DDB_G0275731* |  |
| 31268 | DPU_G0073888 |  | DDB_G0288977 | *DDB_G0288977* |  |
| *Effectors with HR1 domain* | | | | |  |
| 152774 | DPU_G0053940 |  | DDB_G0271812 | *DDB_G0271812* |  |
| 92017 | DPU_G0052560 |  | DDB_G0271676 | *DDB_G0271676* |  |
| 48387 | DPU_G0055482 |  | DDB_G0274695 | *DDB_G0274695* |  |
| PI3 kinases | | | | |  |
| 58194 | DPU_G0065578 | *pikA* | DDB_G0278727 | *pikA* |  |
| 52203 | DPU_G0071394 | *pikB* | DDB_G0283081 | *pikB* |  |
| 149892 | DPU_G0070956 | *pikC* | DDB_G0275011 | *pikC* |  |
| 155819 | DPU_G0061026 | *pikE* | DDB_G0289601 | *pikE* |  |
| 35373 | DPU_G0055696 | *pikG* | DDB_G0282625 | *pikG* |  |
| 96754 | DPU_G0061222 | *pikH* | DDB_G0291093 | *pikH* |  |
| PI4P 5 kinases | | | | |  |
| 52460 | DPU_G0052116 |  | DDB_G0280377 | *DDB_G0280377* |  |
| 148173 | DPU_G0059774 |  | DDB_G0286115 | *DDB_G0286115* |  |
| 148780 | DPU_G0064312 |  | DDB_G0286115 | *DDB_G0286115* |  |
| 97761 | DPU_G0051730 |  | DDB_G0286115 | *DDB_G0286115* |  |
| 82538 | DPU_G0062938 |  | DDB_G0286115 | *DDB_G0286115* |  |
| 157566 | DPU_G0065446 |  | DDB_G0286115 | *DDB_G0286115* |  |
| 41242 | DPU_G0065592 |  | DDB_G0286115 | *DDB_G0286115* |  |
| 84210 | DPU_G0068074 |  | DDB_G0286115 | *DDB_G0286115* |  |
| 159799 | DPU_G0071882 |  | DDB_G0286115 | *DDB_G0286115* |  |
| 160088 | DPU_G0072838 |  | DDB_G0286115 | *DDB_G0286115* |  |
| 55653 | DPU_G0054650 |  | DDB_G0267588 | *DDB_G0267588* |  |
| 82999 | DPU_G0064208 |  | DDB_G0267588 | *DDB_G0267588* |  |
| 154611 | DPU_G0058136 |  | DDB_G0292190 | *DDB_G0292190* |  |
| 37984 | DPU_G0059910 | *PIPkinA* | DDB_G0275635 | *PIPkinA* |  |
| 57810 | DPU_G0063860 | *pip5k3* | DDB_G0279149 | *pip5k3* |  |
| 99415 | DPU_G0064944 | *rpkA* | DDB_G0283615 | *rpkA* |  |
| 159970 | DPU_G0072414 |  | DDB_G0274067,  DDB_G0272638 | *DDB_G0274067,*  *DDB_G0272638* |  |
| 85137 | DPU_G0071608 |  |  | *none* |  |
| DAG kinases | | | | |  |
| 147968 | DPU_G0057790 | *dgkA* | DDB_G0277223 | *dgkA* |  |
| Phospholipase D | | | | |  |
| 95839 | DPU_G0067580 | *pldA* | DDB_G0281031 | *pldA* |  |
| 149141 | DPU_G0067132 | *pldB* | DDB_G0279483 | *pldB* |  |
| 55934 | DPU_G0055620 | *pldC* | DDB_G0277949 | *pldC* |  |
| 218575# | DPU_G0059446 |  | DDB_G0284155 | *DDB_G0284155* |  |
| Phospholipase C | | | | |  |
| 96532 | DPU_G0055284 | *pipA* | DDB_G0292736 | *plc/pipA* |  |
| *NADPH oxidase complex* | | | | |  |
| 99304 | DPU_G0064096 | *ncfA* | DDB_G0288773 | *ncfA* |  |
| 32111 | DPU_G0075352 | *noxA* | DDB_G0289653 | *noxA* |  |
| 41266 | DPU_G0065620 | *noxB* | DDB_G0287101 | *noxB* |  |
| 51890 | DPU_G0073618 | *noxC* | DDB_G0291117 | *noxC* |  |
| 30041 | DPU_G0070392 | *cybA* | DDB_G0267460 | *cybA* |  |
| Exocyst complex | | | | |  |
| 89267 | DPU_G0060854 | *sec3/exoc1* | DDB_G0285067 | *sec3/exoc1* |  |
| 50260 | DPU_G0063716 | *sec5/exoc2* | DDB_G0280081 | *sec5/exoc2* |  |
| 155933 | DPU_G0061340 | *sec6/exoc3* | DDB_G0293520 | *sec6/exoc3* |  |
| 36520 | DPU_G0057344 | *sec8/exoc4* | DDB_G0284833 | *sec8/exoc4* |  |
| 28786 | DPU_G0065644 | *sec10/exoc5* | DDB_G0287881 | *sec10/exoc5* |  |
| 89602 | DPU_G0062970 | *sec15/exoc6* | DDB_G0293936 | *sec15/exoc6* |  |
| 88329 | DPU_G0055210 | *exo70/exoc7* | DDB_G0272991 | *exo70/exoc7* |  |
| 37625 | DPU_G0059426 | *exo84/exoc8* | DDB_G0279991 | *exo84/exoc8* |  |
| Others | | | | |  |
| 94684 | DPU_G0054034 | *limE* | DDB_G0279415 | *limE* |  |
| 46585 | DPU_G0071820 | *lis1* | DDB_G0288375 | *lis1* |  |

#alternative JGI models replacing models originally selected for the Gene Catalog.


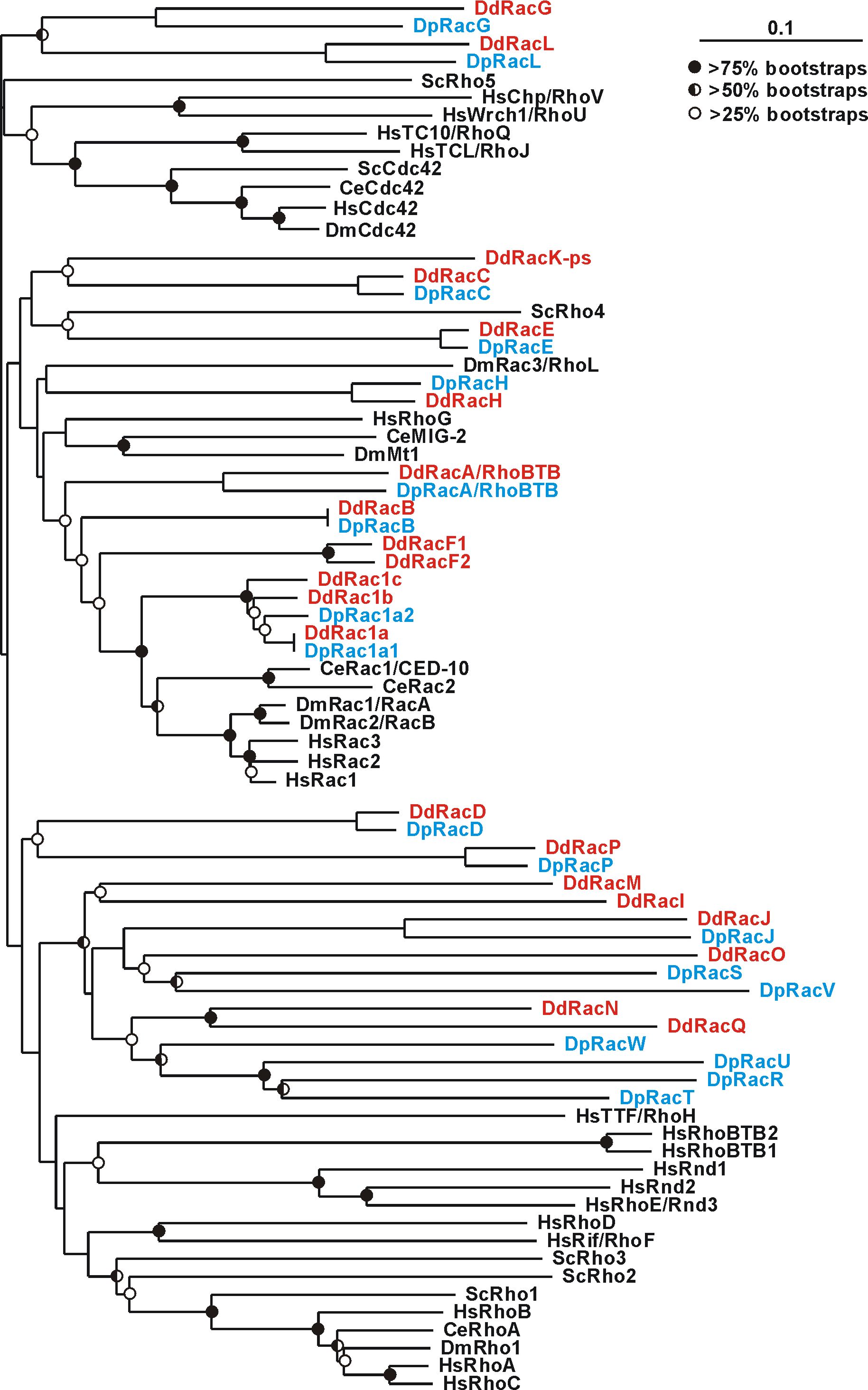


**Figure S14. Phylogenetic tree of the Rho family in *D. purpureum* and *D. discoideum*.** Complete sets of Rho proteins from several species were used. This comprises the 18 D. purpureum Rho proteins(in blue), the 21 *D.* *discoideum* Rho proteins (including a reconstructed sequence of RacK) (red), 6 from *Saccharomyces cerevisiae* (Sc), 5 from *Caenorhabditis elegans* (Ce), 7 from *Drosophila melanogaster* (Dm) and 20 from *Homo sapiens* (Hs). Human RhoBTB3 was not included in the analysis, as its GTPase domain is strongly erased. The phylogenetic tree was constructed using the neighbor joining method with the core GTPase domain devoid of N- and C-terminal repetitive sequences. Bootstraps between homonymous proteins of *D. purpureum* and *D. discoideum* are not indicated; they were 100% in all cases. *Dictyostelium* has representatives of the Rac subfamily, but lacks Rho and Cdc42 proteins. Note that the RacA GTPase domain is more closely related to Rac proteins than to RhoBTB proteins, although RacA has the domain architecture of a RhoBTB protein. The scale bar indicates amino acid substitutions per site.

# The Ras superfamily of GTPases

***The Ras superfamily of GTPases.*** This group comprises classical “small GTPases” and their relatives (the GTPase component of heterotrimeric G-proteins, multidomain ROCO proteins etc.). These proteins generally function as critical regulators of a diverse array of cellular processes (signaling cascades, membrane trafficking, cytoskeleton dynamics etc.), hence the elaboration of the Ras superfamily correlate with the organismal and cell complexity. Comparison of the Ras superfamily complement in *D. purpureum* and *D. discoideum* can be found in Table S15 and Figures S15-S17*.*  The *D. purpureum* genome encodes fewer superfamily members (150, versus 175 in *D. discoideum*). This difference appears to be due to less extensive gene duplication and more extensive gene loss in the *D. purpureum* lineage. Nevertheless, there is a one-to-one orthologous correspondence between most Ras superfamily GTPases from *D. purpureum* and *D. discoideum*. Notable exceptions in the form of independent family expansions include, in addition to those described above for the Rho family, six Ras family (*rasU* to *rasZ*) corresponding to just one gene in *D. purpureum* (DPU_G0070892; Figure S15). A lot of independent evolution is particularly seen in the Rab family of vesicle trafficking regulators (Figure S16), suggesting that there might be substantial differences between the two species in the organization and function of the endomembrane system.

**Table S15. The Ras superfamily of GTPases in *D. purpureum* and *D. discoideum*.**

| *D. purpureum* | | | *D. discoideum* | | notes |
| --- | --- | --- | --- | --- | --- |
| Proposed gene name^a^ | Protein model ID^b^ | dictyBase Gene ID^c^ | Gene name^c^ | dictyBase Gene ID^c^ |  |
|  | | | | | |
| RAS family | | | | | |
| rheb | 147775 | DPU_G0055948 | rheb | DDB_G0277041 |  |
| rapA | 59134 | DPU_G0071072 | rapA | DDB_G0291237 |  |
| rapB | 26434 | DPU_G0053506 | rapB | DDB_G0272857 |  |
| rapC | 74092# | DPU_G0062576 | rapC | DDB_G0270340 |  |
| rasB | 47179 | DPU_G0075064 | rasB | DDB_G0292998 |  |
| rasC | 51488 | DPU_G0070498 | rasC | DDB_G0281385 |  |
| rasD | 91367 | DPU_G0062120 | rasD | DDB_G0292996 |  |
| rasG | 92731 | DPU_G0062342 | rasG | DDB_G0293434 |  |
| rasS | 57324 | DPU_G0061508 | rasS | DDB_G0283537 |  |
| NA | 93239 | DPU_G0070892 | rasU | DDB_G0270138 | Independent family expansion in *D. discoideum* (six paralogs) |
|  |  |  | rasV | DDB_G0270736 |  |
|  |  |  | rasW | DDB_G0270122 |  |
|  |  |  | rasX | DDB_G0270124 |  |
|  |  |  | rasY | DDB_G0270126 |  |
|  |  |  | rasZ | DDB_G0270140 |  |
| rsmA | 90155# | DPU_G0066746 | rsmA | DDB_G0283547 |  |
| rsmB | 79073# | DPU_G0053296 | rsmB | DDB_G0281253 |  |
| - | - | - | rsmC | DDB_G0278449 | Very divergent |
| rsmD | 84962# | DPU_G0070728 | rsmD | DDB_G0292318 |  |
| NA | 55232 | DPU_G0052952 | rsmE-1 | DDB_G0273099 | Possibly independent family expansions in *D. purpureum* (two paralogs) and *D. discoideum* (seven paralogs) |
| NA | CBTG1621.b1 (EST sequence; absent from the genome assembly but recorded by several unassembled reads) |  | rsmE-2 | DDB_G0273825 |  |
|  |  |  | rsmF | DDB_G0278389 |  |
|  |  |  | rsmG | DDB_G0278717 |  |
|  |  |  | rsmH | DDB_G0278403 |  |
|  |  |  | rsmJ | DDB_G0279305 |  |
|  |  |  | rsmK | DDB_G0278217 |  |
| NA | 168758$ | DPU_G0052626 | DDB_G0280437 | DDB_G0280437 | Uncharacterised RAS-like GTPase |
| - | - | - | DDB_G0293786 | DDB_G0293786 | Uncharacterised *D. discoideum*-specific RAS-like GTPase |
| NA | 96273 | DPU_G0062638 | DDB_G0270932 | DDB_G0270932 | Two tandemly arranged divergent RAS-like domains |
| cpras1 | 168760$ | DPU_G0073460 | cpras1 | DDB_G0277381 | Circularly-permuted RAS-like domain (cpRAS) protein |
| cpras2 | 80287# | DPU_G0056356 | cpras2 | DDB_G0293376 | Circularly-permuted RAS-like domain (cpRAS) protein |
|  | | | | | |
| RAB family | | | | | |
| rab1A | 159642 | DPU_G0071228 | rab1A | DDB_G0283757 |  |
| rab1B | 50297 | DPU_G0063876 | rab1B | DDB_G0277867 |  |
| rab1C | 97759 | DPU_G0051726 | rab1C | DDB_G0269174 |  |
| rab1D | 54449 | DPU_G0074050 | rab1D | DDB_G0284985 |  |
| - | - | - | rab1E | DDB_G0275969 |  |
|  |  |  | rabY | DDB_G0282203 |  |
| rab2A | 92882 | DPU_G0064540 | rab2A | DDB_G0292268 |  |
| rab2B | 96118* | DPU_G0071958 | rab2B | DDB_G0272138 |  |
| rab4A | 41201 | DPU_G0065550 | rab4 | DDB_G0292406 |  |
| rab4B | 26958 | DPU_G0056408 |  |  |  |
| rab4C | 39631 | DPU_G0062856 |  |  |  |
| rab5A | 93331 | DPU_G0073260 | rab5A | DDB_G0271984 |  |
| rab5B | 86102 | DPU_G0056432 | rab5B | DDB_G0282847 |  |
| rab6 | 52877 | DPU_G0059370 | rab6 | DDB_G0268068 |  |
| rab7A | 92126 | DPU_G0054224 | rab7A | DDB_G0269236 |  |
| rab7B | 78083 | DPU_G0075452 | rab7B | DDB_G0287553 |  |
| rab8 | 91938 | DPU_G0051692 | rab8A | DDB_G0280043 |  |
|  |  |  | rab8B | DDB_G0276399 |  |
| rab11A | 91321# | DPU_G0060304 | rab11A | DDB_G0269238 |  |
| - | - | - | rab11B | DDB_G0287211 |  |
| rab11C | 96604 | DPU_G0057776 | rab11C | DDB_G0277101 |  |
| rab14 | 92215 | DPU_G0055130 | rab14 | DDB_G0281337 |  |
| rab18 | 57208 | DPU_G0060976 | rab18 | DDB_G0289827 |  |
| rab21 | 54865 | DPU_G0051534 | rab21 | DDB_G0286553 |  |
| rab24 | 78440 | DPU_G0051710 | rab24 | DDB_G0268402 |  |
| rab32A | 74712 | DPU_G0052764 | rab32A | DDB_G0283603 | Local gene duplication in *D. purpureum* comprising an intact gene and an adjacent apparent pseudogene |
| rab32A2_ps | 147455 | DPU_G0052760 |  |  |  |
| rab32B | 148238 | DPU_G0060276 | rab32B | DDB_G0269416 |  |
| rab32C | 91127 | DPU_G0051274 | rab32C | DDB_G0275675 |  |
| rab32D | 46949* | DPU_G0074000 | rab32D | DDB_G0285051 |  |
| rabC | 47972 | DPU_G0053946 | rabC | DDB_G0271736 |  |
| rabG | 96892 | DPU_G0064676 | rabG1 | DDB_G0291738 |  |
|  |  |  | rabG2 | DDB_G0290783 |  |
| - | - | - | rabH | DDB_G0275955 |  |
| rabJ | 156394 | DPU_G0062380 | rabJ | DDB_G0277441 |  |
| rabQ | 82365 | DPU_G0062272 | rabQ | DDB_G0268760 |  |
| - | - |  | rabA | DDB_G0291233 |  |
|  |  |  | rabF1-1 | DDB_G0272905 |  |
|  |  |  | rabF1-2 | DDB_G0273935 |  |
|  |  |  | rabF2_ps | DDB_G0276053 | pseudogene |
| - | - |  | rabK1 | DDB_G0290833 | Large family expansion of divergent Rab7-related paralogs in *D. discoideum* (11 loci, some may be pseudogenes), without orthologs in *D. purpureum* |
|  |  |  | rabK2 | DDB_G0290791 |  |
|  |  |  | rabK3 | DDB_G0290831 |  |
|  |  |  | rabL | DDB_G0290779 |  |
|  |  |  | rabM | DDB_G0290829 |  |
|  |  |  | rabN1 | DDB_G0290789 |  |
|  |  |  | rabN2 | DDB_G0290793 |  |
|  |  |  | DDB_G0294571 | DDB_G0294571 |  |
|  |  |  | DDB_G0290827 | DDB_G0290827 |  |
|  |  |  | rabO | DDB_G0290407 |  |
|  |  |  | rabP | DDB_G0290875 |  |
| rabS | 160488* | DPU_G0074528 | rabS | DDB_G0282537 |  |
| - | - | - | rabR | DDB_G0271980 |  |
|  |  |  | rabT1 | DDB_G0268910 |  |
|  |  |  | rabT2 | DDB_G0269262 |  |
|  |  |  | DDB_G0294583 | DDB_G0294583 |  |
|  |  |  | DDB_G0294569 | DDB_G0294569 |  |
| - | - | - | rabU | DDB_G0291293 |  |
| NA | 77270# | DPU_G0072786 | rabV | DDB_G0282899 | Orthology between *D. purpureum* and *D. discoideum* unsure |
| NA | 82173 | DPU_G0061790 | rabW | DDB_G0276295 | Orthology between *D. purpureum* and *D. discoideum* unsure |
| - | - | - | rabX | DDB_G0275327 | Extremely divergent |
| - | - | - | rabZ | DDB_G0286169 |  |
| - | - | - | DDB_G0276007 | DDB_G0276007 |  |
| NA | 152785 | DPU_G0053958 | - | - |  |
| NA | 78832# | DPU_G0052616 | - | - | Not completely sure that all these paralogs have a common origin (rather divergent sequences, some may be pseudogenes) |
| NA | 83134# | DPU_G0064588 |  |  |  |
| NA | 74151# | DPU_G0066374 |  |  |  |
| NA | 78917 | DPU_G0052950 |  |  |  |
| NA | 84758# | DPU_G0070090 |  |  |  |
| NA | 75757 | DPU_G0063172 |  |  |  |
| NA | 73840 | DPU_G0051070 |  |  |  |
| NA | 75864 | DPU_G0064250 |  |  |  |
| NA | 148542 | DPU_G0062744 |  |  |  |
| NA | 36395 | DPU_G0057254 |  |  |  |
| NA | 80610# | DPU_G0057246 |  |  |  |
| NA | 168762$ | DPU_G0057252 |  |  |  |
| NA | 37398 | DPU_G0059062 |  |  |  |
| NA | 79526# | DPU_G0054478 |  |  |  |
| NA | 38803 | DPU_G0061312 | - | - | Not completely sure that all these paralogs have a common origin (rather divergent sequences) |
| NA | 38788 | DPU_G0061314 |  |  |  |
| NA | 39890 | DPU_G0063268 |  |  |  |
| NA | 25125 | DPU_G0057494 |  |  |  |
|  | | | | | |
| RHO family | | | | | |
| rac1A1 | 92985 | DPU_G0066198 | rac1A | DDB_G0277869 |  |
| rac1A2 | 168754$ | DPU_G0057068 |  |  |  |
| - | - | - | rac1B | DDB_G0268622 |  |
| - | - | - | rac1C | DDB_G0282365 |  |
| racA | 50894 | DPU_G0066986 | racA | DDB_G0286555 |  |
| racB | 168757*$ | DPU_G0061650 | racB | DDB_G0279605 |  |
| racC | 55703 | DPU_G0054880 | racC | DDB_G0293526 |  |
| racD | 93635# | DPU_G0058262 | racD | DDB_G0291976 |  |
| racE | 98135 | DPU_G0054576 | racE | DDB_G0280975 |  |
| - | - | - | racF1 | DDB_G0269176 |  |
|  |  |  | racF2 | DDB_G0276967 |  |
| racG | 75463 | DPU_G0060756 | racG | DDB_G0269178 |  |
| racH | 41217 | DPU_G0065542 | racH | DDB_G0269240 |  |
| - | - | - | racI | DDB_G0277897 |  |
| racJ | 147341 | DPU_G0051392 | racJ | DDB_G0292560 |  |
| - | - | - | racK_ps | DDB_G0268908 | pseudogene |
| racL | 52376 | DPU_G0075220 | racL | DDB_G0292816 |  |
| racP | 89172 | DPU_G0060224 | racP | DDB_G0285453 |  |
| racR | 80321# | DPU_G0056546 | racM | DDB_G0289103 | Independent family expansions in *D. purpureum* (six paralogs) and *D. discoideum* (four paralogs) |
| racS | 81024# | DPU_G0058588 | racN | DDB_G0278009 |  |
| racT | 149292 | DPU_G0067766 | racO | DDB_G0277791 |  |
| racU | 81474 | DPU_G0059740 | racQ | DDB_G0278011 |  |
| racV | 81475 | DPU_G0059742 |  |  |  |
| racW | 158834 | DPU_G0068822 |  |  |  |
| NA | 49642 | DPU_G0060690 | DDB_G0282605 | DDB_G0282605 | Divergent RHO-like domain fused to a LRR domain |
|  | | | | | |
| ARF/Sar1 family |  |  |  |  |  |
| arfA | 93077 | DPU_G0067816 | arfA | DDB_G0289173 |  |
| NA | 46140 | DPU_G0068504 | - | - | Seems to be *D. purpureum*-specific divergent paralogs of arfA (lacking the N-terminal myristoylation motif) |
| NA | 75970 | DPU_G0065016 |  |  |  |
| arl1 | 81368 | DPU_G0059512 | arl1 | DDB_G0288163 |  |
| arl2 | 90855* | DPU_G0072228 | arl2 | DDB_G0281307 |  |
| arl5 | 93016 | DPU_G0066824 | arl5 | DDB_G0271942 |  |
| arl8 | 93516# | DPU_G0052688 | arl8 | DDB_G0283525 |  |
| arfrp1 | 86826# | DPU_G0069592 | arfrp1 | DDB_G0283631 |  |
| - | - | - | arrA | DDB_G0282457 |  |
| arrB | 77595 | DPU_G0074070 | arrB | DDB_G0274445 |  |
| arrC | 41641 | DPU_G0066606 | arrC | DDB_G0271792 |  |
| arrD | 35934 | DPU_G0056396 | arrD | DDB_G0289069 |  |
| NA | 73266 | DPU_G0063870 | arrE | DDB_G0280479 | Possibly independent family expansions in *D. purpureum* (five paralogs) and *D. discoideum* (five paralogs and a pseudogene) |
| NA | 73423 | DPU_G0066132 | arrF | DDB_G0289435 |  |
| NA | 87458 | DPU_G0075268 | arrG_ps | DDB_G0280697 |  |
| NA | 38752 | DPU_G0061148 | arrH | DDB_G0288015 |  |
| NA | 79687 | DPU_G0054922 | arrJ | DDB_G0280621 |  |
|  |  |  | arrK | DDB_G0280633 |  |
| NA | 92653 | DPU_G0061354 | - | - | Uncharacterised *D. purpureum*-specific ARF-like GTPase |
| arrL | 150230 | DPU_G0072342 | arrL | DDB_G0278057 |  |
| - | - | - | DDB_G0292332 | DDB_G0292332 | Uncharacterised *D. discoideum*-specific ARF-like GTPase |
| NA | 147576 | DPU_G0054126 | DDB_G0278443 | DDB_G0278443 | Uncharacterised ARF-like GTPase |
| sarA | 46167 | DPU_G0068760 | sarA | DDB_G0272296 |  |
| sarB | 168733$ | DPU_G0068040 | sarB | DDB_G0278477 |  |
|  | | | | | |
| G-alpha family (a subunit of heterotrimeric G-proteins) | | | | | |
| gpaA | 53968 | DPU_G0071128 | gpaA | DDB_G0283349 |  |
| gpaB | 91607 | DPU_G0070624 | gpaB | DDB_G0276267 |  |
| gpaC | 159561 | DPU_G0071010 | gpaC | DDB_G0287031 |  |
| gpaD | 93271 | DPU_G0071740 | gpaD | DDB_G0285425 |  |
| gpaE | 149474*# | DPU_G0068812 | gpaE | DDB_G0286185 |  |
| gpaF | 93749# | DPU_G0061752 | gpaF | DDB_G0283151 |  |
| gpaG | 92301 | DPU_G0056224 | gpaG | DDB_G0276455 |  |
| gpaH | 91506 | DPU_G0068236 | gpaH | DDB_G0284469 |  |
| gpaI | 36694 | DPU_G0057962 | gpaI | DDB_G0283419 |  |
| - | - | - | gpaJ | DDB_G0268802 |  |
| gpaK | 92510 | DPU_G0059260 | gpaK | DDB_G0276343 |  |
| gpaL | 150924 | DPU_G0074682 | gpaL | DDB_G0280351 |  |
| spnA | 160336 | DPU_G0073856 | spnA | DDB_G0276155 |  |
| gbqA | 153084 | DPU_G0054594 | gbqA | DDB_G0280965 |  |
|  | | | | | |
| Other Ras superfamily GTPases | | | | | |
| ranA | 150415# | DPU_G0073176 | ranA | DDB_G0291235 |  |
| ranB | 29152# | DPU_G0067138 | ranB | DDB_G0274943 | *D. discoideum* protein has two RAN-like domains, while *D. purpureum* protein has only one (corresponding to the C-terminal domain of the former) |
| spg1 | 94854# | DPU_G0055742 | spg1 | DDB_G0291269 |  |
| gemA | 168763*$ | DPU_G0071886 | gemA | DDB_G0267830 |  |
| srpRB | 147655 | DPU_G0054742 | srpRB | DDB_G0278543 |  |
| NA | 150131# | DPU_G0072010 | DDB_G0286513 | DDB_G0286513 | Ortholog of *Arabidopsis* LIP1 and human RABL3 |
| ragA | 35824 | DPU_G0056242 | ragA | DDB_G0288701 |  |
| ragC | 80421* | DPU_G0056746 | ragC | DDB_G0284495 |  |
| NA | 159388# | DPU_G0070482 | DDB_G0270744 | DDB_G0270744 | Ortholog of human RBEL1 |
| dync1li1 | 34712 | DPU_G0054794 | dync1li1 | DDB_G0292904 |  |
| comB | 146844* | DPU_G0066452 | comB | DDB_G0281825 |  |
| NA | CCAB688.g1 (EST sequence, absent from the genome assembly) |  | DDB_G0282647 | DDB_G0282647 | Uncharacterised GTPase, may be divergent member of the RAB family (but lacks a C-terminal prenylation motif), related to DDB_G0294577 |
| NA | 83633# | DPU_G0066126 | DDB_G0294577 | DDB_G0294577 | Uncharacterised GTPase, may be divergent member of the RAB family (but lacks a C-terminal prenylation motif), related to DDB_G0282647 |
|  |  |  | DDB_G0282649_ps | DDB_G0282649 |  |
| NA | 84055 | DPU_G0067564 | DDB_G0276227 | DDB_G0276227 | Divergent RAB-like GTPase domain fused with a LRR domain |
| rsmM | 89854 | DPU_G0064544 | rsmM | DDB_G0292300 | Perhaps should be classified as a divergent member of the RAB family |
| - | - | - | rsmN | DDB_G0282651 | These two genes seems to form a *D. discoideum*-specific family of divergent GTPases |
|  |  |  | DDB_G0282653 | DDB_G0282653 |  |
| NA | 97732 | DPU_G0051548 | DDB_G0287061 | DDB_G0287061 | Large uncharacterised protein with a divergent C-terminal GTPase domain; related to DDB_G0287207 |
| - | - |  | DDB_G0287207 | DDB_G0287207 | Large uncharacterised protein with a divergent C-terminal GTPase domain; related to DDB_G0287061 |
| NA | Absent from the genome assembly but recorded by several unassembled reads (FFAS169538.g1, FFAS99606.b1…) |  | DDB_G0274301 | DDB_G0274301 | Divergent RAB-like GTPase domain protein |
|  | | | | | |
| ROCO family (containing Roc GTPase domain) | | | | | |
| pats1 | 159478* | DPU_G0070698 | pats1 | DDB_G0269250 |  |
| - | - | - | qkgA-1 | DDB_G0273259 |  |
|  |  |  | qkgA-2 | DDB_G0273635 |  |
| gbpC | 168746$ | DPU_G0059624 | gbpC | DDB_G0291079 |  |
| roco4 | 49056 | DPU_G0058498 | roco4 | DDB_G0288251 |  |
| roco5 | 148631 | DPU_G0063182 | roco5 | DDB_G0294533 |  |
| roco6 | 99455# | DPU_G0065240 | roco6 | DDB_G0279417 |  |
| roco7 | 81310 | DPU_G0059300 | roco7 | DDB_G0267472 |  |
| roco8 | 56686# | DPU_G0058976 | roco8 | DDB_G0286127 |  |
| roco9 | 150174 | DPU_G0072160 | roco9 | DDB_G0288183 |  |
| roco10 | 96855# |  | roco10 | DDB_G0291710 |  |
| - | - | - | roco11 | DDB_G0268636 |  |

Individual rows or series of consecutive rows separated by black lines correspond to inferred orthologous groups of genes between *D. purpureum* and *D. discoideum* (based on BLASTP searches and phylogenetic analyses, see Figures S15, S16 and S17). Rows separated by grey lines comprise lineage-specific paralogs presumably originating from gene duplications that occurred independently in *D. purpureum* or *D. discoideum* lineages. Dashes indicate absence of discernible orthologs in one or the other species, either due to gene loss or potentially due to technical limitations (e.g. too extensive divergence) precluding inference of actual orthologous relationships.

^a^Gene names proposed following names of unambiguous *D. discoideum* orthologs; NA – name not assigned.

^b^JGI *Dictyostelium purpureum* QSDP1 protein models [13].

^c^Official *D. discoideum* gene names and corresponding protein IDs taken from dictyBase [14].

*loci affected by a gap or an error in the genome assembly.

#alternative JGI models replacing models originally selected for the Gene Catalog.

$new models created by modification of existing JGI models.

**
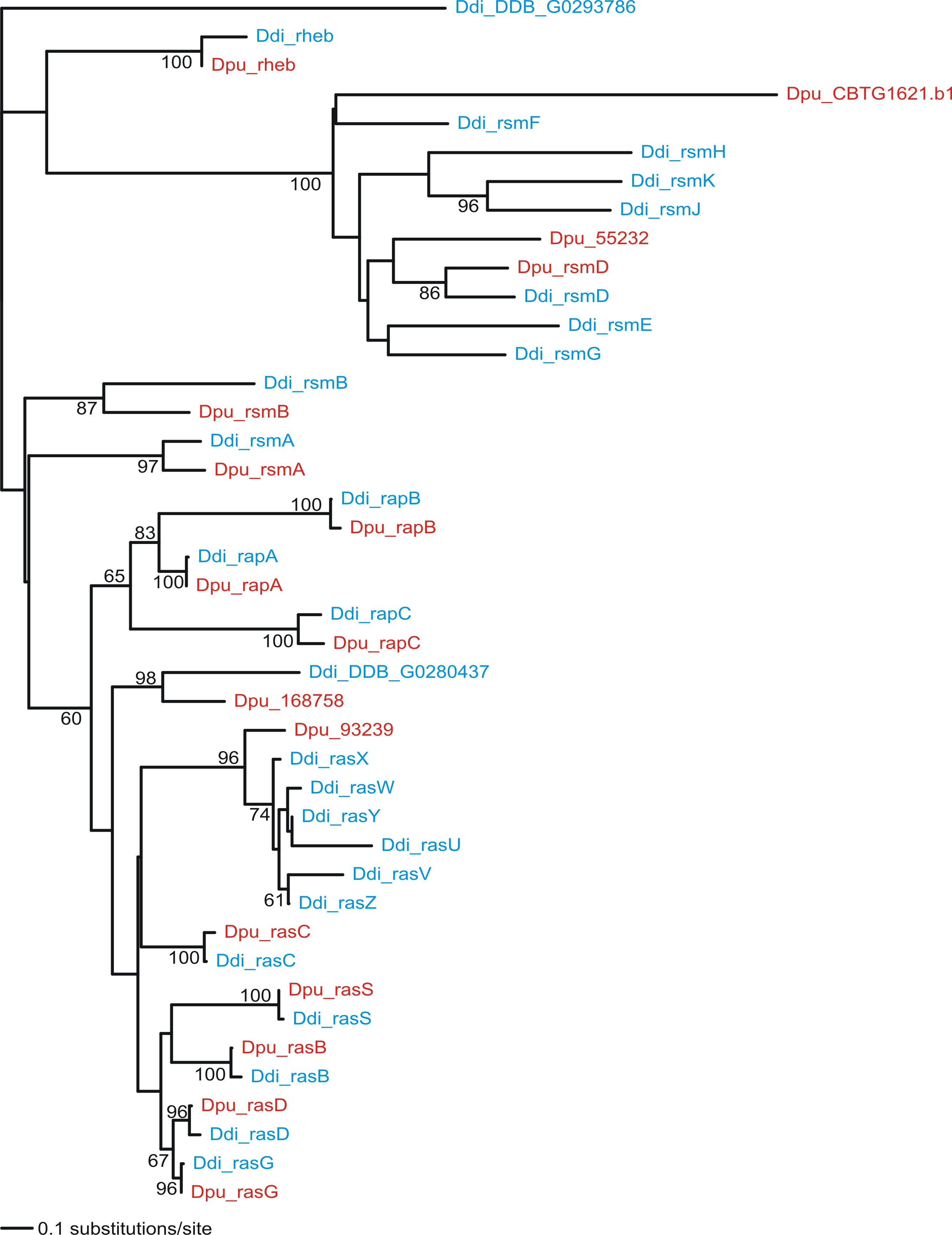
**

**Figure S15. Phylogenetic analysis of the Ras family in *D. discoideum* and *D. purpureum*.** The tree was inferred from an alignment constructed with the aid of ClustalX and extensive manual curation. Ambiguously aligned positions were discarded and the remaining positions were subjected to maximum likelihood tree search using RAxML 7.0.4 (WAG+Γ4+I+F substitution model) and the rapid bootstrapping algorithm (100 bootstrap replicates, branch support values shown only when higher than 50). Sequences that were too divergent and pseudogenes were excluded from the analysis.

**
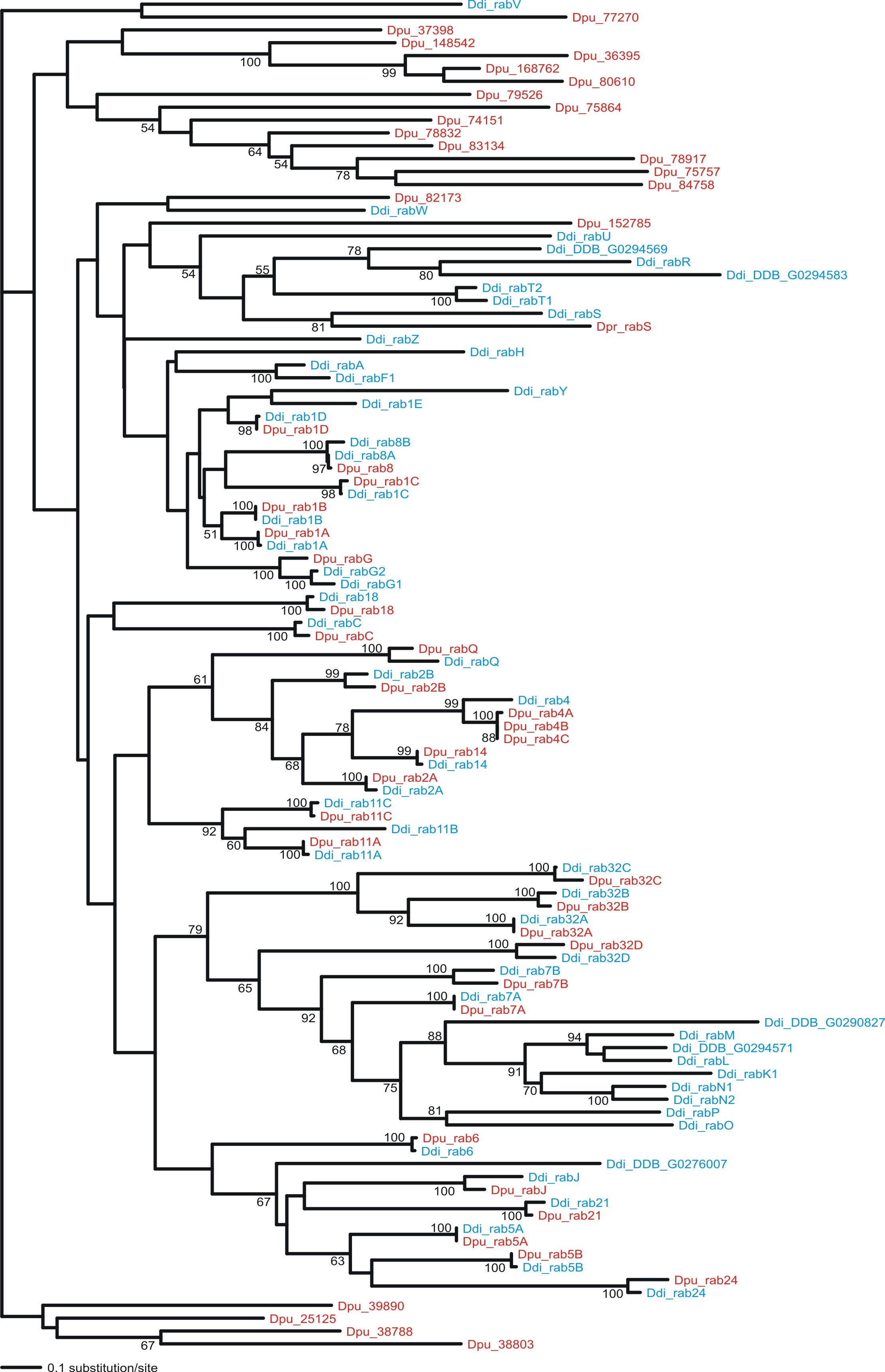
**

**Figure S16. Phylogenetic analysis of the Rab family in *Dictyostelium* spp.** For methodical details see the legend of Figure S15.

**
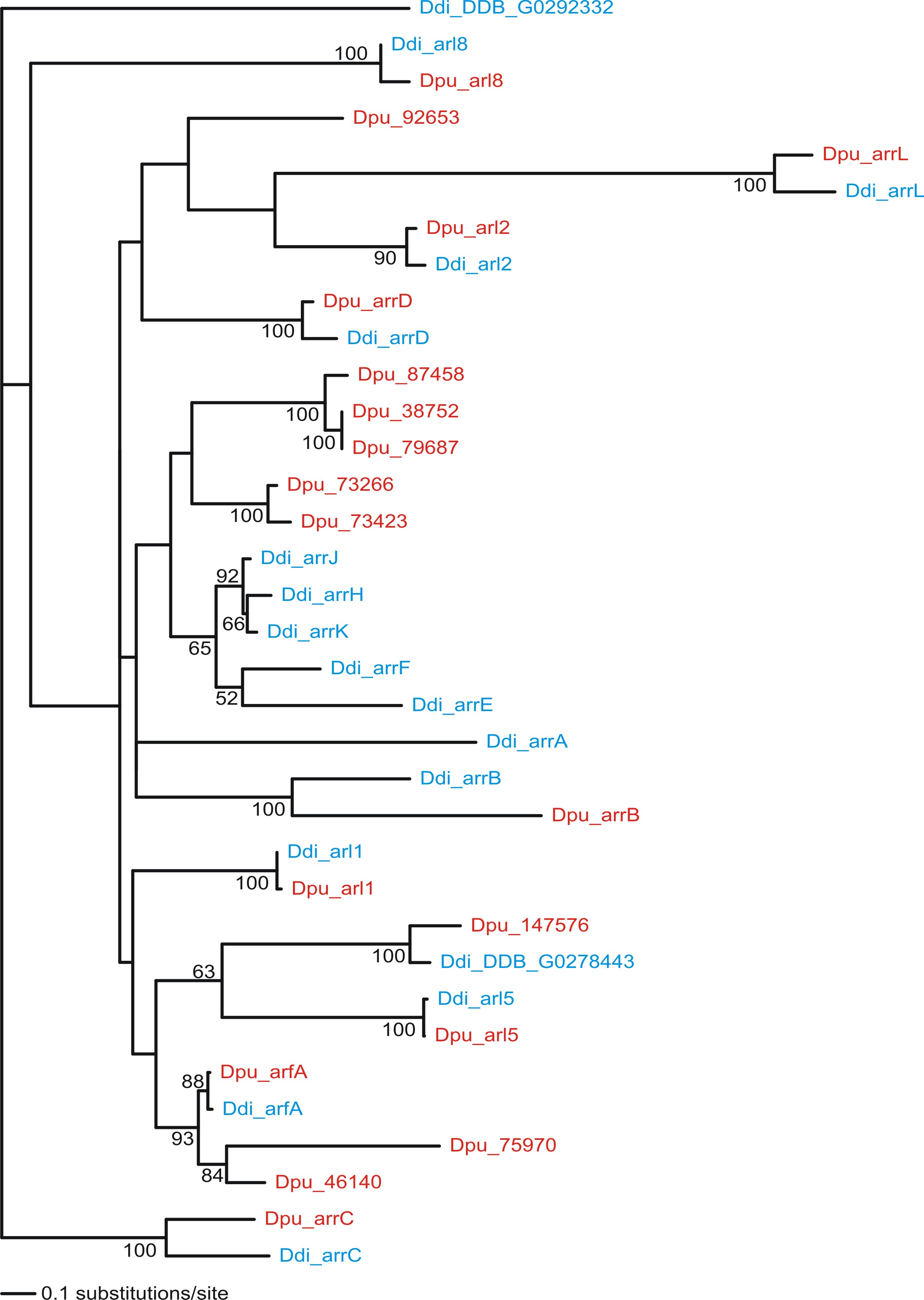
**

**Figure S17. Phylogenetic analysis of the ARF/Sar1 family in *Dictyostelium* spp.** For methodical details see the legend of Figure S15.

###### The *D. pupureum* Glycome

Comparison of predicted protein sequences of *D. purpureum* with the CAZy classification [55] [56] of known glycosyltransferases (GTs), glycohydrolases (GHs), carbohydrate binding modules which are appended to enzyme domains (CBMs), esterases, and expansins are all described in supplemental Table S16. The predicted *D. purpureum* proteins were searched for sequences that could be classified according to the CAZy scheme of glycosyltransferases, glycohydrolases, carbohydrate-binding modules associated with the above, carbohydrate esterases which modify glycans, and expansins. Additional sequences related to glycophosphotransferases, carbohydrate-binding domains (not typically associated with glycoenzymes), and extracellular matrix proteins (some of which have cellulose-binding activity) are also included (denoted as “other”). Proteins with multiple domains fitting these criteria are counted multiple times. Duplicate genes resulting from chromosomal duplications in axenic strains are not counted. Genes within each class are enumerated in Tables S17, S18, and S19 as indicated.

***Storage carbohydrates.*** The cytoplasm is also the site of accumulation of glycogen, an α-glucan polysaccharide that serves as a nutritional store and whose abundance influences cell fate within the slug. Glycogen synthesis and metabolizing enzymes have been described in *D. discoideum* [54], but gene predictions suggest distinctive features that are not fully understood. Both *D. discoideum* and *D. purpureum* lack glycogenin, the primer and initiating glucosyltransferase of glycogen synthesis in animals, plants and fungi, though yeast can synthesize glycogen in the absence of glycogenin [126]. In addition to a conventional family GT3 glycogen synthase-like gene, *D. discoideum* and *D. purpureum* each have a related family GT5 starch synthase-like gene that is similar to that of glaucophytes and red algae, and a GH77 4-α-glucanotransferase (transglycosidase)-like gene [127]. However, other genes associated with starch metabolism are lacking raising the possibility that these genes have unknown alternative functions. Trehalose is a second storage carbohydrate that accumulates to a high level in spores where it may stabilize proteins during desiccation and provide nutrition after germination [128]. *D. discoideum* and *D. purpureum* each have genes associated with trehalose metabolism (GH15, GH37, GH65 and GT20 families) indicative of use of a eukaryotic rather than a prokaryote-type pathway (Table S18).

***Cellulose.*** During development, the slug becomes enveloped within an extracellular, cellulose-based slime sheath and later, during terminal fruiting body formation, each cell acquires its own cellulose-based cell wall. The cellulose is predominantly microfibrillar and assembled via the action of a single CAZy GT2 family synthase [129] that is highly conserved between *D. discoideum* and *D. purpureum*. Sheath and stalk cell walls are invested with distinct classes of small proteins (sheathins) containing or consisting exclusively of cellulose-binding CBDs [130]. Both classes are represented as large multigene families of up to 36 members (Table S16). The spore coat is specialized by the presence of the Gal/GalNAc-rich polysaccharide and 2 diglycosyltransferase genes required for its formation [131] are also conserved in *D. purpureum*. The *D. discoideum* spore coat also contains at least 8 major coat proteins, two of which have cellulose-binding activity *in vitro* [132]. *D. purpureum* orthologs are found for six and are evidently evolving more rapidly than most proteins, as their amino acid sequences are only ~61% similar and 48% identical, and half exhibit additional or alternative Cys-rich domains.

**Table S16. Genes of the Dictyostelid glycome** (gene number differences in orange).

| CAZy Family |  |  | Glycogene Class |
| --- | --- | --- | --- |
| GT family^1^ | D. purpureum | D. discoideum | Glycosyltransferases/glycophosphotransferases |
| 1 | 3 | 4 | UGT small molecule |
| 2 | 9 | 9 | cytoplasmic inverting |
| 3 | 1 | 1 | glycogen synthase |
| 4 | 7 | 7 | E7E retaining |
| 5 | 1 | 1 | starch or α3glucan synthase |
| 8 | 2 | 1 | retaining, secretory |
| 10 | 6 | 11 | α3/4FucT |
| 13 | 2 | 2 | Gnt1 (N-glycan); POMGnt1 |
| 14 | 3 | 3 | Core2, XylT, I-branch β6GlcNAcT |
| 17 | 0 | 4 | bisect/intersect βGlcNAcT |
| 20 | 2 | 3 | trehalose-PO_4_ synthase |
| 22 | 3 | 3 | alg9, alg12, αManT (GPI) |
| 24 | 1 | 1 | Parodi enzyme |
| 33 | 1 | 1 | β4ManT, alg1 |
| 35 | 2 | 2 | glycogen phosphorylase |
| 39 | 1 | 1 | prot O-ManT |
| 41 | 2 | 1 | Spy OGT |
| 49 | 2 | 4 | β3GlcNAcT |
| 50 | 1 | 1 | pigM (GPI α4ManT-I) |
| 57 | 3 | 2 | βGlcT |
| 58 | 2 | 2 | alg3 |
| 59 | 1 | 1 | α2GlcT for N-glycan prec |
| 60 | 2 | 2 | gnt1 (Skp1), gnt2 (modB) |
| 66 | 1 | 1 | stt3 (oligosaccharyltransferase) |
| 74 | 1 | 1 | pgtA (α2FucT for Skp1) |
| 76 | 1 | 1 | α6ManT (pigV GPI) |
| 77 | 1 | 1 | agtA (Skp1) |
| other | 1 | 1 | GT10/23-like, α3/6-FucT-like |
| other | 1 | 1 | alg7 (GlcNAc-PO_4_-T to Dol-P) |
| other | 1 | 1 | GlcNAc-PO_4_-T (Man-6-PO_4_) |
| other | 2 | 9 | glycophosphotransferase |
| Total | 66 | 83 |  |
|  |  |  |  |
| GH family^2^ | D. purpureum | D. discoideum | Glycohydrolases |
| 2 | 1 | 1 | β-glycosidase (potential endo-β-mannosidase) |
| 3 | 1 | 1 | β-glycosidase (bacteria-type) |
| 5 | 6 | 5 | β-glycosidase |
| 7 | 1 | 1 | reducing end cellobiohydrolase |
| 9 | 8 | 8 | cellulase |
| 13 | 5 | 5 | α-amylase |
| 15 | 2 | 2 | trehalase, glucoamylase |
| 16 | 2 | 2 | polysaccharidase |
| 18 | 13 | 7 | chitinase |
| 20 | 3 | 5 | nagA β-GlcNAcase |
| 22 | 3 | 5 | lysozyme C |
| 23 | 3 | 0 | lysozyme G |
| 24 | 0 | 2 | lysozyme |
| 25 | 6 | 6 | lysozyme |
| 27 | 2 | 2 | α-galactosidase, α-GalNAcase |
| 29 | 2 | 2 | α-fucosidase |
| 31 | 2 | 2 | α-glucosidase |
| 35 | 2 | 2 | β-galactosidase |
| 37 | 1 | 1 | trehalase |
| 38 | 11 | 7 | manA (α-mannosidase-II) |
| 39 | 2 | 3 | β-glycosidase |
| 47 | 8 | 6 | α-mannosidase-I |
| 63 | 1 | 1 | α-glucosidase |
| 65 | 1 | 1 | trehalase, trehalose phosphorylase |
| 77 | 1 | 1 | 4-α-glucanotransferase |
| 85 | 1 | 1 | endoglucosaminidase |
| 89 | 1 | 1 | α-N-acetylglucosaminidase |
| Total | 89 | 80 |  |
|  |  |  |  |
| CBM family^2^ | D. purpureum | D. discoideum | Carbohydrate binding modules (appended to enzyme domains) |
| 8 | 3 | 4 | *Dictyostelium*-specific cellulose binding (e.g., celA) |
| 13 | 3 | 10 | Cup (cytoplasmic) |
| 20 | 5 | 5 | glucoamylase, laforin |
| 33 | 3 | 3 | *Dictyostelium*- & bacteria-specific chitin-binding |
| 48 | 3 | 3 | glycogen-binding, AMPK |
| 49 | 39 | 45 | cellulose-binding, sheathins |
| 50 | 7 | 4 | binds chitin or peptidoglycan |
| Total | 63 | 74 |  |
|  |  |  |  |
| CBD family^3^ | D. purpureum | D. discoideum |  |
| other | 13 | 10 | discoidin |
| other | 2 | 2 | comitin or comitin-like |
| Total | 15 | 12 |  |
|  |  |  |  |
| CE family^1^ | D. purpureum | D. discoideum | Carbohydrate esterases |
| 1 | 1 | 1 |  |
| 2 | 2 | 2 |  |
| Total | 3 | 3 |  |
|  |  |  |  |
| Expansin-like^2^ | D. purpureum | D. discoideum | Expansin-related proteins |
|  | 7 | 7 |  |
|  |  |  |  |
| Spore coat^3^ | D. purpureum | D. discoideum | Spore coat proteins |
|  | 6 | 8 |  |
|  |  |  |  |
| Grand Total | 249 | 267 |  |
|  |  |  |  |

^1^enumerated in Table S17

^2^enumerated in Table S18

^3^enumerated in Table S19.

Table S17. Glycosyltransferases/glycophosphotransferases and Carbohydrate esterases of the Dictyostelids.

| D. p.  Protein ID | D. p. dictyBase Gene I.D. | Name | D. discoideum  Ortholog | Family | Predicted function | %  ident. | | % target  (amino acids) |
| --- | --- | --- | --- | --- | --- | --- | --- | --- |
| Glycosyltransferases (GT Families) | | | | | | | | |
| 74375 | DPU_G0073034 |  | DDB_G0278473 | unassigned | distantly related to fucosyltransferases | | 34 | 77(543) |
| 95768 | DPU_G0066680 | ugt52 | DDB_G0288655 | GT Family 1 | UDP-Glc: sterol glucosyltransferase | | 58 | 100(1756) |
| 88818 | DPU_G0058150 | ugt1 | DDB_G0290339 | GT Family 1 | distantly related to β-GTs | | 63 | 99(398) |
| 35006 | DPU_G0055140 | ugt2 | DDB_G0268540 | GT Family 1 | related to β-GTs | | 46 | 100(440) |
| 73885 | DPU_G0051156 |  | DDB_G0289973 | GT Family 2 | candidate β-GT | | 43 | 84(490) |
| 146960 | DPU_G0071316 |  | DDB_G0278817 | GT Family 2 | candidate β-GT | | 62 | 97(612) |
| 92136 | DPU_G0054322 | dcsA | DDB_G0269124 | GT Family 2 | cellulose synthase | | 88 | 100(1067) |
| 35249 | DPU_G0055570 | dgtA | DDB_G0288321 | GT Family 2 | candidate dolichol-phosphate β-glucosyltransferase | | 77 | 100(327) |
| 30502 | DPU_G0072038 | dgtB | DDB_G0286519 | GT Family 2 | candidate GDP-Man: Dol-phosphate β-mannosyltransferase | | 82 | 99(251) |
| 47347 | DPU_G0075876 | pgtB | DDB_G0290079 | GT Family 2; GT Family 4 | bifunctional β-GT/α-GT | | 66 | 99(1343) |
| 56261 | DPU_G0056760 | pgtC | DDB_G0284107 | GT Family 2; GT Family 4 | bifunctional β-GT/α-GT | | 48 | 94(1029) |
| 87341 | DPU_G0074572 | pgtD | DDB_G0278731 | GT Family 2; GT Family 4 | candidate multimodular bifunctional β-GT/α-GT | | 55 | 96(2185) |
| 159362 | DPU_G0070358 | pgtA | DDB_G0283761 | GT Family 2; GT Family 74 | bifunctional β-1,3-GalT/α-1,2-fucT | | 57 | 99(771) |
| 148003 | DPU_G0058290 | glcS | DDB_G0267674 | GT Family 3 | glycogen synthase | | 69 | 100(894) |
| 45300 | DPU_G0058250 | alg11 | DDB_G0292118 | GT Family 4 | α-1,2-mannosyltransferase | | 75 | 95(479) |
| 88372 | DPU_G0055520 | alg2 | DDB_G0272730 | GT Family 4 | α-1,3-mannosyltransferase | | 73 | 99(417) |
| 42846 | DPU_G0069094 | gtr1 | DDB_G0274373 | GT Family 4 | candidate bifunctional GT/sulfotransferase | | 60 | 100(1321) |
| 148633 | DPU_G0063480 | pigA | DDB_G0283965 | GT Family 4 | UDP-GlcNAc: phosphatidylinositol α-1,6-GlcNAcT | | 93 | 88(460) |
| 158449 | DPU_G0067810 | gtr2 | DDB_G0276105 | GT Family 5 | candidate bifunctional protein: distantly related to amylases and glycogen synthases | | 67 | 99(2500) |
| 86718 | DPU_G0068020 | gtr3 | DDB_G0286945 | GT Family 8 | candidate α-GT | | 62 | 99(367) |
| 154205 | DPU_G0057064 |  |  | GT Family 8 | related to α-GTs | |  |  |
| 57337 | DPU_G0061546 | fut10 | DDB_G0286889 | GT Family 10 | candidate α3/4-FucT | | 51 | 97(644) |
| 156375 | DPU_G0062344 | fut9 | DDB_G0293768 | GT Family 10 | candidate α3/4-FucT | | 56 | 100(560) |
| 157080 | DPU_G0064180 | fut7 | DDB_G0283287 | GT Family 10 | candidate α3/4-FucT | | 57 | 88(435) |
| 6690 | DPU_G0064160 | fut4 | DDB_G0284551 | GT Family 10 | candidate α3/4-FucT | |  |  |
| 80087 | DPU_G0055858 | fut8 | DDB_G0280571 | GT Family 10 | potential N-acetyl-β-D-glucosaminide α-1,6-FucT | | 49 | 92(589) |
| 27418 | DPU_G0058850 | fut11 | DDB_G0268190 | GT Family 10 | candidate α3/4-FucT | | 47 | 93(788) |
| 88972 | DPU_G0059144 | gnt3 | DDB_G0288463 | GT Family 13 | candidate β-1,2-GlcNAcT, β2GnT1? (complex) | | 46 | 98(490) |
| 91685 | DPU_G0072472 | gnt4 | DDB_G0268854 | GT Family 13 | related to β-N-acetylglucosaminyltransferases | | 58 | 88(572) |
| 93669 | DPU_G0059368 | gnt10 | DDB_G0269900 | GT Family 14 | candidate β-1,6-GlcNAcT or polypeptide βXylT | | 27 | 78(644) |
| 96706 | DPU_G0060270 | gnt11 | DDB_G0269558 | GT Family 14 | candidate β-1,6-GlcNAcT or polypeptide βXylT | | 40 | 99(501) |
| 149262 | DPU_G0067706 | gnt9 | DDB_G0268204 | GT Family 14 | candidate β-1,6-GlcNAcT or polypeptide βXylT | | 40 | 65(419) |
| 46972 | DPU_G0074060 | tpsB | DDB_G0284975 | GT Family 20 | candidate α,α-trehalose-6-phosphate synthase/phosphatase | | 81 | 99(788) |
| 47494 | DPU_G0051860 | tpsC | DDB_G0290405 | GT Family 20 | candidate α,α-trehalose-6-phosphate synthase/phosphatase | | 70 | 100(897) |
| 149392 | DPU_G0068484 | smp3 | DDB_G0284459 | GT Family 22 | candidate Dol-P-Man: α-1,2-mannosyltransferase | | 41 | 98(1231) |
| 96848 | DPU_G0063556 | alg9 | DDB_G0279349 | GT Family 22 | Dol-P-Man: α-1,2-mannosyltransferase | | 74 | 98(634) |
| 80121 | DPU_G0056042 | alg12 | DDB_G0267884 | GT Family 22 | Dol-P-Man: α-1,6-mannosyltransferase | | 44 | 98(633) |
| 82586 | DPU_G0063038 | ggtA | DDB_G0274103 | GT Family 24 | UDP-glucose:glycoprotein α-glucosyltransferase (Parodi enz.) | | 60 | 100(1714) |
| 52859 | DPU_G0059326 | alg1 | DDB_G0286011 | GT Family 33 | GDP-mannose β-1,4-mannosyltransferase (mntA) | | 70 | 97(477) |
| 45567 | DPU_G0061194 | glpD | DDB_G0291123 | GT Family 35 | glycogen phosphorylase | | 84 | 99(993) |
| 51433 | DPU_G0070164 | glpV | DDB_G0281383 | GT Family 35 | glycogen phosphorylase | | 93 | 99(846) |
| 99420 | DPU_G0065082 | pmtA | DDB_G0284847 | GT Family 39 | related to protein O-mannosyltransferases | | 82 | 100(211) |
| 39606 | DPU_G0062462 | spyA | DDB_G0285263 | GT Family 41 | O-βGlcNAcT, cytoplasmic | | 58 | 92(810) |
| 151710 | DPU_G0051722 | spyB |  | GT Family 41 | O-βGlcNAcT, cytoplasmic | |  |  |
| 51901 | DPU_G0073636 | gnt13 | DDB_G0282469 | GT Family 49 | candidate β-GT | | 45 | 84(536) |
| 152182 | DPU_G0052592 | gnt15 | DDB_G0274741 | GT Family 49 | candidate β-GT | | 48 | 85(440) |
| 158484 | DPU_G0067852 | pigM | DDB_G0288899 | GT Family 50 | Dol-phosphate-mannose α-1,4-mannosyltransferase | | 83 | 98(431) |
| 38502 | DPU_G0060630 | alg8 | DDB_G0275261 | GT Family 57 | Dol-P-Glc: α-1,3-GlcT2 | | 67 | 96(602) |
| 43700 | DPU_G0071234 | alg6 | DDB_G0283841 | GT Family 57 | Dol-P-Glc: α-1,3-GlcT1 | | 72 | 99(525) |
| 37084 | DPU_G0058522 | alg3 | DDB_G0268238 | GT Family 58 | Dol-P-Man: α-1,2/1,3-mannosyltransferase | | 61 | 99(459) |
| 153070 | DPU_G0054568 | pigB | DDB_G0279825 | GT Family 58 | related to Dol-P-Man: α-1,2-mannosyltransferases | | 47 | 98(1215) |
| 85245 | DPU_G0072078 | alg10 | DDB_G0277817 | GT Family 59 | related to Dol-P-Glc: α-1,2-GlcT1 | | 56 | 46(265) |
| EST only |  | gnt2 | DDB_G0291241 | GT Family 60 | polypeptide α-GlcNAcT (mucin-type) | |  |  |
| 89733 | DPU_G0063838 | gnt1 | DDB_G0275699 | GT Family 60 | UDP-GlcNAc:HyPro Skp1 polypeptide α-GlcNAc-transferase | | 56 | 100(425) |
| 150543 | DPU_G0073564 | stt3 | DDB_G0285159 | GT Family 66 | oligosaccharyltransferase subunit STT3 | | 90 | 100(714) |
| 98471 | DPU_G0056892 | pigV | DDB_G0280891 | GT Family 76 | Dol-P-Man: α6-mannosyltransferase | | 56 | 99(647) |
| 152231 | DPU_G0052824 | agtA | DDB_G0283005 | GT Family 77 | UDP-Gal:fucoside α-1,3-galactosyltransferase | | 62 | 99(642) |
| Predicted glycophosphotransferases | | | | | | | | |
| 158811 | DPU_G0068698 | gpt1 | DDB_G0285815 |  | N-glycan: GlcNAc-PO4-transferase | 54 | |  |
| 158821 | DPU_G0068712 | gpt5 | DDB_G0274707 |  | glycophosphotransferase | 49 | |  |
| 97408 | DPU_G0073430 | gpt10 | DDB_G0281103 |  | glycophosphotransferase | 82 | |  |
| 158539 | DPU_G0068028 | alg7 | DDB_G0290751 |  | Dol-P-dependent GlcNAc-1-P transferase (N-glycan) |  | |  |
| Predicted carbohydrate esterases | | | | | | | | |
| 74322 | DPU_G0071384 | esd | DDB_G0283037 | CE Family 1 | candidate esterase, lacks signal peptide | 62 | | 99(282) |
| 27230 | DPU_G0057788 |  | DDB_G0275371 | CE Family 2 | candidate esterase | 66 | | 99(363) |
| 37730 | DPU_G0059526 |  | DDB_G0287647 | CE Family 2 | candidate esterase, lacks signal peptide | 64 | | 82(347) |

Table S18. glycohydrolase, carbohydrate binding module, and expansin-like domain containing proteins of the Dictyostelids.

| D. p.  Protein ID. | D. p. dictyBase Gene I.D. | Name | D.d. Ortholog | Family | Predicted Function | % ident. | % of target  (amino acids) |
| --- | --- | --- | --- | --- | --- | --- | --- |
| Glycohydrolases | | | | | | | |
| 97798 | DPU_G0051944 | manH | DDB_G0275917 | GH Family 02 | candidate β-mannosidase | 57 | 100(1056) |
| 48170 | DPU_G0054796 | gluA | DDB_G0292810 | GH Family 03 | candidate β-glucosidase | 81 | 100(831) |
| 91906 | DPU_G0075826 |  |  | GH Family 05 | candidate β-glycosidase |  |  |
| 52841 | DPU_G0058880 |  | DDB_G0286025 | GH Family 05 | candidate β-glycosidase; CBM8 module; GPI anchor, putative cellulase | 79 | 96(537) |
| 47102 | DPU_G0074860 |  | DDB_G0286061 | GH Family 05 | candidate β-glycosidase; CBM8 module; GPI-anchor | 61 | 100(547) |
| 152047 | DPU_G0052360 |  | DDB_G0289953 | GH Family 05 | candidate endoglucanase | 50 | 100(793) |
| 148310 | DPU_G0060794 |  | DDB_G0270190 | GH Family 05 | candidate endoglucanase; CBM49 module, putative cellulase | 66 | 96(551) |
| 148335 | DPU_G0060842 |  | DDB_G0270074 | GH Family 05 | candidate endoglycoceramidase | 73 | 98(498) |
| 151874 | DPU_G0051994 | cbhA | DDB_G0268446 | GH Family 07 | candidate [reducing end] b-1,4-cellobiohydrolase | 80 | 99(453) |
| 39756 | DPU_G0062978 | celA | DDB_G0271134 | GH Family 09 | endoglucanase, CBM8 expected | 82 | 62(434) |
| 87197 | DPU_G0073568 |  | DDB_G0284295 | GH Family 09 | candidate endoglucanase | 83 | 90(434) |
| 31382 | DPU_G0074154 |  | DDB_G0279995 | GH Family 09 | candidate endoglucanase | 61 | 60(501) |
| 40165 | DPU_G0063752 |  | DDB_G0284239 | GH Family 09 | candidate endoglucanase | 74 | 66(449) |
| 40261 | DPU_G0063986 |  | DDB_G0286321 | GH Family 09 | candidate endoglucanase | 55 | 91(520) |
| 85868 | DPU_G0074378 |  | DDB_G0271314 | GH Family 09 | candidate endoglucanase | 76 | 100(454) |
| 36716 | DPU_G0057932 |  | DDB_G0286277 | GH Family 09 | candidate endoglucanase; GPI anchor | 53 | 82(499) |
| 153014 | DPU_G0054480 |  |  | GH Family 09 | candidate endoglucanase; GPI anchor |  |  |
| 50145 | DPU_G0063042 | glgB | DDB_G0274105 | GH Family 13 | α-1,4-glucan branching enzyme ; CBM48 module, α-amylase | 84 | 100(675) |
| 45888 | DPU_G0065672 | agl | DDB_G0287569 | GH Family 13 | glycogen debranching enzyme amylo-α-1,6-glucosidase | 71 | 100(1608) |
| 55676 | DPU_G0054822 | amyA | DDB_G0281547 | GH Family 13 | candidate α-amylase | 80 | 100(469) |
| 87665 | DPU_G0051736 | amyB | DDB_G0286079 | GH Family 13 | candidate α-amylase | 88 | 96(495) |
| 55969 | DPU_G0055704 | amyC | DDB_G0282715 | GH Family 13 | candidate trehalose synthase, α-amylase | 76 | 100(778) |
| 33519 | DPU_G0052964 |  | DDB_G0277045 | GH Family 15 | candidate α,α-trehalase | 77 | 83(545) |
| 92511 | DPU_G0059274 |  | DDB_G0276215 | GH Family 15 | candidate α-glycosidase | 91 | 98(638) |
| 149133 | DPU_G0066922 |  | DDB_G0280263 | GH Family 16 | candidate endo-β1,3/4-glucanase | 66 | 89(244) |
| 48687 | DPU_G0056598 |  | DDB_G0292848 | GH Family 16 | inactive β-glycosidase (missing catalytic residues) | 67 | 94(951) |
| 50758 | DPU_G0066176 |  | DDB_G0268090 | GH Family 18 | candidate chitinase | 63 | 97(252) |
| 47033 | DPU_G0074484 | ctbsB | DDB_G0276795 | GH Family 18 | candidate di-N-acetylchitobiase | 58 | 99(374) |
| 156870 | DPU_G0063718 | ctbsA | DDB_G0274233 | GH Family 18 | di-N-acetylchitobiase | 58 | 97(372) |
| 75595 | DPU_G0061716 | chid1 | DDB_G0290417 | GH Family 18 | inactive chitinase (missing catalytic residues), chitinase domain-containing protein 1 | 51 | 99(388) |
| 85320 | DPU_G0072384 |  |  | GH Family 18 | candidate inactive chitinase (missing catalytic residues) |  |  |
| 93366 | DPU_G0057482 |  | DDB_G0278071 | GH Family 18 | candidate inactive chitinase (missing catalytic residues) | 33 | 90(292) |
| 75666 | DPU_G0062690 |  | DDB_G0287463 | GH Family 18 | related to chitinases | 25 | 73(331) |
| 88230 | DPU_G0054814 |  |  | GH Family 18 | related to chitinases |  |  |
| 91849 | DPU_G0075114 |  |  | GH Family 18 | related to chitinases |  |  |
| 98002 | DPU_G0053622 |  | DDB_G0287465 | GH Family 18 | related to chitinases | 42 | 83(383) |
| 149996 | DPU_G0071494 |  |  | GH Family 18 | related to chitinases |  |  |
| 151171 | DPU_G0075100 |  |  | GH Family 18 | related to chitinases |  |  |
| 96643 | DPU_G0058838 |  |  | GH Family 18 | related to chitinases |  |  |
| 30528 | DPU_G0072046 | nagA | DDB_G0287033 | GH Family 20 | N-acetyl β-glucosaminidase | 73 | 100(533) |
| 83242 | DPU_G0064898 | nagE | DDB_G0285647 | GH Family 20 | related to N-acetyl β-glucosaminidase | 44 | 94(663) |
| 93851 | DPU_G0065710 | nagD | DDB_G0287659 | GH Family 20 | related to N-acetyl β-glucosaminidase | 46 | 92(563) |
| 41144 | DPU_G0065448 | lysB | DDB_G0278563 | GH Family 22 | distantly related to lysozymes, lysozyme C family | 75 | 94(190) |
| 151645 | DPU_G0051586 |  | DDB_G0288143 | GH Family 22 | distantly related to lysozymes, lysozyme C family | 36 | 57(111) |
| 156720 | DPU_G0063338 | rcdBB | DDB_G0274551 | GH Family 22 | distantly related to lysozymes | 41 | 100(339) |
| 147060 | DPU_G0073036 |  |  | GH Family 23 | distantly related to lysozymes, similar to lysozyme C |  |  |
| 153079 | DPU_G0054584 |  |  | GH Family 23 | distantly related to lysozymes |  |  |
| 153511 | DPU_G0055578 |  |  | GH Family 23 | distantly related to lysozymes |  |  |
| 20023 | DPU_G0055886 | cf45-1 | DDB_G0269248 | GH Family 25 | distantly related to lysozymes, counting factor complex protein | 61 | 99(297) |
| 31893 | DPU_G0075018 | cf50-1 | DDB_G0273175 | GH Family 25 | distantly related to lysozymes | 62 | 99(302) |
| 39207 | DPU_G0061924 |  | DDB_G0293566 | GH Family 25 | distantly related to lysozymes | 58 | 100(217) |
| 81570 | DPU_G0060048 |  | DDB_G0274181 | GH Family 25 | distantly related to lysozymes | 62 | 100(215) |
| 88463 | DPU_G0055994 |  | DDB_G0293492 | GH Family 25 | distantly related to lysozymes | 65 | 100(215) |
| 154665 | DPU_G0058236 |  | DDB_G0276439 | GH Family 25 | distantly related to lysozymes |  |  |
| 58841 | DPU_G0069506 |  | DDB_G0291524 | GH Family 27 | candidate α-galactosidase, or alpha-N-acetylgalactosaminidase | 68 | 84(393) |
| 86485 | DPU_G0064266 |  | DDB_G0294140 | GH Family 27 | candidate α-galactosidase | 74 | 99(384) |
| 58554 | DPU_G0067796 | alfB | DDB_G0276661 | GH Family 29 | α-L-fucosidase | 62 | 99(456) |
| 88361 | DPU_G0055470 | alfA | DDB_G0274391 | GH Family 29 | α-L-fucosidase | 65 | 99(459) |
| 49160 | DPU_G0058762 | modA | DDB_G0269154 | GH Family 31 | α glucosidase II | 75 | 99(938) |
| 157944 | DPU_G0066330 | gaa | DDB_G0269790 | GH Family 31 | candidate α-glucosidase | 78 | 100(867) |
| 157172 | DPU_G0064456 | glb1 | DDB_G0290217 | GH Family 35 | candidate β-galactosidase | 64 | 94(637) |
| 48969 | DPU_G0058132 | glb2 | DDB_G0285637 | GH Family 35 | candidate β-galactosidase | 57 | 99(757) |
| 41704 | DPU_G0066730 |  | DDB_G0283473 | GH Family 37 | candidate α,α-trehalase | 69 | 92(548) |
| 49593 | DPU_G0060478 | manA | DDB_G0292206 | GH Family 38 | candidate α-mannosidase | 70 | 100(1008) |
| 55378 | DPU_G0053628 | manG | DDB_G0287577 | GH Family 38 | candidate α-mannosidase | 67 | 99(1082) |
| 34840 | DPU_G0054962 | manB | DDB_G0278259 | GH Family 38 | related to α-mannosidases | 63 | 100(1043) |
| 47917 | DPU_G0053730 | manF | DDB_G0287231 | GH Family 38 | related to α-mannosidases | 59 | 100(994) |
| 98237 | DPU_G0055394 | manC | DDB_G0268994 | GH Family 38 | related to α-mannosidases | 55 | 99(1074) |
| 40536 | DPU_G0064404 | manD | DDB_G0278653 | GH Family 38 | related to α-mannosidases; C-terminal membrane anchor | 42 | 98(1212) |
| 45100 | DPU_G0054176 | manE | DDB_G0278651 | GH Family 38 | related to α-mannosidases; C-terminal membrane anchor | 44 | 78(1112) |
| 48877 | DPU_G0057412 | manI |  | GH Family 38 | related to α-mannosidases; C-terminal membrane anchor |  |  |
| 96508 | DPU_G0054174 | manJ |  | GH Family 38 | related to α-mannosidases; C-terminal membrane anchor |  |  |
| 99980 | DPU_G0070820 | manK |  | GH Family 38 | related to α-mannosidases; C-terminal membrane anchor |  |  |
| 74852 | DPU_G0054178 | manL |  | GH Family 38 | related to α-mannosidases; N- and C-terminal membrane anchor |  |  |
| 78906 | DPU_G0052928 |  | DDB_G0281313 | GH Family 39 | candidate β-glycosidase; C-terminal membrane anchor | 49 |  |
| 11305 | DPU_G0053732 |  | DDB_G0288289 | GH Family 39 | candidate β-xylosidase | 83 |  |
| 55071 | DPU_G0052284 | msdA | DDB_G0268754 | GH Family 47 | mannosyl-oligosaccharide a1,2-mannosidase | 59 | 75(493) |
| 86464 | DPU_G0063912 | msdB | DDB_G0284391 | GH Family 47 | mannosyl-oligosaccharide α1,2-mannosidase | 54 | 75(441) |
| 155265 | DPU_G0059682 | msdC | DDB_G0284395 | GH Family 47 | mannosyl-oligosaccharide α1,2-mannosidase; N-terminal membrane anchor | 45 | 95(615) |
| 157585 | DPU_G0065480 | msdD | DDB_G0292918 | GH Family 47 | mannosyl-oligosaccharide α1,2-mannosidase; N-terminal membrane anchor | 51 | 89(511) |
| 91147 | DPU_G0052066 | msdE | DDB_G0277051 | GH Family 47 | mannosyl-oligosaccharide α1,2-mannosidase | 47 | 100(796) |
| 74722 | DPU_G0052784 | msdG |  | GH Family 47 | related to α-mannosidases |  |  |
| 147471 | DPU_G0052790 | msdF | DDB_G0284393 | GH Family 47 | mannosyl-oligosaccharide α1,2-mannosidase | 55 | 38(192) |
| 28384 | DPU_G0063916 | msdH |  | GH Family 47 | too short to be reliably annotated |  |  |
| 53711 | DPU_G0069350 |  | DDB_G0280145 | GH Family 63 | Mannosyl-oligosaccharide glucosidase | 54 | 99(878) |
| 97948 | DPU_G0053166 | athl1 | DDB_G0287109 | GH Family 65 | acid trehalase-like protein 1 | 55 | 100(679) |
| 46840 | DPU_G0073540 |  | DDB_G0284259 | GH Family 77 | candidate 4-α-glucanotransferase; two N-terminal CBM20 modules | 84 | 100(909) |
| 95866 | DPU_G0067914 |  | DDB_G0268520 | GH Family 85 | related to endo-β-N-acetylglucosaminidases | 41 | 100(1362) |
| 26247 | DPU_G0052098 | naglu | DDB_G0291998 | GH Family 89 | related to α-N-acetylglucosaminidases | 70 | 89(725) |
| Carbohydrate binding modules | | | | | | | |
| 95208 | DPU_G0059732 |  | DDB_G0277757 | CBM Family 08 | candidate carbohydrate-binding protein | 57 | 97(368) |
| 76197 | DPU_G0066892 | cupK |  | CBM Family 13 | carbohydrate-binding protein, ricin B lectin domain-containing protein |  |  |
| 158837 | DPU_G0068828 | cupL |  | CBM Family 13 | carbohydrate-binding protein, ricin B lectin domain-containing protein |  |  |
| 15974 | DPU_G0072456 | cupI | DDB_G0272242 | CBM Family 13 | carbohydrate-binding protein, ricin B lectin domain-containing protein | 34 | 87(620) |
| 149039 | DPU_G0066074 |  | DDB_G0270014 | CBM Family 20 | candidate carbohydrate-binding protein | 79 | 92(190) |
| 58838 | DPU_G0069496 |  | DDB_G0272062 | CBM Family 20 | candidate carbohydrate-binding protein | 73 | 100(148) |
| 35223 | DPU_G0055550 |  | DDB_G0290085 | CBM Family 20 | candidate multimodular carbohydrate-binding protein | 63 | 100(1590) |
| 53299 | DPU_G0064966 |  | DDB_G0273103 | CBM Family 33 | candidate carbohydrate-binding protein | 62 | 97(233) |
| 55507 | DPU_G0054064 |  | DDB_G0273835 | CBM Family 33 | candidate carbohydrate-binding protein | 45 | 96(241) |
| 79382 | DPU_G0054062 |  | DDB_G0277201 | CBM Family 33 | candidate carbohydrate-binding protein | 36 | 91(233) |
| 99095 | DPU_G0062228 |  | DDB_G0269710 | CBM Family 48 | candidate carbohydrate-binding protein | 51 | 100(1495) |
| 74639 | DPU_G0052060 | ptdC |  | CBM Family 49 | candidate peptidase C1A; sheathin-like |  | (566) |
| 149760 | DPU_G0070402 | ptdA | DDB_G0281079 | CBM Family 49 | candidate peptidase C1A; sheathin-like | 32 | 97(642) |
| 26246 | DPU_G0052058 | ptdB | DDB_G0281077 | CBM Family 49 | candidate peptidase C1A; two CBM49 modules; C-terminal membrane anchor | 40 | 93(740) |
| 33217 | DPU_G0052432 |  | DDB_G0287275 | CBM Family 49 | sheathin-like | 28 | 50(692) |
| 57109 | DPU_G0060486 | ecmG | DDB_G0292224 | CBM Family 49 | sheathin-like, sheathin-like domains may be solitary | 58 | 94(117) |
| 78730 | DPU_G0052390 | ecmL | DDB_G0293762 | CBM Family 49 | sheathin-like | 43 | 98(1124) |
| 78747 | DPU_G0052430 | ecmM |  | CBM Family 49 | sheathin-like |  | (1118) |
| 93241 | DPU_G0070896 | ecmF | DDB_G0291291 | CBM Family 49 | sheathin-like, sheathin-like domains may be solitary | 49 | 80(103) |
| 152083 | DPU_G0052428 | ecmN |  | CBM Family 49 | sheathin-like |  | (1137) |
| 154318 | DPU_G0057266 | celB | DDB_G0269112 | CBM Family 49 | sheathin-like | 46 | 96(519) |
| 157586 | DPU_G0065482 | ecmD | DDB_G0286647 | CBM Family 49 | sheathin |  | (271) |
| 56144 | DPU_G0056304 | staA | DDB_G0294473 | CBM Family 49 | sheathin-like, sheathin-like domains may be solitary |  | (152) |
| 57657 | DPU_G0063026 |  |  | CBM Family 49 | sheathin-like, sheathin-like domains may be solitary |  | (271) |
| 150550 | DPU_G0073576 |  |  | CBM Family 49 | sheathin-like, sheathin-like domains may be solitary |  | (155) |
| 91080 | DPU_G0071334 |  |  | CBM Family 49 | sheathin-like, sheathin-like domains may be solitary |  | (152) |
| 77419 | DPU_G0073538 |  |  | CBM Family 49 | sheathin-like, sheathin-like domains may be solitary |  | (138) |
| 94401 | DPU_G0075866 |  |  | CBM Family 49 | sheathin-like, sheathin-like domains may be solitary |  | (140) |
| 156962 | DPU_G0063962 |  |  | CBM Family 49 | sheathin-like, sheathin-like domains may be solitary |  | (150) |
| 22202 | DPU_G0058510 |  |  | CBM Family 49 | sheathin-like, sheathin-like domains may be solitary |  | (125) |
| 50740 | DPU_G0066108 |  |  | CBM Family 49 | sheathin-like, sheathin-like domains may be solitary |  | (152) |
| 153444 | DPU_G0055454 |  |  | CBM Family 49 | sheathin-like, sheathin-like domains may be solitary |  | (152) |
| 98184 | DPU_G0054972 |  |  | CBM Family 49 | sheathin-like, sheathin-like domains may be solitary |  | (151) |
| 91241 | DPU_G0056960 |  |  | CBM Family 49 | sheathin-like |  | (198) |
| 35222 | DPU_G0055556 |  |  | CBM Family 49 | sheathin-like, sheathin-like domains may be solitary |  | (164) |
| 78284 | DPU_G0075864 |  |  | CBM Family 49 | sheathin-like, sheathin-like domains may be solitary |  | (164) |
| 148310 | DPU_G0060794 |  |  | CBM Family 49 | sheathin-like |  | (560) |
| 79711 | DPU_G0054974 |  |  | CBM Family 49 | sheathin-like, sheathin-like domains may be solitary |  | (152) |
| 91957 | DPU_G0051890 |  |  | CBM Family 49 | sheathin-like |  | (372) |
| 97652 | DPU_G0075514 |  |  | CBM Family 49 | sheathin-like |  | (476) |
| 40477 | DPU_G0064212 |  |  | CBM Family 49 | sheathin-like, sheathin-like domains may be solitary |  | (151) |
| 12263 | DPU_G0061576 |  |  | CBM Family 49 | sheathin-like, sheathin-like domains may be solitary |  | (151) |
| 149279 | DPU_G0067738 |  |  | CBM Family 49 | sheathin-like, sheathin-like domains may be solitary |  | (151) |
| 154567 | DPU_G0058060 |  |  | CBM Family 49 | sheathin-like |  | (321) |
| 36696 | DPU_G0057944 |  |  | CBM Family 49 | sheathin-like, sheathin-like domains may be solitary |  | (151) |
| 93076 | DPU_G0067812 |  |  | CBM Family 49 | sheathin-like, sheathin-like domains may be solitary |  | (151) |
| 73532 | DPU_G0067806 |  |  | CBM Family 49 | sheathin-like, sheathin-like domains may be solitary |  | (151) |
| 158052 | DPU_G0066778 |  |  | CBM Family 49 | sheathin-like |  | (619) |
| 83697 | DPU_G0066272 |  |  | CBM Family 49 | sheathin-like, sheathin-like domains may be solitary |  | (123) |
| Cell Wall Expansins | | | | | | | |
| 155955 | DPU_G0061384 | expl1 | DDB_G0267846 | EXPN | distantly related to plant expansins | 53 | 86(246) |
| 148118 | DPU_G0059332 | expl5 | DDB_G0276937 | EXPN | distantly related to plant expansins | 70 | 99(236) |
| 20287 | DPU_G0054872 | expl3 | DDB_G0276287 | EXPN | distantly related to plant expansins; C-terminal membrane anchor | 73 | 68(229) |
| 55762 | DPU_G0055032 | expl2 | DDB_G0284677 | EXPN | distantly related to plant expansins; C-terminal membrane anchor | 76 | 98(402) |
| 92439 | DPU_G0058204 | expl10 | DDB_G0284549 | EXPN | distantly related to plant expansins; three CBM50 modules | 52 | 93(277) |
| 37473 | DPU_G0059154 | expl11 |  | EXPN | distantly related to plant expansins; two CBM50 modules |  |  |
| 88764 | DPU_G0057922 | expl12 | DDB_G0284479 | EXPN | distantly related to plant expansins; two CBM50 modules | 53 | 100(270) |
|  |  |  |  |  |  |  |  |

Table S19. Proposed carbohydrate-binding-domain containing and spore coat proteins of the Dictyostelids.

| D.p. Protein I.D. | D. p. dictyBase Gene I.D. | Name | D.d. Ortholog | Predicted Function | % ident. | % of target  (amino acids) |
| --- | --- | --- | --- | --- | --- | --- |
| Comitin-related proteins | | | | | | |
| 75958 | DPU_G0064986 | cmr | DDB_G0279711 | comitin-like protein, 134 aa in Dd |  | (135) |
| 38686 | DPU_G0061038 | comA | DDB_G0289599 | comitin, 185 aa in Dd |  | (201) |
| Spore coat-related proteins | | | | | | |
| 97410 | DPU_G0073446 | cotA | DDB_G0276941 | SP96, 600 aa in Dd | 57 | (489) |
| 96596 | DPU_G0057732 | cotB | DDB_G0276761 | SP70, 537 aa in Dd | 34 | (517) |
| 92698 | DPU_G0061834 | cotD | DDB_G0283149 | SP75, 554 aa in Dd | 53 | (598) |
| 92740 | DPU_G0062794 | pspB | DDB_G0276939 | SP85, cellulose-binding, 532 aa in Dd | 39 | (603) |
| 99604 | DPU_G0066804 | pspD | DDB_G0277379 | SP87, 677 aa in Dd | 72 | (515) |
| 150519 | DPU_G0073470 | psvA | DDB_G0276869 | p58/SP35, cellulose-binding, 513 aa in Dd | 61 | (565) |
| Discoidin-related proteins | | | | | | |
| 52865 | DPU_G0059342 | dscA-1 | DDB_G0273063 | discoidin |  | (255) |
| 160079 | DPU_G0072822 |  |  | discoidin |  | (255) |
| 91146 | DPU_G0052050 |  |  | discoidin |  | (255) |
| 146689 | DPU_G0062490 |  |  | discoidin |  | (255) |
| 75318 | DPU_G0059346 |  |  | discoidin |  | (257) |
| 152743 | DPU_G0053890 |  |  | discoidin |  | (256) |
| 57504 | DPU_G0062294 |  |  | discoidin |  | (253) |
| 58954 | DPU_G0070126 |  |  | discoidin |  | (253) |
| 56540 | DPU_G0058448 |  |  | discoidin |  | (252) |
| 91250 | DPU_G0057764 |  |  | discoidin |  | (253) |
| 93835 | DPU_G0065018 |  |  | discoidin |  | (253) |
| 156718 | DPU_G0063334 |  |  | discoidin |  | (254) |
| 52224 | DPU_G0072994 |  |  | discoidin |  | (278) |
| Sheathin-like proteins | | | | | | |
| see CAZy family CBM 49 carbohydrate binding module entries in Table S18 above. | | | | | | |
| Cup-like proteins | | | | | | |
| see CAZy family CBM 13 carbohydrate binding module entries in Table S18 above. | | | | | | |

###### Social Genes

# Analysis of social genes

We used 12,410 predicted coding sequences and protein sequences of *Dictyostelium purpureum* along with 13,349 of *Dictyostelium discoideum* retrieved from dictyBase (version of 10/25/2009 [14]). Illumina RNA-seq reads from cDNA libraries of the vegetative stage and six social stages at four-hour intervals of 12,713 *D. discoiduem* genes and 12,246 *D. purpureum* genes were obtained by personal communication [15].

We constructed a social expression index for each gene to describe its relative expression in social versus vegetative stages, for genes with > 30 RNA-seq reads at least in one time point of development and high reproducibility between biological replicates (> 0.9 Pearson’s correlation between expression profiles from the two biological replicates) in *D. discoideum* (3,039 genes) and *D. purpureum* (5,751 genes). The number of reads per gene was normalized for each time point and then averaged over all social time points to give average social expression. The social expression index is defined as this social average divided by the social average plus the normalized vegetative count. We also constructed gamete expression indices for genes with at least four RNA-seq ESTs expressed in the vegetative and gamete stages from *D. discoideum* (932 genes).

Comparative analyses of social genes used relatively strict orthology requirements to avoid analyzing false matches. We applied Inparanoid 2.0 [116] for searching orthologs and in-paralogs among protein sequences with at least 50 amino acid residues of *D. purpureum* and *D. discoideum*, with parameter settings as follows: score cutoff = 100, out-group cutoff = 50, sequence overlap cutoff = 0.8, confident cutoff = 0.05, group overlap cutoff = 0.5, grey zone = 0, and bootstrap estimation of the confidence. To reduce false positive results caused by biased amino acid usage (due to the very A+T content of the genomes), we adjusted the substitution matrix BLOSUM62 based on amino acid compositions of the two species by using Yu's method of composition-specific substitution matrix adjustment [133]. Our conservative procedure yielded 5,504 *D. purpureum* genes being orthologous counterparts of 5,613 *D. discoideum* genes, from which 5,651 (including 5,334 one-to-one orthologs) pairs of orthologs were constructed. Thus, we analyzed orthologs for fewer than half of the genes, consistent with the large distance between these two species. 232 and 447 orthologs had paralogs in *D. purpureum* and *D. discoideum*, respectively.

Global sequence alignment of the orthologous genes was implemented by using the program GAP4, which uses a dynamic alignment algorithm to improve sensitivity and recognition of homologous regions from sequences with intermittent similarity [134]. Amino acid substitution matrices BLOSUM45, BLOSUM62 and BLOSUM100 addressed by GAP4 were again adjusted based on the amino acid composition of *D. purpureum* and *D. discoideum*. The alignments of protein sequences were subsequently retro-translated into DNA sequence alignments for dN and dS calculation.

Degrees of divergence were measured by rates of non-synonymous change (dN), rates of synonymous change (dS), and conservation score (CS). The value of dN and dS were calculated by using a maximum likelihood method that was implemented by the program codeml of PAML4 [135], with parameter settings as following: runmode = -2, CodonFreq = 1, model = 0, NSsites = 0, icode = 0, Mgene = 0, ncatG = 10, and other parameters are defaults. The conservation score is calculated by dividing the GAP4 similarly score of a protein to its ortholog by the similarity score of the protein to itself [136].


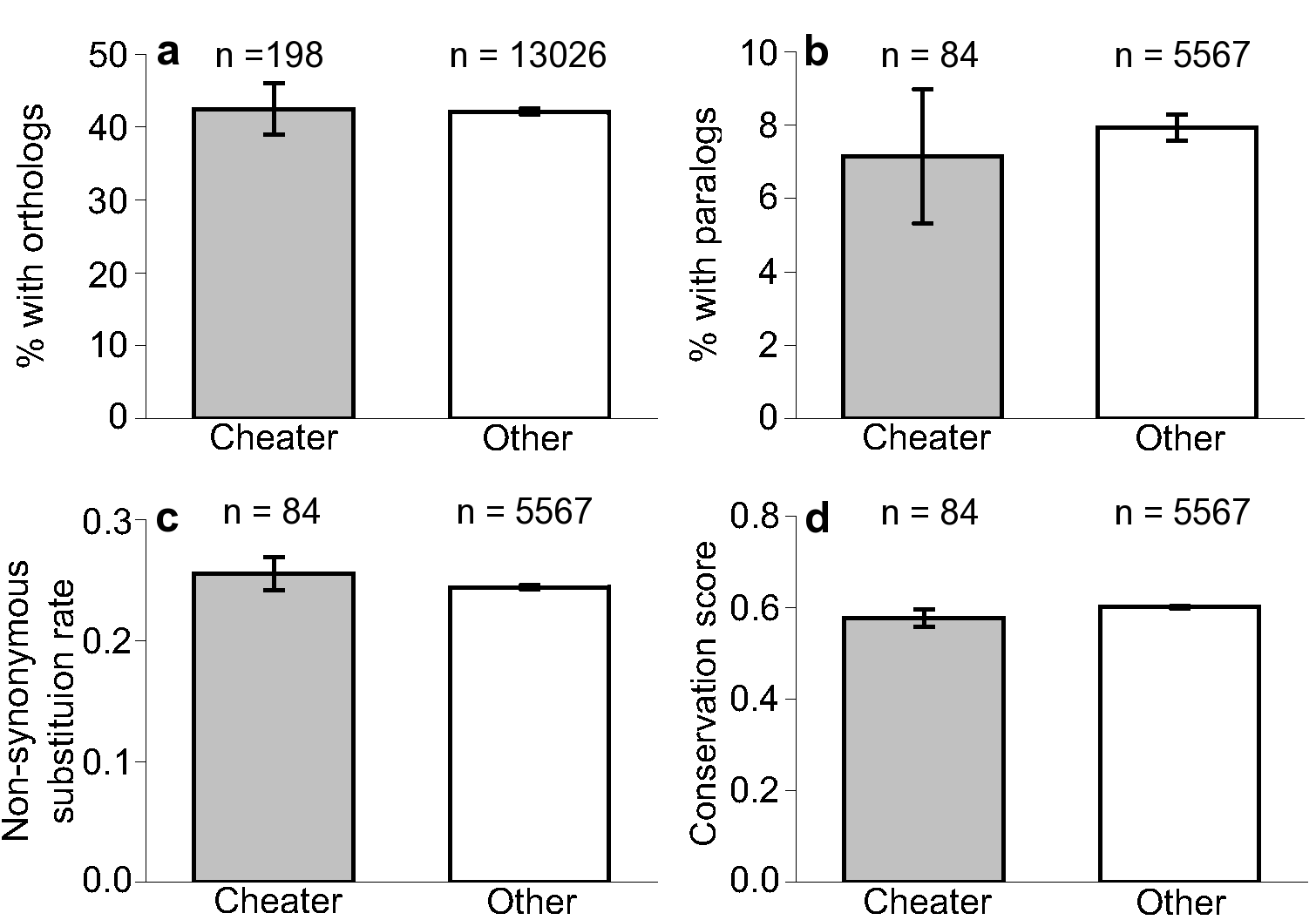


**Figure S18. Cheater genes and other genes do not differ in several measures of evolutionary rates.** A. Percentage with orthologs, |z| = 0.0885, p = 0.929. B. Percentage of orthologs that have inparalogs, |z| = 0.262, p = 0.793. C. Non-synonymous substitution rate, |t| = 1.2454, df = 85.78, p = 0.216. The ratio of non-synonymous to synonymous change (dN/dS) often provides a more sensitive measure of adaptive change, but the large distance between *D. discoideum* and *D. purpureum* means that synonymous changes have saturated and are impossible to estimate accurately. D. Conservation score, |t| = 0.8178, df = 86.64, p = 0.416. All tests are two-tailed.


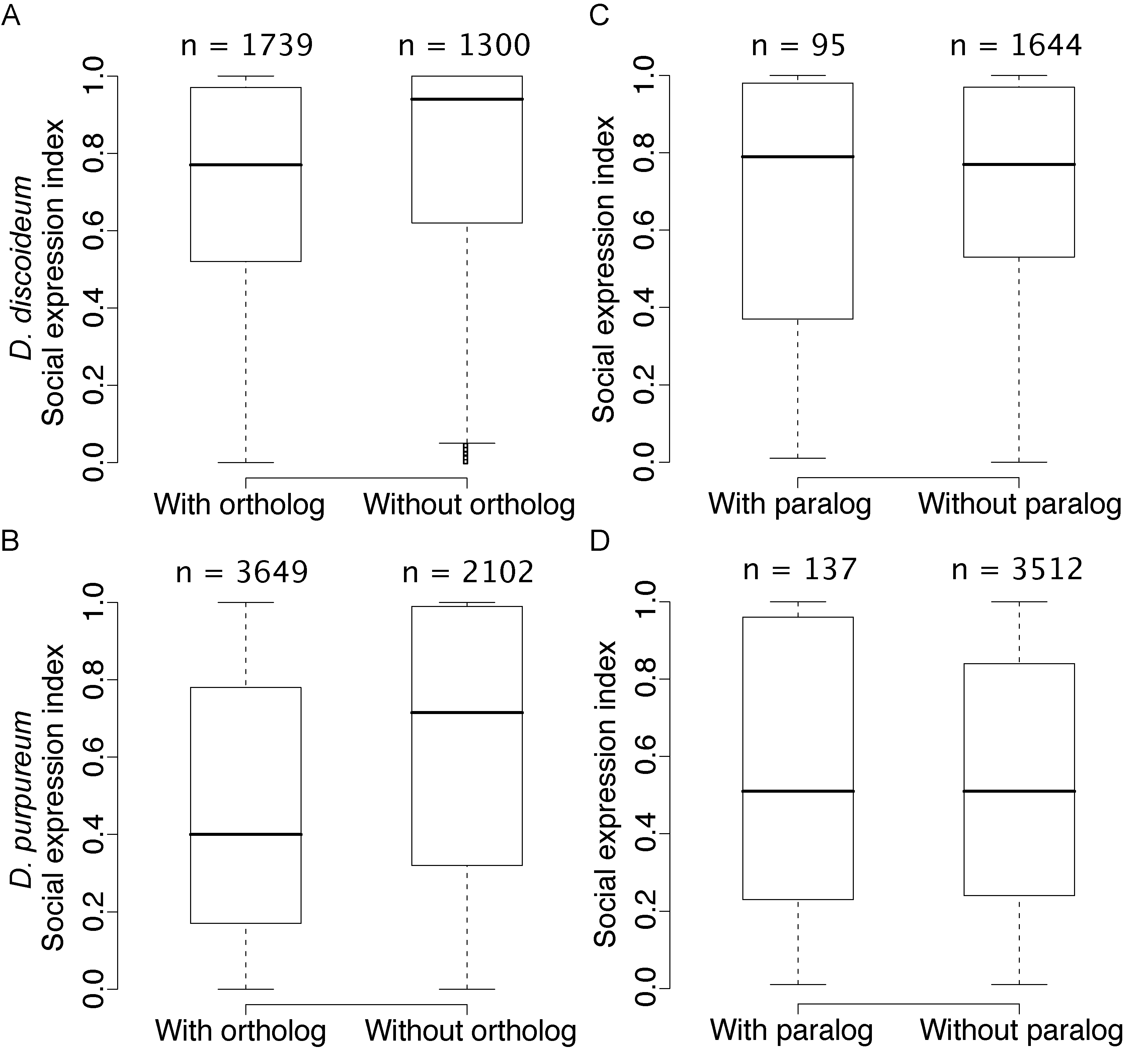


**Figure S19. Social expression index for both *D. discoideum* (A and C) and *D. purpureum* (B and D) depending on whether the genes have orthologs (A and B), or inparalogs (C and D).** Boxes show quartiles, whiskers show the most extreme data point that is no more than 1.5 times the interquartile range from the box, and circles show the outliers. Mann-Whitney U tests are used throughout. Panel A. Genes without orthologs have higher social expression in *D. discoideum* than those with orthologs, p < 0.0001. Panel B. Genes without orthologs also have higher social expression in *D. purpureum* than those with orthologs, p < 0.0001. Panel C. Genes with inparalogs have no significantly different social expression in *D. discoideum* from those without inparalogs, p = 0. 93. Panel D. Genes with inparalogs have no significantly different social expression in *D. purpureum* from those without inparalogs, p = 0.24.


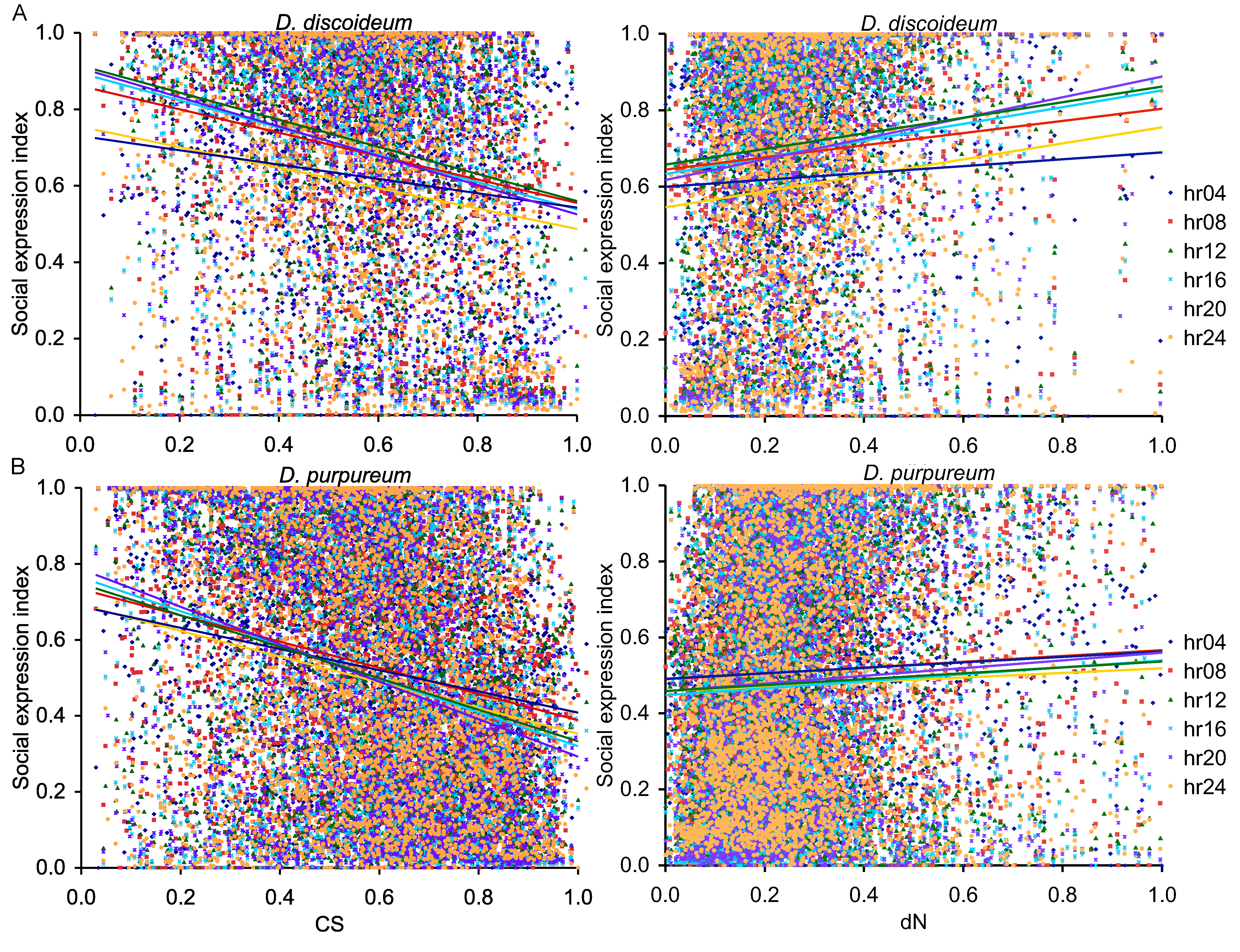


**Figure S20. Percentage of RNA-seq reads in different social stage as a function of conservation score and non-synonymous substitution rate (dN) in *D. discoideum* (A) and *D. purpureum* (B).** A separate social expression index was calculated for each social time point against the vegetative stage (hr00). Social expression index values and regression lines of stage hr04~hr24 were marked in blue, red, green, lightblue, pink and yellow, respectively. Panel A. Using *D. discoideum* RNA-seqs (1,739 genes), all the regressions on CS are significant (R^2^ = 0.036~0.061, p < 0.0001), as well as most of the regressions on dN (R^2^ = 0.0098~0.020, p < 0.0001). Panel B. Using *D. purpureum* RNA-seqs, (3,649 genes), all the regressions on CS are significant (R^2^ = 0.024~0.063, p < 0.0001), as well as all the regressions on dN (R^2^ = 0.0012~0.012, p < 0.0001).


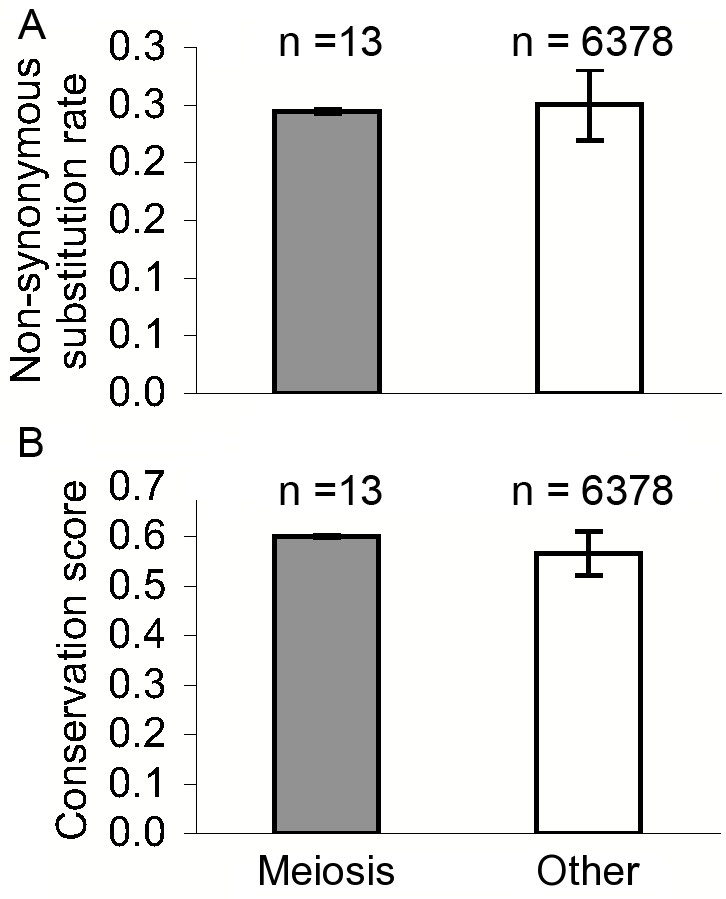


**Figure S21**. **Comparison between meiotic genes and other genes in non-synonymous substitution rate (A) and conservation score (B).** The meiotic genes are *Mlh1*, *Msh2*, *Msh6*, *Pds5*, *Pms1*, *Rad18*, *Rad23*, *Rad50*, *Rad51*, *Scc3*, *Smc2*, *Smc3*, and *Smc4*. Panel A. Non-synonymous substitution rate, |t| = 0.20, df = 12.10, p = 0.85. Panel B. Conservation score, |t| = 0.78, df = 12.08, p = 0.45. Two tailed t-test.
