# Supplementary material for: Comparative genomics of the social amoebae Dictyostelium discoideum and Dictyostelium purpureum
Source: Genome Biol. 2011 Feb 28;12(2):R20. doi: 10.1186/gb-2011-12-2-r20 (PMC3188802; doi:10.1186/gb-2011-12-2-r20)
Supplement: Additional file 2 — Supplementary Table S2. A table listing blocks of partially conserved gene order between the D. discoideum and D. purpureum genomes. [file gb-2011-12-2-r20-S2.DOC]

| block | dd_gene | dd_chr | dd_start | dd_stop | dp_gene | dp_chr | dp_start | dp_stop |
| --- | --- | --- | --- | --- | --- | --- | --- | --- |
| Block 857 | DDB_G0267478 | 1 | 202433 | 203254 | estExt_Genewise1Plus.C_210084 | scaffold_21 | 124125 | 125263 |
| Block 857 | nup93 | 1 | 203419 | 206582 | GID1.0038724 | scaffold_21 | 121263 | 124084 |
| Block 857 | DDB_G0267482 | 1 | 206837 | 207171 | e_gw1.21.100.1 | scaffold_21 | 120382 | 120710 |
| Block 2878 | acp1 | 1 | 208100 | 208842 | e_gw1.8.27.1 | scaffold_8 | 35172 | 35866 |
| Block 2878 | DDB_G0267486 | 1 | 208945 | 209681 | e_gw1.8.100.1 | scaffold_8 | 34528 | 34956 |
| Block 2878 | DDB_G0267488 | 1 | 210356 | 212554 | GID1.0037837 | scaffold_8 | 32239 | 33498 |
| Block 2883 | ChLim | 1 | 212712 | 214898 | estExt_fgeneshDP_pg.C_80063 | scaffold_8 | 152217 | 154521 |
| Block 2883 | DDB_G0267492 | 1 | 215251 | 217562 | estExt_Genewise1.C_80096 | scaffold_8 | 149611 | 151973 |
| Block 2883 | DDB_G0267494 | 1 | 217763 | 218365 | GID1.0037887 | scaffold_8 | 148467 | 149252 |
| Block 2883 | DDB_G0267496 | 1 | 218913 | 225973 | GID1.0037885 | scaffold_8 | 139391 | 145969 |
| Block 2883 | DDB_G0267498 | 1 | 226283 | 227085 | estExt_Genewise1Plus.C_80087 | scaffold_8 | 136541 | 137344 |
| Block 2883 | DDB_G0267500 | 1 | 227165 | 228343 | estExt_fgeneshDP_pg.C_80056 | scaffold_8 | 134803 | 136255 |
| Block 2883 | pakC | 1 | 229145 | 230944 | estExt_fgeneshDP_kg.C_80006 | scaffold_8 | 98394 | 100291 |
| Block 2883 | DDB_G0268264 | 1 | 231364 | 232044 | estExt_fgeneshDP_pm.C_80015 | scaffold_8 | 96612 | 97516 |
| Block 2883 | DDB_G0268266 | 1 | 233800 | 236723 | GID1.0037865 | scaffold_8 | 93143 | 95920 |
| Block 287 | DDB_G0267502 | 1 | 236992 | 238491 | GID1.0043255 | scaffold_130 | 58120 | 59597 |
| Block 287 | msh6 | 1 | 238722 | 242663 | estExt_Genewise1Plus.C_1300051 | scaffold_130 | 60753 | 63974 |
| Block 858 | DDB_G0267504 | 1 | 245416 | 247229 | estExt_Genewise1Plus.C_210091 | scaffold_21 | 139893 | 141786 |
| Block 2319 | DDB_G0267508 | 1 | 254497 | 255225 | e_gw1.53.85.1 | scaffold_53 | 28589 | 28988 |
| Block 2319 | DDB_G0268478 | 1 | 255654 | 256271 | estExt_Genewise1Plus.C_530027 | scaffold_53 | 27208 | 28104 |
| Block 2319 | DDB_G0267510 | 1 | 256535 | 257366 | GID1.0040363 | scaffold_53 | 26307 | 27042 |
| Block 2319 | DDB_G0267512 | 1 | 257711 | 258721 | e_gw1.53.18.1 | scaffold_53 | 25015 | 26022 |
| Block 2397 | dhx57 | 1 | 263217 | 267660 | GID1.0050041 | scaffold_557 | 1571 | 5938 |
| Block 2397 | ssr3 | 1 | 271374 | 272197 | gw1.557.8.1 | scaffold_557 | 6265 | 7146 |
| Block 2397 | DDB_G0267526 | 1 | 272779 | 273967 | estExt_fgeneshDP_kg.C_5570002 | scaffold_557 | 7557 | 8753 |
| Block 2397 | DDB_G0267528 | 1 | 274124 | 275255 | e_gw1.557.5.1 | scaffold_557 | 8925 | 10156 |
| Block 858 | DDB_G0268270 | 1 | 279600 | 281837 | gw1.21.45.1 | scaffold_21 | 137206 | 139098 |
| Block 858 | DDB_G0267532 | 1 | 283495 | 286152 | GID1.0038729 | scaffold_21 | 130565 | 133361 |
| Block 858 | dafE | 1 | 286362 | 287906 | e_gw1.21.53.1 | scaffold_21 | 128820 | 130319 |
| Block 858 | DDB_G0267534 | 1 | 288484 | 289302 | fgeneshDP_pg.C_scaffold_21000049 | scaffold_21 | 127266 | 127991 |
| Block 286 | hibch | 1 | 289882 | 291134 | e_gw1.130.42.1 | scaffold_130 | 56513 | 57875 |
| Block 286 | DDB_G0267538 | 1 | 291771 | 293820 | estExt_fgeneshDP_pg.C_1300023 | scaffold_130 | 53933 | 56057 |
| Block 286 | cdk10 | 1 | 294048 | 295439 | gw1.130.41.1 | scaffold_130 | 51842 | 53571 |
| Block 286 | dicB | 1 | 298527 | 300350 | GID1.0043251 | scaffold_130 | 47872 | 49721 |
| Block 286 | scy1 | 1 | 300608 | 303232 | estExt_Genewise1Plus.C_1300038 | scaffold_130 | 44990 | 47673 |
| Block 286 | DDB_G0267546 | 1 | 311255 | 312670 | GID1.0043249 | scaffold_130 | 43033 | 44140 |
| Block 2378 | DDB_G0267548 | 1 | 313115 | 316757 | fgeneshDP_pg.C_scaffold_55000005 | scaffold_55 | 10118 | 13387 |
| Block 2378 | DDB_G0268278 | 1 | 322112 | 322810 | estExt_fgeneshDP_pg.C_550006 | scaffold_55 | 15253 | 16072 |
| Block 1946 | DDB_G0267552 | 1 | 324845 | 325596 | e_gw1.427.16.1 | scaffold_427 | 8809 | 9274 |
| Block 1946 | DDB_G0267554 | 1 | 325873 | 326929 | fgeneshDP_pm.C_scaffold_427000002 | scaffold_427 | 9472 | 10393 |
| Block 1946 | DDB_G0267556 | 1 | 327145 | 330624 | GID1.0048942 | scaffold_427 | 10583 | 13785 |
| Block 286 | gacO | 1 | 355159 | 357213 | gw1.130.13.1 | scaffold_130 | 67295 | 67855 |
| Block 1307 | DDB_G0267572 | 1 | 361555 | 362932 | GID1.0046936 | scaffold_289 | 38355 | 39625 |
| Block 2378 | DDB_G0268286 | 1 | 363410 | 364574 | estExt_fgeneshDP_kg.C_550001 | scaffold_55 | 8504 | 9854 |
| Block 2378 | cybA | 1 | 364761 | 365278 | e_gw1.55.57.1 | scaffold_55 | 7926 | 8419 |
| Block 1307 | DDB_G0267574 | 1 | 365942 | 366747 | fgeneshDP_pm.C_scaffold_289000007 | scaffold_289 | 29256 | 30062 |
| Block 1307 | DDB_G0267576 | 1 | 366936 | 367540 | fgeneshDP_pm.C_scaffold_289000008 | scaffold_289 | 30220 | 30725 |
| Block 1307 | DDB_G0267578 | 1 | 368155 | 368942 | fgeneshDP_pg.C_scaffold_289000014 | scaffold_289 | 31256 | 31969 |
| Block 1307 | DDB_G0267582 | 1 | 371018 | 373018 | estExt_Genewise1Plus.C_2890030 | scaffold_289 | 32533 | 34546 |
| Block 1307 | adcE | 1 | 373253 | 374947 | fgeneshDP_pg.C_scaffold_289000016 | scaffold_289 | 34664 | 36358 |
| Block 1307 | DDB_G0267586 | 1 | 375348 | 376736 | GID1.0046935 | scaffold_289 | 36899 | 38260 |
| Block 424 | DDB_G0267588 | 1 | 376992 | 379294 | estExt_Genewise1Plus.C_1490016 | scaffold_149 | 38853 | 41311 |
| Block 424 | DDB_G0267590 | 1 | 381029 | 382632 | fgeneshDP_pg.C_scaffold_149000021 | scaffold_149 | 65398 | 67097 |
| Block 286 | DDB_G0267592 | 1 | 383616 | 385211 | fgeneshDP_pg.C_scaffold_130000018 | scaffold_130 | 40934 | 42481 |
| Block 286 | pfdn2 | 1 | 385503 | 385941 | e_gw1.130.15.1 | scaffold_130 | 38452 | 38977 |
| Block 286 | DDB_G0268288 | 1 | 386296 | 390186 | fgeneshDP_pg.C_scaffold_130000015 | scaffold_130 | 34687 | 38360 |
| Block 286 | DDB_G0267598 | 1 | 395715 | 397024 | fgeneshDP_pm.C_scaffold_130000007 | scaffold_130 | 33252 | 34375 |
| Block 1945 | DDB_G0267602 | 1 | 400350 | 405975 | GID1.0048939 | scaffold_427 | 3065 | 8477 |
| Block 1945 | mserS | 1 | 406648 | 408225 | gw1.427.14.1 | scaffold_427 | 916 | 2331 |
| Block 2378 | fcf1 | 1 | 408722 | 409582 | fgeneshDP_pm.C_scaffold_55000011 | scaffold_55 | 67538 | 68609 |
| Block 2378 | DDB_G0267620 | 1 | 436026 | 437354 | fgeneshDP_pg.C_scaffold_55000018 | scaffold_55 | 63023 | 64315 |
| Block 2378 | srp9 | 1 | 438237 | 438607 | e_gw1.55.60.1 | scaffold_55 | 61890 | 62201 |
| Block 2378 | kil1 | 1 | 443514 | 444929 | fgeneshDP_pg.C_scaffold_55000017 | scaffold_55 | 60102 | 61543 |
| Block 2874 | gtaE | 1 | 476398 | 479414 | estExt_fgeneshDP_pg.C_80007 | scaffold_8 | 18103 | 20084 |
| Block 2874 | DDB_G0267448 | 1 | 481685 | 483244 | GID1.0037827 | scaffold_8 | 15479 | 16647 |
| Block 2874 | DDB_G0267642 | 1 | 484012 | 485684 | GID1.0037826 | scaffold_8 | 13644 | 15068 |
| Block 2874 | rpl38 | 1 | 485945 | 486391 | GID1.0037825 | scaffold_8 | 12931 | 13411 |
| Block 2229 | DDB_G0267644 | 1 | 488923 | 490112 | GID1.0049650 | scaffold_500 | 9797 | 11035 |
| Block 2229 | sec20 | 1 | 490625 | 491661 | GID1.0049649 | scaffold_500 | 8433 | 9362 |
| Block 1306 | DDB_G0267648 | 1 | 491895 | 494666 | GID1.0046929 | scaffold_289 | 26520 | 28994 |
| Block 2229 | ireA | 1 | 495110 | 498317 | estExt_Genewise1Plus.C_5000001 | scaffold_500 | 5285 | 8321 |
| Block 288 | DDB_G0267656 | 1 | 518710 | 520050 | e_gw1.130.46.1 | scaffold_130 | 70932 | 72218 |
| Block 288 | DDB_G0268492 | 1 | 520101 | 522397 | GID1.0043260 | scaffold_130 | 72284 | 74245 |
| Block 2394 | DDB_G0267658 | 1 | 523164 | 526205 | gw1.555.2.1 | scaffold_555 | 9736 | 12637 |
| Block 2394 | DDB_G0267660 | 1 | 526607 | 527617 | fgeneshDP_pg.C_scaffold_555000005 | scaffold_555 | 8810 | 9595 |
| Block 2394 | DDB_G0267662 | 1 | 527773 | 528777 | fgeneshDP_pg.C_scaffold_555000004 | scaffold_555 | 7700 | 8539 |
| Block 2394 | DDB_G0267664 | 1 | 529188 | 530978 | fgeneshDP_pm.C_scaffold_555000002 | scaffold_555 | 5621 | 7257 |
| Block 1306 | gefW | 1 | 533108 | 536707 | GID1.0046928 | scaffold_289 | 22369 | 25811 |
| Block 850 | hspL | 1 | 537016 | 537560 | fgeneshDP_pg.C_scaffold_21000025 | scaffold_21 | 64558 | 64977 |
| Block 850 | DDB_G0267670 | 1 | 537622 | 538492 | fgeneshDP_pg.C_scaffold_21000024 | scaffold_21 | 63764 | 64468 |
| Block 1148 | culB | 1 | 551012 | 554227 | estExt_Genewise1Plus.C_2570019 | scaffold_257 | 31886 | 34912 |
| Block 1148 | DDB_G0268496 | 1 | 554652 | 556085 | e_gw1.257.22.1 | scaffold_257 | 35301 | 36698 |
| Block 1148 | DDB_G0267680 | 1 | 557566 | 558286 | fgeneshDP_pm.C_scaffold_257000009 | scaffold_257 | 37063 | 37639 |
| Block 1148 | rpb9 | 1 | 558538 | 559011 | fgeneshDP_pg.C_scaffold_257000016 | scaffold_257 | 38029 | 38607 |
| Block 1148 | ercc1 | 1 | 559492 | 561036 | gw1.257.28.1 | scaffold_257 | 39493 | 40122 |
| Block 850 | DDB_G0267686 | 1 | 564277 | 571070 | fgeneshDP_pg.C_scaffold_21000020 | scaffold_21 | 50484 | 55757 |
| Block 850 | DDB_G0268498 | 1 | 573341 | 576172 | fgeneshDP_pg.C_scaffold_21000019 | scaffold_21 | 46322 | 48972 |
| Block 850 | DDB_G0267698 | 1 | 623530 | 624219 | GID1.0038698 | scaffold_21 | 71570 | 72184 |
| Block 979 | roco7 | 1 | 628403 | 636330 | fgeneshDP_pg.C_scaffold_229000012 | scaffold_229 | 30199 | 37507 |
| Block 59 | DDB_G0268320 | 1 | 637945 | 640745 | estExt_fgeneshDP_pm.C_1040006 | scaffold_104 | 19269 | 22248 |
| Block 979 | mcfT | 1 | 661668 | 662715 | estExt_fgeneshDP_pg.C_2290014 | scaffold_229 | 47756 | 49088 |
| Block 59 | DDB_G0267706 | 1 | 670002 | 670854 | gw1.104.22.1 | scaffold_104 | 368 | 952 |
| Block 59 | vps25 | 1 | 670930 | 671606 | e_gw1.104.20.1 | scaffold_104 | 1160 | 1791 |
| Block 59 | DDB_G0267710 | 1 | 673242 | 674981 | estExt_Genewise1.C_1040005 | scaffold_104 | 3632 | 5145 |
| Block 59 | DDB_G0267712 | 1 | 675419 | 675964 | fgeneshDP_pm.C_scaffold_104000007 | scaffold_104 | 22332 | 22854 |
| Block 59 | sf3b4 | 1 | 676187 | 677352 | GID1.0042396 | scaffold_104 | 23132 | 24001 |
| Block 494 | DDB_G0267718 | 1 | 695658 | 697436 | estExt_fgeneshDP_kg.C_1570005 | scaffold_157 | 24469 | 25154 |
| Block 494 | DDB_G0267722 | 1 | 699431 | 700187 | estExt_fgeneshDP_pg.C_1570012 | scaffold_157 | 23619 | 24445 |
| Block 850 | DDB_G0268334 | 1 | 714031 | 717583 | estExt_fgeneshDP_kg.C_210009 | scaffold_21 | 80274 | 81165 |
| Block 850 | dst2 | 1 | 719427 | 722987 | estExt_fgeneshDP_pm.C_210018 | scaffold_21 | 72212 | 77482 |
| Block 850 | DDB_G0267732 | 1 | 723287 | 729518 | GID1.0038706 | scaffold_21 | 86414 | 92099 |
| Block 922 | DDB_G0267746 | 1 | 774330 | 775937 | estExt_fgeneshDP_kg.C_220016 | scaffold_22 | 102211 | 104286 |
| Block 922 | cdc45 | 1 | 776324 | 778499 | e_gw1.22.50.1 | scaffold_22 | 100163 | 102154 |
| Block 1472 | DDB_G0267750 | 1 | 780178 | 783237 | fgeneshDP_pg.C_scaffold_319000008 | scaffold_319 | 19740 | 22750 |
| Block 1290 | DDB_G0267752 | 1 | 783552 | 786233 | fgeneshDP_pg.C_scaffold_285000009 | scaffold_285 | 27842 | 30399 |
| Block 1472 | tbck | 1 | 792299 | 795646 | fgeneshDP_pg.C_scaffold_319000011 | scaffold_319 | 32780 | 36061 |
| Block 1290 | tbcD | 1 | 802089 | 806703 | GID1.0046861 | scaffold_285 | 37560 | 42152 |
| Block 1290 | gfm2 | 1 | 806804 | 809101 | e_gw1.285.5.1 | scaffold_285 | 35150 | 37475 |
| Block 2777 | DDB_G0267768 | 1 | 809267 | 810455 | e_gw1.729.4.1 | scaffold_729 | 2474 | 3664 |
| Block 2777 | DDB_G0268518 | 1 | 810583 | 811472 | GID1.0050862 | scaffold_729 | 3994 | 4901 |
| Block 2025 | DDB_G0268520 | 1 | 811678 | 815598 | estExt_fgeneshDP_pm.C_4480004 | scaffold_448 | 17759 | 21404 |
| Block 2025 | DDB_G0268522 | 1 | 816236 | 816791 | GID1.0049146 | scaffold_448 | 17047 | 17549 |
| Block 2025 | cnbA | 1 | 816934 | 817967 | estExt_fgeneshDP_pg.C_4480005 | scaffold_448 | 15830 | 16951 |
| Block 1665 | DDB_G0267772 | 1 | 824718 | 825057 | GID1.0048061 | scaffold_360 | 6522 | 6796 |
| Block 1665 | abkC | 1 | 825289 | 827478 | fgeneshDP_pg.C_scaffold_360000002 | scaffold_360 | 6998 | 9062 |
| Block 1665 | DDB_G0267776 | 1 | 827764 | 832445 | gw1.360.1.1 | scaffold_360 | 9635 | 13563 |
| Block 1665 | repD | 1 | 833130 | 835460 | estExt_Genewise1.C_3600006 | scaffold_360 | 14587 | 17370 |
| Block 1665 | DDB_G0267786 | 1 | 852021 | 853903 | e_gw1.360.7.1 | scaffold_360 | 20412 | 26948 |
| Block 584 | DDB_G0267792 | 1 | 858290 | 859778 | estExt_fgeneshDP_pg.C_1710007 | scaffold_171 | 12649 | 14607 |
| Block 584 | DDB_G0267794 | 1 | 860671 | 862593 | estExt_fgeneshDP_pg.C_1710006 | scaffold_171 | 9598 | 11566 |
| Block 584 | DDB_G0267796 | 1 | 863148 | 865925 | estExt_Genewise1.C_1710007 | scaffold_171 | 6582 | 9112 |
| Block 584 | DDB_G0268346 | 1 | 866636 | 867904 | GID1.0044403 | scaffold_171 | 4727 | 5945 |
| Block 1717 | gxcGG | 1 | 891838 | 894228 | estExt_fgeneshDP_kg.C_3730004 | scaffold_373 | 10281 | 14041 |
| Block 1717 | DDB_G0267804 | 1 | 899153 | 902230 | fgeneshDP_pg.C_scaffold_373000007 | scaffold_373 | 23267 | 26392 |
| Block 2574 | DDB_G0267820 | 1 | 919584 | 920681 | GID1.0050423 | scaffold_621 | 3653 | 5236 |
| Block 2574 | DDB_G0267822 | 1 | 920878 | 922078 | gw1.621.9.1 | scaffold_621 | 5757 | 6346 |
| Block 2574 | DDB_G0267824 | 1 | 922136 | 923726 | e_gw1.621.7.1 | scaffold_621 | 6845 | 8211 |
| Block 2574 | gemA | 1 | 929880 | 932237 | estExt_fgeneshDP_pg.C_6210001 | scaffold_621 | 713 | 3578 |
| Block 238 | DDB_G0268358 | 1 | 936052 | 938060 | e_gw1.125.19.1 | scaffold_125 | 334 | 948 |
| Block 238 | DDB_G0267834 | 1 | 939089 | 940508 | estExt_fgeneshDP_pg.C_1250002 | scaffold_125 | 2495 | 4352 |
| Block 1218 | corA | 1 | 943374 | 944711 | estExt_fgeneshDP_pg.C_2720014 | scaffold_272 | 28850 | 30809 |
| Block 1218 | DDB_G0268360 | 1 | 948334 | 948958 | fgeneshDP_pm.C_scaffold_272000007 | scaffold_272 | 27138 | 27416 |
| Block 1218 | DDB_G0267838 | 1 | 949001 | 949753 | estExt_fgeneshDP_kg.C_2720002 | scaffold_272 | 27774 | 28639 |
| Block 1218 | rpmA | 1 | 950030 | 952882 | estExt_fgeneshDP_pm.C_2720006 | scaffold_272 | 24087 | 26754 |
| Block 1218 | DDB_G0267840 | 1 | 954390 | 958768 | estExt_fgeneshDP_pg.C_2720011 | scaffold_272 | 18554 | 22172 |
| Block 1218 | DDB_G0267848 | 1 | 964384 | 965467 | GID1.0046606 | scaffold_272 | 11673 | 12768 |
| Block 1218 | DDB_G0267852 | 1 | 968117 | 968896 | e_gw1.272.29.1 | scaffold_272 | 8812 | 9539 |
| Block 492 | DDB_G0267866 | 1 | 991453 | 992043 | estExt_fgeneshDP_kg.C_1570003 | scaffold_157 | 16222 | 17017 |
| Block 492 | DDB_G0267868 | 1 | 992499 | 994469 | estExt_Genewise1.C_1570008 | scaffold_157 | 13971 | 16047 |
| Block 902 | naprt | 1 | 1010098 | 1012044 | GID1.0045485 | scaffold_217 | 14715 | 18792 |
| Block 902 | DDB_G0267872 | 1 | 1012281 | 1014423 | gw1.217.2.1 | scaffold_217 | 12199 | 13827 |
| Block 902 | DDB_G0268534 | 1 | 1014659 | 1015216 | fgeneshDP_pg.C_scaffold_217000006 | scaffold_217 | 11317 | 11880 |
| Block 902 | DDB_G0268536 | 1 | 1020423 | 1022453 | e_gw1.217.14.1 | scaffold_217 | 3957 | 6002 |
| Block 902 | psmA5 | 1 | 1022895 | 1023978 | estExt_Genewise1.C_2170002 | scaffold_217 | 2350 | 3652 |
| Block 586 | alg12 | 1 | 1032061 | 1034001 | fgeneshDP_pg.C_scaffold_171000011 | scaffold_171 | 30552 | 32156 |
| Block 586 | amtC | 1 | 1034624 | 1036114 | estExt_Genewise1.C_1710016 | scaffold_171 | 28157 | 30051 |
| Block 2350 | DDB_G0267890 | 1 | 1042896 | 1044435 | fgeneshDP_pg.C_scaffold_539000003 | scaffold_539 | 6343 | 7934 |
| Block 2350 | DDB_G0267892 | 1 | 1045342 | 1047918 | fgeneshDP_pg.C_scaffold_539000001 | scaffold_539 | 981 | 3227 |
| Block 492 | DDB_G0267898 | 1 | 1053842 | 1055149 | e_gw1.157.31.1 | scaffold_157 | 60884 | 62179 |
| Block 1123 | DDB_G0267904 | 1 | 1077141 | 1080667 | estExt_fgeneshDP_pg.C_2530008 | scaffold_253 | 16838 | 19923 |
| Block 1123 | DDB_G0267906 | 1 | 1080926 | 1082242 | fgeneshDP_pg.C_scaffold_253000005 | scaffold_253 | 10959 | 12218 |
| Block 1959 | DDB_G0267908 | 1 | 1083269 | 1083568 | e_gw1.43.43.1 | scaffold_43 | 76528 | 76818 |
| Block 1959 | sigK | 1 | 1084166 | 1085446 | estExt_Genewise1.C_430019 | scaffold_43 | 74594 | 75891 |
| Block 1959 | DDB_G0268384 | 1 | 1085918 | 1089019 | fgeneshDP_pm.C_scaffold_43000004 | scaffold_43 | 68864 | 71831 |
| Block 492 | ugt2 | 1 | 1089190 | 1090568 | e_gw1.157.30.1 | scaffold_157 | 4208 | 7574 |
| Block 1168 | pikF | 1 | 1099622 | 1104015 | e_gw1.260.6.1 | scaffold_260 | 22708 | 26184 |
| Block 988 | mtmr15 | 1 | 1114621 | 1117968 | GID1.0038828 | scaffold_23 | 86710 | 90160 |
| Block 988 | DDB_G0267918 | 1 | 1118591 | 1121104 | GID1.0038829 | scaffold_23 | 90675 | 92966 |
| Block 60 | DDB_G0267924 | 1 | 1128799 | 1132710 | estExt_Genewise1.C_1040010 | scaffold_104 | 12454 | 16954 |
| Block 1490 | DDB_G0268394 | 1 | 1135912 | 1139439 | estExt_fgeneshDP_pg.C_3230004 | scaffold_323 | 8618 | 11512 |
| Block 1168 | DDB_G0267930 | 1 | 1143814 | 1144065 | GID1.0046388 | scaffold_260 | 18318 | 18515 |
| Block 60 | fnkB | 1 | 1147906 | 1149691 | estExt_Genewise1.C_1040007 | scaffold_104 | 5165 | 6898 |
| Block 60 | rab24 | 1 | 1157427 | 1158481 | fgeneshDP_pg.C_scaffold_104000009 | scaffold_104 | 17156 | 18003 |
| Block 1304 | DDB_G0267940 | 1 | 1159902 | 1161696 | fgeneshDP_pg.C_scaffold_289000004 | scaffold_289 | 8926 | 10663 |
| Block 1304 | DDB_G0267942 | 1 | 1161933 | 1165193 | estExt_fgeneshDP_pg.C_2890003 | scaffold_289 | 6149 | 8887 |
| Block 2877 | DDB_G0267948 | 1 | 1170991 | 1172151 | fgeneshDP_pg.C_scaffold_8000014 | scaffold_8 | 31203 | 32080 |
| Block 2877 | copE | 1 | 1172814 | 1173875 | fgeneshDP_pg.C_scaffold_8000013 | scaffold_8 | 29635 | 30692 |
| Block 285 | DDB_G0267952 | 1 | 1173990 | 1175066 | e_gw1.130.50.1 | scaffold_130 | 30694 | 31670 |
| Block 285 | DDB_G0267954 | 1 | 1175758 | 1177189 | GID1.0043242 | scaffold_130 | 28856 | 30002 |
| Block 285 | abiA | 1 | 1177308 | 1178404 | fgeneshDP_pm.C_scaffold_130000006 | scaffold_130 | 27649 | 28705 |
| Block 285 | DDB_G0268552 | 1 | 1182084 | 1184169 | fgeneshDP_pg.C_scaffold_130000009 | scaffold_130 | 23449 | 25283 |
| Block 2380 | DDB_G0268404 | 1 | 1184694 | 1187746 | GID1.0040461 | scaffold_55 | 52465 | 54932 |
| Block 2380 | cog4 | 1 | 1187972 | 1190807 | GID1.0040458 | scaffold_55 | 41032 | 43833 |
| Block 2380 | tsuA | 1 | 1191800 | 1198906 | estExt_fgeneshDP_pm.C_550006 | scaffold_55 | 44588 | 51053 |
| Block 2380 | pyd1 | 1 | 1202711 | 1205883 | estExt_fgeneshDP_pm.C_550005 | scaffold_55 | 36833 | 40276 |
| Block 1304 | DDB_G0267974 | 1 | 1214003 | 1217095 | GID1.0046926 | scaffold_289 | 17285 | 20105 |
| Block 1304 | aph1 | 1 | 1217963 | 1219191 | e_gw1.289.17.1 | scaffold_289 | 20642 | 21821 |
| Block 432 | krsB | 1 | 1220851 | 1224573 | estExt_fgeneshDP_pm.C_150020 | scaffold_15 | 91491 | 95674 |
| Block 432 | mocs3 | 1 | 1230144 | 1231512 | GID1.0038337 | scaffold_15 | 97240 | 98561 |
| Block 2380 | copA | 1 | 1232234 | 1235989 | e_gw1.55.2.1 | scaffold_55 | 55752 | 59516 |
| Block 2885 | DDB_G0267992 | 1 | 1244589 | 1245377 | GID1.0037871 | scaffold_8 | 110321 | 111199 |
| Block 2885 | DDB_G0267994 | 1 | 1246116 | 1247617 | GID1.0037872 | scaffold_8 | 111942 | 113346 |
| Block 2885 | DDB_G0267996 | 1 | 1248279 | 1249587 | e_gw1.8.74.1 | scaffold_8 | 113945 | 115237 |
| Block 2885 | lmpA | 1 | 1250161 | 1252599 | GID1.0037874 | scaffold_8 | 116630 | 119152 |
| Block 2885 | lmpC | 1 | 1257306 | 1259874 | GID1.0037877 | scaffold_8 | 124402 | 126935 |
| Block 2885 | DDB_G0267434 | 1 | 1260113 | 1262650 | fgeneshDP_pg.C_scaffold_8000054 | scaffold_8 | 127389 | 130208 |
| Block 2885 | abcF4 | 1 | 1263019 | 1266447 | estExt_Genewise1Plus.C_80085 | scaffold_8 | 131930 | 134013 |
| Block 2380 | snrp70 | 1 | 1286207 | 1287863 | fgeneshDP_pg.C_scaffold_55000022 | scaffold_55 | 69324 | 70741 |
| Block 1167 | DDB_G0268008 | 1 | 1290599 | 1291894 | fgeneshDP_pg.C_scaffold_260000006 | scaffold_260 | 14297 | 15784 |
| Block 1167 | DDB_G0268558 | 1 | 1292814 | 1294391 | GID1.0046387 | scaffold_260 | 16455 | 17950 |
| Block 1627 | DDB_G0268010 | 1 | 1301596 | 1302485 | estExt_fgeneshDP_pm.C_3530001 | scaffold_353 | 749 | 1703 |
| Block 1627 | DDB_G0268012 | 1 | 1302705 | 1303853 | fgeneshDP_pm.C_scaffold_353000002 | scaffold_353 | 1775 | 2812 |
| Block 1627 | gtf2h1 | 1 | 1308296 | 1310389 | fgeneshDP_pm.C_scaffold_353000003 | scaffold_353 | 7288 | 9327 |
| Block 1627 | phg1a | 1 | 1310839 | 1313118 | estExt_fgeneshDP_kg.C_3530002 | scaffold_353 | 9662 | 12114 |
| Block 1627 | DDB_G0268016 | 1 | 1313476 | 1313789 | e_gw1.353.30.1 | scaffold_353 | 12278 | 12577 |
| Block 1627 | DDB_G0268018 | 1 | 1314808 | 1316513 | fgeneshDP_pg.C_scaffold_353000005 | scaffold_353 | 13150 | 14729 |
| Block 1627 | bkdB | 1 | 1317002 | 1318564 | GID1.0047960 | scaffold_353 | 15439 | 16931 |
| Block 1627 | DDB_G0268022 | 1 | 1318763 | 1319387 | GID1.0047961 | scaffold_353 | 17096 | 17686 |
| Block 1627 | DDB_G0268024 | 1 | 1320084 | 1322172 | e_gw1.353.14.1 | scaffold_353 | 17983 | 19708 |
| Block 1167 | DDB_G0268028 | 1 | 1325279 | 1327600 | estExt_Genewise1.C_2600002 | scaffold_260 | 5014 | 8101 |
| Block 1288 | cysB | 1 | 1329298 | 1331157 | estExt_Genewise1.C_2850005 | scaffold_285 | 20418 | 22468 |
| Block 1288 | DDB_G0268030 | 1 | 1334449 | 1338114 | GID1.0046854 | scaffold_285 | 22630 | 25323 |
| Block 2698 | DDB_G0268422 | 1 | 1339034 | 1339601 | fgeneshDP_pm.C_scaffold_68000005 | scaffold_68 | 35284 | 35832 |
| Block 2698 | DDB_G0268032 | 1 | 1339641 | 1339901 | e_gw1.68.70.1 | scaffold_68 | 35962 | 36299 |
| Block 2698 | DDB_G0268034 | 1 | 1340557 | 1342081 | estExt_Genewise1Plus.C_680026 | scaffold_68 | 46275 | 47963 |
| Block 2698 | DDB_G0268036 | 1 | 1342458 | 1343261 | e_gw1.68.17.1 | scaffold_68 | 48252 | 49064 |
| Block 2698 | DDB_G0268038 | 1 | 1344276 | 1344767 | estExt_Genewise1Plus.C_680032 | scaffold_68 | 49531 | 50212 |
| Block 329 | DDB_G0268040 | 1 | 1345125 | 1346305 | GID1.0043431 | scaffold_136 | 54162 | 55271 |
| Block 329 | DDB_G0268042 | 1 | 1346481 | 1347356 | estExt_Genewise1Plus.C_1360030 | scaffold_136 | 48823 | 49589 |
| Block 329 | DDB_G0268044 | 1 | 1347890 | 1350468 | estExt_fgeneshDP_kg.C_1360003 | scaffold_136 | 44527 | 48593 |
| Block 329 | DDB_G0268048 | 1 | 1353351 | 1354182 | fgeneshDP_pg.C_scaffold_136000018 | scaffold_136 | 43292 | 43894 |
| Block 1302 | DDB_G0268054 | 1 | 1365318 | 1365947 | GID1.0046919 | scaffold_289 | 1098 | 1546 |
| Block 1302 | sptA | 1 | 1366267 | 1368004 | GID1.0046923 | scaffold_289 | 10752 | 12323 |
| Block 1302 | DDB_G0268424 | 1 | 1368763 | 1370695 | GID1.0046924 | scaffold_289 | 12831 | 14659 |
| Block 431 | zntA | 1 | 1375259 | 1376890 | fgeneshDP_pg.C_scaffold_15000030 | scaffold_15 | 88512 | 89936 |
| Block 431 | DDB_G0268564 | 1 | 1377494 | 1378332 | e_gw1.15.85.1 | scaffold_15 | 76829 | 77835 |
| Block 431 | DDB_G0268566 | 1 | 1378934 | 1380740 | GID1.0038326 | scaffold_15 | 75164 | 76516 |
| Block 431 | DDB_G0268060 | 1 | 1380972 | 1383578 | estExt_Genewise1Plus.C_150034 | scaffold_15 | 39118 | 41781 |
| Block 431 | aqpA | 1 | 1385036 | 1385981 | estExt_fgeneshDP_kg.C_150004 | scaffold_15 | 42352 | 43451 |
| Block 860 | DDB_G0268062 | 1 | 1391482 | 1393242 | fgeneshDP_pm.C_scaffold_21000026 | scaffold_21 | 143684 | 145324 |
| Block 860 | DDB_G0268064 | 1 | 1393387 | 1394274 | estExt_Genewise1Plus.C_210096 | scaffold_21 | 142702 | 143608 |
| Block 984 | rab6 | 1 | 1398472 | 1399280 | estExt_Genewise1Plus.C_230043 | scaffold_23 | 84236 | 85352 |
| Block 984 | DDB_G0268070 | 1 | 1399546 | 1400979 | fgeneshDP_pm.C_scaffold_23000011 | scaffold_23 | 80633 | 81952 |
| Block 431 | DDB_G0268074 | 1 | 1408319 | 1411401 | fgeneshDP_pg.C_scaffold_15000019 | scaffold_15 | 60001 | 62981 |
| Block 431 | DDB_G0268076 | 1 | 1414125 | 1415255 | estExt_Genewise1.C_150045 | scaffold_15 | 52519 | 58370 |
| Block 984 | rpc3 | 1 | 1420168 | 1422262 | estExt_Genewise1.C_230037 | scaffold_23 | 75945 | 77990 |
| Block 2864 | lvsG | 1 | 1432947 | 1439629 | estExt_fgeneshDP_pm.C_790008 | scaffold_79 | 43981 | 50385 |
| Block 2864 | abcH2 | 1 | 1443157 | 1444962 | GID1.0041478 | scaffold_79 | 41045 | 42380 |
| Block 2864 | DDB_G0268094 | 1 | 1446577 | 1447961 | fgeneshDP_pg.C_scaffold_79000027 | scaffold_79 | 69307 | 70561 |
| Block 2864 | DDB_G0268098 | 1 | 1450677 | 1452718 | e_gw1.79.3.1 | scaffold_79 | 74301 | 76262 |
| Block 2864 | asnS2 | 1 | 1453036 | 1454418 | GID1.0041493 | scaffold_79 | 76589 | 78048 |
| Block 2864 | DDB_G0268102 | 1 | 1455102 | 1460921 | GID1.0041494 | scaffold_79 | 78869 | 83551 |
| Block 2864 | gabT | 1 | 1461475 | 1463206 | estExt_Genewise1.C_790056 | scaffold_79 | 83913 | 85846 |
| Block 1982 | DDB_G0268110 | 1 | 1466318 | 1468006 | GID1.0049058 | scaffold_438 | 8000 | 9736 |
| Block 1982 | DDB_G0268112 | 1 | 1468365 | 1468964 | fgeneshDP_pm.C_scaffold_438000004 | scaffold_438 | 10180 | 10764 |
| Block 1982 | polA | 1 | 1469084 | 1473264 | GID1.0049060 | scaffold_438 | 11019 | 15094 |
| Block 1982 | DDB_G0268114 | 1 | 1473867 | 1474392 | fgeneshDP_pg.C_scaffold_438000008 | scaffold_438 | 15413 | 16053 |
| Block 1170 | DDB_G0268116 | 1 | 1479679 | 1480888 | e_gw1.260.17.1 | scaffold_260 | 31046 | 32224 |
| Block 1170 | DDB_G0268118 | 1 | 1481369 | 1483750 | GID1.0046392 | scaffold_260 | 28395 | 30746 |
| Block 1170 | DDB_G0268126 | 1 | 1489512 | 1491326 | e_gw1.260.13.1 | scaffold_260 | 26574 | 28256 |
| Block 853 | DDB_G0268130 | 1 | 1512409 | 1514206 | fgeneshDP_pg.C_scaffold_21000037 | scaffold_21 | 93589 | 95320 |
| Block 853 | DDB_G0268132 | 1 | 1516724 | 1519586 | e_gw1.21.18.1 | scaffold_21 | 97377 | 97832 |
| Block 853 | H3a | 1 | 1520754 | 1521173 | estExt_Genewise1.C_210076 | scaffold_21 | 98805 | 100592 |
| Block 853 | DDB_G0268140 | 1 | 1529143 | 1531470 | e_gw1.21.41.1 | scaffold_21 | 65171 | 67318 |
| Block 1806 | fam45 | 1 | 1546946 | 1548173 | e_gw1.396.14.1 | scaffold_396 | 13521 | 14717 |
| Block 1806 | DDB_G0268156 | 1 | 1548695 | 1550155 | estExt_fgeneshDP_pm.C_3960002 | scaffold_396 | 11742 | 13341 |
| Block 1806 | DDB_G0268572 | 1 | 1551637 | 1554960 | estExt_fgeneshDP_pg.C_3960004 | scaffold_396 | 7820 | 11295 |
| Block 1806 | eIF4e | 1 | 1556730 | 1557482 | e_gw1.396.20.1 | scaffold_396 | 2499 | 3263 |
| Block 1806 | DDB_G0268576 | 1 | 1558253 | 1561540 | GID1.0048554 | scaffold_396 | 114 | 1725 |
| Block 1445 | cshA | 1 | 1569279 | 1571100 | estExt_Genewise1.C_3120015 | scaffold_312 | 30570 | 32709 |
| Block 1445 | DDB_G0268158 | 1 | 1574564 | 1576904 | GID1.0047338 | scaffold_312 | 36357 | 38563 |
| Block 2095 | DDB_G0268162 | 1 | 1580521 | 1581951 | GID1.0049358 | scaffold_468 | 1384 | 2298 |
| Block 2095 | cdk8 | 1 | 1582311 | 1583530 | estExt_Genewise1Plus.C_4680003 | scaffold_468 | 2643 | 4104 |
| Block 2876 | DDB_G0268450 | 1 | 1586565 | 1588451 | GID1.0037832 | scaffold_8 | 26507 | 28362 |
| Block 2876 | DDB_G0268452 | 1 | 1588545 | 1589537 | GID1.0037833 | scaffold_8 | 28443 | 29325 |
| Block 491 | gppA | 1 | 1607815 | 1609553 | fgeneshDP_pg.C_scaffold_157000006 | scaffold_157 | 11392 | 14039 |
| Block 491 | DDB_G0268462 | 1 | 1630091 | 1632271 | GID1.0044019 | scaffold_157 | 1095 | 3128 |
| Block 920 | fut11 | 1 | 1637375 | 1639913 | e_gw1.22.44.1 | scaffold_22 | 89536 | 91762 |
| Block 920 | DDB_G0268194 | 1 | 1641107 | 1641971 | GID1.0038778 | scaffold_22 | 106136 | 106964 |
| Block 920 | DDB_G0268196 | 1 | 1642718 | 1645146 | fgeneshDP_pg.C_scaffold_22000039 | scaffold_22 | 107520 | 109917 |
| Block 920 | rpa43 | 1 | 1645550 | 1646794 | e_gw1.22.29.1 | scaffold_22 | 110334 | 110851 |
| Block 920 | acpA | 1 | 1647348 | 1648410 | estExt_Genewise1.C_220061 | scaffold_22 | 111934 | 113309 |
| Block 2272 | DDB_G0268200 | 1 | 1653352 | 1653765 | GID1.0049739 | scaffold_513 | 4177 | 4653 |
| Block 320 | kif12 | 1 | 1654404 | 1659091 | gw1.135.3.1 | scaffold_135 | 42057 | 43124 |
| Block 320 | vps46 | 1 | 1659361 | 1660037 | fgeneshDP_pg.C_scaffold_135000008 | scaffold_135 | 45804 | 46592 |
| Block 2272 | sahA | 1 | 1662307 | 1663602 | estExt_fgeneshDP_kg.C_5130001 | scaffold_513 | 6617 | 8092 |
| Block 2272 | DDB_G0268586 | 1 | 1664604 | 1667807 | e_gw1.513.12.1 | scaffold_513 | 1072 | 2330 |
| Block 320 | dlpA | 1 | 1676402 | 1680165 | e_gw1.135.13.1 | scaffold_135 | 2677 | 4858 |
| Block 1282 | DDB_G0268216 | 1 | 1702484 | 1708162 | GID1.0046813 | scaffold_283 | 4474 | 9826 |
| Block 1016 | DDB_G0268218 | 1 | 1708888 | 1711434 | estExt_fgeneshDP_pm.C_2340008 | scaffold_234 | 42695 | 45982 |
| Block 1016 | DDB_G0268220 | 1 | 1712183 | 1713033 | fgeneshDP_pg.C_scaffold_234000012 | scaffold_234 | 41377 | 42192 |
| Block 2272 | DDB_G0268222 | 1 | 1715767 | 1717245 | estExt_fgeneshDP_kg.C_5130002 | scaffold_513 | 12681 | 14566 |
| Block 2272 | DDB_G0268224 | 1 | 1719500 | 1721536 | fgeneshDP_pg.C_scaffold_513000005 | scaffold_513 | 8465 | 11074 |
| Block 1818 | DDB_G0268226 | 1 | 1721883 | 1722152 | GID1.0048597 | scaffold_399 | 3560 | 3984 |
| Block 1818 | DDB_G0268228 | 1 | 1723392 | 1725922 | gw1.399.7.1 | scaffold_399 | 1187 | 3287 |
| Block 884 | DDB_G0268232 | 1 | 1728957 | 1732034 | estExt_fgeneshDP_kg.C_2130002 | scaffold_213 | 32708 | 33386 |
| Block 884 | DDB_G0268234 | 1 | 1732577 | 1733416 | fgeneshDP_pg.C_scaffold_213000011 | scaffold_213 | 25389 | 25889 |
| Block 884 | DDB_G0268236 | 1 | 1733949 | 1735538 | e_gw1.213.17.1 | scaffold_213 | 23304 | 24932 |
| Block 884 | alg3 | 1 | 1736097 | 1737900 | e_gw1.213.18.1 | scaffold_213 | 21025 | 22651 |
| Block 884 | acrA | 1 | 1738891 | 1745341 | GID1.0045405 | scaffold_213 | 14474 | 20231 |
| Block 1282 | DDB_G0268244 | 1 | 1761068 | 1763956 | estExt_Genewise1Plus.C_2830001 | scaffold_283 | 1369 | 3946 |
| Block 2761 | DDB_G0268642 | 1 | 1801909 | 1805309 | GID1.0050807 | scaffold_715 | 3407 | 6411 |
| Block 1282 | atp5D | 1 | 1816266 | 1816778 | estExt_fgeneshDP_kg.C_2830003 | scaffold_283 | 20138 | 20897 |
| Block 2761 | DDB_G0268646 | 1 | 1826960 | 1827855 | estExt_fgeneshDP_kg.C_7150001 | scaffold_715 | 2385 | 3278 |
| Block 1282 | DDB_G0268652 | 1 | 1844690 | 1845832 | fgeneshDP_pg.C_scaffold_283000004 | scaffold_283 | 10307 | 11357 |
| Block 1360 | DDB_G0269048 | 1 | 1858322 | 1860328 | gw1.3.138.1 | scaffold_3 | 144316 | 145068 |
| Block 1813 | aatA | 1 | 1889432 | 1890712 | estExt_fgeneshDP_kg.C_3970002 | scaffold_397 | 16228 | 17658 |
| Block 1813 | cycH | 1 | 1891354 | 1892315 | e_gw1.397.19.1 | scaffold_397 | 14941 | 15695 |
| Block 1813 | DDB_G0269052 | 1 | 1898794 | 1901011 | e_gw1.397.16.1 | scaffold_397 | 11149 | 12890 |
| Block 1813 | DDB_G0268678 | 1 | 1901921 | 1904275 | GID1.0048571 | scaffold_397 | 8310 | 10389 |
| Block 1813 | DDB_G0268680 | 1 | 1904782 | 1906824 | GID1.0048570 | scaffold_397 | 5784 | 7714 |
| Block 1813 | DDB_G0268928 | 1 | 1909652 | 1911457 | GID1.0048569 | scaffold_397 | 1040 | 2599 |
| Block 1360 | DDB_G0268932 | 1 | 1915657 | 1916434 | fgeneshDP_pg.C_scaffold_3000058 | scaffold_3 | 145868 | 146540 |
| Block 1360 | DDB_G0268684 | 1 | 1916941 | 1919356 | GID1.0037416 | scaffold_3 | 97386 | 99882 |
| Block 1813 | ppwd1 | 1 | 1931476 | 1933597 | e_gw1.397.3.1 | scaffold_397 | 18194 | 20375 |
| Block 1813 | DDB_G0268696 | 1 | 1935672 | 1937431 | estExt_Genewise1Plus.C_3970021 | scaffold_397 | 22471 | 24986 |
| Block 1813 | DDB_G0268934 | 1 | 1937906 | 1938403 | fgeneshDP_pg.C_scaffold_397000012 | scaffold_397 | 25234 | 25700 |
| Block 1813 | DDB_G0268698 | 1 | 1938533 | 1940334 | GID1.0048579 | scaffold_397 | 25875 | 27971 |
| Block 1724 | DDB_G0268702 | 1 | 1941102 | 1943114 | estExt_Genewise1.C_3750025 | scaffold_375 | 26039 | 28096 |
| Block 1724 | DDB_G0268704 | 1 | 1943354 | 1944470 | e_gw1.375.10.1 | scaffold_375 | 28216 | 29568 |
| Block 1724 | ints3 | 1 | 1944951 | 1949839 | GID1.0048282 | scaffold_375 | 30320 | 32421 |
| Block 1820 | pinA | 1 | 1957162 | 1958072 | GID1.0048607 | scaffold_399 | 25610 | 26452 |
| Block 1820 | DDB_G0269060 | 1 | 1958550 | 1960468 | e_gw1.399.20.1 | scaffold_399 | 27154 | 28504 |
| Block 1820 | ube2w | 1 | 1961102 | 1961834 | GID1.0048606 | scaffold_399 | 24515 | 25144 |
| Block 1820 | DDB_G0268708 | 1 | 1964490 | 1965662 | GID1.0048603 | scaffold_399 | 17292 | 19476 |
| Block 1216 | pkbA | 1 | 1977388 | 1978925 | e_gw1.272.13.1 | scaffold_272 | 3476 | 4905 |
| Block 1216 | DDB_G0268712 | 1 | 1979347 | 1980121 | e_gw1.272.21.1 | scaffold_272 | 2283 | 3143 |
| Block 380 | DDB_G0269062 | 1 | 1988769 | 1990709 | e_gw1.142.19.1 | scaffold_142 | 64101 | 65762 |
| Block 380 | lsm5 | 1 | 1990918 | 1991418 | GID1.0043628 | scaffold_142 | 63294 | 63760 |
| Block 380 | DDB_G0268718 | 1 | 1991527 | 1993860 | fgeneshDP_pm.C_scaffold_142000009 | scaffold_142 | 60738 | 63184 |
| Block 380 | selk | 1 | 1994316 | 1994923 | fgeneshDP_pg.C_scaffold_142000022 | scaffold_142 | 59793 | 60304 |
| Block 380 | DDB_G0269078 | 1 | 2023544 | 2024644 | estExt_fgeneshDP_pg.C_1420021 | scaffold_142 | 57713 | 59325 |
| Block 380 | DDB_G0268746 | 1 | 2032965 | 2034502 | fgeneshDP_pg.C_scaffold_142000014 | scaffold_142 | 41456 | 42246 |
| Block 380 | DDB_G0268966 | 1 | 2042126 | 2043871 | estExt_Genewise1.C_1420023 | scaffold_142 | 52317 | 53985 |
| Block 1694 | DDB_G0268758 | 1 | 2048942 | 2050448 | fgeneshDP_pg.C_scaffold_369000014 | scaffold_369 | 28045 | 29895 |
| Block 1330 | rabQ | 1 | 2054001 | 2054913 | fgeneshDP_pg.C_scaffold_292000015 | scaffold_292 | 41287 | 42158 |
| Block 1330 | DDB_G0268762 | 1 | 2055281 | 2056126 | fgeneshDP_pm.C_scaffold_292000006 | scaffold_292 | 40405 | 41001 |
| Block 1464 | DDB_G0268774 | 1 | 2073845 | 2075041 | GID1.0047412 | scaffold_317 | 19859 | 20746 |
| Block 1464 | gatB | 1 | 2075252 | 2076868 | GID1.0047413 | scaffold_317 | 20931 | 22577 |
| Block 1464 | DDB_G0268988 | 1 | 2077109 | 2078704 | fgeneshDP_pm.C_scaffold_317000005 | scaffold_317 | 22665 | 24171 |
| Block 1464 | DDB_G0268990 | 1 | 2079025 | 2080332 | fgeneshDP_pm.C_scaffold_317000006 | scaffold_317 | 24576 | 25806 |
| Block 1464 | anapc10 | 1 | 2080758 | 2081980 | e_gw1.317.16.1 | scaffold_317 | 26032 | 27396 |
| Block 1464 | rbbB | 1 | 2082803 | 2086499 | e_gw1.317.1.1 | scaffold_317 | 28253 | 31402 |
| Block 380 | DDB_G0268786 | 1 | 2108112 | 2110076 | estExt_fgeneshDP_pm.C_1420002 | scaffold_142 | 8530 | 10410 |
| Block 380 | DDB_G0269000 | 1 | 2111460 | 2112649 | gw1.142.36.1 | scaffold_142 | 7817 | 7990 |
| Block 2361 | DDB_G0268790 | 1 | 2118381 | 2119262 | estExt_fgeneshDP_pg.C_5400004 | scaffold_540 | 11492 | 12495 |
| Block 2361 | gtaF | 1 | 2120474 | 2122457 | fgeneshDP_pm.C_scaffold_540000001 | scaffold_540 | 12997 | 14805 |
| Block 1694 | bub2 | 1 | 2123318 | 2124574 | GID1.0048197 | scaffold_369 | 11153 | 12318 |
| Block 1694 | DDB_G0268796 | 1 | 2124707 | 2125744 | estExt_Genewise1Plus.C_3690005 | scaffold_369 | 10170 | 11032 |
| Block 1694 | DDB_G0268798 | 1 | 2125974 | 2127217 | e_gw1.369.8.1 | scaffold_369 | 8970 | 9807 |
| Block 1694 | DDB_G0268800 | 1 | 2127909 | 2128811 | fgeneshDP_pg.C_scaffold_369000004 | scaffold_369 | 7190 | 7909 |
| Block 317 | abpA | 1 | 2137156 | 2139957 | estExt_Genewise1.C_1340006 | scaffold_134 | 8590 | 11417 |
| Block 1328 | DDB_G0268808 | 1 | 2141889 | 2145041 | fgeneshDP_pg.C_scaffold_292000011 | scaffold_292 | 30430 | 32078 |
| Block 1328 | DDB_G0268812 | 1 | 2154156 | 2155464 | gw1.292.7.1 | scaffold_292 | 28816 | 29883 |
| Block 1694 | DDB_G0268814 | 1 | 2157480 | 2160359 | estExt_fgeneshDP_pg.C_3690003 | scaffold_369 | 3711 | 7095 |
| Block 1758 | DDB_G0268818 | 1 | 2167055 | 2168395 | GID1.0048433 | scaffold_386 | 10637 | 12505 |
| Block 685 | DDB_G0269006 | 1 | 2170094 | 2173749 | GID1.0044849 | scaffold_189 | 56010 | 59144 |
| Block 685 | DDB_G0268820 | 1 | 2174037 | 2176085 | fgeneshDP_pg.C_scaffold_189000018 | scaffold_189 | 59427 | 60477 |
| Block 317 | DDB_G0268822 | 1 | 2179084 | 2182266 | estExt_fgeneshDP_kg.C_1340001 | scaffold_134 | 1392 | 2852 |
| Block 317 | DDB_G0268824 | 1 | 2182743 | 2184185 | e_gw1.134.30.1 | scaffold_134 | 2883 | 4305 |
| Block 1758 | DDB_G0268826 | 1 | 2184410 | 2187469 | estExt_fgeneshDP_kg.C_3860003 | scaffold_386 | 19650 | 21209 |
| Block 1758 | udkA | 1 | 2213969 | 2215930 | GID1.0048427 | scaffold_386 | 1716 | 3405 |
| Block 1758 | atg10 | 1 | 2216150 | 2216921 | e_gw1.386.15.1 | scaffold_386 | 3934 | 4844 |
| Block 1758 | spsA | 1 | 2217041 | 2218013 | e_gw1.386.9.1 | scaffold_386 | 4890 | 5810 |
| Block 1758 | pykA | 1 | 2219371 | 2220365 | e_gw1.386.5.1 | scaffold_386 | 8287 | 9290 |
| Block 125 | DDB_G0268850 | 1 | 2235139 | 2243566 | fgeneshDP_pg.C_scaffold_112000007 | scaffold_112 | 20471 | 28882 |
| Block 125 | DDB_G0268852 | 1 | 2244141 | 2252535 | estExt_fgeneshDP_pg.C_1120008 | scaffold_112 | 29403 | 37809 |
| Block 2656 | gnt4 | 1 | 2252863 | 2254749 | estExt_fgeneshDP_kg.C_660006 | scaffold_66 | 37439 | 39583 |
| Block 2656 | CYP524A1 | 1 | 2256023 | 2257759 | estExt_Genewise1Plus.C_660017 | scaffold_66 | 28394 | 30557 |
| Block 2357 | rngB | 1 | 2266964 | 2269912 | GID1.0040416 | scaffold_54 | 41269 | 44000 |
| Block 2357 | DDB_G0268862 | 1 | 2271073 | 2273030 | GID1.0040417 | scaffold_54 | 44880 | 46742 |
| Block 2357 | nat10 | 1 | 2281503 | 2284779 | GID1.0040419 | scaffold_54 | 49447 | 54048 |
| Block 1324 | DDB_G0268870 | 1 | 2286518 | 2287261 | estExt_Genewise1Plus.C_2920014 | scaffold_292 | 21969 | 22709 |
| Block 1324 | DDB_G0268872 | 1 | 2287976 | 2294601 | GID1.0046976 | scaffold_292 | 16485 | 21013 |
| Block 2656 | DDB_G0269022 | 1 | 2295154 | 2296270 | GID1.0040933 | scaffold_66 | 6804 | 7936 |
| Block 1135 | DDB_G0268876 | 1 | 2297918 | 2302573 | e_gw1.255.7.1 | scaffold_255 | 29714 | 34046 |
| Block 1135 | DDB_G0268878 | 1 | 2304383 | 2305729 | fgeneshDP_pg.C_scaffold_255000004 | scaffold_255 | 22602 | 23678 |
| Block 1135 | atxn10 | 1 | 2306169 | 2308216 | fgeneshDP_pm.C_scaffold_255000002 | scaffold_255 | 20464 | 22362 |
| Block 1135 | DDB_G0268886 | 1 | 2311232 | 2323957 | GID1.0046260 | scaffold_255 | 5054 | 17591 |
| Block 2357 | DDB_G0268888 | 1 | 2324197 | 2330890 | GID1.0040411 | scaffold_54 | 25908 | 32232 |
| Block 2357 | DDB_G0268890 | 1 | 2331887 | 2332531 | GID1.0040413 | scaffold_54 | 34704 | 35421 |
| Block 2357 | ate1 | 1 | 2333199 | 2335088 | e_gw1.54.25.1 | scaffold_54 | 36070 | 37944 |
| Block 2357 | DDB_G0268892 | 1 | 2336357 | 2337109 | estExt_fgeneshDP_kg.C_540005 | scaffold_54 | 39173 | 39956 |
| Block 1324 | wdr75 | 1 | 2341344 | 2344486 | fgeneshDP_pg.C_scaffold_292000003 | scaffold_292 | 4509 | 7558 |
| Block 1324 | DDB_G0268900 | 1 | 2344800 | 2345556 | fgeneshDP_pg.C_scaffold_292000002 | scaffold_292 | 3527 | 4271 |
| Block 318 | dhak | 1 | 2438501 | 2440838 | estExt_fgeneshDP_pg.C_1340013 | scaffold_134 | 45160 | 47433 |
| Block 318 | DDB_G0269276 | 1 | 2442246 | 2442509 | estExt_fgeneshDP_kg.C_1340006 | scaffold_134 | 48096 | 48656 |
| Block 318 | DDB_G0269280 | 1 | 2444312 | 2445271 | estExt_fgeneshDP_kg.C_1340007 | scaffold_134 | 48785 | 49949 |
| Block 318 | DDB_G0269282 | 1 | 2445906 | 2446880 | e_gw1.134.32.1 | scaffold_134 | 50564 | 51556 |
| Block 318 | DDB_G0269284 | 1 | 2449936 | 2451468 | fgeneshDP_pg.C_scaffold_134000012 | scaffold_134 | 42849 | 44228 |
| Block 318 | DDB_G0269286 | 1 | 2451692 | 2455913 | estExt_fgeneshDP_pg.C_1340011 | scaffold_134 | 38100 | 42495 |
| Block 318 | DDB_G0269288 | 1 | 2456628 | 2467773 | fgeneshDP_pg.C_scaffold_134000008 | scaffold_134 | 23083 | 33349 |
| Block 318 | nap1 | 1 | 2468482 | 2469686 | e_gw1.134.31.1 | scaffold_134 | 21069 | 22105 |
| Block 267 | DDB_G0269294 | 1 | 2471481 | 2474033 | GID1.0043206 | scaffold_129 | 18519 | 21881 |
| Block 318 | gefX | 1 | 2476607 | 2480100 | GID1.0043374 | scaffold_134 | 51920 | 55243 |
| Block 1357 | DDB_G0269300 | 1 | 2481510 | 2482781 | fgeneshDP_pg.C_scaffold_3000029 | scaffold_3 | 67732 | 68823 |
| Block 1357 | DDB_G0269302 | 1 | 2482897 | 2483850 | GID1.0037402 | scaffold_3 | 66463 | 67630 |
| Block 1357 | DDB_G0269304 | 1 | 2484101 | 2485936 | GID1.0037401 | scaffold_3 | 64449 | 66232 |
| Block 1357 | dhx35 | 1 | 2485989 | 2488139 | e_gw1.3.81.1 | scaffold_3 | 63166 | 64323 |
| Block 1357 | DDB_G0270470 | 1 | 2488609 | 2490364 | fgeneshDP_pg.C_scaffold_3000024 | scaffold_3 | 57202 | 58767 |
| Block 1357 | DDB_G0270472 | 1 | 2493841 | 2496701 | estExt_fgeneshDP_pg.C_30025 | scaffold_3 | 59171 | 61710 |
| Block 1357 | DDB_G0269312 | 1 | 2497222 | 2499823 | gw1.3.61.1 | scaffold_3 | 54523 | 57034 |
| Block 1357 | DDB_G0270474 | 1 | 2500071 | 2500579 | GID1.0037396 | scaffold_3 | 53704 | 54223 |
| Block 267 | gins4 | 1 | 2543534 | 2544345 | fgeneshDP_pg.C_scaffold_129000011 | scaffold_129 | 39645 | 40440 |
| Block 267 | zntD | 1 | 2544681 | 2547056 | GID1.0043216 | scaffold_129 | 41053 | 42620 |
| Block 318 | DDB_G0269330 | 1 | 2552986 | 2554021 | e_gw1.134.36.1 | scaffold_134 | 17642 | 18603 |
| Block 318 | DDB_G0269332 | 1 | 2554441 | 2556496 | estExt_fgeneshDP_kg.C_1340004 | scaffold_134 | 18838 | 20889 |
| Block 267 | DDB_G0269334 | 1 | 2556676 | 2557732 | fgeneshDP_kg.C_scaffold_129000003 | scaffold_129 | 37950 | 39619 |
| Block 368 | DDB_G0269336 | 1 | 2560862 | 2561113 | GID1.0043552 | scaffold_140 | 29359 | 29607 |
| Block 368 | gfm1 | 1 | 2561561 | 2563899 | GID1.0043551 | scaffold_140 | 26544 | 28832 |
| Block 1844 | DDB_G0269342 | 1 | 2567416 | 2567916 | GID1.0039778 | scaffold_40 | 109526 | 110057 |
| Block 1844 | DDB_G0269344 | 1 | 2568156 | 2569534 | e_gw1.40.55.1 | scaffold_40 | 107890 | 109254 |
| Block 1844 | DDB_G0269346 | 1 | 2569786 | 2570613 | fgeneshDP_pg.C_scaffold_40000031 | scaffold_40 | 106893 | 107708 |
| Block 2956 | DDB_G0269348 | 1 | 2570683 | 2572593 | GID1.0041803 | scaffold_87 | 33563 | 35519 |
| Block 2956 | DDB_G0269350 | 1 | 2573853 | 2575961 | e_gw1.87.23.1 | scaffold_87 | 67305 | 71787 |
| Block 2956 | mcfD | 1 | 2576335 | 2577600 | GID1.0041804 | scaffold_87 | 35803 | 37162 |
| Block 2956 | DDB_G0270854 | 1 | 2581654 | 2582858 | GID1.0041805 | scaffold_87 | 37939 | 39063 |
| Block 2956 | DDB_G0269356 | 1 | 2583576 | 2584480 | fgeneshDP_pg.C_scaffold_87000016 | scaffold_87 | 39279 | 40123 |
| Block 2956 | DDB_G0269358 | 1 | 2584566 | 2585608 | GID1.0041807 | scaffold_87 | 40268 | 41383 |
| Block 2956 | DDB_G0270856 | 1 | 2591243 | 2593999 | estExt_Genewise1.C_870022 | scaffold_87 | 47130 | 50004 |
| Block 2956 | DDB_G0270488 | 1 | 2595518 | 2606541 | estExt_Genewise1Plus.C_870025 | scaffold_87 | 51680 | 62524 |
| Block 2956 | DDB_G0270492 | 1 | 2607973 | 2609167 | GID1.0041811 | scaffold_87 | 62551 | 63875 |
| Block 1064 | frmB | 1 | 2609297 | 2610806 | GID1.0045995 | scaffold_242 | 31094 | 32560 |
| Block 1064 | DDB_G0270494 | 1 | 2611408 | 2612236 | fgeneshDP_pm.C_scaffold_242000005 | scaffold_242 | 33201 | 33973 |
| Block 370 | rab7A | 1 | 2627248 | 2628493 | estExt_fgeneshDP_kg.C_1400004 | scaffold_140 | 41799 | 43383 |
| Block 370 | DDB_G0269368 | 1 | 2629488 | 2630345 | fgeneshDP_pm.C_scaffold_140000007 | scaffold_140 | 40789 | 41483 |
| Block 2671 | DDB_G0269370 | 1 | 2630812 | 2631796 | GID1.0050619 | scaffold_662 | 8262 | 9201 |
| Block 2671 | DDB_G0269372 | 1 | 2632261 | 2632988 | estExt_Genewise1Plus.C_6620004 | scaffold_662 | 6220 | 6992 |
| Block 2671 | DDB_G0269374 | 1 | 2633520 | 2636041 | estExt_fgeneshDP_pg.C_6620003 | scaffold_662 | 3484 | 5944 |
| Block 2671 | DDB_G0270496 | 1 | 2636802 | 2638688 | GID1.0050614 | scaffold_662 | 846 | 2516 |
| Block 370 | DDB_G0269376 | 1 | 2642577 | 2643428 | e_gw1.140.13.1 | scaffold_140 | 37909 | 38706 |
| Block 370 | rad1 | 1 | 2644089 | 2645514 | e_gw1.140.38.1 | scaffold_140 | 39586 | 40588 |
| Block 2658 | DDB_G0269380 | 1 | 2645714 | 2650717 | estExt_Genewise1.C_660011 | scaffold_66 | 16453 | 23311 |
| Block 2658 | pepD | 1 | 2653185 | 2654791 | estExt_Genewise1Plus.C_660015 | scaffold_66 | 25102 | 26809 |
| Block 1947 | corB | 1 | 2678066 | 2680954 | estExt_fgeneshDP_pg.C_4270006 | scaffold_427 | 14978 | 18087 |
| Block 1947 | DDB_G0270500 | 1 | 2682747 | 2684442 | GID1.0048944 | scaffold_427 | 18304 | 19953 |
| Block 1095 | dhkE | 1 | 2700289 | 2705516 | GID1.0038934 | scaffold_25 | 75038 | 82713 |
| Block 1095 | DDB_G0269400 | 1 | 2710210 | 2711100 | estExt_fgeneshDP_pm.C_250013 | scaffold_25 | 83213 | 84389 |
| Block 1095 | DDB_G0269404 | 1 | 2713558 | 2714296 | GID1.0038936 | scaffold_25 | 84853 | 85501 |
| Block 1095 | DDB_G0269406 | 1 | 2715190 | 2716197 | GID1.0038937 | scaffold_25 | 86428 | 87290 |
| Block 1095 | spc2 | 1 | 2718039 | 2718873 | fgeneshDP_pg.C_scaffold_25000035 | scaffold_25 | 87867 | 88537 |
| Block 1095 | paf1 | 1 | 2724169 | 2725668 | estExt_Genewise1Plus.C_250057 | scaffold_25 | 88888 | 90327 |
| Block 1095 | ebp | 1 | 2725897 | 2726556 | estExt_fgeneshDP_kg.C_250007 | scaffold_25 | 56330 | 57133 |
| Block 1095 | rab32B | 1 | 2727197 | 2728127 | GID1.0038927 | scaffold_25 | 55042 | 55870 |
| Block 1095 | gxcB | 1 | 2732721 | 2736502 | e_gw1.25.35.1 | scaffold_25 | 71053 | 74808 |
| Block 1095 | magoh | 1 | 2747913 | 2749221 | estExt_fgeneshDP_kg.C_250001 | scaffold_25 | 11222 | 12644 |
| Block 1095 | DDB_G0270516 | 1 | 2750055 | 2752861 | e_gw1.25.41.1 | scaffold_25 | 13303 | 16426 |
| Block 1095 | DDB_G0269430 | 1 | 2753471 | 2756444 | GID1.0038907 | scaffold_25 | 5063 | 8064 |
| Block 1095 | DDB_G0269432 | 1 | 2756997 | 2760268 | GID1.0038906 | scaffold_25 | 1618 | 4637 |
| Block 1456 | DDB_G0270520 | 1 | 2765942 | 2767289 | estExt_fgeneshDP_pg.C_3160001 | scaffold_316 | 179 | 1513 |
| Block 1456 | DDB_G0269438 | 1 | 2767401 | 2768924 | estExt_fgeneshDP_pg.C_3160003 | scaffold_316 | 2602 | 4090 |
| Block 743 | DDB_G0270868 | 1 | 2774064 | 2776697 | estExt_fgeneshDP_pg.C_1970008 | scaffold_197 | 38858 | 41490 |
| Block 743 | atp9b | 1 | 2777615 | 2781943 | estExt_Genewise1.C_1970009 | scaffold_197 | 42191 | 46045 |
| Block 743 | DDB_G0269440 | 1 | 2782084 | 2784323 | GID1.0045029 | scaffold_197 | 46068 | 48284 |
| Block 1095 | DDB_G0269442 | 1 | 2784673 | 2787444 | fgeneshDP_pg.C_scaffold_25000022 | scaffold_25 | 52197 | 54668 |
| Block 1095 | hemB | 1 | 2788108 | 2789424 | fgeneshDP_pm.C_scaffold_25000007 | scaffold_25 | 50143 | 51353 |
| Block 1095 | DDB_G0269446 | 1 | 2789495 | 2790655 | estExt_Genewise1.C_250028 | scaffold_25 | 46634 | 48161 |
| Block 1095 | DDB_G0269448 | 1 | 2791078 | 2795119 | e_gw1.25.33.1 | scaffold_25 | 42273 | 46080 |
| Block 154 | DDB_G0294553 | 1 | 2805074 | 2806555 | e_gw1.116.21.1 | scaffold_116 | 59028 | 60383 |
| Block 154 | trpS | 1 | 2807320 | 2808805 | e_gw1.116.26.1 | scaffold_116 | 60950 | 62377 |
| Block 2360 | DDB_G0269460 | 1 | 2818161 | 2825118 | estExt_fgeneshDP_pg.C_5400002 | scaffold_540 | 5633 | 10662 |
| Block 2320 | DDB_G0269462 | 1 | 2825931 | 2833171 | fgeneshDP_pg.C_scaffold_53000014 | scaffold_53 | 29736 | 36696 |
| Block 2320 | DDB_G0269464 | 1 | 2833557 | 2833931 | e_gw1.53.86.1 | scaffold_53 | 40754 | 41064 |
| Block 2320 | DDB_G0269466 | 1 | 2834458 | 2835595 | GID1.0040370 | scaffold_53 | 41757 | 42665 |
| Block 2320 | mcfF | 1 | 2836764 | 2838030 | fgeneshDP_pg.C_scaffold_53000018 | scaffold_53 | 43071 | 44238 |
| Block 2320 | psmB2 | 1 | 2839118 | 2839714 | estExt_Genewise1.C_530041 | scaffold_53 | 45147 | 46037 |
| Block 2320 | fcsB | 1 | 2840597 | 2843560 | estExt_Genewise1.C_530042 | scaffold_53 | 46502 | 49043 |
| Block 1755 | DDB_G0269486 | 1 | 2860257 | 2863558 | GID1.0048419 | scaffold_385 | 11633 | 15113 |
| Block 1755 | DDB_G0269488 | 1 | 2863702 | 2864193 | fgeneshDP_pg.C_scaffold_385000006 | scaffold_385 | 11012 | 11469 |
| Block 1755 | nxnA | 1 | 2864584 | 2866468 | estExt_fgeneshDP_pm.C_3850002 | scaffold_385 | 7475 | 9134 |
| Block 2360 | DDB_G0270538 | 1 | 2869907 | 2873050 | GID1.0049941 | scaffold_540 | 1019 | 3183 |
| Block 2320 | gacZ | 1 | 2878932 | 2882063 | estExt_fgeneshDP_pg.C_530022 | scaffold_53 | 51772 | 54868 |
| Block 2320 | DDB_G0269498 | 1 | 2883107 | 2884312 | GID1.0040376 | scaffold_53 | 54935 | 57200 |
| Block 2660 | DDB_G0269500 | 1 | 2887577 | 2888427 | fgeneshDP_pg.C_scaffold_66000023 | scaffold_66 | 64814 | 65843 |
| Block 2660 | DDB_G0269502 | 1 | 2888658 | 2894331 | GID1.0040954 | scaffold_66 | 59055 | 64247 |
| Block 2660 | abcG1 | 1 | 2895800 | 2898550 | estExt_Genewise1Plus.C_660033 | scaffold_66 | 52281 | 54988 |
| Block 641 | cysA | 1 | 2903199 | 2904480 | GID1.0038537 | scaffold_18 | 132385 | 133681 |
| Block 641 | vps37 | 1 | 2905172 | 2906360 | e_gw1.18.50.1 | scaffold_18 | 134211 | 135411 |
| Block 641 | DG1007 | 1 | 2906854 | 2908497 | fgeneshDP_pg.C_scaffold_18000054 | scaffold_18 | 135905 | 137451 |
| Block 641 | DDB_G0269512 | 1 | 2908706 | 2909031 | gw1.18.75.1 | scaffold_18 | 137713 | 138069 |
| Block 371 | abnA | 1 | 2941556 | 2941915 | estExt_fgeneshDP_pm.C_1400010 | scaffold_140 | 52127 | 52634 |
| Block 41 | DDB_G0269522 | 1 | 2944304 | 2945241 | GID1.0042299 | scaffold_101 | 61376 | 62259 |
| Block 41 | argD | 1 | 2946561 | 2947922 | GID1.0042300 | scaffold_101 | 63826 | 65151 |
| Block 371 | DDB_G0270552 | 1 | 2951187 | 2953550 | fgeneshDP_pg.C_scaffold_140000019 | scaffold_140 | 53773 | 56025 |
| Block 2660 | DDB_G0269546 | 1 | 2969017 | 2974040 | e_gw1.66.3.1 | scaffold_66 | 40230 | 44597 |
| Block 152 | DDB_G0269548 | 1 | 2974419 | 2975425 | e_gw1.116.27.1 | scaffold_116 | 44413 | 45611 |
| Block 371 | snwA | 1 | 2978336 | 2980393 | estExt_fgeneshDP_kg.C_1400006 | scaffold_140 | 64672 | 65465 |
| Block 371 | calB | 1 | 2981116 | 2981944 | fgeneshDP_pm.C_scaffold_140000012 | scaffold_140 | 61322 | 62016 |
| Block 371 | DDB_G0269552 | 1 | 2982129 | 2982680 | gw1.140.49.1 | scaffold_140 | 60652 | 60879 |
| Block 371 | suvA | 1 | 2983180 | 2987875 | estExt_Genewise1Plus.C_1400035 | scaffold_140 | 56038 | 57137 |
| Block 152 | fcsA | 1 | 2990411 | 2993319 | estExt_Genewise1.C_1160022 | scaffold_116 | 46229 | 49548 |
| Block 152 | DDB_G0269560 | 1 | 2996712 | 2998612 | estExt_Genewise1.C_1160020 | scaffold_116 | 42152 | 44339 |
| Block 1838 | abcG14 | 1 | 3002229 | 3006724 | GID1.0039780 | scaffold_40 | 114081 | 120277 |
| Block 152 | DDB_G0270828 | 1 | 3009708 | 3012882 | fgeneshDP_pm.C_scaffold_116000004 | scaffold_116 | 38462 | 41691 |
| Block 723 | DDB_G0269566 | 1 | 3015026 | 3015487 | GID1.0044968 | scaffold_194 | 36455 | 36928 |
| Block 371 | med7 | 1 | 3017741 | 3018878 | GID1.0043567 | scaffold_140 | 65516 | 66477 |
| Block 371 | rabggta | 1 | 3019090 | 3020441 | e_gw1.140.41.1 | scaffold_140 | 66719 | 67945 |
| Block 371 | trfA | 1 | 3020703 | 3025206 | GID1.0043570 | scaffold_140 | 69029 | 71628 |
| Block 723 | DDB_G0269572 | 1 | 3034894 | 3039544 | estExt_Genewise1.C_1940042 | scaffold_194 | 37162 | 41896 |
| Block 1838 | dcd3B | 1 | 3041058 | 3041915 | estExt_fgeneshDP_kg.C_400002 | scaffold_40 | 17741 | 19098 |
| Block 1838 | mybP | 1 | 3042784 | 3047317 | fgeneshDP_pg.C_scaffold_40000007 | scaffold_40 | 11989 | 15941 |
| Block 1838 | acy1 | 1 | 3048022 | 3049597 | estExt_Genewise1Plus.C_400010 | scaffold_40 | 10134 | 11597 |
| Block 2236 | DG1110 | 1 | 3055800 | 3057686 | GID1.0049671 | scaffold_503 | 5229 | 6437 |
| Block 2236 | gapA | 1 | 3059126 | 3062012 | e_gw1.503.2.1 | scaffold_503 | 668 | 3262 |
| Block 1971 | DDB_G0269580 | 1 | 3064049 | 3065362 | gw1.433.2.1 | scaffold_433 | 21342 | 22568 |
| Block 1971 | DDB_G0270566 | 1 | 3070710 | 3072538 | estExt_Genewise1.C_4330001 | scaffold_433 | 15326 | 17330 |
| Block 1838 | DDB_G0270568 | 1 | 3075849 | 3077228 | estExt_Genewise1.C_400056 | scaffold_40 | 70845 | 72190 |
| Block 1838 | DDB_G0269588 | 1 | 3077286 | 3078470 | GID1.0039762 | scaffold_40 | 72259 | 73528 |
| Block 1838 | DDB_G0269596 | 1 | 3090844 | 3091928 | fgeneshDP_pg.C_scaffold_40000020 | scaffold_40 | 77019 | 77808 |
| Block 1838 | DDB_G0269598 | 1 | 3092703 | 3093698 | GID1.0039764 | scaffold_40 | 78849 | 79725 |
| Block 1838 | DDB_G0270890 | 1 | 3096057 | 3096885 | fgeneshDP_pg.C_scaffold_40000001 | scaffold_40 | 556 | 1433 |
| Block 1838 | DDB_G0270892 | 1 | 3109107 | 3109977 | GID1.0039745 | scaffold_40 | 2147 | 3049 |
| Block 1838 | DDB_G0269602 | 1 | 3110201 | 3111767 | fgeneshDP_pg.C_scaffold_40000003 | scaffold_40 | 3334 | 4676 |
| Block 1194 | lvsA | 1 | 3119127 | 3130279 | GID1.0046521 | scaffold_267 | 30794 | 41116 |
| Block 1194 | DDB_G0269608 | 1 | 3131550 | 3134161 | GID1.0046522 | scaffold_267 | 41789 | 44396 |
| Block 2236 | gxcT | 1 | 3136522 | 3141488 | fgeneshDP_pg.C_scaffold_503000005 | scaffold_503 | 12502 | 16848 |
| Block 2236 | DDB_G0269612 | 1 | 3141822 | 3142493 | e_gw1.503.7.1 | scaffold_503 | 11664 | 12319 |
| Block 2236 | DDB_G0270578 | 1 | 3142550 | 3144837 | e_gw1.503.4.1 | scaffold_503 | 9628 | 11606 |
| Block 2236 | DDB_G0269614 | 1 | 3146250 | 3147915 | GID1.0049672 | scaffold_503 | 6850 | 8159 |
| Block 1194 | DDB_G0269616 | 1 | 3148640 | 3149056 | e_gw1.267.18.1 | scaffold_267 | 29914 | 30396 |
| Block 1194 | DDB_G0269618 | 1 | 3150646 | 3151839 | fgeneshDP_pg.C_scaffold_267000011 | scaffold_267 | 27055 | 28359 |
| Block 1194 | DDB_G0270580 | 1 | 3152348 | 3153799 | fgeneshDP_pm.C_scaffold_267000003 | scaffold_267 | 25675 | 26838 |
| Block 1194 | DDB_G0270582 | 1 | 3155421 | 3157129 | gw1.267.8.1 | scaffold_267 | 22482 | 23723 |
| Block 1194 | pyd2 | 1 | 3157203 | 3158848 | e_gw1.267.6.1 | scaffold_267 | 20459 | 22111 |
| Block 724 | DDB_G0270584 | 1 | 3164918 | 3165853 | fgeneshDP_pg.C_scaffold_194000021 | scaffold_194 | 56244 | 57753 |
| Block 724 | celB | 1 | 3166724 | 3168412 | GID1.0044972 | scaffold_194 | 53402 | 55892 |
| Block 724 | mgp3 | 1 | 3170314 | 3174422 | estExt_Genewise1Plus.C_1940045 | scaffold_194 | 48611 | 52295 |
| Block 724 | forE | 1 | 3176439 | 3181124 | GID1.0044970 | scaffold_194 | 43142 | 47409 |
| Block 1843 | DDB_G0269628 | 1 | 3183487 | 3185815 | estExt_Genewise1Plus.C_400063 | scaffold_40 | 84441 | 87823 |
| Block 1843 | DDB_G0269630 | 1 | 3187028 | 3188197 | fgeneshDP_pg.C_scaffold_40000016 | scaffold_40 | 69377 | 70527 |
| Block 758 | irlD | 1 | 3190841 | 3195534 | estExt_fgeneshDP_pg.C_20039 | scaffold_2 | 108445 | 112313 |
| Block 758 | irlC | 1 | 3195968 | 3200425 | estExt_fgeneshDP_pg.C_20026 | scaffold_2 | 74595 | 78795 |
| Block 3029 | DDB_G0269634 | 1 | 3201588 | 3202019 | estExt_fgeneshDP_kg.C_950003 | scaffold_95 | 37818 | 38344 |
| Block 2417 | DDB_G0269636 | 1 | 3202239 | 3203453 | GID1.0050094 | scaffold_564 | 11435 | 12634 |
| Block 2417 | dr1 | 1 | 3204015 | 3204688 | GID1.0050095 | scaffold_564 | 13393 | 14039 |
| Block 1267 | DDB_G0269640 | 1 | 3205382 | 3206294 | fgeneshDP_pm.C_scaffold_280000001 | scaffold_280 | 822 | 1737 |
| Block 1267 | mak16l | 1 | 3206739 | 3208141 | fgeneshDP_pm.C_scaffold_280000002 | scaffold_280 | 2156 | 3430 |
| Block 1267 | DDB_G0270592 | 1 | 3212380 | 3212770 | e_gw1.280.29.1 | scaffold_280 | 6881 | 7254 |
| Block 1267 | DDB_G0269644 | 1 | 3214845 | 3216721 | fgeneshDP_pm.C_scaffold_280000005 | scaffold_280 | 10006 | 11666 |
| Block 1267 | DDB_G0269646 | 1 | 3217078 | 3218041 | fgeneshDP_pm.C_scaffold_280000006 | scaffold_280 | 11968 | 12741 |
| Block 3029 | DDB_G0270900 | 1 | 3225362 | 3225869 | gw1.95.35.1 | scaffold_95 | 23007 | 23406 |
| Block 3029 | rcdP | 1 | 3226604 | 3229060 | estExt_Genewise1Plus.C_950017 | scaffold_95 | 20008 | 22577 |
| Block 3029 | catB | 1 | 3244376 | 3246648 | estExt_fgeneshDP_pg.C_950006 | scaffold_95 | 11005 | 13583 |
| Block 3029 | DDB_G0269658 | 1 | 3247871 | 3248759 | GID1.0042075 | scaffold_95 | 9045 | 9936 |
| Block 2957 | tubB | 1 | 3261649 | 3263130 | estExt_Genewise1.C_870038 | scaffold_87 | 72168 | 74192 |
| Block 2957 | cf45-1 | 1 | 3267307 | 3268805 | e_gw1.87.38.1 | scaffold_87 | 81118 | 82660 |
| Block 913 | DDB_G0269666 | 1 | 3273351 | 3273836 | GID1.0045527 | scaffold_219 | 23006 | 23498 |
| Block 913 | modA | 1 | 3274209 | 3277152 | estExt_Genewise1.C_2190008 | scaffold_219 | 23725 | 26834 |
| Block 913 | DDB_G0269668 | 1 | 3278152 | 3278565 | GID1.0045529 | scaffold_219 | 27325 | 27702 |
| Block 913 | DDB_G0269670 | 1 | 3279591 | 3281482 | fgeneshDP_pg.C_scaffold_219000012 | scaffold_219 | 28721 | 30562 |
| Block 2359 | DDB_G0270606 | 1 | 3287288 | 3289621 | estExt_fgeneshDP_kg.C_540008 | scaffold_54 | 106969 | 107675 |
| Block 2359 | DDB_G0269676 | 1 | 3289699 | 3291350 | fgeneshDP_pg.C_scaffold_54000036 | scaffold_54 | 105428 | 106914 |
| Block 2359 | jcdD | 1 | 3291492 | 3292937 | estExt_fgeneshDP_kg.C_540007 | scaffold_54 | 103520 | 104465 |
| Block 2359 | DDB_G0270910 | 1 | 3293315 | 3296614 | GID1.0040438 | scaffold_54 | 100665 | 103393 |
| Block 2359 | DDB_G0270912 | 1 | 3297321 | 3297636 | GID1.0040437 | scaffold_54 | 99611 | 99889 |
| Block 2359 | DDB_G0270610 | 1 | 3299624 | 3300197 | GID1.0040435 | scaffold_54 | 97113 | 97671 |
| Block 913 | DDB_G0269678 | 1 | 3301231 | 3305445 | estExt_Genewise1.C_2190014 | scaffold_219 | 46089 | 49480 |
| Block 913 | DDB_G0269680 | 1 | 3305788 | 3308565 | fgeneshDP_pg.C_scaffold_219000017 | scaffold_219 | 37616 | 39853 |
| Block 913 | atxn2 | 1 | 3309660 | 3313064 | estExt_fgeneshDP_pg.C_2190015 | scaffold_219 | 34539 | 37407 |
| Block 2359 | DDB_G0269686 | 1 | 3319946 | 3322896 | estExt_fgeneshDP_pg.C_540021 | scaffold_54 | 54442 | 57453 |
| Block 2359 | sdad1 | 1 | 3323737 | 3326298 | GID1.0040421 | scaffold_54 | 59043 | 61460 |
| Block 2359 | DDB_G0270916 | 1 | 3330425 | 3331198 | GID1.0040422 | scaffold_54 | 62026 | 62715 |
| Block 2359 | DDB_G0270918 | 1 | 3332298 | 3336672 | estExt_fgeneshDP_pg.C_540024 | scaffold_54 | 63780 | 67821 |
| Block 1063 | pan2 | 1 | 3339032 | 3343830 | GID1.0045989 | scaffold_242 | 11949 | 16471 |
| Block 1063 | pakD | 1 | 3345400 | 3350536 | GID1.0045990 | scaffold_242 | 17330 | 21940 |
| Block 1063 | DDB_G0269698 | 1 | 3350709 | 3351586 | fgeneshDP_pg.C_scaffold_242000005 | scaffold_242 | 22187 | 23072 |
| Block 1063 | DDB_G0269700 | 1 | 3352107 | 3354728 | estExt_Genewise1Plus.C_2420016 | scaffold_242 | 23586 | 31039 |
| Block 1723 | racH | 1 | 3360340 | 3361265 | e_gw1.375.17.1 | scaffold_375 | 10106 | 11006 |
| Block 1723 | bopA | 1 | 3361680 | 3363770 | fgeneshDP_pm.C_scaffold_375000001 | scaffold_375 | 7756 | 9798 |
| Block 1723 | DDB_G0270618 | 1 | 3363806 | 3365508 | GID1.0048271 | scaffold_375 | 5923 | 7303 |
| Block 1723 | DDB_G0269704 | 1 | 3367372 | 3368994 | GID1.0048276 | scaffold_375 | 15094 | 16544 |
| Block 1981 | fah | 1 | 3369734 | 3371095 | estExt_Genewise1.C_4380005 | scaffold_438 | 6406 | 7971 |
| Block 1981 | cypB | 1 | 3371521 | 3372496 | fgeneshDP_pm.C_scaffold_438000001 | scaffold_438 | 5050 | 5891 |
| Block 1981 | DDB_G0269706 | 1 | 3373104 | 3374984 | GID1.0049055 | scaffold_438 | 2614 | 4003 |
| Block 1981 | DDB_G0270920 | 1 | 3375140 | 3378478 | fgeneshDP_pg.C_scaffold_438000001 | scaffold_438 | 145 | 2535 |
| Block 1322 | DDB_G0269710 | 1 | 3383680 | 3388102 | estExt_fgeneshDP_pg.C_2910005 | scaffold_291 | 11863 | 16123 |
| Block 1322 | ahsa | 1 | 3388870 | 3390021 | GID1.0046954 | scaffold_291 | 5387 | 6521 |
| Block 1322 | DDB_G0270622 | 1 | 3391069 | 3391491 | GID1.0046955 | scaffold_291 | 7269 | 7837 |
| Block 1322 | tdo | 1 | 3391649 | 3393035 | fgeneshDP_pm.C_scaffold_291000002 | scaffold_291 | 8047 | 9416 |
| Block 1361 | DDB_G0269716 | 1 | 3397004 | 3398144 | e_gw1.3.106.1 | scaffold_3 | 229172 | 230464 |
| Block 1361 | DDB_G0270928 | 1 | 3398987 | 3400602 | fgeneshDP_pm.C_scaffold_3000031 | scaffold_3 | 226492 | 228105 |
| Block 1361 | DDB_G0269718 | 1 | 3402124 | 3406095 | estExt_fgeneshDP_pg.C_30084 | scaffold_3 | 221471 | 226106 |
| Block 1361 | abcB7 | 1 | 3410490 | 3413579 | fgeneshDP_pg.C_scaffold_3000082 | scaffold_3 | 209525 | 212085 |
| Block 1361 | DDB_G0269722 | 1 | 3414230 | 3415170 | GID1.0037458 | scaffold_3 | 187210 | 189266 |
| Block 1361 | DDB_G0270932 | 1 | 3419847 | 3423103 | estExt_fgeneshDP_pg.C_30075 | scaffold_3 | 193916 | 198871 |
| Block 1361 | DDB_G0269724 | 1 | 3424394 | 3425573 | estExt_Genewise1Plus.C_30134 | scaffold_3 | 199992 | 201501 |
| Block 1361 | DDB_G0269726 | 1 | 3425762 | 3426901 | GID1.0037461 | scaffold_3 | 201535 | 202683 |
| Block 1361 | ciao1 | 1 | 3427650 | 3428995 | fgeneshDP_pg.C_scaffold_3000078 | scaffold_3 | 202987 | 204292 |
| Block 1355 | abcG22 | 1 | 3432396 | 3434782 | GID1.0037392 | scaffold_3 | 42498 | 44522 |
| Block 1355 | pakA | 1 | 3436536 | 3441900 | estExt_fgeneshDP_pg.C_30016 | scaffold_3 | 35563 | 41045 |
| Block 2356 | armc8 | 1 | 3447052 | 3449746 | GID1.0040409 | scaffold_54 | 20738 | 22973 |
| Block 1137 | DDB_G0269734 | 1 | 3450462 | 3451264 | GID1.0046276 | scaffold_255 | 43100 | 43866 |
| Block 1137 | DDB_G0269736 | 1 | 3451792 | 3454310 | fgeneshDP_pg.C_scaffold_255000012 | scaffold_255 | 40580 | 42806 |
| Block 1137 | desA | 1 | 3455193 | 3456362 | GID1.0046273 | scaffold_255 | 37800 | 38967 |
| Block 1361 | cdsA | 1 | 3466584 | 3468271 | estExt_Genewise1.C_30078 | scaffold_3 | 131454 | 133175 |
| Block 1361 | DDB_G0269744 | 1 | 3468500 | 3469879 | fgeneshDP_pg.C_scaffold_3000041 | scaffold_3 | 110078 | 111208 |
| Block 1355 | ITPK1 | 1 | 3471589 | 3472824 | GID1.0037384 | scaffold_3 | 20413 | 21705 |
| Block 1355 | DDB_G0269748 | 1 | 3473013 | 3474697 | estExt_Genewise1.C_30018 | scaffold_3 | 21807 | 24016 |
| Block 1355 | cas1 | 1 | 3476704 | 3479389 | fgeneshDP_pg.C_scaffold_3000013 | scaffold_3 | 28064 | 30440 |
| Block 2356 | DDB_G0270934 | 1 | 3485944 | 3487242 | GID1.0040410 | scaffold_54 | 24559 | 25559 |
| Block 1076 | DDB_G0270936 | 1 | 3488110 | 3489009 | e_gw1.244.16.1 | scaffold_244 | 27606 | 28286 |
| Block 1076 | DDB_G0269756 | 1 | 3489330 | 3491399 | e_gw1.244.8.1 | scaffold_244 | 24993 | 26968 |
| Block 1076 | DDB_G0270632 | 1 | 3492337 | 3494799 | fgeneshDP_pg.C_scaffold_244000010 | scaffold_244 | 21869 | 24713 |
| Block 381 | dcsA | 1 | 3500588 | 3503767 | estExt_fgeneshDP_kg.C_1420006 | scaffold_142 | 33936 | 41183 |
| Block 1463 | DDB_G0269760 | 1 | 3510769 | 3511836 | GID1.0047420 | scaffold_317 | 35689 | 36894 |
| Block 1321 | DDB_G0269762 | 1 | 3512075 | 3514549 | fgeneshDP_pg.C_scaffold_291000010 | scaffold_291 | 24067 | 26322 |
| Block 1321 | cnrC | 1 | 3515795 | 3517258 | fgeneshDP_pm.C_scaffold_291000008 | scaffold_291 | 21322 | 22968 |
| Block 1321 | DDB_G0270942 | 1 | 3518935 | 3519427 | fgeneshDP_pg.C_scaffold_291000008 | scaffold_291 | 20813 | 21169 |
| Block 1321 | cnrI | 1 | 3520960 | 3522817 | estExt_Genewise1Plus.C_2910018 | scaffold_291 | 18623 | 20453 |
| Block 1321 | DDB_G0269770 | 1 | 3523265 | 3524869 | fgeneshDP_pg.C_scaffold_291000006 | scaffold_291 | 16808 | 18374 |
| Block 381 | psmB3 | 1 | 3525818 | 3526839 | estExt_Genewise1.C_1420016 | scaffold_142 | 30742 | 31949 |
| Block 381 | DDB_G0269774 | 1 | 3527142 | 3528263 | GID1.0043613 | scaffold_142 | 29576 | 30469 |
| Block 381 | DDB_G0269776 | 1 | 3528359 | 3529764 | fgeneshDP_pg.C_scaffold_142000010 | scaffold_142 | 28193 | 29492 |
| Block 381 | DDB_G0270634 | 1 | 3530459 | 3532109 | GID1.0043610 | scaffold_142 | 24680 | 26190 |
| Block 381 | DDB_G0270636 | 1 | 3534092 | 3537641 | estExt_fgeneshDP_pg.C_1420008 | scaffold_142 | 19077 | 22168 |
| Block 1321 | tifA | 1 | 3537960 | 3539386 | GID1.0046957 | scaffold_291 | 10050 | 11524 |
| Block 1321 | gefB | 1 | 3539581 | 3544525 | estExt_fgeneshDP_pg.C_2910001 | scaffold_291 | 1052 | 5133 |
| Block 1463 | DDB_G0269778 | 1 | 3547267 | 3548768 | GID1.0047409 | scaffold_317 | 13461 | 14908 |
| Block 1463 | DDB_G0269780 | 1 | 3549387 | 3553055 | fgeneshDP_pg.C_scaffold_317000009 | scaffold_317 | 15827 | 18966 |
| Block 1354 | DDB_G0269786 | 1 | 3556678 | 3557509 | fgeneshDP_pg.C_scaffold_299000011 | scaffold_299 | 28431 | 29440 |
| Block 1354 | DDB_G0269788 | 1 | 3558146 | 3559074 | GID1.0047096 | scaffold_299 | 26614 | 27514 |
| Block 1819 | fntB | 1 | 3561059 | 3562855 | e_gw1.399.9.1 | scaffold_399 | 8052 | 9508 |
| Block 1819 | gaa | 1 | 3563596 | 3566431 | GID1.0048598 | scaffold_399 | 4189 | 7038 |
| Block 381 | DDB_G0269794 | 1 | 3580948 | 3581715 | estExt_fgeneshDP_pm.C_1420011 | scaffold_142 | 65814 | 66460 |
| Block 1074 | DDB_G0270640 | 1 | 3581899 | 3584968 | GID1.0046030 | scaffold_244 | 8217 | 10993 |
| Block 1074 | DDB_G0270966 | 1 | 3627252 | 3628552 | estExt_Genewise1Plus.C_2440007 | scaffold_244 | 13532 | 14820 |
| Block 1074 | DDB_G0269800 | 1 | 3628624 | 3631009 | fgeneshDP_pm.C_scaffold_244000003 | scaffold_244 | 14868 | 16914 |
| Block 1491 | mvpA | 1 | 3649783 | 3652408 | estExt_Genewise1.C_3230009 | scaffold_323 | 23483 | 26219 |
| Block 1491 | DDB_G0269816 | 1 | 3664995 | 3666427 | e_gw1.323.13.1 | scaffold_323 | 33724 | 35128 |
| Block 1491 | mhisS | 1 | 3669575 | 3671080 | gw1.323.8.1 | scaffold_323 | 28008 | 29310 |
| Block 1491 | DDB_G0269824 | 1 | 3671523 | 3672995 | GID1.0047508 | scaffold_323 | 26398 | 27403 |
| Block 1779 | acbA | 1 | 3673860 | 3674359 | e_gw1.39.106.1 | scaffold_39 | 77861 | 78291 |
| Block 1779 | DDB_G0269826 | 1 | 3675572 | 3680023 | estExt_Genewise1Plus.C_390077 | scaffold_39 | 78738 | 82832 |
| Block 1779 | fbp | 1 | 3681011 | 3682051 | estExt_Genewise1.C_390080 | scaffold_39 | 83696 | 85006 |
| Block 1491 | DDB_G0269828 | 1 | 3682321 | 3683577 | fgeneshDP_pm.C_scaffold_323000006 | scaffold_323 | 32263 | 33441 |
| Block 1491 | DDB_G0269830 | 1 | 3683818 | 3685422 | fgeneshDP_pg.C_scaffold_323000013 | scaffold_323 | 30563 | 32008 |
| Block 577 | DDB_G0270972 | 1 | 3691172 | 3692200 | estExt_fgeneshDP_pg.C_1700001 | scaffold_170 | 364 | 1624 |
| Block 1158 | DDB_G0270662 | 1 | 3696190 | 3696483 | estExt_fgeneshDP_kg.C_260002 | scaffold_26 | 22225 | 22684 |
| Block 577 | DDB_G0269840 | 1 | 3697371 | 3699160 | GID1.0044378 | scaffold_170 | 8276 | 9829 |
| Block 577 | DDB_G0270664 | 1 | 3699523 | 3701813 | estExt_fgeneshDP_pm.C_1700003 | scaffold_170 | 10102 | 12090 |
| Block 577 | DDB_G0269842 | 1 | 3701971 | 3707422 | estExt_fgeneshDP_pg.C_1700008 | scaffold_170 | 13757 | 16658 |
| Block 1158 | DDB_G0269848 | 1 | 3711642 | 3713063 | e_gw1.26.84.1 | scaffold_26 | 20852 | 22162 |
| Block 1158 | psaB | 1 | 3731263 | 3734083 | estExt_Genewise1.C_260017 | scaffold_26 | 27039 | 29968 |
| Block 1158 | DDB_G0269220 | 1 | 3734477 | 3734926 | fgeneshDP_pg.C_scaffold_26000012 | scaffold_26 | 31284 | 31697 |
| Block 1158 | racG | 1 | 3735334 | 3735939 | fgeneshDP_pg.C_scaffold_26000013 | scaffold_26 | 31832 | 32416 |
| Block 1158 | ddcA | 1 | 3743580 | 3744917 | estExt_Genewise1.C_260031 | scaffold_26 | 36266 | 37639 |
| Block 1158 | DDB_G0269858 | 1 | 3745025 | 3746842 | GID1.0038983 | scaffold_26 | 38526 | 40282 |
| Block 1158 | DDB_G0269860 | 1 | 3747132 | 3750705 | fgeneshDP_pg.C_scaffold_26000019 | scaffold_26 | 40604 | 44088 |
| Block 1158 | DDB_G0270980 | 1 | 3766207 | 3767162 | gw1.26.103.1 | scaffold_26 | 23945 | 24454 |
| Block 1158 | tspB | 1 | 3769162 | 3770134 | estExt_fgeneshDP_kg.C_260004 | scaffold_26 | 51658 | 59285 |
| Block 1158 | DDB_G0269880 | 1 | 3785577 | 3786992 | e_gw1.26.13.1 | scaffold_26 | 50024 | 51442 |
| Block 196 | DDB_G0269884 | 1 | 3797962 | 3800493 | fgeneshDP_pm.C_scaffold_120000010 | scaffold_120 | 47162 | 49739 |
| Block 196 | DDB_G0269886 | 1 | 3801076 | 3802258 | estExt_fgeneshDP_pg.C_1200025 | scaffold_120 | 50198 | 51523 |
| Block 2261 | DDB_G0269888 | 1 | 3802780 | 3803589 | estExt_Genewise1Plus.C_510056 | scaffold_51 | 104984 | 105673 |
| Block 2261 | DDB_G0269890 | 1 | 3803772 | 3804667 | GID1.0040304 | scaffold_51 | 103940 | 104744 |
| Block 2261 | DDB_G0269892 | 1 | 3806514 | 3807971 | GID1.0040303 | scaffold_51 | 100596 | 102498 |
| Block 1478 | rsc5 | 1 | 3809035 | 3810441 | fgeneshDP_pg.C_scaffold_32000038 | scaffold_32 | 96859 | 98074 |
| Block 1478 | orcB | 1 | 3810811 | 3812262 | e_gw1.32.59.1 | scaffold_32 | 98347 | 99655 |
| Block 746 | kcnma1 | 1 | 3812473 | 3816336 | estExt_fgeneshDP_pg.C_1980017 | scaffold_198 | 39342 | 43275 |
| Block 746 | phgA | 1 | 3817626 | 3819202 | fgeneshDP_pg.C_scaffold_198000016 | scaffold_198 | 36719 | 38050 |
| Block 746 | DDB_G0269898 | 1 | 3820454 | 3823469 | GID1.0045052 | scaffold_198 | 33280 | 35117 |
| Block 746 | DDB_G0270686 | 1 | 3829651 | 3831106 | estExt_fgeneshDP_pg.C_1980014 | scaffold_198 | 31721 | 32811 |
| Block 1953 | gtf2h5 | 1 | 3831161 | 3831468 | e_gw1.429.11.1 | scaffold_429 | 21421 | 21709 |
| Block 1953 | DDB_G0269908 | 1 | 3831693 | 3832921 | estExt_Genewise1Plus.C_4290009 | scaffold_429 | 17900 | 19697 |
| Block 1953 | DDB_G0270990 | 1 | 3834760 | 3836838 | GID1.0048963 | scaffold_429 | 14222 | 16350 |
| Block 196 | sec31 | 1 | 3837509 | 3841658 | GID1.0042935 | scaffold_120 | 36084 | 39855 |
| Block 2261 | psaA | 1 | 3842009 | 3844717 | estExt_Genewise1.C_510044 | scaffold_51 | 89608 | 92679 |
| Block 1478 | DDB_G0270996 | 1 | 3845549 | 3846750 | GID1.0039363 | scaffold_32 | 101761 | 102857 |
| Block 1478 | DDB_G0269910 | 1 | 3847028 | 3848476 | fgeneshDP_pg.C_scaffold_32000040 | scaffold_32 | 100281 | 101430 |
| Block 1157 | redB | 1 | 3848909 | 3851073 | GID1.0038969 | scaffold_26 | 8933 | 12157 |
| Block 1157 | tpp1 | 1 | 3852049 | 3853851 | GID1.0038970 | scaffold_26 | 13312 | 15456 |
| Block 1157 | atp5b | 1 | 3855247 | 3857504 | estExt_Genewise1Plus.C_260009 | scaffold_26 | 16746 | 19790 |
| Block 1157 | mpl2 | 1 | 3859398 | 3861680 | estExt_Genewise1.C_260038 | scaffold_26 | 44144 | 46786 |
| Block 1359 | DDB_G0270692 | 1 | 3887599 | 3890811 | estExt_fgeneshDP_pm.C_30015 | scaffold_3 | 90308 | 93420 |
| Block 1359 | arcD | 1 | 3892076 | 3893050 | fgeneshDP_pm.C_scaffold_3000015 | scaffold_3 | 87825 | 89005 |
| Block 1359 | uap56 | 1 | 3893359 | 3895841 | e_gw1.3.84.1 | scaffold_3 | 81784 | 83962 |
| Block 319 | DDB_G0269934 | 1 | 3897216 | 3903350 | fgeneshDP_pg.C_scaffold_134000023 | scaffold_134 | 65576 | 71371 |
| Block 1359 | DDB_G0270694 | 1 | 3908437 | 3910342 | GID1.0037457 | scaffold_3 | 185212 | 186541 |
| Block 1359 | DDB_G0269942 | 1 | 3911017 | 3912100 | GID1.0037464 | scaffold_3 | 206534 | 208961 |
| Block 1359 | DDB_G0269944 | 1 | 3913001 | 3914017 | GID1.0037452 | scaffold_3 | 176494 | 177505 |
| Block 1359 | DDB_G0269946 | 1 | 3915625 | 3915986 | fgeneshDP_pm.C_scaffold_3000022 | scaffold_3 | 175915 | 176335 |
| Block 1359 | tnpo | 1 | 3916816 | 3919967 | estExt_Genewise1.C_30104 | scaffold_3 | 171488 | 175136 |
| Block 1359 | serC | 1 | 3920593 | 3921886 | GID1.0037449 | scaffold_3 | 169890 | 171155 |
| Block 1359 | DDB_G0269952 | 1 | 3922020 | 3922814 | fgeneshDP_pg.C_scaffold_3000066 | scaffold_3 | 169053 | 169754 |
| Block 683 | cxeA | 1 | 3937103 | 3937960 | estExt_Genewise1Plus.C_1890005 | scaffold_189 | 10875 | 11771 |
| Block 683 | DDB_G0269964 | 1 | 3938864 | 3940930 | fgeneshDP_pm.C_scaffold_189000001 | scaffold_189 | 8536 | 10693 |
| Block 683 | ddx1 | 1 | 3941617 | 3944019 | GID1.0044832 | scaffold_189 | 15032 | 17347 |
| Block 319 | DDB_G0270698 | 1 | 3944438 | 3944996 | fgeneshDP_pg.C_scaffold_134000019 | scaffold_134 | 58761 | 59297 |
| Block 319 | DDB_G0269968 | 1 | 3945381 | 3946280 | estExt_Genewise1.C_1340039 | scaffold_134 | 59382 | 60544 |
| Block 319 | DDB_G0269970 | 1 | 3946896 | 3947753 | estExt_fgeneshDP_pg.C_1340021 | scaffold_134 | 61084 | 62008 |
| Block 1359 | DDB_G0269974 | 1 | 3955288 | 3956544 | GID1.0037478 | scaffold_3 | 256425 | 257515 |
| Block 1359 | DDB_G0269976 | 1 | 3956916 | 3959123 | GID1.0037477 | scaffold_3 | 254068 | 256141 |
| Block 1359 | DDB_G0269978 | 1 | 3960032 | 3961552 | estExt_fgeneshDP_kg.C_30009 | scaffold_3 | 251599 | 252414 |
| Block 1359 | drpp30 | 1 | 3962023 | 3963455 | e_gw1.3.99.1 | scaffold_3 | 250219 | 251447 |
| Block 1359 | DDB_G0269982 | 1 | 3972852 | 3974733 | estExt_Genewise1Plus.C_30165 | scaffold_3 | 247437 | 249720 |
| Block 3032 | DDB_G0269986 | 1 | 3977474 | 3979864 | GID1.0042093 | scaffold_95 | 47557 | 51240 |
| Block 3032 | DDB_G0271002 | 1 | 3981529 | 3984295 | GID1.0042092 | scaffold_95 | 45976 | 47453 |
| Block 1676 | tbcE | 1 | 3986134 | 3987976 | GID1.0048122 | scaffold_364 | 17998 | 20925 |
| Block 1676 | DDB_G0269994 | 1 | 3990838 | 3992071 | GID1.0048121 | scaffold_364 | 16582 | 17644 |
| Block 1079 | mekA | 1 | 4015409 | 4017699 | estExt_fgeneshDP_pg.C_2450007 | scaffold_245 | 12943 | 15444 |
| Block 1079 | jcdI | 1 | 4018580 | 4021604 | GID1.0046050 | scaffold_245 | 2999 | 5684 |
| Block 1079 | DDB_G0270008 | 1 | 4022273 | 4022909 | estExt_fgeneshDP_pg.C_2450003 | scaffold_245 | 6357 | 6921 |
| Block 1780 | DDB_G0270014 | 1 | 4026325 | 4027243 | GID1.0039728 | scaffold_39 | 86534 | 87975 |
| Block 1780 | pmpA | 1 | 4028749 | 4029268 | GID1.0039727 | scaffold_39 | 85197 | 85694 |
| Block 1842 | DDB_G0270018 | 1 | 4030543 | 4031583 | GID1.0039774 | scaffold_40 | 104010 | 105280 |
| Block 1842 | sf3a3 | 1 | 4032294 | 4034133 | e_gw1.40.39.1 | scaffold_40 | 67432 | 69105 |
| Block 1842 | mgp2 | 1 | 4042887 | 4045665 | gw1.40.36.1 | scaffold_40 | 101462 | 103757 |
| Block 1842 | gefE | 1 | 4047424 | 4051387 | estExt_Genewise1Plus.C_400068 | scaffold_40 | 93836 | 97350 |
| Block 1842 | DDB_G0270026 | 1 | 4051948 | 4052745 | estExt_Genewise1.C_400065 | scaffold_40 | 91758 | 92581 |
| Block 1842 | DDB_G0270028 | 1 | 4053112 | 4054599 | estExt_Genewise1Plus.C_400060 | scaffold_40 | 80026 | 81647 |
| Block 1842 | DDB_G0271012 | 1 | 4054671 | 4057974 | estExt_fgeneshDP_pg.C_400023 | scaffold_40 | 81644 | 83946 |
| Block 2775 | DDB_G0270034 | 1 | 4066936 | 4067804 | estExt_fgeneshDP_pg.C_7280002 | scaffold_728 | 4094 | 5015 |
| Block 2775 | ppp2r4 | 1 | 4068160 | 4069244 | fgeneshDP_pm.C_scaffold_728000003 | scaffold_728 | 5161 | 6199 |
| Block 1100 | DDB_G0270710 | 1 | 4079745 | 4080356 | gw1.25.88.1 | scaffold_25 | 69816 | 70322 |
| Block 1100 | DDB_G0271014 | 1 | 4089972 | 4092037 | gw1.25.53.1 | scaffold_25 | 59238 | 60802 |
| Block 1100 | DDB_G0270044 | 1 | 4092193 | 4093572 | GID1.0038929 | scaffold_25 | 57618 | 59075 |
| Block 268 | DDB_G0270722 | 1 | 4127506 | 4130007 | GID1.0043209 | scaffold_129 | 27807 | 30229 |
| Block 268 | DDB_G0270056 | 1 | 4130694 | 4135261 | estExt_fgeneshDP_pg.C_1290007 | scaffold_129 | 30752 | 34126 |
| Block 268 | DDB_G0270062 | 1 | 4152838 | 4157625 | e_gw1.129.2.1 | scaffold_129 | 49489 | 54232 |
| Block 1163 | DDB_G0270068 | 1 | 4175523 | 4179514 | GID1.0039020 | scaffold_26 | 117411 | 120940 |
| Block 1163 | DDB_G0270070 | 1 | 4180324 | 4181863 | estExt_fgeneshDP_pg.C_260054 | scaffold_26 | 121522 | 123327 |
| Block 1163 | DDB_G0270072 | 1 | 4182087 | 4183901 | fgeneshDP_pg.C_scaffold_26000055 | scaffold_26 | 123499 | 125268 |
| Block 1163 | DDB_G0270074 | 1 | 4187072 | 4188735 | GID1.0039024 | scaffold_26 | 126646 | 128260 |
| Block 2352 | DDB_G0270096 | 1 | 4205387 | 4207964 | fgeneshDP_pg.C_scaffold_54000006 | scaffold_54 | 13859 | 16437 |
| Block 2352 | DDB_G0270098 | 1 | 4208288 | 4210353 | estExt_fgeneshDP_kg.C_540003 | scaffold_54 | 9997 | 12241 |
| Block 2352 | DDB_G0270100 | 1 | 4212241 | 4214067 | GID1.0040400 | scaffold_54 | 815 | 2661 |
| Block 721 | mkcF | 1 | 4222376 | 4224477 | GID1.0044967 | scaffold_194 | 34202 | 36322 |
| Block 102 | DDB_G0270104 | 1 | 4225842 | 4227446 | estExt_fgeneshDP_pg.C_1090022 | scaffold_109 | 73794 | 75739 |
| Block 721 | fslB | 1 | 4235323 | 4237343 | estExt_Genewise1Plus.C_1940001 | scaffold_194 | 1021 | 3706 |
| Block 102 | DDB_G0270106 | 1 | 4238878 | 4241893 | estExt_Genewise1Plus.C_1090038 | scaffold_109 | 70020 | 73178 |
| Block 721 | dhx33 | 1 | 4244035 | 4246332 | fgeneshDP_pg.C_scaffold_194000005 | scaffold_194 | 12330 | 14732 |
| Block 1268 | DDB_G0271036 | 1 | 4262689 | 4263138 | GID1.0046763 | scaffold_280 | 21803 | 22886 |
| Block 1268 | atg6A | 1 | 4264499 | 4268846 | GID1.0046762 | scaffold_280 | 17632 | 21386 |
| Block 1101 | DDB_G0270118 | 1 | 4271883 | 4272478 | GID1.0038940 | scaffold_25 | 90681 | 91398 |
| Block 1101 | sdhB | 1 | 4273481 | 4274624 | estExt_fgeneshDP_pm.C_250016 | scaffold_25 | 95688 | 97270 |
| Block 2451 | ints2 | 1 | 4285180 | 4289651 | estExt_fgeneshDP_pg.C_5780003 | scaffold_578 | 4722 | 8791 |
| Block 1268 | DDB_G0270128 | 1 | 4289930 | 4292995 | GID1.0046761 | scaffold_280 | 14777 | 17517 |
| Block 1101 | DDB_G0270134 | 1 | 4297045 | 4297503 | GID1.0038942 | scaffold_25 | 94100 | 94613 |
| Block 2451 | rasZ | 1 | 4300316 | 4300960 | estExt_fgeneshDP_kg.C_5780002 | scaffold_578 | 2104 | 3136 |
| Block 2388 | DDB_G0270146 | 1 | 4304005 | 4305998 | fgeneshDP_pm.C_scaffold_552000002 | scaffold_552 | 2669 | 4806 |
| Block 2388 | DDB_G0270738 | 1 | 4307771 | 4308595 | fgeneshDP_pm.C_scaffold_552000001 | scaffold_552 | 1098 | 2077 |
| Block 2304 | DDB_G0270150 | 1 | 4316335 | 4319082 | estExt_fgeneshDP_pg.C_5240001 | scaffold_524 | 759 | 3713 |
| Block 2304 | maspS | 1 | 4321908 | 4323986 | GID1.0049828 | scaffold_524 | 3854 | 9864 |
| Block 2304 | DDB_G0270154 | 1 | 4327219 | 4328630 | estExt_Genewise1Plus.C_5240015 | scaffold_524 | 12350 | 13715 |
| Block 2388 | DDB_G0270156 | 1 | 4329080 | 4331994 | GID1.0050013 | scaffold_552 | 5006 | 7675 |
| Block 2388 | DDB_G0270744 | 1 | 4332329 | 4335222 | e_gw1.552.2.1 | scaffold_552 | 8025 | 10719 |
| Block 2388 | DDB_G0270746 | 1 | 4336075 | 4337602 | GID1.0050015 | scaffold_552 | 11142 | 12544 |
| Block 2388 | DDB_G0270158 | 1 | 4337917 | 4338637 | fgeneshDP_pg.C_scaffold_552000006 | scaffold_552 | 12682 | 13346 |
| Block 1161 | DDB_G0271044 | 1 | 4346844 | 4349734 | estExt_fgeneshDP_pg.C_260041 | scaffold_26 | 87593 | 90292 |
| Block 1161 | DDB_G0270750 | 1 | 4356679 | 4358223 | estExt_fgeneshDP_pg.C_260042 | scaffold_26 | 93123 | 94941 |
| Block 1161 | DDB_G0270168 | 1 | 4358663 | 4359839 | e_gw1.26.101.1 | scaffold_26 | 95238 | 96223 |
| Block 1161 | DDB_G0270170 | 1 | 4360881 | 4366105 | fgeneshDP_pg.C_scaffold_26000044 | scaffold_26 | 98486 | 102894 |
| Block 1161 | DDB_G0270172 | 1 | 4366498 | 4367403 | fgeneshDP_pm.C_scaffold_26000023 | scaffold_26 | 103268 | 104278 |
| Block 1161 | aprt | 1 | 4368158 | 4368757 | e_gw1.26.105.1 | scaffold_26 | 105087 | 105683 |
| Block 1161 | wdr85 | 1 | 4375992 | 4377095 | fgeneshDP_pg.C_scaffold_26000028 | scaffold_26 | 62403 | 63452 |
| Block 1161 | DDB_G0270180 | 1 | 4377432 | 4377910 | e_gw1.26.42.1 | scaffold_26 | 63615 | 64090 |
| Block 1161 | DDB_G0270182 | 1 | 4377987 | 4379075 | fgeneshDP_pg.C_scaffold_26000033 | scaffold_26 | 68618 | 69700 |
| Block 1161 | DDB_G0270184 | 1 | 4379266 | 4380433 | estExt_fgeneshDP_pg.C_260032 | scaffold_26 | 67204 | 68473 |
| Block 1161 | DDB_G0270186 | 1 | 4381272 | 4381856 | GID1.0038996 | scaffold_26 | 66117 | 66632 |
| Block 1161 | psmD6 | 1 | 4382128 | 4383766 | estExt_Genewise1Plus.C_260069 | scaffold_26 | 64276 | 65828 |
| Block 1161 | DDB_G0270190 | 1 | 4384621 | 4386448 | GID1.0038999 | scaffold_26 | 70161 | 72163 |
| Block 1161 | DDB_G0270192 | 1 | 4389266 | 4390889 | gw1.26.78.1 | scaffold_26 | 74648 | 75920 |
| Block 1161 | DDB_G0270194 | 1 | 4391158 | 4391944 | GID1.0039003 | scaffold_26 | 79460 | 80108 |
| Block 1161 | DDB_G0270752 | 1 | 4392163 | 4393069 | fgeneshDP_pg.C_scaffold_26000037 | scaffold_26 | 80551 | 81401 |
| Block 1161 | DDB_G0270196 | 1 | 4393614 | 4395336 | fgeneshDP_pg.C_scaffold_26000038 | scaffold_26 | 82004 | 83904 |
| Block 1161 | DDB_G0270200 | 1 | 4397167 | 4399546 | estExt_fgeneshDP_pg.C_260040 | scaffold_26 | 85688 | 87501 |
| Block 1161 | DDB_G0270202 | 1 | 4400104 | 4400817 | GID1.0039006 | scaffold_26 | 84125 | 84950 |
| Block 1161 | DDB_G0270208 | 1 | 4413499 | 4414682 | GID1.0038992 | scaffold_26 | 61183 | 62251 |
| Block 3034 | DDB_G0270216 | 1 | 4420069 | 4423204 | GID1.0042106 | scaffold_95 | 75379 | 78136 |
| Block 3034 | vps16 | 1 | 4423807 | 4426401 | e_gw1.95.1.1 | scaffold_95 | 78522 | 81174 |
| Block 3034 | DDB_G0270220 | 1 | 4432689 | 4435367 | estExt_fgeneshDP_pg.C_950026 | scaffold_95 | 69098 | 71960 |
| Block 1156 | utp11 | 1 | 4445249 | 4446106 | GID1.0038966 | scaffold_26 | 4722 | 5563 |
| Block 1156 | gtaG | 1 | 4446395 | 4449415 | estExt_fgeneshDP_pg.C_260001 | scaffold_26 | 1999 | 4422 |
| Block 367 | DDB_G0270238 | 1 | 4474835 | 4479049 | fgeneshDP_pg.C_scaffold_140000008 | scaffold_140 | 16413 | 20080 |
| Block 367 | magA | 1 | 4479608 | 4480480 | gw1.140.4.1 | scaffold_140 | 15337 | 15888 |
| Block 367 | DDB_G0270242 | 1 | 4484790 | 4485548 | fgeneshDP_pm.C_scaffold_140000003 | scaffold_140 | 13472 | 14307 |
| Block 150 | DDB_G0271052 | 1 | 4502537 | 4512340 | estExt_Genewise1Plus.C_1160004 | scaffold_116 | 4449 | 8835 |
| Block 150 | DDB_G0270246 | 1 | 4514326 | 4516297 | gw1.116.18.1 | scaffold_116 | 15421 | 16995 |
| Block 150 | DDB_G0270248 | 1 | 4516591 | 4517262 | gw1.116.32.1 | scaffold_116 | 14522 | 14851 |
| Block 150 | DDB_G0270250 | 1 | 4517624 | 4519199 | GID1.0042787 | scaffold_116 | 27460 | 28596 |
| Block 150 | DDB_G0270252 | 1 | 4519307 | 4521349 | GID1.0042788 | scaffold_116 | 28851 | 31008 |
| Block 150 | DDB_G0270254 | 1 | 4521755 | 4522105 | fgeneshDP_pm.C_scaffold_116000002 | scaffold_116 | 31519 | 31869 |
| Block 150 | DDB_G0270256 | 1 | 4522306 | 4522903 | GID1.0042779 | scaffold_116 | 1340 | 2286 |
| Block 150 | DDB_G0270258 | 1 | 4523481 | 4524638 | fgeneshDP_pg.C_scaffold_116000001 | scaffold_116 | 138 | 977 |
| Block 2577 | nhp6 | 1 | 4527247 | 4527672 | fgeneshDP_kg.C_scaffold_625000001 | scaffold_625 | 1042 | 1562 |
| Block 2577 | DDB_G0270262 | 1 | 4528758 | 4529451 | e_gw1.625.10.1 | scaffold_625 | 2162 | 2822 |
| Block 2577 | fszB | 1 | 4529920 | 4531250 | e_gw1.625.6.1 | scaffold_625 | 3301 | 4670 |
| Block 2577 | DDB_G0270264 | 1 | 4531797 | 4533209 | e_gw1.625.5.1 | scaffold_625 | 7630 | 9042 |
| Block 1102 | DDB_G0270268 | 1 | 4540670 | 4542577 | fgeneshDP_pg.C_scaffold_25000059 | scaffold_25 | 138720 | 140376 |
| Block 1102 | gpsn2 | 1 | 4542640 | 4543788 | estExt_Genewise1Plus.C_250121 | scaffold_25 | 137380 | 138642 |
| Block 1102 | uae1 | 1 | 4544769 | 4548249 | estExt_fgeneshDP_pg.C_250057 | scaffold_25 | 132602 | 136067 |
| Block 1102 | abpC | 1 | 4554019 | 4556699 | estExt_fgeneshDP_kg.C_250012 | scaffold_25 | 99951 | 112442 |
| Block 1102 | DDB_G0270278 | 1 | 4558267 | 4561757 | GID1.0038960 | scaffold_25 | 129164 | 132570 |
| Block 1098 | sgmA | 1 | 4562741 | 4564611 | estExt_Genewise1Plus.C_250018 | scaffold_25 | 24958 | 27110 |
| Block 1098 | DDB_G0271054 | 1 | 4565256 | 4567067 | GID1.0038916 | scaffold_25 | 27695 | 29489 |
| Block 2218 | DDB_G0270284 | 1 | 4579488 | 4580763 | estExt_fgeneshDP_kg.C_50020 | scaffold_5 | 202531 | 203811 |
| Block 2218 | dcd1A | 1 | 4598954 | 4600454 | GID1.0037640 | scaffold_5 | 200661 | 202015 |
| Block 2218 | pctA | 1 | 4600795 | 4602080 | estExt_fgeneshDP_pg.C_50056 | scaffold_5 | 199060 | 200312 |
| Block 3027 | DDB_G0270304 | 1 | 4607306 | 4608218 | e_gw1.95.33.1 | scaffold_95 | 4498 | 5170 |
| Block 3027 | DDB_G0270306 | 1 | 4610085 | 4611533 | fgeneshDP_pg.C_scaffold_95000003 | scaffold_95 | 6699 | 8036 |
| Block 1270 | rpsA | 1 | 4628419 | 4629156 | estExt_fgeneshDP_kg.C_2800009 | scaffold_280 | 42973 | 43739 |
| Block 1270 | DDB_G0269162 | 1 | 4629764 | 4632502 | estExt_fgeneshDP_pm.C_2800014 | scaffold_280 | 38173 | 40874 |
| Block 1270 | DDB_G0271068 | 1 | 4633487 | 4634149 | estExt_Genewise1Plus.C_2800029 | scaffold_280 | 41557 | 42577 |
| Block 1270 | DDB_G0271070 | 1 | 4634981 | 4635241 | gw1.280.30.1 | scaffold_280 | 37745 | 37969 |
| Block 1159 | med27 | 1 | 4635594 | 4637043 | estExt_fgeneshDP_pg.C_260026 | scaffold_26 | 59499 | 61088 |
| Block 1159 | nup54 | 1 | 4639723 | 4641353 | estExt_Genewise1Plus.C_260016 | scaffold_26 | 25572 | 27006 |
| Block 1080 | DDB_G0270322 | 1 | 4641717 | 4643879 | estExt_fgeneshDP_pg.C_2450008 | scaffold_245 | 15769 | 18026 |
| Block 1080 | DDB_G0270324 | 1 | 4644690 | 4646184 | estExt_Genewise1Plus.C_2450012 | scaffold_245 | 18948 | 20472 |
| Block 1080 | DDB_G0270776 | 1 | 4646538 | 4649118 | GID1.0046058 | scaffold_245 | 20531 | 22948 |
| Block 1358 | ipo4 | 1 | 4662405 | 4666147 | GID1.0037407 | scaffold_3 | 77434 | 81007 |
| Block 1358 | DDB_G0270338 | 1 | 4667184 | 4668853 | fgeneshDP_pm.C_scaffold_3000019 | scaffold_3 | 122139 | 124501 |
| Block 1080 | DDB_G0270344 | 1 | 4674130 | 4678854 | estExt_fgeneshDP_pm.C_2450009 | scaffold_245 | 25533 | 29846 |
| Block 1080 | psmC1 | 1 | 4684536 | 4685855 | estExt_fgeneshDP_pg.C_2450011 | scaffold_245 | 23723 | 25351 |
| Block 1358 | DDB_G0270786 | 1 | 4686330 | 4688375 | e_gw1.3.69.1 | scaffold_3 | 95276 | 97143 |
| Block 1084 | DDB_G0270352 | 1 | 4693514 | 4695492 | estExt_Genewise1.C_2460015 | scaffold_246 | 35401 | 37784 |
| Block 1084 | erg2 | 1 | 4699501 | 4700297 | GID1.0046080 | scaffold_246 | 37894 | 38592 |
| Block 1084 | DDB_G0270358 | 1 | 4701032 | 4702819 | fgeneshDP_pm.C_scaffold_246000007 | scaffold_246 | 39331 | 40968 |
| Block 912 | DDB_G0270362 | 1 | 4713592 | 4714219 | estExt_Genewise1Plus.C_2190002 | scaffold_219 | 6039 | 6834 |
| Block 912 | DDB_G0270378 | 1 | 4727960 | 4729831 | fgeneshDP_pg.C_scaffold_219000005 | scaffold_219 | 8135 | 9885 |
| Block 744 | DDB_G0270382 | 1 | 4732515 | 4733489 | estExt_fgeneshDP_kg.C_1980002 | scaffold_198 | 18413 | 19447 |
| Block 744 | DDB_G0270384 | 1 | 4733677 | 4734627 | fgeneshDP_pm.C_scaffold_198000004 | scaffold_198 | 14872 | 15738 |
| Block 744 | DDB_G0270386 | 1 | 4735593 | 4737533 | estExt_Genewise1Plus.C_1980011 | scaffold_198 | 12113 | 13945 |
| Block 1966 | DDB_G0270388 | 1 | 4737656 | 4740668 | GID1.0048981 | scaffold_431 | 11055 | 13980 |
| Block 744 | cpsf2 | 1 | 4749183 | 4751699 | fgeneshDP_pg.C_scaffold_198000005 | scaffold_198 | 8482 | 10889 |
| Block 744 | DDB_G0270394 | 1 | 4751920 | 4752333 | GID1.0045038 | scaffold_198 | 7937 | 8332 |
| Block 744 | ddx49 | 1 | 4752654 | 4754329 | GID1.0045037 | scaffold_198 | 6037 | 7700 |
| Block 744 | cplA | 1 | 4755270 | 4757427 | e_gw1.198.20.1 | scaffold_198 | 3736 | 5886 |
| Block 1165 | raptor | 1 | 4758927 | 4763559 | GID1.0039027 | scaffold_26 | 132463 | 136884 |
| Block 1165 | orcC | 1 | 4764421 | 4768313 | estExt_fgeneshDP_pg.C_260061 | scaffold_26 | 137862 | 141807 |
| Block 1966 | lkhA | 1 | 4768681 | 4771055 | estExt_Genewise1.C_4310003 | scaffold_431 | 3898 | 6113 |
| Block 461 | wimA | 1 | 4776808 | 4782904 | estExt_fgeneshDP_pg.C_1520016 | scaffold_152 | 45026 | 50761 |
| Block 461 | DDB_G0270402 | 1 | 4784022 | 4784931 | fgeneshDP_pm.C_scaffold_152000009 | scaffold_152 | 42983 | 43846 |
| Block 461 | DDB_G0270404 | 1 | 4785053 | 4791836 | e_gw1.152.15.1 | scaffold_152 | 36458 | 42787 |
| Block 461 | rzpA | 1 | 4793277 | 4794092 | estExt_fgeneshDP_kg.C_1520005 | scaffold_152 | 34496 | 35441 |
| Block 461 | eIF4e3 | 1 | 4795840 | 4798577 | estExt_fgeneshDP_pg.C_1520012 | scaffold_152 | 30968 | 33257 |
| Block 1966 | DDB_G0270806 | 1 | 4800145 | 4802005 | GID1.0048980 | scaffold_431 | 8798 | 10603 |
| Block 1966 | scy2 | 1 | 4802064 | 4805588 | GID1.0048977 | scaffold_431 | 171 | 3406 |
| Block 461 | DDB_G0270412 | 1 | 4819722 | 4821704 | GID1.0043884 | scaffold_152 | 17202 | 19267 |
| Block 2450 | DDB_G0270416 | 1 | 4823455 | 4826412 | GID1.0050178 | scaffold_578 | 12871 | 13879 |
| Block 461 | top2 | 1 | 4831688 | 4836353 | gw1.152.2.1 | scaffold_152 | 52802 | 56484 |
| Block 461 | DDB_G0270420 | 1 | 4840357 | 4841439 | estExt_Genewise1Plus.C_1520019 | scaffold_152 | 28721 | 30014 |
| Block 461 | DDB_G0270422 | 1 | 4842234 | 4844540 | estExt_Genewise1.C_1520017 | scaffold_152 | 25643 | 28351 |
| Block 2450 | H3b | 1 | 4846006 | 4846565 | estExt_fgeneshDP_kg.C_5780001 | scaffold_578 | 1116 | 1669 |
| Block 1776 | rpl35a | 1 | 4849685 | 4850344 | estExt_Genewise1Plus.C_390071 | scaffold_39 | 71391 | 72052 |
| Block 1776 | DDB_G0270426 | 1 | 4851386 | 4851959 | e_gw1.39.86.1 | scaffold_39 | 69041 | 69310 |
| Block 1776 | DDB_G0270428 | 1 | 4852185 | 4852947 | e_gw1.39.80.1 | scaffold_39 | 67849 | 68705 |
| Block 1776 | snpC | 1 | 4853471 | 4854871 | estExt_Genewise1.C_390065 | scaffold_39 | 65970 | 67444 |
| Block 1776 | wipA | 1 | 4856092 | 4856685 | fgeneshDP_pg.C_scaffold_39000028 | scaffold_39 | 65253 | 65849 |
| Block 1776 | DDB_G0270432 | 1 | 4857327 | 4861922 | fgeneshDP_pg.C_scaffold_39000027 | scaffold_39 | 61685 | 64422 |
| Block 1792 | npcA | 1 | 4862383 | 4866613 | GID1.0048512 | scaffold_392 | 14307 | 18466 |
| Block 1792 | med23 | 1 | 4866950 | 4872568 | GID1.0048511 | scaffold_392 | 8606 | 13940 |
| Block 1792 | DDB_G0270436 | 1 | 4873137 | 4875220 | e_gw1.392.6.1 | scaffold_392 | 4190 | 7890 |
| Block 942 | DDB_G0270816 | 1 | 4879000 | 4881006 | GID1.0045643 | scaffold_224 | 17312 | 18862 |
| Block 942 | DDB_G0270438 | 1 | 4881217 | 4881761 | fgeneshDP_pg.C_scaffold_224000010 | scaffold_224 | 19386 | 19898 |
| Block 942 | DDB_G0270440 | 1 | 4882133 | 4882733 | e_gw1.224.29.1 | scaffold_224 | 20329 | 20916 |
| Block 942 | polA3 | 1 | 4882826 | 4884472 | fgeneshDP_pg.C_scaffold_224000015 | scaffold_224 | 28764 | 30351 |
| Block 942 | tcp1 | 1 | 4885046 | 4886981 | estExt_Genewise1.C_2240020 | scaffold_224 | 25878 | 28152 |
| Block 942 | numA | 1 | 4887451 | 4889926 | estExt_fgeneshDP_pg.C_2240013 | scaffold_224 | 22756 | 25571 |
| Block 745 | DDB_G0270444 | 1 | 4890352 | 4894529 | GID1.0045046 | scaffold_198 | 19864 | 23964 |
| Block 745 | DDB_G0270818 | 1 | 4894629 | 4895040 | e_gw1.198.37.1 | scaffold_198 | 45948 | 46395 |
| Block 745 | DDB_G0271088 | 1 | 4895305 | 4899887 | estExt_fgeneshDP_pg.C_1980018 | scaffold_198 | 43417 | 45673 |
| Block 1162 | DDB_G0270446 | 1 | 4899926 | 4903011 | estExt_fgeneshDP_pg.C_260051 | scaffold_26 | 112332 | 115140 |
| Block 1162 | DDB_G0270448 | 1 | 4903242 | 4904821 | GID1.0039017 | scaffold_26 | 110705 | 112121 |
| Block 1162 | DDB_G0270450 | 1 | 4904923 | 4906041 | estExt_fgeneshDP_kg.C_260009 | scaffold_26 | 108552 | 109784 |
| Block 1162 | DDB_G0270452 | 1 | 4906213 | 4908821 | GID1.0039014 | scaffold_26 | 105766 | 108019 |
| Block 2128 | DDB_G0271114 | 2 | 358 | 1851 | fgeneshDP_pm.C_scaffold_475000001 | scaffold_475 | 2240 | 3439 |
| Block 2128 | DDB_G0271112 | 2 | 2575 | 4734 | GID1.0049415 | scaffold_475 | 3707 | 5972 |
| Block 1410 | atg7 | 2 | 6776 | 9169 | GID1.0047218 | scaffold_306 | 28897 | 31141 |
| Block 1410 | DDB_G0271108 | 2 | 15241 | 16379 | e_gw1.306.9.1 | scaffold_306 | 27098 | 28195 |
| Block 1410 | DDB_G0271120 | 2 | 17121 | 19402 | estExt_Genewise1Plus.C_3060007 | scaffold_306 | 24954 | 26833 |
| Block 2309 | ufd1 | 2 | 19983 | 21203 | estExt_Genewise1.C_5260013 | scaffold_526 | 12497 | 13714 |
| Block 2309 | sod2 | 2 | 21524 | 22687 | estExt_Genewise1.C_5260016 | scaffold_526 | 14126 | 15575 |
| Block 2128 | DDB_G0271124 | 2 | 23582 | 25531 | fgeneshDP_pg.C_scaffold_475000009 | scaffold_475 | 19385 | 21190 |
| Block 2309 | DDB_G0271130 | 2 | 31518 | 33840 | fgeneshDP_pm.C_scaffold_526000005 | scaffold_526 | 10079 | 12177 |
| Block 390 | DDB_G0271098 | 2 | 36177 | 36831 | GID1.0043669 | scaffold_144 | 7898 | 8474 |
| Block 390 | DDB_G0271350 | 2 | 40707 | 41744 | estExt_Genewise1Plus.C_1440009 | scaffold_144 | 8865 | 10093 |
| Block 2309 | DDB_G0271354 | 2 | 42708 | 43472 | estExt_Genewise1Plus.C_5260010 | scaffold_526 | 5699 | 6628 |
| Block 2309 | proC | 2 | 44014 | 44394 | fgeneshDP_pm.C_scaffold_526000003 | scaffold_526 | 4236 | 4616 |
| Block 1410 | celA | 2 | 45111 | 47321 | e_gw1.306.8.1 | scaffold_306 | 8210 | 9511 |
| Block 784 | yod1 | 2 | 52705 | 54054 | fgeneshDP_pm.C_scaffold_200000006 | scaffold_200 | 24696 | 25855 |
| Block 784 | idhA | 2 | 54631 | 55952 | fgeneshDP_pg.C_scaffold_200000010 | scaffold_200 | 22938 | 24084 |
| Block 784 | udkD | 2 | 56107 | 57781 | estExt_fgeneshDP_pg.C_2000007 | scaffold_200 | 16471 | 18196 |
| Block 784 | DDB_G0271356 | 2 | 58689 | 59390 | e_gw1.200.36.1 | scaffold_200 | 15098 | 15958 |
| Block 784 | DDB_G0271342 | 2 | 59610 | 62009 | GID1.0045098 | scaffold_200 | 12956 | 14921 |
| Block 784 | DDB_G0271340 | 2 | 62953 | 65314 | estExt_Genewise1.C_2000001 | scaffold_200 | 5922 | 8479 |
| Block 2187 | DDB_G0271358 | 2 | 65908 | 66540 | fgeneshDP_pg.C_scaffold_49000032 | scaffold_49 | 74173 | 74835 |
| Block 2187 | taf7 | 2 | 67349 | 69445 | GID1.0040199 | scaffold_49 | 71555 | 73807 |
| Block 1410 | DDB_G0271334 | 2 | 74400 | 79073 | GID1.0047215 | scaffold_306 | 19531 | 24178 |
| Block 1937 | DDB_G0271324 | 2 | 90704 | 91899 | GID1.0048907 | scaffold_423 | 17263 | 19064 |
| Block 1937 | DDB_G0271366 | 2 | 97610 | 98221 | e_gw1.423.16.1 | scaffold_423 | 16289 | 16985 |
| Block 2882 | DDB_G0271372 | 2 | 104136 | 107135 | estExt_fgeneshDP_pm.C_80009 | scaffold_8 | 82938 | 85887 |
| Block 2882 | DDB_G0271316 | 2 | 107224 | 108084 | GID1.0037860 | scaffold_8 | 85900 | 86701 |
| Block 2882 | sf3b5 | 2 | 111021 | 111494 | fgeneshDP_pm.C_scaffold_8000012 | scaffold_8 | 89484 | 89905 |
| Block 2882 | ucpA | 2 | 112166 | 113349 | e_gw1.8.85.1 | scaffold_8 | 90140 | 91301 |
| Block 2875 | DDB_G0271308 | 2 | 113519 | 116100 | estExt_fgeneshDP_pg.C_80008 | scaffold_8 | 20264 | 22556 |
| Block 2875 | DDB_G0271304 | 2 | 118603 | 119523 | estExt_fgeneshDP_pg.C_80009 | scaffold_8 | 22593 | 23704 |
| Block 2187 | DDB_G0271374 | 2 | 121436 | 122831 | estExt_fgeneshDP_kg.C_490001 | scaffold_49 | 3553 | 4995 |
| Block 2187 | DDB_G0271302 | 2 | 122908 | 125355 | estExt_Genewise1Plus.C_490003 | scaffold_49 | 5014 | 7553 |
| Block 2187 | rpl27 | 2 | 126855 | 127699 | e_gw1.49.23.1 | scaffold_49 | 8998 | 9423 |
| Block 2187 | DDB_G0271296 | 2 | 128177 | 130259 | e_gw1.49.35.1 | scaffold_49 | 10609 | 12699 |
| Block 2187 | DDB_G0271294 | 2 | 130353 | 130938 | e_gw1.49.78.1 | scaffold_49 | 12786 | 13398 |
| Block 1558 | DDB_G0271292 | 2 | 132410 | 133183 | GID1.0039434 | scaffold_34 | 26165 | 26926 |
| Block 1558 | DDB_G0271290 | 2 | 135911 | 137123 | fgeneshDP_pg.C_scaffold_34000025 | scaffold_34 | 54532 | 55671 |
| Block 1558 | DDB_G0271288 | 2 | 140491 | 141852 | GID1.0039425 | scaffold_34 | 6012 | 7446 |
| Block 1558 | clasp | 2 | 141951 | 145023 | fgeneshDP_pg.C_scaffold_34000004 | scaffold_34 | 7645 | 10543 |
| Block 1558 | CSN7 | 2 | 147410 | 148472 | GID1.0039427 | scaffold_34 | 10789 | 11975 |
| Block 1558 | DDB_G0271280 | 2 | 149313 | 151154 | fgeneshDP_pg.C_scaffold_34000007 | scaffold_34 | 14133 | 15779 |
| Block 2665 | DDB_G0271384 | 2 | 154665 | 157887 | e_gw1.66.26.1 | scaffold_66 | 88802 | 91562 |
| Block 2665 | DDB_G0271268 | 2 | 167706 | 168266 | GID1.0040964 | scaffold_66 | 92299 | 92814 |
| Block 2665 | usp14 | 2 | 170091 | 171904 | estExt_Genewise1Plus.C_660046 | scaffold_66 | 86725 | 88620 |
| Block 1558 | cog8 | 2 | 186286 | 189289 | GID1.0039450 | scaffold_34 | 56032 | 58488 |
| Block 1644 | DDB_G0271394 | 2 | 197833 | 199366 | fgeneshDP_pg.C_scaffold_358000001 | scaffold_358 | 1409 | 1876 |
| Block 1644 | DDB_G0271252 | 2 | 199744 | 200013 | GID1.0048029 | scaffold_358 | 130 | 312 |
| Block 1558 | DDB_G0271250 | 2 | 201416 | 202998 | GID1.0039433 | scaffold_34 | 23777 | 25219 |
| Block 1558 | cnxA | 2 | 203269 | 204879 | estExt_fgeneshDP_kg.C_340002 | scaffold_34 | 22061 | 22848 |
| Block 1558 | mon2 | 2 | 205725 | 211675 | estExt_fgeneshDP_pm.C_340005 | scaffold_34 | 15920 | 21443 |
| Block 1558 | DDB_G0271248 | 2 | 212993 | 214344 | fgeneshDP_pg.C_scaffold_34000049 | scaffold_34 | 126380 | 127636 |
| Block 1558 | DDB_G0271400 | 2 | 214423 | 215721 | e_gw1.34.64.1 | scaffold_34 | 127758 | 129032 |
| Block 1558 | med19 | 2 | 215936 | 216913 | estExt_fgeneshDP_pg.C_340016 | scaffold_34 | 34773 | 35509 |
| Block 2444 | DDB_G0271242 | 2 | 218788 | 220665 | fgeneshDP_pg.C_scaffold_575000002 | scaffold_575 | 3638 | 5279 |
| Block 2444 | utp18 | 2 | 221177 | 222968 | GID1.0050154 | scaffold_575 | 1638 | 3230 |
| Block 1558 | vta1 | 2 | 417030 | 418844 | GID1.0039441 | scaffold_34 | 36786 | 38713 |
| Block 1558 | clcA | 2 | 422428 | 425089 | estExt_fgeneshDP_pm.C_340012 | scaffold_34 | 46083 | 49355 |
| Block 2130 | pitrm1 | 2 | 441829 | 445192 | GID1.0049417 | scaffold_475 | 9096 | 12470 |
| Block 2130 | DDB_G0271510 | 2 | 451142 | 453520 | GID1.0049418 | scaffold_475 | 12875 | 15272 |
| Block 2130 | DDB_G0271512 | 2 | 454931 | 455947 | e_gw1.475.13.1 | scaffold_475 | 17230 | 18156 |
| Block 1558 | rpc5 | 2 | 456027 | 458103 | GID1.0039466 | scaffold_34 | 93381 | 95400 |
| Block 1558 | polZ | 2 | 466708 | 474903 | GID1.0039461 | scaffold_34 | 77925 | 84999 |
| Block 1558 | DDB_G0271532 | 2 | 536858 | 538818 | GID1.0039456 | scaffold_34 | 72130 | 74032 |
| Block 282 | DDB_G0271538 | 2 | 555084 | 556837 | GID1.0043232 | scaffold_130 | 4509 | 6090 |
| Block 282 | dlpC | 2 | 558697 | 561529 | e_gw1.130.20.1 | scaffold_130 | 7613 | 10296 |
| Block 1846 | sac1 | 2 | 561917 | 563662 | estExt_Genewise1.C_4000023 | scaffold_400 | 25925 | 27741 |
| Block 1846 | DDB_G0271570 | 2 | 563763 | 564899 | estExt_fgeneshDP_kg.C_4000003 | scaffold_400 | 27794 | 28624 |
| Block 1846 | DDB_G0271544 | 2 | 592310 | 593826 | e_gw1.400.7.1 | scaffold_400 | 5143 | 6342 |
| Block 1846 | DG1106 | 2 | 594743 | 598366 | fgeneshDP_pm.C_scaffold_400000004 | scaffold_400 | 7383 | 10442 |
| Block 1846 | cks1 | 2 | 600662 | 601206 | e_gw1.400.16.1 | scaffold_400 | 16161 | 16912 |
| Block 1846 | DDB_G0271546 | 2 | 606700 | 608144 | GID1.0048620 | scaffold_400 | 20857 | 22211 |
| Block 1053 | lvsD | 2 | 608638 | 617250 | GID1.0045955 | scaffold_240 | 3014 | 11668 |
| Block 1053 | DDB_G0271550 | 2 | 620894 | 622453 | e_gw1.240.24.1 | scaffold_240 | 13543 | 14792 |
| Block 1053 | DDB_G0271552 | 2 | 623594 | 626007 | fgeneshDP_pm.C_scaffold_240000002 | scaffold_240 | 15846 | 18322 |
| Block 1663 | orcD | 2 | 668167 | 669724 | e_gw1.36.65.1 | scaffold_36 | 111562 | 113059 |
| Block 1663 | pdhX | 2 | 669848 | 671089 | gw1.36.16.1 | scaffold_36 | 110307 | 111416 |
| Block 1663 | DDB_G0271786 | 2 | 698866 | 702452 | estExt_fgeneshDP_pg.C_360050 | scaffold_36 | 122638 | 126502 |
| Block 336 | wdr57 | 2 | 702957 | 704143 | GID1.0043467 | scaffold_137 | 45546 | 46706 |
| Block 336 | rabC | 2 | 704978 | 706000 | estExt_Genewise1.C_1370035 | scaffold_137 | 43459 | 44856 |
| Block 336 | DDB_G0271750 | 2 | 707319 | 710524 | fgeneshDP_pg.C_scaffold_137000018 | scaffold_137 | 39611 | 42696 |
| Block 1663 | tubC | 2 | 713462 | 715042 | fgeneshDP_pg.C_scaffold_36000045 | scaffold_36 | 113264 | 114710 |
| Block 1663 | DDB_G0271790 | 2 | 715154 | 715652 | fgeneshDP_pg.C_scaffold_36000049 | scaffold_36 | 122120 | 122597 |
| Block 1845 | arrC | 2 | 716247 | 716981 | e_gw1.400.18.1 | scaffold_400 | 1857 | 2487 |
| Block 1845 | DDB_G0271700 | 2 | 717208 | 718107 | estExt_fgeneshDP_kg.C_4000001 | scaffold_400 | 2610 | 3810 |
| Block 1845 | rpl36 | 2 | 719655 | 720235 | estExt_Genewise1.C_4000017 | scaffold_400 | 17691 | 18285 |
| Block 1525 | DDB_G0271754 | 2 | 729820 | 733274 | estExt_fgeneshDP_pg.C_330031 | scaffold_33 | 104175 | 106752 |
| Block 1845 | captA | 2 | 733878 | 735319 | fgeneshDP_pg.C_scaffold_400000009 | scaffold_400 | 18760 | 20144 |
| Block 1525 | DDB_G0271694 | 2 | 736775 | 739025 | GID1.0039418 | scaffold_33 | 117019 | 119568 |
| Block 1525 | DDB_G0271796 | 2 | 739786 | 740826 | e_gw1.33.48.1 | scaffold_33 | 120244 | 121277 |
| Block 1525 | DDB_G0271798 | 2 | 741606 | 744680 | fgeneshDP_pm.C_scaffold_33000017 | scaffold_33 | 122101 | 124405 |
| Block 1317 | comC | 2 | 744793 | 749389 | e_gw1.290.1.1 | scaffold_290 | 3635 | 13289 |
| Block 170 | DDB_G0271690 | 2 | 750002 | 752612 | estExt_Genewise1Plus.C_1180057 | scaffold_118 | 71495 | 73910 |
| Block 170 | DDB_G0271760 | 2 | 757446 | 758572 | estExt_fgeneshDP_pm.C_1180014 | scaffold_118 | 59314 | 60966 |
| Block 170 | grlB | 2 | 762749 | 765092 | e_gw1.118.17.1 | scaffold_118 | 68740 | 71078 |
| Block 836 | DDB_G0271806 | 2 | 773875 | 778107 | estExt_fgeneshDP_pg.C_2080001 | scaffold_208 | 622 | 4840 |
| Block 836 | DDB_G0271682 | 2 | 778984 | 782463 | gw1.208.5.1 | scaffold_208 | 29683 | 30591 |
| Block 1317 | DDB_G0271680 | 2 | 783129 | 784413 | e_gw1.290.25.1 | scaffold_290 | 32566 | 33731 |
| Block 170 | pckA | 2 | 796359 | 798202 | estExt_Genewise1.C_1180017 | scaffold_118 | 14352 | 16574 |
| Block 170 | DDB_G0271676 | 2 | 799169 | 800770 | estExt_fgeneshDP_kg.C_1180002 | scaffold_118 | 11603 | 13904 |
| Block 1317 | DDB_G0271814 | 2 | 802171 | 803454 | GID1.0046947 | scaffold_290 | 28536 | 29523 |
| Block 790 | tbcA | 2 | 815968 | 816496 | fgeneshDP_pg.C_scaffold_200000021 | scaffold_200 | 48233 | 48758 |
| Block 790 | ddx31 | 2 | 816798 | 819524 | GID1.0045114 | scaffold_200 | 45279 | 48026 |
| Block 594 | DDB_G0271828 | 2 | 839485 | 842046 | GID1.0044444 | scaffold_172 | 47174 | 48277 |
| Block 2348 | DDB_G0271724 | 2 | 843563 | 844538 | fgeneshDP_pm.C_scaffold_538000004 | scaffold_538 | 9606 | 10533 |
| Block 2348 | DDB_G0271726 | 2 | 845080 | 845979 | estExt_Genewise1Plus.C_5380011 | scaffold_538 | 7385 | 8440 |
| Block 170 | DDB_G0271728 | 2 | 852985 | 853488 | GID1.0042860 | scaffold_118 | 32800 | 33322 |
| Block 170 | DDB_G0271730 | 2 | 854663 | 858640 | e_gw1.118.60.1 | scaffold_118 | 35330 | 38820 |
| Block 594 | DDB_G0271732 | 2 | 858789 | 861863 | estExt_fgeneshDP_pg.C_1720017 | scaffold_172 | 43921 | 47070 |
| Block 594 | DDB_G0271830 | 2 | 862780 | 864014 | fgeneshDP_pm.C_scaffold_172000006 | scaffold_172 | 41943 | 43055 |
| Block 170 | DDB_G0271832 | 2 | 873673 | 876190 | fgeneshDP_pg.C_scaffold_118000010 | scaffold_118 | 31017 | 32653 |
| Block 170 | uqcrh | 2 | 876949 | 878329 | GID1.0042858 | scaffold_118 | 28665 | 30281 |
| Block 170 | CYP516B1 | 2 | 881685 | 883368 | estExt_fgeneshDP_kg.C_1180004 | scaffold_118 | 22752 | 25550 |
| Block 2080 | DDB_G0271838 | 2 | 891434 | 895692 | fgeneshDP_pg.C_scaffold_460000002 | scaffold_460 | 8240 | 12186 |
| Block 2080 | DDB_G0271840 | 2 | 896024 | 897020 | fgeneshDP_pg.C_scaffold_460000003 | scaffold_460 | 12536 | 13148 |
| Block 2080 | DDB_G0271742 | 2 | 897362 | 898012 | e_gw1.460.8.1 | scaffold_460 | 16360 | 17010 |
| Block 2080 | scsC | 2 | 899061 | 900513 | GID1.0049272 | scaffold_460 | 13987 | 15464 |
| Block 2622 | DDB_G0271880 | 2 | 917079 | 919079 | GID1.0050526 | scaffold_642 | 6837 | 9886 |
| Block 2622 | mpgB | 2 | 919284 | 920692 | GID1.0050525 | scaffold_642 | 5232 | 6608 |
| Block 2622 | DDB_G0271860 | 2 | 926120 | 927802 | estExt_fgeneshDP_pg.C_6420001 | scaffold_642 | 303 | 3275 |
| Block 2622 | DDB_G0271882 | 2 | 928514 | 929249 | estExt_Genewise1.C_6420006 | scaffold_642 | 3361 | 4490 |
| Block 2080 | gefS | 2 | 947382 | 951071 | estExt_Genewise1Plus.C_4600009 | scaffold_460 | 17430 | 20899 |
| Block 1480 | DDB_G0271866 | 2 | 967310 | 968209 | estExt_Genewise1Plus.C_3200031 | scaffold_320 | 33041 | 34047 |
| Block 1480 | DDB_G0271886 | 2 | 968356 | 971478 | estExt_Genewise1Plus.C_3200030 | scaffold_320 | 30714 | 32892 |
| Block 1871 | DDB_G0271944 | 2 | 1002773 | 1004402 | e_gw1.409.2.1 | scaffold_409 | 12566 | 13907 |
| Block 1871 | DDB_G0271910 | 2 | 1004624 | 1005573 | estExt_fgeneshDP_pg.C_4090006 | scaffold_409 | 10986 | 12011 |
| Block 1871 | arl5 | 2 | 1005812 | 1006902 | estExt_fgeneshDP_kg.C_4090002 | scaffold_409 | 9762 | 10909 |
| Block 37 | DDB_G0271956 | 2 | 1007341 | 1010327 | GID1.0042282 | scaffold_101 | 18466 | 20867 |
| Block 37 | mrrA | 2 | 1011163 | 1011888 | estExt_fgeneshDP_kg.C_1010001 | scaffold_101 | 21589 | 22569 |
| Block 169 | DDB_G0271890 | 2 | 1019896 | 1021032 | fgeneshDP_pg.C_scaffold_118000018 | scaffold_118 | 49094 | 50170 |
| Block 169 | DDB_G0271892 | 2 | 1021494 | 1022350 | estExt_fgeneshDP_kg.C_1180006 | scaffold_118 | 47795 | 48822 |
| Block 524 | DDB_G0271894 | 2 | 1023653 | 1025824 | estExt_Genewise1Plus.C_1620037 | scaffold_162 | 60705 | 64369 |
| Block 169 | DDB_G0271900 | 2 | 1030492 | 1033845 | GID1.0042863 | scaffold_118 | 39891 | 43223 |
| Block 169 | aspA | 2 | 1034955 | 1036338 | fgeneshDP_pm.C_scaffold_118000010 | scaffold_118 | 44046 | 45330 |
| Block 334 | mcfI | 2 | 1036439 | 1037455 | estExt_Genewise1Plus.C_1370025 | scaffold_137 | 32429 | 33421 |
| Block 169 | DDB_G0271928 | 2 | 1038621 | 1043049 | GID1.0042852 | scaffold_118 | 7710 | 11302 |
| Block 334 | DDB_G0271904 | 2 | 1045321 | 1047496 | GID1.0043461 | scaffold_137 | 29554 | 31557 |
| Block 524 | mccB | 2 | 1064824 | 1066590 | estExt_Genewise1Plus.C_1620035 | scaffold_162 | 58258 | 60291 |
| Block 524 | DDB_G0271962 | 2 | 1066886 | 1068286 | e_gw1.162.28.1 | scaffold_162 | 56559 | 58001 |
| Block 524 | DDB_G0271968 | 2 | 1072576 | 1074385 | GID1.0044176 | scaffold_162 | 53891 | 55685 |
| Block 524 | rpl32 | 2 | 1082220 | 1082621 | estExt_Genewise1.C_1620023 | scaffold_162 | 47244 | 49144 |
| Block 37 | DDB_G0272056 | 2 | 1101539 | 1104750 | e_gw1.101.15.1 | scaffold_101 | 11742 | 15466 |
| Block 333 | fsjB | 2 | 1122179 | 1124101 | gw1.137.8.1 | scaffold_137 | 23479 | 24246 |
| Block 333 | DDB_G0272000 | 2 | 1124429 | 1127616 | GID1.0043459 | scaffold_137 | 24357 | 27429 |
| Block 333 | DDB_G0271992 | 2 | 1128892 | 1129408 | fgeneshDP_pm.C_scaffold_137000007 | scaffold_137 | 28777 | 29287 |
| Block 838 | DDB_G0271990 | 2 | 1129784 | 1130716 | estExt_fgeneshDP_pm.C_2080005 | scaffold_208 | 20505 | 21590 |
| Block 838 | DDB_G0271988 | 2 | 1131307 | 1132855 | GID1.0045287 | scaffold_208 | 18304 | 19833 |
| Block 2654 | DDB_G0272006 | 2 | 1154199 | 1156007 | gw1.659.10.1 | scaffold_659 | 4217 | 4552 |
| Block 2654 | DDB_G0272012 | 2 | 1157378 | 1158487 | GID1.0050604 | scaffold_659 | 985 | 2020 |
| Block 540 | DDB_G0272020 | 2 | 1165713 | 1168265 | GID1.0044238 | scaffold_165 | 8608 | 10891 |
| Block 540 | rpc34 | 2 | 1168435 | 1169435 | fgeneshDP_pm.C_scaffold_165000002 | scaffold_165 | 7497 | 8441 |
| Block 35 | DDB_G0272048 | 2 | 1179897 | 1181072 | fgeneshDP_pg.C_scaffold_101000003 | scaffold_101 | 4156 | 5352 |
| Block 35 | DDB_G0272070 | 2 | 1187619 | 1189763 | fgeneshDP_pm.C_scaffold_101000001 | scaffold_101 | 2061 | 3863 |
| Block 83 | DDB_G0272074 | 2 | 1202496 | 1203071 | e_gw1.107.40.1 | scaffold_107 | 45535 | 46104 |
| Block 83 | DDB_G0272090 | 2 | 1203133 | 1205919 | GID1.0042509 | scaffold_107 | 46228 | 49061 |
| Block 1518 | eIF3s6ip | 2 | 1207115 | 1209185 | estExt_Genewise1Plus.C_330004 | scaffold_33 | 8463 | 10513 |
| Block 1518 | eIF3s10 | 2 | 1209925 | 1213181 | e_gw1.33.13.1 | scaffold_33 | 4371 | 7752 |
| Block 2973 | gacHH | 2 | 1225301 | 1230149 | fgeneshDP_pg.C_scaffold_89000016 | scaffold_89 | 55856 | 59992 |
| Block 2973 | DDB_G0272082 | 2 | 1232133 | 1237742 | fgeneshDP_pm.C_scaffold_89000008 | scaffold_89 | 48363 | 50135 |
| Block 2973 | DDB_G0272082 | 2 | 1232133 | 1237742 | e_gw1.89.16.1 | scaffold_89 | 50263 | 51132 |
| Block 2415 | DDB_G0272092 | 2 | 1238036 | 1241826 | e_gw1.562.1.1 | scaffold_562 | 7132 | 10615 |
| Block 2415 | rps29 | 2 | 1245309 | 1245566 | estExt_Genewise1.C_5620008 | scaffold_562 | 13252 | 14218 |
| Block 1911 | spt6 | 2 | 1257785 | 1263215 | e_gw1.418.4.1 | scaffold_418 | 5552 | 9034 |
| Block 1911 | DDB_G0272318 | 2 | 1265242 | 1267143 | estExt_Genewise1.C_4180006 | scaffold_418 | 11784 | 14511 |
| Block 497 | snrpb | 2 | 1270295 | 1271254 | fgeneshDP_pm.C_scaffold_158000008 | scaffold_158 | 59485 | 60327 |
| Block 497 | DDB_G0272322 | 2 | 1271790 | 1272752 | fgeneshDP_pg.C_scaffold_158000016 | scaffold_158 | 60883 | 61833 |
| Block 497 | sbds | 2 | 1273588 | 1274532 | estExt_fgeneshDP_pm.C_1580009 | scaffold_158 | 62454 | 63456 |
| Block 332 | nudE | 2 | 1274622 | 1276054 | estExt_Genewise1Plus.C_1370009 | scaffold_137 | 15168 | 16685 |
| Block 332 | DDB_G0272358 | 2 | 1279792 | 1280825 | gw1.137.43.1 | scaffold_137 | 17855 | 18588 |
| Block 837 | gtf2h2 | 2 | 1283144 | 1284614 | e_gw1.208.28.1 | scaffold_208 | 15007 | 16352 |
| Block 837 | DDB_G0272328 | 2 | 1285206 | 1286051 | estExt_Genewise1Plus.C_2080009 | scaffold_208 | 17136 | 18218 |
| Block 837 | DDB_G0272364 | 2 | 1304225 | 1308675 | GID1.0045295 | scaffold_208 | 37095 | 45182 |
| Block 837 | DDB_G0272366 | 2 | 1309211 | 1312916 | e_gw1.208.11.1 | scaffold_208 | 24927 | 28150 |
| Block 2017 | hd | 2 | 1332807 | 1342356 | estExt_fgeneshDP_pm.C_4440003 | scaffold_444 | 7399 | 16524 |
| Block 2017 | mcfQ | 2 | 1343034 | 1344313 | estExt_fgeneshDP_kg.C_4440001 | scaffold_444 | 5592 | 6822 |
| Block 332 | DDB_G0272130 | 2 | 1353012 | 1353719 | GID1.0043451 | scaffold_137 | 12119 | 12888 |
| Block 1655 | gxcM | 2 | 1354162 | 1357599 | GID1.0039528 | scaffold_36 | 18694 | 21864 |
| Block 1655 | rps23 | 2 | 1360049 | 1361030 | estExt_Genewise1.C_360017 | scaffold_36 | 23740 | 24649 |
| Block 2232 | DDB_G0272128 | 2 | 1361460 | 1362254 | estExt_Genewise1Plus.C_5010003 | scaffold_501 | 4667 | 5747 |
| Block 2232 | DDB_G0272126 | 2 | 1362569 | 1362787 | e_gw1.501.10.1 | scaffold_501 | 6076 | 6300 |
| Block 2232 | DDB_G0272124 | 2 | 1363252 | 1364160 | estExt_Genewise1.C_5010006 | scaffold_501 | 6558 | 7622 |
| Block 2232 | prpf6 | 2 | 1364496 | 1367683 | estExt_Genewise1.C_5010008 | scaffold_501 | 7894 | 10868 |
| Block 2928 | DDB_G0272252 | 2 | 1375218 | 1375889 | GID1.0041713 | scaffold_85 | 8413 | 8913 |
| Block 2928 | DDB_G0272378 | 2 | 1378665 | 1381133 | e_gw1.85.17.1 | scaffold_85 | 12690 | 15605 |
| Block 595 | DDB_G0272380 | 2 | 1381519 | 1382323 | fgeneshDP_pm.C_scaffold_172000007 | scaffold_172 | 50802 | 51631 |
| Block 595 | DDB_G0272254 | 2 | 1382512 | 1386507 | estExt_fgeneshDP_pm.C_1720008 | scaffold_172 | 55255 | 63156 |
| Block 1405 | fam49 | 2 | 1395698 | 1397714 | e_gw1.304.15.1 | scaffold_304 | 13641 | 15118 |
| Block 1405 | DDB_G0272192 | 2 | 1398899 | 1400395 | estExt_Genewise1Plus.C_3040008 | scaffold_304 | 11462 | 13191 |
| Block 2143 | sppA | 2 | 1407657 | 1409140 | GID1.0040119 | scaffold_48 | 1 | 465 |
| Block 2143 | ppp4C | 2 | 1413240 | 1414461 | fgeneshDP_pg.C_scaffold_48000004 | scaffold_48 | 3879 | 5116 |
| Block 2143 | DDB_G0272396 | 2 | 1421494 | 1423584 | estExt_Genewise1.C_480008 | scaffold_48 | 6388 | 7766 |
| Block 2928 | DDB_G0272398 | 2 | 1434624 | 1435328 | e_gw1.85.44.1 | scaffold_85 | 3092 | 3674 |
| Block 1872 | DDB_G0272196 | 2 | 1453487 | 1454548 | estExt_Genewise1.C_4090017 | scaffold_409 | 20224 | 21547 |
| Block 1872 | DDB_G0272272 | 2 | 1457115 | 1458329 | fgeneshDP_pg.C_scaffold_409000012 | scaffold_409 | 23317 | 24454 |
| Block 1872 | DDB_G0272146 | 2 | 1461818 | 1462633 | estExt_fgeneshDP_kg.C_4090006 | scaffold_409 | 25908 | 26810 |
| Block 544 | DDB_G0272404 | 2 | 1465987 | 1466802 | estExt_fgeneshDP_kg.C_1650012 | scaffold_165 | 61253 | 61902 |
| Block 544 | DDB_G0272142 | 2 | 1467358 | 1468101 | gw1.165.51.1 | scaffold_165 | 62997 | 63487 |
| Block 544 | DDB_G0272140 | 2 | 1468342 | 1468868 | fgeneshDP_pg.C_scaffold_165000027 | scaffold_165 | 63836 | 64376 |
| Block 2585 | rab2B | 2 | 1469838 | 1471025 | estExt_fgeneshDP_pm.C_6290001 | scaffold_629 | 980 | 2001 |
| Block 2585 | DDB_G0272406 | 2 | 1472831 | 1476060 | estExt_fgeneshDP_pg.C_6290002 | scaffold_629 | 3698 | 7544 |
| Block 2585 | DDB_G0272418 | 2 | 1490060 | 1490900 | e_gw1.629.8.1 | scaffold_629 | 8061 | 8382 |
| Block 767 | DDB_G0272420 | 2 | 1494277 | 1495359 | estExt_fgeneshDP_kg.C_20016 | scaffold_2 | 249730 | 251207 |
| Block 767 | DDB_G0272186 | 2 | 1495961 | 1499293 | GID1.0037367 | scaffold_2 | 251912 | 255126 |
| Block 767 | DDB_G0272184 | 2 | 1499939 | 1501019 | e_gw1.2.96.1 | scaffold_2 | 256199 | 257164 |
| Block 767 | DDB_G0272182 | 2 | 1501968 | 1504445 | estExt_fgeneshDP_pm.C_20030 | scaffold_2 | 258620 | 261424 |
| Block 767 | DDB_G0272180 | 2 | 1504945 | 1506041 | estExt_fgeneshDP_pg.C_20098 | scaffold_2 | 261752 | 262978 |
| Block 767 | DDB_G0272178 | 2 | 1506498 | 1507001 | GID1.0037371 | scaffold_2 | 263112 | 263578 |
| Block 3040 | DDB_G0272176 | 2 | 1507191 | 1509241 | GID1.0042127 | scaffold_96 | 63276 | 65245 |
| Block 1537 | DDB_G0272432 | 2 | 1546739 | 1548283 | GID1.0047678 | scaffold_334 | 27633 | 29054 |
| Block 3040 | acpB | 2 | 1549151 | 1550511 | estExt_fgeneshDP_pg.C_960020 | scaffold_96 | 68976 | 70599 |
| Block 1537 | DDB_G0272282 | 2 | 1559357 | 1565999 | estExt_Genewise1.C_3340036 | scaffold_334 | 20055 | 25659 |
| Block 767 | DDB_G0272156 | 2 | 1574473 | 1576211 | GID1.0037354 | scaffold_2 | 219600 | 221625 |
| Block 767 | DDB_G0272154 | 2 | 1580645 | 1581638 | estExt_fgeneshDP_kg.C_20012 | scaffold_2 | 226015 | 227218 |
| Block 767 | DDB_G0272152 | 2 | 1582216 | 1582739 | fgeneshDP_pg.C_scaffold_2000086 | scaffold_2 | 224325 | 224883 |
| Block 1888 | rcaA | 2 | 1608827 | 1611361 | e_gw1.410.3.1 | scaffold_410 | 6436 | 8319 |
| Block 1888 | DDB_G0272200 | 2 | 1612599 | 1615397 | e_gw1.410.1.1 | scaffold_410 | 9966 | 12345 |
| Block 1888 | DDB_G0272294 | 2 | 1619189 | 1623033 | GID1.0048746 | scaffold_410 | 12601 | 14310 |
| Block 2147 | DDB_G0272456 | 2 | 1626163 | 1630931 | GID1.0040134 | scaffold_48 | 42572 | 46866 |
| Block 2147 | sarA | 2 | 1631163 | 1632158 | estExt_Genewise1.C_480015 | scaffold_48 | 47658 | 48890 |
| Block 2147 | arpB | 2 | 1633318 | 1634831 | estExt_Genewise1.C_480023 | scaffold_48 | 59551 | 61460 |
| Block 2147 | DDB_G0272202 | 2 | 1637452 | 1639366 | estExt_fgeneshDP_pg.C_480019 | scaffold_48 | 55914 | 57836 |
| Block 2147 | ppp6C | 2 | 1641456 | 1642667 | estExt_Genewise1Plus.C_480018 | scaffold_48 | 53485 | 55237 |
| Block 2147 | DDB_G0272458 | 2 | 1642928 | 1643935 | estExt_fgeneshDP_pg.C_480014 | scaffold_48 | 41391 | 42323 |
| Block 2147 | DDB_G0272206 | 2 | 1644849 | 1650278 | GID1.0040132 | scaffold_48 | 35690 | 40658 |
| Block 840 | orfR1062 | 2 | 1651952 | 1654500 | fgeneshDP_pm.C_scaffold_208000009 | scaffold_208 | 47472 | 50030 |
| Block 840 | idhC | 2 | 1656440 | 1658000 | GID1.0045294 | scaffold_208 | 34767 | 36175 |
| Block 1888 | DDB_G0272212 | 2 | 1660406 | 1662913 | estExt_fgeneshDP_kg.C_4100002 | scaffold_410 | 18722 | 19611 |
| Block 1888 | DDB_G0272214 | 2 | 1664737 | 1666776 | fgeneshDP_pg.C_scaffold_410000009 | scaffold_410 | 24111 | 26117 |
| Block 1888 | DDB_G0272460 | 2 | 1667107 | 1668846 | GID1.0048752 | scaffold_410 | 26692 | 27876 |
| Block 2575 | DDB_G0272218 | 2 | 1676806 | 1678614 | estExt_Genewise1.C_6220001 | scaffold_622 | 593 | 2878 |
| Block 2575 | DDB_G0272302 | 2 | 1679126 | 1679447 | e_gw1.622.7.1 | scaffold_622 | 5081 | 5403 |
| Block 2602 | sgkC | 2 | 1686878 | 1689055 | estExt_fgeneshDP_pg.C_6310005 | scaffold_631 | 7022 | 10841 |
| Block 2602 | qprt | 2 | 1689514 | 1690416 | estExt_Genewise1Plus.C_6310009 | scaffold_631 | 5694 | 6595 |
| Block 2602 | DDB_G0272464 | 2 | 1691771 | 1692277 | e_gw1.631.9.1 | scaffold_631 | 3432 | 3755 |
| Block 543 | DDB_G0272468 | 2 | 1694220 | 1696756 | estExt_fgeneshDP_pg.C_1650020 | scaffold_165 | 51374 | 53800 |
| Block 543 | ybl1 | 2 | 1697348 | 1697842 | estExt_fgeneshDP_kg.C_1650011 | scaffold_165 | 54197 | 54883 |
| Block 543 | DDB_G0272224 | 2 | 1698110 | 1699602 | gw1.165.39.1 | scaffold_165 | 55425 | 56654 |
| Block 2921 | DDB_G0272226 | 2 | 1699668 | 1701323 | fgeneshDP_pg.C_scaffold_84000013 | scaffold_84 | 31575 | 33188 |
| Block 2921 | DDB_G0272230 | 2 | 1702673 | 1703347 | GID1.0041674 | scaffold_84 | 26809 | 27435 |
| Block 2921 | DDB_G0272232 | 2 | 1704105 | 1704593 | GID1.0041673 | scaffold_84 | 25715 | 26035 |
| Block 2921 | pex2 | 2 | 1704902 | 1706265 | GID1.0041672 | scaffold_84 | 24060 | 25434 |
| Block 2921 | aspS1 | 2 | 1708635 | 1710443 | e_gw1.84.2.1 | scaffold_84 | 21646 | 23166 |
| Block 280 | DDB_G0272476 | 2 | 1734394 | 1734901 | estExt_Genewise1Plus.C_130119 | scaffold_13 | 134203 | 135129 |
| Block 280 | DDB_G0272480 | 2 | 1738418 | 1742703 | GID1.0038228 | scaffold_13 | 137179 | 140611 |
| Block 2598 | prsB | 2 | 1745412 | 1746503 | GID1.0050476 | scaffold_630 | 7838 | 8975 |
| Block 2598 | DDB_G0272484 | 2 | 1746988 | 1748640 | GID1.0050477 | scaffold_630 | 9586 | 10981 |
| Block 2598 | prkag | 2 | 1774988 | 1776993 | fgeneshDP_pm.C_scaffold_630000001 | scaffold_630 | 3081 | 4972 |
| Block 2198 | DDB_G0272486 | 2 | 1778071 | 1780952 | estExt_fgeneshDP_pm.C_4910004 | scaffold_491 | 15176 | 18492 |
| Block 2598 | DDB_G0272544 | 2 | 1782585 | 1784685 | GID1.0050468 | scaffold_630 | 216 | 2336 |
| Block 2198 | osbD | 2 | 1790250 | 1791849 | fgeneshDP_pg.C_scaffold_491000005 | scaffold_491 | 11838 | 13550 |
| Block 769 | malA | 2 | 1797717 | 1799893 | estExt_Genewise1.C_20112 | scaffold_2 | 235682 | 237907 |
| Block 769 | DDB_G0272502 | 2 | 1804106 | 1807486 | GID1.0037359 | scaffold_2 | 229409 | 232646 |
| Block 2921 | DDB_G0272504 | 2 | 1807635 | 1808303 | fgeneshDP_pm.C_scaffold_84000007 | scaffold_84 | 74827 | 75480 |
| Block 1985 | DDB_G0272530 | 2 | 1812894 | 1813799 | estExt_fgeneshDP_kg.C_4390003 | scaffold_439 | 9878 | 11184 |
| Block 2180 | sgkA | 2 | 1832589 | 1834463 | GID1.0049540 | scaffold_488 | 2811 | 4727 |
| Block 2180 | DDB_G0272512 | 2 | 1834572 | 1835498 | estExt_fgeneshDP_kg.C_4880001 | scaffold_488 | 1691 | 2742 |
| Block 1985 | DDB_G0272909 | 2 | 1842798 | 1844058 | fgeneshDP_pg.C_scaffold_439000006 | scaffold_439 | 11552 | 12531 |
| Block 1913 | DDB_G0272666 | 2 | 1844804 | 1848829 | estExt_fgeneshDP_pg.C_4180006 | scaffold_418 | 17831 | 20856 |
| Block 1913 | timm9 | 2 | 1849198 | 1849452 | GID1.0048851 | scaffold_418 | 21720 | 21968 |
| Block 2756 | DDB_G0272933 | 2 | 1849914 | 1850503 | e_gw1.708.7.1 | scaffold_708 | 1541 | 2088 |
| Block 2756 | mocos | 2 | 1851663 | 1854686 | estExt_Genewise1Plus.C_7080005 | scaffold_708 | 2506 | 5653 |
| Block 1563 | DDB_G0272724 | 2 | 1861665 | 1861979 | estExt_fgeneshDP_kg.C_340005 | scaffold_34 | 61798 | 62508 |
| Block 2197 | DDB_G0272949 | 2 | 1876102 | 1879953 | estExt_fgeneshDP_pg.C_4910004 | scaffold_491 | 6659 | 10022 |
| Block 2197 | DDB_G0272951 | 2 | 1881029 | 1882708 | GID1.0049570 | scaffold_491 | 3752 | 5287 |
| Block 2197 | psmD14 | 2 | 1883110 | 1884356 | estExt_Genewise1.C_4910001 | scaffold_491 | 1915 | 3542 |
| Block 1563 | hspC | 2 | 1884709 | 1885671 | fgeneshDP_pg.C_scaffold_34000027 | scaffold_34 | 59255 | 60185 |
| Block 274 | DDB_G0272953 | 2 | 1890614 | 1892396 | GID1.0038179 | scaffold_13 | 12477 | 14034 |
| Block 274 | DDB_G0272955 | 2 | 1892469 | 1893836 | estExt_Genewise1Plus.C_130004 | scaffold_13 | 3698 | 5105 |
| Block 274 | DDB_G0272957 | 2 | 1894181 | 1894921 | fgeneshDP_pg.C_scaffold_13000007 | scaffold_13 | 14118 | 14942 |
| Block 274 | DDB_G0272913 | 2 | 1895147 | 1896018 | fgeneshDP_pm.C_scaffold_13000002 | scaffold_13 | 15308 | 16145 |
| Block 274 | DDB_G0272708 | 2 | 1896942 | 1897665 | fgeneshDP_pm.C_scaffold_13000003 | scaffold_13 | 17541 | 18283 |
| Block 1967 | DDB_G0272706 | 2 | 1897975 | 1899063 | GID1.0048984 | scaffold_431 | 19492 | 20574 |
| Block 1967 | CYP515A1 | 2 | 1902373 | 1904386 | e_gw1.431.8.1 | scaffold_431 | 21187 | 22953 |
| Block 274 | DDB_G0272696 | 2 | 1912183 | 1925844 | estExt_Genewise1Plus.C_130034 | scaffold_13 | 18818 | 31691 |
| Block 274 | DDB_G0272965 | 2 | 1933223 | 1935198 | GID1.0038208 | scaffold_13 | 81723 | 83552 |
| Block 274 | psmB1 | 2 | 1939043 | 1940202 | estExt_Genewise1.C_130089 | scaffold_13 | 86857 | 88049 |
| Block 274 | DDB_G0272971 | 2 | 1941389 | 1942639 | e_gw1.13.63.1 | scaffold_13 | 62470 | 63593 |
| Block 274 | gacH | 2 | 1943915 | 1945799 | GID1.0038199 | scaffold_13 | 59700 | 61575 |
| Block 274 | DDB_G0272690 | 2 | 1955378 | 1957800 | GID1.0038216 | scaffold_13 | 102440 | 104312 |
| Block 274 | DDB_G0272975 | 2 | 1960485 | 1962871 | estExt_fgeneshDP_kg.C_130017 | scaffold_13 | 130809 | 134043 |
| Block 1565 | DDB_G0272686 | 2 | 1970271 | 1971638 | GID1.0039462 | scaffold_34 | 85440 | 86840 |
| Block 1565 | qdpr | 2 | 1971701 | 1972500 | e_gw1.34.69.1 | scaffold_34 | 86953 | 87718 |
| Block 1565 | plip | 2 | 1973280 | 1973978 | fgeneshDP_pm.C_scaffold_34000018 | scaffold_34 | 88264 | 88944 |
| Block 1565 | DDB_G0272682 | 2 | 1974279 | 1977711 | GID1.0039465 | scaffold_34 | 89381 | 93087 |
| Block 760 | DDB_G0272680 | 2 | 1989656 | 1993357 | estExt_fgeneshDP_pg.C_20069 | scaffold_2 | 187191 | 190914 |
| Block 760 | DDB_G0272678 | 2 | 1994043 | 1996634 | gw1.2.58.1 | scaffold_2 | 191710 | 194148 |
| Block 760 | DDB_G0272983 | 2 | 1996836 | 1998654 | gw1.2.64.1 | scaffold_2 | 169879 | 171667 |
| Block 760 | vps54 | 2 | 1998974 | 2002002 | e_gw1.2.50.1 | scaffold_2 | 171929 | 174871 |
| Block 760 | rps4 | 2 | 2005501 | 2006734 | fgeneshDP_pg.C_scaffold_2000039 | scaffold_2 | 106409 | 107678 |
| Block 496 | vps5 | 2 | 2012017 | 2014408 | estExt_Genewise1Plus.C_1580024 | scaffold_158 | 54740 | 57682 |
| Block 496 | gacU | 2 | 2015227 | 2019243 | estExt_fgeneshDP_pg.C_1580012 | scaffold_158 | 50784 | 54383 |
| Block 496 | exoc7 | 2 | 2020446 | 2023366 | fgeneshDP_pm.C_scaffold_158000006 | scaffold_158 | 46429 | 49112 |
| Block 496 | sf3a1 | 2 | 2023681 | 2026145 | GID1.0044061 | scaffold_158 | 43947 | 46088 |
| Block 1984 | DDB_G0272871 | 2 | 2043175 | 2043819 | e_gw1.439.10.1 | scaffold_439 | 1890 | 2531 |
| Block 1984 | sun1 | 2 | 2044015 | 2046802 | GID1.0049066 | scaffold_439 | 2662 | 5292 |
| Block 1984 | DDB_G0272995 | 2 | 2047544 | 2051265 | estExt_fgeneshDP_pm.C_4390002 | scaffold_439 | 6046 | 9911 |
| Block 279 | DDB_G0272997 | 2 | 2068279 | 2071820 | gw1.13.56.1 | scaffold_13 | 112467 | 113549 |
| Block 279 | DDB_G0272748 | 2 | 2078511 | 2080559 | GID1.0038235 | scaffold_13 | 156860 | 158667 |
| Block 279 | rapB | 2 | 2080811 | 2081657 | e_gw1.13.20.1 | scaffold_13 | 155859 | 156569 |
| Block 496 | psmA7 | 2 | 2088469 | 2089357 | estExt_Genewise1.C_1580008 | scaffold_158 | 7906 | 8726 |
| Block 496 | DDB_G0272758 | 2 | 2094594 | 2097497 | GID1.0044051 | scaffold_158 | 3836 | 6606 |
| Block 496 | snrpD3 | 2 | 2104522 | 2104962 | fgeneshDP_pg.C_scaffold_158000001 | scaffold_158 | 3140 | 3589 |
| Block 2183 | DDB_G0272769 | 2 | 2132196 | 2134630 | GID1.0049545 | scaffold_488 | 17165 | 19671 |
| Block 541 | DDB_G0272839 | 2 | 2134935 | 2136390 | GID1.0044257 | scaffold_165 | 39925 | 41307 |
| Block 541 | wdr7 | 2 | 2140791 | 2144919 | GID1.0044258 | scaffold_165 | 41416 | 45393 |
| Block 2183 | uglB | 2 | 2146681 | 2148474 | gw1.488.16.1 | scaffold_488 | 14891 | 15550 |
| Block 541 | DDB_G0272777 | 2 | 2151170 | 2153486 | gw1.165.18.1 | scaffold_165 | 29648 | 31910 |
| Block 541 | DDB_G0272785 | 2 | 2160344 | 2162380 | GID1.0044252 | scaffold_165 | 32096 | 34199 |
| Block 541 | sds | 2 | 2163013 | 2164353 | estExt_fgeneshDP_pg.C_1650014 | scaffold_165 | 35036 | 36614 |
| Block 2558 | emg1 | 2 | 2206713 | 2207824 | GID1.0050400 | scaffold_616 | 6909 | 7981 |
| Block 1055 | iunH | 2 | 2213617 | 2214639 | estExt_Genewise1Plus.C_2400029 | scaffold_240 | 46256 | 47441 |
| Block 1055 | DDB_G0272740 | 2 | 2215013 | 2215587 | GID1.0045965 | scaffold_240 | 35535 | 36130 |
| Block 1055 | DDB_G0272811 | 2 | 2219349 | 2221241 | fgeneshDP_pg.C_scaffold_240000011 | scaffold_240 | 33578 | 35408 |
| Block 1055 | DDB_G0273017 | 2 | 2223000 | 2224406 | estExt_fgeneshDP_kg.C_2400002 | scaffold_240 | 25302 | 27812 |
| Block 1055 | DDB_G0272746 | 2 | 2225559 | 2226698 | GID1.0045961 | scaffold_240 | 24309 | 25325 |
| Block 1055 | DDB_G0272841 | 2 | 2227520 | 2229274 | gw1.240.18.1 | scaffold_240 | 21853 | 23334 |
| Block 1055 | serS | 2 | 2229970 | 2231623 | estExt_Genewise1.C_2400011 | scaffold_240 | 19298 | 21137 |
| Block 2558 | ppr2 | 2 | 2234123 | 2235371 | fgeneshDP_pg.C_scaffold_616000004 | scaffold_616 | 10302 | 11446 |
| Block 1658 | CYP525A1 | 2 | 2240074 | 2242191 | e_gw1.36.55.1 | scaffold_36 | 90227 | 91965 |
| Block 1658 | rsc11-1 | 2 | 2254519 | 2256476 | GID1.0039545 | scaffold_36 | 45202 | 47146 |
| Block 1658 | adrm1-1 | 2 | 2256649 | 2258227 | GID1.0039544 | scaffold_36 | 43479 | 44897 |
| Block 2162 | dohh-1 | 2 | 2269454 | 2270482 | GID1.0049482 | scaffold_482 | 10428 | 11498 |
| Block 2162 | DDB_G0273053 | 2 | 2274972 | 2279528 | estExt_fgeneshDP_pg.C_4820005 | scaffold_482 | 13795 | 17599 |
| Block 1332 | DDB_G0273027 | 2 | 2279823 | 2280959 | gw1.293.15.1 | scaffold_293 | 31753 | 32490 |
| Block 2647 | DDB_G0273029 | 2 | 2281164 | 2281613 | fgeneshDP_pg.C_scaffold_656000003 | scaffold_656 | 8008 | 8440 |
| Block 2647 | DDB_G0272638 | 2 | 2283620 | 2287270 | GID1.0050590 | scaffold_656 | 1355 | 4608 |
| Block 331 | 3B-1 | 2 | 2307782 | 2308523 | estExt_fgeneshDP_pm.C_1370002 | scaffold_137 | 11150 | 11989 |
| Block 331 | DDB_G0273031 | 2 | 2309331 | 2311383 | GID1.0043449 | scaffold_137 | 9957 | 10750 |
| Block 863 | DDB_G0273033 | 2 | 2314489 | 2319305 | GID1.0045345 | scaffold_210 | 49530 | 53926 |
| Block 863 | stip-1 | 2 | 2321827 | 2324606 | fgeneshDP_pg.C_scaffold_210000003 | scaffold_210 | 10910 | 13354 |
| Block 1332 | DDB_G0273037 | 2 | 2332610 | 2336477 | GID1.0046996 | scaffold_293 | 23033 | 26596 |
| Block 890 | DDB_G0272612 | 2 | 2337116 | 2338958 | fgeneshDP_pm.C_scaffold_214000008 | scaffold_214 | 44463 | 46228 |
| Block 890 | DDB_G0272614 | 2 | 2339222 | 2339775 | e_gw1.214.33.1 | scaffold_214 | 46391 | 47047 |
| Block 890 | tkt-1 | 2 | 2344406 | 2346517 | estExt_Genewise1Plus.C_2140034 | scaffold_214 | 49361 | 51553 |
| Block 2548 | iptC-1 | 2 | 2346717 | 2348255 | e_gw1.612.1.1 | scaffold_612 | 5362 | 6516 |
| Block 2548 | ksrA-1 | 2 | 2348286 | 2349493 | e_gw1.612.7.1 | scaffold_612 | 2685 | 3811 |
| Block 2548 | DDB_G0272620 | 2 | 2349783 | 2350307 | fgeneshDP_pg.C_scaffold_612000003 | scaffold_612 | 4230 | 4836 |
| Block 1687 | DDB_G0272580 | 2 | 2366874 | 2367775 | GID1.0048171 | scaffold_368 | 21 | 843 |
| Block 1687 | DDB_G0273041 | 2 | 2367964 | 2369307 | gw1.368.11.1 | scaffold_368 | 1276 | 2463 |
| Block 1654 | maoB-1 | 2 | 2371794 | 2373338 | e_gw1.36.59.1 | scaffold_36 | 120008 | 121535 |
| Block 1687 | DDB_G0272586 | 2 | 2376866 | 2377720 | estExt_fgeneshDP_pg.C_3680012 | scaffold_368 | 20327 | 21169 |
| Block 863 | rpl15-1 | 2 | 2381693 | 2382564 | estExt_fgeneshDP_pg.C_2100007 | scaffold_210 | 22232 | 23140 |
| Block 863 | dpm2-1 | 2 | 2383219 | 2383623 | GID1.0045333 | scaffold_210 | 23796 | 24626 |
| Block 863 | psmD8-1 | 2 | 2383995 | 2385114 | estExt_Genewise1.C_2100028 | scaffold_210 | 25001 | 26184 |
| Block 863 | srp14-1 | 2 | 2385325 | 2385874 | fgeneshDP_pg.C_scaffold_210000010 | scaffold_210 | 26297 | 26866 |
| Block 1654 | DDB_G0272600 | 2 | 2424167 | 2428889 | GID1.0039557 | scaffold_36 | 67719 | 72161 |
| Block 1320 | DDB_G0272807 | 2 | 2429610 | 2433567 | fgeneshDP_pg.C_scaffold_290000014 | scaffold_290 | 37615 | 40296 |
| Block 1320 | DDB_G0272576 | 2 | 2452620 | 2453345 | GID1.0046952 | scaffold_290 | 40906 | 41542 |
| Block 1654 | psmB4-1 | 2 | 2469210 | 2470451 | fgeneshDP_pg.C_scaffold_36000029 | scaffold_36 | 72928 | 74047 |
| Block 1654 | DDB_G0273165 | 2 | 2470630 | 2471707 | fgeneshDP_pg.C_scaffold_36000030 | scaffold_36 | 74306 | 75283 |
| Block 1765 | DDB_G0273151 | 2 | 2510290 | 2512562 | estExt_fgeneshDP_pg.C_3880008 | scaffold_388 | 19949 | 22131 |
| Block 1765 | DDB_G0273177 | 2 | 2513131 | 2513748 | GID1.0048464 | scaffold_388 | 18610 | 19075 |
| Block 1765 | DDB_G0273125 | 2 | 2514851 | 2517055 | estExt_fgeneshDP_pg.C_3880006 | scaffold_388 | 16566 | 18475 |
| Block 1765 | pakH-1 | 2 | 2519173 | 2520921 | fgeneshDP_pm.C_scaffold_388000002 | scaffold_388 | 14548 | 16295 |
| Block 1765 | DDB_G0273119 | 2 | 2522321 | 2522763 | GID1.0048461 | scaffold_388 | 13479 | 13975 |
| Block 1765 | ublcp1-1 | 2 | 2523447 | 2524743 | GID1.0048460 | scaffold_388 | 11203 | 12583 |
| Block 1654 | DDB_G0273105 | 2 | 2548517 | 2551298 | estExt_Genewise1.C_360005 | scaffold_36 | 9569 | 12400 |
| Block 1654 | DDB_G0273103 | 2 | 2552197 | 2552991 | estExt_Genewise1Plus.C_360011 | scaffold_36 | 12968 | 13788 |
| Block 865 | cinD-1 | 2 | 2626506 | 2627374 | GID1.0045329 | scaffold_210 | 16984 | 17643 |
| Block 1534 | dhkI-1 | 2 | 2912996 | 2918311 | GID1.0047668 | scaffold_333 | 22892 | 27741 |
| Block 887 | gtf2e1-1 | 2 | 2964684 | 2966772 | estExt_Genewise1Plus.C_2140015 | scaffold_214 | 31930 | 33639 |
| Block 887 | DDB_G0273499 | 2 | 3010779 | 3012146 | GID1.0045429 | scaffold_214 | 27749 | 28907 |
| Block 865 | DDB_G0273509 | 2 | 3024488 | 3025945 | e_gw1.210.33.1 | scaffold_210 | 39437 | 40906 |
| Block 2054 | DDB_G0273515 | 2 | 3030948 | 3031769 | fgeneshDP_pg.C_scaffold_455000005 | scaffold_455 | 10817 | 11527 |
| Block 887 | DDB_G0273523 | 2 | 3036766 | 3037638 | gw1.214.28.1 | scaffold_214 | 30585 | 31436 |
| Block 887 | DDB_G0273527 | 2 | 3041125 | 3042357 | estExt_fgeneshDP_pm.C_2140006 | scaffold_214 | 34749 | 36030 |
| Block 1534 | DDB_G0273529 | 2 | 3042630 | 3045345 | GID1.0047663 | scaffold_333 | 6709 | 9086 |
| Block 1534 | spkA-2 | 2 | 3046597 | 3048840 | estExt_fgeneshDP_pm.C_3330003 | scaffold_333 | 9827 | 12477 |
| Block 1534 | carA-2 | 2 | 3052522 | 3053843 | estExt_fgeneshDP_pg.C_3330004 | scaffold_333 | 14848 | 16835 |
| Block 1534 | DDB_G0273537 | 2 | 3057104 | 3058771 | estExt_fgeneshDP_pg.C_3330005 | scaffold_333 | 16973 | 18457 |
| Block 2054 | DDB_G0273539 | 2 | 3059009 | 3062996 | estExt_Genewise1.C_4550003 | scaffold_455 | 2186 | 5648 |
| Block 2694 | DDB_G0273547 | 2 | 3073196 | 3074768 | GID1.0050672 | scaffold_677 | 2615 | 4208 |
| Block 2694 | DDB_G0273549 | 2 | 3075300 | 3076373 | fgeneshDP_pg.C_scaffold_677000003 | scaffold_677 | 4651 | 5493 |
| Block 2694 | DDB_G0273551 | 2 | 3076526 | 3077896 | e_gw1.677.4.1 | scaffold_677 | 5830 | 6732 |
| Block 1534 | DDB_G0273553 | 2 | 3078562 | 3081255 | estExt_fgeneshDP_pg.C_3330010 | scaffold_333 | 33660 | 35510 |
| Block 1534 | DDB_G0273555 | 2 | 3081447 | 3082268 | e_gw1.333.18.1 | scaffold_333 | 32803 | 33534 |
| Block 1534 | DDB_G0273557 | 2 | 3082837 | 3084666 | GID1.0047669 | scaffold_333 | 29933 | 32096 |
| Block 1534 | DDB_G0273559 | 2 | 3092091 | 3095655 | estExt_Genewise1.C_3330010 | scaffold_333 | 19009 | 22453 |
| Block 2054 | dpp3-2 | 2 | 3097910 | 3100373 | estExt_Genewise1Plus.C_4550019 | scaffold_455 | 17898 | 20465 |
| Block 2054 | DDB_G0273565 | 2 | 3101107 | 3104001 | GID1.0049219 | scaffold_455 | 15394 | 17374 |
| Block 886 | DDB_G0273573 | 2 | 3106300 | 3123499 | estExt_Genewise1.C_2140002 | scaffold_214 | 2029 | 16735 |
| Block 886 | DDB_G0273579 | 2 | 3126865 | 3127422 | e_gw1.214.37.1 | scaffold_214 | 51840 | 52345 |
| Block 2054 | DDB_G0273587 | 2 | 3131685 | 3131831 | e_gw1.455.21.1 | scaffold_455 | 23025 | 23222 |
| Block 167 | DDB_G0273589 | 2 | 3132359 | 3133519 | e_gw1.118.41.1 | scaffold_118 | 1495 | 2868 |
| Block 2145 | DDB_G0273595 | 2 | 3140612 | 3142428 | estExt_Genewise1Plus.C_480035 | scaffold_48 | 80911 | 82656 |
| Block 2145 | DDB_G0273597 | 2 | 3142812 | 3143791 | GID1.0040141 | scaffold_48 | 64282 | 65242 |
| Block 167 | DDB_G0273601 | 2 | 3145806 | 3147044 | gw1.118.31.1 | scaffold_118 | 5514 | 6734 |
| Block 1660 | DDB_G0273603 | 2 | 3147512 | 3148004 | gw1.36.103.1 | scaffold_36 | 84408 | 84572 |
| Block 1660 | psiG-2 | 2 | 3149148 | 3151405 | estExt_fgeneshDP_pg.C_360036 | scaffold_36 | 86954 | 89383 |
| Block 1660 | DDB_G0273607 | 2 | 3151692 | 3152213 | estExt_Genewise1.C_360076 | scaffold_36 | 89556 | 90026 |
| Block 167 | DDB_G0273611 | 2 | 3155620 | 3156853 | fgeneshDP_pg.C_scaffold_118000002 | scaffold_118 | 3345 | 4555 |
| Block 2145 | DDB_G0273613 | 2 | 3157497 | 3158321 | estExt_fgeneshDP_pg.C_480027 | scaffold_48 | 79416 | 80444 |
| Block 2145 | DDB_G0273617 | 2 | 3161370 | 3163430 | GID1.0040136 | scaffold_48 | 49476 | 51403 |
| Block 1660 | g6pd-2 | 2 | 3194335 | 3196222 | estExt_Genewise1.C_360035 | scaffold_36 | 49796 | 52411 |
| Block 2145 | DDB_G0273647 | 2 | 3212490 | 3216157 | fgeneshDP_pg.C_scaffold_48000008 | scaffold_48 | 17845 | 20671 |
| Block 2145 | DDB_G0273651 | 2 | 3218798 | 3223683 | e_gw1.48.17.1 | scaffold_48 | 10841 | 15755 |
| Block 1692 | DDB_G0273675 | 2 | 3248949 | 3252861 | GID1.0048190 | scaffold_368 | 25964 | 29715 |
| Block 1692 | DDB_G0273677 | 2 | 3253110 | 3254228 | fgeneshDP_pg.C_scaffold_368000015 | scaffold_368 | 24707 | 25783 |
| Block 1625 | DDB_G0273723 | 2 | 3311099 | 3314131 | estExt_fgeneshDP_pm.C_3520002 | scaffold_352 | 4456 | 7682 |
| Block 1625 | snf12-2 | 2 | 3315750 | 3317324 | fgeneshDP_pg.C_scaffold_352000004 | scaffold_352 | 9000 | 10534 |
| Block 1660 | DDB_G0273727 | 2 | 3317565 | 3318374 | e_gw1.36.86.1 | scaffold_36 | 58268 | 59110 |
| Block 1660 | DDB_G0273729 | 2 | 3318553 | 3319983 | gw1.36.63.1 | scaffold_36 | 54744 | 55844 |
| Block 885 | DDB_G0273733 | 2 | 3323042 | 3325113 | gw1.214.23.1 | scaffold_214 | 38439 | 39552 |
| Block 1625 | DDB_G0273737 | 2 | 3327786 | 3329396 | estExt_Genewise1.C_3520003 | scaffold_352 | 6 | 1134 |
| Block 590 | DDB_G0273741 | 2 | 3333729 | 3334817 | estExt_Genewise1Plus.C_1720014 | scaffold_172 | 41132 | 41858 |
| Block 590 | rps30-2 | 2 | 3335567 | 3336368 | e_gw1.172.27.1 | scaffold_172 | 39511 | 39711 |
| Block 590 | DDB_G0273745 | 2 | 3336578 | 3339082 | estExt_fgeneshDP_pm.C_1720004 | scaffold_172 | 36763 | 39400 |
| Block 885 | DDB_G0273767 | 2 | 3370553 | 3372184 | estExt_fgeneshDP_pg.C_2140001 | scaffold_214 | 458 | 1990 |
| Block 864 | DDB_G0273773 | 2 | 3375807 | 3376283 | estExt_fgeneshDP_pm.C_2100003 | scaffold_210 | 18593 | 19353 |
| Block 864 | DDB_G0273777 | 2 | 3378433 | 3379590 | e_gw1.210.36.1 | scaffold_210 | 15300 | 16370 |
| Block 2154 | ddo-1 | 2 | 3388862 | 3389902 | estExt_fgeneshDP_kg.C_480009 | scaffold_48 | 101036 | 102245 |
| Block 590 | DDB_G0273789 | 2 | 3393721 | 3394641 | e_gw1.172.21.1 | scaffold_172 | 12234 | 13145 |
| Block 590 | DDB_G0273791 | 2 | 3394712 | 3396298 | GID1.0044432 | scaffold_172 | 13298 | 14558 |
| Block 2154 | DDB_G0273795 | 2 | 3398938 | 3404688 | GID1.0040160 | scaffold_48 | 106350 | 112549 |
| Block 2154 | ndkC-2 | 2 | 3414392 | 3415125 | estExt_fgeneshDP_pg.C_480038 | scaffold_48 | 105084 | 105788 |
| Block 1331 | DDB_G0273811 | 2 | 3419583 | 3423121 | GID1.0046995 | scaffold_293 | 19420 | 22740 |
| Block 1331 | DDB_G0273813 | 2 | 3423516 | 3426735 | GID1.0046990 | scaffold_293 | 9213 | 12227 |
| Block 1331 | DDB_G0273815 | 2 | 3426859 | 3427485 | GID1.0046989 | scaffold_293 | 8370 | 9026 |
| Block 1415 | DDB_G0274757 | 2 | 3767669 | 3768464 | e_gw1.308.25.1 | scaffold_308 | 971 | 1662 |
| Block 2195 | gtr1 | 2 | 3769011 | 3773077 | e_gw1.490.1.1 | scaffold_490 | 1520 | 5188 |
| Block 2195 | mpheS | 2 | 3773217 | 3774581 | gw1.490.6.1 | scaffold_490 | 15689 | 16843 |
| Block 2195 | DDB_G0274771 | 2 | 3774786 | 3775580 | GID1.0049565 | scaffold_490 | 14507 | 15312 |
| Block 2195 | DDB_G0274369 | 2 | 3776684 | 3777742 | GID1.0049564 | scaffold_490 | 8689 | 13066 |
| Block 1415 | DDB_G0274365 | 2 | 3781328 | 3782643 | estExt_fgeneshDP_kg.C_3080002 | scaffold_308 | 3331 | 4693 |
| Block 1718 | DDB_G0274363 | 2 | 3782937 | 3784604 | estExt_fgeneshDP_pg.C_3740001 | scaffold_374 | 12 | 1459 |
| Block 2369 | DDB_G0274775 | 2 | 3790952 | 3793106 | estExt_Genewise1.C_5460004 | scaffold_546 | 13315 | 15349 |
| Block 2369 | ctnA | 2 | 3794134 | 3794997 | e_gw1.546.3.1 | scaffold_546 | 11804 | 12656 |
| Block 635 | DDB_G0274355 | 2 | 3803866 | 3804451 | estExt_fgeneshDP_pg.C_180019 | scaffold_18 | 42236 | 42877 |
| Block 635 | DDB_G0274779 | 2 | 3804733 | 3806021 | e_gw1.18.53.1 | scaffold_18 | 41423 | 41947 |
| Block 1718 | DDB_G0274353 | 2 | 3806791 | 3807972 | estExt_fgeneshDP_kg.C_3740001 | scaffold_374 | 1686 | 3029 |
| Block 1718 | catA | 2 | 3809579 | 3811150 | estExt_Genewise1.C_3740002 | scaffold_374 | 4626 | 6345 |
| Block 1718 | DDB_G0274781 | 2 | 3811687 | 3812827 | estExt_Genewise1Plus.C_3740007 | scaffold_374 | 6721 | 7913 |
| Block 1718 | DDB_G0274785 | 2 | 3813200 | 3816537 | estExt_Genewise1Plus.C_3740008 | scaffold_374 | 8295 | 11533 |
| Block 1718 | DDB_G0274351 | 2 | 3816583 | 3818151 | fgeneshDP_pg.C_scaffold_374000006 | scaffold_374 | 11560 | 12693 |
| Block 1718 | DDB_G0274349 | 2 | 3819679 | 3821667 | fgeneshDP_pg.C_scaffold_374000008 | scaffold_374 | 14691 | 16505 |
| Block 1718 | DDB_G0274347 | 2 | 3822485 | 3824599 | estExt_Genewise1Plus.C_3740009 | scaffold_374 | 17584 | 23321 |
| Block 2897 | DDB_G0274795 | 2 | 3843569 | 3847820 | e_gw1.80.20.1 | scaffold_80 | 52718 | 56219 |
| Block 2897 | dst1 | 2 | 3848457 | 3850862 | GID1.0041521 | scaffold_80 | 49480 | 51813 |
| Block 2897 | DDB_G0274585 | 2 | 3855554 | 3856925 | fgeneshDP_pg.C_scaffold_80000025 | scaffold_80 | 56285 | 57697 |
| Block 2729 | DDB_G0274581 | 2 | 3857487 | 3859303 | GID1.0037768 | scaffold_7 | 91292 | 92961 |
| Block 2729 | pitB | 2 | 3865153 | 3866468 | estExt_fgeneshDP_kg.C_70005 | scaffold_7 | 89555 | 90810 |
| Block 2729 | DDB_G0274329 | 2 | 3866755 | 3867478 | e_gw1.7.132.1 | scaffold_7 | 86286 | 86971 |
| Block 2729 | DDB_G0274331 | 2 | 3873454 | 3874617 | estExt_Genewise1Plus.C_70126 | scaffold_7 | 101706 | 103149 |
| Block 2729 | DDB_G0274333 | 2 | 3874798 | 3875222 | fgeneshDP_pg.C_scaffold_7000045 | scaffold_7 | 103815 | 104290 |
| Block 2729 | DDB_G0274335 | 2 | 3875681 | 3876691 | GID1.0037773 | scaffold_7 | 104580 | 105560 |
| Block 2729 | DDB_G0274337 | 2 | 3877488 | 3879243 | estExt_fgeneshDP_kg.C_70007 | scaffold_7 | 106270 | 108211 |
| Block 2729 | DG1124 | 2 | 3879409 | 3883367 | GID1.0037788 | scaffold_7 | 139535 | 142576 |
| Block 2729 | DDB_G0274339 | 2 | 3884764 | 3886120 | estExt_fgeneshDP_pm.C_70014 | scaffold_7 | 58369 | 60135 |
| Block 2244 | DDB_G0274751 | 2 | 3901311 | 3902618 | estExt_fgeneshDP_pg.C_5070002 | scaffold_507 | 1058 | 2691 |
| Block 2244 | DDB_G0274745 | 2 | 3904495 | 3905754 | estExt_Genewise1.C_5070002 | scaffold_507 | 3371 | 5703 |
| Block 792 | DDB_G0274327 | 2 | 3906527 | 3908579 | estExt_fgeneshDP_pm.C_2010001 | scaffold_201 | 570 | 2611 |
| Block 792 | DDB_G0274743 | 2 | 3908851 | 3911455 | estExt_fgeneshDP_pg.C_2010002 | scaffold_201 | 2597 | 5057 |
| Block 792 | DDB_G0274803 | 2 | 3914962 | 3919478 | GID1.0045122 | scaffold_201 | 5322 | 9264 |
| Block 2375 | DDB_G0274805 | 2 | 3922920 | 3924846 | fgeneshDP_pg.C_scaffold_549000006 | scaffold_549 | 9387 | 10976 |
| Block 2721 | DDB_G0274311 | 2 | 3925403 | 3926030 | GID1.0037726 | scaffold_7 | 2545 | 3258 |
| Block 2375 | pex4 | 2 | 3926398 | 3926951 | GID1.0049998 | scaffold_549 | 8544 | 9169 |
| Block 2375 | DDB_G0274739 | 2 | 3927777 | 3929242 | estExt_Genewise1Plus.C_5490005 | scaffold_549 | 6623 | 7559 |
| Block 2375 | dpiA | 2 | 3929517 | 3930405 | estExt_fgeneshDP_pm.C_5490003 | scaffold_549 | 5488 | 6514 |
| Block 2375 | DDB_G0274317 | 2 | 3930966 | 3932417 | estExt_fgeneshDP_pm.C_5490002 | scaffold_549 | 3632 | 5289 |
| Block 2721 | DDB_G0274323 | 2 | 3942764 | 3943688 | GID1.0037729 | scaffold_7 | 8952 | 9808 |
| Block 2721 | ddx52 | 2 | 3943979 | 3946098 | gw1.7.5.1 | scaffold_7 | 10767 | 12228 |
| Block 2721 | myoK | 2 | 3946676 | 3949481 | e_gw1.7.76.1 | scaffold_7 | 12919 | 15641 |
| Block 1230 | argB | 2 | 3953865 | 3957310 | estExt_Genewise1Plus.C_2750001 | scaffold_275 | 2322 | 5308 |
| Block 1230 | DDB_G0274813 | 2 | 3958144 | 3961274 | estExt_Genewise1Plus.C_2750003 | scaffold_275 | 5792 | 8805 |
| Block 1230 | dlcB | 2 | 3961408 | 3961956 | e_gw1.275.26.1 | scaffold_275 | 8851 | 9280 |
| Block 1230 | tmem32 | 2 | 3962312 | 3962978 | e_gw1.275.23.1 | scaffold_275 | 9497 | 10031 |
| Block 1230 | acgA | 2 | 3963667 | 3966442 | e_gw1.275.10.1 | scaffold_275 | 11050 | 13834 |
| Block 1230 | mtrpS | 2 | 3966523 | 3967656 | GID1.0046668 | scaffold_275 | 13942 | 15174 |
| Block 1230 | DDB_G0274303 | 2 | 3968026 | 3968532 | GID1.0046669 | scaffold_275 | 15407 | 15880 |
| Block 1230 | DDB_G0274305 | 2 | 3968967 | 3970911 | GID1.0046670 | scaffold_275 | 17472 | 19251 |
| Block 1230 | DDB_G0274735 | 2 | 3972236 | 3973477 | estExt_fgeneshDP_pg.C_2750008 | scaffold_275 | 19810 | 21104 |
| Block 2721 | DDB_G0274309 | 2 | 3982874 | 3983930 | estExt_Genewise1Plus.C_70006 | scaffold_7 | 1414 | 2460 |
| Block 2721 | erh | 2 | 3984590 | 3985333 | e_gw1.7.184.1 | scaffold_7 | 400 | 940 |
| Block 2376 | DB10 | 2 | 3986135 | 3988096 | GID1.0050001 | scaffold_549 | 13151 | 14449 |
| Block 3045 | DDB_G0274267 | 2 | 3991368 | 3992657 | gw1.97.34.1 | scaffold_97 | 23430 | 23891 |
| Block 2721 | DDB_G0274273 | 2 | 3996490 | 3997716 | estExt_Genewise1.C_70040 | scaffold_7 | 25945 | 27037 |
| Block 3045 | gefM | 2 | 4011339 | 4014453 | e_gw1.97.16.1 | scaffold_97 | 18331 | 21117 |
| Block 519 | tssc1 | 2 | 4024427 | 4025902 | GID1.0044152 | scaffold_161 | 43559 | 44837 |
| Block 519 | DDB_G0274827 | 2 | 4026024 | 4028953 | gw1.161.24.1 | scaffold_161 | 40615 | 43205 |
| Block 3045 | DDB_G0274283 | 2 | 4031221 | 4032374 | estExt_Genewise1Plus.C_970016 | scaffold_97 | 29151 | 30396 |
| Block 165 | DDB_G0274829 | 2 | 4043880 | 4045327 | GID1.0042835 | scaffold_117 | 42818 | 44169 |
| Block 3045 | DDB_G0274295 | 2 | 4054699 | 4056345 | fgeneshDP_pg.C_scaffold_97000010 | scaffold_97 | 26910 | 28148 |
| Block 3045 | DDB_G0274833 | 2 | 4058436 | 4059473 | fgeneshDP_pg.C_scaffold_97000009 | scaffold_97 | 24210 | 24790 |
| Block 1417 | glgB | 2 | 4075702 | 4078210 | estExt_Genewise1.C_3080016 | scaffold_308 | 16594 | 19315 |
| Block 1417 | ggtA | 2 | 4078334 | 4083606 | fgeneshDP_pg.C_scaffold_308000004 | scaffold_308 | 10376 | 15434 |
| Block 2898 | DDB_G0274253 | 2 | 4096019 | 4096795 | fgeneshDP_pg.C_scaffold_80000026 | scaffold_80 | 58186 | 58928 |
| Block 2898 | DDB_G0274715 | 2 | 4096998 | 4098443 | fgeneshDP_pg.C_scaffold_80000027 | scaffold_80 | 59522 | 60540 |
| Block 2839 | DDB_G0274255 | 2 | 4107028 | 4110496 | estExt_fgeneshDP_pg.C_7720001 | scaffold_772 | 130 | 3397 |
| Block 2839 | DDB_G0274257 | 2 | 4110958 | 4111638 | GID1.0050983 | scaffold_772 | 3584 | 4167 |
| Block 165 | DDB_G0274713 | 2 | 4123627 | 4125363 | GID1.0042837 | scaffold_117 | 45958 | 47439 |
| Block 10 | DDB_G0274265 | 2 | 4125658 | 4127601 | estExt_fgeneshDP_pg.C_10104 | scaffold_1 | 278730 | 280494 |
| Block 10 | DDB_G0274709 | 2 | 4129470 | 4129927 | GID1.0037259 | scaffold_1 | 277564 | 277791 |
| Block 10 | udkB | 2 | 4130463 | 4131290 | e_gw1.1.168.1 | scaffold_1 | 276215 | 277022 |
| Block 10 | fam91 | 2 | 4132762 | 4135722 | GID1.0037257 | scaffold_1 | 272467 | 275172 |
| Block 516 | DDB_G0274251 | 2 | 4139265 | 4140506 | e_gw1.161.32.1 | scaffold_161 | 38476 | 39592 |
| Block 516 | DDB_G0274249 | 2 | 4140717 | 4141216 | GID1.0044149 | scaffold_161 | 37742 | 38315 |
| Block 1231 | prp18 | 2 | 4151258 | 4152427 | GID1.0046673 | scaffold_275 | 23645 | 24882 |
| Block 1231 | vatH | 2 | 4153831 | 4155826 | GID1.0046672 | scaffold_275 | 21250 | 23134 |
| Block 1050 | paxB | 2 | 4160812 | 4162521 | estExt_fgeneshDP_kg.C_240011 | scaffold_24 | 131230 | 133088 |
| Block 1050 | DDB_G0274847 | 2 | 4163999 | 4169898 | estExt_fgeneshDP_pm.C_240019 | scaffold_24 | 134118 | 139246 |
| Block 2737 | DDB_G0274549 | 2 | 4181553 | 4182180 | estExt_fgeneshDP_kg.C_70008 | scaffold_7 | 108355 | 109882 |
| Block 2737 | DDB_G0274547 | 2 | 4183931 | 4185730 | e_gw1.7.88.1 | scaffold_7 | 111355 | 112950 |
| Block 516 | DDB_G0274699 | 2 | 4187622 | 4188059 | GID1.0044143 | scaffold_161 | 25403 | 26058 |
| Block 516 | thrS2 | 2 | 4189324 | 4191420 | e_gw1.161.2.1 | scaffold_161 | 27329 | 29425 |
| Block 516 | taf12 | 2 | 4191460 | 4193639 | gw1.161.14.1 | scaffold_161 | 31096 | 31407 |
| Block 516 | DDB_G0274695 | 2 | 4194284 | 4195864 | estExt_Genewise1.C_1610020 | scaffold_161 | 32590 | 34249 |
| Block 516 | prdx4 | 2 | 4200234 | 4200968 | estExt_Genewise1.C_1610012 | scaffold_161 | 22174 | 23383 |
| Block 6 | gacF | 2 | 4207716 | 4211327 | GID1.0037209 | scaffold_1 | 167182 | 170060 |
| Block 6 | bre1 | 2 | 4213542 | 4217347 | GID1.0037192 | scaffold_1 | 123847 | 127135 |
| Block 6 | eIF1 | 2 | 4218642 | 4219237 | estExt_Genewise1.C_10086 | scaffold_1 | 128561 | 129570 |
| Block 6 | DDB_G0274693 | 2 | 4219919 | 4221580 | GID1.0037194 | scaffold_1 | 130165 | 131862 |
| Block 6 | DDB_G0274691 | 2 | 4224968 | 4227633 | fgeneshDP_pg.C_scaffold_1000106 | scaffold_1 | 281776 | 283888 |
| Block 516 | DDB_G0274689 | 2 | 4231507 | 4232625 | GID1.0044135 | scaffold_161 | 2697 | 3617 |
| Block 516 | DDB_G0274687 | 2 | 4232939 | 4236127 | e_gw1.161.16.1 | scaffold_161 | 5753 | 6535 |
| Block 516 | DDB_G0274865 | 2 | 4239208 | 4240293 | estExt_fgeneshDP_kg.C_1610002 | scaffold_161 | 7959 | 8957 |
| Block 516 | DDB_G0274539 | 2 | 4240701 | 4241459 | estExt_fgeneshDP_kg.C_1610003 | scaffold_161 | 9679 | 10608 |
| Block 516 | DDB_G0274867 | 2 | 4242688 | 4243466 | fgeneshDP_pg.C_scaffold_161000005 | scaffold_161 | 11946 | 12912 |
| Block 2245 | DDB_G0274535 | 2 | 4249570 | 4250937 | GID1.0049695 | scaffold_507 | 6383 | 7375 |
| Block 2245 | DDB_G0274533 | 2 | 4251233 | 4253059 | fgeneshDP_pg.C_scaffold_507000006 | scaffold_507 | 7928 | 9564 |
| Block 2245 | DDB_G0274871 | 2 | 4260104 | 4261589 | GID1.0049697 | scaffold_507 | 11840 | 13396 |
| Block 1044 | rad9 | 2 | 4264823 | 4266467 | e_gw1.24.39.1 | scaffold_24 | 50239 | 51792 |
| Block 1044 | gpgA | 2 | 4271613 | 4271988 | estExt_fgeneshDP_pm.C_240003 | scaffold_24 | 44283 | 45033 |
| Block 597 | DDB_G0274523 | 2 | 4272459 | 4275383 | fgeneshDP_pg.C_scaffold_173000014 | scaffold_173 | 32844 | 35090 |
| Block 597 | DDB_G0274521 | 2 | 4276411 | 4277554 | e_gw1.173.36.1 | scaffold_173 | 30825 | 32039 |
| Block 597 | napA | 2 | 4278933 | 4282676 | GID1.0044464 | scaffold_173 | 26215 | 29832 |
| Block 2744 | DDB_G0274681 | 2 | 4285306 | 4287031 | GID1.0037818 | scaffold_7 | 196513 | 198384 |
| Block 2744 | pex19 | 2 | 4294598 | 4295786 | gw1.7.120.1 | scaffold_7 | 195282 | 196353 |
| Block 2738 | DDB_G0274875 | 2 | 4298174 | 4304028 | estExt_fgeneshDP_pm.C_70025 | scaffold_7 | 121174 | 126622 |
| Block 2738 | DDB_G0274877 | 2 | 4304355 | 4306891 | GID1.0037779 | scaffold_7 | 116430 | 118404 |
| Block 2738 | sfbA | 2 | 4307259 | 4309136 | estExt_fgeneshDP_pg.C_70049 | scaffold_7 | 113399 | 115426 |
| Block 517 | DDB_G0274879 | 2 | 4310976 | 4316495 | estExt_fgeneshDP_pg.C_1610006 | scaffold_161 | 12978 | 18823 |
| Block 1891 | gxcX | 2 | 4335542 | 4339141 | GID1.0048756 | scaffold_411 | 6396 | 9547 |
| Block 159 | DDB_G0274893 | 2 | 4345519 | 4348441 | e_gw1.117.16.1 | scaffold_117 | 9421 | 12095 |
| Block 159 | DDB_G0274897 | 2 | 4350797 | 4354210 | estExt_fgeneshDP_pm.C_1170002 | scaffold_117 | 5469 | 8557 |
| Block 159 | DDB_G0274899 | 2 | 4355083 | 4359978 | estExt_fgeneshDP_pg.C_1170001 | scaffold_117 | 69 | 5073 |
| Block 517 | DDB_G0274379 | 2 | 4362036 | 4362959 | e_gw1.161.39.1 | scaffold_161 | 63572 | 63907 |
| Block 517 | DDB_G0274505 | 2 | 4363158 | 4364848 | gw1.161.10.1 | scaffold_161 | 61432 | 62484 |
| Block 517 | PLK | 2 | 4365581 | 4369322 | estExt_fgeneshDP_pg.C_1610022 | scaffold_161 | 57567 | 60595 |
| Block 1891 | DDB_G0274495 | 2 | 4374270 | 4376904 | GID1.0048757 | scaffold_411 | 10480 | 12994 |
| Block 2743 | lig1 | 2 | 4387143 | 4390846 | e_gw1.7.3.1 | scaffold_7 | 192915 | 194861 |
| Block 2743 | DDB_G0274489 | 2 | 4392026 | 4392628 | fgeneshDP_pg.C_scaffold_7000078 | scaffold_7 | 190807 | 191394 |
| Block 2743 | DDB_G0274487 | 2 | 4393406 | 4395073 | GID1.0037814 | scaffold_7 | 188049 | 189913 |
| Block 2743 | DDB_G0274663 | 2 | 4395230 | 4396693 | GID1.0037808 | scaffold_7 | 177636 | 179287 |
| Block 2743 | DDB_G0274661 | 2 | 4398382 | 4398957 | estExt_fgeneshDP_kg.C_70013 | scaffold_7 | 184765 | 185517 |
| Block 2743 | DDB_G0274915 | 2 | 4429330 | 4435116 | fgeneshDP_pg.C_scaffold_7000071 | scaffold_7 | 172443 | 177526 |
| Block 2743 | DDB_G0274479 | 2 | 4435995 | 4436726 | fgeneshDP_pg.C_scaffold_7000070 | scaffold_7 | 170730 | 171506 |
| Block 2743 | DDB_G0274475 | 2 | 4438378 | 4439592 | estExt_fgeneshDP_pm.C_70030 | scaffold_7 | 169195 | 170391 |
| Block 2743 | DDB_G0274657 | 2 | 4440232 | 4441107 | fgeneshDP_pg.C_scaffold_7000068 | scaffold_7 | 168132 | 168750 |
| Block 2723 | DDB_G0274473 | 2 | 4462831 | 4465274 | GID1.0037741 | scaffold_7 | 36997 | 39509 |
| Block 2723 | tpiA | 2 | 4468057 | 4468921 | estExt_Genewise1.C_70042 | scaffold_7 | 39879 | 40816 |
| Block 162 | mybJ | 2 | 4491578 | 4494024 | GID1.0042831 | scaffold_117 | 32501 | 35032 |
| Block 162 | DDB_G0274461 | 2 | 4495190 | 4496383 | fgeneshDP_pg.C_scaffold_117000013 | scaffold_117 | 36633 | 37785 |
| Block 162 | pfkA | 2 | 4497066 | 4499570 | estExt_Genewise1Plus.C_1170015 | scaffold_117 | 26866 | 29280 |
| Block 615 | DDB_G0274923 | 2 | 4500320 | 4502641 | gw1.176.13.1 | scaffold_176 | 55713 | 58035 |
| Block 615 | DDB_G0274925 | 2 | 4504952 | 4507782 | fgeneshDP_pm.C_scaffold_176000008 | scaffold_176 | 58030 | 60333 |
| Block 2723 | coq9 | 2 | 4511594 | 4512729 | estExt_Genewise1.C_70107 | scaffold_7 | 85218 | 86197 |
| Block 2723 | myoI | 2 | 4520846 | 4528279 | estExt_Genewise1Plus.C_70095 | scaffold_7 | 68442 | 75672 |
| Block 209 | DDB_G0274937 | 2 | 4542990 | 4543832 | GID1.0042982 | scaffold_121 | 72158 | 73731 |
| Block 209 | aslB | 2 | 4544366 | 4546649 | GID1.0042981 | scaffold_121 | 69619 | 71816 |
| Block 1419 | dokA | 2 | 4549616 | 4554631 | GID1.0047255 | scaffold_308 | 33409 | 37889 |
| Block 1419 | DDB_G0274641 | 2 | 4556118 | 4557389 | GID1.0047253 | scaffold_308 | 29582 | 30913 |
| Block 1419 | pyd3 | 2 | 4557923 | 4559284 | GID1.0047252 | scaffold_308 | 27604 | 28965 |
| Block 1419 | scsB | 2 | 4561408 | 4562983 | GID1.0047250 | scaffold_308 | 23718 | 25208 |
| Block 1419 | DDB_G0274447 | 2 | 4563458 | 4564473 | GID1.0047249 | scaffold_308 | 22420 | 23488 |
| Block 2723 | med30 | 2 | 4565565 | 4566423 | e_gw1.7.127.1 | scaffold_7 | 61399 | 62141 |
| Block 2723 | rpl8 | 2 | 4580040 | 4581529 | estExt_fgeneshDP_kg.C_70003 | scaffold_7 | 55844 | 57275 |
| Block 2723 | DDB_G0274439 | 2 | 4582539 | 4582857 | e_gw1.7.186.1 | scaffold_7 | 53564 | 53719 |
| Block 2723 | twfA | 2 | 4583042 | 4584406 | estExt_Genewise1.C_70086 | scaffold_7 | 53771 | 55232 |
| Block 2740 | DDB_G0274435 | 2 | 4590287 | 4592024 | GID1.0037792 | scaffold_7 | 147368 | 148842 |
| Block 2740 | DDB_G0274433 | 2 | 4592733 | 4593984 | GID1.0037793 | scaffold_7 | 149391 | 150549 |
| Block 2740 | DDB_G0274425 | 2 | 4605258 | 4609454 | fgeneshDP_pg.C_scaffold_7000061 | scaffold_7 | 150660 | 154973 |
| Block 2899 | pakF | 2 | 4634757 | 4638287 | GID1.0041535 | scaffold_80 | 82885 | 86442 |
| Block 2899 | DDB_G0274953 | 2 | 4638892 | 4640784 | GID1.0041536 | scaffold_80 | 87053 | 88964 |
| Block 1 | DDB_G0274629 | 2 | 4644502 | 4644711 | GID1.0037146 | scaffold_1 | 636 | 848 |
| Block 1 | DDB_G0274407 | 2 | 4648020 | 4649467 | e_gw1.1.94.1 | scaffold_1 | 1676 | 2850 |
| Block 1 | eif3d | 2 | 4651697 | 4653638 | fgeneshDP_pm.C_scaffold_1000003 | scaffold_1 | 19162 | 21133 |
| Block 1 | DDB_G0274401 | 2 | 4654037 | 4655736 | GID1.0037157 | scaffold_1 | 21377 | 23080 |
| Block 1 | DDB_G0274959 | 2 | 4656148 | 4656831 | fgeneshDP_pg.C_scaffold_1000012 | scaffold_1 | 23557 | 24531 |
| Block 1 | DDB_G0274961 | 2 | 4657028 | 4658483 | fgeneshDP_pg.C_scaffold_1000013 | scaffold_1 | 24660 | 25950 |
| Block 164 | DDB_G0274969 | 2 | 4675613 | 4677882 | estExt_fgeneshDP_pg.C_1170018 | scaffold_117 | 48123 | 50045 |
| Block 164 | adcB | 2 | 4678108 | 4680270 | GID1.0042840 | scaffold_117 | 50126 | 52065 |
| Block 164 | prpf8 | 2 | 4689623 | 4696928 | estExt_fgeneshDP_pg.C_1170020 | scaffold_117 | 53662 | 60949 |
| Block 164 | tcf25 | 2 | 4744257 | 4746576 | GID1.0042844 | scaffold_117 | 68872 | 71101 |
| Block 164 | DDB_G0274981 | 2 | 4747073 | 4749586 | GID1.0042834 | scaffold_117 | 39653 | 42397 |
| Block 1 | DDB_G0274211 | 2 | 4754535 | 4759883 | gw1.1.4.1 | scaffold_1 | 89798 | 94492 |
| Block 1 | DDB_G0274393 | 2 | 4762537 | 4763966 | e_gw1.1.143.1 | scaffold_1 | 175927 | 177309 |
| Block 1 | DDB_G0274207 | 2 | 4765179 | 4769151 | estExt_fgeneshDP_pg.C_10059 | scaffold_1 | 170610 | 175182 |
| Block 1890 | omt3 | 2 | 4779612 | 4781179 | fgeneshDP_pg.C_scaffold_411000002 | scaffold_411 | 4274 | 5883 |
| Block 797 | DDB_G0274201 | 2 | 4784399 | 4785525 | e_gw1.201.28.1 | scaffold_201 | 52819 | 53934 |
| Block 797 | DDB_G0274203 | 2 | 4785655 | 4786471 | GID1.0045141 | scaffold_201 | 54126 | 55000 |
| Block 797 | DDB_G0274205 | 2 | 4786917 | 4787984 | estExt_fgeneshDP_kg.C_2010015 | scaffold_201 | 55438 | 56508 |
| Block 1890 | dhkC | 2 | 4792114 | 4795791 | GID1.0048760 | scaffold_411 | 20842 | 25014 |
| Block 797 | DDB_G0274991 | 2 | 4805511 | 4806029 | estExt_fgeneshDP_kg.C_2010014 | scaffold_201 | 51290 | 52307 |
| Block 1890 | bzpJ | 2 | 4811378 | 4813923 | estExt_fgeneshDP_kg.C_4110001 | scaffold_411 | 1186 | 2502 |
| Block 2078 | pARTf | 2 | 4842740 | 4844712 | GID1.0040068 | scaffold_46 | 97521 | 99573 |
| Block 2078 | dpoA | 2 | 4848760 | 4851124 | estExt_Genewise1Plus.C_460074 | scaffold_46 | 94775 | 97384 |
| Block 1422 | DDB_G0274169 | 2 | 4851501 | 4851845 | e_gw1.31.53.1 | scaffold_31 | 13993 | 14370 |
| Block 1422 | cand1 | 2 | 4852009 | 4855992 | GID1.0039267 | scaffold_31 | 14546 | 18419 |
| Block 1422 | DDB_G0274165 | 2 | 4856535 | 4859018 | estExt_fgeneshDP_pg.C_310008 | scaffold_31 | 20748 | 22970 |
| Block 1422 | DDB_G0275001 | 2 | 4860200 | 4860763 | GID1.0039270 | scaffold_31 | 24033 | 24663 |
| Block 1897 | DDB_G0274385 | 2 | 4862777 | 4864042 | e_gw1.413.7.1 | scaffold_413 | 22211 | 23559 |
| Block 1422 | hisS | 2 | 4865675 | 4867232 | GID1.0039268 | scaffold_31 | 18891 | 20418 |
| Block 1897 | DDB_G0274157 | 2 | 4868002 | 4868388 | estExt_Genewise1Plus.C_4130013 | scaffold_413 | 26776 | 27126 |
| Block 1022 | DDB_G0275005 | 2 | 4884445 | 4889568 | e_gw1.235.6.1 | scaffold_235 | 38191 | 39444 |
| Block 1022 | pdeD | 2 | 4890713 | 4893535 | estExt_fgeneshDP_pg.C_2350014 | scaffold_235 | 43561 | 46757 |
| Block 1022 | DDB_G0274153 | 2 | 4896087 | 4898102 | estExt_fgeneshDP_pm.C_2350008 | scaffold_235 | 48395 | 50461 |
| Block 2459 | DDB_G0274141 | 2 | 4903886 | 4905233 | estExt_Genewise1.C_580043 | scaffold_58 | 58114 | 59403 |
| Block 2459 | vatP | 2 | 4906713 | 4907496 | fgeneshDP_pg.C_scaffold_58000019 | scaffold_58 | 53026 | 54401 |
| Block 263 | cycC | 2 | 4908080 | 4909016 | estExt_Genewise1.C_1280029 | scaffold_128 | 32793 | 33924 |
| Block 263 | DDB_G0274609 | 2 | 4909219 | 4910367 | gw1.128.41.1 | scaffold_128 | 31721 | 32547 |
| Block 263 | captD | 2 | 4926635 | 4928146 | fgeneshDP_pm.C_scaffold_128000009 | scaffold_128 | 48586 | 50017 |
| Block 263 | DDB_G0275237 | 2 | 4928221 | 4930229 | fgeneshDP_pg.C_scaffold_128000014 | scaffold_128 | 46672 | 48441 |
| Block 263 | DDB_G0275239 | 2 | 4931752 | 4939709 | GID1.0043190 | scaffold_128 | 36945 | 44249 |
| Block 2589 | DDB_G0275145 | 2 | 4947242 | 4948810 | estExt_fgeneshDP_pm.C_630004 | scaffold_63 | 17096 | 22264 |
| Block 2589 | DDB_G0275147 | 2 | 4950085 | 4951052 | gw1.63.51.1 | scaffold_63 | 23001 | 23857 |
| Block 2589 | DDB_G0275149 | 2 | 4951571 | 4953844 | GID1.0040810 | scaffold_63 | 24183 | 26294 |
| Block 2914 | DDB_G0275243 | 2 | 4977026 | 4977569 | GID1.0041603 | scaffold_82 | 62442 | 62853 |
| Block 2914 | alyB | 2 | 4992140 | 4993240 | fgeneshDP_pg.C_scaffold_82000020 | scaffold_82 | 63191 | 63907 |
| Block 849 | DDB_G0275213 | 2 | 4996710 | 4997539 | GID1.0038683 | scaffold_21 | 32668 | 33401 |
| Block 849 | srp19 | 2 | 4997904 | 4998622 | e_gw1.21.80.1 | scaffold_21 | 31451 | 32218 |
| Block 849 | DDB_G0275129 | 2 | 4999105 | 5003189 | GID1.0038685 | scaffold_21 | 36844 | 40361 |
| Block 2573 | DDB_G0275107 | 2 | 5011588 | 5013893 | fgeneshDP_pm.C_scaffold_62000012 | scaffold_62 | 98610 | 100977 |
| Block 2573 | commd10 | 2 | 5014263 | 5015075 | e_gw1.62.34.1 | scaffold_62 | 95822 | 96644 |
| Block 926 | DDB_G0275253 | 2 | 5017742 | 5018696 | estExt_fgeneshDP_pm.C_2210002 | scaffold_221 | 4273 | 5323 |
| Block 926 | DDB_G0275109 | 2 | 5020327 | 5022565 | fgeneshDP_pm.C_scaffold_221000006 | scaffold_221 | 30401 | 32348 |
| Block 926 | DDB_G0275111 | 2 | 5023663 | 5024614 | GID1.0045577 | scaffold_221 | 18373 | 19294 |
| Block 926 | top3 | 2 | 5024897 | 5027507 | e_gw1.221.16.1 | scaffold_221 | 15503 | 18126 |
| Block 926 | DDB_G0275113 | 2 | 5027872 | 5029728 | e_gw1.221.21.1 | scaffold_221 | 13188 | 15221 |
| Block 926 | cnrD | 2 | 5030468 | 5031650 | e_gw1.221.24.1 | scaffold_221 | 8039 | 9076 |
| Block 926 | sdhC | 2 | 5032690 | 5033882 | estExt_fgeneshDP_pg.C_2210002 | scaffold_221 | 3156 | 4014 |
| Block 926 | DDB_G0275117 | 2 | 5034378 | 5036419 | estExt_fgeneshDP_kg.C_2210001 | scaffold_221 | 515 | 3081 |
| Block 1146 | DDB_G0275259 | 2 | 5039971 | 5040252 | e_gw1.257.37.1 | scaffold_257 | 46291 | 46554 |
| Block 1146 | sigI | 2 | 5040499 | 5041819 | estExt_fgeneshDP_kg.C_2570004 | scaffold_257 | 45045 | 45871 |
| Block 1146 | DDB_G0275093 | 2 | 5042626 | 5044774 | fgeneshDP_pg.C_scaffold_257000018 | scaffold_257 | 40902 | 43027 |
| Block 849 | mgmt | 2 | 5045296 | 5045913 | fgeneshDP_pg.C_scaffold_21000018 | scaffold_21 | 44878 | 45566 |
| Block 849 | DDB_G0275097 | 2 | 5046445 | 5048700 | estExt_fgeneshDP_pg.C_210017 | scaffold_21 | 42173 | 44391 |
| Block 849 | DDB_G0275099 | 2 | 5048886 | 5050310 | GID1.0038686 | scaffold_21 | 40624 | 41559 |
| Block 1146 | alg8 | 2 | 5055024 | 5057166 | e_gw1.257.17.1 | scaffold_257 | 17181 | 19068 |
| Block 1146 | commd7 | 2 | 5057403 | 5058116 | e_gw1.257.32.1 | scaffold_257 | 19292 | 19987 |
| Block 1146 | DDB_G0275171 | 2 | 5058477 | 5060388 | GID1.0046311 | scaffold_257 | 20217 | 21803 |
| Block 2879 | asnS1 | 2 | 5062272 | 5064483 | estExt_Genewise1.C_80042 | scaffold_8 | 77544 | 80031 |
| Block 2879 | etnkB | 2 | 5065376 | 5066799 | fgeneshDP_pm.C_scaffold_8000005 | scaffold_8 | 62967 | 64273 |
| Block 2879 | DDB_G0275205 | 2 | 5067894 | 5069497 | GID1.0037852 | scaffold_8 | 65067 | 67089 |
| Block 2879 | DDB_G0275169 | 2 | 5070449 | 5074721 | fgeneshDP_pg.C_scaffold_8000029 | scaffold_8 | 67768 | 71780 |
| Block 2879 | DDB_G0275057 | 2 | 5076419 | 5077956 | estExt_Genewise1Plus.C_80036 | scaffold_8 | 72835 | 74586 |
| Block 2879 | DDB_G0275059 | 2 | 5078247 | 5079141 | fgeneshDP_pg.C_scaffold_8000031 | scaffold_8 | 74670 | 75636 |
| Block 2879 | DDB_G0275061 | 2 | 5079325 | 5080770 | fgeneshDP_pg.C_scaffold_8000032 | scaffold_8 | 75875 | 77192 |
| Block 2879 | DDB_G0275063 | 2 | 5081010 | 5081771 | estExt_fgeneshDP_pg.C_80026 | scaffold_8 | 61797 | 62736 |
| Block 2879 | dmpA | 2 | 5083129 | 5088327 | e_gw1.8.39.1 | scaffold_8 | 56240 | 61204 |
| Block 2879 | dstD | 2 | 5089182 | 5091707 | GID1.0037847 | scaffold_8 | 51523 | 53938 |
| Block 2888 | DDB_G0275203 | 2 | 5091989 | 5093921 | e_gw1.8.9.1 | scaffold_8 | 161504 | 162980 |
| Block 2888 | DDB_G0275067 | 2 | 5094206 | 5095366 | GID1.0037895 | scaffold_8 | 164932 | 166573 |
| Block 1782 | fahd1 | 2 | 5105999 | 5106762 | GID1.0039730 | scaffold_39 | 92636 | 93449 |
| Block 1782 | DDB_G0275269 | 2 | 5111542 | 5114550 | fgeneshDP_pm.C_scaffold_39000025 | scaffold_39 | 102009 | 103283 |
| Block 1782 | pwp1 | 2 | 5114842 | 5116551 | e_gw1.39.10.1 | scaffold_39 | 103538 | 105305 |
| Block 1782 | DDB_G0275075 | 2 | 5117418 | 5120536 | GID1.0039737 | scaffold_39 | 105626 | 108556 |
| Block 2224 | DDB_G0275165 | 2 | 5130267 | 5133212 | GID1.0040229 | scaffold_50 | 36434 | 39368 |
| Block 2224 | DDB_G0275081 | 2 | 5135245 | 5135670 | e_gw1.50.25.1 | scaffold_50 | 41650 | 42078 |
| Block 2456 | DDB_G0275083 | 2 | 5139679 | 5140149 | fgeneshDP_pm.C_scaffold_58000013 | scaffold_58 | 59473 | 60120 |
| Block 2456 | gacFF | 2 | 5141120 | 5143970 | GID1.0040587 | scaffold_58 | 61260 | 63960 |
| Block 2822 | DDB_G0275275 | 2 | 5147563 | 5149393 | fgeneshDP_pg.C_scaffold_755000002 | scaffold_755 | 1550 | 3061 |
| Block 2822 | DDB_G0275087 | 2 | 5149683 | 5151296 | fgeneshDP_pg.C_scaffold_755000001 | scaffold_755 | 16 | 1088 |
| Block 1493 | DDB_G0275089 | 2 | 5151468 | 5153317 | GID1.0047515 | scaffold_324 | 575 | 2400 |
| Block 1493 | DDB_G0275277 | 2 | 5154209 | 5156188 | fgeneshDP_pg.C_scaffold_324000002 | scaffold_324 | 3059 | 4990 |
| Block 775 | DDB_G0275091 | 2 | 5158931 | 5161022 | e_gw1.20.34.1 | scaffold_20 | 35109 | 37040 |
| Block 775 | ubpB | 2 | 5164570 | 5166232 | estExt_Genewise1Plus.C_200023 | scaffold_20 | 49763 | 51453 |
| Block 2456 | pikC | 2 | 5198204 | 5203554 | GID1.0040581 | scaffold_58 | 47387 | 52351 |
| Block 2456 | DDB_G0275287 | 2 | 5204918 | 5211779 | estExt_fgeneshDP_pg.C_580008 | scaffold_58 | 18258 | 24517 |
| Block 3053 | DDB_G0275289 | 2 | 5212410 | 5215326 | gw1.98.4.1 | scaffold_98 | 17570 | 18450 |
| Block 3053 | gca | 2 | 5217259 | 5221807 | GID1.0042173 | scaffold_98 | 9129 | 13457 |
| Block 3053 | cmfA | 2 | 5222827 | 5225122 | fgeneshDP_pm.C_scaffold_98000003 | scaffold_98 | 5931 | 8051 |
| Block 3053 | DDB_G0275049 | 2 | 5235880 | 5236964 | fgeneshDP_pg.C_scaffold_98000002 | scaffold_98 | 4174 | 5490 |
| Block 3053 | galE | 2 | 5239680 | 5240979 | estExt_Genewise1Plus.C_980003 | scaffold_98 | 898 | 2459 |
| Block 1510 | gacI | 2 | 5249000 | 5251591 | estExt_Genewise1.C_3280002 | scaffold_328 | 3993 | 6800 |
| Block 1510 | gpbB | 2 | 5253060 | 5254195 | estExt_Genewise1.C_3280004 | scaffold_328 | 8454 | 9699 |
| Block 1412 | DDB_G0275297 | 2 | 5256775 | 5258042 | estExt_Genewise1Plus.C_3070002 | scaffold_307 | 10612 | 12014 |
| Block 1412 | cln5 | 2 | 5258700 | 5259995 | estExt_Genewise1.C_3070005 | scaffold_307 | 12158 | 13468 |
| Block 1494 | DDB_G0275041 | 2 | 5260229 | 5261254 | e_gw1.324.26.1 | scaffold_324 | 8892 | 9794 |
| Block 1494 | DDB_G0275301 | 2 | 5261438 | 5262743 | GID1.0047518 | scaffold_324 | 7728 | 8786 |
| Block 131 | DDB_G0275035 | 2 | 5280206 | 5287159 | GID1.0042683 | scaffold_113 | 24225 | 30958 |
| Block 131 | DDB_G0275305 | 2 | 5288776 | 5297735 | GID1.0042691 | scaffold_113 | 51597 | 59837 |
| Block 2225 | odhB | 2 | 5303412 | 5304931 | estExt_Genewise1.C_500030 | scaffold_50 | 42185 | 43528 |
| Block 2225 | gpdA | 2 | 5311196 | 5312379 | estExt_fgeneshDP_kg.C_500007 | scaffold_50 | 44991 | 46293 |
| Block 2225 | DDB_G0275025 | 2 | 5312636 | 5313948 | fgeneshDP_pg.C_scaffold_50000023 | scaffold_50 | 46480 | 47688 |
| Block 2225 | DDB_G0275181 | 2 | 5314092 | 5314707 | GID1.0040235 | scaffold_50 | 48635 | 49197 |
| Block 2225 | DDB_G0275311 | 2 | 5315557 | 5317702 | estExt_fgeneshDP_kg.C_500010 | scaffold_50 | 50576 | 52476 |
| Block 1037 | dhx9 | 2 | 5318243 | 5323206 | GID1.0045934 | scaffold_239 | 1168 | 5786 |
| Block 387 | DDB_G0275317 | 2 | 5326031 | 5327140 | estExt_fgeneshDP_kg.C_1430008 | scaffold_143 | 69317 | 70932 |
| Block 387 | DDB_G0275179 | 2 | 5332367 | 5334080 | estExt_Genewise1.C_1430053 | scaffold_143 | 66682 | 68917 |
| Block 776 | DDB_G0275329 | 2 | 5344037 | 5344733 | estExt_Genewise1Plus.C_200029 | scaffold_20 | 52818 | 53556 |
| Block 776 | eIF4g | 2 | 5345079 | 5349368 | fgeneshDP_pg.C_scaffold_20000014 | scaffold_20 | 43392 | 47247 |
| Block 776 | DDB_G0275331 | 2 | 5352360 | 5355896 | GID1.0038617 | scaffold_20 | 37618 | 41018 |
| Block 776 | DDB_G0275335 | 2 | 5368550 | 5369291 | e_gw1.20.97.1 | scaffold_20 | 54101 | 54596 |
| Block 776 | DDB_G0275337 | 2 | 5369654 | 5370811 | fgeneshDP_pg.C_scaffold_20000019 | scaffold_20 | 54963 | 56099 |
| Block 776 | DDB_G0275401 | 2 | 5371826 | 5377182 | fgeneshDP_pg.C_scaffold_20000020 | scaffold_20 | 56606 | 61505 |
| Block 57 | DDB_G0275339 | 2 | 5377530 | 5378837 | fgeneshDP_pg.C_scaffold_103000030 | scaffold_103 | 64124 | 65764 |
| Block 57 | DDB_G0275341 | 2 | 5379082 | 5380253 | fgeneshDP_pm.C_scaffold_103000009 | scaffold_103 | 65940 | 67185 |
| Block 57 | DDB_G0275343 | 2 | 5380336 | 5381973 | e_gw1.103.28.1 | scaffold_103 | 67268 | 68899 |
| Block 1037 | DDB_G0275345 | 2 | 5383587 | 5385970 | GID1.0045935 | scaffold_239 | 6074 | 7951 |
| Block 2677 | DDB_G0275349 | 2 | 5387837 | 5388154 | e_gw1.667.10.1 | scaffold_667 | 8546 | 8839 |
| Block 2677 | DDB_G0275351 | 2 | 5388788 | 5389451 | e_gw1.667.7.1 | scaffold_667 | 7418 | 7900 |
| Block 2677 | DDB_G0275403 | 2 | 5389611 | 5391918 | e_gw1.667.1.1 | scaffold_667 | 5040 | 7170 |
| Block 2677 | tipD | 2 | 5392534 | 5394449 | GID1.0050635 | scaffold_667 | 2586 | 4444 |
| Block 457 | DDB_G0275353 | 2 | 5396406 | 5398039 | e_gw1.151.29.1 | scaffold_151 | 65523 | 66466 |
| Block 457 | ap3s1 | 2 | 5398448 | 5399309 | e_gw1.151.46.1 | scaffold_151 | 64365 | 65144 |
| Block 457 | pccA | 2 | 5399832 | 5402247 | estExt_Genewise1Plus.C_1510042 | scaffold_151 | 60987 | 63549 |
| Block 457 | DDB_G0275409 | 2 | 5408276 | 5410781 | estExt_fgeneshDP_pg.C_1510013 | scaffold_151 | 26545 | 28130 |
| Block 457 | DDB_G0275359 | 2 | 5410997 | 5412385 | fgeneshDP_pg.C_scaffold_151000014 | scaffold_151 | 28350 | 29741 |
| Block 457 | DDB_G0275365 | 2 | 5415007 | 5417291 | estExt_Genewise1Plus.C_1510027 | scaffold_151 | 30709 | 33228 |
| Block 457 | DDB_G0275411 | 2 | 5417692 | 5418811 | estExt_fgeneshDP_kg.C_1510004 | scaffold_151 | 33253 | 34736 |
| Block 457 | DDB_G0275367 | 2 | 5421141 | 5421511 | estExt_fgeneshDP_kg.C_1510005 | scaffold_151 | 36989 | 37555 |
| Block 457 | DDB_G0275413 | 2 | 5421883 | 5425883 | GID1.0043866 | scaffold_151 | 37818 | 41434 |
| Block 457 | DDB_G0275415 | 2 | 5426870 | 5436383 | estExt_fgeneshDP_pg.C_1510018 | scaffold_151 | 42419 | 51483 |
| Block 457 | DDB_G0275417 | 2 | 5436896 | 5438103 | fgeneshDP_pg.C_scaffold_151000019 | scaffold_151 | 51860 | 52775 |
| Block 112 | DDB_G0275375 | 2 | 5457102 | 5461460 | e_gw1.110.13.1 | scaffold_110 | 18088 | 22364 |
| Block 112 | DDB_G0275377 | 2 | 5462215 | 5463153 | fgeneshDP_pm.C_scaffold_110000006 | scaffold_110 | 16531 | 17466 |
| Block 112 | DDB_G0275379 | 2 | 5463452 | 5463733 | GID1.0042588 | scaffold_110 | 15944 | 16228 |
| Block 112 | DDB_G0275381 | 2 | 5464164 | 5464540 | fgeneshDP_kg.C_scaffold_110000005 | scaffold_110 | 15252 | 15711 |
| Block 112 | syn8A | 2 | 5465381 | 5465992 | estExt_fgeneshDP_pg.C_1100006 | scaffold_110 | 13611 | 14322 |
| Block 112 | DDB_G0275383 | 2 | 5466499 | 5466854 | estExt_Genewise1Plus.C_1100010 | scaffold_110 | 12932 | 13588 |
| Block 112 | DDB_G0275385 | 2 | 5468622 | 5469383 | GID1.0042584 | scaffold_110 | 11042 | 11771 |
| Block 112 | DDB_G0275431 | 2 | 5469666 | 5474561 | GID1.0042583 | scaffold_110 | 6920 | 10817 |
| Block 112 | DDB_G0275433 | 2 | 5475082 | 5475566 | GID1.0042582 | scaffold_110 | 5631 | 6052 |
| Block 1590 | msh2 | 2 | 5499559 | 5502718 | estExt_Genewise1.C_3440002 | scaffold_344 | 3768 | 6803 |
| Block 1590 | amd1 | 2 | 5504223 | 5505870 | estExt_Genewise1.C_3440004 | scaffold_344 | 7760 | 10045 |
| Block 564 | vps32 | 2 | 5512109 | 5512961 | estExt_Genewise1Plus.C_1690027 | scaffold_169 | 62847 | 63793 |
| Block 564 | DDB_G0275813 | 2 | 5513230 | 5515473 | estExt_Genewise1Plus.C_1690025 | scaffold_169 | 60366 | 62556 |
| Block 383 | DDB_G0275575 | 2 | 5516482 | 5517465 | GID1.0043646 | scaffold_143 | 28963 | 29658 |
| Block 383 | DDB_G0275693 | 2 | 5517857 | 5520139 | GID1.0043645 | scaffold_143 | 26230 | 28682 |
| Block 2457 | DDB_G0275577 | 2 | 5520301 | 5522466 | fgeneshDP_pm.C_scaffold_58000008 | scaffold_58 | 26272 | 28329 |
| Block 2457 | DDB_G0275815 | 2 | 5523034 | 5524923 | gw1.58.1.1 | scaffold_58 | 29294 | 30673 |
| Block 2457 | DDB_G0275821 | 2 | 5528419 | 5530800 | GID1.0040576 | scaffold_58 | 31020 | 33821 |
| Block 2457 | DDB_G0275823 | 2 | 5532993 | 5536729 | GID1.0040578 | scaffold_58 | 35040 | 38250 |
| Block 2457 | DDB_G0275581 | 2 | 5537004 | 5539739 | fgeneshDP_pg.C_scaffold_58000014 | scaffold_58 | 38603 | 41077 |
| Block 2457 | DDB_G0275583 | 2 | 5540226 | 5540954 | e_gw1.58.44.1 | scaffold_58 | 45183 | 45857 |
| Block 2588 | DDB_G0275591 | 2 | 5549208 | 5551289 | estExt_fgeneshDP_kg.C_630002 | scaffold_63 | 28129 | 29000 |
| Block 2588 | DDB_G0275805 | 2 | 5552132 | 5554700 | estExt_Genewise1.C_630021 | scaffold_63 | 29314 | 31910 |
| Block 2588 | DDB_G0275593 | 2 | 5554792 | 5556012 | GID1.0040806 | scaffold_63 | 14538 | 15737 |
| Block 2588 | DDB_G0275595 | 2 | 5558363 | 5560414 | fgeneshDP_pg.C_scaffold_63000006 | scaffold_63 | 12389 | 14360 |
| Block 2588 | DDB_G0275599 | 2 | 5564988 | 5565755 | fgeneshDP_pg.C_scaffold_63000003 | scaffold_63 | 6801 | 7559 |
| Block 383 | DDB_G0275603 | 2 | 5569957 | 5572046 | e_gw1.143.13.1 | scaffold_143 | 63155 | 63695 |
| Block 383 | abcH3 | 2 | 5576898 | 5580254 | gw1.143.15.1 | scaffold_143 | 58542 | 61661 |
| Block 383 | DDB_G0275795 | 2 | 5582769 | 5585296 | estExt_fgeneshDP_kg.C_1430005 | scaffold_143 | 53123 | 53979 |
| Block 383 | myoD | 2 | 5586740 | 5590357 | e_gw1.143.1.1 | scaffold_143 | 48118 | 51805 |
| Block 1511 | DDB_G0275613 | 2 | 5593262 | 5594749 | gw1.328.3.1 | scaffold_328 | 9834 | 11247 |
| Block 1511 | gnt1 | 2 | 5600379 | 5601765 | fgeneshDP_pm.C_scaffold_328000004 | scaffold_328 | 11461 | 12578 |
| Block 832 | DDB_G0275787 | 2 | 5633340 | 5634501 | estExt_Genewise1Plus.C_2070006 | scaffold_207 | 23684 | 24740 |
| Block 1557 | DDB_G0275551 | 2 | 5642348 | 5642807 | estExt_fgeneshDP_kg.C_3390002 | scaffold_339 | 28984 | 30395 |
| Block 1557 | DDB_G0275843 | 2 | 5644351 | 5647666 | estExt_Genewise1Plus.C_3390016 | scaffold_339 | 31234 | 35136 |
| Block 1557 | smg1 | 2 | 5652271 | 5659508 | GID1.0047753 | scaffold_339 | 9090 | 16154 |
| Block 12 | DDB_G0275847 | 2 | 5659772 | 5660844 | e_gw1.10.80.1 | scaffold_10 | 22689 | 23640 |
| Block 12 | DDB_G0275849 | 2 | 5663340 | 5663958 | estExt_fgeneshDP_kg.C_100004 | scaffold_10 | 18712 | 19561 |
| Block 12 | DDB_G0275851 | 2 | 5664434 | 5665493 | GID1.0037978 | scaffold_10 | 17476 | 18429 |
| Block 12 | vatE | 2 | 5665740 | 5666762 | estExt_Genewise1Plus.C_100018 | scaffold_10 | 16028 | 17128 |
| Block 12 | DDB_G0275777 | 2 | 5668155 | 5670887 | GID1.0037976 | scaffold_10 | 11828 | 14475 |
| Block 12 | DDB_G0275555 | 2 | 5671366 | 5672088 | e_gw1.10.95.1 | scaffold_10 | 10652 | 11368 |
| Block 12 | DDB_G0275671 | 2 | 5673471 | 5674799 | GID1.0037973 | scaffold_10 | 7869 | 9194 |
| Block 12 | psmD4 | 2 | 5675617 | 5677400 | estExt_Genewise1.C_100007 | scaffold_10 | 6104 | 7583 |
| Block 12 | DDB_G0275853 | 2 | 5677648 | 5680517 | estExt_Genewise1Plus.C_100005 | scaffold_10 | 3448 | 6064 |
| Block 12 | DDB_G0275673 | 2 | 5681295 | 5681657 | fgeneshDP_pg.C_scaffold_10000002 | scaffold_10 | 2531 | 2902 |
| Block 12 | rab32C | 2 | 5682462 | 5683136 | estExt_fgeneshDP_kg.C_100001 | scaffold_10 | 523 | 1822 |
| Block 486 | DDB_G0275773 | 2 | 5702781 | 5704543 | estExt_fgeneshDP_pm.C_1550004 | scaffold_155 | 57372 | 59817 |
| Block 486 | DDB_G0275683 | 2 | 5705654 | 5706860 | estExt_fgeneshDP_pg.C_1550020 | scaffold_155 | 60718 | 61914 |
| Block 1528 | cytC | 2 | 5711136 | 5711896 | estExt_Genewise1.C_3310003 | scaffold_331 | 8258 | 9065 |
| Block 1528 | DDB_G0275539 | 2 | 5713504 | 5717587 | estExt_fgeneshDP_pg.C_3310002 | scaffold_331 | 4490 | 7865 |
| Block 1528 | DDB_G0275541 | 2 | 5718400 | 5719581 | GID1.0047633 | scaffold_331 | 1390 | 2466 |
| Block 1528 | DDB_G0275765 | 2 | 5721870 | 5722528 | fgeneshDP_pg.C_scaffold_331000001 | scaffold_331 | 509 | 1169 |
| Block 1185 | DDB_G0275543 | 2 | 5723020 | 5724123 | fgeneshDP_pg.C_scaffold_264000001 | scaffold_264 | 732 | 1833 |
| Block 1185 | gefN | 2 | 5727548 | 5731261 | fgeneshDP_pg.C_scaffold_264000002 | scaffold_264 | 4454 | 8978 |
| Block 1185 | DDB_G0275545 | 2 | 5740633 | 5740926 | GID1.0046457 | scaffold_264 | 25448 | 25747 |
| Block 1185 | abcG2 | 2 | 5747021 | 5751204 | estExt_fgeneshDP_pg.C_2640005 | scaffold_264 | 20447 | 24964 |
| Block 933 | helB1 | 2 | 5767120 | 5770678 | e_gw1.222.10.1 | scaffold_222 | 49361 | 51163 |
| Block 933 | DDB_G0275669 | 2 | 5770969 | 5772831 | e_gw1.222.9.1 | scaffold_222 | 45951 | 47474 |
| Block 3052 | DDB_G0275667 | 2 | 5773953 | 5774963 | e_gw1.97.3.1 | scaffold_97 | 79305 | 80266 |
| Block 3052 | DDB_G0275665 | 2 | 5775955 | 5776866 | GID1.0042168 | scaffold_97 | 81867 | 82796 |
| Block 1963 | DDB_G0275871 | 2 | 5782758 | 5786395 | estExt_fgeneshDP_pm.C_430016 | scaffold_43 | 114011 | 117902 |
| Block 1963 | DDB_G0275661 | 2 | 5793058 | 5794446 | GID1.0039933 | scaffold_43 | 111355 | 112927 |
| Block 1185 | elp2 | 2 | 5834298 | 5837126 | GID1.0046455 | scaffold_264 | 15277 | 17936 |
| Block 556 | psmD9 | 2 | 5838066 | 5839444 | e_gw1.168.81.1 | scaffold_168 | 9960 | 10828 |
| Block 556 | DDB_G0275649 | 2 | 5840630 | 5842875 | GID1.0044321 | scaffold_168 | 12587 | 14292 |
| Block 556 | natA | 2 | 5843174 | 5844215 | GID1.0044322 | scaffold_168 | 14948 | 15884 |
| Block 556 | DDB_G0275647 | 2 | 5844630 | 5845219 | e_gw1.168.6.1 | scaffold_168 | 16122 | 16600 |
| Block 556 | acn9 | 2 | 5845547 | 5845954 | fgeneshDP_pg.C_scaffold_168000010 | scaffold_168 | 16824 | 17297 |
| Block 556 | DDB_G0275751 | 2 | 5846136 | 5849271 | estExt_fgeneshDP_pg.C_1680011 | scaffold_168 | 17367 | 20460 |
| Block 556 | DDB_G0275749 | 2 | 5849684 | 5850597 | e_gw1.168.38.1 | scaffold_168 | 20930 | 21939 |
| Block 2466 | DDB_G0275535 | 2 | 5855173 | 5859004 | estExt_Genewise1.C_5830002 | scaffold_583 | 1827 | 5587 |
| Block 556 | DDB_G0275747 | 2 | 5859225 | 5862047 | estExt_fgeneshDP_pg.C_1680004 | scaffold_168 | 4076 | 6604 |
| Block 556 | rpl13a | 2 | 5862942 | 5864085 | estExt_fgeneshDP_pg.C_1680003 | scaffold_168 | 1968 | 3203 |
| Block 2466 | dio3 | 2 | 5871153 | 5871926 | e_gw1.583.7.1 | scaffold_583 | 12595 | 13365 |
| Block 2466 | DDB_G0275885 | 2 | 5872215 | 5873899 | fgeneshDP_pg.C_scaffold_583000004 | scaffold_583 | 11264 | 12292 |
| Block 1185 | DDB_G0275739 | 2 | 5874086 | 5876119 | GID1.0046460 | scaffold_264 | 33216 | 35047 |
| Block 1185 | DDB_G0275737 | 2 | 5886283 | 5888286 | estExt_fgeneshDP_pm.C_2640007 | scaffold_264 | 42512 | 45099 |
| Block 556 | masA | 2 | 5889548 | 5891263 | estExt_Genewise1.C_1680007 | scaffold_168 | 6612 | 9588 |
| Block 483 | med26 | 2 | 5901425 | 5908686 | fgeneshDP_pg.C_scaffold_155000009 | scaffold_155 | 24557 | 29128 |
| Block 483 | hspK | 2 | 5908923 | 5909735 | fgeneshDP_pg.C_scaffold_155000010 | scaffold_155 | 29435 | 30234 |
| Block 483 | DDB_G0275889 | 2 | 5910894 | 5915388 | estExt_Genewise1Plus.C_1550019 | scaffold_155 | 31228 | 35251 |
| Block 215 | DDB_G0275621 | 2 | 5920956 | 5922536 | GID1.0042996 | scaffold_122 | 34777 | 36354 |
| Block 215 | mcm4 | 2 | 5922676 | 5925336 | GID1.0042997 | scaffold_122 | 36429 | 39137 |
| Block 2008 | cchl | 2 | 5926113 | 5926904 | e_gw1.440.16.1 | scaffold_440 | 8402 | 9127 |
| Block 2008 | DDB_G0275731 | 2 | 5927091 | 5931648 | GID1.0049080 | scaffold_440 | 9314 | 13521 |
| Block 2033 | alxA | 2 | 5935176 | 5937972 | GID1.0039999 | scaffold_45 | 20456 | 23124 |
| Block 2033 | sky1 | 2 | 5938373 | 5940343 | estExt_fgeneshDP_kg.C_450002 | scaffold_45 | 23665 | 24708 |
| Block 2033 | DDB_G0275631 | 2 | 5943376 | 5945682 | estExt_fgeneshDP_pg.C_450012 | scaffold_45 | 27636 | 31055 |
| Block 2033 | DDB_G0275633 | 2 | 5958751 | 5962256 | estExt_Genewise1Plus.C_450040 | scaffold_45 | 67208 | 70781 |
| Block 2033 | DDB_G0275901 | 2 | 5962502 | 5962785 | e_gw1.45.70.1 | scaffold_45 | 66870 | 67079 |
| Block 2033 | DDB_G0275491 | 2 | 5984103 | 5984351 | fgeneshDP_pg.C_scaffold_45000025 | scaffold_45 | 65183 | 65548 |
| Block 2033 | lsm6 | 2 | 5985367 | 5985738 | gw1.45.21.1 | scaffold_45 | 63724 | 63912 |
| Block 2033 | cycB | 2 | 5986642 | 5988080 | estExt_Genewise1Plus.C_450033 | scaffold_45 | 61548 | 63288 |
| Block 2033 | DDB_G0275495 | 2 | 5990194 | 5992689 | GID1.0040016 | scaffold_45 | 57399 | 59600 |
| Block 2033 | thoc1 | 2 | 5995833 | 5998580 | e_gw1.45.38.1 | scaffold_45 | 54786 | 56948 |
| Block 2033 | DDB_G0275909 | 2 | 5999452 | 5999844 | GID1.0040013 | scaffold_45 | 53830 | 54255 |
| Block 2033 | omt6 | 2 | 6002314 | 6003084 | estExt_Genewise1Plus.C_450029 | scaffold_45 | 51763 | 52642 |
| Block 88 | DDB_G0275505 | 2 | 6004913 | 6008718 | GID1.0042534 | scaffold_108 | 22661 | 26208 |
| Block 88 | DDB_G0275509 | 2 | 6010147 | 6012753 | fgeneshDP_pg.C_scaffold_108000009 | scaffold_108 | 16389 | 18329 |
| Block 88 | DDB_G0275513 | 2 | 6019814 | 6020251 | GID1.0042532 | scaffold_108 | 18772 | 19206 |
| Block 88 | DDB_G0275917 | 2 | 6020977 | 6024147 | estExt_fgeneshDP_pg.C_1080011 | scaffold_108 | 19510 | 22611 |
| Block 1058 | DDB_G0275919 | 2 | 6024229 | 6026592 | fgeneshDP_pg.C_scaffold_241000010 | scaffold_241 | 22575 | 24350 |
| Block 1058 | nhe1 | 2 | 6028712 | 6031072 | fgeneshDP_pg.C_scaffold_241000012 | scaffold_241 | 30424 | 32506 |
| Block 1058 | PIPkinA | 2 | 6032137 | 6036988 | e_gw1.241.40.1 | scaffold_241 | 24765 | 25943 |
| Block 1058 | abcF3 | 2 | 6043301 | 6045349 | e_gw1.241.22.1 | scaffold_241 | 32832 | 34772 |
| Block 1363 | DDB_G0275923 | 2 | 6045593 | 6046186 | fgeneshDP_pg.C_scaffold_3000043 | scaffold_3 | 114638 | 115158 |
| Block 1363 | gtf2h3 | 2 | 6046326 | 6047769 | gw1.3.26.1 | scaffold_3 | 115487 | 116550 |
| Block 1058 | DDB_G0275519 | 2 | 6048553 | 6049576 | estExt_fgeneshDP_pm.C_2410001 | scaffold_241 | 6310 | 7460 |
| Block 1058 | wdr68 | 2 | 6050662 | 6052207 | GID1.0045978 | scaffold_241 | 20408 | 21566 |
| Block 1058 | DDB_G0275521 | 2 | 6052881 | 6054412 | gw1.241.28.1 | scaffold_241 | 18596 | 19774 |
| Block 324 | DDB_G0275523 | 2 | 6054525 | 6057197 | fgeneshDP_pg.C_scaffold_136000006 | scaffold_136 | 13684 | 16332 |
| Block 1058 | DDB_G0275929 | 2 | 6060863 | 6065293 | estExt_Genewise1Plus.C_2410015 | scaffold_241 | 13898 | 18335 |
| Block 324 | ncsA | 2 | 6074654 | 6075442 | fgeneshDP_pm.C_scaffold_136000003 | scaffold_136 | 21168 | 21872 |
| Block 2708 | rps24 | 2 | 6076150 | 6076783 | estExt_Genewise1Plus.C_6840010 | scaffold_684 | 4833 | 5437 |
| Block 2708 | DDB_G0275933 | 2 | 6077245 | 6077812 | GID1.0050699 | scaffold_684 | 4001 | 4417 |
| Block 2708 | tbpA | 2 | 6078639 | 6079457 | estExt_fgeneshDP_kg.C_6840001 | scaffold_684 | 1998 | 3584 |
| Block 2708 | CSN8 | 2 | 6080636 | 6081602 | GID1.0050697 | scaffold_684 | 723 | 1549 |
| Block 606 | DDB_G0275469 | 2 | 6083128 | 6084744 | GID1.0044503 | scaffold_175 | 29 | 1540 |
| Block 606 | pppB | 2 | 6093317 | 6094282 | estExt_fgeneshDP_kg.C_1750001 | scaffold_175 | 3759 | 4980 |
| Block 606 | srp54 | 2 | 6096542 | 6098278 | GID1.0044506 | scaffold_175 | 6238 | 7864 |
| Block 606 | DDB_G0275457 | 2 | 6098905 | 6099684 | estExt_fgeneshDP_kg.C_1750003 | scaffold_175 | 8481 | 9221 |
| Block 606 | DDB_G0275459 | 2 | 6099955 | 6101055 | gw1.175.15.1 | scaffold_175 | 10191 | 10694 |
| Block 2708 | DDB_G0275467 | 2 | 6106225 | 6108420 | GID1.0050701 | scaffold_684 | 5558 | 7452 |
| Block 2872 | mbtps2 | 2 | 6110020 | 6112082 | fgeneshDP_pm.C_scaffold_795000001 | scaffold_795 | 437 | 2247 |
| Block 213 | DDB_G0275983 | 2 | 6133072 | 6134777 | fgeneshDP_pg.C_scaffold_122000008 | scaffold_122 | 17644 | 19280 |
| Block 213 | DDB_G0275981 | 2 | 6135182 | 6136930 | fgeneshDP_pg.C_scaffold_122000009 | scaffold_122 | 19913 | 21624 |
| Block 2220 | DDB_G0275973 | 2 | 6159783 | 6160731 | fgeneshDP_pg.C_scaffold_50000003 | scaffold_50 | 3267 | 4254 |
| Block 2220 | DDB_G0275971 | 2 | 6161664 | 6164041 | estExt_fgeneshDP_pg.C_500002 | scaffold_50 | 1302 | 2509 |
| Block 2007 | DDB_G0275967 | 2 | 6174382 | 6176681 | GID1.0049083 | scaffold_440 | 18138 | 20470 |
| Block 2872 | DDB_G0275959 | 2 | 6201334 | 6202215 | e_gw1.795.5.1 | scaffold_795 | 2638 | 3479 |
| Block 1973 | DDB_G0276227 | 2 | 6209085 | 6212043 | fgeneshDP_pg.C_scaffold_434000006 | scaffold_434 | 9686 | 12205 |
| Block 219 | clcC | 2 | 6214512 | 6217305 | GID1.0043021 | scaffold_123 | 9776 | 12400 |
| Block 219 | xrn1 | 2 | 6219034 | 6224717 | fgeneshDP_pm.C_scaffold_123000003 | scaffold_123 | 4393 | 5076 |
| Block 219 | DDB_G0276133 | 2 | 6227281 | 6229349 | GID1.0043019 | scaffold_123 | 1442 | 3121 |
| Block 2007 | DDB_G0276131 | 2 | 6229733 | 6230936 | fgeneshDP_pg.C_scaffold_440000003 | scaffold_440 | 4035 | 5189 |
| Block 2007 | cdc123 | 2 | 6231055 | 6232436 | GID1.0049077 | scaffold_440 | 5302 | 6648 |
| Block 2007 | DDB_G0276127 | 2 | 6233660 | 6234172 | estExt_fgeneshDP_pg.C_4400005 | scaffold_440 | 7547 | 8080 |
| Block 2141 | DDB_G0276121 | 2 | 6243100 | 6245241 | e_gw1.479.4.1 | scaffold_479 | 11584 | 13434 |
| Block 2141 | cnrF | 2 | 6245903 | 6248863 | GID1.0049453 | scaffold_479 | 15578 | 18469 |
| Block 992 | DDB_G0276117 | 2 | 6248939 | 6249615 | GID1.0045777 | scaffold_230 | 10120 | 10915 |
| Block 992 | DDB_G0276223 | 2 | 6249978 | 6252163 | GID1.0045778 | scaffold_230 | 11253 | 13287 |
| Block 992 | cct8 | 2 | 6252408 | 6254269 | GID1.0045779 | scaffold_230 | 13400 | 15214 |
| Block 992 | dph5 | 2 | 6254929 | 6255750 | e_gw1.230.21.1 | scaffold_230 | 15809 | 16630 |
| Block 1973 | DDB_G0276113 | 2 | 6255847 | 6256648 | GID1.0049005 | scaffold_434 | 5151 | 5912 |
| Block 1973 | DDB_G0276111 | 2 | 6257257 | 6258504 | GID1.0049006 | scaffold_434 | 6422 | 7749 |
| Block 2011 | DDB_G0276107 | 2 | 6274880 | 6277131 | estExt_fgeneshDP_pg.C_4410007 | scaffold_441 | 21137 | 23522 |
| Block 2011 | gtr2 | 2 | 6278756 | 6286177 | GID1.0049088 | scaffold_441 | 12126 | 19250 |
| Block 2011 | enlA | 2 | 6287576 | 6292087 | estExt_fgeneshDP_pg.C_4410004 | scaffold_441 | 5713 | 10763 |
| Block 2031 | DDB_G0276221 | 2 | 6294216 | 6298970 | estExt_fgeneshDP_pm.C_450008 | scaffold_45 | 32639 | 37113 |
| Block 971 | pdeE | 2 | 6299752 | 6303322 | estExt_Genewise1Plus.C_2280039 | scaffold_228 | 37713 | 41071 |
| Block 2031 | nsfA | 2 | 6305185 | 6307529 | estExt_Genewise1.C_450009 | scaffold_45 | 15648 | 18422 |
| Block 971 | DDB_G0276215 | 2 | 6329242 | 6331560 | estExt_fgeneshDP_kg.C_2280005 | scaffold_228 | 48045 | 50630 |
| Block 2031 | DDB_G0276213 | 2 | 6342477 | 6344154 | gw1.45.44.1 | scaffold_45 | 11312 | 12642 |
| Block 2031 | DDB_G0276087 | 2 | 6345228 | 6346562 | e_gw1.45.49.1 | scaffold_45 | 13628 | 15148 |
| Block 2031 | DDB_G0276085 | 2 | 6346773 | 6349211 | fgeneshDP_pg.C_scaffold_45000005 | scaffold_45 | 9048 | 10913 |
| Block 1436 | DDB_G0276193 | 2 | 6374235 | 6376003 | estExt_Genewise1Plus.C_3110004 | scaffold_311 | 12111 | 13975 |
| Block 1436 | DDB_G0276189 | 2 | 6377269 | 6378579 | fgeneshDP_pm.C_scaffold_311000002 | scaffold_311 | 10168 | 11285 |
| Block 1436 | DDB_G0276149 | 2 | 6386663 | 6387794 | estExt_fgeneshDP_pg.C_3110003 | scaffold_311 | 7638 | 9788 |
| Block 480 | DDB_G0276183 | 2 | 6395035 | 6395934 | GID1.0043968 | scaffold_155 | 688 | 1280 |
| Block 480 | DDB_G0276181 | 2 | 6396502 | 6401346 | fgeneshDP_pg.C_scaffold_155000002 | scaffold_155 | 1696 | 5665 |
| Block 480 | DDB_G0276083 | 2 | 6401967 | 6403331 | gw1.155.4.1 | scaffold_155 | 6715 | 7878 |
| Block 480 | atp5e | 2 | 6403629 | 6403975 | GID1.0043971 | scaffold_155 | 8224 | 8532 |
| Block 480 | DDB_G0276243 | 2 | 6404608 | 6407725 | e_gw1.155.22.1 | scaffold_155 | 8810 | 11825 |
| Block 570 | DDB_G0276081 | 2 | 6407875 | 6408645 | GID1.0038451 | scaffold_17 | 68243 | 68875 |
| Block 570 | mproS | 2 | 6408994 | 6411018 | fgeneshDP_pg.C_scaffold_17000026 | scaffold_17 | 66142 | 67962 |
| Block 480 | DG1040 | 2 | 6422134 | 6430256 | GID1.0043983 | scaffold_155 | 40851 | 48144 |
| Block 211 | ddcB | 2 | 6435122 | 6436509 | GID1.0042986 | scaffold_122 | 9777 | 11140 |
| Block 211 | DDB_G0276065 | 2 | 6436714 | 6438795 | GID1.0042985 | scaffold_122 | 6671 | 9599 |
| Block 1106 | AAC3 | 2 | 6448201 | 6450417 | e_gw1.250.7.1 | scaffold_250 | 38996 | 40864 |
| Block 1106 | DDB_G0276247 | 2 | 6450589 | 6451091 | e_gw1.250.16.1 | scaffold_250 | 41135 | 41509 |
| Block 570 | DDB_G0276051 | 2 | 6470835 | 6471896 | GID1.0038456 | scaffold_17 | 80261 | 81213 |
| Block 1439 | DDB_G0276047 | 2 | 6475432 | 6482377 | GID1.0047316 | scaffold_311 | 24953 | 31827 |
| Block 1439 | DDB_G0276169 | 2 | 6484204 | 6485692 | e_gw1.311.7.1 | scaffold_311 | 23617 | 24569 |
| Block 480 | DDB_G0276251 | 2 | 6486769 | 6491097 | GID1.0043982 | scaffold_155 | 36233 | 40291 |
| Block 2890 | DDB_G0276253 | 2 | 6491319 | 6493574 | GID1.0041500 | scaffold_80 | 6871 | 8812 |
| Block 2890 | DDB_G0276255 | 2 | 6494207 | 6496810 | GID1.0041499 | scaffold_80 | 3728 | 6090 |
| Block 2088 | ifkC | 2 | 6497249 | 6502434 | GID1.0049308 | scaffold_464 | 5896 | 10847 |
| Block 2088 | DDB_G0276167 | 2 | 6503388 | 6504835 | GID1.0049309 | scaffold_464 | 11530 | 12819 |
| Block 2088 | DDB_G0276041 | 2 | 6505743 | 6507464 | e_gw1.464.3.1 | scaffold_464 | 13700 | 14952 |
| Block 2088 | DDB_G0276039 | 2 | 6507654 | 6508708 | GID1.0049311 | scaffold_464 | 15679 | 16587 |
| Block 2268 | dhkF | 2 | 6510206 | 6513834 | estExt_Genewise1.C_5110015 | scaffold_511 | 13499 | 16778 |
| Block 2669 | DDB_G0276259 | 2 | 6516107 | 6517803 | fgeneshDP_pm.C_scaffold_660000001 | scaffold_660 | 2883 | 4426 |
| Block 2669 | mcfV | 2 | 6518859 | 6520587 | e_gw1.660.5.1 | scaffold_660 | 854 | 2057 |
| Block 2890 | DDB_G0276263 | 2 | 6521921 | 6524951 | estExt_fgeneshDP_pm.C_800001 | scaffold_80 | 1475 | 3667 |
| Block 2088 | pdi1 | 2 | 6534599 | 6535780 | estExt_Genewise1.C_4640004 | scaffold_464 | 4012 | 5454 |
| Block 2088 | DDB_G0276033 | 2 | 6536698 | 6537213 | estExt_fgeneshDP_pg.C_4640001 | scaffold_464 | 1986 | 2681 |
| Block 2268 | DDB_G0276297 | 2 | 6542724 | 6543779 | GID1.0049720 | scaffold_511 | 2284 | 3354 |
| Block 2268 | DDB_G0276293 | 2 | 6545533 | 6547005 | gw1.511.9.1 | scaffold_511 | 4031 | 5156 |
| Block 2268 | DDB_G0276317 | 2 | 6548294 | 6550404 | estExt_fgeneshDP_pg.C_5110003 | scaffold_511 | 6108 | 8150 |
| Block 2268 | DDB_G0276291 | 2 | 6551323 | 6551810 | estExt_fgeneshDP_kg.C_5110002 | scaffold_511 | 8657 | 9181 |
| Block 2268 | DDB_G0276319 | 2 | 6552163 | 6553147 | estExt_fgeneshDP_kg.C_5110004 | scaffold_511 | 9293 | 10517 |
| Block 2268 | DDB_G0276321 | 2 | 6553840 | 6556023 | GID1.0049725 | scaffold_511 | 10880 | 12999 |
| Block 402 | sgcA | 2 | 6569374 | 6579404 | GID1.0043711 | scaffold_146 | 4267 | 14356 |
| Block 402 | DDB_G0276285 | 2 | 6580646 | 6582630 | estExt_fgeneshDP_pg.C_1460004 | scaffold_146 | 15710 | 17657 |
| Block 402 | utp13 | 2 | 6586815 | 6589839 | GID1.0043713 | scaffold_146 | 17853 | 20812 |
| Block 402 | DDB_G0276281 | 2 | 6594735 | 6595766 | estExt_fgeneshDP_kg.C_1460004 | scaffold_146 | 20881 | 22016 |
| Block 89 | DDB_G0276279 | 2 | 6596619 | 6597930 | GID1.0042535 | scaffold_108 | 27595 | 28925 |
| Block 89 | DDB_G0276305 | 2 | 6598521 | 6600671 | estExt_Genewise1.C_1080027 | scaffold_108 | 29431 | 31947 |
| Block 555 | H2Bv2 | 2 | 6617583 | 6619007 | GID1.0044315 | scaffold_167 | 61278 | 62863 |
| Block 555 | DDB_G0276325 | 2 | 6620069 | 6623378 | GID1.0044314 | scaffold_167 | 57250 | 60358 |
| Block 824 | DDB_G0276389 | 2 | 6638389 | 6642252 | GID1.0045229 | scaffold_205 | 28373 | 31350 |
| Block 824 | torA | 2 | 6642359 | 6644785 | e_gw1.205.14.1 | scaffold_205 | 25991 | 28282 |
| Block 967 | pccB | 2 | 6649902 | 6651563 | estExt_fgeneshDP_pm.C_2280008 | scaffold_228 | 24472 | 26208 |
| Block 967 | gpaK | 2 | 6656184 | 6657345 | estExt_fgeneshDP_kg.C_2280004 | scaffold_228 | 27820 | 29239 |
| Block 824 | anapc2 | 2 | 6662690 | 6665640 | GID1.0045235 | scaffold_205 | 41885 | 44788 |
| Block 824 | nat5 | 2 | 6665751 | 6666443 | fgeneshDP_pm.C_scaffold_205000007 | scaffold_205 | 44982 | 45671 |
| Block 568 | DDB_G0276347 | 2 | 6683848 | 6684795 | estExt_Genewise1.C_170040 | scaffold_17 | 57891 | 59178 |
| Block 568 | fnkC | 2 | 6685245 | 6689265 | GID1.0038446 | scaffold_17 | 53853 | 57583 |
| Block 2559 | tipB | 2 | 6704235 | 6705693 | GID1.0050405 | scaffold_617 | 250 | 1503 |
| Block 2767 | pyr4 | 2 | 6712677 | 6713789 | e_gw1.72.44.1 | scaffold_72 | 43074 | 44180 |
| Block 2767 | pyr1-3 | 2 | 6715125 | 6721929 | e_gw1.72.26.1 | scaffold_72 | 35167 | 41832 |
| Block 2036 | DDB_G0276383 | 2 | 6722356 | 6728756 | fgeneshDP_pg.C_scaffold_45000030 | scaffold_45 | 78902 | 85035 |
| Block 2036 | DDB_G0276361 | 2 | 6731626 | 6733995 | estExt_fgeneshDP_pm.C_450020 | scaffold_45 | 85488 | 88431 |
| Block 2036 | myoG | 2 | 6735309 | 6745974 | GID1.0040026 | scaffold_45 | 89289 | 98996 |
| Block 609 | DDB_G0276527 | 2 | 6758586 | 6764413 | GID1.0044521 | scaffold_175 | 43121 | 48409 |
| Block 87 | ercc8 | 2 | 6765058 | 6766711 | GID1.0042540 | scaffold_108 | 43400 | 45075 |
| Block 87 | DDB_G0276529 | 2 | 6767314 | 6773989 | e_gw1.108.13.1 | scaffold_108 | 33786 | 40115 |
| Block 609 | DDB_G0276483 | 2 | 6774250 | 6775212 | fgeneshDP_pg.C_scaffold_175000017 | scaffold_175 | 51472 | 52401 |
| Block 609 | vilB | 2 | 6778874 | 6781843 | GID1.0044524 | scaffold_175 | 52763 | 56605 |
| Block 609 | DDB_G0276531 | 2 | 6782346 | 6785288 | GID1.0044525 | scaffold_175 | 57574 | 60517 |
| Block 609 | gpaG | 2 | 6786653 | 6788270 | estExt_fgeneshDP_kg.C_1750006 | scaffold_175 | 60992 | 63030 |
| Block 87 | DDB_G0276535 | 2 | 6792594 | 6793817 | fgeneshDP_pg.C_scaffold_108000001 | scaffold_108 | 682 | 1614 |
| Block 87 | DDB_G0276537 | 2 | 6794229 | 6794676 | fgeneshDP_pg.C_scaffold_108000002 | scaffold_108 | 2137 | 2576 |
| Block 87 | rps15a | 2 | 6795591 | 6795983 | estExt_Genewise1.C_1080004 | scaffold_108 | 2951 | 4005 |
| Block 87 | DDB_G0276485 | 2 | 6796209 | 6797616 | GID1.0042528 | scaffold_108 | 7911 | 9801 |
| Block 1681 | DDB_G0276541 | 2 | 6806094 | 6811742 | estExt_fgeneshDP_pm.C_3650002 | scaffold_365 | 13136 | 18522 |
| Block 2838 | DDB_G0276407 | 2 | 6819071 | 6820655 | GID1.0041413 | scaffold_77 | 78443 | 79972 |
| Block 2838 | DDB_G0276413 | 2 | 6830256 | 6832764 | fgeneshDP_pg.C_scaffold_77000031 | scaffold_77 | 80344 | 82666 |
| Block 2838 | mrt4 | 2 | 6832973 | 6833734 | e_gw1.77.51.1 | scaffold_77 | 82843 | 83594 |
| Block 2838 | pakB | 2 | 6834331 | 6837105 | e_gw1.77.29.1 | scaffold_77 | 84952 | 86472 |
| Block 2035 | DDB_G0276489 | 2 | 6839916 | 6841328 | estExt_fgeneshDP_pg.C_450029 | scaffold_45 | 77158 | 78628 |
| Block 2035 | mettl1 | 2 | 6841951 | 6842892 | e_gw1.45.16.1 | scaffold_45 | 75983 | 76897 |
| Block 87 | DDB_G0276549 | 2 | 6853808 | 6856851 | estExt_Genewise1.C_1080014 | scaffold_108 | 10052 | 14909 |
| Block 1681 | DDB_G0276553 | 2 | 6861964 | 6864751 | GID1.0048137 | scaffold_365 | 23614 | 26258 |
| Block 3055 | hira | 2 | 6867962 | 6871754 | fgeneshDP_pm.C_scaffold_98000006 | scaffold_98 | 31008 | 34590 |
| Block 3055 | DDB_G0276555 | 2 | 6872219 | 6874893 | fgeneshDP_pm.C_scaffold_98000007 | scaffold_98 | 35021 | 37653 |
| Block 2560 | pdhB | 2 | 6875032 | 6876597 | estExt_Genewise1.C_6170007 | scaffold_617 | 5279 | 7297 |
| Block 2560 | rps18 | 2 | 6877572 | 6878351 | estExt_fgeneshDP_kg.C_6170001 | scaffold_617 | 3796 | 4600 |
| Block 484 | DDB_G0276461 | 2 | 6890762 | 6893260 | estExt_fgeneshDP_pg.C_1550017 | scaffold_155 | 51947 | 54515 |
| Block 484 | DDB_G0276503 | 2 | 6895025 | 6896483 | estExt_Genewise1Plus.C_1550029 | scaffold_155 | 48984 | 51541 |
| Block 565 | DDB_G0276561 | 2 | 6899145 | 6901824 | gw1.17.48.1 | scaffold_17 | 88019 | 89416 |
| Block 3055 | DG1112 | 2 | 6902171 | 6905424 | estExt_Genewise1.C_980019 | scaffold_98 | 21699 | 25122 |
| Block 847 | DDB_G0276431 | 2 | 6917381 | 6918823 | estExt_fgeneshDP_pm.C_210003 | scaffold_21 | 7494 | 8988 |
| Block 847 | sepA | 2 | 6929237 | 6933164 | estExt_fgeneshDP_pg.C_210001 | scaffold_21 | 1533 | 5367 |
| Block 847 | DDB_G0276567 | 2 | 6937506 | 6938876 | estExt_fgeneshDP_pm.C_210002 | scaffold_21 | 5895 | 7459 |
| Block 565 | rpl7 | 2 | 6942715 | 6943942 | fgeneshDP_pg.C_scaffold_17000004 | scaffold_17 | 7787 | 8984 |
| Block 565 | DDB_G0276569 | 2 | 6944790 | 6946059 | GID1.0038426 | scaffold_17 | 4715 | 5916 |
| Block 565 | usp7 | 2 | 6946259 | 6950896 | estExt_fgeneshDP_pg.C_170001 | scaffold_17 | 516 | 4503 |
| Block 1810 | DDB_G0276451 | 2 | 6961052 | 6961850 | fgeneshDP_pm.C_scaffold_396000007 | scaffold_396 | 25109 | 25786 |
| Block 1810 | DDB_G0276573 | 2 | 6962416 | 6963678 | GID1.0048564 | scaffold_396 | 23511 | 24560 |
| Block 565 | DDB_G0276449 | 2 | 6963973 | 6965249 | GID1.0038429 | scaffold_17 | 9184 | 10454 |
| Block 565 | DDB_G0276447 | 2 | 6966228 | 6968533 | fgeneshDP_pm.C_scaffold_17000004 | scaffold_17 | 11218 | 13456 |
| Block 565 | DDB_G0276445 | 2 | 6970767 | 6972827 | estExt_Genewise1Plus.C_170013 | scaffold_17 | 17339 | 19676 |
| Block 2404 | syn16A | 2 | 6979737 | 6980894 | GID1.0040491 | scaffold_56 | 26728 | 27948 |
| Block 229 | DDB_G0276519 | 2 | 6995818 | 6998580 | fgeneshDP_pg.C_scaffold_123000026 | scaffold_123 | 69287 | 71395 |
| Block 229 | DDB_G0276521 | 2 | 6998756 | 6999149 | GID1.0043053 | scaffold_123 | 68593 | 68954 |
| Block 3058 | DDB_G0276471 | 2 | 7000828 | 7001278 | gw1.98.42.1 | scaffold_98 | 42699 | 43389 |
| Block 3058 | DDB_G0276473 | 2 | 7001811 | 7003048 | estExt_fgeneshDP_pg.C_980016 | scaffold_98 | 43657 | 44958 |
| Block 2404 | DDB_G0276583 | 2 | 7003791 | 7006681 | estExt_fgeneshDP_pm.C_560004 | scaffold_56 | 24065 | 26624 |
| Block 2404 | DDB_G0276523 | 2 | 7007156 | 7009176 | estExt_Genewise1Plus.C_560010 | scaffold_56 | 21709 | 23622 |
| Block 2404 | myoC | 2 | 7033317 | 7036976 | GID1.0040502 | scaffold_56 | 47502 | 51241 |
| Block 2404 | DDB_G0276623 | 2 | 7037821 | 7038470 | estExt_fgeneshDP_kg.C_560006 | scaffold_56 | 51982 | 52749 |
| Block 2404 | hmgB | 2 | 7040072 | 7041652 | GID1.0040504 | scaffold_56 | 54027 | 55580 |
| Block 2404 | DDB_G0276625 | 2 | 7041890 | 7043333 | GID1.0040505 | scaffold_56 | 55789 | 56966 |
| Block 2404 | DDB_G0276627 | 2 | 7044331 | 7045548 | fgeneshDP_pg.C_scaffold_56000023 | scaffold_56 | 100516 | 101730 |
| Block 2404 | DDB_G0276631 | 2 | 7046544 | 7047592 | e_gw1.56.39.1 | scaffold_56 | 101968 | 102961 |
| Block 778 | DDB_G0276673 | 2 | 7088783 | 7092476 | GID1.0038629 | scaffold_20 | 68748 | 72325 |
| Block 778 | DDB_G0276675 | 2 | 7092722 | 7093857 | e_gw1.20.74.1 | scaffold_20 | 73007 | 74057 |
| Block 567 | DDB_G0276713 | 2 | 7098964 | 7102563 | GID1.0038444 | scaffold_17 | 47595 | 50645 |
| Block 567 | DDB_G0276679 | 2 | 7102976 | 7103468 | GID1.0038443 | scaffold_17 | 46807 | 47245 |
| Block 2443 | tyrS | 2 | 7123835 | 7125181 | GID1.0050150 | scaffold_574 | 6985 | 8420 |
| Block 2443 | DDB_G0276695 | 2 | 7127974 | 7129914 | GID1.0050147 | scaffold_574 | 1032 | 2889 |
| Block 567 | pldG | 2 | 7165567 | 7168640 | estExt_fgeneshDP_pg.C_170024 | scaffold_17 | 60616 | 64299 |
| Block 778 | canA | 2 | 7175839 | 7178164 | GID1.0038628 | scaffold_20 | 65822 | 67954 |
| Block 778 | DDB_G0276927 | 2 | 7178354 | 7179092 | estExt_fgeneshDP_pg.C_200021 | scaffold_20 | 64600 | 65618 |
| Block 778 | cotB | 2 | 7180807 | 7182719 | estExt_fgeneshDP_pg.C_200020 | scaffold_20 | 61592 | 63640 |
| Block 778 | ubqC | 2 | 7185203 | 7186037 | estExt_Genewise1Plus.C_200038 | scaffold_20 | 74172 | 74908 |
| Block 778 | DDB_G0277007 | 2 | 7186890 | 7188420 | e_gw1.20.62.1 | scaffold_20 | 75711 | 77063 |
| Block 778 | DDB_G0277009 | 2 | 7188977 | 7189934 | GID1.0038634 | scaffold_20 | 79902 | 80934 |
| Block 778 | DDB_G0277011 | 2 | 7190188 | 7191382 | GID1.0038635 | scaffold_20 | 81170 | 82289 |
| Block 778 | DDB_G0277013 | 2 | 7191621 | 7192991 | e_gw1.20.56.1 | scaffold_20 | 82631 | 83824 |
| Block 1038 | captC | 2 | 7196595 | 7198174 | estExt_Genewise1.C_2390010 | scaffold_239 | 9626 | 11064 |
| Block 1038 | DDB_G0276961 | 2 | 7198816 | 7200418 | GID1.0045937 | scaffold_239 | 11385 | 12975 |
| Block 700 | gltA | 2 | 7203181 | 7205017 | estExt_fgeneshDP_pg.C_1900008 | scaffold_190 | 23114 | 25071 |
| Block 700 | mcfX | 2 | 7205928 | 7206833 | fgeneshDP_pg.C_scaffold_190000007 | scaffold_190 | 19048 | 20198 |
| Block 700 | DDB_G0276935 | 2 | 7206912 | 7209124 | estExt_fgeneshDP_kg.C_1900002 | scaffold_190 | 18186 | 18984 |
| Block 567 | DDB_G0277015 | 2 | 7211571 | 7212398 | estExt_Genewise1Plus.C_170028 | scaffold_17 | 38820 | 39995 |
| Block 567 | psmC2 | 2 | 7220471 | 7222019 | estExt_fgeneshDP_pg.C_170017 | scaffold_17 | 40601 | 42491 |
| Block 567 | wdr36 | 2 | 7225072 | 7228344 | e_gw1.17.3.1 | scaffold_17 | 43088 | 46212 |
| Block 2091 | plbA | 2 | 7228630 | 7230567 | gw1.465.6.1 | scaffold_465 | 5233 | 7058 |
| Block 2091 | gefH | 2 | 7232285 | 7234301 | GID1.0049315 | scaffold_465 | 1108 | 3010 |
| Block 3047 | DDB_G0276903 | 2 | 7246401 | 7246589 | e_gw1.97.48.1 | scaffold_97 | 44908 | 45069 |
| Block 3047 | DDB_G0277003 | 2 | 7246770 | 7247710 | e_gw1.97.26.1 | scaffold_97 | 43937 | 44769 |
| Block 3047 | cpiC | 2 | 7247886 | 7248167 | estExt_fgeneshDP_kg.C_970003 | scaffold_97 | 43425 | 43913 |
| Block 3047 | DDB_G0277025 | 2 | 7248965 | 7249927 | gw1.97.28.1 | scaffold_97 | 40327 | 41062 |
| Block 3047 | DDB_G0276901 | 2 | 7250086 | 7250349 | estExt_Genewise1.C_970017 | scaffold_97 | 39492 | 39976 |
| Block 2786 | DDB_G0276899 | 2 | 7250605 | 7251583 | estExt_Genewise1.C_7330004 | scaffold_733 | 1579 | 2714 |
| Block 2786 | tmem56B | 2 | 7259023 | 7259983 | gw1.733.5.1 | scaffold_733 | 539 | 1375 |
| Block 825 | DDB_G0277031 | 2 | 7268082 | 7269923 | fgeneshDP_pg.C_scaffold_205000019 | scaffold_205 | 50236 | 51812 |
| Block 825 | DDB_G0276769 | 2 | 7270608 | 7271380 | GID1.0045240 | scaffold_205 | 54303 | 55072 |
| Block 965 | DDB_G0276779 | 2 | 7274549 | 7276368 | estExt_Genewise1Plus.C_2280001 | scaffold_228 | 1 | 1537 |
| Block 965 | DDB_G0276781 | 2 | 7276697 | 7277607 | GID1.0045730 | scaffold_228 | 1824 | 2680 |
| Block 2710 | DDB_G0276783 | 2 | 7277963 | 7278892 | GID1.0050710 | scaffold_687 | 7117 | 7966 |
| Block 2710 | DDB_G0276997 | 2 | 7279793 | 7281449 | e_gw1.687.2.1 | scaffold_687 | 4781 | 6315 |
| Block 2401 | DDB_G0277033 | 2 | 7281705 | 7285614 | GID1.0040483 | scaffold_56 | 3477 | 8427 |
| Block 2401 | DDB_G0276993 | 2 | 7289061 | 7289930 | GID1.0040484 | scaffold_56 | 8995 | 9942 |
| Block 779 | ctxB | 2 | 7291201 | 7292643 | estExt_Genewise1Plus.C_200052 | scaffold_20 | 84015 | 85617 |
| Block 779 | DDB_G0276785 | 2 | 7293352 | 7294681 | GID1.0038645 | scaffold_20 | 99351 | 100785 |
| Block 779 | pgs1 | 2 | 7295554 | 7297396 | estExt_fgeneshDP_pg.C_200039 | scaffold_20 | 102637 | 106321 |
| Block 779 | DDB_G0276895 | 2 | 7301575 | 7302360 | GID1.0038649 | scaffold_20 | 106425 | 107075 |
| Block 572 | rheb | 2 | 7307901 | 7308556 | GID1.0038464 | scaffold_17 | 115966 | 116624 |
| Block 572 | DDB_G0277043 | 2 | 7309037 | 7311422 | e_gw1.17.40.1 | scaffold_17 | 117737 | 119710 |
| Block 572 | DDB_G0276791 | 2 | 7312632 | 7315457 | estExt_fgeneshDP_pm.C_170018 | scaffold_17 | 120262 | 123082 |
| Block 572 | DDB_G0276789 | 2 | 7315749 | 7316266 | GID1.0038467 | scaffold_17 | 123208 | 123673 |
| Block 2401 | lysA | 2 | 7324249 | 7328679 | estExt_fgeneshDP_pm.C_560002 | scaffold_56 | 12995 | 17077 |
| Block 214 | DDB_G0277045 | 2 | 7329544 | 7331445 | e_gw1.122.32.1 | scaffold_122 | 26008 | 27618 |
| Block 214 | tfdp2 | 2 | 7332551 | 7334636 | estExt_fgeneshDP_pg.C_1220013 | scaffold_122 | 28638 | 30981 |
| Block 214 | DDB_G0277047 | 2 | 7334981 | 7338732 | estExt_Genewise1.C_1220028 | scaffold_122 | 31123 | 34635 |
| Block 214 | DDB_G0276801 | 2 | 7338995 | 7341586 | estExt_fgeneshDP_pg.C_1220011 | scaffold_122 | 23562 | 25927 |
| Block 214 | DDB_G0277049 | 2 | 7342293 | 7343669 | GID1.0042991 | scaffold_122 | 21733 | 23153 |
| Block 104 | DDB_G0277051 | 2 | 7344554 | 7346452 | estExt_fgeneshDP_kg.C_110005 | scaffold_11 | 63516 | 65787 |
| Block 104 | prp19 | 2 | 7346815 | 7348845 | estExt_fgeneshDP_pg.C_110009 | scaffold_11 | 29271 | 31566 |
| Block 104 | DDB_G0277053 | 2 | 7350240 | 7352597 | GID1.0038047 | scaffold_11 | 26316 | 28353 |
| Block 104 | DDB_G0276805 | 2 | 7352935 | 7356463 | GID1.0038046 | scaffold_11 | 22955 | 25914 |
| Block 104 | casK | 2 | 7357318 | 7359110 | e_gw1.11.65.1 | scaffold_11 | 21093 | 22519 |
| Block 426 | DDB_G0276991 | 2 | 7363091 | 7364950 | fgeneshDP_pg.C_scaffold_149000022 | scaffold_149 | 67294 | 68547 |
| Block 74 | DDB_G0276815 | 2 | 7377153 | 7378563 | GID1.0042475 | scaffold_106 | 43051 | 44486 |
| Block 74 | DDB_G0276821 | 2 | 7383680 | 7385846 | GID1.0042474 | scaffold_106 | 40531 | 42309 |
| Block 426 | ifkB | 2 | 7399005 | 7403081 | estExt_fgeneshDP_pg.C_1490019 | scaffold_149 | 56732 | 63464 |
| Block 1378 | pspB | 2 | 7404442 | 7406131 | estExt_fgeneshDP_kg.C_3000001 | scaffold_300 | 1545 | 3863 |
| Block 2206 | gacS | 2 | 7409927 | 7413087 | GID1.0049619 | scaffold_496 | 13746 | 16843 |
| Block 2206 | DDB_G0277065 | 2 | 7413757 | 7416189 | fgeneshDP_pg.C_scaffold_496000002 | scaffold_496 | 4555 | 6850 |
| Block 2770 | cotA | 2 | 7435715 | 7437741 | estExt_fgeneshDP_pg.C_720019 | scaffold_72 | 52453 | 54367 |
| Block 1378 | glnA1 | 2 | 7438375 | 7439874 | estExt_Genewise1.C_3000019 | scaffold_300 | 38893 | 40438 |
| Block 1378 | DDB_G0276837 | 2 | 7440494 | 7440712 | estExt_Genewise1Plus.C_3000018 | scaffold_300 | 38481 | 38740 |
| Block 1378 | DDB_G0277073 | 2 | 7441371 | 7442396 | fgeneshDP_pm.C_scaffold_300000008 | scaffold_300 | 37077 | 37943 |
| Block 1899 | DDB_G0276843 | 2 | 7455044 | 7456516 | fgeneshDP_pg.C_scaffold_414000002 | scaffold_414 | 5245 | 6611 |
| Block 1899 | DDB_G0276845 | 2 | 7457047 | 7457682 | fgeneshDP_pg.C_scaffold_414000003 | scaffold_414 | 7204 | 7791 |
| Block 1899 | DDB_G0277079 | 2 | 7457925 | 7458374 | gw1.414.14.1 | scaffold_414 | 8741 | 8962 |
| Block 2770 | DDB_G0277081 | 2 | 7458529 | 7459877 | fgeneshDP_pm.C_scaffold_72000017 | scaffold_72 | 75268 | 76467 |
| Block 52 | DDB_G0276847 | 2 | 7466041 | 7468086 | e_gw1.103.22.1 | scaffold_103 | 7497 | 9239 |
| Block 52 | purH | 2 | 7471628 | 7473256 | estExt_Genewise1.C_1030003 | scaffold_103 | 2900 | 4666 |
| Block 52 | commd4 | 2 | 7473827 | 7474882 | fgeneshDP_pg.C_scaffold_103000003 | scaffold_103 | 5354 | 6390 |
| Block 52 | DDB_G0276853 | 2 | 7475339 | 7476121 | e_gw1.103.47.1 | scaffold_103 | 12498 | 13217 |
| Block 52 | DDB_G0276855 | 2 | 7476418 | 7478610 | estExt_Genewise1.C_1030015 | scaffold_103 | 13346 | 15406 |
| Block 52 | DDB_G0276857 | 2 | 7478852 | 7480340 | GID1.0042354 | scaffold_103 | 15555 | 16837 |
| Block 52 | DDB_G0277093 | 2 | 7481904 | 7482850 | gw1.103.54.1 | scaffold_103 | 17372 | 17811 |
| Block 52 | DDB_G0276861 | 2 | 7489899 | 7491256 | fgeneshDP_pm.C_scaffold_103000003 | scaffold_103 | 22593 | 23959 |
| Block 52 | DDB_G0276977 | 2 | 7491728 | 7494315 | estExt_fgeneshDP_pm.C_1030005 | scaffold_103 | 28617 | 31232 |
| Block 781 | rab11C | 2 | 7495742 | 7496669 | estExt_fgeneshDP_pg.C_200041 | scaffold_20 | 107393 | 108302 |
| Block 781 | DDB_G0277103 | 2 | 7497630 | 7501071 | fgeneshDP_pg.C_scaffold_20000043 | scaffold_20 | 108902 | 112085 |
| Block 781 | crtp1 | 2 | 7503238 | 7505062 | estExt_Genewise1Plus.C_200073 | scaffold_20 | 115212 | 117529 |
| Block 576 | clcB | 2 | 7511780 | 7514334 | e_gw1.17.39.1 | scaffold_17 | 147032 | 149594 |
| Block 576 | DDB_G0276867 | 2 | 7518557 | 7519229 | fgeneshDP_pm.C_scaffold_17000024 | scaffold_17 | 145382 | 146019 |
| Block 2772 | apm4 | 2 | 7519340 | 7521217 | estExt_Genewise1Plus.C_720061 | scaffold_72 | 88649 | 90445 |
| Block 2772 | lsm3 | 2 | 7521506 | 7522057 | e_gw1.72.67.1 | scaffold_72 | 87955 | 88485 |
| Block 2772 | psvA | 2 | 7523443 | 7525185 | GID1.0041208 | scaffold_72 | 84623 | 86462 |
| Block 1342 | rpl10a | 2 | 7526827 | 7527734 | estExt_fgeneshDP_kg.C_2960002 | scaffold_296 | 19512 | 20611 |
| Block 1342 | trap1 | 2 | 7528411 | 7530546 | GID1.0047043 | scaffold_296 | 20906 | 22982 |
| Block 1342 | DDB_G0277111 | 2 | 7541564 | 7543845 | GID1.0047045 | scaffold_296 | 25706 | 27366 |
| Block 2772 | pex16 | 2 | 7544142 | 7545419 | fgeneshDP_pg.C_scaffold_72000033 | scaffold_72 | 90556 | 92077 |
| Block 2208 | nramp1 | 2 | 7550489 | 7552201 | estExt_Genewise1Plus.C_4980003 | scaffold_498 | 1025 | 2944 |
| Block 2527 | DDB_G0277115 | 2 | 7554216 | 7555364 | estExt_Genewise1Plus.C_6040005 | scaffold_604 | 3691 | 4933 |
| Block 2527 | DDB_G0276881 | 2 | 7556328 | 7556849 | estExt_fgeneshDP_kg.C_6040001 | scaffold_604 | 349 | 1474 |
| Block 2208 | DDB_G0277137 | 2 | 7586629 | 7588320 | estExt_Genewise1.C_4980004 | scaffold_498 | 3976 | 5495 |
| Block 2527 | trappc3 | 2 | 7590485 | 7591221 | fgeneshDP_pg.C_scaffold_604000004 | scaffold_604 | 5012 | 5668 |
| Block 2527 | gxcBB | 2 | 7592400 | 7595873 | estExt_Genewise1.C_6040009 | scaffold_604 | 6648 | 10034 |
| Block 2527 | DDB_G0277129 | 2 | 7596113 | 7596769 | e_gw1.604.7.1 | scaffold_604 | 10168 | 10569 |
| Block 823 | DDB_G0277307 | 2 | 7620745 | 7623341 | GID1.0045237 | scaffold_205 | 46764 | 49897 |
| Block 823 | DDB_G0277213 | 2 | 7625386 | 7626401 | e_gw1.205.32.1 | scaffold_205 | 21529 | 22230 |
| Block 823 | cnrJ | 2 | 7627568 | 7629643 | estExt_Genewise1Plus.C_2050014 | scaffold_205 | 18186 | 20270 |
| Block 823 | pkaD | 2 | 7629752 | 7632050 | gw1.205.4.1 | scaffold_205 | 16727 | 17557 |
| Block 823 | DDB_G0277311 | 2 | 7635557 | 7638122 | e_gw1.205.16.1 | scaffold_205 | 9165 | 11768 |
| Block 1300 | iptA | 2 | 7639119 | 7639970 | estExt_fgeneshDP_kg.C_2880001 | scaffold_288 | 13301 | 14296 |
| Block 1300 | DDB_G0277217 | 2 | 7641479 | 7644970 | estExt_fgeneshDP_pm.C_2880003 | scaffold_288 | 14741 | 17593 |
| Block 1300 | DDB_G0277313 | 2 | 7645497 | 7648292 | estExt_fgeneshDP_pg.C_2880007 | scaffold_288 | 17773 | 20184 |
| Block 1300 | DDB_G0277219 | 2 | 7648658 | 7650734 | GID1.0046910 | scaffold_288 | 20404 | 22013 |
| Block 1300 | DDB_G0277315 | 2 | 7651426 | 7652354 | fgeneshDP_pg.C_scaffold_288000009 | scaffold_288 | 22965 | 23957 |
| Block 602 | forG | 2 | 7669104 | 7672455 | GID1.0044486 | scaffold_174 | 24207 | 29837 |
| Block 602 | DDB_G0277179 | 2 | 7675009 | 7676592 | GID1.0044485 | scaffold_174 | 22133 | 23791 |
| Block 602 | atg3 | 2 | 7676988 | 7678399 | estExt_Genewise1.C_1740002 | scaffold_174 | 20468 | 22047 |
| Block 1300 | DDB_G0277185 | 2 | 7685491 | 7692517 | estExt_Genewise1.C_2880010 | scaffold_288 | 28769 | 35674 |
| Block 1300 | DDB_G0277187 | 2 | 7693792 | 7694910 | GID1.0046916 | scaffold_288 | 36663 | 38159 |
| Block 773 | DDB_G0277189 | 2 | 7702153 | 7702749 | e_gw1.20.101.1 | scaffold_20 | 25007 | 25649 |
| Block 773 | chcA | 2 | 7703629 | 7709061 | estExt_Genewise1.C_200017 | scaffold_20 | 26074 | 31955 |
| Block 773 | DDB_G0277327 | 2 | 7710224 | 7713698 | e_gw1.20.29.1 | scaffold_20 | 20417 | 24510 |
| Block 773 | DDB_G0277329 | 2 | 7714001 | 7716524 | fgeneshDP_pg.C_scaffold_20000009 | scaffold_20 | 17354 | 19911 |
| Block 773 | mrps17 | 2 | 7718257 | 7718640 | e_gw1.20.98.1 | scaffold_20 | 14898 | 15281 |
| Block 773 | DDB_G0277197 | 2 | 7718960 | 7719637 | GID1.0038609 | scaffold_20 | 13389 | 14279 |
| Block 783 | dgkA | 2 | 7728034 | 7730784 | GID1.0038657 | scaffold_20 | 121426 | 124053 |
| Block 783 | DDB_G0277205 | 2 | 7732992 | 7733709 | estExt_fgeneshDP_pm.C_200021 | scaffold_20 | 126048 | 126948 |
| Block 966 | DDB_G0277229 | 2 | 7738959 | 7741103 | fgeneshDP_pg.C_scaffold_228000012 | scaffold_228 | 22309 | 24384 |
| Block 966 | ndufs8 | 2 | 7741263 | 7742099 | fgeneshDP_pm.C_scaffold_228000006 | scaffold_228 | 20942 | 22032 |
| Block 966 | mcysS | 2 | 7742358 | 7744329 | GID1.0045738 | scaffold_228 | 18752 | 20649 |
| Block 966 | DDB_G0277235 | 2 | 7746019 | 7747815 | fgeneshDP_pg.C_scaffold_228000009 | scaffold_228 | 15952 | 17475 |
| Block 783 | DDB_G0277237 | 2 | 7748080 | 7749272 | fgeneshDP_pg.C_scaffold_20000052 | scaffold_20 | 126995 | 128135 |
| Block 783 | DDB_G0277331 | 2 | 7749467 | 7751341 | GID1.0038662 | scaffold_20 | 128271 | 129928 |
| Block 783 | DDB_G0277239 | 2 | 7751400 | 7757267 | GID1.0038663 | scaffold_20 | 130141 | 135846 |
| Block 783 | DDB_G0277333 | 2 | 7758300 | 7759343 | e_gw1.20.92.1 | scaffold_20 | 148382 | 149067 |
| Block 966 | DDB_G0277335 | 2 | 7759740 | 7760451 | e_gw1.228.42.1 | scaffold_228 | 3292 | 4014 |
| Block 966 | DDB_G0277149 | 2 | 7763808 | 7764973 | e_gw1.228.34.1 | scaffold_228 | 7621 | 8853 |
| Block 966 | DDB_G0277341 | 2 | 7765694 | 7768987 | fgeneshDP_pg.C_scaffold_228000006 | scaffold_228 | 9854 | 12892 |
| Block 966 | DDB_G0277243 | 2 | 7769456 | 7770896 | e_gw1.228.30.1 | scaffold_228 | 14147 | 15572 |
| Block 699 | scfd2 | 2 | 7771367 | 7774152 | estExt_fgeneshDP_pg.C_1900004 | scaffold_190 | 11717 | 14459 |
| Block 699 | apm2 | 2 | 7774523 | 7776187 | GID1.0044856 | scaffold_190 | 14687 | 16332 |
| Block 699 | rps3a | 2 | 7782031 | 7783618 | estExt_fgeneshDP_pg.C_1900003 | scaffold_190 | 9629 | 11335 |
| Block 2395 | DDB_G0277245 | 2 | 7785663 | 7789396 | estExt_Genewise1.C_5560012 | scaffold_556 | 9837 | 13548 |
| Block 2395 | DDB_G0277349 | 2 | 7789663 | 7790505 | fgeneshDP_pg.C_scaffold_556000004 | scaffold_556 | 8346 | 9576 |
| Block 2395 | phg1b | 2 | 7792635 | 7794903 | estExt_Genewise1Plus.C_5560008 | scaffold_556 | 5405 | 7758 |
| Block 699 | DDB_G0277249 | 2 | 7804358 | 7805206 | gw1.190.6.1 | scaffold_190 | 37826 | 38230 |
| Block 2030 | DDB_G0277251 | 2 | 7807780 | 7809789 | fgeneshDP_pg.C_scaffold_45000004 | scaffold_45 | 6773 | 8694 |
| Block 2030 | DDB_G0277253 | 2 | 7810140 | 7812752 | estExt_Genewise1.C_450001 | scaffold_45 | 2496 | 6744 |
| Block 820 | DDB_G0277257 | 2 | 7819656 | 7821277 | e_gw1.205.8.1 | scaffold_205 | 49 | 596 |
| Block 820 | DDB_G0277259 | 2 | 7821725 | 7822135 | GID1.0045218 | scaffold_205 | 1012 | 1404 |
| Block 820 | DDB_G0277263 | 2 | 7829165 | 7830232 | gw1.205.31.1 | scaffold_205 | 2207 | 2998 |
| Block 907 | DDB_G0277265 | 2 | 7835033 | 7836502 | fgeneshDP_pg.C_scaffold_218000002 | scaffold_218 | 4619 | 6411 |
| Block 907 | poxA | 2 | 7843530 | 7845358 | estExt_fgeneshDP_pm.C_2180001 | scaffold_218 | 984 | 3070 |
| Block 2174 | DDB_G0277361 | 2 | 7847201 | 7849048 | GID1.0049519 | scaffold_486 | 830 | 1351 |
| Block 2174 | ionA | 2 | 7850854 | 7854647 | e_gw1.486.3.1 | scaffold_486 | 4854 | 7875 |
| Block 2174 | DDB_G0277287 | 2 | 7854865 | 7857366 | estExt_Genewise1.C_4860005 | scaffold_486 | 8725 | 11221 |
| Block 2915 | DDB_G0277367 | 2 | 7875821 | 7876306 | fgeneshDP_pm.C_scaffold_82000014 | scaffold_82 | 68649 | 69125 |
| Block 2915 | DDB_G0277151 | 2 | 7876387 | 7877957 | e_gw1.82.21.1 | scaffold_82 | 66849 | 68472 |
| Block 2915 | DDB_G0277369 | 2 | 7878450 | 7879358 | GID1.0041606 | scaffold_82 | 65626 | 66406 |
| Block 1867 | DDB_G0277375 | 2 | 7892061 | 7895493 | estExt_Genewise1Plus.C_4080007 | scaffold_408 | 10057 | 13057 |
| Block 1867 | DDB_G0277163 | 2 | 7896231 | 7897544 | e_gw1.408.3.1 | scaffold_408 | 8053 | 9243 |
| Block 1867 | DDB_G0277279 | 2 | 7897554 | 7898278 | fgeneshDP_pm.C_scaffold_408000003 | scaffold_408 | 7320 | 7952 |
| Block 1867 | DDB_G0277159 | 2 | 7900120 | 7901136 | e_gw1.408.4.1 | scaffold_408 | 2697 | 3377 |
| Block 2764 | DDB_G0277271 | 2 | 7901916 | 7903060 | estExt_Genewise1Plus.C_720008 | scaffold_72 | 7159 | 8405 |
| Block 2764 | nubp2 | 2 | 7903850 | 7904837 | GID1.0041179 | scaffold_72 | 5750 | 6721 |
| Block 2764 | tom40 | 2 | 7905198 | 7906836 | GID1.0041178 | scaffold_72 | 3929 | 5337 |
| Block 1867 | DDB_G0277391 | 2 | 7914101 | 7916497 | estExt_fgeneshDP_pg.C_4080001 | scaffold_408 | 159 | 2553 |
| Block 2915 | DDB_G0277389 | 2 | 7917122 | 7918696 | fgeneshDP_pm.C_scaffold_82000015 | scaffold_82 | 69593 | 71150 |
| Block 1867 | pspD | 2 | 7919127 | 7921344 | estExt_fgeneshDP_pg.C_4080007 | scaffold_408 | 13807 | 15631 |
| Block 2764 | DDB_G0277383 | 2 | 7927885 | 7929534 | estExt_fgeneshDP_pm.C_720015 | scaffold_72 | 64510 | 66759 |
| Block 2764 | DDB_G0277395 | 2 | 7932192 | 7932749 | estExt_fgeneshDP_pg.C_720022 | scaffold_72 | 61486 | 62333 |
| Block 2764 | DDB_G0277481 | 2 | 7940307 | 7941567 | estExt_fgeneshDP_pg.C_720001 | scaffold_72 | 803 | 3733 |
| Block 1382 | DDB_G0277411 | 2 | 7948969 | 7949649 | e_gw1.300.18.1 | scaffold_300 | 21746 | 22502 |
| Block 1382 | DDB_G0277407 | 2 | 7952613 | 7952990 | GID1.0047119 | scaffold_300 | 23481 | 23921 |
| Block 1382 | DDB_G0277405 | 2 | 7953381 | 7954590 | GID1.0047120 | scaffold_300 | 24216 | 25339 |
| Block 1382 | DDB_G0277483 | 2 | 7960430 | 7963088 | estExt_Genewise1Plus.C_3000015 | scaffold_300 | 26642 | 28901 |
| Block 75 | DDB_G0277533 | 2 | 7963300 | 7966911 | estExt_Genewise1Plus.C_1060037 | scaffold_106 | 44636 | 47127 |
| Block 75 | dyrk1 | 2 | 7967587 | 7970471 | estExt_fgeneshDP_pg.C_1060014 | scaffold_106 | 48802 | 51417 |
| Block 1900 | DDB_G0277509 | 2 | 7972253 | 7972635 | GID1.0048792 | scaffold_414 | 17076 | 17769 |
| Block 1900 | DDB_G0277537 | 2 | 7975590 | 7983208 | GID1.0048791 | scaffold_414 | 9599 | 16625 |
| Block 1340 | DG1122 | 2 | 7985091 | 7987449 | fgeneshDP_pg.C_scaffold_296000001 | scaffold_296 | 1383 | 3461 |
| Block 1340 | cct6 | 2 | 7987588 | 7989436 | fgeneshDP_pg.C_scaffold_296000012 | scaffold_296 | 29063 | 30880 |
| Block 1340 | DDB_G0277495 | 2 | 7991481 | 7993460 | GID1.0047037 | scaffold_296 | 7681 | 9467 |
| Block 1340 | vatB | 2 | 7994638 | 7996460 | estExt_Genewise1.C_2960006 | scaffold_296 | 10596 | 12739 |
| Block 914 | DDB_G0277539 | 2 | 7997048 | 8000044 | GID1.0038740 | scaffold_22 | 4448 | 7266 |
| Block 914 | DDB_G0277541 | 2 | 8002164 | 8004711 | estExt_fgeneshDP_pg.C_220004 | scaffold_22 | 8731 | 11310 |
| Block 914 | DDB_G0277543 | 2 | 8007471 | 8012532 | fgeneshDP_pg.C_scaffold_22000005 | scaffold_22 | 11609 | 15732 |
| Block 914 | piaA | 2 | 8014117 | 8017818 | estExt_fgeneshDP_pg.C_220006 | scaffold_22 | 17308 | 21064 |
| Block 914 | hpd | 2 | 8018648 | 8020421 | estExt_fgeneshDP_kg.C_220002 | scaffold_22 | 23765 | 25509 |
| Block 2771 | DDB_G0277429 | 2 | 8026075 | 8028735 | estExt_fgeneshDP_pg.C_720029 | scaffold_72 | 79669 | 83176 |
| Block 2771 | DDB_G0277427 | 2 | 8030093 | 8032114 | GID1.0041205 | scaffold_72 | 76847 | 79038 |
| Block 914 | cycD | 2 | 8052989 | 8055164 | estExt_fgeneshDP_pg.C_220001 | scaffold_22 | 341 | 2035 |
| Block 914 | nubp1 | 2 | 8055359 | 8056502 | e_gw1.22.23.1 | scaffold_22 | 2291 | 3358 |
| Block 914 | dpm3 | 2 | 8056847 | 8057309 | fgeneshDP_pm.C_scaffold_22000002 | scaffold_22 | 3683 | 4143 |
| Block 1341 | capB | 2 | 8083002 | 8083944 | estExt_Genewise1Plus.C_2960016 | scaffold_296 | 31393 | 32324 |
| Block 1341 | rabJ | 2 | 8097388 | 8098468 | GID1.0047048 | scaffold_296 | 33846 | 34716 |
| Block 1341 | amtA | 2 | 8098858 | 8100429 | estExt_Genewise1.C_2960024 | scaffold_296 | 35011 | 36868 |
| Block 1341 | DDB_G0277449 | 2 | 8107750 | 8109220 | e_gw1.296.16.1 | scaffold_296 | 38954 | 40426 |
| Block 1341 | clc | 2 | 8109503 | 8110193 | GID1.0047040 | scaffold_296 | 15528 | 16327 |
| Block 2771 | DDB_G0277451 | 2 | 8110693 | 8111826 | fgeneshDP_pg.C_scaffold_72000030 | scaffold_72 | 83233 | 84327 |
| Block 2270 | ddx20 | 2 | 8114748 | 8117297 | e_gw1.512.22.1 | scaffold_512 | 11944 | 13206 |
| Block 2270 | DDB_G0277453 | 2 | 8117472 | 8117861 | GID1.0049733 | scaffold_512 | 13463 | 13812 |
| Block 2270 | dynC | 2 | 8118175 | 8119000 | e_gw1.512.16.1 | scaffold_512 | 14099 | 14906 |
| Block 1045 | plbE | 2 | 8119377 | 8121155 | GID1.0038876 | scaffold_24 | 59812 | 61739 |
| Block 1045 | DDB_G0277563 | 2 | 8121554 | 8122184 | e_gw1.24.59.1 | scaffold_24 | 62163 | 62744 |
| Block 1942 | DDB_G0277469 | 2 | 8129207 | 8129848 | fgeneshDP_pg.C_scaffold_425000007 | scaffold_425 | 10460 | 10978 |
| Block 1942 | DDB_G0277471 | 2 | 8129998 | 8131503 | estExt_Genewise1.C_4250009 | scaffold_425 | 8507 | 10026 |
| Block 2270 | DDB_G0277475 | 2 | 8134167 | 8135634 | gw1.512.6.1 | scaffold_512 | 9413 | 10246 |
| Block 2270 | DDB_G0277477 | 2 | 8135731 | 8138016 | estExt_Genewise1Plus.C_5120009 | scaffold_512 | 6653 | 8757 |
| Block 2270 | DDB_G0277479 | 2 | 8138895 | 8140490 | gw1.512.7.1 | scaffold_512 | 4696 | 5367 |
| Block 2270 | syn5 | 2 | 8140625 | 8142080 | e_gw1.512.11.1 | scaffold_512 | 3239 | 4590 |
| Block 772 | DDB_G0277571 | 2 | 8144500 | 8146932 | GID1.0038606 | scaffold_20 | 4827 | 7247 |
| Block 772 | DDB_G0277569 | 2 | 8147602 | 8149038 | estExt_Genewise1Plus.C_200004 | scaffold_20 | 3282 | 4551 |
| Block 772 | DDB_G0277567 | 2 | 8149650 | 8151410 | estExt_fgeneshDP_pg.C_200001 | scaffold_20 | 94 | 3286 |
| Block 1045 | DDB_G0277615 | 2 | 8170923 | 8172580 | estExt_fgeneshDP_kg.C_240005 | scaffold_24 | 57931 | 59017 |
| Block 1942 | DDB_G0277663 | 2 | 8252291 | 8255625 | estExt_fgeneshDP_pg.C_4250014 | scaffold_425 | 19285 | 22574 |
| Block 1942 | DDB_G0277665 | 2 | 8258250 | 8258868 | fgeneshDP_pg.C_scaffold_425000002 | scaffold_425 | 1492 | 2034 |
| Block 1942 | DDB_G0277581 | 2 | 8259412 | 8260989 | GID1.0048924 | scaffold_425 | 2408 | 3940 |
| Block 1942 | rpl7a | 2 | 8267159 | 8268624 | GID1.0048926 | scaffold_425 | 6821 | 8323 |
| Block 1942 | DDB_G0277631 | 2 | 8268991 | 8269748 | gw1.425.17.1 | scaffold_425 | 6011 | 6479 |
| Block 1942 | DDB_G0277667 | 2 | 8273248 | 8274742 | GID1.0048937 | scaffold_425 | 22811 | 24134 |
| Block 178 | rpc4 | 2 | 8287649 | 8289022 | GID1.0042914 | scaffold_119 | 74303 | 75594 |
| Block 1868 | rps27 | 2 | 8291607 | 8292596 | e_gw1.408.9.1 | scaffold_408 | 23126 | 23452 |
| Block 1868 | DDB_G0277611 | 2 | 8293130 | 8296752 | GID1.0048724 | scaffold_408 | 24385 | 27648 |
| Block 178 | DDB_G0277613 | 2 | 8297290 | 8297757 | fgeneshDP_pg.C_scaffold_119000014 | scaffold_119 | 34768 | 35235 |
| Block 1040 | DDB_G0277673 | 2 | 8298462 | 8298875 | e_gw1.239.42.1 | scaffold_239 | 32781 | 33017 |
| Block 2581 | DDB_G0277697 | 2 | 8299171 | 8300297 | estExt_fgeneshDP_pg.C_6270002 | scaffold_627 | 1852 | 3300 |
| Block 2581 | DDB_G0277687 | 2 | 8302540 | 8308296 | GID1.0050458 | scaffold_627 | 8456 | 9289 |
| Block 1868 | cog1 | 2 | 8310259 | 8314937 | GID1.0048722 | scaffold_408 | 15612 | 21147 |
| Block 1449 | DDB_G0277675 | 2 | 8318008 | 8320411 | GID1.0047351 | scaffold_313 | 24587 | 27018 |
| Block 1449 | DDB_G0277685 | 2 | 8322248 | 8328904 | fgeneshDP_pm.C_scaffold_313000005 | scaffold_313 | 27880 | 33639 |
| Block 1449 | DDB_G0277695 | 2 | 8330243 | 8332402 | e_gw1.313.12.1 | scaffold_313 | 35343 | 36640 |
| Block 1414 | bzpH | 2 | 8335432 | 8337087 | GID1.0047237 | scaffold_307 | 33616 | 34937 |
| Block 1414 | DDB_G0277705 | 2 | 8339002 | 8340525 | gw1.307.8.1 | scaffold_307 | 36533 | 37150 |
| Block 2092 | apeA | 2 | 8354121 | 8355206 | gw1.465.15.1 | scaffold_465 | 7863 | 8678 |
| Block 2092 | DDB_G0277703 | 2 | 8355870 | 8356564 | fgeneshDP_pm.C_scaffold_465000004 | scaffold_465 | 9543 | 10294 |
| Block 1040 | DDB_G0277717 | 2 | 8356663 | 8357084 | e_gw1.239.41.1 | scaffold_239 | 31512 | 31997 |
| Block 2581 | DDB_G0277787 | 2 | 8357279 | 8359039 | estExt_fgeneshDP_pg.C_6270001 | scaffold_627 | 86 | 1114 |
| Block 180 | DDB_G0277767 | 2 | 8359729 | 8362242 | GID1.0042909 | scaffold_119 | 59363 | 61680 |
| Block 180 | DDB_G0277723 | 2 | 8362418 | 8364226 | estExt_fgeneshDP_kg.C_1190006 | scaffold_119 | 61918 | 63760 |
| Block 180 | thfA | 2 | 8366309 | 8367372 | estExt_Genewise1.C_1190043 | scaffold_119 | 65037 | 66605 |
| Block 180 | DDB_G0277789 | 2 | 8368321 | 8371835 | estExt_Genewise1.C_1190045 | scaffold_119 | 67315 | 70591 |
| Block 180 | uroc1 | 2 | 8372229 | 8374403 | estExt_Genewise1.C_1190049 | scaffold_119 | 70625 | 72837 |
| Block 1040 | valS1 | 2 | 8389174 | 8392491 | estExt_Genewise1.C_2390027 | scaffold_239 | 36880 | 40398 |
| Block 2034 | guaD | 2 | 8403823 | 8405499 | fgeneshDP_pg.C_scaffold_45000016 | scaffold_45 | 44278 | 45774 |
| Block 2034 | DDB_G0277745 | 2 | 8405559 | 8406440 | fgeneshDP_pg.C_scaffold_45000017 | scaffold_45 | 45866 | 46753 |
| Block 2034 | DDB_G0277747 | 2 | 8406857 | 8407795 | GID1.0040010 | scaffold_45 | 47381 | 48445 |
| Block 330 | sec24l | 2 | 8408970 | 8412467 | gw1.136.1.1 | scaffold_136 | 49939 | 53205 |
| Block 330 | DDB_G0277799 | 2 | 8413463 | 8415856 | fgeneshDP_pg.C_scaffold_136000026 | scaffold_136 | 61194 | 63842 |
| Block 330 | gtpbp5 | 2 | 8415951 | 8417837 | gw1.136.21.1 | scaffold_136 | 64009 | 65559 |
| Block 330 | rpl4 | 2 | 8418115 | 8419330 | fgeneshDP_pm.C_scaffold_136000013 | scaffold_136 | 65999 | 67297 |
| Block 330 | DDB_G0277805 | 2 | 8419431 | 8420426 | fgeneshDP_pg.C_scaffold_136000029 | scaffold_136 | 67504 | 68433 |
| Block 2094 | DDB_G0277813 | 2 | 8432351 | 8434762 | estExt_Genewise1Plus.C_4650016 | scaffold_465 | 14184 | 15086 |
| Block 2094 | acsA | 2 | 8435007 | 8437127 | estExt_Genewise1.C_4650022 | scaffold_465 | 16917 | 19406 |
| Block 1040 | dclre1c | 2 | 8438346 | 8441366 | e_gw1.239.5.1 | scaffold_239 | 23624 | 24729 |
| Block 1040 | DDB_G0277757 | 2 | 8442531 | 8443658 | estExt_fgeneshDP_pm.C_2390004 | scaffold_239 | 28092 | 29509 |
| Block 1040 | phyA | 2 | 8444415 | 8445485 | fgeneshDP_pg.C_scaffold_239000011 | scaffold_239 | 30489 | 31417 |
| Block 2094 | fszA | 2 | 8450337 | 8451971 | estExt_fgeneshDP_pg.C_4650008 | scaffold_465 | 15073 | 16715 |
| Block 2536 | pif1 | 3 | 12156 | 14409 | estExt_fgeneshDP_pg.C_610033 | scaffold_61 | 79621 | 81848 |
| Block 2536 | empD | 3 | 15087 | 16133 | estExt_fgeneshDP_pm.C_610005 | scaffold_61 | 38459 | 39375 |
| Block 2536 | mybH | 3 | 16340 | 20087 | GID1.0040716 | scaffold_61 | 16538 | 19828 |
| Block 2326 | parG | 3 | 42510 | 44486 | estExt_Genewise1.C_530084 | scaffold_53 | 100808 | 103005 |
| Block 2326 | nol9 | 3 | 44863 | 47023 | GID1.0040394 | scaffold_53 | 98608 | 100627 |
| Block 537 | pldC | 3 | 49404 | 54446 | estExt_Genewise1Plus.C_1640014 | scaffold_164 | 29816 | 34562 |
| Block 537 | DDB_G0277951 | 3 | 55112 | 55969 | fgeneshDP_pg.C_scaffold_164000009 | scaffold_164 | 27826 | 28588 |
| Block 537 | ints6 | 3 | 56545 | 59868 | fgeneshDP_pg.C_scaffold_164000008 | scaffold_164 | 24824 | 27385 |
| Block 537 | nup43 | 3 | 60115 | 61588 | e_gw1.164.27.1 | scaffold_164 | 23249 | 24560 |
| Block 537 | DDB_G0277957 | 3 | 62453 | 64198 | GID1.0044219 | scaffold_164 | 20978 | 22599 |
| Block 1797 | DDB_G0277967 | 3 | 72018 | 75420 | GID1.0048526 | scaffold_393 | 18758 | 22051 |
| Block 1797 | mlcE | 3 | 76885 | 77611 | estExt_fgeneshDP_kg.C_3930005 | scaffold_393 | 22918 | 23849 |
| Block 2538 | DDB_G0277971 | 3 | 80768 | 81314 | e_gw1.61.74.1 | scaffold_61 | 85971 | 86643 |
| Block 2538 | DDB_G0277973 | 3 | 83008 | 83922 | GID1.0040751 | scaffold_61 | 88438 | 89318 |
| Block 2538 | cxgS | 3 | 84465 | 84632 | GID1.0040752 | scaffold_61 | 89835 | 89999 |
| Block 2538 | xab2 | 3 | 88368 | 91113 | estExt_fgeneshDP_pg.C_610038 | scaffold_61 | 92271 | 96219 |
| Block 2632 | DDB_G0277893 | 3 | 95820 | 96613 | fgeneshDP_pm.C_scaffold_65000004 | scaffold_65 | 23352 | 24133 |
| Block 2632 | rimA | 3 | 96863 | 97960 | e_gw1.65.13.1 | scaffold_65 | 36564 | 37580 |
| Block 2632 | tbcB | 3 | 98599 | 99531 | fgeneshDP_pm.C_scaffold_65000005 | scaffold_65 | 29067 | 29910 |
| Block 537 | dhkJ | 3 | 102264 | 108452 | e_gw1.164.16.1 | scaffold_164 | 53521 | 59975 |
| Block 2538 | psiF | 3 | 109696 | 112817 | estExt_Genewise1.C_610055 | scaffold_61 | 82115 | 84782 |
| Block 1797 | ssbA | 3 | 113091 | 113951 | gw1.393.26.1 | scaffold_393 | 18017 | 18316 |
| Block 1797 | gxcA | 3 | 114291 | 118239 | estExt_fgeneshDP_pg.C_3930007 | scaffold_393 | 13566 | 17161 |
| Block 1797 | etfb | 3 | 125579 | 126515 | estExt_Genewise1.C_3930009 | scaffold_393 | 10662 | 11698 |
| Block 537 | DDB_G0277993 | 3 | 126886 | 128657 | GID1.0044227 | scaffold_164 | 40945 | 44191 |
| Block 537 | DDB_G0277997 | 3 | 130346 | 140572 | GID1.0044228 | scaffold_164 | 44324 | 53241 |
| Block 537 | ugpB | 3 | 140895 | 142743 | estExt_Genewise1Plus.C_1640007 | scaffold_164 | 16780 | 18836 |
| Block 2538 | DDB_G0278013 | 3 | 162120 | 162377 | estExt_Genewise1.C_610043 | scaffold_61 | 64552 | 65407 |
| Block 2538 | DDB_G0278015 | 3 | 164355 | 167062 | GID1.0040739 | scaffold_61 | 67362 | 69819 |
| Block 2538 | DDB_G0278017 | 3 | 167304 | 167864 | estExt_fgeneshDP_kg.C_610006 | scaffold_61 | 70061 | 70770 |
| Block 2538 | DDB_G0278021 | 3 | 170689 | 172404 | GID1.0040743 | scaffold_61 | 74315 | 75803 |
| Block 2538 | mrpl33 | 3 | 172946 | 173351 | GID1.0040744 | scaffold_61 | 76154 | 76479 |
| Block 2538 | DDB_G0278025 | 3 | 174453 | 175235 | fgeneshDP_pm.C_scaffold_61000011 | scaffold_61 | 77261 | 78008 |
| Block 2538 | snrpD1 | 3 | 175913 | 176563 | fgeneshDP_pg.C_scaffold_61000032 | scaffold_61 | 78570 | 79079 |
| Block 537 | cbpP | 3 | 179912 | 182605 | fgeneshDP_pm.C_scaffold_164000005 | scaffold_164 | 36815 | 38239 |
| Block 2538 | rpb8 | 3 | 186227 | 187053 | e_gw1.61.66.1 | scaffold_61 | 32423 | 32983 |
| Block 537 | fimA | 3 | 187950 | 189871 | estExt_fgeneshDP_kg.C_1640003 | scaffold_164 | 38556 | 40617 |
| Block 2400 | DDB_G0278047 | 3 | 191972 | 193220 | estExt_Genewise1.C_5590004 | scaffold_559 | 4058 | 5671 |
| Block 2400 | DDB_G0278049 | 3 | 193898 | 194222 | e_gw1.559.14.1 | scaffold_559 | 6192 | 6552 |
| Block 752 | ilvB | 3 | 198473 | 200536 | estExt_Genewise1Plus.C_20012 | scaffold_2 | 15262 | 17288 |
| Block 2635 | arrL | 3 | 206243 | 207086 | GID1.0040919 | scaffold_65 | 72677 | 73341 |
| Block 2635 | DDB_G0278059 | 3 | 207407 | 209554 | estExt_Genewise1Plus.C_650028 | scaffold_65 | 37680 | 39628 |
| Block 2635 | DDB_G0278061 | 3 | 209901 | 211233 | estExt_fgeneshDP_pg.C_650030 | scaffold_65 | 73658 | 74977 |
| Block 2635 | DDB_G0278063 | 3 | 211823 | 214784 | e_gw1.65.39.1 | scaffold_65 | 75219 | 78098 |
| Block 1795 | DDB_G0278075 | 3 | 233703 | 234872 | e_gw1.393.16.1 | scaffold_393 | 1384 | 3044 |
| Block 1795 | CRTF | 3 | 235235 | 237989 | GID1.0048518 | scaffold_393 | 1 | 690 |
| Block 752 | exdl2A | 3 | 242964 | 245120 | GID1.0037265 | scaffold_2 | 6818 | 8885 |
| Block 752 | pdiA | 3 | 246720 | 247623 | e_gw1.2.115.1 | scaffold_2 | 4398 | 5114 |
| Block 2400 | dymA | 3 | 252381 | 255025 | estExt_Genewise1.C_5590011 | scaffold_559 | 9378 | 11938 |
| Block 1530 | mecr | 3 | 262493 | 263754 | estExt_Genewise1.C_3310011 | scaffold_331 | 22068 | 23855 |
| Block 1530 | DDB_G0278097 | 3 | 264255 | 270651 | GID1.0047642 | scaffold_331 | 24228 | 29756 |
| Block 2400 | gnd | 3 | 273541 | 275022 | estExt_Genewise1.C_5590001 | scaffold_559 | 671 | 2508 |
| Block 752 | DDB_G0278105 | 3 | 280166 | 282713 | fgeneshDP_pm.C_scaffold_2000015 | scaffold_2 | 118874 | 121319 |
| Block 752 | DDB_G0278107 | 3 | 283161 | 283844 | fgeneshDP_pm.C_scaffold_2000016 | scaffold_2 | 122205 | 122831 |
| Block 2400 | DDB_G0278597 | 3 | 287423 | 288337 | GID1.0050059 | scaffold_559 | 7076 | 8356 |
| Block 86 | DDB_G0278111 | 3 | 294308 | 294963 | gw1.107.45.1 | scaffold_107 | 74513 | 74837 |
| Block 86 | DDB_G0278113 | 3 | 295249 | 295926 | GID1.0042519 | scaffold_107 | 73503 | 74159 |
| Block 828 | DDB_G0278115 | 3 | 296602 | 297542 | GID1.0045262 | scaffold_206 | 43635 | 44566 |
| Block 828 | DDB_G0278601 | 3 | 297893 | 302229 | estExt_fgeneshDP_pg.C_2060010 | scaffold_206 | 25809 | 29681 |
| Block 828 | sigE | 3 | 302308 | 303514 | fgeneshDP_pg.C_scaffold_206000011 | scaffold_206 | 29737 | 30730 |
| Block 828 | arcA | 3 | 304397 | 306034 | estExt_fgeneshDP_pm.C_2060004 | scaffold_206 | 31267 | 33041 |
| Block 828 | sec61b | 3 | 306850 | 307279 | e_gw1.206.41.1 | scaffold_206 | 34311 | 34742 |
| Block 86 | mvd | 3 | 313759 | 315215 | e_gw1.107.34.1 | scaffold_107 | 76739 | 77980 |
| Block 482 | DDB_G0278609 | 3 | 316046 | 317868 | GID1.0043976 | scaffold_155 | 18809 | 20754 |
| Block 1901 | DDB_G0278125 | 3 | 327348 | 329843 | estExt_fgeneshDP_pm.C_4140005 | scaffold_414 | 22830 | 25741 |
| Block 1901 | uglA | 3 | 330250 | 331290 | GID1.0048794 | scaffold_414 | 21499 | 22595 |
| Block 1901 | gefI | 3 | 332007 | 334836 | e_gw1.414.3.1 | scaffold_414 | 18015 | 20470 |
| Block 482 | DDB_G0278127 | 3 | 335063 | 335396 | estExt_Genewise1.C_1550015 | scaffold_155 | 18315 | 18618 |
| Block 2077 | vps11 | 3 | 343671 | 346615 | fgeneshDP_pg.C_scaffold_46000029 | scaffold_46 | 84562 | 87601 |
| Block 2077 | DDB_G0278143 | 3 | 347604 | 352895 | estExt_fgeneshDP_pg.C_460030 | scaffold_46 | 88389 | 94570 |
| Block 482 | gxcW | 3 | 354814 | 358184 | GID1.0043974 | scaffold_155 | 14983 | 17751 |
| Block 2325 | patA | 3 | 364618 | 368104 | GID1.0040393 | scaffold_53 | 92128 | 95574 |
| Block 2636 | DDB_G0278151 | 3 | 372488 | 373942 | GID1.0040904 | scaffold_65 | 40004 | 41410 |
| Block 2636 | tmem111 | 3 | 374402 | 375450 | GID1.0040905 | scaffold_65 | 41874 | 42941 |
| Block 2641 | mai | 3 | 376116 | 376878 | estExt_Genewise1Plus.C_650068 | scaffold_65 | 94981 | 95803 |
| Block 2641 | sdhD | 3 | 377175 | 377835 | GID1.0040927 | scaffold_65 | 96045 | 96608 |
| Block 1013 | pssA | 3 | 378150 | 379977 | e_gw1.234.2.1 | scaffold_234 | 20012 | 21242 |
| Block 1013 | ptpB | 3 | 381607 | 383318 | estExt_fgeneshDP_kg.C_2340002 | scaffold_234 | 16099 | 17800 |
| Block 2537 | DDB_G0278161 | 3 | 385354 | 387534 | estExt_Genewise1Plus.C_610031 | scaffold_61 | 44333 | 49597 |
| Block 2537 | DDB_G0278163 | 3 | 387740 | 388320 | GID1.0040729 | scaffold_61 | 39625 | 40227 |
| Block 2537 | DDB_G0278169 | 3 | 391453 | 392277 | GID1.0040717 | scaffold_61 | 19888 | 20670 |
| Block 2537 | DDB_G0278171 | 3 | 393144 | 395891 | GID1.0040718 | scaffold_61 | 20875 | 24379 |
| Block 2325 | DDB_G0278179 | 3 | 400484 | 402497 | e_gw1.53.49.1 | scaffold_53 | 103338 | 105342 |
| Block 1013 | DDB_G0278181 | 3 | 405756 | 408648 | fgeneshDP_pg.C_scaffold_234000003 | scaffold_234 | 12518 | 15024 |
| Block 2537 | ints11 | 3 | 423737 | 426046 | e_gw1.61.5.1 | scaffold_61 | 25863 | 27330 |
| Block 2537 | dak | 3 | 426271 | 427288 | estExt_Genewise1Plus.C_610019 | scaffold_61 | 28371 | 29442 |
| Block 2537 | DDB_G0278621 | 3 | 427974 | 430638 | estExt_fgeneshDP_pg.C_610012 | scaffold_61 | 29923 | 32361 |
| Block 2537 | DDB_G0278627 | 3 | 435839 | 438592 | GID1.0040727 | scaffold_61 | 35561 | 38280 |
| Block 2537 | DDB_G0277899 | 3 | 439645 | 440608 | GID1.0040726 | scaffold_61 | 34238 | 35115 |
| Block 536 | DDB_G0278193 | 3 | 444626 | 446858 | estExt_Genewise1Plus.C_1640002 | scaffold_164 | 2279 | 4510 |
| Block 536 | DDB_G0278195 | 3 | 447655 | 448305 | e_gw1.164.30.1 | scaffold_164 | 4708 | 5355 |
| Block 536 | DDB_G0278197 | 3 | 448708 | 454535 | GID1.0044216 | scaffold_164 | 5603 | 9509 |
| Block 536 | DDB_G0278629 | 3 | 458790 | 461950 | fgeneshDP_pm.C_scaffold_164000002 | scaffold_164 | 14296 | 16724 |
| Block 753 | xrcc4 | 3 | 471533 | 473001 | fgeneshDP_pg.C_scaffold_2000006 | scaffold_2 | 11435 | 12876 |
| Block 753 | eIF1a | 3 | 473374 | 473946 | estExt_fgeneshDP_kg.C_20001 | scaffold_2 | 13184 | 13775 |
| Block 343 | DDB_G0278225 | 3 | 509241 | 509610 | estExt_fgeneshDP_kg.C_1390001 | scaffold_139 | 1088 | 1660 |
| Block 343 | DDB_G0278227 | 3 | 509789 | 511581 | gw1.139.36.1 | scaffold_139 | 1823 | 3859 |
| Block 343 | DDB_G0278229 | 3 | 512537 | 515209 | GID1.0043509 | scaffold_139 | 4636 | 7359 |
| Block 753 | DDB_G0278231 | 3 | 515488 | 515895 | GID1.0037319 | scaffold_2 | 134419 | 134841 |
| Block 753 | DDB_G0278233 | 3 | 516517 | 517431 | fgeneshDP_pg.C_scaffold_2000053 | scaffold_2 | 136955 | 137941 |
| Block 753 | DDB_G0294557 | 3 | 517463 | 519124 | estExt_fgeneshDP_pg.C_20054 | scaffold_2 | 140296 | 142144 |
| Block 2539 | dhkB | 3 | 525688 | 531677 | GID1.0040734 | scaffold_61 | 50569 | 55997 |
| Block 2539 | DDB_G0278239 | 3 | 533251 | 534784 | GID1.0040735 | scaffold_61 | 57163 | 58692 |
| Block 2539 | clcD | 3 | 535599 | 538688 | GID1.0040736 | scaffold_61 | 59161 | 62030 |
| Block 2539 | tcea1 | 3 | 539874 | 541072 | fgeneshDP_pg.C_scaffold_61000026 | scaffold_61 | 62927 | 64336 |
| Block 2637 | DDB_G0278253 | 3 | 566464 | 568173 | fgeneshDP_pm.C_scaffold_65000016 | scaffold_65 | 59895 | 61412 |
| Block 2637 | DDB_G0278255 | 3 | 568794 | 570338 | gw1.65.52.1 | scaffold_65 | 61467 | 63286 |
| Block 2637 | DDB_G0278257 | 3 | 571285 | 572943 | GID1.0040917 | scaffold_65 | 65211 | 66747 |
| Block 467 | manB | 3 | 573780 | 576993 | e_gw1.153.12.1 | scaffold_153 | 39841 | 43313 |
| Block 467 | DDB_G0278261 | 3 | 577308 | 579437 | GID1.0043923 | scaffold_153 | 43775 | 46720 |
| Block 716 | rpa12 | 3 | 579706 | 580766 | e_gw1.193.44.1 | scaffold_193 | 44426 | 44909 |
| Block 716 | DDB_G0278267 | 3 | 586381 | 589126 | GID1.0044938 | scaffold_193 | 36436 | 38907 |
| Block 2203 | snfA | 3 | 589784 | 592434 | estExt_Genewise1Plus.C_4950006 | scaffold_495 | 6010 | 8336 |
| Block 2203 | DDB_G0278269 | 3 | 593095 | 594230 | fgeneshDP_pg.C_scaffold_495000005 | scaffold_495 | 8551 | 9511 |
| Block 2203 | DDB_G0278271 | 3 | 599269 | 601031 | GID1.0049607 | scaffold_495 | 14025 | 15343 |
| Block 2203 | DDB_G0278273 | 3 | 601284 | 602879 | e_gw1.495.4.1 | scaffold_495 | 15410 | 16897 |
| Block 1091 | rpe | 3 | 603034 | 603814 | estExt_fgeneshDP_kg.C_2490003 | scaffold_249 | 20386 | 21306 |
| Block 1091 | cspA | 3 | 604573 | 605887 | estExt_fgeneshDP_pg.C_2490008 | scaffold_249 | 21940 | 23221 |
| Block 1091 | DDB_G0278277 | 3 | 606213 | 607054 | e_gw1.249.33.1 | scaffold_249 | 23553 | 24228 |
| Block 1091 | mlcD | 3 | 607477 | 608249 | fgeneshDP_pg.C_scaffold_249000010 | scaffold_249 | 24597 | 25250 |
| Block 963 | DDB_G0278281 | 3 | 614554 | 615842 | GID1.0045713 | scaffold_227 | 14297 | 15546 |
| Block 1091 | fol1 | 3 | 616133 | 618179 | GID1.0046131 | scaffold_249 | 2955 | 4877 |
| Block 1091 | copB2 | 3 | 618891 | 622611 | estExt_Genewise1.C_2490024 | scaffold_249 | 28558 | 32156 |
| Block 963 | ileS | 3 | 632971 | 636359 | estExt_Genewise1.C_2270013 | scaffold_227 | 7916 | 11532 |
| Block 963 | DDB_G0278649 | 3 | 636762 | 637763 | GID1.0045710 | scaffold_227 | 6487 | 7567 |
| Block 963 | DDB_G0278295 | 3 | 638585 | 639322 | e_gw1.227.28.1 | scaffold_227 | 4572 | 5000 |
| Block 963 | DDB_G0278297 | 3 | 639451 | 640653 | e_gw1.227.23.1 | scaffold_227 | 2289 | 3719 |
| Block 1091 | DDB_G0278301 | 3 | 647938 | 651814 | estExt_fgeneshDP_pg.C_2490001 | scaffold_249 | 197 | 2893 |
| Block 363 | manE | 3 | 655951 | 659720 | fgeneshDP_pg.C_scaffold_14000052 | scaffold_14 | 141816 | 145338 |
| Block 363 | DDB_G0278311 | 3 | 668576 | 671801 | GID1.0038293 | scaffold_14 | 145819 | 148736 |
| Block 963 | DDB_G0278315 | 3 | 683079 | 685187 | GID1.0045716 | scaffold_227 | 20120 | 21770 |
| Block 963 | TFIIIC3 | 3 | 689224 | 692344 | GID1.0045715 | scaffold_227 | 17002 | 19881 |
| Block 84 | DDB_G0278661 | 3 | 695788 | 700246 | estExt_fgeneshDP_pg.C_1070020 | scaffold_107 | 54432 | 58599 |
| Block 84 | DDB_G0278663 | 3 | 701606 | 704101 | estExt_Genewise1Plus.C_1070033 | scaffold_107 | 51587 | 54047 |
| Block 1515 | DDB_G0278327 | 3 | 707798 | 709539 | GID1.0047614 | scaffold_329 | 34262 | 35717 |
| Block 1515 | DDB_G0278329 | 3 | 709648 | 710709 | GID1.0047613 | scaffold_329 | 33362 | 34177 |
| Block 3015 | DDB_G0278333 | 3 | 713907 | 716110 | fgeneshDP_pg.C_scaffold_94000012 | scaffold_94 | 28138 | 30211 |
| Block 3015 | snrpC | 3 | 716610 | 717246 | e_gw1.94.28.1 | scaffold_94 | 27221 | 27475 |
| Block 1515 | DDB_G0278337 | 3 | 717847 | 718532 | GID1.0047600 | scaffold_329 | 3689 | 4284 |
| Block 1515 | DDB_G0278339 | 3 | 719660 | 721905 | GID1.0047599 | scaffold_329 | 377 | 2535 |
| Block 2631 | DDB_G0278341 | 3 | 722601 | 724035 | estExt_Genewise1Plus.C_650034 | scaffold_65 | 43211 | 44796 |
| Block 2631 | acly | 3 | 726196 | 728064 | estExt_fgeneshDP_kg.C_650002 | scaffold_65 | 46775 | 49188 |
| Block 2631 | DDB_G0278347 | 3 | 729623 | 730084 | GID1.0040909 | scaffold_65 | 50542 | 50961 |
| Block 2631 | DDB_G0278349 | 3 | 731119 | 732534 | fgeneshDP_pm.C_scaffold_65000013 | scaffold_65 | 51818 | 52821 |
| Block 2631 | DDB_G0278351 | 3 | 734436 | 737543 | GID1.0040912 | scaffold_65 | 56038 | 57648 |
| Block 2631 | smu1 | 3 | 737677 | 739535 | fgeneshDP_pg.C_scaffold_65000023 | scaffold_65 | 57826 | 59681 |
| Block 1070 | DDB_G0278355 | 3 | 739670 | 741739 | GID1.0046016 | scaffold_243 | 20875 | 22995 |
| Block 1070 | DDB_G0278357 | 3 | 742074 | 742494 | e_gw1.243.65.1 | scaffold_243 | 19978 | 20139 |
| Block 1515 | rab1B | 3 | 743092 | 744234 | estExt_Genewise1.C_3290017 | scaffold_329 | 29651 | 31472 |
| Block 1515 | ctr9 | 3 | 746082 | 749671 | estExt_Genewise1.C_3290013 | scaffold_329 | 25093 | 28351 |
| Block 2631 | DDB_G0278665 | 3 | 753135 | 757040 | fgeneshDP_pm.C_scaffold_65000001 | scaffold_65 | 11424 | 14913 |
| Block 2631 | DDB_G0278367 | 3 | 766301 | 768775 | fgeneshDP_pg.C_scaffold_65000004 | scaffold_65 | 7147 | 10269 |
| Block 2631 | DDB_G0278369 | 3 | 768975 | 770144 | e_gw1.65.56.1 | scaffold_65 | 5612 | 6775 |
| Block 2631 | spc1 | 3 | 770499 | 770858 | gw1.65.74.1 | scaffold_65 | 22827 | 23129 |
| Block 2631 | DDB_G0278373 | 3 | 771365 | 773623 | e_gw1.65.24.1 | scaffold_65 | 16733 | 17449 |
| Block 2631 | helB2 | 3 | 776296 | 778895 | estExt_Genewise1Plus.C_650013 | scaffold_65 | 18081 | 20279 |
| Block 2631 | DDB_G0278375 | 3 | 779475 | 780824 | fgeneshDP_pg.C_scaffold_65000008 | scaffold_65 | 21125 | 22431 |
| Block 1918 | DDB_G0278669 | 3 | 781169 | 782398 | e_gw1.42.38.1 | scaffold_42 | 84350 | 85615 |
| Block 1918 | DDB_G0278671 | 3 | 784969 | 786624 | estExt_fgeneshDP_kg.C_420007 | scaffold_42 | 81632 | 84157 |
| Block 1918 | maf1 | 3 | 788140 | 789126 | estExt_fgeneshDP_pm.C_420013 | scaffold_42 | 80244 | 81279 |
| Block 1918 | apm3 | 3 | 792608 | 794195 | e_gw1.42.36.1 | scaffold_42 | 77891 | 79370 |
| Block 1918 | bzpD | 3 | 795514 | 798263 | GID1.0039857 | scaffold_42 | 73681 | 75832 |
| Block 1918 | gacA | 3 | 800738 | 802121 | e_gw1.42.61.1 | scaffold_42 | 72049 | 73143 |
| Block 1918 | DDB_G0278383 | 3 | 803175 | 803642 | GID1.0039855 | scaffold_42 | 70256 | 70681 |
| Block 1918 | CYP513F1 | 3 | 805261 | 806859 | GID1.0039847 | scaffold_42 | 44594 | 47829 |
| Block 2029 | DDB_G0278395 | 3 | 819227 | 821289 | estExt_fgeneshDP_pg.C_4490010 | scaffold_449 | 18256 | 19992 |
| Block 470 | DDB_G0278407 | 3 | 833181 | 836091 | estExt_fgeneshDP_pg.C_1530027 | scaffold_153 | 63432 | 66080 |
| Block 470 | shkC | 3 | 836437 | 838779 | estExt_Genewise1Plus.C_1530046 | scaffold_153 | 60154 | 62754 |
| Block 710 | rsc12 | 3 | 845743 | 848931 | estExt_Genewise1.C_1920003 | scaffold_192 | 19602 | 22865 |
| Block 710 | DDB_G0278411 | 3 | 849816 | 851130 | fgeneshDP_pg.C_scaffold_192000009 | scaffold_192 | 23174 | 24262 |
| Block 710 | clmA | 3 | 852176 | 854714 | estExt_fgeneshDP_pg.C_1920016 | scaffold_192 | 39264 | 41889 |
| Block 710 | eRF3 | 3 | 855258 | 856931 | e_gw1.192.4.1 | scaffold_192 | 34975 | 36588 |
| Block 710 | DDB_G0278415 | 3 | 857716 | 858660 | GID1.0044910 | scaffold_192 | 33208 | 34237 |
| Block 710 | xacB | 3 | 859128 | 863604 | GID1.0044907 | scaffold_192 | 24341 | 28857 |
| Block 357 | DDB_G0278425 | 3 | 866714 | 870080 | e_gw1.14.96.1 | scaffold_14 | 78852 | 79619 |
| Block 357 | DDB_G0278427 | 3 | 870597 | 871423 | GID1.0038268 | scaffold_14 | 81901 | 83122 |
| Block 357 | rps20 | 3 | 871775 | 872149 | estExt_fgeneshDP_kg.C_140006 | scaffold_14 | 54387 | 54823 |
| Block 357 | DDB_G0278431 | 3 | 872674 | 874217 | GID1.0038260 | scaffold_14 | 56973 | 58491 |
| Block 357 | DDB_G0278433 | 3 | 875590 | 877535 | GID1.0038261 | scaffold_14 | 59516 | 60580 |
| Block 357 | DDB_G0278435 | 3 | 877848 | 880508 | GID1.0038262 | scaffold_14 | 60719 | 63274 |
| Block 357 | DDB_G0278441 | 3 | 883102 | 884257 | estExt_fgeneshDP_pg.C_140029 | scaffold_14 | 77603 | 78690 |
| Block 357 | DDB_G0278443 | 3 | 885094 | 886826 | GID1.0038265 | scaffold_14 | 75321 | 76629 |
| Block 357 | pigO | 3 | 887014 | 890500 | GID1.0038264 | scaffold_14 | 66609 | 75066 |
| Block 2633 | DDB_G0278453 | 3 | 901380 | 904104 | GID1.0040898 | scaffold_65 | 25544 | 28493 |
| Block 2633 | DDB_G0278455 | 3 | 904324 | 905523 | fgeneshDP_pg.C_scaffold_65000010 | scaffold_65 | 24387 | 25334 |
| Block 1553 | ndrA | 3 | 907158 | 908849 | estExt_Genewise1.C_3380017 | scaffold_338 | 31844 | 34170 |
| Block 2843 | DDB_G0278469 | 3 | 924371 | 926602 | estExt_fgeneshDP_pg.C_780004 | scaffold_78 | 7198 | 9925 |
| Block 2843 | DDB_G0278471 | 3 | 926878 | 928461 | estExt_fgeneshDP_kg.C_780002 | scaffold_78 | 9926 | 10739 |
| Block 1939 | DDB_G0278475 | 3 | 932374 | 934337 | GID1.0048915 | scaffold_424 | 4614 | 6276 |
| Block 2042 | DDB_G0278479 | 3 | 936199 | 937743 | e_gw1.450.5.1 | scaffold_450 | 16118 | 17581 |
| Block 1553 | DDB_G0278691 | 3 | 937883 | 939277 | estExt_fgeneshDP_pm.C_3380005 | scaffold_338 | 29473 | 30846 |
| Block 1553 | DDB_G0278693 | 3 | 939361 | 941535 | gw1.338.1.1 | scaffold_338 | 27483 | 29349 |
| Block 1939 | dhkK | 3 | 942210 | 945957 | estExt_Genewise1.C_4240001 | scaffold_424 | 885 | 4542 |
| Block 2690 | DDB_G0278481 | 3 | 950150 | 951676 | fgeneshDP_pm.C_scaffold_674000001 | scaffold_674 | 4404 | 6418 |
| Block 2690 | NGAP | 3 | 951936 | 954731 | GID1.0050658 | scaffold_674 | 1398 | 4082 |
| Block 2042 | DDB_G0278487 | 3 | 959760 | 962183 | e_gw1.450.2.1 | scaffold_450 | 18736 | 20753 |
| Block 2027 | DDB_G0278489 | 3 | 962952 | 964582 | e_gw1.449.8.1 | scaffold_449 | 14025 | 15515 |
| Block 2027 | DDB_G0278491 | 3 | 965453 | 966959 | e_gw1.449.7.1 | scaffold_449 | 11376 | 12770 |
| Block 2027 | DDB_G0278493 | 3 | 967226 | 967869 | GID1.0049154 | scaffold_449 | 10475 | 11086 |
| Block 2027 | alp | 3 | 968065 | 969744 | e_gw1.449.3.1 | scaffold_449 | 8694 | 10343 |
| Block 22 | DDB_G0278497 | 3 | 971318 | 972954 | gw1.10.63.1 | scaffold_10 | 87522 | 88960 |
| Block 22 | DDB_G0278499 | 3 | 974509 | 975668 | estExt_Genewise1.C_100071 | scaffold_10 | 88936 | 91996 |
| Block 22 | DDB_G0278501 | 3 | 976180 | 978681 | fgeneshDP_pg.C_scaffold_10000037 | scaffold_10 | 93480 | 95680 |
| Block 353 | DDB_G0278507 | 3 | 981524 | 983660 | estExt_Genewise1.C_140007 | scaffold_14 | 6105 | 7869 |
| Block 353 | DDB_G0278509 | 3 | 984753 | 988653 | GID1.0038239 | scaffold_14 | 1631 | 5249 |
| Block 2529 | DDB_G0278517 | 3 | 995255 | 996064 | GID1.0050343 | scaffold_605 | 11692 | 11916 |
| Block 2529 | mfeB | 3 | 996509 | 997473 | estExt_Genewise1Plus.C_6050011 | scaffold_605 | 10410 | 11530 |
| Block 353 | DG1105 | 3 | 1006468 | 1011515 | estExt_fgeneshDP_pm.C_140007 | scaffold_14 | 38276 | 43003 |
| Block 353 | DDB_G0278701 | 3 | 1012938 | 1018512 | GID1.0038251 | scaffold_14 | 30848 | 36181 |
| Block 353 | DDB_G0278527 | 3 | 1022106 | 1025121 | GID1.0038250 | scaffold_14 | 27312 | 29977 |
| Block 2529 | rplP1 | 3 | 1026839 | 1027765 | GID1.0050341 | scaffold_605 | 9279 | 10220 |
| Block 2508 | rpl5 | 3 | 1039561 | 1040650 | estExt_Genewise1Plus.C_60020 | scaffold_6 | 30799 | 31956 |
| Block 2508 | DDB_G0278541 | 3 | 1041012 | 1042497 | estExt_Genewise1Plus.C_60016 | scaffold_6 | 28844 | 30534 |
| Block 437 | srpRB | 3 | 1043259 | 1044326 | GID1.0038344 | scaffold_15 | 114841 | 115925 |
| Block 3039 | fimC | 3 | 1046298 | 1049939 | gw1.96.17.1 | scaffold_96 | 56565 | 60207 |
| Block 3039 | DDB_G0278547 | 3 | 1050062 | 1052275 | e_gw1.96.23.1 | scaffold_96 | 60285 | 62372 |
| Block 437 | gacB | 3 | 1056077 | 1057365 | estExt_fgeneshDP_pg.C_150042 | scaffold_15 | 119897 | 122208 |
| Block 2508 | DDB_G0278705 | 3 | 1058155 | 1059582 | fgeneshDP_pg.C_scaffold_6000025 | scaffold_6 | 58646 | 59782 |
| Block 1770 | DDB_G0278551 | 3 | 1060290 | 1062395 | e_gw1.39.8.1 | scaffold_39 | 11057 | 13006 |
| Block 1770 | pdhC | 3 | 1063899 | 1066276 | GID1.0039692 | scaffold_39 | 7790 | 10072 |
| Block 1770 | DDB_G0278709 | 3 | 1084509 | 1086368 | e_gw1.39.67.1 | scaffold_39 | 13581 | 15010 |
| Block 1770 | nedd8 | 3 | 1086802 | 1087339 | e_gw1.39.108.1 | scaffold_39 | 15316 | 15760 |
| Block 1770 | DDB_G0278713 | 3 | 1087624 | 1088613 | e_gw1.39.21.1 | scaffold_39 | 16041 | 17006 |
| Block 1770 | DDB_G0278567 | 3 | 1089249 | 1090634 | fgeneshDP_pg.C_scaffold_39000010 | scaffold_39 | 17509 | 18853 |
| Block 1770 | DDB_G0278569 | 3 | 1091191 | 1092112 | estExt_fgeneshDP_pg.C_390012 | scaffold_39 | 21021 | 21917 |
| Block 1770 | DDB_G0278571 | 3 | 1092876 | 1093321 | estExt_Genewise1.C_390033 | scaffold_39 | 22114 | 22808 |
| Block 1770 | DDB_G0278573 | 3 | 1094317 | 1095126 | estExt_fgeneshDP_kg.C_390006 | scaffold_39 | 23601 | 24465 |
| Block 21 | DDB_G0278747 | 3 | 1113093 | 1114781 | e_gw1.10.57.1 | scaffold_10 | 108066 | 109706 |
| Block 21 | DDB_G0278751 | 3 | 1117749 | 1118645 | fgeneshDP_pm.C_scaffold_10000021 | scaffold_10 | 104527 | 105360 |
| Block 21 | DDB_G0278753 | 3 | 1118934 | 1119938 | gw1.10.106.1 | scaffold_10 | 103887 | 104192 |
| Block 21 | gacR | 3 | 1120346 | 1122813 | fgeneshDP_pm.C_scaffold_10000020 | scaffold_10 | 100693 | 103117 |
| Block 21 | DDB_G0278757 | 3 | 1123519 | 1127550 | estExt_fgeneshDP_pg.C_100038 | scaffold_10 | 96207 | 100163 |
| Block 21 | DDB_G0278761 | 3 | 1131314 | 1136948 | GID1.0038004 | scaffold_10 | 81543 | 86726 |
| Block 1725 | cstf1 | 3 | 1141749 | 1143269 | fgeneshDP_pm.C_scaffold_376000002 | scaffold_376 | 11625 | 13064 |
| Block 1725 | DDB_G0278771 | 3 | 1143468 | 1147597 | GID1.0048290 | scaffold_376 | 13368 | 16527 |
| Block 1725 | DDB_G0278773 | 3 | 1149897 | 1151429 | fgeneshDP_pg.C_scaffold_376000006 | scaffold_376 | 18680 | 19999 |
| Block 1725 | DDB_G0278775 | 3 | 1152307 | 1152877 | GID1.0048292 | scaffold_376 | 20862 | 21411 |
| Block 1725 | pikA | 3 | 1154369 | 1159209 | estExt_Genewise1Plus.C_3760013 | scaffold_376 | 22658 | 27480 |
| Block 350 | aco2 | 3 | 1168705 | 1171255 | estExt_Genewise1.C_1390040 | scaffold_139 | 56798 | 59737 |
| Block 350 | pah | 3 | 1172682 | 1174440 | estExt_Genewise1.C_1390033 | scaffold_139 | 49134 | 50885 |
| Block 350 | elp4 | 3 | 1174736 | 1176055 | GID1.0043527 | scaffold_139 | 41831 | 48998 |
| Block 350 | DDB_G0278785 | 3 | 1176379 | 1177366 | e_gw1.139.39.1 | scaffold_139 | 39035 | 39917 |
| Block 2904 | DDB_G0278789 | 3 | 1182227 | 1183696 | fgeneshDP_pg.C_scaffold_81000019 | scaffold_81 | 43367 | 44890 |
| Block 2904 | DDB_G0278791 | 3 | 1184009 | 1185447 | fgeneshDP_pg.C_scaffold_81000013 | scaffold_81 | 30153 | 31340 |
| Block 2904 | DDB_G0278793 | 3 | 1186077 | 1187040 | e_gw1.81.59.1 | scaffold_81 | 46772 | 47519 |
| Block 2904 | I6KA | 3 | 1187328 | 1189572 | GID1.0041543 | scaffold_81 | 18833 | 20661 |
| Block 2904 | DDB_G0278795 | 3 | 1191521 | 1192327 | fgeneshDP_pm.C_scaffold_81000005 | scaffold_81 | 22373 | 23295 |
| Block 2904 | DDB_G0278797 | 3 | 1192386 | 1193618 | estExt_Genewise1Plus.C_810012 | scaffold_81 | 23339 | 24608 |
| Block 2904 | pgtD | 3 | 1197002 | 1203965 | fgeneshDP_pm.C_scaffold_81000012 | scaffold_81 | 48587 | 57659 |
| Block 203 | DDB_G0278801 | 3 | 1206396 | 1207886 | fgeneshDP_pg.C_scaffold_121000009 | scaffold_121 | 28710 | 29900 |
| Block 203 | dus1l | 3 | 1208252 | 1209559 | GID1.0042963 | scaffold_121 | 30221 | 31429 |
| Block 203 | DDB_G0278805 | 3 | 1209711 | 1210757 | GID1.0042964 | scaffold_121 | 31645 | 32611 |
| Block 203 | DDB_G0278807 | 3 | 1213734 | 1214127 | estExt_fgeneshDP_kg.C_1210003 | scaffold_121 | 35077 | 35964 |
| Block 203 | DDB_G0278937 | 3 | 1214678 | 1217558 | e_gw1.121.2.1 | scaffold_121 | 36719 | 38278 |
| Block 203 | rckA | 3 | 1218919 | 1222296 | GID1.0042968 | scaffold_121 | 41017 | 43152 |
| Block 2507 | eIF5b | 3 | 1232003 | 1235140 | gw1.6.3.1 | scaffold_6 | 16828 | 19641 |
| Block 2507 | DDB_G0278817 | 3 | 1235984 | 1237920 | GID1.0037649 | scaffold_6 | 14004 | 15770 |
| Block 2507 | DDB_G0278819 | 3 | 1238183 | 1240456 | estExt_fgeneshDP_pm.C_60002 | scaffold_6 | 11295 | 13514 |
| Block 429 | snrpF | 3 | 1243062 | 1243559 | fgeneshDP_pm.C_scaffold_15000008 | scaffold_15 | 24845 | 25302 |
| Block 429 | fps | 3 | 1245549 | 1247206 | GID1.0038304 | scaffold_15 | 22726 | 24618 |
| Block 429 | DDB_G0278945 | 3 | 1251221 | 1252764 | e_gw1.15.71.1 | scaffold_15 | 18015 | 19135 |
| Block 429 | DDB_G0278829 | 3 | 1258942 | 1260744 | fgeneshDP_pm.C_scaffold_15000002 | scaffold_15 | 13486 | 15225 |
| Block 429 | DDB_G0278831 | 3 | 1261226 | 1261756 | e_gw1.15.91.1 | scaffold_15 | 15811 | 16630 |
| Block 429 | DDB_G0278839 | 3 | 1264824 | 1265291 | estExt_fgeneshDP_kg.C_150001 | scaffold_15 | 16789 | 17707 |
| Block 2902 | DDB_G0278841 | 3 | 1265394 | 1267160 | fgeneshDP_pg.C_scaffold_81000018 | scaffold_81 | 41279 | 42781 |
| Block 2902 | taf11 | 3 | 1267448 | 1268800 | gw1.81.29.1 | scaffold_81 | 38147 | 38434 |
| Block 2902 | DDB_G0278845 | 3 | 1269374 | 1273426 | fgeneshDP_pm.C_scaffold_81000009 | scaffold_81 | 33423 | 36674 |
| Block 2902 | psmA6 | 3 | 1274786 | 1275953 | estExt_fgeneshDP_pm.C_810008 | scaffold_81 | 31393 | 32601 |
| Block 2902 | etfdh | 3 | 1276262 | 1278082 | GID1.0041552 | scaffold_81 | 38705 | 40710 |
| Block 2179 | DDB_G0278851 | 3 | 1278474 | 1281570 | estExt_Genewise1.C_4870014 | scaffold_487 | 16827 | 19512 |
| Block 2179 | DDB_G0278853 | 3 | 1282301 | 1282690 | GID1.0049534 | scaffold_487 | 15393 | 15746 |
| Block 2179 | DAip1 | 3 | 1283399 | 1285516 | estExt_Genewise1.C_4870011 | scaffold_487 | 12749 | 14941 |
| Block 2902 | DDB_G0278855 | 3 | 1285622 | 1288868 | fgeneshDP_pg.C_scaffold_81000003 | scaffold_81 | 4457 | 7433 |
| Block 2902 | DDB_G0278857 | 3 | 1289087 | 1289795 | GID1.0041538 | scaffold_81 | 3602 | 4293 |
| Block 2902 | captB | 3 | 1290736 | 1292456 | estExt_fgeneshDP_pm.C_810001 | scaffold_81 | 1053 | 2711 |
| Block 20 | fray1 | 3 | 1298188 | 1299912 | estExt_fgeneshDP_kg.C_100012 | scaffold_10 | 79477 | 81001 |
| Block 20 | gefBB | 3 | 1304620 | 1310875 | GID1.0038002 | scaffold_10 | 71174 | 75996 |
| Block 144 | p17 | 3 | 1316821 | 1317345 | estExt_fgeneshDP_kg.C_1150005 | scaffold_115 | 18482 | 19066 |
| Block 144 | DDB_G0278871 | 3 | 1320697 | 1321245 | fgeneshDP_pg.C_scaffold_115000009 | scaffold_115 | 15953 | 16525 |
| Block 144 | gnpda1 | 3 | 1321299 | 1322102 | estExt_fgeneshDP_kg.C_1150004 | scaffold_115 | 16729 | 17555 |
| Block 144 | DDB_G0278953 | 3 | 1335145 | 1336477 | fgeneshDP_pg.C_scaffold_115000007 | scaffold_115 | 13011 | 14249 |
| Block 144 | DDB_G0278883 | 3 | 1337376 | 1338350 | fgeneshDP_pg.C_scaffold_115000006 | scaffold_115 | 11066 | 11935 |
| Block 144 | sec61a | 3 | 1338636 | 1340376 | estExt_Genewise1.C_1150008 | scaffold_115 | 8954 | 10833 |
| Block 144 | DDB_G0278887 | 3 | 1342274 | 1342981 | e_gw1.115.46.1 | scaffold_115 | 3764 | 4580 |
| Block 144 | mrpl27 | 3 | 1343635 | 1344413 | e_gw1.115.14.1 | scaffold_115 | 2114 | 2523 |
| Block 126 | DDB_G0278891 | 3 | 1344963 | 1346111 | fgeneshDP_pg.C_scaffold_112000018 | scaffold_112 | 76249 | 77478 |
| Block 1933 | DDB_G0278895 | 3 | 1348299 | 1352374 | GID1.0048896 | scaffold_422 | 14591 | 18254 |
| Block 1933 | DDB_G0278897 | 3 | 1358187 | 1358840 | GID1.0048899 | scaffold_422 | 23650 | 24335 |
| Block 126 | DDB_G0278901 | 3 | 1361340 | 1366175 | e_gw1.112.8.1 | scaffold_112 | 69654 | 73908 |
| Block 126 | DDB_G0278955 | 3 | 1366879 | 1368192 | GID1.0042666 | scaffold_112 | 74833 | 76058 |
| Block 17 | DDB_G0278903 | 3 | 1368428 | 1369840 | e_gw1.10.61.1 | scaffold_10 | 59497 | 60762 |
| Block 17 | mobA | 3 | 1373685 | 1374560 | estExt_fgeneshDP_kg.C_100009 | scaffold_10 | 57431 | 58841 |
| Block 17 | DDB_G0278909 | 3 | 1376167 | 1382125 | GID1.0037991 | scaffold_10 | 42457 | 47855 |
| Block 17 | DDB_G0278911 | 3 | 1382986 | 1384020 | estExt_fgeneshDP_pg.C_100020 | scaffold_10 | 40907 | 41896 |
| Block 67 | DDB_G0278913 | 3 | 1384248 | 1385606 | GID1.0042413 | scaffold_104 | 61965 | 63362 |
| Block 67 | DDB_G0278957 | 3 | 1385676 | 1386714 | fgeneshDP_pg.C_scaffold_104000026 | scaffold_104 | 58028 | 58836 |
| Block 17 | repB | 3 | 1389108 | 1391685 | estExt_fgeneshDP_pg.C_100031 | scaffold_10 | 66509 | 69256 |
| Block 827 | adprt1A | 3 | 1394183 | 1397588 | e_gw1.206.26.1 | scaffold_206 | 40271 | 43525 |
| Block 827 | DDB_G0278923 | 3 | 1407772 | 1410150 | estExt_Genewise1Plus.C_2060019 | scaffold_206 | 22593 | 25171 |
| Block 439 | DDB_G0278961 | 3 | 1412506 | 1413599 | fgeneshDP_pg.C_scaffold_15000044 | scaffold_15 | 125411 | 126467 |
| Block 439 | DDB_G0278927 | 3 | 1414868 | 1416355 | estExt_fgeneshDP_pg.C_150043 | scaffold_15 | 122665 | 124007 |
| Block 2505 | DDB_G0280845 | 3 | 1421922 | 1424296 | fgeneshDP_pg.C_scaffold_6000001 | scaffold_6 | 399 | 3328 |
| Block 2505 | DDB_G0280831 | 3 | 1432985 | 1434625 | GID1.0037667 | scaffold_6 | 53258 | 54788 |
| Block 2505 | aoxA | 3 | 1437954 | 1439078 | estExt_Genewise1.C_60041 | scaffold_6 | 57083 | 58413 |
| Block 2505 | trappc4 | 3 | 1440057 | 1440625 | fgeneshDP_pg.C_scaffold_6000029 | scaffold_6 | 63966 | 64458 |
| Block 19 | rps6 | 3 | 1442353 | 1443385 | estExt_Genewise1.C_100057 | scaffold_10 | 64566 | 65724 |
| Block 345 | dimA | 3 | 1459749 | 1463597 | GID1.0043511 | scaffold_139 | 8703 | 11623 |
| Block 345 | DDB_G0278979 | 3 | 1466872 | 1468184 | e_gw1.139.37.1 | scaffold_139 | 14827 | 16212 |
| Block 345 | DDB_G0278981 | 3 | 1470681 | 1472622 | estExt_fgeneshDP_kg.C_1390002 | scaffold_139 | 19275 | 21513 |
| Block 446 | DDB_G0278985 | 3 | 1475884 | 1476812 | e_gw1.150.41.1 | scaffold_150 | 7494 | 8221 |
| Block 446 | DDB_G0278987 | 3 | 1477419 | 1478405 | fgeneshDP_pm.C_scaffold_150000003 | scaffold_150 | 6018 | 6890 |
| Block 446 | DDB_G0278989 | 3 | 1479334 | 1479798 | e_gw1.150.48.1 | scaffold_150 | 4969 | 5433 |
| Block 2642 | culE | 3 | 1485914 | 1488923 | estExt_Genewise1.C_650072 | scaffold_65 | 96910 | 100204 |
| Block 2642 | DDB_G0278993 | 3 | 1489337 | 1490448 | GID1.0040930 | scaffold_65 | 100834 | 101255 |
| Block 1862 | DDB_G0278995 | 3 | 1494607 | 1498054 | GID1.0048701 | scaffold_406 | 25922 | 28384 |
| Block 1862 | gmkA | 3 | 1502267 | 1503059 | e_gw1.406.10.1 | scaffold_406 | 24592 | 25411 |
| Block 1862 | DDB_G0279003 | 3 | 1503809 | 1506692 | GID1.0048699 | scaffold_406 | 20950 | 23621 |
| Block 1014 | DDB_G0279005 | 3 | 1507522 | 1509663 | fgeneshDP_pg.C_scaffold_234000006 | scaffold_234 | 21762 | 23531 |
| Block 1014 | gacP | 3 | 1510645 | 1512700 | GID1.0045856 | scaffold_234 | 24227 | 26137 |
| Block 1014 | DDB_G0279011 | 3 | 1513004 | 1513501 | estExt_Genewise1.C_2340018 | scaffold_234 | 26417 | 26978 |
| Block 1014 | nola1 | 3 | 1514463 | 1515327 | gw1.234.29.1 | scaffold_234 | 28031 | 28300 |
| Block 1862 | DDB_G0279015 | 3 | 1515905 | 1517172 | e_gw1.406.5.1 | scaffold_406 | 19414 | 20661 |
| Block 692 | DDB_G0279019 | 3 | 1519155 | 1520138 | e_gw1.19.52.1 | scaffold_19 | 75876 | 76834 |
| Block 692 | DDB_G0279067 | 3 | 1520762 | 1522195 | e_gw1.19.32.1 | scaffold_19 | 73894 | 75126 |
| Block 692 | DDB_G0279021 | 3 | 1522629 | 1525694 | fgeneshDP_pg.C_scaffold_19000023 | scaffold_19 | 68613 | 73590 |
| Block 1014 | DDB_G0279033 | 3 | 1534984 | 1537005 | GID1.0045859 | scaffold_234 | 28765 | 30684 |
| Block 2172 | DDB_G0279035 | 3 | 1538571 | 1539599 | estExt_fgeneshDP_kg.C_4850001 | scaffold_485 | 2304 | 3641 |
| Block 2172 | coq3 | 3 | 1539737 | 1540795 | gw1.485.14.1 | scaffold_485 | 1310 | 2201 |
| Block 2172 | DDB_G0279039 | 3 | 1541078 | 1544303 | GID1.0049506 | scaffold_485 | 67 | 1001 |
| Block 1175 | DDB_G0279045 | 3 | 1551325 | 1553266 | estExt_fgeneshDP_pg.C_2610015 | scaffold_261 | 34705 | 36910 |
| Block 1175 | DDB_G0279047 | 3 | 1553555 | 1554260 | gw1.261.31.1 | scaffold_261 | 34124 | 34574 |
| Block 1175 | DDB_G0279049 | 3 | 1554715 | 1557635 | GID1.0046411 | scaffold_261 | 30782 | 33784 |
| Block 1175 | DDB_G0279053 | 3 | 1563766 | 1566821 | estExt_Genewise1.C_2610017 | scaffold_261 | 24223 | 26949 |
| Block 1949 | rpl31 | 3 | 1578225 | 1578923 | estExt_fgeneshDP_pm.C_4280003 | scaffold_428 | 15040 | 15642 |
| Block 1949 | utp20 | 3 | 1583286 | 1592966 | fgeneshDP_pg.C_scaffold_428000001 | scaffold_428 | 2162 | 11321 |
| Block 3038 | DDB_G0279089 | 3 | 1601965 | 1606352 | e_gw1.96.16.1 | scaffold_96 | 36068 | 40100 |
| Block 3038 | gxcCC | 3 | 1613622 | 1617399 | e_gw1.96.18.1 | scaffold_96 | 46384 | 49604 |
| Block 3038 | DDB_G0279093 | 3 | 1618503 | 1620851 | estExt_Genewise1Plus.C_960031 | scaffold_96 | 52459 | 53293 |
| Block 68 | abpB | 3 | 1621394 | 1622514 | estExt_fgeneshDP_pm.C_1040016 | scaffold_104 | 59038 | 60257 |
| Block 695 | DDB_G0279099 | 3 | 1626037 | 1632840 | estExt_Genewise1Plus.C_190151 | scaffold_19 | 128919 | 135047 |
| Block 695 | DDB_G0279105 | 3 | 1635440 | 1638700 | GID1.0038595 | scaffold_19 | 124009 | 127167 |
| Block 2509 | eIF3s8 | 3 | 1642949 | 1645898 | estExt_Genewise1Plus.C_60031 | scaffold_6 | 41210 | 44547 |
| Block 2509 | DDB_G0279111 | 3 | 1646736 | 1648046 | gw1.6.95.1 | scaffold_6 | 45237 | 46351 |
| Block 2509 | metS | 3 | 1648404 | 1650743 | estExt_fgeneshDP_pm.C_60008 | scaffold_6 | 46730 | 49119 |
| Block 2509 | DDB_G0279115 | 3 | 1651481 | 1654895 | estExt_Genewise1Plus.C_60036 | scaffold_6 | 49976 | 53073 |
| Block 462 | syn7B | 3 | 1672697 | 1673557 | GID1.0043925 | scaffold_153 | 48180 | 49082 |
| Block 351 | DDB_G0279137 | 3 | 1675769 | 1677652 | e_gw1.139.30.1 | scaffold_139 | 51766 | 53586 |
| Block 351 | DDB_G0279139 | 3 | 1678974 | 1680116 | fgeneshDP_pg.C_scaffold_139000023 | scaffold_139 | 54598 | 55859 |
| Block 351 | ap1b1 | 3 | 1680228 | 1683328 | estExt_Genewise1.C_1390045 | scaffold_139 | 63276 | 66519 |
| Block 1516 | DDB_G0279145 | 3 | 1686583 | 1688169 | fgeneshDP_pg.C_scaffold_329000003 | scaffold_329 | 4870 | 6330 |
| Block 1516 | pip5k3 | 3 | 1689106 | 1697393 | estExt_Genewise1Plus.C_3290004 | scaffold_329 | 8398 | 14233 |
| Block 2028 | cenB | 3 | 1697579 | 1698154 | GID1.0049158 | scaffold_449 | 17170 | 17820 |
| Block 2028 | DDB_G0279155 | 3 | 1698727 | 1700268 | GID1.0049157 | scaffold_449 | 15622 | 16931 |
| Block 462 | med16 | 3 | 1701022 | 1705842 | GID1.0043901 | scaffold_153 | 54 | 3487 |
| Block 438 | aco1 | 3 | 1708174 | 1711261 | estExt_Genewise1.C_150087 | scaffold_15 | 135729 | 138970 |
| Block 438 | DDB_G0279179 | 3 | 1715076 | 1717952 | fgeneshDP_pg.C_scaffold_15000041 | scaffold_15 | 116479 | 119325 |
| Block 462 | vps39 | 3 | 1722504 | 1725233 | GID1.0043903 | scaffold_153 | 6115 | 8989 |
| Block 462 | DDB_G0279173 | 3 | 1725633 | 1727571 | estExt_fgeneshDP_kg.C_1530001 | scaffold_153 | 9275 | 9910 |
| Block 462 | hspc300 | 3 | 1727720 | 1728000 | fgeneshDP_pm.C_scaffold_153000003 | scaffold_153 | 11330 | 11625 |
| Block 462 | DDB_G0279181 | 3 | 1728295 | 1730367 | fgeneshDP_pg.C_scaffold_153000006 | scaffold_153 | 11962 | 13817 |
| Block 462 | DDB_G0279183 | 3 | 1731085 | 1734690 | estExt_Genewise1.C_1530010 | scaffold_153 | 14413 | 19315 |
| Block 691 | DDB_G0279177 | 3 | 1734898 | 1736319 | GID1.0038567 | scaffold_19 | 57166 | 58574 |
| Block 691 | DDB_G0279205 | 3 | 1747299 | 1751183 | fgeneshDP_pg.C_scaffold_19000013 | scaffold_19 | 31797 | 35040 |
| Block 1978 | rpl21 | 3 | 1751733 | 1752215 | estExt_fgeneshDP_kg.C_4360002 | scaffold_436 | 4595 | 5921 |
| Block 1978 | rps19 | 3 | 1760514 | 1760960 | estExt_Genewise1.C_4360006 | scaffold_436 | 2997 | 3731 |
| Block 1978 | DDB_G0279209 | 3 | 1761272 | 1761904 | e_gw1.436.18.1 | scaffold_436 | 6247 | 6729 |
| Block 1978 | DDB_G0279211 | 3 | 1762684 | 1765261 | estExt_Genewise1.C_4360019 | scaffold_436 | 9981 | 12740 |
| Block 1978 | rpl11 | 3 | 1766422 | 1767386 | estExt_Genewise1Plus.C_4360021 | scaffold_436 | 14918 | 15865 |
| Block 1978 | DDB_G0279213 | 3 | 1768231 | 1771080 | fgeneshDP_pm.C_scaffold_436000005 | scaffold_436 | 16503 | 18457 |
| Block 2260 | DDB_G0279221 | 3 | 1781444 | 1781813 | GID1.0040294 | scaffold_51 | 84790 | 85026 |
| Block 2260 | rpb1 | 3 | 1783778 | 1789076 | GID1.0040293 | scaffold_51 | 77225 | 82575 |
| Block 2260 | DDB_G0279223 | 3 | 1790126 | 1790778 | fgeneshDP_pg.C_scaffold_51000034 | scaffold_51 | 92979 | 93493 |
| Block 2260 | DDB_G0279225 | 3 | 1791010 | 1793454 | estExt_fgeneshDP_pm.C_510013 | scaffold_51 | 87091 | 89349 |
| Block 2260 | DDB_G0279227 | 3 | 1794406 | 1795711 | e_gw1.51.53.1 | scaffold_51 | 85171 | 86200 |
| Block 23 | DDB_G0279247 | 3 | 1830468 | 1831196 | estExt_fgeneshDP_kg.C_100016 | scaffold_10 | 141089 | 142731 |
| Block 23 | DDB_G0279251 | 3 | 1833200 | 1837979 | estExt_fgeneshDP_pg.C_100053 | scaffold_10 | 137241 | 141048 |
| Block 982 | DDB_G0279253 | 3 | 1838283 | 1839914 | GID1.0038797 | scaffold_23 | 5181 | 6591 |
| Block 23 | DDB_G0279259 | 3 | 1848643 | 1851168 | gw1.10.6.1 | scaffold_10 | 129926 | 131926 |
| Block 982 | DDB_G0279395 | 3 | 1853231 | 1854643 | GID1.0038798 | scaffold_23 | 6660 | 7929 |
| Block 982 | DDB_G0279261 | 3 | 1855053 | 1856963 | e_gw1.23.33.1 | scaffold_23 | 8312 | 10360 |
| Block 690 | DDB_G0279263 | 3 | 1857138 | 1859575 | fgeneshDP_pm.C_scaffold_19000011 | scaffold_19 | 54746 | 56980 |
| Block 690 | DDB_G0279265 | 3 | 1859879 | 1860764 | estExt_Genewise1Plus.C_190038 | scaffold_19 | 46283 | 47341 |
| Block 690 | DDB_G0279267 | 3 | 1861211 | 1865976 | GID1.0038560 | scaffold_19 | 35187 | 38952 |
| Block 690 | DDB_G0279271 | 3 | 1870767 | 1872072 | GID1.0038558 | scaffold_19 | 29971 | 31138 |
| Block 1529 | DDB_G0279277 | 3 | 1876664 | 1877879 | GID1.0047640 | scaffold_331 | 20740 | 21853 |
| Block 1529 | DDB_G0279279 | 3 | 1878087 | 1879323 | fgeneshDP_pg.C_scaffold_331000008 | scaffold_331 | 19156 | 20415 |
| Block 430 | DDB_G0279397 | 3 | 1884785 | 1891355 | GID1.0038308 | scaffold_15 | 30714 | 36455 |
| Block 430 | DDB_G0279287 | 3 | 1892317 | 1893775 | fgeneshDP_pg.C_scaffold_15000011 | scaffold_15 | 37135 | 38566 |
| Block 430 | pmmA | 3 | 1893937 | 1895062 | estExt_fgeneshDP_pg.C_150022 | scaffold_15 | 73938 | 75076 |
| Block 430 | DDB_G0279291 | 3 | 1895354 | 1896877 | estExt_fgeneshDP_pg.C_150015 | scaffold_15 | 47063 | 49093 |
| Block 430 | CAX1 | 3 | 1901303 | 1903976 | estExt_Genewise1.C_150039 | scaffold_15 | 43440 | 46099 |
| Block 2903 | DDB_G0279309 | 3 | 1913378 | 1917128 | GID1.0041542 | scaffold_81 | 15077 | 18527 |
| Block 2903 | cdc5l | 3 | 1918418 | 1921048 | fgeneshDP_pg.C_scaffold_81000005 | scaffold_81 | 11554 | 13970 |
| Block 2903 | pigN | 3 | 1921668 | 1925073 | GID1.0041540 | scaffold_81 | 7540 | 10742 |
| Block 65 | DDB_G0279317 | 3 | 1928580 | 1930763 | fgeneshDP_pm.C_scaffold_104000015 | scaffold_104 | 49815 | 51803 |
| Block 65 | DDB_G0279321 | 3 | 1932547 | 1934192 | e_gw1.104.33.1 | scaffold_104 | 52289 | 53903 |
| Block 1477 | aurK | 3 | 1959552 | 1961164 | estExt_fgeneshDP_pg.C_320051 | scaffold_32 | 118760 | 120880 |
| Block 1477 | amfr | 3 | 1961529 | 1964457 | estExt_Genewise1Plus.C_320089 | scaffold_32 | 115814 | 118525 |
| Block 1477 | alg9 | 3 | 1972184 | 1974605 | estExt_fgeneshDP_pg.C_320046 | scaffold_32 | 109383 | 111868 |
| Block 1477 | DDB_G0279351 | 3 | 1974768 | 1975241 | GID1.0039367 | scaffold_32 | 108637 | 109158 |
| Block 1477 | DDB_G0279405 | 3 | 1975813 | 1977989 | GID1.0039366 | scaffold_32 | 105870 | 107989 |
| Block 1477 | DDB_G0279357 | 3 | 1983573 | 1984613 | fgeneshDP_pm.C_scaffold_32000022 | scaffold_32 | 104338 | 105740 |
| Block 1477 | ap1s1 | 3 | 1984933 | 1985585 | e_gw1.32.33.1 | scaffold_32 | 103650 | 104215 |
| Block 2482 | DDB_G0279361 | 3 | 1986517 | 1987341 | fgeneshDP_pg.C_scaffold_59000024 | scaffold_59 | 59241 | 60014 |
| Block 2482 | DDB_G0279363 | 3 | 1987447 | 1988114 | estExt_Genewise1Plus.C_590057 | scaffold_59 | 81162 | 81771 |
| Block 2630 | DDB_G0279367 | 3 | 1993054 | 1995220 | GID1.0040888 | scaffold_65 | 3920 | 5419 |
| Block 2630 | DDB_G0279369 | 3 | 1995959 | 1998148 | estExt_fgeneshDP_kg.C_650001 | scaffold_65 | 1212 | 2057 |
| Block 1952 | utp14 | 3 | 2000722 | 2004171 | GID1.0048956 | scaffold_429 | 747 | 3853 |
| Block 1952 | DDB_G0279373 | 3 | 2004876 | 2006425 | fgeneshDP_pg.C_scaffold_429000002 | scaffold_429 | 4472 | 5999 |
| Block 1952 | DDB_G0279375 | 3 | 2006606 | 2007698 | GID1.0048958 | scaffold_429 | 6218 | 7385 |
| Block 1477 | DDB_G0279381 | 3 | 2014573 | 2016449 | GID1.0039357 | scaffold_32 | 91739 | 93545 |
| Block 1477 | DDB_G0279383 | 3 | 2017692 | 2018908 | GID1.0039374 | scaffold_32 | 122634 | 123642 |
| Block 1477 | DDB_G0279421 | 3 | 2022765 | 2023750 | GID1.0039377 | scaffold_32 | 129836 | 130642 |
| Block 1173 | DDB_G0279427 | 3 | 2032053 | 2034159 | estExt_fgeneshDP_pg.C_2610006 | scaffold_261 | 13265 | 15760 |
| Block 1173 | DDB_G0279429 | 3 | 2037686 | 2039852 | estExt_fgeneshDP_pg.C_2610005 | scaffold_261 | 7144 | 9856 |
| Block 1173 | DDB_G0279431 | 3 | 2040372 | 2042087 | e_gw1.261.19.1 | scaffold_261 | 5444 | 6601 |
| Block 1173 | metap1 | 3 | 2043258 | 2045009 | estExt_Genewise1.C_2610003 | scaffold_261 | 3006 | 4354 |
| Block 358 | DDB_G0279435 | 3 | 2045716 | 2047346 | GID1.0038271 | scaffold_14 | 89622 | 91280 |
| Block 358 | DDB_G0279437 | 3 | 2047840 | 2049064 | GID1.0038270 | scaffold_14 | 87834 | 89446 |
| Block 358 | bzpC | 3 | 2050082 | 2052571 | estExt_fgeneshDP_pg.C_140032 | scaffold_14 | 84821 | 87483 |
| Block 464 | ctns | 3 | 2059799 | 2060751 | e_gw1.153.6.1 | scaffold_153 | 31334 | 32319 |
| Block 464 | hspJ | 3 | 2061353 | 2061838 | GID1.0043915 | scaffold_153 | 29261 | 30464 |
| Block 464 | DDB_G0279449 | 3 | 2062975 | 2066277 | fgeneshDP_pg.C_scaffold_153000011 | scaffold_153 | 24878 | 27745 |
| Block 464 | DDB_G0279451 | 3 | 2066556 | 2067929 | fgeneshDP_pg.C_scaffold_153000008 | scaffold_153 | 19529 | 20796 |
| Block 1771 | DDB_G0279453 | 3 | 2068536 | 2071742 | estExt_fgeneshDP_pg.C_390015 | scaffold_39 | 24458 | 27025 |
| Block 1771 | DDB_G0279455 | 3 | 2074339 | 2075435 | estExt_fgeneshDP_kg.C_390007 | scaffold_39 | 30019 | 31720 |
| Block 1686 | DDB_G0279461 | 3 | 2082922 | 2086347 | estExt_fgeneshDP_pm.C_3670001 | scaffold_367 | 6074 | 7759 |
| Block 1686 | DDB_G0279463 | 3 | 2087531 | 2089153 | GID1.0048161 | scaffold_367 | 8728 | 11372 |
| Block 1686 | DDB_G0279589 | 3 | 2089639 | 2090237 | GID1.0048162 | scaffold_367 | 11764 | 12323 |
| Block 1686 | roco6 | 3 | 2094570 | 2101013 | estExt_fgeneshDP_pm.C_3670004 | scaffold_367 | 13823 | 20695 |
| Block 208 | ctsD | 3 | 2104523 | 2105867 | estExt_Genewise1.C_1210028 | scaffold_121 | 60936 | 62730 |
| Block 208 | DDB_G0279473 | 3 | 2107184 | 2108727 | e_gw1.121.29.1 | scaffold_121 | 65011 | 66499 |
| Block 1916 | glnA3 | 3 | 2109355 | 2111562 | estExt_fgeneshDP_pm.C_420008 | scaffold_42 | 60139 | 62830 |
| Block 1916 | DDB_G0279475 | 3 | 2113626 | 2114348 | fgeneshDP_pg.C_scaffold_42000018 | scaffold_42 | 58359 | 58994 |
| Block 1916 | DDB_G0279477 | 3 | 2114897 | 2119637 | GID1.0039850 | scaffold_42 | 53581 | 57683 |
| Block 1916 | DDB_G0279479 | 3 | 2120059 | 2120364 | e_gw1.42.58.1 | scaffold_42 | 12415 | 12702 |
| Block 1916 | ccdc94 | 3 | 2120888 | 2121862 | gw1.42.11.1 | scaffold_42 | 11324 | 11992 |
| Block 1916 | pldB | 3 | 2122585 | 2126750 | GID1.0039830 | scaffold_42 | 6458 | 10232 |
| Block 1916 | DDB_G0279485 | 3 | 2127093 | 2131698 | fgeneshDP_pg.C_scaffold_42000020 | scaffold_42 | 63169 | 67555 |
| Block 1916 | DDB_G0279487 | 3 | 2132450 | 2140696 | GID1.0039834 | scaffold_42 | 14149 | 22186 |
| Block 1174 | DDB_G0279489 | 3 | 2144687 | 2147315 | fgeneshDP_pm.C_scaffold_261000006 | scaffold_261 | 19826 | 22038 |
| Block 1174 | DDB_G0279491 | 3 | 2147617 | 2149527 | e_gw1.261.30.1 | scaffold_261 | 16181 | 16774 |
| Block 25 | DDB_G0279501 | 3 | 2160865 | 2165924 | fgeneshDP_pg.C_scaffold_10000062 | scaffold_10 | 161271 | 166099 |
| Block 25 | DDB_G0279505 | 3 | 2172140 | 2172712 | estExt_fgeneshDP_pm.C_100026 | scaffold_10 | 166093 | 167412 |
| Block 1908 | plrg1 | 3 | 2174875 | 2176510 | fgeneshDP_pg.C_scaffold_416000001 | scaffold_416 | 1571 | 3158 |
| Block 1861 | DDB_G0279511 | 3 | 2181977 | 2185666 | estExt_Genewise1.C_4060003 | scaffold_406 | 8489 | 9726 |
| Block 1908 | DDB_G0279513 | 3 | 2186381 | 2188234 | GID1.0048814 | scaffold_416 | 3313 | 5172 |
| Block 347 | pigQ | 3 | 2188620 | 2192094 | GID1.0043515 | scaffold_139 | 21748 | 24443 |
| Block 347 | DDB_G0279517 | 3 | 2192375 | 2194027 | GID1.0043516 | scaffold_139 | 24970 | 26784 |
| Block 347 | mrpl22 | 3 | 2194197 | 2194910 | fgeneshDP_pg.C_scaffold_139000010 | scaffold_139 | 26921 | 27625 |
| Block 347 | limE | 3 | 2195759 | 2196668 | estExt_fgeneshDP_pm.C_1390008 | scaffold_139 | 28879 | 29907 |
| Block 854 | DDB_G0279523 | 3 | 2198274 | 2200498 | estExt_fgeneshDP_pg.C_210027 | scaffold_21 | 67922 | 70538 |
| Block 854 | DDB_G0279525 | 3 | 2201116 | 2201861 | estExt_fgeneshDP_kg.C_210008 | scaffold_21 | 70674 | 71518 |
| Block 1861 | DDB_G0279527 | 3 | 2202606 | 2206383 | GID1.0048693 | scaffold_406 | 4651 | 8097 |
| Block 1732 | bzpF | 3 | 2206682 | 2208577 | estExt_fgeneshDP_kg.C_3780001 | scaffold_378 | 733 | 1928 |
| Block 1861 | crlG | 3 | 2212555 | 2214319 | estExt_Genewise1Plus.C_4060001 | scaffold_406 | 203 | 2024 |
| Block 1861 | utp6 | 3 | 2214566 | 2216943 | GID1.0048692 | scaffold_406 | 2179 | 4414 |
| Block 1732 | DDB_G0279531 | 3 | 2217133 | 2218055 | fgeneshDP_pg.C_scaffold_378000002 | scaffold_378 | 2062 | 2790 |
| Block 1732 | DDB_G0279535 | 3 | 2220986 | 2221876 | fgeneshDP_pg.C_scaffold_378000003 | scaffold_378 | 2989 | 3950 |
| Block 1732 | pkaR | 3 | 2222668 | 2223895 | GID1.0048314 | scaffold_378 | 4187 | 5279 |
| Block 1861 | calA | 3 | 2244074 | 2245039 | estExt_fgeneshDP_kg.C_4060003 | scaffold_406 | 14968 | 16142 |
| Block 1861 | DDB_G0279555 | 3 | 2248374 | 2250101 | estExt_Genewise1.C_4060010 | scaffold_406 | 17602 | 19363 |
| Block 2814 | DDB_G0279561 | 3 | 2261454 | 2263171 | estExt_fgeneshDP_pg.C_750023 | scaffold_75 | 60771 | 62617 |
| Block 2814 | DDB_G0279563 | 3 | 2265052 | 2265670 | GID1.0041323 | scaffold_75 | 59108 | 60129 |
| Block 2814 | DDB_G0279565 | 3 | 2265879 | 2269163 | GID1.0041321 | scaffold_75 | 53894 | 56626 |
| Block 2814 | DDB_G0279567 | 3 | 2271017 | 2271847 | fgeneshDP_pg.C_scaffold_75000019 | scaffold_75 | 51577 | 52395 |
| Block 2814 | gflD | 3 | 2272081 | 2277554 | fgeneshDP_pg.C_scaffold_75000018 | scaffold_75 | 46708 | 51425 |
| Block 2057 | DDB_G0279587 | 3 | 2307458 | 2308309 | GID1.0049228 | scaffold_456 | 14662 | 15519 |
| Block 2057 | cbfA | 3 | 2308619 | 2312032 | GID1.0049229 | scaffold_456 | 15679 | 18823 |
| Block 2057 | DDB_G0279609 | 3 | 2313265 | 2314626 | fgeneshDP_pm.C_scaffold_456000005 | scaffold_456 | 13444 | 14396 |
| Block 2057 | DDB_G0279611 | 3 | 2316340 | 2317367 | e_gw1.456.8.1 | scaffold_456 | 9392 | 10458 |
| Block 2057 | DDB_G0279613 | 3 | 2317988 | 2319847 | GID1.0049226 | scaffold_456 | 6638 | 8481 |
| Block 2057 | DDB_G0279617 | 3 | 2321489 | 2322121 | estExt_fgeneshDP_pg.C_4560001 | scaffold_456 | 3324 | 4489 |
| Block 1874 | DDB_G0279627 | 3 | 2333694 | 2334224 | GID1.0039796 | scaffold_41 | 42017 | 42478 |
| Block 2861 | lkb1 | 3 | 2335074 | 2337125 | estExt_Genewise1.C_790016 | scaffold_79 | 24006 | 26452 |
| Block 2861 | psmD7 | 3 | 2343459 | 2344670 | GID1.0041468 | scaffold_79 | 27829 | 28969 |
| Block 2861 | DDB_G0279635 | 3 | 2345293 | 2346834 | e_gw1.79.30.1 | scaffold_79 | 29646 | 30992 |
| Block 2861 | DDB_G0279637 | 3 | 2347354 | 2348655 | estExt_fgeneshDP_pm.C_790007 | scaffold_79 | 36012 | 37343 |
| Block 1874 | DDB_G0279639 | 3 | 2350407 | 2353794 | fgeneshDP_pm.C_scaffold_41000001 | scaffold_41 | 3338 | 6618 |
| Block 1133 | DDB_G0279643 | 3 | 2356584 | 2358923 | estExt_Genewise1Plus.C_2540019 | scaffold_254 | 30374 | 32764 |
| Block 1133 | DDB_G0279645 | 3 | 2359062 | 2360328 | fgeneshDP_pm.C_scaffold_254000006 | scaffold_254 | 29219 | 30307 |
| Block 1133 | DDB_G0279647 | 3 | 2361407 | 2362691 | GID1.0046248 | scaffold_254 | 26645 | 27839 |
| Block 1252 | DDB_G0279649 | 3 | 2363863 | 2368238 | GID1.0046751 | scaffold_279 | 33466 | 37457 |
| Block 2792 | DDB_G0279657 | 3 | 2378488 | 2383621 | estExt_fgeneshDP_pm.C_740007 | scaffold_74 | 34576 | 39234 |
| Block 2792 | med17 | 3 | 2384965 | 2387825 | estExt_fgeneshDP_pg.C_740014 | scaffold_74 | 29849 | 32496 |
| Block 2792 | forA | 3 | 2388583 | 2393474 | GID1.0041270 | scaffold_74 | 24751 | 29304 |
| Block 1133 | snd1 | 3 | 2397433 | 2400394 | estExt_Genewise1.C_2540022 | scaffold_254 | 36769 | 39991 |
| Block 1133 | H2AX | 3 | 2410613 | 2411077 | gw1.254.9.1 | scaffold_254 | 46534 | 46875 |
| Block 2855 | DDB_G0279675 | 3 | 2426331 | 2428066 | GID1.0041454 | scaffold_78 | 77566 | 80496 |
| Block 2855 | DDB_G0279731 | 3 | 2431375 | 2432326 | fgeneshDP_pg.C_scaffold_78000039 | scaffold_78 | 81028 | 81929 |
| Block 2855 | gxcDD | 3 | 2433796 | 2439149 | estExt_Genewise1.C_780055 | scaffold_78 | 83677 | 88120 |
| Block 2520 | DDB_G0279683 | 3 | 2441695 | 2442513 | e_gw1.60.60.1 | scaffold_60 | 94143 | 94829 |
| Block 1769 | DDB_G0279687 | 3 | 2445627 | 2445896 | fgeneshDP_pg.C_scaffold_39000005 | scaffold_39 | 7238 | 7546 |
| Block 1769 | eftud1 | 3 | 2446020 | 2449616 | GID1.0039704 | scaffold_39 | 31903 | 35332 |
| Block 2520 | DDB_G0279693 | 3 | 2453262 | 2456643 | GID1.0040703 | scaffold_60 | 90309 | 93586 |
| Block 2520 | DDB_G0279695 | 3 | 2457402 | 2457891 | e_gw1.60.72.1 | scaffold_60 | 89308 | 89705 |
| Block 2520 | DDB_G0279697 | 3 | 2458047 | 2458484 | fgeneshDP_pm.C_scaffold_60000022 | scaffold_60 | 88578 | 88935 |
| Block 2520 | DDB_G0279699 | 3 | 2459375 | 2461348 | fgeneshDP_pg.C_scaffold_60000033 | scaffold_60 | 85230 | 86907 |
| Block 2520 | DDB_G0279701 | 3 | 2464297 | 2465421 | estExt_fgeneshDP_pg.C_600031 | scaffold_60 | 81781 | 82809 |
| Block 2520 | prpf4B | 3 | 2466144 | 2468855 | estExt_Genewise1Plus.C_600058 | scaffold_60 | 78441 | 79914 |
| Block 1656 | DDB_G0279709 | 3 | 2471727 | 2472482 | fgeneshDP_pg.C_scaffold_36000015 | scaffold_36 | 36747 | 37571 |
| Block 1656 | cmr | 3 | 2473335 | 2473844 | fgeneshDP_pg.C_scaffold_36000014 | scaffold_36 | 35382 | 35860 |
| Block 2520 | DDB_G0279713 | 3 | 2474053 | 2474909 | estExt_fgeneshDP_pg.C_600029 | scaffold_60 | 77325 | 78415 |
| Block 2520 | mtyrS | 3 | 2475392 | 2476840 | e_gw1.60.38.1 | scaffold_60 | 69025 | 70422 |
| Block 2520 | DDB_G0279717 | 3 | 2477453 | 2479054 | GID1.0040691 | scaffold_60 | 67004 | 68673 |
| Block 1604 | DDB_G0279747 | 3 | 2498789 | 2499747 | GID1.0047875 | scaffold_347 | 31366 | 32309 |
| Block 1604 | DDB_G0279749 | 3 | 2499904 | 2500343 | GID1.0047874 | scaffold_347 | 30711 | 31135 |
| Block 1604 | DDB_G0279751 | 3 | 2501638 | 2505374 | estExt_Genewise1.C_3470018 | scaffold_347 | 17968 | 29498 |
| Block 1604 | DDB_G0279753 | 3 | 2506085 | 2509939 | estExt_Genewise1Plus.C_3470017 | scaffold_347 | 13915 | 17973 |
| Block 1604 | DDB_G0279755 | 3 | 2510417 | 2512172 | GID1.0047869 | scaffold_347 | 10249 | 11951 |
| Block 1311 | top2mt | 3 | 2512313 | 2516167 | e_gw1.29.18.1 | scaffold_29 | 79860 | 83773 |
| Block 1311 | DDB_G0279791 | 3 | 2518903 | 2520445 | GID1.0039178 | scaffold_29 | 76689 | 78266 |
| Block 1311 | lap | 3 | 2524840 | 2526402 | estExt_fgeneshDP_kg.C_290007 | scaffold_29 | 74541 | 76186 |
| Block 2790 | DDB_G0279761 | 3 | 2526528 | 2527832 | GID1.0041274 | scaffold_74 | 41126 | 42344 |
| Block 2790 | DDB_G0279765 | 3 | 2530587 | 2533361 | GID1.0041276 | scaffold_74 | 46577 | 51641 |
| Block 2790 | DDB_G0279769 | 3 | 2535702 | 2536946 | e_gw1.74.43.1 | scaffold_74 | 17580 | 18583 |
| Block 2790 | DDB_G0279785 | 3 | 2547719 | 2548238 | estExt_Genewise1Plus.C_740022 | scaffold_74 | 57996 | 58716 |
| Block 2790 | pufA | 3 | 2548824 | 2551181 | e_gw1.74.9.1 | scaffold_74 | 59200 | 60198 |
| Block 2790 | mrps10 | 3 | 2553750 | 2554145 | fgeneshDP_pm.C_scaffold_74000010 | scaffold_74 | 65211 | 65606 |
| Block 2790 | pex7 | 3 | 2554628 | 2556009 | e_gw1.74.10.1 | scaffold_74 | 66308 | 67483 |
| Block 2790 | DDB_G0279803 | 3 | 2556336 | 2558174 | GID1.0041283 | scaffold_74 | 67653 | 69333 |
| Block 2790 | DDB_G0279805 | 3 | 2558407 | 2559776 | fgeneshDP_pg.C_scaffold_74000029 | scaffold_74 | 69669 | 71009 |
| Block 2790 | rsc22 | 3 | 2560881 | 2563234 | estExt_Genewise1.C_740033 | scaffold_74 | 72165 | 74749 |
| Block 2790 | DDB_G0279807 | 3 | 2563367 | 2565484 | estExt_fgeneshDP_pg.C_740023 | scaffold_74 | 55643 | 57937 |
| Block 1858 | cprB | 3 | 2569081 | 2570624 | estExt_fgeneshDP_pm.C_4040003 | scaffold_404 | 6691 | 8291 |
| Block 1858 | DDB_G0279809 | 3 | 2572023 | 2575055 | estExt_Genewise1.C_4040007 | scaffold_404 | 9738 | 12764 |
| Block 1858 | DDB_G0279813 | 3 | 2578911 | 2581892 | estExt_fgeneshDP_pm.C_4040005 | scaffold_404 | 14104 | 16634 |
| Block 1858 | DDB_G0279821 | 3 | 2586560 | 2588427 | e_gw1.404.12.1 | scaffold_404 | 4734 | 6466 |
| Block 413 | DDB_G0279825 | 3 | 2593261 | 2597278 | GID1.0043759 | scaffold_147 | 56356 | 59934 |
| Block 413 | ivdA | 3 | 2597532 | 2598965 | GID1.0043760 | scaffold_147 | 60082 | 61389 |
| Block 413 | DDB_G0279829 | 3 | 2599428 | 2600334 | estExt_fgeneshDP_kg.C_1470005 | scaffold_147 | 61844 | 62684 |
| Block 361 | wdr12 | 3 | 2678219 | 2679731 | e_gw1.14.13.1 | scaffold_14 | 121621 | 123020 |
| Block 361 | DDB_G0279905 | 3 | 2680036 | 2680911 | e_gw1.14.79.1 | scaffold_14 | 123308 | 124108 |
| Block 361 | DDB_G0279907 | 3 | 2681663 | 2682631 | GID1.0038289 | scaffold_14 | 128615 | 129808 |
| Block 1295 | Dd5P1 | 3 | 2713196 | 2716121 | GID1.0046876 | scaffold_286 | 22947 | 25343 |
| Block 1295 | DDB_G0279939 | 3 | 2716529 | 2716884 | GID1.0046875 | scaffold_286 | 22091 | 22570 |
| Block 92 | dhkH | 3 | 2725463 | 2730232 | GID1.0042546 | scaffold_108 | 60028 | 63901 |
| Block 92 | lipA | 3 | 2734597 | 2736770 | e_gw1.108.23.1 | scaffold_108 | 53244 | 55329 |
| Block 92 | DDB_G0279945 | 3 | 2737032 | 2739323 | GID1.0042548 | scaffold_108 | 70892 | 74204 |
| Block 1815 | mlkA | 3 | 2752719 | 2754560 | GID1.0048582 | scaffold_398 | 424 | 2020 |
| Block 1295 | DDB_G0279959 | 3 | 2758242 | 2759567 | fgeneshDP_kg.C_scaffold_286000001 | scaffold_286 | 40988 | 42714 |
| Block 1295 | abcB4 | 3 | 2759596 | 2762072 | e_gw1.286.4.1 | scaffold_286 | 28841 | 30621 |
| Block 1295 | drpp40 | 3 | 2762286 | 2763560 | e_gw1.286.10.1 | scaffold_286 | 26544 | 27995 |
| Block 2829 | DDB_G0280015 | 3 | 2764702 | 2766927 | GID1.0050950 | scaffold_760 | 7 | 1212 |
| Block 2829 | DDB_G0279963 | 3 | 2767341 | 2767750 | GID1.0050951 | scaffold_760 | 1538 | 2081 |
| Block 1815 | DDB_G0279965 | 3 | 2775437 | 2776753 | e_gw1.398.6.1 | scaffold_398 | 23406 | 24704 |
| Block 1815 | DDB_G0279967 | 3 | 2780826 | 2781737 | GID1.0048590 | scaffold_398 | 21989 | 23117 |
| Block 1815 | DDB_G0279969 | 3 | 2781830 | 2784109 | e_gw1.398.3.1 | scaffold_398 | 19664 | 21883 |
| Block 1815 | apnA | 3 | 2784977 | 2786706 | e_gw1.398.18.1 | scaffold_398 | 17728 | 18636 |
| Block 991 | DDB_G0280017 | 3 | 2788130 | 2795336 | GID1.0045774 | scaffold_230 | 244 | 4441 |
| Block 93 | DDB_G0279979 | 3 | 2806294 | 2808540 | fgeneshDP_pm.C_scaffold_108000009 | scaffold_108 | 74437 | 76446 |
| Block 93 | DDB_G0279981 | 3 | 2809171 | 2810082 | estExt_fgeneshDP_kg.C_1080006 | scaffold_108 | 76634 | 77766 |
| Block 991 | DDB_G0280025 | 3 | 2821744 | 2823688 | estExt_fgeneshDP_pg.C_2300017 | scaffold_230 | 46163 | 48406 |
| Block 991 | exoc8 | 3 | 2824517 | 2827436 | e_gw1.230.16.1 | scaffold_230 | 33195 | 35951 |
| Block 2849 | DDB_G0279995 | 3 | 2833406 | 2836050 | e_gw1.78.20.1 | scaffold_78 | 47496 | 49027 |
| Block 2849 | rpl18 | 3 | 2837555 | 2838534 | estExt_fgeneshDP_kg.C_780007 | scaffold_78 | 39970 | 41593 |
| Block 2463 | DDB_G0280067 | 3 | 2886853 | 2888403 | fgeneshDP_pm.C_scaffold_58000022 | scaffold_58 | 105112 | 106552 |
| Block 2463 | DDB_G0280069 | 3 | 2889026 | 2890227 | estExt_fgeneshDP_pg.C_580038 | scaffold_58 | 103669 | 104881 |
| Block 2463 | DDB_G0280071 | 3 | 2891601 | 2894057 | estExt_fgeneshDP_pg.C_580037 | scaffold_58 | 99761 | 102268 |
| Block 2463 | DDB_G0280073 | 3 | 2894240 | 2895662 | gw1.58.51.1 | scaffold_58 | 98903 | 99535 |
| Block 1495 | DDB_G0280079 | 3 | 2899122 | 2902089 | GID1.0047522 | scaffold_324 | 12309 | 14655 |
| Block 1495 | exoc2 | 3 | 2902757 | 2906284 | estExt_Genewise1.C_3240013 | scaffold_324 | 15234 | 18682 |
| Block 2291 | mcfJ | 3 | 2907001 | 2908252 | fgeneshDP_pg.C_scaffold_52000026 | scaffold_52 | 64816 | 66162 |
| Block 2291 | DDB_G0280085 | 3 | 2908403 | 2909200 | estExt_fgeneshDP_kg.C_520011 | scaffold_52 | 66217 | 67290 |
| Block 2689 | DDB_G0280367 | 3 | 2917996 | 2919019 | fgeneshDP_pg.C_scaffold_673000004 | scaffold_673 | 6701 | 7575 |
| Block 2689 | DDB_G0280091 | 3 | 2919337 | 2920234 | e_gw1.673.3.1 | scaffold_673 | 5739 | 6346 |
| Block 422 | osbA | 3 | 2921032 | 2922532 | estExt_Genewise1Plus.C_1480041 | scaffold_148 | 58488 | 59886 |
| Block 422 | gacGG | 3 | 2923710 | 2927475 | estExt_fgeneshDP_pg.C_1480024 | scaffold_148 | 54138 | 57574 |
| Block 422 | DDB_G0280103 | 3 | 2937406 | 2937942 | estExt_fgeneshDP_kg.C_1480007 | scaffold_148 | 50493 | 51495 |
| Block 422 | DDB_G0280105 | 3 | 2938222 | 2940089 | GID1.0043788 | scaffold_148 | 48152 | 49930 |
| Block 1752 | med11 | 3 | 2948262 | 2948693 | fgeneshDP_pg.C_scaffold_384000007 | scaffold_384 | 22069 | 22527 |
| Block 1752 | DDB_G0280111 | 3 | 2948944 | 2952761 | estExt_fgeneshDP_pm.C_3840001 | scaffold_384 | 18356 | 21890 |
| Block 1752 | sybA | 3 | 2954586 | 2955002 | e_gw1.384.9.1 | scaffold_384 | 15895 | 16167 |
| Block 1752 | anapc5 | 3 | 2955422 | 2958552 | fgeneshDP_pg.C_scaffold_384000005 | scaffold_384 | 12702 | 15532 |
| Block 1883 | yelA | 3 | 2965418 | 2968512 | estExt_fgeneshDP_pg.C_410031 | scaffold_41 | 90380 | 93658 |
| Block 1883 | DDB_G0280119 | 3 | 2971976 | 2976291 | GID1.0039813 | scaffold_41 | 84608 | 87818 |
| Block 2462 | DDB_G0280121 | 3 | 2976788 | 2977787 | gw1.58.10.1 | scaffold_58 | 97382 | 97983 |
| Block 2462 | DDB_G0280123 | 3 | 2978017 | 2978724 | e_gw1.58.40.1 | scaffold_58 | 96496 | 97188 |
| Block 2462 | DDB_G0280125 | 3 | 2979381 | 2983277 | fgeneshDP_pg.C_scaffold_58000032 | scaffold_58 | 84873 | 89213 |
| Block 54 | map1d | 3 | 2983397 | 2984706 | e_gw1.103.8.1 | scaffold_103 | 44881 | 46105 |
| Block 54 | DDB_G0280129 | 3 | 2984895 | 2985253 | e_gw1.103.57.1 | scaffold_103 | 44229 | 44641 |
| Block 54 | DDB_G0280131 | 3 | 2985578 | 2989509 | fgeneshDP_pg.C_scaffold_103000021 | scaffold_103 | 40325 | 44025 |
| Block 54 | DDB_G0280133 | 3 | 2991402 | 2996437 | GID1.0042365 | scaffold_103 | 35988 | 38943 |
| Block 54 | DDB_G0280135 | 3 | 2996500 | 2997587 | fgeneshDP_pg.C_scaffold_103000018 | scaffold_103 | 32783 | 34586 |
| Block 1752 | DDB_G0280141 | 3 | 3002773 | 3004711 | GID1.0048410 | scaffold_384 | 27116 | 28884 |
| Block 385 | DDB_G0280143 | 3 | 3004846 | 3007140 | estExt_fgeneshDP_pg.C_1430016 | scaffold_143 | 34892 | 37127 |
| Block 385 | DDB_G0280371 | 3 | 3007522 | 3009141 | e_gw1.143.6.1 | scaffold_143 | 40759 | 42488 |
| Block 2223 | DDB_G0280373 | 3 | 3009655 | 3010347 | GID1.0040223 | scaffold_50 | 24121 | 24636 |
| Block 2223 | pyr56 | 3 | 3010631 | 3012067 | e_gw1.50.52.1 | scaffold_50 | 24885 | 26394 |
| Block 2223 | DDB_G0280145 | 3 | 3012864 | 3015761 | estExt_Genewise1Plus.C_500021 | scaffold_50 | 27064 | 29905 |
| Block 1783 | ddx47 | 3 | 3015956 | 3017596 | gw1.39.14.1 | scaffold_39 | 108730 | 110031 |
| Block 323 | DDB_G0280163 | 3 | 3036716 | 3038045 | GID1.0043414 | scaffold_136 | 18808 | 19862 |
| Block 323 | DDB_G0280165 | 3 | 3038985 | 3041507 | GID1.0043412 | scaffold_136 | 10430 | 12638 |
| Block 323 | psiP | 3 | 3046316 | 3049419 | estExt_Genewise1.C_1360005 | scaffold_136 | 7093 | 10214 |
| Block 1783 | dynD | 3 | 3050941 | 3052942 | e_gw1.39.66.1 | scaffold_39 | 119174 | 121018 |
| Block 1783 | DDB_G0280177 | 3 | 3054033 | 3057877 | estExt_fgeneshDP_pg.C_390051 | scaffold_39 | 114733 | 118005 |
| Block 1783 | DDB_G0280179 | 3 | 3058313 | 3059360 | fgeneshDP_pm.C_scaffold_39000030 | scaffold_39 | 113653 | 114667 |
| Block 109 | DDB_G0280375 | 3 | 3060332 | 3067092 | GID1.0038088 | scaffold_11 | 139846 | 145442 |
| Block 109 | DDB_G0280181 | 3 | 3067276 | 3068277 | estExt_Genewise1.C_110115 | scaffold_11 | 138624 | 139804 |
| Block 109 | sglB | 3 | 3069484 | 3071171 | GID1.0038089 | scaffold_11 | 146043 | 147710 |
| Block 109 | DDB_G0280377 | 3 | 3071897 | 3074445 | estExt_Genewise1Plus.C_110121 | scaffold_11 | 147934 | 150659 |
| Block 2190 | DDB_G0280187 | 3 | 3078456 | 3080086 | estExt_Genewise1Plus.C_490037 | scaffold_49 | 39754 | 41136 |
| Block 2190 | DDB_G0280191 | 3 | 3082696 | 3084110 | fgeneshDP_pm.C_scaffold_49000008 | scaffold_49 | 46970 | 48314 |
| Block 2190 | DDB_G0280193 | 3 | 3084374 | 3086922 | GID1.0040192 | scaffold_49 | 48603 | 50278 |
| Block 2190 | DDB_G0280195 | 3 | 3087742 | 3093065 | GID1.0040193 | scaffold_49 | 51484 | 56028 |
| Block 2190 | sepsecs | 3 | 3095673 | 3097226 | GID1.0040194 | scaffold_49 | 58989 | 60541 |
| Block 2024 | DDB_G0280383 | 3 | 3119383 | 3120372 | fgeneshDP_pg.C_scaffold_448000001 | scaffold_448 | 1094 | 1848 |
| Block 2024 | DDB_G0280215 | 3 | 3121269 | 3122626 | estExt_Genewise1.C_4480002 | scaffold_448 | 2518 | 4053 |
| Block 342 | grp94 | 3 | 3124645 | 3127046 | estExt_fgeneshDP_pg.C_1380021 | scaffold_138 | 60373 | 62876 |
| Block 995 | vps55 | 3 | 3127670 | 3128628 | e_gw1.230.11.1 | scaffold_230 | 24502 | 25076 |
| Block 995 | DDB_G0280221 | 3 | 3129179 | 3135878 | GID1.0045786 | scaffold_230 | 25702 | 31520 |
| Block 342 | DDB_G0280225 | 3 | 3138804 | 3139214 | estExt_Genewise1Plus.C_1380035 | scaffold_138 | 58050 | 58531 |
| Block 342 | DDB_G0280227 | 3 | 3139657 | 3141535 | GID1.0043502 | scaffold_138 | 63000 | 64795 |
| Block 342 | rpl24 | 3 | 3141943 | 3142654 | estExt_fgeneshDP_pg.C_1380023 | scaffold_138 | 65482 | 66140 |
| Block 2753 | DDB_G0280235 | 3 | 3146638 | 3148177 | estExt_Genewise1Plus.C_700054 | scaffold_70 | 89046 | 90466 |
| Block 2753 | coq5 | 3 | 3148507 | 3149553 | estExt_Genewise1Plus.C_700055 | scaffold_70 | 90735 | 91863 |
| Block 1884 | DDB_G0280243 | 3 | 3154238 | 3155321 | fgeneshDP_pg.C_scaffold_41000035 | scaffold_41 | 99005 | 100015 |
| Block 1884 | DDB_G0280385 | 3 | 3156312 | 3157836 | GID1.0039817 | scaffold_41 | 96701 | 98265 |
| Block 1884 | DDB_G0280247 | 3 | 3159963 | 3161380 | e_gw1.41.46.1 | scaffold_41 | 115167 | 116434 |
| Block 1884 | DDB_G0280249 | 3 | 3162671 | 3165464 | fgeneshDP_pm.C_scaffold_41000019 | scaffold_41 | 117870 | 120513 |
| Block 2289 | DDB_G0280251 | 3 | 3165971 | 3166945 | e_gw1.52.16.1 | scaffold_52 | 50363 | 51370 |
| Block 2289 | DDB_G0280253 | 3 | 3167632 | 3171153 | GID1.0040329 | scaffold_52 | 46266 | 49742 |
| Block 2289 | mdhC | 3 | 3171359 | 3172577 | fgeneshDP_pg.C_scaffold_52000018 | scaffold_52 | 44924 | 46087 |
| Block 2289 | DDB_G0280257 | 3 | 3173383 | 3174731 | fgeneshDP_pg.C_scaffold_52000015 | scaffold_52 | 38429 | 39610 |
| Block 2289 | thyA | 3 | 3175236 | 3176238 | GID1.0040324 | scaffold_52 | 36759 | 37715 |
| Block 2289 | DDB_G0280259 | 3 | 3176428 | 3178260 | fgeneshDP_pm.C_scaffold_52000006 | scaffold_52 | 35012 | 36571 |
| Block 1884 | DDB_G0280261 | 3 | 3178567 | 3180486 | e_gw1.41.30.1 | scaffold_41 | 110062 | 111910 |
| Block 1884 | DDB_G0280263 | 3 | 3180987 | 3182048 | GID1.0039822 | scaffold_41 | 108413 | 109458 |
| Block 1884 | allC | 3 | 3183979 | 3185311 | estExt_fgeneshDP_kg.C_410007 | scaffold_41 | 105789 | 107371 |
| Block 1884 | DDB_G0280269 | 3 | 3190136 | 3192063 | GID1.0039815 | scaffold_41 | 93723 | 95404 |
| Block 1884 | DDB_G0280271 | 3 | 3192876 | 3193504 | e_gw1.41.57.1 | scaffold_41 | 95811 | 96465 |
| Block 1746 | DDB_G0280281 | 3 | 3200265 | 3202279 | estExt_fgeneshDP_pg.C_3800001 | scaffold_380 | 1995 | 3945 |
| Block 1746 | DDB_G0280285 | 3 | 3206520 | 3207210 | estExt_fgeneshDP_pm.C_3800003 | scaffold_380 | 12573 | 13500 |
| Block 1746 | DDB_G0280287 | 3 | 3207518 | 3208475 | e_gw1.380.19.1 | scaffold_380 | 11583 | 12456 |
| Block 1746 | DDB_G0280289 | 3 | 3208817 | 3211777 | e_gw1.380.6.1 | scaffold_380 | 8807 | 11206 |
| Block 1746 | DDB_G0280291 | 3 | 3212417 | 3214010 | GID1.0048341 | scaffold_380 | 6712 | 8154 |
| Block 1746 | coq1 | 3 | 3214568 | 3216167 | GID1.0048340 | scaffold_380 | 4428 | 6057 |
| Block 1746 | myoA | 3 | 3224159 | 3227470 | GID1.0048349 | scaffold_380 | 25755 | 28883 |
| Block 1746 | DDB_G0280307 | 3 | 3228229 | 3229981 | estExt_Genewise1.C_3800014 | scaffold_380 | 23552 | 25443 |
| Block 1411 | DDB_G0280311 | 3 | 3241213 | 3244547 | GID1.0047223 | scaffold_307 | 1758 | 4837 |
| Block 1411 | ssr2 | 3 | 3245211 | 3246276 | fgeneshDP_pg.C_scaffold_307000002 | scaffold_307 | 4992 | 5991 |
| Block 1411 | DDB_G0280315 | 3 | 3247016 | 3247990 | GID1.0047227 | scaffold_307 | 7540 | 8517 |
| Block 2288 | glud2 | 3 | 3249762 | 3253025 | estExt_fgeneshDP_pm.C_520004 | scaffold_52 | 26626 | 30166 |
| Block 2288 | DDB_G0280393 | 3 | 3254509 | 3256772 | fgeneshDP_pg.C_scaffold_52000011 | scaffold_52 | 32416 | 33228 |
| Block 58 | rab8A | 3 | 3266503 | 3267523 | estExt_fgeneshDP_kg.C_1030007 | scaffold_103 | 76431 | 78165 |
| Block 58 | DDB_G0280337 | 3 | 3277418 | 3277913 | estExt_Genewise1Plus.C_1030054 | scaffold_103 | 72303 | 72860 |
| Block 58 | rdiB | 3 | 3280488 | 3280946 | fgeneshDP_pg.C_scaffold_103000034 | scaffold_103 | 71086 | 71547 |
| Block 58 | DDB_G0280051 | 3 | 3281113 | 3282389 | estExt_fgeneshDP_pg.C_1030032 | scaffold_103 | 69543 | 71000 |
| Block 2820 | sgtA | 3 | 3286144 | 3287386 | estExt_Genewise1.C_7520004 | scaffold_752 | 2709 | 3987 |
| Block 2820 | DDB_G0280347 | 3 | 3287782 | 3288431 | estExt_Genewise1.C_7520001 | scaffold_752 | 1331 | 2108 |
| Block 2820 | DDB_G0280349 | 3 | 3288732 | 3289517 | fgeneshDP_pm.C_scaffold_752000001 | scaffold_752 | 585 | 1248 |
| Block 2918 | gpaL | 3 | 3291770 | 3293372 | GID1.0041613 | scaffold_82 | 86855 | 88603 |
| Block 2918 | odhA | 3 | 3293945 | 3296736 | GID1.0041612 | scaffold_82 | 83178 | 86059 |
| Block 2918 | DDB_G0280363 | 3 | 3303410 | 3305794 | e_gw1.82.14.1 | scaffold_82 | 80641 | 82995 |
| Block 2455 | DDB_G0280403 | 3 | 3309264 | 3310324 | fgeneshDP_pg.C_scaffold_58000007 | scaffold_58 | 15442 | 16344 |
| Block 2455 | DDB_G0280405 | 3 | 3310609 | 3311235 | e_gw1.58.52.1 | scaffold_58 | 16632 | 17296 |
| Block 455 | cypD | 3 | 3316821 | 3317345 | e_gw1.151.9.1 | scaffold_151 | 24346 | 25174 |
| Block 455 | DDB_G0280413 | 3 | 3319399 | 3322888 | GID1.0043857 | scaffold_151 | 19149 | 24032 |
| Block 455 | DDB_G0280415 | 3 | 3323965 | 3324460 | GID1.0043853 | scaffold_151 | 10934 | 11382 |
| Block 455 | DDB_G0280417 | 3 | 3324530 | 3325577 | GID1.0043852 | scaffold_151 | 9958 | 10698 |
| Block 455 | DDB_G0280419 | 3 | 3326559 | 3329126 | e_gw1.151.18.1 | scaffold_151 | 4300 | 6780 |
| Block 455 | DDB_G0280421 | 3 | 3329289 | 3330048 | fgeneshDP_pg.C_scaffold_151000003 | scaffold_151 | 8336 | 9195 |
| Block 175 | ap4e1 | 3 | 3335352 | 3338884 | estExt_Genewise1Plus.C_1190009 | scaffold_119 | 7650 | 13333 |
| Block 175 | rio1 | 3 | 3344181 | 3345988 | e_gw1.119.5.1 | scaffold_119 | 13717 | 15063 |
| Block 175 | dynA | 3 | 3347902 | 3352629 | estExt_fgeneshDP_pm.C_1190002 | scaffold_119 | 15689 | 20254 |
| Block 2909 | pelo | 3 | 3368362 | 3369914 | fgeneshDP_pm.C_scaffold_82000004 | scaffold_82 | 11203 | 12574 |
| Block 2909 | pitC | 3 | 3370546 | 3371762 | e_gw1.82.29.1 | scaffold_82 | 9624 | 10740 |
| Block 2909 | DDB_G0280451 | 3 | 3372581 | 3373171 | e_gw1.82.48.1 | scaffold_82 | 8150 | 8887 |
| Block 2909 | DDB_G0280453 | 3 | 3373444 | 3374418 | e_gw1.82.33.1 | scaffold_82 | 6981 | 7814 |
| Block 2909 | DDB_G0280457 | 3 | 3378782 | 3380059 | estExt_fgeneshDP_pg.C_820001 | scaffold_82 | 972 | 2548 |
| Block 1018 | DDB_G0280525 | 3 | 3381127 | 3387444 | e_gw1.235.15.1 | scaffold_235 | 1242 | 5869 |
| Block 1018 | DDB_G0280461 | 3 | 3391535 | 3392987 | estExt_Genewise1.C_2350013 | scaffold_235 | 19558 | 21656 |
| Block 1018 | DDB_G0280463 | 3 | 3394025 | 3396265 | fgeneshDP_pg.C_scaffold_235000005 | scaffold_235 | 17056 | 19251 |
| Block 1018 | DDB_G0280465 | 3 | 3396577 | 3397708 | fgeneshDP_pg.C_scaffold_235000004 | scaffold_235 | 15615 | 16675 |
| Block 1018 | psrA | 3 | 3399574 | 3402079 | estExt_fgeneshDP_pg.C_2350003 | scaffold_235 | 13418 | 15517 |
| Block 1018 | DDB_G0280471 | 3 | 3403854 | 3409622 | GID1.0045865 | scaffold_235 | 6034 | 11633 |
| Block 1395 | coq7 | 3 | 3414983 | 3415636 | estExt_fgeneshDP_kg.C_3030001 | scaffold_303 | 5301 | 6373 |
| Block 1395 | DDB_G0280527 | 3 | 3416378 | 3425292 | GID1.0047164 | scaffold_303 | 9250 | 17143 |
| Block 1090 | DDB_G0280481 | 3 | 3429343 | 3431710 | GID1.0046124 | scaffold_248 | 36232 | 38355 |
| Block 1090 | fsjA | 3 | 3432872 | 3433788 | GID1.0046125 | scaffold_248 | 39263 | 40127 |
| Block 1090 | jcdH | 3 | 3434014 | 3435699 | gw1.248.17.1 | scaffold_248 | 40432 | 42125 |
| Block 1090 | DDB_G0280487 | 3 | 3435776 | 3436690 | fgeneshDP_pg.C_scaffold_248000014 | scaffold_248 | 42341 | 43198 |
| Block 1090 | wdr46 | 3 | 3437042 | 3438999 | fgeneshDP_pg.C_scaffold_248000015 | scaffold_248 | 43485 | 45439 |
| Block 1930 | fumH | 3 | 3450744 | 3452416 | GID1.0048882 | scaffold_421 | 4652 | 6438 |
| Block 1930 | DDB_G0280505 | 3 | 3457915 | 3459485 | estExt_Genewise1.C_4210009 | scaffold_421 | 7703 | 9328 |
| Block 1930 | DDB_G0280507 | 3 | 3459592 | 3460950 | e_gw1.421.10.1 | scaffold_421 | 9499 | 10398 |
| Block 1930 | cstf2 | 3 | 3461453 | 3464060 | fgeneshDP_pg.C_scaffold_421000006 | scaffold_421 | 12731 | 13774 |
| Block 562 | DDB_G0280513 | 3 | 3471532 | 3474399 | estExt_fgeneshDP_pg.C_1690009 | scaffold_169 | 33419 | 36612 |
| Block 2290 | DDB_G0280549 | 3 | 3481319 | 3482516 | estExt_fgeneshDP_kg.C_520009 | scaffold_52 | 61265 | 62660 |
| Block 2290 | DDB_G0280551 | 3 | 3483317 | 3484567 | GID1.0040336 | scaffold_52 | 63273 | 64326 |
| Block 562 | ctps | 3 | 3510967 | 3512917 | GID1.0044358 | scaffold_169 | 31370 | 33304 |
| Block 562 | uqcrq | 3 | 3513680 | 3514010 | fgeneshDP_pm.C_scaffold_169000010 | scaffold_169 | 59337 | 59663 |
| Block 562 | fut8 | 3 | 3514430 | 3516337 | fgeneshDP_pg.C_scaffold_169000019 | scaffold_169 | 56324 | 59022 |
| Block 562 | DDB_G0280575 | 3 | 3519504 | 3522563 | estExt_Genewise1.C_1690020 | scaffold_169 | 51755 | 55768 |
| Block 562 | DDB_G0280579 | 3 | 3523763 | 3524566 | fgeneshDP_pm.C_scaffold_169000008 | scaffold_169 | 50514 | 51254 |
| Block 562 | DDB_G0280581 | 3 | 3524824 | 3526893 | fgeneshDP_pg.C_scaffold_169000015 | scaffold_169 | 48414 | 50063 |
| Block 562 | DDB_G0280593 | 3 | 3539163 | 3543408 | GID1.0044363 | scaffold_169 | 43593 | 46336 |
| Block 562 | sdhA | 3 | 3543819 | 3545699 | estExt_Genewise1.C_1690016 | scaffold_169 | 39732 | 42075 |
| Block 2566 | DDB_G0280705 | 3 | 3648861 | 3656137 | GID1.0040765 | scaffold_62 | 15450 | 22226 |
| Block 2566 | DDB_G0280747 | 3 | 3657513 | 3658445 | fgeneshDP_pg.C_scaffold_62000005 | scaffold_62 | 13372 | 14256 |
| Block 1592 | DDB_G0280765 | 3 | 3715566 | 3718006 | estExt_fgeneshDP_pg.C_3440016 | scaffold_344 | 32272 | 34740 |
| Block 1592 | DDB_G0280767 | 3 | 3718300 | 3719568 | GID1.0047832 | scaffold_344 | 26247 | 28762 |
| Block 1513 | DDB_G0280769 | 3 | 3719733 | 3720516 | e_gw1.328.10.1 | scaffold_328 | 34991 | 35694 |
| Block 2453 | DDB_G0280773 | 3 | 3724009 | 3726993 | estExt_Genewise1.C_580001 | scaffold_58 | 1 | 2778 |
| Block 2453 | rvb2 | 3 | 3727138 | 3728652 | fgeneshDP_pg.C_scaffold_58000002 | scaffold_58 | 2910 | 4354 |
| Block 1513 | DDB_G0280777 | 3 | 3729649 | 3735545 | GID1.0047596 | scaffold_328 | 29293 | 33352 |
| Block 2453 | DDB_G0280783 | 3 | 3739979 | 3740353 | gw1.58.56.1 | scaffold_58 | 4855 | 5184 |
| Block 2453 | DDB_G0280793 | 3 | 3756319 | 3757251 | fgeneshDP_pm.C_scaffold_58000004 | scaffold_58 | 5342 | 6205 |
| Block 2453 | DDB_G0280797 | 3 | 3760317 | 3764888 | GID1.0040568 | scaffold_58 | 9108 | 13479 |
| Block 2453 | DDB_G0280799 | 3 | 3765385 | 3765613 | e_gw1.58.58.1 | scaffold_58 | 14853 | 15023 |
| Block 420 | DDB_G0280813 | 3 | 3771010 | 3774818 | GID1.0043782 | scaffold_148 | 36193 | 39748 |
| Block 420 | trrA | 3 | 3775563 | 3776632 | estExt_fgeneshDP_pm.C_1480011 | scaffold_148 | 40531 | 41936 |
| Block 420 | DDB_G0280803 | 3 | 3777864 | 3779318 | estExt_fgeneshDP_pg.C_1480019 | scaffold_148 | 43004 | 44493 |
| Block 420 | DDB_G0280805 | 3 | 3779782 | 3781110 | GID1.0043785 | scaffold_148 | 44829 | 45977 |
| Block 2266 | DDB_G0280875 | 3 | 3824495 | 3824971 | GID1.0049716 | scaffold_510 | 6086 | 6451 |
| Block 2266 | DDB_G0280877 | 3 | 3825256 | 3827473 | GID1.0049715 | scaffold_510 | 3512 | 5711 |
| Block 681 | arpD | 3 | 3833482 | 3835332 | estExt_Genewise1.C_1870044 | scaffold_187 | 56136 | 57904 |
| Block 681 | DDB_G0280889 | 3 | 3835530 | 3836519 | GID1.0044822 | scaffold_187 | 55165 | 56030 |
| Block 681 | pigV | 3 | 3836784 | 3838939 | estExt_fgeneshDP_pg.C_1870011 | scaffold_187 | 46904 | 48944 |
| Block 681 | rbsk | 3 | 3840063 | 3841393 | fgeneshDP_pm.C_scaffold_187000003 | scaffold_187 | 45454 | 46545 |
| Block 417 | dicA | 3 | 3856978 | 3859184 | fgeneshDP_pg.C_scaffold_148000013 | scaffold_148 | 30521 | 32681 |
| Block 417 | DDB_G0280903 | 3 | 3859774 | 3860817 | fgeneshDP_pg.C_scaffold_148000014 | scaffold_148 | 33136 | 34274 |
| Block 2461 | DDB_G0280905 | 3 | 3861062 | 3861547 | fgeneshDP_pm.C_scaffold_58000016 | scaffold_58 | 75166 | 75753 |
| Block 2461 | xpo4 | 3 | 3862138 | 3865629 | GID1.0040595 | scaffold_58 | 71155 | 74599 |
| Block 2461 | tal | 3 | 3865707 | 3866767 | estExt_Genewise1.C_580051 | scaffold_58 | 75864 | 77073 |
| Block 2461 | DDB_G0280945 | 3 | 3867097 | 3869258 | fgeneshDP_pg.C_scaffold_58000030 | scaffold_58 | 77230 | 79141 |
| Block 2461 | DDB_G0280911 | 3 | 3870282 | 3873485 | fgeneshDP_pg.C_scaffold_58000031 | scaffold_58 | 79506 | 83342 |
| Block 2680 | DDB_G0280919 | 3 | 3879930 | 3880291 | e_gw1.67.51.1 | scaffold_67 | 860 | 1166 |
| Block 2680 | DDB_G0280921 | 3 | 3881788 | 3885032 | fgeneshDP_pg.C_scaffold_67000001 | scaffold_67 | 2079 | 6325 |
| Block 2680 | DDB_G0280923 | 3 | 3886092 | 3886599 | GID1.0040971 | scaffold_67 | 6644 | 6996 |
| Block 2680 | DDB_G0280947 | 3 | 3886790 | 3888946 | e_gw1.67.31.1 | scaffold_67 | 7285 | 8460 |
| Block 246 | udpB | 3 | 3914149 | 3915176 | fgeneshDP_pg.C_scaffold_126000002 | scaffold_126 | 2310 | 3441 |
| Block 246 | DDB_G0280987 | 3 | 3916236 | 3917206 | fgeneshDP_pm.C_scaffold_126000009 | scaffold_126 | 13240 | 14149 |
| Block 711 | DDB_G0280991 | 3 | 3918547 | 3920423 | estExt_fgeneshDP_pg.C_1920020 | scaffold_192 | 51758 | 53568 |
| Block 711 | DDB_G0280993 | 3 | 3921693 | 3923045 | fgeneshDP_pg.C_scaffold_192000019 | scaffold_192 | 49698 | 51394 |
| Block 711 | mps1 | 3 | 3923506 | 3926616 | GID1.0044917 | scaffold_192 | 46996 | 49638 |
| Block 1974 | DDB_G0281025 | 3 | 3944292 | 3946934 | estExt_fgeneshDP_pg.C_4350003 | scaffold_435 | 6955 | 10444 |
| Block 2340 | DDB_G0281027 | 3 | 3947337 | 3947903 | GID1.0049904 | scaffold_534 | 14473 | 14987 |
| Block 1974 | pldA | 3 | 3951371 | 3955510 | estExt_fgeneshDP_pm.C_4350001 | scaffold_435 | 2237 | 5888 |
| Block 1974 | DDB_G0281033 | 3 | 3956014 | 3956307 | e_gw1.435.13.1 | scaffold_435 | 6346 | 6513 |
| Block 711 | DDB_G0281045 | 3 | 3967277 | 3973178 | GID1.0044920 | scaffold_192 | 53668 | 58406 |
| Block 1274 | gxcEE | 3 | 3973435 | 3975824 | fgeneshDP_pg.C_scaffold_281000012 | scaffold_281 | 30112 | 32162 |
| Block 1274 | DDB_G0281049 | 3 | 3977638 | 3978912 | fgeneshDP_pg.C_scaffold_281000011 | scaffold_281 | 27771 | 28601 |
| Block 2340 | psmD12 | 3 | 3979792 | 3981586 | fgeneshDP_pg.C_scaffold_534000005 | scaffold_534 | 15190 | 16261 |
| Block 1878 | DDB_G0281057 | 3 | 3984594 | 3986192 | fgeneshDP_pg.C_scaffold_41000013 | scaffold_41 | 39462 | 41089 |
| Block 1878 | rrpC | 3 | 3986737 | 3993698 | GID1.0039794 | scaffold_41 | 32162 | 39119 |
| Block 1601 | wdr4 | 3 | 3999282 | 4000753 | gw1.346.6.1 | scaffold_346 | 9250 | 10616 |
| Block 1601 | DDB_G0281065 | 3 | 4001320 | 4001787 | fgeneshDP_pg.C_scaffold_346000003 | scaffold_346 | 8017 | 8601 |
| Block 1601 | DDB_G0281067 | 3 | 4002363 | 4003396 | estExt_fgeneshDP_pm.C_3460003 | scaffold_346 | 18807 | 20020 |
| Block 1601 | DDB_G0281069 | 3 | 4003785 | 4006768 | e_gw1.346.4.1 | scaffold_346 | 24422 | 26185 |
| Block 1601 | serA | 3 | 4007757 | 4009455 | estExt_Genewise1Plus.C_3460008 | scaffold_346 | 20249 | 22972 |
| Block 415 | DDB_G0281081 | 3 | 4027472 | 4029494 | GID1.0043775 | scaffold_148 | 23962 | 25871 |
| Block 415 | prkab | 3 | 4037372 | 4038716 | GID1.0043771 | scaffold_148 | 14370 | 15553 |
| Block 415 | DDB_G0281091 | 3 | 4039994 | 4042224 | GID1.0043772 | scaffold_148 | 16797 | 18641 |
| Block 415 | gbqA | 3 | 4042692 | 4044602 | GID1.0043773 | scaffold_148 | 19106 | 20928 |
| Block 415 | rpl37A | 3 | 4045953 | 4046558 | estExt_Genewise1.C_1480015 | scaffold_148 | 22065 | 22547 |
| Block 2766 | ldhA | 3 | 4061672 | 4062828 | estExt_Genewise1Plus.C_720021 | scaffold_72 | 16545 | 17733 |
| Block 2766 | gpt10 | 3 | 4064325 | 4065641 | estExt_fgeneshDP_pg.C_720011 | scaffold_72 | 18668 | 20255 |
| Block 2681 | DDB_G0281143 | 3 | 4098849 | 4099499 | estExt_fgeneshDP_kg.C_670001 | scaffold_67 | 14076 | 14558 |
| Block 2681 | DDB_G0281151 | 3 | 4103245 | 4106511 | estExt_fgeneshDP_pm.C_670004 | scaffold_67 | 15283 | 18304 |
| Block 2681 | eIF3s3 | 3 | 4106976 | 4108033 | e_gw1.67.40.1 | scaffold_67 | 18610 | 19945 |
| Block 2681 | DDB_G0281155 | 3 | 4113808 | 4115562 | e_gw1.67.23.1 | scaffold_67 | 33319 | 35069 |
| Block 1397 | cog2 | 3 | 4125474 | 4128500 | estExt_Genewise1.C_3030009 | scaffold_303 | 22283 | 25622 |
| Block 2766 | dnapkcs | 3 | 4132807 | 4145996 | GID1.0041188 | scaffold_72 | 20758 | 33609 |
| Block 1639 | psmA4 | 3 | 4147505 | 4148552 | estExt_fgeneshDP_kg.C_3560001 | scaffold_356 | 4145 | 5404 |
| Block 1639 | oxaB | 3 | 4148918 | 4150249 | GID1.0048008 | scaffold_356 | 6016 | 7253 |
| Block 1639 | psmD11 | 3 | 4153223 | 4155157 | estExt_fgeneshDP_kg.C_3560002 | scaffold_356 | 7585 | 9478 |
| Block 557 | DDB_G0281175 | 3 | 4163655 | 4165436 | e_gw1.168.4.1 | scaffold_168 | 61607 | 63215 |
| Block 557 | tmem208 | 3 | 4165613 | 4166323 | GID1.0044349 | scaffold_168 | 63396 | 63990 |
| Block 1602 | clkA | 3 | 4167312 | 4170856 | e_gw1.346.13.1 | scaffold_346 | 34544 | 35534 |
| Block 1602 | DDB_G0281181 | 3 | 4171126 | 4172256 | estExt_Genewise1Plus.C_3460011 | scaffold_346 | 33243 | 34325 |
| Block 1397 | DDB_G0281321 | 3 | 4178520 | 4179137 | fgeneshDP_pg.C_scaffold_303000005 | scaffold_303 | 18561 | 19492 |
| Block 2626 | DDB_G0281325 | 3 | 4191095 | 4192891 | estExt_Genewise1.C_6440002 | scaffold_644 | 3676 | 5604 |
| Block 2626 | DDB_G0281327 | 3 | 4193116 | 4194082 | GID1.0050531 | scaffold_644 | 2497 | 3453 |
| Block 261 | DDB_G0281209 | 3 | 4207203 | 4208804 | GID1.0043183 | scaffold_128 | 23489 | 25016 |
| Block 261 | grlL | 3 | 4210774 | 4213017 | gw1.128.21.1 | scaffold_128 | 17094 | 19243 |
| Block 559 | DG1113 | 3 | 4213401 | 4217074 | e_gw1.169.18.1 | scaffold_169 | 3210 | 6795 |
| Block 559 | dhkA | 3 | 4220018 | 4226569 | GID1.0044352 | scaffold_169 | 7248 | 15363 |
| Block 2682 | pex3 | 3 | 4226854 | 4228465 | GID1.0040983 | scaffold_67 | 35257 | 36610 |
| Block 2682 | brf1 | 3 | 4228761 | 4230881 | gw1.67.1.1 | scaffold_67 | 37230 | 38921 |
| Block 2682 | DDB_G0281217 | 3 | 4231408 | 4233727 | fgeneshDP_pg.C_scaffold_67000016 | scaffold_67 | 39499 | 41434 |
| Block 2682 | DDB_G0281219 | 3 | 4234799 | 4235929 | estExt_fgeneshDP_kg.C_670003 | scaffold_67 | 41491 | 42834 |
| Block 2682 | tbccd | 3 | 4236950 | 4238575 | estExt_fgeneshDP_pg.C_670019 | scaffold_67 | 45475 | 46887 |
| Block 2682 | DDB_G0281223 | 3 | 4239018 | 4240493 | fgeneshDP_pg.C_scaffold_67000020 | scaffold_67 | 47181 | 48576 |
| Block 1608 | DDB_G0281225 | 3 | 4242261 | 4243821 | fgeneshDP_pm.C_scaffold_349000004 | scaffold_349 | 30288 | 31592 |
| Block 1608 | DDB_G0281227 | 3 | 4244319 | 4244805 | GID1.0047909 | scaffold_349 | 29541 | 30025 |
| Block 1608 | PNKP | 3 | 4245222 | 4246856 | gw1.349.5.1 | scaffold_349 | 27367 | 28917 |
| Block 414 | crlA | 3 | 4253816 | 4255131 | e_gw1.148.28.1 | scaffold_148 | 7725 | 9107 |
| Block 414 | DDB_G0281235 | 3 | 4255795 | 4257140 | estExt_Genewise1.C_1480004 | scaffold_148 | 6383 | 7538 |
| Block 414 | DDB_G0281237 | 3 | 4257593 | 4260812 | fgeneshDP_pg.C_scaffold_148000002 | scaffold_148 | 3396 | 5944 |
| Block 255 | DDB_G0281331 | 3 | 4278355 | 4280707 | e_gw1.127.17.1 | scaffold_127 | 45909 | 48199 |
| Block 255 | lvsC | 3 | 4286715 | 4294937 | gw1.127.12.1 | scaffold_127 | 53261 | 60848 |
| Block 2993 | DDB_G0281259 | 3 | 4305745 | 4307064 | e_gw1.90.25.1 | scaffold_90 | 47785 | 49033 |
| Block 2993 | DDB_G0281261 | 3 | 4307171 | 4310514 | GID1.0041909 | scaffold_90 | 54181 | 57477 |
| Block 2993 | DDB_G0281265 | 3 | 4311777 | 4313561 | GID1.0041913 | scaffold_90 | 63019 | 64795 |
| Block 2993 | DDB_G0281269 | 3 | 4314924 | 4316454 | fgeneshDP_pg.C_scaffold_90000025 | scaffold_90 | 66626 | 67995 |
| Block 2993 | DDB_G0281271 | 3 | 4316503 | 4317240 | fgeneshDP_pg.C_scaffold_90000026 | scaffold_90 | 68104 | 68820 |
| Block 2993 | med12 | 3 | 4322929 | 4331907 | fgeneshDP_pg.C_scaffold_90000027 | scaffold_90 | 69888 | 75969 |
| Block 247 | DDB_G0281279 | 3 | 4333375 | 4335700 | estExt_fgeneshDP_pm.C_1260005 | scaffold_126 | 6945 | 9663 |
| Block 247 | DDB_G0281281 | 3 | 4335971 | 4336735 | estExt_fgeneshDP_kg.C_1260001 | scaffold_126 | 9650 | 10532 |
| Block 247 | DDB_G0281283 | 3 | 4337681 | 4338432 | e_gw1.126.33.1 | scaffold_126 | 11431 | 12042 |
| Block 414 | racE | 3 | 4354163 | 4355462 | estExt_fgeneshDP_pg.C_1480001 | scaffold_148 | 1682 | 2820 |
| Block 2543 | pcmA | 3 | 4356442 | 4357481 | fgeneshDP_pm.C_scaffold_610000001 | scaffold_610 | 564 | 1589 |
| Block 2543 | DDB_G0281289 | 3 | 4358553 | 4360353 | e_gw1.610.4.1 | scaffold_610 | 2668 | 4015 |
| Block 2543 | DDB_G0281369 | 3 | 4370772 | 4372312 | e_gw1.610.9.1 | scaffold_610 | 10359 | 11066 |
| Block 2543 | DDB_G0281371 | 3 | 4372875 | 4374176 | fgeneshDP_pg.C_scaffold_610000004 | scaffold_610 | 8737 | 9954 |
| Block 2543 | shkD | 3 | 4376038 | 4378565 | GID1.0050363 | scaffold_610 | 4845 | 7085 |
| Block 490 | rab14 | 3 | 4383385 | 4384571 | estExt_fgeneshDP_kg.C_1560007 | scaffold_156 | 53882 | 55541 |
| Block 490 | DDB_G0281351 | 3 | 4385822 | 4387835 | estExt_Genewise1Plus.C_1560041 | scaffold_156 | 56007 | 58071 |
| Block 490 | DDB_G0281353 | 3 | 4388160 | 4394973 | e_gw1.156.17.1 | scaffold_156 | 58417 | 64928 |
| Block 1086 | DDB_G0281361 | 3 | 4403294 | 4405281 | GID1.0046086 | scaffold_247 | 4648 | 6383 |
| Block 1086 | DDB_G0281363 | 3 | 4410114 | 4413041 | GID1.0046097 | scaffold_247 | 33359 | 36237 |
| Block 1250 | DDB_G0281367 | 3 | 4413764 | 4414489 | fgeneshDP_pg.C_scaffold_279000011 | scaffold_279 | 24889 | 25476 |
| Block 1250 | DDB_G0281379 | 3 | 4415599 | 4417045 | GID1.0046748 | scaffold_279 | 26492 | 28297 |
| Block 1086 | cxdA | 3 | 4421049 | 4422099 | e_gw1.247.40.1 | scaffold_247 | 39275 | 40298 |
| Block 1250 | nacA | 3 | 4433625 | 4434218 | estExt_Genewise1.C_2790010 | scaffold_279 | 10536 | 11186 |
| Block 1250 | drpp25 | 3 | 4439811 | 4440920 | estExt_fgeneshDP_kg.C_2790001 | scaffold_279 | 8742 | 10133 |
| Block 1250 | DDB_G0281415 | 3 | 4443494 | 4446173 | estExt_Genewise1Plus.C_2790006 | scaffold_279 | 5639 | 8509 |
| Block 1250 | dstA | 3 | 4448074 | 4450633 | estExt_Genewise1Plus.C_2790002 | scaffold_279 | 962 | 2843 |
| Block 1965 | DDB_G0281425 | 3 | 4468486 | 4470184 | e_gw1.430.5.1 | scaffold_430 | 14490 | 15902 |
| Block 1965 | DDB_G0281427 | 3 | 4470521 | 4474783 | GID1.0048975 | scaffold_430 | 16283 | 19723 |
| Block 394 | DDB_G0281429 | 3 | 4475058 | 4476479 | fgeneshDP_pg.C_scaffold_145000004 | scaffold_145 | 7278 | 9175 |
| Block 394 | DDB_G0281431 | 3 | 4477171 | 4480036 | estExt_fgeneshDP_kg.C_1450002 | scaffold_145 | 4042 | 5347 |
| Block 2858 | DDB_G0281521 | 3 | 4480305 | 4485819 | estExt_fgeneshDP_pg.C_790002 | scaffold_79 | 3601 | 8838 |
| Block 2858 | DDB_G0281523 | 3 | 4486206 | 4487897 | estExt_Genewise1.C_790001 | scaffold_79 | 1289 | 2911 |
| Block 394 | srfA | 3 | 4494019 | 4495275 | estExt_fgeneshDP_kg.C_1450001 | scaffold_145 | 1 | 2102 |
| Block 2858 | DDB_G0281433 | 3 | 4495953 | 4497167 | GID1.0041461 | scaffold_79 | 9038 | 10195 |
| Block 2858 | mrpl17 | 3 | 4497695 | 4498243 | e_gw1.79.12.1 | scaffold_79 | 10608 | 11144 |
| Block 394 | lysS | 3 | 4498697 | 4500739 | estExt_fgeneshDP_pm.C_1450003 | scaffold_145 | 14170 | 23394 |
| Block 394 | ercc6 | 3 | 4502255 | 4507222 | GID1.0043697 | scaffold_145 | 41032 | 45737 |
| Block 394 | DDB_G0281443 | 3 | 4509513 | 4511664 | fgeneshDP_pg.C_scaffold_145000013 | scaffold_145 | 37162 | 39574 |
| Block 1250 | DDB_G0281445 | 3 | 4513064 | 4515098 | estExt_Genewise1Plus.C_2790017 | scaffold_279 | 22974 | 24777 |
| Block 1250 | DDB_G0281447 | 3 | 4516135 | 4517301 | e_gw1.279.23.1 | scaffold_279 | 81 | 664 |
| Block 2390 | rasC | 3 | 4519070 | 4520084 | estExt_Genewise1.C_5530005 | scaffold_553 | 13646 | 14665 |
| Block 2390 | DDB_G0281527 | 3 | 4520387 | 4522554 | e_gw1.553.1.1 | scaffold_553 | 11401 | 13366 |
| Block 927 | DDB_G0281465 | 3 | 4545446 | 4546801 | fgeneshDP_pm.C_scaffold_221000010 | scaffold_221 | 39366 | 40634 |
| Block 927 | rpl29 | 3 | 4548958 | 4549339 | estExt_Genewise1Plus.C_2210030 | scaffold_221 | 38491 | 39051 |
| Block 927 | pdkA | 3 | 4550105 | 4552335 | estExt_Genewise1Plus.C_2210026 | scaffold_221 | 35684 | 38304 |
| Block 927 | DDB_G0281473 | 3 | 4553203 | 4555374 | estExt_Genewise1.C_2210023 | scaffold_221 | 32377 | 34912 |
| Block 1932 | abcG6 | 3 | 4592822 | 4597860 | estExt_Genewise1.C_4220006 | scaffold_422 | 9341 | 14505 |
| Block 1932 | DDB_G0281485 | 3 | 4599041 | 4603561 | GID1.0048894 | scaffold_422 | 3895 | 7640 |
| Block 349 | DDB_G0281487 | 3 | 4603817 | 4604947 | fgeneshDP_pg.C_scaffold_139000016 | scaffold_139 | 37702 | 38814 |
| Block 349 | DDB_G0281489 | 3 | 4605058 | 4605743 | fgeneshDP_pg.C_scaffold_139000015 | scaffold_139 | 36893 | 37564 |
| Block 395 | DDB_G0281497 | 3 | 4615481 | 4617145 | estExt_fgeneshDP_pg.C_1450011 | scaffold_145 | 25630 | 29147 |
| Block 395 | DDB_G0281499 | 3 | 4621318 | 4626772 | GID1.0043695 | scaffold_145 | 31606 | 37082 |
| Block 395 | DDB_G0281541 | 3 | 4627625 | 4629265 | e_gw1.145.23.1 | scaffold_145 | 23698 | 25383 |
| Block 2859 | DDB_G0281543 | 3 | 4629488 | 4633507 | estExt_Genewise1Plus.C_790010 | scaffold_79 | 12116 | 16243 |
| Block 2859 | DDB_G0281501 | 3 | 4634453 | 4636939 | fgeneshDP_pg.C_scaffold_79000006 | scaffold_79 | 16481 | 18908 |
| Block 2628 | DDB_G0281503 | 3 | 4637514 | 4640951 | GID1.0050534 | scaffold_645 | 2 | 1850 |
| Block 2628 | DDB_G0281505 | 3 | 4641293 | 4642444 | GID1.0050535 | scaffold_645 | 2051 | 3116 |
| Block 2628 | colC | 3 | 4643635 | 4646580 | estExt_fgeneshDP_pg.C_6450003 | scaffold_645 | 3533 | 7793 |
| Block 2628 | DDB_G0281509 | 3 | 4647716 | 4648014 | GID1.0050537 | scaffold_645 | 9130 | 9403 |
| Block 117 | cpsf1 | 3 | 4665204 | 4670752 | e_gw1.110.2.1 | scaffold_110 | 54826 | 59934 |
| Block 2081 | yipf1 | 3 | 4674842 | 4676185 | estExt_Genewise1.C_4610002 | scaffold_461 | 993 | 2532 |
| Block 2081 | DDB_G0281589 | 3 | 4676319 | 4677638 | estExt_Genewise1Plus.C_4610005 | scaffold_461 | 2528 | 4074 |
| Block 1881 | DDB_G0281595 | 3 | 4685430 | 4686634 | e_gw1.41.42.1 | scaffold_41 | 54804 | 55954 |
| Block 1881 | nacB | 3 | 4686750 | 4687178 | estExt_fgeneshDP_kg.C_410002 | scaffold_41 | 56024 | 56493 |
| Block 1881 | bkdC | 3 | 4689130 | 4690767 | estExt_fgeneshDP_pm.C_410008 | scaffold_41 | 58168 | 60043 |
| Block 1881 | abkB | 3 | 4693676 | 4696009 | gw1.41.28.1 | scaffold_41 | 65308 | 67398 |
| Block 1881 | DDB_G0281599 | 3 | 4696808 | 4697785 | GID1.0039805 | scaffold_41 | 63687 | 64725 |
| Block 1881 | DDB_G0281601 | 3 | 4697870 | 4698426 | gw1.41.62.1 | scaffold_41 | 62907 | 63514 |
| Block 1881 | DDB_G0281603 | 3 | 4698508 | 4700770 | GID1.0039803 | scaffold_41 | 60209 | 62559 |
| Block 253 | cfaD | 3 | 4701100 | 4702904 | estExt_Genewise1Plus.C_1270011 | scaffold_127 | 27156 | 28938 |
| Block 253 | DDB_G0281607 | 3 | 4703616 | 4704607 | estExt_fgeneshDP_kg.C_1270003 | scaffold_127 | 25000 | 26460 |
| Block 117 | guaA | 3 | 4709076 | 4711232 | estExt_fgeneshDP_kg.C_1100007 | scaffold_110 | 46934 | 48214 |
| Block 117 | DDB_G0281621 | 3 | 4716452 | 4718620 | GID1.0042601 | scaffold_110 | 49460 | 51529 |
| Block 453 | DDB_G0281627 | 3 | 4725130 | 4727134 | GID1.0043840 | scaffold_150 | 55023 | 56968 |
| Block 453 | DDB_G0281639 | 3 | 4746209 | 4748719 | e_gw1.150.25.1 | scaffold_150 | 40757 | 43196 |
| Block 453 | polD2 | 3 | 4748825 | 4750464 | GID1.0043834 | scaffold_150 | 43329 | 45044 |
| Block 453 | DDB_G0281643 | 3 | 4751182 | 4753968 | estExt_fgeneshDP_kg.C_1500004 | scaffold_150 | 48361 | 50144 |
| Block 2332 | mkcE | 3 | 4757942 | 4760392 | estExt_fgeneshDP_pm.C_5310001 | scaffold_531 | 404 | 2701 |
| Block 2332 | DDB_G0281651 | 3 | 4760769 | 4762128 | fgeneshDP_pm.C_scaffold_531000002 | scaffold_531 | 5139 | 7107 |
| Block 2773 | mybC | 3 | 4766011 | 4767857 | e_gw1.723.2.1 | scaffold_723 | 5574 | 6995 |
| Block 2773 | DDB_G0281653 | 3 | 4768142 | 4769409 | e_gw1.723.3.1 | scaffold_723 | 4025 | 5207 |
| Block 2773 | DDB_G0281657 | 3 | 4770616 | 4773886 | e_gw1.723.5.1 | scaffold_723 | 304 | 2478 |
| Block 201 | drkD | 3 | 4777368 | 4781526 | e_gw1.121.13.1 | scaffold_121 | 1422 | 5270 |
| Block 201 | gtaI | 3 | 4784467 | 4786077 | estExt_Genewise1Plus.C_1210006 | scaffold_121 | 7721 | 10507 |
| Block 201 | aprA | 3 | 4787137 | 4789219 | fgeneshDP_pg.C_scaffold_121000003 | scaffold_121 | 11028 | 13299 |
| Block 201 | DDB_G0281669 | 3 | 4791599 | 4794060 | estExt_Genewise1Plus.C_1210011 | scaffold_121 | 13647 | 16345 |
| Block 201 | DDB_G0281671 | 3 | 4794386 | 4798555 | GID1.0042959 | scaffold_121 | 16334 | 23582 |
| Block 1824 | rps26 | 3 | 4801949 | 4802463 | estExt_Genewise1Plus.C_40031 | scaffold_4 | 49585 | 50088 |
| Block 1824 | DDB_G0281679 | 3 | 4804047 | 4805222 | GID1.0037505 | scaffold_4 | 51696 | 52988 |
| Block 1824 | msh3 | 3 | 4806161 | 4810447 | estExt_Genewise1Plus.C_40035 | scaffold_4 | 54776 | 57957 |
| Block 1824 | nol1 | 3 | 4810538 | 4812581 | GID1.0037507 | scaffold_4 | 58115 | 60236 |
| Block 1824 | DDB_G0281687 | 3 | 4813506 | 4816440 | GID1.0037502 | scaffold_4 | 45617 | 48272 |
| Block 1824 | rpl19 | 3 | 4825161 | 4825826 | gw1.4.35.1 | scaffold_4 | 43366 | 44049 |
| Block 120 | gefP | 3 | 4826796 | 4831443 | GID1.0042639 | scaffold_111 | 61534 | 65545 |
| Block 120 | DDB_G0281693 | 3 | 4833441 | 4834541 | fgeneshDP_pg.C_scaffold_111000023 | scaffold_111 | 68201 | 69247 |
| Block 1824 | DDB_G0281705 | 3 | 4853137 | 4854186 | estExt_fgeneshDP_pg.C_40018 | scaffold_4 | 44311 | 45440 |
| Block 1595 | DDB_G0281811 | 3 | 4857991 | 4858413 | estExt_fgeneshDP_kg.C_3450001 | scaffold_345 | 23173 | 24088 |
| Block 1595 | DDB_G0281709 | 3 | 4862322 | 4866125 | GID1.0047845 | scaffold_345 | 18221 | 21562 |
| Block 1595 | ddx27 | 3 | 4866495 | 4869027 | GID1.0047844 | scaffold_345 | 15503 | 18039 |
| Block 1595 | DDB_G0281713 | 3 | 4869216 | 4869587 | GID1.0047843 | scaffold_345 | 14939 | 15160 |
| Block 1595 | fhkC | 3 | 4871788 | 4873878 | GID1.0047842 | scaffold_345 | 12463 | 14337 |
| Block 1406 | DDB_G0281719 | 3 | 4874420 | 4877744 | GID1.0047192 | scaffold_305 | 5409 | 7799 |
| Block 1406 | DDB_G0281723 | 3 | 4880176 | 4881555 | fgeneshDP_pg.C_scaffold_305000005 | scaffold_305 | 9787 | 10459 |
| Block 1406 | ube2m | 3 | 4883727 | 4884819 | fgeneshDP_pg.C_scaffold_305000006 | scaffold_305 | 13691 | 14667 |
| Block 1406 | DDB_G0281729 | 3 | 4886865 | 4888149 | fgeneshDP_kg.C_scaffold_305000001 | scaffold_305 | 2912 | 4301 |
| Block 1406 | DDB_G0281731 | 3 | 4889095 | 4889283 | e_gw1.305.19.1 | scaffold_305 | 2021 | 2272 |
| Block 2526 | gacY | 3 | 4898948 | 4901276 | GID1.0050326 | scaffold_603 | 325 | 3909 |
| Block 2526 | cct3 | 3 | 4903167 | 4904990 | estExt_Genewise1.C_6030007 | scaffold_603 | 5057 | 7042 |
| Block 1406 | DDB_G0281743 | 3 | 4905122 | 4909573 | estExt_fgeneshDP_pg.C_3050009 | scaffold_305 | 21690 | 25799 |
| Block 2526 | DDB_G0281745 | 3 | 4910382 | 4912175 | GID1.0050329 | scaffold_603 | 8418 | 10056 |
| Block 1406 | DDB_G0281747 | 3 | 4912687 | 4913451 | estExt_fgeneshDP_pg.C_3050010 | scaffold_305 | 26205 | 27048 |
| Block 1830 | DDB_G0281815 | 3 | 4913622 | 4914745 | e_gw1.4.90.1 | scaffold_4 | 105066 | 106186 |
| Block 1830 | DDB_G0281749 | 3 | 4915108 | 4916323 | GID1.0037522 | scaffold_4 | 103757 | 104862 |
| Block 1830 | DDB_G0281751 | 3 | 4916395 | 4917360 | e_gw1.4.94.1 | scaffold_4 | 102682 | 103641 |
| Block 1830 | DDB_G0281759 | 3 | 4924793 | 4925983 | gw1.4.78.1 | scaffold_4 | 101375 | 102391 |
| Block 1830 | DDB_G0281761 | 3 | 4926150 | 4927037 | e_gw1.4.92.1 | scaffold_4 | 100343 | 101320 |
| Block 1830 | DDB_G0281763 | 3 | 4927454 | 4928627 | estExt_Genewise1Plus.C_40053 | scaffold_4 | 98653 | 99911 |
| Block 1003 | tor | 3 | 4934791 | 4942180 | estExt_Genewise1.C_2320008 | scaffold_232 | 12610 | 19895 |
| Block 1003 | DDB_G0281771 | 3 | 4943706 | 4944176 | GID1.0045811 | scaffold_232 | 10773 | 11240 |
| Block 2704 | helC | 3 | 4949807 | 4951339 | fgeneshDP_pg.C_scaffold_681000003 | scaffold_681 | 4233 | 5759 |
| Block 2704 | DDB_G0281777 | 3 | 4951421 | 4952644 | fgeneshDP_pg.C_scaffold_681000002 | scaffold_681 | 2990 | 3979 |
| Block 2704 | lsm2 | 3 | 4953475 | 4954320 | fgeneshDP_pg.C_scaffold_681000001 | scaffold_681 | 1267 | 1782 |
| Block 202 | DDB_G0281785 | 3 | 4971939 | 4974761 | GID1.0042960 | scaffold_121 | 23971 | 26595 |
| Block 2906 | coxA | 3 | 4977091 | 4978539 | estExt_Genewise1Plus.C_810042 | scaffold_81 | 58021 | 59495 |
| Block 2906 | DG1080 | 3 | 4978870 | 4981935 | e_gw1.81.8.1 | scaffold_81 | 60801 | 62402 |
| Block 2906 | gpn2 | 3 | 4982225 | 4983345 | e_gw1.81.52.1 | scaffold_81 | 64548 | 65572 |
| Block 2906 | DDB_G0281789 | 3 | 4983430 | 4984948 | GID1.0041565 | scaffold_81 | 65660 | 67138 |
| Block 874 | DDB_G0281791 | 3 | 4985633 | 4985776 | e_gw1.211.32.1 | scaffold_211 | 43343 | 43492 |
| Block 874 | DDB_G0281793 | 3 | 4985912 | 4986452 | e_gw1.211.28.1 | scaffold_211 | 43643 | 44236 |
| Block 2906 | gtaJ | 3 | 4990977 | 4993121 | estExt_fgeneshDP_pg.C_810039 | scaffold_81 | 88308 | 89255 |
| Block 2906 | DDB_G0281833 | 3 | 4994441 | 4995199 | fgeneshDP_pm.C_scaffold_81000020 | scaffold_81 | 86128 | 86776 |
| Block 2906 | DDB_G0281835 | 3 | 4995492 | 4996860 | GID1.0041572 | scaffold_81 | 84492 | 85876 |
| Block 2906 | DDB_G0281837 | 3 | 4996941 | 4997559 | GID1.0041571 | scaffold_81 | 83685 | 84310 |
| Block 2906 | DDB_G0281839 | 3 | 4997894 | 5000502 | GID1.0041570 | scaffold_81 | 80876 | 83385 |
| Block 2906 | ddx24 | 3 | 5000989 | 5003811 | GID1.0041569 | scaffold_81 | 77672 | 80452 |
| Block 202 | DDB_G0281843 | 3 | 5003890 | 5005484 | e_gw1.121.32.1 | scaffold_121 | 62849 | 64247 |
| Block 1586 | DDB_G0281845 | 3 | 5006578 | 5010931 | GID1.0047814 | scaffold_343 | 16390 | 20104 |
| Block 1407 | V4-7 | 3 | 5023432 | 5025642 | fgeneshDP_pg.C_scaffold_305000007 | scaffold_305 | 15784 | 17996 |
| Block 1586 | sre1 | 3 | 5029047 | 5030143 | e_gw1.343.15.1 | scaffold_343 | 14806 | 15762 |
| Block 1586 | DDB_G0281857 | 3 | 5031854 | 5035144 | estExt_Genewise1Plus.C_3430004 | scaffold_343 | 10526 | 14318 |
| Block 1151 | DDB_G0281937 | 3 | 5037215 | 5037808 | estExt_Genewise1Plus.C_2590014 | scaffold_259 | 17046 | 17841 |
| Block 1151 | DDB_G0281861 | 3 | 5038245 | 5040197 | e_gw1.259.2.1 | scaffold_259 | 18256 | 20129 |
| Block 447 | slob1 | 3 | 5040353 | 5042502 | fgeneshDP_pg.C_scaffold_150000007 | scaffold_150 | 12102 | 13985 |
| Block 447 | adh5 | 3 | 5043162 | 5044640 | estExt_Genewise1.C_1500011 | scaffold_150 | 9794 | 11287 |
| Block 1407 | DDB_G0281871 | 3 | 5051942 | 5054758 | GID1.0047198 | scaffold_305 | 18209 | 20964 |
| Block 1831 | pfdn4 | 3 | 5055049 | 5055612 | fgeneshDP_pg.C_scaffold_4000040 | scaffold_4 | 106355 | 107057 |
| Block 1831 | DDB_G0281875 | 3 | 5056031 | 5056821 | GID1.0037530 | scaffold_4 | 116973 | 117715 |
| Block 869 | DDB_G0281945 | 3 | 5071587 | 5072946 | fgeneshDP_pg.C_scaffold_211000019 | scaffold_211 | 47818 | 49317 |
| Block 1831 | DDB_G0281889 | 3 | 5074777 | 5076633 | estExt_Genewise1Plus.C_40071 | scaffold_4 | 114976 | 116875 |
| Block 869 | mrkC | 3 | 5083659 | 5086073 | GID1.0045367 | scaffold_211 | 51614 | 54114 |
| Block 1831 | drkC | 3 | 5088116 | 5090879 | e_gw1.4.63.1 | scaffold_4 | 107584 | 109675 |
| Block 1831 | slu7 | 3 | 5091808 | 5093614 | e_gw1.4.10.1 | scaffold_4 | 110838 | 112499 |
| Block 1831 | DDB_G0281903 | 3 | 5094433 | 5095945 | e_gw1.4.76.1 | scaffold_4 | 113142 | 114583 |
| Block 869 | DDB_G0281907 | 3 | 5099224 | 5103373 | estExt_fgeneshDP_pg.C_2110006 | scaffold_211 | 15558 | 18950 |
| Block 869 | DDB_G0281909 | 3 | 5103620 | 5105863 | GID1.0045351 | scaffold_211 | 19077 | 21105 |
| Block 869 | DDB_G0281911 | 3 | 5106919 | 5108104 | e_gw1.211.15.1 | scaffold_211 | 50247 | 51217 |
| Block 1831 | DDB_G0281913 | 3 | 5108423 | 5109952 | estExt_fgeneshDP_kg.C_40010 | scaffold_4 | 119639 | 121169 |
| Block 1831 | comB | 3 | 5112758 | 5119385 | GID1.0037533 | scaffold_4 | 121407 | 126921 |
| Block 1831 | DDB_G0281917 | 3 | 5121103 | 5122056 | e_gw1.4.86.1 | scaffold_4 | 129096 | 130181 |
| Block 249 | DDB_G0281923 | 3 | 5126418 | 5131891 | e_gw1.126.7.1 | scaffold_126 | 34957 | 40320 |
| Block 249 | DDB_G0281925 | 3 | 5132437 | 5134280 | estExt_Genewise1.C_1260045 | scaffold_126 | 32327 | 34651 |
| Block 2947 | dwwA | 3 | 5135547 | 5137365 | GID1.0041778 | scaffold_86 | 79309 | 81032 |
| Block 2947 | tra1 | 3 | 5137846 | 5151854 | GID1.0041777 | scaffold_86 | 65714 | 78504 |
| Block 545 | nola4 | 3 | 5161271 | 5163028 | e_gw1.166.5.1 | scaffold_166 | 28087 | 33631 |
| Block 545 | DDB_G0281963 | 3 | 5173395 | 5174956 | GID1.0044274 | scaffold_166 | 13676 | 14975 |
| Block 2124 | DDB_G0281965 | 3 | 5175300 | 5175752 | gw1.473.14.1 | scaffold_473 | 5675 | 6007 |
| Block 2124 | slrA | 3 | 5176361 | 5180936 | estExt_Genewise1.C_4730009 | scaffold_473 | 6663 | 10984 |
| Block 1383 | mybE | 3 | 5190182 | 5192638 | estExt_fgeneshDP_pg.C_3010001 | scaffold_301 | 1200 | 3261 |
| Block 1383 | DG1060 | 3 | 5193203 | 5193814 | GID1.0047129 | scaffold_301 | 3332 | 3971 |
| Block 545 | ap1g1 | 3 | 5194623 | 5197502 | estExt_fgeneshDP_pg.C_1660001 | scaffold_166 | 3261 | 6239 |
| Block 545 | DDB_G0281971 | 3 | 5200691 | 5201407 | e_gw1.166.41.1 | scaffold_166 | 8534 | 9187 |
| Block 545 | gxcG | 3 | 5205115 | 5207257 | GID1.0044273 | scaffold_166 | 10228 | 13623 |
| Block 2213 | DDB_G0281979 | 3 | 5208875 | 5210138 | e_gw1.5.57.1 | scaffold_5 | 61282 | 62653 |
| Block 2213 | DDB_G0281981 | 3 | 5213481 | 5214734 | estExt_fgeneshDP_pg.C_50022 | scaffold_5 | 57455 | 58661 |
| Block 2213 | tmem50 | 3 | 5214975 | 5215799 | estExt_Genewise1Plus.C_50019 | scaffold_5 | 56500 | 57341 |
| Block 2213 | sec23 | 3 | 5216752 | 5219266 | estExt_Genewise1.C_50018 | scaffold_5 | 46767 | 49647 |
| Block 2213 | lyrm1 | 3 | 5219597 | 5220032 | e_gw1.5.73.1 | scaffold_5 | 46262 | 46695 |
| Block 2213 | DDB_G0281991 | 3 | 5222837 | 5224029 | GID1.0037595 | scaffold_5 | 44654 | 45792 |
| Block 2518 | DDB_G0281993 | 3 | 5224852 | 5225898 | estExt_fgeneshDP_kg.C_600017 | scaffold_60 | 95983 | 97229 |
| Block 1833 | DDB_G0282007 | 3 | 5236311 | 5237319 | GID1.0037544 | scaffold_4 | 145668 | 146629 |
| Block 1833 | ahhA | 3 | 5237565 | 5238371 | fgeneshDP_pg.C_scaffold_4000057 | scaffold_4 | 144753 | 145556 |
| Block 1833 | DDB_G0282075 | 3 | 5239385 | 5240941 | estExt_fgeneshDP_pg.C_40056 | scaffold_4 | 142893 | 144563 |
| Block 642 | DDB_G0282011 | 3 | 5241503 | 5243356 | GID1.0038542 | scaffold_18 | 140650 | 142352 |
| Block 642 | DDB_G0282017 | 3 | 5245886 | 5247937 | GID1.0038541 | scaffold_18 | 138377 | 140455 |
| Block 2518 | DDB_G0282087 | 3 | 5297220 | 5298359 | GID1.0040684 | scaffold_60 | 52552 | 53721 |
| Block 2518 | eIF2s2 | 3 | 5298628 | 5299779 | estExt_Genewise1.C_600031 | scaffold_60 | 51062 | 52478 |
| Block 2061 | DDB_G0282041 | 3 | 5320048 | 5321192 | gw1.457.8.1 | scaffold_457 | 10360 | 11007 |
| Block 2061 | vps36 | 3 | 5326311 | 5328458 | e_gw1.457.5.1 | scaffold_457 | 11705 | 13539 |
| Block 1640 | DDB_G0282059 | 3 | 5353948 | 5357409 | e_gw1.356.4.1 | scaffold_356 | 30046 | 33741 |
| Block 1468 | DDB_G0282065 | 3 | 5362723 | 5363052 | GID1.0047429 | scaffold_319 | 1121 | 1333 |
| Block 1468 | DDB_G0282067 | 3 | 5363274 | 5364995 | gw1.319.19.1 | scaffold_319 | 8503 | 9825 |
| Block 1468 | rio2 | 3 | 5366397 | 5368196 | GID1.0047432 | scaffold_319 | 5316 | 7056 |
| Block 1468 | cxfA | 3 | 5368452 | 5368825 | GID1.0047431 | scaffold_319 | 4783 | 5046 |
| Block 1468 | DDB_G0282101 | 3 | 5369694 | 5372796 | GID1.0047430 | scaffold_319 | 1472 | 3976 |
| Block 1640 | abcG24 | 3 | 5372934 | 5376503 | estExt_Genewise1Plus.C_3560012 | scaffold_356 | 26523 | 30004 |
| Block 1640 | DDB_G0282105 | 3 | 5377643 | 5380519 | GID1.0048015 | scaffold_356 | 23109 | 25640 |
| Block 1640 | DDB_G0282131 | 3 | 5381150 | 5383925 | GID1.0048014 | scaffold_356 | 19945 | 22756 |
| Block 2563 | DDB_G0282113 | 3 | 5398887 | 5399607 | estExt_fgeneshDP_kg.C_6180001 | scaffold_618 | 3501 | 4224 |
| Block 2563 | DDB_G0282115 | 3 | 5399711 | 5404710 | estExt_fgeneshDP_pg.C_6180003 | scaffold_618 | 4252 | 8719 |
| Block 1385 | DDB_G0282125 | 3 | 5425596 | 5428416 | GID1.0047136 | scaffold_301 | 20021 | 22661 |
| Block 1385 | DDB_G0282127 | 3 | 5429045 | 5430156 | GID1.0047135 | scaffold_301 | 18548 | 19592 |
| Block 1385 | DDB_G0282139 | 3 | 5433286 | 5438655 | estExt_Genewise1.C_3010010 | scaffold_301 | 10316 | 12730 |
| Block 1385 | ptpC | 3 | 5439955 | 5443028 | estExt_fgeneshDP_pg.C_3010006 | scaffold_301 | 15385 | 18330 |
| Block 1837 | nvl | 3 | 5443128 | 5445837 | GID1.0037567 | scaffold_4 | 192458 | 195020 |
| Block 1837 | DDB_G0282149 | 3 | 5446691 | 5453615 | e_gw1.4.50.1 | scaffold_4 | 207813 | 215032 |
| Block 1455 | DDB_G0282151 | 3 | 5456198 | 5458674 | GID1.0047382 | scaffold_315 | 33923 | 36294 |
| Block 1455 | DDB_G0282155 | 3 | 5462477 | 5463732 | e_gw1.315.10.1 | scaffold_315 | 29778 | 30919 |
| Block 1455 | DDB_G0282185 | 3 | 5463972 | 5466440 | gw1.315.25.1 | scaffold_315 | 28969 | 29118 |
| Block 1455 | DDB_G0282157 | 3 | 5467686 | 5469353 | gw1.315.4.1 | scaffold_315 | 24504 | 26126 |
| Block 1455 | DDB_G0282159 | 3 | 5469433 | 5471067 | estExt_Genewise1.C_3150015 | scaffold_315 | 22585 | 24476 |
| Block 1455 | DDB_G0282161 | 3 | 5471894 | 5473402 | fgeneshDP_pg.C_scaffold_315000009 | scaffold_315 | 20501 | 21806 |
| Block 1976 | DDB_G0282177 | 3 | 5490516 | 5491077 | GID1.0049023 | scaffold_435 | 21466 | 22002 |
| Block 1976 | DDB_G0282179 | 3 | 5491825 | 5492262 | estExt_Genewise1.C_4350013 | scaffold_435 | 22743 | 23424 |
| Block 1976 | DDB_G0282187 | 3 | 5492546 | 5495583 | fgeneshDP_pg.C_scaffold_435000008 | scaffold_435 | 23513 | 24626 |
| Block 1271 | CYP515B1 | 3 | 5497711 | 5499541 | estExt_fgeneshDP_pg.C_2810001 | scaffold_281 | 1129 | 2859 |
| Block 1271 | polA1 | 3 | 5500018 | 5504728 | estExt_Genewise1.C_2810004 | scaffold_281 | 3134 | 7812 |
| Block 1976 | DDB_G0282193 | 3 | 5505181 | 5505851 | GID1.0049022 | scaffold_435 | 20213 | 20777 |
| Block 1964 | timm16 | 3 | 5506037 | 5506499 | e_gw1.430.6.1 | scaffold_430 | 9170 | 9650 |
| Block 1964 | DDB_G0282197 | 3 | 5507533 | 5509512 | e_gw1.430.4.1 | scaffold_430 | 7308 | 8332 |
| Block 1964 | DDB_G0282285 | 3 | 5511594 | 5513276 | estExt_fgeneshDP_pg.C_4300003 | scaffold_430 | 4704 | 6580 |
| Block 1271 | allB1 | 3 | 5513834 | 5515875 | estExt_Genewise1Plus.C_2810010 | scaffold_281 | 16888 | 18970 |
| Block 1271 | DDB_G0282201 | 3 | 5515941 | 5517965 | fgeneshDP_pg.C_scaffold_281000006 | scaffold_281 | 19010 | 20722 |
| Block 1399 | DDB_G0282209 | 3 | 5522317 | 5524513 | estExt_fgeneshDP_pg.C_3030010 | scaffold_303 | 29063 | 31282 |
| Block 1399 | DDB_G0282211 | 3 | 5525043 | 5526594 | e_gw1.303.6.1 | scaffold_303 | 31845 | 33363 |
| Block 1399 | DDB_G0282213 | 3 | 5526720 | 5528464 | estExt_fgeneshDP_pg.C_3030012 | scaffold_303 | 33464 | 35711 |
| Block 177 | mfap1 | 3 | 5530765 | 5532147 | estExt_fgeneshDP_kg.C_1190002 | scaffold_119 | 30088 | 30887 |
| Block 177 | DDB_G0282221 | 3 | 5532383 | 5536679 | GID1.0042891 | scaffold_119 | 25255 | 28904 |
| Block 2765 | DDB_G0282223 | 3 | 5536930 | 5539561 | GID1.0041185 | scaffold_72 | 14416 | 16484 |
| Block 177 | DDB_G0282287 | 3 | 5546265 | 5548140 | GID1.0042894 | scaffold_119 | 31877 | 33816 |
| Block 2819 | nhp2l1 | 3 | 5564223 | 5564709 | GID1.0041334 | scaffold_75 | 83326 | 83715 |
| Block 2819 | forD | 3 | 5565585 | 5569532 | estExt_Genewise1.C_750066 | scaffold_75 | 84235 | 87601 |
| Block 2819 | DDB_G0282247 | 3 | 5570015 | 5571588 | estExt_Genewise1.C_750067 | scaffold_75 | 88192 | 90228 |
| Block 2819 | tupA | 3 | 5574241 | 5576373 | fgeneshDP_pg.C_scaffold_75000034 | scaffold_75 | 92093 | 94048 |
| Block 1008 | ech1 | 3 | 5585355 | 5586328 | GID1.0045821 | scaffold_232 | 41735 | 42695 |
| Block 1008 | DDB_G0282263 | 3 | 5586895 | 5589681 | e_gw1.232.15.1 | scaffold_232 | 43062 | 46129 |
| Block 1008 | sgmC | 3 | 5590476 | 5592144 | e_gw1.232.20.1 | scaffold_232 | 46897 | 48378 |
| Block 250 | gxcV | 3 | 5595367 | 5597368 | e_gw1.126.20.1 | scaffold_126 | 54242 | 56004 |
| Block 250 | DDB_G0282275 | 3 | 5598918 | 5599803 | GID1.0043142 | scaffold_126 | 57597 | 58454 |
| Block 250 | DDB_G0282351 | 3 | 5602431 | 5603475 | estExt_fgeneshDP_pg.C_1260021 | scaffold_126 | 59366 | 61124 |
| Block 250 | rnpA | 3 | 5604394 | 5606049 | fgeneshDP_pg.C_scaffold_126000022 | scaffold_126 | 61502 | 63497 |
| Block 250 | DDB_G0282299 | 3 | 5607244 | 5609831 | GID1.0043146 | scaffold_126 | 64833 | 67400 |
| Block 250 | DDB_G0282301 | 3 | 5610071 | 5610561 | fgeneshDP_pm.C_scaffold_126000014 | scaffold_126 | 67585 | 67977 |
| Block 250 | DDB_G0282303 | 3 | 5610805 | 5613253 | GID1.0043148 | scaffold_126 | 68204 | 70538 |
| Block 250 | DDB_G0282305 | 3 | 5613858 | 5616404 | e_gw1.126.21.1 | scaffold_126 | 71867 | 73843 |
| Block 250 | DDB_G0282313 | 3 | 5624970 | 5626340 | estExt_Genewise1.C_1260051 | scaffold_126 | 51308 | 52813 |
| Block 250 | dhkD | 3 | 5632424 | 5637271 | GID1.0043139 | scaffold_126 | 46602 | 50948 |
| Block 250 | forB | 3 | 5641699 | 5645174 | e_gw1.126.11.1 | scaffold_126 | 40837 | 44226 |
| Block 1594 | rpa5 | 3 | 5656917 | 5658092 | fgeneshDP_pm.C_scaffold_345000002 | scaffold_345 | 3779 | 5000 |
| Block 1594 | DDB_G0282341 | 3 | 5658262 | 5659241 | GID1.0047839 | scaffold_345 | 5211 | 6108 |
| Block 1594 | DDB_G0282343 | 3 | 5660192 | 5660841 | fgeneshDP_pm.C_scaffold_345000003 | scaffold_345 | 6996 | 7622 |
| Block 1594 | potA | 3 | 5660926 | 5663954 | e_gw1.345.1.1 | scaffold_345 | 7647 | 10551 |
| Block 2060 | DDB_G0282393 | 3 | 5695519 | 5696365 | fgeneshDP_pm.C_scaffold_457000001 | scaffold_457 | 847 | 1700 |
| Block 2060 | gacQ | 3 | 5696610 | 5698400 | GID1.0049232 | scaffold_457 | 1938 | 3561 |
| Block 2060 | mbtps1 | 3 | 5699822 | 5703817 | e_gw1.457.2.1 | scaffold_457 | 6054 | 9271 |
| Block 1211 | DDB_G0282409 | 3 | 5714264 | 5717976 | estExt_fgeneshDP_pg.C_2700011 | scaffold_270 | 35685 | 39162 |
| Block 1211 | DDB_G0282411 | 3 | 5721347 | 5723844 | estExt_Genewise1Plus.C_2700027 | scaffold_270 | 42435 | 45531 |
| Block 1001 | dhkM | 3 | 5726442 | 5733682 | GID1.0045808 | scaffold_232 | 870 | 6894 |
| Block 1001 | DDB_G0282415 | 3 | 5735049 | 5735769 | fgeneshDP_pg.C_scaffold_232000002 | scaffold_232 | 7928 | 8592 |
| Block 2060 | selD | 3 | 5741356 | 5742554 | fgeneshDP_pg.C_scaffold_457000008 | scaffold_457 | 17575 | 18567 |
| Block 873 | DDB_G0282427 | 3 | 5755702 | 5758268 | gw1.211.8.1 | scaffold_211 | 38406 | 40363 |
| Block 873 | DDB_G0282429 | 3 | 5758659 | 5759806 | estExt_Genewise1.C_2110017 | scaffold_211 | 36864 | 38184 |
| Block 873 | DDB_G0282757 | 3 | 5762312 | 5762476 | GID1.0045358 | scaffold_211 | 34766 | 34930 |
| Block 1801 | DDB_G0282431 | 3 | 5763247 | 5764563 | e_gw1.394.4.1 | scaffold_394 | 27698 | 28963 |
| Block 1801 | DDB_G0282433 | 3 | 5764640 | 5765343 | gw1.394.11.1 | scaffold_394 | 26936 | 27455 |
| Block 252 | DDB_G0282451 | 3 | 5774365 | 5774968 | gw1.127.31.1 | scaffold_127 | 17195 | 17724 |
| Block 252 | med1 | 3 | 5777523 | 5779929 | estExt_fgeneshDP_pg.C_1270008 | scaffold_127 | 21973 | 24118 |
| Block 2788 | gnt13 | 3 | 5807533 | 5809539 | estExt_Genewise1.C_7340004 | scaffold_734 | 4083 | 5430 |
| Block 2788 | ints9 | 3 | 5815575 | 5817857 | GID1.0050877 | scaffold_734 | 1533 | 3843 |
| Block 686 | DDB_G0282477 | 3 | 5822316 | 5822710 | e_gw1.19.177.1 | scaffold_19 | 8047 | 8372 |
| Block 686 | spf27 | 3 | 5822824 | 5823706 | fgeneshDP_pm.C_scaffold_19000003 | scaffold_19 | 5910 | 6763 |
| Block 686 | DDB_G0282479 | 3 | 5826574 | 5827634 | fgeneshDP_pg.C_scaffold_19000001 | scaffold_19 | 82 | 782 |
| Block 2676 | DDB_G0282481 | 3 | 5828096 | 5828706 | fgeneshDP_pg.C_scaffold_666000004 | scaffold_666 | 8470 | 9021 |
| Block 2676 | DDB_G0282483 | 3 | 5829856 | 5831090 | estExt_fgeneshDP_kg.C_6660001 | scaffold_666 | 6504 | 7682 |
| Block 1453 | DDB_G0282765 | 3 | 5832783 | 5833825 | GID1.0047370 | scaffold_315 | 8201 | 9162 |
| Block 1453 | mhkD | 3 | 5835955 | 5838780 | estExt_fgeneshDP_pm.C_3150002 | scaffold_315 | 5152 | 8190 |
| Block 1453 | DDB_G0282491 | 3 | 5839851 | 5843352 | estExt_fgeneshDP_pm.C_3150001 | scaffold_315 | 1730 | 4182 |
| Block 2579 | aatB | 3 | 5845721 | 5847037 | estExt_fgeneshDP_kg.C_6260001 | scaffold_626 | 8271 | 9662 |
| Block 2579 | DDB_G0282497 | 3 | 5849588 | 5850358 | GID1.0050453 | scaffold_626 | 5671 | 6469 |
| Block 450 | DDB_G0282515 | 3 | 5873962 | 5876256 | gw1.150.26.1 | scaffold_150 | 29130 | 31091 |
| Block 450 | DDB_G0282517 | 3 | 5877016 | 5878111 | fgeneshDP_pg.C_scaffold_150000026 | scaffold_150 | 64369 | 65775 |
| Block 638 | DDB_G0282525 | 3 | 5886297 | 5887636 | fgeneshDP_pg.C_scaffold_18000042 | scaffold_18 | 105199 | 106561 |
| Block 638 | pemtA | 3 | 5888056 | 5888814 | fgeneshDP_pg.C_scaffold_18000044 | scaffold_18 | 109181 | 109886 |
| Block 638 | rbbD | 3 | 5888997 | 5890360 | estExt_Genewise1.C_180062 | scaffold_18 | 109907 | 111548 |
| Block 2084 | DDB_G0282531 | 3 | 5890637 | 5891733 | e_gw1.462.8.1 | scaffold_462 | 2534 | 3618 |
| Block 1624 | DDB_G0282781 | 3 | 5914083 | 5916077 | fgeneshDP_pg.C_scaffold_351000015 | scaffold_351 | 31760 | 34472 |
| Block 1624 | DDB_G0282549 | 3 | 5917145 | 5918251 | GID1.0047944 | scaffold_351 | 30532 | 31499 |
| Block 2084 | DDB_G0282783 | 3 | 5921147 | 5930200 | estExt_fgeneshDP_pg.C_4620004 | scaffold_462 | 7528 | 16060 |
| Block 638 | DDB_G0282563 | 3 | 5941283 | 5943136 | estExt_fgeneshDP_pg.C_180057 | scaffold_18 | 142489 | 144451 |
| Block 2516 | DDB_G0282573 | 3 | 5953713 | 5955298 | GID1.0040681 | scaffold_60 | 46383 | 47881 |
| Block 2516 | DDB_G0282575 | 3 | 5957238 | 5960822 | fgeneshDP_pm.C_scaffold_60000007 | scaffold_60 | 27522 | 30887 |
| Block 2516 | pldZ | 3 | 5966744 | 5968161 | e_gw1.60.40.1 | scaffold_60 | 20296 | 21773 |
| Block 2516 | wdr45l | 3 | 5969861 | 5971133 | estExt_Genewise1Plus.C_600010 | scaffold_60 | 22698 | 24262 |
| Block 2516 | DDB_G0282583 | 3 | 5972121 | 5972733 | estExt_fgeneshDP_pm.C_600006 | scaffold_60 | 24500 | 25861 |
| Block 882 | DDB_G0282587 | 3 | 5977758 | 5978198 | gw1.213.23.1 | scaffold_213 | 6363 | 6692 |
| Block 882 | DDB_G0282593 | 3 | 5983138 | 5984226 | e_gw1.213.8.1 | scaffold_213 | 1724 | 2539 |
| Block 882 | gmfA | 3 | 5984604 | 5985197 | fgeneshDP_pm.C_scaffold_213000001 | scaffold_213 | 390 | 1004 |
| Block 1149 | DDB_G0282597 | 3 | 5985289 | 5989281 | estExt_fgeneshDP_pg.C_2590009 | scaffold_259 | 13565 | 16869 |
| Block 1149 | DDB_G0282599 | 3 | 5989441 | 5990577 | fgeneshDP_pg.C_scaffold_259000006 | scaffold_259 | 8877 | 9965 |
| Block 1149 | rps11 | 3 | 5991308 | 5992503 | estExt_fgeneshDP_pm.C_2590004 | scaffold_259 | 7052 | 8197 |
| Block 1149 | DDB_G0282603 | 3 | 5993046 | 5993891 | fgeneshDP_pg.C_scaffold_259000004 | scaffold_259 | 6100 | 6852 |
| Block 1149 | DDB_G0282605 | 3 | 5993980 | 5995716 | estExt_Genewise1.C_2590004 | scaffold_259 | 4060 | 6053 |
| Block 1149 | crsA | 3 | 5996864 | 5998409 | estExt_fgeneshDP_pg.C_2590002 | scaffold_259 | 1683 | 3023 |
| Block 2871 | midA | 3 | 6003476 | 6005010 | GID1.0051016 | scaffold_790 | 547 | 2074 |
| Block 2871 | psmF1 | 3 | 6005174 | 6006260 | GID1.0051017 | scaffold_790 | 2182 | 3251 |
| Block 546 | pikG | 3 | 6013022 | 6018337 | e_gw1.166.21.1 | scaffold_166 | 23244 | 28057 |
| Block 546 | vps15 | 3 | 6019111 | 6025130 | GID1.0044276 | scaffold_166 | 16935 | 22454 |
| Block 1787 | DDB_G0294577 | 3 | 6055905 | 6057036 | gw1.390.13.1 | scaffold_390 | 24717 | 25055 |
| Block 1787 | rpl28 | 3 | 6061184 | 6061582 | estExt_fgeneshDP_kg.C_3900001 | scaffold_390 | 23813 | 24274 |
| Block 1787 | DDB_G0282655 | 3 | 6063029 | 6063729 | e_gw1.390.14.1 | scaffold_390 | 22251 | 22907 |
| Block 1787 | wdr61 | 3 | 6064156 | 6065672 | fgeneshDP_pg.C_scaffold_390000008 | scaffold_390 | 20385 | 21738 |
| Block 2343 | DDB_G0282801 | 3 | 6081891 | 6091580 | fgeneshDP_pg.C_scaffold_536000002 | scaffold_536 | 3771 | 12884 |
| Block 2343 | DDB_G0282661 | 3 | 6092418 | 6092875 | GID1.0049914 | scaffold_536 | 2064 | 2993 |
| Block 546 | DDB_G0282667 | 3 | 6096363 | 6098133 | fgeneshDP_pg.C_scaffold_166000018 | scaffold_166 | 62379 | 63885 |
| Block 546 | spt16 | 3 | 6105920 | 6109250 | estExt_Genewise1Plus.C_1660035 | scaffold_166 | 49357 | 52821 |
| Block 546 | DDB_G0282683 | 3 | 6123377 | 6123829 | estExt_fgeneshDP_kg.C_1660002 | scaffold_166 | 47932 | 48651 |
| Block 546 | rcl1 | 3 | 6124057 | 6125277 | fgeneshDP_pg.C_scaffold_166000014 | scaffold_166 | 46713 | 47859 |
| Block 2023 | DDB_G0282685 | 3 | 6125852 | 6127432 | e_gw1.447.6.1 | scaffold_447 | 12931 | 14401 |
| Block 2718 | DDB_G0282689 | 3 | 6128420 | 6131150 | GID1.0050730 | scaffold_693 | 6836 | 8007 |
| Block 2023 | pksB | 3 | 6131868 | 6132842 | estExt_fgeneshDP_kg.C_4470001 | scaffold_447 | 10825 | 11874 |
| Block 2718 | pex10 | 3 | 6137089 | 6138534 | e_gw1.693.6.1 | scaffold_693 | 2110 | 3081 |
| Block 2718 | DDB_G0282695 | 3 | 6139156 | 6141114 | GID1.0050726 | scaffold_693 | 67 | 1431 |
| Block 2023 | amiB | 3 | 6170766 | 6179193 | GID1.0049131 | scaffold_447 | 14636 | 21581 |
| Block 2343 | DDB_G0282711 | 3 | 6179495 | 6183789 | estExt_fgeneshDP_pg.C_5360003 | scaffold_536 | 13106 | 15899 |
| Block 546 | DDB_G0282713 | 3 | 6189456 | 6191092 | gw1.166.34.1 | scaffold_166 | 44830 | 46217 |
| Block 546 | DDB_G0282715 | 3 | 6191274 | 6194070 | estExt_Genewise1Plus.C_1660022 | scaffold_166 | 42099 | 44834 |
| Block 546 | gxcAA | 3 | 6195317 | 6198952 | GID1.0044280 | scaffold_166 | 38380 | 41468 |
| Block 2718 | tmem104 | 3 | 6203669 | 6205412 | GID1.0050729 | scaffold_693 | 4736 | 6438 |
| Block 2023 | vilD | 3 | 6207123 | 6212648 | estExt_Genewise1.C_4470004 | scaffold_447 | 5573 | 10824 |
| Block 2023 | mcfU | 3 | 6213878 | 6215173 | fgeneshDP_pm.C_scaffold_447000001 | scaffold_447 | 4048 | 5141 |
| Block 2023 | DDB_G0282729 | 3 | 6215725 | 6219713 | GID1.0049126 | scaffold_447 | 143 | 3411 |
| Block 2211 | polA2 | 3 | 6220731 | 6222999 | estExt_fgeneshDP_pg.C_50013 | scaffold_5 | 38555 | 40616 |
| Block 2211 | rpb4 | 3 | 6226742 | 6227440 | fgeneshDP_pm.C_scaffold_5000005 | scaffold_5 | 40956 | 41586 |
| Block 2305 | ddx18 | 3 | 6230156 | 6232041 | GID1.0049836 | scaffold_525 | 9677 | 11565 |
| Block 2305 | haao | 3 | 6232657 | 6233325 | estExt_Genewise1Plus.C_5250014 | scaffold_525 | 12172 | 12851 |
| Block 2305 | DDB_G0282745 | 3 | 6234797 | 6237354 | GID1.0049839 | scaffold_525 | 14657 | 16717 |
| Block 1785 | DDB_G0282747 | 3 | 6239552 | 6240857 | fgeneshDP_pm.C_scaffold_390000001 | scaffold_390 | 1331 | 2512 |
| Block 1785 | bzpG | 3 | 6241172 | 6242290 | e_gw1.390.5.1 | scaffold_390 | 5432 | 6499 |
| Block 1785 | gefC | 3 | 6243320 | 6247805 | GID1.0048488 | scaffold_390 | 7680 | 11968 |
| Block 1785 | DDB_G0282895 | 3 | 6249781 | 6254932 | estExt_fgeneshDP_pg.C_3900006 | scaffold_390 | 13896 | 18665 |
| Block 2305 | DDB_G0282823 | 3 | 6262357 | 6265680 | fgeneshDP_pg.C_scaffold_525000005 | scaffold_525 | 6523 | 9468 |
| Block 2305 | yipf6 | 3 | 6266209 | 6267258 | e_gw1.525.11.1 | scaffold_525 | 2899 | 3898 |
| Block 2305 | arcB | 3 | 6271490 | 6272740 | GID1.0049833 | scaffold_525 | 953 | 1716 |
| Block 2305 | DDB_G0282831 | 3 | 6273125 | 6273518 | GID1.0049832 | scaffold_525 | 295 | 704 |
| Block 634 | DDB_G0282845 | 3 | 6289243 | 6290713 | estExt_Genewise1.C_180016 | scaffold_18 | 26173 | 28130 |
| Block 634 | rab5B | 3 | 6292039 | 6292775 | fgeneshDP_pm.C_scaffold_18000007 | scaffold_18 | 29092 | 29929 |
| Block 634 | DDB_G0282849 | 3 | 6293427 | 6296760 | estExt_fgeneshDP_pg.C_180015 | scaffold_18 | 31603 | 34017 |
| Block 634 | DDB_G0282853 | 3 | 6303739 | 6304648 | estExt_Genewise1Plus.C_180032 | scaffold_18 | 50206 | 51158 |
| Block 634 | DDB_G0282855 | 3 | 6304937 | 6307397 | GID1.0038505 | scaffold_18 | 51417 | 52718 |
| Block 634 | patB | 3 | 6309404 | 6312689 | GID1.0038506 | scaffold_18 | 55970 | 59098 |
| Block 634 | DDB_G0282859 | 3 | 6316518 | 6318407 | fgeneshDP_pg.C_scaffold_18000026 | scaffold_18 | 59424 | 60923 |
| Block 1384 | snrpG | 3 | 6319736 | 6320163 | e_gw1.301.32.1 | scaffold_301 | 6176 | 6764 |
| Block 634 | DDB_G0282879 | 3 | 6331837 | 6332697 | e_gw1.18.51.1 | scaffold_18 | 45984 | 47025 |
| Block 634 | DDB_G0282881 | 3 | 6333061 | 6334356 | estExt_fgeneshDP_kg.C_180002 | scaffold_18 | 44100 | 45533 |
| Block 634 | DDB_G0282901 | 3 | 6334464 | 6335236 | fgeneshDP_pm.C_scaffold_18000010 | scaffold_18 | 43283 | 44076 |
| Block 634 | sglA | 3 | 6335773 | 6337539 | fgeneshDP_pg.C_scaffold_18000017 | scaffold_18 | 39128 | 40893 |
| Block 1384 | trappc2 | 3 | 6341041 | 6341809 | estExt_Genewise1.C_3010006 | scaffold_301 | 4975 | 5820 |
| Block 2123 | DDB_G0282903 | 3 | 6342424 | 6344481 | e_gw1.473.15.1 | scaffold_473 | 4740 | 5277 |
| Block 2123 | DDB_G0282903 | 3 | 6342424 | 6344481 | e_gw1.473.5.1 | scaffold_473 | 3073 | 4671 |
| Block 634 | pakG | 3 | 6346144 | 6349794 | GID1.0038491 | scaffold_18 | 21456 | 24735 |
| Block 193 | abcB6 | 4 | 9046 | 11927 | GID1.0042921 | scaffold_120 | 7784 | 10075 |
| Block 193 | mcm7 | 4 | 12867 | 15427 | GID1.0042923 | scaffold_120 | 13335 | 15947 |
| Block 193 | DDB_G0282935 | 4 | 17607 | 18612 | fgeneshDP_pg.C_scaffold_120000007 | scaffold_120 | 12274 | 13244 |
| Block 193 | DDB_G0282937 | 4 | 19492 | 20019 | e_gw1.120.55.1 | scaffold_120 | 10826 | 11208 |
| Block 2480 | DDB_G0283055 | 4 | 20488 | 22100 | GID1.0040622 | scaffold_59 | 18854 | 20358 |
| Block 2480 | DDB_G0282939 | 4 | 22195 | 23766 | GID1.0040623 | scaffold_59 | 20494 | 21840 |
| Block 2480 | DDB_G0282941 | 4 | 24418 | 26871 | estExt_Genewise1Plus.C_590022 | scaffold_59 | 22693 | 24930 |
| Block 2480 | DDB_G0282943 | 4 | 28010 | 31415 | estExt_fgeneshDP_pm.C_590008 | scaffold_59 | 26746 | 30295 |
| Block 1940 | DDB_G0282945 | 4 | 31559 | 33775 | e_gw1.424.18.1 | scaffold_424 | 20330 | 22266 |
| Block 1940 | DDB_G0283057 | 4 | 34226 | 37566 | fgeneshDP_pg.C_scaffold_424000006 | scaffold_424 | 16671 | 19517 |
| Block 1940 | DDB_G0282947 | 4 | 38803 | 42072 | estExt_fgeneshDP_pg.C_4240004 | scaffold_424 | 10194 | 13138 |
| Block 1940 | DDB_G0282949 | 4 | 42516 | 44512 | estExt_Genewise1.C_4240005 | scaffold_424 | 7558 | 9391 |
| Block 2844 | DDB_G0282951 | 4 | 45329 | 45825 | GID1.0041422 | scaffold_78 | 12248 | 12817 |
| Block 2844 | DDB_G0282953 | 4 | 45934 | 47981 | GID1.0041423 | scaffold_78 | 12945 | 14873 |
| Block 2844 | DDB_G0282955 | 4 | 49492 | 50609 | e_gw1.78.45.1 | scaffold_78 | 15104 | 16158 |
| Block 2514 | DDB_G0282957 | 4 | 50957 | 51163 | GID1.0037688 | scaffold_6 | 99657 | 99866 |
| Block 2514 | DDB_G0282959 | 4 | 52235 | 56945 | estExt_fgeneshDP_pm.C_60027 | scaffold_6 | 156678 | 160730 |
| Block 1551 | DDB_G0282961 | 4 | 57054 | 58860 | GID1.0047739 | scaffold_338 | 10889 | 12605 |
| Block 1551 | DDB_G0282963 | 4 | 60445 | 65808 | fgeneshDP_pg.C_scaffold_338000004 | scaffold_338 | 14995 | 19544 |
| Block 1551 | rdeA | 4 | 67134 | 67898 | estExt_fgeneshDP_kg.C_3380001 | scaffold_338 | 19787 | 20934 |
| Block 1551 | DDB_G0282965 | 4 | 68065 | 69168 | e_gw1.338.12.1 | scaffold_338 | 21936 | 23255 |
| Block 2514 | DDB_G0283061 | 4 | 69391 | 70422 | e_gw1.6.106.1 | scaffold_6 | 165578 | 166549 |
| Block 2514 | acadsb | 4 | 70875 | 72116 | estExt_Genewise1.C_60120 | scaffold_6 | 163815 | 165296 |
| Block 2514 | masB | 4 | 72206 | 73834 | e_gw1.6.81.1 | scaffold_6 | 181966 | 183636 |
| Block 2514 | DDB_G0282971 | 4 | 74287 | 76772 | estExt_Genewise1Plus.C_60126 | scaffold_6 | 178560 | 180972 |
| Block 2514 | DDB_G0282977 | 4 | 81934 | 85011 | fgeneshDP_pg.C_scaffold_6000062 | scaffold_6 | 166788 | 169613 |
| Block 1951 | DDB_G0282995 | 4 | 113431 | 114507 | estExt_fgeneshDP_pm.C_4280005 | scaffold_428 | 21946 | 22917 |
| Block 1951 | DDB_G0283063 | 4 | 115315 | 116295 | GID1.0048955 | scaffold_428 | 23517 | 24410 |
| Block 1600 | rad54 | 4 | 118731 | 121607 | estExt_Genewise1.C_3460001 | scaffold_346 | 2640 | 5681 |
| Block 1600 | DDB_G0283065 | 4 | 122741 | 124654 | fgeneshDP_pg.C_scaffold_346000002 | scaffold_346 | 5723 | 7456 |
| Block 192 | DDB_G0283067 | 4 | 138075 | 139277 | estExt_Genewise1.C_1200001 | scaffold_120 | 146 | 1419 |
| Block 192 | DDB_G0283001 | 4 | 139924 | 140950 | GID1.0042918 | scaffold_120 | 1755 | 2495 |
| Block 192 | DDB_G0283003 | 4 | 141272 | 141856 | estExt_Genewise1Plus.C_1200003 | scaffold_120 | 2906 | 3608 |
| Block 192 | DDB_G0283049 | 4 | 142399 | 143635 | fgeneshDP_pg.C_scaffold_120000004 | scaffold_120 | 4453 | 5578 |
| Block 192 | agtA | 4 | 143819 | 145984 | GID1.0042920 | scaffold_120 | 5654 | 7626 |
| Block 2512 | DDB_G0283007 | 4 | 147313 | 148900 | estExt_fgeneshDP_kg.C_60011 | scaffold_6 | 80488 | 82400 |
| Block 2512 | mcm8 | 4 | 149067 | 151575 | GID1.0037682 | scaffold_6 | 82549 | 85057 |
| Block 2512 | DDB_G0283011 | 4 | 151994 | 152491 | GID1.0037683 | scaffold_6 | 85666 | 86081 |
| Block 2512 | DDB_G0283013 | 4 | 153182 | 160521 | e_gw1.6.50.1 | scaffold_6 | 86471 | 93459 |
| Block 2512 | atg12 | 4 | 160759 | 161341 | fgeneshDP_pg.C_scaffold_6000039 | scaffold_6 | 93597 | 94143 |
| Block 2263 | DDB_G0283017 | 4 | 164286 | 166961 | estExt_fgeneshDP_pg.C_510042 | scaffold_51 | 105828 | 108334 |
| Block 2263 | DDB_G0283019 | 4 | 167136 | 167543 | fgeneshDP_pg.C_scaffold_51000043 | scaffold_51 | 109575 | 110030 |
| Block 2845 | DDB_G0283033 | 4 | 178285 | 180683 | estExt_Genewise1.C_780013 | scaffold_78 | 19450 | 22256 |
| Block 2845 | DDB_G0283071 | 4 | 181322 | 181939 | fgeneshDP_pg.C_scaffold_78000010 | scaffold_78 | 18092 | 18730 |
| Block 2845 | orcA | 4 | 182130 | 184025 | fgeneshDP_pg.C_scaffold_78000009 | scaffold_78 | 16313 | 17944 |
| Block 2512 | esd | 4 | 192516 | 193525 | fgeneshDP_pg.C_scaffold_6000040 | scaffold_6 | 94831 | 95825 |
| Block 2512 | DDB_G0283039 | 4 | 196663 | 198359 | GID1.0037695 | scaffold_6 | 120531 | 121895 |
| Block 2512 | DDB_G0283041 | 4 | 198610 | 199358 | fgeneshDP_pg.C_scaffold_6000032 | scaffold_6 | 74178 | 74877 |
| Block 2512 | DDB_G0283087 | 4 | 203029 | 204917 | GID1.0037675 | scaffold_6 | 64691 | 66579 |
| Block 2512 | DD3-3 | 4 | 210212 | 212062 | estExt_Genewise1Plus.C_60060 | scaffold_6 | 76133 | 78723 |
| Block 2476 | DDB_G0283099 | 4 | 217095 | 223472 | GID1.0050252 | scaffold_589 | 5082 | 11345 |
| Block 2476 | DDB_G0283135 | 4 | 223712 | 224049 | GID1.0050251 | scaffold_589 | 4129 | 4928 |
| Block 2512 | pikB | 4 | 247398 | 252971 | estExt_Genewise1Plus.C_60077 | scaffold_6 | 107858 | 113294 |
| Block 2512 | DDB_G0283107 | 4 | 255521 | 256804 | fgeneshDP_pm.C_scaffold_6000021 | scaffold_6 | 104272 | 105604 |
| Block 2512 | iksA | 4 | 257953 | 261035 | GID1.0037689 | scaffold_6 | 100261 | 102910 |
| Block 959 | DDB_G0283117 | 4 | 267048 | 268356 | fgeneshDP_pg.C_scaffold_226000017 | scaffold_226 | 31317 | 32460 |
| Block 959 | xpot | 4 | 269911 | 273800 | GID1.0045702 | scaffold_226 | 33548 | 37106 |
| Block 959 | DDB_G0283121 | 4 | 275079 | 276215 | GID1.0045696 | scaffold_226 | 17456 | 18480 |
| Block 2926 | D2 | 4 | 276713 | 278420 | GID1.0041702 | scaffold_84 | 76124 | 77877 |
| Block 2926 | DDB_G0283125 | 4 | 281753 | 283546 | estExt_Genewise1Plus.C_840057 | scaffold_84 | 78356 | 80187 |
| Block 959 | DDB_G0283127 | 4 | 283814 | 285345 | e_gw1.226.21.1 | scaffold_226 | 15629 | 17311 |
| Block 1262 | DDB_G0283157 | 4 | 299093 | 300864 | estExt_Genewise1.C_280082 | scaffold_28 | 99593 | 101504 |
| Block 1262 | DDB_G0283159 | 4 | 301718 | 303846 | estExt_fgeneshDP_pg.C_280044 | scaffold_28 | 102053 | 104277 |
| Block 1262 | DDB_G0283161 | 4 | 303938 | 304342 | estExt_Genewise1.C_280091 | scaffold_28 | 111534 | 112454 |
| Block 1584 | pyk | 4 | 344914 | 346711 | estExt_Genewise1.C_3420018 | scaffold_342 | 17180 | 19701 |
| Block 1584 | eIF2b5 | 4 | 349612 | 351949 | e_gw1.342.6.1 | scaffold_342 | 13267 | 15453 |
| Block 618 | DDB_G0283171 | 4 | 358763 | 359168 | GID1.0044558 | scaffold_177 | 27631 | 28141 |
| Block 618 | kmo | 4 | 359274 | 360656 | estExt_Genewise1.C_1770019 | scaffold_177 | 26026 | 27537 |
| Block 618 | prfA | 4 | 360952 | 362348 | estExt_fgeneshDP_kg.C_1770003 | scaffold_177 | 24204 | 24908 |
| Block 618 | DDB_G0283177 | 4 | 362729 | 364548 | GID1.0044555 | scaffold_177 | 22271 | 24051 |
| Block 284 | DDB_G0283211_ps | 4 | 368141 | 369137 | e_gw1.130.60.1 | scaffold_130 | 18319 | 18519 |
| Block 284 | DDB_G0283211_ps | 4 | 368141 | 369137 | gw1.130.52.1 | scaffold_130 | 18700 | 19377 |
| Block 618 | DDB_G0283251 | 4 | 377937 | 380372 | e_gw1.177.20.1 | scaffold_177 | 19694 | 21992 |
| Block 618 | DDB_G0283187 | 4 | 381819 | 383085 | e_gw1.177.33.1 | scaffold_177 | 14423 | 17544 |
| Block 618 | DDB_G0283253 | 4 | 383852 | 384293 | estExt_fgeneshDP_pg.C_1770003 | scaffold_177 | 13449 | 14066 |
| Block 618 | DDB_G0283189 | 4 | 385928 | 393596 | estExt_fgeneshDP_pm.C_1770002 | scaffold_177 | 5186 | 12644 |
| Block 1950 | vps22 | 4 | 415279 | 416235 | e_gw1.428.9.1 | scaffold_428 | 19681 | 20654 |
| Block 1950 | top1 | 4 | 416900 | 419809 | estExt_Genewise1.C_4280009 | scaffold_428 | 15955 | 18620 |
| Block 2479 | DDB_G0283269 | 4 | 428924 | 430693 | GID1.0040634 | scaffold_59 | 40553 | 42315 |
| Block 2479 | eppA | 4 | 431083 | 432443 | estExt_fgeneshDP_pg.C_590019 | scaffold_59 | 42517 | 43888 |
| Block 2479 | mcfO | 4 | 432962 | 435476 | GID1.0040636 | scaffold_59 | 44281 | 46803 |
| Block 2479 | DDB_G0283271 | 4 | 436406 | 437655 | estExt_fgeneshDP_pg.C_590021 | scaffold_59 | 47523 | 49269 |
| Block 2513 | shkA | 4 | 438511 | 441126 | estExt_fgeneshDP_pg.C_60040 | scaffold_6 | 96372 | 98642 |
| Block 2479 | DDB_G0283273 | 4 | 441777 | 442542 | e_gw1.59.22.1 | scaffold_59 | 38293 | 39238 |
| Block 2479 | DDB_G0283275 | 4 | 442808 | 443794 | GID1.0040633 | scaffold_59 | 39471 | 40409 |
| Block 2513 | cdk11 | 4 | 446640 | 447716 | fgeneshDP_pg.C_scaffold_6000046 | scaffold_6 | 116250 | 117269 |
| Block 200 | DDB_G0283281 | 4 | 447879 | 449138 | GID1.0042950 | scaffold_120 | 61534 | 62852 |
| Block 200 | acoA | 4 | 449907 | 452098 | fgeneshDP_pg.C_scaffold_120000029 | scaffold_120 | 63274 | 65369 |
| Block 200 | migA | 4 | 453096 | 455785 | gw1.120.26.1 | scaffold_120 | 71314 | 73182 |
| Block 200 | atp5O | 4 | 456147 | 457641 | estExt_fgeneshDP_pg.C_1200032 | scaffold_120 | 73334 | 75247 |
| Block 1550 | DDB_G0283285 | 4 | 458537 | 462904 | estExt_fgeneshDP_pm.C_3380001 | scaffold_338 | 3263 | 4817 |
| Block 1550 | fut7 | 4 | 463265 | 464749 | GID1.0047734 | scaffold_338 | 4794 | 6158 |
| Block 2479 | DDB_G0283291 | 4 | 467526 | 468617 | e_gw1.59.57.1 | scaffold_59 | 8018 | 9200 |
| Block 40 | ucpB | 4 | 469001 | 470093 | e_gw1.101.24.1 | scaffold_101 | 43155 | 45284 |
| Block 40 | DDB_G0283293 | 4 | 472326 | 474925 | estExt_Genewise1.C_1010019 | scaffold_101 | 40239 | 42700 |
| Block 2479 | DDB_G0283295 | 4 | 476937 | 477164 | GID1.0040617 | scaffold_59 | 7616 | 7843 |
| Block 2513 | DDB_G0283305 | 4 | 498765 | 500435 | GID1.0037697 | scaffold_6 | 123434 | 125229 |
| Block 2513 | DDB_G0283339 | 4 | 501258 | 507721 | GID1.0037698 | scaffold_6 | 125916 | 131512 |
| Block 2513 | prpf39 | 4 | 508620 | 510972 | e_gw1.6.66.1 | scaffold_6 | 143461 | 145495 |
| Block 2513 | DDB_G0283309 | 4 | 511242 | 512282 | fgeneshDP_pm.C_scaffold_6000025 | scaffold_6 | 122330 | 123367 |
| Block 2853 | DDB_G0283311 | 4 | 512897 | 513361 | fgeneshDP_pg.C_scaffold_78000029 | scaffold_78 | 65602 | 66030 |
| Block 2853 | dcp2 | 4 | 515744 | 517800 | estExt_Genewise1.C_780037 | scaffold_78 | 61930 | 63636 |
| Block 2337 | comD | 4 | 539820 | 543347 | estExt_Genewise1Plus.C_5340003 | scaffold_534 | 846 | 4091 |
| Block 2479 | DDB_G0283347 | 4 | 543684 | 545458 | e_gw1.59.43.1 | scaffold_59 | 13347 | 14906 |
| Block 2479 | gpaA | 4 | 547836 | 549106 | estExt_Genewise1Plus.C_590009 | scaffold_59 | 9496 | 12112 |
| Block 2513 | DDB_G0283373 | 4 | 556576 | 558318 | fgeneshDP_pg.C_scaffold_6000045 | scaffold_6 | 114248 | 116106 |
| Block 2337 | DDB_G0283355 | 4 | 559359 | 563272 | e_gw1.534.1.1 | scaffold_534 | 5015 | 8693 |
| Block 807 | eftud2 | 4 | 569125 | 572263 | GID1.0045165 | scaffold_202 | 46347 | 49424 |
| Block 807 | DDB_G0283361 | 4 | 572566 | 574122 | fgeneshDP_pg.C_scaffold_202000021 | scaffold_202 | 49779 | 51263 |
| Block 1404 | scdA | 4 | 574408 | 576993 | estExt_fgeneshDP_kg.C_3040002 | scaffold_304 | 6438 | 7320 |
| Block 1404 | DDB_G0283377 | 4 | 578428 | 579892 | GID1.0047181 | scaffold_304 | 8315 | 9643 |
| Block 1404 | vti1B | 4 | 584359 | 585348 | GID1.0047182 | scaffold_304 | 10073 | 11168 |
| Block 39 | rpb12 | 4 | 585874 | 586210 | e_gw1.101.39.1 | scaffold_101 | 31584 | 31871 |
| Block 39 | DDB_G0283369 | 4 | 587429 | 594000 | GID1.0042288 | scaffold_101 | 32188 | 38164 |
| Block 39 | DDB_G0283381 | 4 | 594665 | 600480 | GID1.0042293 | scaffold_101 | 45556 | 50794 |
| Block 807 | udkC | 4 | 600577 | 602174 | fgeneshDP_pg.C_scaffold_202000019 | scaffold_202 | 44673 | 46133 |
| Block 2107 | DDB_G0283399 | 4 | 609993 | 610496 | e_gw1.47.71.1 | scaffold_47 | 47810 | 48343 |
| Block 39 | DDB_G0283495 | 4 | 611003 | 613564 | estExt_fgeneshDP_pm.C_1010008 | scaffold_101 | 65678 | 67925 |
| Block 2107 | DDB_G0283403 | 4 | 615804 | 618044 | e_gw1.47.41.1 | scaffold_47 | 53095 | 55347 |
| Block 2107 | bdp1 | 4 | 618312 | 619784 | fgeneshDP_pg.C_scaffold_47000025 | scaffold_47 | 55554 | 56854 |
| Block 2107 | splA | 4 | 623097 | 631896 | estExt_Genewise1Plus.C_470034 | scaffold_47 | 61102 | 65508 |
| Block 807 | ssr1 | 4 | 648667 | 650194 | fgeneshDP_pg.C_scaffold_202000018 | scaffold_202 | 40955 | 42249 |
| Block 807 | DDB_G0283413 | 4 | 651190 | 652101 | GID1.0045160 | scaffold_202 | 39524 | 40399 |
| Block 807 | DDB_G0283415 | 4 | 652443 | 653317 | GID1.0045167 | scaffold_202 | 52177 | 52987 |
| Block 807 | DDB_G0283417 | 4 | 654013 | 654705 | GID1.0045168 | scaffold_202 | 53975 | 55916 |
| Block 807 | gpaI | 4 | 656137 | 657400 | e_gw1.202.8.1 | scaffold_202 | 56540 | 57826 |
| Block 1630 | DDB_G0283499 | 4 | 660137 | 660898 | fgeneshDP_pg.C_scaffold_354000001 | scaffold_354 | 714 | 1457 |
| Block 1630 | rplP2 | 4 | 661727 | 662322 | GID1.0047972 | scaffold_354 | 1829 | 4300 |
| Block 1649 | DDB_G0283437 | 4 | 697659 | 698518 | fgeneshDP_pg.C_scaffold_359000003 | scaffold_359 | 4399 | 5225 |
| Block 1649 | DDB_G0283439 | 4 | 699253 | 700845 | estExt_fgeneshDP_pg.C_3590002 | scaffold_359 | 1750 | 3784 |
| Block 1731 | DDB_G0283523 | 4 | 701281 | 702009 | e_gw1.377.19.1 | scaffold_377 | 27148 | 28135 |
| Block 1731 | DDB_G0283451 | 4 | 712043 | 713824 | estExt_fgeneshDP_pg.C_3770013 | scaffold_377 | 28258 | 30581 |
| Block 184 | gcn5 | 4 | 727119 | 728357 | e_gw1.12.3.1 | scaffold_12 | 9276 | 10355 |
| Block 1860 | DDB_G0283473 | 4 | 746464 | 748248 | e_gw1.405.20.1 | scaffold_405 | 2121 | 3848 |
| Block 1860 | kinX | 4 | 749416 | 752964 | e_gw1.405.8.1 | scaffold_405 | 4804 | 5814 |
| Block 1860 | DDB_G0283475 | 4 | 753547 | 760773 | estExt_fgeneshDP_pm.C_4050003 | scaffold_405 | 8622 | 15601 |
| Block 2277 | DDB_G0283591 | 4 | 783267 | 784202 | fgeneshDP_pg.C_scaffold_517000006 | scaffold_517 | 12033 | 12359 |
| Block 2277 | DDB_G0283585 | 4 | 785263 | 790130 | estExt_fgeneshDP_pg.C_5170005 | scaffold_517 | 8050 | 10167 |
| Block 2277 | yipf5 | 4 | 791068 | 791911 | GID1.0049772 | scaffold_517 | 6749 | 7552 |
| Block 2277 | prp40 | 4 | 792074 | 794312 | GID1.0049770 | scaffold_517 | 2568 | 4480 |
| Block 2277 | DDB_G0283545 | 4 | 794803 | 797775 | GID1.0049769 | scaffold_517 | 263 | 1915 |
| Block 1860 | crtA | 4 | 820858 | 822240 | estExt_Genewise1.C_4050015 | scaffold_405 | 24546 | 26053 |
| Block 1860 | CSN1 | 4 | 822737 | 824361 | estExt_Genewise1Plus.C_4050014 | scaffold_405 | 22363 | 24037 |
| Block 1860 | DDB_G0283555 | 4 | 824798 | 826280 | estExt_Genewise1.C_4050011 | scaffold_405 | 20331 | 22113 |
| Block 648 | DDB_G0283557 | 4 | 827565 | 829532 | e_gw1.180.18.1 | scaffold_180 | 57399 | 58592 |
| Block 648 | cbpB | 4 | 829856 | 830504 | e_gw1.180.39.1 | scaffold_180 | 58899 | 59492 |
| Block 3037 | DDB_G0283565 | 4 | 833347 | 836465 | GID1.0042116 | scaffold_96 | 19097 | 22057 |
| Block 3037 | DDB_G0283567 | 4 | 836774 | 837473 | fgeneshDP_pm.C_scaffold_96000008 | scaffold_96 | 22371 | 23072 |
| Block 3037 | DG1104 | 4 | 837650 | 846384 | GID1.0042118 | scaffold_96 | 23305 | 31423 |
| Block 1234 | eIF3s9 | 4 | 847464 | 849687 | GID1.0046681 | scaffold_276 | 6942 | 9148 |
| Block 1234 | rasS | 4 | 849934 | 850518 | estExt_Genewise1Plus.C_2760004 | scaffold_276 | 1330 | 2312 |
| Block 1234 | DDB_G0283571 | 4 | 851425 | 851742 | gw1.276.30.1 | scaffold_276 | 185 | 436 |
| Block 2256 | DDB_G0283577 | 4 | 856410 | 859921 | GID1.0040274 | scaffold_51 | 34734 | 41351 |
| Block 2256 | utp15 | 4 | 863530 | 865415 | e_gw1.51.35.1 | scaffold_51 | 24539 | 26234 |
| Block 2256 | yakA | 4 | 868454 | 873263 | estExt_fgeneshDP_pg.C_510013 | scaffold_51 | 27834 | 32000 |
| Block 2256 | DDB_G0283745 | 4 | 877706 | 881475 | GID1.0040284 | scaffold_51 | 58257 | 61773 |
| Block 2256 | DDB_G0283625 | 4 | 882607 | 883473 | estExt_Genewise1Plus.C_510036 | scaffold_51 | 56643 | 57670 |
| Block 2256 | prsC | 4 | 883884 | 885363 | estExt_Genewise1Plus.C_510033 | scaffold_51 | 54938 | 56190 |
| Block 2256 | DDB_G0283629 | 4 | 885727 | 887358 | estExt_Genewise1Plus.C_510027 | scaffold_51 | 49179 | 54145 |
| Block 2256 | DDB_G0283633 | 4 | 889575 | 891258 | gw1.51.8.1 | scaffold_51 | 45820 | 47095 |
| Block 2256 | wdr89 | 4 | 891645 | 892935 | fgeneshDP_pg.C_scaffold_51000017 | scaffold_51 | 44088 | 45243 |
| Block 2256 | cutA | 4 | 908845 | 910395 | estExt_Genewise1Plus.C_510005 | scaffold_51 | 14501 | 16366 |
| Block 2119 | DDB_G0283649 | 4 | 910617 | 911334 | GID1.0049379 | scaffold_471 | 2679 | 3456 |
| Block 2119 | eb1 | 4 | 912876 | 914543 | estExt_fgeneshDP_pg.C_4710004 | scaffold_471 | 5539 | 7002 |
| Block 2119 | crlC | 4 | 927333 | 928611 | GID1.0049382 | scaffold_471 | 7091 | 8294 |
| Block 900 | ddx3 | 4 | 939305 | 941576 | estExt_fgeneshDP_pm.C_2160005 | scaffold_216 | 41989 | 44783 |
| Block 900 | ppiD | 4 | 943186 | 944382 | GID1.0045475 | scaffold_216 | 39762 | 41000 |
| Block 900 | DDB_G0283667 | 4 | 946222 | 948830 | estExt_fgeneshDP_pg.C_2160012 | scaffold_216 | 36767 | 39385 |
| Block 900 | pppA | 4 | 949430 | 952310 | estExt_fgeneshDP_pg.C_2160011 | scaffold_216 | 33899 | 36612 |
| Block 900 | DDB_G0283669 | 4 | 953002 | 953325 | e_gw1.216.37.1 | scaffold_216 | 32657 | 33144 |
| Block 900 | grn | 4 | 953712 | 954208 | e_gw1.216.24.1 | scaffold_216 | 31937 | 32308 |
| Block 189 | DDB_G0283675 | 4 | 961972 | 963150 | estExt_fgeneshDP_pg.C_120035 | scaffold_12 | 83876 | 85110 |
| Block 189 | DDB_G0283677 | 4 | 963963 | 964844 | e_gw1.12.73.1 | scaffold_12 | 89176 | 90042 |
| Block 189 | rab32A | 4 | 965063 | 966191 | fgeneshDP_pg.C_scaffold_12000040 | scaffold_12 | 90271 | 91494 |
| Block 189 | psmB7 | 4 | 967091 | 968259 | estExt_Genewise1.C_120082 | scaffold_12 | 92238 | 93507 |
| Block 189 | limA | 4 | 968949 | 972603 | estExt_Genewise1.C_120085 | scaffold_12 | 93781 | 94741 |
| Block 189 | DDB_G0283681 | 4 | 973798 | 978136 | estExt_Genewise1.C_120086 | scaffold_12 | 97792 | 101937 |
| Block 189 | DDB_G0283683 | 4 | 978468 | 980033 | e_gw1.12.50.1 | scaffold_12 | 156455 | 158010 |
| Block 189 | utp23 | 4 | 980287 | 981874 | fgeneshDP_pm.C_scaffold_12000032 | scaffold_12 | 154766 | 156050 |
| Block 189 | DDB_G0283687 | 4 | 982205 | 984735 | fgeneshDP_pg.C_scaffold_12000060 | scaffold_12 | 152401 | 154525 |
| Block 703 | DDB_G0283697 | 4 | 992255 | 995015 | fgeneshDP_pg.C_scaffold_190000016 | scaffold_190 | 49830 | 52118 |
| Block 72 | phg2 | 4 | 995559 | 999815 | e_gw1.106.28.1 | scaffold_106 | 29912 | 33743 |
| Block 72 | guaB | 4 | 1000722 | 1002269 | estExt_fgeneshDP_pm.C_1060005 | scaffold_106 | 26614 | 28252 |
| Block 72 | osbH | 4 | 1009391 | 1010770 | estExt_fgeneshDP_kg.C_1060001 | scaffold_106 | 16493 | 18600 |
| Block 703 | DDB_G0283715 | 4 | 1012994 | 1016773 | GID1.0044873 | scaffold_190 | 56759 | 59546 |
| Block 1651 | rpkA | 4 | 1020192 | 1022856 | estExt_fgeneshDP_pg.C_3590007 | scaffold_359 | 13824 | 16561 |
| Block 1651 | DDB_G0283719 | 4 | 1023936 | 1028052 | estExt_fgeneshDP_pg.C_3590005 | scaffold_359 | 7268 | 11232 |
| Block 2052 | DDB_G0283729 | 4 | 1039785 | 1041314 | fgeneshDP_pm.C_scaffold_454000002 | scaffold_454 | 9878 | 11620 |
| Block 2052 | DDB_G0283731 | 4 | 1041764 | 1042491 | GID1.0049209 | scaffold_454 | 13808 | 14590 |
| Block 955 | DDB_G0283733 | 4 | 1042995 | 1044131 | fgeneshDP_pg.C_scaffold_226000006 | scaffold_226 | 9046 | 9990 |
| Block 1484 | dnajc19 | 4 | 1046052 | 1046527 | e_gw1.321.30.1 | scaffold_321 | 23134 | 23583 |
| Block 1484 | DDB_G0283737 | 4 | 1046828 | 1047289 | fgeneshDP_pm.C_scaffold_321000007 | scaffold_321 | 23957 | 24407 |
| Block 955 | rpl26 | 4 | 1048980 | 1049683 | e_gw1.226.11.1 | scaffold_226 | 5728 | 6228 |
| Block 1484 | DDB_G0283753 | 4 | 1050017 | 1052135 | estExt_fgeneshDP_pg.C_3210013 | scaffold_321 | 28412 | 31012 |
| Block 1484 | cdc25 | 4 | 1053164 | 1056325 | estExt_fgeneshDP_pg.C_3210015 | scaffold_321 | 33961 | 36465 |
| Block 955 | DDB_G0283855 | 4 | 1060450 | 1062283 | gw1.226.5.1 | scaffold_226 | 49989 | 51482 |
| Block 955 | lig3 | 4 | 1062604 | 1066349 | fgeneshDP_pg.C_scaffold_226000022 | scaffold_226 | 46000 | 49567 |
| Block 955 | DDB_G0283859 | 4 | 1066871 | 1072160 | fgeneshDP_pg.C_scaffold_226000021 | scaffold_226 | 40873 | 45927 |
| Block 955 | DDB_G0283861 | 4 | 1072483 | 1073637 | estExt_fgeneshDP_pg.C_2260020 | scaffold_226 | 39606 | 40590 |
| Block 511 | DDB_G0283865 | 4 | 1077378 | 1078425 | GID1.0044116 | scaffold_160 | 33222 | 33820 |
| Block 511 | DDB_G0283771 | 4 | 1079549 | 1081769 | estExt_fgeneshDP_pg.C_1600017 | scaffold_160 | 34367 | 36434 |
| Block 511 | DDB_G0283773 | 4 | 1081883 | 1083350 | fgeneshDP_pm.C_scaffold_160000008 | scaffold_160 | 36647 | 37912 |
| Block 1000 | DDB_G0283781 | 4 | 1089062 | 1090168 | GID1.0045803 | scaffold_231 | 39223 | 40110 |
| Block 1000 | cprC | 4 | 1092858 | 1094270 | estExt_fgeneshDP_kg.C_2310001 | scaffold_231 | 40905 | 42208 |
| Block 2342 | dimt1l | 4 | 1107655 | 1109115 | fgeneshDP_pg.C_scaffold_535000005 | scaffold_535 | 11676 | 12867 |
| Block 2342 | DDB_G0283791 | 4 | 1109469 | 1110788 | GID1.0049909 | scaffold_535 | 9973 | 11319 |
| Block 2342 | DDB_G0283793 | 4 | 1114386 | 1116616 | estExt_fgeneshDP_pg.C_5350003 | scaffold_535 | 4270 | 6572 |
| Block 2342 | commd3 | 4 | 1117216 | 1118052 | e_gw1.535.6.1 | scaffold_535 | 3087 | 3868 |
| Block 511 | DDB_G0283797 | 4 | 1118346 | 1119815 | fgeneshDP_pg.C_scaffold_160000013 | scaffold_160 | 26889 | 28154 |
| Block 511 | DDB_G0283799 | 4 | 1120333 | 1121659 | e_gw1.160.11.1 | scaffold_160 | 25214 | 26362 |
| Block 2484 | fam96B | 4 | 1121919 | 1122538 | e_gw1.59.75.1 | scaffold_59 | 100296 | 100715 |
| Block 2484 | DDB_G0283803 | 4 | 1122911 | 1123829 | estExt_Genewise1.C_590074 | scaffold_59 | 99194 | 100018 |
| Block 471 | dct | 4 | 1132285 | 1133349 | estExt_fgeneshDP_kg.C_1540001 | scaffold_154 | 1772 | 3140 |
| Block 471 | dus2l | 4 | 1134008 | 1135291 | e_gw1.154.3.1 | scaffold_154 | 4523 | 5754 |
| Block 1547 | arpC | 4 | 1135639 | 1137330 | estExt_Genewise1.C_3370010 | scaffold_337 | 17864 | 19910 |
| Block 1547 | HBS1 | 4 | 1138835 | 1141696 | gw1.337.3.1 | scaffold_337 | 14049 | 16867 |
| Block 511 | hmgL | 4 | 1145359 | 1146736 | estExt_Genewise1Plus.C_1600027 | scaffold_160 | 28338 | 29813 |
| Block 2334 | DDB_G0283815 | 4 | 1148242 | 1149573 | e_gw1.532.6.1 | scaffold_532 | 8808 | 10121 |
| Block 2334 | hgd | 4 | 1151090 | 1152640 | estExt_Genewise1.C_5320003 | scaffold_532 | 6824 | 8790 |
| Block 2334 | DDB_G0283819 | 4 | 1153703 | 1155501 | GID1.0049886 | scaffold_532 | 3707 | 5484 |
| Block 471 | DDB_G0283821 | 4 | 1155677 | 1158750 | estExt_Genewise1.C_1540005 | scaffold_154 | 5957 | 10144 |
| Block 471 | mlh3 | 4 | 1160700 | 1165945 | GID1.0043953 | scaffold_154 | 43557 | 48237 |
| Block 471 | DDB_G0283885 | 4 | 1166070 | 1167758 | fgeneshDP_pg.C_scaffold_154000017 | scaffold_154 | 42241 | 43419 |
| Block 471 | DDB_G0283887 | 4 | 1170844 | 1172651 | e_gw1.154.25.1 | scaffold_154 | 38286 | 40195 |
| Block 471 | DDB_G0283889 | 4 | 1174977 | 1176878 | GID1.0043950 | scaffold_154 | 33944 | 35800 |
| Block 471 | DDB_G0283823 | 4 | 1177277 | 1177982 | estExt_fgeneshDP_kg.C_1540005 | scaffold_154 | 32891 | 33549 |
| Block 471 | DDB_G0283825 | 4 | 1178221 | 1179642 | estExt_fgeneshDP_pm.C_1540008 | scaffold_154 | 31248 | 32885 |
| Block 471 | DDB_G0283829 | 4 | 1183400 | 1184878 | fgeneshDP_pm.C_scaffold_154000006 | scaffold_154 | 26491 | 28129 |
| Block 1474 | rae1 | 4 | 1191439 | 1192760 | GID1.0039338 | scaffold_32 | 44376 | 45633 |
| Block 1474 | ube1c | 4 | 1193106 | 1194623 | GID1.0039339 | scaffold_32 | 46036 | 47527 |
| Block 2120 | DDB_G0283837 | 4 | 1195917 | 1198139 | fgeneshDP_pg.C_scaffold_471000006 | scaffold_471 | 9198 | 11203 |
| Block 2370 | grlN | 4 | 1198222 | 1201082 | GID1.0049987 | scaffold_547 | 6558 | 9110 |
| Block 2494 | alg6 | 4 | 1202486 | 1204316 | e_gw1.593.1.1 | scaffold_593 | 5177 | 6989 |
| Block 515 | DDB_G0283843 | 4 | 1223477 | 1224134 | estExt_fgeneshDP_pg.C_1600028 | scaffold_160 | 58669 | 59677 |
| Block 515 | DDB_G0283845 | 4 | 1227055 | 1227727 | GID1.0044129 | scaffold_160 | 55802 | 56415 |
| Block 515 | DDB_G0283847 | 4 | 1228686 | 1230545 | estExt_Genewise1.C_1600041 | scaffold_160 | 53296 | 54896 |
| Block 2370 | DDB_G0283759 | 4 | 1232305 | 1235646 | GID1.0049989 | scaffold_547 | 11718 | 14766 |
| Block 2370 | pgtA | 4 | 1235916 | 1238225 | GID1.0049988 | scaffold_547 | 9171 | 11381 |
| Block 2494 | grpE | 4 | 1238328 | 1239107 | fgeneshDP_pm.C_scaffold_593000003 | scaffold_593 | 4259 | 4988 |
| Block 2494 | DDB_G0283849 | 4 | 1239874 | 1240653 | e_gw1.593.12.1 | scaffold_593 | 2782 | 3494 |
| Block 2494 | rab1A | 4 | 1241175 | 1242406 | GID1.0050268 | scaffold_593 | 677 | 1915 |
| Block 2053 | mgp4 | 4 | 1248575 | 1251382 | e_gw1.454.1.1 | scaffold_454 | 18235 | 21174 |
| Block 2053 | DDB_G0283901 | 4 | 1254496 | 1256100 | estExt_fgeneshDP_pg.C_4540008 | scaffold_454 | 15162 | 16797 |
| Block 2120 | DDB_G0283983 | 4 | 1259472 | 1262994 | estExt_fgeneshDP_pg.C_4710009 | scaffold_471 | 17452 | 21778 |
| Block 2120 | DDB_G0283985 | 4 | 1263489 | 1265251 | GID1.0049385 | scaffold_471 | 11282 | 13112 |
| Block 1473 | DDB_G0283917 | 4 | 1272110 | 1273864 | e_gw1.32.51.1 | scaffold_32 | 69615 | 71391 |
| Block 1473 | DDB_G0283919 | 4 | 1275553 | 1278795 | estExt_Genewise1.C_320054 | scaffold_32 | 73208 | 77110 |
| Block 2515 | DDB_G0283989 | 4 | 1284281 | 1287103 | fgeneshDP_pg.C_scaffold_6000059 | scaffold_6 | 160824 | 163624 |
| Block 1473 | DDB_G0283991 | 4 | 1287653 | 1288744 | estExt_Genewise1Plus.C_320057 | scaffold_32 | 77558 | 78888 |
| Block 1473 | DDB_G0283993 | 4 | 1289307 | 1292881 | fgeneshDP_pg.C_scaffold_32000032 | scaffold_32 | 79603 | 83171 |
| Block 2806 | DDB_G0283925 | 4 | 1306681 | 1307437 | fgeneshDP_pg.C_scaffold_749000002 | scaffold_749 | 2754 | 3352 |
| Block 2806 | DDB_G0283927 | 4 | 1307685 | 1309325 | e_gw1.749.2.1 | scaffold_749 | 1082 | 2523 |
| Block 2515 | sec1 | 4 | 1335851 | 1337901 | GID1.0037722 | scaffold_6 | 190986 | 192960 |
| Block 2515 | DDB_G0283939 | 4 | 1338263 | 1341418 | e_gw1.6.48.1 | scaffold_6 | 189082 | 189441 |
| Block 2254 | DDB_G0283943 | 4 | 1342529 | 1344406 | estExt_fgeneshDP_pm.C_510001 | scaffold_51 | 6554 | 8211 |
| Block 1473 | msh4 | 4 | 1370478 | 1373603 | GID1.0039328 | scaffold_32 | 14051 | 17258 |
| Block 1473 | DDB_G0283959 | 4 | 1374141 | 1375052 | GID1.0039327 | scaffold_32 | 12482 | 13398 |
| Block 1473 | spc98 | 4 | 1376667 | 1379177 | GID1.0039325 | scaffold_32 | 7306 | 9849 |
| Block 1473 | DDB_G0284003 | 4 | 1379527 | 1382094 | GID1.0039324 | scaffold_32 | 4504 | 6888 |
| Block 1473 | fam32 | 4 | 1382400 | 1382738 | estExt_fgeneshDP_kg.C_320003 | scaffold_32 | 3923 | 4440 |
| Block 1473 | pigA | 4 | 1382938 | 1384703 | GID1.0039322 | scaffold_32 | 2174 | 3758 |
| Block 1473 | erkB | 4 | 1385123 | 1386548 | estExt_fgeneshDP_pm.C_320001 | scaffold_32 | 96 | 2061 |
| Block 2051 | pkaC | 4 | 1394958 | 1397005 | e_gw1.454.3.1 | scaffold_454 | 1729 | 3506 |
| Block 2051 | DDB_G0284007 | 4 | 1398686 | 1399744 | GID1.0049205 | scaffold_454 | 4814 | 5872 |
| Block 1473 | DDB_G0283971 | 4 | 1402443 | 1405050 | estExt_Genewise1Plus.C_320044 | scaffold_32 | 59466 | 62431 |
| Block 1473 | DDB_G0283973 | 4 | 1405614 | 1407920 | GID1.0039343 | scaffold_32 | 56917 | 59087 |
| Block 1473 | DDB_G0283975 | 4 | 1408395 | 1409228 | e_gw1.32.77.1 | scaffold_32 | 55750 | 56553 |
| Block 2254 | DDB_G0283977 | 4 | 1409294 | 1411915 | GID1.0040262 | scaffold_51 | 8783 | 11213 |
| Block 305 | pms1 | 4 | 1418066 | 1421134 | GID1.0043315 | scaffold_132 | 65354 | 68224 |
| Block 305 | phr2AB | 4 | 1423354 | 1425584 | estExt_Genewise1.C_1320052 | scaffold_132 | 70543 | 73265 |
| Block 2798 | ubcC | 4 | 1428069 | 1429134 | estExt_Genewise1.C_7430002 | scaffold_743 | 13 | 1444 |
| Block 2798 | DDB_G0284011 | 4 | 1429547 | 1430576 | GID1.0050899 | scaffold_743 | 3762 | 4751 |
| Block 661 | DDB_G0284013 | 4 | 1432537 | 1433609 | GID1.0044726 | scaffold_184 | 1170 | 2392 |
| Block 2103 | DDB_G0284015 | 4 | 1433731 | 1436234 | GID1.0040077 | scaffold_47 | 1512 | 7121 |
| Block 661 | DDB_G0284025 | 4 | 1440123 | 1442348 | estExt_fgeneshDP_kg.C_1840006 | scaffold_184 | 35300 | 36611 |
| Block 998 | DDB_G0284019 | 4 | 1444241 | 1446775 | fgeneshDP_pm.C_scaffold_231000001 | scaffold_231 | 7080 | 9566 |
| Block 2103 | DDB_G0284021 | 4 | 1447496 | 1448281 | GID1.0040080 | scaffold_47 | 11391 | 12053 |
| Block 2705 | DDB_G0284033 | 4 | 1456924 | 1458222 | e_gw1.682.1.1 | scaffold_682 | 3207 | 4420 |
| Block 2705 | DDB_G0284031 | 4 | 1458434 | 1459279 | e_gw1.682.3.1 | scaffold_682 | 4576 | 5390 |
| Block 2705 | pkgD | 4 | 1459718 | 1462938 | estExt_fgeneshDP_pg.C_6820003 | scaffold_682 | 5602 | 8565 |
| Block 188 | DDB_G0284053 | 4 | 1473938 | 1474783 | fgeneshDP_pm.C_scaffold_12000021 | scaffold_12 | 82795 | 83693 |
| Block 188 | DDB_G0284059 | 4 | 1478818 | 1482847 | GID1.0038139 | scaffold_12 | 75816 | 80196 |
| Block 188 | DDB_G0284063 | 4 | 1485717 | 1486784 | estExt_fgeneshDP_pm.C_120017 | scaffold_12 | 71784 | 72604 |
| Block 299 | atp7a | 4 | 1486936 | 1490134 | estExt_Genewise1.C_1320003 | scaffold_132 | 999 | 4703 |
| Block 3036 | DDB_G0284065 | 4 | 1493285 | 1494732 | estExt_Genewise1Plus.C_960011 | scaffold_96 | 16175 | 17556 |
| Block 3036 | DDB_G0284069 | 4 | 1498595 | 1499943 | fgeneshDP_pm.C_scaffold_96000003 | scaffold_96 | 9494 | 10801 |
| Block 3036 | rcdII | 4 | 1500251 | 1502171 | estExt_fgeneshDP_pm.C_960002 | scaffold_96 | 7109 | 8747 |
| Block 998 | rnrA | 4 | 1503516 | 1506219 | estExt_Genewise1.C_2310017 | scaffold_231 | 27650 | 30768 |
| Block 998 | dhkG | 4 | 1509861 | 1520159 | GID1.0045798 | scaffold_231 | 17565 | 26766 |
| Block 299 | DDB_G0284145 | 4 | 1523365 | 1525118 | e_gw1.132.32.1 | scaffold_132 | 5193 | 6455 |
| Block 299 | elp1 | 4 | 1525708 | 1529993 | fgeneshDP_pg.C_scaffold_132000003 | scaffold_132 | 6890 | 11123 |
| Block 956 | DDB_G0284077 | 4 | 1530962 | 1532422 | e_gw1.226.24.1 | scaffold_226 | 7199 | 8493 |
| Block 660 | DDB_G0284147 | 4 | 1532512 | 1533541 | estExt_fgeneshDP_pg.C_1840001 | scaffold_184 | 60 | 1134 |
| Block 3036 | DDB_G0284079 | 4 | 1538387 | 1539423 | GID1.0042115 | scaffold_96 | 17822 | 18792 |
| Block 2448 | abcF2 | 4 | 1542455 | 1544698 | GID1.0050163 | scaffold_576 | 5302 | 6832 |
| Block 2448 | DDB_G0284153 | 4 | 1545923 | 1547930 | GID1.0050165 | scaffold_576 | 9398 | 11200 |
| Block 2448 | DDB_G0284085 | 4 | 1549462 | 1550559 | gw1.576.8.1 | scaffold_576 | 11391 | 12350 |
| Block 956 | rps16 | 4 | 1558362 | 1559293 | estExt_fgeneshDP_pg.C_2260007 | scaffold_226 | 10363 | 11354 |
| Block 998 | DDB_G0284155 | 4 | 1561847 | 1565439 | GID1.0045797 | scaffold_231 | 13783 | 17190 |
| Block 660 | DDB_G0284097 | 4 | 1565830 | 1568043 | estExt_fgeneshDP_pg.C_1840016 | scaffold_184 | 57938 | 60309 |
| Block 660 | DDB_G0284099 | 4 | 1569018 | 1570493 | estExt_fgeneshDP_pg.C_1840017 | scaffold_184 | 60854 | 62527 |
| Block 998 | DDB_G0284105 | 4 | 1580531 | 1582704 | GID1.0045793 | scaffold_231 | 1516 | 3160 |
| Block 514 | DG1039 | 4 | 1593040 | 1595399 | estExt_Genewise1Plus.C_1600046 | scaffold_160 | 59908 | 62402 |
| Block 514 | pprA | 4 | 1595576 | 1596770 | fgeneshDP_pg.C_scaffold_160000030 | scaffold_160 | 62441 | 63624 |
| Block 514 | DDB_G0284113 | 4 | 1602053 | 1605870 | GID1.0044127 | scaffold_160 | 50601 | 53086 |
| Block 514 | DDB_G0284165 | 4 | 1606151 | 1606472 | e_gw1.160.53.1 | scaffold_160 | 49939 | 50142 |
| Block 514 | DDB_G0284115 | 4 | 1607949 | 1608528 | estExt_fgeneshDP_kg.C_1600006 | scaffold_160 | 47177 | 47891 |
| Block 2497 | phbB | 4 | 1608856 | 1609984 | fgeneshDP_pm.C_scaffold_596000002 | scaffold_596 | 9420 | 10629 |
| Block 2497 | DDB_G0284167 | 4 | 1610129 | 1611458 | estExt_fgeneshDP_kg.C_5960001 | scaffold_596 | 7987 | 9250 |
| Block 2497 | DDB_G0284169 | 4 | 1612603 | 1613308 | GID1.0050286 | scaffold_596 | 5710 | 6484 |
| Block 2497 | DDB_G0284125 | 4 | 1617357 | 1618926 | e_gw1.596.1.1 | scaffold_596 | 895 | 4658 |
| Block 2497 | DDB_G0284127 | 4 | 1619168 | 1620657 | fgeneshDP_pg.C_scaffold_596000001 | scaffold_596 | 5 | 680 |
| Block 733 | E2F | 4 | 1622284 | 1624946 | estExt_fgeneshDP_pg.C_1960001 | scaffold_196 | 736 | 2986 |
| Block 998 | DDB_G0284135 | 4 | 1634829 | 1637741 | estExt_fgeneshDP_pg.C_2310015 | scaffold_231 | 47128 | 49963 |
| Block 733 | DDB_G0284139 | 4 | 1640056 | 1641798 | fgeneshDP_pg.C_scaffold_196000004 | scaffold_196 | 12538 | 14349 |
| Block 733 | DDB_G0284171 | 4 | 1645755 | 1651739 | GID1.0045007 | scaffold_196 | 20970 | 26660 |
| Block 733 | DDB_G0284173 | 4 | 1652572 | 1653456 | e_gw1.196.36.1 | scaffold_196 | 18978 | 20000 |
| Block 181 | DDB_G0284177 | 4 | 1658818 | 1662279 | estExt_fgeneshDP_pg.C_120001 | scaffold_12 | 298 | 3717 |
| Block 181 | DDB_G0284195 | 4 | 1676837 | 1679614 | estExt_Genewise1Plus.C_120056 | scaffold_12 | 58967 | 61390 |
| Block 181 | proS | 4 | 1680128 | 1681774 | estExt_Genewise1.C_120054 | scaffold_12 | 56434 | 58373 |
| Block 181 | DDB_G0284199 | 4 | 1682534 | 1683645 | e_gw1.12.83.1 | scaffold_12 | 55061 | 55954 |
| Block 181 | DDB_G0284201 | 4 | 1684869 | 1685329 | fgeneshDP_pm.C_scaffold_12000014 | scaffold_12 | 53872 | 54283 |
| Block 181 | kynu | 4 | 1685557 | 1686979 | fgeneshDP_pg.C_scaffold_12000020 | scaffold_12 | 52124 | 53630 |
| Block 181 | ubl5 | 4 | 1687407 | 1687951 | e_gw1.12.36.1 | scaffold_12 | 51390 | 51841 |
| Block 181 | DDB_G0284213 | 4 | 1693957 | 1696086 | GID1.0038120 | scaffold_12 | 48275 | 50292 |
| Block 181 | krsA | 4 | 1697612 | 1699450 | estExt_fgeneshDP_pm.C_120010 | scaffold_12 | 45389 | 47237 |
| Block 181 | DDB_G0284217 | 4 | 1700271 | 1701885 | gw1.12.69.1 | scaffold_12 | 42874 | 44070 |
| Block 181 | DDB_G0284219 | 4 | 1702610 | 1704995 | GID1.0038116 | scaffold_12 | 40014 | 42347 |
| Block 181 | atox1 | 4 | 1705613 | 1706032 | fgeneshDP_pm.C_scaffold_12000008 | scaffold_12 | 31235 | 31885 |
| Block 181 | DDB_G0284307 | 4 | 1706442 | 1708221 | estExt_fgeneshDP_pm.C_120007 | scaffold_12 | 28636 | 30416 |
| Block 2074 | DDB_G0284311 | 4 | 1711370 | 1712269 | GID1.0040057 | scaffold_46 | 67284 | 68081 |
| Block 2074 | DDB_G0284223 | 4 | 1712491 | 1714131 | GID1.0040058 | scaffold_46 | 68387 | 70220 |
| Block 1020 | tbcC | 4 | 1725990 | 1727775 | e_gw1.235.18.1 | scaffold_235 | 32589 | 34209 |
| Block 1020 | DDB_G0284315 | 4 | 1728697 | 1729626 | estExt_fgeneshDP_kg.C_2350002 | scaffold_235 | 31058 | 32265 |
| Block 1500 | DDB_G0284239 | 4 | 1731488 | 1733684 | e_gw1.325.8.1 | scaffold_325 | 21607 | 23113 |
| Block 1500 | DDB_G0284241 | 4 | 1733961 | 1735439 | GID1.0047543 | scaffold_325 | 17625 | 19031 |
| Block 1500 | DDB_G0284243 | 4 | 1735866 | 1737435 | estExt_Genewise1Plus.C_3250008 | scaffold_325 | 15282 | 17433 |
| Block 1500 | sympk | 4 | 1741477 | 1746822 | fgeneshDP_pm.C_scaffold_325000004 | scaffold_325 | 10157 | 14517 |
| Block 1500 | DDB_G0284245 | 4 | 1747466 | 1748910 | estExt_fgeneshDP_pg.C_3250004 | scaffold_325 | 8900 | 9952 |
| Block 1466 | DDB_G0284247 | 4 | 1749570 | 1751523 | GID1.0047425 | scaffold_318 | 24106 | 25863 |
| Block 1466 | DDB_G0284249 | 4 | 1751734 | 1754028 | e_gw1.318.2.1 | scaffold_318 | 22346 | 23890 |
| Block 1466 | DDB_G0284251 | 4 | 1754346 | 1756247 | estExt_fgeneshDP_pm.C_3180001 | scaffold_318 | 19254 | 21002 |
| Block 1466 | DDB_G0284253 | 4 | 1758153 | 1759359 | e_gw1.318.15.1 | scaffold_318 | 15660 | 16069 |
| Block 1466 | DDB_G0284321 | 4 | 1760065 | 1764027 | fgeneshDP_pg.C_scaffold_318000001 | scaffold_318 | 10214 | 13902 |
| Block 2778 | DDB_G0284255 | 4 | 1777902 | 1780775 | estExt_fgeneshDP_pg.C_730001 | scaffold_73 | 1270 | 4451 |
| Block 2778 | dph1 | 4 | 1781348 | 1782846 | GID1.0041214 | scaffold_73 | 4527 | 6055 |
| Block 2778 | ppil2 | 4 | 1783122 | 1785177 | fgeneshDP_pg.C_scaffold_73000003 | scaffold_73 | 6282 | 8116 |
| Block 2778 | DDB_G0284259 | 4 | 1787150 | 1790086 | estExt_Genewise1.C_730010 | scaffold_73 | 9272 | 12321 |
| Block 2778 | DDB_G0284303 | 4 | 1792427 | 1792983 | fgeneshDP_pg.C_scaffold_73000004 | scaffold_73 | 8275 | 8753 |
| Block 2778 | DDB_G0284263 | 4 | 1795281 | 1797504 | fgeneshDP_pg.C_scaffold_73000007 | scaffold_73 | 14580 | 16613 |
| Block 2778 | ppa1 | 4 | 1798190 | 1799209 | estExt_fgeneshDP_pm.C_730005 | scaffold_73 | 19122 | 20097 |
| Block 2778 | eIF2a | 4 | 1799493 | 1801319 | e_gw1.73.5.1 | scaffold_73 | 20320 | 22047 |
| Block 2778 | DDB_G0284273 | 4 | 1806777 | 1808384 | fgeneshDP_pg.C_scaffold_73000011 | scaffold_73 | 22765 | 24380 |
| Block 2778 | DDB_G0284325 | 4 | 1809449 | 1811827 | estExt_fgeneshDP_pg.C_730012 | scaffold_73 | 25320 | 27772 |
| Block 2778 | Dd5P2 | 4 | 1813905 | 1819443 | fgeneshDP_pg.C_scaffold_73000014 | scaffold_73 | 30921 | 35468 |
| Block 2778 | DDB_G0284275 | 4 | 1820423 | 1821487 | GID1.0041229 | scaffold_73 | 36152 | 37074 |
| Block 1549 | DDB_G0284287 | 4 | 1834114 | 1834675 | e_gw1.337.16.1 | scaffold_337 | 32197 | 32708 |
| Block 1549 | DDB_G0284289 | 4 | 1835474 | 1837830 | estExt_fgeneshDP_pg.C_3370015 | scaffold_337 | 33392 | 35210 |
| Block 2778 | DDB_G0284295 | 4 | 1844275 | 1845881 | fgeneshDP_pm.C_scaffold_73000009 | scaffold_73 | 46490 | 49175 |
| Block 1549 | rcdK | 4 | 1846274 | 1849299 | fgeneshDP_pm.C_scaffold_337000008 | scaffold_337 | 28952 | 31488 |
| Block 1549 | DDB_G0284337 | 4 | 1851676 | 1855620 | estExt_fgeneshDP_pg.C_3370012 | scaffold_337 | 23331 | 27485 |
| Block 1549 | lyrm5 | 4 | 1857300 | 1858168 | GID1.0047728 | scaffold_337 | 22696 | 23187 |
| Block 2105 | DDB_G0284443 | 4 | 1858931 | 1860087 | fgeneshDP_pg.C_scaffold_47000017 | scaffold_47 | 38559 | 39634 |
| Block 2105 | DDB_G0284343 | 4 | 1860277 | 1861465 | e_gw1.47.77.1 | scaffold_47 | 37151 | 38341 |
| Block 2105 | CYP556A1 | 4 | 1862286 | 1864421 | fgeneshDP_pg.C_scaffold_47000015 | scaffold_47 | 34251 | 36390 |
| Block 2105 | vps4 | 4 | 1865073 | 1866634 | estExt_Genewise1.C_470018 | scaffold_47 | 32018 | 33791 |
| Block 2073 | osbI | 4 | 1870805 | 1872192 | e_gw1.46.34.1 | scaffold_46 | 50173 | 51198 |
| Block 2073 | DDB_G0284355 | 4 | 1873789 | 1876121 | estExt_fgeneshDP_pg.C_460016 | scaffold_46 | 43926 | 45801 |
| Block 2073 | plbD | 4 | 1876303 | 1878098 | estExt_Genewise1.C_460030 | scaffold_46 | 41615 | 43535 |
| Block 183 | phaZ | 4 | 1884053 | 1885252 | GID1.0038100 | scaffold_12 | 6549 | 7593 |
| Block 1254 | DDB_G0284361 | 4 | 1888546 | 1892124 | GID1.0039091 | scaffold_28 | 11352 | 14761 |
| Block 1254 | cf60 | 4 | 1892663 | 1894210 | estExt_fgeneshDP_pg.C_280005 | scaffold_28 | 15182 | 16977 |
| Block 1254 | DDB_G0284365 | 4 | 1895020 | 1895769 | estExt_fgeneshDP_pm.C_280004 | scaffold_28 | 17189 | 18108 |
| Block 1254 | DDB_G0284367 | 4 | 1896160 | 1897120 | fgeneshDP_pg.C_scaffold_28000007 | scaffold_28 | 18792 | 19660 |
| Block 1254 | DDB_G0284371 | 4 | 1900513 | 1901488 | e_gw1.28.67.1 | scaffold_28 | 22100 | 23119 |
| Block 183 | DDB_G0284455 | 4 | 1906991 | 1909550 | fgeneshDP_pg.C_scaffold_12000007 | scaffold_12 | 15105 | 16998 |
| Block 183 | DDB_G0284377 | 4 | 1909801 | 1912062 | estExt_Genewise1Plus.C_120016 | scaffold_12 | 18284 | 19946 |
| Block 183 | xpo5 | 4 | 1912156 | 1915705 | GID1.0038108 | scaffold_12 | 19975 | 23582 |
| Block 183 | DDB_G0284381 | 4 | 1916862 | 1920131 | e_gw1.12.41.1 | scaffold_12 | 25157 | 28240 |
| Block 2073 | DDB_G0284383 | 4 | 1920700 | 1923303 | e_gw1.46.20.1 | scaffold_46 | 62753 | 65248 |
| Block 2073 | syn10 | 4 | 1923544 | 1924484 | estExt_fgeneshDP_pm.C_460012 | scaffold_46 | 61340 | 62435 |
| Block 2073 | gefA | 4 | 1926792 | 1928739 | estExt_Genewise1.C_460034 | scaffold_46 | 51413 | 54043 |
| Block 2104 | lvsF | 4 | 1946519 | 1950085 | estExt_Genewise1Plus.C_470008 | scaffold_47 | 19930 | 23478 |
| Block 2104 | mybY | 4 | 1950553 | 1952517 | estExt_fgeneshDP_pg.C_470011 | scaffold_47 | 24201 | 26017 |
| Block 2419 | phf5a | 4 | 1954324 | 1955003 | fgeneshDP_pg.C_scaffold_565000004 | scaffold_565 | 11025 | 11659 |
| Block 2419 | mrpl15 | 4 | 1955406 | 1956629 | fgeneshDP_pg.C_scaffold_565000003 | scaffold_565 | 9225 | 10370 |
| Block 2104 | regA | 4 | 1956975 | 1959735 | estExt_fgeneshDP_pg.C_470013 | scaffold_47 | 29347 | 31847 |
| Block 2104 | erg24 | 4 | 1962362 | 1963750 | estExt_fgeneshDP_pm.C_470007 | scaffold_47 | 25986 | 27566 |
| Block 473 | dng1 | 4 | 1965235 | 1966608 | estExt_Genewise1.C_1540016 | scaffold_154 | 23618 | 24903 |
| Block 473 | DDB_G0284413 | 4 | 1967001 | 1967573 | fgeneshDP_pg.C_scaffold_154000009 | scaffold_154 | 22681 | 23253 |
| Block 473 | psmC3 | 4 | 1968066 | 1969603 | estExt_Genewise1Plus.C_1540011 | scaffold_154 | 20625 | 22137 |
| Block 473 | ercc4 | 4 | 1971161 | 1974139 | estExt_Genewise1.C_1540009 | scaffold_154 | 17566 | 20488 |
| Block 512 | DDB_G0284457 | 4 | 1974366 | 1975576 | GID1.0044123 | scaffold_160 | 45750 | 47015 |
| Block 512 | med6 | 4 | 1975693 | 1976671 | GID1.0044115 | scaffold_160 | 31756 | 32651 |
| Block 473 | udpA | 4 | 1983105 | 1983854 | fgeneshDP_pg.C_scaffold_154000022 | scaffold_154 | 53230 | 53979 |
| Block 2104 | DDB_G0284433 | 4 | 1984663 | 1986076 | estExt_Genewise1.C_470007 | scaffold_47 | 18192 | 19565 |
| Block 2104 | pigB | 4 | 1990910 | 1992815 | GID1.0040081 | scaffold_47 | 12205 | 17405 |
| Block 2104 | DDB_G0284437 | 4 | 1993267 | 1994253 | e_gw1.47.66.1 | scaffold_47 | 7496 | 8554 |
| Block 2104 | DDB_G0284439 | 4 | 1994656 | 1997578 | GID1.0040079 | scaffold_47 | 8805 | 11293 |
| Block 71 | DDB_G0284461 | 4 | 2003252 | 2005552 | estExt_Genewise1Plus.C_1060001 | scaffold_106 | 822 | 3371 |
| Block 71 | maea | 4 | 2005913 | 2007284 | GID1.0042464 | scaffold_106 | 3789 | 5037 |
| Block 71 | DDB_G0284475 | 4 | 2010250 | 2017993 | GID1.0042465 | scaffold_106 | 5246 | 12651 |
| Block 71 | DDB_G0284481 | 4 | 2027462 | 2030822 | e_gw1.106.31.1 | scaffold_106 | 13316 | 16427 |
| Block 662 | DDB_G0284485 | 4 | 2034779 | 2035915 | estExt_Genewise1.C_1840002 | scaffold_184 | 2534 | 4066 |
| Block 662 | pdkB | 4 | 2038480 | 2041297 | e_gw1.184.8.1 | scaffold_184 | 4870 | 5935 |
| Block 662 | DDB_G0284491 | 4 | 2041403 | 2045127 | e_gw1.184.18.1 | scaffold_184 | 20822 | 24316 |
| Block 662 | DDB_G0284493 | 4 | 2045896 | 2047208 | e_gw1.184.33.1 | scaffold_184 | 19353 | 20600 |
| Block 662 | ragC | 4 | 2047442 | 2049155 | fgeneshDP_pg.C_scaffold_184000008 | scaffold_184 | 17672 | 19059 |
| Block 999 | gxcQ | 4 | 2058245 | 2061757 | GID1.0045802 | scaffold_231 | 35134 | 38710 |
| Block 999 | sf3b2 | 4 | 2068357 | 2070234 | GID1.0045801 | scaffold_231 | 32435 | 34286 |
| Block 999 | DDB_G0284507 | 4 | 2070281 | 2071515 | gw1.231.31.1 | scaffold_231 | 31323 | 32209 |
| Block 735 | DDB_G0284509 | 4 | 2071986 | 2073330 | GID1.0045002 | scaffold_196 | 11986 | 12310 |
| Block 735 | DDB_G0284513 | 4 | 2079840 | 2084303 | GID1.0045000 | scaffold_196 | 4587 | 7923 |
| Block 2483 | psmC6 | 4 | 2090141 | 2091556 | estExt_fgeneshDP_pg.C_590031 | scaffold_59 | 89202 | 90674 |
| Block 662 | cudA | 4 | 2091815 | 2094223 | estExt_fgeneshDP_pg.C_1840005 | scaffold_184 | 12647 | 14684 |
| Block 662 | forI | 4 | 2097497 | 2100503 | e_gw1.184.21.1 | scaffold_184 | 7043 | 9768 |
| Block 1923 | DDB_G0284521 | 4 | 2100599 | 2101369 | estExt_fgeneshDP_kg.C_420009 | scaffold_42 | 118545 | 119432 |
| Block 1923 | DDB_G0284523 | 4 | 2102761 | 2103650 | fgeneshDP_pg.C_scaffold_42000040 | scaffold_42 | 117021 | 117806 |
| Block 2483 | DDB_G0284557 | 4 | 2110678 | 2111280 | fgeneshDP_pg.C_scaffold_59000030 | scaffold_59 | 88566 | 89132 |
| Block 662 | DDB_G0284525 | 4 | 2111778 | 2113587 | GID1.0044731 | scaffold_184 | 14941 | 16583 |
| Block 2069 | DDB_G0284559 | 4 | 2123220 | 2124500 | GID1.0040045 | scaffold_46 | 32961 | 34178 |
| Block 2069 | rps13 | 4 | 2127182 | 2127908 | estExt_Genewise1Plus.C_460016 | scaffold_46 | 31649 | 32402 |
| Block 2069 | gpaH | 4 | 2128349 | 2129813 | estExt_fgeneshDP_kg.C_460002 | scaffold_46 | 29545 | 31412 |
| Block 2069 | DDB_G0284561 | 4 | 2131768 | 2134716 | GID1.0040042 | scaffold_46 | 24950 | 27685 |
| Block 2069 | cwc2 | 4 | 2134830 | 2136505 | estExt_Genewise1Plus.C_460008 | scaffold_46 | 23299 | 24852 |
| Block 2418 | DDB_G0284537 | 4 | 2139831 | 2140908 | GID1.0050098 | scaffold_565 | 7311 | 8350 |
| Block 2418 | DDB_G0284565 | 4 | 2142583 | 2147590 | fgeneshDP_pg.C_scaffold_565000001 | scaffold_565 | 983 | 6104 |
| Block 2576 | DDB_G0284579 | 4 | 2163129 | 2166028 | estExt_Genewise1.C_6240005 | scaffold_624 | 5369 | 11302 |
| Block 2576 | DDB_G0284569 | 4 | 2167100 | 2169809 | estExt_Genewise1Plus.C_6240002 | scaffold_624 | 1482 | 4984 |
| Block 737 | DDB_G0284577 | 4 | 2176004 | 2178172 | GID1.0045017 | scaffold_196 | 53217 | 55180 |
| Block 737 | dlcA | 4 | 2178851 | 2179736 | fgeneshDP_pg.C_scaffold_196000019 | scaffold_196 | 55792 | 56403 |
| Block 737 | DDB_G0284581 | 4 | 2180042 | 2181240 | GID1.0045019 | scaffold_196 | 56606 | 57668 |
| Block 1583 | glyS | 4 | 2181616 | 2183756 | GID1.0047800 | scaffold_342 | 10571 | 12708 |
| Block 1583 | fslL | 4 | 2184288 | 2186464 | fgeneshDP_pm.C_scaffold_342000002 | scaffold_342 | 4135 | 6199 |
| Block 737 | DDB_G0284587 | 4 | 2187151 | 2188593 | GID1.0045020 | scaffold_196 | 57982 | 58915 |
| Block 1482 | CSN5 | 4 | 2205667 | 2207051 | e_gw1.321.25.1 | scaffold_321 | 661 | 2620 |
| Block 1482 | csnk2b | 4 | 2209637 | 2210617 | GID1.0047463 | scaffold_321 | 5300 | 6133 |
| Block 1482 | DDB_G0284603 | 4 | 2211789 | 2213620 | fgeneshDP_pg.C_scaffold_321000004 | scaffold_321 | 7233 | 8714 |
| Block 737 | DDB_G0284605 | 4 | 2214347 | 2217243 | estExt_Genewise1.C_1960030 | scaffold_196 | 46015 | 49256 |
| Block 2398 | DDB_G0284619 | 4 | 2237635 | 2238396 | estExt_fgeneshDP_kg.C_5580001 | scaffold_558 | 5294 | 6278 |
| Block 2398 | pwp2 | 4 | 2240813 | 2244334 | GID1.0050048 | scaffold_558 | 16 | 3323 |
| Block 737 | DDB_G0284627 | 4 | 2260399 | 2261655 | e_gw1.196.32.1 | scaffold_196 | 40852 | 42159 |
| Block 1546 | DDB_G0284641 | 4 | 2274398 | 2274878 | estExt_fgeneshDP_pm.C_3370001 | scaffold_337 | 1837 | 2443 |
| Block 1546 | D7 | 4 | 2275277 | 2278014 | estExt_fgeneshDP_kg.C_3370003 | scaffold_337 | 2571 | 5290 |
| Block 1546 | DDB_G0284643 | 4 | 2279720 | 2280877 | GID1.0047720 | scaffold_337 | 6848 | 7987 |
| Block 1546 | CYP517A2 | 4 | 2285672 | 2287360 | GID1.0047722 | scaffold_337 | 9468 | 11775 |
| Block 190 | DDB_G0284649 | 4 | 2287653 | 2289871 | fgeneshDP_pg.C_scaffold_12000064 | scaffold_12 | 159577 | 161673 |
| Block 1253 | gcsA | 4 | 2291954 | 2294131 | estExt_fgeneshDP_pg.C_280001 | scaffold_28 | 212 | 2849 |
| Block 1253 | DDB_G0284725 | 4 | 2297325 | 2303099 | e_gw1.28.2.1 | scaffold_28 | 3924 | 9270 |
| Block 1850 | DDB_G0284727 | 4 | 2313169 | 2315723 | estExt_Genewise1.C_4020001 | scaffold_402 | 5 | 2918 |
| Block 1850 | mrd1 | 4 | 2315842 | 2318657 | e_gw1.402.4.1 | scaffold_402 | 3017 | 5740 |
| Block 1850 | pigF | 4 | 2319957 | 2320799 | fgeneshDP_pg.C_scaffold_402000003 | scaffold_402 | 6119 | 7017 |
| Block 1850 | prsA | 4 | 2322296 | 2323334 | estExt_Genewise1Plus.C_4020006 | scaffold_402 | 8437 | 9736 |
| Block 478 | fslK | 4 | 2327899 | 2330183 | GID1.0043960 | scaffold_154 | 56600 | 58621 |
| Block 478 | expl2 | 4 | 2331829 | 2333137 | estExt_Genewise1Plus.C_1540045 | scaffold_154 | 59571 | 61173 |
| Block 478 | DDB_G0284679 | 4 | 2333236 | 2334456 | fgeneshDP_pm.C_scaffold_154000013 | scaffold_154 | 61214 | 62368 |
| Block 478 | DDB_G0284681 | 4 | 2334842 | 2335972 | estExt_Genewise1Plus.C_1540048 | scaffold_154 | 62724 | 63860 |
| Block 190 | DDB_G0284683 | 4 | 2336209 | 2339112 | GID1.0038167 | scaffold_12 | 145758 | 150902 |
| Block 190 | mpi | 4 | 2341606 | 2343049 | e_gw1.12.56.1 | scaffold_12 | 143945 | 145335 |
| Block 190 | DDB_G0284687 | 4 | 2343826 | 2345747 | GID1.0038155 | scaffold_12 | 116666 | 118610 |
| Block 190 | ripA | 4 | 2346926 | 2349655 | estExt_fgeneshDP_pg.C_120044 | scaffold_12 | 112750 | 115869 |
| Block 190 | DDB_G0284689 | 4 | 2350414 | 2355543 | GID1.0038153 | scaffold_12 | 107594 | 112474 |
| Block 190 | DDB_G0284691 | 4 | 2357079 | 2358174 | GID1.0038152 | scaffold_12 | 105146 | 106137 |
| Block 892 | DDB_G0284695 | 4 | 2359622 | 2360974 | GID1.0045446 | scaffold_215 | 16727 | 17626 |
| Block 892 | hemC | 4 | 2361299 | 2362276 | GID1.0045445 | scaffold_215 | 15407 | 16521 |
| Block 892 | mtr | 4 | 2362420 | 2366575 | estExt_fgeneshDP_pg.C_2150005 | scaffold_215 | 11056 | 15360 |
| Block 892 | DDB_G0284701 | 4 | 2371416 | 2372808 | GID1.0045442 | scaffold_215 | 2568 | 6906 |
| Block 301 | polB | 4 | 2376164 | 2378598 | e_gw1.132.10.1 | scaffold_132 | 46652 | 48208 |
| Block 301 | DDB_G0284705 | 4 | 2378839 | 2380249 | e_gw1.132.31.1 | scaffold_132 | 44930 | 46391 |
| Block 301 | DDB_G0284731 | 4 | 2380797 | 2383385 | fgeneshDP_pg.C_scaffold_132000013 | scaffold_132 | 42164 | 44356 |
| Block 301 | DDB_G0284707 | 4 | 2388566 | 2389651 | GID1.0043299 | scaffold_132 | 36583 | 37793 |
| Block 301 | copB | 4 | 2394561 | 2397811 | estExt_Genewise1.C_1320011 | scaffold_132 | 16388 | 19418 |
| Block 301 | DDB_G0284737 | 4 | 2397942 | 2398964 | GID1.0043297 | scaffold_132 | 32522 | 33465 |
| Block 301 | gxcFF | 4 | 2399480 | 2401787 | GID1.0043294 | scaffold_132 | 19721 | 21973 |
| Block 736 | 4cl1 | 4 | 2416042 | 2417784 | e_gw1.196.24.1 | scaffold_196 | 38737 | 40475 |
| Block 736 | exoc4 | 4 | 2418843 | 2422835 | e_gw1.196.19.1 | scaffold_196 | 34075 | 37868 |
| Block 736 | msh5 | 4 | 2423376 | 2426473 | fgeneshDP_pm.C_scaffold_196000007 | scaffold_196 | 30912 | 33793 |
| Block 301 | DDB_G0284749 | 4 | 2426750 | 2430729 | estExt_fgeneshDP_pg.C_1320009 | scaffold_132 | 28714 | 32501 |
| Block 2362 | DDB_G0284753 | 4 | 2432933 | 2434591 | estExt_Genewise1.C_5420008 | scaffold_542 | 10225 | 11784 |
| Block 2362 | DDB_G0284757 | 4 | 2437158 | 2439674 | estExt_fgeneshDP_pg.C_5420006 | scaffold_542 | 12384 | 14950 |
| Block 2362 | fslA | 4 | 2445394 | 2447839 | GID1.0049959 | scaffold_542 | 8094 | 10139 |
| Block 2362 | aass | 4 | 2456087 | 2459084 | estExt_Genewise1.C_5420001 | scaffold_542 | 546 | 3803 |
| Block 275 | DDB_G0284799 | 4 | 2498543 | 2499047 | GID1.0038197 | scaffold_13 | 57541 | 58290 |
| Block 275 | DDB_G0284801 | 4 | 2499782 | 2500150 | e_gw1.13.128.1 | scaffold_13 | 58870 | 59286 |
| Block 1647 | DDB_G0284807 | 4 | 2501891 | 2505360 | e_gw1.358.1.1 | scaffold_358 | 10580 | 13836 |
| Block 1647 | DDB_G0284809 | 4 | 2506040 | 2506788 | GID1.0048035 | scaffold_358 | 14459 | 15137 |
| Block 1647 | DDB_G0284811 | 4 | 2507767 | 2508648 | GID1.0048036 | scaffold_358 | 15629 | 16398 |
| Block 1647 | DDB_G0284813 | 4 | 2508788 | 2509215 | e_gw1.358.15.1 | scaffold_358 | 16568 | 16768 |
| Block 1647 | DDB_G0284841 | 4 | 2510140 | 2513041 | estExt_fgeneshDP_pg.C_3580008 | scaffold_358 | 17985 | 20572 |
| Block 275 | DDB_G0284843 | 4 | 2515068 | 2515796 | estExt_fgeneshDP_kg.C_130016 | scaffold_13 | 115936 | 116991 |
| Block 585 | DDB_G0284819 | 4 | 2520967 | 2522046 | estExt_Genewise1Plus.C_1710020 | scaffold_171 | 37520 | 38846 |
| Block 585 | DDB_G0284825 | 4 | 2526025 | 2531286 | GID1.0044407 | scaffold_171 | 16091 | 21284 |
| Block 3048 | abcC8 | 4 | 2579815 | 2584663 | estExt_Genewise1.C_970030 | scaffold_97 | 46617 | 51619 |
| Block 3048 | DDB_G0284881 | 4 | 2585806 | 2586592 | fgeneshDP_pg.C_scaffold_97000021 | scaffold_97 | 58011 | 58662 |
| Block 3048 | oxaA | 4 | 2586982 | 2588331 | gw1.97.22.1 | scaffold_97 | 58910 | 60043 |
| Block 3048 | DDB_G0284885 | 4 | 2588526 | 2590107 | estExt_fgeneshDP_kg.C_970006 | scaffold_97 | 69809 | 72344 |
| Block 3048 | DDB_G0285049 | 4 | 2595117 | 2596316 | fgeneshDP_pg.C_scaffold_97000025 | scaffold_97 | 66331 | 67330 |
| Block 2833 | rab32D | 4 | 2597422 | 2598751 | estExt_Genewise1.C_770005 | scaffold_77 | 4498 | 5188 |
| Block 2833 | DDB_G0284887 | 4 | 2599094 | 2600545 | estExt_Genewise1Plus.C_770001 | scaffold_77 | 2576 | 3873 |
| Block 2833 | DDB_G0284889 | 4 | 2600817 | 2602140 | fgeneshDP_pm.C_scaffold_77000001 | scaffold_77 | 588 | 1826 |
| Block 3048 | culC | 4 | 2621558 | 2624319 | GID1.0042164 | scaffold_97 | 72611 | 75129 |
| Block 3048 | ap4s1 | 4 | 2625777 | 2626566 | e_gw1.97.36.1 | scaffold_97 | 76185 | 76833 |
| Block 3048 | DDB_G0284907 | 4 | 2626851 | 2627138 | estExt_Genewise1Plus.C_970036 | scaffold_97 | 62850 | 63266 |
| Block 2833 | orf2034 | 4 | 2629450 | 2630669 | estExt_fgeneshDP_pm.C_770002 | scaffold_77 | 5540 | 6662 |
| Block 2833 | timm10 | 4 | 2631200 | 2631555 | e_gw1.77.61.1 | scaffold_77 | 6926 | 7355 |
| Block 226 | DDB_G0285055 | 4 | 2631798 | 2637863 | e_gw1.123.9.1 | scaffold_123 | 55412 | 61055 |
| Block 226 | DDB_G0284913 | 4 | 2638616 | 2643148 | e_gw1.123.11.1 | scaffold_123 | 50193 | 54443 |
| Block 226 | DDB_G0284915 | 4 | 2643291 | 2644175 | e_gw1.123.26.1 | scaffold_123 | 49259 | 50032 |
| Block 226 | DDB_G0284919 | 4 | 2646442 | 2647519 | fgeneshDP_pg.C_scaffold_123000018 | scaffold_123 | 40693 | 42215 |
| Block 226 | DDB_G0284921 | 4 | 2648206 | 2649720 | GID1.0043042 | scaffold_123 | 43288 | 44907 |
| Block 1531 | DDB_G0285057 | 4 | 2666993 | 2667683 | e_gw1.332.18.1 | scaffold_332 | 32162 | 32675 |
| Block 1531 | DDB_G0284939 | 4 | 2667885 | 2668401 | estExt_Genewise1.C_3320021 | scaffold_332 | 31396 | 31866 |
| Block 1531 | DDB_G0284941 | 4 | 2668956 | 2669455 | GID1.0047655 | scaffold_332 | 30570 | 31086 |
| Block 1531 | DDB_G0284943 | 4 | 2669585 | 2671865 | e_gw1.332.6.1 | scaffold_332 | 22293 | 23736 |
| Block 1531 | fsjC | 4 | 2672551 | 2675180 | estExt_Genewise1Plus.C_3320016 | scaffold_332 | 18598 | 21216 |
| Block 1531 | ucr | 4 | 2675998 | 2677138 | fgeneshDP_pg.C_scaffold_332000005 | scaffold_332 | 16457 | 17691 |
| Block 1531 | oplah | 4 | 2680203 | 2684167 | estExt_Genewise1.C_3320004 | scaffold_332 | 8647 | 12502 |
| Block 1452 | DDB_G0285059 | 4 | 2696768 | 2698214 | fgeneshDP_pg.C_scaffold_314000009 | scaffold_314 | 15773 | 16987 |
| Block 1452 | DDB_G0284959 | 4 | 2698477 | 2699183 | gw1.314.15.1 | scaffold_314 | 17257 | 17797 |
| Block 1452 | DDB_G0284961 | 4 | 2699345 | 2699985 | GID1.0047363 | scaffold_314 | 18197 | 18815 |
| Block 1452 | DDB_G0284963 | 4 | 2700406 | 2702424 | fgeneshDP_pm.C_scaffold_314000006 | scaffold_314 | 19198 | 20975 |
| Block 1452 | DDB_G0284969 | 4 | 2719814 | 2721577 | gw1.314.2.1 | scaffold_314 | 33701 | 35110 |
| Block 1452 | DDB_G0285065 | 4 | 2722034 | 2723623 | estExt_Genewise1.C_3140022 | scaffold_314 | 35396 | 37097 |
| Block 1166 | DDB_G0284971 | 4 | 2726690 | 2727950 | fgeneshDP_pg.C_scaffold_260000001 | scaffold_260 | 187 | 1185 |
| Block 1166 | exoc1 | 4 | 2728155 | 2731002 | fgeneshDP_pm.C_scaffold_260000001 | scaffold_260 | 1427 | 4090 |
| Block 2835 | tpsB | 4 | 2735507 | 2737979 | estExt_Genewise1.C_770046 | scaffold_77 | 71369 | 74134 |
| Block 2835 | DDB_G0284977 | 4 | 2738670 | 2742984 | estExt_fgeneshDP_pg.C_770015 | scaffold_77 | 38574 | 42483 |
| Block 2835 | DDB_G0284979 | 4 | 2743512 | 2744492 | GID1.0041395 | scaffold_77 | 43100 | 43979 |
| Block 2835 | DDB_G0284981 | 4 | 2745658 | 2747307 | fgeneshDP_pg.C_scaffold_77000019 | scaffold_77 | 47407 | 48594 |
| Block 2835 | btg | 4 | 2748189 | 2749460 | estExt_fgeneshDP_kg.C_770005 | scaffold_77 | 52318 | 53730 |
| Block 2835 | echs1 | 4 | 2750152 | 2751628 | estExt_Genewise1Plus.C_770033 | scaffold_77 | 54857 | 56013 |
| Block 2835 | colD | 4 | 2752867 | 2755644 | estExt_fgeneshDP_pg.C_770023 | scaffold_77 | 57098 | 60547 |
| Block 2835 | rab1D | 4 | 2756032 | 2757408 | estExt_Genewise1Plus.C_770038 | scaffold_77 | 60534 | 62244 |
| Block 2835 | DDB_G0285073 | 4 | 2758636 | 2761135 | GID1.0041406 | scaffold_77 | 64950 | 67119 |
| Block 2835 | repG | 4 | 2762877 | 2764154 | e_gw1.77.35.1 | scaffold_77 | 67358 | 68596 |
| Block 2835 | eif5a | 4 | 2766075 | 2766554 | estExt_fgeneshDP_kg.C_770010 | scaffold_77 | 70388 | 70987 |
| Block 806 | DDB_G0284995 | 4 | 2782622 | 2783781 | estExt_Genewise1.C_2020020 | scaffold_202 | 35436 | 36707 |
| Block 806 | DDB_G0284997 | 4 | 2784039 | 2785261 | estExt_fgeneshDP_pm.C_2020007 | scaffold_202 | 37061 | 38589 |
| Block 1451 | DDB_G0285007 | 4 | 2796145 | 2797245 | estExt_fgeneshDP_pg.C_3140003 | scaffold_314 | 5752 | 6683 |
| Block 1451 | u2af1 | 4 | 2799520 | 2801698 | estExt_Genewise1Plus.C_3140007 | scaffold_314 | 9870 | 12062 |
| Block 1451 | DDB_G0285079 | 4 | 2802048 | 2802865 | GID1.0047359 | scaffold_314 | 12375 | 13182 |
| Block 1451 | DDB_G0285095 | 4 | 2804851 | 2805754 | GID1.0047360 | scaffold_314 | 13390 | 15348 |
| Block 2835 | DDB_G0285011 | 4 | 2814046 | 2815374 | estExt_Genewise1Plus.C_770018 | scaffold_77 | 28596 | 30167 |
| Block 2835 | DDB_G0285083 | 4 | 2815942 | 2819113 | GID1.0041390 | scaffold_77 | 24713 | 27802 |
| Block 222 | DDB_G0285013 | 4 | 2820652 | 2821932 | e_gw1.123.21.1 | scaffold_123 | 29549 | 30634 |
| Block 222 | DDB_G0285021 | 4 | 2830332 | 2830877 | estExt_fgeneshDP_pm.C_1230007 | scaffold_123 | 35144 | 35865 |
| Block 2701 | alrE | 4 | 2832495 | 2833482 | estExt_fgeneshDP_pg.C_680031 | scaffold_68 | 81161 | 82238 |
| Block 2701 | alrD | 4 | 2833891 | 2834847 | e_gw1.68.53.1 | scaffold_68 | 79159 | 80187 |
| Block 2701 | sfxn | 4 | 2834988 | 2836314 | fgeneshDP_pg.C_scaffold_68000034 | scaffold_68 | 91699 | 93030 |
| Block 2701 | DDB_G0285031 | 4 | 2836987 | 2839374 | fgeneshDP_pg.C_scaffold_68000035 | scaffold_68 | 93611 | 96232 |
| Block 2835 | DDB_G0285033 | 4 | 2839925 | 2840710 | fgeneshDP_pg.C_scaffold_77000014 | scaffold_77 | 37122 | 38074 |
| Block 1255 | DDB_G0285103 | 4 | 2851187 | 2852364 | estExt_Genewise1.C_280017 | scaffold_28 | 23772 | 25482 |
| Block 1255 | psmD13 | 4 | 2852779 | 2854360 | estExt_Genewise1.C_280018 | scaffold_28 | 25766 | 27235 |
| Block 1483 | snpA | 4 | 2863727 | 2864850 | estExt_Genewise1Plus.C_3210014 | scaffold_321 | 11222 | 12630 |
| Block 1483 | DDB_G0285113 | 4 | 2865898 | 2867728 | GID1.0047465 | scaffold_321 | 8814 | 10578 |
| Block 713 | rad54b | 4 | 2873116 | 2876085 | e_gw1.193.1.1 | scaffold_193 | 19758 | 22026 |
| Block 713 | DDB_G0285119 | 4 | 2877924 | 2879489 | estExt_fgeneshDP_pg.C_1930007 | scaffold_193 | 15420 | 16921 |
| Block 713 | psmE3 | 4 | 2880074 | 2881142 | e_gw1.193.36.1 | scaffold_193 | 10893 | 11814 |
| Block 2779 | DDB_G0285123 | 4 | 2882150 | 2884484 | GID1.0041230 | scaffold_73 | 37267 | 39523 |
| Block 713 | DDB_G0285125 | 4 | 2884598 | 2886443 | estExt_fgeneshDP_pm.C_1930006 | scaffold_193 | 22066 | 23922 |
| Block 2779 | derl2 | 4 | 2896240 | 2897333 | e_gw1.73.21.1 | scaffold_73 | 44844 | 45625 |
| Block 2779 | stt3 | 4 | 2899851 | 2902292 | GID1.0041232 | scaffold_73 | 41597 | 43917 |
| Block 2779 | mocs2s | 4 | 2902514 | 2902867 | e_gw1.73.70.1 | scaffold_73 | 58323 | 58658 |
| Block 2779 | DDB_G0285135 | 4 | 2903243 | 2903902 | fgeneshDP_pg.C_scaffold_73000026 | scaffold_73 | 63587 | 64243 |
| Block 2779 | mocs1 | 4 | 2905081 | 2907503 | e_gw1.73.15.1 | scaffold_73 | 64839 | 66150 |
| Block 2779 | gtaL | 4 | 2908294 | 2910216 | fgeneshDP_pg.C_scaffold_73000029 | scaffold_73 | 67733 | 69301 |
| Block 2779 | osbJ | 4 | 2910487 | 2911895 | GID1.0041245 | scaffold_73 | 69462 | 70777 |
| Block 2779 | DDB_G0285143 | 4 | 2913006 | 2914361 | e_gw1.73.49.1 | scaffold_73 | 90549 | 91869 |
| Block 713 | DDB_G0285145 | 4 | 2915991 | 2917295 | fgeneshDP_pm.C_scaffold_193000002 | scaffold_193 | 6610 | 7761 |
| Block 2779 | DDB_G0285167 | 4 | 2919389 | 2920363 | estExt_fgeneshDP_kg.C_730004 | scaffold_73 | 54130 | 55464 |
| Block 2779 | DDB_G0285205 | 4 | 2921996 | 2926921 | estExt_fgeneshDP_pg.C_730022 | scaffold_73 | 49372 | 53315 |
| Block 1450 | vmp1 | 4 | 2937306 | 2938857 | fgeneshDP_pg.C_scaffold_314000002 | scaffold_314 | 3739 | 5325 |
| Block 1450 | DDB_G0285177 | 4 | 2938952 | 2940437 | fgeneshDP_pm.C_scaffold_314000001 | scaffold_314 | 2112 | 3524 |
| Block 2779 | DDB_G0285203 | 4 | 2941521 | 2941979 | GID1.0041253 | scaffold_73 | 88573 | 88982 |
| Block 2779 | gacT | 4 | 2946825 | 2949854 | estExt_fgeneshDP_pg.C_730037 | scaffold_73 | 85127 | 88236 |
| Block 135 | DDB_G0285209 | 4 | 2951533 | 2952479 | fgeneshDP_pm.C_scaffold_114000002 | scaffold_114 | 2699 | 3561 |
| Block 135 | gpn3 | 4 | 2952647 | 2953733 | e_gw1.114.34.1 | scaffold_114 | 1409 | 2478 |
| Block 2834 | DDB_G0285183 | 4 | 2957139 | 2960755 | GID1.0041387 | scaffold_77 | 19057 | 21008 |
[truncated: 208,483 more chars]
