# Supplementary material for: Comparative genomics of the social amoebae Dictyostelium discoideum and Dictyostelium purpureum
Source: Genome Biol. 2011 Feb 28;12(2):R20. doi: 10.1186/gb-2011-12-2-r20 (PMC3188802; doi:10.1186/gb-2011-12-2-r20)
Supplement: Additional file 3 — Supplementary Table S4. A table listing the predicted orthologs that are shared between D. discoideum and D. purpureum. [file gb-2011-12-2-r20-S3.DOC]

| **source** | **jgi_Protein I.D.** | **Dictybase I.D.** | **D. discoideum genename** | **annotation for D. discoideum gene** |
| --- | --- | --- | --- | --- |
| curated | 100074 | DDB_G0270762 | fimD | actin binding protein, calponin homology (CH) domain-containing protein, fimbrin-4 |
| curated | 10267 | DDB_G0267568 | gacO | RhoGAP domain-containing protein |
| curated | 12667 | DDB_G0284261 | nxnB | annexin I, putative actin binding protein |
| curated | 146520 | DDB_G0274243 | gacF | RhoGAP domain-containing protein |
| curated | 147341 | DDB_G0292560 | racJ | Rho GTPase |
| curated | 147379 | DDB_G0292068 | frpA | actin binding protein, calponin homology (CH) domain-containing protein, fimbrin-related protein, fimbrin-related actin binding protein |
| curated | 147382 | DDB_G0292100 | fscH | G-protein-coupled receptor (GPCR) family protein, frizzled and smoothened-like sans CRD protein |
| curated | 147510 | DDB_G0272694 | gacH | RhoGAP domain-containing protein |
| curated | 147802 | DDB_G0282891 | pakG | putative protein serine/threonine kinase, STE20 family protein kinase, protein kinase, STE group, PAKL subfamily protein kinase |
| curated | 148204 | DDB_G0289763 | forF | actin binding protein, formin homology domain-containing protein |
| curated | 148245 | DDB_G0269204 | dhkE | histidine kinase, protein kinase, Atypical group, HisK family protein kinase |
| curated | 148367 | DDB_G0291007 | gxcK | pleckstrin homology (PH) domain-containing protein, RhoGEF domain-containing protein |
| curated | 148817 | DDB_G0290493 | gxcHH | pleckstrin homology (PH) domain-containing protein, RhoGEF domain-containing protein, IQ calmodulin-binding domain-containing protein |
| curated | 148839 | DDB_G0272372 | gxcM | pleckstrin homology (PH) domain-containing protein, RhoGEF domain-containing protein, DH domain-containing protein |
| curated | 148964 | DDB_G0287505 | talB | talinB |
| curated | 149337 | DDB_G0276363 | myoG | unconventional myosin heavy chain |
| curated | 149388 | DDB_G0284035 | efa1B | elongation factor 1b, elongation factor 1 beta |
| curated | 149424 | DDB_G0291840 | gacEE | pleckstrin homology (PH) domain-containing protein, RhoGAP domain-containing protein, C2 calcium/lipid-binding (CaLB) region-containing protein |
| curated | 14945 | DDB_G0278545 | fimC | actin binding protein, calponin homology (CH) domain-containing protein, fimbrin-3 |
| curated | 149722 | DDB_G0268888 | DDB_G0268888 | DOCK family protein, putative guanine nucleotide exchange factor (GEF) |
| curated | 149813 | DDB_G0276617 | myoC | myosin IC, class I myosin |
| curated | 149892 | DDB_G0275011 | pikC | phosphatidylinositol-4,5-diphosphate 3-kinase, PI3kinase |
| curated | 149898 | DDB_G0275085 | gacFF | pleckstrin homology (PH) domain-containing protein, RhoGAP domain-containing protein, F-box-containing protein |
| curated | 150045 | DDB_G0277845 | dhkB | histidine kinase, protein kinase, Atypical group, HisK family protein kinase |
| curated | 150164 | DDB_G0288205 | gacX | RhoGAP domain-containing protein, WD40 repeat-containing protein |
| curated | 150581 | DDB_G0279607 | forA | actin binding protein, formin homology domain-containing protein |
| curated | 150789 | DDB_G0267428 | abcH2 | putative non-transporter ABC protein |
| curated | 150846 | DDB_G0274409 | pakF | putative protein serine/threonine kinase, STE20 family protein kinase, protein kinase, STE group, PAKL subfamily protein kinase |
| curated | 15107 | DDB_G0275697 | abcH3 | putative non-transporter ABC protein, metallophosphoesterase domain-containing protein |
| curated | 151807 | DDB_G0291356 | grlE | GABA-B receptor-like protein, metabotropic glutamate receptor-like protein, G-protein-coupled receptor (GPCR) family 3 protein 5 |
| curated | 151857 | DDB_G0279913 | dhkH | histidine kinase, protein kinase, Atypical group, HisK family protein kinase |
| curated | 151950 | DDB_G0281573 | gefP | Ras guanine nucleotide exchange factor |
| curated | 151994 | DDB_G0275035 | DDB_G0275035 | DOCK family protein, putative guanine nucleotide exchange factor (GEF) |
| curated | 152450 | DDB_G0282289 | dhkD | histidine kinase, protein kinase, Atypical group, HisK family protein kinase |
| curated | 152501 | DDB_G0275239 | DDB_G0275239 | DOCK family protein, putative guanine nucleotide exchange factor (GEF) |
| curated | 152547 | DDB_G0271686 | grlB | G-protein-coupled receptor (GPCR) family 3 protein 2 |
| curated | 152605 | DDB_G0284739 | gxcFF | pleckstrin homology (PH) domain-containing protein, RhoGEF domain-containing protein, IQ calmodulin-binding domain-containing protein |
| curated | 153271 | DDB_G0284729 | fslK | G-protein-coupled receptor (GPCR) family protein, frizzled and smoothened-like protein |
| curated | 153285 | DDB_G0278147 | gxcW | pleckstrin homology (PH) domain-containing protein, RhoGEF domain-containing protein, FYVE-type zinc finger-containing protein |
| curated | 153400 | DDB_G0290873 | gacJJ | pleckstrin homology (PH) domain-containing protein, SH3 domain-containing protein, RhoGAP domain-containing protein |
| curated | 153584 | DDB_G0282073 | gxcG | pleckstrin homology (PH) domain-containing protein, RhoGEF domain-containing protein |
| curated | 153591 | DDB_G0282717 | gxcAA | pleckstrin homology (PH) domain-containing protein, RhoGEF domain-containing protein, IQ calmodulin-binding domain-containing protein, Phox domain-containing protein, calponin homology (CH) domain-containing protein |
| curated | 153663 | DDB_G0280961 | dhkA | histidine kinase, protein kinase, Atypical group, HisK family protein kinase |
| curated | 153700 | DDB_G0293084 | DDB_G0293084 | DOCK family protein, putative guanine nucleotide exchange factor (GEF) |
| curated | 153775 | DDB_G0274519 | napA | component of SCAR regulatory complex |
| curated | 153797 | DDB_G0277175 | forG | actin binding protein, formin homology domain-containing protein |
| curated | 153891 | DDB_G0293654 | gacE | RhoGAP domain-containing protein |
| curated | 154316 | DDB_G0269626 | forE | actin binding protein, formin homology domain-containing protein |
| curated | 154327 | DDB_G0291728 | arpE | actin related protein 5 |
| curated | 154751 | DDB_G0267376 | acrA | adenylyl cyclase, adenylate cyclase |
| curated | 155058 | DDB_G0270672 | fslN | frizzled and smoothened-like protein |
| curated | 155144 | DDB_G0284045 | dhkG | histidine kinase, HisK family protein kinase |
| curated | 155148 | DDB_G0284501 | gxcQ | pleckstrin homology (PH) domain-containing protein, RhoGEF domain-containing protein |
| curated | 155154 | DDB_G0282377 | dhkM | histidine kinase, protein kinase, Atypical group, HisK family protein kinase |
| curated | 155202 | DDB_G0279009 | gacP | RhoGAP domain-containing protein |
| curated | 155336 | DDB_G0269696 | pakD | p21-activated protein kinase, putative protein serine/threonine kinase, STE20 family protein kinase, PKC domain-containing protein, PE/DAG binding, calponin homology (CH) domain-containing protein, protein kinase, STE group, PAKA subfamily protein kinase |
| curated | 155548 | DDB_G0287739 | arpH | actin related protein 11, actin related protein 10 |
| curated | 15574 | DDB_G0290439 | gacW | leucine-rich repeat-containing protein (LRR), RhoGAP domain-containing protein |
| curated | 155819 | DDB_G0289601 | pikE | phosphatidylinositol 3-kinase |
| curated | 156601 | DDB_G0274101 | dokA | histidine kinase, protein kinase, Atypical group |
| curated | 156926 | DDB_G0284845 | gxcC | pleckstrin homology (PH) domain-containing protein, RhoGEF domain-containing protein, armadillo repeat-containing protein |
| curated | 156963 | DDB_G0285589 | forH | actin binding protein, formin, diaphanous-related formin |
| curated | 157660 | DDB_G0279413 | pkaR | protein kinase A regulatory subunit |
| curated | 157695 | DDB_G0280039 | myoA | myosin IA heavy chain, class I myosin |
| curated | 157827 | DDB_G0289859 | kxcA | LISK family protein kinase, pleckstrin homology (PH) domain-containing protein, RhoGEF domain-containing protein, IQ calmodulin-binding domain-containing protein, protein kinase, TKL group, tyrosine kinase-like protein |
| curated | 157834 | DDB_G0282381 | gefC | regulator of chromosome condensation (RCC1) domain-containing protein, RhoGEF domain-containing protein, Ras nucleotide exchange factor |
| curated | 158102 | DDB_G0274889 | gxcX | pleckstrin homology (PH) domain-containing protein, RhoGEF domain-containing protein, FYVE-type zinc finger-containing protein |
| curated | 158106 | DDB_G0274191 | dhkC | histidine kinase, protein kinase, Atypical group, HisK family protein kinase |
| curated | 158218 | DDB_G0287779 | arpG | actin related protein 8 |
| curated | 158397 | DDB_G0279315 | gacN | RhoGAP domain-containing protein |
| curated | 158606 | DDB_G0282395 | gacQ | RhoGAP domain-containing protein |
| curated | 158993 | DDB_G0276833 | gacS | RhoGAP domain-containing protein |
| curated | 15906 | DDB_G0269386 | grlK | G-protein-coupled receptor (GPCR) family 3 protein 10 |
| curated | 159207 | DDB_G0282813 | arcB | actin related protein 2/3 complex, subunit 2, actin related protein 2/3 complex, 34 kDa subunit |
| curated | 159333 | DDB_G0284761 | fslA | G-protein-coupled receptor (GPCR) family protein, frizzled and smoothened-like protein |
| curated | 159347 | DDB_G0289177 | myoF | myosin IF, class I myosin |
| curated | 159361 | DDB_G0272244 | grlG | G-protein-coupled receptor (GPCR) family 3 protein 7 |
| curated | 159515 | DDB_G0292262 | myoM | calmodulin-binding protein, pleckstrin homology (PH) domain-containing protein, RhoGEF domain-containing protein, Rac guanyl-nucleotide exchange factor, unconventional myosin heavy chain |
| curated | 159516 | DDB_G0288749 | abkA | ABC1 family protein kinase, ABC1-A subfamily protein kinase, protein kinase, Atypical group |
| curated | 159700 | DDB_G0281739 | gacY | RhoGAP domain-containing protein |
| curated | 16140 | DDB_G0281211 | grlL | G-protein-coupled receptor (GPCR) family 3 protein 11 |
| curated | 16409 | DDB_G0281799 | abkB | putative protein serine/threonine kinase, ABC1 family protein kinase, protein kinase, Atypical group, ABC1-B subfamily protein kinase |
| curated | 17639 | DDB_G0274773 | fslH | G-protein-coupled receptor (GPCR) family protein, frizzled and smoothened-like protein |
| curated | 25874 | DDB_G0274575 | myoK | myosin IK, unconventional myosin heavy chain, class I myosin |
| curated | 26238 | DDB_G0292064 | fscF | G-protein-coupled receptor (GPCR) family protein, frizzled and smoothened-like sans CRD protein |
| curated | 26275 | DDB_G0291996 | gxcU | pleckstrin homology (PH) domain-containing protein, RhoGEF domain-containing protein, FYVE-type zinc finger-containing protein |
| curated | 26788 | DDB_G0293340 | gxcKK | pleckstrin homology (PH) domain-containing protein, RhoGEF domain-containing protein, IQ calmodulin-binding domain-containing protein |
| curated | 27726 | DDB_G0269424 | gxcB | pleckstrin homology (PH) domain-containing protein, RhoGEF domain-containing protein, calponin homology (CH) domain-containing protein |
| curated | 27977 | DDB_G0284725 | DDB_G0284725 | SH3 domain-containing protein, DOCK family protein, putative guanine nucleotide exchange factor (GEF) |
| curated | 28922 | DDB_G0270658 | acbA | acyl-CoA binding protein |
| curated | 29142 | DDB_G0278381 | gacA | RhoGAP domain-containing protein, papD-like domain-containing protein |
| curated | 30041 | DDB_G0267460 | cybA | p22-like superoxide-generating NADPH oxidase light subunit, flavocytochrome b small subunit |
| curated | 31335 | DDB_G0276459 | pakB | myosin heavy chain kinase, myosin I heavy chain kinase, p21-activated protein kinase, STE20 family protein kinase, protein kinase, STE group, PAKA subfamily protein kinase |
| curated | 31462 | DDB_G0292384 | abcH1 | putative non-transporter ABC protein |
| curated | 32111 | DDB_G0289653 | noxA | superoxide-generating NADPH oxidase flavocytochrome, flavocytochrome b large subunit |
| curated | 32330 | DDB_G0279123 | gxcCC | RhoGEF domain-containing protein, DH domain-containing protein, calponin homology (CH) domain-containing protein, putative actin binding protein |
| curated | 32515 | DDB_G0287507 | limD1 | LIM-type zinc finger-containing protein |
| curated | 33314 | DDB_G0271684 | grlA | G-protein-coupled receptor (GPCR) family 3 protein 1 |
| curated | 33732 | DDB_G0282271 | gxcV | pleckstrin homology (PH) domain-containing protein, RhoGEF domain-containing protein, FYVE-type zinc finger-containing protein |
| curated | 33735 | DDB_G0282297 | forB | actin binding protein, formin homology domain-containing protein |
| curated | 34447 | DDB_G0275447 | myoD | myosin ID heavy chain, class I myosin |
| curated | 34798 | DDB_G0270404 | DDB_G0270404 | DOCK family protein, putative guanine nucleotide exchange factor (GEF) |
| curated | 34997 | DDB_G0286643 | grlM | G-protein-coupled receptor (GPCR) family 3 protein 12 |
| curated | 35308 | DDB_G0277883 | dhkJ | histidine kinase, protein kinase, Atypical group, HisK family protein kinase |
| curated | 35373 | DDB_G0282625 | pikG | phosphatidylinositol 3-kinase, PI3kinase |
| curated | 36102 | DDB_G0284519 | forI | formin homology domain-containing protein |
| curated | 37984 | DDB_G0275635 | PIPkinA | ankyrin repeat-containing protein, phosphatidylinositol phosphate kinase |
| curated | 38686 | DDB_G0289599 | comA | actin binding protein, comitin, mannose binding lectin, B_lectin |
| curated | 39928 | DDB_G0293266 | gxcY | pleckstrin homology (PH) domain-containing protein, RhoGEF domain-containing protein, armadillo repeat-containing protein |
| curated | 40381 | DDB_G0288771 | darA | darlin, armadillo-like helical domain-containing protein |
| curated | 40920 | DDB_G0285253 | scrA | SCAR1 |
| curated | 41217 | DDB_G0269240 | racH | Rho GTPase |
| curated | 41248 | DDB_G0285859 | gxcP | pleckstrin homology (PH) domain-containing protein, RhoGEF domain-containing protein |
| curated | 41266 | DDB_G0287101 | noxB | superoxide-generating NADPH oxidase flavocytochrome, flavocytochrome b large subunit |
| curated | 41820 | DDB_G0286607 | fslP | G-protein-coupled receptor (GPCR) family protein, frizzled and smoothened-like protein |
| curated | 42128 | DDB_G0282927 | dhkL | histidine kinase, protein kinase, Atypical group, HisK family protein kinase |
| curated | 42383 | DDB_G0283907 | pkaC | cAMP-dependent protein kinase, protein serine/threonine kinase, protein kinase, AGC group, PKA family protein kinase, protein kinase A catalytic subunit |
| curated | 44150 | DDB_G0293932 | pakE | putative protein serine/threonine kinase, STE20 family protein kinase, protein kinase, STE group, PAKL subfamily protein kinase |
| curated | 44803 | DDB_G0274437 | twfA | putative protein tyrosine kinase, A6 family protein kinase, twinfilin |
| curated | 45161 | DDB_G0293978 | gxcJ | pleckstrin homology (PH) domain-containing protein, RhoGEF domain-containing protein, DH domain-containing protein |
| curated | 45162 | DDB_G0293898 | coaA | actin binding protein, coactosin |
| curated | 45371 | DDB_G0267374 | acpA | subunit of heterodimeric actin capping protein cap32/34 |
| curated | 46175 | DDB_G0272106 | arpB | actin related protein 2, polyphosphate kinase component, poly P kinase component |
| curated | 46589 | DDB_G0287855 | pirA | component of SCAR regulatory complex |
| curated | 46912 | DDB_G0282245 | forD | actin binding protein, formin homology domain-containing protein |
| curated | 46917 | DDB_G0289117 | myoB | myosin IB, class I myosin |
| curated | 46997 | DDB_G0279733 | gxcDD | pleckstrin homology (PH) domain-containing protein, RhoGEF domain-containing protein, IQ calmodulin-binding domain-containing protein, calponin homology (CH) domain-containing protein, Arf GTPase activating protein |
| curated | 47503 | DDB_G0291974 | docA | SH3 domain-containing protein, DOCK family protein, putative guanine nucleotide exchange factor (GEF) |
| curated | 47922 | DDB_G0268632 | abpA | alpha actinin, actin bundling protein, actin binding protein A, alpha actinin 1 |
| curated | 4855 | DDB_G0267854 | gxcD | RhoGEF domain-containing protein, calponin homology (CH) domain-containing protein, villin headpiece (VHP) domain-containing protein |
| curated | 48616 | DDB_G0286355 | mhcA | myosin II heavy chain, myosin heavy chain |
| curated | 48651 | DDB_G0293528 | arsA | arsenite transport subunit A, arsenite-translocating ATPase |
| curated | 48677 | DDB_G0285703 | hipA | I/LWEQ domain-containing protein, ENTH domain-containing protein |
| curated | 48766 | DDB_G0289115 | mhkB | myosin heavy chain kinase, protein serine/threonine kinase, protein kinase, Atypical group, Alpha kinase family protein, MHCK subfamily protein kinase |
| curated | 48789 | DDB_G0280887 | arpD | actin-like protein 6A, actin related protein 4 |
| curated | 4927 | DDB_G0291125 | gnrA | gelsolin-related protein |
| curated | 50285 | DDB_G0275185 | gacI | RhoGAP domain-containing protein |
| curated | 50380 | DDB_G0283755 | arpC | actin-like protein, actin related protein 3 |
| curated | 50735 | DDB_G0290481 | talA | filopodin, talinA |
| curated | 50865 | DDB_G0287295 | forC | actin binding protein, formin homology domain-containing protein |
| curated | 50894 | DDB_G0286555 | racA | Rho GTPase, RhoBTB family protein |
| curated | 50957 | DDB_G0277887 | dhkK | histidine kinase, protein kinase, Atypical group, HisK family protein kinase, response regulator receiver domain-containing protein |
| curated | 50960 | DDB_G0282949 | elmoC | engulfment and cell motility ELM family protein |
| curated | 51061 | DDB_G0282725 | vilD | villin |
| curated | 51240 | DDB_G0278733 | DAip1 | WD40 repeat-containing protein |
| curated | 51346 | DDB_G0276143 | dhkF | histidine kinase, protein kinase, Atypical group, HisK family protein kinase |
| curated | 51532 | DDB_G0290687 | mhkC | myosin heavy chain kinase, protein serine/threonine kinase, protein kinase, Atypical group, Alpha kinase family protein, MHCK subfamily protein kinase |
| curated | 51654 | DDB_G0277131 | gxcBB | pleckstrin homology (PH) domain-containing protein, RhoGEF domain-containing protein, IQ calmodulin-binding domain-containing protein, calponin homology (CH) domain-containing protein |
| curated | 51890 | DDB_G0291117 | noxC | superoxide-generating NADPH oxidase flavocytochrome, flavocytochrome b large subunit |
| curated | 52129 | DDB_G0293124 | kxcB | putative protein serine/threonine kinase, RhoGEF domain-containing protein, DH domain-containing protein, PH domain-containing protein, protein kinase, STE group |
| curated | 52203 | DDB_G0283081 | pikB | phosphatidylinositol-4,5-diphosphate 3-kinase, PI3kinase |
| curated | 52275 | DDB_G0274455 | myoI | class VII unconventional myosin, myosin VII |
| curated | 52376 | DDB_G0292816 | racL | Rho GTPase |
| curated | 52744 | DDB_G0276893 | ctxB | cortexillin II |
| curated | 52946 | DDB_G0269134 | efaAI | elongation factor 1 alpha, elongation factor 1a |
| curated | 53913 | DDB_G0288769 | cap | cyclase associated protein |
| curated | 54845 | DDB_G0287291 | abpD | interaptin, calponin homology (CH) domain-containing protein, actin binding protein D, alpha actinin 2 |
| curated | 55703 | DDB_G0293526 | racC | Rho GTPase |
| curated | 56349 | DDB_G0270730 | fslB | G-protein-coupled receptor (GPCR) family protein, frizzled and smoothened-like protein |
| curated | 58101 | DDB_G0292804 | arcC | actin related protein 2/3 complex, subunit 3, actin related protein 2/3 complex, 21 kDa subunit |
| curated | 58194 | DDB_G0278727 | pikA | phosphatidylinositol-4,5-diphosphate 3-kinase, PI3kinase |
| curated | 58375 | DDB_G0279599 | crlG | G-protein-coupled receptor (GPCR) family protein, secretin-like receptor |
| curated | 59079 | DDB_G0288319 | arcE | actin related protein 2/3 complex, subunit 5, actin related protein 2/3 complex, 16 kDa subunit |
| curated | 74651 | DDB_G0292102 | fscJ | G-protein-coupled receptor (GPCR) family protein, frizzled and smoothened-like sans CRD protein |
| curated | 75463 | DDB_G0269178 | racG | Rho GTPase |
| curated | 75785 | DDB_G0283579 | crlD | G-protein-coupled receptor (GPCR) family protein |
| curated | 75958 | DDB_G0279711 | cmr | putative actin binding protein, comitin-like protein, putative mannose binding protein, B_lectin domain-containing protein |
| curated | 76346 | DDB_G0289879 | arsB | arsenite transport subunit B |
| curated | 76775 | DDB_G0267548 | elmoF | engulfment and cell motility ELM family protein |
| curated | 77025 | DDB_G0288377 | gxcH | pleckstrin homology (PH) domain-containing protein, RhoGEF domain-containing protein |
| curated | 77030 | DDB_G0288383 | gxcI | RhoGEF domain-containing protein |
| curated | 77869 | DDB_G0283479 | abcH4 | putative non-transporter ABC protein |
| curated | 77981 | DDB_G0272080 | gacHH | Kelch repeat-containing protein, RhoGAP domain-containing protein |
| curated | 78427 | DDB_G0280049 | rdiB | rho GDP-dissociation inhibitor-like protein |
| curated | 78826 | DDB_G0272150 | grlJ | G-protein-coupled receptor (GPCR) family 3 protein 9 |
| curated | 79778 | DDB_G0289725 | fscA | G-protein-coupled receptor (GPCR) family protein, frizzled and smoothened-like sans CRD protein |
| curated | 80288 | DDB_G0289483 | ctxA | actin bundling protein, actin binding protein, cortexillin I |
| curated | 81422 | DDB_G0291085 | gxcE | pleckstrin homology (PH) domain-containing protein, RhoGEF domain-containing protein |
| curated | 81789 | DDB_G0292004 | DDB_G0292004 | DOCK family protein, putative guanine nucleotide exchange factor (GEF), cyclin-like F-box containing protein |
| curated | 82049 | DDB_G0286609 | fslQ | G-protein-coupled receptor (GPCR) family protein, frizzled and smoothened-like protein |
| curated | 82068 | DDB_G0291021 | gacD | RhoGAP domain-containing protein |
| curated | 82126 | DDB_G0286301 | crlE | G-protein-coupled receptor (GPCR) family protein |
| curated | 82194 | DDB_G0281047 | gxcEE | RhoGEF domain-containing protein, IQ calmodulin-binding domain-containing protein |
| curated | 82447 | DDB_G0280087 | gxcS | pleckstrin homology (PH) domain-containing protein, RhoGEF domain-containing protein |
| curated | 83267 | DDB_G0290023 | gxcL | pleckstrin homology (PH) domain-containing protein, RhoGEF domain-containing protein |
| curated | 83271 | DDB_G0267774 | abkC | putative protein serine/threonine kinase, ABC1 family protein kinase, protein kinase, Atypical group, ABC1-C subfamily protein kinase |
| curated | 84596 | DDB_G0269610 | gxcT | C2H2-type zinc finger-containing protein, pleckstrin homology (PH) domain-containing protein, RhoGEF domain-containing protein, IQ calmodulin-binding domain-containing protein |
| curated | 85139 | DDB_G0272885 | fslJ-1 | G-protein-coupled receptor (GPCR) family protein, frizzled and smoothened-like protein, putative cell number regulator |
| curated | 85191 | DDB_G0282475 | gxcF | pleckstrin homology (PH) domain-containing protein, RhoGEF domain-containing protein |
| curated | 85721 | DDB_G0269102 | arcD | actin related protein 2/3 complex, 20 kDa subunit, actin related protein 2/3 complex, subunit 4 |
| curated | 85916 | DDB_G0278755 | gacR | RhoGAP domain-containing protein |
| curated | 86597 | DDB_G0280179 | elmoB | engulfment and cell motility ELM family protein |
| curated | 87985 | DDB_G0267956 | abiA | component of SCAR regulatory complex |
| curated | 88032 | DDB_G0287895 | gacM | RhoGAP domain-containing protein |
| curated | 88267 | DDB_G0279175 | hspc300 | component of SCAR regulatory complex |
| curated | 88548 | DDB_G0288937 | arpA | alpha-centractin, polyphosphate kinase component, poly P kinase component, actin related protein 1 |
| curated | 88870 | DDB_G0282595 | gmfA | putative actin binding protein, ADF/cofilin-like domain-containing protein, glia maturation factor |
| curated | 89172 | DDB_G0285453 | racP | Rho GTPase |
| curated | 89777 | DDB_G0285303 | gxcR | pleckstrin homology (PH) domain-containing protein, RhoGEF domain-containing protein |
| curated | 89818 | DDB_G0284585 | fslL | G-protein-coupled receptor (GPCR) family protein, frizzled and smoothened-like protein |
| curated | 89900 | DDB_G0285641 | gacJ | RhoGAP domain-containing protein |
| curated | 90830 | DDB_G0276571 | gnrC | gelsolin-related protein |
| curated | 91113 | DDB_G0267450 | pakC | p21-activated protein kinase, putative protein serine/threonine kinase, pleckstrin homology (PH) domain-containing protein, STE20 family protein kinase, PH domain-containing protein, protein kinase, STE group, PAKA subfamily protein kinase |
| curated | 91322 | DDB_G0269100 | abpC | gelation factor, actin binding protein, filamin |
| curated | 91686 | DDB_G0277881 | limC | LIM-type zinc finger-containing protein |
| curated | 91862 | DDB_G0289541 | vasP | vasodilator-stimulated phosphoprotein, VASP |
| curated | 92199 | DDB_G0283767 | dct | dynacortin |
| curated | 92251 | DDB_G0277855 | fimA | actin bundling protein, actin binding protein, fimbrin, calponin homology (CH) domain-containing protein |
| curated | 92763 | DDB_G0272112 | myoJ | myosin-5b |
| curated | 92856 | DDB_G0282923 | rdeA | Hpt domain-containing protein, histidine-containing phosphotransfer domain-containing protein |
| curated | 92938 | DDB_G0268354 | gxcGG | pleckstrin homology (PH) domain-containing protein, RhoGEF domain-containing protein, IQ calmodulin-binding domain-containing protein |
| curated | 92985 | DDB_G0277869 | rac1A | Rho GTPase |
| curated | 93648 | DDB_G0285915 | gacK | RhoGAP domain-containing protein |
| curated | 94172 | DDB_G0291231 | mhkA | myosin heavy chain kinase, protein serine/threonine kinase, protein kinase, Atypical group, Alpha kinase family protein, MHCK subfamily protein kinase |
| curated | 94211 | DDB_G0279657 | elmoE | engulfment and cell motility ELM family protein |
| curated | 94447 | DDB_G0279081 | abpB | actin bundling protein, actin binding protein, 34 kDa actin-binding protein |
| curated | 94684 | DDB_G0279415 | limE | LIM-type zinc finger-containing protein |
| curated | 94711 | DDB_G0290185 | crlF | G-protein-coupled receptor (GPCR) family protein |
| curated | 95056 | DDB_G0277825 | arcA | actin related protein 2/3 complex, subunit 1, actin related protein 2/3 complex, 41 kDa subunit |
| curated | 95353 | DDB_G0291077 | rdiA | Rho GDP-dissociation inhibitor |
| curated | 95356 | DDB_G0290891 | gacL | RhoGAP domain-containing protein |
| curated | 95520 | DDB_G0282489 | mhkD | putative protein serine/threonine kinase, putative myosin heavy chain kinase, protein kinase, Atypical group, Alpha kinase family protein, MHCK subfamily protein kinase |
| curated | 95982 | DDB_G0291536 | gnrB | gelsolin-related protein |
| curated | 96166 | DDB_G0277017 | gxcN | pleckstrin homology (PH) domain-containing protein, RhoGEF domain-containing protein |
| curated | 96234 | DDB_G0278051 | elmoA | engulfment and cell motility ELM family protein |
| curated | 96263 | DDB_G0269166 | pakA | p21-activated protein kinase, protein serine/threonine kinase, STE20 family protein kinase, protein kinase, STE group, PAKA subfamily protein kinase |
| curated | 96403 | DDB_G0267490 | ChLim | LIM-type zinc finger-containing protein, calponin homology (CH) domain-containing protein |
| curated | 96526 | DDB_G0278549 | gacB | RhoGAP domain-containing protein |
| curated | 96538 | DDB_G0293928 | gxcZ | RhoGEF domain-containing protein, IQ calmodulin-binding domain-containing protein, calponin homology (CH) domain-containing protein, villin headpiece (VHP) domain-containing protein |
| curated | 96754 | DDB_G0291093 | pikH | PI3kinase, Phosphatidylinositol 3-kinase |
| curated | 97064 | DDB_G0284331 | regA | cAMP phosphodiesterase, response regulator receiver domain-containing protein |
| curated | 97170 | DDB_G0269496 | gacZ | MYND-type zinc finger-containing protein, RhoGAP domain-containing protein |
| curated | 97202 | DDB_G0291378 | forJ | formin homology domain-containing protein |
| curated | 97430 | DDB_G0285163 | gacT | RhoGAP domain-containing protein |
| curated | 97523 | DDB_G0269160 | nxnA | annexin VII |
| curated | 97675 | DDB_G0272104 | acpB | subunit of heterodimeric actin capping protein cap32/34 |
| curated | 97823 | DDB_G0293396 | gxcO | pleckstrin homology (PH) domain-containing protein, RhoGEF domain-containing protein |
| curated | 98122 | DDB_G0267462 | Dd5P4 | RhoGAP domain-containing protein, inositol 5-phosphatase |
| curated | 98135 | DDB_G0280975 | racE | Rho GTPase |
| curated | 98143 | DDB_G0280093 | gacGG | regulator of chromosome condensation (RCC1) domain-containing protein, RhoGAP domain-containing protein |
| curated | 98229 | DDB_G0272925 | gacU | RhoGAP domain-containing protein |
| curated | 98367 | DDB_G0288679 | myoE | myosin IE, class I myosin |
| curated | 98394 | DDB_G0289447 | myoH | myosin, putative class V myosin |
| curated | 98559 | DDB_G0292386 | carmil | leucine-rich repeat-containing protein (LRR) |
| curated | 98759 | DDB_G0287681 | grlR | G-protein-coupled receptor (GPCR) family 3 protein 17 |
| curated | 98812 | DDB_G0292150 | ak1 | putative protein serine/threonine kinase, Arf GTPase activating protein, protein kinase, Atypical group, Alpha kinase family protein |
| curated | 98974 | DDB_G0293510 | gacV | RhoGAP domain-containing protein |
| curated | 98995 | DDB_G0291095 | grlP | G-protein-coupled receptor (GPCR) family 3 protein 15 |
| curated | 99010 | DDB_G0278703 | gxcII | pleckstrin homology (PH) domain-containing protein, RhoGEF domain-containing protein, IQ calmodulin-binding domain-containing protein |
| curated | 99023 | DDB_G0287007 | arpF | actin related protein 6 |
| curated | 99199 | DDB_G0275679 | gxcJJ | pleckstrin homology (PH) domain-containing protein, RhoGEF domain-containing protein, IQ calmodulin-binding domain-containing protein |
| curated | 99304 | DDB_G0288773 | ncfA | p67-like superoxide-generating NADPH oxidase, cytosolic factor p67 |
| curated | 99415 | DDB_G0283615 | rpkA | G-protein-coupled receptor (GPCR) family protein, phosphatidylinositol-4-phosphate 5-kinase (PIP5K), serpentine receptor PIP5K family protein |
| curated | 99508 | DDB_G0280943 | elmoD | ankyrin repeat-containing protein, engulfment and cell motility ELM family protein |
| curated | 99548 | DDB_G0277987 | gxcA | pleckstrin homology (PH) domain-containing protein, RhoGEF domain-containing protein, IQ calmodulin-binding domain-containing protein, Rac guanyl-nucleotide exchange factor, calponin homology (CH) domain-containing protein |
| curated | 99654 | DDB_G0269388 | corB | WD-40 repeat-containing protein, putative actin binding protein, coronin B, double coronin |
| curated | 99974 | DDB_G0289327 | sevA | severin |
| inparanoid | 100013 | DDB_G0289001 | pyk3 | protein tyrosine kinase, protein kinase, TKL group, tyrosine kinase-like protein, DPYK family protein kinase |
| inparanoid | 100030 | DDB_G0285393 | ints1 | integrator complex subunit 1 |
| inparanoid | 100046 | DDB_G0282115 | DDB_G0282115 | SNF2-related domain-containing protein, C3HC4-type zinc finger-containing protein, CHR group protein, helicase, C-terminal domain-containing protein |
| inparanoid | 100047 | DDB_G0267830 | gemA | mitochondrial GTPase, Ras-related GTPase |
| inparanoid | 100061 | DDB_G0272406 | DDB_G0272406 |  |
| inparanoid | 100067 | DDB_G0272350 | sgkC | sphingosine kinase related protein |
| inparanoid | 100069 | DDB_G0288105 | oxct | 3-oxoacid CoA-transferase |
| inparanoid | 100079 | DDB_G0293924 | DDB_G0293924 |  |
| inparanoid | 100082 | DDB_G0281507 | colC | colossin C, Cna B-type domain-containing protein |
| inparanoid | 100086 | DDB_G0272845 | DDB_G0272845 | AB-hydrolase associated lipase region containing protein |
| inparanoid | 100091 | DDB_G0289339 | med21 | putative mediator complex subunit 21 |
| inparanoid | 100116 | DDB_G0284029 | pkgD | protein kinase 4, cAMP-dependent protein kinase, protein serine/threonine kinase, protein kinase, AGC group, PKA family protein kinase, protein kinase A family kinase |
| inparanoid | 100160 | DDB_G0285223 | anapc4 | anaphase promoting complex subunit 4 |
| inparanoid | 100174 | DDB_G0285807 | DDB_G0285807 | unknown |
| inparanoid | 100176 | DDB_G0287911 | DDB_G0287911 | poly(ADP-ribose) polymerase, catalytic region domain-containing protein |
| inparanoid | 100179 | DDB_G0277777 | DDB_G0277777 | Similar to Dictyostelium discoideum (Slime mold). prespore-specific protein. |
| inparanoid | 10189 | DDB_G0279481 | ccdc94 | putative RNA splicing factor |
| inparanoid | 10469 | DDB_G0286713 | DDB_G0286713 |  |
| inparanoid | 10607 | DDB_G0289189 | psmD10 | ankyrin repeat-containing protein, 26S proteasome non-ATPase regulatory subunit 10, 26S proteasome regulatory subunit p28 |
| inparanoid | 10653 | DDB_G0292318 | rsmD | small GTPase |
| inparanoid | 10678 | DDB_G0279037 | coq3 | 3,4-dihydroxy-5-hexaprenylbenzoate methyltransferase, hexaprenyldihydroxybenzoate methyltransferase, methyltransferase type 11 domain-containing protein |
| inparanoid | 10699 | DDB_G0294577 | DDB_G0294577 | Rab GTPase |
| inparanoid | 10734 | DDB_G0289353 | cox11 | cytochrome c oxidase assembly protein |
| inparanoid | 10848 | DDB_G0292946 | DDB_G0292946 |  |
| inparanoid | 10863 | DDB_G0286617 | cycK | cyclin, putative K-type cyclin |
| inparanoid | 10979 | DDB_G0283631 | arfrp1 | ARF-like protein, ADP-ribosylation factor-like protein |
| inparanoid | 11061 | DDB_G0277631 | DDB_G0277631 | Similar to hypothetical ORF; Pth2p. |
| inparanoid | 11127 | DDB_G0281565 | rpl19 | S60 ribosomal protein L19 |
| inparanoid | 11174 | DDB_G0284959 | DDB_G0284959 | optic atrophy 3-like family protein |
| inparanoid | 11209 | DDB_G0291199 | DDB_G0291199 |  |
| inparanoid | 11237 | DDB_G0275459 | DDB_G0275459 | RNA-binding region RNP-1 domain-containing protein, RNA recognition motif-containing protein RRM |
| inparanoid | 11264 | DDB_G0281965 | DDB_G0281965 |  |
| inparanoid | 11322 | DDB_G0292766 | DDB_G0292766 |  |
| inparanoid | 11345 | DDB_G0293246 | fxn | frataxin |
| inparanoid | 11664 | DDB_G0267524 | ssr3 | translocon-associated protein TRAP gamma subunit |
| inparanoid | 11971 | DDB_G0292324 | med28 | putative mediator complex subunit 28 |
| inparanoid | 12023 | DDB_G0267998 | psiS | PA14 domain-containing protein |
| inparanoid | 12134 | DDB_G0277029 | tmem56B | TRAM, LAG1 and CLN8 homology domain-containing protein, TMEM56 family protein 2 |
| inparanoid | 12282 | DDB_G0276339 | DDB_G0276339 |  |
| inparanoid | 12619 | DDB_G0283547 | rsmA | small GTPase |
| inparanoid | 13105 | DDB_G0282433 | DDB_G0282433 |  |
| inparanoid | 13427 | DDB_G0286989 | DDB_G0286989 |  |
| inparanoid | 13578 | DDB_G0271168 | DDB_G0271168 |  |
| inparanoid | 13641 | DDB_G0291942 | DDB_G0291942 |  |
| inparanoid | 13661 | DDB_G0293048 | DDB_G0293048 |  |
| inparanoid | 13673 | DDB_G0289475 | DDB_G0289475 |  |
| inparanoid | 13730 | DDB_G0288433 | DDB_G0288433 |  |
| inparanoid | 13745 | DDB_G0267706 | DDB_G0267706 |  |
| inparanoid | 13772 | DDB_G0271070 | DDB_G0271070 |  |
| inparanoid | 13870 | DDB_G0275719 | lsm6 | LSM (like-Sm) domain-containing protein, putative U6 small nuclear ribonucleoparticle-associated protein |
| inparanoid | 13877 | DDB_G0279667 | H2AX | histone H2A |
| inparanoid | 13929 | DDB_G0285293 | spt4 | transcription initiation factor Spt4 |
| inparanoid | 14102 | DDB_G0283571 | DDB_G0283571 |  |
| inparanoid | 14407 | DDB_G0280971 | lvsC | BEACH domain-containing protein |
| inparanoid | 14626 | DDB_G0278965 | DG2033 | tRNA/rRNA methyltransferase SpoU family protein |
| inparanoid | 146457 | DDB_G0274629 | DDB_G0274629 | unknown |
| inparanoid | 146468 | DDB_G0274401 | DDB_G0274401 |  |
| inparanoid | 146503 | DDB_G0274241 | bre1 | RING zinc finger-containing protein, RmlC-like cupin family protein |
| inparanoid | 146505 | DDB_G0274693 | DDB_G0274693 | BRCT domain-containing protein |
| inparanoid | 146507 | DDB_G0289971 | med24 | putative mediator complex subunit 24 |
| inparanoid | 146509 | DDB_G0289913 | DDB_G0289913 |  |
| inparanoid | 146568 | DDB_G0274247 | fam91 | FAM91 family protein |
| inparanoid | 146630 | DDB_G0278231 | DDB_G0278231 |  |
| inparanoid | 146631 | DDB_G0288341 | DDB_G0288341 |  |
| inparanoid | 146650 | DDB_G0268134 | DDB_G0268134 | Similar to Orgyia pseudotsugata multicapsid polyhedrosis virus (OpMNPV). hypothetical 29.3 kDa protein (ORF92). |
| inparanoid | 146659 | DDB_G0274845 | DDB_G0274845 |  |
| inparanoid | 146669 | DDB_G0280199 | gadA | glutamate decarboxylase |
| inparanoid | 146678 | DDB_G0272186 | DDB_G0272186 | tetratricopeptide-like helical domain-containing protein (TPR), TPR_1 repeat-containing protein |
| inparanoid | 146682 | DDB_G0272178 | DDB_G0272178 | DUF1715 family protein |
| inparanoid | 146695 | DDB_G0269746 | ITPK1 | inositol 1,3,4-triphosphate 5/6 kinase, inositol-tetrakisphosphate 1-kinase |
| inparanoid | 146703 | DDB_G0270826 | abcG22 | ABC transporter G family protein |
| inparanoid | 146707 | DDB_G0270474 | DDB_G0270474 |  |
| inparanoid | 146712 | DDB_G0269304 | DDB_G0269304 | RIKEN cDNA 2810403P18. |
| inparanoid | 146713 | DDB_G0269302 | DDB_G0269302 |  |
| inparanoid | 146718 | DDB_G0270336 | ipo4 | importin 4 |
| inparanoid | 146727 | DDB_G0268684 | DDB_G0268684 |  |
| inparanoid | 146760 | DDB_G0269950 | serC | phosphoserine transaminase, phosphoserine aminotransferase |
| inparanoid | 146763 | DDB_G0295677 | mpdu1 | transmembrane protein, cystinosin/ERS1p repeat-containing protein |
| inparanoid | 146768 | DDB_G0270694 | DDB_G0270694 | Q19958 Stomatin protein 2. |
| inparanoid | 146769 | DDB_G0269722 | DDB_G0269722 | CG10903 protein (RE22146p). |
| inparanoid | 146772 | DDB_G0269726 | DDB_G0269726 |  |
| inparanoid | 146775 | DDB_G0269942 | DDB_G0269942 |  |
| inparanoid | 146788 | DDB_G0269976 | DDB_G0269976 |  |
| inparanoid | 146789 | DDB_G0269974 | DDB_G0269974 |  |
| inparanoid | 146813 | DDB_G0281687 | DDB_G0281687 | SWAP/Surp domain-containing protein, RNA-binding region RNP-1 domain-containing protein, RNA recognition motif-containing protein RRM, regulation of nuclear pre-mRNA domain-containing protein (RPR) |
| inparanoid | 146818 | DDB_G0281685 | nol1 | NOL1/NOP2/Sun family protein |
| inparanoid | 146833 | DDB_G0281749 | DDB_G0281749 |  |
| inparanoid | 146841 | DDB_G0281875 | DDB_G0281875 |  |
| inparanoid | 146844 | DDB_G0281825 | comB | Rab GTPase domain-containing protein |
| inparanoid | 146855 | DDB_G0282007 | uch1 | peptidase C12 family protein, ubiquitin C-terminal hydrolase |
| inparanoid | 146878 | DDB_G0282181 | nvl | AAA ATPase domain-containing protein, nuclear VCP-like protein, valosin-containing protein |
| inparanoid | 146888 | DDB_G0292994 | pdhA | pyruvate dehydrogenase E1 alpha subunit |
| inparanoid | 146906 | DDB_G0281991 | DDB_G0281991 |  |
| inparanoid | 146951 | DDB_G0270296 | dcd1A | acid ceramidase-like protein, acid N-acylsphingosine amidohydrolase-like protein |
| inparanoid | 146978 | DDB_G0280831 | DDB_G0280831 | phosphoglycerate mutase domain-containing protein |
| inparanoid | 146982 | DDB_G0293394 | pter | phosphotriesterase-related protein |
| inparanoid | 146986 | DDB_G0283087 | DDB_G0283087 | putative cholinesterase, carboxylesterase, type B family protein |
| inparanoid | 146993 | DDB_G0283009 | mcm8 | MCM family protein |
| inparanoid | 146994 | DDB_G0283011 | DDB_G0283011 | putative t-SNARE family protein |
| inparanoid | 146999 | DDB_G0282957 | DDB_G0282957 | unknown |
| inparanoid | 147006 | DDB_G0283039 | DDB_G0283039 |  |
| inparanoid | 147008 | DDB_G0283305 | DDB_G0283305 |  |
| inparanoid | 147009 | DDB_G0283339 | DDB_G0283339 |  |
| inparanoid | 147031 | purC%2FE | purC%2FE |  |
| inparanoid | 147033 | DDB_G0283937 | sec1 | Sec1-like family protein, putative syntaxin binding protein |
| inparanoid | 147037 | DDB_G0274311 | DDB_G0274311 | GRIM-19 domain-containing protein, NADH-ubiquinone oxidoreductase |
| inparanoid | 147040 | DDB_G0274323 | DDB_G0274323 |  |
| inparanoid | 147050 | DDB_G0274275 | DDB_G0274275 | AT1-46. 11/98 |
| inparanoid | 147058 | DDB_G0274527 | DDB_G0274527 | COBW domain-containing protein, cobalamin (vitamin B12) biosynthesis family protein, cobalamin synthesis protein/P47K family protein |
| inparanoid | 147060 | DDB_G0274551 | rcdBB | random cDNA clone veg113 |
| inparanoid | 147072 | DDB_G0274643 | DDB_G0274643 | Similar to Dictyostelium discoideum (Slime mold). carbonic anhydrase. |
| inparanoid | 147081 | DDB_G0274797 | aqr | intron-binding protein aquarius homolog |
| inparanoid | 147084 | DDB_G0274335 | DDB_G0274335 |  |
| inparanoid | 147099 | DDB_G0274577 | DG1124 | calcium-binding EF-hand domain-containing protein |
| inparanoid | 147104 | DDB_G0274433 | DDB_G0274433 |  |
| inparanoid | 147119 | DDB_G0274663 | DDB_G0274663 | P90893 Putative serine protease F56F10.1 precursor (EC 3.4.-.-). |
| inparanoid | 147125 | DDB_G0274487 | DDB_G0274487 | Similar to hypothetical ORF; Yor161cp. |
| inparanoid | 147133 | DDB_G0287833 | DDB_G0287833 |  |
| inparanoid | 147134 | DDB_G0287831 | DDB_G0287831 |  |
| inparanoid | 147136 | DDB_G0268302 | rpl38 | S60 ribosomal protein L38 |
| inparanoid | 147137 | DDB_G0267642 | DDB_G0267642 |  |
| inparanoid | 147138 | DDB_G0267448 | DDB_G0267448 | erg4/erg24 family protein |
| inparanoid | 147142 | DDB_G0271306 | nmd3 | 60S ribosomal export protein |
| inparanoid | 147143 | DDB_G0268450 | DDB_G0268450 |  |
| inparanoid | 147144 | DDB_G0268452 | DDB_G0268452 | v-SNARE family protein |
| inparanoid | 147148 | DDB_G0267488 | DDB_G0267488 |  |
| inparanoid | 147158 | DDB_G0275167 | dstD | signal transducer and activator of transcription (STAT) family protein |
| inparanoid | 147163 | DDB_G0275205 | DDB_G0275205 | Similar to Arabidopsis thaliana (Mouse-ear cress). hypothetical 38.7 kDa protein. |
| inparanoid | 147171 | DDB_G0271316 | DDB_G0271316 |  |
| inparanoid | 147180 | DDB_G0267452 | sonA | UAS domain-containing protein, ubiquilin domain-containing protein |
| inparanoid | 147182 | DDB_G0267992 | DDB_G0267992 |  |
| inparanoid | 147183 | DDB_G0267994 | DDB_G0267994 | DNAJ heat shock N-terminal domain-containing protein |
| inparanoid | 147185 | DDB_G0267406 | lmpA | lysosomal integral membrane glycoprotein |
| inparanoid | 147188 | DDB_G0267440 | lmpC | lysosomal integral membrane protein II |
| inparanoid | 147196 | DDB_G0267496 | DDB_G0267496 |  |
| inparanoid | 147249 | DDB_G0292872 | cct4 | chaperonin containing TCP1 delta subunit |
| inparanoid | 147256 | DDB_G0292818 | DDB_G0292818 | Zim17-type zinc finger-containing protein |
| inparanoid | 147284 | DDB_G0275671 | DDB_G0275671 | esterase/lipase/thioesterase domain-containing protein |
| inparanoid | 147287 | DDB_G0275777 | DDB_G0275777 |  |
| inparanoid | 147302 | DDB_G0278909 | DDB_G0278909 | leucine-rich repeat-containing protein (LRR), armadillo repeat-containing protein, protein kinase, TKL group, tyrosine kinase-like protein, ARMK family protein kinase |
| inparanoid | 147313 | DDB_G0278867 | gefBB | Ras guanine nucleotide exchange factor |
| inparanoid | 147315 | DDB_G0278761 | DDB_G0278761 | NF-X1-type zinc finger-containing protein, SAM domain-containing protein |
| inparanoid | 147339 | DDB_G0279245 | mpp10 | U3 snoRNP protein, U3 small nucleolar ribonucleoprotein |
| inparanoid | 147357 | DDB_G0276805 | DDB_G0276805 | MICROTUBULE-ASSOCIATED PROTEIN CP224. 6/101 |
| inparanoid | 147358 | DDB_G0277053 | DDB_G0277053 | Similar to Dictyostelium discoideum (Slime mold). Histidine kinase DhkE. |
| inparanoid | 147378 | DDB_G0292072 | fdfT | farnesyl-diphosphate farnesyltransferase, squalene synthase |
| inparanoid | 147399 | DDB_G0280375 | DDB_G0280375 | Polyprotein (Fragment). |
| inparanoid | 147400 | DDB_G0280183 | sglB | sphingosine-1-phosphate lyase, S1P lyase |
| inparanoid | 147402 | DDB_G0292028 | DDB_G0292028 | BCSC1. |
| inparanoid | 147409 | DDB_G0284693 | DDB_G0284693 |  |
| inparanoid | 147411 | DDB_G0284359 | phaZ | polyhydroxybutyrate depolymerase, PHB depolymerase |
| inparanoid | 147419 | DDB_G0284379 | xpo5 | armadillo-like helical domain-containing protein, exportin 5 |
| inparanoid | 147427 | DDB_G0284219 | DDB_G0284219 |  |
| inparanoid | 147431 | DDB_G0284213 | DDB_G0284213 |  |
| inparanoid | 147443 | DDB_G0284573 | syf2 | putative pre-mRNA-splicing factor |
| inparanoid | 147450 | DDB_G0284059 | DDB_G0284059 | SET domain-containing protein, MYND-type zinc finger-containing protein, TPR repeat-containing protein |
| inparanoid | 147463 | DDB_G0284691 | DDB_G0284691 |  |
| inparanoid | 147464 | DDB_G0284689 | DDB_G0284689 |  |
| inparanoid | 147466 | DDB_G0284687 | DDB_G0284687 | phospholipase D, putative cardiolipin synthetase |
| inparanoid | 147478 | DDB_G0284683 | DDB_G0284683 |  |
| inparanoid | 147508 | DDB_G0284799 | DDB_G0284799 | putative NADH dehydrogenase (ubiquinone), putative NADH-ubiquinone oxidoreductase 19 kDa subunit, CHCH domain-containing protein |
| inparanoid | 147519 | DDB_G0272965 | DDB_G0272965 |  |
| inparanoid | 147525 | DDB_G0275879 | fnkE | putative protein serine/threonine kinase, FNIP repeat-containing protein, protein kinase, STE group, FNIPK subfamily protein kinase |
| inparanoid | 147527 | DDB_G0272690 | DDB_G0272690 | SWIFT. 6/101 |
| inparanoid | 147546 | DDB_G0272748 | DDB_G0272748 |  |
| inparanoid | 147550 | DDB_G0278509 | DDB_G0278509 | protein kinase, TKL group, tyrosine kinase-like protein |
| inparanoid | 147553 | DDB_G0292570 | DDB_G0292570 |  |
| inparanoid | 147562 | DDB_G0278701 | DDB_G0278701 | P42695 Hypothetical protein KIAA0056 (Fragment). |
| inparanoid | 147567 | DDB_G0285653 | DDB_G0285653 |  |
| inparanoid | 147571 | DDB_G0278431 | DDB_G0278431 |  |
| inparanoid | 147573 | DDB_G0278435 | DDB_G0278435 | AAA ATPase domain-containing protein |
| inparanoid | 147575 | DDB_G0278687 | pigO | phosphatidylinositol glycan, class O |
| inparanoid | 147576 | DDB_G0278443 | DDB_G0278443 | ARF/SAR superfamily protein |
| inparanoid | 147579 | DDB_G0278427 | DDB_G0278427 |  |
| inparanoid | 147581 | DDB_G0279437 | DDB_G0279437 | cytochrome b561 / ferric reductase transmembrane domain-containing protein, DOMON domain-containing protein |
| inparanoid | 147582 | DDB_G0279435 | DDB_G0279435 |  |
| inparanoid | 147589 | DDB_G0292586 | DDB_G0292586 | regulator of chromosome condensation (RCC1) domain-containing protein |
| inparanoid | 147604 | DDB_G0278311 | DDB_G0278311 | myotubularin-related protein |
| inparanoid | 147615 | DDB_G0278735 | fps | farnesyl diphosphate synthase, FDP synthase, geranyltranstransferase |
| inparanoid | 147619 | DDB_G0279397 | DDB_G0279397 |  |
| inparanoid | 147637 | DDB_G0268566 | DDB_G0268566 |  |
| inparanoid | 147648 | DDB_G0267980 | mocs3 | molybdenum cofactor synthesis 3 |
| inparanoid | 147655 | DDB_G0278543 | srpRB | signal recognition particle receptor beta subunit |
| inparanoid | 147668 | DDB_G0279327 | cnrP | importin 9, putative cell number regulator |
| inparanoid | 147673 | DDB_G0292756 | DDB_G0292756 |  |
| inparanoid | 147676 | DDB_G0292752 | DDB_G0292752 |  |
| inparanoid | 147678 | DDB_G0292748 | DDB_G0292748 |  |
| inparanoid | 147685 | DDB_G0274171 | DDB_G0274171 | unknown |
| inparanoid | 147709 | DDB_G0293920 | DDB_G0293920 |  |
| inparanoid | 147735 | DDB_G0293976 | DDB_G0293976 |  |
| inparanoid | 147737 | DDB_G0276569 | DDB_G0276569 | unknown |
| inparanoid | 147740 | DDB_G0276449 | DDB_G0276449 | Putative synthase |
| inparanoid | 147754 | DDB_G0276679 | DDB_G0276679 |  |
| inparanoid | 147755 | DDB_G0276713 | DDB_G0276713 |  |
| inparanoid | 147757 | DDB_G0276349 | fnkC | FNIP repeat-containing protein, meprin and TRAF homology (MATH) domain-containing protein, protein kinase, STE group, FNIPK subfamily protein kinase |
| inparanoid | 147762 | DDB_G0276081 | DDB_G0276081 |  |
| inparanoid | 147767 | DDB_G0291530 | DDB_G0291530 |  |
| inparanoid | 147775 | DDB_G0277041 | rheb | Ras GTPase |
| inparanoid | 147778 | DDB_G0276789 | DDB_G0276789 |  |
| inparanoid | 147786 | DDB_G0277199 | rpc1 | RNA polymerase III, largest subunit |
| inparanoid | 147816 | DDB_G0282855 | DDB_G0282855 |  |
| inparanoid | 147817 | DDB_G0282817 | patB | P-type ATPase |
| inparanoid | 147823 | DDB_G0292664 | DDB_G0292664 | calponin homology (CH) domain-containing protein |
| inparanoid | 147828 | DDB_G0292398 | psmE4 | proteasome activator complex subunit 4 |
| inparanoid | 147835 | DDB_G0292666 | DDB_G0292666 |  |
| inparanoid | 147848 | DDB_G0269122 | cysA | cystathionine gamma-lyase |
| inparanoid | 147852 | DDB_G0282017 | DDB_G0282017 | Sec1-like family protein |
| inparanoid | 147853 | DDB_G0282011 | DDB_G0282011 | GPI transamidase subunit PIG-U family protein |
| inparanoid | 147862 | DDB_G0292538 | gtpbp1 | GTP-binding protein 1 |
| inparanoid | 147869 | DDB_G0279271 | DDB_G0279271 |  |
| inparanoid | 147871 | DDB_G0279267 | DDB_G0279267 | histone deacetylase family protein |
| inparanoid | 147878 | DDB_G0279177 | DDB_G0279177 |  |
| inparanoid | 147887 | DDB_G0293166 | DDB_G0293166 |  |
| inparanoid | 14789 | DDB_G0269590 | DDB_G0269590 | P-type ATPase, transmembrane protein |
| inparanoid | 147898 | DDB_G0293038 | DDB_G0293038 |  |
| inparanoid | 147906 | DDB_G0279105 | DDB_G0279105 |  |
| inparanoid | 147922 | DDB_G0269514 | DDB_G0269514 |  |
| inparanoid | 147928 | DDB_G0275331 | DDB_G0275331 | AgCP8645 (Fragment). |
| inparanoid | 147939 | DDB_G0276883 | canA | calcineurin A, CaM-dependent protein phosphatase, catalytic subunit, protein serine/threonine phosphatase, protein phosphatase-2B |
| inparanoid | 147940 | DDB_G0276673 | DDB_G0276673 | CG7074 protein (LD45056p). |
| inparanoid | 147945 | DDB_G0277009 | DDB_G0277009 |  |
| inparanoid | 147946 | DDB_G0277011 | DDB_G0277011 |  |
| inparanoid | 147950 | DDB_G0274971 | DDB_G0274971 |  |
| inparanoid | 147956 | DDB_G0276785 | DDB_G0276785 |  |
| inparanoid | 147960 | DDB_G0276895 | DDB_G0276895 |  |
| inparanoid | 147968 | DDB_G0277223 | dgkA | diacylglycerol kinase |
| inparanoid | 147973 | DDB_G0277331 | DDB_G0277331 | SET domain-containing protein |
| inparanoid | 147974 | DDB_G0277239 | DDB_G0277239 | Similar to Dictyostelium discoideum (Slime mold). Non-receptor tyrosine kinase spore lysis A (EC 2.7.1.112) (Tyrosine-protein kinase 1). |
| inparanoid | 147986 | DDB_G0292186 | DDB_G0292186 | NDT80/PhoG-like protein |
| inparanoid | 147991 | DDB_G0292112 | galK | galactokinase |
| inparanoid | 147994 | DDB_G0275213 | DDB_G0275213 |  |
| inparanoid | 147996 | DDB_G0275129 | DDB_G0275129 |  |
| inparanoid | 147997 | DDB_G0275099 | DDB_G0275099 |  |
| inparanoid | 148002 | DDB_G0293950 | DDB_G0293950 |  |
| inparanoid | 148003 | DDB_G0267674 | glcS | glycogen synthase |
| inparanoid | 148009 | DDB_G0267698 | DDB_G0267698 |  |
| inparanoid | 148017 | DDB_G0267732 | DDB_G0267732 |  |
| inparanoid | 148033 | DDB_G0267984 | DDB_G0267984 | peptidase M20 family protein |
| inparanoid | 148035 | DDB_G0267480 | nup93 | nucleoporin 93 |
| inparanoid | 148040 | DDB_G0267532 | DDB_G0267532 |  |
| inparanoid | 148051 | DDB_G0277539 | DDB_G0277539 | putative protein tyrosine kinase, WEE1 family protein kinase |
| inparanoid | 148069 | DDB_G0285921 | DDB_G0285921 |  |
| inparanoid | 148072 | DDB_G0285927 | DDB_G0285927 | aminoadipate-semialdehyde dehydrogenase-phosphopantetheinyl transferase |
| inparanoid | 148075 | DDB_G0285985 | DDB_G0285985 |  |
| inparanoid | 148089 | DDB_G0268194 | DDB_G0268194 | dCTP deaminase |
| inparanoid | 148100 | DDB_G0285523 | pex12 | RING zinc finger-containing protein, peroxin 12, peroxisomal biogenesis factor 12 |
| inparanoid | 148101 | DDB_G0285525 | DDB_G0285525 | dephospho-CoA kinase |
| inparanoid | 148112 | DDB_G0286021 | DDB_G0286021 | unknown |
| inparanoid | 148116 | DDB_G0285603 | DDB_G0285603 | unknown |
| inparanoid | 148117 | DDB_G0285427 | mkcA | protein serine/threonine kinase, STE20 family protein kinase, protein kinase, STE group, MKC subfamily protein kinase |
| inparanoid | 148127 | DDB_G0285765 | DDB_G0285765 | DNAJ heat shock N-terminal domain-containing protein, heat shock protein DnaJ family protein |
| inparanoid | 148146 | DDB_G0286135 | DDB_G0286135 | myb domain-containing protein, ZZ-type zinc finger-containing protein |
| inparanoid | 148187 | DDB_G0277455 | plbE | phospholipase B-like protein |
| inparanoid | 148193 | DDB_G0289367 | agnE | argonaut-like protein |
| inparanoid | 148196 | DDB_G0289361 | CSN2 | proteasome component region PCI (PINT) domain-containing protein, COP9 signalosome complex subunit 2 |
| inparanoid | 148200 | DDB_G0288847 | DDB_G0288847 |  |
| inparanoid | 148205 | DDB_G0288833 | derl1 | derlin-1 |
| inparanoid | 148208 | DDB_G0289349 | DDB_G0289349 | patatin family protein |
| inparanoid | 148209 | DDB_G0289347 | DDB_G0289347 |  |
| inparanoid | 148217 | DDB_G0269432 | DDB_G0269432 |  |
| inparanoid | 148218 | DDB_G0269430 | DDB_G0269430 | T10O22.22. |
| inparanoid | 148225 | DDB_G0269926 | DDB_G0269926 | RNA-binding region RNP-1 domain-containing protein, splicing factor PWI domain-containing protein |
| inparanoid | 148238 | DDB_G0269416 | rab32B | Rab GTPase |
| inparanoid | 148240 | DDB_G0270044 | DDB_G0270044 |  |
| inparanoid | 148247 | DDB_G0269404 | DDB_G0269404 |  |
| inparanoid | 148248 | DDB_G0269406 | DDB_G0269406 | P36592 DNA repair and recombination protein rad22. |
| inparanoid | 148251 | DDB_G0270118 | DDB_G0270118 |  |
| inparanoid | 148271 | DDB_G0270278 | DDB_G0270278 |  |
| inparanoid | 148277 | DDB_G0270230 | utp11 | U3 snoRNP protein, U3 small nucleolar ribonucleoprotein |
| inparanoid | 148280 | DDB_G0269912 | redB | NADPH-cytochrome-P450 oxidoreductase |
| inparanoid | 148281 | DDB_G0269914 | tpp1 | peptidase S8 and S53 domain-containing protein |
| inparanoid | 148294 | DDB_G0269858 | DDB_G0269858 |  |
| inparanoid | 148303 | DDB_G0270208 | DDB_G0270208 | WD40 repeat-containing protein |
| inparanoid | 148307 | DDB_G0270186 | DDB_G0270186 |  |
| inparanoid | 148310 | DDB_G0270190 | DDB_G0270190 | putative cellulase, cellulose-binding domain-containing protein, glycoside hydrolase family 5 protein |
| inparanoid | 148314 | DDB_G0270194 | DDB_G0270194 | DUF866 family protein |
| inparanoid | 148317 | DDB_G0270202 | DDB_G0270202 |  |
| inparanoid | 148328 | DDB_G0270448 | DDB_G0270448 |  |
| inparanoid | 148335 | DDB_G0270074 | DDB_G0270074 |  |
| inparanoid | 148338 | DDB_G0270398 | raptor | WD40 repeat-containing protein, HEAT repeat-containing protein, armadillo-like helical domain-containing protein, Raptor family protein |
| inparanoid | 148343 | DDB_G0291193 | nubpl | Mrp/NBP35 family protein |
| inparanoid | 148347 | DDB_G0291163 | nup155 | nucleoporin 155 |
| inparanoid | 148349 | DDB_G0291167 | DDB_G0291167 |  |
| inparanoid | 148371 | DDB_G0291009 | DDB_G0291009 |  |
| inparanoid | 148373 | DDB_G0291005 | DDB_G0291005 |  |
| inparanoid | 148387 | DDB_G0291057 | DDB_G0291057 |  |
| inparanoid | 148388 | DDB_G0291037 | rpb6 | RNA polymerase II core subunit, RNA polymerase I core subunit, RNA polymerase III core subunit |
| inparanoid | 148402 | DDB_G0284361 | DDB_G0284361 |  |
| inparanoid | 148411 | DDB_G0285363 | DDB_G0285363 |  |
| inparanoid | 148417 | DDB_G0285349 | anapc1 | anaphase promoting complex subunit 1 |
| inparanoid | 148427 | DDB_G0290451 | shkE | SH2 domain-containing protein, protein kinase, TKL group, tyrosine kinase-like protein, SHK subfamily protein kinase |
| inparanoid | 148428 | DDB_G0290415 | DDB_G0290415 | EbiP3474 (Fragment). |
| inparanoid | 148436 | DDB_G0290419 | DDB_G0290419 |  |
| inparanoid | 148463 | DDB_G0293106 | DDB_G0293106 |  |
| inparanoid | 148469 | DDB_G0292988 | DDB_G0292988 | putative transmembrane protein |
| inparanoid | 148470 | DDB_G0293856 | DDB_G0293856 |  |
| inparanoid | 148489 | DDB_G0279791 | DDB_G0279791 | unknown |
| inparanoid | 148493 | DDB_G0293098 | DDB_G0293098 |  |
| inparanoid | 148495 | DDB_G0288717 | DDB_G0288717 |  |
| inparanoid | 148496 | DDB_G0288719 | scfd1 | Sec1-like family protein |
| inparanoid | 148497 | DDB_G0288681 | prpD | Propionate catabolic protein PrpD. |
| inparanoid | 148513 | DDB_G0293046 | DDB_G0293046 |  |
| inparanoid | 148530 | DDB_G0293416 | abcB1 | ABC transporter B family protein |
| inparanoid | 148531 | DDB_G0293428 | DDB_G0293428 |  |
| inparanoid | 148535 | DDB_G0293460 | DDB_G0293460 |  |
| inparanoid | 148537 | DDB_G0293386 | DDB_G0293386 |  |
| inparanoid | 148551 | DDB_G0295793 | DDB_G0295793 | WD40 repeat-containing protein |
| inparanoid | 148567 | DDB_G0293740 | ddx51 | putative RNA helicase, DEAD/DEAH box helicase |
| inparanoid | 148568 | DDB_G0293682 | DDB_G0293682 |  |
| inparanoid | 148569 | DDB_G0293916 | DDB_G0293916 |  |
| inparanoid | 148578 | DDB_G0274167 | cand1 | HEAT repeat-containing protein, cullin-associated neddylation-disassociated protein 1 |
| inparanoid | 148579 | DDB_G0274159 | hisS | histidine-tRNA ligase, histidyl-tRNA synthetase |
| inparanoid | 148581 | DDB_G0275001 | DDB_G0275001 |  |
| inparanoid | 148582 | DDB_G0288405 | DDB_G0288405 | RabGAP/TBC domain-containing protein, putative GTPase activating protein (GAP), TLDc domain-containing protein |
| inparanoid | 148591 | DDB_G0288141 | DDB_G0288141 | unknown |
| inparanoid | 148600 | DDB_G0287325 | DDB_G0287325 | EPS15 homology (EH) domain-containing protein |
| inparanoid | 148604 | DDB_G0287537 | nop14 | U3 snoRNP protein, U3 small nucleolar ribonucleoprotein |
| inparanoid | 148605 | DDB_G0287997 | DDB_G0287997 |  |
| inparanoid | 148613 | DDB_G0272134 | DDB_G0272134 |  |
| inparanoid | 148631 | DDB_G0294533 | roco5 | pleckstrin homology (PH) domain-containing protein, RhoGEF domain-containing protein, leucine-rich repeat-containing protein (LRR), protein kinase, TKL group, tyrosine kinase-like protein, ROCO family protein kinase |
| inparanoid | 148633 | DDB_G0283965 | pigA | GlcNAc transferase, phosphatidylinositol glycan, class A, phosphatidylinositol N-acetylglucosaminyltransferase subunit A, glycosyltrasferase |
| inparanoid | 148636 | DDB_G0283909 | spc98 | spindle pole body component 98 |
| inparanoid | 148638 | DDB_G0283959 | DDB_G0283959 |  |
| inparanoid | 148639 | DDB_G0283957 | msh4 | mutS homolog, DNA mismatch repair protein |
| inparanoid | 148649 | DDB_G0283835 | rae1 | WD40 repeat-containing protein, putative mRNA export protein |
| inparanoid | 148650 | DDB_G0283891 | ube1c | ubiquitin-activating enzyme E1C, NEDD8-activating enzyme E1 catalytic subunit, ubiquitin-activating enzyme 3 (UBA3) |
| inparanoid | 148654 | DDB_G0283973 | DDB_G0283973 |  |
| inparanoid | 148674 | DDB_G0270996 | DDB_G0270996 |  |
| inparanoid | 148678 | DDB_G0279351 | DDB_G0279351 |  |
| inparanoid | 148685 | DDB_G0279383 | DDB_G0279383 |  |
| inparanoid | 148688 | DDB_G0279421 | DDB_G0279421 |  |
| inparanoid | 148693 | DDB_G0291710 | roco10 | Kelch repeat-containing protein, RGS domain-containing protein, leucine-rich repeat-containing protein (LRR), RhoGAP domain-containing protein, protein kinase, TKL group, tyrosine kinase-like protein, ROCO family protein kinase |
| inparanoid | 148695 | DDB_G0291818 | DDB_G0291818 |  |
| inparanoid | 148699 | DDB_G0276049 | DDB_G0276049 | unknown |
| inparanoid | 148700 | DDB_G0291852 | DDB_G0291852 | UPF0551 family protein |
| inparanoid | 148703 | DDB_G0291606 | vps8 | RING zinc finger-containing protein, WD40 repeat-containing protein |
| inparanoid | 148704 | DDB_G0291472 | DDB_G0291472 | Pirin. |
| inparanoid | 148721 | DDB_G0291908 | DDB_G0291908 |  |
| inparanoid | 148729 | DDB_G0271694 | DDB_G0271694 |  |
| inparanoid | 148736 | DDB_G0271288 | DDB_G0271288 | NPR2 family protein |
| inparanoid | 148738 | DDB_G0271282 | CSN7 | proteasome component region PCI (PINT) domain-containing protein, COP9 signalosome complex subunit 7 |
| inparanoid | 148744 | DDB_G0271250 | DDB_G0271250 |  |
| inparanoid | 148745 | DDB_G0271292 | DDB_G0271292 | NAP (nucleosome assembly protein) family protein |
| inparanoid | 148748 | DDB_G0271484 | DDB_G0271484 |  |
| inparanoid | 148761 | DDB_G0271388 | cog8 | oligomeric Golgi complex component, Dor1-like protein |
| inparanoid | 148772 | DDB_G0271608 | polZ | DNA polymerase zeta catalytic subunit |
| inparanoid | 148776 | DDB_G0272682 | DDB_G0272682 | endonuclease/exonuclease/phosphatase domain-containing protein, cry34 related protein |
| inparanoid | 148777 | DDB_G0271514 | rpc5 | putative RNA polymerase III subunit |
| inparanoid | 148783 | DDB_G0272863 | DDB_G0272863 |  |
| inparanoid | 148788 | DDB_G0294094 | lrrA | leucine-rich repeat-containing protein (LRR) |
| inparanoid | 148794 | DDB_G0269936 | DDB_G0269936 |  |
| inparanoid | 148822 | DDB_G0290785 | DDB_G0290785 |  |
| inparanoid | 148826 | DDB_G0292016 | DDB_G0292016 |  |
| inparanoid | 148855 | DDB_G0272624 | adrm1-1 | adhesion regulating molecule family protein |
| inparanoid | 148856 | DDB_G0272791 | rsc11-1 | alpha/beta hydrolase fold-1 domain-containing protein |
| inparanoid | 148868 | DDB_G0272600 | DDB_G0272600 | unknown |
| inparanoid | 148904 | DDB_G0290919 | smc5 | structural maintenance of chromosome protein, AAA ATPase domain-containing protein |
| inparanoid | 148930 | DDB_G0290471 | DDB_G0290471 | protein kinase, TKL group, tyrosine kinase-like protein, ARK family protein kinase |
| inparanoid | 148931 | DDB_G0290473 | DDB_G0290473 |  |
| inparanoid | 148953 | DDB_G0272158 | DDB_G0272158 | PUTATIVE HELICASE RUVBL |
| inparanoid | 148983 | DDB_G0288215 | DDB_G0288215 |  |
| inparanoid | 148988 | DDB_G0286559 | abcC5 | ABC transporter C family protein |
| inparanoid | 148991 | DDB_G0287733 | syn7A | syntaxin 7, t-SNARE family protein |
| inparanoid | 148995 | DDB_G0289181 | gnb1l | guanine nucleotide-binding protein subunit beta-like protein 1 |
| inparanoid | 148999 | DDB_G0292618 | ogg1 | 8-oxoguanine DNA-glycosylase, DNA-formamidopyrimidine glycosylase, mutM homolog |
| inparanoid | 149003 | DDB_G0277847 | pdhC | dihydrolipoamide acetyltransferase, dihydrolipoyllysine-residue acetyltransferase, pyruvate dehydrogenase E2 component |
| inparanoid | 149015 | DDB_G0279689 | eftud1 | elongation factor Tu domain-containing protein, small GTP-binding protein domain-containing protein, band 7 family protein |
| inparanoid | 149032 | DDB_G0270734 | DDB_G0270734 |  |
| inparanoid | 149033 | DDB_G0270778 | DDB_G0270778 | putative prolyl 4-hydroxylase alpha subunit |
| inparanoid | 149038 | DDB_G0269170 | pmpA | putative membrane protein |
| inparanoid | 149039 | DDB_G0270014 | DDB_G0270014 |  |
| inparanoid | 149041 | DDB_G0275071 | fahd1 | Fumarylacetoacetate (FAA) hydrolase domain-containing protein |
| inparanoid | 149048 | DDB_G0275075 | DDB_G0275075 | tetratricopeptide-like helical domain-containing protein (TPR) |
| inparanoid | 149056 | DDB_G0270892 | DDB_G0270892 |  |
| inparanoid | 149073 | DDB_G0269588 | DDB_G0269588 | FadA6_3. |
| inparanoid | 149075 | DDB_G0269598 | DDB_G0269598 |  |
| inparanoid | 149085 | DDB_G0270018 | DDB_G0270018 |  |
| inparanoid | 149089 | DDB_G0269342 | DDB_G0269342 | unknown |
| inparanoid | 149103 | DDB_G0285553 | cycL | cyclin |
| inparanoid | 149105 | DDB_G0280963 | rrpC | RNA-directed RNA polymerase |
| inparanoid | 149107 | DDB_G0279627 | DDB_G0279627 |  |
| inparanoid | 149114 | DDB_G0281603 | DDB_G0281603 |  |
| inparanoid | 149116 | DDB_G0281599 | DDB_G0281599 | CG15084 protein (GH26994p). |
| inparanoid | 149119 | DDB_G0292886 | DDB_G0292886 |  |
| inparanoid | 149128 | DDB_G0280385 | DDB_G0280385 |  |
| inparanoid | 149133 | DDB_G0280263 | DDB_G0280263 | glycoside hydrolase family 16 protein |
| inparanoid | 149141 | DDB_G0279483 | pldB | phospholipase D1 |
| inparanoid | 149145 | DDB_G0279487 | DDB_G0279487 | HEAT repeat-containing protein, armadillo-like helical domain-containing protein, GCN1-like protein |
| inparanoid | 149158 | DDB_G0278679 | CYP513F1 | cytochrome P450 family protein |
| inparanoid | 149161 | DDB_G0279477 | DDB_G0279477 |  |
| inparanoid | 149165 | DDB_G0278759 | DDB_G0278759 |  |
| inparanoid | 149166 | DDB_G0278383 | DDB_G0278383 |  |
| inparanoid | 149168 | DDB_G0278379 | bzpD | putative basic-leucine zipper (bZIP) transcription factor |
| inparanoid | 14919 | DDB_G0275899 | DDB_G0275899 | Similar to Dictyostelium discoideum (Slime mold). diacylglycerol kinase protein DgkA. |
| inparanoid | 149208 | DDB_G0276355 | dhcA | cytoplasmic dynein heavy chain, dynein beta chain, flagellar outer arm |
| inparanoid | 149221 | DDB_G0286735 | DDB_G0286735 |  |
| inparanoid | 149232 | DDB_G0285827 | DDB_G0285827 |  |
| inparanoid | 149244 | DDB_G0275661 | DDB_G0275661 |  |
| inparanoid | 149252 | DDB_G0278119 | DDB_G0278119 |  |
| inparanoid | 149253 | DDB_G0286603 | DDB_G0286603 | glutamine-fructose-6-phosphate transaminase (isomerizing), glucosamine-fructose-6-phosphate aminotransferase |
| inparanoid | 149255 | DDB_G0286693 | adcD | FYVE-type zinc finger-containing protein, arrestin domain-containing protein |
| inparanoid | 149262 | DDB_G0269558 | gnt11 | GlcNAc transferase, glycosyltransferase, beta-1,6-N-acetylglucosaminyltransferase, core-2/I-branching enzyme |
| inparanoid | 149273 | DDB_G0285875 | DDB_G0285875 | U3 snoRNP protein, U3 small nucleolar ribonucleoprotein, BP28, C-terminal domain-containing protein |
| inparanoid | 149280 | DDB_G0293438 | abcB2 | ABC transporter B family protein |
| inparanoid | 149310 | DDB_G0275451 | alxA | ALG-2 interacting protein X |
| inparanoid | 149347 | DDB_G0288795 | DDB_G0288795 | putative protein serine/threonine kinase, protein kinase, AGC group, AKT family protein kinase |
| inparanoid | 149353 | DDB_G0284561 | DDB_G0284561 |  |
| inparanoid | 149356 | DDB_G0284559 | DDB_G0284559 | Similar to Dictyostelium discoideum (Slime mold). hypothetical 97.7 kDa protein. |
| inparanoid | 149357 | DDB_G0279285 | DDB_G0279285 | UBX domain-containing protein |
| inparanoid | 149369 | DDB_G0284223 | DDB_G0284223 | putative metallophosphoesterase |
| inparanoid | 149370 | DDB_G0290307 | DDB_G0290307 | RNA-binding region RNP-1 domain-containing protein, RNA recognition motif-containing protein RRM, ELAVL1-like protein |
| inparanoid | 149375 | DDB_G0272815 | cprE | cysteine proteinase 5 precursor |
| inparanoid | 149379 | DDB_G0274389 | pARTf | poly(ADP-ribose) polymerase, catalytic region domain-containing protein |
| inparanoid | 149385 | DDB_G0290297 | xrcc2 | AAA ATPase domain-containing protein, putative DNA repair protein XRCC2 |
| inparanoid | 149390 | DDB_G0284439 | DDB_G0284439 |  |
| inparanoid | 149391 | DDB_G0284021 | DDB_G0284021 |  |
| inparanoid | 149427 | DDB_G0291800 | DDB_G0291800 | glutathione-dependent formaldehyde-activating, GFA family protein |
| inparanoid | 149445 | DDB_G0272456 | DDB_G0272456 | HEAT repeat-containing protein, armadillo-like helical domain-containing protein |
| inparanoid | 149447 | DDB_G0273137 | DDB_G0273137 | HYPOTHETICAL 83.2 KDA PROTEIN |
| inparanoid | 149460 | DDB_G0271946 | DDB_G0271946 | Similar to Staphylococcus epidermidis ATCC 12228. streptococcal hemagglutinin protein. |
| inparanoid | 149465 | DDB_G0284767 | DDB_G0284767 | unknown |
| inparanoid | 149471 | DDB_G0273285 | DDB_G0273285 | Similar to Dictyostelium discoideum (Slime mold). prespore-specific protein. |
| inparanoid | 149503 | DDB_G0280193 | DDB_G0280193 |  |
| inparanoid | 149504 | DDB_G0280195 | DDB_G0280195 | FYVE-type zinc finger-containing protein, HMG-I and HMG-Y, DNA-binding domain-containing protein, histone deacetylase family protein |
| inparanoid | 149505 | DDB_G0280197 | sepsecs | O-phosphoseryl-tRNA(Sec) selenium transferase |
| inparanoid | 149510 | DDB_G0271338 | taf7 | transcription initiation factor TFIID subunit, TFIID subunit |
| inparanoid | 149515 | DDB_G0291336 | DDB_G0291336 |  |
| inparanoid | 149523 | DDB_G0291303 | frmA | FERM domain-containing protein, talin family protein |
| inparanoid | 149527 | DDB_G0292010 | DDB_G0292010 |  |
| inparanoid | 149540 | DDB_G0275165 | DDB_G0275165 | LISK family protein kinase, protein kinase, TKL group, tyrosine kinase-like protein |
| inparanoid | 149546 | DDB_G0275181 | DDB_G0275181 | methylmalonyl-CoA epimerase, methylmalonyl coenzyme A racemase |
| inparanoid | 149549 | DDB_G0292124 | DDB_G0292124 |  |
| inparanoid | 149555 | DDB_G0292128 | DDB_G0292128 |  |
| inparanoid | 149573 | DDB_G0283977 | DDB_G0283977 | Probable short-chain dehydrogenase. |
| inparanoid | 149595 | DDB_G0283745 | DDB_G0283745 | armadillo-like helical domain-containing protein, transportin 3-like protein |
| inparanoid | 149604 | DDB_G0279193 | rpb1 | RNA polymerase II largest subunit, RNA polymerase II core subunit |
| inparanoid | 149605 | DDB_G0279221 | DDB_G0279221 |  |
| inparanoid | 149614 | DDB_G0269892 | DDB_G0269892 |  |
| inparanoid | 149615 | DDB_G0269890 | DDB_G0269890 | RH19667p. |
| inparanoid | 149620 | DDB_G0282993 | sodC | superoxide dismutase |
| inparanoid | 149635 | DDB_G0280045 | thyA | thymidylate synthase (FAD) |
| inparanoid | 149647 | DDB_G0280551 | DDB_G0280551 |  |
| inparanoid | 149674 | DDB_G0267510 | DDB_G0267510 |  |
| inparanoid | 149681 | DDB_G0269466 | DDB_G0269466 |  |
| inparanoid | 149688 | DDB_G0295761 | nle1 | WD40 repeat-containing protein, NLE domain-containing protein, putative notchless |
| inparanoid | 149698 | DDB_G0294617 | DDB_G0294617 | unknown |
| inparanoid | 149700 | DDB_G0286803 | vps41 | RING zinc finger-containing protein, WD40 repeat-containing protein, 7-fold repeat in clathrin and VPS proteins repeat-containing protein |
| inparanoid | 149704 | DDB_G0277861 | patA | P-type ATPase, Ca2+-ATPase |
| inparanoid | 149711 | DDB_G0270100 | DDB_G0270100 |  |
| inparanoid | 149720 | DDB_G0270626 | armc8 | armadillo repeat-containing protein |
| inparanoid | 149721 | DDB_G0277473 | DDB_G0277473 | triacylglycerol lipase-like protein triacylglycerol lipase |
| inparanoid | 149723 | DDB_G0291139 | DDB_G0291139 |  |
| inparanoid | 149724 | DDB_G0268890 | DDB_G0268890 |  |
| inparanoid | 149728 | DDB_G0268862 | DDB_G0268862 |  |
| inparanoid | 149730 | DDB_G0268868 | nat10 | putative N-acetyltransferase |
| inparanoid | 149732 | DDB_G0269688 | sdad1 | SDA1 domain-containing protein |
| inparanoid | 149748 | DDB_G0270912 | DDB_G0270912 |  |
| inparanoid | 149767 | DDB_G0268578 | gefV | RasGEF domain-containing protein, leucine-rich repeat-containing protein (LRR) |
| inparanoid | 149769 | DDB_G0267960 | cog4 | oligomeric Golgi complex component |
| inparanoid | 149772 | DDB_G0268404 | DDB_G0268404 | DENN domain-containing protein |
| inparanoid | 149785 | DDB_G0291836 | DDB_G0291836 |  |
| inparanoid | 149786 | DDB_G0291820 | DDB_G0291820 |  |
| inparanoid | 149802 | DDB_G0276575 | syn16A | t-SNARE family protein, putative syntaxin 16 |
| inparanoid | 149815 | DDB_G0276615 | hmgB | hydroxymethylglutaryl CoA reductase, hmg CoA reductase B |
| inparanoid | 149816 | DDB_G0276625 | DDB_G0276625 |  |
| inparanoid | 149843 | DDB_G0277515 | DDB_G0277515 | xanthine/uracil permease family protein |
| inparanoid | 149867 | DDB_G0286701 | DDB_G0286701 | Putative initiator tRNA phosphoribosyl-transferase. |
| inparanoid | 149879 | DDB_G0280797 | DDB_G0280797 |  |
| inparanoid | 149887 | DDB_G0275821 | DDB_G0275821 |  |
| inparanoid | 149889 | DDB_G0275823 | DDB_G0275823 | Similar to Dictyostelium discoideum (Slime mold). hybrid histidine kinase DHKB. |
| inparanoid | 149905 | DDB_G0269484 | DDB_G0269484 |  |
| inparanoid | 149906 | DDB_G0280907 | xpo4 | exportin 4 |
| inparanoid | 149923 | DDB_G0290403 | DDB_G0290403 | unknown |
| inparanoid | 149933 | DDB_G0283055 | DDB_G0283055 | RabGAP/TBC domain-containing protein, TBC1 domain family protein |
| inparanoid | 149934 | DDB_G0282939 | DDB_G0282939 |  |
| inparanoid | 149944 | DDB_G0283275 | DDB_G0283275 |  |
| inparanoid | 149945 | DDB_G0283269 | DDB_G0283269 | putative asparaginase 2 |
| inparanoid | 149947 | DDB_G0283329 | mcfO | EF-hand domain-containing protein, mitochondrial substrate carrier family protein, putative calcium-binding mitochondrial carrier protein |
| inparanoid | 149960 | DDB_G0284471 | kif8 | WD40 repeat-containing protein, kinesin-4, kinesin family member 8 |
| inparanoid | 149992 | DDB_G0282573 | DDB_G0282573 | WD40 repeat-containing protein |
| inparanoid | 149995 | DDB_G0282087 | DDB_G0282087 |  |
| inparanoid | 150002 | DDB_G0279717 | DDB_G0279717 | carboxylesterase, type B family protein |
| inparanoid | 150014 | DDB_G0279693 | DDB_G0279693 |  |
| inparanoid | 150024 | DDB_G0268742 | DDB_G0268742 | Chrysanthemyl diphosphate synthase. |
| inparanoid | 150026 | DDB_G0295679 | DDB_G0295679 | helicase superfamily 1 and 2 domain-containing protein, type III restriction enzyme, res subunit family protein |
| inparanoid | 150028 | DDB_G0278169 | DDB_G0278169 |  |
| inparanoid | 150029 | DDB_G0278171 | DDB_G0278171 |  |
| inparanoid | 150037 | DDB_G0277899 | DDB_G0277899 | RNA-binding region RNP-1 domain-containing protein |
| inparanoid | 150040 | DDB_G0278163 | DDB_G0278163 | TM2 domain-containing protein |
| inparanoid | 150046 | DDB_G0278239 | DDB_G0278239 |  |
| inparanoid | 150047 | DDB_G0278639 | clcD | chloride channel protein, CLC 6/7 family protein |
| inparanoid | 150055 | DDB_G0278023 | mrpl33 | ribosomal protein L33, mitochondrial |
| inparanoid | 150060 | DDB_G0295697 | rabif | Mss4-like family protein, Rab guanyl-nucleotide exchange factor |
| inparanoid | 150062 | DDB_G0277973 | DDB_G0277973 |  |
| inparanoid | 150069 | DDB_G0278249 | DDB_G0278249 |  |
| inparanoid | 150076 | DDB_G0280705 | DDB_G0280705 | myb domain-containing protein, chromo (CHRomatin Organization MOdifier) domain-containing protein, SNF2-related domain-containing protein, CHR group protein, helicase, C-terminal domain-containing protein |
| inparanoid | 150090 | DDB_G0287803 | argS2 | arginyl-tRNA synthetase, arginine-tRNA ligase |
| inparanoid | 150096 | DDB_G0293002 | tagA | ABC transporter B family protein, serine protease |
| inparanoid | 150117 | DDB_G0275593 | DDB_G0275593 | HYPOTHETICAL 38.7 KDA PROTEIN |
| inparanoid | 150121 | DDB_G0275149 | DDB_G0275149 | Ankyrin repeat-containing protein AKR1. |
| inparanoid | 150130 | DDB_G0286511 | DDB_G0286511 | putative ubiquitin-conjugating enzyme E2 |
| inparanoid | 150141 | DDB_G0286753 | eIF5 | eukaryotic translation initiation factor 5 |
| inparanoid | 150147 | DDB_G0286517 | mlysS | leucyl-tRNA synthetase, lysine-tRNA ligase, putative mitochondrial leucyl-tRNA synthetase, putative mitochondrial lysine-tRNA ligase |
| inparanoid | 150161 | DDB_G0288053 | empA | emp24/gp25L/p24 family protein |
| inparanoid | 150163 | DDB_G0288181 | hspA | chaperonin 60, heat shock 60 |
| inparanoid | 150171 | DDB_G0272809 | DDB_G0272809 | Similar to Dictyostelium discoideum (Slime mold). CIGB protein. |
| inparanoid | 150174 | DDB_G0288183 | roco9 | leucine-rich repeat-containing protein (LRR), RhoGAP domain-containing protein, protein kinase, TKL group, tyrosine kinase-like protein, ROCO family protein kinase |
| inparanoid | 150175 | DDB_G0288259 | papA | poly(A) polymerase |
| inparanoid | 150187 | DDB_G0270210 | cbpJ | calcium-binding protein, NCS-1/frequenin-related protein |
| inparanoid | 150195 | DDB_G0290069 | pitA | phosphatidylinositol transfer protein 1 |
| inparanoid | 150199 | DDB_G0279367 | DDB_G0279367 |  |
| inparanoid | 150212 | DDB_G0287043 | DDB_G0287043 |  |
| inparanoid | 150215 | DDB_G0278151 | DDB_G0278151 |  |
| inparanoid | 150216 | DDB_G0278153 | tmem111 | transmembrane protein, DUF850 family protein, TMEM111 family protein |
| inparanoid | 150220 | DDB_G0278347 | DDB_G0278347 |  |
| inparanoid | 150223 | DDB_G0278351 | DDB_G0278351 |  |
| inparanoid | 150228 | DDB_G0278257 | DDB_G0278257 |  |
| inparanoid | 150230 | DDB_G0278057 | arrL | ADP-ribosylation factor-related, ARF-related |
| inparanoid | 150238 | DDB_G0278157 | sdhD | succinate dehydrogenase (ubiquinone), succinic dehydrogenase, complex II, cytochrome b small subunit |
| inparanoid | 150244 | DDB_G0269022 | DDB_G0269022 |  |
| inparanoid | 150265 | DDB_G0269502 | DDB_G0269502 | putative nucleoporin 160 |
| inparanoid | 150274 | DDB_G0293514 | DDB_G0293514 | Transcriptional regulatory protein. |
| inparanoid | 150275 | DDB_G0271268 | DDB_G0271268 |  |
| inparanoid | 150282 | DDB_G0280923 | DDB_G0280923 |  |
| inparanoid | 150294 | DDB_G0281213 | pex3 | transmembrane protein, peroxisomal biogenesis factor 3, peroxin 3 |
| inparanoid | 150326 | DDB_G0285679 | nol5a | NOP5 family protein |
| inparanoid | 150327 | DDB_G0285677 | DDB_G0285677 |  |
| inparanoid | 150380 | DDB_G0293842 | DDB_G0293842 | unknown |
| inparanoid | 150386 | DDB_G0293636 | DDB_G0293636 | putative splicing factor |
| inparanoid | 150403 | DDB_G0293656 | fhkA | putative protein serine/threonine kinase, protein kinase, CAMK group, RAD53 family protein kinase, FHA domain-containing protein |
| inparanoid | 150404 | DDB_G0293658 | DDB_G0293658 |  |
| inparanoid | 150406 | DDB_G0291366 | DDB_G0291366 |  |
| inparanoid | 150412 | DDB_G0291380 | atr1 | protein kinase, Atypical group, phosphatidylinositol 3-kinase-related protein kinase, ATR subfamily protein kinase, PIKK family protein kinase |
| inparanoid | 150414 | DDB_G0291650 | DDB_G0291650 | importin subunit beta-3 |
| inparanoid | 150424 | DDB_G0291436 | DDB_G0291436 | Q86SQ9 Dehydrodolichyl diphosphate synthase (EC 2.5.1.-) (Dedol-PP synthase). |
| inparanoid | 150433 | DDB_G0274675 | DDB_G0274675 |  |
| inparanoid | 150434 | DDB_G0291868 | rfc2 | replication factor C subunit |
| inparanoid | 150438 | DDB_G0280007 | DDB_G0280007 |  |
| inparanoid | 150489 | DDB_G0277155 | tom40 | translocase of outer mitochondrial membrane, mitochondrial porin |
| inparanoid | 150490 | DDB_G0277157 | nubp2 | nucleotide binding protein 2 |
| inparanoid | 150495 | DDB_G0282215 | DDB_G0282215 | AAA ATPase domain-containing protein |
| inparanoid | 150496 | DDB_G0282223 | DDB_G0282223 |  |
| inparanoid | 150499 | DDB_G0281167 | dnapkcs | DNA-dependent protein kinase (DNAPK) subunit, protein kinase, Atypical group, phosphatidylinositol 3-kinase-related protein kinase, PIKK family protein kinase, DNAPK subfamily protein kinase |
| inparanoid | 150514 | DDB_G0277381 | cpras1 | type A von Willebrand factor (VWFA) domain-containing protein, Ras GTPase domain-containing protein |
| inparanoid | 150516 | DDB_G0277427 | DDB_G0277427 |  |
| inparanoid | 150525 | DDB_G0284257 | dph1 | diphthamide biosynthesis protein 1 |
| inparanoid | 150541 | DDB_G0285123 | DDB_G0285123 |  |
| inparanoid | 150543 | DDB_G0285159 | stt3 | oligosaccharyl transferase, STT3 subunit, glycosyltransferase, oligosaccharyltransferase, dolichyl-diphosphooligosaccharide-protein glycotransferase |
| inparanoid | 150556 | DDB_G0285141 | osbJ | oxysterol binding family protein, member 10 |
| inparanoid | 150569 | DDB_G0292820 | adprt2 | poly(ADP-ribosyl)transferase, NAD+ ADP-ribosyltransferase |
| inparanoid | 150570 | DDB_G0292836 | DDB_G0292836 | peptidase A22B family protein |
| inparanoid | 150573 | DDB_G0292840 | cysK | cysteine synthase |
| inparanoid | 150585 | DDB_G0279761 | DDB_G0279761 | putative protein serine/threonine kinase, protein kinase, Atypical group, G11 family protein kinase |
| inparanoid | 150587 | DDB_G0279765 | DDB_G0279765 |  |
| inparanoid | 150594 | DDB_G0279803 | DDB_G0279803 |  |
| inparanoid | 150600 | DDB_G0290457 | DDB_G0290457 |  |
| inparanoid | 150605 | DDB_G0292688 | commd8 | COMM domain-containing protein 8 |
| inparanoid | 150613 | DDB_G0292328 | DDB_G0292328 |  |
| inparanoid | 150614 | DDB_G0292294 | hemE | uroporphyrinogen decarboxylase |
| inparanoid | 150618 | DDB_G0292270 | comG | aldehyde dehydrogenase, putative NAD-dependent aldehyde dehydrogenase |
| inparanoid | 150632 | DDB_G0279565 | DDB_G0279565 |  |
| inparanoid | 150633 | DDB_G0291430 | DDB_G0291430 |  |
| inparanoid | 150642 | DDB_G0293226 | rvb1 | AAA ATPase domain-containing protein, RuvB-like protein 1 |
| inparanoid | 150644 | DDB_G0293222 | DDB_G0293222 |  |
| inparanoid | 150645 | DDB_G0282243 | nhp2l1 | U4/U6 small nuclear ribonucleoprotein, non-histone chromosome protein 2-like 1, high mobility group-like nuclear protein |
| inparanoid | 150653 | DDB_G0270224 | DDB_G0270224 |  |
| inparanoid | 150656 | DDB_G0289121 | pde4 | cAMP-specific phosphodiesterase |
| inparanoid | 150657 | DDB_G0289141 | DDB_G0289141 | ankyrin repeat-containing protein, poly(ADP-ribosyl)transferase, NAD+ ADP-ribosyltransferase, BRCT domain-containing protein |
| inparanoid | 150660 | DDB_G0289137 | DDB_G0289137 |  |
| inparanoid | 150701 | DDB_G0285083 | DDB_G0285083 |  |
| inparanoid | 150706 | DDB_G0284979 | DDB_G0284979 |  |
| inparanoid | 150717 | DDB_G0285073 | DDB_G0285073 | vacuolar sorting protein 9 domain-containing protein |
| inparanoid | 150724 | DDB_G0276407 | DDB_G0276407 |  |
| inparanoid | 150730 | DDB_G0279593 | DDB_G0279593 | D111/G-patch domain-containing protein, ribosomal RNA methyltransferase RrmJ/FtsJ domain-containing protein |
| inparanoid | 150733 | DDB_G0282951 | DDB_G0282951 | unknown |
| inparanoid | 150734 | DDB_G0282953 | DDB_G0282953 | unknown |
| inparanoid | 150753 | DDB_G0274199 | DDB_G0274199 | putative metallophosphoesterase |
| inparanoid | 150765 | DDB_G0279675 | DDB_G0279675 |  |
| inparanoid | 150768 | DDB_G0279059 | DDB_G0279059 | mannose-6-phosphate receptor domain-containing protein |
| inparanoid | 150779 | DDB_G0279633 | psmD7 | 26S proteasome non-ATPase regulatory subunit 7, 26S proteasome regulatory subunit S12, Mov34 domain-containing protein |
| inparanoid | 150797 | DDB_G0275303 | DDB_G0275303 |  |
| inparanoid | 150798 | DDB_G0290431 | DDB_G0290431 |  |
| inparanoid | 150804 | DDB_G0268100 | asnS2 | asparagine-tRNA ligase, asparaginyl-tRNA synthetase |
| inparanoid | 150808 | DDB_G0269708 | DDB_G0269708 | WH2 domain-containing protein, SH3 domain-containing protein |
| inparanoid | 150810 | DDB_G0276255 | DDB_G0276255 | Similar to Protein required for cell viability; Yjl010cp. |
| inparanoid | 150811 | DDB_G0276253 | DDB_G0276253 | Similar to Solanum tuberosum (Potato). Fructose-6-phosphate 2-kinase/fructose-2,6-bisphosphatase (EC 2.7.1.105). |
| inparanoid | 150816 | DDB_G0290395 | DDB_G0290395 |  |
| inparanoid | 150822 | DDB_G0272572 | DDB_G0272572 |  |
| inparanoid | 150832 | DDB_G0274593 | dst1 | putative protein serine/threonine kinase, STE20 family protein kinase, protein kinase, STE group |
| inparanoid | 150847 | DDB_G0274953 | DDB_G0274953 | Similar to Dictyostelium discoideum (Slime mold). inositol 5-phosphatase 2. |
| inparanoid | 150849 | DDB_G0278857 | DDB_G0278857 |  |
| inparanoid | 150851 | DDB_G0279313 | pigN | phosphatidylinositol glycan, class N |
| inparanoid | 150863 | DDB_G0278849 | etfdh | electron transfer flavoprotein-ubiquinone oxidoreductase, electron-transferring-flavoprotein dehydrogenase |
| inparanoid | 150876 | DDB_G0281789 | DDB_G0281789 | Phosphomannomutase (Pmm). |
| inparanoid | 150880 | DDB_G0281841 | ddx24 | putative RNA helicase, DEAD/DEAH box helicase |
| inparanoid | 150881 | DDB_G0281839 | DDB_G0281839 | WD40 repeat-containing protein, BOP1, N-terminal domain-containing protein |
| inparanoid | 150882 | DDB_G0281837 | DDB_G0281837 |  |
| inparanoid | 150883 | DDB_G0281835 | DDB_G0281835 |  |
| inparanoid | 150888 | DDB_G0280455 | ascc2 | ubiquitin system component Cue domain containing protein, activating signal cointegrator 1 complex subunit |
| inparanoid | 150899 | DDB_G0275829 | DDB_G0275829 |  |
| inparanoid | 150900 | DDB_G0294407 | helF | putative RNA helicase, dsRNA-binding Protein, DEAD/DEAH box helicase |
| inparanoid | 150917 | DDB_G0277369 | DDB_G0277369 | NAP (nucleosome assembly protein) family protein |
| inparanoid | 150921 | DDB_G0280801 | DDB_G0280801 |  |
| inparanoid | 150923 | DDB_G0280353 | odhA | oxoglutarate dehydrogenase (succinyl-transferring), alpha-ketoglutarate dehydrogenase, 2-oxoglutarate dehydrogenase E1 component |
| inparanoid | 150924 | DDB_G0280351 | gpaL | G-protein subunit alpha 12 |
| inparanoid | 150981 | DDB_G0272454 | DDB_G0272454 | RGS-containing protein kinase RCK1. |
| inparanoid | 150983 | DDB_G0272234 | pex2 | RING zinc finger-containing protein, peroxisomal biogenesis factor 2, peroxin 2 |
| inparanoid | 150984 | DDB_G0272232 | DDB_G0272232 | putative transmembrane protein |
| inparanoid | 150985 | DDB_G0272230 | DDB_G0272230 | Similar to Protein forms dimers in vivo and in vitro, contains a conserved YPCXXC motif at carboxyl-terminal, binds FAD as a cofactor, and catalyzes the formation of disulfide bonds in protein substrates.; Erv1p. |
| inparanoid | 150991 | DDB_G0275125 | plbF | phospholipase B-like protein |
| inparanoid | 151024 | DDB_G0272252 | DDB_G0272252 |  |
| inparanoid | 151030 | DDB_G0286059 | asns | asparagine synthetase |
| inparanoid | 151033 | DDB_G0286063 | DDB_G0286063 |  |
| inparanoid | 151034 | DDB_G0285541 | DDB_G0285541 | At2g39450 protein (Hypothetical protein). |
| inparanoid | 151074 | DDB_G0287313 | DDB_G0287313 |  |
| inparanoid | 151088 | DDB_G0281947 | tra1 | protein kinase, Atypical group, phosphatidylinositol 3-kinase-related protein kinase, PIKK family protein kinase, TRRAP subfamily protein kinase |
| inparanoid | 151089 | DDB_G0281827 | dwwA | WW domain-containing protein |
| inparanoid | 151095 | DDB_G0286207 | DDB_G0286207 | Similar to Oryza sativa (Rice). hydroxyproline-rich glycoprotein. |
| inparanoid | 151099 | DDB_G0286633 | ints4 | HEAT repeat-containing protein, armadillo-like helical domain-containing protein, integrator complex subunit 4 |
| inparanoid | 151114 | DDB_G0269348 | DDB_G0269348 | CG9386 protein (RE33302p). |
| inparanoid | 151115 | DDB_G0269352 | mcfD | mitochondrial substrate carrier family protein |
| inparanoid | 151116 | DDB_G0270854 | DDB_G0270854 | O94404 Hypothetical UPF0135 protein C126.12 in chromosome III. |
| inparanoid | 151118 | DDB_G0269358 | DDB_G0269358 |  |
| inparanoid | 151122 | DDB_G0270492 | DDB_G0270492 |  |
| inparanoid | 151136 | DDB_G0293306 | uba5 | UBA/THIF-type NAD/FAD binding fold-containing protein, E1-like enzyme family protein |
| inparanoid | 151143 | DDB_G0292744 | DDB_G0292744 |  |
| inparanoid | 151152 | DDB_G0293070 | DDB_G0293070 | WD40 repeat-containing protein, TROVE domain-containing protein, NACHT domain-containing protein, telomerase-associated protein 1-like protein (TEP1) |
| inparanoid | 151153 | DDB_G0293068 | DDB_G0293068 |  |
| inparanoid | 151154 | DDB_G0293064 | DDB_G0293064 | CG10777 protein (LD32873P). |
| inparanoid | 151185 | DDB_G0291528 | iptB | putative isopentenyltransferase, putative isopentenylpyrophosphate (IPP) transferase |
| inparanoid | 151187 | DDB_G0286259 | DDB_G0286259 |  |
| inparanoid | 151213 | DDB_G0290799 | DDB_G0290799 | uncharacterised protein family, carbohydrate kinase-related |
| inparanoid | 151214 | DDB_G0280493 | DDB_G0280493 |  |
| inparanoid | 151217 | DDB_G0281159 | DDB_G0281159 |  |
| inparanoid | 151220 | DDB_G0281261 | DDB_G0281261 | putative G-patch-containing protein |
| inparanoid | 151224 | DDB_G0281265 | DDB_G0281265 |  |
| inparanoid | 151237 | DDB_G0289755 | DDB_G0289755 | B1075D06.2 protein. |
| inparanoid | 151241 | DDB_G0289697 | DDB_G0289697 | FAD dependent oxidoreductase domain-containing protein, berberine domain-containing protein |
| inparanoid | 151248 | DDB_G0289699 | nola2 | ribosomal protein L7Ae, H/ACA RNP complex subunit 2 |
| inparanoid | 151253 | DDB_G0284917 | DDB_G0284917 |  |
| inparanoid | 151263 | DDB_G0289519 | DDB_G0289519 |  |
| inparanoid | 151275 | DDB_G0287947 | DDB_G0287947 |  |
| inparanoid | 151284 | DDB_G0287959 | DDB_G0287959 |  |
| inparanoid | 151302 | DDB_G0287213 | rcdJJ | random cDNA clone veg110 |
| inparanoid | 151347 | DDB_G0292470 | anapc7 | anaphase promoting complex subunit 7 |
| inparanoid | 151351 | DDB_G0292462 | DDB_G0292462 |  |
| inparanoid | 151370 | DDB_G0288569 | DDB_G0288569 | unknown |
| inparanoid | 151371 | DDB_G0287107 | DDB_G0287107 |  |
| inparanoid | 151373 | DDB_G0287495 | pyrK | UMP-CMP kinase, cytidylate kinase, uridylate kinase, uridine monophosphate/cytidine monophosphate kinase |
| inparanoid | 151379 | DDB_G0279671 | DDB_G0279671 |  |
| inparanoid | 151385 | DDB_G0270360 | bud31 | putative RNA splicing factor, putative transcription factor |
| inparanoid | 151386 | DDB_G0269658 | DDB_G0269658 |  |
| inparanoid | 151403 | DDB_G0294597 | DDB_G0294597 | beta-lactamase family protein |
| inparanoid | 151413 | DDB_G0290057 | DDB_G0290057 |  |
| inparanoid | 151417 | DDB_G0270216 | DDB_G0270216 |  |
| inparanoid | 151426 | DDB_G0284079 | DDB_G0284079 | ankyrin repeat-containing protein, RA domain-containing protein |
| inparanoid | 151427 | DDB_G0283565 | DDB_G0283565 |  |
| inparanoid | 151438 | DDB_G0272176 | DDB_G0272176 | las1-like protein |
| inparanoid | 151469 | DDB_G0290877 | DDB_G0290877 |  |
| inparanoid | 151475 | DDB_G0284903 | culC | cullin C |
| inparanoid | 151484 | DDB_G0275009 | gcA | guanylyl cyclase |
| inparanoid | 151501 | DDB_G0276689 | DDB_G0276689 | unknown |
| inparanoid | 151521 | DDB_G0290209 | med10 | putative mediator complex subunit 10 |
| inparanoid | 151533 | DDB_G0290107 | DDB_G0290107 |  |
| inparanoid | 151544 | DDB_G0290077 | mlcB | myoB light chain |
| inparanoid | 151547 | DDB_G0290083 | DDB_G0290083 | phenylacetic acid degradation-related protein |
| inparanoid | 151554 | DDB_G0287517 | DDB_G0287517 |  |
| inparanoid | 151558 | DDB_G0288565 | bysl | bystin |
| inparanoid | 151559 | DDB_G0288485 | pikD | phosphatidylinositol 4-kinase, phosphatidylinositol 4-kinase catalytic subunit, beta polypeptide |
| inparanoid | 151565 | DDB_G0287979 | DDB_G0287979 |  |
| inparanoid | 151566 | DDB_G0285843 | helD | DEAD/DEAH box helicase, putative RNA splicing factor |
| inparanoid | 151574 | DDB_G0287725 | DDB_G0287725 |  |
| inparanoid | 151576 | DDB_G0287557 | pno1 | RNA-binding protein PNO1 |
| inparanoid | 151580 | DDB_G0288045 | DDB_G0288045 |  |
| inparanoid | 151593 | DDB_G0271956 | DDB_G0271956 | RING zinc finger-containing protein, IBR zinc finger-containing protein |
| inparanoid | 151604 | DDB_G0283381 | DDB_G0283381 |  |
| inparanoid | 151610 | DDB_G0269522 | DDB_G0269522 |  |
| inparanoid | 151611 | DDB_G0269526 | argD | acetylornithine transaminase, acetylornithine 5-aminotransferase |
| inparanoid | 151633 | DDB_G0286383 | DDB_G0286383 |  |
| inparanoid | 151634 | DDB_G0286271 | DDB_G0286271 |  |
| inparanoid | 151635 | DDB_G0286269 | DDB_G0286269 | putative transmembrane protein |
| inparanoid | 151645 | DDB_G0288143 | DDB_G0288143 | lysozyme C family protein |
| inparanoid | 151646 | DDB_G0286939 | DDB_G0286939 |  |
| inparanoid | 151665 | DDB_G0276857 | DDB_G0276857 | HYPOTHETICAL 34.2 KDA PROTEIN |
| inparanoid | 151707 | DDB_G0267714 | sf3b4 | RNA-binding region RNP-1 domain-containing protein, RNA recognition motif-containing protein RRM, splicing factor 3B subunit 4 |
| inparanoid | 151708 | DDB_G0285717 | gloB1 | hydroxyacylglutathione hydrolase, glyoxylase II, beta-lactamase domain-containing protein |
| inparanoid | 151723 | DDB_G0279095 | ptsA | 6-pyruvoyltetrahydropterin synthase |
| inparanoid | 151724 | DDB_G0278913 | DDB_G0278913 |  |
| inparanoid | 151725 | DDB_G0278577 | DDB_G0278577 |  |
| inparanoid | 151775 | DDB_G0284463 | maea | lissencephaly type-1-like homology (LisH) motif-containing protein, CT11-RanBPM domain-containing protein |
| inparanoid | 151776 | DDB_G0284475 | DDB_G0284475 |  |
| inparanoid | 151785 | DDB_G0276821 | DDB_G0276821 | aldehyde dehydrogenase |
| inparanoid | 151786 | DDB_G0276815 | DDB_G0276815 | Similar to Homo sapiens (Human). DKFZP564O0463 protein. |
| inparanoid | 151801 | DDB_G0290409 | DDB_G0290409 |  |
| inparanoid | 151809 | DDB_G0291656 | ykt6 | longin domain-containing protein, v-SNARE family protein |
| inparanoid | 151810 | DDB_G0291360 | dus4l | tRNA-dihydrouridine synthase 4-like protein |
| inparanoid | 151812 | DDB_G0291896 | DDB_G0291896 |  |
| inparanoid | 151816 | DDB_G0292052 | sec13 | WD40 repeat-containing protein, putative transport protein |
| inparanoid | 151830 | DDB_G0278113 | DDB_G0278113 | AgCP13922 (Fragment). |
| inparanoid | 151832 | DDB_G0295725 | DDB_G0295725 | unknown |
| inparanoid | 151839 | DDB_G0276485 | DDB_G0276485 |  |
| inparanoid | 151843 | DDB_G0275513 | DDB_G0275513 | HYPOTHETICAL PROTEIN PA3216. 3/101 |
| inparanoid | 151845 | DDB_G0275505 | DDB_G0275505 |  |
| inparanoid | 151846 | DDB_G0276279 | DDB_G0276279 | Similar to Arabidopsis thaliana (Mouse-ear cress). Hypothetical 34.3 kDa protein. |
| inparanoid | 15185 | DDB_G0280511 | DDB_G0280511 | Similar to Strongylocentrotus purpuratus (Purple sea urchin). Fibropellin I (Epidermal growth factor-related protein 1) (UEGF-1). |
| inparanoid | 151851 | DDB_G0276481 | ercc8 | WD40 repeat-containing protein, DNA excision repair protein 8 |
| inparanoid | 151859 | DDB_G0279945 | DDB_G0279945 |  |
| inparanoid | 151873 | DDB_G0289485 | vacA | vacuolin A, prohibitin domain-containing protein |
| inparanoid | 151874 | DDB_G0268446 | DDB_G0268446 | glycoside hydrolase family 7 protein, cellulose 1,4-beta-cellobiosidase, cellobiohydrolase I |
| inparanoid | 151875 | DDB_G0278859 | DDB_G0278859 |  |
| inparanoid | 151891 | DDB_G0290103 | elp3 | elongation protein 3, RNA polymerase II elongator complex subunit, HAG group protein, GCN5-related N-acetyltransferase, GNAT family protein |
| inparanoid | 151893 | DDB_G0275433 | DDB_G0275433 |  |
| inparanoid | 151895 | DDB_G0275385 | DDB_G0275385 | putative prolyl 4-hydroxylase alpha subunit |
| inparanoid | 151899 | DDB_G0275379 | DDB_G0275379 | unknown |
| inparanoid | 151903 | DDB_G0287851 | DDB_G0287851 | Q9JHR7 Insulin-degrading enzyme (EC 3.4.24.56) (Insulysin) (Insulinase) (Insulin protease). |
| inparanoid | 151904 | DDB_G0287645 | DDB_G0287645 | putative transmembrane protein, DUF1222 family protein |
| inparanoid | 151926 | DDB_G0293390 | DDB_G0293390 |  |
| inparanoid | 151977 | DDB_G0278955 | DDB_G0278955 | GLP_680_34352_35572. |
| inparanoid | 151996 | DDB_G0292922 | DDB_G0292922 |  |
| inparanoid | 151998 | DDB_G0292850 | phlp1 | phosducin-like protein |
| inparanoid | 152002 | DDB_G0275305 | DDB_G0275305 | unknown |
| inparanoid | 152036 | DDB_G0290671 | rabggtb | protein geranylgeranyltransferase type II, Rab geranylgeranyltransferase beta subunit |
| inparanoid | 152042 | DDB_G0290893 | DDB_G0290893 |  |
| inparanoid | 152043 | DDB_G0290857 | DDB_G0290857 |  |
| inparanoid | 152044 | DDB_G0289885 | sigF | unknown |
| inparanoid | 152047 | DDB_G0289953 | DDB_G0289953 |  |
| inparanoid | 152048 | DDB_G0289955 | DDB_G0289955 |  |
| inparanoid | 152069 | DDB_G0285599 | mcfB | EF-hand domain-containing protein, mitochondrial substrate carrier family protein, calcium-dependent mitochondrial substrate carrier |
| inparanoid | 152073 | DDB_G0285759 | mmetS | methionyl-tRNA synthetase, methionine-tRNA ligase, methionyl-tRNA synthetase, mitochondrial, methionine-tRNA ligase, mitochondrial |
| inparanoid | 152090 | DDB_G0270256 | DDB_G0270256 |  |
| inparanoid | 152099 | DDB_G0270252 | DDB_G0270252 |  |
| inparanoid | 152110 | DDB_G0295693 | DDB_G0295693 | unknown |
| inparanoid | 152111 | DDB_G0288519 | DDB_G0288519 | unknown |
| inparanoid | 152120 | DDB_G0270022 | DDB_G0270022 | cellular retinaldehyde-binding/triple function domain-containing protein |
| inparanoid | 152122 | DDB_G0271022 | adcC | SAM domain-containing protein, arrestin domain-containing protein, C2 calcium-dependent membrane targeting domain-containing protein |
| inparanoid | 152138 | DDB_G0274791 | commd5 | COMM domain-containing protein 5 |
| inparanoid | 152145 | DDB_G0274981 | DDB_G0274981 |  |
| inparanoid | 152146 | DDB_G0274829 | DDB_G0274829 | Similar to Arabidopsis thaliana (Mouse-ear cress). hypothetical 44.4 kDa protein. |
| inparanoid | 152148 | DDB_G0274713 | DDB_G0274713 | 1-aminocyclopropane-1-carboxylate synthase, S-adenosyl-L-methionine methylthioadenosine-lyase, ACC synthase |
| inparanoid | 152151 | DDB_G0274395 | adcB | SAM domain-containing protein, arrestin domain-containing protein, C2 calcium-dependent membrane targeting domain-containing protein |
| inparanoid | 152155 | DDB_G0274217 | tcf25 | Nulp1-type basic helix-loop-helix domain-containing protein |
| inparanoid | 152156 | DDB_G0274537 | DDB_G0274537 | EbiP7426 (Fragment). |
| inparanoid | 152163 | DDB_G0271928 | DDB_G0271928 |  |
| inparanoid | 152169 | DDB_G0271774 | uqcrh | ubiquinol-cytochrome-c reductase hinge protein, ubiquinol-cytochrome-c reductase subunit |
| inparanoid | 152171 | DDB_G0271728 | DDB_G0271728 |  |
| inparanoid | 152202 | DDB_G0282221 | DDB_G0282221 |  |
| inparanoid | 152205 | DDB_G0282287 | DDB_G0282287 |  |
| inparanoid | 152207 | DDB_G0277211 | DDB_G0277211 | DEAD/DEAH box helicase, superkiller viralicidic activity 2-like protein |
| inparanoid | 152220 | DDB_G0277767 | DDB_G0277767 | Similar to Dictyostelium discoideum (Slime mold). CIGB protein. |
| inparanoid | 152225 | DDB_G0277671 | rpc4 | putative RNA polymerase III subunit |
| inparanoid | 152229 | DDB_G0283001 | DDB_G0283001 |  |
| inparanoid | 152231 | DDB_G0283005 | agtA | WD40 repeat-containing protein, glycosyltransferase, alpha-galactosyltransferase, UDP-Gal:fucoside alpha3-galactosyltransferase |
| inparanoid | 152232 | DDB_G0282931 | abcB6 | ABC transporter B family protein |
| inparanoid | 152234 | DDB_G0282933 | mcm7 | MCM family protein, putative DNA replication licensing factor |
| inparanoid | 152246 | DDB_G0270992 | sec31 | WD40 repeat-containing protein, putative transport protein |
| inparanoid | 152248 | DDB_G0270228 | gtpbp3 | GTP-binding protein 3, putative tRNA modification GTPase 3 |
| inparanoid | 152261 | DDB_G0283281 | DDB_G0283281 | strictosidine synthase family protein |
| inparanoid | 152270 | DDB_G0281671 | DDB_G0281671 |  |
| inparanoid | 152271 | DDB_G0281785 | DDB_G0281785 |  |
| inparanoid | 152274 | DDB_G0278803 | dus1l | tRNA-dihydrouridine synthase 1-like protein |
| inparanoid | 152275 | DDB_G0278805 | DDB_G0278805 |  |
| inparanoid | 152282 | DDB_G0279237 | DDB_G0279237 |  |
| inparanoid | 152297 | DDB_G0276067 | ddcB | group IV decarboxylase, putative diaminopimelate decarboxylase |
| inparanoid | 152302 | DDB_G0277049 | DDB_G0277049 | mitochondrial matrix Mmp37 family protein |
| inparanoid | 152307 | DDB_G0275621 | DDB_G0275621 | Similar to Arabidopsis thaliana (Mouse-ear cress). hypothetical 59.5 kDa protein. |
| inparanoid | 152308 | DDB_G0275623 | mcm4 | MCM family protein, putative DNA replication licensing factor |
| inparanoid | 152315 | DDB_G0291922 | taz | tafazzin family protein, phospholipid/glycerol acyltransferase domain-containing protein |
| inparanoid | 152320 | DDB_G0291484 | DDB_G0291484 |  |
| inparanoid | 152321 | DDB_G0291662 | pop5 | RNase P protein subunit, RNase MRP protein subunit |
| inparanoid | 152330 | DDB_G0276133 | DDB_G0276133 |  |
| inparanoid | 152332 | DDB_G0276229 | clcC | chloride channel protein, CLC 6/7 family protein |
| inparanoid | 152333 | DDB_G0292192 | DDB_G0292192 |  |
| inparanoid | 152350 | DDB_G0277737 | DDB_G0277737 | Similar to Oryza sativa (Rice). hydroxyproline-rich glycoprotein. |
| inparanoid | 152353 | DDB_G0284921 | DDB_G0284921 |  |
| inparanoid | 152364 | DDB_G0276521 | DDB_G0276521 | Putative nuclease. |
| inparanoid | 152381 | DDB_G0288207 | DDB_G0288207 |  |
| inparanoid | 152382 | DDB_G0288267 | thrS1 | threonyl-tRNA synthetase, threonine-tRNA ligase |
| inparanoid | 152383 | DDB_G0288249 | mkpA | MAP kinase phosphatase, putative protein tyrosine phosphatase, dual specificity, gelsolin family protein |
| inparanoid | 152384 | DDB_G0287639 | DDB_G0287639 |  |
| inparanoid | 152394 | DDB_G0288029 | DDB_G0288029 | amino acid kinase |
| inparanoid | 152401 | DDB_G0279551 | DDB_G0279551 |  |
| inparanoid | 152412 | DDB_G0287025 | DDB_G0287025 |  |
| inparanoid | 152418 | DDB_G0267430 | abcG20 | ABC transporter G family protein |
| inparanoid | 152421 | DDB_G0286239 | DDB_G0286239 | alpha/beta hydrolase fold-1 domain-containing protein, serine hydrolase-like protein |
| inparanoid | 152429 | DDB_G0286219 | DDB_G0286219 | SNF2-related domain-containing protein, HEAT repeat-containing protein, armadillo-like helical domain-containing protein, CHR group protein, helicase, C-terminal domain-containing protein, putative TBP-associated factor |
| inparanoid | 152437 | DDB_G0280989 | hop2 | homologous-pairing protein 2 |
| inparanoid | 152453 | DDB_G0282275 | DDB_G0282275 |  |
| inparanoid | 152457 | DDB_G0282299 | DDB_G0282299 | BRCT domain-containing protein, DNA ligase |
| inparanoid | 152459 | DDB_G0282303 | DDB_G0282303 | ankyrin repeat-containing protein, RabGAP/TBC domain-containing protein, putative GTPase activating protein (GAP) |
| inparanoid | 152490 | DDB_G0281309 | DDB_G0281309 | Conserved hypothetical protein TIGR00730. |
| inparanoid | 152494 | DDB_G0281209 | DDB_G0281209 |  |
| inparanoid | 152517 | DDB_G0269294 | DDB_G0269294 |  |
| inparanoid | 152519 | DDB_G0270722 | DDB_G0270722 |  |
| inparanoid | 152527 | DDB_G0269326 | zntD | zinc/iron permease, zinc transporter |
| inparanoid | 152543 | DDB_G0271538 | DDB_G0271538 | LISK family protein kinase, protein kinase, TKL group, tyrosine kinase-like protein |
| inparanoid | 152553 | DDB_G0267954 | DDB_G0267954 | Y17G7B.18a protein. |
| inparanoid | 152560 | DDB_G0267546 | DDB_G0267546 |  |
| inparanoid | 152562 | DDB_G0267470 | dicB | discoidin-inducing complex (DIC) protein |
| inparanoid | 152566 | DDB_G0267502 | DDB_G0267502 |  |
| inparanoid | 152579 | DDB_G0287935 | DDB_G0287935 | DENN domain-containing protein |
| inparanoid | 152580 | DDB_G0295777 | DDB_G0295777 | UbiA prenyltransferase family protein |
| inparanoid | 152582 | DDB_G0294591 | DDB_G0294591 | putative transmembrane protein |
| inparanoid | 152608 | DDB_G0284737 | DDB_G0284737 | Putative 4-nitrophenylphosphatase (At5g47760). |
| inparanoid | 152610 | DDB_G0284707 | DDB_G0284707 |  |
| inparanoid | 152618 | DDB_G0285415 | nup85 | nucleoporin 85 |
| inparanoid | 152626 | DDB_G0283981 | pms1 | MutL DNA mismatch repair protein |
| inparanoid | 152631 | DDB_G0288327 | DDB_G0288327 |  |
| inparanoid | 152633 | DDB_G0288025 | hal | histidase, histidine ammonia-lyase |
| inparanoid | 152635 | DDB_G0288027 | DDB_G0288027 |  |
| inparanoid | 152639 | DDB_G0287823 | DDB_G0287823 |  |
| inparanoid | 152641 | DDB_G0288061 | p2xE | putative purinergic receptor, putative P2X receptor |
| inparanoid | 152647 | DDB_G0287449 | DDB_G0287449 |  |
| inparanoid | 152657 | DDB_G0288329 | DDB_G0288329 |  |
| inparanoid | 152720 | DDB_G0292146 | DDB_G0292146 |  |
| inparanoid | 152723 | DDB_G0280165 | DDB_G0280165 | vacuolar sorting protein 9 domain-containing protein |
| inparanoid | 152734 | DDB_G0269296 | DDB_G0269296 |  |
| inparanoid | 152735 | DDB_G0276475 | cfaC | counting factor associated protein |
| inparanoid | 152742 | DDB_G0268040 | DDB_G0268040 |  |
| inparanoid | 152760 | DDB_G0273031 | DDB_G0273031 | Similar to Oryza sativa (Rice). ESTs AU069374(C61044). |
| inparanoid | 152762 | DDB_G0272130 | DDB_G0272130 | recoverin family protein |
| inparanoid | 152770 | DDB_G0272000 | DDB_G0272000 | LMBR1 LONG FORM |
| inparanoid | 152772 | DDB_G0271904 | DDB_G0271904 | phosphoenolpyruvate carboxykinase (GTP), phosphoenolpyruvate carboxykinase |
| inparanoid | 152774 | DDB_G0271812 | DDB_G0271812 |  |
| inparanoid | 152778 | DDB_G0271788 | wdr57 | WD40 repeat-containing protein, putative U5 small nuclear ribonucleoprotein |
| inparanoid | 152793 | DDB_G0291338 | DDB_G0291338 |  |
| inparanoid | 152794 | DDB_G0291332 | DDB_G0291332 |  |
| inparanoid | 152806 | DDB_G0291830 | thg1 | tRNA-histidine guanylyltransferase 1 |
| inparanoid | 152810 | DDB_G0291952 | DDB_G0291952 | unknown |
| inparanoid | 152813 | DDB_G0280227 | DDB_G0280227 |  |
| inparanoid | 152820 | DDB_G0278229 | DDB_G0278229 | Q8K4G6 Protein LRP16 (Fragment). |
| inparanoid | 152826 | DDB_G0279515 | pigQ | putative glycosyltransferase, putative GlcNAc transferase, phosphatidylinositol glycan, class Q, phosphatidylinositol N-acetylglucosaminyltransferase subunit Q |
| inparanoid | 152838 | DDB_G0278827 | DDB_G0278827 | Putative RNA helicase of the SKI2 subfamily. |
| inparanoid | 152862 | DDB_G0270482 | gfm1 | mitochondrial translation elongation factor G |
| inparanoid | 152863 | DDB_G0269336 | DDB_G0269336 |  |
| inparanoid | 152871 | DDB_G0270882 | nosip | putative nitric oxide synthase-interacting protein |
| inparanoid | 152921 | DDB_G0270634 | DDB_G0270634 | RNA-binding region RNP-1 domain-containing protein, RNA recognition motif-containing protein RRM, putative splicing regulator |
| inparanoid | 152939 | DDB_G0268716 | lsm5 | LSM (like-Sm) domain-containing protein, putative U6 small nuclear ribonucleoparticle-associated protein |
| inparanoid | 152946 | DDB_G0290179 | ube3a | ubiquitin-protein ligase E3A |
| inparanoid | 152947 | DDB_G0290181 | DDB_G0290181 | HAT repeat-containing protein, RNA binding S1 domain-containing protein |
| inparanoid | 152956 | DDB_G0275693 | DDB_G0275693 | peptidase C53 family protein |
| inparanoid | 152980 | DDB_G0271098 | DDB_G0271098 |  |
| inparanoid | 152983 | DDB_G0290421 | DDB_G0290421 |  |
| inparanoid | 152991 | DDB_G0289985 | DDB_G0289985 | putative transmembrane protein |
| inparanoid | 153006 | DDB_G0281499 | DDB_G0281499 | putative actin binding protein, vinculin-related protein |
| inparanoid | 153008 | DDB_G0281441 | ercc6 | SNF2-related domain-containing protein, CHR group protein, helicase, C-terminal domain-containing protein, DNA excision repair protein 6 |
| inparanoid | 153022 | DDB_G0276269 | sgcA | guanylyl cyclase |
| inparanoid | 153024 | DDB_G0276283 | utp13 | WD40 repeat-containing protein, U3 snoRNP protein, U3 small nucleolar ribonucleoprotein |
| inparanoid | 153035 | DDB_G0285499 | DDB_G0285499 | TAR RNA loop binding protein (DJ792D7.1) (TAR (HIV) RNA-binding protein 1). |
| inparanoid | 153040 | DDB_G0285489 | DDB_G0285489 |  |
| inparanoid | 153053 | DDB_G0291912 | DDB_G0291912 | peptidase S10 family protein, serine carboxypeptidase, carboxypeptidase C |
| inparanoid | 153070 | DDB_G0279825 | DDB_G0279825 |  |
| inparanoid | 153071 | DDB_G0279827 | ivdA | isovaleryl-CoA dehydrogenase, mitochondrial |
| inparanoid | 153082 | DDB_G0281089 | prkab | putative glycoside hydrolase, AMP-activated protein kinase beta subunit, AMPK complex beta subunit |
| inparanoid | 153084 | DDB_G0280965 | gbqA | G-protein alpha subunit family protein, guanine nucleotide binding protein |
| inparanoid | 153086 | DDB_G0281081 | DDB_G0281081 | F1E22.1. |
| inparanoid | 153091 | DDB_G0290319 | DDB_G0290319 | bolA family protein |
| inparanoid | 153093 | DDB_G0280813 | DDB_G0280813 | phosphatidylinositol 3-kinase, FYVE-type zinc finger-containing protein |
| inparanoid | 153096 | DDB_G0280805 | DDB_G0280805 | P25586 Hypothetical 37.2 kDa protein in CHA1-PRD1 intergenic region. |
| inparanoid | 153099 | DDB_G0280105 | DDB_G0280105 | peptidase S10 family protein, serine carboxypeptidase, carboxypeptidase D |
| inparanoid | 153106 | DDB_G0291992 | DG1003 | unknown |
| inparanoid | 153109 | DDB_G0292168 | CYP516A1 | cytochrome P450 family protein |
| inparanoid | 15314 | DDB_G0293622 | DDB_G0293622 |  |
| inparanoid | 153140 | DDB_G0282235 | rfc5 | replication factor C subunit |
| inparanoid | 153145 | DDB_G0281641 | polD2 | DNA polymerase delta subunit 2, DNA polymerase subunit delta p50 |
| inparanoid | 153163 | DDB_G0280417 | DDB_G0280417 |  |
| inparanoid | 153164 | DDB_G0280415 | DDB_G0280415 |  |
| inparanoid | 153168 | DDB_G0280413 | DDB_G0280413 |  |
| inparanoid | 15317 | DDB_G0286705 | cog7 | oligomeric Golgi complex component |
| inparanoid | 153177 | DDB_G0275413 | DDB_G0275413 | transmembrane protein, vacuolar sorting protein 9 domain-containing protein, DUF726 family protein |
| inparanoid | 153190 | DDB_G0293534 | DDB_G0293534 | unknown |
| inparanoid | 153212 | DDB_G0279157 | med16 | putative mediator complex subunit 16 |
| inparanoid | 153214 | DDB_G0279169 | vps39 | tetratricopeptide-like helical domain-containing protein (TPR), citron-like domain-containing protein |
| inparanoid | 153226 | DDB_G0279447 | hspJ | heat shock protein Hsp20 domain-containing protein, putative alpha-crystallin-type heat shock protein |
| inparanoid | 153229 | DDB_G0267522 | DDB_G0267522 |  |
| inparanoid | 153230 | DDB_G0278459 | DDB_G0278459 |  |
| inparanoid | 153234 | DDB_G0278261 | DDB_G0278261 | sugar transporter family protein, putative myo-inositol cotransporter, solute carrier family 2 member protein |
| inparanoid | 153236 | DDB_G0279133 | syn7B | putative syntaxin 7, putative t-SNARE family protein |
| inparanoid | 153261 | DDB_G0283889 | DDB_G0283889 | unknown |
| inparanoid | 153264 | DDB_G0283883 | mlh3 | MutL DNA mismatch repair protein |
| inparanoid | 153265 | DDB_G0268092 | DDB_G0268092 |  |
| inparanoid | 153279 | DDB_G0276183 | DDB_G0276183 |  |
| inparanoid | 153282 | DDB_G0276241 | atp5e | ATP synthase epsilon chain, mitochondrial |
| inparanoid | 153287 | DDB_G0278609 | DDB_G0278609 |  |
| inparanoid | 153293 | DDB_G0276251 | DDB_G0276251 | transmembrane protein |
| inparanoid | 153294 | DDB_G0276029 | DG1040 | putative CCR4-NOT complex subunit 1 |
| inparanoid | 153304 | DDB_G0289655 | abcG7 | ABC transporter G family protein |
| inparanoid | 153313 | DDB_G0289737 | DDB_G0289737 |  |
| inparanoid | 153314 | DDB_G0289735 | DDB_G0289735 | RabGAP/TBC domain-containing protein, putative GTPase activating protein (GAP) |
| inparanoid | 153322 | DDB_G0292768 | dcd1B | acid ceramidase-like protein, acid N-acylsphingosine amidohydrolase-like protein |
| inparanoid | 153330 | DDB_G0268462 | DDB_G0268462 | putative transmembrane protein |
| inparanoid | 153349 | DDB_G0286637 | DDB_G0286637 | 3-methyl-2-oxobutanoate hydroxymethyltransferase, 2-dehydropantoate 2-reductase |
| inparanoid | 153350 | DDB_G0286809 | DDB_G0286809 |  |
| inparanoid | 153352 | DDB_G0286811 | DDB_G0286811 | putative transmembrane protein |
| inparanoid | 153358 | DDB_G0286645 | cstf3 | cleavage stimulation factor subunit 3, suppressor of forked (suf) domain-containing protein |
| inparanoid | 153362 | DDB_G0272758 | DDB_G0272758 | AAA ATPase domain-containing protein, replication factor C conserved region domain-containing protein |
| inparanoid | 153380 | DDB_G0272014 | DDB_G0272014 | Similar to Rattus norvegicus (Rat). kynurenine/alpha-aminoadipate aminotransferase (KAT/AADAT). |
| inparanoid | 153384 | DDB_G0290921 | DDB_G0290921 |  |
| inparanoid | 153420 | DDB_G0290695 | DDB_G0290695 |  |
| inparanoid | 153426 | DDB_G0284421 | med6 | putative mediator complex subunit 6 |
| inparanoid | 153427 | DDB_G0283865 | DDB_G0283865 |  |
| inparanoid | 153434 | DDB_G0284457 | DDB_G0284457 |  |
| inparanoid | 153440 | DDB_G0283845 | DDB_G0283845 |  |
| inparanoid | 153446 | DDB_G0274689 | DDB_G0274689 |  |
| inparanoid | 153460 | DDB_G0274249 | DDB_G0274249 |  |
| inparanoid | 153463 | DDB_G0274279 | tssc1 | WD40 repeat-containing protein |
| inparanoid | 153473 | DDB_G0271572 | exosc10 | 3'-5' exonuclease, exosome component 10 |
| inparanoid | 153478 | DDB_G0291600 | DDB_G0291600 |  |
| inparanoid | 153506 | DDB_G0288359 | dcd3A | alkaline dihydroceramidase, N-acylsphingosine amidohydrolase |
| inparanoid | 153508 | DDB_G0288047 | dnmA | DNA (cytosine-5-)-methyltransferase, DNA methyltransferase 2 |
| inparanoid | 153513 | DDB_G0287627 | timm17 | mitochondrial import inner membrane translocase subunit 17 |
| inparanoid | 153519 | DDB_G0287391 | ranbp1 | Ran binding protein 1 domain-containing protein |
| inparanoid | 153522 | DDB_G0287899 | DDB_G0287899 |  |
| inparanoid | 153527 | DDB_G0278197 | DDB_G0278197 |  |
| inparanoid | 153538 | DDB_G0277993 | DDB_G0277993 | putative importin subunit alpha C |
| inparanoid | 153539 | DDB_G0277997 | DDB_G0277997 |  |
| inparanoid | 153547 | DDB_G0272032 | gefG | Ras guanine nucleotide exchange factor |
| inparanoid | 153563 | DDB_G0272785 | DDB_G0272785 | ACYLOXYACYL HYDROLASE |
| inparanoid | 153568 | DDB_G0272839 | DDB_G0272839 | histidine triad (HIT) family protein |
| inparanoid | 153569 | DDB_G0272771 | wdr7 | WD40 repeat-containing protein |
| inparanoid | 153587 | DDB_G0282627 | vps15 | putative protein serine/threonine kinase, VPS15 family protein kinase |
| inparanoid | 153601 | DDB_G0295483 | DDB_G0295483 | RUN domain-containing protein |
| inparanoid | 153611 | DDB_G0292022 | ubr7 | ubiquitin protein ligase E3 component n-recognin 7 |
| inparanoid | 153613 | DDB_G0291251 | limD | LIM-type zinc finger-containing protein |
| inparanoid | 153614 | DDB_G0291267 | dst3 | putative protein serine/threonine kinase, STE20 family protein kinase, protein kinase, STE group |
| inparanoid | 153616 | DDB_G0291271 | eIF2b2 | translation initiation factor eIF-2B beta subunit |
| inparanoid | 153625 | DDB_G0276325 | DDB_G0276325 |  |
| inparanoid | 153626 | DDB_G0276273 | H2Bv2 | histone H2B domain-containing protein |
| inparanoid | 153632 | DDB_G0275649 | DDB_G0275649 |  |
| inparanoid | 153633 | DDB_G0275449 | natA | N-acetyltransferase, N-acetyltransferase complex subunit |
| inparanoid | 153660 | DDB_G0281177 | tmem208 | transmembrane protein, DUF788 family protein |
| inparanoid | 153661 | DDB_G0281085 | TFIIIA | C2H2-type zinc finger-containing protein, transcription initiation factor IIIA |
| inparanoid | 153664 | DDB_G0295713 | DDB_G0295713 | regulator of chromosome condensation (RCC1) domain-containing protein |
| inparanoid | 153668 | DDB_G0275399 | DDB_G0275399 |  |
| inparanoid | 153669 | DDB_G0280567 | ctps | CTP synthase |
| inparanoid | 153694 | DDB_G0293608 | wdr53 | WD40 repeat-containing protein |
| inparanoid | 153704 | DDB_G0293072 | DDB_G0293072 |  |
| inparanoid | 153705 | DDB_G0293074 | DDB_G0293074 |  |
| inparanoid | 153714 | DDB_G0268346 | DDB_G0268346 | patatin family protein |
| inparanoid | 153718 | DDB_G0284825 | DDB_G0284825 | RapGAP/RanGAP domain-containing protein, putative RanGAP |
| inparanoid | 153743 | DDB_G0273085 | DDB_G0273085 |  |
| inparanoid | 153778 | DDB_G0287251 | DDB_G0287251 |  |
| inparanoid | 153779 | DDB_G0270572 | pks2 | putative polyketide synthase, beta-ketoacyl synthase family protein |
| inparanoid | 153793 | DDB_G0289807 | DDB_G0289807 | Similar to hypothetical protein FLJ22405. |
| inparanoid | 153799 | DDB_G0276299 | agnA | argonaut-like protein |
| inparanoid | 153802 | DDB_G0275655 | malaS | alanyl-tRNA synthetase, alanine-tRNA ligase, putative mitochondrial alanyl-tRNA synthetase, putative mitochondrial alanine-tRNA ligase |
| inparanoid | 153817 | DDB_G0275455 | srp54 | signal recognition particle 54 kDa subunit |
| inparanoid | 153830 | DDB_G0292230 | DDB_G0292230 |  |
| inparanoid | 153831 | DDB_G0279943 | DDB_G0279943 |  |
| inparanoid | 153832 | DDB_G0276527 | DDB_G0276527 | putative protein serine/threonine kinase, regulator of chromosome condensation (RCC1) domain-containing protein, ubiquitin-protein ligase (HECT) domain-containing protein, protein kinase, CAMK group, CAMKL family protein kinase |
| inparanoid | 153835 | DDB_G0276453 | vilB | villin, protovillin |
| inparanoid | 153836 | DDB_G0276531 | DDB_G0276531 | Gb|AAF29402.1. |
| inparanoid | 153846 | DDB_G0288699 | tmem120 | transmembrane protein, TMPIT-like family protein, TMEM120 family protein |
| inparanoid | 153859 | DDB_G0287843 | DDB_G0287843 |  |
| inparanoid | 153860 | DDB_G0291824 | DDB_G0291824 |  |
| inparanoid | 153866 | DDB_G0283177 | DDB_G0283177 |  |
| inparanoid | 153869 | DDB_G0283171 | DDB_G0283171 |  |
| inparanoid | 153877 | DDB_G0291756 | gshB | glutathione synthase |
| inparanoid | 153883 | DDB_G0291450 | DDB_G0291450 |  |
| inparanoid | 153885 | DDB_G0291700 | DDB_G0291700 |  |
| inparanoid | 153888 | DDB_G0291109 | DDB_G0291109 |  |
| inparanoid | 153916 | DDB_G0288933 | cda | cytidine deaminase |
| inparanoid | 153918 | DDB_G0288935 | panC | pantoate-beta-alanine ligase |
| inparanoid | 153927 | DDB_G0289047 | DDB_G0289047 | AAA ATPase domain-containing protein, putative endopeptidase Clp |
| inparanoid | 153930 | DDB_G0289059 | DDB_G0289059 |  |
| inparanoid | 153934 | DDB_G0289075 | spiA | spore coat protein |
| inparanoid | 153938 | DDB_G0286387 | DDB_G0286387 | BTB/POZ domain-containing protein, meprin and TRAF homology (MATH) domain-containing protein |
| inparanoid | 153940 | DDB_G0286695 | DDB_G0286695 | pleckstrin homology (PH) domain-containing protein, Arf GTPase activating protein |
| inparanoid | 153945 | DDB_G0287163 | DDB_G0287163 |  |
| inparanoid | 153948 | DDB_G0285709 | DDB_G0285709 | heat shock protein Hsp70 family protein |
| inparanoid | 153960 | DDB_G0284357 | DDB_G0284357 | unknown |
| inparanoid | 153984 | DDB_G0293188 | DDB_G0293188 |  |
| inparanoid | 15402 | DDB_G0278613 | DDB_G0278613 | EGF-like domain-containing protein, matrilin-like protein |
| inparanoid | 154027 | DDB_G0292884 | DDB_G0292884 |  |
| inparanoid | 154030 | DDB_G0292974 | vti1A | v-SNARE family protein |
| inparanoid | 154037 | DDB_G0284013 | DDB_G0284013 |  |
| inparanoid | 154042 | DDB_G0284525 | DDB_G0284525 |  |
| inparanoid | 154051 | DDB_G0283893 | DDB_G0283893 | unknown |
| inparanoid | 154070 | DDB_G0289021 | DDB_G0289021 |  |
| inparanoid | 154090 | DDB_G0287197 | ncbp2 | RNA-binding region RNP-1 domain-containing protein, RNA recognition motif-containing protein RRM, nuclear cap-binding protein subunit 2 |
| inparanoid | 154097 | DDB_G0285657 | DDB_G0285657 |  |
| inparanoid | 154099 | DDB_G0287081 | DDB_G0287081 |  |
| inparanoid | 154113 | DDB_G0286391 | jcdC | transcription factor jumonji, jmjC domain-containing protein |
| inparanoid | 154129 | DDB_G0280477 | DDB_G0280477 | leucine-rich repeat-containing protein (LRR) |
| inparanoid | 154133 | DDB_G0280889 | DDB_G0280889 |  |
| inparanoid | 154135 | DDB_G0290313 | DDB_G0290313 |  |
| inparanoid | 154178 | DDB_G0269966 | ddx1 | putative RNA helicase, DEAD/DEAH box helicase, SPla/RYanodine receptor SPRY domain-containing protein |
| inparanoid | 154195 | DDB_G0269006 | DDB_G0269006 | C2H2-type zinc finger-containing protein, RNA-binding region RNP-1 domain-containing protein, RNA recognition motif-containing protein RRM, D111/G-patch domain-containing protein |
| inparanoid | 154197 | DDB_G0288979 | trmu | tRNA (5-methylaminomethyl-2-thiouridylate)-methyltransferase |
| inparanoid | 154198 | DDB_G0289311 | DDB_G0289311 |  |
| inparanoid | 154202 | DDB_G0277139 | apm2 | AP-2 medium chain, AP-2 ?2, clathrin-adaptor medium chain |
| inparanoid | 154223 | DDB_G0290621 | DDB_G0290621 | protein kinase, TKL group, tyrosine kinase-like protein |
| inparanoid | 154253 | DDB_G0278417 | xacB | pleckstrin homology (PH) domain-containing protein, RhoGEF domain-containing protein, RhoGAP domain-containing protein, IQ calmodulin-binding domain-containing protein |
| inparanoid | 154263 | DDB_G0280995 | mps1 | TTK family protein kinase, putative protein threonine/tyrosine kinase |
| inparanoid | 154266 | DDB_G0281045 | DDB_G0281045 | paired amphipathic helix (PAH) containing protein |
| inparanoid | 154284 | DDB_G0278267 | DDB_G0278267 | pleckstrin homology (PH) domain-containing protein, SH3 domain-containing protein, Arf GTPase activating protein |
| inparanoid | 154292 | DDB_G0285433 | phlp2 | phosducin-like protein |
| inparanoid | 154313 | DDB_G0270102 | mkcF | putative protein serine/threonine kinase, STE20 family protein kinase, SH3 domain-containing protein, protein kinase, STE group, MKC subfamily protein kinase |
| inparanoid | 154314 | DDB_G0269566 | DDB_G0269566 |  |
| inparanoid | 154328 | DDB_G0291730 | DDB_G0291730 |  |
| inparanoid | 154335 | DDB_G0291452 | DDB_G0291452 |  |
| inparanoid | 154339 | DDB_G0291636 | rpb5 | RNA polymerase II core subunit, RNA polymerase I core subunit, RNA polymerase III core subunit |
| inparanoid | 154348 | DDB_G0284509 | DDB_G0284509 |  |
| inparanoid | 154353 | DDB_G0284171 | DDB_G0284171 | chromo (CHRomatin Organization MOdifier) domain-containing protein, SNF2-related domain-containing protein, CHR group protein, helicase, C-terminal domain-containing protein |
| inparanoid | 154365 | DDB_G0284581 | DDB_G0284581 |  |
| inparanoid | 154370 | DDB_G0268686 | DDB_G0268686 |  |
| inparanoid | 154375 | DDB_G0269440 | DDB_G0269440 |  |
| inparanoid | 154383 | DDB_G0270396 | ddx49 | putative RNA helicase, DEAD/DEAH box helicase |
| inparanoid | 154398 | DDB_G0269898 | DDB_G0269898 |  |
| inparanoid | 154434 | DDB_G0290035 | DDB_G0290035 | Aminotransferase, putative. |
| inparanoid | 154444 | DDB_G0271342 | dgat1 | diacylglycerol O-acyltransferase 1 |
| inparanoid | 154453 | DDB_G0291318 | DDB_G0291318 | putative mitochondrial protein |
| inparanoid | 154455 | DDB_G0292040 | hemG | protoporphyrinogen oxidase |
| inparanoid | 154457 | DDB_G0292038 | DDB_G0292038 |  |
| inparanoid | 154460 | DDB_G0271708 | ddx31 | putative RNA helicase, DEAD/DEAH box helicase |
| inparanoid | 154464 | DDB_G0271536 | rcnA | modulatory calcineurin-interacting protein |
| inparanoid | 154468 | DDB_G0274803 | DDB_G0274803 | Similar to Dictyostelium discoideum (Slime mold). histidine kinase DhkG. |
| inparanoid | 154487 | DDB_G0274203 | DDB_G0274203 |  |
| inparanoid | 154491 | DDB_G0286545 | vps13D | vacuolar protein sorting-associated protein 13 family protein |
| inparanoid | 154497 | DDB_G0286287 | DDB_G0286287 |  |
| inparanoid | 154498 | DDB_G0286285 | DDB_G0286285 |  |
| inparanoid | 154506 | DDB_G0283413 | DDB_G0283413 |  |
| inparanoid | 154511 | DDB_G0283359 | eftud2 | U5 small nuclear ribonucleoprotein subunit, elongation factor Tu domain-containing protein, small GTP-binding protein domain-containing protein |
| inparanoid | 154513 | DDB_G0283415 | DDB_G0283415 |  |
| inparanoid | 154514 | DDB_G0283417 | DDB_G0283417 | putative protein tyrosine phosphatase, dual specificity |
| inparanoid | 154527 | DDB_G0292478 | DDB_G0292478 |  |
| inparanoid | 154531 | DDB_G0292678 | atp12 | ATP synthase mitochondrial F1 complex assembly factor 2 |
| inparanoid | 154532 | DDB_G0292680 | DDB_G0292680 | 4633402D15Rik protein. |
| inparanoid | 154535 | DDB_G0292862 | ppt3 | palmitoyl-protein thioesterase 3 |
| inparanoid | 154564 | DDB_G0277259 | DDB_G0277259 |  |
| inparanoid | 154579 | DDB_G0276369 | kif5 | kinesin-1, kinesin family member 5 |
| inparanoid | 154581 | DDB_G0276377 | anapc2 | anaphase promoting complex subunit 2 |
| inparanoid | 154583 | DDB_G0277307 | DDB_G0277307 | peptidase S16, Lon protease family protein |
| inparanoid | 154586 | DDB_G0276769 | DDB_G0276769 |  |
| inparanoid | 154588 | DDB_G0291440 | coq6 | monooxygenase |
| inparanoid | 154593 | DDB_G0291438 | nol6 | U3 snoRNP protein, U3 small nucleolar ribonucleoprotein, Nrap family protein |
| inparanoid | 154608 | DDB_G0278115 | DDB_G0278115 | putative transmembrane protein, DUF829 family protein |
| inparanoid | 154614 | DDB_G0295765 | mdn1 | type A von Willebrand factor (VWFA) domain-containing protein, putative AAA+ ATPase |
| inparanoid | 154620 | DDB_G0290341 | DDB_G0290341 |  |
| inparanoid | 154633 | DDB_G0271988 | DDB_G0271988 |  |
| inparanoid | 154640 | DDB_G0272208 | idhC | isocitrate dehydrogenase (NADP+), isocitrate dehydrogenase (NADP+), cytosolic |
| inparanoid | 154647 | DDB_G0287353 | fdxr | adrenodoxin reductase, NADPH:ferredoxin oxidoreductase |
| inparanoid | 154652 | DDB_G0287775 | DDB_G0287775 |  |
| inparanoid | 154656 | DDB_G0287805 | DDB_G0287805 |  |
| inparanoid | 154675 | DDB_G0273061 | cinD-1 | putative helix-turn-helix transcription factor |
| inparanoid | 154679 | DDB_G0272588 | dpm2-1 | dolichyl-phosphate mannosyltransferase 2 regulatory subunit |
| inparanoid | 154682 | DDB_G0271100 | DDB_G0271100 | O74918 Putative exosome complex exonuclease RRP45 (EC 3.1.13.-) (Ribosomal RNA processing protein 45). |
| inparanoid | 154697 | DDB_G0281909 | DDB_G0281909 |  |
| inparanoid | 154698 | DDB_G0282117 | DDB_G0282117 |  |
| inparanoid | 154704 | DDB_G0282757 | DDB_G0282757 |  |
| inparanoid | 154707 | DDB_G0282425 | DDB_G0282425 |  |
| inparanoid | 154713 | DDB_G0281895 | mrkC | putative protein serine/threonine kinase, protein kinase, CAMK group, CAMKL family protein kinase, MARK subfamily protein kinase |
| inparanoid | 154716 | DDB_G0274825 | DDB_G0274825 | putative cell surface glycoprotein |
| inparanoid | 154731 | DDB_G0287657 | tpsA | glycosyltransferase, alpha,alpha-trehalose-phosphate synthase, trehalose 6-phosphate synthase, trehalose-phosphatase |
| inparanoid | 154738 | DDB_G0288227 | DDB_G0288227 |  |
| inparanoid | 154741 | DDB_G0287555 | DDB_G0287555 | cysteine dioxygenase |
| inparanoid | 154775 | DDB_G0273463 | DDB_G0273463 | Similar to Dictyostelium discoideum (Slime mold). prespore-specific protein. |
| inparanoid | 154788 | DDB_G0295717 | DDB_G0295717 | RabGAP/TBC domain-containing protein, calcium-binding EF-hand domain-containing protein |
| inparanoid | 154791 | DDB_G0284697 | hemC | porphobilinogen deaminase, hydroxymethylbilane synthase |
| inparanoid | 154812 | DDB_G0285879 | DDB_G0285879 |  |
| inparanoid | 154821 | DDB_G0283663 | ppiD | tetratricopeptide-like helical domain-containing protein (TPR), cyclophilin-type peptidylprolyl cis-trans isomerase (PPIase), peptidylprolyl isomerase |
| inparanoid | 154831 | DDB_G0268472 | naprt | nicotinate phosphoribosyltransferase-like protein |
| inparanoid | 154840 | DDB_G0291396 | DDB_G0291396 | dilute domain-containing protein |
| inparanoid | 154842 | DDB_G0291394 | DDB_G0291394 |  |
| inparanoid | 154856 | DDB_G0288941 | DDB_G0288941 | unknown |
| inparanoid | 154861 | DDB_G0288909 | pus3 | tRNA pseudouridylate synthase |
| inparanoid | 154864 | DDB_G0289235 | DDB_G0289235 | Hypothetical glutamic acid-rich region containing protein. |
| inparanoid | 154873 | DDB_G0269666 | DDB_G0269666 |  |
| inparanoid | 154875 | DDB_G0269668 | DDB_G0269668 |  |
| inparanoid | 15488 | DDB_G0277531 | DDB_G0277531 | EGF-like domain-containing protein |
| inparanoid | 154923 | DDB_G0275111 | DDB_G0275111 |  |
| inparanoid | 154975 | DDB_G0286149 | dnpep | aspartyl aminopeptidase |
| inparanoid | 154980 | DDB_G0295787 | hbx11 | putative homeobox transcription factor, homeodomain (HOX) containing protein |
| inparanoid | 154986 | DDB_G0270090 | DDB_G0270090 |  |
| inparanoid | 154989 | DDB_G0270816 | DDB_G0270816 |  |
| inparanoid | 155003 | DDB_G0293614 | DDB_G0293614 | putative exoribonuclease |
| inparanoid | 155005 | DDB_G0293618 | pncA | nicotinamidase, pyrazinamidase |
| inparanoid | 155008 | DDB_G0289547 | DDB_G0289547 | RNA-binding region RNP-1 domain-containing protein, RNA recognition motif-containing protein RRM |
| inparanoid | 155018 | DDB_G0287991 | DDB_G0287991 |  |
| inparanoid | 155023 | DDB_G0287983 | redC | NADPH-dependent diflavin oxidoreductase 1 |
| inparanoid | 155024 | DDB_G0288685 | fam40 | FAM40 family protein |
| inparanoid | 155029 | DDB_G0288465 | DDB_G0288465 | putative pantetheine-phosphate adenylyltransferase |
| inparanoid | 155042 | DDB_G0283121 | DDB_G0283121 | methenyl tetrahydrofolate cyclohydrolase / NADP-dependent methylene H4F dehydrogenase |
| inparanoid | 155043 | DDB_G0285459 | DDB_G0285459 | putative protein tyrosine phosphatase, dual specificity |
| inparanoid | 155044 | DDB_G0282925 | lvsE | BEACH domain-containing protein |
| inparanoid | 155048 | DDB_G0283119 | xpot | armadillo-like helical domain-containing protein, exportin, tRNA |
| inparanoid | 155056 | DDB_G0278649 | DDB_G0278649 |  |
| inparanoid | 155059 | DDB_G0278281 | DDB_G0278281 |  |
| inparanoid | 155061 | DDB_G0278321 | TFIIIC3 | transcription factor IIIC-gamma subunit |
| inparanoid | 155062 | DDB_G0278315 | DDB_G0278315 | DUF647 family protein |
| inparanoid | 155076 | DDB_G0276781 | DDB_G0276781 |  |
| inparanoid | 155078 | DDB_G0277337 | DDB_G0277337 | beta-lactamase family protein |
| inparanoid | 155084 | DDB_G0277295 | mcysS | cysteinyl-tRNA synthetase, cysteine-tRNA ligase, putative mitochondrial cysteine-tRNA ligase, putative mitochondrial cysteinyl-tRNA synthetase |
| inparanoid | 155103 | DDB_G0283921 | ctsB | peptidase C1A family protein, papain family cysteine protease, cathepsin B precursor |
| inparanoid | 155106 | DDB_G0286131 | DDB_G0286131 |  |
| inparanoid | 155123 | DDB_G0276117 | DDB_G0276117 | HYPOTHETICAL 24.6 KDA PROTEIN C56G2.15 FROM CHROMOSOME III. 6/101 |
| inparanoid | 155125 | DDB_G0276233 | cct8 | chaperonin containing TCP1 theta subunit |
| inparanoid | 155132 | DDB_G0280221 | DDB_G0280221 | WD40 repeat-containing protein, putative guanine nucleotide exchange factor (GEF), DENN domain-containing protein, C2 calcium-dependent membrane targeting domain-containing protein |
| inparanoid | 155135 | DDB_G0292244 | rpb3 | RNA polymerase II core subunit |
| inparanoid | 155139 | DDB_G0284105 | DDB_G0284105 | C2H2-type zinc finger-containing protein |
| inparanoid | 155143 | DDB_G0284155 | DDB_G0284155 | phospholipase D |
| inparanoid | 155147 | DDB_G0284555 | sf3b2 | PSP proline-rich domain-containing protein, splicing factor 3B subunit 2 |
| inparanoid | 155149 | DDB_G0283781 | DDB_G0283781 |  |
| inparanoid | 155156 | DDB_G0282055 | DDB_G0282055 | C2 domain-containing protein, Ras GTPase activation domain-containing protein |
| inparanoid | 155157 | DDB_G0281771 | DDB_G0281771 |  |
| inparanoid | 155163 | DDB_G0282485 | DDB_G0282485 |  |
| inparanoid | 155167 | DDB_G0282261 | ech1 | enoyl Coenzyme A hydratase, Delta(3,5)-Delta(2,4)-dienoyl-CoA isomerase |
| inparanoid | 155178 | DDB_G0287695 | DDB_G0287695 | FAD-binding monooxygenase |
| inparanoid | 155181 | DDB_G0287675 | DDB_G0287675 |  |
| inparanoid | 155188 | DDB_G0287233 | spoT | guanosine-3',5'-bis(diphosphate) 3'-diphosphatase, guanosine polyphosphate pyrophosphohydrolase |
| inparanoid | 155205 | DDB_G0279033 | DDB_G0279033 |  |
| inparanoid | 155211 | DDB_G0280471 | DDB_G0280471 |  |
| inparanoid | 155221 | DDB_G0284227 | DDB_G0284227 |  |
| inparanoid | 155232 | DDB_G0290599 | DDB_G0290599 |  |
| inparanoid | 155255 | DDB_G0287157 | DDB_G0287157 | DUF1683 family protein |
| inparanoid | 155256 | DDB_G0287159 | cysS | cysteinyl-tRNA synthetase, cysteine-tRNA ligase |
| inparanoid | 155275 | DDB_G0286613 | DDB_G0286613 | type A von Willebrand factor (VWFA) domain-containing protein, poly(ADP-ribose) polymerase, catalytic region domain-containing protein, BRCT domain-containing protein, 14-3-3 family protein, vault protein inter-alpha-trypsin (VIT) domain-containing protein |
| inparanoid | 155279 | DDB_G0286455 | DDB_G0286455 |  |
| inparanoid | 155281 | DDB_G0275345 | DDB_G0275345 |  |
| inparanoid | 155283 | DDB_G0276961 | DDB_G0276961 | RabGAP/TBC domain-containing protein, putative GTPase activating protein (GAP) |
| inparanoid | 155299 | DDB_G0277075 | DDB_G0277075 | Similar to Mus musculus (Mouse). sex-determining region Y protein (Testis-determining factor). |
| inparanoid | 155301 | DDB_G0271502 | lvsD | BEACH domain-containing protein |
| inparanoid | 155307 | DDB_G0272746 | DDB_G0272746 |  |
| inparanoid | 155311 | DDB_G0272740 | DDB_G0272740 | putative histone-like transcription factor |
| inparanoid | 155324 | DDB_G0275925 | wdr68 | WD40 repeat-containing protein |
| inparanoid | 155334 | DDB_G0295739 | DDB_G0295739 | unknown |
| inparanoid | 155335 | DDB_G0269694 | pan2 | WD40-like domain-containing protein, peptidase C19 family protein, putative ubiquitin carboxyl-terminal hydrolase (UCH), putative exonuclease, polyA specific ribonuclease subunit |
| inparanoid | 155341 | DDB_G0269362 | frmB | Band 4.1 domain-containing protein, FERM domain-containing protein |
| inparanoid | 155352 | DDB_G0289455 | DDB_G0289455 |  |
| inparanoid | 155362 | DDB_G0278355 | DDB_G0278355 |  |
| inparanoid | 155369 | DDB_G0288625 | DDB_G0288625 |  |
| inparanoid | 155370 | DDB_G0288627 | DDB_G0288627 |  |
| inparanoid | 155371 | DDB_G0288643 | DDB_G0288643 |  |
| inparanoid | 155376 | DDB_G0270640 | DDB_G0270640 |  |
| inparanoid | 155396 | DDB_G0270006 | jcdI | transcription factor jumonji, jmjC domain-containing protein |
| inparanoid | 155404 | DDB_G0270776 | DDB_G0270776 | DUF255 family protein |
| inparanoid | 155426 | DDB_G0270356 | erg2 | C-8 sterol isomerase |
| inparanoid | 155432 | DDB_G0281361 | DDB_G0281361 |  |
| inparanoid | 155439 | DDB_G0290147 | DDB_G0290147 |  |
| inparanoid | 155440 | DDB_G0290145 | DDB_G0290145 |  |
| inparanoid | 155470 | DDB_G0280481 | DDB_G0280481 | unknown |
| inparanoid | 155471 | DDB_G0280483 | fsjA | rRNA methyltransferase, rRNA (uridine-2'-O-)-methyltransferase |
| inparanoid | 155477 | DDB_G0278283 | fol1 | dihydro-6-hydroxymethylpterin pyrophosphokinase (HPPK), dihydropteroate synthetase (DHPS), dihydroneopterin aldolase (DHNA) domain-containing protein |
| inparanoid | 155533 | DDB_G0289423 | DDB_G0289423 | SPX domain-containing protein, EXS domain-containing protein, SPX/EXS domain-containing protein 4 |
| inparanoid | 155536 | DDB_G0289395 | crlB | cAMP receptor-like protein, G-protein-coupled receptor (GPCR) family protein |
| inparanoid | 155553 | DDB_G0288551 | DDB_G0288551 | Pumilio RNA-binding region-containing protein, PUF domain-containing protein |
| inparanoid | 155555 | DDB_G0287561 | DDB_G0287561 | NADH:flavin oxidoreductase/NADH oxidase domain-containing protein |
| inparanoid | 155557 | DDB_G0288545 | nup210 | nucleoporin 210 |
| inparanoid | 155563 | DDB_G0286623 | mcm2 | MCM family protein, putative DNA replication licensing factor |
| inparanoid | 155594 | DDB_G0279647 | DDB_G0279647 |  |
| inparanoid | 155599 | DDB_G0292832 | DDB_G0292832 |  |
| inparanoid | 155606 | DDB_G0268886 | vps13E | vacuolar protein sorting-associated protein 13 family protein |
| inparanoid | 155619 | DDB_G0269738 | desA | sphingolipid delta 4 desaturase, dihydroceramide desaturase |
| inparanoid | 155622 | DDB_G0269734 | DDB_G0269734 |  |
| inparanoid | 155625 | DDB_G0268500 | DDB_G0268500 | unknown |
| inparanoid | 155627 | DDB_G0291299 | DDB_G0291299 | O-sialoglycoprotein endopeptidase. |
| inparanoid | 155634 | DDB_G0286759 | DDB_G0286759 | unknown |
| inparanoid | 155636 | DDB_G0286857 | glod5 | glyoxalase domain-containing protein 5 |
| inparanoid | 155644 | DDB_G0286189 | sumo | small ubiquitin-like protein |
| inparanoid | 155651 | DDB_G0290237 | acbd6 | ankyrin repeat-containing protein, acyl-CoA binding protein, acyl-CoA-binding domain-containing protein 6 |
| inparanoid | 155654 | DDB_G0290233 | mgp1 | RhoGAP domain-containing protein, Cdc15/Fes/CIP4 domain-containing protein, PCH family protein |
| inparanoid | 155657 | DDB_G0275171 | DDB_G0275171 |  |
| inparanoid | 155718 | DDB_G0285601 | cdc20 | WD40 repeat-containing protein, cdc20/fizzy domain-containing protein |
| inparanoid | 155722 | DDB_G0285967 | DDB_G0285967 | LIM-type zinc finger-containing protein, phosphatase tensin type domain-containing protein, C2 tensin-type domain-containing protein |
| inparanoid | 155733 | DDB_G0268558 | DDB_G0268558 |  |
| inparanoid | 155735 | DDB_G0268168 | DDB_G0268168 |  |
| inparanoid | 155738 | DDB_G0268118 | DDB_G0268118 | Erwinia chrysanthemi IndA protein homolog-like. |
| inparanoid | 155757 | DDB_G0279049 | DDB_G0279049 |  |
| inparanoid | 155777 | DDB_G0284857 | DDB_G0284857 |  |
| inparanoid | 155787 | DDB_G0289613 | gefAA | leucine-rich repeat-containing protein (LRR), Ras guanine nucleotide exchange factor |
| inparanoid | 155793 | DDB_G0289829 | DDB_G0289829 |  |
| inparanoid | 155800 | DDB_G0275687 | abcG18 | ABC transporter G family protein |
| inparanoid | 155801 | DDB_G0275651 | elp2 | WD-40 repeat-containing protein, elongation protein 2, RNA polymerase II elongator complex subunit |
| inparanoid | 155803 | DDB_G0275545 | DDB_G0275545 |  |
| inparanoid | 155806 | DDB_G0275739 | DDB_G0275739 |  |
| inparanoid | 155807 | DDB_G0275863 | nmt | glycylpeptide N-tetradecanoyltransferase, peptide N-myristoyltransferase |
| inparanoid | 155809 | DDB_G0291308 | DDB_G0291308 |  |
| inparanoid | 155823 | DDB_G0289593 | tufM | elongation factor Tu domain-containing protein, small GTP-binding protein domain-containing protein |
| inparanoid | 155824 | DDB_G0275865 | DDB_G0275865 |  |
| inparanoid | 155867 | DDB_G0269150 | lvsA | BEACH domain-containing protein |
| inparanoid | 155868 | DDB_G0269608 | DDB_G0269608 |  |
| inparanoid | 155909 | DDB_G0293192 | DG1098 | armadillo-like helical domain-containing protein |
| inparanoid | 155911 | DDB_G0293258 | DDB_G0293258 | myotubularin-related protein |
| inparanoid | 155915 | DDB_G0292992 | helA | putative RNA helicase |
| inparanoid | 155933 | DDB_G0293520 | exoc3 | exocyst complex subunit 3 |
| inparanoid | 155951 | DDB_G0267850 | DDB_G0267850 | ribosomal protein L1 family protein |
| inparanoid | 155952 | DDB_G0267848 | DDB_G0267848 | beta-lactamase-type transpeptidase fold containing protein |
| inparanoid | 155977 | DDB_G0290523 | DDB_G0290523 | putative transmembrane protein |
| inparanoid | 155993 | DDB_G0291097 | vps33 | Sec1-like family protein |
| inparanoid | 155996 | DDB_G0291103 | DDB_G0291103 |  |
| inparanoid | 155997 | DDB_G0291099 | DDB_G0291099 |  |
| inparanoid | 155998 | DDB_G0284043 | sapA | saposin A |
| inparanoid | 155999 | DDB_G0291181 | DDB_G0291181 |  |
| inparanoid | 156005 | DDB_G0291175 | DDB_G0291175 |  |
| inparanoid | 156014 | DDB_G0274567 | mtrpS | tryptophanyl-tRNA synthetase, tryptophan-tRNA ligase, putative mitochondrial tryptophanyl-tRNA synthetase, putative mitochondrial tryptophan-tRNA ligase |
| inparanoid | 156015 | DDB_G0274303 | DDB_G0274303 |  |
| inparanoid | 156016 | DDB_G0274305 | DDB_G0274305 | unknown |
| inparanoid | 156018 | DDB_G0274553 | vatH | vacuolar ATP synthase subunit H |
| inparanoid | 156019 | DDB_G0274555 | prp18 | putative RNA splicing factor |
| inparanoid | 156020 | DDB_G0267438 | abcA2 | ABC transporter A family protein |
| inparanoid | 156027 | DDB_G0283597 | eIF3s9 | RNA-binding region RNP-1 domain-containing protein, RNA recognition motif-containing protein RRM, eIF-3 eta, eukaryotic translation initiation factor 3 (eIF3) subunit 9 |
| inparanoid | 156047 | DDB_G0289211 | DDB_G0289211 |  |
| inparanoid | 156071 | DDB_G0286469 | DDB_G0286469 |  |
| inparanoid | 156072 | DDB_G0286421 | DDB_G0286421 |  |
| inparanoid | 156077 | DDB_G0286199 | DDB_G0286199 | Q92QZ1 Dimethyladenosine transferase (EC 2.1.1.-) (S-adenosylmethionine-6-N', N'-adenosyl(rRNA) dimethyltransferase) (16S rRNA dimethylase) (High level kasugamycin resistance protein ksgA) (Kasugamycin dimethyltransferase). |
| inparanoid | 156078 | DDB_G0286409 | DDB_G0286409 |  |
| inparanoid | 156079 | DDB_G0286413 | DDB_G0286413 |  |
| inparanoid | 156097 | DDB_G0279649 | DDB_G0279649 | ankyrin repeat-containing protein, pleckstrin homology (PH) domain-containing protein, Arf GTPase activating protein, BAR domain-containing protein |
| inparanoid | 156100 | DDB_G0279653 | DDB_G0279653 |  |
| inparanoid | 156107 | DDB_G0270128 | DDB_G0270128 |  |
| inparanoid | 156147 | DDB_G0288651 | DDB_G0288651 | patatin family protein |
| inparanoid | 156159 | DDB_G0268216 | DDB_G0268216 |  |
| inparanoid | 156163 | DDB_G0268616 | mtaA | centrosomal protein CP224, XMAP215 family protein |
| inparanoid | 156172 | DDB_G0270764 | MYH | mutY homolog, A/G-specific adenine DNA glycosylase, monofunctional DNA glycosylase |
| inparanoid | 156179 | DDB_G0293560 | rpa2 | RNA polymerase I, second largest subunit |
| inparanoid | 156198 | DDB_G0284973 | DDB_G0284973 |  |
| inparanoid | 156200 | DDB_G0268030 | DDB_G0268030 |  |
| inparanoid | 156207 | DDB_G0268516 | tbcD | tubulin folding cofactor D |
| inparanoid | 156209 | DDB_G0275985 | mcfH | mitochondrial substrate carrier family protein |
| inparanoid | 156210 | DDB_G0276069 | DDB_G0276069 | unknown |
| inparanoid | 156220 | DDB_G0286523 | DDB_G0286523 |  |
| inparanoid | 156221 | DDB_G0279939 | DDB_G0279939 |  |
| inparanoid | 156222 | DDB_G0279927 | Dd5P1 | inositol 5-phosphatase |
| inparanoid | 156249 | DDB_G0276125 | DDB_G0276125 |  |
| inparanoid | 156256 | DDB_G0277219 | vps13l | unknown |
| inparanoid | 156262 | DDB_G0277187 | DDB_G0277187 |  |
| inparanoid | 156265 | DDB_G0268054 | DDB_G0268054 |  |
| inparanoid | 156269 | DDB_G0268056 | sptA | serine C-palmitoyltransferase subunit |
| inparanoid | 156270 | DDB_G0268424 | DDB_G0268424 |  |
| inparanoid | 156272 | DDB_G0267974 | DDB_G0267974 |  |
| inparanoid | 156274 | DDB_G0267666 | gefW | RasGEF domain-containing protein |
| inparanoid | 156275 | DDB_G0267648 | DDB_G0267648 |  |
| inparanoid | 156281 | DDB_G0267586 | DDB_G0267586 |  |
| inparanoid | 156282 | DDB_G0267572 | DDB_G0267572 |  |
| inparanoid | 156290 | DDB_G0284805 | DDB_G0284805 |  |
| inparanoid | 156293 | DDB_G0271814 | DDB_G0271814 |  |
| inparanoid | 156298 | DDB_G0272576 | DDB_G0272576 |  |
| inparanoid | 156300 | DDB_G0269712 | ahsa | activator of Hsp90 ATPase family protein |
| inparanoid | 156301 | DDB_G0270622 | DDB_G0270622 |  |
| inparanoid | 156303 | DDB_G0269192 | tifA | DEAD/DEAH box helicase domain-containing protein, eukaryotic translation initiation factor 4A |
| inparanoid | 156321 | DDB_G0269216 | cypE | cyclophilin E, cyclophilin-type peptidylprolyl cis-trans isomerase (PPIase) |
| inparanoid | 156331 | DDB_G0293578 | DDB_G0293578 |  |
| inparanoid | 156335 | DDB_G0273095 | DDB_G0273095 |  |
| inparanoid | 156336 | DDB_G0273093 | DDB_G0273093 | heat shock protein 70 (Hsp70) family member |
| inparanoid | 156341 | DDB_G0273339 | DDB_G0273339 |  |
| inparanoid | 156342 | DDB_G0273037 | DDB_G0273037 | Similar to plasmodium falciparum (Isolate 3D7). Metacaspase-like protein. |
| inparanoid | 156344 | DDB_G0273331 | DDB_G0273331 |  |
| inparanoid | 156375 | DDB_G0293768 | fut9 | glycosyltransferase, alpha-3/4-fucosyltransferase |
| inparanoid | 156380 | DDB_G0293914 | med15 | putative mediator complex subunit 15 |
| inparanoid | 156383 | DDB_G0277495 | DDB_G0277495 | unknown |
| inparanoid | 156386 | DDB_G0277403 | clc | clathrin light chain |
| inparanoid | 156389 | DDB_G0276947 | trap1 | heat shock protein Hsp90 family protein |
| inparanoid | 156394 | DDB_G0277441 | rabJ | Rab GTPase |
| inparanoid | 156398 | DDB_G0291195 | DDB_G0291195 | putative methyltransferase |
| inparanoid | 156403 | DDB_G0291157 | cln3 | Batten's disease protein Cln3 family protein |
| inparanoid | 156413 | DDB_G0291155 | DDB_G0291155 | RNA-binding region RNP-1 domain-containing protein, RNA recognition motif-containing protein RRM |
| inparanoid | 156422 | DDB_G0289465 | DDB_G0289465 |  |
| inparanoid | 156432 | DDB_G0285229 | DDB_G0285229 |  |
| inparanoid | 156442 | DDB_G0269788 | DDB_G0269788 | Similar to sterol-C4-methyl oxidase-like. |
| inparanoid | 156466 | DDB_G0277405 | DDB_G0277405 | SAP DNA-binding domain-containing protein |
| inparanoid | 156475 | DDB_G0281953 | DG1060 | putative tyrosine phosphatase family protein |
| inparanoid | 156481 | DDB_G0282127 | DDB_G0282127 |  |
| inparanoid | 156482 | DDB_G0282125 | DDB_G0282125 |  |
| inparanoid | 156483 | DDB_G0272760 | mcm6 | MCM family protein, putative DNA replication licensing factor |
| inparanoid | 156484 | DDB_G0292402 | DDB_G0292402 | Putative oxalyl-CoA decarboxylase. |
| inparanoid | 156495 | DDB_G0287279 | DDB_G0287279 |  |
| inparanoid | 156510 | DDB_G0280527 | DDB_G0280527 | RapGAP/RanGAP domain-containing protein, armadillo repeat-containing protein, tuberin family protein |
| inparanoid | 156521 | DDB_G0280239 | DDB_G0280239 | unknown |
| inparanoid | 156527 | DDB_G0283377 | DDB_G0283377 |  |
| inparanoid | 156528 | DDB_G0283363 | vti1B | putative v-SNARE family protein |
| inparanoid | 156538 | DDB_G0281719 | DDB_G0281719 |  |
| inparanoid | 156561 | DDB_G0271334 | DDB_G0271334 | C2 calcium/lipid-binding (CaLB) region-containing protein, dilute domain-containing protein |
| inparanoid | 156564 | DDB_G0271096 | atg7 | autophagy protein 7, E1-like enzyme family protein |
| inparanoid | 156596 | DDB_G0274449 | scsB | succinate-CoA ligase (GDP-forming), succinyl-CoA synthetase (GDP-forming), GTP-specific succinyl-CoA synthetase beta subunit |
| inparanoid | 156598 | DDB_G0274123 | pyd3 | beta-alanine synthase |
| inparanoid | 156599 | DDB_G0274641 | DDB_G0274641 |  |
| inparanoid | 15662 | DDB_G0270024 | mgp2 | RhoGAP domain-containing protein, Cdc15/Fes/CIP4 domain-containing protein, PCH family protein |
| inparanoid | 156632 | DDB_G0293248 | grpA | golgi reassembly stacking protein |
| inparanoid | 156647 | DDB_G0284535 | CYP508A4 | cytochrome P450 family protein |
| inparanoid | 156651 | DDB_G0278121 | DDB_G0278121 |  |
| inparanoid | 156662 | DDB_G0276047 | DDB_G0276047 |  |
| inparanoid | 156697 | DDB_G0277675 | DDB_G0277675 | RasGTPase-activating protein |
| inparanoid | 156705 | DDB_G0285079 | DDB_G0285079 |  |
| inparanoid | 156706 | DDB_G0285009 | hemD | putative uroporphyrinogen III synthase |
| inparanoid | 156709 | DDB_G0284961 | DDB_G0284961 |  |
| inparanoid | 156716 | DDB_G0282765 | DDB_G0282765 | short-chain dehydrogenase/reductase (SDR) family protein |
| inparanoid | 156755 | DDB_G0269778 | DDB_G0269778 | cell differentiation family, Rcd1-like protein |
| inparanoid | 156758 | DDB_G0268774 | DDB_G0268774 |  |
| inparanoid | 156759 | DDB_G0268776 | gatB | glutamyl-tRNA(Gln) amidotransferase B subunit |
| inparanoid | 156766 | DDB_G0269760 | DDB_G0269760 | BTB/POZ domain-containing protein, potassium channel tetramerization domain-containing protein |
| inparanoid | 156771 | DDB_G0284247 | DDB_G0284247 | CG8351 protein (LD47396p). |
| inparanoid | 156776 | DDB_G0282101 | DDB_G0282101 |  |
| inparanoid | 156777 | DDB_G0282097 | cxfA | cytochrome c oxidase subunit VI |
| inparanoid | 156778 | DDB_G0282099 | rio2 | putative protein serine/threonine kinase, protein kinase, Atypical group, RIO family protein kinase, RIO2 subfamily protein kinase |
| inparanoid | 156809 | DDB_G0284601 | csnk2b | putative casein kinase II beta chain (CK2), putative casein kinase II regulatory subunit |
| inparanoid | 156811 | DDB_G0285113 | DDB_G0285113 |  |
| inparanoid | 156819 | DDB_G0284349 | gdap2 | ganglioside induced differentiation associated protein 2 |
| inparanoid | 156844 | DDB_G0271370 | DDB_G0271370 |  |
| inparanoid | 156854 | DDB_G0269824 | DDB_G0269824 |  |
| inparanoid | 156861 | DDB_G0275089 | DDB_G0275089 |  |
| inparanoid | 156864 | DDB_G0275301 | DDB_G0275301 | unknown |
| inparanoid | 156868 | DDB_G0280079 | DDB_G0280079 | myb domain-containing protein, SWIRM domain-containing protein, ZZ-type zinc finger-containing protein |
| inparanoid | 156870 | DDB_G0274233 | ctbsA | glycoside hydrolase family 18 protein, putative di-N-acetylchitobiase |
| inparanoid | 156871 | DDB_G0276435 | DDB_G0276435 |  |
| inparanoid | 156873 | DDB_G0277895 | DDB_G0277895 | TM2 domain containing protein |
| inparanoid | 156874 | DDB_G0275793 | DDB_G0275793 | Similar to hypothetical ORF; Yml018cp. |
| inparanoid | 156889 | DDB_G0284241 | DDB_G0284241 | FLJ00071 protein (Fragment). |
| inparanoid | 156893 | DDB_G0288441 | DDB_G0288441 |  |
| inparanoid | 156894 | DDB_G0288475 | DDB_G0288475 | unknown |
| inparanoid | 156902 | DDB_G0293444 | DDB_G0293444 |  |
| inparanoid | 156942 | DDB_G0280777 | DDB_G0280777 | bromodomain-containing protein, BRD group protein |
| inparanoid | 156945 | DDB_G0278339 | DDB_G0278339 | CG6700 protein. |
| inparanoid | 156946 | DDB_G0278337 | DDB_G0278337 | unknown |
| inparanoid | 156959 | DDB_G0278329 | DDB_G0278329 |  |
| inparanoid | 156960 | DDB_G0278327 | DDB_G0278327 |  |
| inparanoid | 156983 | DDB_G0286279 | DDB_G0286279 |  |
| inparanoid | 156986 | DDB_G0279277 | DDB_G0279277 |  |
| inparanoid | 156988 | DDB_G0278097 | DDB_G0278097 |  |
| inparanoid | 157001 | DDB_G0284941 | DDB_G0284941 | thioredoxin domain-containing protein |
| inparanoid | 157009 | DDB_G0273421 | DDB_G0273421 |  |
| inparanoid | 157024 | DDB_G0272432 | DDB_G0272432 |  |
| inparanoid | 157031 | DDB_G0288807 | ppt2 | palmitoyl-protein thioesterase 2 |
| inparanoid | 157047 | DDB_G0285279 | DDB_G0285279 |  |
| inparanoid | 157052 | DDB_G0285997 | abcF1 | ABC transporter-related protein |
| inparanoid | 157066 | DDB_G0284643 | DDB_G0284643 |  |
| inparanoid | 157068 | DDB_G0294563 | DDB_G0294563 | EGF-like domain-containing protein, kazal-type protease inhibitor domain-containing protein |
| inparanoid | 157074 | DDB_G0284341 | lyrm5 |  |
| inparanoid | 157080 | DDB_G0283287 | fut7 | glycosyltransferase, alpha-3/4-fucosyltransferase |
| inparanoid | 157090 | DDB_G0290645 | DDB_G0290645 |  |
| inparanoid | 157099 | DDB_G0275845 | smg1 | protein kinase, Atypical group, phosphatidylinositol 3-kinase-related protein kinase, PIKK family protein kinase, SMG1 subfamily protein kinase |
| inparanoid | 157120 | DDB_G0288595 | DDB_G0288595 |  |
| inparanoid | 157122 | DDB_G0288599 | DDB_G0288599 | membrane bound O-acyl transferase family protein |
| inparanoid | 157146 | DDB_G0284583 | glyS | glycyl-tRNA synthetase, glycine-tRNA ligase |
| inparanoid | 157150 | DDB_G0282063 | DDB_G0282063 |  |
| inparanoid | 157172 | DDB_G0290217 | glb1 | glycoside hydrolase family 35 protein, beta-galactosidase |
| inparanoid | 157173 | DDB_G0290221 | DDB_G0290221 |  |
| inparanoid | 157178 | DDB_G0280767 | DDB_G0280767 | P44765 Aminobenzoyl-glutamate utilization protein A homolog. |
| inparanoid | 157185 | DDB_G0282341 | DDB_G0282341 |  |
| inparanoid | 157188 | DDB_G0281567 | fhkC | protein kinase 1, putative protein serine/threonine kinase, protein kinase, CAMK group, RAD53 family protein kinase, FHA domain-containing protein |
| inparanoid | 157189 | DDB_G0281713 | DDB_G0281713 |  |
| inparanoid | 157190 | DDB_G0281711 | ddx27 | putative RNA helicase, DEAD/DEAH box helicase |
| inparanoid | 157191 | DDB_G0281709 | DDB_G0281709 |  |
| inparanoid | 157194 | DDB_G0295825 | mrpl34 | ribosomal protein L34, mitochondrial |
| inparanoid | 157197 | DDB_G0282633 | DDB_G0282633 | ankyrin repeat-containing protein, regulator of chromosome condensation (RCC1) domain-containing protein, BTB/POZ domain-containing protein, putative guanine nucleotide exchange factor (GEF) |
| inparanoid | 157215 | DDB_G0279755 | DDB_G0279755 | OB fold-containing protein, nucleic acid binding |
| inparanoid | 157220 | DDB_G0279749 | DDB_G0279749 |  |
| inparanoid | 157221 | DDB_G0279747 | DDB_G0279747 |  |
| inparanoid | 157251 | DDB_G0289445 | prmt2 | protein arginine methyltransferase |
| inparanoid | 157255 | DDB_G0281227 | DDB_G0281227 |  |
| inparanoid | 157280 | DDB_G0287259 | cdh1 | WD40 repeat-containing protein, cdc20/fizzy domain-containing protein |
| inparanoid | 157290 | DDB_G0282549 | DDB_G0282549 |  |
| inparanoid | 157299 | DDB_G0291464 | DDB_G0291464 |  |
| inparanoid | 157306 | DDB_G0268020 | bkdB | 3-methyl-2-oxobutanoate dehydrogenase (lipoamide), branched-chain alpha-keto acid dehydrogenase E1 beta chain |
| inparanoid | 157307 | DDB_G0268022 | DDB_G0268022 |  |
| inparanoid | 157318 | DDB_G0277689 | DDB_G0277689 |  |
| inparanoid | 157323 | DDB_G0284937 | DDB_G0284937 | rhomboid family protein |
| inparanoid | 157347 | DDB_G0288437 | DDB_G0288437 |  |
| inparanoid | 157354 | DDB_G0281171 | oxaB | putative oxidase assembly protein |
| inparanoid | 157360 | DDB_G0282131 | DDB_G0282131 |  |
| inparanoid | 157361 | DDB_G0282105 | DDB_G0282105 | protein phosphatase 2C |
| inparanoid | 157371 | DDB_G0285643 | mrkB | putative protein serine/threonine kinase, protein kinase, CAMK group, CAMKL family protein kinase, MARK subfamily protein kinase |
| inparanoid | 157372 | DDB_G0285645 | DDB_G0285645 |  |
| inparanoid | 157374 | DDB_G0285649 | DDB_G0285649 | protein phosphatase 2C-related protein |
| inparanoid | 157375 | DDB_G0271252 | DDB_G0271252 |  |
| inparanoid | 157379 | DDB_G0278213 | DDB_G0278213 |  |
| inparanoid | 157381 | DDB_G0284809 | DDB_G0284809 | HesB/YadR/YfhF domain-containing protein |
| inparanoid | 157382 | DDB_G0284811 | DDB_G0284811 |  |
| inparanoid | 157395 | DDB_G0272440 | DDB_G0272440 | putative quinone oxidoreductase, alcohol dehydrogenase |
| inparanoid | 157402 | DDB_G0290009 | sr | sepiapterin reductase |
| inparanoid | 157421 | DDB_G0286051 | leo1 | RNA polymerase II complex component |
| inparanoid | 157422 | DDB_G0286053 | DDB_G0286053 | unknown |
| inparanoid | 157425 | DDB_G0285533 | ppt1 | palmitoyl-protein thioesterase 1 |
| inparanoid | 157428 | DDB_G0285537 | ost3 | dolichyl-diphosphooligosaccharide-protein glycotransferase, oligosaccharyltransferase gamma subunit |
| inparanoid | 157442 | DDB_G0292788 | pex6 | AAA ATPase domain-containing protein, peroxin 6, peroxisomal biogenesis factor 6 |
| inparanoid | 157443 | DDB_G0292770 | jcdE | transcription factor jumonji, jmjC domain-containing protein |
| inparanoid | 157447 | DDB_G0292794 | culD | cullin |
| inparanoid | 157467 | DDB_G0269994 | DDB_G0269994 |  |
| inparanoid | 157468 | DDB_G0269990 | tbcE | tubulin binding cofactor E |
| inparanoid | 157483 | DDB_G0276553 | DDB_G0276553 |  |
| inparanoid | 157489 | DDB_G0288823 | lzic | leucine zipper and ICAT homologous protein LZIC |
| inparanoid | 157492 | DDB_G0288817 | osbK | oxysterol binding family protein, member 11 |
| inparanoid | 157498 | DDB_G0288831 | DDB_G0288831 |  |
| inparanoid | 157501 | DDB_G0289067 | smkA | EVH1 domain-containing protein |
| inparanoid | 157507 | DDB_G0279463 | DDB_G0279463 |  |
| inparanoid | 157508 | DDB_G0279589 | DDB_G0279589 |  |
| inparanoid | 157517 | DDB_G0272580 | DDB_G0272580 |  |
| inparanoid | 157534 | DDB_G0273315 | DDB_G0273315 |  |
| inparanoid | 157536 | DDB_G0273235 | DDB_G0273235 | P-type ATPase |
| inparanoid | 157543 | DDB_G0268794 | bub2 | RabGAP/TBC domain-containing protein, putative GTPase activating protein (GAP), putative mitotic checkpoint protein |
| inparanoid | 157554 | DDB_G0292208 | DDB_G0292208 | transmembrane protein |
| inparanoid | 157558 | DDB_G0288247 | aif | apoptosis inducing factor |
| inparanoid | 157560 | DDB_G0287337 | taf5 | transcription initiation factor TFIID subunit, TFIID subunit |
| inparanoid | 157565 | DDB_G0290487 | DDB_G0290487 |  |
| inparanoid | 157581 | DDB_G0274917 | vps13B |  |
| inparanoid | 157583 | DDB_G0284821 | DDB_G0284821 |  |
| inparanoid | 157609 | DDB_G0271336 | DDB_G0271336 | Similar to amidase. |
| inparanoid | 157617 | DDB_G0270618 | DDB_G0270618 |  |
| inparanoid | 157622 | DDB_G0269704 | DDB_G0269704 |  |
| inparanoid | 157636 | DDB_G0278771 | DDB_G0278771 |  |
| inparanoid | 157638 | DDB_G0278775 | DDB_G0278775 | Q95044 Ubiquitin-conjugating enzyme E2-C (EC 6.3.2.19) (Ubiquitin-protein ligase) (Ubiquitin carrier protein). |
| inparanoid | 157686 | DDB_G0280293 | coq1 | trans-prenyltransferase, hexaprenyl pyrophosphate synthetase |
| inparanoid | 157687 | DDB_G0280291 | DDB_G0280291 | methenyl tetrahydrofolate cyclohydrolase / NADP-dependent methylene H4F dehydrogenase, methenyl tetrahydrofolate cyclohydrolase / NADP-dependent methylene H4F dehydrogenase, mitochondrial |
| inparanoid | 157744 | DDB_G0287035 | lmpB | lysosomal integral membrane protein II |
| inparanoid | 157756 | DDB_G0280141 | DDB_G0280141 |  |
| inparanoid | 157758 | DDB_G0269622 | DDB_G0269622 |  |
| inparanoid | 157765 | DDB_G0269486 | DDB_G0269486 |  |
| inparanoid | 157773 | DDB_G0269034 | udkA | uridine kinase, uridine phosphokinase |
| inparanoid | 157779 | DDB_G0268818 | DDB_G0268818 |  |
| inparanoid | 157783 | DDB_G0268948 | DDB_G0268948 | putative SAM dependent methyltransferase |
| inparanoid | 157788 | DDB_G0290573 | DDB_G0290573 |  |
| inparanoid | 157792 | DDB_G0290577 | dnase2 | deoxyribonuclease II |
| inparanoid | 157793 | DDB_G0290557 | DDB_G0290557 |  |
| inparanoid | 157797 | DDB_G0290541 | DDB_G0290541 |  |
| inparanoid | 157799 | DDB_G0283863 | DDB_G0283863 |  |
| inparanoid | 15780 | DDB_G0268066 | DDB_G0268066 | putative ATPase |
| inparanoid | 157806 | DDB_G0273117 | ublcp1-1 | ubiquitin-like domain-containing CTD phosphatase 1 |
| inparanoid | 157807 | DDB_G0273119 | DDB_G0273119 |  |
| inparanoid | 157810 | DDB_G0273177 | DDB_G0273177 |  |
| inparanoid | 157828 | DDB_G0289835 | DDB_G0289835 | phosphoglycerate/bisphosphoglycerate mutase family protein |
| inparanoid | 157845 | DDB_G0288001 | DDB_G0288001 |  |
| inparanoid | 157851 | DDB_G0289201 | mfsd1 | major facilitator superfamily domain-containing protein 1 |
| inparanoid | 157857 | DDB_G0270434 | med23 | putative mediator complex subunit 23 |
| inparanoid | 157858 | DDB_G0269158 | npcA | Niemann-Pick C type protein |
| inparanoid | 157863 | DDB_G0288355 | DDB_G0288355 | RmlC-like cupin family protein |
| inparanoid | 157872 | DDB_G0277967 | DDB_G0277967 | RabGAP/TBC domain-containing protein, putative GTPase activating protein (GAP) |
| inparanoid | 157882 | DDB_G0279837 | lsm1 | LSM (like-Sm) domain-containing protein |
| inparanoid | 157891 | DDB_G0281111 | DDB_G0281111 |  |
| inparanoid | 157902 | DDB_G0280309 | mcm3 | MCM family protein, putative DNA replication licensing factor |
| inparanoid | 157906 | DDB_G0277763 | DDB_G0277763 | Similar to Arabidopsis thaliana (Mouse-ear cress). phosphonopyruvate decarboxylase-like protein. |
| inparanoid | 157910 | DDB_G0276573 | DDB_G0276573 |  |
| inparanoid | 157915 | DDB_G0268928 | DDB_G0268928 | phosphoesterase, PA-phosphatase related-family protein |
| inparanoid | 157916 | DDB_G0268680 | DDB_G0268680 |  |
| inparanoid | 157917 | DDB_G0268678 | DDB_G0268678 | TH1 family protein, negative transcription elongation factor NELF subunit |
| inparanoid | 157925 | DDB_G0268698 | DDB_G0268698 |  |
| inparanoid | 157928 | DDB_G0279925 | mlkA | myosin light chain kinase, protein serine/threonine kinase, CAMK1 family protein kinase, protein kinase, CAMK group, CAM kinase |
| inparanoid | 157936 | DDB_G0279967 | DDB_G0279967 | UPF0406 family protein |
| inparanoid | 157944 | DDB_G0269790 | gaa | alpha-glucosidase, alpha-glucoside hydrolase |
| inparanoid | 157949 | DDB_G0268708 | DDB_G0268708 |  |
| inparanoid | 157952 | DDB_G0268938 | ube2w | ubiquitin-conjugating enzyme E2W |
| inparanoid | 157953 | DDB_G0268618 | pinA | PinA (Fragment). |
| inparanoid | 157955 | DDB_G0270924 | DDB_G0270924 |  |
| inparanoid | 157966 | DDB_G0271546 | DDB_G0271546 |  |
| inparanoid | 157974 | DDB_G0283727 | DDB_G0283727 | short-chain dehydrogenase/reductase (SDR) family protein |
| inparanoid | 157996 | DDB_G0288839 | DDB_G0288839 |  |
| inparanoid | 157997 | DDB_G0285437 | DDB_G0285437 |  |
| inparanoid | 158011 | DDB_G0270318 | DDB_G0270318 |  |
| inparanoid | 158013 | DDB_G0288389 | DDB_G0288389 |  |
| inparanoid | 158016 | DDB_G0292742 | DDB_G0292742 | C2 calcium/lipid-binding (CaLB) region-containing protein, dilute domain-containing protein |
| inparanoid | 158024 | DDB_G0293210 | sgmB | saposin B domain-containing protein, metallophosphoesterase domain-containing protein, sphingomyelinase |
| inparanoid | 158038 | DDB_G0279601 | utp6 | U3 snoRNP protein, U3 small nucleolar ribonucleoprotein, tetratricopeptide-like helical domain-containing protein (TPR) |
| inparanoid | 158039 | DDB_G0279527 | DDB_G0279527 |  |
| inparanoid | 158047 | DDB_G0278995 | DDB_G0278995 | Rsc12 (Fragment). |
| inparanoid | 158049 | DDB_G0288429 | DDB_G0288429 |  |
| inparanoid | 158053 | DDB_G0287345 | ankrd39 | ankyrin repeat-containing protein 39 |
| inparanoid | 158068 | DDB_G0277691 | cog1 | oligomeric Golgi complex component |
| inparanoid | 158071 | DDB_G0282229 | DDB_G0282229 |  |
| inparanoid | 158074 | DDB_G0276581 | DDB_G0276581 |  |
| inparanoid | 158104 | DDB_G0274799 | cpsf3 | beta-lactamase domain-containing protein, DNA repair metallo-beta-lactamase domain-containing protein, cleavage and polyadenylation specificity factor 73 kDa subunit |
| inparanoid | 158109 | DDB_G0286583 | DDB_G0286583 |  |
| inparanoid | 158110 | DDB_G0286581 | DDB_G0286581 |  |
| inparanoid | 158137 | DDB_G0277537 | DDB_G0277537 | phosphatidylinositol 4-kinase, phosphatidylinositol 4-kinase catalytic subunit, alpha polypeptide |
| inparanoid | 158140 | DDB_G0277877 | uglA | uracil glycosylase, uracil-DNA glycosylase |
| inparanoid | 158154 | DDB_G0288635 | DDB_G0288635 | transmembrane protein |
| inparanoid | 158156 | DDB_G0274853 | DDB_G0274853 |  |
| inparanoid | 158160 | DDB_G0279513 | DDB_G0279513 |  |
| inparanoid | 158197 | DDB_G0272931 | timm9 | mitochondrial import inner membrane translocase subunit 9 |
| inparanoid | 158216 | DDB_G0287891 | bms1l | BMS1-like ribosome biogenesis protein |
| inparanoid | 158224 | DDB_G0288481 | gchA | GTP cyclohydrolase I |
| inparanoid | 158240 | DDB_G0281485 | DDB_G0281485 |  |
| inparanoid | 158242 | DDB_G0278895 | DDB_G0278895 |  |
| inparanoid | 158245 | DDB_G0278897 | DDB_G0278897 |  |
| inparanoid | 158258 | DDB_G0271752 | DDB_G0271752 | NADH:flavin oxidoreductase/NADH oxidase domain-containing protein |
| inparanoid | 158261 | DDB_G0278475 | DDB_G0278475 |  |
| inparanoid | 158270 | DDB_G0277581 | DDB_G0277581 | putative aspartyl protease contains Pfam profile: PF00026 Eukaryotic aspartyl protease |
| inparanoid | 158272 | DDB_G0277629 | rpl7a | 60S ribosomal protein L7a |
| inparanoid | 158278 | DDB_G0276551 | DDB_G0276551 | Similar to plasmodium falciparum (Isolate 3D7). hypothetical 231.8 kDa protein. |
| inparanoid | 158283 | DDB_G0277667 | DDB_G0277667 | cyclin domain-containing protein |
| inparanoid | 158300 | DDB_G0267602 | DDB_G0267602 |  |
| inparanoid | 158303 | DDB_G0267556 | DDB_G0267556 | SNF2-related domain-containing protein, CHR group protein, helicase, C-terminal domain-containing protein |
| inparanoid | 158305 | DDB_G0270500 | DDB_G0270500 | Putative actin interacting protein. |
| inparanoid | 158316 | DDB_G0283063 | DDB_G0283063 | unknown |
| inparanoid | 158317 | DDB_G0279371 | utp14 | U3 snoRNP protein, U3 small nucleolar ribonucleoprotein |
| inparanoid | 158320 | DDB_G0279377 | gtf2f1 | transcription initiation factor IIF subunit alpha |
| inparanoid | 158324 | DDB_G0270990 | DDB_G0270990 | putative acyl-CoA oxidase |
| inparanoid | 158336 | DDB_G0281427 | DDB_G0281427 | Putative sac domain-containing inositol phosphatase 3. |
| inparanoid | 158338 | DDB_G0270808 | scy2 | SCY1 family protein kinase |
| inparanoid | 158341 | DDB_G0270806 | DDB_G0270806 | Glycolate oxidase subunit D-like, D-lactate dehydrogenase-like. |
| inparanoid | 158345 | DDB_G0272706 | DDB_G0272706 |  |
| inparanoid | 158352 | DDB_G0290359 | DDB_G0290359 |  |
| inparanoid | 158359 | DDB_G0269014 | DDB_G0269014 |  |
| inparanoid | 158366 | DDB_G0276113 | DDB_G0276113 |  |
| inparanoid | 158367 | DDB_G0276111 | DDB_G0276111 | F15D4.4 PROTEIN. 6/101 |
| inparanoid | 158383 | DDB_G0282193 | DDB_G0282193 |  |
| inparanoid | 158384 | DDB_G0282177 | DDB_G0282177 |  |
| inparanoid | 158398 | DDB_G0286173 | DDB_G0286173 |  |
| inparanoid | 158416 | DDB_G0269706 | DDB_G0269706 |  |
| inparanoid | 158419 | DDB_G0268110 | DDB_G0268110 | Kelch repeat-containing protein, SAM domain-containing protein |
| inparanoid | 158421 | DDB_G0267464 | polA | mitochondrial DNA polymerase A, DNA polymerase A family protein, 5'-3' exonuclease domain-containing protein, 3'-5' exonuclease domain-containing protein |
| inparanoid | 158427 | DDB_G0272869 | sun1 | SUN domain-containing protein 1 |
| inparanoid | 158435 | DDB_G0293066 | DDB_G0293066 |  |
| inparanoid | 158438 | DDB_G0276129 | cdc123 | cell division cycle protein 123 |
| inparanoid | 158441 | DDB_G0275731 | DDB_G0275731 | calponin homology (CH) domain-containing protein, fimbrin-related RasGAP protein, Ras GTPase activation domain-containing protein |
| inparanoid | 158443 | DDB_G0276711 | rpiA | ribose-5-phosphate isomerase |
| inparanoid | 158444 | DDB_G0275967 | DDB_G0275967 | amidase family protein |
| inparanoid | 158449 | DDB_G0276105 | gtr2 | alpha amylase domain-containing protein, starch synthase-like protein |
| inparanoid | 158466 | DDB_G0288617 | shkB | SH2 domain-containing protein, protein kinase, TKL group, tyrosine kinase-like protein, SHK subfamily protein kinase |
| inparanoid | 158467 | DDB_G0272997 | DDB_G0272997 |  |
| inparanoid | 158468 | DDB_G0288875 | ndufv1 | ubiquinone oxidoreductase, NADH dehydrogenase ubiquinone flavoprotein 1, NADH-ubiquinone oxidoreductase |
| inparanoid | 158484 | DDB_G0288899 | pigM | glycosyltransferase, dolichyl-phosphate-mannose alpha-1,4-mannosyltransferase, DXD motif-containing protein, phosphatidylinositol glycan, class M |
| inparanoid | 158497 | DDB_G0291129 | ndufa6 | NADH dehydrogenase ubiquinone 1 alpha subcomplex subunit 6 |
| inparanoid | 158500 | DDB_G0282729 | DDB_G0282729 | unknown |
| inparanoid | 158520 | DDB_G0268522 | psmG3 | proteasome assembly chaperone 3 |
| inparanoid | 158528 | DDB_G0278493 | DDB_G0278493 | unknown |
| inparanoid | 158531 | DDB_G0279155 | DDB_G0279155 |  |
| inparanoid | 158532 | DDB_G0279151 | cenB | centrin |
| inparanoid | 158535 | DDB_G0278387 | bloc1s2 | biogenesis of lysosome-related organelles complex-1, subunit 2 |
| inparanoid | 158544 | DDB_G0290723 | mkcB | putative protein serine/threonine kinase, STE20 family protein kinase, protein kinase, STE group, MKC subfamily protein kinase |
| inparanoid | 158565 | DDB_G0287825 | DDB_G0287825 |  |
| inparanoid | 158575 | DDB_G0278445 | mpl3 | leucine-rich repeat-containing protein (LRR), putative protein tyrosine phosphatase, dual specificity |
| inparanoid | 158579 | DDB_G0284007 | DDB_G0284007 |  |
| inparanoid | 158583 | DDB_G0283731 | DDB_G0283731 |  |
| inparanoid | 158593 | DDB_G0273453 | DDB_G0273453 | unknown |
| inparanoid | 158595 | DDB_G0268248 | DDB_G0268248 |  |
| inparanoid | 158600 | DDB_G0279613 | DDB_G0279613 | aldehyde dehydrogenase |
| inparanoid | 158602 | DDB_G0279587 | DDB_G0279587 |  |
| inparanoid | 158603 | DDB_G0279409 | cbfA | C-module-binding factor, transcription factor jumonji, jmjC domain-containing protein |
| inparanoid | 158604 | DDB_G0295731 | DDB_G0295731 | ankyrin repeat-containing protein |
| inparanoid | 158630 | DDB_G0292888 | DDB_G0292888 |  |
| inparanoid | 158635 | DDB_G0279835 | DDB_G0279835 |  |
| inparanoid | 158646 | DDB_G0271842 | scsC | succinate-CoA ligase (ADP-forming), succinyl-CoA synthetase (ADP-forming), ATP-specific succinyl-CoA synthetase beta subunit |
| inparanoid | 158674 | DDB_G0289749 | DDB_G0289749 | peptidase S28 family protein |
| inparanoid | 158675 | DDB_G0286303 | ku80 | ATP-dependent DNA helicase, DNA-dependent protein kinase (DNAPK) subunit |
| inparanoid | 158682 | DDB_G0276043 | ifkC | putative protein serine/threonine kinase, putative eukaryotic translation initiation factor 2 alpha (eIF2alpha) kinase, RWD domain-containing protein, anticodon-binding domain-containing protein, GCN2 subfamily protein kinase, PEK family protein kinase |
| inparanoid | 158683 | DDB_G0276167 | DDB_G0276167 |  |
| inparanoid | 158689 | DDB_G0276963 | gefH | Ras guanine nucleotide exchange factor |
| inparanoid | 158734 | DDB_G0267676 | DDB_G0267676 |  |
| inparanoid | 158736 | DDB_G0267422 | tipC |  |
| inparanoid | 158740 | DDB_G0286449 | DDB_G0286449 |  |
| inparanoid | 158742 | DDB_G0286445 | DDB_G0286445 |  |
| inparanoid | 158751 | DDB_G0268186 | DDB_G0268186 | Putative mitotic control protein dis3 (At2g17510/MJB20.7). |
| inparanoid | 158753 | DDB_G0283649 | DDB_G0283649 | NADPH-dependent FMN reductase family protein, putative FMN-dependent NAD(P)H:quinone reductase |
| inparanoid | 158756 | DDB_G0283619 | crlC | cAMP receptor-like protein, G-protein-coupled receptor (GPCR) family protein |
| inparanoid | 158759 | DDB_G0283985 | DDB_G0283985 |  |
| inparanoid | 158763 | DDB_G0288735 | DDB_G0288735 | putative acyl-CoA oxidase |
| inparanoid | 158776 | DDB_G0268230 | DDB_G0268230 |  |
| inparanoid | 158789 | DDB_G0271112 | DDB_G0271112 |  |
| inparanoid | 158791 | DDB_G0271506 | pitrm1 | peptidase M16 family protein, pitrilysin metalloprotease |
| inparanoid | 158792 | DDB_G0271510 | DDB_G0271510 |  |
| inparanoid | 158801 | DDB_G0287055 | vps13F | vacuolar protein sorting-associated protein 13 family protein |
| inparanoid | 158804 | DDB_G0279641 | sae1 | sumo-activating enzyme subunit 1, ubiquitin-like 1-activating enzyme E1A |
| inparanoid | 158808 | DDB_G0286793 | DDB_G0286793 | transmembrane protein |
| inparanoid | 158809 | DDB_G0285811 | DDB_G0285811 |  |
| inparanoid | 158810 | DDB_G0285813 | DDB_G0285813 |  |
| inparanoid | 158821 | DDB_G0274707 | gpt5 | putative glycophosphotransferase |
| inparanoid | 158827 | DDB_G0276119 | cnrF | RabGAP/TBC domain-containing protein, recoverin family protein, EF-hand domain-containing protein |
| inparanoid | 158856 | DDB_G0272634 | dohh-1 | PBS lyase HEAT-like repeat-containing protein, deoxyhypusine monooxygenase, deoxyhypusine hydroxylase, deoxyhypusine dioxygenase |
| inparanoid | 158867 | DDB_G0291133 | DDB_G0291133 | putative protein tyrosine kinase, WEE1 family protein kinase |
| inparanoid | 158874 | DDB_G0293300 | DDB_G0293300 |  |
| inparanoid | 158887 | DDB_G0292446 | DDB_G0292446 | At5g49900. |
| inparanoid | 158914 | DDB_G0272522 | sgkA | sphingosine kinase |
| inparanoid | 158919 | DDB_G0272769 | DDB_G0272769 | TRANSMEMBRANE PROTEIN NRF-6 |
| inparanoid | 158926 | DDB_G0281183 | DDB_G0281183 | RING zinc finger-containing protein |
| inparanoid | 158929 | DDB_G0288511 | sadA | substrate adhesion molecule |
| inparanoid | 158939 | DDB_G0274771 | DDB_G0274771 |  |
| inparanoid | 158944 | DDB_G0272951 | DDB_G0272951 |  |
| inparanoid | 158954 | DDB_G0287875 | DDB_G0287875 | pleckstrin homology (PH) domain-containing protein, calponin homology (CH) domain-containing protein, putative actin binding protein, ras association (RA) domain-containing protein |
| inparanoid | 158968 | DDB_G0293728 | DDB_G0293728 |  |
| inparanoid | 158969 | DDB_G0293628 | DDB_G0293628 |  |
| inparanoid | 158975 | DDB_G0291780 | wbp1 | dolichyl-diphosphooligosaccharide-protein glycotransferase, oligosaccharyltransferase beta subunit |
| inparanoid | 158981 | DDB_G0278271 | DDB_G0278271 |  |
| inparanoid | 159023 | DDB_G0267646 | sec20 | sec20 family protein |
| inparanoid | 159024 | DDB_G0267644 | DDB_G0267644 |  |
| inparanoid | 159025 | DDB_G0268560 | DDB_G0268560 |  |
| inparanoid | 159036 | DDB_G0271844 | DDB_G0271844 | Phox domain-containing protein, Rho GTPase domain containing protein, vacuolar sorting protein 9 domain-containing protein |
| inparanoid | 159058 | DDB_G0271856 | vps52C | Vps52 / Sac2 family protein |
| inparanoid | 159064 | DDB_G0271504 | lvsB | BEACH domain-containing protein |
| inparanoid | 159071 | DDB_G0274871 | DDB_G0274871 | 3-hydroxy-3-methylglutaryl-coenzyme A synthase 2. |
| inparanoid | 159076 | DDB_G0275759 | rpa1 | RNA polymerase I, largest subunit |
| inparanoid | 159078 | DDB_G0291544 | DDB_G0291544 |  |
| inparanoid | 159085 | DDB_G0292608 | med4 | putative mediator complex subunit 4 |
| inparanoid | 159090 | DDB_G0280875 | DDB_G0280875 |  |
| inparanoid | 159091 | DDB_G0269046 | DDB_G0269046 | NAD(P)+ transhydrogenase (AB-specific) |
| inparanoid | 159094 | DDB_G0276297 | DDB_G0276297 |  |
| inparanoid | 159099 | DDB_G0276321 | DDB_G0276321 | putative acetyl-CoA synthetase, putative acetoacetyl-CoA synthetase, AMP-dependent synthetase and ligase domain-containing protein |
| inparanoid | 159101 | DDB_G0287277 | DDB_G0287277 | NAD-dependent epimerase/dehydratase family protein |
| inparanoid | 159107 | DDB_G0277453 | DDB_G0277453 |  |
| inparanoid | 159113 | DDB_G0268200 | DDB_G0268200 |  |
| inparanoid | 159135 | DDB_G0284175 | DDB_G0284175 |  |
| inparanoid | 159138 | DDB_G0291558 | DDB_G0291558 |  |
| inparanoid | 159142 | DDB_G0291534 | DDB_G0291534 |  |
| inparanoid | 159143 | DDB_G0283545 | DDB_G0283545 | Similar to CSRP2 binding protein. |
| inparanoid | 159144 | DDB_G0283543 | prp40 | WW domain-containing protein, putative RNA splicing factor, FF domain-containing protein |
| inparanoid | 159146 | DDB_G0283541 | yipf5 | Yip1 domain-containing protein |
| inparanoid | 159166 | DDB_G0268584 | DDB_G0268584 |  |
| inparanoid | 159167 | DDB_G0268512 | wrn | ATP-dependent DNA helicase RecQ family protein, Werner syndrome protein |
| inparanoid | 159187 | DDB_G0289473 | DDB_G0289473 | P-type ATPase, Ca2+-ATPase |
| inparanoid | 159206 | DDB_G0282831 | DDB_G0282831 |  |
| inparanoid | 159210 | DDB_G0282741 | ddx18 | putative RNA helicase, DEAD/DEAH box helicase |
| inparanoid | 159218 | DDB_G0289919 | ponJ | ponticulin-related protein |
| inparanoid | 159222 | DDB_G0281891 | DDB_G0281891 | RabGAP/TBC domain-containing protein, putative GTPase activating protein (GAP) |
| inparanoid | 159223 | DDB_G0282611 | DDB_G0282611 | phosphopantothenatecysteine ligase |
| inparanoid | 159229 | DDB_G0289325 | scsA | succinate-CoA ligase (GDP-forming), succinyl-CoA synthetase (GDP-forming), GTP-specific succinyl-CoA synthetase alpha subunit |
| inparanoid | 159231 | DDB_G0288939 | valS2 | valyl tRNA synthetase, valine-tRNA ligase |
| inparanoid | 159232 | DDB_G0288017 | DDB_G0288017 | putative sphingomyelinase |
| inparanoid | 159234 | DDB_G0283831 | DDB_G0283831 |  |
| inparanoid | 159259 | DDB_G0284087 | DDB_G0284087 |  |
| inparanoid | 159260 | DDB_G0283819 | DDB_G0283819 | Esterase HDE. |
| inparanoid | 159278 | DDB_G0281027 | DDB_G0281027 |  |
| inparanoid | 159283 | DDB_G0283791 | DDB_G0283791 |  |
| inparanoid | 159298 | DDB_G0281511 | cog3 | oligomeric Golgi complex component, Sec34-like family protein |
| inparanoid | 159300 | DDB_G0273437 | DDB_G0273437 | Similar to Escherichia coli CFT073. glycerol dehydrogenase (EC 1.1.1.6). |
| inparanoid | 159346 | DDB_G0289237 | tirA | Toll-Interleukin (TIR) receptor domain-containing protein |
| inparanoid | 159351 | DDB_G0290853 | stlB | putative polyketide synthase, beta-ketoacyl synthase family protein |
| inparanoid | 159362 | DDB_G0283761 | pgtA | bifunctional glycosyltransferase, N-acetylgalactosamine 3-beta-galactosyltransferase, GlcNAc transferase, alpha-1,2-fucosyltransferase |
| inparanoid | 159363 | DDB_G0283759 | DDB_G0283759 | unknown |
| inparanoid | 159366 | DDB_G0286293 | dnajc13 | DnaJ (Hsp40) homolog, subfamily C, member 13 |
| inparanoid | 159372 | DDB_G0274313 | pex4 | putative ubiquitin-conjugating enzyme E2, peroxisomal biogenesis factor 4, peroxin 4 |
| inparanoid | 159376 | DDB_G0270414 | tmem56C | TRAM, LAG1 and CLN8 homology domain-containing protein, TMEM56 family protein 3 |
| inparanoid | 159389 | DDB_G0270746 | DDB_G0270746 | WD40 repeat-containing protein |
| inparanoid | 159415 | DDB_G0267516 | dhx57 | DEAD/DEAH box helicase, RWD domain-containing protein |
| inparanoid | 159422 | DDB_G0284621 | pwp2 | WD40 repeat-containing protein, periodic tryptophan protein |
| inparanoid | 159427 | DDB_G0274573 | DDB_G0274573 | putative transmembrane protein |
| inparanoid | 159438 | DDB_G0285913 | DDB_G0285913 |  |
| inparanoid | 159443 | DDB_G0293570 | DDB_G0293570 | RUN domain-containing protein |
| inparanoid | 159467 | DDB_G0290697 | DDB_G0290697 |  |
| inparanoid | 159468 | DDB_G0269636 | DDB_G0269636 | EF-hand domain-containing protein, EPS15 homology (EH) domain-containing protein |
| inparanoid | 159469 | DDB_G0269638 | dr1 | putative histone-like transcription factor |
| inparanoid | 159472 | DDB_G0284537 | DDB_G0284537 |  |
| inparanoid | 159481 | DDB_G0289875 | uppA | UDP-glucose pyrophosphorylase, UTP-glucose-1-phosphate uridylyltransferase |
| inparanoid | 159488 | DDB_G0288369 | DDB_G0288369 |  |
| inparanoid | 159502 | DDB_G0286595 | DDB_G0286595 |  |
| inparanoid | 159503 | DDB_G0286205 | DDB_G0286205 |  |
| inparanoid | 159511 | DDB_G0292320 | DDB_G0292320 | UNC-50 family protein |
| inparanoid | 159521 | DDB_G0276695 | DDB_G0276695 |  |
| inparanoid | 159524 | DDB_G0276721 | tyrS | tyrosine-tRNA ligase, tyrosyl-tRNA synthetase |
| inparanoid | 159528 | DDB_G0271240 | utp18 | WD40 repeat-containing protein, U3 snoRNP protein, U3 small nucleolar ribonucleoprotein |
| inparanoid | 159536 | DDB_G0284081 | DDB_G0284081 | Non-transporter ABC protein AbcF2. |
| inparanoid | 159537 | DDB_G0284047 | abcF2 | ABC transporter-related protein |
| inparanoid | 159538 | DDB_G0283655 | DDB_G0283655 |  |
| inparanoid | 159561 | DDB_G0287031 | gpaC | G-protein subunit alpha 3 |
| inparanoid | 159564 | DDB_G0277077 | DDB_G0277077 | UvrB/UvrC domain-containing protein |
| inparanoid | 159593 | DDB_G0289429 | DDB_G0289429 |  |
| inparanoid | 159600 | DDB_G0287815 | DDB_G0287815 |  |
| inparanoid | 159601 | DDB_G0287839 | DDB_G0287839 | Probable adenylate kinase (EC 2.7.4.3). |
| inparanoid | 159603 | DDB_G0291324 | DDB_G0291324 |  |
| inparanoid | 159608 | DDB_G0292042 | DDB_G0292042 | glucose-methanol-choline oxidoreductase |
| inparanoid | 159621 | DDB_G0288895 | DDB_G0288895 |  |
| inparanoid | 159626 | DDB_G0283099 | DDB_G0283099 | nipped-B protein, HEAT repeat-containing protein, armadillo-like helical domain-containing protein |
| inparanoid | 159629 | DDB_G0288757 | DDB_G0288757 | nicotinamide-nucleotide adenylyltransferase |
| inparanoid | 159642 | DDB_G0283757 | rab1A | Rab GTPase |
| inparanoid | 159660 | DDB_G0284169 | DDB_G0284169 |  |
| inparanoid | 159667 | DDB_G0287063 | DDB_G0287063 | unknown |
| inparanoid | 159697 | DDB_G0286761 | DDB_G0286761 |  |
| inparanoid | 159715 | DDB_G0277907 | rplP1 | ribosomal acidic phosphoprotein P1, 60S acidic ribosomal protein P1 |
| inparanoid | 159723 | DDB_G0274483 | DDB_G0274483 | Similar to Homo sapiens (Human). protein KIAA1404. |
| inparanoid | 15973 | DDB_G0286237 | DDB_G0286237 |  |
| inparanoid | 159737 | DDB_G0281343 | shkD | SH2 domain-containing protein, protein kinase, TKL group, tyrosine kinase-like protein, SHK subfamily protein kinase |
| inparanoid | 15974 | DDB_G0272242 | cupI | ricin B lectin domain-containing protein, cup family protein |
| inparanoid | 159756 | DDB_G0274183 | DDB_G0274183 | leucine-rich repeat-containing protein (LRR) |
| inparanoid | 159759 | DDB_G0282095 | DDB_G0282095 | beta-lactamase family protein |
| inparanoid | 159760 | DDB_G0282413 | DDB_G0282413 |  |
| inparanoid | 159774 | DDB_G0272732 | emg1 | putative ribosome biogenesis protein |
| inparanoid | 159779 | DDB_G0276333 | tipB | unknown |
| inparanoid | 159783 | DDB_G0276157 | pkgA | protein serine/threonine kinase, protein kinase, AGC group, MAST family protein kinase |
| inparanoid | 159833 | DDB_G0282497 | DDB_G0282497 |  |
| inparanoid | 159841 | DDB_G0291990 | febA | 4E-binding protein homolog |
| inparanoid | 159842 | DDB_G0292024 | DDB_G0292024 | putative protein tyrosine phosphatase, dual specificity |
| inparanoid | 159848 | DDB_G0272544 | DDB_G0272544 | reduced folate carrier family protein |
| inparanoid | 159856 | DDB_G0272246 | prsB | phosphoribosyl pyrophosphate synthetase |
| inparanoid | 159857 | DDB_G0272484 | DDB_G0272484 | HD phosphohydrolase domain-containing protein |
| inparanoid | 159871 | DDB_G0286443 | DDB_G0286443 |  |
| inparanoid | 159885 | DDB_G0288965 | DDB_G0288965 |  |
| inparanoid | 159892 | DDB_G0273101 | DDB_G0273101 | armadillo-like helical domain-containing protein, SEC7-like domain-containing protein |
| inparanoid | 159895 | DDB_G0267836 | DDB_G0267836 |  |
| inparanoid | 159905 | DDB_G0271858 | mpgB | mannose-1-phosphate guanylyltransferase, GTP:alpha-D-mannose-1-phosphate guanylyltransferase |
| inparanoid | 159906 | DDB_G0271880 | DDB_G0271880 | UPF0183 family protein |
| inparanoid | 159911 | DDB_G0281327 | DDB_G0281327 |  |
| inparanoid | 159914 | DDB_G0281503 | DDB_G0281503 | Similar to Babesia bigemina. 200 kDa antigen p200. |
| inparanoid | 159915 | DDB_G0281505 | DDB_G0281505 |  |
| inparanoid | 159919 | DDB_G0292140 | DDB_G0292140 | SET domain-containing protein |
| inparanoid | 159921 | DDB_G0292138 | DDB_G0292138 |  |
| inparanoid | 159955 | DDB_G0274975 | DDB_G0274975 |  |
| inparanoid | 159956 | DDB_G0286167 | DDB_G0286167 | WD-40 repeat-containing protein |
| inparanoid | 159964 | DDB_G0289495 | DDB_G0289495 | vacuolar sorting protein 9 domain-containing protein |
| inparanoid | 159968 | DDB_G0282623 | wdr3 | WD40 repeat-containing protein, U3 snoRNP protein, U3 small nucleolar ribonucleoprotein |
| inparanoid | 159970 | DDB_G0272638 | DDB_G0272638 | phosphatidylinositol-4-phosphate 5-kinase (PIP5K) family protein |
| inparanoid | 159971 | DDB_G0271926 | DDB_G0271926 |  |
| inparanoid | 159984 | DDB_G0272012 | DDB_G0272012 | GNS1/SUR4 family protein |
| inparanoid | 160000 | DDB_G0270496 | DDB_G0270496 | TRAUB family protein |
| inparanoid | 160005 | DDB_G0269370 | DDB_G0269370 | NUDIX hydrolase family protein, cleavage and polyadenylation specificity factor 5-like protein |
| inparanoid | 160021 | DDB_G0275323 | tipD | autophagy protein 16 |
| inparanoid | 16003 | DDB_G0282515 | DDB_G0282515 |  |
| inparanoid | 160044 | DDB_G0278483 | ngap | RasGTPase-activating protein, NGAP-like protein, Ras p21 protein activator, C2 domain-containing protein |
| inparanoid | 160058 | DDB_G0273417 | DDB_G0273417 |  |
| inparanoid | 16006 | DDB_G0272777 | DDB_G0272777 | VCP-like ATPase. |
| inparanoid | 160083 | DDB_G0275471 | CSN8 | COP9 signalosome complex subunit 8 |
| inparanoid | 160085 | DDB_G0275933 | DDB_G0275933 |  |
| inparanoid | 160087 | DDB_G0275467 | DDB_G0275467 | 5'-nucleotidase |
| inparanoid | 160096 | DDB_G0276783 | DDB_G0276783 | Putative nuclease |
| inparanoid | 160097 | DDB_G0272767 | DDB_G0272767 |  |
| inparanoid | 160114 | DDB_G0288575 | DDB_G0288575 |  |
| inparanoid | 160116 | DDB_G0288469 | DDB_G0288469 |  |
| inparanoid | 160117 | DDB_G0282695 | DDB_G0282695 |  |
| inparanoid | 160120 | DDB_G0282723 | tmem104 | transmembrane protein, amino acid/polyamine transporter II, amino acid transporter, amino acid permease |
| inparanoid | 160141 | DDB_G0286479 | DDB_G0286479 | phosphatidylinositol 4-kinase |
| inparanoid | 160156 | DDB_G0271862 | eIF2s1 | eukaryotic translation initiation factor 2 (eIF2) subunit 1, eIF-2 alpha |
| inparanoid | 16016 | DDB_G0272678 | DDB_G0272678 | Phosphatidylethanolamine N-methyltransferase |
| inparanoid | 160184 | DDB_G0289527 | DDB_G0289527 |  |
| inparanoid | 160203 | DDB_G0291532 | DDB_G0291532 | Q15404 Ras suppressor protein 1 (Rsu-1) (RSP-1). |
| inparanoid | 160208 | DDB_G0268642 | DDB_G0268642 | putative protein serine/threonine kinase, putative eukaryotic translation initiation factor 2 alpha (eIF2alpha) kinase, PEK family protein kinase |
| inparanoid | 160210 | DDB_G0276427 | osbF | oxysterol binding family protein, member 6 |
| inparanoid | 160263 | DDB_G0268518 | DDB_G0268518 |  |
| inparanoid | 160267 | DDB_G0278299 | DDB_G0278299 |  |
| inparanoid | 160270 | DDB_G0288529 | DDB_G0288529 |  |
| inparanoid | 160278 | DDB_G0282473 | ints9 | integrator complex subunit 9 |
| inparanoid | 160291 | DDB_G0272424 | DDB_G0272424 | GCN5-related N-acetyltransferase |
| inparanoid | 160299 | DDB_G0286487 | DDB_G0286487 | seven transmembrane domain protein |
| inparanoid | 160300 | DDB_G0286495 | itpa | inosine triphosphate pyrophosphatase, inosine triphosphatase |
| inparanoid | 160307 | DDB_G0284011 | DDB_G0284011 |  |
| inparanoid | 160311 | DDB_G0284777 | DDB_G0284777 | DUF1183 family protein |
| inparanoid | 160363 | DDB_G0280015 | DDB_G0280015 |  |
| inparanoid | 160364 | DDB_G0279963 | DDB_G0279963 |  |
| inparanoid | 160380 | DDB_G0280895 | DDB_G0280895 | WD40 repeat-containing protein, cyclin-like F-box containing protein, START domain-containing protein |
| inparanoid | 160396 | DDB_G0274257 | DDB_G0274257 | FRAGILE HISTIDINE TRIAD PROTEIN |
| inparanoid | 160424 | DDB_G0292306 | atp5C1 | ATP synthase gamma chain, F1-ATPase gamma subunit |
| inparanoid | 160426 | DDB_G0287253 | gemin2 | component of gems 2 |
| inparanoid | 160448 | DDB_G0282615 | midA | DUF185 family protein |
| inparanoid | 160449 | DDB_G0282617 | psmF1 | proteasome inhibitor PI31 subunit |
| inparanoid | 160469 | DDB_G0285269 | DDB_G0285269 | CAF1 family protein, putative CCR4-NOT complex subunit |
| inparanoid | 160487 | DDB_G0281097 | DDB_G0281097 | pseudouridine synthase family protein |
| inparanoid | 16127 | DDB_G0283263 | migA | BTB/POZ domain-containing protein, coagulation factor 5/8 type C-terminal domain-containing protein |
| inparanoid | 16141 | DDB_G0292414 | DDB_G0292414 |  |
| inparanoid | 16311 | DDB_G0268270 | DDB_G0268270 |  |
| inparanoid | 16456 | DDB_G0278535 | DDB_G0278535 | ankyrin repeat-containing protein, SAM domain-containing protein, protein kinase, TKL group, tyrosine kinase-like protein, ARK family protein kinase |
| inparanoid | 16531 | DDB_G0276561 | DDB_G0276561 |  |
| inparanoid | 16576 | DDB_G0274923 | DDB_G0274923 |  |
| inparanoid | 16626 | DDB_G0277801 | gtpbp5 | GTP1/OBG family protein, GTP-binding protein 5 |
| inparanoid | 16710 | DDB_G0286763 | DDB_G0286763 |  |
| inparanoid | 16805 | DDB_G0271014 | DDB_G0271014 |  |
| inparanoid | 16883 | DDB_G0272983 | DDB_G0272983 |  |
| inparanoid | 16972 | DDB_G0293380 | DDB_G0293380 | putative RNA methylase domain-containing protein |
| inparanoid | 16980 | DDB_G0270246 | DDB_G0270246 | regulator of chromosome condensation (RCC1) domain-containing protein |
| inparanoid | 17048 | DDB_G0272841 | DDB_G0272841 | Similar to Arabidopsis thaliana (Mouse-ear cress). Purple acid phosphatase, putative. |
| inparanoid | 17195 | DDB_G0280485 | jcdH | transcription factor jumonji, jmjC domain-containing protein |
| inparanoid | 17426 | DDB_G0287297 | p80 | endosomal membrane protein |
| inparanoid | 17436 | DDB_G0278255 | DDB_G0278255 | regulator of chromosome condensation (RCC1) domain-containing protein |
| inparanoid | 17481 | DDB_G0290153 | DDB_G0290153 |  |
| inparanoid | 17501 | DDB_G0287303 | DDB_G0287303 | putative amino acid/polyamine transporter I, putative amino acid permease |
| inparanoid | 17550 | DDB_G0291287 | DDB_G0291287 |  |
| inparanoid | 17662 | DDB_G0282713 | DDB_G0282713 |  |
| inparanoid | 17677 | DDB_G0286743 | CYP555A1 | cytochrome P450 family protein |
| inparanoid | 17822 | DDB_G0275521 | DDB_G0275521 |  |
| inparanoid | 17837 | DDB_G0287713 | DDB_G0287713 |  |
| inparanoid | 17903 | DDB_G0273199 | DDB_G0273199 | putative protein tyrosine phosphatase, dual specificity |
| inparanoid | 17925 | DDB_G0276213 | DDB_G0276213 |  |
| inparanoid | 18166 | DDB_G0267392 | plbG | phospholipase B-like protein |
| inparanoid | 18219 | DDB_G0278313 | DDB_G0278313 | pectin lyase-like family protein, polymorphic membrane protein repeat-containing protein |
| inparanoid | 18260 | DDB_G0270192 | DDB_G0270192 |  |
| inparanoid | 18286 | DDB_G0267770 | DDB_G0267770 |  |
| inparanoid | 18291 | DDB_G0278227 | DDB_G0278227 |  |
| inparanoid | 18398 | DDB_G0278497 | DDB_G0278497 |  |
| inparanoid | 18468 | DDB_G0273261 | sigL | EGF-like domain-containing protein |
| inparanoid | 18522 | DDB_G0268480 | cdk10 | putative protein serine/threonine kinase, CDK family protein kinase, putative cyclin-dependent kinase, protein kinase, CMGC group, PITSLRE subfamily protein kinase |
| inparanoid | 18544 | DDB_G0272500 | DDB_G0272500 |  |
| inparanoid | 18614 | DDB_G0289035 | DDB_G0289035 |  |
| inparanoid | 18720 | DDB_G0281759 | DDB_G0281759 |  |
| inparanoid | 18752 | DDB_G0284883 | oxaA | putative oxidase assembly protein |
| inparanoid | 18795 | DDB_G0272224 | DDB_G0272224 | NAD+ kinase family protein |
| inparanoid | 18799 | DDB_G0294370 | DDB_G0294370 | Reverse transcriptase-like polymerase (Fragment). |
| inparanoid | 18874 | DDB_G0284507 | DDB_G0284507 | putative DNA repair protein, putative Rad51C protein |
| inparanoid | 18880 | DDB_G0279111 | DDB_G0279111 | AAA ATPase domain-containing protein |
| inparanoid | 19018 | DDB_G0293054 | DDB_G0293054 |  |
| inparanoid | 19119 | DDB_G0273279 | DDB_G0273279 |  |
| inparanoid | 19562 | DDB_G0292288 | DDB_G0292288 |  |
| inparanoid | 19653 | DDB_G0291774 | DDB_G0291774 |  |
| inparanoid | 19819 | DDB_G0293990 | DDB_G0293990 |  |
| inparanoid | 19838 | DDB_G0273299 | DDB_G0273299 |  |
| inparanoid | 19840 | DDB_G0291031 | ccs | copper chaperone for superoxide dismutase |
| inparanoid | 19880 | DDB_G0290427 | DDB_G0290427 |  |
| inparanoid | 19917 | DDB_G0281139 | DDB_G0281139 |  |
| inparanoid | 20033 | DDB_G0284217 | DDB_G0284217 | CAF1 family protein, putative CCR4-NOT complex subunit |
| inparanoid | 20077 | DDB_G0287655 | DDB_G0287655 |  |
| inparanoid | 20225 | DDB_G0289891 | DDB_G0289891 |  |
| inparanoid | 20262 | DDB_G0280207 | DDB_G0280207 |  |
| inparanoid | 20344 | DDB_G0285593 | nfu1 | NIF system FeS cluster assembly domain-containing protein |
| inparanoid | 20418 | DDB_G0271854 | DDB_G0271854 | HYPOTHETICAL 24.4 KDA PROTEIN |
| inparanoid | 20507 | DDB_G0285413 | DDB_G0285413 |  |
| inparanoid | 20552 | DDB_G0274873 | pex19 | peroxin 19, peroxisomal biogenesis factor 19 |
| inparanoid | 20609 | DDB_G0277263 | DDB_G0277263 | Similar to Actinobacillus pleuropneumoniae (Haemophilus pleuropneumoniae). urea transport protein Utp. |
| inparanoid | 20708 | DDB_G0280573 | DDB_G0280573 |  |
| inparanoid | 20741 | DDB_G0283871 | DDB_G0283871 | unknown |
| inparanoid | 20776 | DDB_G0273425 | rpc25 | RNA polymerase III subunit |
| inparanoid | 20777 | DDB_G0274609 | DDB_G0274609 | Similar to Dictyostelium discoideum (Slime mold). hypothetical 98.7 kDa protein (Fragment). |
| inparanoid | 20813 | DDB_G0272546 | DDB_G0272546 |  |
| inparanoid | 20838 | DDB_G0272358 | DDB_G0272358 |  |
| inparanoid | 20860 | DDB_G0269836 | DDB_G0269836 | R2005 protein. |
| inparanoid | 20966 | DDB_G0277025 | DDB_G0277025 |  |
| inparanoid | 21106 | DDB_G0291416 | DDB_G0291416 |  |
| inparanoid | 21228 | DDB_G0269238 | rab11A | Rab GTPase |
| inparanoid | 21278 | DDB_G0275147 | DDB_G0275147 |  |
| inparanoid | 21416 | DDB_G0281459 | DDB_G0281459 | Similar to Dictyostelium discoideum (Slime mold). hypothetical 127.0 kDa protein. |
| inparanoid | 21469 | DDB_G0276849 | DDB_G0276849 |  |
| inparanoid | 21493 | DDB_G0270980 | DDB_G0270980 |  |
| inparanoid | 21542 | DDB_G0285491 | DDB_G0285491 |  |
| inparanoid | 21561 | DDB_G0270710 | DDB_G0270710 |  |
| inparanoid | 21784 | DDB_G0268984 | DDB_G0268984 |  |
| inparanoid | 21788 | DDB_G0270340 | rapC | Ras GTPase |
| inparanoid | 21913 | DDB_G0286005 | DDB_G0286005 | putative transmembrane protein |
| inparanoid | 22037 | DDB_G0282451 | DDB_G0282451 |  |
| inparanoid | 22069 | DDB_G0267216 | DDB_G0267216 |  |
| inparanoid | 22138 | DDB_G0293766 | MTH | NUDIX hydrolase family protein, 7,8-dihydro-8-oxoguanine triphosphatase , mutT homolog |
| inparanoid | 22139 | DDB_G0270248 | DDB_G0270248 |  |
| inparanoid | 22143 | DDB_G0274465 | DDB_G0274465 | unknown |
| inparanoid | 22158 | DDB_G0269066 | DDB_G0269066 |  |
| inparanoid | 22179 | DDB_G0271864 | mocs2l | molybdenum cofactor synthesis protein 2 large subunit |
| inparanoid | 22387 | DDB_G0289997 | DDB_G0289997 |  |
| inparanoid | 22402 | DDB_G0292254 | DDB_G0292254 |  |
| inparanoid | 22470 | DDB_G0272142 | DDB_G0272142 | 1110001A02RIK PROTEIN. 6/101 |
| inparanoid | 22474 | DDB_G0281601 | DDB_G0281601 |  |
| inparanoid | 22475 | DDB_G0282587 | DDB_G0282587 |  |
| inparanoid | 22488 | DDB_G0287731 | DDB_G0287731 |  |
| inparanoid | 22541 | DDB_G0291015 | DDB_G0291015 | putative small nuclear ribonucleoparticle-associated protein, LSM (like-Sm) domain-containing protein |
| inparanoid | 22581 | DDB_G0279047 | DDB_G0279047 | unknown |
| inparanoid | 22622 | DDB_G0287601 | DDB_G0287601 |  |
| inparanoid | 22775 | DDB_G0270900 | DDB_G0270900 |  |
| inparanoid | 23422 | DDB_G0269492 | DDB_G0269492 |  |
| inparanoid | 23568 | DDB_G0280783 | DDB_G0280783 |  |
| inparanoid | 23687 | DDB_G0269552 | DDB_G0269552 |  |
| inparanoid | 23747 | DDB_G0268926 | DDB_G0268926 |  |
| inparanoid | 23749 | DDB_G0276471 | DDB_G0276471 |  |
| inparanoid | 23781 | DDB_G0268998 | DDB_G0268998 |  |
| inparanoid | 23857 | DDB_G0278753 | cyb5C | cytochrome b5 C |
| inparanoid | 23930 | DDB_G0273319 | DDB_G0273319 |  |
| inparanoid | 24005 | DDB_G0278371 | spc1 | signal peptidase complex subunit 1, microsomal signal peptidase subunit |
| inparanoid | 24036 | DDB_G0269512 | DDB_G0269512 |  |
| inparanoid | 24069 | DDB_G0294400 | DDB_G0294400 |  |
| inparanoid | 24114 | DDB_G0291420 | DDB_G0291420 |  |
| inparanoid | 24460 | DDB_G0273027 | DDB_G0273027 | Putative RNaseIII. |
| inparanoid | 24907 | DDB_G0274911 | DDB_G0274911 | Similar to Dictyostelium discoideum (Slime mold). coronin binding protein. |
| inparanoid | 24920 | DDB_G0274559 | udkB | uridine kinase |
| inparanoid | 24961 | DDB_G0274407 | DDB_G0274407 | Similar to ribosomal protein; protein id: At4g36420.1, supported by cDNA: gi_15450414, supported by cDNA: gi_17104652. |
| inparanoid | 24967 | DDB_G0274393 | DDB_G0274393 |  |
| inparanoid | 25009 | DDB_G0289979 | DDB_G0289979 | pleckstrin homology (PH) domain-containing protein, putative tyrosine phosphatase, C2 calcium/lipid-binding (CaLB) region-containing protein |
| inparanoid | 25046 | DDB_G0272184 | DDB_G0272184 | zinc-containing alcohol dehydrogenase (ADH) |
| inparanoid | 25072 | DDB_G0282467 | DDB_G0282467 | 1-aminocyclopropane-1-carboxylate synthase, S-adenosyl-L-methionine methylthioadenosine-lyase, ACC synthase |
| inparanoid | 25134 | DDB_G0272985 | vps54 | vacuolar protein sorting 54 family protein |
| inparanoid | 25197 | DDB_G0269980 | drpp30 | RNase P protein subunit, RNase MRP protein subunit |
| inparanoid | 25203 | DDB_G0272198 | DDB_G0272198 |  |
| inparanoid | 25225 | DDB_G0269306 | dhx35 | DEAD/DEAH box helicase |
| inparanoid | 25237 | DDB_G0270786 | DDB_G0270786 |  |
| inparanoid | 25293 | DDB_G0269932 | uap56 | DEAD/DEAH box helicase, putative RNA splicing factor, ATP-dependent RNA helicase |
| inparanoid | 25333 | DDB_G0269716 | DDB_G0269716 |  |
| inparanoid | 25363 | DDB_G0281761 | DDB_G0281761 |  |
| inparanoid | 25367 | DDB_G0281751 | DDB_G0281751 |  |
| inparanoid | 25383 | DDB_G0281899 | drkC | protein tyrosine kinase, E set domain-containing protein, protein kinase, TKL group, DRK subfamily protein kinase |
| inparanoid | 25406 | DDB_G0281901 | slu7 | putative RNA splicing factor |
| inparanoid | 25418 | DDB_G0281903 | DDB_G0281903 |  |
| inparanoid | 25422 | DDB_G0281815 | DDB_G0281815 | DUF410 family protein, UPF0363 family protein |
| inparanoid | 25433 | DDB_G0282149 | DDB_G0282149 |  |
| inparanoid | 25440 | DDB_G0281917 | DDB_G0281917 |  |
| inparanoid | 25505 | DDB_G0281987 | lyrm1 | LYR motif-containing protein 1 |
| inparanoid | 25523 | DDB_G0281979 | DDB_G0281979 |  |
| inparanoid | 25545 | DDB_G0286671 | sir2B | ankyrin repeat-containing protein, NAD(+)-dependent deacetylase, silent information regulator protein (Sir2) family protein |
| inparanoid | 25595 | DDB_G0283013 | DDB_G0283013 |  |
| inparanoid | 25596 | DDB_G0283061 | DDB_G0283061 |  |
| inparanoid | 25624 | DDB_G0283307 | prpf39 | tetratricopeptide-like helical domain-containing protein (TPR), putative U1 small nuclear ribonucleoparticle-associated protein, pre-mRNA processing factor 39 |
| inparanoid | 25628 | DDB_G0282969 | masB | malate synthase |
| inparanoid | 25738 | DDB_G0274439 | DDB_G0274439 | bolA family protein |
| inparanoid | 25751 | DDB_G0274493 | lig1 | DNA ligase I |
| inparanoid | 25815 | DDB_G0274547 | DDB_G0274547 |  |
| inparanoid | 25816 | DDB_G0288255 | DDB_G0288255 | Cfr family protein. |
| inparanoid | 25863 | DDB_G0274329 | DDB_G0274329 | cyclophilin-type peptidylprolyl cis-trans isomerase (PPIase) |
| inparanoid | 25866 | DDB_G0274565 | erh | enhancer of rudimentary family protein |
| inparanoid | 25909 | DDB_G0271310 | ucpA | transmembrane protein, mitochondrial substrate carrier family protein, putative mitochondrial oxaloacetate transporter |
| inparanoid | 25949 | DDB_G0267486 | DDB_G0267486 | Y73F8A.27 protein. |
| inparanoid | 25959 | DDB_G0275065 | dmpA | transmembrane protein |
| inparanoid | 25972 | DDB_G0267484 | acp1 | acid phosphatase 1 |
| inparanoid | 25982 | DDB_G0287829 | ecd | SGT1 protein, ecdysoneless-like protein |
| inparanoid | 25984 | DDB_G0267996 | DDB_G0267996 |  |
| inparanoid | 26064 | DDB_G0275847 | DDB_G0275847 |  |
| inparanoid | 26115 | DDB_G0278903 | DDB_G0278903 |  |
| inparanoid | 26130 | DDB_G0292558 | fam96A | DUF59 family protein, FAM96 family protein |
| inparanoid | 26132 | DDB_G0293206 | DDB_G0293206 |  |
| inparanoid | 26158 | DDB_G0278747 | DDB_G0278747 |  |
| inparanoid | 26201 | DDB_G0292078 | DDB_G0292078 |  |
| inparanoid | 26215 | DDB_G0292074 | commd1 | COMM domain-containing protein 1 |
| inparanoid | 26223 | DDB_G0286103 | DDB_G0286103 | small MutS related (smr) family protein |
| inparanoid | 26247 | DDB_G0291998 | naglu | alpha-N-acetylglucosaminidase |
| inparanoid | 26270 | DDB_G0276885 | casK | protein serine/threonine kinase, protein kinase, CMGC group, CK2 family protein kinase, casein kinase II alpha chain |
| inparanoid | 26301 | DDB_G0284381 | DDB_G0284381 | peptidase C19 family protein, putative ubiquitin carboxyl-terminal hydrolase (UCH) |
| inparanoid | 26332 | DDB_G0284685 | mpi | mannose-6-phosphate isomerase, phosphomannose isomerase |
| inparanoid | 26339 | DDB_G0290041 | DDB_G0290041 |  |
| inparanoid | 26366 | DDB_G0284199 | DDB_G0284199 |  |
| inparanoid | 26369 | DDB_G0284205 | ubl5 | ubiquitin-like protein 5 |
| inparanoid | 26374 | DDB_G0283683 | DDB_G0283683 | leucine-rich repeat-containing protein (LRR) |
| inparanoid | 26389 | DDB_G0283677 | DDB_G0283677 | AgCP15508 (Fragment). |
| inparanoid | 26398 | DDB_G0283459 | gcn5 | bromodomain-containing protein, HAG group protein, GCN5-related N-acetyltransferase, GNAT family protein, putative histone acetyltransferase |
| inparanoid | 26402 | DDB_G0284801 | DDB_G0284801 |  |
| inparanoid | 26434 | DDB_G0272857 | rapB | Ras GTPase |
| inparanoid | 26502 | DDB_G0272971 | DDB_G0272971 |  |
| inparanoid | 26513 | DDB_G0272710 | nat9 | N-acetyltransferase 9 |
| inparanoid | 26566 | DDB_G0279903 | wdr12 | WD40 repeat-containing protein, NLE domain-containing protein |
| inparanoid | 26606 | DDB_G0272604 | CYP508A2-1 | cytochrome P450 family protein |
| inparanoid | 26607 | DDB_G0279905 | DDB_G0279905 |  |
| inparanoid | 26608 | DDB_G0292584 | rnaseh2a | ribonuclease H2 subunit A, RNase H2 subunit A |
| inparanoid | 26615 | DDB_G0292588 | rer1 | retention in endoplasmic reticulum 1 homolog |
| inparanoid | 26622 | DDB_G0292596 | DDB_G0292596 | putative ubiquitin-conjugating enzyme E2 |
| inparanoid | 26627 | DDB_G0268564 | DDB_G0268564 | transmembrane protein, putative peroxisomal membrane protein |
| inparanoid | 26651 | DDB_G0278945 | DDB_G0278945 |  |
| inparanoid | 26655 | DDB_G0271272 | DDB_G0271272 | DNA polymerase V family protein |
| inparanoid | 26662 | DDB_G0278831 | DDB_G0278831 |  |
| inparanoid | 26752 | DDB_G0293312 | DDB_G0293312 |  |
| inparanoid | 26757 | DDB_G0293866 | DDB_G0293866 |  |
| inparanoid | 26772 | DDB_G0293986 | qpct | peptidase M28E domain containing-protein, glutaminyl-peptide cyclotransferase |
| inparanoid | 26774 | DDB_G0282833 | DDB_G0282833 |  |
| inparanoid | 26830 | DDB_G0277043 | DDB_G0277043 |  |
| inparanoid | 26840 | DDB_G0277019 | wdr36 | WD40 repeat-containing protein, U3 snoRNP protein, U3 small nucleolar ribonucleoprotein |
| inparanoid | 26867 | DDB_G0276865 | clcB | chloride channel protein, CLC 6/7 family protein |
| inparanoid | 26932 | DDB_G0292408 | DDB_G0292408 |  |
| inparanoid | 26951 | DDB_G0269510 | vps37 | Modifier of rudimentary (Modr) family protein |
| inparanoid | 26967 | DDB_G0274779 | DDB_G0274779 |  |
| inparanoid | 26975 | DDB_G0292410 | DDB_G0292410 | CCCH-type zinc finger-containing protein |
| inparanoid | 26995 | DDB_G0282879 | DDB_G0282879 |  |
| inparanoid | 27048 | DDB_G0279019 | DDB_G0279019 |  |
| inparanoid | 27061 | DDB_G0279067 | DDB_G0279067 |  |
| inparanoid | 27070 | DDB_G0278575 | DDB_G0278575 |  |
| inparanoid | 27074 | DDB_G0293168 | ddx17 | putative RNA helicase, DEAD/DEAH box helicase |
| inparanoid | 27078 | DDB_G0282477 | DDB_G0282477 |  |
| inparanoid | 27081 | DDB_G0279107 | DDB_G0279107 |  |
| inparanoid | 27183 | DDB_G0277189 | DDB_G0277189 |  |
| inparanoid | 27193 | DDB_G0276237 | DDB_G0276237 | putative protein arginine methyltransferase |
| inparanoid | 27199 | DDB_G0277327 | DDB_G0277327 |  |
| inparanoid | 27213 | DDB_G0275091 | DDB_G0275091 |  |
| inparanoid | 27214 | DDB_G0277013 | DDB_G0277013 |  |
| inparanoid | 27230 | DDB_G0275371 | DDB_G0275371 | unknown |
| inparanoid | 27234 | DDB_G0276675 | DDB_G0276675 |  |
| inparanoid | 27237 | DDB_G0277195 | mrps17 | ribosomal protein S17, mitochondrial |
| inparanoid | 27239 | DDB_G0277007 | DDB_G0277007 | Similar to Homo sapiens (Human). GDP-fucose transporter 1. |
| inparanoid | 27255 | DDB_G0277333 | DDB_G0277333 |  |
| inparanoid | 27302 | DDB_G0275211 | srp19 | signal recognition particle 19 kDa subunit |
| inparanoid | 27344 | DDB_G0267482 | DDB_G0267482 |  |
| inparanoid | 27346 | DDB_G0268140 | DDB_G0268140 |  |
| inparanoid | 27357 | DDB_G0267388 | dafE | diaminopimelate epimerase |
| inparanoid | 27418 | DDB_G0268190 | fut11 | glycosyltransferase, alpha-3/4-fucosyltransferase, LamG-like jellyroll fold domain-containing protein |
| inparanoid | 27423 | DDB_G0285513 | asf1 | anti-silencing protein 1 |
| inparanoid | 27432 | DDB_G0277437 | nubp1 | nucleotide binding protein 1 |
| inparanoid | 27445 | DDB_G0285527 | uch2 | peptidase C12 family protein, ubiquitin C-terminal hydrolase |
| inparanoid | 27447 | DDB_G0288393 | DDB_G0288393 |  |
| inparanoid | 27457 | DDB_G0276425 | nutf2 | nuclear transport factor 2 |
| inparanoid | 27471 | DDB_G0267748 | cdc45 | cell division cycle protein 45 |
| inparanoid | 27499 | DDB_G0285605 | DDB_G0285605 |  |
| inparanoid | 27527 | DDB_G0279261 | DDB_G0279261 |  |
| inparanoid | 27567 | DDB_G0277563 | DDB_G0277563 |  |
| inparanoid | 27582 | DDB_G0288799 | mrpl21 | ribosomal protein L21, mitochondrial |
| inparanoid | 27593 | DDB_G0274525 | rad9 | component of 9-1-1 complex |
| inparanoid | 27634 | DDB_G0269448 | DDB_G0269448 | BcDNA:GH04637 protein. |
| inparanoid | 27692 | DDB_G0270516 | DDB_G0270516 |  |
| inparanoid | 27751 | DDB_G0270174 | aprt | adenine phosphoribosyltransferase |
| inparanoid | 27770 | DDB_G0291061 | DDB_G0291061 |  |
| inparanoid | 27789 | DDB_G0270180 | DDB_G0270180 | GLP_38_25174_24752. |
| inparanoid | 27790 | DDB_G0270312 | DDB_G0270312 | GNS1/SUR4 family protein |
| inparanoid | 27799 | DDB_G0270168 | DDB_G0270168 |  |
| inparanoid | 27830 | DDB_G0269880 | DDB_G0269880 | CoA-transferase family III protein, CAIB/BAIF family protein |
| inparanoid | 27831 | DDB_G0269848 | DDB_G0269848 |  |
| inparanoid | 27887 | DDB_G0291149 | DDB_G0291149 | nuclear prelamin A recognition factor-like protein, iron hydrogenase domain-containing protein |
| inparanoid | 27911 | DDB_G0291049 | ost2 | dolichyl-diphosphooligosaccharide-protein glycotransferase, oligosaccharyltransferase epsilon subunit |
| inparanoid | 27918 | DDB_G0269998 | DDB_G0269998 |  |
| inparanoid | 27952 | DDB_G0285107 | DDB_G0285107 |  |
| inparanoid | 28013 | DDB_G0271270 | DDB_G0271270 | beta-lactamase-like domain-containing protein |
| inparanoid | 28038 | DDB_G0284371 | DDB_G0284371 |  |
| inparanoid | 28052 | DDB_G0293148 | DDB_G0293148 |  |
| inparanoid | 28055 | DDB_G0290603 | DDB_G0290603 |  |
| inparanoid | 28071 | DDB_G0279737 | top2mt | DNA topoisomerase II |
| inparanoid | 28078 | DDB_G0293108 | DDB_G0293108 |  |
| inparanoid | 28207 | DDB_G0287327 | prmt5 | protein arginine methyltransferase, Skb1 family protein |
| inparanoid | 28208 | DDB_G0285381 | polD1 | DNA polymerase delta catalytic subunit, DNA polymerase delta subunit 1, DNA polymerase subunit delta p125 |
| inparanoid | 28210 | DDB_G0274169 | DDB_G0274169 | bolA family protein |
| inparanoid | 28222 | DDB_G0278721 | cprD | cysteine protease 4 |
| inparanoid | 28225 | DDB_G0295753 | DDB_G0295753 | mitochondrial substrate carrier family protein, putative mitochondrial phosphate carrier protein |
| inparanoid | 28243 | DDB_G0287361 | ddx41 | DEAD/DEAH box helicase, DEAD box protein abstrakt |
| inparanoid | 28265 | DDB_G0269894 | orcB | origin recognition complex subunit 2 |
| inparanoid | 28273 | DDB_G0283975 | DDB_G0283975 |  |
| inparanoid | 28313 | DDB_G0279359 | ap1s1 | clathrin-adaptor small chain, adapter-related protein complex 1 sigma 1B subunit, sigma adaptin |
| inparanoid | 28319 | DDB_G0283917 | sir2A | NAD(+)-dependent deacetylase, silent information regulator protein (Sir2) family protein, UBP-type zinc finger-containing protein |
| inparanoid | 28339 | DDB_G0272078 | eIF3s10 | proteasome component region PCI (PINT) domain-containing protein, eIF-3 theta, eukaryotic translation initiation factor 3 (eIF3) subunit 10 |
| inparanoid | 28373 | DDB_G0291910 | bcs1lB | mitochondrial chaperone BCS1, mitochondrial ATPase |
| inparanoid | 28422 | DDB_G0272684 | qdpr | dihydropteridine reductase, quinoid dihydropteridine reductase |
| inparanoid | 28467 | DDB_G0271400 | DDB_G0271400 |  |
| inparanoid | 28495 | DDB_G0272066 | DDB_G0272066 | Similar to Dictyostelium discoideum (Slime mold). hypothetical 97.7 kDa protein. |
| inparanoid | 28557 | DDB_G0290497 | DDB_G0290497 |  |
| inparanoid | 28562 | DDB_G0290631 | DDB_G0290631 | pmp22 family protein |
| inparanoid | 28572 | DDB_G0272652 | CYP525A1 | cytochrome P450 family protein |
| inparanoid | 28638 | DDB_G0271562 | orcD | origin recognition complex subunit 4 |
| inparanoid | 28664 | DDB_G0273201 | DDB_G0273201 | DNA-binding HORMA domain-containing protein, mitotic spindle assembly checkpoint protein |
| inparanoid | 28786 | DDB_G0287881 | exoc5 | exocyst complex subunit 5 |
| inparanoid | 28858 | DDB_G0278709 | DDB_G0278709 | At3g59500. |
| inparanoid | 28875 | DDB_G0278713 | DDB_G0278713 | proline synthetase co-transcribed bacterial homolog protein |
| inparanoid | 28879 | DDB_G0270426 | DDB_G0270426 |  |
| inparanoid | 28884 | DDB_G0292610 | DDB_G0292610 |  |
| inparanoid | 28891 | DDB_G0278551 | DDB_G0278551 | CDNA FLJ45739 fis, clone KIDNE2010049, weakly similar to Glycerol kinase 2 (EC 2.7.1.30). |
| inparanoid | 28909 | DDB_G0278711 | nedd8 | neddylin, ubiquitin-like protein Nedd8 |
| inparanoid | 28921 | DDB_G0280173 | isca1 | iron-sulfur cluster assembly 1 homolog |
| inparanoid | 28940 | DDB_G0275073 | pwp1 | WD40 repeat-containing protein, periodic tryptophan protein |
| inparanoid | 28941 | DDB_G0270428 | DDB_G0270428 |  |
| inparanoid | 28946 | DDB_G0280175 | dynD | dynactin 62 kDa subunit, dynactin subunit p62 |
| inparanoid | 28977 | DDB_G0269344 | DDB_G0269344 | WD40 repeat-containing protein |
| inparanoid | 28986 | DDB_G0270020 | sf3a3 | C2H2-type zinc finger-containing protein, splicing factor 3A subunit 3 |
| inparanoid | 29025 | DDB_G0269576 | DDB_G0269576 |  |
| inparanoid | 29047 | DDB_G0280321 | fhkE | putative protein serine/threonine kinase, protein kinase, CAMK group, RAD53 family protein kinase, FHA domain-containing protein |
| inparanoid | 29050 | DDB_G0280261 | DDB_G0280261 | Genomic DNA, chromosome 5, TAC clone:K8K14 (AT5g67320/K8K14_4). |
| inparanoid | 29057 | DDB_G0280247 | DDB_G0280247 |  |
| inparanoid | 29076 | DDB_G0281595 | DDB_G0281595 |  |
| inparanoid | 29091 | DDB_G0285885 | DDB_G0285885 |  |
| inparanoid | 29096 | DDB_G0280271 | DDB_G0280271 |  |
| inparanoid | 29109 | DDB_G0277901 | apm3 | clathrin-adaptor medium chain apm3, ?3, mu3 |
| inparanoid | 29118 | DDB_G0278669 | DDB_G0278669 |  |
| inparanoid | 29119 | DDB_G0279479 | DDB_G0279479 |  |
| inparanoid | 29130 | DDB_G0289999 | DDB_G0289999 |  |
| inparanoid | 29143 | DDB_G0275999 | msh1 | mutS homolog, DNA mismatch repair protein |
| inparanoid | 29164 | DDB_G0285829 | bxdc5 | brix domain-containing protein, ribosome production factor |
| inparanoid | 29220 | DDB_G0285689 | DDB_G0285689 |  |
| inparanoid | 29225 | DDB_G0285417 | cdk7 | p34-cdc2 protein, protein serine/threonine kinase, CDK family protein kinase, cyclin-dependent kinase, CTD kinase, protein kinase, CMGC group |
| inparanoid | 29253 | DDB_G0285943 | DDB_G0285943 | seven transmembrane domain protein, lung seven transmembrane receptor family protein |
| inparanoid | 29263 | DDB_G0285239 | ndufs7 | NADH-ubiquinone oxidoreductase 20 kDa subunit, NADH dehydrogenase (ubiquinone) Fe-S protein 7 |
| inparanoid | 29264 | DDB_G0285871 | DDB_G0285871 | Ran GTPase binding protein, Mog1 family protein, RAN guanine nucleotide release factor |
| inparanoid | 29294 | DDB_G0286941 | DDB_G0286941 |  |
| inparanoid | 29296 | DDB_G0275717 | thoc1 | putative THO1 protein (nuclear matrix protein p84) |
| inparanoid | 29301 | DDB_G0276491 | mettl1 | tRNA (guanine-N7-)-methyltransferase, tRNA(m7G46)-methyltransferase |
| inparanoid | 29312 | DDB_G0275901 | DDB_G0275901 |  |
| inparanoid | 29341 | DDB_G0276087 | DDB_G0276087 | PUTATIVE PHOSPHOLIPASE |
| inparanoid | 29357 | DDB_G0273221 | DDB_G0273221 | unknown |
| inparanoid | 29382 | DDB_G0284383 | DDB_G0284383 | Q9VIF0 UPF0120 protein CG9246. |
| inparanoid | 29412 | DDB_G0284353 | osbI | oxysterol binding family protein, member 9 |
| inparanoid | 29429 | DDB_G0291816 | DDB_G0291816 |  |
| inparanoid | 29435 | DDB_G0291448 | CYP519D1 | cytochrome P450 family protein |
| inparanoid | 29443 | DDB_G0291842 | DDB_G0291842 | putative protein tyrosine kinase, WEE1 family protein kinase |
| inparanoid | 29459 | DDB_G0283403 | DDB_G0283403 |  |
| inparanoid | 29464 | DDB_G0291490 | pigT |  |
| inparanoid | 29471 | DDB_G0269808 | DDB_G0269808 |  |
| inparanoid | 29500 | DDB_G0283399 | DDB_G0283399 |  |
| inparanoid | 29501 | DDB_G0284343 | DDB_G0284343 |  |
| inparanoid | 29503 | DDB_G0291848 | CSN3 | proteasome component region PCI (PINT) domain-containing protein, COP9 signalosome complex subunit 3 |
| inparanoid | 29512 | DDB_G0273247 | DDB_G0273247 |  |
| inparanoid | 29527 | DDB_G0272570 | DDB_G0272570 |  |
| inparanoid | 29534 | DDB_G0272394 | ndufa5 | NADH dehydrogenase (ubiquinone), NADH-ubiquinone oxidoreductase B13 subunit, NADH dehydrogenase [ubiquinone] 1 alpha subcomplex subunit 5 |
| inparanoid | 29548 | DDB_G0278463 | DDB_G0278463 |  |
| inparanoid | 29604 | DDB_G0282119 | DDB_G0282119 |  |
| inparanoid | 29612 | DDB_G0271296 | DDB_G0271296 |  |
| inparanoid | 29618 | DDB_G0271294 | DDB_G0271294 |  |
| inparanoid | 29630 | DDB_G0271298 | rpl27 | S60 ribosomal protein L27 |
| inparanoid | 29684 | DDB_G0280041 | pyr56 | bifunctional UMP-synthetase, uridine 5'-monophosphate synthase, orotate phosphoribosyltransferase (OPRtase), orotidine 5'-phosphate decarboxylase (OMPdecase), UMP synthase |
| inparanoid | 29694 | DDB_G0292012 | DDB_G0292012 |  |
| inparanoid | 29711 | DDB_G0275081 | DDB_G0275081 | 2-AMINOMUCONATE DEAMINASE |
| inparanoid | 29739 | DDB_G0279227 | DDB_G0279227 |  |
| inparanoid | 29786 | DDB_G0283581 | utp15 | WD40 repeat-containing protein, U3 snoRNP protein, U3 small nucleolar ribonucleoprotein |
| inparanoid | 29826 | DDB_G0280251 | DDB_G0280251 |  |
| inparanoid | 29840 | DDB_G0287169 | DDB_G0287169 |  |
| inparanoid | 29882 | DDB_G0269464 | DDB_G0269464 |  |
| inparanoid | 29884 | DDB_G0267512 | DDB_G0267512 | Glycoprotein endopeptidase - like protein. |
| inparanoid | 29910 | DDB_G0286159 | nudc | nuclear distribution protein C homolog, nuclear migration protein nudC |
| inparanoid | 29915 | DDB_G0267508 | DDB_G0267508 | UPF0041 family protein |
| inparanoid | 29916 | DDB_G0284499 | smc2 | structural maintenance of chromosome protein |
| inparanoid | 29919 | DDB_G0278179 | DDB_G0278179 | myb domain-containing protein, DNAJ heat shock N-terminal domain-containing protein |
| inparanoid | 29956 | DDB_G0269024 | ate1 | arginyltransferase |
| inparanoid | 30022 | DDB_G0267982 | copA | WD40 repeat-containing protein, coatomer protein complex alpha subunit |
| inparanoid | 30027 | DDB_G0267622 | srp9 | signal recognition particle 9 kDa subunit |
| inparanoid | 30030 | DDB_G0291716 | DDB_G0291716 |  |
| inparanoid | 30075 | DDB_G0276631 | DDB_G0276631 |  |
| inparanoid | 30076 | DDB_G0276359 | sec11 | microsomal signal peptidase subunit |
| inparanoid | 30114 | DDB_G0286909 | anapc11 | anaphase promoting complex subunit 11 |
| inparanoid | 30157 | DDB_G0280405 | DDB_G0280405 |  |
| inparanoid | 30169 | DDB_G0280799 | DDB_G0280799 |  |
| inparanoid | 30175 | DDB_G0280123 | DDB_G0280123 |  |
| inparanoid | 30182 | DDB_G0275583 | DDB_G0275583 | phosphatidylethanolamine-binding protein PEBP |
| inparanoid | 30205 | DDB_G0289149 | litaf | lipopolysaccharide-induced TNF factor homolog |
| inparanoid | 30237 | DDB_G0283291 | DDB_G0283291 | Probable iron/ascorbate oxidoreductase. |
| inparanoid | 30238 | DDB_G0283347 | DDB_G0283347 |  |
| inparanoid | 30259 | DDB_G0283273 | DDB_G0283273 |  |
| inparanoid | 30280 | DDB_G0283801 | fam96B | DUF59 family protein, FAM96 family protein |
| inparanoid | 30303 | DDB_G0279695 | DDB_G0279695 | RNA recognition motif-containing protein RRM |
| inparanoid | 30308 | DDB_G0282579 | pldZ | phospholipase D3 |
| inparanoid | 30367 | DDB_G0279715 | mtyrS | tyrosine-tRNA ligase, tyrosyl-tRNA synthetase, putative mitochondrial tyrosyl-tRNA synthetase, putative mitochondrial tyrosine-tRNA ligase |
| inparanoid | 30371 | DDB_G0278189 | ints11 | integrator complex subunit 11, beta-lactamase-like domain-containing protein, RNA-metabolising metallo-beta-lactamase domain-containing protein |
| inparanoid | 30387 | DDB_G0277971 | DDB_G0277971 | vacuolar ATP synthase subunit G |
| inparanoid | 30399 | DDB_G0278039 | rpb8 | RNA polymerase II core subunit, RNA polymerase I core subunit, RNA polymerase III core subunit |
| inparanoid | 30479 | DDB_G0275249 | commd10 | COMM domain-containing protein 10 |
| inparanoid | 30483 | DDB_G0288385 | DDB_G0288385 |  |
| inparanoid | 30493 | DDB_G0286507 | DDB_G0286507 | RNA-binding region RNP-1 domain-containing protein, RNA recognition motif-containing protein RRM, putative acyl-CoA thioester hydrolase |
| inparanoid | 30502 | DDB_G0286519 | dgtB | glycosyltransferase, dolichol-phosphate-mannose synthase |
| inparanoid | 30504 | DDB_G0282233 | DDB_G0282233 | PV1H14130_P. |
| inparanoid | 30528 | DDB_G0287033 | nagA | beta-N-acetylhexosaminidase, glycoside hydrolase family 20 protein, beta-hexosaminidase |
| inparanoid | 30555 | DDB_G0290089 | DDB_G0290089 |  |
| inparanoid | 30610 | DDB_G0287761 | DDB_G0287761 |  |
| inparanoid | 30626 | DDB_G0278369 | DDB_G0278369 |  |
| inparanoid | 30635 | DDB_G0278063 | DDB_G0278063 | peptidase S16, Lon protease family protein |
| inparanoid | 30670 | DDB_G0277891 | rimA | mitochondrial substrate carrier family protein, putative mitochondrial pyrimidine nucleotide transporter |
| inparanoid | 30729 | DDB_G0271384 | DDB_G0271384 |  |
| inparanoid | 30730 | DDB_G0269546 | DDB_G0269546 | C2 calcium-dependent membrane targeting domain-containing protein |
| inparanoid | 30756 | DDB_G0281153 | eIF3s3 | Mov34/MPN/PAD-1 family protein, eukaryotic translation initiation factor 3 (eIF3) subunit 3, eIF-3 gamma |
| inparanoid | 30759 | DDB_G0280919 | DDB_G0280919 |  |
| inparanoid | 30768 | DDB_G0281155 | DDB_G0281155 | sugar transporter family protein |
| inparanoid | 30795 | DDB_G0280947 | DDB_G0280947 | UPF0171 family protein |
| inparanoid | 30810 | DDB_G0268036 | DDB_G0268036 | 2,3-diketo-5-methylthio-1-phosphopentane enolase |
| inparanoid | 30821 | DDB_G0275979 | thoc7 | unknown |
| inparanoid | 30837 | DDB_G0291566 | grwd1 | glutamate-rich WD repeat-containing protein 1 |
| inparanoid | 30843 | DDB_G0268032 | DDB_G0268032 |  |
| inparanoid | 30850 | DDB_G0285027 | alrD | aldo-keto reductase |
| inparanoid | 30876 | DDB_G0293588 | ggps1 | geranylgeranyl diphosphate synthase 1 |
| inparanoid | 30895 | DDB_G0293828 | DDB_G0293828 |  |
| inparanoid | 30899 | DDB_G0293840 | DDB_G0293840 | putative glutathione S-transferase, putative glutathione transferase |
| inparanoid | 30919 | DDB_G0293590 | DDB_G0293590 | NF-X1-type zinc finger-containing protein |
| inparanoid | 30952 | DDB_G0291235 | ranA | GTP-binding nuclear protein Ran |
| inparanoid | 30992 | DDB_G0277107 | lsm3 | LSM (like-Sm) domain-containing protein, putative U6 small nuclear ribonucleoparticle-associated protein |
| inparanoid | 31008 | DDB_G0276335 | pyr1-3 | dihydroorotase, aspartate carbamoyltransferase, glutamine-dependent carbamoyl-phosphate synthase |
| inparanoid | 31033 | DDB_G0276331 | pyr4 | dihydroorotate oxidase, dihydroorotate dehydrogenase |
| inparanoid | 31066 | DDB_G0285133 | mocs2s | molybdenum cofactor synthesis protein 2 small subunit |
| inparanoid | 31087 | DDB_G0285137 | mocs1 | molybdenum cofactor synthesis 1 |
| inparanoid | 31095 | DDB_G0284267 | eIF2a | eukaryotic translation initiation factor 2A |
| inparanoid | 31114 | DDB_G0285131 | derl2 | derlin-2 |
| inparanoid | 31119 | DDB_G0285143 | DDB_G0285143 | unknown |
| inparanoid | 31125 | DDB_G0292686 | dus3l | CCCH-type zinc finger-containing protein, tRNA-dihydrouridine synthase 3-like protein |
| inparanoid | 31131 | DDB_G0292690 | trappc2l | trafficking protein particle complex subunit 2-like protein |
| inparanoid | 31135 | DDB_G0279801 | pex7 | WD40 repeat-containing protein, peroxisomal biogenesis factor 7, peroxin 7, peroxisome targeting signal type 2 receptor |
| inparanoid | 31145 | DDB_G0292958 | mcm5 | MCM family protein, putative DNA replication licensing factor |
| inparanoid | 31155 | DDB_G0292692 | DDB_G0292692 |  |
| inparanoid | 31172 | DDB_G0279769 | psmG1 | proteasome assembly chaperone 1 |
| inparanoid | 31198 | DDB_G0293228 | gtf2h4 | general transcription factor IIH, polypeptide 4, TFIIH subunit |
| inparanoid | 31218 | DDB_G0291277 | DDB_G0291277 |  |
| inparanoid | 31219 | DDB_G0293230 | cdc26 | subunit of anaphase promoting complex |
| inparanoid | 31231 | DDB_G0281233 | DDB_G0281233 | cellulose-binding domain-containing protein |
| inparanoid | 31241 | DDB_G0292428 | DDB_G0292428 |  |
| inparanoid | 31265 | DDB_G0289135 | bcs1lA | mitochondrial chaperone BCS1, mitochondrial ATPase |
| inparanoid | 31268 | DDB_G0288977 | DDB_G0288977 | RasGTPase-activating protein, IQGAP-related protein |
| inparanoid | 31269 | DDB_G0289037 | ufc1 | ubiquitin-like (UBL) post-translational modifier |
| inparanoid | 31270 | DDB_G0289125 | DDB_G0289125 |  |
| inparanoid | 31276 | DDB_G0289079 | DDB_G0289079 |  |
| inparanoid | 31325 | DDB_G0284911 | timm10 | mitochondrial import inner membrane translocase subunit 10 |
| inparanoid | 31349 | DDB_G0276411 | mrt4 | ribosomal protein L10 family protein, mRNA turnover protein 4 |
| inparanoid | 31355 | DDB_G0284987 | repG | XPG, flap structure specific endonuclease 1 |
| inparanoid | 31376 | DDB_G0275911 | DDB_G0275911 |  |
| inparanoid | 31382 | DDB_G0279995 | DDB_G0279995 | putative glycoside hydrolase |
| inparanoid | 31392 | DDB_G0280399 | tmem93 | transmembrane protein, DUF786 family protein |
| inparanoid | 31417 | DDB_G0282955 | DDB_G0282955 |  |
| inparanoid | 31424 | DDB_G0279763 | CYP513D1 | cytochrome P450 family protein |
| inparanoid | 31439 | DDB_G0273435 | DDB_G0273435 |  |
| inparanoid | 31443 | DDB_G0268098 | DDB_G0268098 | MAR-binding protein. |
| inparanoid | 31483 | DDB_G0281435 | mrpl17 | ribosomal protein L17, mitochondrial |
| inparanoid | 31489 | DDB_G0279635 | DDB_G0279635 |  |
| inparanoid | 31545 | DDB_G0274795 | DDB_G0274795 | ubiquitin-specific protease 48-like protein, peptidase C19 family protein |
| inparanoid | 31574 | DDB_G0281787 | gpn2 | GPN-loop GTPase 2 |
| inparanoid | 31594 | DDB_G0281559 | DG1080 | SNF2-related domain-containing protein, CHR group protein, helicase, C-terminal domain-containing protein |
| inparanoid | 31606 | DDB_G0278793 | DDB_G0278793 |  |
| inparanoid | 31630 | DDB_G0280453 | DDB_G0280453 |  |
| inparanoid | 31641 | DDB_G0280451 | DDB_G0280451 | galactose-binding domain-containing protein |
| inparanoid | 31643 | DDB_G0280363 | DDB_G0280363 |  |
| inparanoid | 31644 | DDB_G0280449 | pitC | phosphatidylinositol transfer protein |
| inparanoid | 31657 | DDB_G0277151 | DDB_G0277151 |  |
| inparanoid | 31743 | DDB_G0272236 | aspS1 | aspartyl-tRNA synthetase, aspartate-tRNA ligase |
| inparanoid | 31754 | DDB_G0272398 | DDB_G0272398 |  |
| inparanoid | 31764 | DDB_G0272378 | DDB_G0272378 | Similar to Oryza sativa (Japonica cultivar-group). P0446G04.23 protein (Fragment). |
| inparanoid | 31794 | DDB_G0287307 | gtf2e2 | transcription initiation factor IIE2, transcription initiation factor IIE subunit beta |
| inparanoid | 31848 | DDB_G0287315 | DDB_G0287315 |  |
| inparanoid | 31882 | DDB_G0286197 | DDB_G0286197 |  |
| inparanoid | 31883 | DDB_G0288363 | DDB_G0288363 |  |
| inparanoid | 31892 | DDB_G0286535 | gghA | peptidase C26 family protein, gamma-glutamyl hydrolase |
| inparanoid | 31893 | DDB_G0269248 | cf45-1 | component of the counting factor (CF) complex |
| inparanoid | 31920 | DDB_G0289285 | DDB_G0289285 |  |
| inparanoid | 31942 | DDB_G0293052 | eIF3s6 | proteasome component region PCI (PINT) domain-containing protein, eukaryotic translation initiation factor 3 (eIF3) subunit 6, eIF-3 p48 |
| inparanoid | 31945 | DDB_G0287099 | DDB_G0287099 | phosphoglycerate/bisphosphoglycerate mutase family protein |
| inparanoid | 31947 | DDB_G0293062 | DDB_G0293062 |  |
| inparanoid | 31953 | DDB_G0293296 | DDB_G0293296 |  |
| inparanoid | 31960 | DDB_G0291632 | DDB_G0291632 |  |
| inparanoid | 31990 | DDB_G0291994 | abcA1 | ABC transporter A family protein |
| inparanoid | 32017 | DDB_G0291554 | DDB_G0291554 |  |
| inparanoid | 32061 | DDB_G0281259 | DDB_G0281259 | putative DREV methyltransferase |
| inparanoid | 32062 | DDB_G0282267 | DDB_G0282267 | FKBP-type peptidylprolyl cis-trans isomerase (PPIase) |
| inparanoid | 32083 | DDB_G0289557 | DDB_G0289557 |  |
| inparanoid | 32126 | DDB_G0287969 | cnrN | phosphatase tensin type domain-containing protein, C2 tensin-type domain-containing protein, putative cell number regulator |
| inparanoid | 32138 | DDB_G0287513 | jcdG | transcription factor jumonji, jmjC domain-containing protein, cupin region-containing protein |
| inparanoid | 32147 | DDB_G0287515 | tat | tyrosine transaminase, tyrosine aminotransferase |
| inparanoid | 32183 | DDB_G0287603 | DDB_G0287603 | unknown |
| inparanoid | 32199 | DDB_G0287939 | nit2 | nitrilase 2 |
| inparanoid | 32201 | DDB_G0287607 | pcna | proliferating cell nuclear antigen |
| inparanoid | 32209 | DDB_G0292468 | DDB_G0292468 | F54C9.9 protein. |
| inparanoid | 32241 | DDB_G0292466 | nol10 | NUC153 domain-containing protein |
| inparanoid | 32286 | DDB_G0270754 | vps16 |  |
| inparanoid | 32295 | DDB_G0270304 | DDB_G0270304 |  |
| inparanoid | 32303 | DDB_G0278547 | DDB_G0278547 |  |
| inparanoid | 32306 | DDB_G0284067 | clybl | citrate lyase subunit beta-like protein |
| inparanoid | 32310 | DDB_G0279089 | DDB_G0279089 |  |
| inparanoid | 32345 | DDB_G0277003 | DDB_G0277003 |  |
| inparanoid | 32350 | DDB_G0275667 | DDB_G0275667 | Similar to similarity to S. cerevisiae kti12 protein. |
| inparanoid | 32359 | DDB_G0284905 | ap4s1 | adapter-related protein complex 4 sigma 1 subunit, clathrin-adaptor small chain, sigma adaptin |
| inparanoid | 32378 | DDB_G0274607 | gefM | Ras guanine nucleotide exchange factor |
| inparanoid | 32387 | DDB_G0276903 | DDB_G0276903 |  |
| inparanoid | 32417 | DDB_G0277669 | DDB_G0277669 | aldose 1-epimerase family protein |
| inparanoid | 32433 | DDB_G0288157 | DDB_G0288157 |  |
| inparanoid | 32449 | DDB_G0290127 | DDB_G0290127 | WD40 repeat-containing protein, cyclin-like F-box containing protein |
| inparanoid | 32454 | DDB_G0290213 | vps45 | Sec1-like family protein |
| inparanoid | 32466 | DDB_G0287857 | DDB_G0287857 |  |
| inparanoid | 32479 | DDB_G0287241 | DDB_G0287241 |  |
| inparanoid | 32483 | DDB_G0287261 | gphn | gephyrin |
| inparanoid | 32523 | DDB_G0283333 | ucpB | mitochondrial substrate carrier family protein |
| inparanoid | 32553 | DDB_G0283365 | rpb12 | RNA polymerase, 7 kDa subunit, RNA polymerase II core subunit, RNA polymerase I core subunit, RNA polymerase III core subunit |
| inparanoid | 32594 | DDB_G0287009 | mcfC | EF-hand domain-containing protein, mitochondrial substrate carrier family protein, calcium-dependent mitochondrial substrate carrier |
| inparanoid | 32596 | DDB_G0287067 | DDB_G0287067 |  |
| inparanoid | 32613 | DDB_G0275343 | DDB_G0275343 |  |
| inparanoid | 32620 | DDB_G0276853 | DDB_G0276853 |  |
| inparanoid | 32622 | DDB_G0280129 | DDB_G0280129 |  |
| inparanoid | 32636 | DDB_G0276847 | DDB_G0276847 |  |
| inparanoid | 32637 | DDB_G0280127 | map1d | methionine aminopeptidase |
| inparanoid | 32656 | DDB_G0277169 | DDB_G0277169 |  |
| inparanoid | 32669 | DDB_G0279321 | DDB_G0279321 |  |
| inparanoid | 32675 | DDB_G0290795 | hddc2 | HD domain-containing protein 2 |
| inparanoid | 32706 | DDB_G0286077 | commd6 | COMM domain-containing protein 6 |
| inparanoid | 32729 | DDB_G0267708 | vps25 | vacuolar protein sorting 25 |
| inparanoid | 32742 | DDB_G0270708 | DDB_G0270708 | Similar to Dictyostelium discoideum (Slime mold). hypothetical 127.0 kDa protein. |
| inparanoid | 32760 | DDB_G0290027 | DDB_G0290027 | bolA family protein |
| inparanoid | 32762 | DDB_G0284481 | DDB_G0284481 | AgCP8363 (Fragment). |
| inparanoid | 32773 | DDB_G0290031 | DDB_G0290031 | O95478 TGF beta-inducible nuclear protein 1 (Hairy cell leukemia protein 1) (HUSSY-29). |
| inparanoid | 32822 | DDB_G0272074 | DDB_G0272074 |  |
| inparanoid | 32831 | DDB_G0291362 | rpc19 | putative RNA polymerase III subunit, putative RNA polymerase I subunit |
| inparanoid | 32847 | DDB_G0278607 | mvd | diphosphomevalonate decarboxylase, mevalonate 5-diphosphate decarboxylase |
| inparanoid | 32869 | DDB_G0276529 | DDB_G0276529 | Similar to Dictyostelium discoideum (Slime mold). Non-receptor tyrosine kinase spore lysis A (EC 2.7.1.112) (Tyrosine-protein kinase 1). |
| inparanoid | 32915 | DDB_G0279909 | lipA | putative arachidonate 12-lipoxygenase, lipoxygenase |
| inparanoid | 32959 | DDB_G0293742 | rps2 | ribosomal protein S2 |
| inparanoid | 32960 | DDB_G0293946 | DDB_G0293946 | DUF339 family protein |
| inparanoid | 32967 | DDB_G0281585 | cpsf1 | CPSF domain-containing protein, cleavage and polyadenylation specificity factor 160 kDa subunit, WD40-like domain-containing protein |
| inparanoid | 33009 | DDB_G0302418 | imp4 | U3 small nucleolar ribonucleoprotein protein |
| inparanoid | 33056 | DDB_G0278901 | DDB_G0278901 | putative protein serine/threonine kinase, protein kinase, STE group |
| inparanoid | 33071 | DDB_G0292436 | triA | BTB/POZ domain-containing protein |
| inparanoid | 33074 | DDB_G0292920 | DDB_G0292920 | phosphatase tensin type domain-containing protein, C2 tensin-type domain-containing protein |
| inparanoid | 33087 | DDB_G0279797 | ctnB | countin2 |
| inparanoid | 33096 | DDB_G0292928 | DDB_G0292928 |  |
| inparanoid | 33147 | DDB_G0285197 | gpn3 | GPN-loop GTPase 3 |
| inparanoid | 33196 | DDB_G0278889 | mrpl27 | ribosomal protein L27, mitochondrial |
| inparanoid | 33197 | DDB_G0278887 | prafA | PRA1 family protein 1 |
| inparanoid | 33256 | DDB_G0269548 | DDB_G0269548 |  |
| inparanoid | 33259 | DDB_G0294553 | DDB_G0294553 | delta 5 fatty acid desaturase |
| inparanoid | 33261 | DDB_G0269454 | trpS | tryptophanyl-tRNA synthetase, tryptophan-tRNA ligase |
| inparanoid | 33274 | DDB_G0273089 | coq10-1 | putative coenzyme Q-binding protein |
| inparanoid | 33276 | DDB_G0274893 | DDB_G0274893 | unknown |
| inparanoid | 33308 | DDB_G0273253 | DDB_G0273253 | SET domain-containing protein |
| inparanoid | 33321 | DDB_G0291810 | DDB_G0291810 |  |
| inparanoid | 33329 | DDB_G0271730 | DDB_G0271730 |  |
| inparanoid | 33357 | DDB_G0293758 | H2Bv1 | histone H2B domain-containing protein |
| inparanoid | 33388 | DDB_G0281099 | DDB_G0281099 | TMS membrane protein/tumour differentially expressed (TDE) family protein |
| inparanoid | 33395 | DDB_G0280431 | rio1 | putative protein serine/threonine kinase, protein kinase, Atypical group, RIO family protein kinase, RIO1 subfamily protein kinase |
| inparanoid | 33426 | DDB_G0283113 | eriA | putative RNase III, RNA exonuclease |
| inparanoid | 33450 | DDB_G0282937 | DDB_G0282937 | unknown |
| inparanoid | 33473 | DDB_G0281843 | DDB_G0281843 |  |
| inparanoid | 33484 | DDB_G0281557 | drkD | leucine-rich repeat-containing protein (LRR), protein kinase, TKL group, tyrosine kinase-like protein, DRK subfamily protein kinase |
| inparanoid | 33488 | DDB_G0278937 | DDB_G0278937 | Mitochondrial RNA helicase (At4g14790). |
| inparanoid | 33519 | DDB_G0277045 | DDB_G0277045 |  |
| inparanoid | 33526 | DDB_G0291442 | DDB_G0291442 |  |
| inparanoid | 33545 | DDB_G0269920 | DDB_G0269920 |  |
| inparanoid | 33571 | DDB_G0284915 | DDB_G0284915 | class II aldolase/adducin, N-terminal domain-containing protein |
| inparanoid | 33578 | DDB_G0284913 | DDB_G0284913 |  |
| inparanoid | 33583 | DDB_G0285013 | DDB_G0285013 |  |
| inparanoid | 33592 | DDB_G0276563 | DDB_G0276563 | Similar to LIM domains. |
| inparanoid | 33594 | DDB_G0285055 | DDB_G0285055 |  |
| inparanoid | 33630 | DDB_G0288131 | cmbB | calmodulin-binding protein, FNIP repeat-containing protein |
| inparanoid | 33638 | DDB_G0286241 | aifD | putative apoptosis inducing factor |
| inparanoid | 33651 | DDB_G0284951 | vamp7A | vesicle-associated membrane protein 7, synaptobrevin domain-containing protein, longin domain-containing protein, v-SNARE family protein |
| inparanoid | 33662 | DDB_G0287027 | DDB_G0287027 |  |
| inparanoid | 33672 | DDB_G0287015 | DDB_G0287015 | TM2 domain-containing protein |
| inparanoid | 33680 | DDB_G0286043 | DDB_G0286043 |  |
| inparanoid | 33709 | DDB_G0281283 | DDB_G0281283 |  |
| inparanoid | 33711 | DDB_G0281923 | DDB_G0281923 |  |
| inparanoid | 33721 | DDB_G0282305 | DDB_G0282305 | Gp63 homolog. |
| inparanoid | 33767 | DDB_G0281059 | DDB_G0281059 | methyltransferase type 12 domain-containing protein |
| inparanoid | 33770 | DDB_G0281331 | DDB_G0281331 | putative protein serine/threonine kinase, putative transmembrane protein |
| inparanoid | 33776 | DDB_G0271066 | DDB_G0271066 |  |
| inparanoid | 33780 | DDB_G0289993 | DDB_G0289993 | acyl-CoA oxidase |
| inparanoid | 33784 | DDB_G0291063 | thoc2 | putative THO2 protein |
| inparanoid | 33829 | DDB_G0270062 | DDB_G0270062 |  |
| inparanoid | 33852 | DDB_G0281859 | DDB_G0281859 |  |
| inparanoid | 33855 | DDB_G0271628 | dlpC | dynamin like protein |
| inparanoid | 33863 | DDB_G0267536 | hibch | 3-hydroxyisobutyryl-Coenzyme A hydrolase |
| inparanoid | 33881 | DDB_G0267656 | DDB_G0267656 | Similar to hypothetical protein FLJ12618. |
| inparanoid | 33883 | DDB_G0267952 | DDB_G0267952 |  |
| inparanoid | 33907 | DDB_G0267594 | pfdn2 | prefoldin beta-like domain containing protein, prefoldin subunit 2 |
| inparanoid | 33913 | DDB_G0287757 | DDB_G0287757 | impact protein homolog |
| inparanoid | 33930 | DDB_G0281495 | DDB_G0281495 |  |
| inparanoid | 33948 | DDB_G0284145 | DDB_G0284145 | regulator of chromosome condensation (RCC1) domain-containing protein |
| inparanoid | 33962 | DDB_G0285377 | DDB_G0285377 | Q9Y3B2 3'-5' exoribonuclease CSL4 homolog (EC 3.1.13.-) (Exosome component 1) (CGI-108). |
| inparanoid | 33968 | DDB_G0284713 | polB | DNA polymerase beta, DNA polymerase X family protein |
| inparanoid | 33979 | DDB_G0285375 | atg18 | autophagy protein 18, WD repeat domain phosphoinositide-interacting family protein |
| inparanoid | 33983 | DDB_G0284705 | DDB_G0284705 | unknown |
| inparanoid | 33993 | DDB_G0276725 | DDB_G0276725 |  |
| inparanoid | 33997 | DDB_G0285379 | DDB_G0285379 |  |
| inparanoid | 34025 | DDB_G0288331 | DDB_G0288331 | putative arabinofuranohydrolase |
| inparanoid | 34030 | DDB_G0288333 | purB | adenylosuccinate lyase |
| inparanoid | 34067 | DDB_G0269290 | nap1 | putative nucleosome assembly protein |
| inparanoid | 34073 | DDB_G0269282 | DDB_G0269282 | Heat shock protein HslV (EC 3.4.99.-). |
| inparanoid | 34083 | DDB_G0269330 | DDB_G0269330 |  |
| inparanoid | 34085 | DDB_G0268824 | DDB_G0268824 |  |
| inparanoid | 34221 | DDB_G0291828 | DDB_G0291828 |  |
| inparanoid | 34242 | DDB_G0291344 | DDB_G0291344 | putative cholinesterase, carboxylesterase, type B family protein |
| inparanoid | 34246 | DDB_G0291834 | cpiA | cystatin A1, cysteine protease inhibitor |
| inparanoid | 34248 | DDB_G0291340 | DDB_G0291340 |  |
| inparanoid | 34262 | DDB_G0279137 | DDB_G0279137 | methylenetetrahydrofolate reductase, MTHFR |
| inparanoid | 34288 | DDB_G0291408 | DDB_G0291408 |  |
| inparanoid | 34292 | DDB_G0278785 | DDB_G0278785 |  |
| inparanoid | 34297 | DDB_G0278979 | DDB_G0278979 |  |
| inparanoid | 34300 | DDB_G0278675 | DDB_G0278675 |  |
| inparanoid | 34318 | DDB_G0269378 | rad1 | exonuclease, checkpoint clamp complex protein |
| inparanoid | 34335 | DDB_G0269376 | DDB_G0269376 |  |
| inparanoid | 34340 | DDB_G0269570 | rabggta | protein geranylgeranyltransferase type II, Rab geranylgeranyltransferase alpha subunit |
| inparanoid | 34376 | DDB_G0286035 | mcm9 | MCM family protein |
| inparanoid | 34383 | DDB_G0269062 | DDB_G0269062 | DUF974 family protein |
| inparanoid | 34436 | DDB_G0280371 | DDB_G0280371 | Q827G1 Glycerol kinase 2 (EC 2.7.1.30) (ATP:glycerol 3-phosphotransferase 2) (Glycerokinase 2) (GK 2). |
| inparanoid | 34471 | DDB_G0290183 | DDB_G0290183 |  |
| inparanoid | 34493 | DDB_G0289995 | DDB_G0289995 |  |
| inparanoid | 34496 | DDB_G0291083 | DDB_G0291083 |  |
| inparanoid | 34522 | DDB_G0281541 | DDB_G0281541 | CG11323 protein. |
| inparanoid | 34531 | DDB_G0277751 | DDB_G0277751 | Similar to Mus musculus (Mouse). DNA polymerase theta short isoform. |
| inparanoid | 34584 | DDB_G0293276 | DDB_G0293276 | putative protein serine/threonine kinase, protein kinase, AGC group, MAST family protein kinase |
| inparanoid | 34586 | DDB_G0282421 | DDB_G0282421 |  |
| inparanoid | 34601 | DDB_G0280983 | crlA | cAMP receptor-like protein, G-protein-coupled receptor (GPCR) family protein |
| inparanoid | 34623 | DDB_G0280607 | pfdn5 | prefoldin subunit 5, prefoldin alpha-like domain containing protein |
| inparanoid | 34643 | DDB_G0292048 | DDB_G0292048 |  |
| inparanoid | 34644 | DDB_G0292046 | DDB_G0292046 |  |
| inparanoid | 34673 | DDB_G0292806 | cluA | 150 kDa protein |
| inparanoid | 34679 | DDB_G0278985 | DDB_G0278985 |  |
| inparanoid | 34685 | DDB_G0281639 | DDB_G0281639 | guanylate-binding protein, GTP-binding protein |
| inparanoid | 34686 | DDB_G0292906 | DDB_G0292906 |  |
| inparanoid | 34691 | DDB_G0292898 | pgl | 6-phosphogluconolactonase |
| inparanoid | 34701 | DDB_G0295485 | DDB_G0295485 | EGF-like domain-containing protein |
| inparanoid | 34705 | DDB_G0278989 | DDB_G0278989 |  |
| inparanoid | 34708 | DDB_G0278983 | imp3 | U3 small nucleolar ribonucleoprotein protein |
| inparanoid | 34712 | DDB_G0292904 | dync1li1 | dynein light intermediate chain |
| inparanoid | 34727 | DDB_G0280401 | cypD | cyclophilin D |
| inparanoid | 34742 | DDB_G0280419 | DDB_G0280419 |  |
| inparanoid | 34747 | DDB_G0275353 | DDB_G0275353 |  |
| inparanoid | 34761 | DDB_G0275405 | ap3s1 | sigma adaptin |
| inparanoid | 34779 | DDB_G0293546 | mre11 | DNA repair exonuclease, 3'-5' exonuclease |
| inparanoid | 34783 | DDB_G0293702 | rfc3 | replication factor C subunit |
| inparanoid | 34820 | DDB_G0279445 | ctns | cystinosin |
| inparanoid | 34837 | DDB_G0278331 | DDB_G0278331 | FLJ10534 protein (Fragment). |
| inparanoid | 34840 | DDB_G0278259 | manB | alpha-mannosidase |
| inparanoid | 34848 | DDB_G0278925 | DDB_G0278925 |  |
| inparanoid | 34855 | DDB_G0283887 | DDB_G0283887 | unknown |
| inparanoid | 34883 | DDB_G0283877 | dus2l | tRNA-dihydrouridine synthase 2-like protein |
| inparanoid | 34890 | DDB_G0283573 | DDB_G0283573 |  |
| inparanoid | 34933 | DDB_G0276243 | DDB_G0276243 | Similar to Arabidopsis thaliana (Mouse-ear cress). Hypothetical 105.9 kDa protein. |
| inparanoid | 34947 | DDB_G0281353 | DDB_G0281353 |  |
| inparanoid | 34975 | DDB_G0289675 | adprh | ADP-ribosylarginine hydrolase |
| inparanoid | 35014 | DDB_G0267898 | DDB_G0267898 | EGF-like domain-containing protein |
| inparanoid | 35027 | DDB_G0286639 | DDB_G0286639 |  |
| inparanoid | 35069 | DDB_G0290851 | spc3 | microsomal signal peptidase subunit, signal peptidase complex subunit 3 |
| inparanoid | 35082 | DDB_G0290869 | jcdF | transcription factor jumonji, jmjC domain-containing protein |
| inparanoid | 35083 | DDB_G0290605 | DDB_G0290605 |  |
| inparanoid | 35109 | DDB_G0283799 | DDB_G0283799 |  |
| inparanoid | 35121 | DDB_G0284165 | DDB_G0284165 | ras association (RA) domain-containing protein |
| inparanoid | 35158 | DDB_G0274251 | DDB_G0274251 | ABL-PHILIN 2 |
| inparanoid | 35164 | DDB_G0274379 | DDB_G0274379 |  |
| inparanoid | 35169 | DDB_G0274545 | thrS2 | threonyl-tRNA synthetase, threonine-tRNA ligase |
| inparanoid | 35206 | DDB_G0281939 | DDB_G0281939 |  |
| inparanoid | 35212 | DDB_G0271962 | DDB_G0271962 | HYPOTHETICAL 54.5 KDA PROTEIN C1685.06 IN CHROMOSOME II. 6/101 |
| inparanoid | 35223 | DDB_G0290085 | DDB_G0290085 |  |
| inparanoid | 35229 | DDB_G0287937 | bxdc2 | brix domain-containing protein |
| inparanoid | 35231 | DDB_G0302414 | snrpE | small nuclear ribonucleoprotein E |
| inparanoid | 35249 | DDB_G0288321 | dgtA | glycosyltransferase, dolichyl phosphate glucosyltransferase, dolichyl-phosphate-glucose synthase |
| inparanoid | 35300 | DDB_G0277955 | nup43 | WD40 repeat-containing protein, nucleoporin 43 |
| inparanoid | 35306 | DDB_G0278195 | DDB_G0278195 |  |
| inparanoid | 35395 | DDB_G0281971 | DDB_G0281971 |  |
| inparanoid | 35400 | DDB_G0281933 | nola4 | pseudouridine synthase family protein, DKCLD domain-containing protein, PUA domain-containing protein, H/ACA RNP complex subunit 4 |
| inparanoid | 35425 | DDB_G0285887 | DDB_G0285887 |  |
| inparanoid | 35486 | DDB_G0275647 | DDB_G0275647 | cytochrome b5 domain-containing protein |
| inparanoid | 35506 | DDB_G0281175 | DDB_G0281175 | tRNA(m1A58)-methyltransferase subunit, tRNA (adenine-N(1)-)-methyltransferase non-catalytic subunit |
| inparanoid | 35514 | DDB_G0275749 | DDB_G0275749 |  |
| inparanoid | 35527 | DDB_G0275753 | psmD9 | 26S proteasome non-ATPase regulatory subunit 9, 26S proteasome regulatory subunit p27, PDZ domain-containing protein |
| inparanoid | 35564 | DDB_G0280959 | DG1113 | C2 domain-containing protein |
| inparanoid | 35593 | DDB_G0293606 | DDB_G0293606 | cellular retinaldehyde-binding/triple function domain-containing protein, cellular retinaldehyde binding/alpha-tocopherol transport family protein |
| inparanoid | 35595 | DDB_G0276151 | DDB_G0276151 | Similar to Mycobacterium tuberculosis. Putative enoyl CoA-hydratase (Enoyl-CoA hydratase/isomerase family protein). |
| inparanoid | 35633 | DDB_G0273191 | rps30-1 | 40S ribosomal protein S30 |
| inparanoid | 35644 | DDB_G0273789 | DDB_G0273789 | Dyp-type peroxidase family protein |
| inparanoid | 35656 | DDB_G0285503 | dynB | dynactin 50 kDa subunit, dynamitin |
| inparanoid | 35659 | DDB_G0274521 | DDB_G0274521 | Similar to Mus musculus (Mouse). ribosome biogenesis regulatory protein homolog. |
| inparanoid | 35824 | DDB_G0288701 | ragA | Ras-related GTP-binding protein |
| inparanoid | 35825 | DDB_G0287749 | DDB_G0287749 |  |
| inparanoid | 35831 | DDB_G0283251 | DDB_G0283251 |  |
| inparanoid | 35834 | DDB_G0283187 | DDB_G0283187 | putative protein serine/threonine phosphatase, protein phosphatase |
| inparanoid | 35883 | DDB_G0293376 | cpras2 | Ras GTPase domain-containing protein |
| inparanoid | 35893 | DDB_G0292774 | DDB_G0292774 |  |
| inparanoid | 35909 | DDB_G0289743 | DDB_G0289743 |  |
| inparanoid | 35924 | DDB_G0288957 | DDB_G0288957 | Similar to Homo sapiens (Human). Tenascin (TN) (Hexabrachion) (Cytotactin) (Neuronectin) (GMEM) (JI) (Miotendinous antigen) (Glioma-associated-extracellular matrix antigen) (GP 150-225) (Tenascin-C) (TN-C). |
| inparanoid | 35934 | DDB_G0289069 | arrD | ADP-ribosylation factor-related, ARF-related |
| inparanoid | 35949 | DDB_G0283533 | cbpB | calcium-binding protein |
| inparanoid | 35950 | DDB_G0285685 | DDB_G0285685 |  |
| inparanoid | 35954 | DDB_G0283557 | DDB_G0283557 |  |
| inparanoid | 35993 | DDB_G0292814 | symA | calmodulin-binding protein, sugar transporter family protein, solute carrier family 2 member protein |
| inparanoid | 36006 | DDB_G0293014 | DDB_G0293014 |  |
| inparanoid | 36081 | DDB_G0289225 | DDB_G0289225 |  |
| inparanoid | 36115 | DDB_G0284493 | DDB_G0284493 |  |
| inparanoid | 36123 | DDB_G0284491 | DDB_G0284491 | putative protein kinase |
| inparanoid | 36163 | DDB_G0286375 | DDB_G0286375 |  |
| inparanoid | 36174 | DDB_G0287079 | DDB_G0287079 |  |
| inparanoid | 36325 | DDB_G0277919 | eRF3 | eukaryotic release factor 3 |
| inparanoid | 36346 | DDB_G0285099 | psmE3 | proteasome activator complex subunit 3, proteasome activator 28 subunit |
| inparanoid | 36352 | DDB_G0285455 | impA | FKBP-type peptidylprolyl cis-trans isomerase (PPIase) |
| inparanoid | 36354 | DDB_G0285117 | rad54b | SNF2-related domain-containing protein, DEAD/DEAH box helicase domain-containing protein, CHR group protein, helicase, C-terminal domain-containing protein |
| inparanoid | 36363 | DDB_G0290757 | dynF | dynactin subunit p27 |
| inparanoid | 36380 | DDB_G0285353 | hus1 | checkpoint clamp complex protein |
| inparanoid | 36445 | DDB_G0293600 | DDB_G0293600 |  |
| inparanoid | 36493 | DDB_G0284627 | DDB_G0284627 |  |
| inparanoid | 36498 | DDB_G0284173 | DDB_G0284173 |  |
| inparanoid | 36500 | DDB_G0283805 | adkA | adenylate kinase |
| inparanoid | 36512 | DDB_G0285461 | pigK | phosphatidylinositol glycan, class K |
| inparanoid | 36520 | DDB_G0284833 | exoc4 | exocyst complex subunit 4 |
| inparanoid | 36556 | DDB_G0269200 | cplA | calpain-like cysteine protease |
| inparanoid | 36559 | DDB_G0270818 | DDB_G0270818 |  |
| inparanoid | 36620 | DDB_G0271356 | DDB_G0271356 |  |
| inparanoid | 36629 | DDB_G0291320 | pop1 | RNase P protein subunit, RNase MRP protein subunit |
| inparanoid | 36659 | DDB_G0274201 | DDB_G0274201 | short-chain dehydrogenase/reductase (SDR) family protein |
| inparanoid | 36694 | DDB_G0283419 | gpaI | G-protein subunit alpha 9 |
| inparanoid | 36695 | DDB_G0283717 | pigX | phosphatidylinositol glycan, class X |
| inparanoid | 36729 | DDB_G0292498 | DDB_G0292498 |  |
| inparanoid | 36733 | DDB_G0293190 | DDB_G0293190 |  |
| inparanoid | 36746 | DDB_G0292682 | DDB_G0292682 |  |
| inparanoid | 36750 | DDB_G0292494 | DDB_G0292494 |  |
| inparanoid | 36758 | DDB_G0274491 | DDB_G0274491 |  |
| inparanoid | 36779 | DDB_G0276353 | torA | tortoise |
| inparanoid | 36795 | DDB_G0277311 | DDB_G0277311 | Similar to Dictyostelium discoideum (Slime mold). DG2033 protein. |
| inparanoid | 36797 | DDB_G0277213 | DDB_G0277213 | unknown |
| inparanoid | 36817 | DDB_G0278741 | adprt1A | poly(ADP-ribosyl)transferase, NAD+ ADP-ribosyltransferase |
| inparanoid | 36820 | DDB_G0278117 | sec61b | protein transport protein SEC61 beta subunit |
| inparanoid | 36877 | DDB_G0272362 | gtf2h2 | TFIIH subunit, general transcription factor IIH component |
| inparanoid | 36905 | DDB_G0289331 | abcG16 | ABC transporter G family protein |
| inparanoid | 36912 | DDB_G0287807 | DDB_G0287807 |  |
| inparanoid | 36934 | DDB_G0287889 | DDB_G0287889 |  |
| inparanoid | 36954 | DDB_G0273433 | DDB_G0273433 |  |
| inparanoid | 36990 | DDB_G0281793 | DDB_G0281793 |  |
| inparanoid | 37008 | DDB_G0281911 | DDB_G0281911 |  |
| inparanoid | 37011 | DDB_G0281791 | DDB_G0281791 |  |
| inparanoid | 37021 | DDB_G0287711 | DDB_G0287711 | MGC68542 protein. |
| inparanoid | 37027 | DDB_G0286731 | DDB_G0286731 |  |
| inparanoid | 37036 | DDB_G0287707 | rpc11 | RNA polymerase III subunit |
| inparanoid | 37066 | DDB_G0282593 | DDB_G0282593 | XAP5 protein, XAP-5 protein |
| inparanoid | 37069 | DDB_G0268236 | DDB_G0268236 |  |
| inparanoid | 37081 | DDB_G0277933 | DDB_G0277933 |  |
| inparanoid | 37084 | DDB_G0268238 | alg3 | glycosyltransferase, dolichyl-phosphate-mannose alpha-1,3-mannosyltransferase |
| inparanoid | 37089 | DDB_G0292764 | DDB_G0292764 |  |
| inparanoid | 37115 | DDB_G0272614 | DDB_G0272614 | DUF1794 family protein |
| inparanoid | 37126 | DDB_G0273467 | DDB_G0273467 | Similar to Homo sapiens (Human). MUM2 protein (Fragment). |
| inparanoid | 37177 | DDB_G0283671 | grn | granulin domain-containing protein |
| inparanoid | 37187 | DDB_G0283445 | DDB_G0283445 |  |
| inparanoid | 37192 | DDB_G0283669 | DDB_G0283669 | ubiquinol-cytochrome-c reductase subunit |
| inparanoid | 37207 | DDB_G0268536 | DDB_G0268536 |  |
| inparanoid | 37235 | DDB_G0291404 | eIF3s12 | eukaryotic translation initiation factor 3, subunit 12 |
| inparanoid | 37241 | DDB_G0288913 | DDB_G0288913 |  |
| inparanoid | 37243 | DDB_G0288947 | DDB_G0288947 | unknown |
| inparanoid | 37244 | DDB_G0288963 | DDB_G0288963 | peptidase M14 family protein, putative carboxypeptidase D (CPD) |
| inparanoid | 37248 | DDB_G0288911 | DDB_G0288911 | unknown |
| inparanoid | 37305 | DDB_G0275209 | cnrD | ubiquitin system component Cue domain containing protein, putative cell number regulator |
| inparanoid | 37309 | DDB_G0275257 | top3 | DNA topoisomerase III |
| inparanoid | 37322 | DDB_G0275669 | DDB_G0275669 | Similar to Arabidopsis thaliana (Mouse-ear cress). At3g30775/MIF6.16. |
| inparanoid | 37328 | DDB_G0275443 | helB1 | putative RNA helicase, DEAD/DEAH box helicase, putative RNA splicing factor |
| inparanoid | 37348 | DDB_G0286183 | agps | alkyl-dihydroxyacetonephosphate synthase, alkyldihydroxyacetonephosphate synthase |
| inparanoid | 37360 | DDB_G0286405 | DDB_G0286405 |  |
| inparanoid | 37370 | DDB_G0270440 | DDB_G0270440 |  |
| inparanoid | 37430 | DDB_G0287989 | DDB_G0287989 |  |
| inparanoid | 37434 | DDB_G0274271 | DDB_G0274271 |  |
| inparanoid | 37459 | DDB_G0283741 | rpl26 | S60 ribosomal protein L26 |
| inparanoid | 37479 | DDB_G0283127 | DDB_G0283127 |  |
| inparanoid | 37481 | DDB_G0284077 | DDB_G0284077 | putative metallophosphoesterase |
| inparanoid | 37483 | DDB_G0284473 | vatC | H(+)-transporting ATPase |
| inparanoid | 37512 | DDB_G0278295 | DDB_G0278295 |  |
| inparanoid | 37528 | DDB_G0278297 | DDB_G0278297 | metallophosphoesterase domain-containing protein |
| inparanoid | 37554 | DDB_G0277149 | DDB_G0277149 |  |
| inparanoid | 37568 | DDB_G0277173 | vamp7B | synaptobrevin domain-containing protein, longin domain-containing protein |
| inparanoid | 37572 | DDB_G0277335 | DDB_G0277335 | pmp22 family protein |
| inparanoid | 37582 | DDB_G0277243 | DDB_G0277243 | TRAF-type zinc finger-containing protein, meprin and TRAF homology (MATH) domain-containing protein |
| inparanoid | 37625 | DDB_G0279991 | exoc8 | exocyst complex subunit 8 |
| inparanoid | 37634 | DDB_G0280219 | vps55 | vacuolar protein sorting 55 family protein |
| inparanoid | 37635 | DDB_G0276115 | dph5 | diphthamide biosynthesis protein 5, methyltransferase |
| inparanoid | 37697 | DDB_G0282263 | DDB_G0282263 | leucine-rich repeat-containing protein (LRR) |
| inparanoid | 37701 | DDB_G0282265 | sgmC | metallophosphoesterase domain-containing protein, putative sphingomyelinase |
| inparanoid | 37728 | DDB_G0288161 | DDB_G0288161 |  |
| inparanoid | 37730 | DDB_G0287647 | DDB_G0287647 | esterase, SGNH hydrolase-type |
| inparanoid | 37736 | DDB_G0278159 | pssA | phosphatidylserine synthase |
| inparanoid | 37764 | DDB_G0284313 | tbcC | tubulin folding cofactor C |
| inparanoid | 37773 | DDB_G0280525 | DDB_G0280525 |  |
| inparanoid | 37781 | DDB_G0288443 | DDB_G0288443 |  |
| inparanoid | 37798 | DDB_G0290499 | slmo | slowmo family protein |
| inparanoid | 37818 | DDB_G0286741 | DDB_G0286741 |  |
| inparanoid | 37878 | DDB_G0287721 | DDB_G0287721 |  |
| inparanoid | 37893 | DDB_G0277717 | DDB_G0277717 |  |
| inparanoid | 37897 | DDB_G0277673 | DDB_G0277673 |  |
| inparanoid | 37919 | DDB_G0276239 | DDB_G0276239 | Sec1-like family protein |
| inparanoid | 37923 | DDB_G0277545 | CYP518A1 | cytochrome P450 family protein |
| inparanoid | 37955 | DDB_G0271550 | DDB_G0271550 | putative protein serine/threonine kinase, putative myosin light chain kinase, CAMK1 family protein kinase, protein kinase, CAMK group, putative CAM kinase |
| inparanoid | 37987 | DDB_G0275637 | abcF3 | ABC transporter-related protein |
| inparanoid | 38006 | DDB_G0272450 | DDB_G0272450 | Similar to Homo sapiens (Human). Tenascin (TN) (Hexabrachion) (Cytotactin) (Neuronectin) (GMEM) (JI) (Miotendinous antigen) (Glioma-associated-extracellular matrix antigen) (GP 150-225) (Tenascin-C) (TN-C). |
| inparanoid | 38045 | DDB_G0278357 | DDB_G0278357 |  |
| inparanoid | 38054 | DDB_G0288641 | DDB_G0288641 |  |
| inparanoid | 38082 | DDB_G0289713 | DDB_G0289713 | SAP DNA-binding domain-containing protein |
| inparanoid | 38112 | DDB_G0270936 | DDB_G0270936 |  |
| inparanoid | 38118 | DDB_G0268624 | DDB_G0268624 | UPF0405 family protein |
| inparanoid | 38120 | DDB_G0269756 | DDB_G0269756 |  |
| inparanoid | 38158 | DDB_G0279579 | DDB_G0279579 |  |
| inparanoid | 38166 | DDB_G0270040 | DDB_G0270040 |  |
| inparanoid | 38199 | DDB_G0281393 | cxdA | cytochrome c oxidase subunit IV |
| inparanoid | 38281 | DDB_G0285445 | DDB_G0285445 | WD40 repeat-containing protein, cyclin-like F-box containing protein, START domain-containing protein |
| inparanoid | 38290 | DDB_G0278277 | DDB_G0278277 |  |
| inparanoid | 38307 | DDB_G0276031 | DDB_G0276031 | WD40 repeat-containing protein |
| inparanoid | 38311 | DDB_G0276247 | DDB_G0276247 | unknown |
| inparanoid | 38317 | DDB_G0282921 | ctu1 | ATP-binding domain-containing protein 3, UPF0021 family protein, PP-loop ATPase domain-containing protein, cytosolic thiouridylase subunit 1 |
| inparanoid | 38323 | DDB_G0289499 | lsm7 | LSM (like-Sm) domain-containing protein, putative U6 small nuclear ribonucleoparticle-associated protein |
| inparanoid | 38327 | DDB_G0288903 | DDB_G0288903 |  |
| inparanoid | 38337 | DDB_G0289501 | cdc40 | WD40 repeat-containing protein, putative RNA splicing factor |
| inparanoid | 38349 | DDB_G0287013 | DDB_G0287013 | Proline iminopeptidase. |
| inparanoid | 38359 | DDB_G0274921 | DDB_G0274921 | Similar to Homo sapiens (Human). BK1048E9.3 (Novel protein). |
| inparanoid | 38363 | DDB_G0288041 | DDB_G0288041 |  |
| inparanoid | 38380 | DDB_G0287131 | gtf2f2 | TFIIF subunit |
| inparanoid | 38386 | DDB_G0269142 | hmgA | hmg CoA reductase A, hydroxymethylglutaryl CoA reductase |
| inparanoid | 38395 | DDB_G0286521 | DDB_G0286521 |  |
| inparanoid | 38410 | DDB_G0293364 | sigB | peptidase M8, leishmanolysin family protein |
| inparanoid | 38421 | DDB_G0292676 | bub1 | putative protein serine/threonine kinase, putative mitotic checkpoint protein, BUB family protein kinase |
| inparanoid | 38439 | DDB_G0270558 | DDB_G0270558 |  |
| inparanoid | 38446 | DDB_G0289823 | omt9 | O-methyltransferase family 2 protein |
| inparanoid | 38463 | DDB_G0286231 | DDB_G0286231 |  |
| inparanoid | 38478 | DDB_G0286253 | DDB_G0286253 |  |
| inparanoid | 38491 | DDB_G0268496 | DDB_G0268496 | HesA/moeB/thiF family protein. |
| inparanoid | 38502 | DDB_G0275261 | alg8 | glycosyltransferase, dolichyl-phosphate-glucose alpha-1,3-glucosyltransferase |
| inparanoid | 38509 | DDB_G0275055 | commd7 | COMM domain-containing protein 7 |
| inparanoid | 38530 | DDB_G0292418 | itfg1 | putative integrin alpha FG-GAP repeat-containing protein |
| inparanoid | 38535 | DDB_G0285963 | fhkD | putative protein serine/threonine kinase, protein kinase, CAMK group, RAD53 family protein kinase, FHA domain-containing protein |
| inparanoid | 38536 | DDB_G0281861 | DDB_G0281861 |  |
| inparanoid | 38568 | DDB_G0268548 | pikF | phosphatidylinositol 3-kinase, PI3kinase |
| inparanoid | 38570 | DDB_G0268116 | DDB_G0268116 |  |
| inparanoid | 38579 | DDB_G0268126 | DDB_G0268126 |  |
| inparanoid | 38615 | DDB_G0293330 | DDB_G0293330 | Similar to unknown protein YycE of Bacillus subtilis. |
| inparanoid | 38624 | DDB_G0287643 | DDB_G0287643 | unknown |
| inparanoid | 38642 | DDB_G0289607 | DDB_G0289607 |  |
| inparanoid | 38648 | DDB_G0289615 | DDB_G0289615 |  |
| inparanoid | 38705 | DDB_G0269246 | pyd2 | dihydropyrimidinase, pyrimidine hydrase |
| inparanoid | 38760 | DDB_G0293252 | DDB_G0293252 |  |
| inparanoid | 38766 | DDB_G0293254 | eIF3s5 | Mov34/MPN/PAD-1 family protein, eukaryotic translation initiation factor 3 (eIF3) subunit 5, eIF-3 epsilon |
| inparanoid | 38798 | DDB_G0293036 | ddx5 | putative RNA helicase, DEAD/DEAH box helicase |
| inparanoid | 38806 | DDB_G0293504 | DDB_G0293504 | tRNA nucleotidyltransferase (EC 2.7.7.25). |
| inparanoid | 38810 | DDB_G0293512 | DDB_G0293512 |  |
| inparanoid | 38826 | DDB_G0275515 | asnB | asparagine synthetase |
| inparanoid | 38833 | DDB_G0268712 | DDB_G0268712 | unknown |
| inparanoid | 38848 | DDB_G0267852 | DDB_G0267852 |  |
| inparanoid | 38849 | DDB_G0268620 | pkbA | protein serine/threonine kinase, protein kinase, AGC group, AKT family protein kinase, AKT/PKB protein kinase |
| inparanoid | 38905 | DDB_G0290649 | DDB_G0290649 | CCCH-type zinc finger-containing protein, RNA-binding region RNP-1 domain-containing protein, RNA recognition motif-containing protein RRM |
| inparanoid | 38928 | DDB_G0291177 | tspE | tetraspanin family protein |
| inparanoid | 38950 | DDB_G0274569 | acgA | adenylyl cyclase, non-G-protein-coupled adenylyl cyclase, adenylate cyclase |
| inparanoid | 38966 | DDB_G0274571 | mmgt | putative transmembrane protein, TMEM32 family protein, membrane magnesium transporter |
| inparanoid | 38971 | DDB_G0274815 | dlcB | cytoplasmic dynein light chain, dynein light chain LC8-type 2, dynein light chain type 1 family protein |
| inparanoid | 38991 | DDB_G0285935 | DDB_G0285935 |  |
| inparanoid | 39021 | DDB_G0292134 | bub3 | WD40 repeat-containing protein, putative mitotic checkpoint protein |
| inparanoid | 39053 | DDB_G0286345 | zntB | zinc/iron permease, zinc transporter |
| inparanoid | 39055 | DDB_G0286875 | gatA | glutamyl-tRNA(Gln) amidotransferase A subunit |
| inparanoid | 39072 | DDB_G0281447 | DDB_G0281447 |  |
| inparanoid | 39079 | DDB_G0288989 | DDB_G0288989 |  |
| inparanoid | 39093 | DDB_G0270592 | DDB_G0270592 |  |
| inparanoid | 39155 | DDB_G0288549 | DDB_G0288549 | DUF602 family protein |
| inparanoid | 39172 | DDB_G0288547 | DDB_G0288547 | Putative PRIP-interacting protein, 5'-partial (Fragment). |
| inparanoid | 39207 | DDB_G0293566 | DDB_G0293566 | glycoside hydrolase family 25 protein |
| inparanoid | 39209 | DDB_G0293374 | dut | dUTP diphosphatase, dUTPase |
| inparanoid | 39223 | DDB_G0293564 | gins2 | GINS complex subunit 2 |
| inparanoid | 39242 | DDB_G0267766 | gfm2 | mitochondrial translation elongation factor G |
| inparanoid | 39254 | DDB_G0279915 | abcB4 | ABC transporter B family protein |
| inparanoid | 39281 | DDB_G0279961 | drpp40 | RNase P protein subunit, RNase MRP protein subunit |
| inparanoid | 39282 | DDB_G0288985 | purN | phosphoribosylglycinamide formyltransferase |
| inparanoid | 39284 | DDB_G0288609 | DDB_G0288609 |  |
| inparanoid | 39301 | DDB_G0288983 | mrpl3 | ribosomal protein L3, mitochondrial |
| inparanoid | 39314 | DDB_G0285511 | DDB_G0285511 |  |
| inparanoid | 39331 | DDB_G0267976 | aph1 | gamma-secretase subunit Aph-1 |
| inparanoid | 39420 | DDB_G0269202 | gdcA | gp64 and disintegrin-like, cysteine-rich protein |
| inparanoid | 39437 | DDB_G0268946 | DDB_G0268946 |  |
| inparanoid | 39475 | DDB_G0285899 | DDB_G0285899 | alanine transaminase, alanine aminotransferase, glutamate pyruvate transaminase |
| inparanoid | 39483 | DDB_G0293646 | mcfN | mitochondrial substrate carrier family protein, putative mitochondrial phosphate carrier protein |
| inparanoid | 39515 | DDB_G0277449 | DDB_G0277449 | putative protein serine/threonine kinase, protein kinase, AGC group, SGK family protein kinase |
| inparanoid | 39573 | DDB_G0288533 | macA | cell fusion related protein |
| inparanoid | 39575 | DDB_G0289471 | auh | enoyl-CoA hydratase/isomerase domain-containing protein, methylglutaconyl-CoA hydratase |
| inparanoid | 39585 | DDB_G0285261 | DDB_G0285261 | regulator of chromosome condensation (RCC1) domain-containing protein |
| inparanoid | 39606 | DDB_G0285263 | spyA | glycosyltransferase, TPR repeat-containing protein, UDP-N-acetylglucosamine:peptide N-acetylglucosaminyltransferase, O-linked GlcNAc transferase |
| inparanoid | 39626 | DDB_G0277411 | DDB_G0277411 |  |
| inparanoid | 39635 | DDB_G0282863 | snrpG | LSM (like-Sm) domain-containing protein, putative small nuclear ribonucleoprotein G |
| inparanoid | 39668 | DDB_G0289015 | DDB_G0289015 |  |
| inparanoid | 39689 | DDB_G0288981 | DDB_G0288981 |  |
| inparanoid | 39699 | DDB_G0282211 | DDB_G0282211 |  |
| inparanoid | 39723 | DDB_G0272190 | fam49 | FAM49 family protein |
| inparanoid | 39740 | DDB_G0281731 | DDB_G0281731 |  |
| inparanoid | 39748 | DDB_G0271108 | DDB_G0271108 |  |
| inparanoid | 39776 | DDB_G0274591 | dolpp1 | dolichyldiphosphatase 1 |
| inparanoid | 39798 | DDB_G0274757 | DDB_G0274757 |  |
| inparanoid | 39853 | DDB_G0279499 | DDB_G0279499 |  |
| inparanoid | 39856 | DDB_G0276169 | DDB_G0276169 | AAA ATPase domain-containing protein |
| inparanoid | 39871 | DDB_G0278579 | dynE | dynactin 25 kDa subunit, dynactin subunit p25 |
| inparanoid | 39887 | DDB_G0277695 | DDB_G0277695 |  |
| inparanoid | 39893 | DDB_G0286565 | DDB_G0286565 |  |
| inparanoid | 39970 | DDB_G0268778 | rbbB | ARID/BRIGHT DNA binding domain-containing protein, transcription factor jumonji, jmjC domain-containing protein, transcription factor jumonji, jmjN domain-containing protein, putative Retinoblastoma (Rb) binding protein |
| inparanoid | 39975 | DDB_G0292440 | DDB_G0292440 | carbohydrate/purine kinase domain-containing protein |
| inparanoid | 39988 | DDB_G0268992 | anapc10 | anaphase promoting complex subunit 10 |
| inparanoid | 39991 | DDB_G0284249 | DDB_G0284249 | peptidase M41, FtsH domain-containing protein |
| inparanoid | 40046 | DDB_G0273135 | DDB_G0273135 |  |
| inparanoid | 40086 | DDB_G0284597 | CSN5 | Mov34/MPN/PAD-1 family protein, COP9 signalosome complex subunit 5 |
| inparanoid | 40098 | DDB_G0283735 | dnajc19 | DnaJ homolog subfamily C member 19, mitochondrial import inner membrane translocase subunit 14 |
| inparanoid | 40109 | DDB_G0293056 | DDB_G0293056 |  |
| inparanoid | 40129 | DDB_G0269816 | DDB_G0269816 | kin17-like protein |
| inparanoid | 40138 | DDB_G0275041 | DDB_G0275041 |  |
| inparanoid | 40165 | DDB_G0284239 | DDB_G0284239 | putative glycoside hydrolase |
| inparanoid | 40183 | DDB_G0293484 | psenen | gamma-secretase subunit, presenilin |
| inparanoid | 40196 | DDB_G0293474 | DDB_G0293474 |  |
| inparanoid | 40205 | DDB_G0285463 | DDB_G0285463 | putative transmembrane protein, protein kinase, TKL group, tyrosine kinase-like protein, CZAK family protein kinase |
| inparanoid | 40215 | DDB_G0290347 | DDB_G0290347 |  |
| inparanoid | 40221 | DDB_G0280769 | DDB_G0280769 |  |
| inparanoid | 40224 | DDB_G0268182 | DDB_G0268182 |  |
| inparanoid | 40304 | DDB_G0284943 | DDB_G0284943 |  |
| inparanoid | 40315 | DDB_G0285057 | DDB_G0285057 |  |
| inparanoid | 40321 | DDB_G0273411 | DDB_G0273411 |  |
| inparanoid | 40327 | DDB_G0273073 | abcG17-1 | ABC transporter G family protein |
| inparanoid | 40387 | DDB_G0285803 | DDB_G0285803 | Putative acetyltransferase. |
| inparanoid | 40410 | DDB_G0286007 | rbm8A | RNA recognition motif-containing protein RRM, RNA-binding motif protein 8A |
| inparanoid | 40441 | DDB_G0284287 | DDB_G0284287 |  |
| inparanoid | 40464 | DDB_G0282965 | DDB_G0282965 |  |
| inparanoid | 40482 | DDB_G0288835 | prfB | class I peptide chain release factor, peptide chain release factor 2 |
| inparanoid | 40511 | DDB_G0290387 | DDB_G0290387 | unknown |
| inparanoid | 40521 | DDB_G0288493 | DDB_G0288493 | Similar to unknown protein. |
| inparanoid | 40532 | DDB_G0283163 | eIF2b5 | bacterial transferase hexapeptide repeat-containing protein, eukaryotic translation initiation factor 2B, subunit 5 epsilon |
| inparanoid | 40557 | DDB_G0281821 | sre1 | steroid isomerase |
| inparanoid | 40617 | DDB_G0281849 | cct5 | chaperonin containing TCP1 epsilon subunit |
| inparanoid | 40619 | DDB_G0278643 | DDB_G0278643 | Gb|AAD34459.1 (SPP30-like protein) (At5g58030). |
| inparanoid | 40636 | DDB_G0282291 | potA | calcium-activated BK potassium channel, alpha subunit family protein, putative calcium-activated potassium channel |
| inparanoid | 40649 | DDB_G0275627 | sky1 | putative protein serine/threonine kinase, protein kinase, CMGC group, SRPK family protein kinase |
| inparanoid | 40660 | DDB_G0281847 | DDB_G0281847 |  |
| inparanoid | 40671 | DDB_G0292298 | DDB_G0292298 |  |
| inparanoid | 40694 | DDB_G0289667 | gefD | RhoGAP domain-containing protein, Ras guanine nucleotide exchange factor |
| inparanoid | 40766 | DDB_G0267468 | adprt4 | poly(ADP-ribosyl)transferase, NAD+ ADP-ribosyltransferase, BRCT domain-containing protein |
| inparanoid | 40779 | DDB_G0268024 | DDB_G0268024 | histone deacetylase family protein |
| inparanoid | 40780 | DDB_G0268016 | DDB_G0268016 | unknown |
| inparanoid | 40818 | DDB_G0293122 | DDB_G0293122 |  |
| inparanoid | 40822 | DDB_G0287795 | gcvH4 | glycine cleavage system H-protein |
| inparanoid | 40833 | DDB_G0276037 | DDB_G0276037 | zinc-containing alcohol dehydrogenase (ADH) |
| inparanoid | 40849 | DDB_G0282059 | DDB_G0282059 |  |
| inparanoid | 40868 | DDB_G0284807 | DDB_G0284807 | IPT/TIG domain-containing protein, EGF-like domain-containing protein |
| inparanoid | 40873 | DDB_G0284813 | DDB_G0284813 |  |
| inparanoid | 40919 | DDB_G0267786 | DDB_G0267786 | pleckstrin homology (PH) domain-containing protein |
| inparanoid | 40939 | DDB_G0286057 | adk | adenosine kinase, ATP:adenosine 5'-phosphotransferase |
| inparanoid | 40956 | DDB_G0292730 | adkB | adenylate kinase |
| inparanoid | 40972 | DDB_G0289591 | DDB_G0289591 |  |
| inparanoid | 40977 | DDB_G0289563 | DDB_G0289563 | calcium-binding EF-hand domain-containing protein |
| inparanoid | 41017 | DDB_G0288821 | DDB_G0288821 | small MutS related (smr) family protein, K homology (KH), type 1 domain containing-protein |
| inparanoid | 41069 | DDB_G0292212 | vps26l | vacuolar protein sorting-associated protein 26 family protein |
| inparanoid | 41082 | DDB_G0268798 | DDB_G0268798 |  |
| inparanoid | 41144 | DDB_G0278563 | DDB_G0278563 |  |
| inparanoid | 41176 | DDB_G0287551 | DDB_G0287551 |  |
| inparanoid | 41225 | DDB_G0268704 | DDB_G0268704 |  |
| inparanoid | 41242 | DDB_G0285179 | DDB_G0285179 |  |
| inparanoid | 41247 | DDB_G0283523 | DDB_G0283523 | unknown |
| inparanoid | 41299 | DDB_G0280287 | DDB_G0280287 |  |
| inparanoid | 41313 | DDB_G0291071 | DDB_G0291071 |  |
| inparanoid | 41316 | DDB_G0280289 | DDB_G0280289 | Putative GTP-binding protein. |
| inparanoid | 41330 | DDB_G0280061 | sybA | synaptobrevin domain-containing protein |
| inparanoid | 41353 | DDB_G0269648 | DDB_G0269648 |  |
| inparanoid | 41363 | DDB_G0268630 | spsA | spermidine synthase |
| inparanoid | 41378 | DDB_G0268840 | atg10 | autophagy protein 10 |
| inparanoid | 41380 | DDB_G0268628 | pykA | pyridoxal kinase |
| inparanoid | 41389 | DDB_G0290007 | DDB_G0290007 | Conserved hypothetical protein. |
| inparanoid | 41394 | DDB_G0290553 | DDB_G0290553 | NOL1/NOP2/Sun family protein, PUA domain-containing protein |
| inparanoid | 41400 | DDB_G0290555 | DDB_G0290555 | WD40 repeat-containing protein |
| inparanoid | 41438 | DDB_G0282655 | DDB_G0282655 |  |
| inparanoid | 41462 | DDB_G0270436 | DDB_G0270436 |  |
| inparanoid | 41476 | DDB_G0278075 | DDB_G0278075 |  |
| inparanoid | 41477 | DDB_G0278631 | DDB_G0278631 | solute carrier family 35 member protein, DUF250 family protein |
| inparanoid | 41507 | DDB_G0282431 | DDB_G0282431 |  |
| inparanoid | 41536 | DDB_G0268448 | fam45 | FAM45 family protein |
| inparanoid | 41544 | DDB_G0268574 | eIF4e | eukaryotic translation initiation factor 4E |
| inparanoid | 41556 | DDB_G0268668 | cycH | cyclin, putative H-type cyclin |
| inparanoid | 41569 | DDB_G0269052 | DDB_G0269052 | Q8R3D1 TBC1 domain family member 13. |
| inparanoid | 41571 | DDB_G0269054 | ppwd1 | WD40 repeat-containing protein, cyclophilin-type peptidylprolyl cis-trans isomerase (PPIase) |
| inparanoid | 41579 | DDB_G0279965 | DDB_G0279965 | NAD-dependent epimerase/dehydratase family protein, DUF1731 family protein |
| inparanoid | 41582 | DDB_G0293370 | DDB_G0293370 |  |
| inparanoid | 41583 | DDB_G0279923 | apnA | apurinic/apyrimidinic endonuclease, endonuclease IV |
| inparanoid | 41586 | DDB_G0279969 | DDB_G0279969 |  |
| inparanoid | 41593 | DDB_G0270948 | fntB | protein farnesyltransferase beta subunit |
| inparanoid | 41600 | DDB_G0269060 | DDB_G0269060 |  |
| inparanoid | 41631 | DDB_G0271544 | DDB_G0271544 | acetyl-CoA C-acetyltransferase, acetoacetyl-CoA thiolase |
| inparanoid | 41641 | DDB_G0271792 | arrC | ADP-ribosylation factor-related, ARF-related |
| inparanoid | 41643 | DDB_G0295477 | nip7 | 60S ribosome subunit biogenesis protein |
| inparanoid | 41662 | DDB_G0284663 | mrd1 | RNA-binding region RNP-1 domain-containing protein, RNA recognition motif-containing protein RRM |
| inparanoid | 41691 | DDB_G0279821 | DDB_G0279821 | ART-4 protein. |
| inparanoid | 41704 | DDB_G0283473 | DDB_G0283473 | P32359 Trehalase precursor (EC 3.2.1.28) (Alpha,alpha-trehalase) (Alpha,alpha-trehalose glucohydrolase). |
| inparanoid | 41727 | DDB_G0279015 | DDB_G0279015 |  |
| inparanoid | 41734 | DDB_G0279001 | gmkA | guanylate kinase |
| inparanoid | 41749 | DDB_G0288501 | ddx42 | putative RNA helicase, DEAD/DEAH box helicase |
| inparanoid | 41760 | DDB_G0277635 | rps27 | 40S ribosomal protein S27 |
| inparanoid | 41765 | DDB_G0277163 | DDB_G0277163 | HYPOTHETICAL PROTEIN VCA0903. 6/101 |
| inparanoid | 41767 | DDB_G0277159 | DDB_G0277159 |  |
| inparanoid | 41770 | DDB_G0271944 | DDB_G0271944 |  |
| inparanoid | 41790 | DDB_G0272120 | rcaA | peptidase M41, FtsH domain-containing protein |
| inparanoid | 41793 | DDB_G0272386 | DDB_G0272386 | Kelch repeat-containing protein, cyclin-like F-box containing protein |
| inparanoid | 41797 | DDB_G0272200 | DDB_G0272200 |  |
| inparanoid | 41822 | DDB_G0286579 | dnaja5 | DnaJ homolog subfamily A member 5 |
| inparanoid | 41832 | DDB_G0286679 | DDB_G0286679 |  |
| inparanoid | 41833 | DDB_G0274385 | DDB_G0274385 | Similar to Arabidopsis thaliana (Mouse-ear cress). SAG12 protein. |
| inparanoid | 41848 | DDB_G0277915 | gefI | Ras guanine nucleotide exchange factor |
| inparanoid | 41865 | DDB_G0279195 | adprt1B | poly(ADP-ribosyl)transferase, NAD+ ADP-ribosyltransferase |
| inparanoid | 41903 | DDB_G0272813 | cdk1 | p34-cdc2 protein, protein serine/threonine kinase, CDK family protein kinase, cyclin-dependent kinase, protein kinase, CMGC group, CDC2 subfamily protein kinase |
| inparanoid | 41908 | DDB_G0272354 | spt6 | SH2 domain-containing protein |
| inparanoid | 41918 | DDB_G0287783 | DDB_G0287783 |  |
| inparanoid | 41953 | DDB_G0280507 | DDB_G0280507 | O66677 Peptidyl-tRNA hydrolase (EC 3.1.1.29) (PTH). |
| inparanoid | 41960 | DDB_G0280843 | dgk | deoxyguanosine kinase |
| inparanoid | 41968 | DDB_G0278929 | usp39 | SAP DNA-binding domain-containing protein, putative U4/U6.U5 small nuclear ribonucleoparticle-associated protein, UBP-type zinc finger-containing protein, peptidase C19 family protein, putative ubiquitin carboxyl-terminal hydrolase (UCH) |
| inparanoid | 41990 | DDB_G0282945 | DDB_G0282945 |  |
| inparanoid | 42013 | DDB_G0276913 | DDB_G0276913 | Similar to plasmodium falciparum. elongation factor Tu, putative. |
| inparanoid | 42045 | DDB_G0267552 | DDB_G0267552 | putative NADH dehydrogenase (ubiquinone), putative NADH-ubiquinone oxidoreductase 13 kDa subunit |
| inparanoid | 42052 | DDB_G0283203 | vps22 | EAP30 family protein |
| inparanoid | 42067 | DDB_G0269906 | gtf2h5 | transcription initiation factor IIH5 |
| inparanoid | 42073 | DDB_G0281425 | DDB_G0281425 |  |
| inparanoid | 42075 | DDB_G0282197 | DDB_G0282197 | membrane bound O-acyl transferase family protein |
| inparanoid | 42077 | DDB_G0282195 | timm16 | presequence translocated-associated motor subunit, mitochondrial import inner membrane translocase subunit 16 |
| inparanoid | 42091 | DDB_G0272704 | CYP515A1 | cytochrome P450 family protein |
| inparanoid | 42098 | DDB_G0290277 | DDB_G0290277 |  |
| inparanoid | 42108 | DDB_G0290001 | DDB_G0290001 |  |
| inparanoid | 42131 | DDB_G0281033 | DDB_G0281033 |  |
| inparanoid | 42156 | DDB_G0279209 | DDB_G0279209 |  |
| inparanoid | 42185 | DDB_G0272871 | DDB_G0272871 | YNL222W. |
| inparanoid | 42199 | DDB_G0275895 | cchl | cytochrome c heme-lyase, holocytochrome c synthase |
| inparanoid | 42238 | DDB_G0288929 | DDB_G0288929 | Galactose transporter. |
| inparanoid | 42283 | DDB_G0282685 | DDB_G0282685 |  |
| inparanoid | 42305 | DDB_G0278489 | DDB_G0278489 |  |
| inparanoid | 42307 | DDB_G0278491 | DDB_G0278491 |  |
| inparanoid | 42309 | DDB_G0278495 | alp | alkaline phosphatase |
| inparanoid | 42322 | DDB_G0278487 | DDB_G0278487 | putative protein serine/threonine kinase, CDK family protein kinase, protein kinase, CMGC group |
| inparanoid | 42327 | DDB_G0290849 | zmpste24 | CAAX prenyl protease, zinc metalloproteinase, STE24 endopeptidase |
| inparanoid | 42334 | DDB_G0278479 | DDB_G0278479 |  |
| inparanoid | 42342 | DDB_G0290725 | lyrm4 | LYR motif-containing protein 4 |
| inparanoid | 42369 | DDB_G0289207 | tmcB | HAT repeat-containing protein |
| inparanoid | 42377 | DDB_G0287827 | pfdn1 | prefoldin beta-like domain containing protein, prefoldin subunit 1 |
| inparanoid | 42386 | DDB_G0283899 | mgp4 | RhoGAP domain-containing protein, Cdc15/Fes/CIP4 domain-containing protein, PCH family protein |
| inparanoid | 42435 | DDB_G0282397 | mbtps1 | membrane-bound transcription factor peptidase, site 1, subtilase family protein |
| inparanoid | 42455 | DDB_G0292892 | DDB_G0292892 |  |
| inparanoid | 42465 | DDB_G0271742 | DDB_G0271742 |  |
| inparanoid | 42473 | DDB_G0282387 | DDB_G0282387 | 3'-5' exonuclease domain-containing protein |
| inparanoid | 42477 | DDB_G0282531 | DDB_G0282531 | deoxyribonuclease, tatD-related DNAse |
| inparanoid | 42510 | DDB_G0276041 | DDB_G0276041 |  |
| inparanoid | 42591 | DDB_G0283035 | osbG | oxysterol binding family protein, member 7 |
| inparanoid | 42599 | DDB_G0288729 | DDB_G0288729 | zinc-containing alcohol dehydrogenase (ADH) |
| inparanoid | 42606 | DDB_G0282903 | DDB_G0282903 | unknown |
| inparanoid | 42637 | DDB_G0271512 | DDB_G0271512 | tRNA(m1A58)-methyltransferase subunit, tRNA (adenine-N(1)-)-methyltransferase catalytic subunit |
| inparanoid | 42650 | DDB_G0284879 | commd2 | COMM domain-containing protein 2 |
| inparanoid | 42693 | DDB_G0276121 | DDB_G0276121 | ELONGATION FACTOR TU FAMILY PROTEIN |
| inparanoid | 42712 | DDB_G0283303 | DDB_G0283303 |  |
| inparanoid | 42738 | DDB_G0291135 | DDB_G0291135 |  |
| inparanoid | 42748 | DDB_G0293576 | esf2 | RNA-binding region RNP-1 domain-containing protein, U3 snoRNP-associated protein Esf2 |
| inparanoid | 42780 | DDB_G0277269 | ionA | P-type ATPase, ATPase, P-type cation exchange, alpha subunit, putative sodium/potassium-transporting ATPase |
| inparanoid | 42810 | DDB_G0273081 | DDB_G0273081 | putative histidine ammonia-lyase, putative histidase |
| inparanoid | 42819 | DDB_G0288439 | syn8B | putative syntaxin 8, putative t-SNARE family protein |
| inparanoid | 42846 | DDB_G0274373 | gtr1 | putative glycosyltransferase, putative sulfotransferase |
| inparanoid | 42859 | DDB_G0287737 | DDB_G0287737 | transmembrane protein, cystinosin/ERS1p repeat-containing protein |
| inparanoid | 42873 | DDB_G0278273 | DDB_G0278273 |  |
| inparanoid | 42915 | DDB_G0272126 | DDB_G0272126 |  |
| inparanoid | 42924 | DDB_G0270578 | DDB_G0270578 |  |
| inparanoid | 42926 | DDB_G0269140 | gapA | RasGTPase-activating protein, IQGAP-related protein |
| inparanoid | 42928 | DDB_G0269612 | DDB_G0269612 |  |
| inparanoid | 42958 | DDB_G0275859 | DDB_G0275859 | leucine-rich repeat-containing protein (LRR) |
| inparanoid | 42964 | DDB_G0292626 | DDB_G0292626 | GTP-binding protein. |
| inparanoid | 42970 | DDB_G0292722 | DDB_G0292722 |  |
| inparanoid | 43005 | DDB_G0277561 | dynC | putative dynactin 22 kDa subunit |
| inparanoid | 43010 | DDB_G0277527 | ddx20 | putative RNA helicase, DEAD/DEAH box helicase, component of gems 3 |
| inparanoid | 43015 | DDB_G0277565 | syn5 | t-SNARE family protein, putative syntaxin 5 |
| inparanoid | 43022 | DDB_G0268586 | DDB_G0268586 |  |
| inparanoid | 43053 | DDB_G0291708 | guf1 | GTP-binding protein, LepA family protein |
| inparanoid | 43081 | DDB_G0285295 | vps28 | vacuolar protein sorting 28 family protein |
| inparanoid | 43101 | DDB_G0289711 | morg1 | WD40 repeat-containing protein, mitogen-activated protein kinase organizer 1 |
| inparanoid | 43131 | DDB_G0282825 | yipf6 | Yip1 domain-containing protein |
| inparanoid | 43149 | DDB_G0271376 | DDB_G0271376 | GLP_549_31342_32085. |
| inparanoid | 43200 | DDB_G0283815 | DDB_G0283815 |  |
| inparanoid | 43212 | DDB_G0283355 | DDB_G0283355 | unknown |
| inparanoid | 43221 | DDB_G0285855 | dlcC | cytoplasmic dynein light chain, roadblock/LC7 family protein |
| inparanoid | 43230 | DDB_G0283795 | commd3 | COMM domain-containing protein 3 |
| inparanoid | 43279 | DDB_G0289247 | apm1 | clathrin-adaptor medium chain apm1, ?1, mu1 |
| inparanoid | 43294 | DDB_G0274597 | ctnA | component of the counting factor (CF) complex, countin |
| inparanoid | 43332 | DDB_G0292784 | cog6 | oligomeric Golgi complex component |
| inparanoid | 43361 | DDB_G0281527 | DDB_G0281527 | Coatamer protein, beta subunit, putative. |
| inparanoid | 43402 | DDB_G0267528 | DDB_G0267528 | Genomic DNA, chromosome 5, P1 clone:MDN11 (Hypothetical protein) (At5g48020). |
| inparanoid | 43431 | DDB_G0278049 | DDB_G0278049 | unknown |
| inparanoid | 43460 | DDB_G0291520 | DDB_G0291520 |  |
| inparanoid | 43464 | DDB_G0290693 | eIF2b3 | eukaryotic translation initiation factor 2B, subunit 3 gamma, bacterial transferase hexapeptide repeat-containing protein |
| inparanoid | 43466 | DDB_G0290689 | DDB_G0290689 | NUDIX hydrolase family protein, dinucleoside polyphosphate hydrolase |
| inparanoid | 43486 | DDB_G0289355 | DDB_G0289355 |  |
| inparanoid | 43500 | DDB_G0288347 | nola3 | Nop10 family protein, H/ACA RNP complex subunit 3 |
| inparanoid | 43505 | DDB_G0288351 | DDB_G0288351 |  |
| inparanoid | 43525 | DDB_G0269322 | DDB_G0269322 |  |
| inparanoid | 43555 | DDB_G0292326 | gcvT | aminomethyltransferase, glycine cleavage system T-protein |
| inparanoid | 43563 | DDB_G0292322 | DDB_G0292322 | peptidoglycan-binding LysM domain-containing protein |
| inparanoid | 43611 | DDB_G0287077 | DDB_G0287077 |  |
| inparanoid | 43625 | DDB_G0275741 | dio3 | thyroxine 5'-deiodinase, putative type III iodothyronine deiodinase |
| inparanoid | 43642 | DDB_G0291326 | DDB_G0291326 |  |
| inparanoid | 43649 | DDB_G0292036 | DDB_G0292036 |  |
| inparanoid | 43660 | DDB_G0288785 | cbpL | NCS-1/frequenin-related protein |
| inparanoid | 43673 | DDB_G0287975 | DDB_G0287975 |  |
| inparanoid | 43687 | DDB_G0284751 | DDB_G0284751 |  |
| inparanoid | 43700 | DDB_G0283841 | alg6 | glycosyltransferase, dolichyl-phosphate-glucose alpha-1,3-glucosyltransferase |
| inparanoid | 43706 | DDB_G0283849 | DDB_G0283849 |  |
| inparanoid | 43718 | DDB_G0284125 | DDB_G0284125 |  |
| inparanoid | 43741 | DDB_G0284225 | ucpC | mitochondrial substrate carrier family protein, mitochondrial 2-oxoglutarate/malate carrier protein, mitochondrial brown fat uncoupling family protein |
| inparanoid | 43781 | DDB_G0277129 | DDB_G0277129 |  |
| inparanoid | 43792 | DDB_G0292592 | lst8 | WD40 repeat-containing protein |
| inparanoid | 43805 | DDB_G0285347 | DDB_G0285347 |  |
| inparanoid | 43818 | DDB_G0281289 | DDB_G0281289 |  |
| inparanoid | 43824 | DDB_G0270766 | DDB_G0270766 | short-chain dehydrogenase/reductase (SDR) family protein |
| inparanoid | 43829 | DDB_G0273097 | ptpA1-1 | protein-tyrosine phosphatase 1 |
| inparanoid | 43831 | DDB_G0272883 | ksrA-1 | 3-dehydrosphinganine reductase, 3-ketosphinganine reductase, short-chain dehydrogenase/reductase (SDR) family protein |
| inparanoid | 43833 | DDB_G0273039 | iptC-1 | putative isopentenyltransferase, putative isopentenylpyrophosphate (IPP) transferase |
| inparanoid | 43851 | DDB_G0274809 | mppA | mitochondrial processing peptidase alpha subunit |
| inparanoid | 4386 | DDB_G0274211 | DDB_G0274211 | Ras GTPase activation domain-containing protein |
| inparanoid | 43883 | DDB_G0267824 | DDB_G0267824 |  |
| inparanoid | 43889 | DDB_G0272302 | DDB_G0272302 |  |
| inparanoid | 43907 | DDB_G0270262 | DDB_G0270262 |  |
| inparanoid | 43915 | DDB_G0270264 | DDB_G0270264 | Similar to methionine aminopeptidase 2. |
| inparanoid | 43916 | DDB_G0269224 | fszB | mitochondrial cell division protein |
| inparanoid | 43939 | DDB_G0272418 | DDB_G0272418 | thioredoxin fold domain-containing protein |
| inparanoid | 43962 | DDB_G0272464 | DDB_G0272464 |  |
| inparanoid | 43974 | DDB_G0286481 | DDB_G0286481 | putative protein serine/threonine kinase, putative casein kinase II alpha chain (CK2), protein kinase, CMGC group, CK2 family protein kinase |
| inparanoid | 44008 | DDB_G0273237 | DDB_G0273237 | esterase/lipase/thioesterase domain-containing protein |
| inparanoid | 44017 | DDB_G0286027 | rfc4 | replication factor C subunit |
| inparanoid | 44031 | DDB_G0281043 | DDB_G0281043 |  |
| inparanoid | 44092 | DDB_G0286227 | DDB_G0286227 |  |
| inparanoid | 44097 | DDB_G0276261 | mcfV | mitochondrial substrate carrier family protein |
| inparanoid | 44127 | DDB_G0275403 | DDB_G0275403 | Similar to hypothetical protein MGC3162. |
| inparanoid | 44131 | DDB_G0275351 | DDB_G0275351 |  |
| inparanoid | 44134 | DDB_G0273273 | DDB_G0273273 |  |
| inparanoid | 44136 | DDB_G0272578 | ap3b-1 | beta adaptin |
| inparanoid | 44151 | DDB_G0293402 | DDB_G0293402 |  |
| inparanoid | 44157 | DDB_G0280091 | DDB_G0280091 | Putative E2, ubiquitin-conjugating enzyme UBC5. |
| inparanoid | 44167 | DDB_G0273413 | DDB_G0273413 | Similar to Xanthomonas axonopodis (Pv. citri). Pirin. |
| inparanoid | 4417 | DDB_G0286773 | gflB | RasGEF domain-containing protein, RhoGAP domain-containing protein |
| inparanoid | 44187 | DDB_G0284031 | DDB_G0284031 |  |
| inparanoid | 44210 | DDB_G0276997 | DDB_G0276997 | transmembrane protein, DHHC-type zinc finger-containing protein |
| inparanoid | 44221 | DDB_G0291384 | DDB_G0291384 | putative CorA family magnesium ion transporterr |
| inparanoid | 44226 | DDB_G0282693 | pex10 | RING zinc finger-containing protein, transmembrane protein, peroxisome biogenesis factor 10, peroxin 10 |
| inparanoid | 44239 | DDB_G0269802 | DDB_G0269802 |  |
| inparanoid | 44241 | DDB_G0286085 | DDB_G0286085 |  |
| inparanoid | 44242 | DDB_G0283967 | DDB_G0283967 |  |
| inparanoid | 44282 | DDB_G0272933 | DDB_G0272933 | unknown |
| inparanoid | 44291 | DDB_G0277889 | amtB | ammonium transporter |
| inparanoid | 44314 | DDB_G0281657 | DDB_G0281657 |  |
| inparanoid | 44316 | DDB_G0281563 | mybC | myb transcription factor |
| inparanoid | 44318 | DDB_G0281653 | DDB_G0281653 |  |
| inparanoid | 44326 | DDB_G0267768 | DDB_G0267768 | metal-dependent protein hydrolase domain-containing protein |
| inparanoid | 44338 | DDB_G0289051 | DDB_G0289051 |  |
| inparanoid | 44383 | DDB_G0283401 | ctsZ | cathepsin Z precursor, peptidase C1A family protein, papain family cysteine protease |
| inparanoid | 44385 | DDB_G0288851 | DDB_G0288851 |  |
| inparanoid | 44395 | DDB_G0283927 | DDB_G0283927 | putative protein serine/threonine kinase, NEK family protein kinase |
| inparanoid | 44399 | DDB_G0293868 | DDB_G0293868 |  |
| inparanoid | 44415 | DDB_G0278505 | DDB_G0278505 |  |
| inparanoid | 44467 | DDB_G0275959 | DDB_G0275959 | cellular retinaldehyde-binding/triple function domain-containing protein |
| inparanoid | 44503 | DDB_G0274239 | eIF1 | eukaryotic translation initiation factor 1, translation initiation factor SUI1 |
| inparanoid | 44520 | DDB_G0285903 | DDB_G0285903 |  |
| inparanoid | 44565 | DDB_G0272524 | malA | NADP-dependent malate dehydrogenase (oxaloacetate-decarboxylating), NADP malic enzyme |
| inparanoid | 44582 | DDB_G0269748 | DDB_G0269748 |  |
| inparanoid | 44595 | DDB_G0269742 | cdsA | CDP-diacylglycerol synthase, phosphatidate cytidylyltransferase |
| inparanoid | 44597 | DDB_G0269948 | tnpo | transportin |
| inparanoid | 4465 | DDB_G0267776 | DDB_G0267776 |  |
| inparanoid | 44673 | DDB_G0281985 | sec23 | putative transport protein |
| inparanoid | 44676 | DDB_G0281951 | cyrA | cysteine rich protein |
| inparanoid | 44721 | DDB_G0280819 | aoxA | alternative oxidase |
| inparanoid | 44750 | DDB_G0282967 | acadsb | acyl-Coenzyme A dehydrogenase, short/branched chain, acyl-CoA dehydrogenase |
| inparanoid | 44765 | DDB_G0274273 | DDB_G0274273 | Similar to Dictyostelium discoideum (Slime mold). R2005 protein. |
| inparanoid | 44767 | DDB_G0274471 | tpiA | triose phosphate isomerase, triosephosphate isomerase |
| inparanoid | 44848 | DDB_G0275263 | asnS1 | asparagine-tRNA ligase, asparaginyl-tRNA synthetase |
| inparanoid | 44882 | DDB_G0284553 | gmd | GDP-mannose 4,6-dehydratase, GDP-mannose 4,6-hydro-lyase |
| inparanoid | 44885 | DDB_G0267492 | DDB_G0267492 | Putative FtsH protease. |
| inparanoid | 44919 | DDB_G0275775 | psmD4 | type A von Willebrand factor (VWFA) domain-containing protein, 26S proteasome regulatory subunit S5A, 26S proteasome non-ATPase regulatory subunit 4, ubiquitin interacting motif (UIM) domain-containing protein |
| inparanoid | 44938 | DDB_G0280823 | rps6 | 40S ribosomal protein S6 |
| inparanoid | 44948 | DDB_G0278499 | DDB_G0278499 | protein phosphatase 2C |
| inparanoid | 44981 | DDB_G0291972 | culA | cullin A |
| inparanoid | 44993 | DDB_G0280181 | DDB_G0280181 | ankyrin repeat-containing protein, RA domain-containing protein |
| inparanoid | 44998 | DDB_G0283525 | arl8 | ARF-like protein, ADP-ribosylation factor-like protein |
| inparanoid | 45022 | DDB_G0284197 | proS | prolyl-tRNA synthetase, proline-tRNA ligase |
| inparanoid | 45029 | DDB_G0283679 | psmB7 | proteasome subunit beta type 7, 20S proteasome subunit beta-7 |
| inparanoid | 45032 | DDB_G0275157 | limF | LIM-type zinc finger-containing protein |
| inparanoid | 45058 | DDB_G0272969 | psmB1 | proteasome subunit beta type 1, 20S proteasome subunit beta-1 |
| inparanoid | 45073 | DDB_G0278507 | DDB_G0278507 |  |
| inparanoid | 45078 | DDB_G0282673 | DDB_G0282673 |  |
| inparanoid | 45088 | DDB_G0267420 | sodA | superoxide dismutase |
| inparanoid | 45096 | DDB_G0292564 | iplA | inositol 1,4,5-trisphosphate receptor-like protein, Ca2+ channel |
| inparanoid | 45117 | DDB_G0279301 | CAX1 | DUF307 family protein, type II CAX family calcium cation antiporter |
| inparanoid | 45123 | DDB_G0268076 | DDB_G0268076 |  |
| inparanoid | 45137 | DDB_G0279159 | aco1 | putative iron regulatory protein, aconitate hydratase, aconitase |
| inparanoid | 45148 | DDB_G0293858 | DDB_G0293858 |  |
| inparanoid | 45156 | DDB_G0293298 | mhsp70 | mitochondrial Hsp70 precursor |
| inparanoid | 45165 | DDB_G0293984 | DDB_G0293984 |  |
| inparanoid | 45187 | DDB_G0276347 | DDB_G0276347 | microtubule interacting and transport domain-containing protein (MIT) |
| inparanoid | 45198 | DDB_G0282845 | DDB_G0282845 |  |
| inparanoid | 45207 | DDB_G0292400 | vps2B | SNF7 family protein |
| inparanoid | 45211 | DDB_G0292396 | DDB_G0292396 |  |
| inparanoid | 45215 | DDB_G0282529 | rbbD | WD-40 repeat-containing protein, WD40 repeat-containing protein, putative Retinoblastoma (Rb) binding protein |
| inparanoid | 45258 | DDB_G0277221 | chcA | clathrin heavy chain |
| inparanoid | 45300 | DDB_G0292118 | alg11 | glycosyltransferase, alpha-1,2-mannosyltransferase |
| inparanoid | 45334 | DDB_G0267402 | H3a | histone H3 |
| inparanoid | 45375 | DDB_G0286111 | pigW | phosphatidylinositol glycan, class W |
| inparanoid | 45403 | DDB_G0268080 | rpc3 | RNA polymerase III subunit |
| inparanoid | 45411 | DDB_G0277761 | DDB_G0277761 |  |
| inparanoid | 45417 | DDB_G0285797 | DDB_G0285797 | unknown |
| inparanoid | 45443 | DDB_G0289371 | copG | adaptin N-terminal domain-containing protein, coatomer protein complex gamma subunit |
| inparanoid | 45484 | DDB_G0269446 | DDB_G0269446 |  |
| inparanoid | 45488 | DDB_G0270042 | ascc3l | DEAD/DEAH box helicase, U5 small nuclear ribonucleoprotein 200 kDa helicase, Sec63 domain-containing protein |
| inparanoid | 45518 | DDB_G0270670 | psaB | puromycin-sensitive aminopeptidase-like protein, metallopeptidase |
| inparanoid | 45522 | DDB_G0269856 | ddcA | group IV decarboxylase, Orn/DAP/Arg decarboxylase 2 domain-containing protein, putative diaminopimelate decarboxylase |
| inparanoid | 45567 | DDB_G0291123 | glpD | glycogen phosphorylase 2, glycogen phosphorylase a |
| inparanoid | 45589 | DDB_G0290987 | pus1 | tRNA pseudouridylate synthase |
| inparanoid | 45603 | DDB_G0291141 | DDB_G0291141 | putative zinc transporter, cation diffusion facilitator (CDF) family protein |
| inparanoid | 45609 | DDB_G0285103 | DDB_G0285103 |  |
| inparanoid | 45610 | DDB_G0285105 | psmD13 | 26S proteasome non-ATPase regulatory subunit 13, 26S proteasome regulatory subunit S11 |
| inparanoid | 45625 | DDB_G0283157 | DDB_G0283157 | Protein phosphatase 5, catalytic subunit. |
| inparanoid | 45627 | DDB_G0283161 | DDB_G0283161 |  |
| inparanoid | 45639 | DDB_G0285323 | atg9 | autophagy protein 9 |
| inparanoid | 45662 | DDB_G0288453 | DDB_G0288453 | COP1 homolog. |
| inparanoid | 45674 | DDB_G0293524 | ppk1 | polyphosphate kinase, poly P kinase |
| inparanoid | 45704 | DDB_G0287359 | DDB_G0287359 |  |
| inparanoid | 45776 | DDB_G0274215 | DDB_G0274215 |  |
| inparanoid | 45798 | DDB_G0289497 | DDB_G0289497 | unknown |
| inparanoid | 45823 | DDB_G0273105 | DDB_G0273105 | delta 9 fatty acid desaturase, stearoyl-CoA 9-desaturase, cytochrome b5 domain-containing protein |
| inparanoid | 45833 | DDB_G0272250 | rps23 | 40S ribosomal protein S23 |
| inparanoid | 45843 | DDB_G0273131 | g6pd-1 | glucose 6-phosphate-1-dehydrogenase |
| inparanoid | 45854 | DDB_G0273143 | DDB_G0273143 |  |
| inparanoid | 45887 | DDB_G0287529 | DDB_G0287529 |  |
| inparanoid | 45888 | DDB_G0287569 | agl | glycogen debranching enzyme, amylo-alpha-1,6-glucosidase |
| inparanoid | 45904 | DDB_G0287239 | DDB_G0287239 |  |
| inparanoid | 45921 | DDB_G0278571 | DDB_G0278571 | hypoxia induced family protein |
| inparanoid | 45931 | DDB_G0292388 | rpl27a | S60 ribosomal protein L27a |
| inparanoid | 45934 | DDB_G0269186 | snpC | soluble NSF attachment protein gamma isoform |
| inparanoid | 45946 | DDB_G0270836 | fbp | D-fructose-1,6-bisphosphate 1-phosphohydrolase, fructose-1,6-bisphosphatase |
| inparanoid | 45964 | DDB_G0270568 | DDB_G0270568 |  |
| inparanoid | 45970 | DDB_G0270026 | DDB_G0270026 |  |
| inparanoid | 45996 | DDB_G0277823 | alaS | alanyl-tRNA synthetase, alanine-tRNA ligase |
| inparanoid | 46012 | DDB_G0269784 | DDB_G0269784 |  |
| inparanoid | 46021 | DDB_G0267476 | sigK | EGF-like domain-containing protein |
| inparanoid | 46034 | DDB_G0273195 | DDB_G0273195 | oxalate/formate antiporter |
| inparanoid | 46037 | DDB_G0286691 | DDB_G0286691 |  |
| inparanoid | 46038 | DDB_G0285861 | DDB_G0285861 |  |
| inparanoid | 46059 | DDB_G0276153 | nsfA | N-ethylmaleimide-sensitive fusion protein |
| inparanoid | 46100 | DDB_G0284449 | plbD | phospholipase B-like protein |
| inparanoid | 46103 | DDB_G0284329 | gefA | Ras guanine nucleotide exchange factor |
| inparanoid | 46126 | DDB_G0284433 | DDB_G0284433 | F26G16.16. |
| inparanoid | 46137 | DDB_G0284347 | vps4 | AAA ATPase domain-containing protein, MIT domain-containing protein |
| inparanoid | 46161 | DDB_G0272396 | DDB_G0272396 | putative transmembrane protein |
| inparanoid | 46167 | DDB_G0272296 | sarA | GTP-binding protein Sar1A, ARF/SAR superfamily protein |
| inparanoid | 46211 | DDB_G0291314 | DDB_G0291314 | AAA ATPase domain-containing protein, putative endopeptidase Clp |
| inparanoid | 46221 | DDB_G0291648 | lpd | dihydrolipoamide:NAD oxidoreductase, dihydrolipoyl dehydrogenase, dihydrolipoamide dehydrogenase, glycine cleavage system L-protein |
| inparanoid | 46241 | DDB_G0275029 | odhB | dihydrolipoamide S-succinyltransferase, 2-oxoglutarate dehydrogenase E2 component, dihydrolipoyllysine-residue succinyltransferase |
| inparanoid | 46256 | DDB_G0292122 | psmA2 | proteasome subunit alpha type 2, 20S proteasome subunit alpha-2 |
| inparanoid | 46283 | DDB_G0270994 | psaA | puromycin-sensitive aminopeptidase-like protein, metallopeptidase |
| inparanoid | 46298 | DDB_G0288557 | vilA | villin, villidin |
| inparanoid | 46322 | DDB_G0286929 | DDB_G0286929 |  |
| inparanoid | 46345 | DDB_G0269472 | psmB2 | proteasome subunit beta type 2, 20S proteasome subunit beta-2 |
| inparanoid | 46346 | DDB_G0269474 | fcsB | fatty acyl-CoA synthetase, long-chain-fatty-acid-CoA ligase |
| inparanoid | 46357 | DDB_G0277913 | gefJ | Ras guanine nucleotide exchange factor |
| inparanoid | 46365 | DDB_G0277943 | parG | poly (ADP-ribose) glycohydrolase |
| inparanoid | 46429 | DDB_G0288891 | hemH | ferrochelatase |
| inparanoid | 46457 | DDB_G0280773 | DDB_G0280773 |  |
| inparanoid | 46478 | DDB_G0274141 | DDB_G0274141 | Vacuolar ATP synthase 21 kDa proteolipid subunit |
| inparanoid | 46484 | DDB_G0280909 | tal | transaldolase |
| inparanoid | 46490 | DDB_G0290391 | DDB_G0290391 |  |
| inparanoid | 46501 | DDB_G0278959 | rpl9 | 60S ribosomal protein L9 |
| inparanoid | 46512 | DDB_G0283803 | DDB_G0283803 | phosphatidylethanolamine-binding protein PEBP |
| inparanoid | 46530 | DDB_G0282035 | eIF2s2 | eIF-3 beta, eukaryotic translation initiation factor 2 (eIF2) subunit 2 |
| inparanoid | 46562 | DDB_G0278013 | DDB_G0278013 | unknown |
| inparanoid | 46567 | DDB_G0278581 | psiF | PA14 domain-containing protein, discoidin-inducing complex (DIC) protein |
| inparanoid | 46585 | DDB_G0288375 | lis1 | putative dynein regulator, WD40 repeat-containing protein, lissencephaly type-1-like homology (LisH) motif-containing protein |
| inparanoid | 46595 | DDB_G0287497 | nup133 | nucleoporin 133 |
| inparanoid | 46602 | DDB_G0288313 | DDB_G0288313 | WD repeat protein. |
| inparanoid | 46613 | DDB_G0275805 | DDB_G0275805 |  |
| inparanoid | 46649 | DDB_G0288257 | rpb2 | RNA polymerase II core subunit, RNA polymerase II, second largest subunit |
| inparanoid | 46683 | DDB_G0278991 | culE | cullin E |
| inparanoid | 46686 | DDB_G0269380 | DDB_G0269380 | P-type ATPase |
| inparanoid | 46762 | DDB_G0293816 | DDB_G0293816 |  |
| inparanoid | 46763 | DDB_G0293830 | DDB_G0293830 |  |
| inparanoid | 46764 | DDB_G0293838 | DDB_G0293838 | Hly-III related family protein |
| inparanoid | 46765 | DDB_G0293844 | CSN4 | proteasome component region PCI (PINT) domain-containing protein, COP9 signalosome complex subunit 4 |
| inparanoid | 46773 | DDB_G0293650 | fahd2 | Fumarylacetoacetate (FAA) hydrolase domain-containing protein |
| inparanoid | 46785 | DDB_G0291306 | xpo1 | exportin 1, chromosome region maintenance protein 1 |
| inparanoid | 46791 | DDB_G0270690 | DDB_G0270690 |  |
| inparanoid | 46840 | DDB_G0284259 | DDB_G0284259 | 4-alpha-glucanotransferase (EC 2.4.1.25). |
| inparanoid | 46880 | DDB_G0279795 | adamts | thrombospondin type I repeat-containing protein, catalytic peptidase aspartic domain-containing protein, a disintegrin-like and metallopeptidase (reprolysin type) with thrombospondin type 1 motif |
| inparanoid | 46881 | DDB_G0292266 | amdA | AMP deaminase |
| inparanoid | 46913 | DDB_G0282247 | rapgapB | RapGAP/RanGAP domain-containing protein, putative RapGAP |
| inparanoid | 46952 | DDB_G0285193 | hprT | hypoxanthine phosphoribosyltransferase |
| inparanoid | 46972 | DDB_G0284975 | tpsB | glycosyltransferase, alpha,alpha-trehalose-phosphate synthase, trehalose 6-phosphate synthase, trehalose-phosphatase |
| inparanoid | 46984 | DDB_G0283033 | DDB_G0283033 | peptidase S8 and S53 domain-containing protein |
| inparanoid | 46996 | DDB_G0283315 | dcp2 | mRNA-decapping enzyme 2 |
| inparanoid | 47001 | DDB_G0281523 | DDB_G0281523 |  |
| inparanoid | 47007 | DDB_G0279629 | lkb1 | putative protein serine/threonine kinase, protein kinase, CAMK group, CAMKL family protein kinase, LKB subfamily protein kinase |
| inparanoid | 47017 | DDB_G0268104 | gabT | 4-aminobutyrate transaminase, GABA transaminase, GABA transferase |
| inparanoid | 47028 | DDB_G0290055 | uap1 | UDP-N-acetylglucosamine pyrophosphorylase, UDP-N-acetylgalactosamine pyrophosphorylase |
| inparanoid | 47033 | DDB_G0276795 | ctbsB | glycoside hydrolase family 18 protein |
| inparanoid | 47102 | DDB_G0286061 | DDB_G0286061 |  |
| inparanoid | 47119 | DDB_G0286033 | pex5 | tetratricopeptide-like helical domain-containing protein (TPR), peroxisomal biogenesis factor 5, peroxin 5, putative peroxisome receptor 1 |
| inparanoid | 47154 | DDB_G0280763 | hemA | 5-aminolevulinate synthase, ALA synthase |
| inparanoid | 47156 | DDB_G0286887 | DDB_G0286887 |  |
| inparanoid | 47157 | DDB_G0270856 | DDB_G0270856 | RabGAP/TBC domain-containing protein, GRAM domain-containing protein |
| inparanoid | 47166 | DDB_G0269196 | tubB | beta tubulin |
| inparanoid | 47179 | DDB_G0292998 | rasB | Ras GTPase |
| inparanoid | 47201 | DDB_G0288473 | DDB_G0288473 |  |
| inparanoid | 47204 | DDB_G0290913 | mcfS | mitochondrial substrate carrier family protein, putative mitochondrial carnitine/acylcarnitine transporter |
| inparanoid | 47225 | DDB_G0288723 | DDB_G0288723 | UPF0553 family protein |
| inparanoid | 47236 | DDB_G0287953 | psmD1 | 26S proteasome regulatory subunit S1, 26S proteasome non-ATPase regulatory subunit 1 |
| inparanoid | 47274 | DDB_G0287661 | spt5 | transcription initiation factor Spt5 |
| inparanoid | 47276 | DDB_G0290825 | DDB_G0290825 |  |
| inparanoid | 47305 | DDB_G0276901 | DDB_G0276901 |  |
| inparanoid | 47315 | DDB_G0284867 | abcC8 | ABC transporter C family protein |
| inparanoid | 47329 | DDB_G0276463 | DG1112 | RasGTPase-activating protein, leucine-rich repeat-containing protein (LRR) |
| inparanoid | 47339 | DDB_G0284237 | rps12 | 40S ribosomal protein S12 |
| inparanoid | 47347 | DDB_G0290079 | pgtB | putative glycosyltransferase |
| inparanoid | 47368 | DDB_G0283293 | DDB_G0283293 | putative delta-1-pyrroline-5-carboxylate dehydrogenase |
| inparanoid | 47397 | DDB_G0287141 | pheSA | phenylalanyl-tRNA synthetase, alpha subunit, phenylalanine-tRNA ligase, alpha subunit |
| inparanoid | 47400 | DDB_G0287127 | vatA | vacuolar H+-ATPase A subunit |
| inparanoid | 47407 | DDB_G0287053 | lsm4 | LSM (like-Sm) domain-containing protein, putative U6 small nuclear ribonucleoparticle-associated protein |
| inparanoid | 47411 | DDB_G0277087 | purH | AICAR transformylase / IMP cyclohydrolase, inosine monophosphate cyclohydrolase / phosphoribosylaminoimidazolecarboxamide formyltransferase |
| inparanoid | 47412 | DDB_G0276855 | DDB_G0276855 | Hypothetical 54.5 kDa Trp-Asp repeats containing protein ZC302.2 |
| inparanoid | 47436 | DDB_G0276075 | DDB_G0276075 |  |
| inparanoid | 47438 | DDB_G0267934 | fnkB | FNIP repeat-containing protein, protein kinase, STE group, FNIPK subfamily protein kinase |
| inparanoid | 47441 | DDB_G0267924 | DDB_G0267924 | P-type ATPase, transmembrane protein, sodium/potassium-transporting ATPase alpha chain 2, Na+/K+ ATPase |
| inparanoid | 47448 | DDB_G0286137 | DDB_G0286137 |  |
| inparanoid | 47452 | DDB_G0285429 | purA | adenylosuccinate synthetase |
| inparanoid | 47494 | DDB_G0290405 | tpsC | glycosyltransferase, alpha,alpha-trehalose-phosphate synthase, trehalose 6-phosphate synthase, trehalose-phosphatase |
| inparanoid | 47510 | DDB_G0276457 | rps15a | 40S ribosomal protein S15a |
| inparanoid | 47518 | DDB_G0276549 | DDB_G0276549 | RapGAP/RanGAP domain-containing protein, putative RapGAP |
| inparanoid | 47524 | DDB_G0276305 | DDB_G0276305 | aldehyde dehydrogenase |
| inparanoid | 47540 | DDB_G0293982 | DDB_G0293982 |  |
| inparanoid | 47551 | DDB_G0290245 | psiC | PA14 domain-containing protein |
| inparanoid | 47601 | DDB_G0278885 | sec61a | protein transport protein SEC61 alpha subunit, SecY family protein |
| inparanoid | 47614 | DDB_G0285597 | rps28 | 40S ribosomal protein S28 |
| inparanoid | 47628 | DDB_G0269560 | DDB_G0269560 |  |
| inparanoid | 47629 | DDB_G0269242 | fcsA | fatty acyl-CoA synthetase, long-chain-fatty-acid-CoA ligase |
| inparanoid | 47664 | DDB_G0271678 | pckA | phosphoenolpyruvate carboxykinase |
| inparanoid | 47694 | DDB_G0277725 | thfA | methylenetetrahydrofolate dehydrogenase (NAD+) |
| inparanoid | 47696 | DDB_G0277789 | DDB_G0277789 | transmembrane protein, vacuolar sorting protein 9 domain-containing protein, DUF726 family protein |
| inparanoid | 47700 | DDB_G0277727 | uroc1 | urocanate hydratase, urocanase |
| inparanoid | 47703 | DDB_G0283067 | DDB_G0283067 | unknown |
| inparanoid | 47729 | DDB_G0279411 | ctsD | cathepsin D, preprocathepsin D |
| inparanoid | 47751 | DDB_G0277047 | DDB_G0277047 |  |
| inparanoid | 47757 | DDB_G0291916 | argJ | amino-acid N-acetyltransferase, N-acetylglutamate synthetase, ornithine transacetylase |
| inparanoid | 47784 | DDB_G0287037 | DDB_G0287037 |  |
| inparanoid | 47799 | DDB_G0281925 | DDB_G0281925 | putative RNA helicase, DEAD/DEAH box helicase |
| inparanoid | 47801 | DDB_G0282313 | DDB_G0282313 | heat shock protein DnaJ family protein |
| inparanoid | 47824 | DDB_G0274139 | cycC | cyclin, putative C-type cyclin |
| inparanoid | 47864 | DDB_G0287927 | DDB_G0287927 |  |
| inparanoid | 47891 | DDB_G0284141 | atp7a | P-type ATPase, copper-transporting ATPase |
| inparanoid | 47895 | DDB_G0284735 | copB | coatomer protein complex beta subunit, adaptin N-terminal domain-containing protein |
| inparanoid | 47902 | DDB_G0279275 | DDB_G0279275 |  |
| inparanoid | 47905 | DDB_G0283905 | phr2AB | protein phosphatase 2A regulatory subunit, protein phosphatase 2A B55 regulatory subunit, serine/threonine-protein phosphatase 2A regulatory subunit |
| inparanoid | 47917 | DDB_G0287231 | manF | alpha-mannosidase |
| inparanoid | 47921 | DDB_G0287621 | DDB_G0287621 |  |
| inparanoid | 47938 | DDB_G0269968 | DDB_G0269968 |  |
| inparanoid | 47972 | DDB_G0271736 | rabC | Rab GTPase |
| inparanoid | 47974 | DDB_G0276579 | DDB_G0276579 | Similar to Arabidopsis thaliana (Mouse-ear cress). peptide methionine sulfoxide reductase (Msr). |
| inparanoid | 47976 | DDB_G0276657 | DDB_G0276657 | NPC1 protein. |
| inparanoid | 4800 | DDB_G0278815 | eIF5b | eukaryotic translation initiation factor 5B |
| inparanoid | 48007 | DDB_G0278781 | pah | phenylalanine 4-monooxygenase, phenylalanine hydroxylase |
| inparanoid | 48011 | DDB_G0278779 | aco2 | aconitase, mitochondrial, aconitate hydratase, mitochondrial |
| inparanoid | 48016 | DDB_G0279141 | ap1b1 | adaptor-related protein complex 1, beta 1 subunit, beta adaptin |
| inparanoid | 48051 | DDB_G0279583 | DDB_G0279583 |  |
| inparanoid | 48061 | DDB_G0269772 | psmB3 | proteasome subunit beta type 3, 20S proteasome subunit beta-3 |
| inparanoid | 48090 | DDB_G0275179 | DDB_G0275179 | adenosine deaminase-related growth factor |
| inparanoid | 48119 | DDB_G0293216 | DDB_G0293216 |  |
| inparanoid | 48120 | DDB_G0293218 | vps35 | vacuolar sorting protein 35 |
| inparanoid | 48138 | DDB_G0281235 | DDB_G0281235 |  |
| inparanoid | 48145 | DDB_G0281093 | rpl37A | S60 ribosomal protein L37A, large subunit ribosomal protein |
| inparanoid | 48169 | DDB_G0281865 | adh5 | zinc-containing alcohol dehydrogenase (ADH), alcohol dehydrogenase, class 3, formaldehyde dehydrogenase |
| inparanoid | 48170 | DDB_G0292810 | gluA | beta glucosidase |
| inparanoid | 48207 | DDB_G0293700 | rps21 | 40S ribosomal protein S21 |
| inparanoid | 48214 | DDB_G0270422 | DDB_G0270422 | Q8NBS3 Sodium bicarbonate transporter-like protein 11 (Bicarbonate transporter-related protein-1). |
| inparanoid | 4822 | DDB_G0292948 | isw | myb domain-containing protein, SNF2-related protein, ATP-dependent chromatin-remodelling factor, CHR group protein, helicase, C-terminal domain-containing protein |
| inparanoid | 48233 | DDB_G0279183 | DDB_G0279183 | P-type ATPase |
| inparanoid | 48249 | DDB_G0283821 | DDB_G0283821 | putative protein serine/threonine kinase, protein kinase, STE group |
| inparanoid | 48253 | DDB_G0284419 | ercc4 | DNA excision repair protein 4 |
| inparanoid | 48259 | DDB_G0284411 | dng1 | PHD zinc finger-containing protein, inhibitor of growth (ING) family protein |
| inparanoid | 4830 | DDB_G0277797 | sec24l | putative transport protein |
| inparanoid | 48322 | DDB_G0267868 | DDB_G0267868 | Similar to Dictyostelium discoideum (Slime mold). hypothetical 97.7 kDa protein. |
| inparanoid | 48337 | DDB_G0272831 | psmA7 | proteasome subunit alpha type 7, 20S proteasome subunit alpha-7 |
| inparanoid | 48369 | DDB_G0283847 | DDB_G0283847 | cyclopropane fatty acid synthase, cyclopropane-fatty-acyl-phospholipid synthase |
| inparanoid | 48385 | DDB_G0274859 | prdx4 | peroxiredoxin, thioredoxin peroxidase |
| inparanoid | 48387 | DDB_G0274695 | DDB_G0274695 | SH3 domain-containing protein, Cdc15/Fes/CIP4 domain-containing protein |
| inparanoid | 48405 | DDB_G0271976 | rpl32 | S60 ribosomal protein L32 |
| inparanoid | 48413 | DDB_G0290123 | phbA |  |
| inparanoid | 48414 | DDB_G0288155 | DDB_G0288155 | trimeric LpxA-like domain-containing protein |
| inparanoid | 48421 | DDB_G0287595 | pgkA | phosphoglycerate kinase, calmodulin-binding protein |
| inparanoid | 48481 | DDB_G0284865 | ubcB | ubiquitin conjugating enzyme |
| inparanoid | 48492 | DDB_G0291273 | dtd | D-tyrosyl-tRNA(Tyr) deacylase |
| inparanoid | 48509 | DDB_G0275887 | masA | malate synthase |
| inparanoid | 48520 | DDB_G0280535 | sdhA | succinate dehydrogenase (ubiquinone), succinic dehydrogenase, complex II, flavoprotein subunit |
| inparanoid | 48521 | DDB_G0290593 | DDB_G0290593 | unknown |
| inparanoid | 48523 | DDB_G0280575 | DDB_G0280575 |  |
| inparanoid | 48539 | DDB_G0293076 | xpo6 | exportin 6 |
| inparanoid | 48542 | DDB_G0267796 | DDB_G0267796 | initiation factor eIF-4 gamma middle domain-containing protein, putative RNA splicing factor, initiation factor eIF-4 gamma, MA3 domain-containing protein |
| inparanoid | 48548 | DDB_G0267424 | amtC | ammonium transporter |
| inparanoid | 48551 | DDB_G0285941 | DDB_G0285941 |  |
| inparanoid | 48588 | DDB_G0277319 | atg3 | autophagy protein 3 |
| inparanoid | 48629 | DDB_G0283173 | kmo | kynurenine 3-monooxygenase, kynurenine 3-hydroxylase |
| inparanoid | 48640 | DDB_G0280037 | ddj1 | heat shock protein |
| inparanoid | 48646 | DDB_G0292780 | DDB_G0292780 | RE27154p. |
| inparanoid | 48687 | DDB_G0292848 | DDB_G0292848 |  |
| inparanoid | 48733 | DDB_G0270726 | DDB_G0270726 | pyridoxal phosphate-dependent decarboxylase family protein |
| inparanoid | 48737 | DDB_G0284485 | DDB_G0284485 | short-chain dehydrogenase/reductase (SDR) family protein |
| inparanoid | 48748 | DDB_G0284575 | DDB_G0284575 | Q9VK89 Probable N(2),N(2)-dimethylguanosine tRNA methyltransferase (EC 2.1.1.32) (tRNA(guanine-26,N(2)-N(2)) methyltransferase) (tRNA 2,2-dimethylguanosine-26 methyltransferase) (tRNA(m(2,2)G26)dimethyltransferase). |
| inparanoid | 48824 | DDB_G0277871 | rsc12 |  |
| inparanoid | 48828 | DDB_G0290901 | gefK | Ras guanine nucleotide exchange factor |
| inparanoid | 48839 | DDB_G0285741 | prdx5 | peroxiredoxin |
| inparanoid | 48841 | DDB_G0285739 | DDB_G0285739 |  |
| inparanoid | 48847 | DDB_G0269572 | DDB_G0269572 |  |
| inparanoid | 48852 | DDB_G0291714 | abcB3 | ABC transporter B family protein |
| inparanoid | 48856 | DDB_G0291858 | vatM | vacuolar proton ATPase 100-kDa subunit, vacuolar ATPase transmembrane subunit |
| inparanoid | 48864 | DDB_G0284605 | DDB_G0284605 | P-type ATPase, Ca2+-ATPase |
| inparanoid | 48870 | DDB_G0270870 | atp9b | ATPase, class II, type 9B |
| inparanoid | 48883 | DDB_G0291283 | sptB | serine C-palmitoyltransferase subunit |
| inparanoid | 48894 | DDB_G0271340 | DDB_G0271340 | acyl-CoA oxidase |
| inparanoid | 48931 | DDB_G0284995 | DDB_G0284995 | ankyrin repeat-containing protein, RabGAP/TBC domain-containing protein, putative GTPase activating protein (GAP) |
| inparanoid | 48962 | DDB_G0291247 | mfeA | peroxisomal multifunctional enzyme MFE homolog |
| inparanoid | 48969 | DDB_G0285637 | glb2 | glycoside hydrolase family 35 protein, beta-galactosidase |
| inparanoid | 48998 | DDB_G0287773 | gcvH1 | glycine cleavage system H-protein |
| inparanoid | 49010 | DDB_G0276371 | gefL | Ras guanine nucleotide exchange factor |
| inparanoid | 49021 | DDB_G0272564 | psmD8-1 | 26S proteasome regulatory subunit S14, 26S proteasome non-ATPase regulatory subunit 8 |
| inparanoid | 49032 | DDB_G0282429 | DDB_G0282429 | putative protein serine/threonine kinase, putative myosin light chain kinase, CAMK1 family protein kinase, protein kinase, CAMK group, putative CAM kinase |
| inparanoid | 49047 | DDB_G0288295 | rpl12 | S60 ribosomal protein L12 |
| inparanoid | 49097 | DDB_G0289179 | thyB | thymidine kinase, calmodulin-binding protein |
| inparanoid | 49129 | DDB_G0268538 | psmA5 | proteasome subunit alpha type 5, 20S proteasome subunit alpha-5 |
| inparanoid | 49138 | DDB_G0291402 | DDB_G0291402 |  |
| inparanoid | 49156 | DDB_G0289233 | DDB_G0289233 | Expressed protein. |
| inparanoid | 49160 | DDB_G0269154 | modA | alpha-glucosidase II |
| inparanoid | 49164 | DDB_G0269678 | DDB_G0269678 | Q8N0W3 L-fucose kinase (EC 2.7.1.52) (Fucokinase). |
| inparanoid | 49171 | DDB_G0281473 | DDB_G0281473 |  |
| inparanoid | 49184 | DDB_G0285161 | dagA | cytosolic regulator of adenylyl cyclase, PH domain-containing protein, cytosolic regulator of adenylate cyclase |
| inparanoid | 49207 | DDB_G0269190 | tcp1 | t-complex polypeptide 1, chaperonin containing TCP1 alpha subunit |
| inparanoid | 49251 | DDB_G0278293 | ileS | isoleucyl-tRNA synthetase, isoleucine-tRNA ligase |
| inparanoid | 49264 | DDB_G0276663 | DDB_G0276663 | Q9FY46 Sulfate transporter 4.1, chloroplast precursor (AST82). |
| inparanoid | 49280 | DDB_G0279941 | DDB_G0279941 | Gll1113 protein. |
| inparanoid | 49298 | DDB_G0284071 | rnrA | ribonucleotide reductase large subunit, ribonucleoside-diphosphate reductase |
| inparanoid | 49312 | DDB_G0281569 | tor | protein kinase, Atypical group, phosphatidylinositol 3-kinase-related protein kinase, PIKK family protein kinase, FRAP subfamily protein kinase |
| inparanoid | 49338 | DDB_G0279011 | DDB_G0279011 | thioredoxin domain-containing protein |
| inparanoid | 49348 | DDB_G0280461 | DDB_G0280461 | LISK family protein kinase, protein kinase, TKL group, tyrosine kinase-like protein |
| inparanoid | 49377 | DDB_G0286765 | DDB_G0286765 | AAA ATPase domain-containing protein, BCS1-like protein |
| inparanoid | 49381 | DDB_G0286315 | DDB_G0286315 | transmembrane protein |
| inparanoid | 49392 | DDB_G0276763 | captC | CDP-alcohol phosphatidyltransferase |
| inparanoid | 49396 | DDB_G0277733 | valS1 | valyl tRNA synthetase, valine-tRNA ligase |
| inparanoid | 49409 | DDB_G0272660 | serS | seryl-tRNA synthetase, serine-tRNA ligase |
| inparanoid | 49425 | DDB_G0291862 | rpl3 | 60S ribosomal protein L3 |
| inparanoid | 49487 | DDB_G0274375 | fba | fructose-bisphosphate aldolase, fructose 1,6-bisphosphate aldolase |
| inparanoid | 49513 | DDB_G0270352 | DDB_G0270352 | Probable acyl-CoA dehydrogenase (EC 1.3.99.-). |
| inparanoid | 49516 | DDB_G0277165 | DDB_G0277165 | putative protein serine/threonine kinase, protein kinase, CAMK group, CAMKL family protein kinase, putative CAM kinase, BRSK subfamily protein kinase |
| inparanoid | 49534 | DDB_G0290161 | miox | inositol oxygenase, myo-inositol oxygenase |
| inparanoid | 49561 | DDB_G0278285 | copB2 | WD40 repeat-containing protein, coatomer protein complex beta 2 subunit (beta prime) |
| inparanoid | 49581 | DDB_G0288039 | DDB_G0288039 |  |
| inparanoid | 49582 | DDB_G0287585 | rgaA | RasGTPase-activating protein, IQGAP-related protein |
| inparanoid | 49593 | DDB_G0292206 | manA | alpha-mannosidase |
| inparanoid | 49599 | DDB_G0281561 | tipA | protein phosphatase 2C-related protein, winged helix repressor DNA-binding domain-containing protein |
| inparanoid | 49610 | DDB_G0279659 | snd1 | Staphylococcus nuclease (SNase-like) domain-containing protein, tudor domain-containing protein |
| inparanoid | 49625 | DDB_G0286335 | bkdA | 3-methyl-2-oxobutanoate dehydrogenase (lipoamide), branched-chain alpha-keto acid dehydrogenase E1 alpha chain |
| inparanoid | 49642 | DDB_G0282605 | DDB_G0282605 | leucine-rich repeat-containing protein (LRR), Rho GTPase domain containing protein |
| inparanoid | 49657 | DDB_G0268028 | DDB_G0268028 | unknown |
| inparanoid | 49665 | DDB_G0279433 | metap1 | methionine aminopeptidase 1 |
| inparanoid | 49670 | DDB_G0279053 | DDB_G0279053 | 5'-nucleotidase |
| inparanoid | 49683 | DDB_G0289623 | pex11 | transmembrane protein, peroxisomal biogenesis factor 11, peroxin 11 |
| inparanoid | 49721 | DDB_G0285505 | ino1 | inositol-3-phosphate synthase, myo-inositol-1-phosphate synthase |
| inparanoid | 49739 | DDB_G0293034 | DDB_G0293034 |  |
| inparanoid | 49777 | DDB_G0267408 | psmA3 | 20S proteasome subunit alpha-3, proteasome subunit alpha type 3, 20S proteasome subunit C8 |
| inparanoid | 49816 | DDB_G0285939 | DDB_G0285939 | putative actin binding protein, vinculin-related protein |
| inparanoid | 49817 | DDB_G0286891 | DDB_G0286891 | P-type ATPase, transmembrane protein |
| inparanoid | 49821 | DDB_G0289175 | DG1091 | tetratricopeptide-like helical domain-containing protein (TPR) |
| inparanoid | 49833 | DDB_G0287005 | DDB_G0287005 | proteasome component region PCI (PINT) domain-containing protein |
| inparanoid | 49836 | DDB_G0286427 | uox | urate oxidase, uricase |
| inparanoid | 49852 | DDB_G0281403 | nacA | putative nascent polypeptide-associated complex alpha subunit |
| inparanoid | 49867 | DDB_G0269144 | hspB | heat shock protein, heat shock cognate protein Hsc70-1 |
| inparanoid | 49884 | DDB_G0282191 | polA1 | DNA polymerase alpha catalytic subunit, DNA polymerase alpha-primase |
| inparanoid | 49896 | DDB_G0283265 | mkkA | protein serine/threonine kinase, STE11 family protein kinase, MEK kinase, WD40 repeat-containing protein, protein kinase, STE group, F-box-containing protein, octicosapeptide/Phox/Bem1p domain-containing protein |
| inparanoid | 49903 | DDB_G0288647 | acad8 | acyl-CoA dehydrogenase, acyl-Coenzyme A dehydrogenase family member 8 |
| inparanoid | 49927 | DDB_G0293584 | sqle | squalene monooxygenase, squalene epoxidase |
| inparanoid | 49928 | DDB_G0293476 | DDB_G0293476 | unknown |
| inparanoid | 49929 | DDB_G0267386 | cysB | cystathionine beta-synthase |
| inparanoid | 49939 | DDB_G0288593 | DDB_G0288593 | Heat shock protein HslVU, ATPase subunit HslU, putative. |
| inparanoid | 49947 | DDB_G0277185 | DDB_G0277185 |  |
| inparanoid | 49949 | DDB_G0267400 | hspD | heat shock protein Hsp90 family protein |
| inparanoid | 50009 | DDB_G0293772 | vps52A | Vps52 / Sac2 family protein |
| inparanoid | 50012 | DDB_G0277401 | vatB | vacuolar H+ ATPase B subunit |
| inparanoid | 50024 | DDB_G0277503 | amtA | ammonium transporter |
| inparanoid | 50034 | DDB_G0291161 | DDB_G0291161 |  |
| inparanoid | 50048 | DDB_G0288541 | maoA | amine oxidase (flavin-containing), monoamine oxidase |
| inparanoid | 50060 | DDB_G0277303 | pheSB | phenylalanine-tRNA ligase, beta subunit, phenylalanyl-tRNA synthetase, beta subunit |
| inparanoid | 50065 | DDB_G0276835 | glnA1 | glutamate-ammonia ligase, glutamine synthetase type I |
| inparanoid | 50070 | DDB_G0282887 | trappc2 | trafficking protein particle complex subunit 2 |
| inparanoid | 50074 | DDB_G0282139 | DDB_G0282139 |  |
| inparanoid | 50092 | DDB_G0287281 | DDB_G0287281 | citrate synthase |
| inparanoid | 50094 | DDB_G0287631 | glk | glucokinase |
| inparanoid | 50132 | DDB_G0275299 | cln5 | unknown |
| inparanoid | 50145 | DDB_G0274105 | glgB | 1,4-alpha-glucan branching enzyme, branching enzyme |
| inparanoid | 50166 | DDB_G0286821 | DDB_G0286821 |  |
| inparanoid | 50173 | DDB_G0267426 | cshA | citrate synthase |
| inparanoid | 50193 | DDB_G0285065 | DDB_G0285065 |  |
| inparanoid | 50200 | DDB_G0282159 | DDB_G0282159 | Gll4250 protein. |
| inparanoid | 50257 | DDB_G0269156 | mvpA | major vault protein, major vault protein-alpha |
| inparanoid | 50260 | DDB_G0280081 | exoc2 | exocyst complex subunit 2 |
| inparanoid | 50286 | DDB_G0275045 | gpbB | G beta like protein, Guanine nucleotide-binding protein beta subunit-like protein., TRANS-SIALIDASE. 6/101 |
| inparanoid | 50293 | DDB_G0277841 | ctr9 | RNA polymerase II complex component |
| inparanoid | 50299 | DDB_G0285561 | rps15 | 40S ribosomal protein S15 |
| inparanoid | 50316 | DDB_G0275537 | cytC | cytochrome c |
| inparanoid | 50320 | DDB_G0278095 | mecr | trans-2-enoyl-CoA reductase, 2-enoyl thioester reductase |
| inparanoid | 50323 | DDB_G0284953 | oplah | 5-oxoprolinase (ATP-hydrolyzing), 5-oxo-L-proline amidohydrolase |
| inparanoid | 50334 | DDB_G0284939 | DDB_G0284939 | thioredoxin domain-containing protein |
| inparanoid | 50342 | DDB_G0273473 | DDB_G0273473 | unknown |
| inparanoid | 50351 | DDB_G0271734 | rapgap1 | RapGAP/RanGAP domain-containing protein |
| inparanoid | 50356 | DDB_G0272282 | DDB_G0272282 | protein serine/threonine kinase, protein kinase, AGC group, MAST family protein kinase |
| inparanoid | 50359 | DDB_G0286309 | DDB_G0286309 | zinc-containing alcohol dehydrogenase (ADH) |
| inparanoid | 50372 | DDB_G0285277 | rpl17 | S60 ribosomal protein L17 |
| inparanoid | 50391 | DDB_G0283115 | cnrB | cleft lip and palate transmembrane 1 family protein, putative cell number regulator |
| inparanoid | 50393 | DDB_G0278457 | ndrA | putative protein serine/threonine kinase, protein kinase, AGC group, NDR family protein kinase |
| inparanoid | 50407 | DDB_G0289157 | cmfB | putative CMF receptor CMFR1 |
| inparanoid | 50418 | DDB_G0288639 | DDB_G0288639 | DNAJ heat shock N-terminal domain-containing protein, TPR repeat-containing protein |
| inparanoid | 50432 | DDB_G0284773 | DDB_G0284773 |  |
| inparanoid | 50437 | DDB_G0283247 | pyk | pyruvate kinase, phosphoenolpyruvate kinase |
| inparanoid | 50441 | DDB_G0279741 | DDB_G0279741 |  |
| inparanoid | 50444 | DDB_G0275809 | msh2 | mutS homolog, DNA mismatch repair protein |
| inparanoid | 50446 | DDB_G0275567 | amd1 | S-adenosylmethionine decarboxylase |
| inparanoid | 50468 | DDB_G0282997 | rad54 | SNF2-related domain-containing protein, DEAD/DEAH box helicase domain-containing protein, CHR group protein, helicase, C-terminal domain-containing protein |
| inparanoid | 50476 | DDB_G0292354 | DDB_G0292354 | putative protein serine/threonine kinase, protein kinase, CK1 group, TTBK family protein kinase, putative tau-tubulin kinase, putative casein kinase I |
| inparanoid | 50499 | DDB_G0289461 | DDB_G0289461 | endonuclease/exonuclease/phosphatase domain-containing protein, putative CCR4-NOT complex subunit 6 |
| inparanoid | 50512 | DDB_G0273059 | cak1-1 | protein serine/threonine kinase, protein kinase, CK1 group, casein kinase I |
| inparanoid | 50524 | DDB_G0287689 | tubA | alpha tubulin |
| inparanoid | 50558 | DDB_G0290389 | ascc3 | DEAD/DEAH box helicase, Sec63 domain-containing protein, activating signal cointegrator 1 complex subunit |
| inparanoid | 50564 | DDB_G0267414 | repD | transcription factor IIH component |
| inparanoid | 50598 | DDB_G0293694 | DDB_G0293694 |  |
| inparanoid | 50656 | DDB_G0274595 | catA | catalase |
| inparanoid | 50668 | DDB_G0274399 | DDB_G0274399 | DNA2/NAM7 helicase family protein, SEN1-related protein |
| inparanoid | 50675 | DDB_G0285881 | rpl18a | S60 ribosomal protein L18a |
| inparanoid | 50700 | DDB_G0280307 | DDB_G0280307 | beta-lactamase family protein |
| inparanoid | 50727 | DDB_G0273441 | DDB_G0273441 |  |
| inparanoid | 50733 | DDB_G0290575 | DDB_G0290575 | LIM-type zinc finger-containing protein |
| inparanoid | 50749 | DDB_G0289199 | DDB_G0289199 |  |
| inparanoid | 50758 | DDB_G0268090 | DDB_G0268090 | glycoside hydrolase family 18 protein |
| inparanoid | 50761 | DDB_G0277991 | etfb | electron transfer flavoprotein beta subunit |
| inparanoid | 50803 | DDB_G0271668 | rpl36 | S60 ribosomal protein L36 |
| inparanoid | 50805 | DDB_G0271630 | sac1 | putative phosphoinositide phosphatase |
| inparanoid | 50813 | DDB_G0284727 | DDB_G0284727 |  |
| inparanoid | 50833 | DDB_G0279809 | DDB_G0279809 |  |
| inparanoid | 50838 | DDB_G0293194 | abcD2 | ABC transporter D family protein |
| inparanoid | 50845 | DDB_G0283555 | DDB_G0283555 | putative spore coat protein |
| inparanoid | 50849 | DDB_G0283539 | crtA | calreticulin |
| inparanoid | 50859 | DDB_G0279555 | DDB_G0279555 | Similar to Mus musculus (Mouse). 16 days embryo head cDNA, RIKEN full-length enriched library, clone:C130036O19 product:DJ5O6.2 (NOVEL PROTEIN similar to C. ELEGANS F40E10.6 (ISOFORM 1)) homolog. |
| inparanoid | 50879 | DDB_G0272196 | DDB_G0272196 | Similar to B0228.2.p. |
| inparanoid | 50892 | DDB_G0286681 | DDB_G0286681 |  |
| inparanoid | 50897 | DDB_G0288387 | accA | acetyl-CoA carboxylase, biotin carboxylase |
| inparanoid | 50915 | DDB_G0272318 | DDB_G0272318 | putative importin subunit alpha B |
| inparanoid | 50928 | DDB_G0288461 | hgsA | hydroxymethylglutaryl-CoA synthase |
| inparanoid | 50939 | DDB_G0280505 | DDB_G0280505 |  |
| inparanoid | 50950 | DDB_G0285285 | DDB_G0285285 |  |
| inparanoid | 50965 | DDB_G0277471 | DDB_G0277471 | Putative WD40 repeat protein (Hypothetical protein). |
| inparanoid | 50979 | DDB_G0283205 | top1 | DNA topoisomerase I |
| inparanoid | 50991 | DDB_G0269148 | lkhA | leukotriene A4 hydrolase |
| inparanoid | 50994 | DDB_G0290279 | dgat2 | diacylglycerol O-acyltransferase 2 |
| inparanoid | 51005 | DDB_G0282179 | DDB_G0282179 |  |
| inparanoid | 51008 | DDB_G0279207 | rps19 | 40S ribosomal protein S19 |
| inparanoid | 51014 | DDB_G0279211 | DDB_G0279211 | 5-methyltetrahydropteroyltriglutamate-homocysteine-S-methyltransferase, cobalamin-independent methionine synthase |
| inparanoid | 51018 | DDB_G0271094 | fah | fumarylacetoacetase, fumarylacetoacetate hydrolase |
| inparanoid | 51056 | DDB_G0288955 | DDB_G0288955 |  |
| inparanoid | 51067 | DDB_G0280215 | DDB_G0280215 | heat shock protein Hsp20 domain-containing protein, putative alpha-crystallin-type heat shock protein |
| inparanoid | 51071 | DDB_G0292456 | apeh | acylaminoacyl-peptidase, N-acylpeptide hydrolase, acylamino-acid-releasing enzyme |
| inparanoid | 51090 | DDB_G0283969 | trmt11 | tRNA guanosine-2'-O-methyltransferase 11 |
| inparanoid | 51094 | DDB_G0273419 | DDB_G0273419 | Similar to Haemonchus contortus (Barber pole worm). membrane aminopeptidase H11-4, isoform 4. |
| inparanoid | 51109 | DDB_G0282795 | DDB_G0282795 |  |
| inparanoid | 51128 | DDB_G0276141 | pdi1 | protein disulfide isomerase |
| inparanoid | 51144 | DDB_G0277815 | acsA | acetyl-CoA synthetase, acetyl-CoA ligase |
| inparanoid | 51175 | DDB_G0282069 | slrA | leucine-rich repeat-containing protein (LRR), S-cell enriched leucine-rich repeat protein |
| inparanoid | 51206 | DDB_G0291179 | metK | S-adenosylmethionine synthetase |
| inparanoid | 51234 | DDB_G0277287 | DDB_G0277287 |  |
| inparanoid | 51243 | DDB_G0278851 | DDB_G0278851 |  |
| inparanoid | 51261 | DDB_G0272566 | psmD14 | 26S proteasome non-ATPase regulatory subunit 14, putative multidrug resistance protein |
| inparanoid | 5127 | DDB_G0267658 | DDB_G0267658 |  |
| inparanoid | 51281 | DDB_G0277377 | srpR | signal recognition particle receptor alpha subunit |
| inparanoid | 51289 | DDB_G0277137 | DDB_G0277137 | SUR2-type hydroxylase/desaturase catalytic region-containing protein |
| inparanoid | 51299 | DDB_G0272374 | prpf6 | TPR repeat-containing protein, U5 small nuclear ribonucleoprotein-associated protein, PRP1 splicing factor domain-containing protein, pre-mRNA processing factor 6 |
| inparanoid | 51328 | DDB_G0274745 | DDB_G0274745 |  |
| inparanoid | 51357 | DDB_G0267380 | argE | acetylornithine deacetylase |
| inparanoid | 51398 | DDB_G0271122 | ufd1 | ubiquitin fusion degradation protein UFD1 family protein |
| inparanoid | 51401 | DDB_G0271106 | sod2 | superoxide dismutase, putative mitochondrial superoxide dismutase |
| inparanoid | 51410 | DDB_G0292380 | fhbB | flavohemoglobin |
| inparanoid | 51415 | DDB_G0283765 | hgd | homogentisate 1,2-dioxygenase, homogentisic acid oxidase |
| inparanoid | 51418 | DDB_G0292304 | mrkA | putative protein serine/threonine kinase, protein kinase, CAMK group, CAMKL family protein kinase, MARK subfamily protein kinase |
| inparanoid | 51421 | DDB_G0292654 | ndrJ | ribonucleoside triphosphate reductase, class II ribonucleoside reductase |
| inparanoid | 51433 | DDB_G0281383 | glpV | glycogen phosphorylase 1, glycogen phosphorylase b |
| inparanoid | 51448 | DDB_G0284835 | aass | aminoadipic semialdehyde synthase, alanine dehydrogenase/PNT domain-containing protein, saccharopine dehydrogenase, lysine-ketoglutarate reductase |
| inparanoid | 51452 | DDB_G0284755 | DDB_G0284755 | histidine acid phosphatase family protein |
| inparanoid | 51465 | DDB_G0274775 | DDB_G0274775 | pleckstrin homology (PH) domain-containing protein |
| inparanoid | 51485 | DDB_G0269138 | fttB |  |
| inparanoid | 51488 | DDB_G0281385 | rasC | Ras GTPase |
| inparanoid | 51500 | DDB_G0277245 | DDB_G0277245 | threonine ammonia-lyase, serine/threonine dehydratase, threonine deaminase |
| inparanoid | 51512 | DDB_G0277885 | gnd | 6-phosphogluconate dehydrogenase (decarboxylating) |
| inparanoid | 51521 | DDB_G0277849 | dymA | dynamin like protein |
| inparanoid | 51527 | DDB_G0293580 | DDB_G0293580 | dCTP deaminase |
| inparanoid | 51529 | DDB_G0272308 | rps29 | 40S ribosomal protein S29 |
| inparanoid | 51556 | DDB_G0275407 | txnl4a | thioredoxin-like U5 small nuclear ribonucleoprotein, DIM1-like protein |
| inparanoid | 51572 | DDB_G0288753 | ndrB | putative protein serine/threonine kinase, protein kinase, AGC group, NDR family protein kinase |
| inparanoid | 51588 | DDB_G0275535 | DDB_G0275535 | transmembrane protein, phospholipid-translocating P-type ATPase family protein |
| inparanoid | 51594 | DDB_G0289085 | mmsdh | methylmalonate-semialdehyde dehydrogenase (acylating) |
| inparanoid | 51614 | DDB_G0281545 | acaA | adenylyl cyclase, adenylate cyclase |
| inparanoid | 51636 | DDB_G0288783 | mybG | myb domain-containing protein |
| inparanoid | 51646 | DDB_G0281741 | cct3 | chaperonin containing TCP1 gamma subunit |
| inparanoid | 51655 | DDB_G0292594 | cyc1 | cytochrome c1 |
| inparanoid | 51682 | DDB_G0276417 | pdhB | pyruvate dehydrogenase E1 beta subunit |
| inparanoid | 51693 | DDB_G0272218 | DDB_G0272218 | Similar to Dictyostelium discoideum (Slime mold). MigA. |
| inparanoid | 51698 | DDB_G0284579 | DDB_G0284579 |  |
| inparanoid | 51741 | DDB_G0288877 | aarA | aardvark |
| inparanoid | 51758 | DDB_G0271882 | DDB_G0271882 | Similar to H+-transporting ATPase. |
| inparanoid | 51759 | DDB_G0281325 | DDB_G0281325 |  |
| inparanoid | 51772 | DDB_G0291802 | qtrt1 | queuine tRNA-ribosyltransferase, tRNA-guanine transglycosylase |
| inparanoid | 51779 | DDB_G0276931 | DDB_G0276931 |  |
| inparanoid | 51782 | DDB_G0290111 | adhfe1 | iron-containing alcohol dehydrogenase, alcohol dehydrogenase iron-containing 1 |
| inparanoid | 51786 | DDB_G0279719 | DDB_G0279719 | putative protein serine/threonine kinase |
| inparanoid | 51794 | DDB_G0284051 | DDB_G0284051 |  |
| inparanoid | 51830 | DDB_G0291386 | DDB_G0291386 | RNA recognition motif RNP-1 domain-containing protein |
| inparanoid | 51831 | DDB_G0288471 | taf6 | TATA-binding protein-associated-factor |
| inparanoid | 51857 | DDB_G0288563 | DDB_G0288563 |  |
| inparanoid | 51887 | DDB_G0268644 | DDB_G0268644 |  |
| inparanoid | 51896 | DDB_G0276899 | DDB_G0276899 | glutathione S-transferase domain-containing protein, thioredoxin fold domain-containing protein |
| inparanoid | 51901 | DDB_G0282469 | gnt13 | putative glycosyltransferase, putative GlcNAc transferase, putative beta-1,3-N-acetylglucosaminyltransferase |
| inparanoid | 51907 | DDB_G0284009 | ubcC | ubiquitin-conjugating enzyme |
| inparanoid | 51911 | DDB_G0289317 | DDB_G0289317 | GTP-binding-like protein. |
| inparanoid | 51917 | DDB_G0293532 | dstC | transcriptional repressor, signal transducer and activator of transcription (STAT) family protein |
| inparanoid | 51920 | DDB_G0280347 | DDB_G0280347 | ubiquitin-conjugating enzyme |
| inparanoid | 51923 | DDB_G0280345 | sgtA | tetratricopeptide-like helical domain-containing protein (TPR) |
| inparanoid | 51938 | DDB_G0291468 | DDB_G0291468 |  |
| inparanoid | 51947 | DDB_G0292310 | psenB | presenilin family protein, peptidase A22 family protein |
| inparanoid | 51969 | DDB_G0289909 | DDB_G0289909 |  |
| inparanoid | 51993 | DDB_G0278053 | ilvB | thiamine pyrophosphate-binding enzyme family protein, 2-hydroxyphytanoyl-CoA lyase |
| inparanoid | 52080 | DDB_G0269724 | DDB_G0269724 |  |
| inparanoid | 52083 | DDB_G0269982 | DDB_G0269982 | TBC domain protein. |
| inparanoid | 52099 | DDB_G0281677 | rps26 | 40S ribosomal protein S26 |
| inparanoid | 52102 | DDB_G0281683 | msh3 | mutS homolog, DNA mismatch repair protein |
| inparanoid | 52105 | DDB_G0281763 | DDB_G0281763 | Lupus La protein, La ribonucleoprotein |
| inparanoid | 52108 | DDB_G0281889 | DDB_G0281889 | P04105 Tubulin alpha-1B chain (Tubulin alpha-N chain). |
| inparanoid | 52118 | DDB_G0282363 | psmA1 | 20S proteasome subunit C2, proteasome subunit alpha type 1, 20S proteasome subunit alpha-1 |
| inparanoid | 52125 | DDB_G0282569 | sf3b3 | CPSF domain-containing protein, splicing factor 3B subunit 3 |
| inparanoid | 52137 | DDB_G0281983 | tmem50 | transmembrane protein, UPF0220 family protein, TMEM50 family protein |
| inparanoid | 52167 | DDB_G0278893 | DDB_G0278893 | Putative sulfite oxidase (SOX) (Moco containing protein). |
| inparanoid | 52171 | DDB_G0278541 | DDB_G0278541 | membrane bound O-acyl transferase family protein |
| inparanoid | 52175 | DDB_G0278539 | rpl5 | 60S ribosomal protein L5 |
| inparanoid | 52181 | DDB_G0279109 | eIF3s8 | proteasome component region PCI (PINT) domain-containing protein, eukaryotic translation initiation factor 3 (eIF3) subunit 8, eIF-3 p110 |
| inparanoid | 52183 | DDB_G0279115 | DDB_G0279115 |  |
| inparanoid | 52193 | DDB_G0283095 | DD3-3 | unknown |
| inparanoid | 52216 | DDB_G0282971 | DDB_G0282971 |  |
| inparanoid | 52224 | DDB_G0274309 | DDB_G0274309 | discoidin-like protein |
| inparanoid | 52292 | DDB_G0274331 | DDB_G0274331 | vacuolar ATP synthase subunit D |
| inparanoid | 52300 | DDB_G0274321 | purF | amidophosphoribosyltransferase, phosphoribosylpyrophosphate amidotransferase |
| inparanoid | 52310 | DDB_G0275057 | DDB_G0275057 | putative protein serine/threonine kinase, putative myosin light chain kinase, CAMK1 family protein kinase, protein kinase, CAMK group, putative CAM kinase |
| inparanoid | 52316 | DDB_G0292002 | ergic3 | endoplasmic reticulum-golgi intermediate compartment protein 3 |
| inparanoid | 52338 | DDB_G0267436 | abcF4 | putative non-transporter ABC protein |
| inparanoid | 52340 | DDB_G0267498 | DDB_G0267498 |  |
| inparanoid | 52377 | DDB_G0292866 | DDB_G0292866 | oligopeptidase B, protease II |
| inparanoid | 52381 | DDB_G0275853 | DDB_G0275853 | transmembrane protein |
| inparanoid | 52389 | DDB_G0275701 | vatE | vacuolar H+-ATPase E subunit |
| inparanoid | 5246 | DDB_G0283769 | HBS1 | Hsp70 subfamily B suppressor 1 |
| inparanoid | 52460 | DDB_G0280377 | DDB_G0280377 | pleckstrin homology (PH) domain-containing protein, putative phosphatidylinositol phosphate kinase |
| inparanoid | 52489 | DDB_G0284195 | DDB_G0284195 |  |
| inparanoid | 52501 | DDB_G0272955 | DDB_G0272955 | putative phytanoyl-CoA dioxygenase |
| inparanoid | 52503 | DDB_G0272696 | DDB_G0272696 |  |
| inparanoid | 52529 | DDB_G0272476 | DDB_G0272476 | putative NADH dehydrogenase (ubiquinone), putative NADH-ubiquinone oxidoreductase 8B subunit, mitochondrial ribosome domain-containing protein |
| inparanoid | 52531 | DDB_G0289907 | DDB_G0289907 | EGF-like domain-containing protein, C-type lectin domain-containing protein |
| inparanoid | 52580 | DDB_G0268060 | DDB_G0268060 | Q9FY46 Sulfate transporter 4.1, chloroplast precursor (AST82). |
| inparanoid | 52603 | DDB_G0285971 | rpl37 | ribosomal protein L37 |
| inparanoid | 52641 | DDB_G0276445 | DDB_G0276445 | heat shock protein Hsp70 family protein |
| inparanoid | 5265 | DDB_G0293388 | DDB_G0293388 | Putative FtsH protease. |
| inparanoid | 52650 | DDB_G0277015 | msp | membrane selenoprotein |
| inparanoid | 52661 | DDB_G0275663 | bpnt1 | bisphosphate nucleotidase, 3'(2'),5'-bisphosphate nucleotidase 1, PAP-inositol-1,4-phosphatase |
| inparanoid | 52670 | DDB_G0282853 | DDB_G0282853 | Q10344 Translationally controlled tumor protein homolog (TCTP) (p23fyp). |
| inparanoid | 52698 | DDB_G0282361 | prlA | proliferation associated protein |
| inparanoid | 52718 | DDB_G0279265 | DDB_G0279265 | F11P17.12 protein. |
| inparanoid | 52722 | DDB_G0279099 | DDB_G0279099 | O75140 Hypothetical protein KIAA0645. |
| inparanoid | 52726 | DDB_G0277569 | DDB_G0277569 | GNS1/SUR4 family protein |
| inparanoid | 52731 | DDB_G0275021 | ubpB | ubiquitin hydrolase B, deubiquinating enzyme, peptidase C19 family protein, putative ubiquitin carboxyl-terminal hydrolase (UCH) |
| inparanoid | 52734 | DDB_G0275329 | DDB_G0275329 | unknown |
| inparanoid | 52740 | DDB_G0276765 | ubqC | ubiquitin/ribosomal protein S27a fusion protein |
| inparanoid | 52756 | DDB_G0276943 | crtp1 | putative transmembrane protein, chloroquine resistance transporter-like protein |
| inparanoid | 52772 | DDB_G0292116 | DDB_G0292116 | MGC68498 protein. |
| inparanoid | 52776 | DDB_G0291986 | xpo7 | exportin 7, RAN binding protein 16 |
| inparanoid | 52804 | DDB_G0267478 | DDB_G0267478 |  |
| inparanoid | 52805 | DDB_G0267504 | DDB_G0267504 |  |
| inparanoid | 52810 | DDB_G0268064 | DDB_G0268064 | esterase/lipase/thioesterase domain-containing protein |
| inparanoid | 52831 | DDB_G0285999 | cnbB | calcium-binding EF-hand domain-containing protein, putative CaM-dependent protein phosphatase, regulatory subunit, putative calcineurin B |
| inparanoid | 52841 | DDB_G0286025 | DDB_G0286025 | galactose-binding domain-containing protein, putative cellulase |
| inparanoid | 52847 | DDB_G0285521 | isy1 | ISY1-like protein |
| inparanoid | 52851 | DDB_G0285509 | bcaA | branched-chain amino acid aminotransferase, branched-chain-amino-acid transaminase |
| inparanoid | 52854 | DDB_G0285507 | clp1 | pre-mRNA cleavage complex subunit |
| inparanoid | 52859 | DDB_G0286011 | alg1 | glycosyltransferase, beta-1,4-mannosyltransferase |
| inparanoid | 52874 | DDB_G0272210 | idhM | isocitrate dehydrogenase (NADP+), isocitrate dehydrogenase (NADP+), mitochondrial |
| inparanoid | 52877 | DDB_G0268068 | rab6 | Rab GTPase |
| inparanoid | 52896 | DDB_G0286171 | uqcrb | cytochrome bd ubiquinol oxidase, 14 kDa subunit |
| inparanoid | 52910 | DDB_G0289385 | DDB_G0289385 |  |
| inparanoid | 52923 | DDB_G0289329 | dmtA | des-methyl-DIF-1 methyltransferase, O-methyltransferase family 2 protein |
| inparanoid | 52931 | DDB_G0288801 | DDB_G0288801 | delta 9 fatty acid desaturase, stearoyl-CoA 9-desaturase, cytochrome b5 domain-containing protein |
| inparanoid | 52947 | DDB_G0270834 | sgmA | sphingomyelinase |
| inparanoid | 52960 | DDB_G0269412 | paf1 | RNA polymerase II-associated factor 1 |
| inparanoid | 52983 | DDB_G0270270 | gpsn2 | synaptic glycoprotein SC2-like protein |
| inparanoid | 52984 | DDB_G0269916 | atp5b | ATP synthase beta chain, mitochondrial |
| inparanoid | 52987 | DDB_G0270320 | nup54 | nucleoporin 54 |
| inparanoid | 53005 | DDB_G0270188 | psmD6 | 26S proteasome non-ATPase regulatory subunit 6, 26S proteasome regulatory subunit S10 |
| inparanoid | 53036 | DDB_G0291127 | mvpB | major vault protein, major vault protein B |
| inparanoid | 53050 | DDB_G0291013 | DDB_G0291013 |  |
| inparanoid | 53053 | DDB_G0291003 | DDB_G0291003 |  |
| inparanoid | 53091 | DDB_G0290929 | gtf2b | transcription initiation factor IIB |
| inparanoid | 53101 | DDB_G0283151 | gpaF | G-protein subunit alpha 6 |
| inparanoid | 53119 | DDB_G0293186 | DDB_G0293186 |  |
| inparanoid | 53138 | DDB_G0293004 | ctaA | putative cation-transporting ATPase |
| inparanoid | 53159 | DDB_G0293008 | cpnA | phospholipid-binding protein, copine A |
| inparanoid | 53172 | DDB_G0288449 | rpc2 | RNA polymerase III, second largest subunit |
| inparanoid | 53182 | DDB_G0295755 | glnA2 | glutamine synthetase type I |
| inparanoid | 53209 | DDB_G0283991 | DDB_G0283991 |  |
| inparanoid | 53219 | DDB_G0279345 | amfr | putative ubiquitin-protein ligase (E3) |
| inparanoid | 53222 | DDB_G0272076 | eIF3s6ip | proteasome component region PCI (PINT) domain-containing protein, eukaryotic translation initiation factor 3 (eIF3) subunit 6 interacting protein |
| inparanoid | 53257 | DDB_G0272470 | DDB_G0272470 | Similar to Multicopy Suppressor of STA10-11; Mss11p. |
| inparanoid | 53332 | DDB_G0290491 | DDB_G0290491 | MAP1-LC3 domain-containing protein |
| inparanoid | 53337 | DDB_G0291187 | DDB_G0291187 | Phytochelatins synthase. |
| inparanoid | 53342 | DDB_G0286563 | sigM | EGF-like domain-containing protein, serglycin domain-containing protein |
| inparanoid | 53355 | DDB_G0302511 | rpl39 | 60S ribosomal protein L39 |
| inparanoid | 53408 | DDB_G0270424 | rpl35a | S60 ribosomal protein L35a |
| inparanoid | 53413 | DDB_G0269826 | DDB_G0269826 |  |
| inparanoid | 53427 | DDB_G0270562 | acy1 | N-acyl-L-amino-acid amidohydrolase, aminoacylase |
| inparanoid | 53435 | DDB_G0270028 | DDB_G0270028 | aldehyde dehydrogenase, putative NAD-dependent aldehyde dehydrogenase |
| inparanoid | 53438 | DDB_G0269628 | DDB_G0269628 | putative protein serine/threonine kinase |
| inparanoid | 53443 | DDB_G0269252 | gefE | Ras guanine nucleotide exchange factor |
| inparanoid | 53497 | DDB_G0285821 | srp68 | signal recognition particle 68 kDa subunit |
| inparanoid | 53518 | DDB_G0285345 | DDB_G0285345 | unknown |
| inparanoid | 53545 | DDB_G0275493 | cycB | cyclin, G2/M-specific cyclinB |
| inparanoid | 53548 | DDB_G0275633 | DDB_G0275633 | DEAD/DEAH box helicase, superkiller viralicidic activity 2-like protein |
| inparanoid | 53556 | DDB_G0275039 | DDB_G0275039 | putative transmembrane protein |
| inparanoid | 53558 | DDB_G0286353 | erkA | extracellular signal-regulated protein kinase, protein serine/threonine kinase, MAP kinase, MAPK family protein kinase, ERK subfamily protein kinase, mitogen-activated protein kinase |
| inparanoid | 53560 | DDB_G0288863 | DDB_G0288863 |  |
| inparanoid | 53563 | DDB_G0284563 | cwc2 | pre-mRNA-splicing factor cwc2 |
| inparanoid | 53570 | DDB_G0284533 | rps13 | 40S ribosomal protein S13 |
| inparanoid | 53574 | DDB_G0277947 | shmt1 | serine hydroxymethyltransferase |
| inparanoid | 53590 | DDB_G0274387 | dpoA | prolyl oligopeptidase |
| inparanoid | 53602 | DDB_G0284333 | lvsF | BEACH domain-containing protein |
| inparanoid | 53617 | DDB_G0283385 | splA | non-receptor tyrosine kinase, dual-specificity protein kinase, protein kinase, TKL group, tyrosine kinase-like protein, CZAK family protein kinase |
| inparanoid | 53645 | DDB_G0272118 | ppp6C | protein phosphatase 6 catalytic subunit |
| inparanoid | 53689 | DDB_G0280187 | DDB_G0280187 | peptidase C1A family protein, papain family cysteine protease, cathepsin Z-like protein |
| inparanoid | 53711 | DDB_G0280145 | DDB_G0280145 | glycoside hydrolase family 63 protein |
| inparanoid | 53735 | DDB_G0291968 | DDB_G0291968 |  |
| inparanoid | 53740 | DDB_G0283621 | cutA | fatty acid elongase 3-ketoacyl-CoA synthase, long chain fatty acid elongase |
| inparanoid | 53745 | DDB_G0283629 | DDB_G0283629 | putative zinc transporter, cation diffusion facilitator (CDF) family protein |
| inparanoid | 53750 | DDB_G0283627 | prsC | phosphoribosyl pyrophosphate synthetase |
| inparanoid | 53753 | DDB_G0283625 | DDB_G0283625 | Mob1-like protein |
| inparanoid | 53766 | DDB_G0269888 | DDB_G0269888 |  |
| inparanoid | 53816 | DDB_G0268478 | DDB_G0268478 | UPF0041 family protein |
| inparanoid | 53830 | DDB_G0286163 | DDB_G0286163 | FNIP repeat-containing protein, B-box zinc finger-containing protein |
| inparanoid | 53882 | DDB_G0276523 | DDB_G0276523 | membrane bound O-acyl transferase family protein |
| inparanoid | 53888 | DDB_G0286229 | alyL | amy family lysozyme-like protein |
| inparanoid | 53921 | DDB_G0286703 | DDB_G0286703 | EbiP667 (Fragment). |
| inparanoid | 53930 | DDB_G0286491 | clcE | chloride channel protein, CLC 1/2 family protein |
| inparanoid | 53943 | DDB_G0273197 | alyD-1 | lysozyme |
| inparanoid | 53966 | DDB_G0290393 | DDB_G0290393 |  |
| inparanoid | 53968 | DDB_G0283349 | gpaA | G-protein subunit alpha 1 |
| inparanoid | 53972 | DDB_G0282941 | DDB_G0282941 | Ras GTPase activation domain-containing protein |
| inparanoid | 53982 | DDB_G0279363 | DDB_G0279363 |  |
| inparanoid | 53992 | DDB_G0289877 | rps9 | ribosomal protein 1024, 40S ribosomal protein S9 |
| inparanoid | 54001 | DDB_G0282581 | wdr45l | WD repeat domain phosphoinositide-interacting protein 3, wdr45 like protein |
| inparanoid | 54020 | DDB_G0279703 | prpf4B | putative protein serine/threonine kinase, DYRK family protein kinase, pre-mRNA processing factor 4 homolog B, protein kinase, CMGC group, PRP4 subfamily protein kinase |
| inparanoid | 54025 | DDB_G0278191 | dak | deoxyadenosine kinase, deoxyribonucleoside kinase |
| inparanoid | 54031 | DDB_G0278161 | DDB_G0278161 | multi antimicrobial extrusion (MatE) family protein |
| inparanoid | 54064 | DDB_G0290315 | rpl23 | S60 ribosomal protein L23 |
| inparanoid | 54085 | DDB_G0275601 | ltv1 | LVT1 homolog |
| inparanoid | 54123 | DDB_G0288271 | DDB_G0288271 |  |
| inparanoid | 54136 | DDB_G0277857 | helB2 | putative RNA helicase, DEAD/DEAH box helicase, putative RNA splicing factor, putative U5 small nuclear ribonucleoprotein |
| inparanoid | 54139 | DDB_G0278059 | DDB_G0278059 | putative acetyl-CoA transporter |
| inparanoid | 54141 | DDB_G0278341 | DDB_G0278341 | putative ATP citrate synthase, putative ATP citrate lyase |
| inparanoid | 54156 | DDB_G0278155 | mai | maleylacetoacetate isomerase |
| inparanoid | 54167 | DDB_G0269382 | pepD | peptidase D |
| inparanoid | 54169 | DDB_G0269016 | CYP524A1 | cytochrome P450 family protein |
| inparanoid | 54176 | DDB_G0269214 | abcG1 | ABC transporter G family protein |
| inparanoid | 54181 | DDB_G0271264 | usp14 | ubiquitin domain-containing protein, peptidase C19 family protein, putative ubiquitin carboxyl-terminal hydrolase (UCH) |
| inparanoid | 54199 | DDB_G0286305 | DDB_G0286305 | zinc-containing alcohol dehydrogenase (ADH) |
| inparanoid | 54218 | DDB_G0268034 | DDB_G0268034 | Rab GDP dissociation inhibitor alpha |
| inparanoid | 54221 | DDB_G0268038 | DDB_G0268038 | 12 days embryo spinal ganglion cDNA, RIKEN full-length enriched library, clone:D130045P06 product:granule cell differentiation protein, full insert sequence. |
| inparanoid | 54227 | DDB_G0291590 | DDB_G0291590 | CG8368 protein. |
| inparanoid | 54237 | DDB_G0284863 | limB | LIM-type zinc finger-containing protein |
| inparanoid | 54285 | DDB_G0280235 | DDB_G0280235 | DUF1682 family protein |
| inparanoid | 54286 | DDB_G0280237 | coq5 | ubiE/COQ5 methyltransferase family protein |
| inparanoid | 54296 | DDB_G0277271 | DDB_G0277271 |  |
| inparanoid | 54317 | DDB_G0276945 | apm4 | clathrin-adaptor medium chain apm 4, ?4, mu4 |
| inparanoid | 54340 | DDB_G0291265 | gloA | lactoylglutathione lyase, glyoxylase I |
| inparanoid | 54356 | DDB_G0292838 | taf13 | transcription initiation factor TFIID subunit, TFIID subunit |
| inparanoid | 54361 | DDB_G0279785 | DDB_G0279785 |  |
| inparanoid | 54372 | DDB_G0292358 | ino80 | SNF2-related domain-containing protein, DEAD/DEAH box helicase domain-containing protein, CHR group protein, helicase, C-terminal domain-containing protein |
| inparanoid | 54379 | DDB_G0292362 | DDB_G0292362 | peptidase M3A and M3B domain-containing protein |
| inparanoid | 54421 | DDB_G0289081 | DDB_G0289081 |  |
| inparanoid | 54428 | DDB_G0284887 | DDB_G0284887 |  |
| inparanoid | 54438 | DDB_G0285011 | DDB_G0285011 | putative mitochondrial transferase |
| inparanoid | 54444 | DDB_G0285071 | echs1 | enoyl-CoA hydratase, enoyl Coenzyme A hydratase, mitochondrial |
| inparanoid | 54449 | DDB_G0284985 | rab1D | Rab GTPase |
| inparanoid | 54488 | DDB_G0281543 | DDB_G0281543 |  |
| inparanoid | 54525 | DDB_G0278797 | DDB_G0278797 |  |
| inparanoid | 54540 | DDB_G0281549 | coxA | cytochrome c oxidase assembly protein |
| inparanoid | 54584 | DDB_G0286993 | DDB_G0286993 |  |
| inparanoid | 54646 | DDB_G0270488 | DDB_G0270488 |  |
| inparanoid | 54711 | DDB_G0289515 | pigS | phosphatidylinositol glycan, class S |
| inparanoid | 54721 | DDB_G0287619 | mpgA | mannose-1-phosphate guanylyltransferase, GTP:alpha-D-mannose-1-phosphate guanylyltransferase |
| inparanoid | 54729 | DDB_G0288089 | DDB_G0288089 | GC-rich sequence DNA-binding factor-like protein |
| inparanoid | 54735 | DDB_G0287371 | ada | adenosine deaminase |
| inparanoid | 54757 | DDB_G0278413 | ppcdc | phosphopantothenoylcysteine decarboxylase |
| inparanoid | 54769 | DDB_G0269180 | rcdP | random cDNA clone veg114 |
| inparanoid | 54771 | DDB_G0284065 | DDB_G0284065 | RapGAP/RanGAP domain-containing protein, putative RapGAP |
| inparanoid | 54791 | DDB_G0274283 | DDB_G0274283 | Hemolysin III |
| inparanoid | 54806 | DDB_G0284907 | DDB_G0284907 | unknown |
| inparanoid | 54812 | DDB_G0275295 | galE | UDP-glucose 4-epimerase |
| inparanoid | 54831 | DDB_G0290121 | purD | phosphoribosylamine-glycine ligase, phosphoribosylformylglycinamide cyclo-ligase |
| inparanoid | 54835 | DDB_G0290081 | sort1 | PDZ domain-containing protein, sortilin |
| inparanoid | 54842 | DDB_G0287977 | ftcd | formimidoyltransferase-cyclodeaminase |
| inparanoid | 54865 | DDB_G0286553 | rab21 | Rab GTPase |
| inparanoid | 54877 | DDB_G0268214 | enoB | phosphopyruvate hydratase, 2-phospho-D-glycerate hydrolase, enolase |
| inparanoid | 54918 | DDB_G0280337 | DDB_G0280337 |  |
| inparanoid | 54947 | DDB_G0284461 | DDB_G0284461 | C2 domain-containing protein, leucine-rich repeat-containing protein (LRR) |
| inparanoid | 54966 | DDB_G0277533 | DDB_G0277533 | WD40 repeat-containing protein |
| inparanoid | 54979 | DDB_G0290401 | DDB_G0290401 | pantothenate kinase |
| inparanoid | 54993 | DDB_G0278663 | DDB_G0278663 |  |
| inparanoid | 55023 | DDB_G0293784 | psmB5 | proteasome subunit beta type 5, 20S proteasome subunit beta-5 |
| inparanoid | 55026 | DDB_G0293752 | psmD2 | 26S proteasome regulatory subunit S2, 26S proteasome non-ATPase regulatory subunit 2 |
| inparanoid | 55028 | DDB_G0293572 | DDB_G0293572 |  |
| inparanoid | 55031 | DDB_G0270106 | DDB_G0270106 | Bll7928 protein. |
| inparanoid | 55042 | DDB_G0275383 | DDB_G0275383 | transmembrane protein |
| inparanoid | 55079 | DDB_G0290591 | DDB_G0290591 | winged helix DNA-binding domain-containing protein |
| inparanoid | 55106 | DDB_G0285575 | alkB | alkylated DNA repair protein |
| inparanoid | 55124 | DDB_G0269814 | ncbp1 | initiation factor eIF-4 gamma middle domain-containing protein, nuclear cap-binding protein subunit 1 |
| inparanoid | 55132 | DDB_G0274111 | pfkA | 6-phosphofructokinase |
| inparanoid | 55169 | DDB_G0280427 | ap4e1 | adaptin N-terminal domain-containing protein, epsilon adaptin, adaptor-related protein complex 4, epsilon 1 subunit |
| inparanoid | 55192 | DDB_G0295657 | ndufs4 | ETC complex I subunit conserved family protein, NADH dehydrogenase (ubiquinone) Fe-S protein 4 |
| inparanoid | 55195 | DDB_G0283003 | DDB_G0283003 | NifU-like protein. |
| inparanoid | 55210 | DDB_G0281661 | gtaI | putative GATA-binding transcription factor, GATA zinc finger domain-containing protein 9 |
| inparanoid | 55212 | DDB_G0281669 | DDB_G0281669 |  |
| inparanoid | 55232 | DDB_G0278717 | rsmG | small GTPase |
| inparanoid | 55252 | DDB_G0291482 | gloB2 | hydroxyacylglutathione hydrolase, glyoxylase II, beta-lactamase domain-containing protein |
| inparanoid | 55253 | DDB_G0291412 | srp72 | signal recognition particle 72 kDa subunit |
| inparanoid | 55281 | DDB_G0286223 | DDB_G0286223 |  |
| inparanoid | 55299 | DDB_G0281605 | cfaD | counting factor associated protein, cathepsin L-like proteinase, peptidase C1A family protein |
| inparanoid | 55347 | DDB_G0267540 | scy1 | N-terminal kinase-like (NTKL) protein, SCY1 family protein kinase |
| inparanoid | 55353 | DDB_G0268614 | msh6 | mutS homolog, DNA mismatch repair protein |
| inparanoid | 55370 | DDB_G0287469 | glud1 | NAD-dependent glutamate dehydrogenase, glutamate/phenylalanine/leucine/valine dehydrogenase |
| inparanoid | 55375 | DDB_G0287461 | abcG3 | ABC transporter G family protein |
| inparanoid | 55378 | DDB_G0287577 | manG | alpha-mannosidase |
| inparanoid | 55408 | DDB_G0287317 | DDB_G0287317 | putative transcriptional regulator |
| inparanoid | 55448 | DDB_G0268042 | DDB_G0268042 |  |
| inparanoid | 55455 | DDB_G0272326 | nudE | Lis-interacting protein |
| inparanoid | 55461 | DDB_G0271922 | mcfI | mitochondrial substrate carrier family protein |
| inparanoid | 55483 | DDB_G0280225 | DDB_G0280225 | unknown |
| inparanoid | 55519 | DDB_G0287691 | abcC3 | ABC transporter C family protein |
| inparanoid | 55539 | DDB_G0293530 | pefB | penta EF hand calcium binding protein |
| inparanoid | 55591 | DDB_G0271350 | DDB_G0271350 |  |
| inparanoid | 55596 | DDB_G0290641 | DDB_G0290641 |  |
| inparanoid | 5561 | DDB_G0286621 | BRIP1 | DEAD/DEAH box helicase |
| inparanoid | 55620 | DDB_G0281773 | mon1 | DUF254 family protein, SAND family protein |
| inparanoid | 55625 | DDB_G0268180 | DDB_G0268180 |  |
| inparanoid | 55652 | DDB_G0280053 | osbA | oxysterol binding family protein, member 1 |
| inparanoid | 55653 | DDB_G0267588 | DDB_G0267588 | putative phosphatidylinositol phosphate kinase |
| inparanoid | 55676 | DDB_G0281547 | amyA | putative alpha-amylase |
| inparanoid | 55687 | DDB_G0284321 | DDB_G0284321 | RNA-binding region RNP-1 domain-containing protein, RNA recognition motif-containing protein RRM, putative polypyrimidine tract binding protein (PTBP1) |
| inparanoid | 55697 | DDB_G0275355 | pccA | propionyl-CoA carboxylase, propanoyl-CoA:carbon dioxide ligase alpha subunit |
| inparanoid | 55708 | DDB_G0293540 | empB | emp24/gp25L/p24 family protein |
| inparanoid | 5571 | DDB_G0289183 | DDB_G0289183 | Q92JI3 Glucose inhibited division protein A. |
| inparanoid | 55714 | DDB_G0270420 | DDB_G0270420 | sucraseferredoxin-like family protein |
| inparanoid | 55744 | DDB_G0278409 | shkC | SH2 domain-containing protein, protein kinase, TKL group, tyrosine kinase-like protein, SHK subfamily protein kinase |
| inparanoid | 55753 | DDB_G0284415 | psmC3 | 26S proteasome ATPase 3 subunit, 26S protease regulatory subunit 6A, TAT-binding protein 1 |
| inparanoid | 55762 | DDB_G0284677 | expl2 | expansin-like protein |
| inparanoid | 55765 | DDB_G0284681 | DDB_G0284681 | P11915 Nonspecific lipid-transfer protein, mitochondrial precursor (NSL-TP) (Sterol carrier protein 2) (SCP-2) (Sterol carrier protein X) (SCP-X) (SCPX). |
| inparanoid | 55773 | DDB_G0275889 | DDB_G0275889 |  |
| inparanoid | 55778 | DDB_G0276503 | DDB_G0276503 | phosphatidylserine decarboxylase |
| inparanoid | 55807 | DDB_G0289733 | DDB_G0289733 |  |
| inparanoid | 55809 | DDB_G0289723 | abhd | alpha/beta hydrolase fold-1 domain-containing protein, abhydrolase domain-containing protein |
| inparanoid | 55816 | DDB_G0281351 | DDB_G0281351 |  |
| inparanoid | 55843 | DDB_G0272989 | vps5 | Phox domain-containing protein, putative sorting nexin |
| inparanoid | 55855 | DDB_G0290765 | jcdA | transcription factor jumonji, jmjC domain-containing protein, Kame |
| inparanoid | 55863 | DDB_G0283813 | hmgL | hydroxymethylglutaryl-CoA lyase |
| inparanoid | 55871 | DDB_G0284037 | DG1039 | MPN/PAD-1 domain-containing protein |
| inparanoid | 55908 | DDB_G0271960 | mccB | methylcrotonyl-CoA carboxylase, 3-methylcrotonyl-CoA:carbon dioxide ligase beta subunit |
| inparanoid | 55909 | DDB_G0271894 | DDB_G0271894 |  |
| inparanoid | 55916 | DDB_G0288323 | DDB_G0288323 |  |
| inparanoid | 55923 | DDB_G0287393 | mlh1 | MutL DNA mismatch repair protein |
| inparanoid | 55929 | DDB_G0278193 | DDB_G0278193 |  |
| inparanoid | 55932 | DDB_G0277879 | ugpB | UDP-glucose pyrophosphorylase 2 |
| inparanoid | 55934 | DDB_G0277949 | pldC | phospholipase D1 |
| inparanoid | 55969 | DDB_G0282715 | DDB_G0282715 | Alpha amylase family protein. |
| inparanoid | 55977 | DDB_G0282677 | spt16 | FACT complex subunit SPT16 |
| inparanoid | 55994 | DDB_G0291275 | DDB_G0291275 | mucolipin, putative Ca2+ channel |
| inparanoid | 55995 | DDB_G0291279 | DDB_G0291279 |  |
| inparanoid | 56024 | DDB_G0275813 | DDB_G0275813 | Similar to putative mitochondrial rna splicing protein. |
| inparanoid | 56026 | DDB_G0275573 | vps32 | SNF7 family protein |
| inparanoid | 56050 | DDB_G0284819 | DDB_G0284819 |  |
| inparanoid | 56059 | DDB_G0273355 | DDB_G0273355 |  |
| inparanoid | 56106 | DDB_G0292250 | sqrdl | putative sulfide quinone reductase |
| inparanoid | 56193 | DDB_G0291766 | DDB_G0291766 |  |
| inparanoid | 56257 | DDB_G0291301 | DDB_G0291301 | amine oxidase (flavin-containing), putative sarcosine oxidase, putative L-amino acid oxidase |
| inparanoid | 56261 | DDB_G0284107 | pgtC | putative glycosyltransferase |
| inparanoid | 56266 | DDB_G0288095 | DDB_G0288095 |  |
| inparanoid | 56277 | DDB_G0287153 | DDB_G0287153 | putative ubiquitin-conjugating enzyme E2 |
| inparanoid | 56279 | DDB_G0285663 | DDB_G0285663 | DUF214 family protein, FtsX domain-containing protein |
| inparanoid | 56283 | DDB_G0286395 | u2af2 | RNA-binding region RNP-1 domain-containing protein, RNA recognition motif-containing protein RRM, U2 snRNP auxiliary factor large subunit |
| inparanoid | 56286 | DDB_G0286389 | rpl34 | S60 ribosomal protein L34 |
| inparanoid | 56297 | DDB_G0269118 | cxeA | cytochrome c oxidase subunit V |
| inparanoid | 56354 | DDB_G0269624 | mgp3 | RhoGAP domain-containing protein, Cdc15/Fes/CIP4 domain-containing protein, MEGAP-like protein 1 |
| inparanoid | 56378 | DDB_G0270386 | DDB_G0270386 | P57772 Selenocysteine-specific elongation factor (Elongation factor sec). |
| inparanoid | 56441 | DDB_G0293130 | clcF | chloride channel protein, CLC 1/2 family protein |
| inparanoid | 56445 | DDB_G0279655 | DDB_G0279655 | putative phosphotyrosyl phosphatase activator |
| inparanoid | 56449 | DDB_G0279553 | DDB_G0279553 |  |
| inparanoid | 56450 | DDB_G0277309 | cnrJ | carbohydrate-binding domain-containing protein, putative cell number regulator |
| inparanoid | 56468 | DDB_G0278923 | DDB_G0278923 |  |
| inparanoid | 56481 | DDB_G0275787 | DDB_G0275787 | P35130 Ubiquitin-conjugating enzyme E2-17 kDa (EC 6.3.2.19) (Ubiquitin-protein ligase) (Ubiquitin carrier protein). |
| inparanoid | 56486 | DDB_G0272328 | DDB_G0272328 | Similar to Mus musculus (Mouse). 12 days embryo spinal ganglion cDNA, RIKEN full-length enriched library, clone:D130061K05 product:MEGF11 PROTEIN (KIAA1781) homolog. |
| inparanoid | 56584 | DDB_G0273525 | gtf2e1-1 | transcription factor IIE |
| inparanoid | 56591 | DDB_G0272616 | rnrB-1 | ribonucleotide reductase small subunit, ribonucleoside-diphosphate reductase |
| inparanoid | 56594 | DDB_G0272618 | tkt-1 | transketolase, glycolaldehydetransferase |
| inparanoid | 56621 | DDB_G0285877 | nadsyn1 | glutamine-dependent NAD(+) synthetase, NAD+ synthase (glutamine-hydrolysing) |
| inparanoid | 56653 | DDB_G0288917 | DDB_G0288917 |  |
| inparanoid | 56665 | DDB_G0270362 | DDB_G0270362 |  |
| inparanoid | 56680 | DDB_G0281471 | pdkA | putative protein serine/threonine kinase, protein kinase, AGC group, PDK1 family protein kinase |
| inparanoid | 56684 | DDB_G0281469 | rpl29 | S60 ribosomal protein L29 |
| inparanoid | 56694 | DDB_G0285213 | dhx15 | DEAD/DEAH box helicase, putative RNA splicing factor |
| inparanoid | 56726 | DDB_G0287993 | vps60 | SNF7 family protein |
| inparanoid | 56740 | DDB_G0288127 | ogdh | 2-oxoglutarate dehydrogenase, E1 subunit |
| inparanoid | 56766 | DDB_G0276779 | DDB_G0276779 | WD40 repeat-containing protein |
| inparanoid | 56777 | DDB_G0276027 | pdeE | cAMP/cGMP-stimulated cAMP/cGMP phosphodiesterase, beta-lactamase domain-containing protein |
| inparanoid | 56782 | DDB_G0285409 | DDB_G0285409 |  |
| inparanoid | 56804 | DDB_G0292246 | DDB_G0292246 | Similar to Pseudomonas putida KT2440. glutamine amidotransferase, class I. |
| inparanoid | 56832 | DDB_G0288165 | DDB_G0288165 | molybdenum cofactor sulfurase domain-containing protein |
| inparanoid | 56876 | DDB_G0286073 | DDB_G0286073 |  |
| inparanoid | 56877 | DDB_G0286075 | rps5 | 40S ribosomal protein S5 |
| inparanoid | 56890 | DDB_G0286767 | DDB_G0286767 |  |
| inparanoid | 56928 | DDB_G0272738 | iunH | N-D-ribosylpurine ribohydrolase, inosine-uridine nucleoside N-ribohydrolase |
| inparanoid | 56943 | DDB_G0275929 | DDB_G0275929 |  |
| inparanoid | 56948 | DDB_G0269364 | stlA | putative polyketide synthase, beta-ketoacyl synthase family protein |
| inparanoid | 56953 | DDB_G0269700 | DDB_G0269700 |  |
| inparanoid | 56985 | DDB_G0289451 | tmem167 | TMEM167 family protein |
| inparanoid | 56997 | DDB_G0288007 | DDB_G0288007 | MKIAA0368 protein (Fragment). |
| inparanoid | 57002 | DDB_G0270966 | DDB_G0270966 |  |
| inparanoid | 57008 | DDB_G0269864 | DDB_G0269864 |  |
| inparanoid | 57015 | DDB_G0270324 | DDB_G0270324 | NDR family protein |
| inparanoid | 57022 | DDB_G0271030 | DDB_G0271030 |  |
| inparanoid | 5706 | DDB_G0278693 | DDB_G0278693 |  |
| inparanoid | 57081 | DDB_G0294633 | DDB_G0294633 | ankyrin repeat-containing protein |
| inparanoid | 57084 | DDB_G0289481 | glnS | glutamine-tRNA ligase, glutaminyl-tRNA synthetase, vegetative specific protein H4 |
| inparanoid | 57109 | DDB_G0292224 | ecmG | cellulose-binding domain-containing protein, putative extracellular matrix protein |
| inparanoid | 57120 | DDB_G0279643 | DDB_G0279643 |  |
| inparanoid | 57126 | DDB_G0292834 | DDB_G0292834 | WD40 repeat-containing protein, cyclin-like F-box containing protein |
| inparanoid | 57131 | DDB_G0291239 | ubpA | deubiquitinating enzyme, UBP-type zinc finger-containing protein, peptidase C19 family protein, putative ubiquitin carboxyl-terminal hydrolase (UCH) |
| inparanoid | 57141 | DDB_G0290311 | DDB_G0290311 |  |
| inparanoid | 57147 | DDB_G0267384 | culB | cullin B |
| inparanoid | 57163 | DDB_G0281937 | DDB_G0281937 | maf family protein |
| inparanoid | 57189 | DDB_G0287221 | DDB_G0287221 | ubiquitin-protein ligase (HECT) domain-containing protein, armadillo repeat-containing protein |
| inparanoid | 57208 | DDB_G0289827 | rab18 | Rab GTPase |
| inparanoid | 57270 | DDB_G0293496 | vps52B | Vps52 / Sac2 family protein |
| inparanoid | 57306 | DDB_G0291173 | ndufv2 | NADH dehydrogenase (ubiquinone), NADH-ubiquinone oxidoreductase 24 kDa subunit |
| inparanoid | 57308 | DDB_G0274811 | argB | acetylglutamate kinase, N-acetylglutamate kinase |
| inparanoid | 57310 | DDB_G0274813 | DDB_G0274813 | WD40-like domain-containing protein |
| inparanoid | 57324 | DDB_G0283537 | rasS | Ras GTPase |
| inparanoid | 57337 | DDB_G0286889 | fut10 | glycosyltransferase, alpha-3/4-fucosyltransferase |
| inparanoid | 57346 | DDB_G0286871 | DDB_G0286871 |  |
| inparanoid | 57361 | DDB_G0281381 | dstA | signal transducer and activator of transcription (STAT) family protein |
| inparanoid | 57365 | DDB_G0281415 | DDB_G0281415 | Similar to dipeptidylpeptidase 8. |
| inparanoid | 57393 | DDB_G0271068 | DDB_G0271068 |  |
| inparanoid | 57406 | DDB_G0282199 | allB1 | allantoinase |
| inparanoid | 57477 | DDB_G0267582 | DDB_G0267582 |  |
| inparanoid | 57488 | DDB_G0269768 | cnrI | SET domain-containing protein, putative cell number regulator |
| inparanoid | 57498 | DDB_G0268870 | DDB_G0268870 |  |
| inparanoid | 57519 | DDB_G0285901 | DDB_G0285901 | Vps53-like domain-containing protein |
| inparanoid | 57548 | DDB_G0291201 | ctrC | putative cationic amino acid transporter, solute carrier family 7 member protein |
| inparanoid | 57580 | DDB_G0277483 | DDB_G0277483 |  |
| inparanoid | 57581 | DDB_G0276837 | DDB_G0276837 |  |
| inparanoid | 57630 | DDB_G0272192 | DDB_G0272192 |  |
| inparanoid | 57646 | DDB_G0275297 | DDB_G0275297 |  |
| inparanoid | 57680 | DDB_G0276193 | DDB_G0276193 | putative transmembrane protein, polymorphic membrane protein repeat-containing protein |
| inparanoid | 57749 | DDB_G0286533 | DDB_G0286533 |  |
| inparanoid | 57754 | DDB_G0271886 | DDB_G0271886 | PHD zinc finger-containing protein, bromodomain-containing protein |
| inparanoid | 57755 | DDB_G0271866 | DDB_G0271866 | Similar to Leptospira interrogans serovar lai str. 56601. enoyl-CoA hydratase (EC 4.2.1.17). |
| inparanoid | 57761 | DDB_G0285111 | snpA | soluble NSF attachment protein alpha isoform |
| inparanoid | 57785 | DDB_G0284243 | DDB_G0284243 | protein phosphatase 2C-related protein |
| inparanoid | 57790 | DDB_G0293078 | DDB_G0293078 |  |
| inparanoid | 57796 | DDB_G0285467 | DDB_G0285467 | putative amidohydrolase, metal-dependent hydrolase family protein |
| inparanoid | 57799 | DDB_G0285465 | nog1 | nucleolar GTP-binding protein 1 |
| inparanoid | 57810 | DDB_G0279149 | pip5k3 | FYVE-type zinc finger-containing protein, FYVE finger-containing phosphoinositide kinase, PIP kinase, 1-phosphatidylinositol-3-phosphate 5-kinase |
| inparanoid | 57851 | DDB_G0284945 | fsjC | rRNA methyltransferase, putative rRNA (uridine-2'-O-)-methyltransferase |
| inparanoid | 57886 | DDB_G0276905 | DDB_G0276905 |  |
| inparanoid | 57918 | DDB_G0277851 | dymB | dynamin B |
| inparanoid | 57925 | DDB_G0275843 | DDB_G0275843 | pleckstrin homology (PH) domain-containing protein, Arf GTPase activating protein |
| inparanoid | 57934 | DDB_G0288613 | erf1 | eukaryotic translation termination factor 1, eukaryotic petide chain release factor subunit 1 |
| inparanoid | 57949 | DDB_G0289013 | slc44a2 | solute carrier family 44 protein member 2 |
| inparanoid | 57961 | DDB_G0281857 | DDB_G0281857 |  |
| inparanoid | 57975 | DDB_G0290187 | hspH | heat shock protein Hsp70 family protein |
| inparanoid | 57993 | DDB_G0281071 | serA | 3-phosphoglycerate dehydrogenase |
| inparanoid | 57996 | DDB_G0281181 | DDB_G0281181 |  |
| inparanoid | 58007 | DDB_G0287217 | DDB_G0287217 |  |
| inparanoid | 58027 | DDB_G0287407 | DDB_G0287407 | TPR repeat-containing protein |
| inparanoid | 58029 | DDB_G0287405 | DDB_G0287405 | LMBR1-like conserved region-containing protein |
| inparanoid | 58099 | DDB_G0292830 | DDB_G0292830 | Na+/H+ antiporter. |
| inparanoid | 58140 | DDB_G0272799 | icmA-1 | prenylcysteine methyltransferase, protein-S-isoprenylcysteine O-methyltransferase |
| inparanoid | 58152 | DDB_G0268796 | DDB_G0268796 |  |
| inparanoid | 58166 | DDB_G0274221 | colB | colossin B, Cna B-type domain-containing protein |
| inparanoid | 58183 | DDB_G0274781 | DDB_G0274781 | calcium-binding protein, recoverin family protein, NCS-1/frequenin-related protein, S100/CaBP-9k-type calcium-binding domain-containing protein |
| inparanoid | 58184 | DDB_G0274785 | DDB_G0274785 | TMF1-like protein |
| inparanoid | 58185 | DDB_G0274347 | DDB_G0274347 |  |
| inparanoid | 58305 | DDB_G0268696 | DDB_G0268696 |  |
| inparanoid | 58324 | DDB_G0271642 | cks1 | cyclin-dependent kinases regulatory subunit |
| inparanoid | 58333 | DDB_G0283319 | ap4b1 | adaptor-related protein complex 4, beta 1 subunit, beta adaptin |
| inparanoid | 58337 | DDB_G0284669 | prsA | phosphoribosyl pyrophosphate synthetase |
| inparanoid | 58348 | DDB_G0288145 | purL | phosphoribosylformylglycinamide synthase, PFAS |
| inparanoid | 58371 | DDB_G0283587 | CSN1 | COP9 signalosome complex subunit 1, proteasome component region PCI (PINT) domain-containing protein |
| inparanoid | 5838 | DDB_G0290759 | eIF2b4 | eIF2B GDP-GTP exchange factor, translation initiation factor eIF2B delta subunit |
| inparanoid | 58424 | DDB_G0288373 | efbA | elongation factor 2 |
| inparanoid | 58428 | DDB_G0288921 | hspI | heat shock protein Hsp20 domain-containing protein, putative alpha-crystallin-type heat shock protein |
| inparanoid | 58429 | DDB_G0274157 | DDB_G0274157 |  |
| inparanoid | 58447 | DDB_G0287777 | sec61g | protein transport protein SEC61 gamma subunit |
| inparanoid | 58449 | DDB_G0287693 | ubc9 | putative E2 enzyme Ubc9 |
| inparanoid | 58458 | DDB_G0282255 | DDB_G0282255 | carbohydrate-binding domain-containing protein, CMP/dCMP deaminase, zinc-binding domain-containing protein |
| inparanoid | 58479 | DDB_G0269854 | amdhd1 | imidazolonepropionase, amidohydrolase domain containing protein 1 |
| inparanoid | 58486 | DDB_G0282999 | maoD | amine oxidase (flavin-containing), monoamine oxidase |
| inparanoid | 58510 | DDB_G0269908 | DDB_G0269908 | Fatty acid hydroxylase FAH1P (AT2G34770/T29F13.2). |
| inparanoid | 58541 | DDB_G0279189 | rpl11 | S60 ribosomal protein L11 |
| inparanoid | 58605 | DDB_G0288523 | DDB_G0288523 | Putative acetyltransferase protein. |
| inparanoid | 58624 | DDB_G0273471 | dpp3-1 | dipeptidyl-peptidase III |
| inparanoid | 58639 | DDB_G0271868 | gefS | Ras guanine nucleotide exchange factor |
| inparanoid | 58645 | DDB_G0281589 | DDB_G0281589 | COBW domain-containing protein, cobalamin (vitamin B12) biosynthesis family protein, cobalamin synthesis protein/P47K family protein |
| inparanoid | 58676 | DDB_G0267442 | cdk8 | protein serine/threonine kinase, CDK family protein kinase, cyclin-dependent kinase, CTD kinase, protein kinase, CMGC group |
| inparanoid | 58708 | DDB_G0291434 | pdi2 | protein disulfide isomerase |
| inparanoid | 58748 | DDB_G0292448 | DDB_G0292448 | Hypothetical Generic methyl-transferase/SAM. |
| inparanoid | 58751 | DDB_G0292378 | fhbA | flavohemoglobin |
| inparanoid | 58785 | DDB_G0282359 | ppiA | cyclophilin-type peptidylprolyl cis-trans isomerase (PPIase) |
| inparanoid | 58802 | DDB_G0277905 | snfA | putative protein serine/threonine kinase, SNF/AMP-activated kinase (AMPK), protein kinase, CAMK group, CAMKL family protein kinase, putative CAM kinase, AMP-activated protein kinase alpha subunit, AMPK complex alpha subunit |
| inparanoid | 58815 | DDB_G0276973 | nramp1 | solute carrier family 11 member 1, natural resistance-associated macrophage protein |
| inparanoid | 58819 | DDB_G0267650 | ireA | putative protein serine/threonine kinase, putative endoribonuclease, IRE family protein kinase |
| inparanoid | 58822 | DDB_G0272128 | DDB_G0272128 | 3-hydroxyacyl-CoA dehydrogenase type II |
| inparanoid | 58838 | DDB_G0272062 | DDB_G0272062 |  |
| inparanoid | 58841 | DDB_G0291524 | DDB_G0291524 | putative alpha-N-acetylgalactosaminidase, glycoside hydrolase family 27 protein |
| inparanoid | 58843 | DDB_G0272342 | DDB_G0272342 | Similar to Mus musculus (Mouse). DnaJ homolog subfamily B member 5 (Heat shock protein Hsp40-3) (Heat shock protein cognate 40) (Hsc40). |
| inparanoid | 58856 | DDB_G0279443 | DDB_G0279443 |  |
| inparanoid | 58874 | DDB_G0277477 | DDB_G0277477 | acyl-CoA oxidase |
| inparanoid | 58919 | DDB_G0282743 | haao | 3-hydroxyanthranilate 3,4-dioxygenase |
| inparanoid | 58924 | DDB_G0271354 | DDB_G0271354 |  |
| inparanoid | 58958 | DDB_G0283345 | comD | major facilitator superfamily protein, drug resistance transporter, EmrB/QacA subfamily protein |
| inparanoid | 58973 | DDB_G0271906 | mgluS | glutamate-tRNA ligase, glutamyl-tRNA synthetase, glutamate-tRNA ligase, mitochondrial, glutamyl-tRNA synthetase, mitochondrial |
| inparanoid | 58977 | DDB_G0271726 | DDB_G0271726 | Putative Ligase |
| inparanoid | 59006 | DDB_G0274739 | DDB_G0274739 | Similar to plasmodium falciparum. cell cycle regulator with zn-finger domain, putative. |
| inparanoid | 59013 | DDB_G0292782 | mybN | putative myb transcription factor |
| inparanoid | 59022 | DDB_G0293552 | DDB_G0293552 | AFR050Wp. |
| inparanoid | 59030 | DDB_G0277273 | phg1b | putative phagocytic receptor 1b |
| inparanoid | 59083 | DDB_G0292264 | nosA | U box domain-containing protein, ubiquitin-protein ligase (E3) |
| inparanoid | 59134 | DDB_G0291237 | rapA | Ras GTPase |
| inparanoid | 59136 | DDB_G0288827 | DDB_G0288827 |  |
| inparanoid | 59149 | DDB_G0286473 | pfdn3 | prefoldin alpha-like domain containing protein, prefoldin subunit 3 |
| inparanoid | 59156 | DDB_G0291428 | DDB_G0291428 | paramecium surface antigen repeat-containing protein |
| inparanoid | 59184 | DDB_G0277115 | DDB_G0277115 | doublecortin domain-containing protein |
| inparanoid | 59190 | DDB_G0277911 | mfeB | hypothetical peroxisomal multifunctional enzyme 2 |
| inparanoid | 59197 | DDB_G0284661 | DDB_G0284661 | putative protein serine/threonine kinase, CAMK1 family protein kinase, protein kinase, CAMK group, putative CAM kinase |
| inparanoid | 59227 | DDB_G0284569 | DDB_G0284569 |  |
| inparanoid | 59260 | DDB_G0272462 | qprt | nicotinate-nucleotide diphosphorylase (carboxylating) |
| inparanoid | 59316 | DDB_G0269372 | DDB_G0269372 |  |
| inparanoid | 59352 | DDB_G0275473 | rps24 | 40S ribosomal protein S24 |
| inparanoid | 59395 | DDB_G0282311 | DDB_G0282311 | major facilitator superfamily protein, MFS family protein |
| inparanoid | 59401 | DDB_G0272935 | mocos | molybdenum cofactor sulfurase |
| inparanoid | 5958 | DDB_G0289861 | DDB_G0289861 | unknown |
| inparanoid | 5960 | DDB_G0284783 | DDB_G0284783 |  |
| inparanoid | 6001 | DDB_G0274325 | ddx52 | putative RNA helicase, DEAD/DEAH box helicase |
| inparanoid | 6034 | DDB_G0276767 | plbA | phospholipase B |
| inparanoid | 6061 | DDB_G0268228 | DDB_G0268228 |  |
| inparanoid | 6126 | DDB_G0288659 | wdr18 | WD40 repeat-containing protein |
| inparanoid | 6215 | DDB_G0281229 | PNKP | SAP DNA-binding domain-containing protein, putative bifunctional polynucleotide phosphatase/kinase |
| inparanoid | 6223 | DDB_G0282157 | DDB_G0282157 |  |
| inparanoid | 6368 | DDB_G0275815 | DDB_G0275815 | natural resistance-associated macrophage protein |
| inparanoid | 6436 | DDB_G0285535 | DDB_G0285535 |  |
| inparanoid | 6501 | DDB_G0270582 | DDB_G0270582 |  |
| inparanoid | 6688 | DDB_G0271902 | pscA | putative peptidase S13, D-Ala-D-Ala carboxypeptidase C, carboxypeptidase |
| inparanoid | 6769 | DDB_G0284969 | DDB_G0284969 | tetratricopeptide-like helical domain-containing protein (TPR), Sel1-like repeat-containing protein |
| inparanoid | 6794 | DDB_G0281215 | brf1 | transcription factor IIIB, TATA box-binding protein-associated factor, RNA polymerase III, subunit 2 |
| inparanoid | 6811 | DDB_G0281483 | ppr1 | protein phosphatase 4 regulatory subunit 1 |
| inparanoid | 6820 | DDB_G0267872 | DDB_G0267872 | NOL1/NOP2/Sun family protein |
| inparanoid | 6958 | DDB_G0281061 | wdr4 | WD40 repeat-containing protein |
| inparanoid | 7134 | DDB_G0280147 | ddx47 | putative RNA helicase, DEAD/DEAH box helicase |
| inparanoid | 7152 | DDB_G0283633 | DDB_G0283633 | At3g49310. |
| inparanoid | 7189 | DDB_G0275613 | DDB_G0275613 | METHIONYL-TRNA SYNTHETASE BETA SUBUNIT. 6/101 |
| inparanoid | 7229 | DDB_G0276293 | DDB_G0276293 | putative transmembrane protein |
| inparanoid | 72325 | DDB_G0277535 | rgn | senescence marker protein-30 family protein, regucalcin |
| inparanoid | 72350 | DDB_G0275381 | DDB_G0275381 |  |
| inparanoid | 72456 | DDB_G0269334 | DDB_G0269334 | C2 calcium/lipid-binding (CaLB) region-containing protein, C2 calcium-dependent membrane targeting domain-containing protein |
| inparanoid | 7275 | DDB_G0268748 | DDB_G0268748 | putative actin fragmin kinase, putative AFK family protein kinase |
| inparanoid | 73130 | DDB_G0279959 | DDB_G0279959 |  |
| inparanoid | 73189 | DDB_G0281729 | DDB_G0281729 |  |
| inparanoid | 7342 | DDB_G0286069 | ku70 | ATP-dependent DNA helicase, DNA-dependent protein kinase (DNAPK) subunit |
| inparanoid | 73849 | DDB_G0274959 | DDB_G0274959 | Similar to Synechocystis sp. (Strain PCC 6803). 50S ribosomal protein L13. |
| inparanoid | 73850 | DDB_G0274961 | DDB_G0274961 |  |
| inparanoid | 73870 | DDB_G0295827 | DDB_G0295827 | leucine-rich repeat-containing protein (LRR) |
| inparanoid | 73875 | DDB_G0275419 | DDB_G0275419 |  |
| inparanoid | 73885 | DDB_G0289973 | DDB_G0289973 |  |
| inparanoid | 73982 | DDB_G0272825 | rps4 | 40S ribosomal protein S4 |
| inparanoid | 73996 | DDB_G0278233 | DDB_G0278233 |  |
| inparanoid | 73997 | DDB_G0294557 | DDB_G0294557 | putative actin binding protein |
| inparanoid | 74029 | DDB_G0272152 | DDB_G0272152 | unknown |
| inparanoid | 74044 | DDB_G0268336 | DDB_G0268336 |  |
| inparanoid | 74059 | DDB_G0269226 | cas1 | cycloartenol synthase |
| inparanoid | 74070 | DDB_G0270470 | DDB_G0270470 |  |
| inparanoid | 74075 | DDB_G0269300 | DDB_G0269300 |  |
| inparanoid | 74089 | DDB_G0275923 | DDB_G0275923 |  |
| inparanoid | 74095 | DDB_G0270176 | DDB_G0270176 | protein phosphatase 2C, leucine-rich repeat-containing protein (LRR) |
| inparanoid | 74100 | DDB_G0267880 | DDB_G0267880 | Transcriptional repressor of sporulation, septation and degradation. |
| inparanoid | 74104 | DDB_G0268932 | DDB_G0268932 |  |
| inparanoid | 74112 | DDB_G0269952 | DDB_G0269952 |  |
| inparanoid | 74124 | DDB_G0269728 | ciao1 | WD40 repeat-containing protein, putative cytosolic iron-sulfur assembly |
| inparanoid | 74128 | DDB_G0269720 | abcB7 | ABC transporter B family protein |
| inparanoid | 74149 | DDB_G0293718 | DDB_G0293718 |  |
| inparanoid | 74166 | DDB_G0268252 | DDB_G0268252 |  |
| inparanoid | 74181 | DDB_G0281873 | pfdn4 | prefoldin beta-like domain containing protein, prefoldin subunit 4 |
| inparanoid | 74198 | DDB_G0282009 | ahhA | alpha adducin |
| inparanoid | 74307 | DDB_G0278705 | DDB_G0278705 |  |
| inparanoid | 74311 | DDB_G0280827 | trappc4 | trafficking protein particle complex subunit 4 |
| inparanoid | 74314 | DDB_G0283041 | DDB_G0283041 |  |
| inparanoid | 74321 | DDB_G0282929 | atg12 | autophagy protein 12 |
| inparanoid | 74322 | DDB_G0283037 | esd | esterase, S-formylglutathione hydrolase |
| inparanoid | 74327 | DDB_G0283373 | DDB_G0283373 |  |
| inparanoid | 74328 | DDB_G0283279 | cdk11 | putative protein serine/threonine kinase, CDK family protein kinase, cyclin-dependent kinase, protein kinase, CMGC group, PITSLRE subfamily protein kinase |
| inparanoid | 74341 | DDB_G0283989 | DDB_G0283989 | unknown |
| inparanoid | 74375 | DDB_G0278473 | DDB_G0278473 |  |
| inparanoid | 74399 | DDB_G0274333 | DDB_G0274333 |  |
| inparanoid | 74415 | DDB_G0274425 | DDB_G0274425 | protein phosphatase 2C-related protein |
| inparanoid | 74422 | DDB_G0274657 | DDB_G0274657 | glutaredoxin-related family protein |
| inparanoid | 74424 | DDB_G0274479 | DDB_G0274479 |  |
| inparanoid | 74430 | DDB_G0270308 | DDB_G0270308 |  |
| inparanoid | 74432 | DDB_G0274489 | DDB_G0274489 |  |
| inparanoid | 74448 | DDB_G0267950 | copE | coatomer protein complex epsilon subunit |
| inparanoid | 74459 | DDB_G0282005 | DDB_G0282005 | phospholipase/carboxylesterase family protein |
| inparanoid | 74464 | DDB_G0275169 | DDB_G0275169 | Similar to Y51A2D.19.p. |
| inparanoid | 74466 | DDB_G0275059 | DDB_G0275059 |  |
| inparanoid | 74467 | DDB_G0275061 | DDB_G0275061 | Putative Permease |
| inparanoid | 7448 | DDB_G0285339 | mipp1 | multiple inositol polyphosphate phosphatase |
| inparanoid | 74489 | DDB_G0267434 | DDB_G0267434 | unknown |
| inparanoid | 74537 | DDB_G0292876 | DDB_G0292876 |  |
| inparanoid | 74538 | DDB_G0292878 | DDB_G0292878 |  |
| inparanoid | 74539 | DDB_G0292504 | rad17 |  |
| inparanoid | 74540 | DDB_G0292502 | DDB_G0292502 |  |
| inparanoid | 74542 | DDB_G0292868 | golt1 | golgi transport protein 1 |
| inparanoid | 74560 | DDB_G0275673 | DDB_G0275673 |  |
| inparanoid | 74595 | DDB_G0278501 | DDB_G0278501 |  |
| inparanoid | 74614 | DDB_G0292638 | DDB_G0292638 |  |
| inparanoid | 74620 | DDB_G0279501 | DDB_G0279501 |  |
| inparanoid | 7463 | DDB_G0287003 | DDB_G0287003 | putative transmembrane protein, solute carrier family 35 member protein, DUF914 family protein |
| inparanoid | 74635 | DDB_G0292108 | DDB_G0292108 |  |
| inparanoid | 7467 | DDB_G0269822 | mhisS | histidine-tRNA ligase, histidyl-tRNA synthetase, putative mitochondrial histidyl-tRNA synthetase, putative mitochondrial histidine-tRNA ligase |
| inparanoid | 74692 | DDB_G0284203 | kynu | kynureninase, L-kynurenine hydrolase |
| inparanoid | 74712 | DDB_G0283603 | rab32A | Rab GTPase |
| inparanoid | 74736 | DDB_G0284649 | DDB_G0284649 |  |
| inparanoid | 74743 | DDB_G0272957 | DDB_G0272957 |  |
| inparanoid | 74764 | DDB_G0271702 | DDB_G0271702 |  |
| inparanoid | 74768 | DDB_G0271378 | DDB_G0271378 | tRNA ADENILYL transferase. |
| inparanoid | 74807 | DDB_G0292568 | DDB_G0292568 |  |
| inparanoid | 74818 | DDB_G0285781 | DDB_G0285781 | unknown |
| inparanoid | 74854 | DDB_G0284817 | DDB_G0284817 |  |
| inparanoid | 74855 | DDB_G0278215 | DDB_G0278215 |  |
| inparanoid | 74868 | DDB_G0279287 | DDB_G0279287 | cysteine desulfurase, mitochondrial precursor |
| inparanoid | 74876 | DDB_G0268074 | DDB_G0268074 | Q9FY46 Sulfate transporter 4.1, chloroplast precursor (AST82). |
| inparanoid | 74887 | DDB_G0286049 | zntC | zinc/iron permease, zinc transporter |
| inparanoid | 74898 | DDB_G0279179 | DDB_G0279179 |  |
| inparanoid | 74901 | DDB_G0278961 | DDB_G0278961 |  |
| inparanoid | 74905 | DDB_G0291808 | DDB_G0291808 |  |
| inparanoid | 74907 | DDB_G0278939 | DDB_G0278939 |  |
| inparanoid | 74916 | DDB_G0292750 | DDB_G0292750 |  |
| inparanoid | 74923 | DDB_G0293860 | DDB_G0293860 |  |
| inparanoid | 74948 | DDB_G0293980 | DDB_G0293980 |  |
| inparanoid | 74956 | DDB_G0293904 | redA | NADPH-cytochrome-P450 oxidoreductase |
| inparanoid | 74957 | DDB_G0293988 | DDB_G0293988 |  |
| inparanoid | 74971 | DDB_G0284137 | DDB_G0284137 |  |
| inparanoid | 74972 | DDB_G0276441 | rpl7 | S60 ribosomal protein L7 |
| inparanoid | 74994 | DDB_G0276079 | mproS | prolyl-tRNA synthetase, proline-tRNA ligase, putative mitochondrial prolyl-tRNA synthetase, putative mitochondrial proline-tRNA ligase |
| inparanoid | 75040 | DDB_G0282819 | sglA | sphingosine-1-phosphate lyase, S1P lyase |
| inparanoid | 75049 | DDB_G0282859 | DDB_G0282859 |  |
| inparanoid | 75055 | DDB_G0292662 | DDB_G0292662 |  |
| inparanoid | 75065 | DDB_G0282525 | DDB_G0282525 |  |
| inparanoid | 75067 | DDB_G0282527 | pemtA | phospholipid methyltransferase family protein, phosphatidylethanolamine N-methyltransferase |
| inparanoid | 75077 | DDB_G0269232 | DG1007 | unknown |
| inparanoid | 75082 | DDB_G0282479 | DDB_G0282479 |  |
| inparanoid | 75094 | DDB_G0279205 | DDB_G0279205 | CAATT-binding protein |
| inparanoid | 75104 | DDB_G0288459 | DDB_G0288459 |  |
| inparanoid | 75115 | DDB_G0293030 | mileS | isoleucyl-tRNA synthetase, isoleucine-tRNA ligase, isoleucyl-tRNA synthetase, mitochondrial, isoleucine-tRNA ligase, mitochondrial |
| inparanoid | 75136 | DDB_G0277329 | DDB_G0277329 |  |
| inparanoid | 75147 | DDB_G0275401 | DDB_G0275401 |  |
| inparanoid | 75170 | DDB_G0277103 | DDB_G0277103 |  |
| inparanoid | 75179 | DDB_G0277237 | DDB_G0277237 |  |
| inparanoid | 75205 | DDB_G0275095 | mgmt | methylated-DNA-[protein]-cysteine S-methyltransferase, 6-O-methylguanine-DNA methyltransferase |
| inparanoid | 75206 | DDB_G0268498 | DDB_G0268498 | unknown |
| inparanoid | 75207 | DDB_G0267686 | DDB_G0267686 | RGS domain-containing protein, protein kinase, TKL group, tyrosine kinase-like protein, ARK family protein kinase |
| inparanoid | 75211 | DDB_G0267670 | DDB_G0267670 |  |
| inparanoid | 75212 | DDB_G0267668 | hspL | heat shock protein Hsp20 domain-containing protein, putative alpha-crystallin-type heat shock protein |
| inparanoid | 75224 | DDB_G0268130 | DDB_G0268130 |  |
| inparanoid | 75236 | DDB_G0267534 | DDB_G0267534 |  |
| inparanoid | 75248 | DDB_G0277543 | DDB_G0277543 | CCCH-type zinc finger-containing protein |
| inparanoid | 75271 | DDB_G0285991 | DDB_G0285991 |  |
| inparanoid | 75282 | DDB_G0268196 | DDB_G0268196 |  |
| inparanoid | 75292 | DDB_G0285519 | DDB_G0285519 |  |
| inparanoid | 75301 | DDB_G0285563 | DDB_G0285563 |  |
| inparanoid | 75413 | DDB_G0269442 | DDB_G0269442 | P87053 F-box/WD-repeat protein pof1 (Skp1-binding protein 1). |
| inparanoid | 75426 | DDB_G0270510 | spc2 | microsomal signal peptidase subunit, signal peptidase complex subunit 2 |
| inparanoid | 75450 | DDB_G0270268 | DDB_G0270268 |  |
| inparanoid | 75462 | DDB_G0269220 | DDB_G0269220 | cytochrome c oxidase subunit VIb |
| inparanoid | 75469 | DDB_G0269860 | DDB_G0269860 | OSJNBa0008A08.6 protein. |
| inparanoid | 75478 | DDB_G0270178 | wdr85 | WD40 repeat-containing protein |
| inparanoid | 75483 | DDB_G0270182 | DDB_G0270182 |  |
| inparanoid | 75487 | DDB_G0270752 | DDB_G0270752 | cytochrome b5 domain-containing protein |
| inparanoid | 75488 | DDB_G0270196 | DDB_G0270196 | putative mitochondrial import inner membrane translocase subunit 50, NIF domain-containing protein |
| inparanoid | 75494 | DDB_G0270170 | DDB_G0270170 | bromodomain-containing protein, protein kinase, Atypical group, BRD family protein kinase, GTE group protein |
| inparanoid | 75527 | DDB_G0290993 | DDB_G0290993 |  |
| inparanoid | 75529 | DDB_G0290997 | DDB_G0290997 | peptidase M16 family protein |
| inparanoid | 75550 | DDB_G0287753 | DDB_G0287753 |  |
| inparanoid | 75557 | DDB_G0291059 | DDB_G0291059 | ankyrin repeat-containing protein, K homology (KH), type 1 domain containing-protein |
| inparanoid | 75571 | DDB_G0284367 | DDB_G0284367 | phosphoesterase, PA-phosphatase related-family protein |
| inparanoid | 75576 | DDB_G0285365 | DDB_G0285365 |  |
| inparanoid | 75579 | DDB_G0285359 | DDB_G0285359 |  |
| inparanoid | 75581 | DDB_G0285355 | DDB_G0285355 |  |
| inparanoid | 75590 | DDB_G0290425 | DDB_G0290425 |  |
| inparanoid | 75595 | DDB_G0290417 | chid1 | chitinase domain-containing protein 1, glycosyl hydrolase 18 family protein |
| inparanoid | 75603 | DDB_G0290801 | DDB_G0290801 |  |
| inparanoid | 75605 | DDB_G0290927 | etfa | electron transfer flavoprotein alpha subunit |
| inparanoid | 75617 | DDB_G0285373 | mybL | putative myb transcription factor |
| inparanoid | 75630 | DDB_G0293118 | DDB_G0293118 |  |
| inparanoid | 75664 | DDB_G0293044 | DDB_G0293044 |  |
| inparanoid | 75665 | DDB_G0287463 | DDB_G0287463 |  |
| inparanoid | 75670 | DDB_G0293556 | mcfG | mitochondrial substrate carrier family protein, mitochondrial carnitine/acylcarnitine carrier protein, putative mitochondrial carnitine/acylcarnitine transporter |
| inparanoid | 75673 | DDB_G0293704 | DDB_G0293704 | seven transmembrane domain protein |
| inparanoid | 75678 | DDB_G0293440 | DDB_G0293440 |  |
| inparanoid | 75689 | DDB_G0293404 | DDB_G0293404 |  |
| inparanoid | 75690 | DDB_G0293414 | DDB_G0293414 | P0701D05.6 protein. |
| inparanoid | 75698 | DDB_G0289863 | DDB_G0289863 |  |
| inparanoid | 75703 | DDB_G0285725 | dhps | deoxyhypusine synthase |
| inparanoid | 75733 | DDB_G0287445 | DDB_G0287445 | unknown |
| inparanoid | 75798 | DDB_G0269182 | rsc5 | cellular retinaldehyde-binding/triple function domain-containing protein |
| inparanoid | 75859 | DDB_G0271280 | DDB_G0271280 |  |
| inparanoid | 75869 | DDB_G0271486 | DDB_G0271486 |  |
| inparanoid | 75877 | DDB_G0271290 | DDB_G0271290 |  |
| inparanoid | 75879 | DDB_G0272819 | hspC | heat shock protein |
| inparanoid | 75901 | DDB_G0271248 | DDB_G0271248 | Q8IVH4 Methylmalonic aciduria type A protein, mitochondrial precursor. |
| inparanoid | 75973 | DDB_G0273163 | psmB4-1 | proteasome subunit beta type 4, 20S proteasome subunit beta-4 |
| inparanoid | 75974 | DDB_G0273165 | DDB_G0273165 | endonuclease V |
| inparanoid | 75989 | DDB_G0271738 | tubC | gamma tubulin, tubulin gamma-2 chain |
| inparanoid | 75993 | DDB_G0271790 | DDB_G0271790 |  |
| inparanoid | 76006 | DDB_G0267570 | DDB_G0267570 |  |
| inparanoid | 76062 | DDB_G0286919 | uba2 | sumo-activating enzyme subunit 2, ubiquitin-like 1-activating enzyme E1B |
| inparanoid | 76067 | DDB_G0287519 | DDB_G0287519 | acetyl-coA hydrolase |
| inparanoid | 76077 | DDB_G0288291 | DDB_G0288291 |  |
| inparanoid | 76088 | DDB_G0279687 | DDB_G0279687 |  |
| inparanoid | 76093 | DDB_G0278567 | DDB_G0278567 | cytochrome b5 reductase |
| inparanoid | 76108 | DDB_G0288187 | ddi1 | ubiquitin-associated (UBA) domain-containing protein |
| inparanoid | 76111 | DDB_G0270430 | wipA | WH2 domain-containing protein |
| inparanoid | 76134 | DDB_G0284017 | ddx10 | putative RNA helicase, DEAD/DEAH box helicase |
| inparanoid | 76139 | DDB_G0270890 | DDB_G0270890 |  |
| inparanoid | 76141 | DDB_G0269602 | DDB_G0269602 | SD04658p. |
| inparanoid | 76145 | DDB_G0270560 | mybP | myb domain-containing protein |
| inparanoid | 76154 | DDB_G0269630 | DDB_G0269630 | tatD-related DNAse, TatD-related deoxyribonuclease |
| inparanoid | 76169 | DDB_G0269346 | DDB_G0269346 |  |
| inparanoid | 76173 | DDB_G0279831 | DDB_G0279831 | putative protein serine/threonine kinase, CAMK1 family protein kinase, protein kinase, CAMK group |
| inparanoid | 76175 | DDB_G0278235 | DDB_G0278235 |  |
| inparanoid | 76180 | DDB_G0267754 | DDB_G0267754 |  |
| inparanoid | 76185 | DDB_G0281057 | DDB_G0281057 |  |
| inparanoid | 76207 | DDB_G0280243 | DDB_G0280243 |  |
| inparanoid | 76233 | DDB_G0279475 | DDB_G0279475 |  |
| inparanoid | 76235 | DDB_G0279485 | DDB_G0279485 |  |
| inparanoid | 76255 | DDB_G0284523 | DDB_G0284523 |  |
| inparanoid | 76293 | DDB_G0279985 | DDB_G0279985 | putative transmembrane protein |
| inparanoid | 76324 | DDB_G0285853 | DDB_G0285853 | DNA polymerase alpha primase subunit, DNA primase small subunit |
| inparanoid | 76335 | DDB_G0290879 | DDB_G0290879 |  |
| inparanoid | 76343 | DDB_G0285243 | DDB_G0285243 | putative splicing endonuclease |
| inparanoid | 76363 | DDB_G0277743 | guaD | guanine deaminase |
| inparanoid | 76364 | DDB_G0277745 | DDB_G0277745 | HAD superfamily hydrolase-like, type 3 |
| inparanoid | 76416 | DDB_G0278141 | vps11 | RING zinc finger-containing protein, tetratricopeptide-like helical domain-containing protein (TPR), 7-fold repeat in clathrin and VPS proteins repeat-containing protein |
| inparanoid | 76420 | DDB_G0290289 | DDB_G0290289 |  |
| inparanoid | 76423 | DDB_G0290351 | DDB_G0290351 |  |
| inparanoid | 76426 | DDB_G0290295 | DDB_G0290295 |  |
| inparanoid | 76443 | DDB_G0284443 | DDB_G0284443 |  |
| inparanoid | 76451 | DDB_G0283405 | bdp1 | myb domain-containing protein, putative RNA polymerase III transcription factor TFIIIB (90 kDa subunit) |
| inparanoid | 76474 | DDB_G0272116 | ppp4C | protein phosphatase 4 catalytic subunit |
| inparanoid | 76504 | DDB_G0281609 | DDB_G0281609 |  |
| inparanoid | 76529 | DDB_G0291312 | mcfA | transmembrane protein, mitochondrial substrate carrier family protein |
| inparanoid | 76538 | DDB_G0279087 | med22 | putative mediator complex subunit 22 |
| inparanoid | 76545 | DDB_G0291310 | DDB_G0291310 | Q13868 Exosome complex exonuclease RRP4 (EC 3.1.13.-) (Ribosomal RNA processing protein 4) (Exosome component 2). |
| inparanoid | 76549 | DDB_G0291334 | DDB_G0291334 |  |
| inparanoid | 76560 | DDB_G0275973 | DDB_G0275973 |  |
| inparanoid | 76580 | DDB_G0275025 | DDB_G0275025 |  |
| inparanoid | 76622 | DDB_G0283635 | wdr89 | WD40 repeat-containing protein |
| inparanoid | 76639 | DDB_G0279223 | DDB_G0279223 | OB fold-containing protein, nucleic acid binding |
| inparanoid | 76648 | DDB_G0283019 | DDB_G0283019 | BRCA2 repeat-containing protein |
| inparanoid | 76653 | DDB_G0288589 | DDB_G0288589 |  |
| inparanoid | 76655 | DDB_G0281419 | DDB_G0281419 |  |
| inparanoid | 76658 | DDB_G0276405 | PFP1 | DJ-1/ThiJ/PfpI family protein, peptidase C56 family protein |
| inparanoid | 76668 | DDB_G0280255 | mdhC | malate dehydrogenase |
| inparanoid | 76676 | DDB_G0280083 | mcfJ | mitochondrial substrate carrier family protein |
| inparanoid | 76684 | DDB_G0287165 | DDB_G0287165 | AAA ATPase domain-containing protein, MIT domain-containing protein |
| inparanoid | 76703 | DDB_G0269462 | DDB_G0269462 | ubiquitin domain-containing protein |
| inparanoid | 76707 | DDB_G0269470 | mcfF | mitochondrial substrate carrier family protein, putative mitoferrin, putative mitochondrial iron transporter |
| inparanoid | 76739 | DDB_G0270096 | DDB_G0270096 |  |
| inparanoid | 76769 | DDB_G0269676 | DDB_G0269676 | Putative nucleolar protein. |
| inparanoid | 76787 | DDB_G0267630 | kil1 | sulfotransferase |
| inparanoid | 76788 | DDB_G0267620 | DDB_G0267620 |  |
| inparanoid | 76798 | DDB_G0291376 | adi1 | ARD/ARD' family protein, acireductone dioxygenase 1 |
| inparanoid | 76800 | DDB_G0272438 | DDB_G0272438 |  |
| inparanoid | 76816 | DDB_G0276057 | DDB_G0276057 | ZZ-type zinc finger-containing protein, thioredoxin domain-containing protein, UBX domain-containing protein, UBA domain-containing protein |
| inparanoid | 76826 | DDB_G0276627 | DDB_G0276627 |  |
| inparanoid | 76837 | DDB_G0288787 | vps29 | metallophosphoesterase domain-containing protein, vacuolar sorting protein 29 |
| inparanoid | 76866 | DDB_G0280775 | rvb2 | AAA ATPase domain-containing protein, RuvB-like protein 2 |
| inparanoid | 7687 | DDB_G0276083 | DDB_G0276083 | carboxylic ester hydrolase |
| inparanoid | 76871 | DDB_G0280403 | DDB_G0280403 |  |
| inparanoid | 76878 | DDB_G0275581 | DDB_G0275581 |  |
| inparanoid | 76894 | DDB_G0280945 | DDB_G0280945 |  |
| inparanoid | 76895 | DDB_G0280911 | DDB_G0280911 |  |
| inparanoid | 76929 | DDB_G0279361 | DDB_G0279361 |  |
| inparanoid | 76935 | DDB_G0284557 | DDB_G0284557 | gluconokinase, gluconate kinase |
| inparanoid | 76939 | DDB_G0284545 | sgkB | sphingosine kinase |
| inparanoid | 76963 | DDB_G0278199 | DDB_G0278199 |  |
| inparanoid | 76975 | DDB_G0279699 | DDB_G0279699 | HAM group protein, MYST family protein, MOZ/SAS-like domain-containing protein, putative histone acetyltransferase |
| inparanoid | 76980 | DDB_G0282761 | DDB_G0282761 | unknown |
| inparanoid | 76997 | DDB_G0295721 | nat13 | GCN5-related N-acetyltransferase |
| inparanoid | 77009 | DDB_G0278241 | tcea1 | RNA polymerase II elongation factor |
| inparanoid | 77015 | DDB_G0278027 | snrpD1 | LSM (like-Sm) domain-containing protein, putative small nuclear ribonucleoprotein D1 |
| inparanoid | 77028 | DDB_G0280747 | DDB_G0280747 |  |
| inparanoid | 77048 | DDB_G0293178 | DDB_G0293178 |  |
| inparanoid | 77060 | DDB_G0275599 | DDB_G0275599 | DUF599 family protein |
| inparanoid | 77063 | DDB_G0275595 | DDB_G0275595 |  |
| inparanoid | 77082 | DDB_G0277289 | pigH | putative glycosyltransferase, putative GlcNAc transferase, phosphatidylinositol glycan, class H |
| inparanoid | 77109 | DDB_G0288237 | DDB_G0288237 | putative guanine nucleotide exchange factor (GEF), DENN domain-containing protein |
| inparanoid | 77133 | DDB_G0290087 | vps2A | SNF7 family protein |
| inparanoid | 77138 | DDB_G0278367 | DDB_G0278367 | putative DNA polymerase epsilon subunit B |
| inparanoid | 77142 | DDB_G0278375 | DDB_G0278375 |  |
| inparanoid | 77144 | DDB_G0278455 | DDB_G0278455 |  |
| inparanoid | 77157 | DDB_G0278353 | smu1 | WD40 repeat-containing protein, lissencephaly type-1-like homology (LisH) motif-containing protein, suppressor of mec-8 and unc-52-like protein |
| inparanoid | 77187 | DDB_G0276841 | DDB_G0276841 | unknown |
| inparanoid | 77194 | DDB_G0269500 | DDB_G0269500 |  |
| inparanoid | 77206 | DDB_G0280921 | DDB_G0280921 | Phox domain-containing protein |
| inparanoid | 77221 | DDB_G0281217 | DDB_G0281217 |  |
| inparanoid | 77225 | DDB_G0281223 | DDB_G0281223 |  |
| inparanoid | 77246 | DDB_G0285675 | DDB_G0285675 |  |
| inparanoid | 77250 | DDB_G0285671 | DDB_G0285671 |  |
| inparanoid | 77260 | DDB_G0283407 | DDB_G0283407 |  |
| inparanoid | 77267 | DDB_G0291596 | wdr13 | WD40 repeat-containing protein |
| inparanoid | 77275 | DDB_G0285029 | sfxn | sideroflexin, tricarboxylate carrier (MTC) family protein |
| inparanoid | 77276 | DDB_G0285031 | DDB_G0285031 |  |
| inparanoid | 77300 | DDB_G0274895 | DDB_G0274895 |  |
| inparanoid | 77304 | DDB_G0293294 | DDB_G0293294 |  |
| inparanoid | 77338 | DDB_G0291860 | DDB_G0291860 | unknown |
| inparanoid | 77398 | DDB_G0288623 | cbpH | calcium-binding protein |
| inparanoid | 77411 | DDB_G0277451 | mgm101 | mitochondrial genome maintenance protein |
| inparanoid | 77414 | DDB_G0277113 | pex16 | peroxisomal biogenesis factor 16, peroxin 16 |
| inparanoid | 77418 | DDB_G0284323 | ppil2 | cyclophilin-type peptidylprolyl cis-trans isomerase (PPIase), Ubox domain-containing protein |
| inparanoid | 77419 | DDB_G0284303 | DDB_G0284303 | cellulose-binding domain-containing protein |
| inparanoid | 77422 | DDB_G0284263 | DDB_G0284263 | DNAJ heat shock N-terminal domain-containing protein |
| inparanoid | 77426 | DDB_G0284273 | DDB_G0284273 |  |
| inparanoid | 77429 | DDB_G0284187 | Dd5P2 | inositol 5-phosphatase |
| inparanoid | 77441 | DDB_G0285135 | DDB_G0285135 |  |
| inparanoid | 77484 | DDB_G0279805 | DDB_G0279805 |  |
| inparanoid | 77505 | DDB_G0268816 | DDB_G0268816 |  |
| inparanoid | 77507 | DDB_G0292632 | folC | tetrahydrofolylpolyglutamate synthase, folylpolyglutamate synthase |
| inparanoid | 77520 | DDB_G0293234 | DDB_G0293234 | N-acetyltransferase, non-catalytic subunit |
| inparanoid | 77529 | DDB_G0282189 | tupA | transcriptional repressor TUP1 |
| inparanoid | 77535 | DDB_G0289143 | DDB_G0289143 |  |
| inparanoid | 77539 | DDB_G0289139 | drpp20 | RNase P protein subunit, RNase MRP protein subunit |
| inparanoid | 77552 | DDB_G0289689 | DDB_G0289689 | Similar to Strongylocentrotus purpuratus (Purple sea urchin). Fibropellin I (Epidermal growth factor-related protein 1) (UEGF-1). |
| inparanoid | 77555 | DDB_G0289413 | DDB_G0289413 |  |
| inparanoid | 77570 | DDB_G0285185 | DDB_G0285185 |  |
| inparanoid | 77576 | DDB_G0285033 | DDB_G0285033 | Q9RPT1 Rhamnolipids biosynthesis 3-oxoacyl-[acyl-carrier protein] reductase (EC 1.1.1.100) (3-ketoacyl-acyl carrier protein reductase). |
| inparanoid | 77605 | DDB_G0283073 | orcA | origin recognition complex subunit 1 |
| inparanoid | 77606 | DDB_G0283071 | DDB_G0283071 |  |
| inparanoid | 77611 | DDB_G0291750 | mnd1 | meiotic nuclear division protein 1 |
| inparanoid | 77614 | DDB_G0291822 | wdr92 | WD40 repeat-containing protein |
| inparanoid | 77620 | DDB_G0291794 | DDB_G0291794 |  |
| inparanoid | 77625 | DDB_G0283311 | DDB_G0283311 | unknown |
| inparanoid | 77635 | DDB_G0279731 | DDB_G0279731 |  |
| inparanoid | 77644 | DDB_G0281501 | DDB_G0281501 |  |
| inparanoid | 77665 | DDB_G0268094 | DDB_G0268094 |  |
| inparanoid | 77679 | DDB_G0290453 | usp12 | peptidase C19 family protein, putative ubiquitin carboxyl-terminal hydrolase (UCH) |
| inparanoid | 77680 | DDB_G0290455 | DDB_G0290455 |  |
| inparanoid | 77698 | DDB_G0274585 | DDB_G0274585 | putative acyl-CoA oxidase |
| inparanoid | 77699 | DDB_G0274253 | DDB_G0274253 |  |
| inparanoid | 77713 | DDB_G0278855 | DDB_G0278855 |  |
| inparanoid | 77715 | DDB_G0279311 | cdc5l | myb domain-containing protein |
| inparanoid | 77720 | DDB_G0278935 | DDB_G0278935 | SAP DNA-binding domain-containing protein, C3HC4-type zinc finger-containing protein |
| inparanoid | 77723 | DDB_G0278791 | DDB_G0278791 |  |
| inparanoid | 77728 | DDB_G0278841 | DDB_G0278841 | cytidine deaminase-like protein |
| inparanoid | 77729 | DDB_G0278789 | DDB_G0278789 | heat shock protein DnaJ family protein |
| inparanoid | 77743 | DDB_G0282337 | DDB_G0282337 | Phosphatidylserine decarboxylase proenzyme 2 precursor. |
| inparanoid | 77786 | DDB_G0289479 | swp1 | dolichyl-diphosphooligosaccharide-protein glycotransferase, oligosaccharyltransferase delta subunit |
| inparanoid | 77821 | DDB_G0269550 | DDB_G0269550 |  |
| inparanoid | 77822 | DDB_G0272226 | DDB_G0272226 |  |
| inparanoid | 77880 | DDB_G0286105 | DDB_G0286105 |  |
| inparanoid | 77892 | DDB_G0287309 | DDB_G0287309 |  |
| inparanoid | 77902 | DDB_G0280423 | DDB_G0280423 | UAS domain-containing protein, UBX domain-containing protein |
| inparanoid | 77914 | DDB_G0269278 | DDB_G0269278 |  |
| inparanoid | 77919 | DDB_G0269356 | DDB_G0269356 | Short-chain dehydrogenase/reductase (SDR) superfamily (EC 1.1.1.100). |
| inparanoid | 77947 | DDB_G0286191 | atg8 | autophagy protein 8 |
| inparanoid | 77982 | DDB_G0291526 | rps14 | 40S ribosomal protein S14 |
| inparanoid | 77995 | DDB_G0287741 | DDB_G0287741 | enoyl-CoA hydratase/isomerase domain-containing protein |
| inparanoid | 77998 | DDB_G0288495 | DDB_G0288495 |  |
| inparanoid | 78014 | DDB_G0281269 | DDB_G0281269 |  |
| inparanoid | 78015 | DDB_G0281271 | DDB_G0281271 |  |
| inparanoid | 78016 | DDB_G0281277 | med12 | putative mediator complex subunit 12 |
| inparanoid | 78047 | DDB_G0289535 | cog5 | oligomeric Golgi complex component |
| inparanoid | 78051 | DDB_G0287967 | DDB_G0287967 | unknown |
| inparanoid | 78060 | DDB_G0287617 | DDB_G0287617 |  |
| inparanoid | 78063 | DDB_G0287949 | DDB_G0287949 | unknown |
| inparanoid | 78070 | DDB_G0288087 | DDB_G0288087 |  |
| inparanoid | 78083 | DDB_G0287553 | rab7B | Rab GTPase |
| inparanoid | 78124 | DDB_G0278333 | DDB_G0278333 | SppA. |
| inparanoid | 78128 | DDB_G0287663 | DDB_G0287663 |  |
| inparanoid | 78139 | DDB_G0287499 | DDB_G0287499 |  |
| inparanoid | 78145 | DDB_G0279669 | DDB_G0279669 |  |
| inparanoid | 78209 | DDB_G0274833 | DDB_G0274833 |  |
| inparanoid | 78210 | DDB_G0274295 | DDB_G0274295 | RWP-RK domain-containing protein |
| inparanoid | 78221 | DDB_G0284881 | DDB_G0284881 |  |
| inparanoid | 78235 | DDB_G0275049 | DDB_G0275049 | STEROID DEHYDROGENASE HOMOLOG, short-chain dehydrogenase/reductase (SDR) family protein |
| inparanoid | 78261 | DDB_G0290197 | DDB_G0290197 | calcium-binding EF-hand domain-containing protein, putative NADH dehydrogenase |
| inparanoid | 78276 | DDB_G0275391 | tacc1 | transforming acidic coiled-coil-containing protein |
| inparanoid | 78286 | DDB_G0287763 | DDB_G0287763 |  |
| inparanoid | 78293 | DDB_G0287243 | DDB_G0287243 |  |
| inparanoid | 78299 | DDB_G0288567 | DDB_G0288567 |  |
| inparanoid | 78308 | DDB_G0287377 | mccA | methylcrotonyl-CoA carboxylase, 3-methylcrotonyl-CoA:carbon dioxide ligase alpha subunit |
| inparanoid | 78309 | DDB_G0287255 | gcvP | glycine dehydrogenase (decarboxylating), glycine cleavage system P-protein |
| inparanoid | 78310 | DDB_G0287269 | ccbl | kynurenine-oxoglutarate transaminase, kynurenine aminotransferase, cysteine-S-conjugate beta-lyase |
| inparanoid | 78317 | DDB_G0288081 | DDB_G0288081 |  |
| inparanoid | 78321 | DDB_G0288111 | DDB_G0288111 |  |
| inparanoid | 78323 | DDB_G0288121 | DDB_G0288121 |  |
| inparanoid | 78326 | DDB_G0272048 | DDB_G0272048 |  |
| inparanoid | 78336 | DDB_G0286323 | DDB_G0286323 | Similar to Dictyostelium discoideum (Slime mold). hypothetical 127.0 kDa protein. |
| inparanoid | 78354 | DDB_G0279143 | DDB_G0279143 |  |
| inparanoid | 78374 | DDB_G0271574 | DDB_G0271574 |  |
| inparanoid | 78391 | DDB_G0287071 | DDB_G0287071 |  |
| inparanoid | 78396 | DDB_G0277089 | commd4 | COMM domain-containing protein 4 |
| inparanoid | 78411 | DDB_G0280135 | DDB_G0280135 | methionine adenosyltransferase regulatory beta subunit |
| inparanoid | 78414 | DDB_G0280131 | DDB_G0280131 | armadillo repeat-containing protein, protein kinase, TKL group, tyrosine kinase-like protein, ARMK family protein kinase |
| inparanoid | 78423 | DDB_G0275339 | DDB_G0275339 |  |
| inparanoid | 78440 | DDB_G0268402 | rab24 | Rab GTPase |
| inparanoid | 78457 | DDB_G0278957 | DDB_G0278957 |  |
| inparanoid | 78515 | DDB_G0291350 | DDB_G0291350 | putative protein serine/threonine kinase, putative MPSK, NAK family protein kinase |
| inparanoid | 78519 | DDB_G0291358 | cct2 | chaperonin containing TCP1 beta subunit |
| inparanoid | 78544 | DDB_G0276535 | DDB_G0276535 |  |
| inparanoid | 78545 | DDB_G0276537 | DDB_G0276537 |  |
| inparanoid | 78552 | DDB_G0275509 | DDB_G0275509 |  |
| inparanoid | 78558 | DDB_G0294619 | DDB_G0294619 | transmembrane protein |
| inparanoid | 78588 | DDB_G0293624 | DDB_G0293624 |  |
| inparanoid | 78591 | DDB_G0274931 | DDB_G0274931 | Similar to Dictyostelium discoideum (Slime mold). hypothetical 127.0 kDa protein. |
| inparanoid | 78592 | DDB_G0269812 | DDB_G0269812 |  |
| inparanoid | 78609 | DDB_G0287299 | DDB_G0287299 |  |
| inparanoid | 78642 | DDB_G0281693 | DDB_G0281693 |  |
| inparanoid | 78651 | DDB_G0292432 | DDB_G0292432 |  |
| inparanoid | 78660 | DDB_G0292392 | Dd5P3 | inositol 5-phosphatase |
| inparanoid | 78667 | DDB_G0287751 | DDB_G0287751 | unknown |
| inparanoid | 78678 | DDB_G0292924 | adcA | FYVE-type zinc finger-containing protein, arrestin domain-containing protein |
| inparanoid | 78683 | DDB_G0292912 | psiN | PA14 domain-containing protein |
| inparanoid | 78724 | DDB_G0278883 | DDB_G0278883 |  |
| inparanoid | 78725 | DDB_G0278953 | DDB_G0278953 | CBS (cystathionine-beta-synthase) domain-containing protein |
| inparanoid | 78739 | DDB_G0285577 | DDB_G0285577 | HSPC219. |
| inparanoid | 78743 | DDB_G0285969 | DDB_G0285969 | DJ-1/ThiJ/PfpI family protein, peptidase C56 family protein |
| inparanoid | 78792 | DDB_G0274461 | DDB_G0274461 |  |
| inparanoid | 78801 | DDB_G0274793 | DDB_G0274793 |  |
| inparanoid | 78821 | DDB_G0271890 | DDB_G0271890 | BH1308 PROTEIN. 6/101 |
| inparanoid | 78858 | DDB_G0283049 | DDB_G0283049 |  |
| inparanoid | 78861 | DDB_G0282935 | DDB_G0282935 |  |
| inparanoid | 78883 | DDB_G0283261 | acoA | acyl-CoA oxidase |
| inparanoid | 78889 | DDB_G0281663 | aprA | PhoPQ-activated pathogenicity-related protein |
| inparanoid | 78895 | DDB_G0278801 | DDB_G0278801 |  |
| inparanoid | 78901 | DDB_G0279329 | DDB_G0279329 |  |
| inparanoid | 78906 | DDB_G0281313 | DDB_G0281313 |  |
| inparanoid | 78920 | DDB_G0275983 | DDB_G0275983 |  |
| inparanoid | 78921 | DDB_G0275981 | DDB_G0275981 | unknown |
| inparanoid | 78936 | DDB_G0291480 | DDB_G0291480 |  |
| inparanoid | 78961 | DDB_G0284919 | DDB_G0284919 |  |
| inparanoid | 78969 | DDB_G0276519 | mus81 | crossover junction endonuclease |
| inparanoid | 78981 | DDB_G0292506 | DDB_G0292506 |  |
| inparanoid | 78987 | DDB_G0287635 | prpf4 | WD40 repeat-containing protein, U4/U6 small nuclear ribonucleoprotein, pre-mRNA processing factor 4 |
| inparanoid | 78988 | DDB_G0287415 | DDB_G0287415 |  |
| inparanoid | 78990 | DDB_G0288057 | DDB_G0288057 |  |
| inparanoid | 79010 | DDB_G0287017 | DDB_G0287017 |  |
| inparanoid | 79023 | DDB_G0286225 | DDB_G0286225 |  |
| inparanoid | 79031 | DDB_G0280985 | udpB | uridine phosphorylase |
| inparanoid | 79089 | DDB_G0275237 | DDB_G0275237 |  |
| inparanoid | 79107 | DDB_G0292424 | DDB_G0292424 |  |
| inparanoid | 79109 | DDB_G0269324 | gins4 | GINS complex subunit 4 |
| inparanoid | 79130 | DDB_G0268552 | DDB_G0268552 |  |
| inparanoid | 79136 | DDB_G0268288 | DDB_G0268288 |  |
| inparanoid | 79139 | DDB_G0267592 | DDB_G0267592 |  |
| inparanoid | 79155 | DDB_G0282407 | DDB_G0282407 |  |
| inparanoid | 79158 | DDB_G0287933 | DDB_G0287933 |  |
| inparanoid | 79161 | DDB_G0287813 | DDB_G0287813 |  |
| inparanoid | 79171 | DDB_G0287509 | DDB_G0287509 | UbiA prenyltransferase family protein, putative 1,4-dihydroxy-2-naphthoate octaprenyltransferase |
| inparanoid | 79181 | DDB_G0284075 | elp1 | elongation protein 1, IKI3 domain-containing protein, RNA polymerase II elongator complex subunit |
| inparanoid | 79230 | DDB_G0287387 | DDB_G0287387 |  |
| inparanoid | 79236 | DDB_G0287623 | DDB_G0287623 |  |
| inparanoid | 79246 | DDB_G0269288 | DDB_G0269288 |  |
| inparanoid | 79257 | DDB_G0270698 | DDB_G0270698 |  |
| inparanoid | 79261 | DDB_G0269934 | DDB_G0269934 | regulator of chromosome condensation (RCC1) domain-containing protein, RhoGEF domain-containing protein, Ras GTPase activation domain-containing protein |
| inparanoid | 79270 | DDB_G0267394 | vps46 | SNF7 family protein |
| inparanoid | 79295 | DDB_G0268048 | DDB_G0268048 |  |
| inparanoid | 79326 | DDB_G0271750 | nfaA | Ras GTPase activation domain-containing protein |
| inparanoid | 79367 | DDB_G0279519 | mrpl22 | ribosomal protein L22, mitochondrial |
| inparanoid | 79372 | DDB_G0281489 | DDB_G0281489 | TU37B2. |
| inparanoid | 79373 | DDB_G0281487 | DDB_G0281487 | 3beta-hydroxysteroid dehydrogenase, putative sterol-4alpha-carboxylate 3-dehydrogenase (decarboxylating) |
| inparanoid | 79391 | DDB_G0269692 | DDB_G0269692 |  |
| inparanoid | 79395 | DDB_G0270238 | DDB_G0270238 |  |
| inparanoid | 79397 | DDB_G0268856 | DDB_G0268856 |  |
[truncated: 255,922 more chars]
