# Supplementary material for: Comparative genomics of the social amoebae Dictyostelium discoideum and Dictyostelium purpureum
Source: Genome Biol. 2011 Feb 28;12(2):R20. doi: 10.1186/gb-2011-12-2-r20 (PMC3188802; doi:10.1186/gb-2011-12-2-r20)
Supplement: Additional file 4 — Supplementary Tables S5. A table listing the predicted paralogs that are shared between D. discoideum and D. purpureum. [file gb-2011-12-2-r20-S4.DOC]

| **D. discoideum genes with close paralogs in D. purpureum** | | | | | | | | | | |  |
| --- | --- | --- | --- | --- | --- | --- | --- | --- | --- | --- | --- |
| **ddb_g** | | | | **genename** | | | | **annotation** | | | |
| DDB_G0276401 | | | | DDB_G0276401 | | | |  | | | |
| DDB_G0276403 | | | | DDB_G0276403 | | | |  | | | |
| DDB_G0276409 | | | | DDB_G0276409 | | | |  | | | |
| DDB_G0276429 | | | | DDB_G0276429 | | | |  | | | |
| DDB_G0276593 | | | | DDB_G0276593 | | | |  | | | |
| DDB_G0276601 | | | | DDB_G0276601 | | | |  | | | |
| DDB_G0276603 | | | | DDB_G0276603 | | | |  | | | |
| DDB_G0275475 | | | | DDB_G0275475 | | | |  | | | |
| DDB_G0275465 | | | | DDB_G0275465 | | | |  | | | |
| DDB_G0275481 | | | | DDB_G0275481 | | | |  | | | |
| DDB_G0275507 | | | | DDB_G0275507 | | | | PUTATIVE ACETYL TRANSFERASE. 6/101 | | | |
| DDB_G0275511 | | | | DDB_G0275511 | | | |  | | | |
| DDB_G0275527 | | | | DDB_G0275527 | | | |  | | | |
| DDB_G0275677 | | | | DDB_G0275677 | | | |  | | | |
| DDB_G0275563 | | | | DDB_G0275563 | | | | Similar to Mus musculus (Mouse). GABA-A receptor epsilon-like subunit. | | | |
| DDB_G0275585 | | | | DDB_G0275585 | | | | PUTATIVE OXIDOREDUCTASE, OXYGEN DEPENDENT, FAD-DEPENDENT PROTEIN | | | |
| DDB_G0275605 | | | | DDB_G0275605 | | | |  | | | |
| DDB_G0275607 | | | | DDB_G0275607 | | | |  | | | |
| DDB_G0275615 | | | | DDB_G0275615 | | | |  | | | |
| DDB_G0275557 | | | | DDB_G0275557 | | | | Similar to Neurospora crassa. hypothetical 79.1 kDa protein. | | | |
| DDB_G0275553 | | | | DDB_G0275553 | | | |  | | | |
| DDB_G0275027 | | | | DDB_G0275027 | | | |  | | | |
| DDB_G0275053 | | | | DDB_G0275053 | | | | Putative epimerase | | | |
| DDB_G0275151 | | | | DDB_G0275151 | | | |  | | | |
| DDB_G0275103 | | | | DDB_G0275103 | | | |  | | | |
| DDB_G0275143 | | | | DDB_G0275143 | | | | PUTATIVE CALMODULIN-BINDING PROTEIN CAM-BP46 | | | |
| DDB_G0275139 | | | | DDB_G0275139 | | | |  | | | |
| DDB_G0275133 | | | | DDB_G0275133 | | | |  | | | |
| DDB_G0274143 | | | | DDB_G0274143 | | | |  | | | |
| DDB_G0274161 | | | | DDB_G0274161 | | | |  | | | |
| DDB_G0274175 | | | | DDB_G0274175 | | | |  | | | |
| DDB_G0274187 | | | | DDB_G0274187 | | | |  | | | |
| DDB_G0274195 | | | | DDB_G0274195 | | | |  | | | |
| DDB_G0274209 | | | | DDB_G0274209 | | | | TRANSMEMBRANE PROTEIN. 6/101 | | | |
| DDB_G0274219 | | | | DDB_G0274219 | | | |  | | | |
| DDB_G0274225 | | | | DDB_G0274225 | | | |  | | | |
| DDB_G0274397 | | | | DDB_G0274397 | | | |  | | | |
| DDB_G0274411 | | | | DDB_G0274411 | | | | Similar to Dictyostelium discoideum (Slime mold). hypothetical 97.7 kDa protein. | | | |
| DDB_G0274419 | | | | DDB_G0274419 | | | |  | | | |
| DDB_G0274421 | | | | DDB_G0274421 | | | | Similar to Thermotoga maritima. DNA-directed DNA polymerase I. | | | |
| DDB_G0274427 | | | | DDB_G0274427 | | | | Similar to Dictyostelium discoideum (Slime mold). hypothetical 127.0 kDa protein. | | | |
| DDB_G0274429 | | | | DDB_G0274429 | | | | Similar to Dictyostelium discoideum (Slime mold). hypothetical 127.0 kDa protein. | | | |
| DDB_G0274431 | | | | DDB_G0274431 | | | | Similar to Dictyostelium discoideum (Slime mold). hypothetical 127.0 kDa protein. | | | |
| DDB_G0274453 | | | | DDB_G0274453 | | | |  | | | |
| DDB_G0274509 | | | | DDB_G0274509 | | | |  | | | |
| DDB_G0274515 | | | | DDB_G0274515 | | | |  | | | |
| DDB_G0274529 | | | | DDB_G0274529 | | | | Similar to Dictyostelium discoideum (Slime mold). cell surface glycoprotein GP138B. | | | |
| DDB_G0274541 | | | | DDB_G0274541 | | | |  | | | |
| DDB_G0274245 | | | | DDB_G0274245 | | | |  | | | |
| DDB_G0274319 | | | | DDB_G0274319 | | | |  | | | |
| DDB_G0274341 | | | | DDB_G0274341 | | | |  | | | |
| DDB_G0274369 | | | | DDB_G0274369 | | | |  | | | |
| DDB_G0272626 | | | | DDB_G0272626 | | | | AT2G26590/T9J22.26. 6/101 | | | |
| DDB_G0272793 | | | | DDB_G0272793 | | | |  | | | |
| DDB_G0272630 | | | | DDB_G0272630 | | | | Putative Zn-dependent alcohol dehydrogenases | | | |
| DDB_G0272636 | | | | DDB_G0272636 | | | |  | | | |
| DDB_G0272644 | | | | DDB_G0272644 | | | |  | | | |
| DDB_G0272648 | | | | DDB_G0272648 | | | |  | | | |
| DDB_G0272606 | | | | DDB_G0272606 | | | |  | | | |
| DDB_G0272612 | | | | DDB_G0272612 | | | |  | | | |
| DDB_G0272620 | | | | DDB_G0272620 | | | |  | | | |
| DDB_G0272580 | | | | DDB_G0272580 | | | |  | | | |
| DDB_G0272801 | | | | DDB_G0272801 | | | |  | | | |
| DDB_G0272807 | | | | DDB_G0272807 | | | | Similar to hypothetical ORF; Ypl110cp. | | | |
| DDB_G0272602 | | | | DDB_G0272602 | | | | Similar to Dictyostelium discoideum (Slime mold). CIGB protein. | | | |
| DDB_G0272570 | | | | DDB_G0272570 | | | |  | | | |
| DDB_G0272572 | | | | DDB_G0272572 | | | |  | | | |
| DDB_G0272576 | | | | DDB_G0272576 | | | |  | | | |
| DDB_G0273157 | | | | DDB_G0273157 | | | |  | | | |
| DDB_G0273169 | | | | DDB_G0273169 | | | |  | | | |
| DDB_G0273173 | | | | DDB_G0273173 | | | |  | | | |
| DDB_G0273151 | | | | DDB_G0273151 | | | |  | | | |
| DDB_G0273177 | | | | DDB_G0273177 | | | |  | | | |
| DDB_G0273125 | | | | DDB_G0273125 | | | |  | | | |
| DDB_G0273123 | | | | DDB_G0273123 | | | | Prestalk protein precursor. | | | |
| DDB_G0273119 | | | | DDB_G0273119 | | | |  | | | |
| DDB_G0273113 | | | | DDB_G0273113 | | | |  | | | |
| DDB_G0273109 | | | | DDB_G0273109 | | | |  | | | |
| DDB_G0273103 | | | | DDB_G0273103 | | | | FUSOLIN PRECURSOR | | | |
| DDB_G0273179 | | | | DDB_G0273179 | | | |  | | | |
| DDB_G0273095 | | | | DDB_G0273095 | | | |  | | | |
| DDB_G0273087 | | | | DDB_G0273087 | | | |  | | | |
| DDB_G0273085 | | | | DDB_G0273085 | | | |  | | | |
| DDB_G0273077 | | | | DDB_G0273077 | | | |  | | | |
| DDB_G0273185 | | | | DDB_G0273185 | | | | Similar to Dictyostelium discoideum (Slime mold). histidine kinase DhkE. | | | |
| DDB_G0273217 | | | | DDB_G0273217 | | | |  | | | |
| DDB_G0273219 | | | | DDB_G0273219 | | | |  | | | |
| DDB_G0273231 | | | | DDB_G0273231 | | | | Similar to plasmodium falciparum. SET-domain protein, putative. | | | |
| DDB_G0273233 | | | | DDB_G0273233 | | | |  | | | |
| DDB_G0273245 | | | | DDB_G0273245 | | | |  | | | |
| DDB_G0273247 | | | | DDB_G0273247 | | | |  | | | |
| DDB_G0273135 | | | | DDB_G0273135 | | | |  | | | |
| DDB_G0273137 | | | | DDB_G0273137 | | | | HYPOTHETICAL 83.2 KDA PROTEIN | | | |
| DDB_G0273143 | | | | DDB_G0273143 | | | |  | | | |
| DDB_G0273145 | | | | DDB_G0273145 | | | |  | | | |
| DDB_G0273411 | | | | DDB_G0273411 | | | |  | | | |
| DDB_G0273413 | | | | DDB_G0273413 | | | | Similar to Xanthomonas axonopodis (Pv. citri). Pirin. | | | |
| DDB_G0273415 | | | | DDB_G0273415 | | | |  | | | |
| DDB_G0273417 | | | | DDB_G0273417 | | | |  | | | |
| DDB_G0273419 | | | | DDB_G0273419 | | | | Similar to Haemonchus contortus (Barber pole worm). membrane aminopeptidase H11-4, isoform 4. | | | |
| DDB_G0273421 | | | | DDB_G0273421 | | | |  | | | |
| DDB_G0273423 | | | | DDB_G0273423 | | | | Similar to Homo sapiens (Human). mucin 2 (Intestinal mucin 2). | | | |
| DDB_G0273431 | | | | DDB_G0273431 | | | |  | | | |
| DDB_G0273433 | | | | DDB_G0273433 | | | |  | | | |
| DDB_G0273435 | | | | DDB_G0273435 | | | |  | | | |
| DDB_G0273437 | | | | DDB_G0273437 | | | | Similar to Escherichia coli CFT073. glycerol dehydrogenase (EC 1.1.1.6). | | | |
| DDB_G0273441 | | | | DDB_G0273441 | | | |  | | | |
| DDB_G0272742 | | | | DDB_G0272742 | | | | CG5367 PROTEIN. 6/101 | | | |
| DDB_G0272734 | | | | DDB_G0272734 | | | |  | | | |
| DDB_G0276827 | | | | DDB_G0276827 | | | |  | | | |
| DDB_G0276817 | | | | DDB_G0276817 | | | | K08H10.2A PROTEIN. 6/100 | | | |
| DDB_G0276807 | | | | DDB_G0276807 | | | |  | | | |
| DDB_G0276773 | | | | DDB_G0276773 | | | |  | | | |
| DDB_G0276793 | | | | DDB_G0276793 | | | |  | | | |
| DDB_G0276911 | | | | DDB_G0276911 | | | |  | | | |
| DDB_G0276929 | | | | DDB_G0276929 | | | |  | | | |
| DDB_G0276935 | | | | DDB_G0276935 | | | |  | | | |
| DDB_G0271508 | | | | DDB_G0271508 | | | | Similar to Dictyostelium discoideum (Slime mold). CIGB protein. | | | |
| DDB_G0271518 | | | | DDB_G0271518 | | | |  | | | |
| DDB_G0271556 | | | | DDB_G0271556 | | | |  | | | |
| DDB_G0271674 | | | | DDB_G0271674 | | | |  | | | |
| DDB_G0271670 | | | | DDB_G0271670 | | | | Similar to Leishmania major. Ppg3. | | | |
| DDB_G0271986 | | | | DDB_G0271986 | | | | Similar to Mus musculus (Mouse). similar to CCR4-NOT transcription complex, subunit 3. | | | |
| DDB_G0272016 | | | | DDB_G0272016 | | | |  | | | |
| DDB_G0272024 | | | | DDB_G0272024 | | | |  | | | |
| DDB_G0272028 | | | | DDB_G0272028 | | | | Similar to Dictyostelium discoideum (Slime mold). vegetative stage specific V4-7. | | | |
| DDB_G0272030 | | | | DDB_G0272030 | | | |  | | | |
| DDB_G0272072 | | | | DDB_G0272072 | | | | Similar to Dictyostelium discoideum (Slime mold). CIGB protein. | | | |
| DDB_G0272122 | | | | DDB_G0272122 | | | |  | | | |
| DDB_G0272144 | | | | DDB_G0272144 | | | |  | | | |
| DDB_G0272174 | | | | DDB_G0272174 | | | |  | | | |
| DDB_G0272162 | | | | DDB_G0272162 | | | |  | | | |
| DDB_G0272492 | | | | DDB_G0272492 | | | | Similar to Dictyostelium discoideum (Slime mold). Hypothetical 127.0 kDa protein. | | | |
| DDB_G0272490 | | | | DDB_G0272490 | | | |  | | | |
| DDB_G0272494 | | | | DDB_G0272494 | | | |  | | | |
| DDB_G0272496 | | | | DDB_G0272496 | | | |  | | | |
| DDB_G0272508 | | | | DDB_G0272508 | | | |  | | | |
| DDB_G0272510 | | | | DDB_G0272510 | | | |  | | | |
| DDB_G0272726 | | | | DDB_G0272726 | | | |  | | | |
| DDB_G0272712 | | | | DDB_G0272712 | | | |  | | | |
| DDB_G0272823 | | | | DDB_G0272823 | | | | Similar to Dictyostelium discoideum (Slime mold). hypothetical 97.7 kDa protein. | | | |
| DDB_G0272829 | | | | DDB_G0272829 | | | |  | | | |
| DDB_G0272750 | | | | DDB_G0272750 | | | |  | | | |
| DDB_G0276831 | | | | DDB_G0276831 | | | |  | | | |
| DDB_G0276969 | | | | DDB_G0276969 | | | | P18142 cAMP-regulated D2 protein precursor. | | | |
| DDB_G0276851 | | | | DDB_G0276851 | | | |  | | | |
| DDB_G0276859 | | | | DDB_G0276859 | | | |  | | | |
| DDB_G0276879 | | | | DDB_G0276879 | | | |  | | | |
| DDB_G0276955 | | | | DDB_G0276955 | | | |  | | | |
| DDB_G0276957 | | | | DDB_G0276957 | | | |  | | | |
| DDB_G0276959 | | | | DDB_G0276959 | | | | Similar to Podocoryne carnea. EGF-like protein (Fragment). | | | |
| DDB_G0277171 | | | | DDB_G0277171 | | | |  | | | |
| DDB_G0277177 | | | | DDB_G0277177 | | | |  | | | |
| DDB_G0277181 | | | | DDB_G0277181 | | | |  | | | |
| DDB_G0277201 | | | | DDB_G0277201 | | | | ENHANCING FACTOR PRECURSOR | | | |
| DDB_G0277225 | | | | DDB_G0277225 | | | |  | | | |
| DDB_G0277153 | | | | DDB_G0277153 | | | |  | | | |
| DDB_G0277407 | | | | DDB_G0277407 | | | |  | | | |
| DDB_G0277409 | | | | DDB_G0277409 | | | |  | | | |
| DDB_G0277385 | | | | DDB_G0277385 | | | |  | | | |
| DDB_G0277387 | | | | DDB_G0277387 | | | | Similar to Dictyostelium discoideum (Slime mold). hypothetical 97.7 kDa protein. | | | |
| DDB_G0277487 | | | | DDB_G0277487 | | | |  | | | |
| DDB_G0277431 | | | | DDB_G0277431 | | | |  | | | |
| DDB_G0277443 | | | | DDB_G0277443 | | | |  | | | |
| DDB_G0277447 | | | | DDB_G0277447 | | | |  | | | |
| DDB_G0277457 | | | | DDB_G0277457 | | | |  | | | |
| DDB_G0277573 | | | | DDB_G0277573 | | | |  | | | |
| DDB_G0277585 | | | | DDB_G0277585 | | | | Similar to G-protein-coupled receptor at plasma membrane; interactions in two-hybrid system with Gpa2p; Gpr1p. | | | |
| DDB_G0277599 | | | | DDB_G0277599 | | | |  | | | |
| DDB_G0277603 | | | | DDB_G0277603 | | | | Similar to Dictyostelium discoideum (Slime mold). Hypothetical 127.0 kDa protein. | | | |
| DDB_G0277679 | | | | DDB_G0277679 | | | | Similar to Arabidopsis thaliana (Mouse-ear cress). genomic DNA, chromosome 5, P1 clone:MNB8 (EC 3.3.2.3) (Epoxide hydrolase). | | | |
| DDB_G0276275 | | | | DDB_G0276275 | | | |  | | | |
| DDB_G0276147 | | | | DDB_G0276147 | | | | Putative nuclease. | | | |
| DDB_G0276091 | | | | DDB_G0276091 | | | | putative protein 150-kD protein cluA - Dictyostelium discoideum, PID:g2281117 | | | |
| DDB_G0291261 | | | | DDB_G0291261 | | | |  | | | |
| DDB_G0291289 | | | | DDB_G0291289 | | | |  | | | |
| DDB_G0291316 | | | | DDB_G0291316 | | | |  | | | |
| DDB_G0291406 | | | | DDB_G0291406 | | | | Q9Z765 Preprotein translocase secA subunit. | | | |
| DDB_G0291426 | | | | DDB_G0291426 | | | |  | | | |
| DDB_G0291432 | | | | DDB_G0291432 | | | |  | | | |
| DDB_G0291454 | | | | DDB_G0291454 | | | | P42523 Loose aggregate C protein precursor. | | | |
| DDB_G0291456 | | | | DDB_G0291456 | | | |  | | | |
| DDB_G0291458 | | | | DDB_G0291458 | | | |  | | | |
| DDB_G0291462 | | | | DDB_G0291462 | | | |  | | | |
| DDB_G0291474 | | | | DDB_G0291474 | | | |  | | | |
| DDB_G0291492 | | | | DDB_G0291492 | | | |  | | | |
| DDB_G0291538 | | | | DDB_G0291538 | | | | Maltose O-acetyltransferase (EC 2.3.1.79). | | | |
| DDB_G0291540 | | | | DDB_G0291540 | | | |  | | | |
| DDB_G0291582 | | | | DDB_G0291582 | | | |  | | | |
| DDB_G0291586 | | | | DDB_G0291586 | | | |  | | | |
| DDB_G0291592 | | | | DDB_G0291592 | | | |  | | | |
| DDB_G0291706 | | | | DDB_G0291706 | | | |  | | | |
| DDB_G0291718 | | | | DDB_G0291718 | | | |  | | | |
| DDB_G0291722 | | | | DDB_G0291722 | | | |  | | | |
| DDB_G0291732 | | | | DDB_G0291732 | | | |  | | | |
| DDB_G0291744 | | | | DDB_G0291744 | | | |  | | | |
| DDB_G0291746 | | | | DDB_G0291746 | | | |  | | | |
| DDB_G0291748 | | | | DDB_G0291748 | | | |  | | | |
| DDB_G0291758 | | | | DDB_G0291758 | | | |  | | | |
| DDB_G0291760 | | | | DDB_G0291760 | | | |  | | | |
| DDB_G0291790 | | | | DDB_G0291790 | | | |  | | | |
| DDB_G0291792 | | | | DDB_G0291792 | | | |  | | | |
| DDB_G0291874 | | | | DDB_G0291874 | | | |  | | | |
| DDB_G0291878 | | | | DDB_G0291878 | | | | Pol. | | | |
| DDB_G0291880 | | | | DDB_G0291880 | | | | Pol. | | | |
| DDB_G0291882 | | | | DDB_G0291882 | | | | Pol. | | | |
| DDB_G0291884 | | | | DDB_G0291884 | | | | Multifunctional protein (Fragment). | | | |
| DDB_G0291888 | | | | DDB_G0291888 | | | |  | | | |
| DDB_G0291920 | | | | DDB_G0291920 | | | |  | | | |
| DDB_G0292008 | | | | DDB_G0292008 | | | |  | | | |
| DDB_G0292020 | | | | DDB_G0292020 | | | |  | | | |
| DDB_G0292030 | | | | DDB_G0292030 | | | |  | | | |
| DDB_G0292056 | | | | DDB_G0292056 | | | |  | | | |
| DDB_G0292060 | | | | DDB_G0292060 | | | | Pol. | | | |
| DDB_G0292062 | | | | DDB_G0292062 | | | | Pol. | | | |
| DDB_G0292080 | | | | DDB_G0292080 | | | |  | | | |
| DDB_G0292084 | | | | DDB_G0292084 | | | |  | | | |
| DDB_G0292086 | | | | DDB_G0292086 | | | |  | | | |
| DDB_G0292090 | | | | DDB_G0292090 | | | |  | | | |
| DDB_G0292114 | | | | DDB_G0292114 | | | |  | | | |
| DDB_G0292126 | | | | DDB_G0292126 | | | |  | | | |
| DDB_G0292238 | | | | DDB_G0292238 | | | |  | | | |
| DDB_G0292274 | | | | DDB_G0292274 | | | |  | | | |
| DDB_G0292280 | | | | DDB_G0292280 | | | |  | | | |
| DDB_G0292282 | | | | DDB_G0292282 | | | | Similar to Dictyostelium discoideum (Slime mold). putative calmodulin-binding protein CaM-BP46. | | | |
| DDB_G0292284 | | | | DDB_G0292284 | | | |  | | | |
| DDB_G0292336 | | | | DDB_G0292336 | | | |  | | | |
| DDB_G0292338 | | | | DDB_G0292338 | | | | Multifunctional protein (Fragment). | | | |
| DDB_G0292394 | | | | DDB_G0292394 | | | |  | | | |
| DDB_G0292452 | | | | DDB_G0292452 | | | | Group-specific antigen. | | | |
| DDB_G0292458 | | | | DDB_G0292458 | | | |  | | | |
| DDB_G0292474 | | | | DDB_G0292474 | | | |  | | | |
| DDB_G0292476 | | | | DDB_G0292476 | | | |  | | | |
| DDB_G0292572 | | | | DDB_G0292572 | | | |  | | | |
| DDB_G0292576 | | | | DDB_G0292576 | | | |  | | | |
| DDB_G0292578 | | | | DDB_G0292578 | | | |  | | | |
| DDB_G0292590 | | | | DDB_G0292590 | | | |  | | | |
| DDB_G0292606 | | | | DDB_G0292606 | | | |  | | | |
| DDB_G0292630 | | | | DDB_G0292630 | | | |  | | | |
| DDB_G0292634 | | | | DDB_G0292634 | | | |  | | | |
| DDB_G0292644 | | | | DDB_G0292644 | | | |  | | | |
| DDB_G0292656 | | | | DDB_G0292656 | | | |  | | | |
| DDB_G0292658 | | | | DDB_G0292658 | | | |  | | | |
| DDB_G0292668 | | | | DDB_G0292668 | | | |  | | | |
| DDB_G0292672 | | | | DDB_G0292672 | | | |  | | | |
| DDB_G0292674 | | | | DDB_G0292674 | | | |  | | | |
| DDB_G0292684 | | | | DDB_G0292684 | | | |  | | | |
| DDB_G0292700 | | | | DDB_G0292700 | | | |  | | | |
| DDB_G0292762 | | | | DDB_G0292762 | | | |  | | | |
| DDB_G0292772 | | | | DDB_G0292772 | | | |  | | | |
| DDB_G0272520 | | | | act15 | | | | actin | | | |
| DDB_G0272560 | | | | capA-1 | | | | cAMP-binding protein | | | |
| DDB_G0273397 | | | | carA-1 | | | | G-protein-coupled receptor, cAMP receptor 1 | | | |
| DDB_G0272827 | | | | cbpI | | | | EF-hand domain-containing protein | | | |
| DDB_G0273251 | | | | fpaB-1 | | | | cytosolic glycoprotein FP21, ubiquitin ligase subunit SKP1 | | | |
| DDB_G0273249 | | | | hspE-1 | | | | heat shock cognate protein Hsc70-2, heat shock protein | | | |
| DDB_G0273069 | | | | ndkC-1 | | | | NDP kinase, nucleoside diphosphate kinase | | | |
| DDB_G0272616 | | | | rnrB-1 | | | | ribonucleoside-diphosphate reductase, ribonucleotide reductase small subunit | | | |
| DDB_G0273175 | | | | cf50-1 | | | | component of the counting factor (CF) complex | | | |
| DDB_G0272564 | | | | psmD8-1 | | | | 26S proteasome non-ATPase regulatory subunit 8, 26S proteasome regulatory subunit S14 | | | |
| DDB_G0272799 | | | | icmA-1 | | | | prenylcysteine methyltransferase, protein-S-isoprenylcysteine O-methyltransferase | | | |
| DDB_G0271666 | | | | prtB | | | | proteosomal alpha-subunit 7-1 | | | |
| DDB_G0273097 | | | | ptpA1-1 | | | | protein-tyrosine phosphatase 1 | | | |
| DDB_G0272791 | | | | rsc11-1 | | | | alpha/beta hydrolase fold-1 domain-containing protein | | | |
| DDB_G0274133 | | | | act2 | | | | actin | | | |
| DDB_G0274131 | | | | act17 | | | | actin | | | |
| DDB_G0274135 | | | | act6 | | | | actin | | | |
| DDB_G0272248 | | | | act16 | | | | actin | | | |
| DDB_G0276077 | | | | mlcR | | | | Regulatory myosin light chain (RLC) | | | |
| DDB_G0273443 | | | | atg4 | | | | autophagy protein 4 | | | |
| DDB_G0272110 | | | | gskA | | | | GSK family protein kinase, glycogen synthase kinase 3, protein kinase, CMGC group, protein serine/threonine kinase | | | |
| DDB_G0273059 | | | | cak1 | | | | casein kinase I, protein kinase, CK1 group, protein serine/threonine kinase | | | |
| DDB_G0274099 | | | | cbpE | | | | calcium-binding protein | | | |
| DDB_G0276025 | | | | zakA | | | | CZAK family protein kinase, NZAK subfamily protein kinase, protein kinase, STE group, protein kinase, TKL group, protein tyrosine kinase, tyrosine kinase-like protein | | | |
| DDB_G0291241 | | | | gnt2 | | | | GlcNAc transferase, glycosyltransferase | | | |
| DDB_G0277141 | | | | cotC | | | | spore coat protein SP60 | | | |
| DDB_G0291227 | | | | ctrA | | | | putative cationic amino acid transporter, solute carrier family 7 member protein | | | |
| DDB_G0272568 | | | | cofC-1 | | | | cofilin-2 | | | |
| DDB_G0291253 | | | | dia2 | | | | unknown | | | |
| DDB_G0273445 | | | | spkA-1 | | | | ARK family protein kinase, SAM domain-containing protein, ankyrin repeat-containing protein, protein kinase, TKL group, stress-activated MEK-like kinase, tyrosine kinase-like protein | | | |
| DDB_G0271848 | | | | porA | | | | porin | | | |
| DDB_G0273259 | | | | qkgA | | | | ROCO family protein kinase, WD40 repeat-containing protein, leucine-rich repeat-containing protein (LRR), protein kinase, TKL group, tyrosine kinase-like protein | | | |
| DDB_G0276399 | | | | rab8B | | | | Rab GTPase | | | |
| DDB_G0272837 | | | | ifkA | | | | GCN2 subfamily protein kinase, PEK family protein kinase, anticodon-binding domain-containing protein, eukaryotic translation initiation factor 2 alpha (eIF2alpha) kinase, protein kinase, STE group | | | |
| DDB_G0273071 | | | | vatD | | | | vacuolar ATPase subunit DVA41 | | | |
| DDB_G0282985 | | | | DDB_G0282985 | | | |  | | | |
| DDB_G0282991 | | | | DDB_G0282991 | | | |  | | | |
| DDB_G0283025 | | | | DDB_G0283025 | | | |  | | | |
| DDB_G0283089 | | | | DDB_G0283089 | | | |  | | | |
| DDB_G0283093 | | | | DDB_G0283093 | | | |  | | | |
| DDB_G0283097 | | | | DDB_G0283097 | | | |  | | | |
| DDB_G0283105 | | | | DDB_G0283105 | | | |  | | | |
| DDB_G0283123 | | | | DDB_G0283123 | | | |  | | | |
| DDB_G0283169 | | | | DDB_G0283169 | | | | RIKEN cDNA 0610042E07. | | | |
| DDB_G0283185 | | | | DDB_G0283185 | | | |  | | | |
| DDB_G0283191 | | | | DDB_G0283191 | | | |  | | | |
| DDB_G0283199 | | | | DDB_G0283199 | | | |  | | | |
| DDB_G0283201 | | | | DDB_G0283201 | | | |  | | | |
| DDB_G0283289 | | | | DDB_G0283289 | | | | Putative calmodulin-binding protein CaM-BP46. | | | |
| DDB_G0283317 | | | | DDB_G0283317 | | | |  | | | |
| DDB_G0283321 | | | | DDB_G0283321 | | | |  | | | |
| DDB_G0283351 | | | | DDB_G0283351 | | | | Similar to Plasmodium falciparum (Isolate 3D7). Asparagine-rich antigen. | | | |
| DDB_G0283423 | | | | DDB_G0283423 | | | |  | | | |
| DDB_G0283425 | | | | DDB_G0283425 | | | |  | | | |
| DDB_G0283441 | | | | DDB_G0283441 | | | |  | | | |
| DDB_G0283471 | | | | DDB_G0283471 | | | |  | | | |
| DDB_G0283477 | | | | DDB_G0283477 | | | |  | | | |
| DDB_G0283549 | | | | DDB_G0283549 | | | |  | | | |
| DDB_G0283577 | | | | DDB_G0283577 | | | | Similar to Dictyostelium discoideum (Slime mold). hypothetical 127.0 kDa protein. | | | |
| DDB_G0283623 | | | | DDB_G0283623 | | | | Similar to Mus musculus (Mouse). tenascin C. | | | |
| DDB_G0283657 | | | | DDB_G0283657 | | | |  | | | |
| DDB_G0283739 | | | | DDB_G0283739 | | | |  | | | |
| DDB_G0283775 | | | | DDB_G0283775 | | | |  | | | |
| DDB_G0283785 | | | | DDB_G0283785 | | | |  | | | |
| DDB_G0283809 | | | | DDB_G0283809 | | | |  | | | |
| DDB_G0283817 | | | | DDB_G0283817 | | | | Probable PIRIN-like protein. | | | |
| DDB_G0283833 | | | | DDB_G0283833 | | | |  | | | |
| DDB_G0283851 | | | | DDB_G0283851 | | | |  | | | |
| DDB_G0283923 | | | | DDB_G0283923 | | | |  | | | |
| DDB_G0283945 | | | | DDB_G0283945 | | | |  | | | |
| DDB_G0283961 | | | | DDB_G0283961 | | | |  | | | |
| DDB_G0284055 | | | | DDB_G0284055 | | | |  | | | |
| DDB_G0284057 | | | | DDB_G0284057 | | | |  | | | |
| DDB_G0284061 | | | | DDB_G0284061 | | | |  | | | |
| DDB_G0284083 | | | | DDB_G0284083 | | | |  | | | |
| DDB_G0284095 | | | | DDB_G0284095 | | | |  | | | |
| DDB_G0284109 | | | | DDB_G0284109 | | | |  | | | |
| DDB_G0284111 | | | | DDB_G0284111 | | | |  | | | |
| DDB_G0284119 | | | | DDB_G0284119 | | | |  | | | |
| DDB_G0284123 | | | | DDB_G0284123 | | | |  | | | |
| DDB_G0284131 | | | | DDB_G0284131 | | | |  | | | |
| DDB_G0284215 | | | | DDB_G0284215 | | | |  | | | |
| DDB_G0284233 | | | | DDB_G0284233 | | | |  | | | |
| DDB_G0284271 | | | | DDB_G0284271 | | | |  | | | |
| DDB_G0284279 | | | | DDB_G0284279 | | | |  | | | |
| DDB_G0284373 | | | | DDB_G0284373 | | | |  | | | |
| DDB_G0284375 | | | | DDB_G0284375 | | | |  | | | |
| DDB_G0284387 | | | | DDB_G0284387 | | | |  | | | |
| DDB_G0284477 | | | | DDB_G0284477 | | | |  | | | |
| DDB_G0284479 | | | | DDB_G0284479 | | | |  | | | |
| DDB_G0284487 | | | | DDB_G0284487 | | | |  | | | |
| DDB_G0284527 | | | | DDB_G0284527 | | | |  | | | |
| DDB_G0284531 | | | | DDB_G0284531 | | | |  | | | |
| DDB_G0284629 | | | | DDB_G0284629 | | | |  | | | |
| DDB_G0284637 | | | | DDB_G0284637 | | | |  | | | |
| DDB_G0284639 | | | | DDB_G0284639 | | | |  | | | |
| DDB_G0284645 | | | | DDB_G0284645 | | | |  | | | |
| DDB_G0284653 | | | | DDB_G0284653 | | | |  | | | |
| DDB_G0284655 | | | | DDB_G0284655 | | | |  | | | |
| DDB_G0284659 | | | | DDB_G0284659 | | | |  | | | |
| DDB_G0284675 | | | | DDB_G0284675 | | | |  | | | |
| DDB_G0284753 | | | | DDB_G0284753 | | | |  | | | |
| DDB_G0284763 | | | | DDB_G0284763 | | | |  | | | |
| DDB_G0284789 | | | | DDB_G0284789 | | | |  | | | |
| DDB_G0284793 | | | | DDB_G0284793 | | | |  | | | |
| DDB_G0284797 | | | | DDB_G0284797 | | | |  | | | |
| DDB_G0284899 | | | | DDB_G0284899 | | | |  | | | |
| DDB_G0284935 | | | | DDB_G0284935 | | | |  | | | |
| DDB_G0284965 | | | | DDB_G0284965 | | | |  | | | |
| DDB_G0284967 | | | | DDB_G0284967 | | | |  | | | |
| DDB_G0284991 | | | | DDB_G0284991 | | | |  | | | |
| DDB_G0285001 | | | | DDB_G0285001 | | | |  | | | |
| DDB_G0285015 | | | | DDB_G0285015 | | | |  | | | |
| DDB_G0285019 | | | | DDB_G0285019 | | | |  | | | |
| DDB_G0285109 | | | | DDB_G0285109 | | | |  | | | |
| DDB_G0285127 | | | | DDB_G0285127 | | | |  | | | |
| DDB_G0285129 | | | | DDB_G0285129 | | | |  | | | |
| DDB_G0285169 | | | | DDB_G0285169 | | | |  | | | |
| DDB_G0285173 | | | | DDB_G0285173 | | | |  | | | |
| DDB_G0285225 | | | | DDB_G0285225 | | | |  | | | |
| DDB_G0285271 | | | | DDB_G0285271 | | | |  | | | |
| DDB_G0285325 | | | | DDB_G0285325 | | | | Pol. | | | |
| DDB_G0285351 | | | | DDB_G0285351 | | | |  | | | |
| DDB_G0285369 | | | | DDB_G0285369 | | | |  | | | |
| DDB_G0285385 | | | | DDB_G0285385 | | | |  | | | |
| DDB_G0285439 | | | | DDB_G0285439 | | | |  | | | |
| DDB_G0285457 | | | | DDB_G0285457 | | | |  | | | |
| DDB_G0285479 | | | | DDB_G0285479 | | | | Similar to Mus musculus (Mouse). tenascin X. | | | |
| DDB_G0285481 | | | | DDB_G0285481 | | | |  | | | |
| DDB_G0285483 | | | | DDB_G0285483 | | | |  | | | |
| DDB_G0285485 | | | | DDB_G0285485 | | | | Similar to plasmodium falciparum (Isolate 3D7). hypothetical 231.8 kDa protein. | | | |
| DDB_G0285487 | | | | DDB_G0285487 | | | | Similar to plasmodium falciparum (Isolate 3D7). hypothetical 231.8 kDa protein. | | | |
| DDB_G0285529 | | | | DDB_G0285529 | | | |  | | | |
| DDB_G0285531 | | | | DDB_G0285531 | | | |  | | | |
| DDB_G0285547 | | | | DDB_G0285547 | | | | Pol. | | | |
| DDB_G0285555 | | | | DDB_G0285555 | | | | Probable esterase D2 precursor. | | | |
| DDB_G0285565 | | | | DDB_G0285565 | | | | Similar to Mus musculus (Mouse). tenascin X. | | | |
| DDB_G0285585 | | | | DDB_G0285585 | | | | Similar to Mus musculus (Mouse). tenascin X. | | | |
| DDB_G0285611 | | | | DDB_G0285611 | | | |  | | | |
| DDB_G0285623 | | | | DDB_G0285623 | | | |  | | | |
| DDB_G0285625 | | | | DDB_G0285625 | | | |  | | | |
| DDB_G0285627 | | | | DDB_G0285627 | | | |  | | | |
| DDB_G0285629 | | | | DDB_G0285629 | | | |  | | | |
| DDB_G0285639 | | | | DDB_G0285639 | | | |  | | | |
| DDB_G0285661 | | | | DDB_G0285661 | | | |  | | | |
| DDB_G0285665 | | | | DDB_G0285665 | | | |  | | | |
| DDB_G0285667 | | | | DDB_G0285667 | | | |  | | | |
| DDB_G0285687 | | | | DDB_G0285687 | | | |  | | | |
| DDB_G0285691 | | | | DDB_G0285691 | | | |  | | | |
| DDB_G0285695 | | | | DDB_G0285695 | | | |  | | | |
| DDB_G0285707 | | | | DDB_G0285707 | | | |  | | | |
| DDB_G0285723 | | | | DDB_G0285723 | | | |  | | | |
| DDB_G0285799 | | | | DDB_G0285799 | | | |  | | | |
| DDB_G0285873 | | | | DDB_G0285873 | | | |  | | | |
| DDB_G0285883 | | | | DDB_G0285883 | | | | Similar to Dictyostelium discoideum (Slime mold). hypothetical 127.0 kDa protein. | | | |
| DDB_G0285905 | | | | DDB_G0285905 | | | |  | | | |
| DDB_G0286017 | | | | DDB_G0286017 | | | |  | | | |
| DDB_G0286039 | | | | DDB_G0286039 | | | |  | | | |
| DDB_G0286091 | | | | DDB_G0286091 | | | |  | | | |
| DDB_G0286145 | | | | DDB_G0286145 | | | | NIF. | | | |
| DDB_G0286153 | | | | DDB_G0286153 | | | |  | | | |
| DDB_G0286155 | | | | DDB_G0286155 | | | |  | | | |
| DDB_G0286157 | | | | DDB_G0286157 | | | | Similar to Drosophila melanogaster (Fruit fly). RE12057p. | | | |
| DDB_G0286201 | | | | DDB_G0286201 | | | |  | | | |
| DDB_G0286203 | | | | DDB_G0286203 | | | |  | | | |
| DDB_G0286209 | | | | DDB_G0286209 | | | |  | | | |
| DDB_G0286211 | | | | DDB_G0286211 | | | |  | | | |
| DDB_G0286215 | | | | DDB_G0286215 | | | |  | | | |
| DDB_G0286217 | | | | DDB_G0286217 | | | |  | | | |
| DDB_G0286243 | | | | DDB_G0286243 | | | |  | | | |
| DDB_G0286245 | | | | DDB_G0286245 | | | |  | | | |
| DDB_G0286267 | | | | DDB_G0286267 | | | |  | | | |
| DDB_G0286281 | | | | DDB_G0286281 | | | |  | | | |
| DDB_G0286283 | | | | DDB_G0286283 | | | |  | | | |
| DDB_G0286291 | | | | DDB_G0286291 | | | |  | | | |
| DDB_G0286367 | | | | DDB_G0286367 | | | |  | | | |
| DDB_G0286369 | | | | DDB_G0286369 | | | |  | | | |
| DDB_G0286373 | | | | DDB_G0286373 | | | |  | | | |
| DDB_G0286381 | | | | DDB_G0286381 | | | |  | | | |
| DDB_G0286415 | | | | DDB_G0286415 | | | |  | | | |
| DDB_G0286417 | | | | DDB_G0286417 | | | | Similar to Homo sapiens (Human). Tenascin (TN) (Hexabrachion) (Cytotactin) (Neuronectin) (GMEM) (JI) (Miotendinous antigen) (Glioma-associated-extracellular matrix antigen) (GP 150-225) (Tenascin-C) (TN-C). | | | |
| DDB_G0286431 | | | | DDB_G0286431 | | | |  | | | |
| DDB_G0286433 | | | | DDB_G0286433 | | | |  | | | |
| DDB_G0286437 | | | | DDB_G0286437 | | | |  | | | |
| DDB_G0286537 | | | | DDB_G0286537 | | | |  | | | |
| DDB_G0286567 | | | | DDB_G0286567 | | | |  | | | |
| DDB_G0286575 | | | | DDB_G0286575 | | | |  | | | |
| DDB_G0286649 | | | | DDB_G0286649 | | | |  | | | |
| DDB_G0286659 | | | | DDB_G0286659 | | | |  | | | |
| DDB_G0286673 | | | | DDB_G0286673 | | | |  | | | |
| DDB_G0286737 | | | | DDB_G0286737 | | | |  | | | |
| DDB_G0286771 | | | | DDB_G0286771 | | | |  | | | |
| DDB_G0286849 | | | | DDB_G0286849 | | | | Similar to Rhizobium loti (Mesorhizobium loti). short-chain oxidoreductase. | | | |
| DDB_G0286869 | | | | DDB_G0286869 | | | |  | | | |
| DDB_G0286873 | | | | DDB_G0286873 | | | |  | | | |
| DDB_G0286877 | | | | DDB_G0286877 | | | |  | | | |
| DDB_G0286881 | | | | DDB_G0286881 | | | |  | | | |
| DDB_G0286911 | | | | DDB_G0286911 | | | |  | | | |
| DDB_G0286913 | | | | DDB_G0286913 | | | |  | | | |
| DDB_G0286917 | | | | DDB_G0286917 | | | |  | | | |
| DDB_G0286921 | | | | DDB_G0286921 | | | |  | | | |
| DDB_G0286933 | | | | DDB_G0286933 | | | |  | | | |
| DDB_G0286935 | | | | DDB_G0286935 | | | |  | | | |
| DDB_G0286947 | | | | DDB_G0286947 | | | |  | | | |
| DDB_G0286955 | | | | DDB_G0286955 | | | |  | | | |
| DDB_G0286957 | | | | DDB_G0286957 | | | |  | | | |
| DDB_G0287047 | | | | DDB_G0287047 | | | |  | | | |
| DDB_G0287065 | | | | DDB_G0287065 | | | |  | | | |
| DDB_G0287083 | | | | DDB_G0287083 | | | | Putative calmodulin-binding protein CaM-BP46. | | | |
| DDB_G0287085 | | | | DDB_G0287085 | | | |  | | | |
| DDB_G0287089 | | | | DDB_G0287089 | | | |  | | | |
| DDB_G0287091 | | | | DDB_G0287091 | | | |  | | | |
| DDB_G0287097 | | | | DDB_G0287097 | | | |  | | | |
| DDB_G0287133 | | | | DDB_G0287133 | | | |  | | | |
| DDB_G0287137 | | | | DDB_G0287137 | | | | Vacuolar H+-ATPase A1 subunit isoform. | | | |
| DDB_G0287151 | | | | DDB_G0287151 | | | |  | | | |
| DDB_G0287167 | | | | DDB_G0287167 | | | | Similar to Dictyostelium discoideum (Slime mold). hypothetical 127.0 kDa protein. | | | |
| DDB_G0287215 | | | | DDB_G0287215 | | | |  | | | |
| DDB_G0287235 | | | | DDB_G0287235 | | | |  | | | |
| DDB_G0287275 | | | | DDB_G0287275 | | | |  | | | |
| DDB_G0287301 | | | | DDB_G0287301 | | | |  | | | |
| DDB_G0287305 | | | | DDB_G0287305 | | | |  | | | |
| DDB_G0287311 | | | | DDB_G0287311 | | | |  | | | |
| DDB_G0287329 | | | | DDB_G0287329 | | | |  | | | |
| DDB_G0287339 | | | | DDB_G0287339 | | | |  | | | |
| DDB_G0287349 | | | | DDB_G0287349 | | | |  | | | |
| DDB_G0287357 | | | | DDB_G0287357 | | | | Similar to Dictyostelium discoideum (Slime mold). vegetative stage specific V4-7. | | | |
| DDB_G0287375 | | | | DDB_G0287375 | | | |  | | | |
| DDB_G0287389 | | | | DDB_G0287389 | | | |  | | | |
| DDB_G0287411 | | | | DDB_G0287411 | | | |  | | | |
| DDB_G0287475 | | | | DDB_G0287475 | | | |  | | | |
| DDB_G0287511 | | | | DDB_G0287511 | | | | Similar to G-protein-coupled receptor at plasma membrane; interactions in two-hybrid system with Gpa2p; Gpr1p. | | | |
| DDB_G0287523 | | | | DDB_G0287523 | | | |  | | | |
| DDB_G0287535 | | | | DDB_G0287535 | | | |  | | | |
| DDB_G0287549 | | | | DDB_G0287549 | | | | P15519 Spore germination protein 1 precursor (SPG1). | | | |
| DDB_G0287605 | | | | DDB_G0287605 | | | |  | | | |
| DDB_G0287611 | | | | DDB_G0287611 | | | | P22549 Cyclic nucleotide phosphodiesterase inhibitor precursor (PDI). | | | |
| DDB_G0287613 | | | | DDB_G0287613 | | | |  | | | |
| DDB_G0287615 | | | | DDB_G0287615 | | | |  | | | |
| DDB_G0287641 | | | | DDB_G0287641 | | | |  | | | |
| DDB_G0287701 | | | | DDB_G0287701 | | | |  | | | |
| DDB_G0287703 | | | | DDB_G0287703 | | | | Similar to Dictyostelium discoideum (Slime mold). hypothetical 127.0 kDa protein. | | | |
| DDB_G0287705 | | | | DDB_G0287705 | | | |  | | | |
| DDB_G0287771 | | | | DDB_G0287771 | | | |  | | | |
| DDB_G0287785 | | | | DDB_G0287785 | | | |  | | | |
| DDB_G0287789 | | | | DDB_G0287789 | | | |  | | | |
| DDB_G0287793 | | | | DDB_G0287793 | | | |  | | | |
| DDB_G0287797 | | | | DDB_G0287797 | | | |  | | | |
| DDB_G0287841 | | | | DDB_G0287841 | | | |  | | | |
| DDB_G0287845 | | | | DDB_G0287845 | | | |  | | | |
| DDB_G0287931 | | | | DDB_G0287931 | | | |  | | | |
| DDB_G0287955 | | | | DDB_G0287955 | | | |  | | | |
| DDB_G0287971 | | | | DDB_G0287971 | | | |  | | | |
| DDB_G0287985 | | | | DDB_G0287985 | | | |  | | | |
| DDB_G0288013 | | | | DDB_G0288013 | | | |  | | | |
| DDB_G0288049 | | | | DDB_G0288049 | | | |  | | | |
| DDB_G0288085 | | | | DDB_G0288085 | | | |  | | | |
| DDB_G0288097 | | | | DDB_G0288097 | | | |  | | | |
| DDB_G0288151 | | | | DDB_G0288151 | | | |  | | | |
| DDB_G0288189 | | | | DDB_G0288189 | | | |  | | | |
| DDB_G0288221 | | | | DDB_G0288221 | | | | Gamete and mating-type specific protein A. | | | |
| DDB_G0288301 | | | | DDB_G0288301 | | | | Similar to Dictyostelium discoideum (Slime mold). Hypothetical 127.0 kDa protein. | | | |
| DDB_G0288339 | | | | DDB_G0288339 | | | |  | | | |
| DDB_G0288343 | | | | DDB_G0288343 | | | |  | | | |
| DDB_G0288381 | | | | DDB_G0288381 | | | |  | | | |
| DDB_G0288403 | | | | DDB_G0288403 | | | |  | | | |
| DDB_G0288435 | | | | DDB_G0288435 | | | |  | | | |
| DDB_G0288497 | | | | DDB_G0288497 | | | |  | | | |
| DDB_G0288535 | | | | DDB_G0288535 | | | | Similar to Rhizobium meliloti (Sinorhizobium meliloti). putative transport transmembrane protein. | | | |
| DDB_G0288619 | | | | DDB_G0288619 | | | |  | | | |
| DDB_G0288631 | | | | DDB_G0288631 | | | | Gll1470 protein. | | | |
| DDB_G0288665 | | | | DDB_G0288665 | | | |  | | | |
| DDB_G0288669 | | | | DDB_G0288669 | | | |  | | | |
| DDB_G0288687 | | | | DDB_G0288687 | | | |  | | | |
| DDB_G0288689 | | | | DDB_G0288689 | | | |  | | | |
| DDB_G0288695 | | | | DDB_G0288695 | | | |  | | | |
| DDB_G0288733 | | | | DDB_G0288733 | | | |  | | | |
| DDB_G0288775 | | | | DDB_G0288775 | | | |  | | | |
| DDB_G0288781 | | | | DDB_G0288781 | | | |  | | | |
| DDB_G0288789 | | | | DDB_G0288789 | | | |  | | | |
| DDB_G0288791 | | | | DDB_G0288791 | | | |  | | | |
| DDB_G0288793 | | | | DDB_G0288793 | | | |  | | | |
| DDB_G0288837 | | | | DDB_G0288837 | | | |  | | | |
| DDB_G0288843 | | | | DDB_G0288843 | | | |  | | | |
| DDB_G0288845 | | | | DDB_G0288845 | | | |  | | | |
| DDB_G0288853 | | | | DDB_G0288853 | | | |  | | | |
| DDB_G0288881 | | | | DDB_G0288881 | | | |  | | | |
| DDB_G0288885 | | | | DDB_G0288885 | | | |  | | | |
| DDB_G0288889 | | | | DDB_G0288889 | | | |  | | | |
| DDB_G0288901 | | | | DDB_G0288901 | | | |  | | | |
| DDB_G0288995 | | | | DDB_G0288995 | | | |  | | | |
| DDB_G0289023 | | | | DDB_G0289023 | | | |  | | | |
| DDB_G0289057 | | | | DDB_G0289057 | | | |  | | | |
| DDB_G0289065 | | | | DDB_G0289065 | | | |  | | | |
| DDB_G0289083 | | | | DDB_G0289083 | | | |  | | | |
| DDB_G0289091 | | | | DDB_G0289091 | | | |  | | | |
| DDB_G0289095 | | | | DDB_G0289095 | | | |  | | | |
| DDB_G0289097 | | | | DDB_G0289097 | | | |  | | | |
| DDB_G0289133 | | | | DDB_G0289133 | | | |  | | | |
| DDB_G0289153 | | | | DDB_G0289153 | | | |  | | | |
| DDB_G0289147 | | | | DDB_G0289147 | | | |  | | | |
| DDB_G0289167 | | | | DDB_G0289167 | | | | Similar to Rhizobium loti (Mesorhizobium loti). Short-chain oxidoreductase. | | | |
| DDB_G0289227 | | | | DDB_G0289227 | | | |  | | | |
| DDB_G0289249 | | | | DDB_G0289249 | | | |  | | | |
| DDB_G0289287 | | | | DDB_G0289287 | | | |  | | | |
| DDB_G0289297 | | | | DDB_G0289297 | | | |  | | | |
| DDB_G0289299 | | | | DDB_G0289299 | | | |  | | | |
| DDB_G0289357 | | | | DDB_G0289357 | | | |  | | | |
| DDB_G0289399 | | | | DDB_G0289399 | | | | Similar to Gallus gallus (Chicken). 190 kDa tenascin. | | | |
| DDB_G0289403 | | | | DDB_G0289403 | | | |  | | | |
| DDB_G0289409 | | | | DDB_G0289409 | | | |  | | | |
| DDB_G0289503 | | | | DDB_G0289503 | | | | Q9VYS3 Regulator of nonsense transcripts 1 homolog. | | | |
| DDB_G0289575 | | | | DDB_G0289575 | | | |  | | | |
| DDB_G0289603 | | | | DDB_G0289603 | | | |  | | | |
| DDB_G0289605 | | | | DDB_G0289605 | | | |  | | | |
| DDB_G0289617 | | | | DDB_G0289617 | | | |  | | | |
| DDB_G0289619 | | | | DDB_G0289619 | | | | Q94481 Protein cigB (Fragment). | | | |
| DDB_G0289669 | | | | DDB_G0289669 | | | |  | | | |
| DDB_G0289683 | | | | DDB_G0289683 | | | |  | | | |
| DDB_G0289685 | | | | DDB_G0289685 | | | |  | | | |
| DDB_G0289687 | | | | DDB_G0289687 | | | |  | | | |
| DDB_G0289717 | | | | DDB_G0289717 | | | | Q94481 Protein cigB (Fragment). | | | |
| DDB_G0289739 | | | | DDB_G0289739 | | | | Q94481 Protein cigB (Fragment). | | | |
| DDB_G0289747 | | | | DDB_G0289747 | | | |  | | | |
| DDB_G0289751 | | | | DDB_G0289751 | | | | Q94481 Protein cigB (Fragment). | | | |
| DDB_G0289757 | | | | DDB_G0289757 | | | |  | | | |
| DDB_G0289759 | | | | DDB_G0289759 | | | |  | | | |
| DDB_G0289761 | | | | DDB_G0289761 | | | |  | | | |
| DDB_G0289775 | | | | DDB_G0289775 | | | |  | | | |
| DDB_G0289777 | | | | DDB_G0289777 | | | | Similar to Dictyostelium discoideum (Slime mold). CIGB protein. | | | |
| DDB_G0289781 | | | | DDB_G0289781 | | | |  | | | |
| DDB_G0289817 | | | | DDB_G0289817 | | | | Q94481 Protein cigB (Fragment). | | | |
| DDB_G0289821 | | | | DDB_G0289821 | | | | Q94481 Protein cigB (Fragment). | | | |
| DDB_G0289825 | | | | DDB_G0289825 | | | |  | | | |
| DDB_G0289837 | | | | DDB_G0289837 | | | |  | | | |
| DDB_G0289839 | | | | DDB_G0289839 | | | |  | | | |
| DDB_G0289841 | | | | DDB_G0289841 | | | |  | | | |
| DDB_G0289843 | | | | DDB_G0289843 | | | |  | | | |
| DDB_G0289845 | | | | DDB_G0289845 | | | |  | | | |
| DDB_G0289847 | | | | DDB_G0289847 | | | |  | | | |
| DDB_G0289889 | | | | DDB_G0289889 | | | |  | | | |
| DDB_G0289895 | | | | DDB_G0289895 | | | |  | | | |
| DDB_G0289897 | | | | DDB_G0289897 | | | |  | | | |
| DDB_G0289903 | | | | DDB_G0289903 | | | |  | | | |
| DDB_G0289927 | | | | DDB_G0289927 | | | |  | | | |
| DDB_G0289929 | | | | DDB_G0289929 | | | |  | | | |
| DDB_G0289931 | | | | DDB_G0289931 | | | |  | | | |
| DDB_G0289933 | | | | DDB_G0289933 | | | |  | | | |
| DDB_G0289937 | | | | DDB_G0289937 | | | |  | | | |
| DDB_G0289947 | | | | DDB_G0289947 | | | |  | | | |
| DDB_G0289991 | | | | DDB_G0289991 | | | |  | | | |
| DDB_G0290039 | | | | DDB_G0290039 | | | |  | | | |
| DDB_G0290043 | | | | DDB_G0290043 | | | | Q94481 Protein cigB (Fragment). | | | |
| DDB_G0290175 | | | | DDB_G0290175 | | | |  | | | |
| DDB_G0290199 | | | | DDB_G0290199 | | | |  | | | |
| DDB_G0290219 | | | | DDB_G0290219 | | | |  | | | |
| DDB_G0290285 | | | | DDB_G0290285 | | | | Similar to Dictyostelium discoideum (Slime mold). hypothetical 127.0 kDa protein. | | | |
| DDB_G0290287 | | | | DDB_G0290287 | | | | Similar to Dictyostelium discoideum (Slime mold). Hypothetical 127.0 kDa protein. | | | |
| DDB_G0290321 | | | | DDB_G0290321 | | | |  | | | |
| DDB_G0290323 | | | | DDB_G0290323 | | | |  | | | |
| DDB_G0290335 | | | | DDB_G0290335 | | | |  | | | |
| DDB_G0290399 | | | | DDB_G0290399 | | | |  | | | |
| DDB_G0290411 | | | | DDB_G0290411 | | | |  | | | |
| DDB_G0290423 | | | | DDB_G0290423 | | | |  | | | |
| DDB_G0290441 | | | | DDB_G0290441 | | | |  | | | |
| DDB_G0290489 | | | | DDB_G0290489 | | | |  | | | |
| DDB_G0290505 | | | | DDB_G0290505 | | | | Q94481 Protein cigB (Fragment). | | | |
| DDB_G0290507 | | | | DDB_G0290507 | | | | Similar to Dictyostelium discoideum (Slime mold). CIGB protein. | | | |
| DDB_G0290511 | | | | DDB_G0290511 | | | |  | | | |
| DDB_G0290513 | | | | DDB_G0290513 | | | |  | | | |
| DDB_G0290525 | | | | DDB_G0290525 | | | |  | | | |
| DDB_G0290527 | | | | DDB_G0290527 | | | | Similar to Dictyostelium discoideum (Slime mold). CIGB protein. | | | |
| DDB_G0290547 | | | | DDB_G0290547 | | | | P46595 Ubiquitin-conjugating enzyme E2 4 (EC 6.3.2.19) (Ubiquitin-protein ligase 4) (Ubiquitin carrier protein 4). | | | |
| DDB_G0290559 | | | | DDB_G0290559 | | | | Q94481 Protein cigB (Fragment). | | | |
| DDB_G0290561 | | | | DDB_G0290561 | | | | Q94481 Protein cigB (Fragment). | | | |
| DDB_G0290565 | | | | DDB_G0290565 | | | | Q94481 Protein cigB (Fragment). | | | |
| DDB_G0290567 | | | | DDB_G0290567 | | | | Similar to Dictyostelium discoideum (Slime mold). CIGB protein. | | | |
| DDB_G0290569 | | | | DDB_G0290569 | | | | Q94481 Protein cigB (Fragment). | | | |
| DDB_G0290571 | | | | DDB_G0290571 | | | | Q94481 Protein cigB (Fragment). | | | |
| DDB_G0290601 | | | | DDB_G0290601 | | | |  | | | |
| DDB_G0290609 | | | | DDB_G0290609 | | | |  | | | |
| DDB_G0290611 | | | | DDB_G0290611 | | | | Q94481 Protein cigB (Fragment). | | | |
| DDB_G0290613 | | | | DDB_G0290613 | | | | Q94481 Protein cigB (Fragment). | | | |
| DDB_G0290623 | | | | DDB_G0290623 | | | | Similar to Mus musculus (Mouse). DNA segment, Chr 11, Wayne state University 80, expressed. | | | |
| DDB_G0290625 | | | | DDB_G0290625 | | | | Similar to Dictyostelium discoideum (Slime mold). CIGB protein. | | | |
| DDB_G0290627 | | | | DDB_G0290627 | | | |  | | | |
| DDB_G0290635 | | | | DDB_G0290635 | | | | Similar to Dictyostelium discoideum (Slime mold). hypothetical 127.0 kDa protein. | | | |
| DDB_G0290727 | | | | DDB_G0290727 | | | |  | | | |
| DDB_G0290797 | | | | DDB_G0290797 | | | |  | | | |
| DDB_G0290805 | | | | DDB_G0290805 | | | |  | | | |
| DDB_G0290807 | | | | DDB_G0290807 | | | |  | | | |
| DDB_G0290809 | | | | DDB_G0290809 | | | |  | | | |
| DDB_G0290811 | | | | DDB_G0290811 | | | |  | | | |
| DDB_G0290813 | | | | DDB_G0290813 | | | |  | | | |
| DDB_G0290815 | | | | DDB_G0290815 | | | |  | | | |
| DDB_G0290817 | | | | DDB_G0290817 | | | | Q94481 Protein cigB (Fragment). | | | |
| DDB_G0290821 | | | | DDB_G0290821 | | | |  | | | |
| DDB_G0290841 | | | | DDB_G0290841 | | | | Similar to Dictyostelium discoideum (Slime mold). Hypothetical 127.0 kDa protein. | | | |
| DDB_G0290861 | | | | DDB_G0290861 | | | | Q94481 Protein cigB (Fragment). | | | |
| DDB_G0290881 | | | | DDB_G0290881 | | | |  | | | |
| DDB_G0290915 | | | | DDB_G0290915 | | | |  | | | |
| DDB_G0290933 | | | | DDB_G0290933 | | | |  | | | |
| DDB_G0290935 | | | | DDB_G0290935 | | | | P11467 DG17 protein. | | | |
| DDB_G0290941 | | | | DDB_G0290941 | | | | P11467 DG17 protein. | | | |
| DDB_G0290969 | | | | DDB_G0290969 | | | |  | | | |
| DDB_G0290971 | | | | DDB_G0290971 | | | |  | | | |
| DDB_G0290989 | | | | DDB_G0290989 | | | |  | | | |
| DDB_G0290995 | | | | DDB_G0290995 | | | | Erythrocyte band 7 membrane protein homolog. | | | |
| DDB_G0290999 | | | | DDB_G0290999 | | | |  | | | |
| DDB_G0291011 | | | | DDB_G0291011 | | | |  | | | |
| DDB_G0291023 | | | | DDB_G0291023 | | | |  | | | |
| DDB_G0291025 | | | | DDB_G0291025 | | | |  | | | |
| DDB_G0291027 | | | | DDB_G0291027 | | | | Similar to Dictyostelium discoideum (Slime mold). hypothetical 127.0 kDa protein. | | | |
| DDB_G0291033 | | | | DDB_G0291033 | | | |  | | | |
| DDB_G0291137 | | | | DDB_G0291137 | | | |  | | | |
| DDB_G0291035 | | | | DDB_G0291035 | | | | P11467 DG17 protein. | | | |
| DDB_G0291153 | | | | DDB_G0291153 | | | |  | | | |
| DDB_G0291169 | | | | DDB_G0291169 | | | |  | | | |
| DDB_G0291171 | | | | DDB_G0291171 | | | |  | | | |
| DDB_G0267530 | | | | DDB_G0267530 | | | |  | | | |
| DDB_G0267550 | | | | DDB_G0267550 | | | |  | | | |
| DDB_G0267564 | | | | DDB_G0267564 | | | |  | | | |
| DDB_G0267580 | | | | DDB_G0267580 | | | |  | | | |
| DDB_G0267612 | | | | DDB_G0267612 | | | |  | | | |
| DDB_G0267614 | | | | DDB_G0267614 | | | |  | | | |
| DDB_G0267616 | | | | DDB_G0267616 | | | |  | | | |
| DDB_G0267624 | | | | DDB_G0267624 | | | |  | | | |
| DDB_G0267634 | | | | DDB_G0267634 | | | | Cell differentiation protein rcd1, putative. | | | |
| DDB_G0267654 | | | | DDB_G0267654 | | | |  | | | |
| DDB_G0267672 | | | | DDB_G0267672 | | | |  | | | |
| DDB_G0267694 | | | | DDB_G0267694 | | | |  | | | |
| DDB_G0267700 | | | | DDB_G0267700 | | | |  | | | |
| DDB_G0267710 | | | | DDB_G0267710 | | | |  | | | |
| DDB_G0267724 | | | | DDB_G0267724 | | | |  | | | |
| DDB_G0267740 | | | | DDB_G0267740 | | | |  | | | |
| DDB_G0267744 | | | | DDB_G0267744 | | | |  | | | |
| DDB_G0267756 | | | | DDB_G0267756 | | | |  | | | |
| DDB_G0267758 | | | | DDB_G0267758 | | | | AW551984 protein. | | | |
| DDB_G0267780 | | | | DDB_G0267780 | | | | Pol. | | | |
| DDB_G0267798 | | | | DDB_G0267798 | | | |  | | | |
| DDB_G0267804 | | | | DDB_G0267804 | | | |  | | | |
| DDB_G0267814 | | | | DDB_G0267814 | | | |  | | | |
| DDB_G0267856 | | | | DDB_G0267856 | | | |  | | | |
| DDB_G0267858 | | | | DDB_G0267858 | | | |  | | | |
| DDB_G0267878 | | | | DDB_G0267878 | | | |  | | | |
| DDB_G0267886 | | | | DDB_G0267886 | | | |  | | | |
| DDB_G0267892 | | | | DDB_G0267892 | | | |  | | | |
| DDB_G0267914 | | | | DDB_G0267914 | | | |  | | | |
| DDB_G0267938 | | | | DDB_G0267938 | | | |  | | | |
| DDB_G0267968 | | | | DDB_G0267968 | | | |  | | | |
| DDB_G0267972 | | | | DDB_G0267972 | | | |  | | | |
| DDB_G0268072 | | | | DDB_G0268072 | | | |  | | | |
| DDB_G0268096 | | | | DDB_G0268096 | | | |  | | | |
| DDB_G0268106 | | | | DDB_G0268106 | | | |  | | | |
| DDB_G0268108 | | | | DDB_G0268108 | | | |  | | | |
| DDB_G0268142 | | | | DDB_G0268142 | | | |  | | | |
| DDB_G0268152 | | | | DDB_G0268152 | | | |  | | | |
| DDB_G0268164 | | | | DDB_G0268164 | | | |  | | | |
| DDB_G0268176 | | | | DDB_G0268176 | | | |  | | | |
| DDB_G0268184 | | | | DDB_G0268184 | | | |  | | | |
| DDB_G0268188 | | | | DDB_G0268188 | | | |  | | | |
| DDB_G0268206 | | | | DDB_G0268206 | | | |  | | | |
| DDB_G0268210 | | | | DDB_G0268210 | | | |  | | | |
| DDB_G0268250 | | | | DDB_G0268250 | | | |  | | | |
| DDB_G0268254 | | | | DDB_G0268254 | | | |  | | | |
| DDB_G0268256 | | | | DDB_G0268256 | | | |  | | | |
| DDB_G0268662 | | | | DDB_G0268662 | | | | Similar to Neurospora crassa. related to SEN1 protein. | | | |
| DDB_G0268682 | | | | DDB_G0268682 | | | |  | | | |
| DDB_G0268692 | | | | DDB_G0268692 | | | |  | | | |
| DDB_G0268700 | | | | DDB_G0268700 | | | |  | | | |
| DDB_G0268710 | | | | DDB_G0268710 | | | |  | | | |
| DDB_G0268724 | | | | DDB_G0268724 | | | | Pol. | | | |
| DDB_G0268734 | | | | DDB_G0268734 | | | |  | | | |
| DDB_G0268756 | | | | DDB_G0268756 | | | |  | | | |
| DDB_G0268764 | | | | DDB_G0268764 | | | |  | | | |
| DDB_G0268772 | | | | DDB_G0268772 | | | |  | | | |
| DDB_G0268782 | | | | DDB_G0268782 | | | |  | | | |
| DDB_G0268788 | | | | DDB_G0268788 | | | |  | | | |
| DDB_G0268806 | | | | DDB_G0268806 | | | |  | | | |
| DDB_G0268846 | | | | DDB_G0268846 | | | |  | | | |
| DDB_G0268858 | | | | DDB_G0268858 | | | | P80 protein. | | | |
| DDB_G0268864 | | | | DDB_G0268864 | | | |  | | | |
| DDB_G0268906 | | | | DDB_G0268906 | | | |  | | | |
| DDB_G0269258 | | | | DDB_G0269258 | | | | Pol. | | | |
| DDB_G0269264 | | | | DDB_G0269264 | | | | Pol. | | | |
| DDB_G0269266 | | | | DDB_G0269266 | | | | Non-LTR retroelement reverse transcriptase-like protein. | | | |
| DDB_G0269272 | | | | DDB_G0269272 | | | | BcDNA:GH04637 protein. | | | |
| DDB_G0269320 | | | | DDB_G0269320 | | | |  | | | |
| DDB_G0269350 | | | | DDB_G0269350 | | | |  | | | |
| DDB_G0269384 | | | | DDB_G0269384 | | | |  | | | |
| DDB_G0269398 | | | | DDB_G0269398 | | | |  | | | |
| DDB_G0269426 | | | | DDB_G0269426 | | | |  | | | |
| DDB_G0269428 | | | | DDB_G0269428 | | | |  | | | |
| DDB_G0269452 | | | | DDB_G0269452 | | | |  | | | |
| DDB_G0269494 | | | | DDB_G0269494 | | | |  | | | |
| DDB_G0269504 | | | | DDB_G0269504 | | | |  | | | |
| DDB_G0269506 | | | | DDB_G0269506 | | | |  | | | |
| DDB_G0269508 | | | | DDB_G0269508 | | | |  | | | |
| DDB_G0269530 | | | | DDB_G0269530 | | | |  | | | |
| DDB_G0269534 | | | | DDB_G0269534 | | | |  | | | |
| DDB_G0269536 | | | | DDB_G0269536 | | | |  | | | |
| DDB_G0269538 | | | | DDB_G0269538 | | | |  | | | |
| DDB_G0269540 | | | | DDB_G0269540 | | | |  | | | |
| DDB_G0269542 | | | | DDB_G0269542 | | | | Pol. | | | |
| DDB_G0269562 | | | | DDB_G0269562 | | | |  | | | |
| DDB_G0269582 | | | | DDB_G0269582 | | | |  | | | |
| DDB_G0269584 | | | | DDB_G0269584 | | | |  | | | |
| DDB_G0269592 | | | | DDB_G0269592 | | | |  | | | |
| DDB_G0269600 | | | | DDB_G0269600 | | | |  | | | |
| DDB_G0269606 | | | | DDB_G0269606 | | | |  | | | |
| DDB_G0269654 | | | | DDB_G0269654 | | | |  | | | |
| DDB_G0269732 | | | | DDB_G0269732 | | | |  | | | |
| DDB_G0269758 | | | | DDB_G0269758 | | | |  | | | |
| DDB_G0269766 | | | | DDB_G0269766 | | | |  | | | |
| DDB_G0269782 | | | | DDB_G0269782 | | | |  | | | |
| DDB_G0269852 | | | | DDB_G0269852 | | | |  | | | |
| DDB_G0269874 | | | | DDB_G0269874 | | | |  | | | |
| DDB_G0269930 | | | | DDB_G0269930 | | | |  | | | |
| DDB_G0269954 | | | | DDB_G0269954 | | | |  | | | |
| DDB_G0269956 | | | | DDB_G0269956 | | | | Pol. | | | |
| DDB_G0269992 | | | | DDB_G0269992 | | | |  | | | |
| DDB_G0270000 | | | | DDB_G0270000 | | | |  | | | |
| DDB_G0270016 | | | | DDB_G0270016 | | | |  | | | |
| DDB_G0270030 | | | | DDB_G0270030 | | | |  | | | |
| DDB_G0270034 | | | | DDB_G0270034 | | | |  | | | |
| DDB_G0270038 | | | | DDB_G0270038 | | | |  | | | |
| DDB_G0270048 | | | | DDB_G0270048 | | | |  | | | |
| DDB_G0270050 | | | | DDB_G0270050 | | | |  | | | |
| DDB_G0270058 | | | | DDB_G0270058 | | | |  | | | |
| DDB_G0270066 | | | | DDB_G0270066 | | | |  | | | |
| DDB_G0270076 | | | | DDB_G0270076 | | | |  | | | |
| DDB_G0270078 | | | | DDB_G0270078 | | | |  | | | |
| DDB_G0270080 | | | | DDB_G0270080 | | | |  | | | |
| DDB_G0270094 | | | | DDB_G0270094 | | | |  | | | |
| DDB_G0270116 | | | | DDB_G0270116 | | | |  | | | |
| DDB_G0270132 | | | | DDB_G0270132 | | | |  | | | |
| DDB_G0270160 | | | | DDB_G0270160 | | | |  | | | |
| DDB_G0270232 | | | | DDB_G0270232 | | | |  | | | |
| DDB_G0270244 | | | | DDB_G0270244 | | | |  | | | |
| DDB_G0270286 | | | | DDB_G0270286 | | | |  | | | |
| DDB_G0270300 | | | | DDB_G0270300 | | | |  | | | |
| DDB_G0270310 | | | | DDB_G0270310 | | | |  | | | |
| DDB_G0270326 | | | | DDB_G0270326 | | | |  | | | |
| DDB_G0270350 | | | | DDB_G0270350 | | | |  | | | |
| DDB_G0270390 | | | | DDB_G0270390 | | | |  | | | |
| DDB_G0270408 | | | | DDB_G0270408 | | | |  | | | |
| DDB_G0270454 | | | | DDB_G0270454 | | | |  | | | |
| DDB_G0291081 | | | | pefA | | | | penta EF hand calcium binding protein | | | |
| DDB_G0268638 | | | | dstB | | | | signal transducer and activator of transcription (STAT) family protein | | | |
| DDB_G0288245 | | | | cigA | | | | P14606 Cyclic AMP-inducible protein BP74 precursor. | | | |
| kif2 | | | | kif2 | | | | belongs to the NCD%2FKar3 subfamily%2C predicted to play a role in mitosis | | | |
| DDB_G0269136 | | | | efaAII | | | | elongation factor 1 alpha, elongation factor 1a | | | |
| DDB_G0283389 | | | | rhgA | | | | Rh-like protein/ammonium transporter, Rhesus-like glycoprotein | | | |
| DDB_G0283599 | | | | limA | | | | LIM-type zinc finger-containing protein | | | |
| DDB_G0269106 | | | | cahA | | | | carbonic anhydrase | | | |
| DDB_G0269168 | | | | vps26 | | | | vacuolar protein sorting-associated protein 26 | | | |
| DDB_G0286119 | | | | tagB | | | | ABC transporter B family protein, serine protease | | | |
| DDB_G0274119 | | | | abcA5 | | | | ABC transporter A family protein | | | |
| DDB_G0274117 | | | | abcG8 | | | | ABC transporter G family protein | | | |
| DDB_G0267432 | | | | abcG15 | | | | ABC transporter G family protein | | | |
| DDB_G0269208 | | | | abcG19 | | | | ABC transporter G family protein | | | |
| DDB_G0272795 | | | | 3B-1 | | | | prespore-specific protein | | | |
| DDB_G0286185 | | | | gpaE | | | | G-protein subunit alpha 5 | | | |
| DDB_G0286187 | | | | proB | | | | profilin II | | | |
| DDB_G0285419 | | | | cryS | | | | crystal protein | | | |
| DDB_G0269146 | | | | ifdA | | | | DEAD/DEAH box helicase domain-containing protein, eukaryotic translation initiation factor 4A | | | |
| DDB_G0289389 | | | | dtfA | | | | cell surface protein DTFA | | | |
| DDB_G0267474 | | | | sigD | | | | spore coat protein | | | |
| DDB_G0268636 | | | | roco11 | | | | ROCO family protein kinase, leucine-rich repeat-containing protein (LRR), protein kinase, TKL group, tyrosine kinase-like protein | | | |
| DDB_G0275439 | | | | cad2 | | | | putative adhesion molecule | | | |
| DDB_G0290067 | | | | cigB | | | | B-box zinc finger-containing protein, FNIP repeat-containing protein | | | |
| DDB_G0269164 | | | | egeB | | | | C2 domain-containing protein | | | |
| DDB_G0269228 | | | | DDB_G0269228 | | | |  | | | |
| DDB_G0269126 | | | | DG1041 | | | | unknown | | | |
| DDB_G0273399 | | | | mip1-1 | | | | MEK1 interacting protein 1 | | | |
| DDB_G0276967 | | | | racF2 | | | | Rho GTPase | | | |
| DDB_G0273261 | | | | sigL | | | | EGF-like domain-containing protein | | | |
| DDB_G0290779 | | | | rabL | | | | GTP binding protein RARE7L, Rab GTPase | | | |
| DDB_G0289815 | | | | cupB | | | | calcium up-regulated protein, ricin B lectin domain-containing protein | | | |
| DDB_G0289283 | | | | cupC | | | | calcium up-regulated protein, ricin B lectin domain-containing protein | | | |
| DDB_G0289813 | | | | cupF | | | | calcium up-regulated protein, ricin B lectin domain-containing protein | | | |
| DDB_G0289883 | | | | cupG | | | | calcium up-regulated protein, ricin B lectin domain-containing protein | | | |
| DDB_G0287687 | | | | gerB | | | | 109 gene 2 protein, germination protein (p109) | | | |
| DDB_G0283609 | | | | cbpG | | | | calcium-binding protein | | | |
| DDB_G0283611 | | | | cbpF | | | | calcium-binding protein | | | |
| kif4 | | | | kif4 | | | | belongs to the CENP-E subfamily%2C predicted to play a role in mitosis%3B has a putative nuclear localization signal C-terminal to its motor domain | | | |
| DDB_G0283613 | | | | cbpC | | | | calcium-binding protein | | | |
| DDB_G0286561 | | | | salA | | | | SAPLIP C protein | | | |
| DDB_G0283083 | | | | cbpD2 | | | | calcium-binding protein | | | |
| DDB_G0287211 | | | | rab11B | | | | Rab GTPase | | | |
| DDB_G0289393 | | | | psiH | | | | PA14 domain-containing protein | | | |
| DDB_G0286123 | | | | tagD | | | | ABC transporter B family protein, serine protease | | | |
| DDB_G0290259 | | | | GP138B | | | | cell surface glycoprotein gp138 | | | |
| DDB_G0283393 | | | | rplP2 | | | | 60S acidic ribosomal protein P2, ribosomal acidic phosphoprotein P2 | | | |
| DDB_G0286985 | | | | zipA | | | | zipper-like domain-containing protein | | | |
| kif6 | | | | kif6 | | | | belongs to the MCAK%2FKif2 subfamily%2C predicted to play a role in mitosis | | | |
| DDB_G0269110 | | | | tspA | | | | tetraspanin family protein | | | |
| DDB_G0289659 | | | | rrpA | | | | RNA-directed RNA polymerase | | | |
| DDB_G0269212 | | | | abcG11 | | | | ABC transporter G family protein | | | |
| DDB_G0269116 | | | | cmbC | | | | putative calmodulin-binding protein CaM-BP15 | | | |
| DDB_G0267466 | | | | cupA | | | | calcium up-regulated protein, ricin B lectin domain-containing protein | | | |
| DDB_G0287587 | | | | smlA | | | | unknown | | | |
| DDB_G0283387 | | | | abcA10 | | | | ABC transporter A family protein | | | |
| DDB_G0290265 | | | | GP138D | | | | DdFRP1alpha | | | |
| DDB_G0291213 | | | | DDB_G0291213 | | | | LTR-RETROTRANSPOSON SKIPPER, GAG (Fragment). | | | |
| DDB_G0291608 | | | | DDB_G0291608 | | | |  | | | |
| DDB_G0291610 | | | | DDB_G0291610 | | | |  | | | |
| DDB_G0291620 | | | | DDB_G0291620 | | | | Pol. | | | |
| DDB_G0291930 | | | | DDB_G0291930 | | | | Similar to plasmodium falciparum (Isolate 3D7). hypothetical 98.3 kDa protein. | | | |
| DDB_G0291934 | | | | DDB_G0291934 | | | |  | | | |
| DDB_G0292154 | | | | DDB_G0292154 | | | |  | | | |
| DDB_G0292162 | | | | DDB_G0292162 | | | |  | | | |
| DDB_G0292854 | | | | DDB_G0292854 | | | |  | | | |
| DDB_G0292856 | | | | DDB_G0292856 | | | |  | | | |
| DDB_G0292890 | | | | DDB_G0292890 | | | |  | | | |
| DDB_G0292900 | | | | DDB_G0292900 | | | |  | | | |
| DDB_G0292926 | | | | DDB_G0292926 | | | |  | | | |
| DDB_G0292934 | | | | DDB_G0292934 | | | |  | | | |
| DDB_G0292936 | | | | DDB_G0292936 | | | |  | | | |
| DDB_G0293016 | | | | DDB_G0293016 | | | |  | | | |
| DDB_G0293040 | | | | DDB_G0293040 | | | |  | | | |
| DDB_G0293060 | | | | DDB_G0293060 | | | | Possible blackjack. | | | |
| DDB_G0293240 | | | | DDB_G0293240 | | | |  | | | |
| DDB_G0293242 | | | | DDB_G0293242 | | | |  | | | |
| DDB_G0293302 | | | | DDB_G0293302 | | | |  | | | |
| DDB_G0293334 | | | | DDB_G0293334 | | | | Similar to Plasmodium falciparum (Isolate 3D7). Hypothetical 111.5 kDa protein. | | | |
| DDB_G0293346 | | | | DDB_G0293346 | | | |  | | | |
| DDB_G0293350 | | | | DDB_G0293350 | | | | Pol. | | | |
| DDB_G0293368 | | | | DDB_G0293368 | | | |  | | | |
| DDB_G0293406 | | | | DDB_G0293406 | | | |  | | | |
| DDB_G0293592 | | | | DDB_G0293592 | | | |  | | | |
| DDB_G0293594 | | | | DDB_G0293594 | | | |  | | | |
| DDB_G0293598 | | | | DDB_G0293598 | | | |  | | | |
| DDB_G0293602 | | | | DDB_G0293602 | | | |  | | | |
| DDB_G0293612 | | | | DDB_G0293612 | | | |  | | | |
| DDB_G0293630 | | | | DDB_G0293630 | | | | Pol. | | | |
| DDB_G0293662 | | | | DDB_G0293662 | | | |  | | | |
| DDB_G0293664 | | | | DDB_G0293664 | | | | Similar to Dictyostelium discoideum (Slime mold). Hypothetical 127.0 kDa protein. | | | |
| DDB_G0293668 | | | | DDB_G0293668 | | | |  | | | |
| DDB_G0293670 | | | | DDB_G0293670 | | | |  | | | |
| DDB_G0293672 | | | | DDB_G0293672 | | | | BiP (Fragment). | | | |
| DDB_G0293674 | | | | DDB_G0293674 | | | | Hsc70 protein. | | | |
| DDB_G0293678 | | | | DDB_G0293678 | | | | P36415 Heat shock cognate protein (Aginactin). | | | |
| DDB_G0293744 | | | | DDB_G0293744 | | | |  | | | |
| DDB_G0293760 | | | | DDB_G0293760 | | | |  | | | |
| DDB_G0293764 | | | | DDB_G0293764 | | | | Multifunctional protein (Fragment). | | | |
| DDB_G0293774 | | | | DDB_G0293774 | | | |  | | | |
| DDB_G0293804 | | | | DDB_G0293804 | | | |  | | | |
| DDB_G0293910 | | | | DDB_G0293910 | | | |  | | | |
| DDB_G0293926 | | | | DDB_G0293926 | | | |  | | | |
| DDB_G0293948 | | | | DDB_G0293948 | | | |  | | | |
| DDB_G0293954 | | | | DDB_G0293954 | | | |  | | | |
| DDB_G0291940 | | | | DDB_G0291940 | | | | Pol. | | | |
| DDB_G0293996 | | | | DDB_G0293996 | | | |  | | | |
| DDB_G0293998 | | | | DDB_G0293998 | | | |  | | | |
| abpE-1 | | | | abpE-1 | | | | involved in pseudopod formation%3B contains an N-terminal ADF%2Fcofilin-like actin binding domain and a c-terminal SH3 domain%3B there is a second copy of this gene%2C %3Ca href%3D%22%2Fdb%2Fcgi-bin%2Fgene_page.pl?primary_id%3DDDB_G0273517%22%3E%3Ci%3EabpE-2%3C%2Fi%3E%3C%2Fa%3E | | | |
| DDB_G0294423 | | | | ai2b | | | | homing endonuclease | | | |
| DDB_G0294012 | | | | atp1 | | | | ATP synthase F1 alpha, ATPase subunit 1 | | | |
| DDB_G0294036 | | | | mrpl2 | | | | ribosomal protein L2, mitochondrial | | | |
| DDB_G0292562 | | | | lagC3 | | | | LagC-like protein | | | |
| DDB_G0291245 | | | | abcA6 | | | | ABC transporter A family protein | | | |
| DDB_G0291243 | | | | abcC7 | | | | ABC transporter C family protein | | | |
| DDB_G0284185 | | | | syn6 | | | | putative syntaxin 6, putative t-SNARE family protein | | | |
| DDB_G0290261 | | | | GP138C | | | | cell surface glycoprotein gp138 | | | |
| DDB_G0290479 | | | | hydA | | | | aldehyde dehydrogenase, putative NAD-dependent aldehyde dehydrogenase | | | |
| DDB_G0289449 | | | | ubqF | | | | ubiquitin | | | |
| DDB_G0291856 | | | | gerD | | | | germination protein (270G) | | | |
| DDB_G0291982 | | | | psiA | | | | PA14 domain-containing protein, prespore-cell-inducing factor, psi factor | | | |
| DDB_G0291233 | | | | rabA | | | | Rab GTPase | | | |
| DDB_G0270458 | | | | DDB_G0270458 | | | | Pol. | | | |
| DDB_G0270460 | | | | DDB_G0270460 | | | | Pol. | | | |
| DDB_G0270464 | | | | DDB_G0270464 | | | | 5'-B-MODULE (Fragment). | | | |
| DDB_G0270476 | | | | DDB_G0270476 | | | |  | | | |
| DDB_G0270506 | | | | DDB_G0270506 | | | |  | | | |
| DDB_G0270514 | | | | DDB_G0270514 | | | |  | | | |
| DDB_G0270522 | | | | DDB_G0270522 | | | |  | | | |
| DDB_G0270524 | | | | DDB_G0270524 | | | |  | | | |
| DDB_G0270540 | | | | DDB_G0270540 | | | |  | | | |
| DDB_G0270564 | | | | DDB_G0270564 | | | |  | | | |
| DDB_G0270570 | | | | DDB_G0270570 | | | |  | | | |
| DDB_G0270574 | | | | DDB_G0270574 | | | |  | | | |
| DDB_G0270586 | | | | DDB_G0270586 | | | | Similar to Rhizobium meliloti (Sinorhizobium meliloti). putative transport transmembrane protein. | | | |
| DDB_G0270596 | | | | DDB_G0270596 | | | |  | | | |
| DDB_G0270628 | | | | DDB_G0270628 | | | |  | | | |
| DDB_G0270642 | | | | DDB_G0270642 | | | |  | | | |
| DDB_G0270644 | | | | DDB_G0270644 | | | |  | | | |
| DDB_G0270646 | | | | DDB_G0270646 | | | | Kinesin-like protein, putative. | | | |
| DDB_G0270648 | | | | DDB_G0270648 | | | | Pol. | | | |
| DDB_G0270650 | | | | DDB_G0270650 | | | | Pol. | | | |
| DDB_G0270700 | | | | DDB_G0270700 | | | | Similar to Dictyostelium discoideum (Slime mold). hypothetical 127.0 kDa protein. | | | |
| DDB_G0270702 | | | | DDB_G0270702 | | | |  | | | |
| DDB_G0270704 | | | | DDB_G0270704 | | | | Similar to Mus musculus (Mouse). 12 days embryo spinal ganglion cDNA, RIKEN full-length enriched library, clone:D130061K05 product:MEGF11 PROTEIN (KIAA1781) homolog. | | | |
| DDB_G0270706 | | | | DDB_G0270706 | | | |  | | | |
| DDB_G0270724 | | | | DDB_G0270724 | | | |  | | | |
| DDB_G0270728 | | | | DDB_G0270728 | | | |  | | | |
| DDB_G0270732 | | | | DDB_G0270732 | | | |  | | | |
| DDB_G0270770 | | | | DDB_G0270770 | | | | Multifunctional protein (Fragment). | | | |
| DDB_G0270792 | | | | DDB_G0270792 | | | | Pol. | | | |
| DDB_G0270796 | | | | DDB_G0270796 | | | | Putative calmodulin-binding protein CaM-BP15. | | | |
| DDB_G0270802 | | | | DDB_G0270802 | | | | Similar to Homo sapiens (Human). Tenascin (TN) (Hexabrachion) (Cytotactin) (Neuronectin) (GMEM) (JI) (Miotendinous antigen) (Glioma-associated-extracellular matrix antigen) (GP 150-225) (Tenascin-C) (TN-C). | | | |
| DDB_G0270810 | | | | DDB_G0270810 | | | |  | | | |
| DDB_G0268284 | | | | DDB_G0268284 | | | |  | | | |
| DDB_G0268292 | | | | DDB_G0268292 | | | |  | | | |
| DDB_G0268304 | | | | DDB_G0268304 | | | |  | | | |
| DDB_G0268326 | | | | DDB_G0268326 | | | |  | | | |
| DDB_G0268340 | | | | DDB_G0268340 | | | | Multifunctional protein (Fragment). | | | |
| DDB_G0268342 | | | | DDB_G0268342 | | | |  | | | |
| DDB_G0268348 | | | | DDB_G0268348 | | | |  | | | |
| DDB_G0268352 | | | | DDB_G0268352 | | | |  | | | |
| DDB_G0268362 | | | | DDB_G0268362 | | | |  | | | |
| DDB_G0268386 | | | | DDB_G0268386 | | | |  | | | |
| DDB_G0268388 | | | | DDB_G0268388 | | | |  | | | |
| DDB_G0268390 | | | | DDB_G0268390 | | | |  | | | |
| DDB_G0268406 | | | | DDB_G0268406 | | | |  | | | |
| DDB_G0268412 | | | | DDB_G0268412 | | | |  | | | |
| DDB_G0268420 | | | | DDB_G0268420 | | | |  | | | |
| DDB_G0268428 | | | | DDB_G0268428 | | | |  | | | |
| DDB_G0268430 | | | | DDB_G0268430 | | | | P07271 DNA repair and recombination protein PIF1, mitochondrial precursor. | | | |
| DDB_G0268432 | | | | DDB_G0268432 | | | |  | | | |
| DDB_G0268438 | | | | DDB_G0268438 | | | |  | | | |
| DDB_G0268440 | | | | DDB_G0268440 | | | | Pol. | | | |
| DDB_G0268464 | | | | DDB_G0268464 | | | | Pol. | | | |
| DDB_G0268918 | | | | DDB_G0268918 | | | |  | | | |
| DDB_G0268924 | | | | DDB_G0268924 | | | |  | | | |
| DDB_G0268930 | | | | DDB_G0268930 | | | |  | | | |
| DDB_G0268940 | | | | DDB_G0268940 | | | |  | | | |
| DDB_G0268944 | | | | DDB_G0268944 | | | | Pol. | | | |
| DDB_G0268960 | | | | DDB_G0268960 | | | |  | | | |
| DDB_G0268964 | | | | DDB_G0268964 | | | |  | | | |
| DDB_G0268978 | | | | DDB_G0268978 | | | | Pol. | | | |
| DDB_G0268980 | | | | DDB_G0268980 | | | |  | | | |
| DDB_G0268986 | | | | DDB_G0268986 | | | | Pol. | | | |
| DDB_G0268996 | | | | DDB_G0268996 | | | |  | | | |
| DDB_G0269010 | | | | DDB_G0269010 | | | |  | | | |
| DDB_G0269018 | | | | DDB_G0269018 | | | |  | | | |
| DDB_G0275715 | | | | DDB_G0275715 | | | |  | | | |
| DDB_G0275721 | | | | DDB_G0275721 | | | |  | | | |
| DDB_G0275727 | | | | DDB_G0275727 | | | | Q94481 Protein cigB (Fragment). | | | |
| DDB_G0275785 | | | | DDB_G0275785 | | | |  | | | |
| DDB_G0275789 | | | | DDB_G0275789 | | | | Similar to Dictyostelium discoideum (Slime mold). Hypothetical 127.0 kDa protein. | | | |
| DDB_G0275799 | | | | DDB_G0275799 | | | |  | | | |
| DDB_G0275803 | | | | DDB_G0275803 | | | | Similar to Dictyostelium discoideum (Slime mold). hypothetical 97.7 kDa protein. | | | |
| DDB_G0275347 | | | | DDB_G0275347 | | | |  | | | |
| DDB_G0275361 | | | | DDB_G0275361 | | | |  | | | |
| DDB_G0275365 | | | | DDB_G0275365 | | | |  | | | |
| DDB_G0275389 | | | | DDB_G0275389 | | | |  | | | |
| DDB_G0276979 | | | | DDB_G0276979 | | | | Pol. | | | |
| DDB_G0276981 | | | | DDB_G0276981 | | | | Pol. | | | |
| DDB_G0276983 | | | | DDB_G0276983 | | | | Multifunctional protein (Fragment). | | | |
| DDB_G0276985 | | | | DDB_G0276985 | | | | Similar to Arabidopsis thaliana (Mouse-ear cress). Putative GTP-binding protein CGPA. | | | |
| DDB_G0276991 | | | | DDB_G0276991 | | | |  | | | |
| DDB_G0276995 | | | | DDB_G0276995 | | | |  | | | |
| DDB_G0277005 | | | | DDB_G0277005 | | | | Q94481 Protein cigB (Fragment). | | | |
| DDB_G0271150 | | | | DDB_G0271150 | | | |  | | | |
| DDB_G0271152 | | | | DDB_G0271152 | | | |  | | | |
| DDB_G0271164 | | | | DDB_G0271164 | | | |  | | | |
| DDB_G0271166 | | | | DDB_G0271166 | | | |  | | | |
| DDB_G0271176 | | | | DDB_G0271176 | | | |  | | | |
| DDB_G0271186 | | | | DDB_G0271186 | | | |  | | | |
| DDB_G0271188 | | | | DDB_G0271188 | | | |  | | | |
| DDB_G0271200 | | | | DDB_G0271200 | | | |  | | | |
| DDB_G0271214 | | | | DDB_G0271214 | | | |  | | | |
| DDB_G0271216 | | | | DDB_G0271216 | | | |  | | | |
| DDB_G0271218 | | | | DDB_G0271218 | | | |  | | | |
| DDB_G0271228 | | | | DDB_G0271228 | | | |  | | | |
| DDB_G0271232 | | | | DDB_G0271232 | | | | Similar to Dictyostelium discoideum (Slime mold). Prestalk protein. | | | |
| DDB_G0271234 | | | | DDB_G0271234 | | | |  | | | |
| DDB_G0271244 | | | | DDB_G0271244 | | | |  | | | |
| DDB_G0271262 | | | | DDB_G0271262 | | | |  | | | |
| DDB_G0271274 | | | | DDB_G0271274 | | | |  | | | |
| DDB_G0271320 | | | | DDB_G0271320 | | | |  | | | |
| DDB_G0271322 | | | | DDB_G0271322 | | | |  | | | |
| DDB_G0271324 | | | | DDB_G0271324 | | | |  | | | |
| DDB_G0271330 | | | | DDB_G0271330 | | | |  | | | |
| DDB_G0276729 | | | | DDB_G0276729 | | | |  | | | |
| DDB_G0276731 | | | | DDB_G0276731 | | | |  | | | |
| DDB_G0276733 | | | | DDB_G0276733 | | | |  | | | |
| DDB_G0276735 | | | | DDB_G0276735 | | | | Similar to K08H10.2a.p. | | | |
| DDB_G0276739 | | | | DDB_G0276739 | | | |  | | | |
| DDB_G0276745 | | | | DDB_G0276745 | | | |  | | | |
| DDB_G0276661 | | | | DDB_G0276661 | | | | P10901 Alpha-L-fucosidase precursor (EC 3.2.1.51) (Alpha-L-fucoside fucohydrolase). | | | |
| DDB_G0276681 | | | | DDB_G0276681 | | | |  | | | |
| DDB_G0276691 | | | | DDB_G0276691 | | | |  | | | |
| DDB_G0276697 | | | | DDB_G0276697 | | | |  | | | |
| DDB_G0276705 | | | | DDB_G0276705 | | | | Similar to K08H10.2a.p. | | | |
| DDB_G0272873 | | | | DDB_G0272873 | | | |  | | | |
| DDB_G0272877 | | | | DDB_G0272877 | | | |  | | | |
| DDB_G0272891 | | | | DDB_G0272891 | | | |  | | | |
| DDB_G0272895 | | | | DDB_G0272895 | | | |  | | | |
| DDB_G0273263 | | | | DDB_G0273263 | | | | Pol. | | | |
| DDB_G0273273 | | | | DDB_G0273273 | | | |  | | | |
| DDB_G0273275 | | | | DDB_G0273275 | | | | Similar to Dictyostelium discoideum (Slime mold). putative calmodulin-binding protein CaM-BP46. | | | |
| DDB_G0273277 | | | | DDB_G0273277 | | | | Similar to delayed anaerobic Gene; Dan4p. | | | |
| DDB_G0273279 | | | | DDB_G0273279 | | | |  | | | |
| DDB_G0273281 | | | | DDB_G0273281 | | | | Similar to Dictyostelium discoideum (Slime mold). hypothetical 97.7 kDa protein. | | | |
| DDB_G0273285 | | | | DDB_G0273285 | | | | Similar to Dictyostelium discoideum (Slime mold). prespore-specific protein. | | | |
| DDB_G0273287 | | | | DDB_G0273287 | | | | Similar to Dictyostelium discoideum (Slime mold). prespore-specific protein. | | | |
| DDB_G0273297 | | | | DDB_G0273297 | | | |  | | | |
| DDB_G0273307 | | | | DDB_G0273307 | | | |  | | | |
| DDB_G0275221 | | | | DDB_G0275221 | | | | Pol. | | | |
| DDB_G0274631 | | | | DDB_G0274631 | | | | Similar to Dictyostelium discoideum (Slime mold). hypothetical 97.7 kDa protein. | | | |
| DDB_G0274635 | | | | DDB_G0274635 | | | | Similar to Dictyostelium discoideum (Slime mold). hypothetical 97.7 kDa protein. | | | |
| DDB_G0274637 | | | | DDB_G0274637 | | | | Similar to Dictyostelium discoideum (Slime mold). hypothetical 97.7 kDa protein. | | | |
| DDB_G0274653 | | | | DDB_G0274653 | | | |  | | | |
| DDB_G0274655 | | | | DDB_G0274655 | | | |  | | | |
| DDB_G0274669 | | | | DDB_G0274669 | | | | Similar to GNS1/SUR4 family protein. | | | |
| DDB_G0274673 | | | | DDB_G0274673 | | | | Pol. | | | |
| DDB_G0274677 | | | | DDB_G0274677 | | | |  | | | |
| DDB_G0274679 | | | | DDB_G0274679 | | | |  | | | |
| DDB_G0274685 | | | | DDB_G0274685 | | | |  | | | |
| DDB_G0274719 | | | | DDB_G0274719 | | | | Pol. | | | |
| DDB_G0274747 | | | | DDB_G0274747 | | | |  | | | |
| DDB_G0274753 | | | | DDB_G0274753 | | | | Clone 9.10 TDD-3 and red repetitive elements, partial sequence. | | | |
| DDB_G0273315 | | | | DDB_G0273315 | | | |  | | | |
| DDB_G0273319 | | | | DDB_G0273319 | | | |  | | | |
| DDB_G0273459 | | | | DDB_G0273459 | | | |  | | | |
| DDB_G0273463 | | | | DDB_G0273463 | | | | Similar to Dictyostelium discoideum (Slime mold). prespore-specific protein. | | | |
| DDB_G0277521 | | | | DDB_G0277521 | | | | Pol. | | | |
| DDB_G0277619 | | | | DDB_G0277619 | | | | Similar to G-protein-coupled receptor at plasma membrane; interactions in two-hybrid system with Gpa2p; Gpr1p. | | | |
| DDB_G0277623 | | | | DDB_G0277623 | | | | Similar to G-protein-coupled receptor at plasma membrane; interactions in two-hybrid system with Gpa2p; Gpr1p. | | | |
| DDB_G0277627 | | | | DDB_G0277627 | | | |  | | | |
| DDB_G0276303 | | | | DDB_G0276303 | | | |  | | | |
| DDB_G0276307 | | | | DDB_G0276307 | | | | Similar to Plasmodium falciparum (Isolate 3D7). Asparagine-rich antigen. | | | |
| DDB_G0276309 | | | | DDB_G0276309 | | | | Similar to Gallus gallus (Chicken). 190 kDa tenascin. | | | |
| DDB_G0276171 | | | | DDB_G0276171 | | | | Pol. | | | |
| DDB_G0276197 | | | | DDB_G0276197 | | | | Pol. | | | |
| DDB_G0276203 | | | | DDB_G0276203 | | | | Similar to Dictyostelium discoideum (Slime mold). Phosphatidylinositol 3-kinase 3 (EC 2.7.1.137) (PI3-kinase) (PtdIns-3-kinase) (PI3K). | | | |
| DDB_G0276207 | | | | DDB_G0276207 | | | |  | | | |
| DDB_G0276211 | | | | DDB_G0276211 | | | | Pol. | | | |
| DDB_G0276225 | | | | DDB_G0276225 | | | |  | | | |
| DDB_G0275961 | | | | DDB_G0275961 | | | |  | | | |
| DDB_G0275963 | | | | DDB_G0275963 | | | |  | | | |
| DDB_G0275991 | | | | DDB_G0275991 | | | |  | | | |
| DDB_G0271568 | | | | DDB_G0271568 | | | | Multifunctional protein (Fragment). | | | |
| DDB_G0271576 | | | | DDB_G0271576 | | | | Pol. | | | |
| DDB_G0271592 | | | | DDB_G0271592 | | | |  | | | |
| DDB_G0271746 | | | | DDB_G0271746 | | | |  | | | |
| DDB_G0271748 | | | | DDB_G0271748 | | | | Similar to Lactococcus lactis (Subsp. lactis) (Streptococcus lactis). Oxidoreductase. | | | |
| DDB_G0271758 | | | | DDB_G0271758 | | | |  | | | |
| DDB_G0272088 | | | | DDB_G0272088 | | | |  | | | |
| DDB_G0272268 | | | | DDB_G0272268 | | | | Clone 9.10 TDD-3 and red repetitive elements, partial sequence. | | | |
| DDB_G0272270 | | | | DDB_G0272270 | | | | Pol. | | | |
| DDB_G0272278 | | | | DDB_G0272278 | | | | Similar to Dictyostelium discoideum (Slime mold). Hypothetical 97.7 kDa protein. | | | |
| DDB_G0272288 | | | | DDB_G0272288 | | | | Similar to Drosophila melanogaster (Fruit fly). RE12057p. | | | |
| DDB_G0272290 | | | | DDB_G0272290 | | | | Multifunctional protein (Fragment). | | | |
| DDB_G0272294 | | | | DDB_G0272294 | | | | Similar to Arabidopsis thaliana (Mouse-ear cress). At1g10390/F14N23_29. | | | |
| DDB_G0272298 | | | | DDB_G0272298 | | | | Similar to Sarcophaga peregrina (Flesh fly) (Boettcherisca peregrina). cathepsin L (EC 3.4.22.15). | | | |
| DDB_G0271876 | | | | DDB_G0271876 | | | | Similar to Dictyostelium discoideum (Slime mold). CIGB protein. | | | |
| DDB_G0272528 | | | | DDB_G0272528 | | | | Similar to Plasmodium falciparum. FHA domain protein, putative. | | | |
| DDB_G0272911 | | | | DDB_G0272911 | | | |  | | | |
| DDB_G0272915 | | | | DDB_G0272915 | | | |  | | | |
| DDB_G0272917 | | | | DDB_G0272917 | | | |  | | | |
| DDB_G0276373 | | | | DDB_G0276373 | | | |  | | | |
| DDB_G0276487 | | | | DDB_G0276487 | | | |  | | | |
| DDB_G0276515 | | | | DDB_G0276515 | | | |  | | | |
| DDB_G0276517 | | | | DDB_G0276517 | | | |  | | | |
| DDB_G0281037 | | | | DDB_G0281037 | | | |  | | | |
| DDB_G0281079 | | | | DDB_G0281079 | | | |  | | | |
| DDB_G0281083 | | | | DDB_G0281083 | | | |  | | | |
| DDB_G0281115 | | | | DDB_G0281115 | | | | Pirin. | | | |
| DDB_G0281117 | | | | DDB_G0281117 | | | |  | | | |
| DDB_G0281119 | | | | DDB_G0281119 | | | | Pirin. | | | |
| DDB_G0281121 | | | | DDB_G0281121 | | | |  | | | |
| DDB_G0281123 | | | | DDB_G0281123 | | | |  | | | |
| DDB_G0281125 | | | | DDB_G0281125 | | | |  | | | |
| DDB_G0281131 | | | | DDB_G0281131 | | | |  | | | |
| DDB_G0281133 | | | | DDB_G0281133 | | | | Beta-eliminating lyase. | | | |
| DDB_G0281135 | | | | DDB_G0281135 | | | |  | | | |
| DDB_G0281141 | | | | DDB_G0281141 | | | |  | | | |
| DDB_G0281147 | | | | DDB_G0281147 | | | |  | | | |
| DDB_G0281149 | | | | DDB_G0281149 | | | |  | | | |
| DDB_G0281157 | | | | DDB_G0281157 | | | |  | | | |
| DDB_G0281203 | | | | DDB_G0281203 | | | | Similar to Dictyostelium discoideum (Slime mold). hypothetical 127.0 kDa protein. | | | |
| DDB_G0281207 | | | | DDB_G0281207 | | | | Similar to Dictyostelium discoideum (Slime mold). hypothetical 127.0 kDa protein. | | | |
| DDB_G0281231 | | | | DDB_G0281231 | | | | Similar to Homo sapiens (Human). Tenascin (TN) (Hexabrachion) (Cytotactin) (Neuronectin) (GMEM) (JI) (Miotendinous antigen) (Glioma-associated-extracellular matrix antigen) (GP 150-225) (Tenascin-C) (TN-C). | | | |
| DDB_G0281249 | | | | DDB_G0281249 | | | |  | | | |
| DDB_G0281257 | | | | DDB_G0281257 | | | |  | | | |
| DDB_G0281267 | | | | DDB_G0281267 | | | |  | | | |
| DDB_G0281285 | | | | DDB_G0281285 | | | |  | | | |
| DDB_G0281287 | | | | DDB_G0281287 | | | |  | | | |
| DDB_G0281349 | | | | DDB_G0281349 | | | |  | | | |
| DDB_G0281355 | | | | DDB_G0281355 | | | | Pol. | | | |
| DDB_G0281359 | | | | DDB_G0281359 | | | | Similar to Aquifex aeolicus. methionyl-tRNA synthetase beta subunit. | | | |
| DDB_G0282385 | | | | DDB_G0282385 | | | |  | | | |
| DDB_G0282405 | | | | DDB_G0282405 | | | | Group-specific antigen. | | | |
| DDB_G0281851 | | | | DDB_G0281851 | | | |  | | | |
| DDB_G0281905 | | | | DDB_G0281905 | | | |  | | | |
| DDB_G0281927 | | | | DDB_G0281927 | | | |  | | | |
| DDB_G0277937 | | | | DDB_G0277937 | | | |  | | | |
| DDB_G0277961 | | | | DDB_G0277961 | | | |  | | | |
| DDB_G0277963 | | | | DDB_G0277963 | | | |  | | | |
| DDB_G0277965 | | | | DDB_G0277965 | | | |  | | | |
| DDB_G0278001 | | | | DDB_G0278001 | | | |  | | | |
| DDB_G0278035 | | | | DDB_G0278035 | | | | P10819 Adenosylhomocysteinase (EC 3.3.1.1) (S-adenosyl-L-homocysteine hydrolase) (AdoHcyase). | | | |
| DDB_G0278037 | | | | DDB_G0278037 | | | | P10819 Adenosylhomocysteinase (EC 3.3.1.1) (S-adenosyl-L-homocysteine hydrolase) (AdoHcyase). | | | |
| DDB_G0278067 | | | | DDB_G0278067 | | | |  | | | |
| DDB_G0278071 | | | | DDB_G0278071 | | | |  | | | |
| DDB_G0278093 | | | | DDB_G0278093 | | | |  | | | |
| DDB_G0278103 | | | | DDB_G0278103 | | | |  | | | |
| DDB_G0278131 | | | | DDB_G0278131 | | | |  | | | |
| DDB_G0278135 | | | | DDB_G0278135 | | | |  | | | |
| DDB_G0278137 | | | | DDB_G0278137 | | | |  | | | |
| DDB_G0278139 | | | | DDB_G0278139 | | | |  | | | |
| DDB_G0278145 | | | | DDB_G0278145 | | | |  | | | |
| DDB_G0278183 | | | | DDB_G0278183 | | | |  | | | |
| DDB_G0278209 | | | | DDB_G0278209 | | | |  | | | |
| DDB_G0278211 | | | | DDB_G0278211 | | | |  | | | |
| DDB_G0278219 | | | | DDB_G0278219 | | | |  | | | |
| DDB_G0281579 | | | | DDB_G0281579 | | | | Polyprotein (Fragment). | | | |
| DDB_G0281581 | | | | DDB_G0281581 | | | | 78 kDa glucose-regulated protein homolog. | | | |
| DDB_G0281629 | | | | DDB_G0281629 | | | | Similar to Dictyostelium discoideum (Slime mold). histidine kinase DhkE. | | | |
| DDB_G0281631 | | | | DDB_G0281631 | | | |  | | | |
| DDB_G0281633 | | | | DDB_G0281633 | | | |  | | | |
| DDB_G0281635 | | | | DDB_G0281635 | | | |  | | | |
| DDB_G0281645 | | | | DDB_G0281645 | | | |  | | | |
| DDB_G0281665 | | | | DDB_G0281665 | | | |  | | | |
| DDB_G0281675 | | | | DDB_G0281675 | | | |  | | | |
| DDB_G0281737 | | | | DDB_G0281737 | | | |  | | | |
| DDB_G0281783 | | | | DDB_G0281783 | | | |  | | | |
| DDB_G0279135 | | | | DDB_G0279135 | | | |  | | | |
| DDB_G0280779 | | | | DDB_G0280779 | | | |  | | | |
| DDB_G0280781 | | | | DDB_G0280781 | | | |  | | | |
| DDB_G0280789 | | | | DDB_G0280789 | | | |  | | | |
| DDB_G0280825 | | | | DDB_G0280825 | | | | Acetyltransferase, CysE/LacA/LpxA/NodL family. | | | |
| DDB_G0282435 | | | | DDB_G0282435 | | | |  | | | |
| DDB_G0282437 | | | | DDB_G0282437 | | | |  | | | |
| DDB_G0282439 | | | | DDB_G0282439 | | | |  | | | |
| DDB_G0282441 | | | | DDB_G0282441 | | | |  | | | |
| DDB_G0282443 | | | | DDB_G0282443 | | | |  | | | |
| DDB_G0282445 | | | | DDB_G0282445 | | | |  | | | |
| DDB_G0282487 | | | | DDB_G0282487 | | | |  | | | |
| DDB_G0282495 | | | | DDB_G0282495 | | | |  | | | |
| DDB_G0282501 | | | | DDB_G0282501 | | | |  | | | |
| DDB_G0282503 | | | | DDB_G0282503 | | | |  | | | |
| DDB_G0282511 | | | | DDB_G0282511 | | | |  | | | |
| DDB_G0282513 | | | | DDB_G0282513 | | | |  | | | |
| DDB_G0282535 | | | | DDB_G0282535 | | | | Similar to Dictyostelium discoideum (Slime mold). histidine kinase A. | | | |
| DDB_G0282543 | | | | DDB_G0282543 | | | | Group-specific antigen. | | | |
| DDB_G0282545 | | | | DDB_G0282545 | | | | Gag. | | | |
| DDB_G0282547 | | | | DDB_G0282547 | | | |  | | | |
| DDB_G0282555 | | | | DDB_G0282555 | | | |  | | | |
| DDB_G0282557 | | | | DDB_G0282557 | | | | Similar to Dictyostelium discoideum (Slime mold). Inositol 5-phosphatase 2. | | | |
| DDB_G0282559 | | | | DDB_G0282559 | | | |  | | | |
| DDB_G0282565 | | | | DDB_G0282565 | | | |  | | | |
| DDB_G0282567 | | | | DDB_G0282567 | | | |  | | | |
| DDB_G0282577 | | | | DDB_G0282577 | | | |  | | | |
| DDB_G0282619 | | | | DDB_G0282619 | | | |  | | | |
| DDB_G0282639 | | | | DDB_G0282639 | | | | Similar to Dictyostelium discoideum (Slime mold). CIGB protein. | | | |
| DDB_G0282659 | | | | DDB_G0282659 | | | | Pol. | | | |
| DDB_G0282661 | | | | DDB_G0282661 | | | |  | | | |
| DDB_G0282665 | | | | DDB_G0282665 | | | |  | | | |
| DDB_G0282681 | | | | DDB_G0282681 | | | |  | | | |
| DDB_G0282699 | | | | DDB_G0282699 | | | |  | | | |
| DDB_G0282701 | | | | DDB_G0282701 | | | |  | | | |
| DDB_G0282707 | | | | DDB_G0282707 | | | | Similar to Dictyostelium discoideum (Slime mold). hypothetical 127.0 kDa protein. | | | |
| DDB_G0282709 | | | | DDB_G0282709 | | | | Similar to Dictyostelium discoideum (Slime mold). Hypothetical 127.0 kDa protein. | | | |
| DDB_G0282719 | | | | DDB_G0282719 | | | |  | | | |
| DDB_G0282737 | | | | DDB_G0282737 | | | | P54661 Small aggregate formation protein. | | | |
| DDB_G0282827 | | | | DDB_G0282827 | | | |  | | | |
| DDB_G0282829 | | | | DDB_G0282829 | | | |  | | | |
| DDB_G0282843 | | | | DDB_G0282843 | | | |  | | | |
| DDB_G0282857 | | | | DDB_G0282857 | | | |  | | | |
| DDB_G0282861 | | | | DDB_G0282861 | | | |  | | | |
| DDB_G0282885 | | | | DDB_G0282885 | | | | FLJ21908 protein. | | | |
| DDB_G0282893 | | | | DDB_G0282893 | | | |  | | | |
| DDB_G0279097 | | | | DDB_G0279097 | | | |  | | | |
| DDB_G0279121 | | | | DDB_G0279121 | | | |  | | | |
| DDB_G0281975 | | | | DDB_G0281975 | | | | Similar to Dictyostelium discoideum (Slime mold) serine/threonine protein phosphatase calcineurin A (EC 3.1.3.16). | | | |
| DDB_G0281993 | | | | DDB_G0281993 | | | |  | | | |
| DDB_G0281999 | | | | DDB_G0281999 | | | |  | | | |
| DDB_G0282001 | | | | DDB_G0282001 | | | |  | | | |
| DDB_G0282019 | | | | DDB_G0282019 | | | | Similar to Dictyostelium discoideum (Slime mold). CIGB protein. | | | |
| DDB_G0282065 | | | | DDB_G0282065 | | | |  | | | |
| DDB_G0282109 | | | | DDB_G0282109 | | | |  | | | |
| DDB_G0282111 | | | | DDB_G0282111 | | | |  | | | |
| DDB_G0282153 | | | | DDB_G0282153 | | | |  | | | |
| DDB_G0282163 | | | | DDB_G0282163 | | | |  | | | |
| DDB_G0282171 | | | | DDB_G0282171 | | | |  | | | |
| DDB_G0280409 | | | | DDB_G0280409 | | | |  | | | |
| DDB_G0280411 | | | | DDB_G0280411 | | | |  | | | |
| DDB_G0280425 | | | | DDB_G0280425 | | | | Similar to Dictyostelium discoideum (Slime mold). Non-receptor tyrosine kinase spore lysis A (EC 2.7.1.112) (Tyrosine-protein kinase 1). | | | |
| DDB_G0280429 | | | | DDB_G0280429 | | | | Similar to Boophilus microplus (Cattle tick). notch-like protein. | | | |
| DDB_G0280443 | | | | DDB_G0280443 | | | |  | | | |
| DDB_G0280509 | | | | DDB_G0280509 | | | |  | | | |
| DDB_G0282205 | | | | DDB_G0282205 | | | |  | | | |
| DDB_G0282225 | | | | DDB_G0282225 | | | |  | | | |
| DDB_G0282227 | | | | DDB_G0282227 | | | |  | | | |
| DDB_G0282241 | | | | DDB_G0282241 | | | | Q9ZPR1 Cell division control protein 48 homolog B (AtCDC48b). | | | |
| DDB_G0282251 | | | | DDB_G0282251 | | | |  | | | |
| DDB_G0278247 | | | | DDB_G0278247 | | | |  | | | |
| DDB_G0278251 | | | | DDB_G0278251 | | | |  | | | |
| DDB_G0278279 | | | | DDB_G0278279 | | | |  | | | |
| DDB_G0278291 | | | | DDB_G0278291 | | | |  | | | |
| DDB_G0278359 | | | | DDB_G0278359 | | | |  | | | |
| DDB_G0278385 | | | | DDB_G0278385 | | | |  | | | |
| DDB_G0278391 | | | | DDB_G0278391 | | | |  | | | |
| DDB_G0278393 | | | | DDB_G0278393 | | | |  | | | |
| DDB_G0278401 | | | | DDB_G0278401 | | | | Similar to Dictyostelium discoideum (Slime mold). cysteine proteinase 5 (EC 3.4.22.-). | | | |
| DDB_G0278405 | | | | DDB_G0278405 | | | |  | | | |
| DDB_G0278421 | | | | DDB_G0278421 | | | |  | | | |
| DDB_G0278423 | | | | DDB_G0278423 | | | |  | | | |
| DDB_G0278437 | | | | DDB_G0278437 | | | |  | | | |
| DDB_G0278439 | | | | DDB_G0278439 | | | |  | | | |
| DDB_G0278451 | | | | DDB_G0278451 | | | |  | | | |
| DDB_G0278453 | | | | DDB_G0278453 | | | |  | | | |
| DDB_G0278503 | | | | DDB_G0278503 | | | |  | | | |
| DDB_G0278519 | | | | DDB_G0278519 | | | |  | | | |
| DDB_G0278531 | | | | DDB_G0278531 | | | | Similar to Dictyostelium discoideum (Slime mold). hypothetical 127.0 kDa protein. | | | |
| DDB_G0278553 | | | | DDB_G0278553 | | | |  | | | |
| DDB_G0278555 | | | | DDB_G0278555 | | | |  | | | |
| DDB_G0278557 | | | | DDB_G0278557 | | | |  | | | |
| DDB_G0278559 | | | | DDB_G0278559 | | | |  | | | |
| DDB_G0278561 | | | | DDB_G0278561 | | | |  | | | |
| DDB_G0278565 | | | | DDB_G0278565 | | | |  | | | |
| DDB_G0281399 | | | | DDB_G0281399 | | | |  | | | |
| DDB_G0281421 | | | | DDB_G0281421 | | | |  | | | |
| DDB_G0281423 | | | | DDB_G0281423 | | | |  | | | |
| DDB_G0281455 | | | | DDB_G0281455 | | | | Pol. | | | |
| DDB_G0281481 | | | | DDB_G0281481 | | | |  | | | |
| DDB_G0281491 | | | | DDB_G0281491 | | | | Pol. | | | |
| DDB_G0279203 | | | | DDB_G0279203 | | | |  | | | |
| DDB_G0279219 | | | | DDB_G0279219 | | | |  | | | |
| DDB_G0279243 | | | | DDB_G0279243 | | | |  | | | |
| DDB_G0279325 | | | | DDB_G0279325 | | | |  | | | |
| DDB_G0279333 | | | | DDB_G0279333 | | | |  | | | |
| DDB_G0279335 | | | | DDB_G0279335 | | | |  | | | |
| DDB_G0279339 | | | | DDB_G0279339 | | | |  | | | |
| DDB_G0279355 | | | | DDB_G0279355 | | | |  | | | |
| DDB_G0279493 | | | | DDB_G0279493 | | | |  | | | |
| DDB_G0279541 | | | | DDB_G0279541 | | | |  | | | |
| DDB_G0279545 | | | | DDB_G0279545 | | | |  | | | |
| DDB_G0279575 | | | | DDB_G0279575 | | | |  | | | |
| DDB_G0279577 | | | | DDB_G0279577 | | | |  | | | |
| DDB_G0279929 | | | | DDB_G0279929 | | | | Pol. | | | |
| DDB_G0279931 | | | | DDB_G0279931 | | | | Polyprotein. | | | |
| DDB_G0279935 | | | | DDB_G0279935 | | | |  | | | |
| DDB_G0279953 | | | | DDB_G0279953 | | | |  | | | |
| DDB_G0279955 | | | | DDB_G0279955 | | | |  | | | |
| DDB_G0279957 | | | | DDB_G0279957 | | | |  | | | |
| DDB_G0280001 | | | | DDB_G0280001 | | | | Similar to Mus musculus (Mouse). tenascin C. | | | |
| DDB_G0280003 | | | | DDB_G0280003 | | | | Similar to Mus musculus (Mouse). tenascin C. | | | |
| DDB_G0280005 | | | | DDB_G0280005 | | | | Similar to Mus musculus (Mouse). tenascin C. | | | |
| DDB_G0279615 | | | | DDB_G0279615 | | | |  | | | |
| DDB_G0279617 | | | | DDB_G0279617 | | | |  | | | |
| DDB_G0279621 | | | | DDB_G0279621 | | | |  | | | |
| DDB_G0279623 | | | | DDB_G0279623 | | | |  | | | |
| DDB_G0279679 | | | | DDB_G0279679 | | | |  | | | |
| DDB_G0279691 | | | | DDB_G0279691 | | | | Multifunctional protein (Fragment). | | | |
| DDB_G0279705 | | | | DDB_G0279705 | | | |  | | | |
| DDB_G0279743 | | | | DDB_G0279743 | | | |  | | | |
| DDB_G0279745 | | | | DDB_G0279745 | | | |  | | | |
| DDB_G0279753 | | | | DDB_G0279753 | | | |  | | | |
| DDB_G0279757 | | | | DDB_G0279757 | | | |  | | | |
| DDB_G0279759 | | | | DDB_G0279759 | | | |  | | | |
| DDB_G0279767 | | | | DDB_G0279767 | | | |  | | | |
| DDB_G0279773 | | | | DDB_G0279773 | | | | Pol. | | | |
| DDB_G0279783 | | | | DDB_G0279783 | | | |  | | | |
| DDB_G0280603 | | | | DDB_G0280603 | | | |  | | | |
| DDB_G0280613 | | | | DDB_G0280613 | | | |  | | | |
| DDB_G0280619 | | | | DDB_G0280619 | | | |  | | | |
| DDB_G0280623 | | | | DDB_G0280623 | | | | P42523 Loose aggregate C protein precursor. | | | |
| DDB_G0280629 | | | | DDB_G0280629 | | | |  | | | |
| DDB_G0280635 | | | | DDB_G0280635 | | | |  | | | |
| DDB_G0280637 | | | | DDB_G0280637 | | | |  | | | |
| DDB_G0280647 | | | | DDB_G0280647 | | | |  | | | |
| DDB_G0280685 | | | | DDB_G0280685 | | | | Similar to Homo sapiens (Human). Piwi-like 1 (Drosophila). | | | |
| DDB_G0280707 | | | | DDB_G0280707 | | | |  | | | |
| DDB_G0280711 | | | | DDB_G0280711 | | | |  | | | |
| DDB_G0280713 | | | | DDB_G0280713 | | | |  | | | |
| DDB_G0280725 | | | | DDB_G0280725 | | | | Pirin. | | | |
| DDB_G0280727 | | | | DDB_G0280727 | | | |  | | | |
| DDB_G0280733 | | | | DDB_G0280733 | | | |  | | | |
| DDB_G0280737 | | | | DDB_G0280737 | | | |  | | | |
| DDB_G0278743 | | | | DDB_G0278743 | | | |  | | | |
| DDB_G0278745 | | | | DDB_G0278745 | | | |  | | | |
| DDB_G0278763 | | | | DDB_G0278763 | | | |  | | | |
| DDB_G0278777 | | | | DDB_G0278777 | | | |  | | | |
| DDB_G0278787 | | | | DDB_G0278787 | | | |  | | | |
| DDB_G0278823 | | | | DDB_G0278823 | | | | Farnesyl diphosphate synthase. | | | |
| DDB_G0278825 | | | | DDB_G0278825 | | | |  | | | |
| DDB_G0278861 | | | | DDB_G0278861 | | | |  | | | |
| DDB_G0278915 | | | | DDB_G0278915 | | | |  | | | |
| DDB_G0278919 | | | | DDB_G0278919 | | | | Similar to Dictyostelium discoideum (Slime mold). hypothetical 127.0 kDa protein. | | | |
| DDB_G0279817 | | | | DDB_G0279817 | | | |  | | | |
| DDB_G0279819 | | | | DDB_G0279819 | | | |  | | | |
| DDB_G0279833 | | | | DDB_G0279833 | | | |  | | | |
| DDB_G0279845 | | | | DDB_G0279845 | | | |  | | | |
| DDB_G0279847 | | | | expl4 | | | | expansin-like protein | | | |
| DDB_G0279871 | | | | DDB_G0279871 | | | |  | | | |
| DDB_G0279873 | | | | DDB_G0279873 | | | |  | | | |
| DDB_G0279877 | | | | DDB_G0279877 | | | |  | | | |
| DDB_G0279879 | | | | DDB_G0279879 | | | |  | | | |
| DDB_G0279881 | | | | DDB_G0279881 | | | |  | | | |
| DDB_G0279885 | | | | DDB_G0279885 | | | |  | | | |
| DDB_G0279887 | | | | DDB_G0279887 | | | |  | | | |
| DDB_G0279891 | | | | DDB_G0279891 | | | |  | | | |
| DDB_G0279893 | | | | DDB_G0279893 | | | |  | | | |
| DDB_G0280063 | | | | DDB_G0280063 | | | |  | | | |
| DDB_G0280075 | | | | DDB_G0280075 | | | |  | | | |
| DDB_G0280115 | | | | DDB_G0280115 | | | |  | | | |
| DDB_G0280149 | | | | DDB_G0280149 | | | |  | | | |
| DDB_G0280151 | | | | DDB_G0280151 | | | |  | | | |
| DDB_G0280155 | | | | DDB_G0280155 | | | |  | | | |
| DDB_G0280203 | | | | DDB_G0280203 | | | |  | | | |
| DDB_G0280223 | | | | DDB_G0280223 | | | |  | | | |
| DDB_G0280265 | | | | DDB_G0280265 | | | |  | | | |
| DDB_G0280333 | | | | DDB_G0280333 | | | |  | | | |
| DDB_G0280335 | | | | DDB_G0280335 | | | |  | | | |
| DDB_G0280339 | | | | DDB_G0280339 | | | | P22549 Cyclic nucleotide phosphodiesterase inhibitor precursor (PDI). | | | |
| DDB_G0272330 | | | | DDB_G0272330 | | | | Similar to Mus musculus (Mouse). 12 days embryo spinal ganglion cDNA, RIKEN full-length enriched library, clone:D130061K05 product:MEGF11 PROTEIN (KIAA1781) homolog. | | | |
| DDB_G0272334 | | | | DDB_G0272334 | | | | Similar to Mus musculus (Mouse). 12 days embryo spinal ganglion cDNA, RIKEN full-length enriched library, clone:D130061K05 product:MEGF11 PROTEIN (KIAA1781) homolog. | | | |
| DDB_G0272336 | | | | DDB_G0272336 | | | | Similar to Mus musculus (Mouse). 12 days embryo spinal ganglion cDNA, RIKEN full-length enriched library, clone:D130061K05 product:MEGF11 PROTEIN (KIAA1781) homolog. | | | |
| DDB_G0272340 | | | | DDB_G0272340 | | | | Similar to Dictyostelium discoideum (Slime mold). DG17 protein. | | | |
| DDB_G0272348 | | | | DDB_G0272348 | | | | Similar to Dictyostelium discoideum (Slime mold). Adenylyl cyclase. | | | |
| kif7 | | | | kif7 | | | |  | | | |
| DDB_G0280973 | | | | abcC12 | | | | ABC transporter C family protein | | | |
| DDB_G0282365 | | | | rac1C | | | | Rho GTPase | | | |
| DDB_G0277897 | | | | racI | | | | Rho GTPase | | | |
| DDB_G0273257 | | | | comF | | | | unknown | | | |
| DDB_G0277873 | | | | sqpA | | | | glutamine-asparagine rich protein | | | |
| DDB_G0293450 | | | | abcG9 | | | | ABC transporter G family protein | | | |
| DDB_G0292986 | | | | abcG10 | | | | ABC transporter G family protein | | | |
| DDB_G0273073 | | | | abcG17-1 | | | | ABC transporter G family protein | | | |
| DDB_G0279191 | | | | vacB | | | | prohibitin domain-containing protein, vacuolin B | | | |
| DDB_G0280537 | | | | wacA | | | | aquaporin-like protein, major intrinsic protein family protein | | | |
| DDB_G0282369 | | | | ubqG | | | | ubiquitin | | | |
| DDB_G0280755 | | | | ubqB | | | | ubiquitin/ribosomal protein L40 fusion protein | | | |
| DDB_G0279921 | | | | gp130 | | | | glycoprotein 130 | | | |
| DDB_G0282815 | | | | DDB_G0282815 | | | | unknown | | | |
| DDB_G0277833 | | | | cofA | | | | cofilin | | | |
| DDB_G0277903 | | | | cotE | | | | spore coat protein | | | |
| DDB_G0282147 | | | | fadB | | | | delta 5 fatty acid desaturase | | | |
| DDB_G0279185 | | | | cprF | | | | cysteine proteinase | | | |
| DDB_G0278723 | | | | gdt1 | | | | GDT family protein kinase, protein kinase, TKL group, putative transmembrane protein, tyrosine kinase-like protein | | | |
| DDB_G0279187 | | | | cprG | | | | cysteine proteinase | | | |
| DDB_G0294164 | | | | DDB_G0294164 | | | | Protease homolog (Fragment). | | | |
| DDB_G0294188 | | | | DDB_G0294188 | | | |  | | | |
| DDB_G0294194 | | | | DDB_G0294194 | | | |  | | | |
| DDB_G0294200 | | | | DDB_G0294200 | | | |  | | | |
| DDB_G0294208 | | | | DDB_G0294208 | | | |  | | | |
| DDB_G0294220 | | | | DDB_G0294220 | | | |  | | | |
| DDB_G0294228 | | | | DDB_G0294228 | | | | Protease homolog (Fragment). | | | |
| DDB_G0294236 | | | | DDB_G0294236 | | | | Polyprotein. | | | |
| DDB_G0294240 | | | | DDB_G0294240 | | | |  | | | |
| DDB_G0294250 | | | | DDB_G0294250 | | | | LTR-RETROTRANSPOSON SKIPPER, GAG (Fragment). | | | |
| DDB_G0294252 | | | | DDB_G0294252 | | | | LTR-RETROTRANSPOSON SKIPPER, GAG (Fragment). | | | |
| DDB_G0294254 | | | | DDB_G0294254 | | | | LTR-RETROTRANSPOSON SKIPPER, GAG (Fragment). | | | |
| DDB_G0294256 | | | | DDB_G0294256 | | | | LTR-RETROTRANSPOSON SKIPPER, GAG (Fragment). | | | |
| DDB_G0294258 | | | | DDB_G0294258 | | | |  | | | |
| DDB_G0294320 | | | | DDB_G0294320 | | | | LTR-RETROTRANSPOSON SKIPPER, GAG (Fragment). | | | |
| DDB_G0294322 | | | | DDB_G0294322 | | | | LTR-RETROTRANSPOSON SKIPPER, GAG (Fragment). | | | |
| DDB_G0294336 | | | | DDB_G0294336 | | | |  | | | |
| DDB_G0294338 | | | | DDB_G0294338 | | | |  | | | |
| DDB_G0280861 | | | | DDB_G0280861 | | | |  | | | |
| DDB_G0280869 | | | | DDB_G0280869 | | | |  | | | |
| DDB_G0280879 | | | | DDB_G0280879 | | | |  | | | |
| DDB_G0278973 | | | | DDB_G0278973 | | | |  | | | |
| DDB_G0278997 | | | | DDB_G0278997 | | | |  | | | |
| DDB_G0279017 | | | | DDB_G0279017 | | | |  | | | |
| DDB_G0279021 | | | | DDB_G0279021 | | | |  | | | |
| DDB_G0279055 | | | | DDB_G0279055 | | | | Pol. | | | |
| DDB_G0282141 | | | | hatA | | | | actin binding protein, hisactophilin I | | | |
| DDB_G0271140 | | | | abcA7 | | | | ABC transporter A family protein | | | |
| DDB_G0280539 | | | | abcC13 | | | | ABC transporter C family protein | | | |
| DDB_G0281391 | | | | abcG5 | | | | ABC transporter G family protein | | | |
| DDB_G0278969 | | | | empC | | | | emp24/gp25L/p24 family protein | | | |
| DDB_G0271148 | | | | rps25 | | | | 40S ribosomal protein S25 | | | |
| DDB_G0280541 | | | | abcC1 | | | | ABC transporter C family protein | | | |
| DDB_G0280977 | | | | abcC10 | | | | ABC transporter C family protein | | | |
| DDB_G0279917 | | | | abcD1 | | | | ABC transporter D family protein | | | |
| DDB_G0269176 | | | | racF1 | | | | Rho GTPase | | | |
| DDB_G0273063 | | | | dscA-1 | | | | discoidin I, A chain, discoidin I, alpha chain | | | |
| DDB_G0273065 | | | | dscC-1 | | | | discoidin I, C chain and B chain, discoidin I, beta chain, discoidin I, gamma chain | | | |
| DDB_G0291624 | | | | DDB_G0291624 | | | |  | | | |
| DDB_G0291630 | | | | DDB_G0291630 | | | |  | | | |
| DDB_G0291770 | | | | DDB_G0291770 | | | |  | | | |
| DDB_G0292512 | | | | DDB_G0292512 | | | |  | | | |
| DDB_G0292514 | | | | DDB_G0292514 | | | |  | | | |
| DDB_G0292702 | | | | DDB_G0292702 | | | |  | | | |
| DDB_G0292950 | | | | DDB_G0292950 | | | |  | | | |
| DDB_G0292952 | | | | DDB_G0292952 | | | |  | | | |
| DDB_G0293140 | | | | DDB_G0293140 | | | | Pol. | | | |
| DDB_G0293274 | | | | DDB_G0293274 | | | |  | | | |
| DDB_G0293964 | | | | DDB_G0293964 | | | | Pol. | | | |
| DDB_G0293778 | | | | DDB_G0293778 | | | |  | | | |
| DDB_G0283323 | | | | DDB_G0283323 | | | |  | | | |
| DDB_G0283485 | | | | DDB_G0283485 | | | |  | | | |
| DDB_G0283487 | | | | DDB_G0283487 | | | |  | | | |
| DDB_G0283853 | | | | DDB_G0283853 | | | |  | | | |
| DDB_G0286963 | | | | DDB_G0286963 | | | |  | | | |
| DDB_G0287183 | | | | DDB_G0287183 | | | |  | | | |
| DDB_G0287185 | | | | DDB_G0287185 | | | |  | | | |
| DDB_G0285041 | | | | DDB_G0285041 | | | |  | | | |
| DDB_G0285297 | | | | DDB_G0285297 | | | |  | | | |
| DDB_G0285299 | | | | DDB_G0285299 | | | | Putative purine permease ycdG (Similar to Agrobacterium tumefaciens (Strain C58/ATCC 33970) uracil transport protein). | | | |
| DDB_G0286317 | | | | DDB_G0286317 | | | |  | | | |
| DDB_G0288233 | | | | DDB_G0288233 | | | |  | | | |
| DDB_G0288499 | | | | DDB_G0288499 | | | |  | | | |
| DDB_G0290349 | | | | DDB_G0290349 | | | | Similar to Dictyostelium discoideum (Slime mold). hypothetical 127.0 kDa protein. | | | |
| DDB_G0288527 | | | | DDB_G0288527 | | | |  | | | |
| DDB_G0291045 | | | | DDB_G0291045 | | | | Similar to Dictyostelium discoideum (Slime mold). Hypothetical 127.0 kDa protein. | | | |
| DDB_G0289633 | | | | DDB_G0289633 | | | | Similar to Dictyostelium discoideum (Slime mold). CIGB protein. | | | |
| DDB_G0289789 | | | | DDB_G0289789 | | | |  | | | |
| DDB_G0289849 | | | | DDB_G0289849 | | | | Q94481 Protein cigB (Fragment). | | | |
| DDB_G0289851 | | | | DDB_G0289851 | | | | Similar to Dictyostelium discoideum (Slime mold). CIGB protein. | | | |
| DDB_G0290443 | | | | DDB_G0290443 | | | |  | | | |
| DDB_G0287583 | | | | gerC | | | | 109 gene 3 protein | | | |
| DDB_G0277829 | | | | carC | | | | G-protein-coupled receptor, cAMP receptor 3 | | | |
| DDB_G0269234 | | | | act8 | | | | actin | | | |
| DDB_G0274129 | | | | act12 | | | | actin | | | |
| DDB_G0289391 | | | | pkiA | | | | histidine triad (HIT) family protein, putative protein kinase C inhibitor | | | |
| DDB_G0287685 | | | | cinC | | | | elongation factor 2, vegetative specific protein H6 | | | |
| DDB_G0285165 | | | | abcC9 | | | | ABC transporter C family protein | | | |
| DDB_G0290529 | | | | cpnE | | | | copine E, phospholipid-binding protein | | | |
| DDB_G0267688 | | | | cpnD | | | | copine D, phospholipid-binding protein | | | |
| DDB_G0272875 | | | | cpnB-1 | | | | copine B, phospholipid-binding protein | | | |
| DDB_G0271138 | | | | abcA8 | | | | ABC transporter A family protein | | | |
| DDB_G0277831 | | | | carD | | | | G-protein-coupled receptor, cAMP receptor 4 | | | |
| DDB_G0290173 | | | | bzpI | | | | putative basic-leucine zipper (bZIP) transcription factor | | | |
| DDB_G0272036 | | | | rpb10 | | | | RNA polymerase I core subunit, RNA polymerase II core subunit, RNA polymerase III core subunit, RNA polymerase N/8 kDa subunit | | | |
| DDB_G0270814 | | | | nek2 | | | | NEK family protein kinase, protein serine/threonine kinase | | | |
| DDB_G0273425 | | | | rpc25 | | | | RNA polymerase III subunit | | | |
| DDB_G0276863 | | | | H4b | | | | histone H4 | | | |
| DDB_G0275561 | | | | fnkA | | | | FNIP repeat-containing protein, FNIPK subfamily protein kinase, protein kinase, STE group | | | |
| DDB_G0277591 | | | | gtaH | | | | GATA zinc finger domain-containing protein 8, putative GATA-binding transcription factor | | | |
| DDB_G0286843 | | | | gtaQ | | | | GATA zinc finger domain-containing protein 17, putative GATA-binding transcription factor | | | |
| DDB_G0280639 | | | | gtaT | | | | GATA zinc finger domain-containing protein 20, putative GATA-binding transcription factor | | | |
| DDB_G0278783 | | | | elp4 | | | | RNA polymerase II elongator complex subunit, elongation protein 4 | | | |
| DDB_G0280459 | | | | abcC15 | | | | ABC transporter C family protein | | | |
| DDB_G0280717 | | | | DDB_G0280717 | | | | MAST family protein kinase, protein kinase, AGC group, putative protein serine/threonine kinase | | | |
| DDB_G0290169 | | | | bzpO | | | | putative basic-leucine zipper (bZIP) transcription factor | | | |
| DDB_G0279977 | | | | bud32 | | | | BUD32 family protein kinase, putative protein serine/threonine kinase | | | |
| DDB_G0284897 | | | | abkD | | | | ABC1 family protein kinase, ABC1-B subfamily protein kinase, putative protein serine/threonine kinase | | | |
| DDB_G0284795 | | | | DDB_G0284795 | | | | NAD(+)-dependent deacetylase, silent information regulator protein (Sir2) family protein, UBP-type zinc finger-containing protein | | | |
| DDB_G0267226 | | | | DDB_G0267226 | | | | Putative retroelement pol polyprotein. | | | |
| DDB_G0267304 | | | | DDB_G0267304 | | | | Slime mold (D.discoideum) transposon DIRS-1, complete, clone SB41. | | | |
| DDB_G0267306 | | | | DDB_G0267306 | | | | LTR-RETROTRANSPOSON SKIPPER, GAG (Fragment). | | | |
| DDB_G0267356 | | | | DDB_G0267356 | | | | LTR-RETROTRANSPOSON SKIPPER, GAG (Fragment). | | | |
| DDB_G0267366 | | | | DDB_G0267366 | | | | LTR-RETROTRANSPOSON SKIPPER, GAG (Fragment). | | | |
| DDB_G0268482 | | | | DDB_G0268482 | | | |  | | | |
| DDB_G0268488 | | | | DDB_G0268488 | | | |  | | | |
| DDB_G0268490 | | | | DDB_G0268490 | | | |  | | | |
| DDB_G0268494 | | | | DDB_G0268494 | | | |  | | | |
| DDB_G0268502 | | | | DDB_G0268502 | | | |  | | | |
| DDB_G0268506 | | | | DDB_G0268506 | | | | Transcription factor (Fragment). | | | |
| DDB_G0268508 | | | | DDB_G0268508 | | | | Pol. | | | |
| DDB_G0268524 | | | | DDB_G0268524 | | | | Pol. | | | |
| DDB_G0268528 | | | | DDB_G0268528 | | | | Pol. | | | |
| DDB_G0268546 | | | | DDB_G0268546 | | | |  | | | |
| DDB_G0268562 | | | | DDB_G0268562 | | | |  | | | |
| DDB_G0268570 | | | | DDB_G0268570 | | | |  | | | |
| DDB_G0268580 | | | | DDB_G0268580 | | | | Pol. | | | |
| DDB_G0268588 | | | | DDB_G0268588 | | | |  | | | |
| DDB_G0268598 | | | | DDB_G0268598 | | | | Pol. | | | |
| DDB_G0268608 | | | | DDB_G0268608 | | | | Multifunctional protein (Fragment). | | | |
| DDB_G0268610 | | | | DDB_G0268610 | | | | Pol. | | | |
| DDB_G0268612 | | | | DDB_G0268612 | | | | Similar to Dictyostelium discoideum (Slime mold). hypothetical 97.7 kDa protein. | | | |
| DDB_G0269036 | | | | DDB_G0269036 | | | | Clone 9.10 TDD-3 and red repetitive elements, partial sequence. | | | |
| DDB_G0269042 | | | | DDB_G0269042 | | | | Similar to Dictyostelium discoideum (Slime mold). Hypothetical 97.7 kDa protein. | | | |
| DDB_G0269048 | | | | DDB_G0269048 | | | |  | | | |
| DDB_G0269050 | | | | DDB_G0269050 | | | |  | | | |
| DDB_G0269056 | | | | DDB_G0269056 | | | | Group-specific antigen. | | | |
| DDB_G0269068 | | | | DDB_G0269068 | | | |  | | | |
| DDB_G0269080 | | | | DDB_G0269080 | | | |  | | | |
| DDB_G0269084 | | | | DDB_G0269084 | | | |  | | | |
| DDB_G0269090 | | | | DDB_G0269090 | | | | Similar to Dictyostelium discoideum (Slime mold). hypothetical 97.7 kDa protein. | | | |
| DDB_G0269098 | | | | DDB_G0269098 | | | | Multifunctional protein (Fragment). | | | |
| DDB_G0270848 | | | | DDB_G0270848 | | | |  | | | |
| DDB_G0270850 | | | | DDB_G0270850 | | | |  | | | |
| DDB_G0270852 | | | | DDB_G0270852 | | | | Pol. | | | |
| DDB_G0270860 | | | | DDB_G0270860 | | | | Pol. | | | |
| DDB_G0270878 | | | | DDB_G0270878 | | | |  | | | |
| DDB_G0270886 | | | | DDB_G0270886 | | | | Multifunctional protein (Fragment). | | | |
| DDB_G0270926 | | | | DDB_G0270926 | | | |  | | | |
| DDB_G0270934 | | | | DDB_G0270934 | | | | Triacylglycerol lipase-like protein triacylglycerol lipase (Similar to Oryza sativa (Japonica cultivar-group) putative triacylglycerol lipase). | | | |
| DDB_G0270944 | | | | DDB_G0270944 | | | |  | | | |
| DDB_G0270946 | | | | DDB_G0270946 | | | | Putative sterol 4-alpha-methyl-oxidase. | | | |
| DDB_G0270950 | | | | DDB_G0270950 | | | | Pol. | | | |
| DDB_G0270954 | | | | DDB_G0270954 | | | | LTR-RETROTRANSPOSON SKIPPER, GAG (Fragment). | | | |
| DDB_G0270998 | | | | DDB_G0270998 | | | | Similar to Dictyostelium discoideum (Slime mold). Hypothetical 127.0 kDa protein. | | | |
| DDB_G0271004 | | | | DDB_G0271004 | | | | Clone 9.10 TDD-3 and red repetitive elements, partial sequence. | | | |
| DDB_G0271006 | | | | DDB_G0271006 | | | | Pol. | | | |
| DDB_G0271018 | | | | DDB_G0271018 | | | | Pol. | | | |
| DDB_G0271024 | | | | DDB_G0271024 | | | | unknown | | | |
| DDB_G0271026 | | | | DDB_G0271026 | | | | Pol. | | | |
| DDB_G0271034 | | | | DDB_G0271034 | | | | Similar to Homo sapiens (Human). Tenascin (TN) (Hexabrachion) (Cytotactin) (Neuronectin) (GMEM) (JI) (Miotendinous antigen) (Glioma-associated-extracellular matrix antigen) (GP 150-225) (Tenascin-C) (TN-C). | | | |
| DDB_G0271042 | | | | DDB_G0271042 | | | | Similar to Mus musculus (Mouse). tenascin X. | | | |
| DDB_G0271060 | | | | DDB_G0271060 | | | |  | | | |
| DDB_G0271084 | | | | DDB_G0271084 | | | |  | | | |
| DDB_G0271362 | | | | DDB_G0271362 | | | |  | | | |
| DDB_G0271368 | | | | DDB_G0271368 | | | |  | | | |
| DDB_G0271386 | | | | DDB_G0271386 | | | |  | | | |
| DDB_G0271396 | | | | DDB_G0271396 | | | |  | | | |
| DDB_G0271404 | | | | DDB_G0271404 | | | |  | | | |
| DDB_G0271406 | | | | DDB_G0271406 | | | |  | | | |
| DDB_G0271408 | | | | DDB_G0271408 | | | |  | | | |
| DDB_G0271412 | | | | DDB_G0271412 | | | |  | | | |
| DDB_G0271414 | | | | DDB_G0271414 | | | |  | | | |
| DDB_G0271418 | | | | DDB_G0271418 | | | |  | | | |
| DDB_G0271424 | | | | DDB_G0271424 | | | |  | | | |
| DDB_G0271428 | | | | DDB_G0271428 | | | |  | | | |
| DDB_G0271438 | | | | DDB_G0271438 | | | |  | | | |
| DDB_G0271448 | | | | DDB_G0271448 | | | | Multifunctional protein (Fragment). | | | |
| DDB_G0271450 | | | | DDB_G0271450 | | | | Clone 9.10 TDD-3 and red repetitive elements, partial sequence. | | | |
| DDB_G0271452 | | | | DDB_G0271452 | | | | Multifunctional protein (Fragment). | | | |
| DDB_G0271458 | | | | DDB_G0271458 | | | |  | | | |
| DDB_G0271460 | | | | DDB_G0271460 | | | |  | | | |
| DDB_G0271462 | | | | DDB_G0271462 | | | |  | | | |
| DDB_G0271468 | | | | DDB_G0271468 | | | |  | | | |
| DDB_G0271472 | | | | DDB_G0271472 | | | |  | | | |
| DDB_G0271476 | | | | DDB_G0271476 | | | |  | | | |
| DDB_G0271478 | | | | DDB_G0271478 | | | |  | | | |
| DDB_G0271480 | | | | DDB_G0271480 | | | |  | | | |
| DDB_G0271482 | | | | DDB_G0271482 | | | |  | | | |
| DDB_G0271594 | | | | DDB_G0271594 | | | | Similar to Dictyostelium discoideum (Slime mold). cell surface protein DTFA. | | | |
| DDB_G0271598 | | | | DDB_G0271598 | | | | Lagz1 to Dictyostelium discoideum (Slime mold). LagC2. | | | |
| DDB_G0271600 | | | | DDB_G0271600 | | | | Similar to Dictyostelium discoideum (Slime mold). CIGB protein. | | | |
| DDB_G0271602 | | | | DDB_G0271602 | | | | Similar to Dictyostelium discoideum (Slime mold). CIGB protein. | | | |
| DDB_G0271624 | | | | DDB_G0271624 | | | |  | | | |
| DDB_G0271634 | | | | DDB_G0271634 | | | | Similar to Dictyostelium discoideum (Slime mold). Hypothetical 127.0 kDa protein. | | | |
| DDB_G0271636 | | | | DDB_G0271636 | | | | Similar to Homo sapiens (Human). Tenascin (TN) (Hexabrachion) (Cytotactin) (Neuronectin) (GMEM) (JI) (Miotendinous antigen) (Glioma-associated-extracellular matrix antigen) (GP 150-225) (Tenascin-C) (TN-C). | | | |
| DDB_G0271638 | | | | DDB_G0271638 | | | | Similar to Dictyostelium discoideum (Slime mold). hypothetical 127.0 kDa protein. | | | |
| DDB_G0271640 | | | | DDB_G0271640 | | | | P36417 G-box binding factor (GBF). | | | |
| DDB_G0271644 | | | | DDB_G0271644 | | | | Similar to Dictyostelium discoideum (Slime mold). LvsD. | | | |
| DDB_G0271646 | | | | DDB_G0271646 | | | | lagV3 to Dictyostelium discoideum (Slime mold). LagC2. | | | |
| DDB_G0271652 | | | | DDB_G0271652 | | | | lagV2 ctyostelium discoideum (Slime mold). LagC2. | | | |
| DDB_G0271654 | | | | DDB_G0271654 | | | |  | | | |
| DDB_G0271660 | | | | DDB_G0271660 | | | | Similar to Caenorhabditis elegans. F53F4.5 protein. | | | |
| DDB_G0271802 | | | | DDB_G0271802 | | | | Similar to short chain dehydrogenase. | | | |
| DDB_G0271826 | | | | DDB_G0271826 | | | | Similar to Plasmodium falciparum (Isolate 3D7). Asparagine-rich protein, putative. | | | |
| DDB_G0271936 | | | | DDB_G0271936 | | | |  | | | |
| DDB_G0271938 | | | | DDB_G0271938 | | | | Similar to Dictyostelium discoideum (Slime mold). CIGB protein. | | | |
| DDB_G0271950 | | | | DDB_G0271950 | | | | Similar to Dictyostelium discoideum (Slime mold). CIGB protein. | | | |
| DDB_G0272098 | | | | DDB_G0272098 | | | |  | | | |
| DDB_G0272102 | | | | DDB_G0272102 | | | |  | | | |
| DDB_G0272356 | | | | DDB_G0272356 | | | | Similar to Dictyostelium discoideum (Slime mold). R2005 protein. | | | |
| DDB_G0272364 | | | | DDB_G0272364 | | | | Similar to Mus musculus (Mouse). 12 days embryo spinal ganglion cDNA, RIKEN full-length enriched library, clone:D130061K05 product:MEGF11 PROTEIN (KIAA1781) homolog. | | | |
| DDB_G0272366 | | | | DDB_G0272366 | | | | Similar to Mus musculus (Mouse). 12 days embryo spinal ganglion cDNA, RIKEN full-length enriched library, clone:D130061K05 product:MEGF11 PROTEIN (KIAA1781) homolog. | | | |
| DDB_G0272370 | | | | DDB_G0272370 | | | |  | | | |
| DDB_G0272382 | | | | DDB_G0272382 | | | |  | | | |
| DDB_G0272400 | | | | DDB_G0272400 | | | | Pol. | | | |
| DDB_G0272408 | | | | DDB_G0272408 | | | | Pol. | | | |
| DDB_G0272434 | | | | DDB_G0272434 | | | | Similar to Mus musculus (Mouse). Latent transforming growth factor beta binding protein 4 short splice variant. | | | |
| DDB_G0272442 | | | | DDB_G0272442 | | | |  | | | |
| DDB_G0272452 | | | | DDB_G0272452 | | | | Similar to Leishmania major. L411.4. | | | |
| DDB_G0272474 | | | | DDB_G0272474 | | | |  | | | |
| DDB_G0272482 | | | | DDB_G0272482 | | | |  | | | |
| DDB_G0272536 | | | | DDB_G0272536 | | | |  | | | |
| DDB_G0272538 | | | | DDB_G0272538 | | | | Similar to Plasmodium falciparum (Isolate 3D7). Hypothetical 111.5 kDa protein. | | | |
| DDB_G0272558 | | | | DDB_G0272558 | | | | Similar to Babesia bigemina. 200 kDa antigen p200. | | | |
| DDB_G0272941 | | | | DDB_G0272941 | | | |  | | | |
| DDB_G0272945 | | | | DDB_G0272945 | | | | Similar to plasmodium falciparum. hypothetical 220.3 kDa protein. | | | |
| DDB_G0272961 | | | | DDB_G0272961 | | | |  | | | |
| DDB_G0272963 | | | | DDB_G0272963 | | | |  | | | |
| DDB_G0272973 | | | | DDB_G0272973 | | | |  | | | |
| DDB_G0272977 | | | | DDB_G0272977 | | | | Similar to plasmodium falciparum (Isolate 3D7). hypothetical 98.3 kDa protein. | | | |
| DDB_G0272995 | | | | DDB_G0272995 | | | |  | | | |
| DDB_G0273009 | | | | DDB_G0273009 | | | |  | | | |
| DDB_G0273011 | | | | DDB_G0273011 | | | |  | | | |
| DDB_G0273015 | | | | DDB_G0273015 | | | | Similar to Dictyostelium discoideum (Slime mold). Hypothetical 127.0 kDa protein. | | | |
| DDB_G0273027 | | | | DDB_G0273027 | | | | Putative RNaseIII. | | | |
| DDB_G0273029 | | | | DDB_G0273029 | | | | Similar to Dictyostelium discoideum (Slime mold). putative RNaseIII. | | | |
| DDB_G0273031 | | | | DDB_G0273031 | | | | Similar to Oryza sativa (Rice). ESTs AU069374(C61044). | | | |
| DDB_G0273033 | | | | DDB_G0273033 | | | |  | | | |
| DDB_G0273037 | | | | DDB_G0273037 | | | | Similar to plasmodium falciparum (Isolate 3D7). Metacaspase-like protein. | | | |
| DDB_G0273041 | | | | DDB_G0273041 | | | |  | | | |
| DDB_G0273049 | | | | DDB_G0273049 | | | | Similar to Dictyostelium discoideum (Slime mold). Ras-related protein RabA. | | | |
| DDB_G0273321 | | | | DDB_G0273321 | | | | Similar to Dictyostelium discoideum (Slime mold). phosphatidylinositol 3-kinase 2 (EC 2.7.1.137) (PI3-kinase) (PtdIns-3-kinase) (PI3K). | | | |
| DDB_G0273327 | | | | DDB_G0273327 | | | | Pol. | | | |
| DDB_G0273331 | | | | DDB_G0273331 | | | |  | | | |
| DDB_G0273339 | | | | DDB_G0273339 | | | | Similar to plasmodium falciparum. CCAAT-box DNA binding protein subunit B. | | | |
| DDB_G0273343 | | | | DDB_G0273343 | | | |  | | | |
| DDB_G0273345 | | | | DDB_G0273345 | | | | Pol. | | | |
| DDB_G0273355 | | | | DDB_G0273355 | | | |  | | | |
| DDB_G0273357 | | | | DDB_G0273357 | | | | Similar to Homo sapiens (Human). mucin 2 (Intestinal mucin 2). | | | |
| DDB_G0273369 | | | | DDB_G0273369 | | | | Similar to Dictyostelium discoideum (Slime mold). protein-tyrosine phosphatase 3 (EC 3.1.3.48) (Protein-tyrosine-phosphate phosphohydrolase 3). | | | |
| DDB_G0273371 | | | | DDB_G0273371 | | | | Similar to Dictyostelium discoideum (Slime mold). protein-tyrosine phosphatase 3 (EC 3.1.3.48) (Protein-tyrosine-phosphate phosphohydrolase 3). | | | |
| DDB_G0273379 | | | | DDB_G0273379 | | | |  | | | |
| DDB_G0273381 | | | | DDB_G0273381 | | | |  | | | |
| DDB_G0273385 | | | | DDB_G0273385 | | | |  | | | |
| DDB_G0273391 | | | | DDB_G0273391 | | | |  | | | |
| DDB_G0273395 | | | | DDB_G0273395 | | | |  | | | |
| DDB_G0273467 | | | | DDB_G0273467 | | | | Similar to Homo sapiens (Human). MUM2 protein (Fragment). | | | |
| DDB_G0273477 | | | | DDB_G0273477 | | | | DHHC zinc finger domain, putative. | | | |
| DDB_G0273481 | | | | DDB_G0273481 | | | |  | | | |
| DDB_G0273483 | | | | DDB_G0273483 | | | |  | | | |
| DDB_G0273487 | | | | DDB_G0273487 | | | |  | | | |
| DDB_G0273491 | | | | DDB_G0273491 | | | |  | | | |
| DDB_G0273543 | | | | DDB_G0273543 | | | |  | | | |
| DDB_G0273591 | | | | DDB_G0273591 | | | |  | | | |
| DDB_G0273599 | | | | DDB_G0273599 | | | |  | | | |
| DDB_G0273629 | | | | DDB_G0273629 | | | |  | | | |
| DDB_G0273635 | | | | DDB_G0273635 | | | |  | | | |
| DDB_G0273641 | | | | DDB_G0273641 | | | | Similar to Arabidopsis thaliana (Mouse-ear cress). T3F20.12 protein (MEK kinase MAP3Ka, putative) (MAP3K alpha protein kinase, putative). | | | |
| DDB_G0273643 | | | | DDB_G0273643 | | | |  | | | |
| DDB_G0273653 | | | | DDB_G0273653 | | | |  | | | |
| DDB_G0273671 | | | | DDB_G0273671 | | | |  | | | |
| DDB_G0273679 | | | | DDB_G0273679 | | | | Similar to plasmodium falciparum. SET-domain protein, putative. | | | |
| DDB_G0273681 | | | | DDB_G0273681 | | | | Similar to Dictyostelium discoideum (Slime mold). protein-tyrosine phosphatase 3 (EC 3.1.3.48) (Protein-tyrosine-phosphate phosphohydrolase 3). | | | |
| DDB_G0273683 | | | | DDB_G0273683 | | | | Similar to Dictyostelium discoideum (Slime mold). protein-tyrosine phosphatase 3 (EC 3.1.3.48) (Protein-tyrosine-phosphate phosphohydrolase 3). | | | |
| DDB_G0273703 | | | | DDB_G0273703 | | | |  | | | |
| DDB_G0273705 | | | | DDB_G0273705 | | | |  | | | |
| DDB_G0273733 | | | | DDB_G0273733 | | | | Similar to Homo sapiens (Human). mucin 2 (Intestinal mucin 2). | | | |
| DDB_G0273749 | | | | DDB_G0273749 | | | | Similar to Dictyostelium discoideum (Slime mold). histidine kinase DhkE. | | | |
| DDB_G0273761 | | | | DDB_G0273761 | | | | Pol. | | | |
| DDB_G0273763 | | | | DDB_G0273763 | | | |  | | | |
| DDB_G0273769 | | | | DDB_G0273769 | | | |  | | | |
| DDB_G0273793 | | | | DDB_G0273793 | | | | Similar to Dictyostelium discoideum (Slime mold). prespore-specific protein. | | | |
| DDB_G0273801 | | | | DDB_G0273801 | | | | Similar to Dictyostelium discoideum (Slime mold). hypothetical 127.0 kDa protein. | | | |
| DDB_G0273819 | | | | DDB_G0273819 | | | |  | | | |
| DDB_G0273823 | | | | DDB_G0273823 | | | | Similar to Dictyostelium discoideum (Slime mold). hypothetical 97.7 kDa protein. | | | |
| DDB_G0273829 | | | | DDB_G0273829 | | | | Similar to delayed anaerobic Gene; Dan4p. | | | |
| DDB_G0273833 | | | | DDB_G0273833 | | | | Similar to Dictyostelium discoideum (Slime mold). putative calmodulin-binding protein CaM-BP46. | | | |
| DDB_G0273841 | | | | DDB_G0273841 | | | | Similar to Dictyostelium discoideum (Slime mold). MkpA protein. | | | |
| DDB_G0273851 | | | | DDB_G0273851 | | | |  | | | |
| DDB_G0273867 | | | | DDB_G0273867 | | | | Similar to Dictyostelium discoideum (Slime mold). prestalk protein. | | | |
| DDB_G0273879 | | | | DDB_G0273879 | | | | Pol. | | | |
| DDB_G0273893 | | | | DDB_G0273893 | | | |  | | | |
| DDB_G0273895 | | | | DDB_G0273895 | | | |  | | | |
| DDB_G0273901 | | | | DDB_G0273901 | | | | Pol. | | | |
| DDB_G0273933 | | | | DDB_G0273933 | | | | Similar to Dictyostelium discoideum (Slime mold). Ras-related protein RabA. | | | |
| DDB_G0273971 | | | | DDB_G0273971 | | | |  | | | |
| DDB_G0273985 | | | | DDB_G0273985 | | | |  | | | |
| DDB_G0273987 | | | | DDB_G0273987 | | | |  | | | |
| DDB_G0274037 | | | | DDB_G0274037 | | | |  | | | |
| DDB_G0274049 | | | | DDB_G0274049 | | | |  | | | |
| DDB_G0274087 | | | | DDB_G0274087 | | | |  | | | |
| DDB_G0274089 | | | | DDB_G0274089 | | | |  | | | |
| DDB_G0274091 | | | | DDB_G0274091 | | | | Similar to Dictyostelium discoideum (Slime mold). MkpA protein. | | | |
| DDB_G0274807 | | | | DDB_G0274807 | | | |  | | | |
| DDB_G0274817 | | | | DDB_G0274817 | | | |  | | | |
| DDB_G0274819 | | | | DDB_G0274819 | | | |  | | | |
| DDB_G0274837 | | | | DDB_G0274837 | | | | Pol. | | | |
| DDB_G0274839 | | | | DDB_G0274839 | | | | Pol. | | | |
| DDB_G0274841 | | | | DDB_G0274841 | | | | Similar to ATP-dependent RNA helicase, putative; protein id: At1g35530.1. | | | |
| DDB_G0274851 | | | | DDB_G0274851 | | | |  | | | |
| DDB_G0274869 | | | | DDB_G0274869 | | | | Similar to Dictyostelium discoideum (Slime mold). cell surface glycoprotein GP138B. | | | |
| DDB_G0274881 | | | | DDB_G0274881 | | | |  | | | |
| DDB_G0274883 | | | | DDB_G0274883 | | | |  | | | |
| DDB_G0274887 | | | | DDB_G0274887 | | | | Similar to Acanthamoeba castellanii (Amoeba). disulfide-like protein. | | | |
| DDB_G0274903 | | | | DDB_G0274903 | | | |  | | | |
| DDB_G0274907 | | | | DDB_G0274907 | | | |  | | | |
| DDB_G0274909 | | | | DDB_G0274909 | | | | Similar to Dictyostelium discoideum (Slime mold). coronin binding protein. | | | |
| DDB_G0274919 | | | | DDB_G0274919 | | | |  | | | |
| DDB_G0274937 | | | | DDB_G0274937 | | | | Similar to Dictyostelium discoideum (Slime mold). phosphatidylinositol 3-kinase 2 (EC 2.7.1.137) (PI3-kinase) (PtdIns-3-kinase) (PI3K). | | | |
| DDB_G0274949 | | | | DDB_G0274949 | | | | Similar to Dictyostelium discoideum (Slime mold). hypothetical 97.7 kDa protein. | | | |
| DDB_G0274963 | | | | DDB_G0274963 | | | |  | | | |
| DDB_G0274967 | | | | DDB_G0274967 | | | |  | | | |
| DDB_G0274983 | | | | DDB_G0274983 | | | |  | | | |
| DDB_G0274987 | | | | DDB_G0274987 | | | |  | | | |
| DDB_G0274995 | | | | DDB_G0274995 | | | |  | | | |
| DDB_G0275003 | | | | DDB_G0275003 | | | |  | | | |
| DDB_G0275243 | | | | DDB_G0275243 | | | |  | | | |
| DDB_G0275245 | | | | DDB_G0275245 | | | | Similar to Dictyostelium discoideum (Slime mold). Hypothetical 127.0 kDa protein. | | | |
| DDB_G0275251 | | | | DDB_G0275251 | | | |  | | | |
| DDB_G0275281 | | | | DDB_G0275281 | | | |  | | | |
| DDB_G0275283 | | | | DDB_G0275283 | | | |  | | | |
| DDB_G0275285 | | | | DDB_G0275285 | | | |  | | | |
| DDB_G0275307 | | | | DDB_G0275307 | | | | Similar to Pseudomonas putida KT2440. glutamine amidotransferase, class I. | | | |
| DDB_G0275309 | | | | DDB_G0275309 | | | |  | | | |
| DDB_G0275319 | | | | DDB_G0275319 | | | |  | | | |
| DDB_G0275393 | | | | DDB_G0275393 | | | |  | | | |
| DDB_G0275423 | | | | DDB_G0275423 | | | |  | | | |
| DDB_G0275427 | | | | DDB_G0275427 | | | |  | | | |
| DDB_G0275855 | | | | DDB_G0275855 | | | |  | | | |
| DDB_G0275893 | | | | DDB_G0275893 | | | |  | | | |
| DDB_G0275897 | | | | DDB_G0275897 | | | | Similar to Dictyostelium discoideum (Slime mold). CIGB protein. | | | |
| DDB_G0275927 | | | | DDB_G0275927 | | | |  | | | |
| DDB_G0275937 | | | | DDB_G0275937 | | | |  | | | |
| DDB_G0275943 | | | | DDB_G0275943 | | | | Pol. | | | |
| DDB_G0275945 | | | | DDB_G0275945 | | | | Pol. | | | |
| DDB_G0275947 | | | | DDB_G0275947 | | | | Pol. | | | |
| DDB_G0275993 | | | | DDB_G0275993 | | | |  | | | |
| DDB_G0275997 | | | | DDB_G0275997 | | | |  | | | |
| DDB_G0276005 | | | | DDB_G0276005 | | | |  | | | |
| DDB_G0276015 | | | | DDB_G0276015 | | | |  | | | |
| DDB_G0276017 | | | | DDB_G0276017 | | | |  | | | |
| DDB_G0276021 | | | | DDB_G0276021 | | | |  | | | |
| DDB_G0276387 | | | | DDB_G0276387 | | | | Multifunctional protein (Fragment). | | | |
| DDB_G0276391 | | | | DDB_G0276391 | | | | Similar to Gallus gallus (Chicken). 190 kDa tenascin. | | | |
| DDB_G0276539 | | | | DDB_G0276539 | | | |  | | | |
| DDB_G0276547 | | | | DDB_G0276547 | | | |  | | | |
| DDB_G0276559 | | | | DDB_G0276559 | | | |  | | | |
| DDB_G0276565 | | | | DDB_G0276565 | | | | Pol. | | | |
| DDB_G0276747 | | | | DDB_G0276747 | | | | Similar to K08H10.2a.p. | | | |
| DDB_G0277021 | | | | DDB_G0277021 | | | |  | | | |
| DDB_G0277023 | | | | DDB_G0277023 | | | |  | | | |
| DDB_G0277039 | | | | DDB_G0277039 | | | |  | | | |
| DDB_G0277055 | | | | DDB_G0277055 | | | | Similar to Dictyostelium discoideum (Slime mold). Phosphatidylinositol 3-kinase 3 (EC 2.7.1.137) (PI3-kinase) (PtdIns-3-kinase) (PI3K). | | | |
| DDB_G0277105 | | | | DDB_G0277105 | | | | Similar to Dictyostelium discoideum (Slime mold). MkpA protein. | | | |
| DDB_G0277109 | | | | DDB_G0277109 | | | | Similar to Dictyostelium discoideum (Slime mold). hypothetical 127.0 kDa protein (Fragment). | | | |
| DDB_G0277117 | | | | DDB_G0277117 | | | |  | | | |
| DDB_G0277121 | | | | DDB_G0277121 | | | |  | | | |
| DDB_G0277127 | | | | DDB_G0277127 | | | | Similar to Podocoryne carnea. EGF-like protein (Fragment). | | | |
| DDB_G0277301 | | | | DDB_G0277301 | | | | Similar to Dictyostelium discoideum (Slime mold). hypothetical 12.5 kDa protein. | | | |
| DDB_G0277325 | | | | DDB_G0277325 | | | | Similar to Xanthomonas campestris (Pv. campestris). tetracycline-efflux transporter. | | | |
| DDB_G0277355 | | | | DDB_G0277355 | | | |  | | | |
| DDB_G0277365 | | | | DDB_G0277365 | | | | Pol. | | | |
| DDB_G0277547 | | | | DDB_G0277547 | | | |  | | | |
| DDB_G0277555 | | | | DDB_G0277555 | | | |  | | | |
| DDB_G0277557 | | | | DDB_G0277557 | | | | Similar to Homo sapiens (Human). Tenascin (TN) (Hexabrachion) (Cytotactin) (Neuronectin) (GMEM) (JI) (Miotendinous antigen) (Glioma-associated-extracellular matrix antigen) (GP 150-225) (Tenascin-C) (TN-C). | | | |
| DDB_G0277559 | | | | DDB_G0277559 | | | |  | | | |
| DDB_G0277577 | | | | DDB_G0277577 | | | |  | | | |
| DDB_G0277637 | | | | DDB_G0277637 | | | |  | | | |
| DDB_G0277639 | | | | DDB_G0277639 | | | | Similar to G-protein-coupled receptor at plasma membrane; interactions in two-hybrid system with Gpa2p; Gpr1p. | | | |
| DDB_G0277641 | | | | DDB_G0277641 | | | | Similar to G-protein-coupled receptor at plasma membrane; interactions in two-hybrid system with Gpa2p; Gpr1p. | | | |
| DDB_G0277643 | | | | DDB_G0277643 | | | | Similar to G-protein-coupled receptor at plasma membrane; interactions in two-hybrid system with Gpa2p; Gpr1p. | | | |
| DDB_G0277645 | | | | DDB_G0277645 | | | | Similar to G-protein-coupled receptor at plasma membrane; interactions in two-hybrid system with Gpa2p; Gpr1p. | | | |
| DDB_G0277647 | | | | DDB_G0277647 | | | | Similar to G-protein-coupled receptor at plasma membrane; interactions in two-hybrid system with Gpa2p; Gpr1p. | | | |
| DDB_G0277653 | | | | DDB_G0277653 | | | | Similar to G-protein-coupled receptor at plasma membrane; interactions in two-hybrid system with Gpa2p; Gpr1p. | | | |
| DDB_G0277655 | | | | DDB_G0277655 | | | | Similar to G-protein-coupled receptor at plasma membrane; interactions in two-hybrid system with Gpa2p; Gpr1p. | | | |
| DDB_G0277657 | | | | DDB_G0277657 | | | | Similar to G-protein-coupled receptor at plasma membrane; interactions in two-hybrid system with Gpa2p; Gpr1p. | | | |
| DDB_G0277659 | | | | DDB_G0277659 | | | | Similar to G-protein-coupled receptor at plasma membrane; interactions in two-hybrid system with Gpa2p; Gpr1p. | | | |
| DDB_G0277661 | | | | DDB_G0277661 | | | | Similar to G-protein-coupled receptor at plasma membrane; interactions in two-hybrid system with Gpa2p; Gpr1p. | | | |
| DDB_G0277711 | | | | DDB_G0277711 | | | | Group-specific antigen. | | | |
| DDB_G0277715 | | | | DDB_G0277715 | | | | Pol. | | | |
| DDB_G0278611 | | | | DDB_G0278611 | | | |  | | | |
| DDB_G0278633 | | | | DDB_G0278633 | | | | Clone 9.10 TDD-3 and red repetitive elements, partial sequence. | | | |
| DDB_G0278647 | | | | DDB_G0278647 | | | |  | | | |
| DDB_G0278659 | | | | DDB_G0278659 | | | |  | | | |
| DDB_G0278677 | | | | DDB_G0278677 | | | |  | | | |
| DDB_G0279065 | | | | DDB_G0279065 | | | |  | | | |
| DDB_G0279071 | | | | DDB_G0279071 | | | |  | | | |
| DDB_G0279127 | | | | DDB_G0279127 | | | | Pol. | | | |
| DDB_G0279389 | | | | DDB_G0279389 | | | | Pol. | | | |
| DDB_G0279393 | | | | DDB_G0279393 | | | |  | | | |
| DDB_G0279597 | | | | DDB_G0279597 | | | |  | | | |
| DDB_G0279603 | | | | DDB_G0279603 | | | |  | | | |
| DDB_G0279727 | | | | DDB_G0279727 | | | |  | | | |
| DDB_G0279859 | | | | DDB_G0279859 | | | | Similar to Boophilus microplus (Cattle tick). notch-like protein. | | | |
| DDB_G0279897 | | | | DDB_G0279897 | | | |  | | | |
| DDB_G0279899 | | | | DDB_G0279899 | | | |  | | | |
| DDB_G0279901 | | | | DDB_G0279901 | | | |  | | | |
| DDB_G0280011 | | | | DDB_G0280011 | | | | Pol. | | | |
| DDB_G0280029 | | | | DDB_G0280029 | | | | Similar to Mus musculus (Mouse). tenascin C. | | | |
| DDB_G0280031 | | | | DDB_G0280031 | | | | Pol. | | | |
| DDB_G0280379 | | | | DDB_G0280379 | | | |  | | | |
| DDB_G0280381 | | | | DDB_G0280381 | | | |  | | | |
| DDB_G0280395 | | | | DDB_G0280395 | | | |  | | | |
| DDB_G0280397 | | | | DDB_G0280397 | | | |  | | | |
| DDB_G0280521 | | | | DDB_G0280521 | | | | Polyprotein. | | | |
| DDB_G0280739 | | | | DDB_G0280739 | | | |  | | | |
| DDB_G0280741 | | | | DDB_G0280741 | | | |  | | | |
| DDB_G0280937 | | | | DDB_G0280937 | | | | Similar to C08B11.4.p. | | | |
| DDB_G0281297 | | | | DDB_G0281297 | | | | Pol. | | | |
| DDB_G0281299 | | | | DDB_G0281299 | | | |  | | | |
| DDB_G0281303 | | | | DDB_G0281303 | | | |  | | | |
| DDB_G0281319 | | | | DDB_G0281319 | | | |  | | | |
| DDB_G0281329 | | | | DDB_G0281329 | | | |  | | | |
| DDB_G0281333 | | | | DDB_G0281333 | | | |  | | | |
| DDB_G0281373 | | | | DDB_G0281373 | | | |  | | | |
| DDB_G0281375 | | | | DDB_G0281375 | | | |  | | | |
| DDB_G0281531 | | | | DDB_G0281531 | | | |  | | | |
| DDB_G0281535 | | | | DDB_G0281535 | | | | LTR-RETROTRANSPOSON SKIPPER, GAG (Fragment). | | | |
| DDB_G0281807 | | | | DDB_G0281807 | | | | Probable pirin-like protein. | | | |
| DDB_G0281813 | | | | DDB_G0281813 | | | |  | | | |
| DDB_G0281941 | | | | DDB_G0281941 | | | | Pol. | | | |
| DDB_G0282137 | | | | DDB_G0282137 | | | | Similar to Mus musculus (Mouse). adult male hypothalamus cDNA, RIKEN full-length enriched library, clone:A230106L22 product:hyp (Fragment). | | | |
| DDB_G0282753 | | | | DDB_G0282753 | | | | Non-LTR retroelement reverse transcriptase-like protein. | | | |
| DDB_G0282759 | | | | DDB_G0282759 | | | | Fructose-6-phosphate-2-kinase/fructose-2, 6-bisphosphatase. | | | |
| DDB_G0282779 | | | | DDB_G0282779 | | | |  | | | |
| DDB_G0282787 | | | | DDB_G0282787 | | | | Pol. | | | |
| DDB_G0282797 | | | | DDB_G0282797 | | | | Pol. | | | |
| DDB_G0282807 | | | | DDB_G0282807 | | | | Similar to Dictyostelium discoideum (Slime mold). Hypothetical 127.0 kDa protein. | | | |
| DDB_G0282809 | | | | DDB_G0282809 | | | | Similar to Dictyostelium discoideum (Slime mold). hypothetical 127.0 kDa protein. | | | |
| DDB_G0282905 | | | | DDB_G0282905 | | | |  | | | |
| DDB_G0283079 | | | | DDB_G0283079 | | | | Clone 9.10 TDD-3 and red repetitive elements, partial sequence. | | | |
| DDB_G0283143 | | | | DDB_G0283143 | | | | Similar to Dictyostelium discoideum (Slime mold). hypothetical 127.0 kDa protein. | | | |
| DDB_G0283145 | | | | DDB_G0283145 | | | |  | | | |
| DDB_G0283219 | | | | DDB_G0283219 | | | |  | | | |
| DDB_G0283221 | | | | DDB_G0283221 | | | |  | | | |
| DDB_G0283225 | | | | DDB_G0283225 | | | |  | | | |
| DDB_G0283227 | | | | DDB_G0283227 | | | |  | | | |
| DDB_G0283229 | | | | DDB_G0283229 | | | |  | | | |
| DDB_G0283233 | | | | DDB_G0283233 | | | |  | | | |
| DDB_G0283243 | | | | DDB_G0283243 | | | |  | | | |
| DDB_G0283249 | | | | DDB_G0283249 | | | |  | | | |
| DDB_G0283335 | | | | DDB_G0283335 | | | |  | | | |
| DDB_G0283379 | | | | DDB_G0283379 | | | | Similar to Mus musculus (Mouse). GABA-A receptor epsilon-like subunit. | | | |
| DDB_G0283521 | | | | DDB_G0283521 | | | |  | | | |
| DDB_G0283529 | | | | DDB_G0283529 | | | |  | | | |
| DDB_G0283593 | | | | DDB_G0283593 | | | |  | | | |
| DDB_G0283747 | | | | DDB_G0283747 | | | |  | | | |
| DDB_G0283869 | | | | DDB_G0283869 | | | |  | | | |
| DDB_G0283995 | | | | DDB_G0283995 | | | | Similar to Mus musculus (Mouse). tenascin C. | | | |
| DDB_G0283997 | | | | DDB_G0283997 | | | |  | | | |
| DDB_G0284143 | | | | DDB_G0284143 | | | |  | | | |
| DDB_G0284157 | | | | DDB_G0284157 | | | |  | | | |
| DDB_G0284179 | | | | DDB_G0284179 | | | |  | | | |
| DDB_G0284305 | | | | DDB_G0284305 | | | | Pol. | | | |
| DDB_G0284453 | | | | DDB_G0284453 | | | |  | | | |
| DDB_G0284459 | | | | DDB_G0284459 | | | |  | | | |
| DDB_G0284565 | | | | DDB_G0284565 | | | |  | | | |
| DDB_G0284591 | | | | DDB_G0284591 | | | | P18480 Transcription regulatory protein SNF5 (SWI/SNF complex component SNF5) (Transcription factor TYE4). | | | |
| DDB_G0285047 | | | | DDB_G0285047 | | | |  | | | |
| DDB_G0285075 | | | | DDB_G0285075 | | | |  | | | |
| DDB_G0285085 | | | | DDB_G0285085 | | | |  | | | |
| DDB_G0285089 | | | | DDB_G0285089 | | | | Pol. | | | |
| DDB_G0285097 | | | | DDB_G0285097 | | | |  | | | |
| DDB_G0285155 | | | | DDB_G0285155 | | | |  | | | |
| DDB_G0285237 | | | | DDB_G0285237 | | | |  | | | |
| DDB_G0285301 | | | | DDB_G0285301 | | | |  | | | |
| DDB_G0285305 | | | | DDB_G0285305 | | | | Pol. | | | |
| DDB_G0285309 | | | | DDB_G0285309 | | | |  | | | |
| DDB_G0285315 | | | | DDB_G0285315 | | | | Pol. | | | |
| DDB_G0285317 | | | | DDB_G0285317 | | | | Multifunctional protein (Fragment). | | | |
| DDB_G0285743 | | | | DDB_G0285743 | | | |  | | | |
| DDB_G0285745 | | | | DDB_G0285745 | | | | Similar to Mus musculus (Mouse). tenascin X. | | | |
| DDB_G0285747 | | | | DDB_G0285747 | | | |  | | | |
| DDB_G0285751 | | | | DDB_G0285751 | | | |  | | | |
| DDB_G0285753 | | | | DDB_G0285753 | | | | Similar to Mus musculus (Mouse). tenascin X. | | | |
| DDB_G0285761 | | | | DDB_G0285761 | | | |  | | | |
| DDB_G0285771 | | | | DDB_G0285771 | | | |  | | | |
| DDB_G0285835 | | | | DDB_G0285835 | | | | Pol. | | | |
| DDB_G0285841 | | | | DDB_G0285841 | | | | Pol. | | | |
| DDB_G0285975 | | | | DDB_G0285975 | | | | Mast cell surface antigen-1. | | | |
| DDB_G0285977 | | | | DDB_G0285977 | | | |  | | | |
| DDB_G0285983 | | | | DDB_G0285983 | | | |  | | | |
| DDB_G0285987 | | | | DDB_G0285987 | | | |  | | | |
| DDB_G0286175 | | | | DDB_G0286175 | | | |  | | | |
| DDB_G0286325 | | | | DDB_G0286325 | | | |  | | | |
| DDB_G0286327 | | | | DDB_G0286327 | | | |  | | | |
| DDB_G0286329 | | | | DDB_G0286329 | | | |  | | | |
| DDB_G0286471 | | | | DDB_G0286471 | | | |  | | | |
| DDB_G0286497 | | | | DDB_G0286497 | | | | Pol. | | | |
| DDB_G0286503 | | | | DDB_G0286503 | | | | Polyprotein. | | | |
| DDB_G0286527 | | | | DDB_G0286527 | | | | putative SAM dependent methyltransferase | | | |
| DDB_G0286529 | | | | DDB_G0286529 | | | |  | | | |
| DDB_G0286531 | | | | DDB_G0286531 | | | | Group-specific antigen. | | | |
| DDB_G0286547 | | | | DDB_G0286547 | | | | Multifunctional protein (Fragment). | | | |
| DDB_G0286813 | | | | DDB_G0286813 | | | | Similar to Dictyostelium discoideum (Slime mold). hypothetical 127.0 kDa protein. | | | |
| DDB_G0286861 | | | | DDB_G0286861 | | | | Similar to Rhizobium loti (Mesorhizobium loti). short-chain oxidoreductase. | | | |
| DDB_G0286973 | | | | DDB_G0286973 | | | |  | | | |
| DDB_G0286975 | | | | DDB_G0286975 | | | |  | | | |
| DDB_G0286977 | | | | DDB_G0286977 | | | |  | | | |
| DDB_G0286979 | | | | DDB_G0286979 | | | |  | | | |
| DDB_G0286981 | | | | DDB_G0286981 | | | |  | | | |
| DDB_G0287117 | | | | DDB_G0287117 | | | | Putative transposase. | | | |
| DDB_G0287121 | | | | DDB_G0287121 | | | |  | | | |
| DDB_G0287187 | | | | DDB_G0287187 | | | |  | | | |
| DDB_G0287195 | | | | DDB_G0287195 | | | |  | | | |
| DDB_G0287265 | | | | DDB_G0287265 | | | |  | | | |
| DDB_G0287423 | | | | DDB_G0287423 | | | |  | | | |
| DDB_G0287427 | | | | DDB_G0287427 | | | |  | | | |
| DDB_G0287431 | | | | DDB_G0287431 | | | |  | | | |
| DDB_G0287453 | | | | DDB_G0287453 | | | |  | | | |
| DDB_G0287581 | | | | DDB_G0287581 | | | |  | | | |
| DDB_G0287651 | | | | DDB_G0287651 | | | | Similar to G-protein-coupled receptor at plasma membrane; interactions in two-hybrid system with Gpa2p; Gpr1p. | | | |
| DDB_G0287665 | | | | DDB_G0287665 | | | |  | | | |
| DDB_G0287667 | | | | DDB_G0287667 | | | | Similar to Gallus gallus (Chicken). 190 kDa tenascin. | | | |
| DDB_G0287671 | | | | DDB_G0287671 | | | |  | | | |
| DDB_G0287877 | | | | DDB_G0287877 | | | |  | | | |
| DDB_G0287885 | | | | DDB_G0287885 | | | |  | | | |
| DDB_G0287915 | | | | DDB_G0287915 | | | |  | | | |
| DDB_G0287923 | | | | DDB_G0287923 | | | |  | | | |
| DDB_G0287943 | | | | DDB_G0287943 | | | |  | | | |
| DDB_G0288317 | | | | DDB_G0288317 | | | | Similar to Dictyostelium discoideum (Slime mold). Hypothetical 127.0 kDa protein. | | | |
| DDB_G0288455 | | | | DDB_G0288455 | | | |  | | | |
| DDB_G0288607 | | | | DDB_G0288607 | | | |  | | | |
| DDB_G0288865 | | | | DDB_G0288865 | | | |  | | | |
| DDB_G0288867 | | | | DDB_G0288867 | | | |  | | | |
| DDB_G0289049 | | | | DDB_G0289049 | | | | Pol. | | | |
| DDB_G0289107 | | | | DDB_G0289107 | | | | Pol. | | | |
| DDB_G0289111 | | | | DDB_G0289111 | | | | Probable pirin-like protein. | | | |
| DDB_G0289113 | | | | DDB_G0289113 | | | | Similar to Dictyostelium discoideum (Slime mold). Hypothetical 127.0 kDa protein. | | | |
| DDB_G0289261 | | | | DDB_G0289261 | | | |  | | | |
| DDB_G0289433 | | | | DDB_G0289433 | | | |  | | | |
| DDB_G0289529 | | | | DDB_G0289529 | | | | Q9VYS3 Regulator of nonsense transcripts 1 homolog. | | | |
| DDB_G0289531 | | | | DDB_G0289531 | | | |  | | | |
| DDB_G0289561 | | | | DDB_G0289561 | | | |  | | | |
| DDB_G0289579 | | | | DDB_G0289579 | | | |  | | | |
| DDB_G0289635 | | | | DDB_G0289635 | | | |  | | | |
| DDB_G0289637 | | | | DDB_G0289637 | | | | Q94481 Protein cigB (Fragment). | | | |
| DDB_G0289641 | | | | DDB_G0289641 | | | | Q94481 Protein cigB (Fragment). | | | |
| DDB_G0289647 | | | | DDB_G0289647 | | | |  | | | |
| DDB_G0289795 | | | | DDB_G0289795 | | | | Q94481 Protein cigB (Fragment). | | | |
| DDB_G0289797 | | | | DDB_G0289797 | | | |  | | | |
| DDB_G0289799 | | | | DDB_G0289799 | | | | Q94481 Protein cigB (Fragment). | | | |
| DDB_G0289801 | | | | DDB_G0289801 | | | | Q94481 Protein cigB (Fragment). | | | |
| DDB_G0289803 | | | | DDB_G0289803 | | | | Q94481 Protein cigB (Fragment). | | | |
| DDB_G0289805 | | | | DDB_G0289805 | | | | Pol. | | | |
| DDB_G0289853 | | | | DDB_G0289853 | | | |  | | | |
| DDB_G0289855 | | | | DDB_G0289855 | | | | Q94481 Protein cigB (Fragment). | | | |
| DDB_G0289865 | | | | DDB_G0289865 | | | |  | | | |
| DDB_G0289869 | | | | DDB_G0289869 | | | |  | | | |
| DDB_G0289977 | | | | DDB_G0289977 | | | |  | | | |
| DDB_G0289983 | | | | DDB_G0289983 | | | |  | | | |
| DDB_G0290051 | | | | DDB_G0290051 | | | | Similar to Dictyostelium discoideum (Slime mold). CIGB protein. | | | |
| DDB_G0290059 | | | | DDB_G0290059 | | | | Similar to Dictyostelium discoideum (Slime mold). hypothetical 127.0 kDa protein. | | | |
| DDB_G0290061 | | | | DDB_G0290061 | | | | Similar to Dictyostelium discoideum (Slime mold). hypothetical 127.0 kDa protein. | | | |
| DDB_G0290065 | | | | DDB_G0290065 | | | | Similar to Dictyostelium discoideum (Slime mold). CIGB protein. | | | |
| DDB_G0290095 | | | | DDB_G0290095 | | | |  | | | |
| DDB_G0290109 | | | | DDB_G0290109 | | | | Q94481 Protein cigB (Fragment). | | | |
| DDB_G0290151 | | | | DDB_G0290151 | | | |  | | | |
| DDB_G0290241 | | | | DDB_G0290241 | | | | Pol. | | | |
| DDB_G0290249 | | | | DDB_G0290249 | | | |  | | | |
| DDB_G0290255 | | | | DDB_G0290255 | | | |  | | | |
| DDB_G0290361 | | | | DDB_G0290361 | | | |  | | | |
| DDB_G0290367 | | | | DDB_G0290367 | | | |  | | | |
| DDB_G0290371 | | | | DDB_G0290371 | | | |  | | | |
| DDB_G0290459 | | | | DDB_G0290459 | | | |  | | | |
| DDB_G0290661 | | | | DDB_G0290661 | | | |  | | | |
| DDB_G0290679 | | | | DDB_G0290679 | | | | Similar to Dictyostelium discoideum (Slime mold). CIGB protein. | | | |
| DDB_G0290681 | | | | DDB_G0290681 | | | | Q94481 Protein cigB (Fragment). | | | |
| DDB_G0290683 | | | | DDB_G0290683 | | | |  | | | |
| DDB_G0290715 | | | | DDB_G0290715 | | | |  | | | |
| DDB_G0290749 | | | | DDB_G0290749 | | | | Pol. | | | |
| DDB_G0290763 | | | | DDB_G0290763 | | | |  | | | |
| DDB_G0290777 | | | | DDB_G0290777 | | | |  | | | |
| DDB_G0290837 | | | | DDB_G0290837 | | | |  | | | |
| DDB_G0290895 | | | | DDB_G0290895 | | | | Q94481 Protein cigB (Fragment). | | | |
| DDB_G0290897 | | | | DDB_G0290897 | | | |  | | | |
| DDB_G0291051 | | | | DDB_G0291051 | | | |  | | | |
| DDB_G0291053 | | | | DDB_G0291053 | | | |  | | | |
| DDB_G0291065 | | | | DDB_G0291065 | | | |  | | | |
| DDB_G0291067 | | | | DDB_G0291067 | | | | Pol. | | | |
| DDB_G0291069 | | | | DDB_G0291069 | | | | Pol. | | | |
| DDB_G0291111 | | | | DDB_G0291111 | | | |  | | | |
| DDB_G0291119 | | | | DDB_G0291119 | | | |  | | | |
| DDB_G0291189 | | | | DDB_G0291189 | | | |  | | | |
| DDB_G0291654 | | | | DDB_G0291654 | | | |  | | | |
| DDB_G0291674 | | | | DDB_G0291674 | | | | Similar to Lactococcus lactis (Subsp. lactis) (Streptococcus lactis). Oxidoreductase. | | | |
| DDB_G0291676 | | | | DDB_G0291676 | | | | Similar to Lactococcus lactis (Subsp. lactis) (Streptococcus lactis). Oxidoreductase. | | | |
| DDB_G0291678 | | | | DDB_G0291678 | | | |  | | | |
| DDB_G0291680 | | | | DDB_G0291680 | | | |  | | | |
| DDB_G0291686 | | | | DDB_G0291686 | | | | Pol. | | | |
| DDB_G0291688 | | | | DDB_G0291688 | | | |  | | | |
| DDB_G0291772 | | | | DDB_G0291772 | | | |  | | | |
| DDB_G0291958 | | | | DDB_G0291958 | | | | Pol. | | | |
| DDB_G0291964 | | | | DDB_G0291964 | | | | Pol. | | | |
| DDB_G0292172 | | | | DDB_G0292172 | | | | Pol. | | | |
| DDB_G0292194 | | | | DDB_G0292194 | | | |  | | | |
| DDB_G0292198 | | | | DDB_G0292198 | | | | Pol. | | | |
| DDB_G0292260 | | | | DDB_G0292260 | | | | Multifunctional protein (Fragment). | | | |
| DDB_G0292364 | | | | DDB_G0292364 | | | |  | | | |
| DDB_G0292370 | | | | DDB_G0292370 | | | | Pol. | | | |
| DDB_G0292372 | | | | DDB_G0292372 | | | | Multifunctional protein (Fragment). | | | |
| DDB_G0292540 | | | | DDB_G0292540 | | | | Pol. | | | |
| DDB_G0292542 | | | | DDB_G0292542 | | | |  | | | |
| DDB_G0292546 | | | | DDB_G0292546 | | | |  | | | |
| DDB_G0292712 | | | | DDB_G0292712 | | | | Pol. | | | |
| DDB_G0292728 | | | | DDB_G0292728 | | | |  | | | |
| DDB_G0292796 | | | | DDB_G0292796 | | | | Pol. | | | |
| DDB_G0292800 | | | | DDB_G0292800 | | | |  | | | |
| DDB_G0293152 | | | | DDB_G0293152 | | | | Pol. | | | |
| DDB_G0293382 | | | | DDB_G0293382 | | | |  | | | |
| DDB_G0293408 | | | | DDB_G0293408 | | | |  | | | |
| DDB_G0293488 | | | | DDB_G0293488 | | | |  | | | |
| DDB_G0293490 | | | | DDB_G0293490 | | | | Pol. | | | |
| DDB_G0292966 | | | | DDB_G0292966 | | | |  | | | |
| DDB_G0292990 | | | | DDB_G0292990 | | | |  | | | |
| DDB_G0293160 | | | | DDB_G0293160 | | | | Pol. | | | |
| DDB_G0293174 | | | | DDB_G0293174 | | | |  | | | |
| DDB_G0293176 | | | | DDB_G0293176 | | | |  | | | |
| DDB_G0293182 | | | | DDB_G0293182 | | | |  | | | |
| DDB_G0293282 | | | | DDB_G0293282 | | | | Similar to Boophilus microplus (Cattle tick). notch-like protein. | | | |
| DDB_G0293290 | | | | DDB_G0293290 | | | |  | | | |
| DDB_G0293338 | | | | DDB_G0293338 | | | |  | | | |
| DDB_G0293446 | | | | DDB_G0293446 | | | | Similar to Dictyostelium discoideum (Slime mold). hypothetical 127.0 kDa protein. | | | |
| DDB_G0293518 | | | | DDB_G0293518 | | | |  | | | |
| DDB_G0293714 | | | | DDB_G0293714 | | | |  | | | |
| DDB_G0293720 | | | | DDB_G0293720 | | | | Similar to Dictyostelium discoideum (Slime mold). hypothetical 127.0 kDa protein. | | | |
| DDB_G0293736 | | | | DDB_G0293736 | | | | Pol. | | | |
| DDB_G0293782 | | | | DDB_G0293782 | | | |  | | | |
| DDB_G0293818 | | | | DDB_G0293818 | | | | Pol. | | | |
| DDB_G0293822 | | | | DDB_G0293822 | | | |  | | | |
| DDB_G0293974 | | | | DDB_G0293974 | | | |  | | | |
| DDB_G0294004 | | | | DDB_G0294004 | | | |  | | | |
| DDB_G0294008 | | | | DDB_G0294008 | | | |  | | | |
| DDB_G0283153 | | | | cbpD1 | | | | calcium-binding protein | | | |
| DDB_G0291752 | | | | smc1 | | | | structural maintenance of chromosome protein | | | |
| DDB_G0288427 | | | | cenA | | | | centrin | | | |
| DDB_G0276101 | | | | smc3 | | | | structural maintenance of chromosome protein | | | |
| DDB_G0290257 | | | | GP138A | | | | cell surface glycoprotein gp138 | | | |
| DDB_G0268622 | | | | rac1B | | | | Rho GTPase | | | |
| DDB_G0284467 | | | | fut1 | | | | alpha-3/4-fucosyltransferase, glycosyltransferase | | | |
| DDB_G0287593 | | | | abcC6 | | | | ABC transporter C family protein | | | |
| DDB_G0287589 | | | | abcC14 | | | | ABC transporter C family protein | | | |
| DDB_G0277719 | | | | DG1054 | | | | EGF-like domain-containing protein | | | |
| DDB_G0275453 | | | | abcA11 | | | | ABC transporter A family protein | | | |
| DDB_G0274121 | | | | abcA4 | | | | ABC transporter A family protein | | | |
| DDB_G0284839 | | | | ndrC | | | | NDR family protein kinase, protein kinase, AGC group, putative protein serine/threonine kinase | | | |
| DDB_G0291185 | | | | H3v1 | | | | histone H3 domain-containing protein | | | |
| DDB_G0290665 | | | | gtaX | | | | GATA zinc finger domain-containing protein 24, putative GATA-binding transcription factor | | | |
| DDB_G0271948 | | | | DDB_G0271948 | | | | YEATS family protein | | | |
| DDB_G0281803 | | | | DDB_G0281803 | | | | SAP DNA-binding domain-containing protein | | | |
| DDB_G0273333 | | | | irlB-1 | | | | IRE family protein kinase, putative protein serine/threonine kinase | | | |
| DDB_G0271696 | | | | prtA | | | | proteosomal alpha-subunit M3 | | | |
| DDB_G0281571 | | | | comE | | | | FNIP repeat-containing protein, ankyrin repeat-containing protein | | | |
| DDB_G0285423 | | | | DG2044 | | | | unknown | | | |
| DDB_G0294358 | | | | DDB_G0294358 | | | | LTR-RETROTRANSPOSON SKIPPER, GAG (Fragment). | | | |
| DDB_G0294360 | | | | DDB_G0294360 | | | | LTR-RETROTRANSPOSON SKIPPER, GAG (Fragment). | | | |
| DDB_G0294372 | | | | DDB_G0294372 | | | | LTR-RETROTRANSPOSON SKIPPER, GAG (Fragment). | | | |
| DDB_G0286411 | | | | bzpN | | | | putative basic-leucine zipper (bZIP) transcription factor | | | |
| DDB_G0290171 | | | | bzpS | | | | putative basic-leucine zipper (bZIP) transcription factor | | | |
| DDB_G0290303 | | | | bzpP | | | | putative basic-leucine zipper (bZIP) transcription factor | | | |
| DDB_G0290165 | | | | bzpR | | | | putative basic-leucine zipper (bZIP) transcription factor | | | |
| DDB_G0282047 | | | | bzpM | | | | putative basic-leucine zipper (bZIP) transcription factor | | | |
| DDB_G0282049 | | | | bzpK | | | | putative basic-leucine zipper (bZIP) transcription factor | | | |
| nfyC-1 | | | | nfyC-1 | | | | there is a second copy of this gene%2C %3Ca href%3D%22gene_page.pl?primary_id%3DDDB_G0273545%22%3E%3Ci%3EnfyC-2%3C%2Fi%3E%3C%2Fa%3E | | | |
| nfyA | | | | nfyA | | | | putative ortholog of the A subunit of the heterotrimeric transcription factor NFY%2C composed of NfyA%2C NfyB%2C and NfyC | | | |
| DDB_G0291121 | | | | cinB | | | | esterase/lipase/thioesterase domain-containing protein, vegetative specific protein H5 | | | |
| DDB_G0287591 | | | | DDB_G0287591 | | | | FNIP repeat-containing protein | | | |
| DDB_G0287649 | | | | pldY | | | | phospholipase D3 | | | |
| DDB_G0273067 | | | | dscD-1 | | | | discoidin I, D chain | | | |
| DDB_G0292812 | | | | mipA | | | | putative transmembrane protein | | | |
| DDB_G0268626 | | | | DDB_G0268626 | | | | EGF-like domain-containing protein | | | |
| DDB_G0286125 | | | | pkgC | | | | protein kinase 3, protein kinase, AGC group, protein serine/threonine kinase | | | |
| DDB_G0286549 | | | | flpA | | | | FKBP-like protein, FKBP-type peptidylprolyl cis-trans isomerase (PPIase) | | | |
| DDB_G0277853 | | | | ecmA | | | | extracellular matrix protein ST430 | | | |
| DDB_G0290377 | | | | agnB | | | | argonaut-like protein | | | |
| DDB_G0271870 | | | | agnC | | | | argonaut-like protein | | | |
| DDB_G0273051 | | | | drnA-1 | | | | dicer-like protein, putative RNase III | | | |
| DDB_G0289553 | | | | act1 | | | | actin | | | |
| DDB_G0280545 | | | | act7 | | | | actin | | | |
| DDB_G0274727 | | | | act19 | | | | actin | | | |
| DDB_G0289663 | | | | act5 | | | | actin | | | |
| DDB_G0289005 | | | | act4 | | | | actin | | | |
| DDB_G0288879 | | | | act11 | | | | actin | | | |
| DDB_G0274285 | | | | act20 | | | | actin | | | |
| DDB_G0274561 | | | | act21 | | | | actin | | | |
| DDB_G0274599 | | | | act13 | | | | actin | | | |
| DDB_G0274137 | | | | act14 | | | | actin | | | |
| DDB_G0274601 | | | | act9 | | | | actin | | | |
| DDB_G0289487 | | | | act3 | | | | actin | | | |
| DDB_G0289489 | | | | act18 | | | | actin | | | |
| DDB_G0275023 | | | | act22 | | | | actin | | | |
| DDB_G0268744 | | | | act23 | | | | actin | | | |
| DDB_G0289505 | | | | act24 | | | | actin | | | |
| DDB_G0289507 | | | | act25 | | | | actin | | | |
| DDB_G0269902 | | | | act26 | | | | actin | | | |
| DDB_G0286839 | | | | gtaW | | | | GATA zinc finger domain-containing protein 23, putative GATA-binding transcription factor | | | |
| DDB_G0279331 | | | | gtaR | | | | GATA zinc finger domain-containing protein 18, putative GATA-binding transcription factor | | | |
| DDB_G0280853 | | | | gtaU | | | | GATA zinc finger domain-containing protein 21, putative GATA-binding transcription factor | | | |
| DDB_G0289677 | | | | DDB_G0289677 | | | | homeodomain (HOX) containing protein, putative homeobox transcription factor | | | |
| DDB_G0273127 | | | | DDB_G0273127 | | | | homeodomain (HOX) containing protein, putative homeobox transcription factor | | | |
| DDB_G0277505 | | | | DDB_G0277505 | | | | homeodomain (HOX) containing protein, putative homeobox transcription factor | | | |
| DDB_G0282835 | | | | srfB | | | | putative MADS-box transcription factor | | | |
| DDB_G0287637 | | | | mybD | | | | myb domain-containing protein | | | |
| DDB_G0278963 | | | | DDB_G0278963 | | | |  | | | |
| DDB_G0286823 | | | | DDB_G0286823 | | | |  | | | |
| DDB_G0289315 | | | | DDB_G0289315 | | | |  | | | |
| DDB_G0290115 | | | | DDB_G0290115 | | | |  | | | |
| DDB_G0278317 | | | | mybF | | | | myb domain-containing protein | | | |
| DDB_G0270054 | | | | DDB_G0270054 | | | | ssDNA-binding transcriptional regulator | | | |
| DDB_G0272688 | | | | DDB_G0272688 | | | | NmrA-like protein | | | |
| DDB_G0270590 | | | | DDB_G0270590 | | | | fungal transcriptional regulatory protein, N-terminal domain-containing protein, putative zinc cluster transcription factor | | | |
| DDB_G0270666 | | | | gdt2 | | | | GDT family protein kinase, protein kinase, TKL group, putative transmembrane protein, tyrosine kinase-like protein | | | |
| DDB_G0270550 | | | | gdt4 | | | | GDT family protein kinase, protein kinase, TKL group, putative transmembrane protein, tyrosine kinase-like protein | | | |
| DDB_G0285895 | | | | gdt5 | | | | putative transmembrane protein | | | |
| DDB_G0292314 | | | | gdt7 | | | | putative transmembrane protein | | | |
| DDB_G0270668 | | | | gdt8 | | | | GDT family protein kinase, protein kinase, TKL group, putative transmembrane protein, tyrosine kinase-like protein | | | |
| DDB_G0289187 | | | | H2Av1 | | | | histone H2A domain-containing protein | | | |
| DDB_G0289193 | | | | H2Av2 | | | | histone H2A domain-containing protein | | | |
| DDB_G0282987 | | | | hcpB | | | | HP1-like protein, chromo (CHRomatin Organization MOdifier) domain-containing protein, heterochromatin protein | | | |
| DDB_G0283023 | | | | hcpA | | | | HP1-like protein, chromo (CHRomatin Organization MOdifier) domain-containing protein, heterochromatin protein | | | |
| DDB_G0280439 | | | | cpiB | | | | cystatin A2, cysteine protease inhibitor | | | |
| DDB_G0283197 | | | | DDB_G0283197 | | | | C2H2-type zinc finger-containing protein | | | |
| DDB_G0281095 | | | | DDB_G0281095 | | | | SAP DNA-binding domain-containing protein | | | |
| DDB_G0278625 | | | | DDB_G0278625 | | | | SAP DNA-binding domain-containing protein | | | |
| DDB_G0280785 | | | | DDB_G0280785 | | | | SAP DNA-binding domain-containing protein | | | |
| DDB_G0280809 | | | | DDB_G0280809 | | | | SAP DNA-binding domain-containing protein | | | |
| DDB_G0280811 | | | | DDB_G0280811 | | | | unknown | | | |
| DDB_G0274997 | | | | DDB_G0274997 | | | | SAP DNA-binding domain-containing protein | | | |
| DDB_G0271118 | | | | DDB_G0271118 | | | | bromodomain-containing protein | | | |
| DDB_G0276479 | | | | ctnC | | | | countin3 | | | |
| DDB_G0286841 | | | | DDB_G0286841 | | | | MAST family protein kinase, protein kinase, AGC group, putative protein serine/threonine kinase | | | |
| DDB_G0278469 | | | | DDB_G0278469 | | | | SMAD/FHA domain-containing protein, bromodomain-containing protein | | | |
| DDB_G0273393 | | | | DDB_G0273393 | | | | MYND-type zinc finger-containing protein, SET domain-containing protein | | | |
| DDB_G0273253 | | | | DDB_G0273253 | | | | SET domain-containing protein | | | |
| DDB_G0283443 | | | | DDB_G0283443 | | | | SET domain-containing protein | | | |
| DDB_G0280557 | | | | DDB_G0280557 | | | | protein kinase, CMGC group, putative protein serine/threonine kinase | | | |
| DDB_G0280855 | | | | DDB_G0280855 | | | | protein kinase, CMGC group, putative protein serine/threonine kinase | | | |
| DDB_G0268656 | | | | DDB_G0268656 | | | | ULK-related protein kinase, putative protein serine/threonine kinase | | | |
| DDB_G0283301 | | | | DDB_G0283301 | | | | putative protein kinase | | | |
| DDB_G0280559 | | | | DDB_G0280559 | | | | kinase motif-containing (KMC) protein | | | |
| DDB_G0291664 | | | | DDB_G0291664 | | | | ankyrin repeat-containing protein, putative protein kinase | | | |
| DDB_G0289533 | | | | act27 | | | | actin | | | |
| DDB_G0289511 | | | | act28 | | | | actin, poly P kinase component, polyphosphate kinase component | | | |
| DDB_G0286893 | | | | act29 | | | | actin | | | |
| DDB_G0273171 | | | | irlF-1 | | | | IRE family protein kinase, putative protein serine/threonine kinase | | | |
| DDB_G0289555 | | | | arkA | | | | ARK family protein kinase, GRAM domain-containing protein, PKC domain-containing protein, PE/DAG binding, ankyrin repeat-containing protein, protein kinase, TKL group, tyrosine kinase-like protein | | | |
| DDB_G0288015 | | | | arrH | | | | ADP-ribosylation factor-related, ARF-related | | | |
| DDB_G0282457 | | | | arrA | | | | ADP-ribosylation factor-related, ARF-related | | | |
| DDB_G0280479 | | | | arrE | | | | ADP-ribosylation factor-related, ARF-related | | | |
| DDB_G0280633 | | | | arrK | | | | ADP-ribosylation factor-related, ARF-related | | | |
| DDB_G0286627 | | | | DDB_G0286627 | | | | protein kinase, STE group, putative protein serine/threonine kinase | | | |
| DDB_G0289435 | | | | arrF | | | | ADP-ribosylation factor-related, ARF-related | | | |
| DDB_G0288803 | | | | irlE | | | | IRE family protein kinase, putative protein serine/threonine kinase | | | |
| DDB_G0274445 | | | | arrB | | | | ADP-ribosylation factor-related, ARF-related | | | |
| DDB_G0276295 | | | | rabW | | | | Rab GTPase | | | |
| DDB_G0270680 | | | | samkD | | | | SAM domain-containing protein, SAMK family protein kinase, putative protein serine/threonine kinase | | | |
| DDB_G0275955 | | | | rabH | | | | Rab GTPase | | | |
| DDB_G0273121 | | | | pakH-1 | | | | PAKL subfamily protein kinase, STE20 family protein kinase, protein kinase, STE group, putative protein serine/threonine kinase | | | |
| DDB_G0290783 | | | | rabG2 | | | | Rab GTPase | | | |
| DDB_G0268910 | | | | rabT1 | | | | Rab GTPase | | | |
| DDB_G0269262 | | | | rabT2 | | | | Rab GTPase | | | |
| DDB_G0272905 | | | | rabF1-1 | | | | Rab GTPase | | | |
| DDB_G0275969 | | | | rab1E | | | | Rab GTPase | | | |
| DDB_G0282203 | | | | rabY | | | | Rab GTPase | | | |
| DDB_G0292550 | | | | DDB_G0292550 | | | | CDK family protein kinase, protein kinase, CMGC group, putative protein serine/threonine kinase | | | |
| DDB_G0274943 | | | | ranB | | | | GTP-binding nuclear protein Ran | | | |
| cdk9-1 | | | | cdk9-1 | | | | similar to the cell division cycle 2-related protein kinase 7 (CRK7) and other cell division cycle 2-like protein kinases%3B there is a second copy of this gene%2C %3Ca href%3D%22gene_page.pl?primary_id%3DDDB_G0273721%22%3E%3Ci%3Ecdk9-2%3C%2Fi%3E%3C%2Fa%3E | | | |
| DDB_G0268078 | | | | DDB_G0268078 | | | | RCK family protein kinase, protein kinase, CMGC group, putative protein serine/threonine kinase | | | |
| DDB_G0270126 | | | | rasY | | | | Ras GTPase | | | |
| DDB_G0270140 | | | | rasZ | | | | Ras GTPase | | | |
| DDB_G0270736 | | | | rasV | | | | Ras GTPase | | | |
| DDB_G0270138 | | | | rasU | | | | Ras GTPase | | | |
| DDB_G0270122 | | | | rasW | | | | Ras GTPase | | | |
| DDB_G0289543 | | | | ndrD | | | | NDR family protein kinase, protein kinase, AGC group, putative protein serine/threonine kinase | | | |
| DDB_G0275949 | | | | DDB_G0275949 | | | |  | | | |
| DDB_G0290155 | | | | DDB_G0290155 | | | |  | | | |
| DDB_G0277809 | | | | cfaA | | | | counting factor associated protein | | | |
| DDB_G0269026 | | | | abcG23 | | | | ABC transporter G family protein | | | |
| DDB_G0280643 | | | | DDB_G0280643 | | | | protein kinase, CMGC group, putative protein serine/threonine kinase | | | |
| DDB_G0278101 | | | | cnrO | | | | C2 domain-containing protein, putative cell number regulator | | | |
| DDB_G0284859 | | | | samkB | | | | SAM domain-containing protein, SAMK family protein kinase, putative protein serine/threonine kinase | | | |
| DDB_G0294376 | | | | fcpA | | | | putative CTD phosphatase | | | |
| DDB_G0293888 | | | | omt12 | | | | O-methyltransferase family 2 protein | | | |
| DDB_G0275499 | | | | omt5 | | | | O-methyltransferase family 3 protein | | | |
| DDB_G0273213 | | | | aslA-1 | | | | putative acetyl-CoA synthetase | | | |
| DDB_G0274299 | | | | DDB_G0274299 | | | | unknown | | | |
| DDB_G0275255 | | | | DDB_G0275255 | | | | unknown | | | |
| DDB_G0292218 | | | | st15 | | | | cellulose-binding domain-containing protein, putative beta-1, 4-endoglucanase | | | |
| DDB_G0291870 | | | | rpl13 | | | | S60 ribosomal protein L13 | | | |
| DDB_G0277975 | | | | rpl14 | | | | S60 ribosomal protein L14 | | | |
| DDB_G0272893 | | | | rpl15-1 | | | | S60 ribosomal protein L15 | | | |
| DDB_G0289709 | | | | drkB | | | | DRK subfamily protein kinase, protein kinase, TKL group, tyrosine kinase-like protein | | | |
| DDB_G0276187 | | | | DDB_G0276187 | | | | CZAK family protein kinase, NZAK subfamily protein kinase, protein kinase, STE group, protein kinase, TKL group, tyrosine kinase-like protein | | | |
| DDB_G0287853 | | | | mkcC | | | | MKC subfamily protein kinase, STE20 family protein kinase, protein kinase, STE group, putative protein serine/threonine kinase | | | |
| DDB_G0290407 | | | | rabO | | | | Rab GTPase | | | |
| DDB_G0280437 | | | | DDB_G0280437 | | | | Ras GTPase | | | |
| DDB_G0278389 | | | | rsmF | | | | small GTPase | | | |
| DDB_G0278403 | | | | rsmH | | | | small GTPase | | | |
| DDB_G0279305 | | | | rsmJ | | | | small GTPase | | | |
| DDB_G0270678 | | | | samkC | | | | SAM domain-containing protein, SAMK family protein kinase | | | |
| DDB_G0293786 | | | | DDB_G0293786 | | | | Ras GTPase | | | |
| DDB_G0273099 | | | | rsmE-1 | | | | small GTPase | | | |
| DDB_G0289103 | | | | racM | | | | Rho GTPase | | | |
| DDB_G0290829 | | | | rabM | | | | Rab GTPase | | | |
| DDB_G0290789 | | | | rabN1 | | | | Rab GTPase | | | |
| DDB_G0290833 | | | | rabK1 | | | | Rab GTPase | | | |
| DDB_G0290793 | | | | rabN2 | | | | Rab GTPase | | | |
| DDB_G0290875 | | | | rabP | | | | Rab GTPase | | | |
| DDB_G0291293 | | | | rabU | | | | Rab GTPase | | | |
| DDB_G0271980 | | | | rabR | | | | Rab GTPase | | | |
| DDB_G0277791 | | | | racO | | | | Rho GTPase | | | |
| DDB_G0278009 | | | | racN | | | | Rho GTPase | | | |
| DDB_G0277989 | | | | DDB_G0277989 | | | | putative protein kinase | | | |
| DDB_G0278217 | | | | rsmK | | | | small GTPase | | | |
| DDB_G0278449 | | | | rsmC | | | | small GTPase | | | |
| DDB_G0282899 | | | | rabV | | | | Rab GTPase | | | |
| DDB_G0275327 | | | | rabX | | | | Rab GTPase | | | |
| DDB_G0282651 | | | | rsmN | | | | small GTPase | | | |
| DDB_G0282653 | | | | DDB_G0282653 | | | | Rab GTPase | | | |
| DDB_G0272797 | | | | DDB_G0272797 | | | | CDK family protein kinase, protein kinase, CMGC group, putative protein serine/threonine kinase | | | |
| DDB_G0268740 | | | | DDB_G0268740 | | | | carboxylic ester hydrolase | | | |
| DDB_G0275077 | | | | pks17 | | | | beta-ketoacyl synthase family protein, putative fatty acid synthase | | | |
| DDB_G0283931 | | | | pks23 | | | | beta-ketoacyl synthase family protein, putative polyketide synthase | | | |
| DDB_G0284001 | | | | pks22 | | | | beta-ketoacyl synthase family protein, putative polyketide synthase | | | |
| DDB_G0293902 | | | | pks44 | | | | beta-ketoacyl synthase family protein, putative polyketide synthase | | | |
| DDB_G0293912 | | | | pks45 | | | | beta-ketoacyl synthase family protein, putative polyketide synthase | | | |
| DDB_G0287095 | | | | pks25 | | | | beta-ketoacyl synthase family protein, putative polyketide synthase | | | |
| DDB_G0287119 | | | | pks24 | | | | beta-ketoacyl synthase family protein, putative polyketide synthase | | | |
| DDB_G0272981 | | | | pks13 | | | | beta-ketoacyl synthase family protein, putative polyketide synthase | | | |
| DDB_G0273387 | | | | DDB_G0273387 | | | | unknown | | | |
| DDB_G0269940 | | | | osbC | | | | oxysterol binding family protein, member 3 | | | |
| DDB_G0281179 | | | | clkA | | | | CLK family protein kinase, protein kinase, CMGC group, putative protein serine/threonine kinase | | | |
| DDB_G0271688 | | | | grlO | | | | G-protein-coupled receptor (GPCR) family 3 protein 14 | | | |
| DDB_G0268802 | | | | gpaJ | | | | G-protein subunit alpha 10 | | | |
| DDB_G0288101 | | | | rpl22 | | | | S60 ribosomal protein L22 | | | |
| DDB_G0282107 | | | | DDB_G0282107 | | | | putative phospholipid transfer protein | | | |
| DDB_G0284551 | | | | fut4 | | | | alpha-3/4-fucosyltransferase, glycosyltransferase | | | |
| DDB_G0273191 | | | | rps30-1 | | | | 40S ribosomal protein S30 | | | |
| DDB_G0270294 | | | | DDB_G0270294 | | | | cellulose-binding domain-containing protein | | | |
| DDB_G0270292 | | | | DDB_G0270292 | | | | cellulose-binding domain-containing protein | | | |
| DDB_G0270290 | | | | DDB_G0270290 | | | | cellulose-binding domain-containing protein | | | |
| DDB_G0270288 | | | | DDB_G0270288 | | | | cellulose-binding domain-containing protein | | | |
| DDB_G0274189 | | | | ecmJ | | | | cellulose-binding domain-containing protein, putative extracellular matrix protein | | | |
| DDB_G0294473 | | | | staA | | | | cellulose-binding domain-containing protein | | | |
| DDB_G0280245 | | | | ecmL | | | | cellulose-binding domain-containing protein, putative extracellular matrix protein | | | |
| DDB_G0287821 | | | | DDB_G0287821 | | | | cellulose-binding domain-containing protein | | | |
| DDB_G0287819 | | | | ecmK | | | | cellulose-binding domain-containing protein, putative extracellular matrix protein | | | |
| DDB_G0277191 | | | | DDB_G0277191 | | | | cellulose-binding domain-containing protein | | | |
| DDB_G0292222 | | | | ecmI | | | | cellulose-binding domain-containing protein, putative extracellular matrix protein | | | |
| DDB_G0292220 | | | | ecmH | | | | cellulose-binding domain-containing protein, putative extracellular matrix protein | | | |
| DDB_G0286663 | | | | ecmC | | | | cellulose-binding domain-containing protein, extracellular matrix protein, sheathin | | | |
| DDB_G0271092 | | | | H3c | | | | histone H3 | | | |
| DDB_G0283337 | | | | DDB_G0283337 | | | | ankyrin repeat-containing protein, putative protein kinase | | | |
| DDB_G0291516 | | | | DDB_G0291516 | | | | ankyrin repeat-containing protein, putative protein kinase | | | |
| DDB_G0273129 | | | | fnkD | | | | FNIP repeat-containing protein, FNIPK subfamily protein kinase, protein kinase, STE group | | | |
| DDB_G0287791 | | | | gcvH3 | | | | glycine cleavage system H-protein | | | |
| DDB_G0272987 | | | | irlA | | | | IRE family protein kinase, putative protein serine/threonine kinase | | | |
| DDB_G0269632 | | | | irlD | | | | IRE family protein kinase, putative protein serine/threonine kinase | | | |
| DDB_G0272618 | | | | tkt-1 | | | | glycolaldehydetransferase, transketolase | | | |
| DDB_G0288265 | | | | DDB_G0288265 | | | | chaperonin 10, chaperonin Cpn10 family protein | | | |
| DDB_G0275123 | | | | alyA | | | | lysozyme | | | |
| DDB_G0275121 | | | | alyC | | | | lysozyme | | | |
| DDB_G0273197 | | | | alyD-1 | | | | lysozyme | | | |
| DDB_G0268058 | | | | alrC | | | | aldo-keto reductase | | | |
| DDB_G0273131 | | | | g6pd-1 | | | | glucose 6-phosphate-1-dehydrogenase | | | |
| DDB_G0285023 | | | | alrF | | | | aldo-keto reductase | | | |
| DDB_G0275951 | | | | DDB_G0275951 | | | | putative transmembrane protein | | | |
| DDB_G0269876 | | | | samkA | | | | SAM domain-containing protein, SAMK family protein kinase, calcium-binding EF-hand domain-containing protein | | | |
| DDB_G0272304 | | | | aspS2 | | | | aspartate-tRNA ligase, aspartyl-tRNA synthetase | | | |
| DDB_G0288269 | | | | fslE | | | | G-protein-coupled receptor (GPCR) family protein, frizzled and smoothened-like protein | | | |
| DDB_G0288253 | | | | fslF | | | | G-protein-coupled receptor (GPCR) family protein, frizzled and smoothened-like protein | | | |
| DDB_G0288261 | | | | fslG | | | | G-protein-coupled receptor (GPCR) family protein, frizzled and smoothened-like protein | | | |
| DDB_G0272867 | | | | argS1 | | | | arginine-tRNA ligase, arginyl-tRNA synthetase | | | |
| DDB_G0273293 | | | | DDB_G0273293 | | | | unknown | | | |
| DDB_G0273079 | | | | DDB_G0273079 | | | | unknown | | | |
| DDB_G0293886 | | | | omt11 | | | | O-methyltransferase family 2 protein | | | |
| DDB_G0270162 | | | | allB2 | | | | allantoinase | | | |
| DDB_G0283137 | | | | enoA | | | | 2-phospho-D-glycerate hydrolase, enolase, phosphopyruvate hydratase | | | |
| DDB_G0286095 | | | | plbC | | | | phospholipase B-like protein | | | |
| DDB_G0273195 | | | | DDB_G0273195 | | | | oxalate/formate antiporter | | | |
| DDB_G0271530 | | | | pks9 | | | | beta-ketoacyl synthase family protein, putative polyketide synthase | | | |
| DDB_G0285095 | | | | DDB_G0285095 | | | | TPR repeat-containing protein | | | |
| DDB_G0274263 | | | | DDB_G0274263 | | | | putative glutathione S-transferase, putative glutathione transferase | | | |
| DDB_G0272632 | | | | DDB_G0272632 | | | | putative glutathione S-transferase, putative glutathione transferase | | | |
| DDB_G0273153 | | | | DDB_G0273153 | | | | putative glutathione transferase | | | |
| DDB_G0288715 | | | | gadB | | | | glutamate decarboxylase | | | |
| DDB_G0290537 | | | | DDB_G0290537 | | | | aldehyde dehydrogenase, putative NAD-dependent aldehyde dehydrogenase | | | |
| DDB_G0289253 | | | | tmcC | | | | HAT repeat-containing protein | | | |
| DDB_G0293354 | | | | DDB_G0293354 | | | | enoyl Coenzyme A hydratase, cytosolic, enoyl-CoA hydratase | | | |
| DDB_G0293286 | | | | psiR | | | | PA14 domain-containing protein, putative extracellular matrix protein | | | |
| DDB_G0293288 | | | | psiQ | | | | PA14 domain-containing protein, putative extracellular matrix protein | | | |
| DDB_G0278289 | | | | fslO | | | | frizzled and smoothened-like protein | | | |
| DDB_G0267566 | | | | DDB_G0267566 | | | | ankyrin repeat-containing protein, putative protein serine/threonine kinase | | | |
| DDB_G0287199 | | | | 5NT | | | | 5'-nucleotidase | | | |
| DDB_G0279119 | | | | DDB_G0279119 | | | | unknown | | | |
| DDB_G0278651 | | | | manE | | | | alpha-mannosidase | | | |
| DDB_G0277323 | | | | DDB_G0277323 | | | | FNIP repeat-containing protein | | | |
| DDB_G0276937 | | | | expl5 | | | | expansin-like protein | | | |
| DDB_G0267844 | | | | expl6 | | | | expansin-like protein | | | |
| DDB_G0267846 | | | | expl1 | | | | expansin-like protein | | | |
| DDB_G0283077 | | | | DDB_G0283077 | | | | putative glycoside hydrolase | | | |
| DDB_G0271314 | | | | DDB_G0271314 | | | | putative glycoside hydrolase | | | |
| DDB_G0286277 | | | | DDB_G0286277 | | | | putative glycoside hydrolase | | | |
| DDB_G0272586 | | | | DDB_G0272586 | | | | unknown | | | |
| DDB_G0272781 | | | | pmmB | | | | phosphomannomutase B | | | |
| DDB_G0269518 | | | | abnB | | | | actobindin | | | |
| DDB_G0269520 | | | | abnC | | | | actobindin | | | |
| DDB_G0275279 | | | | DDB_G0275279 | | | | unknown | | | |
| DDB_G0270214 | | | | DD7-1 | | | | galactose-binding domain-containing protein | | | |
| DDB_G0273225 | | | | aslC-1 | | | | putative acetyl-CoA synthetase | | | |
| DDB_G0273211 | | | | aslD-1 | | | | putative acetyl-CoA synthetase | | | |
| trappc10-1 | | | | trappc10-1 | | | | ortholog of the trafficking protein particle complex subunit 10%2C part of the multisubunit TRAPP (transport protein particle) complex%3B there is a second copy of this gene%2C %3Ca href%3D%22gene_page.pl?primary_id%3DDDB_G0273719%22%3E%3Ci%3Etrappc10-2%3C%2Fi%3E%3C%2Fa%3E | | | |
| DDB_G0272216 | | | | aslF | | | | putative acetyl-CoA synthetase | | | |
| DDB_G0273215 | | | | aslG-1 | | | | putative acetyl-CoA synthetase | | | |
| DDB_G0267506 | | | | aslH | | | | putative acetyl-CoA synthetase | | | |
| DDB_G0273227 | | | | aslI-1 | | | | putative acetyl-CoA synthetase | | | |
| DDB_G0280491 | | | | DDB_G0280491 | | | | RWP-RK domain-containing protein | | | |
| DDB_G0273305 | | | | aslJ-1 | | | | putative acetyl-CoA synthetase | | | |
| DDB_G0273229 | | | | aslK-1 | | | | putative acetyl-CoA synthetase | | | |
| DDB_G0273365 | | | | aslL-1 | | | | putative acetyl-CoA synthetase | | | |
| DDB_G0273223 | | | | aslO-1 | | | | putative acetyl-CoA synthetase | | | |
| DDB_G0267544 | | | | aslM | | | | AMP-dependent synthetase and ligase domain-containing protein, putative acetyl-CoA synthetase | | | |
| DDB_G0291660 | | | | smp3 | | | | glycosyltransferase, putative dolichyl-phosphate-mannose alpha-1, 2-mannosyltransferase | | | |
| DDB_G0272582 | | | | maoB-1 | | | | amine oxidase (flavin-containing), monoamine oxidase | | | |
| DDB_G0272584 | | | | maoC-1 | | | | amine oxidase (flavin-containing), monoamine oxidase | | | |
| DDB_G0289265 | | | | DDB_G0289265 | | | | putative amino oxidase | | | |
| DDB_G0272646 | | | | cofD-1 | | | | cofilin | | | |
| DDB_G0292156 | | | | fscG | | | | G-protein-coupled receptor (GPCR) family protein, frizzled and smoothened-like sans CRD protein | | | |
| DDB_G0283839 | | | | grlN | | | | G-protein-coupled receptor (GPCR) family 3 protein 13 | | | |
| DDB_G0281735 | | | | DDB_G0281735 | | | | putative transmembrane protein | | | |
| DDB_G0273081 | | | | DDB_G0273081 | | | | putative histidase, putative histidine ammonia-lyase | | | |
| DDB_G0284759 | | | | psiM | | | | PA14 domain-containing protein | | | |
| DDB_G0270366 | | | | DDB_G0270366 | | | | unknown | | | |
| DDB_G0270368 | | | | DDB_G0270368 | | | | unknown | | | |
| DDB_G0270328 | | | | DDB_G0270328 | | | | unknown | | | |
| DDB_G0284745 | | | | 4cl2 | | | | 4-coumarate-CoA ligase | | | |
| DDB_G0279465 | | | | DDB_G0279465 | | | | putative dTDP-D-glucose 4, 6-dehydratase | | | |
| DDB_G0290225 | | | | DDB_G0290225 | | | | patatin family protein | | | |
| DDB_G0273449 | | | | DDB_G0273449 | | | | unknown | | | |
| DDB_G0293762 | | | | DDB_G0293762 | | | | carbohydrate-binding domain-containing protein | | | |
| DDB_G0274287 | | | | fslC | | | | G-protein-coupled receptor (GPCR) family protein, frizzled and smoothened-like protein | | | |
| DDB_G0269528 | | | | fslD | | | | G-protein-coupled receptor (GPCR) family protein, frizzled and smoothened-like protein | | | |
| DDB_G0284775 | | | | fut2 | | | | alpha-3/4-fucosyltransferase, glycosyltransferase | | | |
| DDB_G0274297 | | | | fut3 | | | | alpha-3/4-fucosyltransferase, glycosyltransferase | | | |
| DDB_G0284505 | | | | fut5 | | | | alpha-3/4-fucosyltransferase, glycosyltransferase | | | |
| DDB_G0284503 | | | | fut6 | | | | alpha-3/4-fucosyltransferase, glycosyltransferase | | | |
| DDB_G0269900 | | | | gnt10 | | | | GlcNAc transferase, beta-1, 6-N-acetylglucosaminyltransferase, core-2/I-branching enzyme, glycosyltransferase | | | |
| DDB_G0274741 | | | | gnt15 | | | | putative GlcNAc transferase, putative beta-1, 3-N-acetylglucosaminyltransferase, putative glycosyltransferase | | | |
| DDB_G0268160 | | | | gnt12 | | | | putative GlcNAc transferase, putative beta-1, 3-N-acetylglucosaminyltransferase, putative glycosyltransferase | | | |
| DDB_G0270528 | | | | gpt2 | | | | putative glycophosphotransferase | | | |
| DDB_G0277597 | | | | gpt3 | | | | Stealth family protein, putative glycophosphotransferase | | | |
| DDB_G0274651 | | | | gpt4 | | | | Stealth family protein, putative glycophosphotransferase | | | |
| DDB_G0268950 | | | | gpt6 | | | | putative glycophosphotransferase | | | |
| DDB_G0274755 | | | | gpt7 | | | | Stealth family protein, putative glycophosphotransferase | | | |
| DDB_G0286711 | | | | gpt8 | | | | putative glycophosphotransferase | | | |
| DDB_G0286709 | | | | gpt9 | | | | putative glycophosphotransferase | | | |
| DDB_G0282461 | | | | grlC | | | | G-protein-coupled receptor (GPCR) family 3 protein 3 | | | |
| DDB_G0286895 | | | | grlD | | | | G-protein-coupled receptor (GPCR) family 3 protein 4 | | | |
| DDB_G0282175 | | | | grlF | | | | G-protein-coupled receptor (GPCR) family 3 protein 6 | | | |
| DDB_G0268544 | | | | ugt3 | | | | putative glycosyltransferase | | | |
| DDB_G0282459 | | | | grlH | | | | G-protein-coupled receptor (GPCR) family 3 protein 8 | | | |
| DDB_G0289171 | | | | DDB_G0289171 | | | | unknown | | | |
| DDB_G0274177 | | | | DDB_G0274177 | | | | EGF-like domain-containing protein | | | |
| DDB_G0272883 | | | | ksrA-1 | | | | 3-dehydrosphinganine reductase, 3-ketosphinganine reductase, short-chain dehydrogenase/reductase (SDR) family protein | | | |
| DDB_G0273035 | | | | fslM-1 | | | | G-protein-coupled receptor (GPCR) family protein, frizzled and smoothened-like protein | | | |
| DDB_G0282989 | | | | fscB | | | | G-protein-coupled receptor (GPCR) family protein, frizzled and smoothened-like sans CRD protein | | | |
| DDB_G0272240 | | | | fscD | | | | G-protein-coupled receptor (GPCR) family protein, frizzled and smoothened-like sans CRD protein | | | |
| DDB_G0272306 | | | | fscE | | | | G-protein-coupled receptor (GPCR) family protein, frizzled and smoothened-like sans CRD protein | | | |
| DDB_G0285981 | | | | DDB_G0285981 | | | | type A von Willebrand factor (VWFA) domain-containing protein | | | |
| DDB_G0287323 | | | | DDB_G0287323 | | | | putative oxidoreductase, Gfo/Idh/MocA family | | | |
| DDB_G0283367 | | | | cofE | | | | cofilin | | | |
| DDB_G0290755 | | | | DDB_G0290755 | | | | C2 domain-containing protein | | | |
| DDB_G0278699 | | | | DDB_G0278699 | | | | unknown | | | |
| DDB_G0273091 | | | | DDB_G0273091 | | | | ZIP family zinc transporter, zinc/iron permease | | | |
| DDB_G0281921 | | | | DDB_G0281921 | | | | ZIP zinc transporter protein, zinc/iron permease | | | |
| DDB_G0289197 | | | | H2Av3 | | | | histone H2A domain-containing protein | | | |
| DDB_G0275079 | | | | hspG1 | | | | heat shock protein Hsp20 domain-containing protein, putative alpha-crystallin-type heat shock protein | | | |
| DDB_G0276953 | | | | hspG9 | | | | heat shock protein Hsp20 domain-containing protein | | | |
| DDB_G0277491 | | | | hspG12 | | | | heat shock protein Hsp20 domain-containing protein, putative alpha-crystallin-type heat shock protein | | | |
| DDB_G0276949 | | | | hspG6 | | | | heat shock protein Hsp20 domain-containing protein | | | |
| DDB_G0276875 | | | | hspG5 | | | | heat shock protein Hsp20 domain-containing protein | | | |
| DDB_G0277119 | | | | hspG8 | | | | heat shock protein Hsp20 domain-containing protein | | | |
| DDB_G0277125 | | | | hspG11 | | | | heat shock protein Hsp20 domain-containing protein, putative alpha-crystallin-type heat shock protein | | | |
| DDB_G0276607 | | | | hspG3 | | | | heat shock protein Hsp20 domain-containing protein | | | |
| DDB_G0276003 | | | | hspG2 | | | | heat shock protein Hsp20 domain-containing protein | | | |
| DDB_G0276587 | | | | hspG4 | | | | heat shock protein Hsp20 domain-containing protein | | | |
| DDB_G0277123 | | | | hspG10 | | | | HSP20-like chaperone domain-containing protein | | | |
| DDB_G0280013 | | | | hspM | | | | heat shock protein Hsp20 domain-containing protein | | | |
| DDB_G0271058 | | | | vilC | | | | villin | | | |
| DDB_G0271238 | | | | DDB_G0271238 | | | | unknown | | | |
| DDB_G0271056 | | | | DDB_G0271056 | | | |  | | | |
| DDB_G0288861 | | | | DDB_G0288861 | | | | heat shock protein Hsp20 domain-containing protein | | | |
| DDB_G0283913 | | | | DDB_G0283913 | | | | heat shock protein Hsp20 domain-containing protein | | | |
| DDB_G0268374 | | | | dcd2B | | | | neutral/alkaline nonlysosomal ceramidase family protein | | | |
| DDB_G0283827 | | | | DDB_G0283827 | | | | WASP-related protein, putative actin binding protein | | | |
| DDB_G0277027 | | | | tmem56A | | | | TMEM56 family protein 1, TRAM, LAG1 and CLN8 homology domain-containing protein | | | |
| DDB_G0281493 | | | | sodD | | | | superoxide dismutase | | | |
| DDB_G0280703 | | | | DDB_G0280703 | | | | elongation factor 1b-related protein, elongation factor 1beta-related protein | | | |
| DDB_G0283195 | | | | DDB_G0283195 | | | | B-box zinc finger-containing protein, FNIP repeat-containing protein | | | |
| DDB_G0284515 | | | | DDB_G0284515 | | | | B-box zinc finger-containing protein, FNIP repeat-containing protein | | | |
| DDB_G0274617 | | | | DDB_G0274617 | | | | FNIP repeat-containing protein | | | |
| DDB_G0271966 | | | | DDB_G0271966 | | | | poly(ADP-ribose) polymerase, catalytic region domain-containing protein | | | |
| DDB_G0277321 | | | | crtp2 | | | | chloroquine resistance transporter-like protein, putative transmembrane protein | | | |
| DDB_G0270204 | | | | crtp3 | | | | chloroquine resistance transporter-like protein, putative transmembrane protein | | | |
| DDB_G0272927 | | | | DDB_G0272927 | | | | cystine knot domain-containing protein | | | |
| DDB_G0275755 | | | | DDB_G0275755 | | | | AhpC/TSA family protein | | | |
| DDB_G0273201 | | | | DDB_G0273201 | | | | DNA-binding HORMA domain-containing protein, mitotic spindle assembly checkpoint protein | | | |
| DDB_G0283637 | | | | xrcc3 | | | | AAA ATPase domain-containing protein, putative DNA repair protein XRCC3 | | | |
| DDB_G0285273 | | | | DDB_G0285273 | | | | AhpC/TSA family protein | | | |
| DDB_G0268192 | | | | DDB_G0268192 | | | | AhpC/TSA family protein | | | |
| DDB_G0269270 | | | | DDB_G0269270 | | | | AhpC/TSA family protein | | | |
| DDB_G0269260 | | | | DDB_G0269260 | | | | AhpC/TSA family protein | | | |
| DDB_G0269392 | | | | DDB_G0269392 | | | | AhpC/TSA family protein | | | |
| DDB_G0279951 | | | | DDB_G0279951 | | | | AhpC/TSA family protein | | | |
| DDB_G0269256 | | | | DDB_G0269256 | | | | AhpC/TSA family protein | | | |
| DDB_G0292136 | | | | hatC | | | | actin binding protein, hisactophilin III | | | |
| DDB_G0273139 | | | | DDB_G0273139 | | | | putative DNA repair protein, putative Rad51 protein | | | |
| DDB_G0271516 | | | | DDB_G0271516 | | | | phosphoesterase, PA-phosphatase related-family protein | | | |
| DDB_G0271784 | | | | DDB_G0271784 | | | | unknown | | | |
| DDB_G0282183 | | | | CYP513A1 | | | | cytochrome P450 family protein | | | |
| DDB_G0287087 | | | | CYP513B1 | | | | cytochrome P450 family protein | | | |
| DDB_G0282353 | | | | CYP513E1 | | | | cytochrome P450 family protein | | | |
| DDB_G0273045 | | | | CYP508A1-1 | | | | cytochrome P450 family protein | | | |
| DDB_G0272604 | | | | CYP508A2-1 | | | | cytochrome P450 family protein | | | |
| DDB_G0268444 | | | | DDB_G0268444 | | | | TRAF-type zinc finger-containing protein, meprin and TRAF homology (MATH) domain-containing protein | | | |
| DDB_G0272338 | | | | DDB_G0272338 | | | | unknown | | | |
| DDB_G0273047 | | | | CYP508A3-1 | | | | cytochrome P450 family protein | | | |
| DDB_G0281967 | | | | DDB_G0281967 | | | | putative cell surface glycoprotein | | | |
| DDB_G0272590 | | | | srp14-1 | | | | signal recognition particle 14 kDa subunit | | | |
| DDB_G0283481 | | | | DDB_G0283481 | | | | putative cell surface glycoprotein | | | |
| DDB_G0269740 | | | | DDB_G0269740 | | | | putative cell surface glycoprotein | | | |
| DDB_G0272052 | | | | DDB_G0272052 | | | | leucine-rich repeat-containing protein (LRR), putative cell surface glycoprotein | | | |
| DDB_G0281377 | | | | DDB_G0281377 | | | | putative cell surface glycoprotein | | | |
| DDB_G0284073 | | | | DDB_G0284073 | | | | putative cell surface glycoprotein | | | |
| DDB_G0281853 | | | | DDB_G0281853 | | | | putative cell surface glycoprotein | | | |
| DDB_G0267684 | | | | DDB_G0267684 | | | | putative cell surface glycoprotein | | | |
| DDB_G0283929 | | | | CYP517A1 | | | | cytochrome P450 family protein | | | |
| DDB_G0273389 | | | | psiG-1 | | | | PA14 domain-containing protein | | | |
| DDB_G0274441 | | | | psiL | | | | PA14 domain-containing protein | | | |
| DDB_G0280171 | | | | psiP | | | | PA14 domain-containing protein | | | |
| DDB_G0280167 | | | | psiO | | | | PA14 domain-containing protein | | | |
| DDB_G0294559 | | | | DDB_G0294559 | | | | RNA recognition motif-containing protein RRM, RNA-binding region RNP-1 domain-containing protein | | | |
| DDB_G0273163 | | | | psmB4-1 | | | | 20S proteasome subunit beta-4, proteasome subunit beta type 4 | | | |
| DDB_G0278879 | | | | gdt9 | | | | GDT family protein kinase, dusty protein kinase, protein kinase, TKL group, putative transmembrane protein, tyrosine kinase-like protein | | | |
| DDB_G0284015 | | | | DDB_G0284015 | | | | BRCT domain-containing protein, PARP domain-containing protein, WWE domain-containing protein, poly(ADP-ribose) polymerase, catalytic region domain-containing protein | | | |
| DDB_G0282811 | | | | gtaK | | | | GATA zinc finger domain-containing protein 11, putative GATA-binding transcription factor | | | |
| DDB_G0277249 | | | | DDB_G0277249 | | | | ankyrin repeat-containing protein | | | |
| DDB_G0290707 | | | | CYP514A4 | | | | cytochrome P450 family protein | | | |
| DDB_G0290743 | | | | CYP514A1 | | | | cytochrome P450 family protein | | | |
| DDB_G0284647 | | | | CYP517A2 | | | | cytochrome P450 family protein | | | |
| DDB_G0291572 | | | | DDB_G0291572 | | | | unknown | | | |
| DDB_G0291574 | | | | DDB_G0291574 | | | | putative transmembrane protein | | | |
| DDB_G0283933 | | | | CYP517A4 | | | | cytochrome P450 family protein | | | |
| DDB_G0275197 | | | | CYP518B1 | | | | cytochrome P450 family protein | | | |
| DDB_G0284089 | | | | CYP519B1 | | | | cytochrome P450 family protein | | | |
| DDB_G0272556 | | | | CYP519C1 | | | | cytochrome P450 family protein | | | |
| DDB_G0286419 | | | | CYP519E1 | | | | cytochrome P450 family protein | | | |
| DDB_G0292496 | | | | CYP520A1 | | | | cytochrome P450 family protein | | | |
| DDB_G0293738 | | | | CYP521A1 | | | | cytochrome P450 family protein | | | |
| DDB_G0282769 | | | | CYP522A1 | | | | cytochrome P450 family protein | | | |
| DDB_G0288067 | | | | MF12 | | | | prespore protein MF12 | | | |
| DDB_G0284923 | | | | CYP554A1 | | | | cytochrome P450 family protein | | | |
| DDB_G0294489 | | | | trxC | | | | thioredoxin | | | |
| DDB_G0294499 | | | | staB | | | | cellulose-binding domain-containing protein | | | |
| DDB_G0281955 | | | | agnD | | | | argonaut-like protein | | | |
| DDB_G0281809 | | | | DDB_G0281809 | | | | RapGAP/RanGAP domain-containing protein | | | |
| DDB_G0273165 | | | | DDB_G0273165 | | | | endonuclease V | | | |
| DDB_G0274777 | | | | DDB_G0274777 | | | | DEAD/DEAH box helicase domain-containing protein, DNA replication helicase | | | |
| DDB_G0293180 | | | | CSN6 | | | | COP9 signalosome complex subunit 6, Mov34/MPN/PAD-1 family protein | | | |
| DDB_G0291498 | | | | cpnF | | | | copine F, phospholipid-binding protein | | | |
| DDB_G0285911 | | | | DDB_G0285911 | | | | LIM-type zinc finger-containing protein | | | |
| DDB_G0284101 | | | | snrpA1 | | | | U2 small nuclear ribonucleoprotein A', leucine-rich repeat-containing protein (LRR) | | | |
| DDB_G0279681 | | | | DDB_G0279681 | | | | calcium-binding EF-hand domain-containing protein | | | |
| DDB_G0294567 | | | | DDB_G0294567 | | | | unknown | | | |
| DDB_G0286169 | | | | rabZ | | | | Rab GTPase | | | |
| DDB_G0294569 | | | | DDB_G0294569 | | | | Rab GTPase | | | |
| DDB_G0276007 | | | | DDB_G0276007 | | | | Rab GTPase | | | |
| DDB_G0290827 | | | | DDB_G0290827 | | | | Rab GTPase domain-containing protein | | | |
| DDB_G0290791 | | | | rabK2 | | | | Rab GTPase domain-containing protein | | | |
| DDB_G0294571 | | | | DDB_G0294571 | | | | Rab GTPase | | | |
| DDB_G0278011 | | | | racQ | | | | Rho GTPase | | | |
| DDB_G0294575 | | | | DDB_G0294575 | | | | ankyrin repeat-containing protein | | | |
| DDB_G0286625 | | | | DDB_G0286625 | | | | PhoPQ-activated pathogenicity-related protein | | | |
| DDB_G0292332 | | | | DDB_G0292332 | | | | ADP-ribosylation factor-like protein | | | |
| DDB_G0290831 | | | | rabK3 | | | | Rab GTPase domain-containing protein | | | |
| DDB_G0276897 | | | | DDB_G0276897 | | | | PhoPQ-activated pathogenicity-related protein | | | |
| DDB_G0284625 | | | | DDB_G0284625 | | | | EGF-like domain-containing protein | | | |
| DDB_G0269354 | | | | DDB_G0269354 | | | | EGF-like domain-containing protein | | | |
| DDB_G0287207 | | | | DDB_G0287207 | | | | Rab GTPase domain-containing protein | | | |
| DDB_G0287957 | | | | DDB_G0287957 | | | | putative AFK family protein kinase, putative actin fragmin kinase | | | |
| DDB_G0268812 | | | | DDB_G0268812 | | | | putative AFK family protein kinase, putative actin fragmin kinase | | | |
| DDB_G0287403 | | | | DDB_G0287403 | | | | unknown | | | |
| DDB_G0272058 | | | | DDB_G0272058 | | | | unknown | | | |
| DDB_G0280599 | | | | fhkB | | | | FHA domain-containing protein, RAD53 family protein kinase, protein kinase, CAMK group, putative CAM kinase (CAMK), putative protein serine/threonine kinase | | | |
| DDB_G0294585 | | | | DDB_G0294585 | | | | unknown | | | |
| DDB_G0294583 | | | | DDB_G0294583 | | | | Ras GTPase | | | |
| DDB_G0282647 | | | | DDB_G0282647 | | | | weakly similar to Rab small GTPases | | | |
| DDB_G0287849 | | | | trxD | | | | thioredoxin | | | |
| DDB_G0274301 | | | | DDB_G0274301 | | | | Ras GTPase domain-containing protein | | | |
| DDB_G0273205 | | | | DDB_G0273205 | | | | calcium-binding EF-hand domain-containing protein | | | |
| DDB_G0277941 | | | | DDB_G0277941 | | | | WD40 repeat-containing protein | | | |
| DDB_G0283953 | | | | mybU | | | | myb domain-containing protein | | | |
| DDB_G0272588 | | | | dpm2-1 | | | | dolichyl-phosphate mannosyltransferase 2 regulatory subunit | | | |
| DDB_G0276097 | | | | DDB_G0276097 | | | | putative transmembrane protein | | | |
| DDB_G0276095 | | | | DDB_G0276095 | | | | unknown | | | |
| DDB_G0294589 | | | | DDB_G0294589 | | | | short-chain dehydrogenase/reductase (SDR) family protein | | | |
| DDB_G0270110 | | | | dhx33 | | | | DEAD/DEAH box helicase | | | |
| DDB_G0273053 | | | | DDB_G0273053 | | | | SNF2-related domain-containing protein, putative DEAD/DEAH box helicase | | | |
| DDB_G0279239 | | | | gtaY | | | | GATA zinc finger domain-containing protein 25 | | | |
| DDB_G0274965 | | | | DDB_G0274965 | | | | CHR group protein, SNF2-related domain-containing protein, helicase, C-terminal domain-containing protein | | | |
| DDB_G0281691 | | | | DDB_G0281691 | | | | unknown | | | |
| DDB_G0293220 | | | | gpn1 | | | | AAA ATPase domain-containing protein, GPN-loop GTPase 1, Ras GTPase domain-containing protein | | | |
| DDB_G0281055 | | | | DDB_G0281055 | | | | putative SAM dependent methyltransferase | | | |
| DDB_G0273203 | | | | snf12-1 | | | | CHC group protein, SWIB/MDM2 domain-containing protein | | | |
| DDB_G0294593 | | | | DDB_G0294593 | | | | unknown | | | |
| DDB_G0288239 | | | | sibE | | | | type A von Willebrand factor (VWFA) domain-containing protein | | | |
| DDB_G0288103 | | | | sibB | | | | type A von Willebrand factor (VWFA) domain-containing protein | | | |
| DDB_G0288197 | | | | sibD | | | | integrin beta family protein%3B cytosolic domain binds talin | | | |
| DDB_G0286851 | | | | DDB_G0286851 | | | | IPT/TIG domain-containing protein | | | |
| DDB_G0270920 | | | | DDB_G0270920 | | | | unknown | | | |
| DDB_G0294595 | | | | DDB_G0294595 | | | | MYND-type zinc finger-containing protein, SET domain-containing protein, tetratricopeptide-like helical domain-containing protein (TPR) | | | |
| DDB_G0275227 | | | | DDB_G0275227 | | | | FKBP-type peptidylprolyl cis-trans isomerase (PPIase) | | | |
| DDB_G0277729 | | | | DDB_G0277729 | | | | unknown | | | |
| DDB_G0272608 | | | | stip-1 | | | | D111/G-patch domain-containing protein | | | |
| DDB_G0272658 | | | | DDB_G0272658 | | | | hssA/2C/7E family protein | | | |
| cwc15-1 | | | | cwc15-1 | | | | ortholog of S. pombe Cwf15 and S. cerevisiae Cwc15%2C thought to be non-essential components of an mRNA splicing complex%3B there is a second copy of this gene%2C %3Ca href%3D%22%2Fdb%2Fcgi-bin%2Fgene_page.pl?primary_id%3DDDB_G0273631%22%3E%3Ci%3Ecwc15-2%3C%2Fi%3E%3C%2Fa%3E | | | |
| DDB_G0286641 | | | | DDB_G0286641 | | | | putative nucleolar GTP-binding protein 1 | | | |
| DDB_G0270556 | | | | syn1B | | | | putative syntaxin 1, t-SNARE family protein | | | |
| DDB_G0276469 | | | | syn16B | | | | putative syntaxin 16, t-SNARE family protein | | | |
| srrm1 | | | | srrm1 | | | | very similar to the mammalian serine%2Farginine repetitive matrix protein 1 (SRRM1)%2C which is part of pre- and post-splicing multiprotein mRNP complexes involved in numerous pre-mRNA processing events | | | |
| DDB_G0273101 | | | | DDB_G0273101 | | | | SEC7-like domain-containing protein, armadillo-like helical domain-containing protein | | | |
| DDB_G0275017 | | | | DDB_G0275017 | | | | HAM group protein, MOZ/SAS-like domain-containing protein, MYST family protein, putative histone acetyltransferase | | | |
| DDB_G0287385 | | | | DDB_G0287385 | | | | putative transmembrane protein | | | |
| DDB_G0271714 | | | | DDB_G0271714 | | | | hssA/2C/7E family protein | | | |
| DDB_G0284327 | | | | tmem34 | | | | DUF300 family protein, transmembrane protein | | | |
| DDB_G0291668 | | | | DDB_G0291668 | | | | putative transmembrane protein | | | |
| DDB_G0273039 | | | | iptC-1 | | | | putative isopentenylpyrophosphate (IPP) transferase, putative isopentenyltransferase | | | |
| pabpn1 | | | | pabpn1 | | | | similar to human PABPN1 (PAB2)%2C which is involved in the 3%27-end formation of mRNA precursors (pre-mRNA) by the addition of a poly(A) tail%3B defects in PABPN1 may be the cause of an autosomal dominant oculopharyngeal muscular dystrophy | | | |
| DDB_G0270148 | | | | cpsf4 | | | | CCCH-type zinc finger-containing protein, cleavage and polyadenylation specificity factor 30 kDa subunit | | | |
| DDB_G0275745 | | | | DDB_G0275745 | | | | unknown | | | |
| DDB_G0275767 | | | | DDB_G0275767 | | | | EGF-like domain-containing protein | | | |
| DDB_G0291113 | | | | DDB_G0291113 | | | | saposin B domain-containing protein | | | |
| DDB_G0284339 | | | | DDB_G0284339 | | | | saposin B domain-containing protein | | | |
| DDB_G0287335 | | | | DDB_G0287335 | | | | RNA recognition motif-containing protein RRM, RNA-binding region RNP-1 domain-containing protein | | | |
| DDB_G0286297 | | | | DDB_G0286297 | | | | RNA recognition motif-containing protein RRM, RNA-binding region RNP-1 domain-containing protein, putative splicing factor 3B 14 kDa subunit | | | |
| DDB_G0281613 | | | | DDB_G0281613 | | | | unknown | | | |
| DDB_G0281201 | | | | DDB_G0281201 | | | | unknown | | | |
| DDB_G0281199 | | | | DDB_G0281199 | | | | unknown | | | |
| DDB_G0293724 | | | | DDB_G0293724 | | | | unknown | | | |
| DDB_G0278069 | | | | gacII | | | | RhoGAP domain-containing protein, SH3 domain-containing protein | | | |
| DDB_G0281617 | | | | DDB_G0281617 | | | | unknown | | | |
| DDB_G0271254 | | | | DDB_G0271254 | | | | unknown | | | |
| DDB_G0281187 | | | | DDB_G0281187 | | | | unknown | | | |
| DDB_G0281193 | | | | DDB_G0281193 | | | | unknown | | | |
| DDB_G0277421 | | | | DDB_G0277421 | | | | unknown | | | |
| DDB_G0277417 | | | | DDB_G0277417 | | | | unknown | | | |
| DDB_G0277423 | | | | DDB_G0277423 | | | | unknown | | | |
| DDB_G0277425 | | | | DDB_G0277425 | | | | unknown | | | |
| DDB_G0284999 | | | | DDB_G0284999 | | | | unknown | | | |
| DDB_G0284571 | | | | gacC | | | | RhoGAP domain-containing protein | | | |
| DDB_G0267600 | | | | DDB_G0267600 | | | | enoyl-CoA hydratase/isomerase domain-containing protein | | | |
| DDB_G0283429 | | | | DDB_G0283429 | | | | similar to proteins in bacteria and protozoa%3B COG4271%2C predicted nucleotide-binding protein containing TIR -like domain (Transcription) | | | |
| DDB_G0283955 | | | | gacG | | | | RhoGAP domain-containing protein | | | |
| DDB_G0289231 | | | | DDB_G0289231 | | | | peptidase S28 family protein | | | |
| DDB_G0289939 | | | | DDB_G0289939 | | | | unknown | | | |
| DDB_G0290509 | | | | DDB_G0290509 | | | | unknown | | | |
| DDB_G0291928 | | | | ubqI | | | | ubiquitin | | | |
| DDB_G0267726 | | | | DDB_G0267726 | | | | unknown | | | |
| DDB_G0268212 | | | | DDB_G0268212 | | | | putative DEAD/DEAH box helicase | | | |
| DDB_G0272624 | | | | adrm1-1 | | | | adhesion regulating molecule family protein | | | |
| DDB_G0272634 | | | | dohh-1 | | | | PBS lyase HEAT-like repeat-containing protein, deoxyhypusine dioxygenase, deoxyhypusine hydroxylase, deoxyhypusine monooxygenase | | | |
| DDB_G0268600 | | | | DDB_G0268600 | | | | unknown | | | |
| DDB_G0269850 | | | | DDB_G0269850 | | | | DG1041 family protein, putative transmembrane protein | | | |
| DDB_G0279549 | | | | eIF3s4 | | | | RNA recognition motif-containing protein RRM, RNA-binding region RNP-1 domain-containing protein, eIF-3 delta, eukaryotic translation initiation factor 3 (eIF3) subunit 4 | | | |
| DDB_G0273083 | | | | DDB_G0273083 | | | | Dyp-type peroxidase family protein | | | |
| DDB_G0277203 | | | | DDB_G0277203 | | | | NAD-dependent epimerase/dehydratase family protein | | | |
| DDB_G0287981 | | | | act33 | | | | actin | | | |
| DDB_G0269476 | | | | act31 | | | | actin related protein | | | |
| DDB_G0269544 | | | | act32 | | | | actin | | | |
| DDB_G0275033 | | | | DDB_G0275033 | | | | unknown | | | |
| DDB_G0292188 | | | | DDB_G0292188 | | | | unknown | | | |
| DDB_G0269482 | | | | DDB_G0269482 | | | | unknown | | | |
| DDB_G0284769 | | | | vps24 | | | | SNF7 family protein | | | |
| DDB_G0279307 | | | | vacC | | | | band 7 family protein, vacuolin C | | | |
| DDB_G0284369 | | | | DDB_G0284369 | | | | DUF298 family protein, calcium-binding EF-hand domain-containing protein | | | |
| DDB_G0273237 | | | | DDB_G0273237 | | | | esterase/lipase/thioesterase domain-containing protein | | | |
| DDB_G0283811 | | | | mcfR | | | | mitochondrial substrate carrier family protein, putative mitochondrial coenzyme A transporter | | | |
| DDB_G0276811 | | | | DDB_G0276811 | | | | ankyrin repeat-containing protein | | | |
| DDB_G0294627 | | | | DDB_G0294627 | | | | MaoC-like dehydratase domain-containing protein | | | |
| DDB_G0274501 | | | | mcfY | | | | mitochondrial substrate carrier family protein | | | |
| DDB_G0276245 | | | | DDB_G0276245 | | | | acyl-CoA oxidase | | | |
| DDB_G0291588 | | | | ddx55 | | | | DEAD/DEAH box helicase, putative RNA helicase | | | |
| DDB_G0272849 | | | | DDB_G0272849 | | | | EGF-like domain-containing protein | | | |
| ap2a1-1 | | | | ap2a1-1 | | | | there is a second copy of this gene %3Ca href%3D%22%2Fdb%2Fcgi-bin%2Fgene_page.pl?primary_id%3DDDB_G0273501%22%3E%3Ci%3Eap2a1-1%3C%2Fi%3E%3C%2Fa%3E | | | |
| DDB_G0289721 | | | | ap2s1 | | | | adaptor-related protein complex 2, sigma 1 subunit, sigma adaptin | | | |
| DDB_G0279537 | | | | ap3d1 | | | | adaptor-related protein complex 3, delta 1 subunit, delta adaptin | | | |
| DDB_G0279539 | | | | DDB_G0279539 | | | | RNA-binding region RNP-1 domain-containing protein | | | |
| DDB_G0294635 | | | | DDB_G0294635 | | | | peptidase S8 and S53 domain-containing protein, putative transmembrane protein, serine peptidase | | | |
| DDB_G0271524 | | | | pks6 | | | | beta-ketoacyl synthase family protein, putative polyketide synthase | | | |
| DDB_G0271618 | | | | pks8 | | | | beta-ketoacyl synthase family protein, putative polyketide synthase | | | |
| DDB_G0281255 | | | | sec24 | | | | putative transport protein | | | |
| DDB_G0279043 | | | | DDB_G0279043 | | | | ankyrin repeat-containing protein | | | |
| DDB_G0271614 | | | | pks7 | | | | beta-ketoacyl synthase family protein, putative polyketide synthase | | | |
| DDB_G0282029 | | | | pks21 | | | | beta-ketoacyl synthase family protein, putative polyketide synthase | | | |
| DDB_G0271662 | | | | pks10 | | | | beta-ketoacyl synthase family protein, putative polyketide synthase | | | |
| DDB_G0273007 | | | | pks15 | | | | beta-ketoacyl synthase family protein, putative polyketide synthase | | | |
| DDB_G0281475 | | | | pks18 | | | | beta-ketoacyl synthase family protein, putative polyketide synthase | | | |
| DDB_G0287219 | | | | DDB_G0287219 | | | | unknown | | | |
| DDB_G0275989 | | | | DDB_G0275989 | | | | beta-ketoacyl synthase family protein | | | |
| DDB_G0275987 | | | | DDB_G0275987 | | | | beta-ketoacyl synthase family protein | | | |
| DDB_G0282027 | | | | pks19 | | | | beta-ketoacyl synthase family protein, putative polyketide synthase | | | |
| DDB_G0295475 | | | | DDB_G0295475 | | | | unknown | | | |
| DDB_G0288457 | | | | pks26 | | | | beta-ketoacyl synthase family protein, putative polyketide synthase | | | |
| DDB_G0287501 | | | | DDB_G0287501 | | | | beta-ketoacyl synthase family protein | | | |
| DDB_G0290467 | | | | pks27 | | | | beta-ketoacyl synthase family protein, putative polyketide synthase | | | |
| DDB_G0290469 | | | | pks28 | | | | beta-ketoacyl synthase family protein, putative polyketide synthase | | | |
| DDB_G0295479 | | | | DDB_G0295479 | | | | beta-ketoacyl synthase family protein | | | |
| DDB_G0290729 | | | | pks33 | | | | beta-ketoacyl synthase family protein, putative polyketide synthase | | | |
| DDB_G0281239 | | | | impa1 | | | | inositol monophosphatase (IMPase), inositol-phosphate phosphatase | | | |
| DDB_G0290699 | | | | pks29 | | | | beta-ketoacyl synthase family protein, putative polyketide synthase | | | |
| DDB_G0290701 | | | | pks30 | | | | beta-ketoacyl synthase family protein, putative polyketide synthase | | | |
| DDB_G0290703 | | | | pks31 | | | | beta-ketoacyl synthase family protein, putative polyketide synthase | | | |
| DDB_G0287351 | | | | dhx37 | | | | DEAD/DEAH box helicase | | | |
| DDB_G0291604 | | | | DDB_G0291604 | | | | beta-ketoacyl synthase family protein | | | |
| DDB_G0292544 | | | | pks42 | | | | beta-ketoacyl synthase family protein, putative polyketide synthase | | | |
| DDB_G0278525 | | | | plaA | | | | calcium-independent PLA2 (iPLA2), patatin family protein, phospholipase A2 | | | |
| DDB_G0274499 | | | | DDB_G0274499 | | | | patatin family protein | | | |
| DDB_G0283645 | | | | DDB_G0283645 | | | | calcium-binding EF-hand domain-containing protein, calcium-binding protein | | | |
| DDB_G0271520 | | | | pks5 | | | | beta-ketoacyl synthase family protein, putative polyketide synthase | | | |
| DDB_G0291684 | | | | pks41 | | | | beta-ketoacyl synthase family protein, putative polyketide synthase | | | |
| DDB_G0291614 | | | | pks40 | | | | beta-ketoacyl synthase family protein, putative polyketide synthase | | | |
| DDB_G0290737 | | | | pks34 | | | | beta-ketoacyl synthase family protein, putative polyketide synthase | | | |
| DDB_G0290937 | | | | pks38 | | | | beta-ketoacyl synthase family protein, putative polyketide synthase | | | |
| DDB_G0290597 | | | | DDB_G0290597 | | | | B-box zinc finger-containing protein, RabGAP/TBC domain-containing protein, putative GTPase activating protein (GAP) | | | |
| DDB_G0280201 | | | | DDB_G0280201 | | | | unknown | | | |
| DDB_G0279253 | | | | DDB_G0279253 | | | | putative guanine nucleotide exchange factor (GEF), regulator of chromosome condensation (RCC1) domain-containing protein | | | |
| DDB_G0281767 | | | | DDB_G0281767 | | | | BTB/POZ domain-containing protein, ankyrin repeat-containing protein, regulator of chromosome condensation (RCC1) domain-containing protein | | | |
| DDB_G0283691 | | | | DDB_G0283691 | | | | putative cell surface glycoprotein | | | |
| DDB_G0274359 | | | | cbpK | | | | NCS-1/frequenin-related protein, calcium-binding protein | | | |
| DDB_G0274357 | | | | DDB_G0274357 | | | | NCS-1/frequenin-related protein, calcium-binding protein | | | |
| DDB_G0273473 | | | | DDB_G0273473 | | | | unknown | | | |
| DDB_G0280021 | | | | mcfL | | | | mitochondrial substrate carrier family protein | | | |
| DDB_G0283751 | | | | mcfM | | | | mitochondrial substrate carrier family protein, putative mitochondrial folate transporter | | | |
| DDB_G0286573 | | | | ponH | | | | ponticulin-related protein | | | |
| DDB_G0284955 | | | | DDB_G0284955 | | | | P-type ATPase | | | |
| DDB_G0272728 | | | | DDB_G0272728 | | | | unknown | | | |
| DDB_G0295659 | | | | pks36 | | | | beta-ketoacyl synthase family protein, putative polyketide synthase | | | |
| DDB_G0295667 | | | | pks35 | | | | beta-ketoacyl synthase family protein, putative polyketide synthase | | | |
| DDB_G0285149 | | | | DDB_G0285149 | | | | RING zinc finger-containing protein, TRAF-type zinc finger-containing protein, meprin and TRAF homology (MATH) domain-containing protein | | | |
| DDB_G0293202 | | | | DDB_G0293202 | | | | TRAF-type zinc finger-containing protein, meprin and TRAF homology (MATH) domain-containing protein | | | |
| DDB_G0277267 | | | | DDB_G0277267 | | | | putative ubiquitin-conjugating enzyme E2 | | | |
| DDB_G0273235 | | | | DDB_G0273235 | | | | P-type ATPase | | | |
| DDB_G0290943 | | | | pks39 | | | | beta-ketoacyl synthase family protein, putative polyketide synthase | | | |
| DDB_G0272578 | | | | ap3b-1 | | | | beta adaptin | | | |
| DDB_G0268272 | | | | aslN | | | | AMP-dependent synthetase and ligase domain-containing protein, putative acetyl-CoA synthetase | | | |
| DDB_G0278767 | | | | DDB_G0278767 | | | | cyclin-like F-box containing protein | | | |
| DDB_G0282983 | | | | DDB_G0282983 | | | | cyclin-like F-box containing protein | | | |
| DDB_G0292404 | | | | sti1 | | | | stress-induced-phosphoprotein 1, tetratricopeptide-like helical domain-containing protein (TPR) | | | |
| DDB_G0292574 | | | | DDB_G0292574 | | | | glucose/ribitol dehydrogenase family protein | | | |
| DDB_G0269938 | | | | osbB | | | | oxysterol binding family protein, member 2 | | | |
| DDB_G0274517 | | | | osbE | | | | oxysterol binding family protein, member 5 | | | |
| DDB_G0281513 | | | | DDB_G0281513 | | | | equilibrative nucleoside transporter (ENT) family protein | | | |
| DDB_G0283911 | | | | DDB_G0283911 | | | | HSP20-like chaperone domain-containing protein | | | |
| DDB_G0274269 | | | | DDB_G0274269 | | | | putative histone acetyltransferase | | | |
| DDB_G0281625 | | | | DDB_G0281625 | | | | unknown | | | |
| DDB_G0271722 | | | | DDB_G0271722 | | | | putative extracellular matrix protein | | | |
| DDB_G0276013 | | | | DDB_G0276013 | | | | WD40 repeat-containing protein, cyclin-like F-box containing protein | | | |
| DDB_G0295675 | | | | DDB_G0295675 | | | | agglutinin domain-containing protein, leucine-rich repeat-containing protein (LRR), putative transmembrane protein | | | |
| DDB_G0285773 | | | | DDB_G0285773 | | | | putative transmembrane protein | | | |
| DDB_G0285775 | | | | DDB_G0285775 | | | | putative transmembrane protein | | | |
| DDB_G0285631 | | | | DDB_G0285631 | | | | putative transmembrane protein | | | |
| DDB_G0287477 | | | | DDB_G0287477 | | | | putative transmembrane protein | | | |
| DDB_G0287609 | | | | DDB_G0287609 | | | | alpha/beta hydrolase fold-3 domain-containing protein | | | |
| DDB_G0269944 | | | | DDB_G0269944 | | | | unknown | | | |
| DDB_G0281673 | | | | DDB_G0281673 | | | | short-chain dehydrogenase/reductase (SDR) family protein | | | |
| DDB_G0268426 | | | | zntA | | | | zinc transporter, zinc/iron permease | | | |
| DDB_G0288011 | | | | DDB_G0288011 | | | | methyltransferase type 11 domain-containing protein | | | |
| DDB_G0290343 | | | | sodE | | | | putative cytoplasmatic superoxide dismutase, superoxide dismutase | | | |
| DDB_G0281461 | | | | sodF | | | | superoxide dismutase | | | |
| DDB_G0290113 | | | | DDB_G0290113 | | | | B-box zinc finger-containing protein, FNIP repeat-containing protein | | | |
| DDB_G0279949 | | | | DDB_G0279949 | | | | AhpC/TSA family protein | | | |
| DDB_G0289867 | | | | pex1 | | | | peroxin 1, peroxisomal biogenesis factor 1 | | | |
| DDB_G0284209 | | | | DDB_G0284209 | | | | short-chain dehydrogenase/reductase (SDR) family protein | | | |
| DDB_G0284207 | | | | DDB_G0284207 | | | | short-chain dehydrogenase/reductase (SDR) family protein | | | |
| DDB_G0288591 | | | | DDB_G0288591 | | | | short-chain dehydrogenase/reductase (SDR) family protein | | | |
| DDB_G0269340 | | | | DDB_G0269340 | | | | short-chain dehydrogenase/reductase (SDR) family protein | | | |
| DDB_G0283553 | | | | DDB_G0283553 | | | | patatin family protein | | | |
| DDB_G0283595 | | | | DDB_G0283595 | | | | patatin family protein | | | |
| DDB_G0293604 | | | | DDB_G0293604 | | | | short-chain dehydrogenase/reductase (SDR) family protein | | | |
| DDB_G0272094 | | | | DDB_G0272094 | | | | unknown | | | |
| DDB_G0270788 | | | | DDB_G0270788 | | | | unknown | | | |
| DDB_G0295695 | | | | DDB_G0295695 | | | | unknown | | | |
| DDB_G0271062 | | | | DDB_G0271062 | | | | FNIP repeat-containing protein | | | |
| DDB_G0292740 | | | | DDB_G0292740 | | | | type A von Willebrand factor (VWFA) domain-containing protein, vault protein inter-alpha-trypsin (VIT) domain-containing protein | | | |
| DDB_G0270212 | | | | DDB_G0270212 | | | | galactose-binding domain-containing protein | | | |
| DDB_G0287765 | | | | DDB_G0287765 | | | | NADH:flavin oxidoreductase/NADH oxidase domain-containing protein | | | |
| DDB_G0286923 | | | | DDB_G0286923 | | | | NADH:flavin oxidoreductase/NADH oxidase domain-containing protein | | | |
| DDB_G0293114 | | | | DDB_G0293114 | | | | NADH:flavin oxidoreductase/NADH oxidase domain-containing protein | | | |
| DDB_G0275559 | | | | DDB_G0275559 | | | | glucose/ribitol dehydrogenase family protein, short-chain dehydrogenase/reductase (SDR) family protein | | | |
| DDB_G0283723 | | | | DDB_G0283723 | | | | pyridoxal phosphate-dependent decarboxylase family protein | | | |
| DDB_G0290885 | | | | DDB_G0290885 | | | | unknown | | | |
| DDB_G0270518 | | | | DDB_G0270518 | | | | unknown | | | |
| DDB_G0269436 | | | | DDB_G0269436 | | | | unknown | | | |
| DDB_G0267728 | | | | DDB_G0267728 | | | | unknown | | | |
| DDB_G0268332 | | | | DDB_G0268332 | | | | unknown | | | |
| DDB_G0272614 | | | | DDB_G0272614 | | | | DUF1794 family protein | | | |
| DDB_G0281113 | | | | DDB_G0281113 | | | | unknown | | | |
| DDB_G0272628 | | | | DDB_G0272628 | | | | zinc-containing alcohol dehydrogenase (ADH) | | | |
| DDB_G0274085 | | | | DDB_G0274085 | | | | zinc-containing alcohol dehydrogenase (ADH) | | | |
| DDB_G0273155 | | | | DDB_G0273155 | | | | zinc-containing alcohol dehydrogenase (ADH) | | | |
| DDB_G0273921 | | | | DDB_G0273921 | | | | zinc-containing alcohol dehydrogenase (ADH) | | | |
| DDB_G0271680 | | | | DDB_G0271680 | | | | zinc-containing alcohol dehydrogenase (ADH) | | | |
| DDB_G0271740 | | | | DDB_G0271740 | | | | zinc-containing alcohol dehydrogenase (ADH) | | | |
| DDB_G0271780 | | | | DDB_G0271780 | | | | zinc-containing alcohol dehydrogenase (ADH) | | | |
| DDB_G0272280 | | | | DDB_G0272280 | | | | AhpC/TSA family protein | | | |
| DDB_G0291764 | | | | DDB_G0291764 | | | | ribonucleotide reductase-related | | | |
| DDB_G0287987 | | | | elp | | | | elongation factor 2-like protein | | | |
| DDB_G0292918 | | | | DDB_G0292918 | | | | glycoside hydrolase family 47 protein, mannosyl-oligosaccharide 1, 2-alpha-mannosidase | | | |
| DDB_G0284395 | | | | DDB_G0284395 | | | | glycoside hydrolase family 47 protein, mannosyl-oligosaccharide 1, 2-alpha-mannosidase | | | |
| DDB_G0284391 | | | | DDB_G0284391 | | | | glycoside hydrolase family 47 protein, mannosyl-oligosaccharide 1, 2-alpha-mannosidase | | | |
| DDB_G0278655 | | | | DDB_G0278655 | | | | glucose/ribitol dehydrogenase family protein, short-chain dehydrogenase/reductase (SDR) family protein | | | |
| DDB_G0286845 | | | | DDB_G0286845 | | | | glucose/ribitol dehydrogenase family protein, short-chain dehydrogenase/reductase (SDR) family protein | | | |
| DDB_G0284393 | | | | DDB_G0284393 | | | | glycoside hydrolase family 47 protein, mannosyl-oligosaccharide 1, 2-alpha-mannosidase | | | |
| DDB_G0273623 | | | | hspE-2 | | | | heat shock cognate protein Hsc70-2, heat shock protein | | | |
| DDB_G0272466 | | | | DDB_G0272466 | | | | glucose/ribitol dehydrogenase family protein, short-chain dehydrogenase/reductase (SDR) family protein | | | |
| DDB_G0289129 | | | | DDB_G0289129 | | | | glucose/ribitol dehydrogenase family protein, short-chain dehydrogenase/reductase (SDR) family protein | | | |
| DDB_G0271914 | | | | DDB_G0271914 | | | | CMP/dCMP deaminase, zinc-binding domain-containing protein | | | |
| DDB_G0285769 | | | | DDB_G0285769 | | | | unknown | | | |
| DDB_G0288711 | | | | DDB_G0288711 | | | | IPT/TIG domain-containing protein | | | |
| DDB_G0290883 | | | | DDB_G0290883 | | | | RING zinc finger-containing protein, TRAF-type zinc finger-containing protein, meprin and TRAF homology (MATH) domain-containing protein | | | |
| DDB_G0273199 | | | | DDB_G0273199 | | | | putative protein tyrosine phosphatase, dual specificity | | | |
| DDB_G0269040 | | | | DDB_G0269040 | | | | C-type lectin domain-containing protein, EGF-like domain-containing protein, IPT/TIG domain-containing protein | | | |
| DDB_G0273105 | | | | DDB_G0273105 | | | | cytochrome b5 domain-containing protein, delta 9 fatty acid desaturase, stearoyl-CoA 9-desaturase | | | |
| DDB_G0278593 | | | | exdl2B | | | | 3'-5' exonuclease domain-containing protein | | | |
| DDB_G0268178 | | | | jcdB | | | | transcription factor jumonji, jmjC domain-containing protein | | | |
| DDB_G0272004 | | | | p2xA | | | | P2X receptor, P2X receptor ATP-gated ion channel, purinergic receptor | | | |
| DDB_G0275293 | | | | p2xB | | | | putative P2X receptor, putative purinergic receptor | | | |
| DDB_G0288335 | | | | p2xD | | | | putative P2X receptor, putative purinergic receptor | | | |
| cblA-1 | | | | cblA-1 | | | | contains putative CBL proto-oncogene N-terminus%2C EF-hand-like domain%3B Cbl adaptor proteins are RING-type E3 ubiquitin ligases%3B%3Cbr%3E there is a second copy of this gene%2C %3Ca href%3D%22%2Fdb%2Fcgi-bin%2Fgene_page.pl?primary_id%3DDDB_G0273609%22%3E%3Ci%3EcblA-2%3C%2Fi%3E%3C%2Fa%3E | | | |
| cblA-2 | | | | cblA-2 | | | | contains putative CBL proto-oncogene N-terminus%2C EF-hand-like domain%3B Cbl adaptor proteins are RING-type E3 ubiquitin ligases%3B there is a second copy of this gene%2C %3Ca href%3D%22%2Fdb%2Fcgi-bin%2Fgene_page.pl?primary_id%3DDDB_G0273141%22%3E%3Ci%3EcblA-1%3C%2Fi%3E%3C%2Fa%3E | | | |
| DDB_G0276365 | | | | DDB_G0276365 | | | | glycoside hydrolase family 18 protein | | | |
| DDB_G0286795 | | | | DDB_G0286795 | | | | unknown | | | |
| DDB_G0273291 | | | | ddo-2 | | | | D-amino-acid:oxygen oxidoreductase (deaminating), D-aspartate oxidase | | | |
| DDB_G0271958 | | | | DDB_G0271958 | | | | putative glutathione S-transferase | | | |
| DDB_G0285893 | | | | DDB_G0285893 | | | | unknown | | | |
| DDB_G0287859 | | | | DDB_G0287859 | | | | Zinc finger, N-recognin domain-containing protein | | | |
| DDB_G0288093 | | | | DDB_G0288093 | | | | RING zinc finger-containing protein, Zinc finger, N-recognin domain-containing protein | | | |
| DDB_G0285449 | | | | DDB_G0285449 | | | | putative protein tyrosine phosphatase, dual specificity | | | |
| DDB_G0285909 | | | | DDB_G0285909 | | | | putative protein tyrosine phosphatase | | | |
| DDB_G0285965 | | | | DDB_G0285965 | | | | AWS domain-containing protein | | | |
| DDB_G0278587 | | | | DDB_G0278587 | | | |  | | | |
| DDB_G0292548 | | | | DDB_G0292548 | | | | Pol. | | | |
| DDB_G0271996 | | | | DDB_G0271996 | | | | FNIP repeat-containing protein | | | |
| DDB_G0287491 | | | | DDB_G0287491 | | | | paramecium surface antigen repeat-containing protein | | | |
| DDB_G0287483 | | | | cnrG | | | | paramecium surface antigen repeat-containing protein, putative cell number regulator | | | |
| DDB_G0287471 | | | | DDB_G0287471 | | | | paramecium surface antigen repeat-containing protein | | | |
| DDB_G0295701 | | | | DDB_G0295701 | | | | paramecium surface antigen repeat-containing protein | | | |
| DDB_G0289145 | | | | pde7 | | | | cAMP phosphodiesterase, cGMP phosphodiesterase | | | |
| DDB_G0289925 | | | | DDB_G0289925 | | | | N, N-dimethylaniline, NADPH:oxygen oxidoreductase, N-oxide-forming, flavin-containing monooxygenase | | | |
| DDB_G0269092 | | | | DDB_G0269092 | | | | unknown | | | |
| DDB_G0268828 | | | | DDB_G0268828 | | | | unknown | | | |
| DDB_G0269008 | | | | DDB_G0269008 | | | | unknown | | | |
| DDB_G0281407 | | | | DDB_G0281407 | | | | IPT/TIG domain-containing protein | | | |
| DDB_G0288869 | | | | DDB_G0288869 | | | | EGF-like domain-containing protein | | | |
| DDB_G0288813 | | | | DDB_G0288813 | | | | EGF-like domain-containing protein | | | |
| DDB_G0288815 | | | | DDB_G0288815 | | | | EGF-like domain-containing protein | | | |
| DDB_G0274059 | | | | cofC-2 | | | | cofilin-2 | | | |
| DDB_G0272903 | | | | DDB_G0272903 | | | | unknown | | | |
| DDB_G0273953 | | | | DDB_G0273953 | | | | unknown | | | |
| DDB_G0272600 | | | | DDB_G0272600 | | | | unknown | | | |
| DDB_G0272901 | | | | DDB_G0272901 | | | | EGF-like domain-containing protein | | | |
| DDB_G0273955 | | | | DDB_G0273955 | | | | EGF-like domain-containing protein | | | |
| DDB_G0273043 | | | | DDB_G0273043 | | | | EGF-like domain-containing protein | | | |
| DDB_G0273961 | | | | DDB_G0273961 | | | | EGF-like domain-containing protein | | | |
| DDB_G0268368 | | | | mybAA | | | | myb domain-containing protein | | | |
| DDB_G0272897 | | | | DDB_G0272897 | | | | EGF-like domain-containing protein | | | |
| DDB_G0273965 | | | | DDB_G0273965 | | | | EGF-like domain-containing protein | | | |
| DDB_G0272805 | | | | DDB_G0272805 | | | | EGF-like domain-containing protein | | | |
| DDB_G0273967 | | | | DDB_G0273967 | | | | EGF-like domain-containing protein | | | |
| DDB_G0273775 | | | | cinD-2 | | | | putative helix-turn-helix transcription factor | | | |
| DDB_G0279721 | | | | ubqH | | | | ubiquitin | | | |
| DDB_G0269458 | | | | ubqJ | | | | ubiquitin | | | |
| DDB_G0274077 | | | | drnA-2 | | | | dicer-like protein, putative RNase III | | | |
| DDB_G0280587 | | | | ubqL | | | | ubiquitin | | | |
| DDB_G0280589 | | | | ubqM | | | | ubiquitin | | | |
| DDB_G0292984 | | | | ubqN | | | | ubiquitin | | | |
| DDB_G0292908 | | | | ubqO | | | | ubiquitin | | | |
| DDB_G0286031 | | | | ubqP | | | | ubiquitin domain-containing protein | | | |
| DDB_G0293836 | | | | DDB_G0293836 | | | | unknown | | | |
| DDB_G0295691 | | | | DDB_G0295691 | | | | transmembrane protein | | | |
| DDB_G0269418 | | | | DDB_G0269418 | | | | osmotically inducible family protein | | | |
| DDB_G0272783 | | | | DDB_G0272783 | | | | major facilitator superfamily protein | | | |
| DDB_G0273221 | | | | DDB_G0273221 | | | | unknown | | | |
| DDB_G0272592 | | | | DDB_G0272592 | | | | EGF-like domain-containing protein | | | |
| DDB_G0273975 | | | | DDB_G0273975 | | | | EGF-like domain-containing protein | | | |
| DDB_G0288003 | | | | DDB_G0288003 | | | | EGF-like domain-containing protein | | | |
| DDB_G0280867 | | | | DDB_G0280867 | | | | EGF-like domain-containing protein | | | |
| DDB_G0271824 | | | | DDB_G0271824 | | | | hssA/2C/7E family protein | | | |
| DDB_G0271716 | | | | DDB_G0271716 | | | | hssA/2C/7E family protein | | | |
| DDB_G0271718 | | | | DDB_G0271718 | | | | hssA/2C/7E family protein | | | |
| DDB_G0271822 | | | | DDB_G0271822 | | | | hssA/2C/7E family protein | | | |
| DDB_G0271712 | | | | DDB_G0271712 | | | | coiled-coil family protein, hssA/2C/7E family protein | | | |
| DDB_G0271818 | | | | DDB_G0271818 | | | | hssA/2C/7E family protein | | | |
| DDB_G0277291 | | | | DDB_G0277291 | | | | EGF-like domain-containing protein | | | |
| DDB_G0277731 | | | | DDB_G0277731 | | | | EGF-like domain-containing protein | | | |
| DDB_G0286867 | | | | DDB_G0286867 | | | | EGF-like domain-containing protein | | | |
| DDB_G0284615 | | | | DDB_G0284615 | | | | EGF-like domain-containing protein | | | |
| DDB_G0271820 | | | | DDB_G0271820 | | | | short-chain dehydrogenase/reductase (SDR) family protein | | | |
| DDB_G0271720 | | | | DDB_G0271720 | | | | short-chain dehydrogenase/reductase (SDR) family protein | | | |
| DDB_G0273189 | | | | DDB_G0273189 | | | | transmembrane protein | | | |
| DDB_G0273943 | | | | CYP508A1-2 | | | | cytochrome P450 family protein | | | |
| DDB_G0273941 | | | | CYP508A3-2 | | | | cytochrome P450 family protein | | | |
| DDB_G0272610 | | | | DDB_G0272610 | | | | unknown | | | |
| DDB_G0274031 | | | | DDB_G0274031 | | | | unknown | | | |
| DDB_G0272642 | | | | DDB_G0272642 | | | | FNIP repeat-containing protein | | | |
| DDB_G0274063 | | | | DDB_G0274063 | | | | FNIP repeat-containing protein | | | |
| DDB_G0292006 | | | | DDB_G0292006 | | | | GCN5-related N-acetyltransferase | | | |
| DDB_G0274057 | | | | cofD-2 | | | | cofilin | | | |
| DDB_G0280997 | | | | DDB_G0280997 | | | | zinc-containing alcohol dehydrogenase (ADH) | | | |
| DDB_G0290037 | | | | DDB_G0290037 | | | | EGF-like domain-containing protein | | | |
| DDB_G0271032 | | | | DDB_G0271032 | | | | EGF-like domain-containing protein | | | |
| DDB_G0295687 | | | | dph2 | | | | diphthamide biosynthesis protein 2 | | | |
| DDB_G0269918 | | | | mpl1 | | | | leucine-rich repeat-containing protein (LRR), putative protein tyrosine phosphatase, dual specificity | | | |
| DDB_G0295705 | | | | DDB_G0295705 | | | | unknown | | | |
| DDB_G0295707 | | | | gtaP | | | | GATA zinc finger domain-containing protein 16, putative GATA-binding transcription factor | | | |
| DDB_G0269086 | | | | DDB_G0269086 | | | | alpha/beta hydrolase fold-1 domain-containing protein | | | |
| DDB_G0295703 | | | | DDB_G0295703 | | | | pentapeptide repeat-containing protein | | | |
| DDB_G0270986 | | | | tspC | | | | tetraspanin family protein | | | |
| DDB_G0270682 | | | | tspD | | | | tetraspanin family protein | | | |
| DDB_G0268590 | | | | DDB_G0268590 | | | | unknown | | | |
| DDB_G0295747 | | | | DDB_G0295747 | | | | unknown | | | |
| DDB_G0293492 | | | | DDB_G0293492 | | | | glycoside hydrolase family 25 protein | | | |
| DDB_G0295749 | | | | DDB_G0295749 | | | | deoxyribonuclease II family protein | | | |
| DDB_G0276439 | | | | DDB_G0276439 | | | | glycoside hydrolase family 25 protein | | | |
| DDB_G0295751 | | | | DDB_G0295751 | | | | unknown | | | |
| DDB_G0270604 | | | | DDB_G0270604 | | | | unknown | | | |
| DDB_G0278243 | | | | DDB_G0278243 | | | | unknown | | | |
| DDB_G0268848 | | | | DDB_G0268848 | | | | putative ATP binding protein | | | |
| DDB_G0268850 | | | | DDB_G0268850 | | | | putative ATP binding protein | | | |
| DDB_G0295779 | | | | DDB_G0295779 | | | | EGF-like domain-containing protein | | | |
| DDB_G0295785 | | | | DDB_G0295785 | | | | cyclin-related 2 family protein | | | |
| DDB_G0295791 | | | | DDB_G0295791 | | | | unknown | | | |
| DDB_G0290931 | | | | DDB_G0290931 | | | | RING zinc finger-containing protein, TRAF-type zinc finger-containing protein, meprin and TRAF homology (MATH) domain-containing protein | | | |
| DDB_G0295799 | | | | DDB_G0295799 | | | | unknown | | | |
| DDB_G0295801 | | | | DDB_G0295801 | | | | B_lectin domain-containing protein | | | |
| DDB_G0295803 | | | | DDB_G0295803 | | | | heat shock protein Hsp20 domain-containing protein | | | |
| DDB_G0285613 | | | | DDB_G0285613 | | | | DNAJ heat shock N-terminal domain-containing protein | | | |
| DDB_G0285729 | | | | DDB_G0285729 | | | | unknown | | | |
| DDB_G0295807 | | | | DDB_G0295807 | | | | glucose/ribitol dehydrogenase family protein, short-chain dehydrogenase/reductase (SDR) family protein | | | |
| DDB_G0273453 | | | | DDB_G0273453 | | | | unknown | | | |
| DDB_G0295811 | | | | DDB_G0295811 | | | | thioredoxin fold domain-containing protein | | | |
| DDB_G0275713 | | | | DDB_G0275713 | | | | DUF781 family protein | | | |
| DDB_G0291576 | | | | DDB_G0291576 | | | | DUF781 family protein | | | |
| DDB_G0289303 | | | | DDB_G0289303 | | | | SET domain-containing protein | | | |
| DDB_G0295813 | | | | DDB_G0295813 | | | | unknown | | | |
| DDB_G0276755 | | | | DDB_G0276755 | | | | unknown | | | |
| DDB_G0271132 | | | | DDB_G0271132 | | | | unknown | | | |
| DDB_G0295821 | | | | DDB_G0295821 | | | | putative transmembrane protein | | | |
| DDB_G0291702 | | | | CYP520B1 | | | | cytochrome P450 family protein | | | |
| DDB_G0295757 | | | | DDB_G0295757 | | | | IPT/TIG domain-containing protein | | | |
| DDB_G0270964 | | | | DDB_G0270964 | | | |  | | | |
| DDB_G0295763 | | | | DDB_G0295763 | | | |  | | | |
| DDB_G0270962 | | | | DDB_G0270962 | | | | IPT/TIG domain-containing protein | | | |
| DDB_G0295771 | | | | DDB_G0295771 | | | | unknown | | | |
| DDB_G0295773 | | | | DDB_G0295773 | | | | DUF1077 family protein | | | |
| DDB_G0282641 | | | | DDB_G0282641 | | | | unknown | | | |
| DDB_G0275215 | | | | DDB_G0275215 | | | | ankyrin repeat-containing protein | | | |
| DDB_G0292896 | | | | eloA | | | | GNS1/SUR4 family protein, long chain fatty acid elongase | | | |
| DDB_G0284701 | | | | DDB_G0284701 | | | | mandelate racemase/muconate lactonizing enzyme domain-containing protein | | | |
| DDB_G0284853 | | | | DDB_G0284853 | | | | B-box zinc finger-containing protein, PHR domain-containing protein, RING zinc finger-containing protein | | | |
| DDB_G0285063 | | | | DDB_G0285063 | | | | putative E3 ubiquitin-protein ligase | | | |
| DDB_G0291191 | | | | DDB_G0291191 | | | | cysteine protease | | | |
| DDB_G0292828 | | | | DDB_G0292828 | | | |  | | | |
| DDB_G0295723 | | | | DDB_G0295723 | | | | IPT/TIG domain-containing protein | | | |
| DDB_G0295727 | | | | DDB_G0295727 | | | | unknown | | | |
| DDB_G0286115 | | | | DDB_G0286115 | | | | phosphatidylinositol-4-phosphate 5-kinase (PIP5K) family protein | | | |
| DDB_G0295733 | | | | DDB_G0295733 | | | | thioesterase superfamily protein | | | |
| DDB_G0286969 | | | | DDB_G0286969 | | | | MIF4G-like, type 3 domain-containing protein, putative eukaryotic translation initiation factor 4 gamma (eIF4G), type A von Willebrand factor (VWFA) domain-containing protein | | | |
| DDB_G0295735 | | | | DDB_G0295735 | | | | transmembrane protein | | | |
| DDB_G0289351 | | | | DDB_G0289351 | | | | EGF-like domain-containing protein | | | |
| DDB_G0289887 | | | | DDB_G0289887 | | | | unknown | | | |
| DDB_G0288219 | | | | DDB_G0288219 | | | |  | | | |
| DDB_G0292822 | | | | DDB_G0292822 | | | |  | | | |
| DDB_G0292826 | | | | DDB_G0292826 | | | |  | | | |
| DDB_G0295737 | | | | DDB_G0295737 | | | | PAP/25A-associated domain-containing protein, nucleotidyltransferase domain-containing protein | | | |
| DDB_G0290651 | | | | DDB_G0290651 | | | | unknown | | | |
| DDB_G0295741 | | | | DDB_G0295741 | | | | putative acyl-CoA N-acyltransferase | | | |
| DDB_G0280565 | | | | DDB_G0280565 | | | | calponin homology (CH) domain-containing protein | | | |
| DDB_G0295715 | | | | DDB_G0295715 | | | | NOL1/NOP2/Sun family protein | | | |
| DDB_G0292528 | | | | DDB_G0292528 | | | | pirin family protein | | | |
| DDB_G0293162 | | | | DDB_G0293162 | | | | unknown | | | |
| DDB_G0295841 | | | | DDB_G0295841 | | | | FNIP repeat-containing protein | | | |
| DDB_G0295843 | | | | DDB_G0295843 | | | | putative eukaryotic translation initiation factor 2-alpha kinase | | | |
| DDB_G0271532 | | | | DDB_G0271532 | | | | pyridine nucleotide-disulphide oxidoreductase, NAD-binding region domain-containing protein | | | |
| DDB_G0268556 | | | | psiE | | | | PA14 domain-containing protein | | | |
| DDB_G0295829 | | | | DDB_G0295829 | | | | EGF-like domain-containing protein, leucine-rich repeat-containing protein (LRR) | | | |
| DDB_G0293536 | | | | cupD | | | | calcium up-regulated protein, ricin B lectin domain-containing protein | | | |
| DDB_G0284743 | | | | 4cl3 | | | | 4-coumarate-CoA ligase | | | |
| DDB_G0290563 | | | | cupH | | | | cup family protein, ricin B lectin domain-containing protein | | | |
| DDB_G0277959 | | | | cupJ | | | | cup family protein, ricin B lectin domain-containing protein | | | |
| DDB_G0292914 | | | | DDB_G0292914 | | | | DEAD-box RNA helicase | | | |
| DDB_G0289365 | | | | gghB | | | | gamma-glutamyl hydrolase, peptidase C26 family protein | | | |
| DDB_G0277133 | | | | DDB_G0277133 | | | | SNF7 family protein | | | |
| DDB_G0273583 | | | | gtf2e1-2 | | | | transcription factor IIE | | | |
| DDB_G0273991 | | | | maoC-2 | | | | amine oxidase (flavin-containing), monoamine oxidase | | | |
| DDB_G0272662 | | | | DDB_G0272662 | | | | putative protein tyrosine phosphatase, dual specificity | | | |
| DDB_G0273875 | | | | cf50-2 | | | | component of the counting factor (CF) complex | | | |
| DDB_G0271664 | | | | DDB_G0271664 | | | | EXS domain-containing protein, SPX domain-containing protein, SPX/EXS domain-containing protein 1 | | | |
| DDB_G0290647 | | | | DDB_G0290647 | | | | EXS domain-containing protein, SPX domain-containing protein, SPX/EXS domain-containing protein 5 | | | |
| DDB_G0290617 | | | | DDB_G0290617 | | | | FNIP repeat-containing protein | | | |
| DDB_G0274481 | | | | DDB_G0274481 | | | | EXS domain-containing protein, SPX domain-containing protein, SPX/EXS domain-containing protein 2 | | | |
| DDB_G0274023 | | | | capA-2 | | | | cAMP-binding protein | | | |
| DDB_G0290639 | | | | DDB_G0290639 | | | | FNIP repeat-containing protein | | | |
| DDB_G0289975 | | | | DDB_G0289975 | | | | unknown | | | |
| DDB_G0290005 | | | | DDB_G0290005 | | | | myotubularin-related protein | | | |
| DDB_G0273093 | | | | DDB_G0273093 | | | | heat shock protein 70 (Hsp70) family member | | | |
| DDB_G0286927 | | | | nedd8l2 | | | | neddylin-like protein, ubiquitin-like protein | | | |
| DDB_G0273919 | | | | dscA-2 | | | | discoidin I, A chain, discoidin I, alpha chain | | | |
| DDB_G0273887 | | | | dscD-2 | | | | discoidin I, D chain | | | |
| DDB_G0273809 | | | | DDB_G0273809 | | | | ZIP family zinc transporter, zinc/iron permease | | | |
| DDB_G0281707 | | | | DDB_G0281707 | | | | putative transmembrane protein | | | |
| DDB_G0285003 | | | | aifC | | | | putative apoptosis inducing factor | | | |
| DDB_G0273089 | | | | coq10-1 | | | | putative coenzyme Q-binding protein | | | |
| DDB_G0274511 | | | | DDB_G0274511 | | | | carboxypeptidase, vitellogenic-like, peptidase S10 family protein, serine carboxypeptidase | | | |
| DDB_G0274941 | | | | omt2 | | | | O-methyltransferase family 2 protein | | | |
| DDB_G0275013 | | | | omt4 | | | | O-methyltransferase family 2 protein | | | |
| DDB_G0282591 | | | | omt7 | | | | O-methyltransferase family 2 protein | | | |
| DDB_G0273615 | | | | fpaB-2 | | | | cytosolic glycoprotein FP21, ubiquitin ligase subunit SKP1 | | | |
| DDB_G0273903 | | | | irlF-2 | | | | IRE family protein kinase, putative protein serine/threonine kinase | | | |
| DDB_G0295849 | | | | DDB_G0295849 | | | | rhomboid family protein | | | |
| DDB_G0270718 | | | | DDB_G0270718 | | | | EGF-like domain-containing protein | | | |
| DDB_G0270714 | | | | DDB_G0270714 | | | | phytanoyl-CoA dioxygenase family protein | | | |
| DDB_G0286883 | | | | DDB_G0286883 | | | | NUDIX hydrolase family protein, dinucleoside polyphosphate hydrolase | | | |
| DDB_G0273471 | | | | dpp3-1 | | | | dipeptidyl-peptidase III | | | |
| DDB_G0273193 | | | | DDB_G0273193 | | | | PH domain-containing protein | | | |
| DDB_G0273857 | | | | irlB-2 | | | | IRE family protein kinase, putative protein serine/threonine kinase | | | |
| DDB_G0295833 | | | | DDB_G0295833 | | | | short-chain dehydrogenase/reductase (SDR) family protein | | | |
| DDB_G0295835 | | | | dhkI-2 | | | | HisK family protein kinase, histidine kinase, protein kinase, Atypical group | | | |
| DDB_G0273561 | | | | hspF-2 | | | | heat shock protein Hsp20 domain-containing protein, putative alpha-crystallin-type heat shock protein | | | |
| DDB_G0273935 | | | | rabF1-2 | | | | Rab GTPase | | | |
| DDB_G0273107 | | | | fip-1 | | | | filament-interacting protein | | | |
| DDB_G0273717 | | | | aslD-2 | | | | putative acetyl-CoA synthetase | | | |
| DDB_G0273709 | | | | aslG-2 | | | | putative acetyl-CoA synthetase | | | |
| DDB_G0273697 | | | | aslC-2 | | | | putative acetyl-CoA synthetase | | | |
| DDB_G0273695 | | | | aslI-2 | | | | putative acetyl-CoA synthetase | | | |
| DDB_G0273689 | | | | aslK-2 | | | | putative acetyl-CoA synthetase | | | |
| DDB_G0273687 | | | | aslL-2 | | | | putative acetyl-CoA synthetase | | | |
| DDB_G0272300 | | | | aslE | | | | putative acetyl-CoA synthetase | | | |
| DDB_G0273361 | | | | DDB_G0273361 | | | | HPP family protein | | | |
| DDB_G0273699 | | | | aslO-2 | | | | putative acetyl-CoA synthetase | | | |
| DDB_G0274067 | | | | DDB_G0274067 | | | | phosphatidylinositol-4-phosphate 5-kinase (PIP5K) family protein | | | |
| DDB_G0274033 | | | | fslM-2 | | | | G-protein-coupled receptor (GPCR) family protein, frizzled and smoothened-like protein | | | |
| DDB_G0271430 | | | | DDB_G0271430 | | | | unkown | | | |
| DDB_G0273573 | | | | DDB_G0273573 | | | | AAA+ ATPase, core domain-containing protein | | | |
| DDB_G0273117 | | | | ublcp1-1 | | | | ubiquitin-like domain-containing CTD phosphatase 1 | | | |
| DDB_G0280689 | | | | DDB_G0280689 | | | | IPT/TIG domain-containing protein | | | |
| DDB_G0280835 | | | | DDB_G0280835 | | | | unkown | | | |
| DDB_G0274011 | | | | fslJ-2 | | | | G-protein-coupled receptor (GPCR) family protein, frizzled and smoothened-like protein, putative cell number regulator | | | |
| DDB_G0273825 | | | | rsmE-2 | | | | small GTPase | | | |
| DDB_G0274955 | | | | etnkA | | | | ethanolamine kinase A | | | |
| DDB_G0295847 | | | | DDB_G0295847 | | | | EGF-like domain-containing protein | | | |
| DDB_G0267692 | | | | DDB_G0267692 | | | | EGF-like domain-containing protein | | | |
| DDB_G0269292 | | | | sugt1 | | | | SGS domain-contantaing protein, TPR repeat-containing protein | | | |
| DDB_G0288503 | | | | DDB_G0288503 | | | | arylamine N-acetyltransferase family protein | | | |
| DDB_G0288507 | | | | DDB_G0288507 | | | | arylamine N-acetyltransferase family protein | | | |
| DDB_G0292214 | | | | DDB_G0292214 | | | | protein phosphatase 2C-like domain-containing protein | | | |
| DDB_G0289731 | | | | DDB_G0289731 | | | | N-terminal delta endotoxin domain-containing protein | | | |
| DDB_G0269000 | | | | DDB_G0269000 | | | | unknown | | | |
| DDB_G0295839 | | | | DDB_G0295839 | | | | unknown | | | |
| DDB_G0280097 | | | | DDB_G0280097 | | | | contains a signal peptide%2C a weak PA14 domain%2C and 2 %3Ci%3EDictyostelium%3C%2Fi%3E-specific repeats | | | |
| DDB_G0293832 | | | | DDB_G0293832 | | | |  | | | |
| DDB_G0278529 | | | | DDB_G0278529 | | | | putative 22 kDa peroxisomal membrane protein that may be involved in pore forming activity and may contribute to the unspecific permeability of the organelle membrane | | | |
| cdk9-2 | | | | cdk9-2 | | | | similar to the cell division cycle 2-related protein kinase 7 (CRK7) and other cell division cycle 2-like protein kinases%3B there is a second copy of this gene%2C %3Ca href%3D%22gene_page.pl?primary_id%3DDDB_G0273207%22%3E%3Ci%3Ecdk9-1%3C%2Fi%3E%3C%2Fa%3E | | | |
| DDB_G0275953 | | | | DDB_G0275953 | | | | HYPOTHETICAL 37.8 KDA PROTEIN | | | |
| DDB_G0277999 | | | | DDB_G0277999 | | | | sulfurates the molybdenum cofactor%2C which is essential for xanthine dehydrogenase and aldehyde oxidase | | | |
| DDB_G0272939 | | | | DDB_G0272939 | | | | highly similar to %3Ca href%3D%22%2Fdb%2Fcgi-bin%2Fgene_page.pl?primary_id%3DDDB_G0272881%22%3E%3Ci%3EDDB_G0272881%3C%2Fi%3E%3C%2Fa%3E%3B has also similarity to hypothetical proteins in other organisms | | | |
| DDB_G0274039 | | | | DDB_G0274039 | | | | highly similar to %3Ca href%3D%22%2Fdb%2Fcgi-bin%2Fgene_page.pl?primary_id%3DDDB_G0272939%22%3E%3Ci%3EDDB_G0272939 %3C%2Fi%3E%3C%2Fa%3E%3B has also similarity to hypothetical proteins in other organisms%3B there is a second copy of this gene%2C %3Ca href%3D%22%2Fdb%2Fcgi-bin%2Fgene_page.pl?primary_i | | | |
| DDB_G0272881 | | | | DDB_G0272881 | | | | highly similar to %3Ca href%3D%22%2Fdb%2Fcgi-bin%2Fgene_page.pl?primary_id%3DDDB_G0272939%22%3E%3Ci%3EDDB_G0272939 %3C%2Fi%3E%3C%2Fa%3E%3B has also similarity to hypothetical proteins in other organisms%3B there is a second copy of this gene%2C %3Ca href%3D%22%2Fdb%2Fcgi-bin%2Fgene_page.pl?primary_i | | | |
| ap2a1-2 | | | | ap2a1-2 | | | | there is a second copy of this gene %3Ca href%3D%22%2Fdb%2Fcgi-bin%2Fgene_page.pl?primary_id%3DDDB_G0273439%22%3E%3Ci%3Eap2a1-1%3C%2Fi%3E%3C%2Fa%3E | | | |
| hpdl-1 | | | | hpdl-1 | | | | catalyzes the reaction 4-hydroxyphenylpyruvate %2B O%3Csub%3E2%3C%2Fsub%3E %3C%3D%3E homogentisate %2B CO%3Csub%3E2%3C%2Fsub%3E%3B there is a second copy of this gene%2C %3Ca href%3D%22%2Fdb%2Fcgi-bin%2Fgene_page.pl?primary_id%3DDDB_G0273513%22%3E%3Ci%3Ehpdl-2%3C%2Fi%3E%3C%2Fa%3E | | | |
| hpdl-2 | | | | hpdl-2 | | | | catalyzes the reaction 4-hydroxyphenylpyruvate %2B O%3Csub%3E2%3C%2Fsub%3E %3C%3D%3E homogentisate %2B CO%3Csub%3E2%3C%2Fsub%3E%3B there is a second copy of this gene%2C %3Ca href%3D%22%2Fdb%2Fcgi-bin%2Fgene_page.pl?primary_id%3DDDB_G0273429%22%3E%3Ci%3Ehpdl-1%3C%2Fi%3E%3C%2Fa%3E | | | |
| abpE-2 | | | | abpE-2 | | | | involved in pseudopod formation%3B contains an N-terminal ADF%2Fcofilin-like actin binding domain and a c-terminal SH3 domain%3B there is a second copy of this gene%2C %3Ca href%3D%22%2Fdb%2Fcgi-bin%2Fgene_page.pl?primary_id%3DDDB_G0273447%22%3E%3Ci%3EabpE-1%3C%2Fi%3E%3C%2Fa%3E | | | |
| nit1-1 | | | | nit1-1 | | | | similar to mammalian NIT1%3B there is a second copy of this gene%2C %3Ca href%3D%22%2Fdb%2Fcgi-bin%2Fgene_page.pl?primary_id%3DDDB_G0273519%22%3E%3Ci%3Enit1-2%3C%2Fi%3E%3C%2Fa%3E | | | |
| nit1-2 | | | | nit1-2 | | | | similar to mammalian NIT1%3B there is a second copy of this gene%2C %3Ca href%3D%22%2Fdb%2Fcgi-bin%2Fgene_page.pl?primary_id%3DDDB_G0273457%22%3E%3Ci%3Enit1-1%3C%2Fi%3E%3C%2Fa%3E | | | |
| rmp | | | | rmp | | | | similar to the human unconventional prefoldin RPB5 interactor a putative scaffolding protein with roles in transcription and ubiquitination | | | |
| ctu2 | | | | ctu2 | | | | ortholog of S. pombe ctu2 (cytosolic thiouridylase subunit 2)%3B with ctu1%2C required for thiolation of the uridine at the wobble position of Lys(UUU) and Glu(UUC) tRNAs | | | |
| DDB_G0302530 | | | | DDB_G0302530 | | | |  | | | |
| nfyC-2 | | | | nfyC-2 | | | | there is a second copy of this gene%2C %3Ca href%3D%22gene_page.pl?primary_id%3DDDB_G0273479%22%3E%3Ci%3EnfyC-1%3C%2Fi%3E%3C%2Fa%3E | | | |
| gemin1 | | | | gemin1 | | | | putative ortholog of Gemin 1%2FSNM1 (Survival of motor neuron protein 1)%2C a component of Cajal bodies (CBs) and Gems%2C nuclear organelles responsible for spliceosomal small nuclear ribonucleoprotein (snRNP) biogenesis | | | |
| gemin5 | | | | gemin5 | | | | ortholog of Gemin 5%2C a component of Cajal bodies (CBs) and Gems%2C nuclear organelles responsible for spliceosomal small nuclear ribonucleoprotein (snRNP) biogenesis | | | |
| DDB_G0273149 | | | | DDB_G0273149 | | | | functions in nuclear protein import via a substrate-importin alpha-beta transport complex that passes though the nuclear pore complexes (NPC)%3B contains a N-terminal importin beta binding domain (IBB domain)%3B there is a second copy of this gene%2C %3Ca href%3D%22%2Fdb%2Fcgi-bin%2Fgene_page.pl?pri | | | |
| DDB_G0273147 | | | | DDB_G0273147 | | | | has similarity to the %3C%2Fi%3ES. cerevisisae%3C%2Fi%3E ATP10%2C a mitochondrial inner membrane protein required for assembly of the mitochondrial F1F0 ATP synthase%3B there is a second copy of this gene%2C %3Ca href%3D%22%2Fdb%2Fcgi-bin%2Fgene_page.pl?primary_id%3DDDB_G0273597%22%3E%3Ci%3EDDB_G027 | | | |
| DDB_G0283091 | | | | DDB_G0283091 | | | |  | | | |
| DDB_G0277083 | | | | DDB_G0277083 | | | | there is an almost identical %3Ca href%3D%22%2Fdb%2Fcgi-bin%2Fgene_page.pl?primary_id%3DDDB_G0283125%22%3E%3Ci%3E gene%3C%2Fi%3E%3C%2Fa%3E on chromosome 4 | | | |
| DDB_G0276051 | | | | DDB_G0276051 | | | |  | | | |
| DDB_G0302588 | | | | DDB_G0302588 | | | | similar to a D. purpureum protein | | | |
| DDB_G0273075 | | | | DDB_G0273075 | | | | there is a second copy of this gene%2C %3Ca href%3D%22gene_page.pl?primary_id%3DDDB_G0273765%22%3E%3Ci%3EDDB_G0273765%3C%2Fi%3E%3C%2Fa%3E | | | |
| DDB_G0273765 | | | | DDB_G0273765 | | | | there is a second copy of this gene%2C %3Ca href%3D%22gene_page.pl?primary_id%3DDDB_G0273075%22%3E%3Ci%3EDDB_G0273075%3C%2Fi%3E%3C%2Fa%3E | | | |
| DDB_G0276179 | | | | DDB_G0276179 | | | | Similar to Dictyostelium discoideum (Slime mold). Myosin regulatory light chain (RMLC). | | | |
| DDB_G0291580 | | | | DDB_G0291580 | | | | member of a small Dictyostelium gene family | | | |
| DDB_G0291578 | | | | DDB_G0291578 | | | | member of a small Dictyostelium gene family%3B missing the carboxyl half compared to related proteins | | | |
| DDB_G0279353 | | | | DDB_G0279353 | | | |  | | | |
| trappc10-2 | | | | trappc10-2 | | | | ortholog of the trafficking protein particle complex subunit 10%2C part of the multisubunit TRAPP (transport protein particle) complex%3B there is a second copy of this gene%2C %3Ca href%3D%22gene_page.pl?primary_id%3DDDB_G0273209%22%3E%3Ci%3Etrappc10-1%3C%2Fi%3E%3C%2Fa%3E | | | |
| cwc15-2 | | | | cwc15-2 | | | | ortholog of S. pombe Cwf15 and S. cerevisiae Cwc15%2C thought to be non-essential components of an mRNA splicing complex%3B there is a second copy of this gene%2C %3Ca href%3D%22%2Fdb%2Fcgi-bin%2Fgene_page.pl?primary_id%3DDDB_G0273383%22%3E%3Ci%3Ecwc15-1%3C%2Fi%3E%3C%2Fa%3E | | | |
|  | | | | | | | | |  | | |
| **D. purpureum genes with close paralogs in D. discoideum** | | | | | | | | |  | | |
| **JGI p_id** | | **JGI genename** | | | | **annotation** | | |  | | |
| 100004 | | estExt_fgeneshDP_pg.C_5880003 | | | | | | |  | | |
| 100068 | | estExt_fgeneshDP_pg.C_6330001 | | | | | | |  | | |
| 100104 | | estExt_fgeneshDP_pg.C_6670001 | | | | | | |  | | |
| 100122 | | estExt_fgeneshDP_pg.C_6860001 | | | | | | |  | | |
| 100137 | | estExt_fgeneshDP_pg.C_7070001 | | | | | | |  | | |
| 10036 | | gw1.76.17.1 | | | | | | |  | | |
| 10325 | | gw1.299.10.1 | | | | | | |  | | |
| 10349 | | gw1.381.2.1 | | | | | | |  | | |
| 10404 | | gw1.286.17.1 | | | | | | |  | | |
| 10471 | | gw1.248.10.1 | | | | | | |  | | |
| 10592 | | gw1.1.63.1 | | | |  | | |  | | |
| 10642 | | gw1.42.15.1 | | | | | | |  | | |
| 10731 | | gw1.18.17.1 | | | | | | |  | | |
| 10755 | | gw1.286.20.1 | | | | | | |  | | |
| 10759 | | gw1.68.18.1 | | | | | | |  | | |
| 10866 | | gw1.530.5.1 | | | | | | |  | | |
| 10887 | | gw1.270.17.1 | | | | | | |  | | |
| 10903 | | gw1.182.4.1 | | | | | | |  | | |
| 11068 | | gw1.529.1.1 | | | | | | |  | | |
| 11134 | | gw1.119.18.1 | | | | | | |  | | |
| 11157 | | gw1.276.24.1 | | | | | | |  | | |
| 11392 | | gw1.73.26.1 | | | | | | |  | | |
| 11414 | | gw1.145.8.1 | | | | | | |  | | |
| 11435 | | gw1.365.11.1 | | | | | | |  | | |
| 11881 | | gw1.301.22.1 | | | | | | |  | | |
| 11972 | | gw1.4.42.1 | | | |  | | |  | | |
| 12008 | | gw1.213.10.1 | | | | | | |  | | |
| 12010 | | gw1.706.1.1 | | | | | | |  | | |
| 12102 | | gw1.171.8.1 | | | | | | |  | | |
| 12128 | | gw1.353.23.1 | | | | | | |  | | |
| 12191 | | gw1.1.80.1 | | | |  | | |  | | |
| 12263 | | gw1.278.27.1 | | | | | | |  | | |
| 12292 | | gw1.24.13.1 | | | | | | |  | | |
| 12317 | | gw1.694.6.1 | | | | | | |  | | |
| 12366 | | gw1.242.7.1 | | | | | | |  | | |
| 12367 | | gw1.677.5.1 | | | | | | |  | | |
| 12401 | | gw1.38.26.1 | | | | | | |  | | |
| 12612 | | gw1.481.10.1 | | | | | | |  | | |
| 12785 | | gw1.194.11.1 | | | | | | |  | | |
| 12802 | | gw1.226.13.1 | | | | | | |  | | |
| 12804 | | gw1.63.15.1 | | | | | | |  | | |
| 12844 | | gw1.322.9.1 | | | | | | |  | | |
| 13118 | | gw1.166.17.1 | | | | | | |  | | |
| 13138 | | gw1.327.14.1 | | | | | | |  | | |
| 13167 | | gw1.23.10.1 | | | | | | |  | | |
| 13262 | | gw1.376.12.1 | | | | | | |  | | |
| 13266 | | gw1.380.21.1 | | | | | | |  | | |
| 13300 | | gw1.312.16.1 | | | | | | |  | | |
| 13314 | | gw1.32.40.1 | | | | | | |  | | |
| 13347 | | gw1.591.9.1 | | | | | | |  | | |
| 13362 | | gw1.277.19.1 | | | | | | |  | | |
| 13388 | | gw1.204.1.1 | | | | | | |  | | |
| 13409 | | gw1.56.9.1 | | | |  | | |  | | |
| 13442 | | gw1.282.42.1 | | | | | | |  | | |
| 13462 | | gw1.20.23.1 | | | | | | |  | | |
| 13500 | | gw1.309.1.1 | | | | | | |  | | |
| 13521 | | gw1.282.43.1 | | | | | | |  | | |
| 13528 | | gw1.655.6.1 | | | | | | |  | | |
| 13537 | | gw1.434.7.1 | | | | | | |  | | |
| 13646 | | gw1.269.23.1 | | | | | | |  | | |
| 13655 | | gw1.127.7.1 | | | | | | |  | | |
| 13692 | | gw1.421.17.1 | | | | | | |  | | |
| 13707 | | gw1.657.2.1 | | | | | | |  | | |
| 13763 | | gw1.182.16.1 | | | | | | |  | | |
| 13792 | | gw1.294.26.1 | | | | | | |  | | |
| 13832 | | gw1.586.10.1 | | | | | | |  | | |
| 13896 | | gw1.39.46.1 | | | | | | |  | | |
| 13914 | | gw1.229.12.1 | | | | | | |  | | |
| 14104 | | gw1.469.14.1 | | | | | | |  | | |
| 14225 | | gw1.792.3.1 | | | | | | |  | | |
| 14334 | | gw1.9.14.1 | | | |  | | |  | | |
| 14379 | | gw1.245.9.1 | | | | | | |  | | |
| 14576 | | gw1.37.23.1 | | | | | | |  | | |
| 14599 | | gw1.9.20.1 | | | |  | | |  | | |
| 146461 | | GID1.0037150 | | | | | | |  | | |
| 146481 | | GID1.0037170 | | | | | | |  | | |
| 146482 | | GID1.0037171 | | | | | | |  | | |
| 146484 | | GID1.0037173 | | | | | | |  | | |
| 146490 | | GID1.0037179 | | | | | | |  | | |
| 146497 | | GID1.0037186 | | | | | | |  | | |
| 146500 | | GID1.0037189 | | | | | | |  | | |
| 146518 | | GID1.0037207 | | | | | | |  | | |
| 146524 | | GID1.0037213 | | | | | | |  | | |
| 146526 | | GID1.0037215 | | | | | | |  | | |
| 146538 | | GID1.0037227 | | | | | | |  | | |
| 146540 | | GID1.0037229 | | | | | | |  | | |
| 146542 | | GID1.0037231 | | | | | | |  | | |
| 146543 | | GID1.0037232 | | | | | | |  | | |
| 146553 | | GID1.0037242 | | | | | | |  | | |
| 146554 | | GID1.0037243 | | | | | | |  | | |
| 146557 | | GID1.0037246 | | | | | | |  | | |
| 146559 | | GID1.0037248 | | | | | | |  | | |
| 146560 | | GID1.0037249 | | | | | | |  | | |
| 146567 | | GID1.0037256 | | | | | | |  | | |
| 146570 | | GID1.0037259 | | | | | | |  | | |
| 146582 | | GID1.0037271 | | | | | | |  | | |
| 146587 | | GID1.0037276 | | | | | | |  | | |
| 146592 | | GID1.0037281 | | | | | | |  | | |
| 146599 | | GID1.0037288 | | | | | | |  | | |
| 146605 | | GID1.0037294 | | | | | | |  | | |
| 146606 | | GID1.0037295 | | | | | | |  | | |
| 146609 | | GID1.0037298 | | | | | | |  | | |
| 146611 | | GID1.0037300 | | | | | | |  | | |
| 146624 | | GID1.0037313 | | | | | | |  | | |
| 146663 | | GID1.0037352 | | | | | | |  | | |
| 146671 | | GID1.0037360 | | | | | | |  | | |
| 146675 | | GID1.0037364 | | | | | | |  | | |
| 146676 | | GID1.0037365 | | | | | | |  | | |
| 146684 | | GID1.0037373 | | | | | | |  | | |
| 146686 | | GID1.0037375 | | | | | | |  | | |
| 146688 | | GID1.0037377 | | | | | | |  | | |
| 146689 | | GID1.0037378 | | | | | | |  | | |
| 146705 | | GID1.0037394 | | | | | | |  | | |
| 146731 | | GID1.0037420 | | | | | | |  | | |
| 146743 | | GID1.0037432 | | | | | | |  | | |
| 146753 | | GID1.0037442 | | | | | | |  | | |
| 146808 | | GID1.0037497 | | | | | | |  | | |
| 146827 | | GID1.0037516 | | | | | | |  | | |
| 146850 | | GID1.0037539 | | | | | | |  | | |
| 146856 | | GID1.0037545 | | | | | | |  | | |
| 146859 | | GID1.0037548 | | | | | | |  | | |
| 146891 | | GID1.0037580 | | | | | | |  | | |
| 146893 | | GID1.0037582 | | | | | | |  | | |
| 146905 | | GID1.0037594 | | | | | | |  | | |
| 146909 | | GID1.0037598 | | | | | | |  | | |
| 146910 | | GID1.0037599 | | | | | | |  | | |
| 146955 | | GID1.0037644 | | | | | | |  | | |
| 147156 | | GID1.0037845 | | | | | | |  | | |
| 147179 | | GID1.0037868 | | | | | | |  | | |
| 147195 | | GID1.0037884 | | | | | | |  | | |
| 147203 | | GID1.0037892 | | | | | | |  | | |
| 147217 | | GID1.0037906 | | | | | | |  | | |
| 147228 | | GID1.0037917 | | | | | | |  | | |
| 147236 | | GID1.0037925 | | | | | | |  | | |
| 147240 | | GID1.0037929 | | | | | | |  | | |
| 147245 | | GID1.0037934 | | | | | | |  | | |
| 147248 | | GID1.0037937 | | | | | | |  | | |
| 147264 | | GID1.0037953 | | | | | | |  | | |
| 147269 | | GID1.0037958 | | | | | | |  | | |
| 147272 | | GID1.0037961 | | | | | | |  | | |
| 147279 | | GID1.0037968 | | | | | | |  | | |
| 147305 | | GID1.0037994 | | | | | | |  | | |
| 147307 | | GID1.0037996 | | | | | | |  | | |
| 147331 | | GID1.0038020 | | | | | | |  | | |
| 147361 | | GID1.0038050 | | | | | | |  | | |
| 147366 | | GID1.0038055 | | | | | | |  | | |
| 147372 | | GID1.0038061 | | | | | | |  | | |
| 147384 | | GID1.0038073 | | | | | | |  | | |
| 147386 | | GID1.0038075 | | | | | | |  | | |
| 147394 | | GID1.0038083 | | | | | | |  | | |
| 147424 | | GID1.0038113 | | | | | | |  | | |
| 147455 | | GID1.0038144 | | | | | | |  | | |
| 147471 | | GID1.0038160 | | | | | | |  | | |
| 14748 | | gw1.245.14.1 | | | | | | |  | | |
| 147515 | | GID1.0038204 | | | | | | |  | | |
| 147516 | | GID1.0038205 | | | | | | |  | | |
| 147523 | | GID1.0038212 | | | | | | |  | | |
| 147534 | | GID1.0038223 | | | | | | |  | | |
| 147536 | | GID1.0038225 | | | | | | |  | | |
| 147574 | | GID1.0038263 | | | | | | |  | | |
| 147598 | | GID1.0038287 | | | | | | |  | | |
| 147601 | | GID1.0038290 | | | | | | |  | | |
| 147609 | | GID1.0038298 | | | | | | |  | | |
| 147633 | | GID1.0038322 | | | | | | |  | | |
| 147650 | | GID1.0038339 | | | | | | |  | | |
| 147652 | | GID1.0038341 | | | | | | |  | | |
| 147667 | | GID1.0038356 | | | | | | |  | | |
| 147705 | | GID1.0038394 | | | | | | |  | | |
| 147720 | | GID1.0038409 | | | | | | |  | | |
| 147727 | | GID1.0038416 | | | | | | |  | | |
| 147744 | | GID1.0038433 | | | | | | |  | | |
| 147748 | | GID1.0038437 | | | | | | |  | | |
| 147756 | | GID1.0038445 | | | | | | |  | | |
| 147784 | | GID1.0038473 | | | | | | |  | | |
| 147800 | | GID1.0038489 | | | | | | |  | | |
| 147806 | | GID1.0038495 | | | | | | |  | | |
| 147861 | | GID1.0038550 | | | | | | |  | | |
| 147882 | | GID1.0038571 | | | | | | |  | | |
| 147900 | | GID1.0038589 | | | | | | |  | | |
| 147904 | | GID1.0038593 | | | | | | |  | | |
| 147953 | | GID1.0038642 | | | | | | |  | | |
| 147963 | | GID1.0038652 | | | | | | |  | | |
| 148018 | | GID1.0038707 | | | | | | |  | | |
| 148081 | | GID1.0038770 | | | | | | |  | | |
| 148118 | | GID1.0038807 | | | | | | |  | | |
| 148120 | | GID1.0038809 | | | | | | |  | | |
| 148130 | | GID1.0038819 | | | | | | |  | | |
| 148141 | | GID1.0038830 | | | | | | |  | | |
| 148150 | | GID1.0038839 | | | | | | |  | | |
| 148170 | | GID1.0038859 | | | | | | |  | | |
| 148173 | | GID1.0038862 | | | | | | |  | | |
| 148175 | | GID1.0038864 | | | | | | |  | | |
| 148176 | | GID1.0038865 | | | | | | |  | | |
| 148183 | | GID1.0038872 | | | | | | |  | | |
| 148184 | | GID1.0038873 | | | | | | |  | | |
| 148191 | | GID1.0038880 | | | | | | |  | | |
| 148215 | | GID1.0038904 | | | | | | |  | | |
| 148293 | | GID1.0038982 | | | | | | |  | | |
| 148330 | | GID1.0039019 | | | | | | |  | | |
| 148350 | | GID1.0039039 | | | | | | |  | | |
| 148358 | | GID1.0039047 | | | | | | |  | | |
| 148381 | | GID1.0039070 | | | | | | |  | | |
| 148385 | | GID1.0039074 | | | | | | |  | | |
| 148424 | | GID1.0039113 | | | | | | |  | | |
| 148435 | | GID1.0039124 | | | | | | |  | | |
| 148542 | | GID1.0039231 | | | | | | |  | | |
| 148543 | | GID1.0039232 | | | | | | |  | | |
| 148547 | | GID1.0039236 | | | | | | |  | | |
| 148565 | | GID1.0039254 | | | | | | |  | | |
| 148566 | | GID1.0039255 | | | | | | |  | | |
| 148621 | | GID1.0039310 | | | | | | |  | | |
| 148628 | | GID1.0039317 | | | | | | |  | | |
| 148629 | | GID1.0039318 | | | | | | |  | | |
| 148637 | | GID1.0039326 | | | | | | |  | | |
| 148651 | | GID1.0039340 | | | | | | |  | | |
| 148702 | | GID1.0039391 | | | | | | |  | | |
| 148714 | | GID1.0039403 | | | | | | |  | | |
| 148728 | | GID1.0039417 | | | | | | |  | | |
| 148758 | | GID1.0039447 | | | | | | |  | | |
| 148780 | | GID1.0039469 | | | | | | |  | | |
| 148793 | | GID1.0039482 | | | | | | |  | | |
| 148800 | | GID1.0039489 | | | | | | |  | | |
| 148802 | | GID1.0039491 | | | | | | |  | | |
| 148808 | | GID1.0039497 | | | | | | |  | | |
| 148809 | | GID1.0039498 | | | | | | |  | | |
| 148824 | | GID1.0039513 | | | | | | |  | | |
| 148837 | | GID1.0039526 | | | | | | |  | | |
| 148864 | | GID1.0039553 | | | | | | |  | | |
| 148871 | | GID1.0039560 | | | | | | |  | | |
| 148872 | | GID1.0039561 | | | | | | |  | | |
| 148873 | | GID1.0039562 | | | | | | |  | | |
| 148893 | | GID1.0039582 | | | | | | |  | | |
| 148914 | | GID1.0039603 | | | | | | |  | | |
| 148933 | | GID1.0039622 | | | | | | |  | | |
| 148935 | | GID1.0039624 | | | | | | |  | | |
| 148936 | | GID1.0039625 | | | | | | |  | | |
| 148939 | | GID1.0039628 | | | | | | |  | | |
| 148944 | | GID1.0039633 | | | | | | |  | | |
| 148955 | | GID1.0039644 | | | | | | |  | | |
| 148956 | | GID1.0039645 | | | | | | |  | | |
| 148963 | | GID1.0039652 | | | | | | |  | | |
| 148970 | | GID1.0039659 | | | | | | |  | | |
| 148975 | | GID1.0039664 | | | | | | |  | | |
| 149063 | | GID1.0039752 | | | | | | |  | | |
| 149064 | | GID1.0039753 | | | | | | |  | | |
| 149067 | | GID1.0039756 | | | | | | |  | | |
| 149068 | | GID1.0039757 | | | | | | |  | | |
| 149090 | | GID1.0039779 | | | | | | |  | | |
| 149109 | | GID1.0039798 | | | | | | |  | | |
| 149150 | | GID1.0039839 | | | | | | |  | | |
| 149152 | | GID1.0039841 | | | | | | |  | | |
| 149153 | | GID1.0039842 | | | | | | |  | | |
| 149159 | | GID1.0039848 | | | | | | |  | | |
| 149186 | | GID1.0039875 | | | | | | |  | | |
| 149192 | | GID1.0039881 | | | | | | |  | | |
| 149198 | | GID1.0039887 | | | | | | |  | | |
| 149199 | | GID1.0039888 | | | | | | |  | | |
| 149220 | | GID1.0039909 | | | | | | |  | | |
| 149258 | | GID1.0039947 | | | | | | |  | | |
| 149259 | | GID1.0039948 | | | | | | |  | | |
| 149266 | | GID1.0039955 | | | | | | |  | | |
| 149271 | | GID1.0039960 | | | | | | |  | | |
| 149277 | | GID1.0039966 | | | | | | |  | | |
| 149279 | | GID1.0039968 | | | | | | |  | | |
| 149290 | | GID1.0039979 | | | | | | |  | | |
| 149292 | | GID1.0039981 | | | | | | |  | | |
| 149339 | | GID1.0040028 | | | | | | |  | | |
| 149346 | | GID1.0040035 | | | | | | |  | | |
| 149351 | | GID1.0040040 | | | | | | |  | | |
| 149363 | | GID1.0040052 | | | | | | |  | | |
| 149365 | | GID1.0040054 | | | | | | |  | | |
| 149418 | | GID1.0040107 | | | | | | |  | | |
| 149433 | | GID1.0040122 | | | | | | |  | | |
| 149439 | | GID1.0040128 | | | | | | |  | | |
| 149441 | | GID1.0040130 | | | | | | |  | | |
| 149442 | | GID1.0040131 | | | | | | |  | | |
| 149461 | | GID1.0040150 | | | | | | |  | | |
| 149475 | | GID1.0040164 | | | | | | |  | | |
| 149476 | | GID1.0040165 | | | | | | |  | | |
| 149485 | | GID1.0040174 | | | | | | |  | | |
| 149491 | | GID1.0040180 | | | | | | |  | | |
| 149493 | | GID1.0040182 | | | | | | |  | | |
| 149516 | | GID1.0040205 | | | | | | |  | | |
| 149521 | | GID1.0040210 | | | | | | |  | | |
| 149551 | | GID1.0040240 | | | | | | |  | | |
| 149557 | | GID1.0040246 | | | | | | |  | | |
| 149558 | | GID1.0040247 | | | | | | |  | | |
| 149579 | | GID1.0040268 | | | | | | |  | | |
| 149585 | | GID1.0040274 | | | | | | |  | | |
| 149596 | | GID1.0040285 | | | | | | |  | | |
| 149610 | | GID1.0040299 | | | | | | |  | | |
| 149619 | | GID1.0040308 | | | | | | |  | | |
| 149628 | | GID1.0040317 | | | | | | |  | | |
| 149741 | | GID1.0040430 | | | | | | |  | | |
| 149744 | | GID1.0040433 | | | | | | |  | | |
| 149753 | | GID1.0040442 | | | | | | |  | | |
| 149760 | | GID1.0040449 | | | | | | |  | | |
| 149766 | | GID1.0040455 | | | | | | |  | | |
| 149807 | | GID1.0040496 | | | | | | |  | | |
| 149817 | | GID1.0040506 | | | | | | |  | | |
| 149859 | | GID1.0040548 | | | | | | |  | | |
| 149884 | | GID1.0040573 | | | | | | |  | | |
| 149902 | | GID1.0040591 | | | | | | |  | | |
| 149912 | | GID1.0040601 | | | | | | |  | | |
| 149913 | | GID1.0040602 | | | | | | |  | | |
| 149914 | | GID1.0040603 | | | | | | |  | | |
| 149932 | | GID1.0040621 | | | | | | |  | | |
| 149955 | | GID1.0040644 | | | | | | |  | | |
| 149956 | | GID1.0040645 | | | | | | |  | | |
| 149957 | | GID1.0040646 | | | | | | |  | | |
| 149964 | | GID1.0040653 | | | | | | |  | | |
| 149976 | | GID1.0040665 | | | | | | |  | | |
| 149984 | | GID1.0040673 | | | | | | |  | | |
| 149999 | | GID1.0040688 | | | | | | |  | | |
| 150000 | | GID1.0040689 | | | | | | |  | | |
| 150022 | | GID1.0040711 | | | | | | |  | | |
| 150063 | | GID1.0040752 | | | | | | |  | | |
| 150085 | | GID1.0040774 | | | | | | |  | | |
| 150105 | | GID1.0040794 | | | | | | |  | | |
| 150114 | | GID1.0040803 | | | | | | |  | | |
| 150125 | | GID1.0040814 | | | | | | |  | | |
| 150126 | | GID1.0040815 | | | | | | |  | | |
| 150160 | | GID1.0040849 | | | | | | |  | | |
| 150192 | | GID1.0040881 | | | | | | |  | | |
| 150209 | | GID1.0040898 | | | | | | |  | | |
| 150222 | | GID1.0040911 | | | | | | |  | | |
| 150229 | | GID1.0040918 | | | | | | |  | | |
| 150253 | | GID1.0040942 | | | | | | |  | | |
| 150257 | | GID1.0040946 | | | | | | |  | | |
| 150285 | | GID1.0040974 | | | | | | |  | | |
| 150298 | | GID1.0040987 | | | | | | |  | | |
| 150319 | | GID1.0041008 | | | | | | |  | | |
| 150320 | | GID1.0041009 | | | | | | |  | | |
| 150325 | | GID1.0041014 | | | | | | |  | | |
| 150413 | | GID1.0041102 | | | | | | |  | | |
| 150419 | | GID1.0041108 | | | | | | |  | | |
| 150425 | | GID1.0041114 | | | | | | |  | | |
| 150464 | | GID1.0041153 | | | | | | |  | | |
| 150468 | | GID1.0041157 | | | | | | |  | | |
| 150542 | | GID1.0041231 | | | | | | |  | | |
| 150550 | | GID1.0041239 | | | | | | |  | | |
| 150554 | | GID1.0041243 | | | | | | |  | | |
| 150557 | | GID1.0041246 | | | | | | |  | | |
| 150561 | | GID1.0041250 | | | | | | |  | | |
| 150571 | | GID1.0041260 | | | | | | |  | | |
| 150584 | | GID1.0041273 | | | | | | |  | | |
| 150598 | | GID1.0041287 | | | | | | |  | | |
| 150699 | | GID1.0041388 | | | | | | |  | | |
| 150759 | | GID1.0041448 | | | | | | |  | | |
| 150762 | | GID1.0041451 | | | | | | |  | | |
| 150791 | | GID1.0041480 | | | | | | |  | | |
| 150801 | | GID1.0041490 | | | | | | |  | | |
| 150823 | | GID1.0041512 | | | | | | |  | | |
| 150824 | | GID1.0041513 | | | | | | |  | | |
| 150826 | | GID1.0041515 | | | | | | |  | | |
| 150840 | | GID1.0041529 | | | | | | |  | | |
| 150858 | | GID1.0041547 | | | | | | |  | | |
| 150878 | | GID1.0041567 | | | | | | |  | | |
| 150905 | | GID1.0041594 | | | | | | |  | | |
| 150926 | | GID1.0041615 | | | | | | |  | | |
| 150953 | | GID1.0041642 | | | | | | |  | | |
| 150971 | | GID1.0041660 | | | | | | |  | | |
| 150977 | | GID1.0041666 | | | | | | |  | | |
| 150987 | | GID1.0041676 | | | | | | |  | | |
| 151025 | | GID1.0041714 | | | | | | |  | | |
| 151026 | | GID1.0041715 | | | | | | |  | | |
| 151045 | | GID1.0041734 | | | | | | |  | | |
| 151051 | | GID1.0041740 | | | | | | |  | | |
| 151056 | | GID1.0041745 | | | | | | |  | | |
| 151059 | | GID1.0041748 | | | | | | |  | | |
| 151061 | | GID1.0041750 | | | | | | |  | | |
| 151109 | | GID1.0041798 | | | | | | |  | | |
| 151131 | | GID1.0041820 | | | | | | |  | | |
| 151134 | | GID1.0041823 | | | | | | |  | | |
| 151139 | | GID1.0041828 | | | | | | |  | | |
| 151141 | | GID1.0041830 | | | | | | |  | | |
| 151161 | | GID1.0041850 | | | | | | |  | | |
| 151180 | | GID1.0041869 | | | | | | |  | | |
| 151193 | | GID1.0041882 | | | | | | |  | | |
| 151208 | | GID1.0041897 | | | | | | |  | | |
| 151221 | | GID1.0041910 | | | | | | |  | | |
| 151268 | | GID1.0041957 | | | | | | |  | | |
| 151292 | | GID1.0041981 | | | | | | |  | | |
| 151299 | | GID1.0041988 | | | | | | |  | | |
| 151306 | | GID1.0041995 | | | | | | |  | | |
| 151317 | | GID1.0042006 | | | | | | |  | | |
| 151319 | | GID1.0042008 | | | | | | |  | | |
| 151325 | | GID1.0042014 | | | | | | |  | | |
| 151354 | | GID1.0042043 | | | | | | |  | | |
| 151401 | | GID1.0042090 | | | | | | |  | | |
| 151405 | | GID1.0042094 | | | | | | |  | | |
| 151407 | | GID1.0042096 | | | | | | |  | | |
| 151408 | | GID1.0042097 | | | | | | |  | | |
| 151410 | | GID1.0042099 | | | | | | |  | | |
| 151460 | | GID1.0042149 | | | | | | |  | | |
| 151479 | | GID1.0042168 | | | | | | |  | | |
| 151490 | | GID1.0042179 | | | | | | |  | | |
| 151518 | | GID1.0042207 | | | | | | |  | | |
| 151519 | | GID1.0042208 | | | | | | |  | | |
| 15157 | | gw1.2.46.1 | | | |  | | |  | | |
| 151668 | | GID1.0042357 | | | | | | |  | | |
| 151672 | | GID1.0042361 | | | | | | |  | | |
| 151675 | | GID1.0042364 | | | | | | |  | | |
| 151682 | | GID1.0042371 | | | | | | |  | | |
| 151710 | | GID1.0042399 | | | | | | |  | | |
| 151726 | | GID1.0042415 | | | | | | |  | | |
| 151768 | | GID1.0042457 | | | | | | |  | | |
| 151784 | | GID1.0042473 | | | | | | |  | | |
| 151821 | | GID1.0042510 | | | | | | |  | | |
| 151841 | | GID1.0042530 | | | | | | |  | | |
| 151870 | | GID1.0042559 | | | | | | |  | | |
| 151905 | | GID1.0042594 | | | | | | |  | | |
| 151923 | | GID1.0042612 | | | | | | |  | | |
| 151927 | | GID1.0042616 | | | | | | |  | | |
| 151929 | | GID1.0042618 | | | | | | |  | | |
| 151957 | | GID1.0042646 | | | | | | |  | | |
| 151960 | | GID1.0042649 | | | | | | |  | | |
| 151989 | | GID1.0042678 | | | | | | |  | | |
| 151990 | | GID1.0042679 | | | | | | |  | | |
| 151991 | | GID1.0042680 | | | | | | |  | | |
| 152011 | | GID1.0042700 | | | | | | |  | | |
| 152102 | | GID1.0042791 | | | | | | |  | | |
| 152190 | | GID1.0042879 | | | | | | |  | | |
| 152192 | | GID1.0042881 | | | | | | |  | | |
| 152238 | | GID1.0042927 | | | | | | |  | | |
| 152280 | | GID1.0042969 | | | | | | |  | | |
| 152284 | | GID1.0042973 | | | | | | |  | | |
| 152286 | | GID1.0042975 | | | | | | |  | | |
| 152291 | | GID1.0042980 | | | | | | |  | | |
| 152386 | | GID1.0043075 | | | | | | |  | | |
| 152402 | | GID1.0043091 | | | | | | |  | | |
| 152431 | | GID1.0043120 | | | | | | |  | | |
| 152464 | | GID1.0043153 | | | | | | |  | | |
| 152469 | | GID1.0043158 | | | | | | |  | | |
| 152477 | | GID1.0043166 | | | | | | |  | | |
| 152492 | | GID1.0043181 | | | | | | |  | | |
| 152496 | | GID1.0043185 | | | | | | |  | | |
| 152537 | | GID1.0043226 | | | | | | |  | | |
| 152568 | | GID1.0043257 | | | | | | |  | | |
| 15257 | | gw1.187.17.1 | | | | | | |  | | |
| 152571 | | GID1.0043260 | | | | | | |  | | |
| 152575 | | GID1.0043264 | | | | | | |  | | |
| 152593 | | GID1.0043282 | | | | | | |  | | |
| 152664 | | GID1.0043353 | | | | | | |  | | |
| 152678 | | GID1.0043367 | | | | | | |  | | |
| 152686 | | GID1.0043375 | | | | | | |  | | |
| 152708 | | GID1.0043397 | | | | | | |  | | |
| 152709 | | GID1.0043398 | | | | | | |  | | |
| 152710 | | GID1.0043399 | | | | | | |  | | |
| 152743 | | GID1.0043432 | | | | | | |  | | |
| 152759 | | GID1.0043448 | | | | | | |  | | |
| 152779 | | GID1.0043468 | | | | | | |  | | |
| 152785 | | GID1.0043474 | | | | | | |  | | |
| 152796 | | GID1.0043485 | | | | | | |  | | |
| 152850 | | GID1.0043539 | | | | | | |  | | |
| 152880 | | GID1.0043569 | | | | | | |  | | |
| 152882 | | GID1.0043571 | | | | | | |  | | |
| 152887 | | GID1.0043576 | | | | | | |  | | |
| 152895 | | GID1.0043584 | | | | | | |  | | |
| 152903 | | GID1.0043592 | | | | | | |  | | |
| 152935 | | GID1.0043624 | | | | | | |  | | |
| 152967 | | GID1.0043656 | | | | | | |  | | |
| 152984 | | GID1.0043673 | | | | | | |  | | |
| 153011 | | GID1.0043700 | | | | | | |  | | |
| 153065 | | GID1.0043754 | | | | | | |  | | |
| 153079 | | GID1.0043768 | | | | | | |  | | |
| 153149 | | GID1.0043838 | | | | | | |  | | |
| 153204 | | GID1.0043893 | | | | | | |  | | |
| 153222 | | GID1.0043911 | | | | | | |  | | |
| 153321 | | GID1.0044010 | | | | | | |  | | |
| 153345 | | GID1.0044034 | | | | | | |  | | |
| 153346 | | GID1.0044035 | | | | | | |  | | |
| 153347 | | GID1.0044036 | | | | | | |  | | |
| 153365 | | GID1.0044054 | | | | | | |  | | |
| 153368 | | GID1.0044057 | | | | | | |  | | |
| 153371 | | GID1.0044060 | | | | | | |  | | |
| 153390 | | GID1.0044079 | | | | | | |  | | |
| 153444 | | GID1.0044133 | | | | | | |  | | |
| 153464 | | GID1.0044153 | | | | | | |  | | |
| 153476 | | GID1.0044165 | | | | | | |  | | |
| 153496 | | GID1.0044185 | | | | | | |  | | |
| 153503 | | GID1.0044192 | | | | | | |  | | |
| 153511 | | GID1.0044200 | | | | | | |  | | |
| 153570 | | GID1.0044259 | | | | | | |  | | |
| 153576 | | GID1.0044265 | | | | | | |  | | |
| 153621 | | GID1.0044310 | | | | | | |  | | |
| 153653 | | GID1.0044342 | | | | | | |  | | |
| 153665 | | GID1.0044354 | | | | | | |  | | |
| 153667 | | GID1.0044356 | | | | | | |  | | |
| 153685 | | GID1.0044374 | | | | | | |  | | |
| 153691 | | GID1.0044380 | | | | | | |  | | |
| 153713 | | GID1.0044402 | | | | | | |  | | |
| 153721 | | GID1.0044410 | | | | | | |  | | |
| 153725 | | GID1.0044414 | | | | | | |  | | |
| 153746 | | GID1.0044435 | | | | | | |  | | |
| 153747 | | GID1.0044436 | | | | | | |  | | |
| 153756 | | GID1.0044445 | | | | | | |  | | |
| 153785 | | GID1.0044474 | | | | | | |  | | |
| 153804 | | GID1.0044493 | | | | | | |  | | |
| 153848 | | GID1.0044537 | | | | | | |  | | |
| 153870 | | GID1.0044559 | | | | | | |  | | |
| 153872 | | GID1.0044561 | | | | | | |  | | |
| 153876 | | GID1.0044565 | | | | | | |  | | |
| 153878 | | GID1.0044567 | | | | | | |  | | |
| 153881 | | GID1.0044570 | | | | | | |  | | |
| 153906 | | GID1.0044595 | | | | | | |  | | |
| 153923 | | GID1.0044612 | | | | | | |  | | |
| 153931 | | GID1.0044620 | | | | | | |  | | |
| 153949 | | GID1.0044638 | | | | | | |  | | |
| 153957 | | GID1.0044646 | | | | | | |  | | |
| 153973 | | GID1.0044662 | | | | | | |  | | |
| 153979 | | GID1.0044668 | | | | | | |  | | |
| 153981 | | GID1.0044670 | | | | | | |  | | |
| 153988 | | GID1.0044677 | | | | | | |  | | |
| 154024 | | GID1.0044713 | | | | | | |  | | |
| 154059 | | GID1.0044748 | | | | | | |  | | |
| 154060 | | GID1.0044749 | | | | | | |  | | |
| 154062 | | GID1.0044751 | | | | | | |  | | |
| 154063 | | GID1.0044752 | | | | | | |  | | |
| 154065 | | GID1.0044754 | | | | | | |  | | |
| 154093 | | GID1.0044782 | | | | | | |  | | |
| 154106 | | GID1.0044795 | | | | | | |  | | |
| 154121 | | GID1.0044810 | | | | | | |  | | |
| 154132 | | GID1.0044821 | | | | | | |  | | |
| 154172 | | GID1.0044826 | | | | | | |  | | |
| 154173 | | GID1.0044827 | | | | | | |  | | |
| 154174 | | GID1.0044828 | | | | | | |  | | |
| 154205 | | GID1.0044859 | | | | | | |  | | |
| 154248 | | GID1.0044902 | | | | | | |  | | |
| 154255 | | GID1.0044909 | | | | | | |  | | |
| 154262 | | GID1.0044916 | | | | | | |  | | |
| 154295 | | GID1.0044949 | | | | | | |  | | |
| 154329 | | GID1.0044983 | | | | | | |  | | |
| 154330 | | GID1.0044984 | | | | | | |  | | |
| 154350 | | GID1.0045004 | | | | | | |  | | |
| 154354 | | GID1.0045008 | | | | | | |  | | |
| 154376 | | GID1.0045030 | | | | | | |  | | |
| 154381 | | GID1.0045035 | | | | | | |  | | |
| 154393 | | GID1.0045047 | | | | | | |  | | |
| 154414 | | GID1.0045068 | | | | | | |  | | |
| 154425 | | GID1.0045079 | | | | | | |  | | |
| 154430 | | GID1.0045084 | | | | | | |  | | |
| 154432 | | GID1.0045086 | | | | | | |  | | |
| 154448 | | GID1.0045102 | | | | | | |  | | |
| 154475 | | GID1.0045129 | | | | | | |  | | |
| 154480 | | GID1.0045134 | | | | | | |  | | |
| 154528 | | GID1.0045182 | | | | | | |  | | |
| 154530 | | GID1.0045184 | | | | | | |  | | |
| 154552 | | GID1.0045206 | | | | | | |  | | |
| 154553 | | GID1.0045207 | | | | | | |  | | |
| 154557 | | GID1.0045211 | | | | | | |  | | |
| 154580 | | GID1.0045234 | | | | | | |  | | |
| 154589 | | GID1.0045243 | | | | | | |  | | |
| 154592 | | GID1.0045246 | | | | | | |  | | |
| 154597 | | GID1.0045251 | | | | | | |  | | |
| 154629 | | GID1.0045283 | | | | | | |  | | |
| 154630 | | GID1.0045284 | | | | | | |  | | |
| 154641 | | GID1.0045295 | | | | | | |  | | |
| 154651 | | GID1.0045305 | | | | | | |  | | |
| 154665 | | GID1.0045319 | | | | | | |  | | |
| 154683 | | GID1.0045337 | | | | | | |  | | |
| 154693 | | GID1.0045347 | | | | | | |  | | |
| 154723 | | GID1.0045377 | | | | | | |  | | |
| 154724 | | GID1.0045378 | | | | | | |  | | |
| 154745 | | GID1.0045399 | | | | | | |  | | |
| 154758 | | GID1.0045412 | | | | | | |  | | |
| 154760 | | GID1.0045414 | | | | | | |  | | |
| 154762 | | GID1.0045416 | | | | | | |  | | |
| 154763 | | GID1.0045417 | | | | | | |  | | |
| 154787 | | GID1.0045441 | | | | | | |  | | |
| 154833 | | GID1.0045487 | | | | | | |  | | |
| 154839 | | GID1.0045493 | | | | | | |  | | |
| 154850 | | GID1.0045504 | | | | | | |  | | |
| 154897 | | GID1.0045551 | | | | | | |  | | |
| 154906 | | GID1.0045560 | | | | | | |  | | |
| 154919 | | GID1.0045573 | | | | | | |  | | |
| 154925 | | GID1.0045579 | | | | | | |  | | |
| 154944 | | GID1.0045598 | | | | | | |  | | |
| 154950 | | GID1.0045604 | | | | | | |  | | |
| 154953 | | GID1.0045607 | | | | | | |  | | |
| 154982 | | GID1.0045636 | | | | | | |  | | |
| 155001 | | GID1.0045655 | | | | | | |  | | |
| 155013 | | GID1.0045667 | | | | | | |  | | |
| 155021 | | GID1.0045675 | | | | | | |  | | |
| 155049 | | GID1.0045703 | | | | | | |  | | |
| 155063 | | GID1.0045717 | | | | | | |  | | |
| 155071 | | GID1.0045725 | | | | | | |  | | |
| 155142 | | GID1.0045796 | | | | | | |  | | |
| 155169 | | GID1.0045823 | | | | | | |  | | |
| 155190 | | GID1.0045844 | | | | | | |  | | |
| 155216 | | GID1.0045870 | | | | | | |  | | |
| 155227 | | GID1.0045881 | | | | | | |  | | |
| 155236 | | GID1.0045890 | | | | | | |  | | |
| 155244 | | GID1.0045898 | | | | | | |  | | |
| 155245 | | GID1.0045899 | | | | | | |  | | |
| 155265 | | GID1.0045919 | | | | | | |  | | |
| 155266 | | GID1.0045920 | | | | | | |  | | |
| 155267 | | GID1.0045921 | | | | | | |  | | |
| 155273 | | GID1.0045927 | | | | | | |  | | |
| 155274 | | GID1.0045928 | | | | | | |  | | |
| 155300 | | GID1.0045954 | | | | | | |  | | |
| 155344 | | GID1.0045998 | | | | | | |  | | |
| 155347 | | GID1.0046001 | | | | | | |  | | |
| 155357 | | GID1.0046011 | | | | | | |  | | |
| 155373 | | GID1.0046027 | | | | | | |  | | |
| 155380 | | GID1.0046034 | | | | | | |  | | |
| 155388 | | GID1.0046042 | | | | | | |  | | |
| 155407 | | GID1.0046061 | | | | | | |  | | |
| 155414 | | GID1.0046068 | | | | | | |  | | |
| 155417 | | GID1.0046071 | | | | | | |  | | |
| 155418 | | GID1.0046072 | | | | | | |  | | |
| 155433 | | GID1.0046087 | | | | | | |  | | |
| 155444 | | GID1.0046098 | | | | | | |  | | |
| 155452 | | GID1.0046106 | | | | | | |  | | |
| 155455 | | GID1.0046109 | | | | | | |  | | |
| 155478 | | GID1.0046132 | | | | | | |  | | |
| 155482 | | GID1.0046136 | | | | | | |  | | |
| 155550 | | GID1.0046204 | | | | | | |  | | |
| 155584 | | GID1.0046238 | | | | | | |  | | |
| 155597 | | GID1.0046251 | | | | | | |  | | |
| 155616 | | GID1.0046270 | | | | | | |  | | |
| 155629 | | GID1.0046283 | | | | | | |  | | |
| 155692 | | GID1.0046346 | | | | | | |  | | |
| 155715 | | GID1.0046369 | | | | | | |  | | |
| 155716 | | GID1.0046370 | | | | | | |  | | |
| 155754 | | GID1.0046408 | | | | | | |  | | |
| 155762 | | GID1.0046416 | | | | | | |  | | |
| 155763 | | GID1.0046417 | | | | | | |  | | |
| 155773 | | GID1.0046427 | | | | | | |  | | |
| 155776 | | GID1.0046430 | | | | | | |  | | |
| 155845 | | GID1.0046499 | | | | | | |  | | |
| 155854 | | GID1.0046508 | | | | | | |  | | |
| 155856 | | GID1.0046510 | | | | | | |  | | |
| 155857 | | GID1.0046511 | | | | | | |  | | |
| 155871 | | GID1.0046525 | | | | | | |  | | |
| 155872 | | GID1.0046526 | | | | | | |  | | |
| 155879 | | GID1.0046533 | | | | | | |  | | |
| 155880 | | GID1.0046534 | | | | | | |  | | |
| 155882 | | GID1.0046536 | | | | | | |  | | |
| 155904 | | GID1.0046558 | | | | | | |  | | |
| 155921 | | GID1.0046575 | | | | | | |  | | |
| 155955 | | GID1.0046609 | | | | | | |  | | |
| 155989 | | GID1.0046643 | | | | | | |  | | |
| 155990 | | GID1.0046644 | | | | | | |  | | |
| 156021 | | GID1.0046675 | | | | | | |  | | |
| 156068 | | GID1.0046722 | | | | | | |  | | |
| 156089 | | GID1.0046743 | | | | | | |  | | |
| 156111 | | GID1.0046765 | | | | | | |  | | |
| 156150 | | GID1.0046804 | | | | | | |  | | |
| 156167 | | GID1.0046821 | | | | | | |  | | |
| 156168 | | GID1.0046822 | | | | | | |  | | |
| 156213 | | GID1.0046867 | | | | | | |  | | |
| 156215 | | GID1.0046869 | | | | | | |  | | |
| 156240 | | GID1.0046894 | | | | | | |  | | |
| 156246 | | GID1.0046900 | | | | | | |  | | |
| 156283 | | GID1.0046937 | | | | | | |  | | |
| 156286 | | GID1.0046940 | | | | | | |  | | |
| 156310 | | GID1.0046964 | | | | | | |  | | |
| 156314 | | GID1.0046968 | | | | | | |  | | |
| 156337 | | GID1.0046991 | | | | | | |  | | |
| 156339 | | GID1.0046993 | | | | | | |  | | |
| 156343 | | GID1.0046997 | | | | | | |  | | |
| 156348 | | GID1.0047002 | | | | | | |  | | |
| 156349 | | GID1.0047003 | | | | | | |  | | |
| 156353 | | GID1.0047007 | | | | | | |  | | |
| 156371 | | GID1.0047025 | | | | | | |  | | |
| 156385 | | GID1.0047039 | | | | | | |  | | |
| 156407 | | GID1.0047061 | | | | | | |  | | |
| 156409 | | GID1.0047063 | | | | | | |  | | |
| 156411 | | GID1.0047065 | | | | | | |  | | |
| 156412 | | GID1.0047066 | | | | | | |  | | |
| 156446 | | GID1.0047100 | | | | | | |  | | |
| 156452 | | GID1.0047106 | | | | | | |  | | |
| 156460 | | GID1.0047114 | | | | | | |  | | |
| 156498 | | GID1.0047152 | | | | | | |  | | |
| 156516 | | GID1.0047170 | | | | | | |  | | |
| 156533 | | GID1.0047187 | | | | | | |  | | |
| 156555 | | GID1.0047209 | | | | | | |  | | |
| 156565 | | GID1.0047219 | | | | | | |  | | |
| 156603 | | GID1.0047257 | | | | | | |  | | |
| 156621 | | GID1.0047275 | | | | | | |  | | |
| 156657 | | GID1.0047311 | | | | | | |  | | |
| 156675 | | GID1.0047329 | | | | | | |  | | |
| 156679 | | GID1.0047333 | | | | | | |  | | |
| 156718 | | GID1.0047372 | | | | | | |  | | |
| 156720 | | GID1.0047374 | | | | | | |  | | |
| 156739 | | GID1.0047393 | | | | | | |  | | |
| 156745 | | GID1.0047399 | | | | | | |  | | |
| 156747 | | GID1.0047401 | | | | | | |  | | |
| 156785 | | GID1.0047439 | | | | | | |  | | |
| 156792 | | GID1.0047446 | | | | | | |  | | |
| 156797 | | GID1.0047451 | | | | | | |  | | |
| 156799 | | GID1.0047453 | | | | | | |  | | |
| 156829 | | GID1.0047483 | | | | | | |  | | |
| 156839 | | GID1.0047493 | | | | | | |  | | |
| 156867 | | GID1.0047521 | | | | | | |  | | |
| 156917 | | GID1.0047571 | | | | | | |  | | |
| 156955 | | GID1.0047609 | | | | | | |  | | |
| 156962 | | GID1.0047616 | | | | | | |  | | |
| 156971 | | GID1.0047625 | | | | | | |  | | |
| 157019 | | GID1.0047673 | | | | | | |  | | |
| 157035 | | GID1.0047689 | | | | | | |  | | |
| 157044 | | GID1.0047698 | | | | | | |  | | |
| 157060 | | GID1.0047714 | | | | | | |  | | |
| 157061 | | GID1.0047715 | | | | | | |  | | |
| 157073 | | GID1.0047727 | | | | | | |  | | |
| 157100 | | GID1.0047754 | | | | | | |  | | |
| 157103 | | GID1.0047757 | | | | | | |  | | |
| 157121 | | GID1.0047775 | | | | | | |  | | |
| 157139 | | GID1.0047793 | | | | | | |  | | |
| 157156 | | GID1.0047810 | | | | | | |  | | |
| 157163 | | GID1.0047817 | | | | | | |  | | |
| 157187 | | GID1.0047841 | | | | | | |  | | |
| 157203 | | GID1.0047857 | | | | | | |  | | |
| 157204 | | GID1.0047858 | | | | | | |  | | |
| 157233 | | GID1.0047887 | | | | | | |  | | |
| 157234 | | GID1.0047888 | | | | | | |  | | |
| 157248 | | GID1.0047902 | | | | | | |  | | |
| 157293 | | GID1.0047947 | | | | | | |  | | |
| 157296 | | GID1.0047950 | | | | | | |  | | |
| 157297 | | GID1.0047951 | | | | | | |  | | |
| 157313 | | GID1.0047967 | | | | | | |  | | |
| 157314 | | GID1.0047968 | | | | | | |  | | |
| 157315 | | GID1.0047969 | | | | | | |  | | |
| 157324 | | GID1.0047978 | | | | | | |  | | |
| 157335 | | GID1.0047989 | | | | | | |  | | |
| 157336 | | GID1.0047990 | | | | | | |  | | |
| 157341 | | GID1.0047995 | | | | | | |  | | |
| 157342 | | GID1.0047996 | | | | | | |  | | |
| 157344 | | GID1.0047998 | | | | | | |  | | |
| 157359 | | GID1.0048013 | | | | | | |  | | |
| 157378 | | GID1.0048032 | | | | | | |  | | |
| 157431 | | GID1.0048085 | | | | | | |  | | |
| 157473 | | GID1.0048127 | | | | | | |  | | |
| 157477 | | GID1.0048131 | | | | | | |  | | |
| 157493 | | GID1.0048147 | | | | | | |  | | |
| 157525 | | GID1.0048179 | | | | | | |  | | |
| 157551 | | GID1.0048205 | | | | | | |  | | |
| 157564 | | GID1.0048218 | | | | | | |  | | |
| 157566 | | GID1.0048220 | | | | | | |  | | |
| 157571 | | GID1.0048225 | | | | | | |  | | |
| 157574 | | GID1.0048228 | | | | | | |  | | |
| 157585 | | GID1.0048239 | | | | | | |  | | |
| 157624 | | GID1.0048278 | | | | | | |  | | |
| 157643 | | GID1.0048297 | | | | | | |  | | |
| 157675 | | GID1.0048329 | | | | | | |  | | |
| 157708 | | GID1.0048362 | | | | | | |  | | |
| 157717 | | GID1.0048371 | | | | | | |  | | |
| 157718 | | GID1.0048372 | | | | | | |  | | |
| 157719 | | GID1.0048373 | | | | | | |  | | |
| 157723 | | GID1.0048377 | | | | | | |  | | |
| 157771 | | GID1.0048425 | | | | | | |  | | |
| 157798 | | GID1.0048452 | | | | | | |  | | |
| 157802 | | GID1.0048456 | | | | | | |  | | |
| 157823 | | GID1.0048477 | | | | | | |  | | |
| 157824 | | GID1.0048478 | | | | | | |  | | |
| 157859 | | GID1.0048513 | | | | | | |  | | |
| 157892 | | GID1.0048546 | | | | | | |  | | |
| 157893 | | GID1.0048547 | | | | | | |  | | |
| 157894 | | GID1.0048548 | | | | | | |  | | |
| 15792 | | gw1.151.19.1 | | | | | | |  | | |
| 157930 | | GID1.0048584 | | | | | | |  | | |
| 157950 | | GID1.0048604 | | | | | | |  | | |
| 157956 | | GID1.0048610 | | | | | | |  | | |
| 157979 | | GID1.0048633 | | | | | | |  | | |
| 157981 | | GID1.0048635 | | | | | | |  | | |
| 157999 | | GID1.0048653 | | | | | | |  | | |
| 158003 | | GID1.0048657 | | | | | | |  | | |
| 158075 | | GID1.0048729 | | | | | | |  | | |
| 158080 | | GID1.0048734 | | | | | | |  | | |
| 158081 | | GID1.0048735 | | | | | | |  | | |
| 158084 | | GID1.0048738 | | | | | | |  | | |
| 158092 | | GID1.0048746 | | | | | | |  | | |
| 158094 | | GID1.0048748 | | | | | | |  | | |
| 158100 | | GID1.0048754 | | | | | | |  | | |
| 158112 | | GID1.0048766 | | | | | | |  | | |
| 158153 | | GID1.0048807 | | | | | | |  | | |
| 158168 | | GID1.0048822 | | | | | | |  | | |
| 158174 | | GID1.0048828 | | | | | | |  | | |
| 158177 | | GID1.0048831 | | | | | | |  | | |
| 158187 | | GID1.0048841 | | | | | | |  | | |
| 158192 | | GID1.0048846 | | | | | | |  | | |
| 158200 | | GID1.0048854 | | | | | | |  | | |
| 158208 | | GID1.0048862 | | | | | | |  | | |
| 158209 | | GID1.0048863 | | | | | | |  | | |
| 158253 | | GID1.0048907 | | | | | | |  | | |
| 158259 | | GID1.0048913 | | | | | | |  | | |
| 158279 | | GID1.0048933 | | | | | | |  | | |
| 158280 | | GID1.0048934 | | | | | | |  | | |
| 158323 | | GID1.0048962 | | | | | | |  | | |
| 158329 | | GID1.0048968 | | | | | | |  | | |
| 158344 | | GID1.0048983 | | | | | | |  | | |
| 158368 | | GID1.0049007 | | | | | | |  | | |
| 158371 | | GID1.0049010 | | | | | | |  | | |
| 158374 | | GID1.0049013 | | | | | | |  | | |
| 158377 | | GID1.0049016 | | | | | | |  | | |
| 158399 | | GID1.0049038 | | | | | | |  | | |
| 158400 | | GID1.0049039 | | | | | | |  | | |
| 158432 | | GID1.0049071 | | | | | | |  | | |
| 158479 | | GID1.0049105 | | | | | | |  | | |
| 158488 | | GID1.0049114 | | | | | | |  | | |
| 158490 | | GID1.0049116 | | | | | | |  | | |
| 158539 | | GID1.0049165 | | | | | | |  | | |
| 158552 | | GID1.0049178 | | | | | | |  | | |
| 158589 | | GID1.0049215 | | | | | | |  | | |
| 158609 | | GID1.0049235 | | | | | | |  | | |
| 158615 | | GID1.0049241 | | | | | | |  | | |
| 158657 | | GID1.0049283 | | | | | | |  | | |
| 158676 | | GID1.0049302 | | | | | | |  | | |
| 158686 | | GID1.0049312 | | | | | | |  | | |
| 158710 | | GID1.0049336 | | | | | | |  | | |
| 158786 | | GID1.0049412 | | | | | | |  | | |
| 158807 | | GID1.0049433 | | | | | | |  | | |
| 158834 | | GID1.0049460 | | | | | | |  | | |
| 158837 | | GID1.0049463 | | | | | | |  | | |
| 158842 | | GID1.0049468 | | | | | | |  | | |
| 158853 | | GID1.0049479 | | | | | | |  | | |
| 158855 | | GID1.0049481 | | | | | | |  | | |
| 158870 | | GID1.0049496 | | | | | | |  | | |
| 158927 | | GID1.0049553 | | | | | | |  | | |
| 158938 | | GID1.0049564 | | | | | | |  | | |
| 158972 | | GID1.0049598 | | | | | | |  | | |
| 158973 | | GID1.0049599 | | | | | | |  | | |
| 158988 | | GID1.0049614 | | | | | | |  | | |
| 158998 | | GID1.0049624 | | | | | | |  | | |
| 159011 | | GID1.0049637 | | | | | | |  | | |
| 159012 | | GID1.0049638 | | | | | | |  | | |
| 159043 | | GID1.0049669 | | | | | | |  | | |
| 159055 | | GID1.0049681 | | | | | | |  | | |
| 159072 | | GID1.0049698 | | | | | | |  | | |
| 159079 | | GID1.0049705 | | | | | | |  | | |
| 159081 | | GID1.0049707 | | | | | | |  | | |
| 159084 | | GID1.0049710 | | | | | | |  | | |
| 159092 | | GID1.0049718 | | | | | | |  | | |
| 159112 | | GID1.0049738 | | | | | | |  | | |
| 159121 | | GID1.0049747 | | | | | | |  | | |
| 159129 | | GID1.0049755 | | | | | | |  | | |
| 159140 | | GID1.0049766 | | | | | | |  | | |
| 159150 | | GID1.0049776 | | | | | | |  | | |
| 159154 | | GID1.0049780 | | | | | | |  | | |
| 159162 | | GID1.0049788 | | | | | | |  | | |
| 159169 | | GID1.0049795 | | | | | | |  | | |
| 159186 | | GID1.0049812 | | | | | | |  | | |
| 159192 | | GID1.0049818 | | | | | | |  | | |
| 159239 | | GID1.0049865 | | | | | | |  | | |
| 159242 | | GID1.0049868 | | | | | | |  | | |
| 159276 | | GID1.0049902 | | | | | | |  | | |
| 159329 | | GID1.0049955 | | | | | | |  | | |
| 159355 | | GID1.0049981 | | | | | | |  | | |
| 159360 | | GID1.0049986 | | | | | | |  | | |
| 159393 | | GID1.0050019 | | | | | | |  | | |
| 159425 | | GID1.0050051 | | | | | | |  | | |
| 159426 | | GID1.0050052 | | | | | | |  | | |
| 159452 | | GID1.0050078 | | | | | | |  | | |
| 159493 | | GID1.0050119 | | | | | | |  | | |
| 159501 | | GID1.0050127 | | | | | | |  | | |
| 159504 | | GID1.0050130 | | | | | | |  | | |
| 159508 | | GID1.0050134 | | | | | | |  | | |
| 159532 | | GID1.0050158 | | | | | | |  | | |
| 159545 | | GID1.0050171 | | | | | | |  | | |
| 159551 | | GID1.0050177 | | | | | | |  | | |
| 159613 | | GID1.0050239 | | | | | | |  | | |
| 159622 | | GID1.0050248 | | | | | | |  | | |
| 159631 | | GID1.0050257 | | | | | | |  | | |
| 159637 | | GID1.0050263 | | | | | | |  | | |
| 159676 | | GID1.0050302 | | | | | | |  | | |
| 159687 | | GID1.0050313 | | | | | | |  | | |
| 159734 | | GID1.0050360 | | | | | | |  | | |
| 159750 | | GID1.0050376 | | | | | | |  | | |
| 159796 | | GID1.0050416 | | | | | | |  | | |
| 159799 | | GID1.0050419 | | | | | | |  | | |
| 159808 | | GID1.0050428 | | | | | | |  | | |
| 159813 | | GID1.0050433 | | | | | | |  | | |
| 159816 | | GID1.0050436 | | | | | | |  | | |
| 159896 | | GID1.0050516 | | | | | | |  | | |
| 159898 | | GID1.0050518 | | | | | | |  | | |
| 159900 | | GID1.0050520 | | | | | | |  | | |
| 159930 | | GID1.0050550 | | | | | | |  | | |
| 159931 | | GID1.0050551 | | | | | | |  | | |
| 159932 | | GID1.0050552 | | | | | | |  | | |
| 159936 | | GID1.0050556 | | | | | | |  | | |
| 159937 | | GID1.0050557 | | | | | | |  | | |
| 159949 | | GID1.0050569 | | | | | | |  | | |
| 159982 | | GID1.0050602 | | | | | | |  | | |
| 159993 | | GID1.0050613 | | | | | | |  | | |
| 160009 | | GID1.0050623 | | | | | | |  | | |
| 160010 | | GID1.0050624 | | | | | | |  | | |
| 160013 | | GID1.0050627 | | | | | | |  | | |
| 160017 | | GID1.0050631 | | | | | | |  | | |
| 160031 | | GID1.0050645 | | | | | | |  | | |
| 160049 | | GID1.0050663 | | | | | | |  | | |
| 160066 | | GID1.0050680 | | | | | | |  | | |
| 160068 | | GID1.0050682 | | | | | | |  | | |
| 160079 | | GID1.0050693 | | | | | | |  | | |
| 160088 | | GID1.0050702 | | | | | | |  | | |
| 160091 | | GID1.0050705 | | | | | | |  | | |
| 160104 | | GID1.0050718 | | | | | | |  | | |
| 160136 | | GID1.0050745 | | | | | | |  | | |
| 160153 | | GID1.0050757 | | | | | | |  | | |
| 160158 | | GID1.0050762 | | | | | | |  | | |
| 160171 | | GID1.0050775 | | | | | | |  | | |
| 160189 | | GID1.0050788 | | | | | | |  | | |
| 160200 | | GID1.0050799 | | | | | | |  | | |
| 160223 | | GID1.0050822 | | | | | | |  | | |
| 160229 | | GID1.0050828 | | | | | | |  | | |
| 160234 | | GID1.0050833 | | | | | | |  | | |
| 160249 | | GID1.0050848 | | | | | | |  | | |
| 160265 | | GID1.0050864 | | | | | | |  | | |
| 160294 | | GID1.0050886 | | | | | | |  | | |
| 160295 | | GID1.0050887 | | | | | | |  | | |
| 160365 | | GID1.0050952 | | | | | | |  | | |
| 160371 | | GID1.0050958 | | | | | | |  | | |
| 160378 | | GID1.0050965 | | | | | | |  | | |
| 160382 | | GID1.0050969 | | | | | | |  | | |
| 160398 | | GID1.0050985 | | | | | | |  | | |
| 160399 | | GID1.0050986 | | | | | | |  | | |
| 160425 | | GID1.0051004 | | | | | | |  | | |
| 160477 | | GID1.0051034 | | | | | | |  | | |
| 160510 | | GID1.0051057 | | | | | | |  | | |
| 16069 | | gw1.86.31.1 | | | | | | |  | | |
| 16622 | | gw1.114.14.1 | | | | | | |  | | |
| 16645 | | gw1.195.23.1 | | | | | | |  | | |
| 16769 | | gw1.16.57.1 | | | | | | |  | | |
| 16837 | | gw1.12.48.1 | | | | | | |  | | |
| 16881 | | gw1.244.10.1 | | | | | | |  | | |
| 16899 | | gw1.67.21.1 | | | | | | |  | | |
| 17103 | | gw1.89.38.1 | | | | | | |  | | |
| 17674 | | gw1.125.31.1 | | | | | | |  | | |
| 17773 | | gw1.163.30.1 | | | | | | |  | | |
| 17819 | | gw1.16.64.1 | | | | | | |  | | |
| 17821 | | gw1.182.25.1 | | | | | | |  | | |
| 18109 | | gw1.117.30.1 | | | | | | |  | | |
| 18179 | | gw1.182.26.1 | | | | | | |  | | |
| 18207 | | gw1.183.28.1 | | | | | | |  | | |
| 18224 | | gw1.117.31.1 | | | | | | |  | | |
| 18249 | | gw1.199.18.1 | | | | | | |  | | |
| 18369 | | gw1.1.137.1 | | | | | | |  | | |
| 18395 | | gw1.185.15.1 | | | | | | |  | | |
| 18408 | | gw1.10.64.1 | | | | | | |  | | |
| 18410 | | gw1.120.38.1 | | | | | | |  | | |
| 18418 | | gw1.57.39.1 | | | | | | |  | | |
| 18421 | | gw1.239.29.1 | | | | | | |  | | |
| 18495 | | gw1.73.46.1 | | | | | | |  | | |
| 18554 | | gw1.167.33.1 | | | | | | |  | | |
| 18577 | | gw1.120.39.1 | | | | | | |  | | |
| 18650 | | gw1.114.23.1 | | | | | | |  | | |
| 18684 | | gw1.3.89.1 | | | |  | | |  | | |
| 18761 | | gw1.191.14.1 | | | | | | |  | | |
| 18839 | | gw1.91.30.1 | | | | | | |  | | |
| 18843 | | gw1.17.59.1 | | | | | | |  | | |
| 18996 | | gw1.2.85.1 | | | |  | | |  | | |
| 19217 | | gw1.71.6.1 | | | |  | | |  | | |
| 19228 | | gw1.48.32.1 | | | | | | |  | | |
| 19369 | | gw1.14.71.1 | | | | | | |  | | |
| 19388 | | gw1.20.69.1 | | | | | | |  | | |
| 19394 | | gw1.4.82.1 | | | |  | | |  | | |
| 19420 | | gw1.26.87.1 | | | | | | |  | | |
| 19461 | | gw1.223.25.1 | | | | | | |  | | |
| 19470 | | gw1.13.60.1 | | | | | | |  | | |
| 19530 | | gw1.49.59.1 | | | | | | |  | | |
| 19579 | | gw1.78.40.1 | | | | | | |  | | |
| 19583 | | gw1.19.44.1 | | | | | | |  | | |
| 19597 | | gw1.83.6.1 | | | |  | | |  | | |
| 19701 | | gw1.11.73.1 | | | | | | |  | | |
| 19832 | | gw1.10.71.1 | | | | | | |  | | |
| 19937 | | gw1.20.73.1 | | | | | | |  | | |
| 20023 | | gw1.17.68.1 | | | | | | |  | | |
| 20568 | | gw1.137.41.1 | | | | | | |  | | |
| 20587 | | gw1.221.31.1 | | | | | | |  | | |
| 20605 | | gw1.119.42.1 | | | | | | |  | | |
| 20666 | | gw1.25.79.1 | | | | | | |  | | |
| 20697 | | gw1.160.42.1 | | | | | | |  | | |
| 20816 | | gw1.264.21.1 | | | | | | |  | | |
| 20820 | | gw1.255.16.1 | | | | | | |  | | |
| 21024 | | gw1.80.59.1 | | | | | | |  | | |
| 21115 | | gw1.120.46.1 | | | | | | |  | | |
| 21135 | | gw1.189.16.1 | | | | | | |  | | |
| 21421 | | gw1.90.32.1 | | | | | | |  | | |
| 21602 | | gw1.90.34.1 | | | | | | |  | | |
| 21604 | | gw1.19.63.1 | | | | | | |  | | |
| 21623 | | gw1.190.33.1 | | | | | | |  | | |
| 21640 | | gw1.119.48.1 | | | | | | |  | | |
| 21659 | | gw1.17.86.1 | | | | | | |  | | |
| 21660 | | gw1.17.87.1 | | | | | | |  | | |
| 21681 | | gw1.20.88.1 | | | | | | |  | | |
| 21687 | | gw1.49.76.1 | | | | | | |  | | |
| 21969 | | gw1.7.142.1 | | | | | | |  | | |
| 22083 | | gw1.45.60.1 | | | | | | |  | | |
| 22089 | | gw1.79.44.1 | | | | | | |  | | |
| 22412 | | gw1.35.59.1 | | | | | | |  | | |
| 22458 | | gw1.96.39.1 | | | | | | |  | | |
| 22794 | | gw1.116.34.1 | | | | | | |  | | |
| 22820 | | gw1.18.67.1 | | | | | | |  | | |
| 22918 | | gw1.222.13.1 | | | | | | |  | | |
| 23207 | | gw1.19.96.1 | | | | | | |  | | |
| 23451 | | gw1.101.34.1 | | | | | | |  | | |
| 23586 | | gw1.226.48.1 | | | | | | |  | | |
| 23669 | | gw1.21.98.1 | | | | | | |  | | |
| 23676 | | gw1.6.134.1 | | | | | | |  | | |
| 23677 | | gw1.4.116.1 | | | | | | |  | | |
| 23706 | | gw1.3.166.1 | | | | | | |  | | |
| 23784 | | gw1.63.57.1 | | | | | | |  | | |
| 23821 | | gw1.36.100.1 | | | | | | |  | | |
| 23823 | | gw1.189.22.1 | | | | | | |  | | |
| 23830 | | gw1.2.139.1 | | | | | | |  | | |
| 23835 | | gw1.18.74.1 | | | | | | |  | | |
| 23841 | | gw1.45.67.1 | | | | | | |  | | |
| 23859 | | gw1.21.99.1 | | | | | | |  | | |
| 23904 | | gw1.182.54.1 | | | | | | |  | | |
| 24118 | | gw1.56.45.1 | | | | | | |  | | |
| 24131 | | gw1.9.71.1 | | | |  | | |  | | |
| 24132 | | gw1.110.41.1 | | | | | | |  | | |
| 24137 | | gw1.9.72.1 | | | |  | | |  | | |
| 24139 | | gw1.9.73.1 | | | |  | | |  | | |
| 24140 | | gw1.3.170.1 | | | | | | |  | | |
| 24144 | | gw1.119.51.1 | | | | | | |  | | |
| 24153 | | gw1.159.20.1 | | | | | | |  | | |
| 24178 | | gw1.9.74.1 | | | |  | | |  | | |
| 24530 | | gw1.4.122.1 | | | | | | |  | | |
| 24589 | | gw1.180.40.1 | | | | | | |  | | |
| 24641 | | gw1.278.35.1 | | | | | | |  | | |
| 24662 | | gw1.194.47.1 | | | | | | |  | | |
| 24690 | | gw1.279.25.1 | | | | | | |  | | |
| 24773 | | gw1.714.9.1 | | | | | | |  | | |
| 24800 | | gw1.762.3.1 | | | | | | |  | | |
| 24875 | | e_gw1.1.103.1 | | | | | | |  | | |
| 24880 | | e_gw1.1.109.1 | | | | | | |  | | |
| 24909 | | e_gw1.1.35.1 | | | | | | |  | | |
| 24943 | | e_gw1.1.161.1 | | | | | | |  | | |
| 25037 | | e_gw1.1.115.1 | | | | | | |  | | |
| 25087 | | e_gw1.2.129.1 | | | | | | |  | | |
| 25123 | | e_gw1.2.102.1 | | | | | | |  | | |
| 25125 | | e_gw1.2.31.1 | | | | | | |  | | |
| 25145 | | e_gw1.2.38.1 | | | | | | |  | | |
| 25157 | | e_gw1.2.122.1 | | | | | | |  | | |
| 25174 | | e_gw1.2.115.1 | | | | | | |  | | |
| 25187 | | e_gw1.3.132.1 | | | | | | |  | | |
| 25267 | | e_gw1.3.123.1 | | | | | | |  | | |
| 25268 | | e_gw1.3.141.1 | | | | | | |  | | |
| 25407 | | e_gw1.4.40.1 | | | | | | |  | | |
| 25410 | | e_gw1.4.54.1 | | | | | | |  | | |
| 25457 | | e_gw1.4.108.1 | | | | | | |  | | |
| 25559 | | e_gw1.6.103.1 | | | | | | |  | | |
| 25577 | | e_gw1.6.123.1 | | | | | | |  | | |
| 25613 | | e_gw1.6.69.1 | | | | | | |  | | |
| 25657 | | e_gw1.6.102.1 | | | | | | |  | | |
| 25724 | | e_gw1.7.135.1 | | | | | | |  | | |
| 25810 | | e_gw1.7.138.1 | | | | | | |  | | |
| 26008 | | e_gw1.9.27.1 | | | | | | |  | | |
| 26079 | | e_gw1.10.68.1 | | | | | | |  | | |
| 26153 | | e_gw1.10.5.1 | | | | | | |  | | |
| 26414 | | e_gw1.13.109.1 | | | | | | |  | | |
| 26515 | | e_gw1.13.95.1 | | | | | | |  | | |
| 26531 | | e_gw1.14.58.1 | | | | | | |  | | |
| 26632 | | e_gw1.15.38.1 | | | | | | |  | | |
| 26751 | | e_gw1.16.87.1 | | | | | | |  | | |
| 26791 | | e_gw1.16.78.1 | | | | | | |  | | |
| 26825 | | e_gw1.17.90.1 | | | | | | |  | | |
| 26866 | | e_gw1.17.63.1 | | | | | | |  | | |
| 26881 | | e_gw1.17.33.1 | | | | | | |  | | |
| 26886 | | e_gw1.17.26.1 | | | | | | |  | | |
| 26888 | | e_gw1.17.32.1 | | | | | | |  | | |
| 26907 | | e_gw1.17.91.1 | | | | | | |  | | |
| 26908 | | e_gw1.17.92.1 | | | | | | |  | | |
| 26917 | | e_gw1.18.84.1 | | | | | | |  | | |
| 26958 | | e_gw1.18.26.1 | | | | | | |  | | |
| 27131 | | e_gw1.19.41.1 | | | | | | |  | | |
| 27133 | | e_gw1.19.176.1 | | | | | | |  | | |
| 27137 | | e_gw1.19.48.1 | | | | | | |  | | |
| 27204 | | e_gw1.20.35.1 | | | | | | |  | | |
| 27222 | | e_gw1.20.41.1 | | | | | | |  | | |
| 27227 | | e_gw1.20.38.1 | | | | | | |  | | |
| 27259 | | e_gw1.20.19.1 | | | | | | |  | | |
| 27275 | | e_gw1.20.4.1 | | | | | | |  | | |
| 27507 | | e_gw1.23.70.1 | | | | | | |  | | |
| 27579 | | e_gw1.24.51.1 | | | | | | |  | | |
| 27694 | | e_gw1.25.45.1 | | | | | | |  | | |
| 27934 | | e_gw1.28.83.1 | | | | | | |  | | |
| 27943 | | e_gw1.28.34.1 | | | | | | |  | | |
| 27966 | | e_gw1.28.55.1 | | | | | | |  | | |
| 28021 | | e_gw1.28.70.1 | | | | | | |  | | |
| 28046 | | e_gw1.29.65.1 | | | | | | |  | | |
| 28057 | | e_gw1.29.66.1 | | | | | | |  | | |
| 28096 | | e_gw1.29.55.1 | | | | | | |  | | |
| 28115 | | e_gw1.30.56.1 | | | | | | |  | | |
| 28127 | | e_gw1.30.57.1 | | | | | | |  | | |
| 28215 | | e_gw1.31.39.1 | | | | | | |  | | |
| 28281 | | e_gw1.32.52.1 | | | | | | |  | | |
| 28384 | | e_gw1.33.91.1 | | | | | | |  | | |
| 28404 | | e_gw1.33.25.1 | | | | | | |  | | |
| 28418 | | e_gw1.33.7.1 | | | | | | |  | | |
| 28428 | | e_gw1.34.59.1 | | | | | | |  | | |
| 28444 | | e_gw1.34.71.1 | | | | | | |  | | |
| 28523 | | e_gw1.35.14.1 | | | | | | |  | | |
| 28539 | | e_gw1.35.42.1 | | | | | | |  | | |
| 28544 | | e_gw1.35.34.1 | | | | | | |  | | |
| 28616 | | e_gw1.36.6.1 | | | | | | |  | | |
| 28619 | | e_gw1.36.89.1 | | | | | | |  | | |
| 28679 | | e_gw1.37.50.1 | | | | | | |  | | |
| 28690 | | e_gw1.37.25.1 | | | | | | |  | | |
| 28723 | | e_gw1.37.40.1 | | | | | | |  | | |
| 28816 | | e_gw1.38.41.1 | | | | | | |  | | |
| 28834 | | e_gw1.38.46.1 | | | | | | |  | | |
| 28978 | | e_gw1.40.68.1 | | | | | | |  | | |
| 28991 | | e_gw1.40.49.1 | | | | | | |  | | |
| 29016 | | e_gw1.40.53.1 | | | | | | |  | | |
| 29106 | | e_gw1.41.59.1 | | | | | | |  | | |
| 29193 | | e_gw1.43.39.1 | | | | | | |  | | |
| 29272 | | e_gw1.44.27.1 | | | | | | |  | | |
| 29455 | | e_gw1.47.49.1 | | | | | | |  | | |
| 29461 | | e_gw1.47.68.1 | | | | | | |  | | |
| 29506 | | e_gw1.48.11.1 | | | | | | |  | | |
| 29588 | | e_gw1.49.29.1 | | | | | | |  | | |
| 29615 | | e_gw1.49.47.1 | | | | | | |  | | |
| 29652 | | e_gw1.50.53.1 | | | | | | |  | | |
| 29680 | | e_gw1.50.74.1 | | | | | | |  | | |
| 29825 | | e_gw1.52.40.1 | | | | | | |  | | |
| 29874 | | e_gw1.53.37.1 | | | | | | |  | | |
| 29973 | | e_gw1.54.43.1 | | | | | | |  | | |
| 30018 | | e_gw1.55.29.1 | | | | | | |  | | |
| 30216 | | e_gw1.58.11.1 | | | | | | |  | | |
| 30324 | | e_gw1.60.67.1 | | | | | | |  | | |
| 30335 | | e_gw1.60.3.1 | | | | | | |  | | |
| 30524 | | e_gw1.63.44.1 | | | | | | |  | | |
| 30544 | | e_gw1.63.17.1 | | | | | | |  | | |
| 30558 | | e_gw1.64.52.1 | | | | | | |  | | |
| 30603 | | e_gw1.64.46.1 | | | | | | |  | | |
| 30692 | | e_gw1.66.52.1 | | | | | | |  | | |
| 30722 | | e_gw1.66.39.1 | | | | | | |  | | |
| 30800 | | e_gw1.68.57.1 | | | | | | |  | | |
| 30830 | | e_gw1.68.37.1 | | | | | | |  | | |
| 30860 | | e_gw1.68.63.1 | | | | | | |  | | |
| 30913 | | e_gw1.69.16.1 | | | | | | |  | | |
| 30965 | | e_gw1.70.49.1 | | | | | | |  | | |
| 31259 | | e_gw1.76.68.1 | | | | | | |  | | |
| 31348 | | e_gw1.77.25.1 | | | | | | |  | | |
| 31357 | | e_gw1.77.24.1 | | | | | | |  | | |
| 31378 | | e_gw1.78.35.1 | | | | | | |  | | |
| 31532 | | e_gw1.80.50.1 | | | | | | |  | | |
| 31694 | | e_gw1.84.11.1 | | | | | | |  | | |
| 31748 | | e_gw1.85.26.1 | | | | | | |  | | |
| 31836 | | e_gw1.86.34.1 | | | | | | |  | | |
| 31970 | | e_gw1.89.16.1 | | | | | | |  | | |
| 31981 | | e_gw1.89.40.1 | | | | | | |  | | |
| 31983 | | e_gw1.89.46.1 | | | | | | |  | | |
| 32029 | | e_gw1.89.48.1 | | | | | | |  | | |
| 32093 | | e_gw1.91.22.1 | | | | | | |  | | |
| 32114 | | e_gw1.91.34.1 | | | | | | |  | | |
| 32136 | | e_gw1.92.27.1 | | | | | | |  | | |
| 32198 | | e_gw1.94.47.1 | | | | | | |  | | |
| 32261 | | e_gw1.95.37.1 | | | | | | |  | | |
| 32271 | | e_gw1.95.27.1 | | | | | | |  | | |
| 32360 | | e_gw1.97.30.1 | | | | | | |  | | |
| 32493 | | e_gw1.100.19.1 | | | | | | |  | | |
| 32589 | | e_gw1.102.40.1 | | | | | | |  | | |
| 32600 | | e_gw1.102.47.1 | | | | | | |  | | |
| 32880 | | e_gw1.108.33.1 | | | | | | |  | | |
| 32968 | | e_gw1.110.42.1 | | | | | | |  | | |
| 32986 | | e_gw1.110.31.1 | | | | | | |  | | |
| 33016 | | e_gw1.111.42.1 | | | | | | |  | | |
| 33021 | | e_gw1.111.41.1 | | | | | | |  | | |
| 33217 | | e_gw1.115.18.1 | | | | | | |  | | |
| 33223 | | e_gw1.115.54.1 | | | | | | |  | | |
| 33247 | | e_gw1.116.30.1 | | | | | | |  | | |
| 33347 | | e_gw1.118.48.1 | | | | | | |  | | |
| 33350 | | e_gw1.118.9.1 | | | | | | |  | | |
| 33494 | | e_gw1.121.44.1 | | | | | | |  | | |
| 33573 | | e_gw1.123.17.1 | | | | | | |  | | |
| 33678 | | e_gw1.125.39.1 | | | | | | |  | | |
| 33697 | | e_gw1.126.15.1 | | | | | | |  | | |
| 33703 | | e_gw1.126.23.1 | | | | | | |  | | |
| 33785 | | e_gw1.128.36.1 | | | | | | |  | | |
| 33801 | | e_gw1.128.47.1 | | | | | | |  | | |
| 33826 | | e_gw1.129.10.1 | | | | | | |  | | |
| 33844 | | e_gw1.129.23.1 | | | | | | |  | | |
| 33846 | | e_gw1.129.27.1 | | | | | | |  | | |
| 33897 | | e_gw1.130.30.1 | | | | | | |  | | |
| 34047 | | e_gw1.134.24.1 | | | | | | |  | | |
| 34065 | | e_gw1.134.25.1 | | | | | | |  | | |
| 34092 | | e_gw1.135.14.1 | | | | | | |  | | |
| 34222 | | e_gw1.138.21.1 | | | | | | |  | | |
| 34230 | | e_gw1.138.37.1 | | | | | | |  | | |
| 34235 | | e_gw1.138.20.1 | | | | | | |  | | |
| 34245 | | e_gw1.138.29.1 | | | | | | |  | | |
| 34411 | | e_gw1.142.24.1 | | | | | | |  | | |
| 34423 | | e_gw1.143.31.1 | | | | | | |  | | |
| 34444 | | e_gw1.143.34.1 | | | | | | |  | | |
| 34489 | | e_gw1.144.9.1 | | | | | | |  | | |
| 34543 | | e_gw1.146.14.1 | | | | | | |  | | |
| 34568 | | e_gw1.147.13.1 | | | | | | |  | | |
| 34575 | | e_gw1.147.19.1 | | | | | | |  | | |
| 34641 | | e_gw1.149.13.1 | | | | | | |  | | |
| 34650 | | e_gw1.149.6.1 | | | | | | |  | | |
| 34656 | | e_gw1.149.28.1 | | | | | | |  | | |
| 34699 | | e_gw1.150.43.1 | | | | | | |  | | |
| 34985 | | e_gw1.156.35.1 | | | | | | |  | | |
| 34999 | | e_gw1.157.38.1 | | | | | | |  | | |
| 35211 | | e_gw1.162.11.1 | | | | | | |  | | |
| 35222 | | e_gw1.163.54.1 | | | | | | |  | | |
| 35226 | | e_gw1.163.24.1 | | | | | | |  | | |
| 35246 | | e_gw1.163.60.1 | | | | | | |  | | |
| 35628 | | e_gw1.171.37.1 | | | | | | |  | | |
| 35661 | | e_gw1.173.40.1 | | | | | | |  | | |
| 35721 | | e_gw1.175.28.1 | | | | | | |  | | |
| 35951 | | e_gw1.180.17.1 | | | | | | |  | | |
| 35956 | | e_gw1.180.20.1 | | | | | | |  | | |
| 35975 | | e_gw1.180.3.1 | | | | | | |  | | |
| 36074 | | e_gw1.183.16.1 | | | | | | |  | | |
| 36089 | | e_gw1.183.32.1 | | | | | | |  | | |
| 36225 | | e_gw1.187.18.1 | | | | | | |  | | |
| 36268 | | e_gw1.190.36.1 | | | | | | |  | | |
| 36298 | | e_gw1.191.21.1 | | | | | | |  | | |
| 36311 | | e_gw1.191.27.1 | | | | | | |  | | |
| 36315 | | e_gw1.192.28.1 | | | | | | |  | | |
| 36390 | | e_gw1.194.24.1 | | | | | | |  | | |
| 36395 | | e_gw1.194.10.1 | | | | | | |  | | |
| 36456 | | e_gw1.195.32.1 | | | | | | |  | | |
| 36533 | | e_gw1.198.26.1 | | | | | | |  | | |
| 36588 | | e_gw1.199.16.1 | | | | | | |  | | |
| 36589 | | e_gw1.199.6.1 | | | | | | |  | | |
| 36615 | | e_gw1.200.20.1 | | | | | | |  | | |
| 36636 | | e_gw1.200.25.1 | | | | | | |  | | |
| 36683 | | e_gw1.201.27.1 | | | | | | |  | | |
| 36696 | | e_gw1.202.37.1 | | | | | | |  | | |
| 36716 | | e_gw1.202.13.1 | | | | | | |  | | |
| 36836 | | e_gw1.207.17.1 | | | | | | |  | | |
| 36866 | | e_gw1.207.13.1 | | | | | | |  | | |
| 36874 | | e_gw1.208.30.1 | | | | | | |  | | |
| 36890 | | e_gw1.208.11.1 | | | | | | |  | | |
| 36894 | | e_gw1.208.36.1 | | | | | | |  | | |
| 36946 | | e_gw1.210.5.1 | | | | | | |  | | |
| 36979 | | e_gw1.210.20.1 | | | | | | |  | | |
| 37055 | | e_gw1.212.42.1 | | | | | | |  | | |
| 37110 | | e_gw1.214.36.1 | | | | | | |  | | |
| 37191 | | e_gw1.216.39.1 | | | | | | |  | | |
| 37274 | | e_gw1.219.9.1 | | | | | | |  | | |
| 37279 | | e_gw1.220.4.1 | | | | | | |  | | |
| 37355 | | e_gw1.223.32.1 | | | | | | |  | | |
| 37392 | | e_gw1.224.23.1 | | | | | | |  | | |
| 37398 | | e_gw1.224.4.1 | | | | | | |  | | |
| 37473 | | e_gw1.226.32.1 | | | | | | |  | | |
| 37644 | | e_gw1.231.32.1 | | | | | | |  | | |
| 37686 | | e_gw1.232.25.1 | | | | | | |  | | |
| 37832 | | e_gw1.237.6.1 | | | | | | |  | | |
| 37835 | | e_gw1.237.32.1 | | | | | | |  | | |
| 37853 | | e_gw1.237.33.1 | | | | | | |  | | |
| 37855 | | e_gw1.237.15.1 | | | | | | |  | | |
| 38177 | | e_gw1.246.17.1 | | | | | | |  | | |
| 38203 | | e_gw1.247.33.1 | | | | | | |  | | |
| 38252 | | e_gw1.248.16.1 | | | | | | |  | | |
| 38388 | | e_gw1.253.22.1 | | | | | | |  | | |
| 38391 | | e_gw1.253.19.1 | | | | | | |  | | |
| 38425 | | e_gw1.254.29.1 | | | | | | |  | | |
| 38429 | | e_gw1.254.30.1 | | | | | | |  | | |
| 38569 | | e_gw1.260.15.1 | | | | | | |  | | |
| 38588 | | e_gw1.261.11.1 | | | | | | |  | | |
| 38611 | | e_gw1.261.20.1 | | | | | | |  | | |
| 38629 | | e_gw1.262.9.1 | | | | | | |  | | |
| 38722 | | e_gw1.268.25.1 | | | | | | |  | | |
| 38743 | | e_gw1.269.26.1 | | | | | | |  | | |
| 38752 | | e_gw1.269.17.1 | | | | | | |  | | |
| 38762 | | e_gw1.269.7.1 | | | | | | |  | | |
| 38775 | | e_gw1.269.15.1 | | | | | | |  | | |
| 38788 | | e_gw1.270.15.1 | | | | | | |  | | |
| 38792 | | e_gw1.270.21.1 | | | | | | |  | | |
| 38843 | | e_gw1.272.22.1 | | | | | | |  | | |
| 38852 | | e_gw1.272.4.1 | | | | | | |  | | |
| 38883 | | e_gw1.273.15.1 | | | | | | |  | | |
| 38985 | | e_gw1.276.18.1 | | | | | | |  | | |
| 39101 | | e_gw1.280.16.1 | | | | | | |  | | |
| 39232 | | e_gw1.285.14.1 | | | | | | |  | | |
| 39291 | | e_gw1.287.30.1 | | | | | | |  | | |
| 39457 | | e_gw1.294.9.1 | | | | | | |  | | |
| 39462 | | e_gw1.294.14.1 | | | | | | |  | | |
| 39465 | | e_gw1.294.5.1 | | | | | | |  | | |
| 39591 | | e_gw1.299.19.1 | | | | | | |  | | |
| 39778 | | e_gw1.308.26.1 | | | | | | |  | | |
| 39824 | | e_gw1.310.11.1 | | | | | | |  | | |
| 39826 | | e_gw1.310.6.1 | | | | | | |  | | |
| 39837 | | e_gw1.310.32.1 | | | | | | |  | | |
| 39877 | | e_gw1.313.14.1 | | | | | | |  | | |
| 39879 | | e_gw1.313.9.1 | | | | | | |  | | |
| 39890 | | e_gw1.313.16.1 | | | | | | |  | | |
| 39910 | | e_gw1.314.5.1 | | | | | | |  | | |
| 39933 | | e_gw1.315.9.1 | | | | | | |  | | |
| 39962 | | e_gw1.316.7.1 | | | | | | |  | | |
| 40017 | | e_gw1.319.2.1 | | | | | | |  | | |
| 40083 | | e_gw1.321.8.1 | | | | | | |  | | |
| 40145 | | e_gw1.324.3.1 | | | | | | |  | | |
| 40191 | | e_gw1.326.4.1 | | | | | | |  | | |
| 40261 | | e_gw1.330.6.1 | | | | | | |  | | |
| 40278 | | e_gw1.330.18.1 | | | | | | |  | | |
| 40286 | | e_gw1.331.1.1 | | | | | | |  | | |
| 40318 | | e_gw1.332.17.1 | | | | | | |  | | |
| 40402 | | e_gw1.335.10.1 | | | | | | |  | | |
| 40477 | | e_gw1.339.12.1 | | | | | | |  | | |
| 40536 | | e_gw1.342.2.1 | | | | | | |  | | |
| 40546 | | e_gw1.342.3.1 | | | | | | |  | | |
| 40548 | | e_gw1.342.21.1 | | | | | | |  | | |
| 40565 | | e_gw1.343.6.1 | | | | | | |  | | |
| 40704 | | e_gw1.349.6.1 | | | | | | |  | | |
| 40708 | | e_gw1.349.17.1 | | | | | | |  | | |
| 40793 | | e_gw1.354.3.1 | | | | | | |  | | |
| 40804 | | e_gw1.354.6.1 | | | | | | |  | | |
| 40819 | | e_gw1.355.15.1 | | | | | | |  | | |
| 40821 | | e_gw1.355.7.1 | | | | | | |  | | |
| 40826 | | e_gw1.355.26.1 | | | | | | |  | | |
| 41003 | | e_gw1.365.10.1 | | | | | | |  | | |
| 41027 | | e_gw1.367.10.1 | | | | | | |  | | |
| 41061 | | e_gw1.368.12.1 | | | | | | |  | | |
| 41097 | | e_gw1.370.5.1 | | | | | | |  | | |
| 41102 | | e_gw1.370.13.1 | | | | | | |  | | |
| 41106 | | e_gw1.371.6.1 | | | | | | |  | | |
| 41108 | | e_gw1.371.2.1 | | | | | | |  | | |
| 41155 | | e_gw1.371.4.1 | | | | | | |  | | |
| 41201 | | e_gw1.375.14.1 | | | | | | |  | | |
| 41210 | | e_gw1.375.23.1 | | | | | | |  | | |
| 41231 | | e_gw1.376.3.1 | | | | | | |  | | |
| 41326 | | e_gw1.382.6.1 | | | | | | |  | | |
| 41371 | | e_gw1.386.21.1 | | | | | | |  | | |
| 41424 | | e_gw1.389.5.1 | | | | | | |  | | |
| 41458 | | e_gw1.391.12.1 | | | | | | |  | | |
| 41502 | | e_gw1.394.10.1 | | | | | | |  | | |
| 41509 | | e_gw1.394.5.1 | | | | | | |  | | |
| 41520 | | e_gw1.395.4.1 | | | | | | |  | | |
| 41521 | | e_gw1.395.9.1 | | | | | | |  | | |
| 41541 | | e_gw1.396.8.1 | | | | | | |  | | |
| 41580 | | e_gw1.398.10.1 | | | | | | |  | | |
| 41654 | | e_gw1.401.7.1 | | | | | | |  | | |
| 41742 | | e_gw1.407.12.1 | | | | | | |  | | |
| 41864 | | e_gw1.415.8.1 | | | | | | |  | | |
| 41889 | | e_gw1.416.6.1 | | | | | | |  | | |
| 41979 | | e_gw1.423.18.1 | | | | | | |  | | |
| 42055 | | e_gw1.428.7.1 | | | | | | |  | | |
| 42082 | | e_gw1.431.11.1 | | | | | | |  | | |
| 42092 | | e_gw1.431.12.1 | | | | | | |  | | |
| 42148 | | e_gw1.436.6.1 | | | | | | |  | | |
| 42183 | | e_gw1.439.3.1 | | | | | | |  | | |
| 42301 | | e_gw1.448.11.1 | | | | | | |  | | |
| 42388 | | e_gw1.455.13.1 | | | | | | |  | | |
| 42396 | | e_gw1.455.21.1 | | | | | | |  | | |
| 42423 | | e_gw1.457.11.1 | | | | | | |  | | |
| 42444 | | e_gw1.458.1.1 | | | | | | |  | | |
| 42446 | | e_gw1.458.10.1 | | | | | | |  | | |
| 42475 | | e_gw1.461.8.1 | | | | | | |  | | |
| 42480 | | e_gw1.462.2.1 | | | | | | |  | | |
| 42538 | | e_gw1.467.2.1 | | | | | | |  | | |
| 42576 | | e_gw1.471.2.1 | | | | | | |  | | |
| 42586 | | e_gw1.471.10.1 | | | | | | |  | | |
| 42595 | | e_gw1.472.9.1 | | | | | | |  | | |
| 42617 | | e_gw1.473.9.1 | | | | | | |  | | |
| 42628 | | e_gw1.474.2.1 | | | | | | |  | | |
| 42674 | | e_gw1.478.10.1 | | | | | | |  | | |
| 42680 | | e_gw1.478.1.1 | | | | | | |  | | |
| 42718 | | e_gw1.482.9.1 | | | | | | |  | | |
| 42743 | | e_gw1.484.13.1 | | | | | | |  | | |
| 42812 | | e_gw1.489.14.1 | | | | | | |  | | |
| 42820 | | e_gw1.489.2.1 | | | | | | |  | | |
| 42864 | | e_gw1.493.1.1 | | | | | | |  | | |
| 42895 | | e_gw1.498.8.1 | | | | | | |  | | |
| 42897 | | e_gw1.498.3.1 | | | | | | |  | | |
| 42899 | | e_gw1.498.10.1 | | | | | | |  | | |
| 42973 | | e_gw1.510.9.1 | | | | | | |  | | |
| 43033 | | e_gw1.513.2.1 | | | | | | |  | | |
| 43040 | | e_gw1.515.2.1 | | | | | | |  | | |
| 43082 | | e_gw1.520.7.1 | | | | | | |  | | |
| 43087 | | e_gw1.520.10.1 | | | | | | |  | | |
| 43246 | | e_gw1.537.13.1 | | | | | | |  | | |
| 43262 | | e_gw1.539.2.1 | | | | | | |  | | |
| 43334 | | e_gw1.551.4.1 | | | | | | |  | | |
| 43381 | | e_gw1.555.3.1 | | | | | | |  | | |
| 43581 | | e_gw1.575.3.1 | | | | | | |  | | |
| 43619 | | e_gw1.582.2.1 | | | | | | |  | | |
| 43628 | | e_gw1.584.6.1 | | | | | | |  | | |
| 43640 | | e_gw1.585.1.1 | | | | | | |  | | |
| 43717 | | e_gw1.596.7.1 | | | | | | |  | | |
| 43722 | | e_gw1.597.4.1 | | | | | | |  | | |
| 43761 | | e_gw1.602.3.1 | | | | | | |  | | |
| 43797 | | e_gw1.606.4.1 | | | | | | |  | | |
| 43840 | | e_gw1.613.4.1 | | | | | | |  | | |
| 43894 | | e_gw1.622.2.1 | | | | | | |  | | |
| 43938 | | e_gw1.629.4.1 | | | | | | |  | | |
| 43951 | | e_gw1.630.15.1 | | | | | | |  | | |
| 43969 | | e_gw1.632.3.1 | | | | | | |  | | |
| 44058 | | e_gw1.650.1.1 | | | | | | |  | | |
| 44091 | | e_gw1.659.5.1 | | | | | | |  | | |
| 44248 | | e_gw1.699.1.1 | | | | | | |  | | |
| 44312 | | e_gw1.720.2.1 | | | | | | |  | | |
| 44321 | | e_gw1.725.2.1 | | | | | | |  | | |
| 44441 | | e_gw1.773.1.1 | | | | | | |  | | |
| 44485 | | e_gw1.812.1.1 | | | | | | |  | | |
| 44491 | | e_gw1.814.1.1 | | | | | | |  | | |
| 44541 | | estExt_Genewise1.C_20045 | | | | | | |  | | |
| 44602 | | estExt_Genewise1.C_30113 | | | | | | |  | | |
| 45100 | | estExt_Genewise1.C_140087 | | | | | | |  | | |
| 4515 | | gw1.394.3.1 | | | | | | |  | | |
| 45505 | | estExt_Genewise1.C_250090 | | | | | | |  | | |
| 45645 | | estExt_Genewise1.C_290016 | | | | | | |  | | |
| 45676 | | estExt_Genewise1.C_300025 | | | | | | |  | | |
| 45800 | | estExt_Genewise1.C_350011 | | | | | | |  | | |
| 46140 | | estExt_Genewise1.C_470027 | | | | | | |  | | |
| 46519 | | estExt_Genewise1.C_600002 | | | | | | |  | | |
| 46831 | | estExt_Genewise1.C_720046 | | | | | | |  | | |
| 47068 | | estExt_Genewise1.C_840007 | | | | | | |  | | |
| 47080 | | estExt_Genewise1.C_840045 | | | | | | |  | | |
| 47162 | | estExt_Genewise1.C_870028 | | | | | | |  | | |
| 47250 | | estExt_Genewise1.C_930005 | | | | | | |  | | |
| 47371 | | estExt_Genewise1.C_1010025 | | | | | | |  | | |
| 47417 | | estExt_Genewise1.C_1030023 | | | | | | |  | | |
| 47774 | | estExt_Genewise1.C_1230042 | | | | | | |  | | |
| 47852 | | estExt_Genewise1.C_1300033 | | | | | | |  | | |
| 47873 | | estExt_Genewise1.C_1310018 | | | | | | |  | | |
| 47947 | | estExt_Genewise1.C_1360005 | | | | | | |  | | |
| 48018 | | estExt_Genewise1.C_1400001 | | | | | | |  | | |
| 48126 | | estExt_Genewise1.C_1470014 | | | | | | |  | | |
| 48221 | | estExt_Genewise1.C_1520031 | | | | | | |  | | |
| 48394 | | estExt_Genewise1.C_1620003 | | | | | | |  | | |
| 48717 | | estExt_Genewise1.C_1820036 | | | | | | |  | | |
| 48793 | | estExt_Genewise1.C_1890009 | | | | | | |  | | |
| 48822 | | estExt_Genewise1.C_1910012 | | | | | | |  | | |
| 48914 | | estExt_Genewise1.C_2010026 | | | | | | |  | | |
| 49064 | | estExt_Genewise1.C_2130013 | | | | | | |  | | |
| 4908 | | gw1.399.1.1 | | | | | | |  | | |
| 49131 | | estExt_Genewise1.C_2170009 | | | | | | |  | | |
| 49369 | | estExt_Genewise1.C_2370009 | | | | | | |  | | |
| 49420 | | estExt_Genewise1.C_2410001 | | | | | | |  | | |
| 49886 | | estExt_Genewise1.C_2810007 | | | | | | |  | | |
| 50425 | | estExt_Genewise1.C_3410011 | | | | | | |  | | |
| 50561 | | estExt_Genewise1.C_3590028 | | | | | | |  | | |
| 50740 | | estExt_Genewise1.C_3900003 | | | | | | |  | | |
| 50844 | | estExt_Genewise1.C_4050010 | | | | | | |  | | |
| 50943 | | estExt_Genewise1.C_4210013 | | | | | | |  | | |
| 50951 | | estExt_Genewise1.C_4230003 | | | | | | |  | | |
| 51478 | | estExt_Genewise1.C_5500004 | | | | | | |  | | |
| 51509 | | estExt_Genewise1.C_5580005 | | | | | | |  | | |
| 51633 | | estExt_Genewise1.C_5970014 | | | | | | |  | | |
| 51750 | | estExt_Genewise1.C_6410003 | | | | | | |  | | |
| 51827 | | estExt_Genewise1.C_6890001 | | | | | | |  | | |
| 51834 | | estExt_Genewise1.C_6940001 | | | | | | |  | | |
| 51961 | | estExt_Genewise1Plus.C_10071 | | | | | | |  | | |
| 51972 | | estExt_Genewise1Plus.C_10095 | | | | | | |  | | |
| 51976 | | estExt_Genewise1Plus.C_10127 | | | | | | |  | | |
| 52042 | | estExt_Genewise1Plus.C_30009 | | | | | | |  | | |
| 52087 | | estExt_Genewise1Plus.C_40013 | | | | | | |  | | |
| 52532 | | estExt_Genewise1Plus.C_130126 | | | | | | |  | | |
| 52761 | | estExt_Genewise1Plus.C_200096 | | | | | | |  | | |
| 52786 | | estExt_Genewise1Plus.C_210032 | | | | | | |  | | |
| 52884 | | estExt_Genewise1Plus.C_230051 | | | | | | |  | | |
| 53135 | | estExt_Genewise1Plus.C_290064 | | | | | | |  | | |
| 53210 | | estExt_Genewise1Plus.C_320070 | | | | | | |  | | |
| 53304 | | estExt_Genewise1Plus.C_360018 | | | | | | |  | | |
| 53490 | | estExt_Genewise1Plus.C_430015 | | | | | | |  | | |
| 53662 | | estExt_Genewise1Plus.C_480043 | | | | | | |  | | |
| 53683 | | estExt_Genewise1Plus.C_490016 | | | | | | |  | | |
| 53869 | | estExt_Genewise1Plus.C_550031 | | | | | | |  | | |
| 53928 | | estExt_Genewise1Plus.C_570059 | | | | | | |  | | |
| 54009 | | estExt_Genewise1Plus.C_600035 | | | | | | |  | | |
| 54015 | | estExt_Genewise1Plus.C_600053 | | | | | | |  | | |
| 54069 | | estExt_Genewise1Plus.C_620024 | | | | | | |  | | |
| 54214 | | estExt_Genewise1Plus.C_680019 | | | | | | |  | | |
| 54298 | | estExt_Genewise1Plus.C_720011 | | | | | | |  | | |
| 54420 | | estExt_Genewise1Plus.C_760049 | | | | | | |  | | |
| 54554 | | estExt_Genewise1Plus.C_840025 | | | | | | |  | | |
| 54559 | | estExt_Genewise1Plus.C_840033 | | | | | | |  | | |
| 54607 | | estExt_Genewise1Plus.C_860002 | | | | | | |  | | |
| 54676 | | estExt_Genewise1Plus.C_890048 | | | | | | |  | | |
| 54851 | | estExt_Genewise1Plus.C_1000046 | | | | | | |  | | |
| 5486 | | gw1.476.2.1 | | | | | | |  | | |
| 55004 | | estExt_Genewise1Plus.C_1080011 | | | | | | |  | | |
| 55328 | | estExt_Genewise1Plus.C_1290023 | | | | | | |  | | |
| 55507 | | estExt_Genewise1Plus.C_1390042 | | | | | | |  | | |
| 55517 | | estExt_Genewise1Plus.C_1400009 | | | | | | |  | | |
| 55541 | | estExt_Genewise1Plus.C_1410004 | | | | | | |  | | |
| 55588 | | estExt_Genewise1Plus.C_1440002 | | | | | | |  | | |
| 55593 | | estExt_Genewise1Plus.C_1440011 | | | | | | |  | | |
| 55899 | | estExt_Genewise1Plus.C_1620013 | | | | | | |  | | |
| 56055 | | estExt_Genewise1Plus.C_1720002 | | | | | | |  | | |
| 56203 | | estExt_Genewise1Plus.C_1820016 | | | | | | |  | | |
| 56217 | | estExt_Genewise1Plus.C_1820032 | | | | | | |  | | |
| 56276 | | estExt_Genewise1Plus.C_1850026 | | | | | | |  | | |
| 56540 | | estExt_Genewise1Plus.C_2110025 | | | | | | |  | | |
| 56580 | | estExt_Genewise1Plus.C_2140004 | | | | | | |  | | |
| 5674 | | gw1.82.6.1 | | | |  | | |  | | |
| 56925 | | estExt_Genewise1Plus.C_2400026 | | | | | | |  | | |
| 57144 | | estExt_Genewise1Plus.C_2570016 | | | | | | |  | | |
| 57285 | | estExt_Genewise1Plus.C_2730020 | | | | | | |  | | |
| 57286 | | estExt_Genewise1Plus.C_2730022 | | | | | | |  | | |
| 57459 | | estExt_Genewise1Plus.C_2870030 | | | | | | |  | | |
| 57493 | | estExt_Genewise1Plus.C_2920005 | | | | | | |  | | |
| 57504 | | estExt_Genewise1Plus.C_2930012 | | | | | | |  | | |
| 57657 | | estExt_Genewise1Plus.C_3070015 | | | | | | |  | | |
| 57688 | | estExt_Genewise1Plus.C_3120013 | | | | | | |  | | |
| 57722 | | estExt_Genewise1Plus.C_3150021 | | | | | | |  | | |
| 57884 | | estExt_Genewise1Plus.C_3350018 | | | | | | |  | | |
| 57957 | | estExt_Genewise1Plus.C_3420017 | | | | | | |  | | |
| 58162 | | estExt_Genewise1Plus.C_3700009 | | | | | | |  | | |
| 58236 | | estExt_Genewise1Plus.C_3840010 | | | | | | |  | | |
| 58554 | | estExt_Genewise1Plus.C_4400012 | | | | | | |  | | |
| 58784 | | estExt_Genewise1Plus.C_4900012 | | | | | | |  | | |
| 59115 | | estExt_Genewise1Plus.C_5800005 | | | | | | |  | | |
| 59376 | | estExt_Genewise1Plus.C_7010001 | | | | | | |  | | |
| 5964 | | gw1.488.3.1 | | | | | | |  | | |
| 6017 | | gw1.432.5.1 | | | | | | |  | | |
| 6027 | | gw1.349.4.1 | | | | | | |  | | |
| 6028 | | gw1.551.2.1 | | | | | | |  | | |
| 6117 | | gw1.483.3.1 | | | | | | |  | | |
| 6348 | | gw1.291.5.1 | | | | | | |  | | |
| 6628 | | gw1.670.4.1 | | | | | | |  | | |
| 6690 | | gw1.337.5.1 | | | | | | |  | | |
| 6826 | | gw1.88.7.1 | | | |  | | |  | | |
| 71387 | | fgeneshDP_kg.C_scaffold_5000009 | | | | | | |  | | |
| 71595 | | fgeneshDP_kg.C_scaffold_21000011 | | | | | | |  | | |
| 71962 | | fgeneshDP_kg.C_scaffold_57000005 | | | | | | |  | | |
| 72172 | | fgeneshDP_kg.C_scaffold_83000007 | | | | | | |  | | |
| 72223 | | fgeneshDP_kg.C_scaffold_91000002 | | | | | | |  | | |
| 72244 | | fgeneshDP_kg.C_scaffold_93000006 | | | | | | |  | | |
| 72359 | | fgeneshDP_kg.C_scaffold_111000007 | | | | | | |  | | |
| 72679 | | fgeneshDP_kg.C_scaffold_173000006 | | | | | | |  | | |
| 72750 | | fgeneshDP_kg.C_scaffold_185000002 | | | | | | |  | | |
| 73240 | | fgeneshDP_kg.C_scaffold_321000004 | | | | | | |  | | |
| 73373 | | fgeneshDP_kg.C_scaffold_368000002 | | | | | | |  | | |
| 73423 | | fgeneshDP_kg.C_scaffold_391000001 | | | | | | |  | | |
| 73526 | | fgeneshDP_kg.C_scaffold_437000002 | | | | | | |  | | |
| 73532 | | fgeneshDP_kg.C_scaffold_441000001 | | | | | | |  | | |
| 73554 | | fgeneshDP_kg.C_scaffold_453000001 | | | | | | |  | | |
| 7370 | | gw1.310.20.1 | | | | | | |  | | |
| 73705 | | fgeneshDP_kg.C_scaffold_572000001 | | | | | | |  | | |
| 73725 | | fgeneshDP_kg.C_scaffold_597000002 | | | | | | |  | | |
| 73840 | | fgeneshDP_pg.C_scaffold_1000003 | | | | | | |  | | |
| 73854 | | fgeneshDP_pg.C_scaffold_1000017 | | | | | | |  | | |
| 73857 | | fgeneshDP_pg.C_scaffold_1000020 | | | | | | |  | | |
| 73865 | | fgeneshDP_pg.C_scaffold_1000028 | | | | | | |  | | |
| 73872 | | fgeneshDP_pg.C_scaffold_1000035 | | | | | | |  | | |
| 73873 | | fgeneshDP_pg.C_scaffold_1000036 | | | | | | |  | | |
| 73876 | | fgeneshDP_pg.C_scaffold_1000039 | | | | | | |  | | |
| 73890 | | fgeneshDP_pg.C_scaffold_1000053 | | | | | | |  | | |
| 73900 | | fgeneshDP_pg.C_scaffold_1000063 | | | | | | |  | | |
| 73904 | | fgeneshDP_pg.C_scaffold_1000067 | | | | | | |  | | |
| 73911 | | fgeneshDP_pg.C_scaffold_1000074 | | | | | | |  | | |
| 73913 | | fgeneshDP_pg.C_scaffold_1000076 | | | | | | |  | | |
| 73920 | | fgeneshDP_pg.C_scaffold_1000083 | | | | | | |  | | |
| 73922 | | fgeneshDP_pg.C_scaffold_1000085 | | | | | | |  | | |
| 73924 | | fgeneshDP_pg.C_scaffold_1000087 | | | | | | |  | | |
| 73925 | | fgeneshDP_pg.C_scaffold_1000088 | | | | | | |  | | |
| 73927 | | fgeneshDP_pg.C_scaffold_1000090 | | | | | | |  | | |
| 73928 | | fgeneshDP_pg.C_scaffold_1000091 | | | | | | |  | | |
| 73930 | | fgeneshDP_pg.C_scaffold_1000093 | | | | | | |  | | |
| 73933 | | fgeneshDP_pg.C_scaffold_1000096 | | | | | | |  | | |
| 73934 | | fgeneshDP_pg.C_scaffold_1000097 | | | | | | |  | | |
| 73937 | | fgeneshDP_pg.C_scaffold_1000100 | | | | | | |  | | |
| 73944 | | fgeneshDP_pg.C_scaffold_2000001 | | | | | | |  | | |
| 73962 | | fgeneshDP_pg.C_scaffold_2000019 | | | | | | |  | | |
| 73963 | | fgeneshDP_pg.C_scaffold_2000020 | | | | | | |  | | |
| 73966 | | fgeneshDP_pg.C_scaffold_2000023 | | | | | | |  | | |
| 73979 | | fgeneshDP_pg.C_scaffold_2000036 | | | | | | |  | | |
| 73981 | | fgeneshDP_pg.C_scaffold_2000038 | | | | | | |  | | |
| 73989 | | fgeneshDP_pg.C_scaffold_2000046 | | | | | | |  | | |
| 73991 | | fgeneshDP_pg.C_scaffold_2000048 | | | | | | |  | | |
| 74005 | | fgeneshDP_pg.C_scaffold_2000062 | | | | | | |  | | |
| 74010 | | fgeneshDP_pg.C_scaffold_2000067 | | | | | | |  | | |
| 74017 | | fgeneshDP_pg.C_scaffold_2000074 | | | | | | |  | | |
| 74019 | | fgeneshDP_pg.C_scaffold_2000076 | | | | | | |  | | |
| 74023 | | fgeneshDP_pg.C_scaffold_2000080 | | | | | | |  | | |
| 74027 | | fgeneshDP_pg.C_scaffold_2000084 | | | | | | |  | | |
| 74028 | | fgeneshDP_pg.C_scaffold_2000085 | | | | | | |  | | |
| 74065 | | fgeneshDP_pg.C_scaffold_3000019 | | | | | | |  | | |
| 74076 | | fgeneshDP_pg.C_scaffold_3000030 | | | | | | |  | | |
| 74091 | | fgeneshDP_pg.C_scaffold_3000045 | | | | | | |  | | |
| 74099 | | fgeneshDP_pg.C_scaffold_3000053 | | | | | | |  | | |
| 74102 | | fgeneshDP_pg.C_scaffold_3000056 | | | | | | |  | | |
| 74109 | | fgeneshDP_pg.C_scaffold_3000063 | | | | | | |  | | |
| 74110 | | fgeneshDP_pg.C_scaffold_3000064 | | | | | | |  | | |
| 74111 | | fgeneshDP_pg.C_scaffold_3000065 | | | | | | |  | | |
| 74116 | | fgeneshDP_pg.C_scaffold_3000070 | | | | | | |  | | |
| 74129 | | fgeneshDP_pg.C_scaffold_3000083 | | | | | | |  | | |
| 74135 | | fgeneshDP_pg.C_scaffold_3000089 | | | | | | |  | | |
| 74143 | | fgeneshDP_pg.C_scaffold_4000002 | | | | | | |  | | |
| 74144 | | fgeneshDP_pg.C_scaffold_4000003 | | | | | | |  | | |
| 74167 | | fgeneshDP_pg.C_scaffold_4000026 | | | | | | |  | | |
| 74193 | | fgeneshDP_pg.C_scaffold_4000052 | | | | | | |  | | |
| 74204 | | fgeneshDP_pg.C_scaffold_4000063 | | | | | | |  | | |
| 74206 | | fgeneshDP_pg.C_scaffold_4000065 | | | | | | |  | | |
| 74208 | | fgeneshDP_pg.C_scaffold_4000067 | | | | | | |  | | |
| 74209 | | fgeneshDP_pg.C_scaffold_4000068 | | | | | | |  | | |
| 74211 | | fgeneshDP_pg.C_scaffold_4000070 | | | | | | |  | | |
| 74212 | | fgeneshDP_pg.C_scaffold_4000071 | | | | | | |  | | |
| 74219 | | fgeneshDP_pg.C_scaffold_4000078 | | | | | | |  | | |
| 74220 | | fgeneshDP_pg.C_scaffold_4000079 | | | | | | |  | | |
| 74222 | | fgeneshDP_pg.C_scaffold_4000081 | | | | | | |  | | |
| 74233 | | fgeneshDP_pg.C_scaffold_5000010 | | | | | | |  | | |
| 74234 | | fgeneshDP_pg.C_scaffold_5000011 | | | | | | |  | | |
| 74249 | | fgeneshDP_pg.C_scaffold_5000026 | | | | | | |  | | |
| 74252 | | fgeneshDP_pg.C_scaffold_5000029 | | | | | | |  | | |
| 74298 | | fgeneshDP_pg.C_scaffold_6000016 | | | | | | |  | | |
| 74305 | | fgeneshDP_pg.C_scaffold_6000023 | | | | | | |  | | |
| 74315 | | fgeneshDP_pg.C_scaffold_6000033 | | | | | | |  | | |
| 74329 | | fgeneshDP_pg.C_scaffold_6000047 | | | | | | |  | | |
| 74345 | | fgeneshDP_pg.C_scaffold_6000063 | | | | | | |  | | |
| 74353 | | fgeneshDP_pg.C_scaffold_6000071 | | | | | | |  | | |
| 74354 | | fgeneshDP_pg.C_scaffold_6000072 | | | | | | |  | | |
| 74358 | | fgeneshDP_pg.C_scaffold_7000004 | | | | | | |  | | |
| 74366 | | fgeneshDP_pg.C_scaffold_7000012 | | | | | | |  | | |
| 74418 | | fgeneshDP_pg.C_scaffold_7000064 | | | | | | |  | | |
| 74419 | | fgeneshDP_pg.C_scaffold_7000065 | | | | | | |  | | |
| 74420 | | fgeneshDP_pg.C_scaffold_7000066 | | | | | | |  | | |
| 74427 | | fgeneshDP_pg.C_scaffold_7000073 | | | | | | |  | | |
| 74428 | | fgeneshDP_pg.C_scaffold_7000074 | | | | | | |  | | |
| 74453 | | fgeneshDP_pg.C_scaffold_8000018 | | | | | | |  | | |
| 74455 | | fgeneshDP_pg.C_scaffold_8000020 | | | | | | |  | | |
| 74456 | | fgeneshDP_pg.C_scaffold_8000021 | | | | | | |  | | |
| 74505 | | fgeneshDP_pg.C_scaffold_9000001 | | | | | | |  | | |
| 74510 | | fgeneshDP_pg.C_scaffold_9000006 | | | | | | |  | | |
| 74513 | | fgeneshDP_pg.C_scaffold_9000009 | | | | | | |  | | |
| 74514 | | fgeneshDP_pg.C_scaffold_9000010 | | | | | | |  | | |
| 74525 | | fgeneshDP_pg.C_scaffold_9000021 | | | | | | |  | | |
| 74526 | | fgeneshDP_pg.C_scaffold_9000022 | | | | | | |  | | |
| 74527 | | fgeneshDP_pg.C_scaffold_9000023 | | | | | | |  | | |
| 74528 | | fgeneshDP_pg.C_scaffold_9000024 | | | | | | |  | | |
| 74533 | | fgeneshDP_pg.C_scaffold_9000029 | | | | | | |  | | |
| 74587 | | fgeneshDP_pg.C_scaffold_10000029 | | | | | | |  | | |
| 74604 | | fgeneshDP_pg.C_scaffold_10000046 | | | | | | |  | | |
| 74608 | | fgeneshDP_pg.C_scaffold_10000050 | | | | | | |  | | |
| 74610 | | fgeneshDP_pg.C_scaffold_10000052 | | | | | | |  | | |
| 74639 | | fgeneshDP_pg.C_scaffold_11000018 | | | | | | |  | | |
| 74660 | | fgeneshDP_pg.C_scaffold_11000039 | | | | | | |  | | |
| 74670 | | fgeneshDP_pg.C_scaffold_11000049 | | | | | | |  | | |
| 74671 | | fgeneshDP_pg.C_scaffold_11000050 | | | | | | |  | | |
| 74674 | | fgeneshDP_pg.C_scaffold_12000002 | | | | | | |  | | |
| 74701 | | fgeneshDP_pg.C_scaffold_12000029 | | | | | | |  | | |
| 74702 | | fgeneshDP_pg.C_scaffold_12000030 | | | | | | |  | | |
| 74704 | | fgeneshDP_pg.C_scaffold_12000032 | | | | | | |  | | |
| 74709 | | fgeneshDP_pg.C_scaffold_12000037 | | | | | | |  | | |
| 74722 | | fgeneshDP_pg.C_scaffold_12000050 | | | | | | |  | | |
| 74740 | | fgeneshDP_pg.C_scaffold_13000004 | | | | | | |  | | |
| 74767 | | fgeneshDP_pg.C_scaffold_13000031 | | | | | | |  | | |
| 74775 | | fgeneshDP_pg.C_scaffold_13000039 | | | | | | |  | | |
| 74778 | | fgeneshDP_pg.C_scaffold_13000042 | | | | | | |  | | |
| 74797 | | fgeneshDP_pg.C_scaffold_13000061 | | | | | | |  | | |
| 74801 | | fgeneshDP_pg.C_scaffold_14000001 | | | | | | |  | | |
| 74835 | | fgeneshDP_pg.C_scaffold_14000035 | | | | | | |  | | |
| 74842 | | fgeneshDP_pg.C_scaffold_14000042 | | | | | | |  | | |
| 74852 | | fgeneshDP_pg.C_scaffold_14000052 | | | | | | |  | | |
| 7486 | | gw1.477.4.1 | | | | | | |  | | |
| 74877 | | fgeneshDP_pg.C_scaffold_15000020 | | | | | | |  | | |
| 74886 | | fgeneshDP_pg.C_scaffold_15000029 | | | | | | |  | | |
| 74920 | | fgeneshDP_pg.C_scaffold_16000008 | | | | | | |  | | |
| 74929 | | fgeneshDP_pg.C_scaffold_16000017 | | | | | | |  | | |
| 74935 | | fgeneshDP_pg.C_scaffold_16000023 | | | | | | |  | | |
| 74949 | | fgeneshDP_pg.C_scaffold_16000037 | | | | | | |  | | |
| 74964 | | fgeneshDP_pg.C_scaffold_16000052 | | | | | | |  | | |
| 74975 | | fgeneshDP_pg.C_scaffold_17000007 | | | | | | |  | | |
| 74976 | | fgeneshDP_pg.C_scaffold_17000008 | | | | | | |  | | |
| 75016 | | fgeneshDP_pg.C_scaffold_17000048 | | | | | | |  | | |
| 75027 | | fgeneshDP_pg.C_scaffold_18000004 | | | | | | |  | | |
| 75030 | | fgeneshDP_pg.C_scaffold_18000007 | | | | | | |  | | |
| 75060 | | fgeneshDP_pg.C_scaffold_18000037 | | | | | | |  | | |
| 75061 | | fgeneshDP_pg.C_scaffold_18000038 | | | | | | |  | | |
| 75062 | | fgeneshDP_pg.C_scaffold_18000039 | | | | | | |  | | |
| 75066 | | fgeneshDP_pg.C_scaffold_18000043 | | | | | | |  | | |
| 75070 | | fgeneshDP_pg.C_scaffold_18000047 | | | | | | |  | | |
| 75074 | | fgeneshDP_pg.C_scaffold_18000051 | | | | | | |  | | |
| 75097 | | fgeneshDP_pg.C_scaffold_19000016 | | | | | | |  | | |
| 75102 | | fgeneshDP_pg.C_scaffold_19000021 | | | | | | |  | | |
| 75111 | | fgeneshDP_pg.C_scaffold_19000030 | | | | | | |  | | |
| 75112 | | fgeneshDP_pg.C_scaffold_19000031 | | | | | | |  | | |
| 75114 | | fgeneshDP_pg.C_scaffold_19000033 | | | | | | |  | | |
| 75116 | | fgeneshDP_pg.C_scaffold_19000035 | | | | | | |  | | |
| 75120 | | fgeneshDP_pg.C_scaffold_19000039 | | | | | | |  | | |
| 75125 | | fgeneshDP_pg.C_scaffold_19000044 | | | | | | |  | | |
| 75162 | | fgeneshDP_pg.C_scaffold_20000035 | | | | | | |  | | |
| 75182 | | fgeneshDP_pg.C_scaffold_20000055 | | | | | | |  | | |
| 75219 | | fgeneshDP_pg.C_scaffold_21000032 | | | | | | |  | | |
| 75221 | | fgeneshDP_pg.C_scaffold_21000034 | | | | | | |  | | |
| 75252 | | fgeneshDP_pg.C_scaffold_22000009 | | | | | | |  | | |
| 75255 | | fgeneshDP_pg.C_scaffold_22000012 | | | | | | |  | | |
| 75277 | | fgeneshDP_pg.C_scaffold_22000034 | | | | | | |  | | |
| 75285 | | fgeneshDP_pg.C_scaffold_22000042 | | | | | | |  | | |
| 75290 | | fgeneshDP_pg.C_scaffold_22000047 | | | | | | |  | | |
| 75314 | | fgeneshDP_pg.C_scaffold_23000015 | | | | | | |  | | |
| 75315 | | fgeneshDP_pg.C_scaffold_23000016 | | | | | | |  | | |
| 75318 | | fgeneshDP_pg.C_scaffold_23000019 | | | | | | |  | | |
| 75322 | | fgeneshDP_pg.C_scaffold_23000023 | | | | | | |  | | |
| 75324 | | fgeneshDP_pg.C_scaffold_23000025 | | | | | | |  | | |
| 75325 | | fgeneshDP_pg.C_scaffold_23000026 | | | | | | |  | | |
| 75345 | | fgeneshDP_pg.C_scaffold_24000002 | | | | | | |  | | |
| 75349 | | fgeneshDP_pg.C_scaffold_24000006 | | | | | | |  | | |
| 75352 | | fgeneshDP_pg.C_scaffold_24000009 | | | | | | |  | | |
| 75357 | | fgeneshDP_pg.C_scaffold_24000014 | | | | | | |  | | |
| 75366 | | fgeneshDP_pg.C_scaffold_24000023 | | | | | | |  | | |
| 75368 | | fgeneshDP_pg.C_scaffold_24000025 | | | | | | |  | | |
| 75373 | | fgeneshDP_pg.C_scaffold_24000030 | | | | | | |  | | |
| 75405 | | fgeneshDP_pg.C_scaffold_25000014 | | | | | | |  | | |
| 75432 | | fgeneshDP_pg.C_scaffold_25000041 | | | | | | |  | | |
| 75439 | | fgeneshDP_pg.C_scaffold_25000048 | | | | | | |  | | |
| 75441 | | fgeneshDP_pg.C_scaffold_25000050 | | | | | | |  | | |
| 75443 | | fgeneshDP_pg.C_scaffold_25000052 | | | | | | |  | | |
| 75444 | | fgeneshDP_pg.C_scaffold_25000053 | | | | | | |  | | |
| 75453 | | fgeneshDP_pg.C_scaffold_26000003 | | | | | | |  | | |
| 75508 | | fgeneshDP_pg.C_scaffold_26000058 | | | | | | |  | | |
| 75535 | | fgeneshDP_pg.C_scaffold_27000024 | | | | | | |  | | |
| 75546 | | fgeneshDP_pg.C_scaffold_27000035 | | | | | | |  | | |
| 75548 | | fgeneshDP_pg.C_scaffold_27000037 | | | | | | |  | | |
| 75556 | | fgeneshDP_pg.C_scaffold_27000045 | | | | | | |  | | |
| 75567 | | fgeneshDP_pg.C_scaffold_28000003 | | | | | | |  | | |
| 75586 | | fgeneshDP_pg.C_scaffold_28000022 | | | | | | |  | | |
| 75594 | | fgeneshDP_pg.C_scaffold_28000030 | | | | | | |  | | |
| 75596 | | fgeneshDP_pg.C_scaffold_28000032 | | | | | | |  | | |
| 75606 | | fgeneshDP_pg.C_scaffold_28000042 | | | | | | |  | | |
| 75611 | | fgeneshDP_pg.C_scaffold_28000047 | | | | | | |  | | |
| 75639 | | fgeneshDP_pg.C_scaffold_29000017 | | | | | | |  | | |
| 75643 | | fgeneshDP_pg.C_scaffold_29000021 | | | | | | |  | | |
| 75655 | | fgeneshDP_pg.C_scaffold_29000033 | | | | | | |  | | |
| 75661 | | fgeneshDP_pg.C_scaffold_29000039 | | | | | | |  | | |
| 75666 | | fgeneshDP_pg.C_scaffold_30000005 | | | | | | |  | | |
| 75674 | | fgeneshDP_pg.C_scaffold_30000013 | | | | | | |  | | |
| 75685 | | fgeneshDP_pg.C_scaffold_30000024 | | | | | | |  | | |
| 75704 | | fgeneshDP_pg.C_scaffold_30000043 | | | | | | |  | | |
| 75713 | | fgeneshDP_pg.C_scaffold_30000052 | | | | | | |  | | |
| 75727 | | fgeneshDP_pg.C_scaffold_31000014 | | | | | | |  | | |
| 75731 | | fgeneshDP_pg.C_scaffold_31000018 | | | | | | |  | | |
| 75738 | | fgeneshDP_pg.C_scaffold_31000025 | | | | | | |  | | |
| 75751 | | fgeneshDP_pg.C_scaffold_31000038 | | | | | | |  | | |
| 75757 | | fgeneshDP_pg.C_scaffold_31000044 | | | | | | |  | | |
| 75770 | | fgeneshDP_pg.C_scaffold_32000010 | | | | | | |  | | |
| 75781 | | fgeneshDP_pg.C_scaffold_32000021 | | | | | | |  | | |
| 75786 | | fgeneshDP_pg.C_scaffold_32000026 | | | | | | |  | | |
| 75815 | | fgeneshDP_pg.C_scaffold_32000055 | | | | | | |  | | |
| 75824 | | fgeneshDP_pg.C_scaffold_33000008 | | | | | | |  | | |
| 75833 | | fgeneshDP_pg.C_scaffold_33000017 | | | | | | |  | | |
| 75837 | | fgeneshDP_pg.C_scaffold_33000021 | | | | | | |  | | |
| 75839 | | fgeneshDP_pg.C_scaffold_33000023 | | | | | | |  | | |
| 75854 | | fgeneshDP_pg.C_scaffold_34000002 | | | | | | |  | | |
| 75864 | | fgeneshDP_pg.C_scaffold_34000012 | | | | | | |  | | |
| 75881 | | fgeneshDP_pg.C_scaffold_34000029 | | | | | | |  | | |
| 75943 | | fgeneshDP_pg.C_scaffold_35000040 | | | | | | |  | | |
| 75945 | | fgeneshDP_pg.C_scaffold_36000001 | | | | | | |  | | |
| 75970 | | fgeneshDP_pg.C_scaffold_36000026 | | | | | | |  | | |
| 76005 | | fgeneshDP_pg.C_scaffold_37000011 | | | | | | |  | | |
| 76007 | | fgeneshDP_pg.C_scaffold_37000013 | | | | | | |  | | |
| 76009 | | fgeneshDP_pg.C_scaffold_37000015 | | | | | | |  | | |
| 76012 | | fgeneshDP_pg.C_scaffold_37000018 | | | | | | |  | | |
| 76014 | | fgeneshDP_pg.C_scaffold_37000020 | | | | | | |  | | |
| 76015 | | fgeneshDP_pg.C_scaffold_37000021 | | | | | | |  | | |
| 76017 | | fgeneshDP_pg.C_scaffold_37000023 | | | | | | |  | | |
| 76018 | | fgeneshDP_pg.C_scaffold_37000024 | | | | | | |  | | |
| 76019 | | fgeneshDP_pg.C_scaffold_37000025 | | | | | | |  | | |
| 76021 | | fgeneshDP_pg.C_scaffold_37000027 | | | | | | |  | | |
| 76022 | | fgeneshDP_pg.C_scaffold_37000028 | | | | | | |  | | |
| 76027 | | fgeneshDP_pg.C_scaffold_37000033 | | | | | | |  | | |
| 76029 | | fgeneshDP_pg.C_scaffold_37000035 | | | | | | |  | | |
| 76035 | | fgeneshDP_pg.C_scaffold_37000041 | | | | | | |  | | |
| 76039 | | fgeneshDP_pg.C_scaffold_37000045 | | | | | | |  | | |
| 76048 | | fgeneshDP_pg.C_scaffold_38000008 | | | | | | |  | | |
| 76064 | | fgeneshDP_pg.C_scaffold_38000024 | | | | | | |  | | |
| 76065 | | fgeneshDP_pg.C_scaffold_38000025 | | | | | | |  | | |
| 76069 | | fgeneshDP_pg.C_scaffold_38000029 | | | | | | |  | | |
| 76104 | | fgeneshDP_pg.C_scaffold_39000021 | | | | | | |  | | |
| 76105 | | fgeneshDP_pg.C_scaffold_39000022 | | | | | | |  | | |
| 76128 | | fgeneshDP_pg.C_scaffold_39000045 | | | | | | |  | | |
| 76178 | | fgeneshDP_pg.C_scaffold_41000006 | | | | | | |  | | |
| 76179 | | fgeneshDP_pg.C_scaffold_41000007 | | | | | | |  | | |
| 76197 | | fgeneshDP_pg.C_scaffold_41000025 | | | | | | |  | | |
| 76200 | | fgeneshDP_pg.C_scaffold_41000028 | | | | | | |  | | |
| 76201 | | fgeneshDP_pg.C_scaffold_41000029 | | | | | | |  | | |
| 76217 | | fgeneshDP_pg.C_scaffold_42000002 | | | | | | |  | | |
| 76228 | | fgeneshDP_pg.C_scaffold_42000013 | | | | | | |  | | |
| 76231 | | fgeneshDP_pg.C_scaffold_42000016 | | | | | | |  | | |
| 76259 | | fgeneshDP_pg.C_scaffold_43000002 | | | | | | |  | | |
| 76261 | | fgeneshDP_pg.C_scaffold_43000004 | | | | | | |  | | |
| 76266 | | fgeneshDP_pg.C_scaffold_43000009 | | | | | | |  | | |
| 76273 | | fgeneshDP_pg.C_scaffold_43000016 | | | | | | |  | | |
| 76292 | | fgeneshDP_pg.C_scaffold_43000035 | | | | | | |  | | |
| 76360 | | fgeneshDP_pg.C_scaffold_45000013 | | | | | | |  | | |
| 76406 | | fgeneshDP_pg.C_scaffold_46000019 | | | | | | |  | | |
| 76449 | | fgeneshDP_pg.C_scaffold_47000023 | | | | | | |  | | |
| 76460 | | fgeneshDP_pg.C_scaffold_47000034 | | | | | | |  | | |
| 76476 | | fgeneshDP_pg.C_scaffold_48000006 | | | | | | |  | | |
| 76519 | | fgeneshDP_pg.C_scaffold_49000008 | | | | | | |  | | |
| 76521 | | fgeneshDP_pg.C_scaffold_49000010 | | | | | | |  | | |
| 76524 | | fgeneshDP_pg.C_scaffold_49000013 | | | | | | |  | | |
| 76554 | | fgeneshDP_pg.C_scaffold_49000043 | | | | | | |  | | |
| 76564 | | fgeneshDP_pg.C_scaffold_50000007 | | | | | | |  | | |
| 76588 | | fgeneshDP_pg.C_scaffold_50000031 | | | | | | |  | | |
| 76595 | | fgeneshDP_pg.C_scaffold_50000038 | | | | | | |  | | |
| 76603 | | fgeneshDP_pg.C_scaffold_50000046 | | | | | | |  | | |
| 76604 | | fgeneshDP_pg.C_scaffold_50000047 | | | | | | |  | | |
| 76605 | | fgeneshDP_pg.C_scaffold_50000048 | | | | | | |  | | |
| 76606 | | fgeneshDP_pg.C_scaffold_51000001 | | | | | | |  | | |
| 76612 | | fgeneshDP_pg.C_scaffold_51000007 | | | | | | |  | | |
| 76632 | | fgeneshDP_pg.C_scaffold_51000027 | | | | | | |  | | |
| 76642 | | fgeneshDP_pg.C_scaffold_51000037 | | | | | | |  | | |
| 76662 | | fgeneshDP_pg.C_scaffold_52000012 | | | | | | |  | | |
| 76691 | | fgeneshDP_pg.C_scaffold_53000002 | | | | | | |  | | |
| 76697 | | fgeneshDP_pg.C_scaffold_53000008 | | | | | | |  | | |
| 76704 | | fgeneshDP_pg.C_scaffold_53000015 | | | | | | |  | | |
| 76726 | | fgeneshDP_pg.C_scaffold_53000037 | | | | | | |  | | |
| 76758 | | fgeneshDP_pg.C_scaffold_54000025 | | | | | | |  | | |
| 76764 | | fgeneshDP_pg.C_scaffold_54000031 | | | | | | |  | | |
| 76778 | | fgeneshDP_pg.C_scaffold_55000008 | | | | | | |  | | |
| 76802 | | fgeneshDP_pg.C_scaffold_55000032 | | | | | | |  | | |
| 76812 | | fgeneshDP_pg.C_scaffold_56000009 | | | | | | |  | | |
| 76823 | | fgeneshDP_pg.C_scaffold_56000020 | | | | | | |  | | |
| 76824 | | fgeneshDP_pg.C_scaffold_56000021 | | | | | | |  | | |
| 76834 | | fgeneshDP_pg.C_scaffold_57000005 | | | | | | |  | | |
| 76843 | | fgeneshDP_pg.C_scaffold_57000014 | | | | | | |  | | |
| 76844 | | fgeneshDP_pg.C_scaffold_57000015 | | | | | | |  | | |
| 76853 | | fgeneshDP_pg.C_scaffold_57000024 | | | | | | |  | | |
| 76920 | | fgeneshDP_pg.C_scaffold_59000015 | | | | | | |  | | |
| 76937 | | fgeneshDP_pg.C_scaffold_59000032 | | | | | | |  | | |
| 76955 | | fgeneshDP_pg.C_scaffold_60000013 | | | | | | |  | | |
| 76981 | | fgeneshDP_pg.C_scaffold_60000039 | | | | | | |  | | |
| 76985 | | fgeneshDP_pg.C_scaffold_61000002 | | | | | | |  | | |
| 77002 | | fgeneshDP_pg.C_scaffold_61000019 | | | | | | |  | | |
| 77031 | | fgeneshDP_pg.C_scaffold_62000008 | | | | | | |  | | |
| 77037 | | fgeneshDP_pg.C_scaffold_62000014 | | | | | | |  | | |
| 77086 | | fgeneshDP_pg.C_scaffold_63000029 | | | | | | |  | | |
| 77097 | | fgeneshDP_pg.C_scaffold_64000001 | | | | | | |  | | |
| 77118 | | fgeneshDP_pg.C_scaffold_64000022 | | | | | | |  | | |
| 77119 | | fgeneshDP_pg.C_scaffold_64000023 | | | | | | |  | | |
| 77130 | | fgeneshDP_pg.C_scaffold_64000034 | | | | | | |  | | |
| 77160 | | fgeneshDP_pg.C_scaffold_65000026 | | | | | | |  | | |
| 77168 | | fgeneshDP_pg.C_scaffold_65000034 | | | | | | |  | | |
| 77205 | | fgeneshDP_pg.C_scaffold_66000034 | | | | | | |  | | |
| 77214 | | fgeneshDP_pg.C_scaffold_67000009 | | | | | | |  | | |
| 77215 | | fgeneshDP_pg.C_scaffold_67000010 | | | | | | |  | | |
| 77216 | | fgeneshDP_pg.C_scaffold_67000011 | | | | | | |  | | |
| 77234 | | fgeneshDP_pg.C_scaffold_67000029 | | | | | | |  | | |
| 77241 | | fgeneshDP_pg.C_scaffold_67000036 | | | | | | |  | | |
| 77261 | | fgeneshDP_pg.C_scaffold_68000020 | | | | | | |  | | |
| 77278 | | fgeneshDP_pg.C_scaffold_69000002 | | | | | | |  | | |
| 77297 | | fgeneshDP_pg.C_scaffold_69000021 | | | | | | |  | | |
| 77298 | | fgeneshDP_pg.C_scaffold_69000022 | | | | | | |  | | |
| 77301 | | fgeneshDP_pg.C_scaffold_69000025 | | | | | | |  | | |
| 77309 | | fgeneshDP_pg.C_scaffold_69000033 | | | | | | |  | | |
| 77336 | | fgeneshDP_pg.C_scaffold_70000023 | | | | | | |  | | |
| 77337 | | fgeneshDP_pg.C_scaffold_70000024 | | | | | | |  | | |
| 77345 | | fgeneshDP_pg.C_scaffold_70000032 | | | | | | |  | | |
| 77360 | | fgeneshDP_pg.C_scaffold_71000012 | | | | | | |  | | |
| 77367 | | fgeneshDP_pg.C_scaffold_71000019 | | | | | | |  | | |
| 77370 | | fgeneshDP_pg.C_scaffold_71000022 | | | | | | |  | | |
| 77371 | | fgeneshDP_pg.C_scaffold_71000023 | | | | | | |  | | |
| 77386 | | fgeneshDP_pg.C_scaffold_72000005 | | | | | | |  | | |
| 77394 | | fgeneshDP_pg.C_scaffold_72000013 | | | | | | |  | | |
| 77397 | | fgeneshDP_pg.C_scaffold_72000016 | | | | | | |  | | |
| 77401 | | fgeneshDP_pg.C_scaffold_72000020 | | | | | | |  | | |
| 77451 | | fgeneshDP_pg.C_scaffold_73000036 | | | | | | |  | | |
| 77466 | | fgeneshDP_pg.C_scaffold_74000011 | | | | | | |  | | |
| 77467 | | fgeneshDP_pg.C_scaffold_74000012 | | | | | | |  | | |
| 77486 | | fgeneshDP_pg.C_scaffold_74000031 | | | | | | |  | | |
| 77502 | | fgeneshDP_pg.C_scaffold_75000007 | | | | | | |  | | |
| 77541 | | fgeneshDP_pg.C_scaffold_76000012 | | | | | | |  | | |
| 77595 | | fgeneshDP_pg.C_scaffold_77000033 | | | | | | |  | | |
| 77609 | | fgeneshDP_pg.C_scaffold_78000013 | | | | | | |  | | |
| 77659 | | fgeneshDP_pg.C_scaffold_79000021 | | | | | | |  | | |
| 77682 | | fgeneshDP_pg.C_scaffold_80000009 | | | | | | |  | | |
| 77693 | | fgeneshDP_pg.C_scaffold_80000020 | | | | | | |  | | |
| 77695 | | fgeneshDP_pg.C_scaffold_80000022 | | | | | | |  | | |
| 77706 | | fgeneshDP_pg.C_scaffold_80000033 | | | | | | |  | | |
| 77757 | | fgeneshDP_pg.C_scaffold_82000007 | | | | | | |  | | |
| 77767 | | fgeneshDP_pg.C_scaffold_82000017 | | | | | | |  | | |
| 77771 | | fgeneshDP_pg.C_scaffold_82000021 | | | | | | |  | | |
| 77795 | | fgeneshDP_pg.C_scaffold_83000015 | | | | | | |  | | |
| 77798 | | fgeneshDP_pg.C_scaffold_83000018 | | | | | | |  | | |
| 77811 | | fgeneshDP_pg.C_scaffold_84000002 | | | | | | |  | | |
| 77840 | | fgeneshDP_pg.C_scaffold_84000031 | | | | | | |  | | |
| 77848 | | fgeneshDP_pg.C_scaffold_84000039 | | | | | | |  | | |
| 77849 | | fgeneshDP_pg.C_scaffold_84000040 | | | | | | |  | | |
| 77883 | | fgeneshDP_pg.C_scaffold_86000003 | | | | | | |  | | |
| 77904 | | fgeneshDP_pg.C_scaffold_87000001 | | | | | | |  | | |
| 77931 | | fgeneshDP_pg.C_scaffold_87000028 | | | | | | |  | | |
| 77933 | | fgeneshDP_pg.C_scaffold_87000030 | | | | | | |  | | |
| 77962 | | fgeneshDP_pg.C_scaffold_88000028 | | | | | | |  | | |
| 77990 | | fgeneshDP_pg.C_scaffold_90000001 | | | | | | |  | | |
| 78007 | | fgeneshDP_pg.C_scaffold_90000018 | | | | | | |  | | |
| 78030 | | fgeneshDP_pg.C_scaffold_91000011 | | | | | | |  | | |
| 78061 | | fgeneshDP_pg.C_scaffold_92000011 | | | | | | |  | | |
| 78066 | | fgeneshDP_pg.C_scaffold_92000016 | | | | | | |  | | |
| 78073 | | fgeneshDP_pg.C_scaffold_92000023 | | | | | | |  | | |
| 78088 | | fgeneshDP_pg.C_scaffold_93000003 | | | | | | |  | | |
| 78135 | | fgeneshDP_pg.C_scaffold_94000023 | | | | | | |  | | |
| 78156 | | fgeneshDP_pg.C_scaffold_95000010 | | | | | | |  | | |
| 78158 | | fgeneshDP_pg.C_scaffold_95000012 | | | | | | |  | | |
| 78159 | | fgeneshDP_pg.C_scaffold_95000013 | | | | | | |  | | |
| 78170 | | fgeneshDP_pg.C_scaffold_95000024 | | | | | | |  | | |
| 78182 | | fgeneshDP_pg.C_scaffold_96000006 | | | | | | |  | | |
| 78187 | | fgeneshDP_pg.C_scaffold_96000011 | | | | | | |  | | |
| 78189 | | fgeneshDP_pg.C_scaffold_96000013 | | | | | | |  | | |
| 7819 | | gw1.405.11.1 | | | | | | |  | | |
| 78214 | | fgeneshDP_pg.C_scaffold_97000014 | | | | | | |  | | |
| 78232 | | fgeneshDP_pg.C_scaffold_97000032 | | | | | | |  | | |
| 78242 | | fgeneshDP_pg.C_scaffold_98000009 | | | | | | |  | | |
| 78246 | | fgeneshDP_pg.C_scaffold_98000013 | | | | | | |  | | |
| 78255 | | fgeneshDP_pg.C_scaffold_98000022 | | | | | | |  | | |
| 78256 | | fgeneshDP_pg.C_scaffold_98000023 | | | | | | |  | | |
| 78265 | | fgeneshDP_pg.C_scaffold_99000006 | | | | | | |  | | |
| 78284 | | fgeneshDP_pg.C_scaffold_99000025 | | | | | | |  | | |
| 78298 | | fgeneshDP_pg.C_scaffold_100000007 | | | | | | |  | | |
| 78328 | | fgeneshDP_pg.C_scaffold_101000005 | | | | | | |  | | |
| 78362 | | fgeneshDP_pg.C_scaffold_102000007 | | | | | | |  | | |
| 78375 | | fgeneshDP_pg.C_scaffold_102000020 | | | | | | |  | | |
| 78377 | | fgeneshDP_pg.C_scaffold_102000022 | | | | | | |  | | |
| 78380 | | fgeneshDP_pg.C_scaffold_102000025 | | | | | | |  | | |
| 78381 | | fgeneshDP_pg.C_scaffold_102000026 | | | | | | |  | | |
| 78398 | | fgeneshDP_pg.C_scaffold_103000005 | | | | | | |  | | |
| 78404 | | fgeneshDP_pg.C_scaffold_103000011 | | | | | | |  | | |
| 78430 | | fgeneshDP_pg.C_scaffold_103000037 | | | | | | |  | | |
| 78436 | | fgeneshDP_pg.C_scaffold_104000005 | | | | | | |  | | |
| 78465 | | fgeneshDP_pg.C_scaffold_104000034 | | | | | | |  | | |
| 78466 | | fgeneshDP_pg.C_scaffold_104000035 | | | | | | |  | | |
| 78473 | | fgeneshDP_pg.C_scaffold_105000007 | | | | | | |  | | |
| 78511 | | fgeneshDP_pg.C_scaffold_106000025 | | | | | | |  | | |
| 78547 | | fgeneshDP_pg.C_scaffold_108000004 | | | | | | |  | | |
| 78560 | | fgeneshDP_pg.C_scaffold_108000017 | | | | | | |  | | |
| 78565 | | fgeneshDP_pg.C_scaffold_108000022 | | | | | | |  | | |
| 78589 | | fgeneshDP_pg.C_scaffold_109000017 | | | | | | |  | | |
| 78615 | | fgeneshDP_pg.C_scaffold_110000021 | | | | | | |  | | |
| 78617 | | fgeneshDP_pg.C_scaffold_110000023 | | | | | | |  | | |
| 78619 | | fgeneshDP_pg.C_scaffold_110000025 | | | | | | |  | | |
| 78653 | | fgeneshDP_pg.C_scaffold_112000006 | | | | | | |  | | |
| 78654 | | fgeneshDP_pg.C_scaffold_112000007 | | | | | | |  | | |
| 78671 | | fgeneshDP_pg.C_scaffold_113000006 | | | | | | |  | | |
| 78715 | | fgeneshDP_pg.C_scaffold_114000024 | | | | | | |  | | |
| 78719 | | fgeneshDP_pg.C_scaffold_115000001 | | | | | | |  | | |
| 78730 | | fgeneshDP_pg.C_scaffold_115000012 | | | | | | |  | | |
| 78740 | | fgeneshDP_pg.C_scaffold_115000022 | | | | | | |  | | |
| 78745 | | fgeneshDP_pg.C_scaffold_115000027 | | | | | | |  | | |
| 78747 | | fgeneshDP_pg.C_scaffold_115000029 | | | | | | |  | | |
| 78756 | | fgeneshDP_pg.C_scaffold_116000006 | | | | | | |  | | |
| 78757 | | fgeneshDP_pg.C_scaffold_116000007 | | | | | | |  | | |
| 78762 | | fgeneshDP_pg.C_scaffold_116000012 | | | | | | |  | | |
| 78800 | | fgeneshDP_pg.C_scaffold_117000021 | | | | | | |  | | |
| 78829 | | fgeneshDP_pg.C_scaffold_118000026 | | | | | | |  | | |
| 78830 | | fgeneshDP_pg.C_scaffold_119000001 | | | | | | |  | | |
| 78846 | | fgeneshDP_pg.C_scaffold_119000017 | | | | | | |  | | |
| 78868 | | fgeneshDP_pg.C_scaffold_120000014 | | | | | | |  | | |
| 78876 | | fgeneshDP_pg.C_scaffold_120000022 | | | | | | |  | | |
| 78880 | | fgeneshDP_pg.C_scaffold_120000026 | | | | | | |  | | |
| 78903 | | fgeneshDP_pg.C_scaffold_121000017 | | | | | | |  | | |
| 78913 | | fgeneshDP_pg.C_scaffold_122000001 | | | | | | |  | | |
| 78917 | | fgeneshDP_pg.C_scaffold_122000005 | | | | | | |  | | |
| 78919 | | fgeneshDP_pg.C_scaffold_122000007 | | | | | | |  | | |
| 78931 | | fgeneshDP_pg.C_scaffold_122000019 | | | | | | |  | | |
| 78950 | | fgeneshDP_pg.C_scaffold_123000007 | | | | | | |  | | |
| 78953 | | fgeneshDP_pg.C_scaffold_123000010 | | | | | | |  | | |
| 78959 | | fgeneshDP_pg.C_scaffold_123000016 | | | | | | |  | | |
| 79042 | | fgeneshDP_pg.C_scaffold_126000013 | | | | | | |  | | |
| 79055 | | fgeneshDP_pg.C_scaffold_127000001 | | | | | | |  | | |
| 79092 | | fgeneshDP_pg.C_scaffold_128000017 | | | | | | |  | | |
| 79102 | | fgeneshDP_pg.C_scaffold_129000004 | | | | | | |  | | |
| 79111 | | fgeneshDP_pg.C_scaffold_129000013 | | | | | | |  | | |
| 79120 | | fgeneshDP_pg.C_scaffold_129000022 | | | | | | |  | | |
| 7927 | | gw1.714.1.1 | | | | | | |  | | |
| 79274 | | fgeneshDP_pg.C_scaffold_135000012 | | | | | | |  | | |
| 79290 | | fgeneshDP_pg.C_scaffold_136000013 | | | | | | |  | | |
| 79293 | | fgeneshDP_pg.C_scaffold_136000016 | | | | | | |  | | |
| 79294 | | fgeneshDP_pg.C_scaffold_136000017 | | | | | | |  | | |
| 79302 | | fgeneshDP_pg.C_scaffold_136000025 | | | | | | |  | | |
| 79309 | | fgeneshDP_pg.C_scaffold_137000001 | | | | | | |  | | |
| 79331 | | fgeneshDP_pg.C_scaffold_137000023 | | | | | | |  | | |
| 79371 | | fgeneshDP_pg.C_scaffold_139000014 | | | | | | |  | | |
| 79382 | | fgeneshDP_pg.C_scaffold_139000025 | | | | | | |  | | |
| 79392 | | fgeneshDP_pg.C_scaffold_140000005 | | | | | | |  | | |
| 79436 | | fgeneshDP_pg.C_scaffold_141000023 | | | | | | |  | | |
| 79454 | | fgeneshDP_pg.C_scaffold_142000016 | | | | | | |  | | |
| 79487 | | fgeneshDP_pg.C_scaffold_143000023 | | | | | | |  | | |
| 79510 | | fgeneshDP_pg.C_scaffold_145000002 | | | | | | |  | | |
| 79514 | | fgeneshDP_pg.C_scaffold_145000006 | | | | | | |  | | |
| 79533 | | fgeneshDP_pg.C_scaffold_146000002 | | | | | | |  | | |
| 79541 | | fgeneshDP_pg.C_scaffold_146000010 | | | | | | |  | | |
| 79555 | | fgeneshDP_pg.C_scaffold_147000008 | | | | | | |  | | |
| 79572 | | fgeneshDP_pg.C_scaffold_148000007 | | | | | | |  | | |
| 79591 | | fgeneshDP_pg.C_scaffold_148000026 | | | | | | |  | | |
| 79613 | | fgeneshDP_pg.C_scaffold_149000022 | | | | | | |  | | |
| 79673 | | fgeneshDP_pg.C_scaffold_152000007 | | | | | | |  | | |
| 79687 | | fgeneshDP_pg.C_scaffold_152000021 | | | | | | |  | | |
| 79692 | | fgeneshDP_pg.C_scaffold_153000005 | | | | | | |  | | |
| 79701 | | fgeneshDP_pg.C_scaffold_153000014 | | | | | | |  | | |
| 79707 | | fgeneshDP_pg.C_scaffold_153000020 | | | | | | |  | | |
| 79711 | | fgeneshDP_pg.C_scaffold_153000024 | | | | | | |  | | |
| 79716 | | fgeneshDP_pg.C_scaffold_154000001 | | | | | | |  | | |
| 79772 | | fgeneshDP_pg.C_scaffold_156000008 | | | | | | |  | | |
| 79781 | | fgeneshDP_pg.C_scaffold_156000017 | | | | | | |  | | |
| 79811 | | fgeneshDP_pg.C_scaffold_157000025 | | | | | | |  | | |
| 79814 | | fgeneshDP_pg.C_scaffold_157000028 | | | | | | |  | | |
| 79845 | | fgeneshDP_pg.C_scaffold_159000013 | | | | | | |  | | |
| 79848 | | fgeneshDP_pg.C_scaffold_159000016 | | | | | | |  | | |
| 79852 | | fgeneshDP_pg.C_scaffold_159000020 | | | | | | |  | | |
| 79859 | | fgeneshDP_pg.C_scaffold_160000004 | | | | | | |  | | |
| 79899 | | fgeneshDP_pg.C_scaffold_161000013 | | | | | | |  | | |
| 79907 | | fgeneshDP_pg.C_scaffold_161000021 | | | | | | |  | | |
| 79968 | | fgeneshDP_pg.C_scaffold_164000011 | | | | | | |  | | |
| 79975 | | fgeneshDP_pg.C_scaffold_165000001 | | | | | | |  | | |
| 79998 | | fgeneshDP_pg.C_scaffold_165000024 | | | | | | |  | | |
| 80058 | | fgeneshDP_pg.C_scaffold_168000013 | | | | | | |  | | |
| 80059 | | fgeneshDP_pg.C_scaffold_168000014 | | | | | | |  | | |
| 80060 | | fgeneshDP_pg.C_scaffold_168000015 | | | | | | |  | | |
| 80063 | | fgeneshDP_pg.C_scaffold_168000018 | | | | | | |  | | |
| 80064 | | fgeneshDP_pg.C_scaffold_168000019 | | | | | | |  | | |
| 80079 | | fgeneshDP_pg.C_scaffold_169000011 | | | | | | |  | | |
| 80109 | | fgeneshDP_pg.C_scaffold_170000020 | | | | | | |  | | |
| 80123 | | fgeneshDP_pg.C_scaffold_171000013 | | | | | | |  | | |
| 80130 | | fgeneshDP_pg.C_scaffold_171000020 | | | | | | |  | | |
| 80131 | | fgeneshDP_pg.C_scaffold_171000021 | | | | | | |  | | |
| 80155 | | fgeneshDP_pg.C_scaffold_172000020 | | | | | | |  | | |
| 80190 | | fgeneshDP_pg.C_scaffold_174000011 | | | | | | |  | | |
| 80206 | | fgeneshDP_pg.C_scaffold_175000008 | | | | | | |  | | |
| 80207 | | fgeneshDP_pg.C_scaffold_175000009 | | | | | | |  | | |
| 80208 | | fgeneshDP_pg.C_scaffold_175000010 | | | | | | |  | | |
| 80221 | | fgeneshDP_pg.C_scaffold_176000002 | | | | | | |  | | |
| 80225 | | fgeneshDP_pg.C_scaffold_176000006 | | | | | | |  | | |
| 80286 | | fgeneshDP_pg.C_scaffold_178000023 | | | | | | |  | | |
| 80312 | | fgeneshDP_pg.C_scaffold_179000025 | | | | | | |  | | |
| 80316 | | fgeneshDP_pg.C_scaffold_180000004 | | | | | | |  | | |
| 80347 | | fgeneshDP_pg.C_scaffold_181000008 | | | | | | |  | | |
| 80348 | | fgeneshDP_pg.C_scaffold_181000009 | | | | | | |  | | |
| 80349 | | fgeneshDP_pg.C_scaffold_181000010 | | | | | | |  | | |
| 80380 | | fgeneshDP_pg.C_scaffold_182000020 | | | | | | |  | | |
| 80384 | | fgeneshDP_pg.C_scaffold_182000024 | | | | | | |  | | |
| 80399 | | fgeneshDP_pg.C_scaffold_183000001 | | | | | | |  | | |
| 80413 | | fgeneshDP_pg.C_scaffold_183000015 | | | | | | |  | | |
| 80442 | | fgeneshDP_pg.C_scaffold_185000011 | | | | | | |  | | |
| 80492 | | fgeneshDP_pg.C_scaffold_187000007 | | | | | | |  | | |
| 80509 | | fgeneshDP_pg.C_scaffold_189000009 | | | | | | |  | | |
| 80513 | | fgeneshDP_pg.C_scaffold_189000013 | | | | | | |  | | |
| 80515 | | fgeneshDP_pg.C_scaffold_189000015 | | | | | | |  | | |
| 80543 | | fgeneshDP_pg.C_scaffold_191000007 | | | | | | |  | | |
| 80554 | | fgeneshDP_pg.C_scaffold_192000003 | | | | | | |  | | |
| 80578 | | fgeneshDP_pg.C_scaffold_193000006 | | | | | | |  | | |
| 80600 | | fgeneshDP_pg.C_scaffold_193000028 | | | | | | |  | | |
| 80603 | | fgeneshDP_pg.C_scaffold_194000003 | | | | | | |  | | |
| 80605 | | fgeneshDP_pg.C_scaffold_194000005 | | | | | | |  | | |
| 80607 | | fgeneshDP_pg.C_scaffold_194000007 | | | | | | |  | | |
| 80608 | | fgeneshDP_pg.C_scaffold_194000008 | | | | | | |  | | |
| 80611 | | fgeneshDP_pg.C_scaffold_194000011 | | | | | | |  | | |
| 80612 | | fgeneshDP_pg.C_scaffold_194000012 | | | | | | |  | | |
| 80649 | | fgeneshDP_pg.C_scaffold_196000006 | | | | | | |  | | |
| 80676 | | fgeneshDP_pg.C_scaffold_197000012 | | | | | | |  | | |
| 80697 | | fgeneshDP_pg.C_scaffold_198000020 | | | | | | |  | | |
| 80699 | | fgeneshDP_pg.C_scaffold_198000022 | | | | | | |  | | |
| 80703 | | fgeneshDP_pg.C_scaffold_199000004 | | | | | | |  | | |
| 80709 | | fgeneshDP_pg.C_scaffold_199000010 | | | | | | |  | | |
| 80710 | | fgeneshDP_pg.C_scaffold_199000011 | | | | | | |  | | |
| 80717 | | fgeneshDP_pg.C_scaffold_199000018 | | | | | | |  | | |
| 80719 | | fgeneshDP_pg.C_scaffold_199000020 | | | | | | |  | | |
| 80733 | | fgeneshDP_pg.C_scaffold_200000013 | | | | | | |  | | |
| 80748 | | fgeneshDP_pg.C_scaffold_201000004 | | | | | | |  | | |
| 80754 | | fgeneshDP_pg.C_scaffold_201000010 | | | | | | |  | | |
| 80809 | | fgeneshDP_pg.C_scaffold_203000021 | | | | | | |  | | |
| 80813 | | fgeneshDP_pg.C_scaffold_204000004 | | | | | | |  | | |
| 80821 | | fgeneshDP_pg.C_scaffold_204000012 | | | | | | |  | | |
| 80825 | | fgeneshDP_pg.C_scaffold_204000016 | | | | | | |  | | |
| 80826 | | fgeneshDP_pg.C_scaffold_204000017 | | | | | | |  | | |
| 80842 | | fgeneshDP_pg.C_scaffold_205000013 | | | | | | |  | | |
| 80849 | | fgeneshDP_pg.C_scaffold_205000020 | | | | | | |  | | |
| 80858 | | fgeneshDP_pg.C_scaffold_206000008 | | | | | | |  | | |
| 80871 | | fgeneshDP_pg.C_scaffold_207000001 | | | | | | |  | | |
| 80875 | | fgeneshDP_pg.C_scaffold_207000005 | | | | | | |  | | |
| 80876 | | fgeneshDP_pg.C_scaffold_207000006 | | | | | | |  | | |
| 80888 | | fgeneshDP_pg.C_scaffold_208000005 | | | | | | |  | | |
| 80907 | | fgeneshDP_pg.C_scaffold_209000005 | | | | | | |  | | |
| 80924 | | fgeneshDP_pg.C_scaffold_210000002 | | | | | | |  | | |
| 80935 | | fgeneshDP_pg.C_scaffold_210000013 | | | | | | |  | | |
| 80939 | | fgeneshDP_pg.C_scaffold_210000017 | | | | | | |  | | |
| 80941 | | fgeneshDP_pg.C_scaffold_210000019 | | | | | | |  | | |
| 80951 | | fgeneshDP_pg.C_scaffold_211000009 | | | | | | |  | | |
| 80955 | | fgeneshDP_pg.C_scaffold_211000013 | | | | | | |  | | |
| 80966 | | fgeneshDP_pg.C_scaffold_212000003 | | | | | | |  | | |
| 80999 | | fgeneshDP_pg.C_scaffold_213000012 | | | | | | |  | | |
| 81004 | | fgeneshDP_pg.C_scaffold_213000017 | | | | | | |  | | |
| 81016 | | fgeneshDP_pg.C_scaffold_214000011 | | | | | | |  | | |
| 81027 | | fgeneshDP_pg.C_scaffold_215000004 | | | | | | |  | | |
| 81044 | | fgeneshDP_pg.C_scaffold_216000001 | | | | | | |  | | |
| 81060 | | fgeneshDP_pg.C_scaffold_217000001 | | | | | | |  | | |
| 81081 | | fgeneshDP_pg.C_scaffold_217000022 | | | | | | |  | | |
| 81086 | | fgeneshDP_pg.C_scaffold_218000005 | | | | | | |  | | |
| 81099 | | fgeneshDP_pg.C_scaffold_219000001 | | | | | | |  | | |
| 81101 | | fgeneshDP_pg.C_scaffold_219000003 | | | | | | |  | | |
| 81105 | | fgeneshDP_pg.C_scaffold_219000007 | | | | | | |  | | |
| 81125 | | fgeneshDP_pg.C_scaffold_220000008 | | | | | | |  | | |
| 81130 | | fgeneshDP_pg.C_scaffold_220000013 | | | | | | |  | | |
| 81152 | | fgeneshDP_pg.C_scaffold_222000002 | | | | | | |  | | |
| 81155 | | fgeneshDP_pg.C_scaffold_222000005 | | | | | | |  | | |
| 81156 | | fgeneshDP_pg.C_scaffold_222000006 | | | | | | |  | | |
| 81159 | | fgeneshDP_pg.C_scaffold_222000009 | | | | | | |  | | |
| 81174 | | fgeneshDP_pg.C_scaffold_223000006 | | | | | | |  | | |
| 81177 | | fgeneshDP_pg.C_scaffold_223000009 | | | | | | |  | | |
| 81190 | | fgeneshDP_pg.C_scaffold_224000002 | | | | | | |  | | |
| 81192 | | fgeneshDP_pg.C_scaffold_224000004 | | | | | | |  | | |
| 81218 | | fgeneshDP_pg.C_scaffold_225000007 | | | | | | |  | | |
| 81233 | | fgeneshDP_pg.C_scaffold_225000022 | | | | | | |  | | |
| 81247 | | fgeneshDP_pg.C_scaffold_226000009 | | | | | | |  | | |
| 81275 | | fgeneshDP_pg.C_scaffold_227000014 | | | | | | |  | | |
| 81276 | | fgeneshDP_pg.C_scaffold_227000015 | | | | | | |  | | |
| 81296 | | fgeneshDP_pg.C_scaffold_228000019 | | | | | | |  | | |
| 81297 | | fgeneshDP_pg.C_scaffold_228000020 | | | | | | |  | | |
| 81316 | | fgeneshDP_pg.C_scaffold_230000003 | | | | | | |  | | |
| 81322 | | fgeneshDP_pg.C_scaffold_230000009 | | | | | | |  | | |
| 81331 | | fgeneshDP_pg.C_scaffold_230000018 | | | | | | |  | | |
| 81345 | | fgeneshDP_pg.C_scaffold_231000014 | | | | | | |  | | |
| 81384 | | fgeneshDP_pg.C_scaffold_234000001 | | | | | | |  | | |
| 81474 | | fgeneshDP_pg.C_scaffold_239000013 | | | | | | |  | | |
| 81475 | | fgeneshDP_pg.C_scaffold_239000014 | | | | | | |  | | |
| 81531 | | fgeneshDP_pg.C_scaffold_243000007 | | | | | | |  | | |
| 81532 | | fgeneshDP_pg.C_scaffold_243000008 | | | | | | |  | | |
| 81555 | | fgeneshDP_pg.C_scaffold_244000009 | | | | | | |  | | |
| 81561 | | fgeneshDP_pg.C_scaffold_244000015 | | | | | | |  | | |
| 81562 | | fgeneshDP_pg.C_scaffold_244000016 | | | | | | |  | | |
| 81564 | | fgeneshDP_pg.C_scaffold_244000018 | | | | | | |  | | |
| 81570 | | fgeneshDP_pg.C_scaffold_245000006 | | | | | | |  | | |
| 81587 | | fgeneshDP_pg.C_scaffold_246000008 | | | | | | |  | | |
| 81598 | | fgeneshDP_pg.C_scaffold_246000019 | | | | | | |  | | |
| 81614 | | fgeneshDP_pg.C_scaffold_247000016 | | | | | | |  | | |
| 81621 | | fgeneshDP_pg.C_scaffold_248000005 | | | | | | |  | | |
| 81624 | | fgeneshDP_pg.C_scaffold_248000008 | | | | | | |  | | |
| 81636 | | fgeneshDP_pg.C_scaffold_249000005 | | | | | | |  | | |
| 81647 | | fgeneshDP_pg.C_scaffold_249000016 | | | | | | |  | | |
| 81651 | | fgeneshDP_pg.C_scaffold_250000002 | | | | | | |  | | |
| 81652 | | fgeneshDP_pg.C_scaffold_250000003 | | | | | | |  | | |
| 81655 | | fgeneshDP_pg.C_scaffold_250000006 | | | | | | |  | | |
| 81659 | | fgeneshDP_pg.C_scaffold_250000010 | | | | | | |  | | |
| 81710 | | fgeneshDP_pg.C_scaffold_253000007 | | | | | | |  | | |
| 81713 | | fgeneshDP_pg.C_scaffold_253000010 | | | | | | |  | | |
| 81716 | | fgeneshDP_pg.C_scaffold_253000013 | | | | | | |  | | |
| 81721 | | fgeneshDP_pg.C_scaffold_253000018 | | | | | | |  | | |
| 81748 | | fgeneshDP_pg.C_scaffold_255000005 | | | | | | |  | | |
| 81749 | | fgeneshDP_pg.C_scaffold_255000006 | | | | | | |  | | |
| 81752 | | fgeneshDP_pg.C_scaffold_255000009 | | | | | | |  | | |
| 81772 | | fgeneshDP_pg.C_scaffold_256000014 | | | | | | |  | | |
| 81797 | | fgeneshDP_pg.C_scaffold_257000019 | | | | | | |  | | |
| 81803 | | fgeneshDP_pg.C_scaffold_258000005 | | | | | | |  | | |
| 81810 | | fgeneshDP_pg.C_scaffold_259000001 | | | | | | |  | | |
| 81817 | | fgeneshDP_pg.C_scaffold_259000008 | | | | | | |  | | |
| 81822 | | fgeneshDP_pg.C_scaffold_259000013 | | | | | | |  | | |
| 81825 | | fgeneshDP_pg.C_scaffold_259000016 | | | | | | |  | | |
| 81850 | | fgeneshDP_pg.C_scaffold_261000002 | | | | | | |  | | |
| 81918 | | fgeneshDP_pg.C_scaffold_265000012 | | | | | | |  | | |
| 81926 | | fgeneshDP_pg.C_scaffold_266000002 | | | | | | |  | | |
| 81929 | | fgeneshDP_pg.C_scaffold_266000005 | | | | | | |  | | |
| 81940 | | fgeneshDP_pg.C_scaffold_266000016 | | | | | | |  | | |
| 81941 | | fgeneshDP_pg.C_scaffold_266000017 | | | | | | |  | | |
| 81949 | | fgeneshDP_pg.C_scaffold_267000006 | | | | | | |  | | |
| 81962 | | fgeneshDP_pg.C_scaffold_268000005 | | | | | | |  | | |
| 81963 | | fgeneshDP_pg.C_scaffold_268000006 | | | | | | |  | | |
| 81972 | | fgeneshDP_pg.C_scaffold_268000015 | | | | | | |  | | |
| 81974 | | fgeneshDP_pg.C_scaffold_268000017 | | | | | | |  | | |
| 82001 | | fgeneshDP_pg.C_scaffold_270000010 | | | | | | |  | | |
| 82004 | | fgeneshDP_pg.C_scaffold_270000013 | | | | | | |  | | |
| 82006 | | fgeneshDP_pg.C_scaffold_271000002 | | | | | | |  | | |
| 82008 | | fgeneshDP_pg.C_scaffold_271000004 | | | | | | |  | | |
| 82015 | | fgeneshDP_pg.C_scaffold_271000011 | | | | | | |  | | |
| 82025 | | fgeneshDP_pg.C_scaffold_272000001 | | | | | | |  | | |
| 82028 | | fgeneshDP_pg.C_scaffold_272000004 | | | | | | |  | | |
| 82056 | | fgeneshDP_pg.C_scaffold_273000012 | | | | | | |  | | |
| 82062 | | fgeneshDP_pg.C_scaffold_273000018 | | | | | | |  | | |
| 82094 | | fgeneshDP_pg.C_scaffold_275000014 | | | | | | |  | | |
| 82106 | | fgeneshDP_pg.C_scaffold_276000012 | | | | | | |  | | |
| 82154 | | fgeneshDP_pg.C_scaffold_279000009 | | | | | | |  | | |
| 82158 | | fgeneshDP_pg.C_scaffold_279000013 | | | | | | |  | | |
| 82173 | | fgeneshDP_pg.C_scaffold_280000010 | | | | | | |  | | |
| 82212 | | fgeneshDP_pg.C_scaffold_282000016 | | | | | | |  | | |
| 82214 | | fgeneshDP_pg.C_scaffold_282000018 | | | | | | |  | | |
| 82228 | | fgeneshDP_pg.C_scaffold_283000013 | | | | | | |  | | |
| 82246 | | fgeneshDP_pg.C_scaffold_285000002 | | | | | | |  | | |
| 82247 | | fgeneshDP_pg.C_scaffold_285000003 | | | | | | |  | | |
| 82264 | | fgeneshDP_pg.C_scaffold_286000006 | | | | | | |  | | |
| 82265 | | fgeneshDP_pg.C_scaffold_286000007 | | | | | | |  | | |
| 82266 | | fgeneshDP_pg.C_scaffold_286000008 | | | | | | |  | | |
| 82276 | | fgeneshDP_pg.C_scaffold_287000003 | | | | | | |  | | |
| 82277 | | fgeneshDP_pg.C_scaffold_287000004 | | | | | | |  | | |
| 82326 | | fgeneshDP_pg.C_scaffold_290000004 | | | | | | |  | | |
| 82327 | | fgeneshDP_pg.C_scaffold_290000005 | | | | | | |  | | |
| 82387 | | fgeneshDP_pg.C_scaffold_294000008 | | | | | | |  | | |
| 82394 | | fgeneshDP_pg.C_scaffold_295000001 | | | | | | |  | | |
| 82409 | | fgeneshDP_pg.C_scaffold_296000002 | | | | | | |  | | |
| 82434 | | fgeneshDP_pg.C_scaffold_297000011 | | | | | | |  | | |
| 82439 | | fgeneshDP_pg.C_scaffold_298000001 | | | | | | |  | | |
| 82511 | | fgeneshDP_pg.C_scaffold_302000012 | | | | | | |  | | |
| 82514 | | fgeneshDP_pg.C_scaffold_302000015 | | | | | | |  | | |
| 82538 | | fgeneshDP_pg.C_scaffold_304000010 | | | | | | |  | | |
| 82549 | | fgeneshDP_pg.C_scaffold_305000011 | | | | | | |  | | |
| 82558 | | fgeneshDP_pg.C_scaffold_306000005 | | | | | | |  | | |
| 82559 | | fgeneshDP_pg.C_scaffold_306000006 | | | | | | |  | | |
| 82561 | | fgeneshDP_pg.C_scaffold_306000008 | | | | | | |  | | |
| 82576 | | fgeneshDP_pg.C_scaffold_307000009 | | | | | | |  | | |
| 82577 | | fgeneshDP_pg.C_scaffold_307000010 | | | | | | |  | | |
| 82589 | | fgeneshDP_pg.C_scaffold_308000007 | | | | | | |  | | |
| 82596 | | fgeneshDP_pg.C_scaffold_309000001 | | | | | | |  | | |
| 82600 | | fgeneshDP_pg.C_scaffold_309000005 | | | | | | |  | | |
| 82636 | | fgeneshDP_pg.C_scaffold_312000006 | | | | | | |  | | |
| 82637 | | fgeneshDP_pg.C_scaffold_312000007 | | | | | | |  | | |
| 82644 | | fgeneshDP_pg.C_scaffold_312000014 | | | | | | |  | | |
| 82703 | | fgeneshDP_pg.C_scaffold_316000011 | | | | | | |  | | |
| 82705 | | fgeneshDP_pg.C_scaffold_317000001 | | | | | | |  | | |
| 82707 | | fgeneshDP_pg.C_scaffold_317000003 | | | | | | |  | | |
| 82710 | | fgeneshDP_pg.C_scaffold_317000006 | | | | | | |  | | |
| 82736 | | fgeneshDP_pg.C_scaffold_319000005 | | | | | | |  | | |
| 82744 | | fgeneshDP_pg.C_scaffold_320000001 | | | | | | |  | | |
| 82767 | | fgeneshDP_pg.C_scaffold_321000014 | | | | | | |  | | |
| 82769 | | fgeneshDP_pg.C_scaffold_322000001 | | | | | | |  | | |
| 82784 | | fgeneshDP_pg.C_scaffold_323000006 | | | | | | |  | | |
| 82785 | | fgeneshDP_pg.C_scaffold_323000007 | | | | | | |  | | |
| 82806 | | fgeneshDP_pg.C_scaffold_324000012 | | | | | | |  | | |
| 82847 | | fgeneshDP_pg.C_scaffold_327000007 | | | | | | |  | | |
| 82855 | | fgeneshDP_pg.C_scaffold_328000006 | | | | | | |  | | |
| 82874 | | fgeneshDP_pg.C_scaffold_329000012 | | | | | | |  | | |
| 82882 | | fgeneshDP_pg.C_scaffold_330000006 | | | | | | |  | | |
| 82898 | | fgeneshDP_pg.C_scaffold_331000007 | | | | | | |  | | |
| 82906 | | fgeneshDP_pg.C_scaffold_332000001 | | | | | | |  | | |
| 82914 | | fgeneshDP_pg.C_scaffold_332000009 | | | | | | |  | | |
| 82935 | | fgeneshDP_pg.C_scaffold_334000003 | | | | | | |  | | |
| 82967 | | fgeneshDP_pg.C_scaffold_336000013 | | | | | | |  | | |
| 82999 | | fgeneshDP_pg.C_scaffold_339000002 | | | | | | |  | | |
| 83033 | | fgeneshDP_pg.C_scaffold_341000010 | | | | | | |  | | |
| 83036 | | fgeneshDP_pg.C_scaffold_341000013 | | | | | | |  | | |
| 83052 | | fgeneshDP_pg.C_scaffold_342000011 | | | | | | |  | | |
| 83061 | | fgeneshDP_pg.C_scaffold_343000009 | | | | | | |  | | |
| 83102 | | fgeneshDP_pg.C_scaffold_346000007 | | | | | | |  | | |
| 83123 | | fgeneshDP_pg.C_scaffold_348000002 | | | | | | |  | | |
| 83124 | | fgeneshDP_pg.C_scaffold_348000003 | | | | | | |  | | |
| 83142 | | fgeneshDP_pg.C_scaffold_349000010 | | | | | | |  | | |
| 83152 | | fgeneshDP_pg.C_scaffold_350000006 | | | | | | |  | | |
| 83162 | | fgeneshDP_pg.C_scaffold_351000007 | | | | | | |  | | |
| 83201 | | fgeneshDP_pg.C_scaffold_354000009 | | | | | | |  | | |
| 83202 | | fgeneshDP_pg.C_scaffold_354000010 | | | | | | |  | | |
| 83219 | | fgeneshDP_pg.C_scaffold_355000012 | | | | | | |  | | |
| 83253 | | fgeneshDP_pg.C_scaffold_358000010 | | | | | | |  | | |
| 83254 | | fgeneshDP_pg.C_scaffold_358000011 | | | | | | |  | | |
| 83255 | | fgeneshDP_pg.C_scaffold_358000012 | | | | | | |  | | |
| 83256 | | fgeneshDP_pg.C_scaffold_358000013 | | | | | | |  | | |
| 83274 | | fgeneshDP_pg.C_scaffold_360000005 | | | | | | |  | | |
| 83291 | | fgeneshDP_pg.C_scaffold_361000010 | | | | | | |  | | |
| 83292 | | fgeneshDP_pg.C_scaffold_361000011 | | | | | | |  | | |
| 83293 | | fgeneshDP_pg.C_scaffold_361000012 | | | | | | |  | | |
| 83299 | | fgeneshDP_pg.C_scaffold_362000004 | | | | | | |  | | |
| 83328 | | fgeneshDP_pg.C_scaffold_364000013 | | | | | | |  | | |
| 83331 | | fgeneshDP_pg.C_scaffold_365000003 | | | | | | |  | | |
| 83362 | | fgeneshDP_pg.C_scaffold_367000008 | | | | | | |  | | |
| 83364 | | fgeneshDP_pg.C_scaffold_367000010 | | | | | | |  | | |
| 83371 | | fgeneshDP_pg.C_scaffold_368000006 | | | | | | |  | | |
| 83372 | | fgeneshDP_pg.C_scaffold_368000007 | | | | | | |  | | |
| 83390 | | fgeneshDP_pg.C_scaffold_369000008 | | | | | | |  | | |
| 83413 | | fgeneshDP_pg.C_scaffold_371000006 | | | | | | |  | | |
| 83415 | | fgeneshDP_pg.C_scaffold_371000008 | | | | | | |  | | |
| 83416 | | fgeneshDP_pg.C_scaffold_371000009 | | | | | | |  | | |
| 83418 | | fgeneshDP_pg.C_scaffold_371000011 | | | | | | |  | | |
| 83432 | | fgeneshDP_pg.C_scaffold_373000009 | | | | | | |  | | |
| 83433 | | fgeneshDP_pg.C_scaffold_373000010 | | | | | | |  | | |
| 83434 | | fgeneshDP_pg.C_scaffold_373000011 | | | | | | |  | | |
| 83464 | | fgeneshDP_pg.C_scaffold_376000002 | | | | | | |  | | |
| 83472 | | fgeneshDP_pg.C_scaffold_376000010 | | | | | | |  | | |
| 83500 | | fgeneshDP_pg.C_scaffold_378000015 | | | | | | |  | | |
| 83501 | | fgeneshDP_pg.C_scaffold_379000001 | | | | | | |  | | |
| 83502 | | fgeneshDP_pg.C_scaffold_379000002 | | | | | | |  | | |
| 83503 | | fgeneshDP_pg.C_scaffold_379000003 | | | | | | |  | | |
| 83521 | | fgeneshDP_pg.C_scaffold_381000002 | | | | | | |  | | |
| 83537 | | fgeneshDP_pg.C_scaffold_382000006 | | | | | | |  | | |
| 83542 | | fgeneshDP_pg.C_scaffold_382000011 | | | | | | |  | | |
| 83543 | | fgeneshDP_pg.C_scaffold_382000012 | | | | | | |  | | |
| 83554 | | fgeneshDP_pg.C_scaffold_383000009 | | | | | | |  | | |
| 83555 | | fgeneshDP_pg.C_scaffold_383000010 | | | | | | |  | | |
| 83577 | | fgeneshDP_pg.C_scaffold_385000009 | | | | | | |  | | |
| 83578 | | fgeneshDP_pg.C_scaffold_385000010 | | | | | | |  | | |
| 83589 | | fgeneshDP_pg.C_scaffold_386000009 | | | | | | |  | | |
| 83593 | | fgeneshDP_pg.C_scaffold_386000013 | | | | | | |  | | |
| 83605 | | fgeneshDP_pg.C_scaffold_388000001 | | | | | | |  | | |
| 83613 | | fgeneshDP_pg.C_scaffold_388000009 | | | | | | |  | | |
| 83617 | | fgeneshDP_pg.C_scaffold_389000003 | | | | | | |  | | |
| 83639 | | fgeneshDP_pg.C_scaffold_391000006 | | | | | | |  | | |
| 83640 | | fgeneshDP_pg.C_scaffold_391000007 | | | | | | |  | | |
| 83657 | | fgeneshDP_pg.C_scaffold_393000005 | | | | | | |  | | |
| 83666 | | fgeneshDP_pg.C_scaffold_393000014 | | | | | | |  | | |
| 83702 | | fgeneshDP_pg.C_scaffold_397000006 | | | | | | |  | | |
| 83706 | | fgeneshDP_pg.C_scaffold_397000010 | | | | | | |  | | |
| 8371 | | gw1.1.34.1 | | | |  | | |  | | |
| 83730 | | fgeneshDP_pg.C_scaffold_399000007 | | | | | | |  | | |
| 83753 | | fgeneshDP_pg.C_scaffold_401000008 | | | | | | |  | | |
| 83775 | | fgeneshDP_pg.C_scaffold_403000008 | | | | | | |  | | |
| 83785 | | fgeneshDP_pg.C_scaffold_404000006 | | | | | | |  | | |
| 83862 | | fgeneshDP_pg.C_scaffold_412000002 | | | | | | |  | | |
| 83879 | | fgeneshDP_pg.C_scaffold_414000001 | | | | | | |  | | |
| 83910 | | fgeneshDP_pg.C_scaffold_417000001 | | | | | | |  | | |
| 83925 | | fgeneshDP_pg.C_scaffold_419000002 | | | | | | |  | | |
| 83965 | | fgeneshDP_pg.C_scaffold_423000010 | | | | | | |  | | |
| 83976 | | fgeneshDP_pg.C_scaffold_425000001 | | | | | | |  | | |
| 84023 | | fgeneshDP_pg.C_scaffold_430000009 | | | | | | |  | | |
| 84044 | | fgeneshDP_pg.C_scaffold_433000002 | | | | | | |  | | |
| 84045 | | fgeneshDP_pg.C_scaffold_433000003 | | | | | | |  | | |
| 84048 | | fgeneshDP_pg.C_scaffold_433000006 | | | | | | |  | | |
| 84058 | | fgeneshDP_pg.C_scaffold_434000009 | | | | | | |  | | |
| 84060 | | fgeneshDP_pg.C_scaffold_434000011 | | | | | | |  | | |
| 84090 | | fgeneshDP_pg.C_scaffold_438000001 | | | | | | |  | | |
| 84109 | | fgeneshDP_pg.C_scaffold_439000010 | | | | | | |  | | |
| 84123 | | fgeneshDP_pg.C_scaffold_441000002 | | | | | | |  | | |
| 84141 | | fgeneshDP_pg.C_scaffold_444000004 | | | | | | |  | | |
| 84149 | | fgeneshDP_pg.C_scaffold_445000008 | | | | | | |  | | |
| 84156 | | fgeneshDP_pg.C_scaffold_446000007 | | | | | | |  | | |
| 84157 | | fgeneshDP_pg.C_scaffold_446000008 | | | | | | |  | | |
| 84166 | | fgeneshDP_pg.C_scaffold_447000007 | | | | | | |  | | |
| 84200 | | fgeneshDP_pg.C_scaffold_451000005 | | | | | | |  | | |
| 84210 | | fgeneshDP_pg.C_scaffold_453000003 | | | | | | |  | | |
| 84227 | | fgeneshDP_pg.C_scaffold_455000003 | | | | | | |  | | |
| 84230 | | fgeneshDP_pg.C_scaffold_455000006 | | | | | | |  | | |
| 84266 | | fgeneshDP_pg.C_scaffold_459000004 | | | | | | |  | | |
| 84267 | | fgeneshDP_pg.C_scaffold_459000005 | | | | | | |  | | |
| 84282 | | fgeneshDP_pg.C_scaffold_461000006 | | | | | | |  | | |
| 84286 | | fgeneshDP_pg.C_scaffold_462000003 | | | | | | |  | | |
| 84315 | | fgeneshDP_pg.C_scaffold_466000004 | | | | | | |  | | |
| 84324 | | fgeneshDP_pg.C_scaffold_467000004 | | | | | | |  | | |
| 84337 | | fgeneshDP_pg.C_scaffold_469000002 | | | | | | |  | | |
| 84366 | | fgeneshDP_pg.C_scaffold_473000001 | | | | | | |  | | |
| 84371 | | fgeneshDP_pg.C_scaffold_473000006 | | | | | | |  | | |
| 84383 | | fgeneshDP_pg.C_scaffold_475000001 | | | | | | |  | | |
| 84424 | | fgeneshDP_pg.C_scaffold_480000001 | | | | | | |  | | |
| 84430 | | fgeneshDP_pg.C_scaffold_480000007 | | | | | | |  | | |
| 84439 | | fgeneshDP_pg.C_scaffold_481000007 | | | | | | |  | | |
| 84449 | | fgeneshDP_pg.C_scaffold_483000001 | | | | | | |  | | |
| 84511 | | fgeneshDP_pg.C_scaffold_491000001 | | | | | | |  | | |
| 84523 | | fgeneshDP_pg.C_scaffold_493000001 | | | | | | |  | | |
| 84525 | | fgeneshDP_pg.C_scaffold_493000003 | | | | | | |  | | |
| 84548 | | fgeneshDP_pg.C_scaffold_496000003 | | | | | | |  | | |
| 84572 | | fgeneshDP_pg.C_scaffold_500000002 | | | | | | |  | | |
| 84589 | | fgeneshDP_pg.C_scaffold_502000004 | | | | | | |  | | |
| 84621 | | fgeneshDP_pg.C_scaffold_508000001 | | | | | | |  | | |
| 84678 | | fgeneshDP_pg.C_scaffold_517000003 | | | | | | |  | | |
| 84687 | | fgeneshDP_pg.C_scaffold_519000002 | | | | | | |  | | |
| 84702 | | fgeneshDP_pg.C_scaffold_521000005 | | | | | | |  | | |
| 84709 | | fgeneshDP_pg.C_scaffold_522000006 | | | | | | |  | | |
| 84711 | | fgeneshDP_pg.C_scaffold_523000001 | | | | | | |  | | |
| 84712 | | fgeneshDP_pg.C_scaffold_523000002 | | | | | | |  | | |
| 84713 | | fgeneshDP_pg.C_scaffold_523000003 | | | | | | |  | | |
| 84751 | | fgeneshDP_pg.C_scaffold_529000004 | | | | | | |  | | |
| 84795 | | fgeneshDP_pg.C_scaffold_537000004 | | | | | | |  | | |
| 84808 | | fgeneshDP_pg.C_scaffold_539000002 | | | | | | |  | | |
| 84814 | | fgeneshDP_pg.C_scaffold_540000003 | | | | | | |  | | |
| 84819 | | fgeneshDP_pg.C_scaffold_541000003 | | | | | | |  | | |
| 84822 | | fgeneshDP_pg.C_scaffold_542000003 | | | | | | |  | | |
| 84830 | | fgeneshDP_pg.C_scaffold_543000005 | | | | | | |  | | |
| 84843 | | fgeneshDP_pg.C_scaffold_547000001 | | | | | | |  | | |
| 84906 | | fgeneshDP_pg.C_scaffold_558000003 | | | | | | |  | | |
| 84919 | | fgeneshDP_pg.C_scaffold_560000003 | | | | | | |  | | |
| 84927 | | fgeneshDP_pg.C_scaffold_562000003 | | | | | | |  | | |
| 84938 | | fgeneshDP_pg.C_scaffold_564000005 | | | | | | |  | | |
| 84964 | | fgeneshDP_pg.C_scaffold_569000005 | | | | | | |  | | |
| 84965 | | fgeneshDP_pg.C_scaffold_569000006 | | | | | | |  | | |
| 84966 | | fgeneshDP_pg.C_scaffold_570000001 | | | | | | |  | | |
| 85002 | | fgeneshDP_pg.C_scaffold_577000001 | | | | | | |  | | |
| 85013 | | fgeneshDP_pg.C_scaffold_579000003 | | | | | | |  | | |
| 85017 | | fgeneshDP_pg.C_scaffold_579000007 | | | | | | |  | | |
| 85023 | | fgeneshDP_pg.C_scaffold_581000001 | | | | | | |  | | |
| 85026 | | fgeneshDP_pg.C_scaffold_582000002 | | | | | | |  | | |
| 85033 | | fgeneshDP_pg.C_scaffold_584000001 | | | | | | |  | | |
| 85037 | | fgeneshDP_pg.C_scaffold_584000005 | | | | | | |  | | |
| 85060 | | fgeneshDP_pg.C_scaffold_590000001 | | | | | | |  | | |
| 85064 | | fgeneshDP_pg.C_scaffold_591000001 | | | | | | |  | | |
| 85079 | | fgeneshDP_pg.C_scaffold_593000006 | | | | | | |  | | |
| 85080 | | fgeneshDP_pg.C_scaffold_593000007 | | | | | | |  | | |
| 85090 | | fgeneshDP_pg.C_scaffold_596000005 | | | | | | |  | | |
| 85108 | | fgeneshDP_pg.C_scaffold_600000002 | | | | | | |  | | |
| 85124 | | fgeneshDP_pg.C_scaffold_603000004 | | | | | | |  | | |
| 85132 | | fgeneshDP_pg.C_scaffold_605000001 | | | | | | |  | | |
| 85137 | | fgeneshDP_pg.C_scaffold_606000001 | | | | | | |  | | |
| 85174 | | fgeneshDP_pg.C_scaffold_615000001 | | | | | | |  | | |
| 85175 | | fgeneshDP_pg.C_scaffold_615000002 | | | | | | |  | | |
| 85182 | | fgeneshDP_pg.C_scaffold_616000002 | | | | | | |  | | |
| 85217 | | fgeneshDP_pg.C_scaffold_625000004 | | | | | | |  | | |
| 85220 | | fgeneshDP_pg.C_scaffold_626000001 | | | | | | |  | | |
| 85248 | | fgeneshDP_pg.C_scaffold_633000002 | | | | | | |  | | |
| 85295 | | fgeneshDP_pg.C_scaffold_645000004 | | | | | | |  | | |
| 85304 | | fgeneshDP_pg.C_scaffold_648000001 | | | | | | |  | | |
| 85336 | | fgeneshDP_pg.C_scaffold_656000004 | | | | | | |  | | |
| 85382 | | fgeneshDP_pg.C_scaffold_671000002 | | | | | | |  | | |
| 85395 | | fgeneshDP_pg.C_scaffold_675000003 | | | | | | |  | | |
| 85404 | | fgeneshDP_pg.C_scaffold_677000005 | | | | | | |  | | |
| 85435 | | fgeneshDP_pg.C_scaffold_688000002 | | | | | | |  | | |
| 85466 | | fgeneshDP_pg.C_scaffold_702000001 | | | | | | |  | | |
| 85472 | | fgeneshDP_pg.C_scaffold_705000001 | | | | | | |  | | |
| 85484 | | fgeneshDP_pg.C_scaffold_710000002 | | | | | | |  | | |
| 85501 | | fgeneshDP_pg.C_scaffold_716000001 | | | | | | |  | | |
| 85505 | | fgeneshDP_pg.C_scaffold_718000001 | | | | | | |  | | |
| 85522 | | fgeneshDP_pg.C_scaffold_726000002 | | | | | | |  | | |
| 85563 | | fgeneshDP_pg.C_scaffold_744000001 | | | | | | |  | | |
| 85580 | | fgeneshDP_pg.C_scaffold_753000001 | | | | | | |  | | |
| 85585 | | fgeneshDP_pg.C_scaffold_757000001 | | | | | | |  | | |
| 85603 | | fgeneshDP_pg.C_scaffold_769000001 | | | | | | |  | | |
| 85613 | | fgeneshDP_pg.C_scaffold_773000004 | | | | | | |  | | |
| 85620 | | fgeneshDP_pg.C_scaffold_779000001 | | | | | | |  | | |
| 85628 | | fgeneshDP_pg.C_scaffold_785000001 | | | | | | |  | | |
| 85641 | | fgeneshDP_pg.C_scaffold_803000001 | | | | | | |  | | |
| 85644 | | fgeneshDP_pg.C_scaffold_808000001 | | | | | | |  | | |
| 85646 | | fgeneshDP_pg.C_scaffold_812000001 | | | | | | |  | | |
| 8579 | | gw1.33.42.1 | | | | | | |  | | |
| 8582 | | gw1.363.12.1 | | | | | | |  | | |
| 85868 | | fgeneshDP_pm.C_scaffold_8000011 | | | | | | |  | | |
| 85931 | | fgeneshDP_pm.C_scaffold_11000009 | | | | | | |  | | |
| 86395 | | fgeneshDP_pm.C_scaffold_30000011 | | | | | | |  | | |
| 86464 | | fgeneshDP_pm.C_scaffold_33000008 | | | | | | |  | | |
| 86546 | | fgeneshDP_pm.C_scaffold_37000007 | | | | | | |  | | |
| 86772 | | fgeneshDP_pm.C_scaffold_48000011 | | | | | | |  | | |
| 86785 | | fgeneshDP_pm.C_scaffold_49000004 | | | | | | |  | | |
| 86974 | | fgeneshDP_pm.C_scaffold_60000002 | | | | | | |  | | |
| 87084 | | fgeneshDP_pm.C_scaffold_66000003 | | | | | | |  | | |
| 87172 | | fgeneshDP_pm.C_scaffold_72000007 | | | | | | |  | | |
| 87255 | | fgeneshDP_pm.C_scaffold_76000011 | | | | | | |  | | |
| 87305 | | fgeneshDP_pm.C_scaffold_79000009 | | | | | | |  | | |
| 87387 | | fgeneshDP_pm.C_scaffold_84000009 | | | | | | |  | | |
| 87430 | | fgeneshDP_pm.C_scaffold_88000005 | | | | | | |  | | |
| 87448 | | fgeneshDP_pm.C_scaffold_89000006 | | | | | | |  | | |
| 87458 | | fgeneshDP_pm.C_scaffold_90000004 | | | | | | |  | | |
| 87578 | | fgeneshDP_pm.C_scaffold_98000011 | | | | | | |  | | |
| 87778 | | fgeneshDP_pm.C_scaffold_114000004 | | | | | | |  | | |
| 87819 | | fgeneshDP_pm.C_scaffold_117000005 | | | | | | |  | | |
| 87843 | | fgeneshDP_pm.C_scaffold_118000017 | | | | | | |  | | |
| 87954 | | fgeneshDP_pm.C_scaffold_127000007 | | | | | | |  | | |
| 88023 | | fgeneshDP_pm.C_scaffold_132000016 | | | | | | |  | | |
| 88026 | | fgeneshDP_pm.C_scaffold_132000019 | | | | | | |  | | |
| 88289 | | fgeneshDP_pm.C_scaffold_154000010 | | | | | | |  | | |
| 88311 | | fgeneshDP_pm.C_scaffold_156000012 | | | | | | |  | | |
| 88339 | | fgeneshDP_pm.C_scaffold_159000005 | | | | | | |  | | |
| 88429 | | fgeneshDP_pm.C_scaffold_167000003 | | | | | | |  | | |
| 88591 | | fgeneshDP_pm.C_scaffold_182000009 | | | | | | |  | | |
| 88622 | | fgeneshDP_pm.C_scaffold_185000006 | | | | | | |  | | |
| 8867 | | gw1.347.12.1 | | | | | | |  | | |
| 88965 | | fgeneshDP_pm.C_scaffold_225000007 | | | | | | |  | | |
| 88993 | | fgeneshDP_pm.C_scaffold_227000008 | | | | | | |  | | |
| 89013 | | fgeneshDP_pm.C_scaffold_229000008 | | | | | | |  | | |
| 89071 | | fgeneshDP_pm.C_scaffold_237000005 | | | | | | |  | | |
| 89388 | | fgeneshDP_pm.C_scaffold_276000007 | | | | | | |  | | |
| 89525 | | fgeneshDP_pm.C_scaffold_295000006 | | | | | | |  | | |
| 89601 | | fgeneshDP_pm.C_scaffold_305000007 | | | | | | |  | | |
| 8976 | | gw1.276.19.1 | | | | | | |  | | |
| 89860 | | fgeneshDP_pm.C_scaffold_349000002 | | | | | | |  | | |
| 90079 | | fgeneshDP_pm.C_scaffold_393000008 | | | | | | |  | | |
| 90232 | | fgeneshDP_pm.C_scaffold_424000002 | | | | | | |  | | |
| 90349 | | fgeneshDP_pm.C_scaffold_454000004 | | | | | | |  | | |
| 90414 | | fgeneshDP_pm.C_scaffold_474000001 | | | | | | |  | | |
| 90467 | | fgeneshDP_pm.C_scaffold_486000005 | | | | | | |  | | |
| 90488 | | fgeneshDP_pm.C_scaffold_490000003 | | | | | | |  | | |
| 90513 | | fgeneshDP_pm.C_scaffold_499000001 | | | | | | |  | | |
| 90624 | | fgeneshDP_pm.C_scaffold_541000001 | | | | | | |  | | |
| 90740 | | fgeneshDP_pm.C_scaffold_583000002 | | | | | | |  | | |
| 90776 | | fgeneshDP_pm.C_scaffold_599000001 | | | | | | |  | | |
| 90815 | | fgeneshDP_pm.C_scaffold_617000002 | | | | | | |  | | |
| 90846 | | fgeneshDP_pm.C_scaffold_636000001 | | | | | | |  | | |
| 90884 | | fgeneshDP_pm.C_scaffold_659000002 | | | | | | |  | | |
| 91024 | | estExt_fgeneshDP_kg.C_20011 | | | | | | |  | | |
| 91034 | | estExt_fgeneshDP_kg.C_30002 | | | | | | |  | | |
| 91068 | | estExt_fgeneshDP_kg.C_50015 | | | | | | |  | | |
| 91080 | | estExt_fgeneshDP_kg.C_60006 | | | | | | |  | | |
| 91146 | | estExt_fgeneshDP_kg.C_110004 | | | | | | |  | | |
| 91159 | | estExt_fgeneshDP_kg.C_120004 | | | | | | |  | | |
| 91170 | | estExt_fgeneshDP_kg.C_130001 | | | | | | |  | | |
| 91198 | | estExt_fgeneshDP_kg.C_150002 | | | | | | |  | | |
| 91210 | | estExt_fgeneshDP_kg.C_150014 | | | | | | |  | | |
| 91250 | | estExt_fgeneshDP_kg.C_200008 | | | | | | |  | | |
| 91251 | | estExt_fgeneshDP_kg.C_200009 | | | | | | |  | | |
| 91300 | | estExt_fgeneshDP_kg.C_240001 | | | | | | |  | | |
| 91328 | | estExt_fgeneshDP_kg.C_260002 | | | | | | |  | | |
| 91339 | | estExt_fgeneshDP_kg.C_270002 | | | | | | |  | | |
| 91361 | | estExt_fgeneshDP_kg.C_280013 | | | | | | |  | | |
| 91392 | | estExt_fgeneshDP_kg.C_320006 | | | | | | |  | | |
| 91410 | | estExt_fgeneshDP_kg.C_340003 | | | | | | |  | | |
| 91414 | | estExt_fgeneshDP_kg.C_350001 | | | | | | |  | | |
| 91416 | | estExt_fgeneshDP_kg.C_350003 | | | | | | |  | | |
| 91426 | | estExt_fgeneshDP_kg.C_360007 | | | | | | |  | | |
| 91427 | | estExt_fgeneshDP_kg.C_360009 | | | | | | |  | | |
| 91428 | | estExt_fgeneshDP_kg.C_360012 | | | | | | |  | | |
| 91433 | | estExt_fgeneshDP_kg.C_360017 | | | | | | |  | | |
| 91459 | | estExt_fgeneshDP_kg.C_400001 | | | | | | |  | | |
| 91491 | | estExt_fgeneshDP_kg.C_440007 | | | | | | |  | | |
| 91538 | | estExt_fgeneshDP_kg.C_490006 | | | | | | |  | | |
| 91543 | | estExt_fgeneshDP_kg.C_500003 | | | | | | |  | | |
| 91548 | | estExt_fgeneshDP_kg.C_500008 | | | | | | |  | | |
| 91559 | | estExt_fgeneshDP_kg.C_510006 | | | | | | |  | | |
| 91566 | | estExt_fgeneshDP_kg.C_520003 | | | | | | |  | | |
| 91582 | | estExt_fgeneshDP_kg.C_540001 | | | | | | |  | | |
| 91615 | | estExt_fgeneshDP_kg.C_570009 | | | | | | |  | | |
| 91644 | | estExt_fgeneshDP_kg.C_600017 | | | | | | |  | | |
| 91656 | | estExt_fgeneshDP_kg.C_620002 | | | | | | |  | | |
| 91672 | | estExt_fgeneshDP_kg.C_640006 | | | | | | |  | | |
| 91690 | | estExt_fgeneshDP_kg.C_670004 | | | | | | |  | | |
| 91719 | | estExt_fgeneshDP_kg.C_710001 | | | | | | |  | | |
| 91743 | | estExt_fgeneshDP_kg.C_730011 | | | | | | |  | | |
| 91761 | | estExt_fgeneshDP_kg.C_760002 | | | | | | |  | | |
| 91767 | | estExt_fgeneshDP_kg.C_760008 | | | | | | |  | | |
| 91810 | | estExt_fgeneshDP_kg.C_830006 | | | | | | |  | | |
| 91822 | | estExt_fgeneshDP_kg.C_840009 | | | | | | |  | | |
| 91824 | | estExt_fgeneshDP_kg.C_850002 | | | | | | |  | | |
| 91831 | | estExt_fgeneshDP_kg.C_860002 | | | | | | |  | | |
| 91861 | | estExt_fgeneshDP_kg.C_910006 | | | | | | |  | | |
| 91876 | | estExt_fgeneshDP_kg.C_930004 | | | | | | |  | | |
| 91878 | | estExt_fgeneshDP_kg.C_930008 | | | | | | |  | | |
| 91900 | | estExt_fgeneshDP_kg.C_970004 | | | | | | |  | | |
| 91906 | | estExt_fgeneshDP_kg.C_990002 | | | | | | |  | | |
| 91919 | | estExt_fgeneshDP_kg.C_1010002 | | | | | | |  | | |
| 91926 | | estExt_fgeneshDP_kg.C_1020004 | | | | | | |  | | |
| 91943 | | estExt_fgeneshDP_kg.C_1050001 | | | | | | |  | | |
| 91945 | | estExt_fgeneshDP_kg.C_1050003 | | | | | | |  | | |
| 91948 | | estExt_fgeneshDP_kg.C_1050006 | | | | | | |  | | |
| 91956 | | estExt_fgeneshDP_kg.C_1060007 | | | | | | |  | | |
| 92003 | | estExt_fgeneshDP_kg.C_1160003 | | | | | | |  | | |
| 92032 | | estExt_fgeneshDP_kg.C_1200001 | | | | | | |  | | |
| 92048 | | estExt_fgeneshDP_kg.C_1230004 | | | | | | |  | | |
| 92057 | | estExt_fgeneshDP_kg.C_1250005 | | | | | | |  | | |
| 92075 | | estExt_fgeneshDP_kg.C_1300001 | | | | | | |  | | |
| 92081 | | estExt_fgeneshDP_kg.C_1310003 | | | | | | |  | | |
| 92090 | | estExt_fgeneshDP_kg.C_1330001 | | | | | | |  | | |
| 92112 | | estExt_fgeneshDP_kg.C_1370003 | | | | | | |  | | |
| 92131 | | estExt_fgeneshDP_kg.C_1420001 | | | | | | |  | | |
| 92182 | | estExt_fgeneshDP_kg.C_1510001 | | | | | | |  | | |
| 92221 | | estExt_fgeneshDP_kg.C_1570006 | | | | | | |  | | |
| 92232 | | estExt_fgeneshDP_kg.C_1600004 | | | | | | |  | | |
| 92233 | | estExt_fgeneshDP_kg.C_1600005 | | | | | | |  | | |
| 92254 | | estExt_fgeneshDP_kg.C_1650004 | | | | | | |  | | |
| 92282 | | estExt_fgeneshDP_kg.C_1720001 | | | | | | |  | | |
| 92295 | | estExt_fgeneshDP_kg.C_1740004 | | | | | | |  | | |
| 92319 | | estExt_fgeneshDP_kg.C_1800001 | | | | | | |  | | |
| 92333 | | estExt_fgeneshDP_kg.C_1820009 | | | | | | |  | | |
| 92334 | | estExt_fgeneshDP_kg.C_1820010 | | | | | | |  | | |
| 92341 | | estExt_fgeneshDP_kg.C_1830001 | | | | | | |  | | |
| 92342 | | estExt_fgeneshDP_kg.C_1830002 | | | | | | |  | | |
| 92348 | | estExt_fgeneshDP_kg.C_1840002 | | | | | | |  | | |
| 92366 | | estExt_fgeneshDP_kg.C_1900002 | | | | | | |  | | |
| 92405 | | estExt_fgeneshDP_kg.C_2010008 | | | | | | |  | | |
| 92413 | | estExt_fgeneshDP_kg.C_2020001 | | | | | | |  | | |
| 92415 | | estExt_fgeneshDP_kg.C_2030001 | | | | | | |  | | |
| 92439 | | estExt_fgeneshDP_kg.C_2090001 | | | | | | |  | | |
| 92440 | | estExt_fgeneshDP_kg.C_2090002 | | | | | | |  | | |
| 92451 | | estExt_fgeneshDP_kg.C_2130001 | | | | | | |  | | |
| 92482 | | estExt_fgeneshDP_kg.C_2210003 | | | | | | |  | | |
| 92524 | | estExt_fgeneshDP_kg.C_2330003 | | | | | | |  | | |
| 92560 | | estExt_fgeneshDP_kg.C_2430001 | | | | | | |  | | |
| 92579 | | estExt_fgeneshDP_kg.C_2480001 | | | | | | |  | | |
| 92587 | | estExt_fgeneshDP_kg.C_2510001 | | | | | | |  | | |
| 92653 | | estExt_fgeneshDP_kg.C_2710002 | | | | | | |  | | |
| 92689 | | estExt_fgeneshDP_kg.C_2800005 | | | | | | |  | | |
| 92697 | | estExt_fgeneshDP_kg.C_2810004 | | | | | | |  | | |
| 92705 | | estExt_fgeneshDP_kg.C_2830004 | | | | | | |  | | |
| 92718 | | estExt_fgeneshDP_kg.C_2900001 | | | | | | |  | | |
| 92744 | | estExt_fgeneshDP_kg.C_3000005 | | | | | | |  | | |
| 92755 | | estExt_fgeneshDP_kg.C_3020002 | | | | | | |  | | |
| 92793 | | estExt_fgeneshDP_kg.C_3130007 | | | | | | |  | | |
| 92810 | | estExt_fgeneshDP_kg.C_3220001 | | | | | | |  | | |
| 92832 | | estExt_fgeneshDP_kg.C_3300003 | | | | | | |  | | |
| 92838 | | estExt_fgeneshDP_kg.C_3310002 | | | | | | |  | | |
| 92875 | | estExt_fgeneshDP_kg.C_3440006 | | | | | | |  | | |
| 92877 | | estExt_fgeneshDP_kg.C_3450002 | | | | | | |  | | |
| 92885 | | estExt_fgeneshDP_kg.C_3500002 | | | | | | |  | | |
| 92909 | | estExt_fgeneshDP_kg.C_3610005 | | | | | | |  | | |
| 92914 | | estExt_fgeneshDP_kg.C_3630001 | | | | | | |  | | |
| 92915 | | estExt_fgeneshDP_kg.C_3630002 | | | | | | |  | | |
| 92921 | | estExt_fgeneshDP_kg.C_3650004 | | | | | | |  | | |
| 92934 | | estExt_fgeneshDP_kg.C_3710002 | | | | | | |  | | |
| 92947 | | estExt_fgeneshDP_kg.C_3760002 | | | | | | |  | | |
| 92958 | | estExt_fgeneshDP_kg.C_3810001 | | | | | | |  | | |
| 92962 | | estExt_fgeneshDP_kg.C_3830001 | | | | | | |  | | |
| 92963 | | estExt_fgeneshDP_kg.C_3830002 | | | | | | |  | | |
| 92966 | | estExt_fgeneshDP_kg.C_3830006 | | | | | | |  | | |
| 92987 | | estExt_fgeneshDP_kg.C_3940002 | | | | | | |  | | |
| 93001 | | estExt_fgeneshDP_kg.C_4030003 | | | | | | |  | | |
| 93052 | | estExt_fgeneshDP_kg.C_4290001 | | | | | | |  | | |
| 93058 | | estExt_fgeneshDP_kg.C_4320002 | | | | | | |  | | |
| 93076 | | estExt_fgeneshDP_kg.C_4410004 | | | | | | |  | | |
| 93101 | | estExt_fgeneshDP_kg.C_4560002 | | | | | | |  | | |
| 93118 | | estExt_fgeneshDP_kg.C_4730002 | | | | | | |  | | |
| 93178 | | estExt_fgeneshDP_kg.C_5180001 | | | | | | |  | | |
| 93186 | | estExt_fgeneshDP_kg.C_5300001 | | | | | | |  | | |
| 93197 | | estExt_fgeneshDP_kg.C_5370001 | | | | | | |  | | |
| 93198 | | estExt_fgeneshDP_kg.C_5370002 | | | | | | |  | | |
| 93208 | | estExt_fgeneshDP_kg.C_5490001 | | | | | | |  | | |
| 93225 | | estExt_fgeneshDP_kg.C_5600001 | | | | | | |  | | |
| 93248 | | estExt_fgeneshDP_kg.C_5890001 | | | | | | |  | | |
| 93261 | | estExt_fgeneshDP_kg.C_6040002 | | | | | | |  | | |
| 93277 | | estExt_fgeneshDP_kg.C_6180001 | | | | | | |  | | |
| 93279 | | estExt_fgeneshDP_kg.C_6220002 | | | | | | |  | | |
| 93297 | | estExt_fgeneshDP_kg.C_6470001 | | | | | | |  | | |
| 93300 | | estExt_fgeneshDP_kg.C_6500001 | | | | | | |  | | |
| 93323 | | estExt_fgeneshDP_kg.C_6860002 | | | | | | |  | | |
| 93329 | | estExt_fgeneshDP_kg.C_7050001 | | | | | | |  | | |
| 93353 | | estExt_fgeneshDP_kg.C_7640001 | | | | | | |  | | |
| 93366 | | estExt_fgeneshDP_pm.C_20003 | | | | | | |  | | |
| 93552 | | estExt_fgeneshDP_pm.C_150009 | | | | | | |  | | |
| 93561 | | estExt_fgeneshDP_pm.C_150021 | | | | | | |  | | |
| 93717 | | estExt_fgeneshDP_pm.C_260026 | | | | | | |  | | |
| 93791 | | estExt_fgeneshDP_pm.C_320017 | | | | | | |  | | |
| 93835 | | estExt_fgeneshDP_pm.C_360013 | | | | | | |  | | |
| 94163 | | estExt_fgeneshDP_pm.C_690014 | | | | | | |  | | |
| 94214 | | estExt_fgeneshDP_pm.C_750002 | | | | | | |  | | |
| 94229 | | estExt_fgeneshDP_pm.C_760005 | | | | | | |  | | |
| 94283 | | estExt_fgeneshDP_pm.C_830003 | | | | | | |  | | |
| 94290 | | estExt_fgeneshDP_pm.C_840001 | | | | | | |  | | |
| 94309 | | estExt_fgeneshDP_pm.C_860005 | | | | | | |  | | |
| 94401 | | estExt_fgeneshDP_pm.C_990011 | | | | | | |  | | |
| 94420 | | estExt_fgeneshDP_pm.C_1020001 | | | | | | |  | | |
| 94509 | | estExt_fgeneshDP_pm.C_1140014 | | | | | | |  | | |
| 94634 | | estExt_fgeneshDP_pm.C_1320006 | | | | | | |  | | |
| 94639 | | estExt_fgeneshDP_pm.C_1330002 | | | | | | |  | | |
| 94663 | | estExt_fgeneshDP_pm.C_1360005 | | | | | | |  | | |
| 94666 | | estExt_fgeneshDP_pm.C_1370001 | | | | | | |  | | |
| 94756 | | estExt_fgeneshDP_pm.C_1510003 | | | | | | |  | | |
| 94806 | | estExt_fgeneshDP_pm.C_1580003 | | | | | | |  | | |
| 94922 | | estExt_fgeneshDP_pm.C_1790008 | | | | | | |  | | |
| 94946 | | estExt_fgeneshDP_pm.C_1820011 | | | | | | |  | | |
| 94960 | | estExt_fgeneshDP_pm.C_1840009 | | | | | | |  | | |
| 95143 | | estExt_fgeneshDP_pm.C_2230008 | | | | | | |  | | |
| 95243 | | estExt_fgeneshDP_pm.C_2460005 | | | | | | |  | | |
| 95300 | | estExt_fgeneshDP_pm.C_2600003 | | | | | | |  | | |
| 95334 | | estExt_fgeneshDP_pm.C_2690002 | | | | | | |  | | |
| 95343 | | estExt_fgeneshDP_pm.C_2720005 | | | | | | |  | | |
| 95462 | | estExt_fgeneshDP_pm.C_3000002 | | | | | | |  | | |
| 95724 | | estExt_fgeneshDP_pm.C_3850004 | | | | | | |  | | |
| 95748 | | estExt_fgeneshDP_pm.C_3940002 | | | | | | |  | | |
| 95923 | | estExt_fgeneshDP_pm.C_4820004 | | | | | | |  | | |
| 96157 | | estExt_fgeneshDP_pm.C_6760004 | | | | | | |  | | |
| 96216 | | estExt_fgeneshDP_pg.C_10006 | | | | | | |  | | |
| 96241 | | estExt_fgeneshDP_pg.C_20035 | | | | | | |  | | |
| 96243 | | estExt_fgeneshDP_pg.C_20039 | | | | | | |  | | |
| 96266 | | estExt_fgeneshDP_pg.C_30033 | | | | | | |  | | |
| 96277 | | estExt_fgeneshDP_pg.C_30091 | | | | | | |  | | |
| 96288 | | estExt_fgeneshDP_pg.C_40029 | | | | | | |  | | |
| 96289 | | estExt_fgeneshDP_pg.C_40030 | | | | | | |  | | |
| 96290 | | estExt_fgeneshDP_pg.C_40034 | | | | | | |  | | |
| 96302 | | estExt_fgeneshDP_pg.C_40075 | | | | | | |  | | |
| 96306 | | estExt_fgeneshDP_pg.C_50012 | | | | | | |  | | |
| 96313 | | estExt_fgeneshDP_pg.C_50027 | | | | | | |  | | |
| 96314 | | estExt_fgeneshDP_pg.C_50028 | | | | | | |  | | |
| 96315 | | estExt_fgeneshDP_pg.C_50030 | | | | | | |  | | |
| 96316 | | estExt_fgeneshDP_pg.C_50031 | | | | | | |  | | |
| 96319 | | estExt_fgeneshDP_pg.C_50039 | | | | | | |  | | |
| 96320 | | estExt_fgeneshDP_pg.C_50040 | | | | | | |  | | |
| 9634 | | gw1.299.8.1 | | | | | | |  | | |
| 96469 | | estExt_fgeneshDP_pg.C_120041 | | | | | | |  | | |
| 96479 | | estExt_fgeneshDP_pg.C_130027 | | | | | | |  | | |
| 96485 | | estExt_fgeneshDP_pg.C_130047 | | | | | | |  | | |
| 96490 | | estExt_fgeneshDP_pg.C_130059 | | | | | | |  | | |
| 96508 | | estExt_fgeneshDP_pg.C_140050 | | | | | | |  | | |
| 96541 | | estExt_fgeneshDP_pg.C_160032 | | | | | | |  | | |
| 96549 | | estExt_fgeneshDP_pg.C_170015 | | | | | | |  | | |
| 96578 | | estExt_fgeneshDP_pg.C_180058 | | | | | | |  | | |
| 96581 | | estExt_fgeneshDP_pg.C_190003 | | | | | | |  | | |
| 96671 | | estExt_fgeneshDP_pg.C_230037 | | | | | | |  | | |
| 96674 | | estExt_fgeneshDP_pg.C_230043 | | | | | | |  | | |
| 96692 | | estExt_fgeneshDP_pg.C_240043 | | | | | | |  | | |
| 96696 | | estExt_fgeneshDP_pg.C_250001 | | | | | | |  | | |
| 96706 | | estExt_fgeneshDP_pg.C_250020 | | | | | | |  | | |
| 96815 | | estExt_fgeneshDP_pg.C_300045 | | | | | | |  | | |
| 96816 | | estExt_fgeneshDP_pg.C_300046 | | | | | | |  | | |
| 96887 | | estExt_fgeneshDP_pg.C_350022 | | | | | | |  | | |
| 96896 | | estExt_fgeneshDP_pg.C_350038 | | | | | | |  | | |
| 96905 | | estExt_fgeneshDP_pg.C_360011 | | | | | | |  | | |
| 96909 | | estExt_fgeneshDP_pg.C_360021 | | | | | | |  | | |
| 96910 | | estExt_fgeneshDP_pg.C_360023 | | | | | | |  | | |
| 96918 | | estExt_fgeneshDP_pg.C_360047 | | | | | | |  | | |
| 96926 | | estExt_fgeneshDP_pg.C_370046 | | | | | | |  | | |
| 96983 | | estExt_fgeneshDP_pg.C_420001 | | | | | | |  | | |
| 96999 | | estExt_fgeneshDP_pg.C_430011 | | | | | | |  | | |
| 97088 | | estExt_fgeneshDP_pg.C_480021 | | | | | | |  | | |
| 97110 | | estExt_fgeneshDP_pg.C_500008 | | | | | | |  | | |
| 97156 | | estExt_fgeneshDP_pg.C_520027 | | | | | | |  | | |
| 97157 | | estExt_fgeneshDP_pg.C_520028 | | | | | | |  | | |
| 97164 | | estExt_fgeneshDP_pg.C_530006 | | | | | | |  | | |
| 97165 | | estExt_fgeneshDP_pg.C_530009 | | | | | | |  | | |
| 97231 | | estExt_fgeneshDP_pg.C_580015 | | | | | | |  | | |
| 97267 | | estExt_fgeneshDP_pg.C_600036 | | | | | | |  | | |
| 97307 | | estExt_fgeneshDP_pg.C_630031 | | | | | | |  | | |
| 97330 | | estExt_fgeneshDP_pg.C_650032 | | | | | | |  | | |
| 97354 | | estExt_fgeneshDP_pg.C_670027 | | | | | | |  | | |
| 97376 | | estExt_fgeneshDP_pg.C_690019 | | | | | | |  | | |
| 9742 | | gw1.206.13.1 | | | | | | |  | | |
| 97512 | | estExt_fgeneshDP_pg.C_790023 | | | | | | |  | | |
| 97541 | | estExt_fgeneshDP_pg.C_830003 | | | | | | |  | | |
| 97554 | | estExt_fgeneshDP_pg.C_840017 | | | | | | |  | | |
| 97634 | | estExt_fgeneshDP_pg.C_920006 | | | | | | |  | | |
| 97644 | | estExt_fgeneshDP_pg.C_930004 | | | | | | |  | | |
| 97647 | | estExt_fgeneshDP_pg.C_930007 | | | | | | |  | | |
| 97677 | | estExt_fgeneshDP_pg.C_970001 | | | | | | |  | | |
| 97695 | | estExt_fgeneshDP_pg.C_980019 | | | | | | |  | | |
| 97761 | | estExt_fgeneshDP_pg.C_1040019 | | | | | | |  | | |
| 97766 | | estExt_fgeneshDP_pg.C_1050011 | | | | | | |  | | |
| 97781 | | estExt_fgeneshDP_pg.C_1060019 | | | | | | |  | | |
| 97802 | | estExt_fgeneshDP_pg.C_1080019 | | | | | | |  | | |
| 97831 | | estExt_fgeneshDP_pg.C_1120008 | | | | | | |  | | |
| 97832 | | estExt_fgeneshDP_pg.C_1120009 | | | | | | |  | | |
| 97847 | | estExt_fgeneshDP_pg.C_1150004 | | | | | | |  | | |
| 97912 | | estExt_fgeneshDP_pg.C_1210013 | | | | | | |  | | |
| 97929 | | estExt_fgeneshDP_pg.C_1230006 | | | | | | |  | | |
| 97937 | | estExt_fgeneshDP_pg.C_1240009 | | | | | | |  | | |
| 97977 | | estExt_fgeneshDP_pg.C_1280022 | | | | | | |  | | |
| 98003 | | estExt_fgeneshDP_pg.C_1310026 | | | | | | |  | | |
| 98104 | | estExt_fgeneshDP_pg.C_1430017 | | | | | | |  | | |
| 98108 | | estExt_fgeneshDP_pg.C_1430026 | | | | | | |  | | |
| 98199 | | estExt_fgeneshDP_pg.C_1550008 | | | | | | |  | | |
| 98231 | | estExt_fgeneshDP_pg.C_1580014 | | | | | | |  | | |
| 98233 | | estExt_fgeneshDP_pg.C_1590001 | | | | | | |  | | |
| 98236 | | estExt_fgeneshDP_pg.C_1600001 | | | | | | |  | | |
| 98287 | | estExt_fgeneshDP_pg.C_1650019 | | | | | | |  | | |
| 98336 | | estExt_fgeneshDP_pg.C_1710021 | | | | | | |  | | |
| 98383 | | estExt_fgeneshDP_pg.C_1780006 | | | | | | |  | | |
| 98396 | | estExt_fgeneshDP_pg.C_1790012 | | | | | | |  | | |
| 98412 | | estExt_fgeneshDP_pg.C_1810004 | | | | | | |  | | |
| 98424 | | estExt_fgeneshDP_pg.C_1820013 | | | | | | |  | | |
| 98426 | | estExt_fgeneshDP_pg.C_1820017 | | | | | | |  | | |
| 98485 | | estExt_fgeneshDP_pg.C_1900013 | | | | | | |  | | |
| 98488 | | estExt_fgeneshDP_pg.C_1910005 | | | | | | |  | | |
| 98489 | | estExt_fgeneshDP_pg.C_1910010 | | | | | | |  | | |
| 98501 | | estExt_fgeneshDP_pg.C_1930014 | | | | | | |  | | |
| 98560 | | estExt_fgeneshDP_pg.C_2030009 | | | | | | |  | | |
| 98568 | | estExt_fgeneshDP_pg.C_2040014 | | | | | | |  | | |
| 98569 | | estExt_fgeneshDP_pg.C_2040018 | | | | | | |  | | |
| 98570 | | estExt_fgeneshDP_pg.C_2040019 | | | | | | |  | | |
| 98573 | | estExt_fgeneshDP_pg.C_2050010 | | | | | | |  | | |
| 98602 | | estExt_fgeneshDP_pg.C_2100006 | | | | | | |  | | |
| 98640 | | estExt_fgeneshDP_pg.C_2150008 | | | | | | |  | | |
| 98669 | | estExt_fgeneshDP_pg.C_2180014 | | | | | | |  | | |
| 98688 | | estExt_fgeneshDP_pg.C_2220013 | | | | | | |  | | |
| 98693 | | estExt_fgeneshDP_pg.C_2230012 | | | | | | |  | | |
| 98719 | | estExt_fgeneshDP_pg.C_2270003 | | | | | | |  | | |
| 98747 | | estExt_fgeneshDP_pg.C_2310013 | | | | | | |  | | |
| 98751 | | estExt_fgeneshDP_pg.C_2320006 | | | | | | |  | | |
| 98839 | | estExt_fgeneshDP_pg.C_2460012 | | | | | | |  | | |
| 98865 | | estExt_fgeneshDP_pg.C_2510012 | | | | | | |  | | |
| 98898 | | estExt_fgeneshDP_pg.C_2570005 | | | | | | |  | | |
| 98950 | | estExt_fgeneshDP_pg.C_2650010 | | | | | | |  | | |
| 98973 | | estExt_fgeneshDP_pg.C_2710010 | | | | | | |  | | |
| 98990 | | estExt_fgeneshDP_pg.C_2730011 | | | | | | |  | | |
| 98991 | | estExt_fgeneshDP_pg.C_2730013 | | | | | | |  | | |
| 99025 | | estExt_fgeneshDP_pg.C_2780011 | | | | | | |  | | |
| 99067 | | estExt_fgeneshDP_pg.C_2830008 | | | | | | |  | | |
| 99118 | | estExt_fgeneshDP_pg.C_2960007 | | | | | | |  | | |
| 99132 | | estExt_fgeneshDP_pg.C_2980011 | | | | | | |  | | |
| 99159 | | estExt_fgeneshDP_pg.C_3020011 | | | | | | |  | | |
| 99161 | | estExt_fgeneshDP_pg.C_3030004 | | | | | | |  | | |
| 99194 | | estExt_fgeneshDP_pg.C_3100007 | | | | | | |  | | |
| 99209 | | estExt_fgeneshDP_pg.C_3140004 | | | | | | |  | | |
| 99223 | | estExt_fgeneshDP_pg.C_3160010 | | | | | | |  | | |
| 99248 | | estExt_fgeneshDP_pg.C_3240003 | | | | | | |  | | |
| 99251 | | estExt_fgeneshDP_pg.C_3240016 | | | | | | |  | | |
| 99260 | | estExt_fgeneshDP_pg.C_3260006 | | | | | | |  | | |
| 9928 | | gw1.57.8.1 | | | |  | | |  | | |
| 99311 | | estExt_fgeneshDP_pg.C_3360001 | | | | | | |  | | |
| 99319 | | estExt_fgeneshDP_pg.C_3370004 | | | | | | |  | | |
| 99342 | | estExt_fgeneshDP_pg.C_3410008 | | | | | | |  | | |
| 99393 | | estExt_fgeneshDP_pg.C_3540013 | | | | | | |  | | |
| 99401 | | estExt_fgeneshDP_pg.C_3560006 | | | | | | |  | | |
| 99434 | | estExt_fgeneshDP_pg.C_3630004 | | | | | | |  | | |
| 99462 | | estExt_fgeneshDP_pg.C_3690002 | | | | | | |  | | |
| 99505 | | estExt_fgeneshDP_pg.C_3810005 | | | | | | |  | | |
| 99515 | | estExt_fgeneshDP_pg.C_3850003 | | | | | | |  | | |
| 99550 | | estExt_fgeneshDP_pg.C_3940001 | | | | | | |  | | |
| 99606 | | estExt_fgeneshDP_pg.C_4090002 | | | | | | |  | | |
| 99638 | | estExt_fgeneshDP_pg.C_4210010 | | | | | | |  | | |
| 99750 | | estExt_fgeneshDP_pg.C_4630001 | | | | | | |  | | |
| 99765 | | estExt_fgeneshDP_pg.C_4690007 | | | | | | |  | | |
| 99782 | | estExt_fgeneshDP_pg.C_4740007 | | | | | | |  | | |
| 99798 | | estExt_fgeneshDP_pg.C_4820006 | | | | | | |  | | |
| 9980 | | gw1.313.11.1 | | | | | | |  | | |
| 99837 | | estExt_fgeneshDP_pg.C_4970003 | | | | | | |  | | |
| 99847 | | estExt_fgeneshDP_pg.C_5020003 | | | | | | |  | | |
| 99877 | | estExt_fgeneshDP_pg.C_5150006 | | | | | | |  | | |
| 99980 | | estExt_fgeneshDP_pg.C_5710002 | | | | | | |  | | |
| **D. discoideum genes with no hit (Bit score >50) in D. purpureum** | | | | | | | | | |  | |
| **D. discoideum I.D. (DDB_G)** | | | **Gene name** | | | | **annotation** | | |  | |
| DDB_G0267178 | | | DDB_G0267178 | | | | Slime mold (D.discoideum) transposon DIRS-1, complete, clone SB41. | | |  | |
| DDB_G0267180 | | | DDB_G0267180 | | | | Slime mold (D.discoideum) transposon DIRS-1, complete, clone SB41. | | |  | |
| DDB_G0267182 | | | DDB_G0267182 | | | | Slime mold (D.discoideum) transposon DIRS-1, complete, clone SB41. | | |  | |
| DDB_G0267184 | | | DDB_G0267184 | | | | Slime mold (D.discoideum) transposon DIRS-1, complete, clone SB41. | | |  | |
| DDB_G0267186 | | | DDB_G0267186 | | | | Slime mold (D.discoideum) transposon DIRS-1, complete, clone SB41. | | |  | |
| DDB_G0267188 | | | DDB_G0267188 | | | | Slime mold (D.discoideum) transposon DIRS-1, complete, clone SB41. | | |  | |
| DDB_G0267190 | | | DDB_G0267190 | | | |  | | |  | |
| DDB_G0267192 | | | DDB_G0267192 | | | |  | | |  | |
| DDB_G0267194 | | | DDB_G0267194 | | | |  | | |  | |
| DDB_G0267196 | | | DDB_G0267196 | | | |  | | |  | |
| DDB_G0267198 | | | DDB_G0267198 | | | |  | | |  | |
| DDB_G0267200 | | | DDB_G0267200 | | | |  | | |  | |
| DDB_G0267202 | | | DDB_G0267202 | | | |  | | |  | |
| DDB_G0267204 | | | DDB_G0267204 | | | |  | | |  | |
| DDB_G0267206 | | | DDB_G0267206 | | | | Slime mold (D.discoideum) transposon DIRS-1, complete, clone SB41. | | |  | |
| DDB_G0267208 | | | DDB_G0267208 | | | | Slime mold (D.discoideum) transposon DIRS-1, complete, clone SB41. | | |  | |
| DDB_G0267210 | | | DDB_G0267210 | | | | Slime mold (D.discoideum) transposon DIRS-1, complete, clone SB41. | | |  | |
| DDB_G0267212 | | | DDB_G0267212 | | | | Slime mold (D.discoideum) transposon DIRS-1, complete, clone SB41. | | |  | |
| DDB_G0267214 | | | DDB_G0267214 | | | |  | | |  | |
| DDB_G0267218 | | | DDB_G0267218 | | | |  | | |  | |
| DDB_G0267220 | | | DDB_G0267220 | | | |  | | |  | |
| DDB_G0267222 | | | DDB_G0267222 | | | |  | | |  | |
| DDB_G0267224 | | | DDB_G0267224 | | | |  | | |  | |
| DDB_G0267228 | | | DDB_G0267228 | | | |  | | |  | |
| DDB_G0267230 | | | DDB_G0267230 | | | |  | | |  | |
| DDB_G0267232 | | | DDB_G0267232 | | | |  | | |  | |
| DDB_G0267234 | | | DDB_G0267234 | | | |  | | |  | |
| DDB_G0267236 | | | DDB_G0267236 | | | | Slime mold (D.discoideum) transposon DIRS-1, complete, clone SB41. | | |  | |
| DDB_G0294483 | | | M11340_2 | | | |  | | |  | |
| DDB_G0294485 | | | M11340_3 | | | |  | | |  | |
| DDB_G0267238 | | | DDB_G0267238 | | | | Slime mold (D.discoideum) transposon DIRS-1, complete, clone SB41. | | |  | |
| DDB_G0267240 | | | DDB_G0267240 | | | | Slime mold (D.discoideum) transposon DIRS-1, complete, clone SB41. | | |  | |
| DDB_G0267242 | | | DDB_G0267242 | | | | Slime mold (D.discoideum) transposon DIRS-1, complete, clone SB41. | | |  | |
| DDB_G0267264 | | | DDB_G0267264 | | | |  | | |  | |
| DDB_G0267244 | | | DDB_G0267244 | | | | Slime mold (D.discoideum) transposon DIRS-1, complete, clone SB41. | | |  | |
| DDB_G0267246 | | | DDB_G0267246 | | | | Slime mold (D.discoideum) transposon DIRS-1, complete, clone SB41. | | |  | |
| DDB_G0267248 | | | DDB_G0267248 | | | | Slime mold (D.discoideum) transposon DIRS-1, complete, clone SB41. | | |  | |
| DDB_G0267250 | | | DDB_G0267250 | | | |  | | |  | |
| DDB_G0267252 | | | DDB_G0267252 | | | |  | | |  | |
| DDB_G0267254 | | | DDB_G0267254_ps | | | | pseudogene | | |  | |
| DDB_G0267258 | | | DDB_G0267258 | | | | Slime mold (D.discoideum) transposon DIRS-1, complete, clone SB41. | | |  | |
| DDB_G0267260 | | | DDB_G0267260 | | | | Slime mold (D.discoideum) transposon DIRS-1, complete, clone SB41. | | |  | |
| DDB_G0267262 | | | DDB_G0267262 | | | | Slime mold (D.discoideum) transposon DIRS-1, complete, clone SB41. | | |  | |
| DDB_G0294481 | | | M11340_1 | | | |  | | |  | |
| DDB_G0294493 | | | M11339_1 | | | |  | | |  | |
| DDB_G0267270 | | | DDB_G0267270 | | | | Slime mold (D.discoideum) transposon DIRS-1, complete, clone SB41. | | |  | |
| DDB_G0267272 | | | DDB_G0267272 | | | | Slime mold (D.discoideum) transposon DIRS-1, complete, clone SB41. | | |  | |
| DDB_G0267274 | | | DDB_G0267274 | | | |  | | |  | |
| DDB_G0267276 | | | DDB_G0267276 | | | |  | | |  | |
| DDB_G0267278 | | | DDB_G0267278 | | | |  | | |  | |
| DDB_G0267280 | | | DDB_G0267280 | | | |  | | |  | |
| DDB_G0267282 | | | DDB_G0267282 | | | | Slime mold (D.discoideum) transposon DIRS-1, complete, clone SB41. | | |  | |
| DDB_G0267284 | | | DDB_G0267284 | | | |  | | |  | |
| DDB_G0267286 | | | DDB_G0267286 | | | |  | | |  | |
| DDB_G0267290 | | | DDB_G0267290 | | | | Slime mold (D.discoideum) transposon DIRS-1, complete, clone SB41. | | |  | |
| DDB_G0267292 | | | DDB_G0267292 | | | | Slime mold (D.discoideum) transposon DIRS-1, complete, clone SB41. | | |  | |
| DDB_G0267294 | | | DDB_G0267294 | | | | Slime mold (D.discoideum) transposon DIRS-1, complete, clone SB41. | | |  | |
| DDB_G0267296 | | | DDB_G0267296 | | | | Slime mold (D.discoideum) transposon DIRS-1, complete, clone SB41. | | |  | |
| DDB_G0267298 | | | DDB_G0267298 | | | |  | | |  | |
| DDB_G0267300 | | | DDB_G0267300 | | | | Slime mold (D.discoideum) transposon DIRS-1, complete, clone SB41. | | |  | |
| DDB_G0267302 | | | DDB_G0267302 | | | |  | | |  | |
| DDB_G0267308 | | | DDB_G0267308 | | | | LTR-RETROTRANSPOSON SKIPPER, GAG (GAG). | | |  | |
| DDB_G0267310 | | | DDB_G0267310 | | | | LTR-RETROTRANSPOSON SKIPPER, GAG (GAG). | | |  | |
| DDB_G0267312 | | | DDB_G0267312 | | | |  | | |  | |
| DDB_G0267314 | | | DDB_G0267314 | | | | Slime mold (D.discoideum) transposon DIRS-1, complete, clone SB41. | | |  | |
| DDB_G0267316 | | | DDB_G0267316 | | | | Slime mold (D.discoideum) transposon DIRS-1, complete, clone SB41. | | |  | |
| DDB_G0267318 | | | DDB_G0267318 | | | |  | | |  | |
| DDB_G0267320 | | | DDB_G0267320 | | | |  | | |  | |
| DDB_G0267322 | | | DDB_G0267322 | | | | Slime mold (D.discoideum) transposon DIRS-1, complete, clone SB41. | | |  | |
| DDB_G0267324 | | | DDB_G0267324 | | | | Slime mold (D.discoideum) transposon DIRS-1, complete, clone SB41. | | |  | |
| DDB_G0294495 | | | M11339_2 | | | |  | | |  | |
| DDB_G0294497 | | | M11339_3 | | | |  | | |  | |
| DDB_G0267326 | | | DDB_G0267326 | | | | Slime mold (D.discoideum) transposon DIRS-1, complete, clone SB41. | | |  | |
| DDB_G0267328 | | | DDB_G0267328 | | | | Slime mold (D.discoideum) transposon DIRS-1, complete, clone SB41. | | |  | |
| DDB_G0267330 | | | DDB_G0267330 | | | | Slime mold (D.discoideum) transposon DIRS-1, complete, clone SB41. | | |  | |
| DDB_G0267332 | | | DDB_G0267332 | | | |  | | |  | |
| DDB_G0267338 | | | DDB_G0267338 | | | | Slime mold (D.discoideum) transposon DIRS-1, complete, clone SB41. | | |  | |
| DDB_G0267334 | | | DDB_G0267334 | | | | Slime mold (D.discoideum) transposon DIRS-1, complete, clone SB41. | | |  | |
| DDB_G0267336 | | | DDB_G0267336 | | | |  | | |  | |
| DDB_G0267342 | | | DDB_G0267342 | | | | Slime mold (D.discoideum) transposon DIRS-1, complete, clone SB41. | | |  | |
| DDB_G0267344 | | | DDB_G0267344 | | | |  | | |  | |
| DDB_G0267346 | | | DDB_G0267346 | | | |  | | |  | |
| DDB_G0267348 | | | DDB_G0267348 | | | |  | | |  | |
| DDB_G0267350 | | | DDB_G0267350 | | | |  | | |  | |
| DDB_G0267352 | | | DDB_G0267352 | | | |  | | |  | |
| DDB_G0267354 | | | DDB_G0267354 | | | |  | | |  | |
| DDB_G0267358 | | | DDB_G0267358 | | | | LTR-RETROTRANSPOSON SKIPPER, GAG (GAG). | | |  | |
| DDB_G0267360 | | | DDB_G0267360 | | | |  | | |  | |
| DDB_G0267364 | | | DDB_G0267364 | | | | LTR-RETROTRANSPOSON SKIPPER, GAG (GAG). | | |  | |
| DDB_G0294439 | | | pro | | | | protease homolog | | |  | |
| DDB_G0267368 | | | DDB_G0267368 | | | | LTR-RETROTRANSPOSON SKIPPER, GAG (GAG). | | |  | |
| DDB_G0267370 | | | DDB_G0267370 | | | |  | | |  | |
| DDB_G0267372 | | | DDB_G0267372 | | | | 5'-B-MODULE (Fragment). | | |  | |
| DDB_G0268474 | | | DDB_G0268474 | | | | 5'-B-MODULE (Fragment). | | |  | |
| DDB_G0268476 | | | DDB_G0268476 | | | |  | | |  | |
| DDB_G0268260 | | | DDB_G0268260 | | | |  | | |  | |
| DDB_G0267458 | | | AAC4 | | | | AAC-rich mRNA | | |  | |
| DDB_G0268262 | | | DDB_G0268262 | | | |  | | |  | |
| DDB_G0267494 | | | DDB_G0267494 | | | |  | | |  | |
| DDB_G0268268 | | | DDB_G0268268 | | | |  | | |  | |
| DDB_G0267518 | | | DDB_G0267518 | | | |  | | |  | |
| DDB_G0267520 | | | DDB_G0267520 | | | |  | | |  | |
| DDB_G0267542 | | | DDB_G0267542 | | | |  | | |  | |
| DDB_G0268274 | | | DDB_G0268274 | | | |  | | |  | |
| DDB_G0268276 | | | DDB_G0268276 | | | |  | | |  | |
| DDB_G0267558 | | | DDB_G0267558 | | | |  | | |  | |
| DDB_G0267596 | | | DDB_G0267596 | | | |  | | |  | |
| DDB_G0268290 | | | DDB_G0268290 | | | |  | | |  | |
| DDB_G0268294 | | | DDB_G0268294 | | | |  | | |  | |
| DDB_G0268484 | | | DDB_G0268484 | | | |  | | |  | |
| DDB_G0267618 | | | DDB_G0267618 | | | |  | | |  | |
| DDB_G0267626 | | | DDB_G0267626 | | | |  | | |  | |
| DDB_G0267628 | | | DDB_G0267628 | | | |  | | |  | |
| DDB_G0268296 | | | DDB_G0268296 | | | |  | | |  | |
| DDB_G0268298 | | | DDB_G0268298 | | | |  | | |  | |
| tRNA-Lys-UUU-2 | | | tRNA-Lys-UUU-2 | | | | transfers a lysine residue to a growing polypeptide chain during protein synthesis%0A | | |  | |
| DDB_G0268300 | | | DDB_G0268300 | | | |  | | |  | |
| DDB_G0268486 | | | DDB_G0268486_ps | | | | pseudogene | | |  | |
| DDB_G0267652 | | | DDB_G0267652 | | | |  | | |  | |
| DDB_G0267662 | | | DDB_G0267662 | | | |  | | |  | |
| DDB_G0267678 | | | DDB_G0267678 | | | |  | | |  | |
| DDB_G0268308 | | | DDB_G0268308 | | | |  | | |  | |
| DDB_G0268310 | | | DDB_G0268310 | | | |  | | |  | |
| DDB_G0268312 | | | DDB_G0268312 | | | |  | | |  | |
| DDB_G0267696 | | | DDB_G0267696 | | | |  | | |  | |
| DDB_G0267702 | | | DDB_G0267702 | | | |  | | |  | |
| DDB_G0268318 | | | DDB_G0268318 | | | |  | | |  | |
| DDB_G0268324 | | | DDB_G0268324 | | | |  | | |  | |
| DDB_G0267720 | | | DDB_G0267720 | | | |  | | |  | |
| DDB_G0268338 | | | DDB_G0268338 | | | |  | | |  | |
| DDB_G0267736 | | | DDB_G0267736 | | | | Group-specific antigen. | | |  | |
| DDB_G0267738 | | | DDB_G0267738 | | | | Group-specific antigen. | | |  | |
| tRNA-Thr-AGU-3 | | | tRNA-Thr-AGU-3 | | | | transfers a threonine residue to a growing polypeptide chain during protein synthesis%0A | | |  | |
| DDB_G0268510 | | | DDB_G0268510 | | | | Pol. | | |  | |
| tRNA-Asn-GUU-8 | | | tRNA-Asn-GUU-8 | | | | transfers an asparagine residue to a growing polypeptide chain during protein synthesis%0A | | |  | |
| DDB_G0268344 | | | DDB_G0268344 | | | |  | | |  | |
| DDB_G0267764 | | | DDB_G0267764 | | | |  | | |  | |
| tRNA-Ser-UGA-1 | | | tRNA-Ser-UGA-1 | | | | transfers a serine residue to a growing polypeptide chain during protein synthesis%0A | | |  | |
| tRNA-Cys-GCA-1 | | | tRNA-Cys-GCA-1 | | | | transfers a cysteine residue to a growing polypeptide chain during protein synthesis%0A | | |  | |
| tRNA-Ser-UGA-4 | | | tRNA-Ser-UGA-4 | | | | transfers a serine residue to a growing polypeptide chain during protein synthesis%0A | | |  | |
| DDB_G0267772 | | | DDB_G0267772 | | | |  | | |  | |
| tRNA-Pro-UGG-2 | | | tRNA-Pro-UGG-2 | | | | transfers a proline residue to a growing polypeptide chain during protein synthesis%0A | | |  | |
| DDB_G0267778 | | | DDB_G0267778 | | | | Pol. | | |  | |
| tRNA-Pro-UGG-3 | | | tRNA-Pro-UGG-3 | | | | transfers a proline residue to a growing polypeptide chain during protein synthesis%0A | | |  | |
| DDB_G0267782 | | | DDB_G0267782 | | | |  | | |  | |
| DDB_G0267790 | | | DDB_G0267790 | | | |  | | |  | |
| DDB_G0267802 | | | DDB_G0267802 | | | |  | | |  | |
| DDB_G0267806 | | | DDB_G0267806 | | | |  | | |  | |
| DDB_G0267808 | | | DDB_G0267808 | | | |  | | |  | |
| DDB_G0267812 | | | DDB_G0267812 | | | |  | | |  | |
| DDB_G0267816 | | | DDB_G0267816 | | | |  | | |  | |
| DDB_G0267826 | | | DDB_G0267826 | | | |  | | |  | |
| DDB_G0267832 | | | DDB_G0267832 | | | |  | | |  | |
[truncated: 365,886 more chars]
